# Still beyond a chance: Distribution of faults in elite show-jumping horses

**DOI:** 10.1371/journal.pone.0264615

**Published:** 2022-03-16

**Authors:** Klára Ničová, Jitka Bartošová

**Affiliations:** 1 Department of Ethology, Institute of Animal Science, Prague, Czech Republic; 2 Department of Ethology and Companion Animal Science, Faculty of Agrobiology, Food and Natural Resources, Czech University of Life Sciences in Prague, Prague, Czech Republic; Massey University, NEW ZEALAND

## Abstract

This study aimed to identify factors that can influence show-jumping performance during top level competitions in the Western European League (2017/2018, CSI5*). The performance data of 144 riders and 222 horses were obtained from video records (FEI TV/ website). Riders with horses achieved a total of 9114 jumping-efforts over 320 obstacles including oxers (n = 142), oxers with water (n = 15), triple bars (n = 6), verticals (n = 136), verticals with water (n = 14) and walls (n = 7). Obstacles in the first round (FR) or in jump off (JO) were standing either as single (n = 6290) or as a combination of two or three fences in a row (n = 2824). The overall fault rate (dropping the pole or refusal/run-out to jump) was 7.85%. The probability of a fault increased with the rank of the fence in the course of FR (F _(1, 7649)_ = 5.29, P < 0.0001, GzLMM; PROC GLIMMIX, SAS). The highest probability of a fault (F _(5, 7649)_ = 2.51, P < 0.03) in FR was found on the vertical obstacle with water (0.125 ± 0.021, LSMEAN ± standard error) while the lowest was on the triple bar (0.037 ± 0.015) and wall (0.048 ± 0.020). Riders who completed more starts in previous competitions achieved a lower fault rate (F _(1, 7649)_ = 6.17, P < 0.02) in FR as well as in JO (F _(1, 931)_ = 7.05, P < 0.01). The probability of faults in JO decreased with a higher speed (F _(1, 931)_ = 6.66, P < 0.01) but there was no significant correlation between the speed in JO and FR in individual horses (r = 0.26, P < 0.01). More faults were found on the fences within combinations in both rounds (FR, JO). The highest probability of faults was found on the first fence (FR 0.095 ± 0.016; JO 0.043 ± 0.008) or second fence (JO 0.055 ± 0.020) of the double combination compared to the least probability of faults on single obstacles (FR 0.057 ± 0.007, F _(5, 7649)_ = 5.29, P < 0.0001; JO 0.100 ± 0.027, F _(1, 931)_ = 3.39, P < 0.04). Other tested factors did not affect show-jumping performance. Some faults are still more likely and not random in a show-jumping course, therefore, the appropriate training focus can improve performance and safety in competitions.

## Introduction

The behavioural flexibility of horses has led to a broad range of equestrian disciplines. In equestrian sport, the horse and its rider are two athletes who should work together as a pair even though they differ in anatomy, movement abilities and experiences. Generally, the declared objective of horse training by Fédération Equestre Internationale (FEI) should be achieved such that an attentive, calm and flexible horse is working in interest and harmony with its rider [1, 2]. The task of the pair in show-jumping, the most popular equestrian discipline, is to pass the prescribed course with the lowest number of penalty points (fault rate) for the dropped poles, obstacles or run-outs with refusals and exceeding the time limit which is defined in the Jumping rules [2]. The long-term goal of the owners and breeders is then to achieve a good score in various competition rankings.

Horse performance and competition success result from many involved factors. For show-jumping horses, the jumping ability and willingness to jump are the most important determinants [3, 4]. Parameters regarding the take-off, landing and lifting of limbs above the obstacle and the angle of the bascule seem to play a crucial role in the show-jumping horse selection [5]. It is known that these traits are affected by length of the training [5, 6]. In addition to physical fitness [7], the mental state of the horse, which is based on personality aspects such as emotionality, reactivity and learning abilities, has to be taken into account [8–10]. These traits can impact the rideability of the horse, which is one of the most important traits for riding horses and frequently evaluated in many breeding programs [11, 12]. During competitions, horses are exposed to various stimuli, including those which impose stress on the animal, such as light, noise, a variety of obstacles, psychological factors, spectators, rider, and equipment [13–15]. All these stimuli and factors can compromise the welfare of the horse, and thus, its performance. For this reason, gradual habituation and associative learning should be a part of the training [16].

The psychological dynamics between a rider and the horse is still poorly understood [17]. The harmony of the rider and the horse working together sometimes disappears, making it possible to observe conflict behaviour of the ridden horse (drawn back ears, pulling reins, head shaking, frequent tail swishing etc.) [2, 18]. In show-jumping, pulling the reins out of the rider’s hands was the most frequent conflict behaviour [18]. The level of discomfort in the horse, in terms of behavioural as well as physiological (saliva cortisol, heart rate) responses, depended on the riding style of an individual rider [19] but also on the number of riders working with a particular horse [20]. As it was found the lower number of riders working with a particular horse produced the less severe stress response (adrenal reaction) compared to a horse with more than one rider [20]. Future studies are needed to fully understand these relationships in sport athletes.

Only a few studies systematically analysed show-jumping performance and the effects of jumping course, obstacle type and further factors during competitions. Until now, the performance analyses in show-jumping have only been conducted twice [21, 22]. The preliminary results of Marlin and Williams [21] suggested a non-random distribution of faults in elite show-jumping. This outcome agrees with the previous study where horses most frequently only dropped some types of obstacles in competition. Walls or triple bars were the most often run-out while the vertical obstacles and oxers were the most frequently dropped obstacles during regional show-jumping competitions [22]. In a previous study [21], faults were distributed across all fence types, however a similar quantity of faults was found on vertical obstacles (49%), as well as within combination fences (41%). In some competitions, horses may encounter obstacles with water trays and it is known that horses are also presumed to be fearful of water, therefore the presence of water trays under the obstacle may further complicate jump estimation [23]. According to the rank of obstacle faults, horses were 9 times more likely to fault at the 9^th^ to 14^th^ obstacles [21] in course of the competition. However, on the regional level, the 3^rd^ and 4^th^ obstacles in the course were the faultiest [22].

The study by Marlin and Williams [21] only analysed the second round of 10 outdoor competitions in the FEI Nations Cup 2017 (1.30/1.60 m obstacle height), meanwhile that of Stachurska et al. [22], only observed classes under 1.40 m height of obstacles in 9 arenas. The performance analyses of the regional level showed that increasing the height of the obstacles from 1.00m to 1.40 m lead to more knocked-down obstacles [22]. Therefore, analyses the performance of the regional level showed that from 1.00 to 1.40mlead to more knocked-down obstacles [22]. Therefore, analyse of performance on the top level competition in show-jumping in the first round and second round in indoor arenas was missing. According to the pre-set course of the competition, a horse either approaches the obstacle straight (from the straight line of the course) or from the left or right arc. The non-straight approach angle from the previous fence induced at higher chance of faults in competition compared to the straight approach [21]. The pre-set course of the competitions determines whether the horse can see the obstacle first with their eye and a limited view using the right eye, and vice versa. In a species with laterally positioned eyes, such as a horse, the impulses of visual input coming at a certain angle only from the right or left eye may employ lateralization, i.e. this phenomenon that a preferential use of the left or right visual field [24]. It was observed that horses fixate a novel stimulus with their left eye to a contralateral hemispheric assessment of each eye´s visual input, processed by the right hemisphere of the brain (associated with new/unexpected, fear and flight reaction) [25] while the left hemisphere of the brain is always associated with control of routine tasks and learning [24].

The present study aimed to determine key factors that affect the show-jumping performance in top sport horses, in that participants (horse-rider pairs) are intensely selected based on their previous successful career. The tested factors were horse/rider’s age and gender, experience in the competition, as well as course-based characteristics (obstacle type, jump order, single obstacles vs. combinations). Due to the fact that mistakes are a disqualifying reason for the competitors, it is very desirable for riders and trainers to know a possible risk factor for higher fault rate in competitions. According to the general belief of trainers and riders, we hypothesised that the competitors (riders and horses) will make mistakes more often on jumps within a combination compared to the single obstacles. This is likely due to a more difficult estimation of the take-off location and the requirement for an adequate tempo to get over the set of two or three fences in line. We also hypothesized that even top horses more often make mistakes on jumps with water trays because they appear less often within a course than other obstacle types and/or given that water trays can make an assessment of a jump trajectory more difficult. We also focused on how the probability of fault was affected by the direction of approach in which the obstacle was jumped. In a highly variable environment of top competition, horses should fail more often on jumps coming from the right than the left arc due to slower and less precise evaluation of the take-off location and jumping trajectory because the obstacle is visible only by the right eye during a portion of the approach (left hemisphere specialization). Further, the effects of time and speed were investigated during the jump off round of the competition to understand if the higher speed induced more faults given that it may compromise precise jump estimation.

## Materials and methods

### Ethics approval

Approval of the Institutional Animal Care and Use Committee of the Institute of Animal Science in Prague was not required as a public medium (FEI TV, FEI website) was used for the present study.

### Competitions

The data were collected within the Western European League (league), a part of Longines FEI World cup (season 2017/2018), covering 13 different cities around Western Europe (Oslo, Helsinki, Verona, Lyon, Stuttgart, Madrid, La Coruna, London, Mechelen, Leipzig, Zurich, Bordeaux, Göteborg). Competitions were of the CSI5* level, ran under the FEI rules, held indoors and consisted of the first round and jump off round. Courses were required to contain single obstacles and combinations of various types, such as vertical, spread, vertical or spread with water trays, and heights with 1.40–1.60 m. Combinations are defined as a group of two or more obstacles with a distance between the fences of double, treble or higher combinations at least 7 m, with a maximum of 12 metres. The vertical obstacles consist of from vertically located poles while the spread obstacles are built to require a jump effort, both in spread and in height. Spread obstacles must not exceed 2.00 m in spread with the exception of triple bars (maximum spread of 2.20 m). The obstacle with water trays involves a water element that can be placed either under a vertical or spread obstacle. The jump off round must contain at least 6 obstacles (combinations count as one obstacle) [2].

In league, first f rounds involved 15–17 jumps while jump offs involved 8 to 10 jumps. Show-jumping courses were built by professional course designers to differentiate as much as possible within the league, i.e. in terms of arrangement and colour of obstacles or decorations around the course. Knocked down obstacles, refusals and run-outs were both classified as ‘faults’ with a cost of 4 penalties for the purposes of this study. The minimum overall speed in the course and the time limit were set up for each competition according to the FEI rules [2]. See S1 Table for details.

### Type of obstacles

The obstacles were arranged either as single obstacles (n = 227, 70.94%), double combination (n = 54, 16.88%) or triple combination (n = 39, 12.19%), consisting of a set of two or three fences in line at a distance of 7 m to 12 m. No triple combinations were included within the jump off courses, however, double combinations were in courses. Individual fences ranging in height from 1.40 m to 1.60 m were generally categorized as a spread (“oxers”, n = 168) or vertical (n = 152). Spread obstacles were further classified as oxers or triple bars. Oxers are obstacles which combine two vertical fences with a distance between them while a triple bar has three components (ascending). Oxers can either be ascending or parallel and the width varies as well as height. Oxers require an effort both in the spread and in height. According to the competition rules, the maximal width of obstacles was 2.00 m in oxers, 2.20 m in a triple bar and 4.50 m in water obstacles. Vertical obstacles were either simple vertical poles or planks placed directly above each other, or “a wall” constructed from light material which can easily fall when knocked [2]. The water trays under the poles were present in 9.53% of obstacles.

In total, there were 320 obstacles either spread (51.70%) or vertical (48.30%), that were further divided into 6 types for the purpose of analyses: simple oxer (44.78%), oxer with water (4.91%), triple bar (2.02%), simple vertical (41.36%), vertical with water (4.63%) or wall (2.30%). The obstacles were further categorized as approached from the left arc (n = 3732), right arc (n = 3782) or from the straight line (n = 1600) with at least 3 canter strides in a row on the straight line after the previous fence. During the outdoor competitions that were analysed in study by Marlin and Williams [21], there were 4 or more strides following on a previous fence or after a turn classified with a straight approach. The Western European League was organised in indoor arenas with shorter courses and a smaller range of arena, therefore, we set up our study as 3 strides on straight line.

### Competitors

In total, 645 show-jumping performances (504 in first rounds, 141 in jump offs) were analysed. Information about the performance of each rider and horse was obtained from video records and the FEI website (https://inside.fei.org/). In total, 144 riders with mean age 38.06 ± 9.74 years (mean ± SD, range: 20–63 years) participated in the observed competitions. Each rider was allowed to compete with more horses during the entire league season but only with one which was within the relevant competition. Riders differed in so-called Longines ranking, an index of success calculated by the FEI from the number of starts and wins in different competitions (starts: 33–2861 / wins: 0–366). The variables describing the riders’ experience (number of previous starts, number of wins, Longines ranking) were correlated. Finally, the number of previous starts was selected for further analyses according to its normal distribution (Kolmogorov-Smirnov test, PROC UNIVARIATE, SAS).

Altogether, 222 horses (63 mares, 59 stallions, 100 geldings) consisting of 22 breeds (mainly Belgian Warm Blood) and by the rule, older than 7 years, attended the competitions. The age of the horses ranged between 9 and 18 years (12.08 ± 1.88 years old). The horses experienced between 5 and 368 previous competitions from which they won in range of 0 to 29. The number of penalty points did not increase during the series of competitions (S1 Fig) ensuring that all rounds of qualifiers were suggested to be equally challenging for the competitors.

### Data analysis

Statistical analyses were performed using SAS System software (version 9.4, SAS Institute Inc. Cary, USA.). A generalized linear mixed model (GzLMM) for categorical data analysis (binomial distribution, link function = logit) was fitted for first rounds and the other for jump offs. The GLIMMIX (SAS) procedure was used to determine whether tested factors affected the probability of a fault on the obstacle. The following fixed effects relating to the course of the competition, the characteristics of the riders and horses, or their common performance entered the model: the rank of the fence within the course, type of fence (vertical, vertical with water, oxer, oxer with water, triple bar, wall), the rank of the fence within an obstacle (single obstacle, first or second fence in double combination, first, second or third fence in triple combination), the direction of the approach (from the left arc or the right arc, or straight in the case that the single obstacle or the first fence were in combination), rider’s age (years), gender (male, female), experience (number of previous starts), horses age (year), sex (stallion, gelding, mare), number of previous starts, speed in the competition (m/s). The horse-rider pair and particular competition entered the model as random factors to account for possible repeated measures on the same individuals across the period of the league circuit. From the initial model that included the tested effects, the non-significant effects (P < 0.05) were subsequently dropped. Within-group least squares means were calculated for classes of tested categorical variables (LSMEANS statement) and differences between them were appropriately adjusted for multiple comparisons applying the Tukey–Kramer adjustment.

The relationship between continuous variables (speed in the first round and jump off) was assessed via the Pearson correlation coefficient (PROC CORR, SAS). Comparison of the speed in the first round and jump off was tested with a general linear mixed model (GLMM) on 118 horse-rider pairs that finished the jump off, speed was included as a dependent variable, the round (first round or jump off) was a fixed factor and the identity of a rider-horse pair and the competition of interest as were random factors (PROC GLIMMIX, SAS; distribution = normal, link function = identity). Another GLMM with the same random effect was fitted to evaluate the effects of tested factors on the speed in jump offs. The fixed effects were age, gender and experience (number of previous starts) of the rider and age, sex and experience of the horse.

## Results

We analysed 13 competitions of the 2017/2018 Western European League season where 9114 jumps were performed. 144 riders and 222 horses participated and jumped over 320 obstacles within 504 starts in first rounds of competitions and 141 starts in jump offs. The overall rate of faults was 7.85% (8.12% in the first round, 6.08% in the jump off). The effects of the tested factors in the first round and jump off described in the following paragraphs.

### First rounds of competition

**Fig 1:**
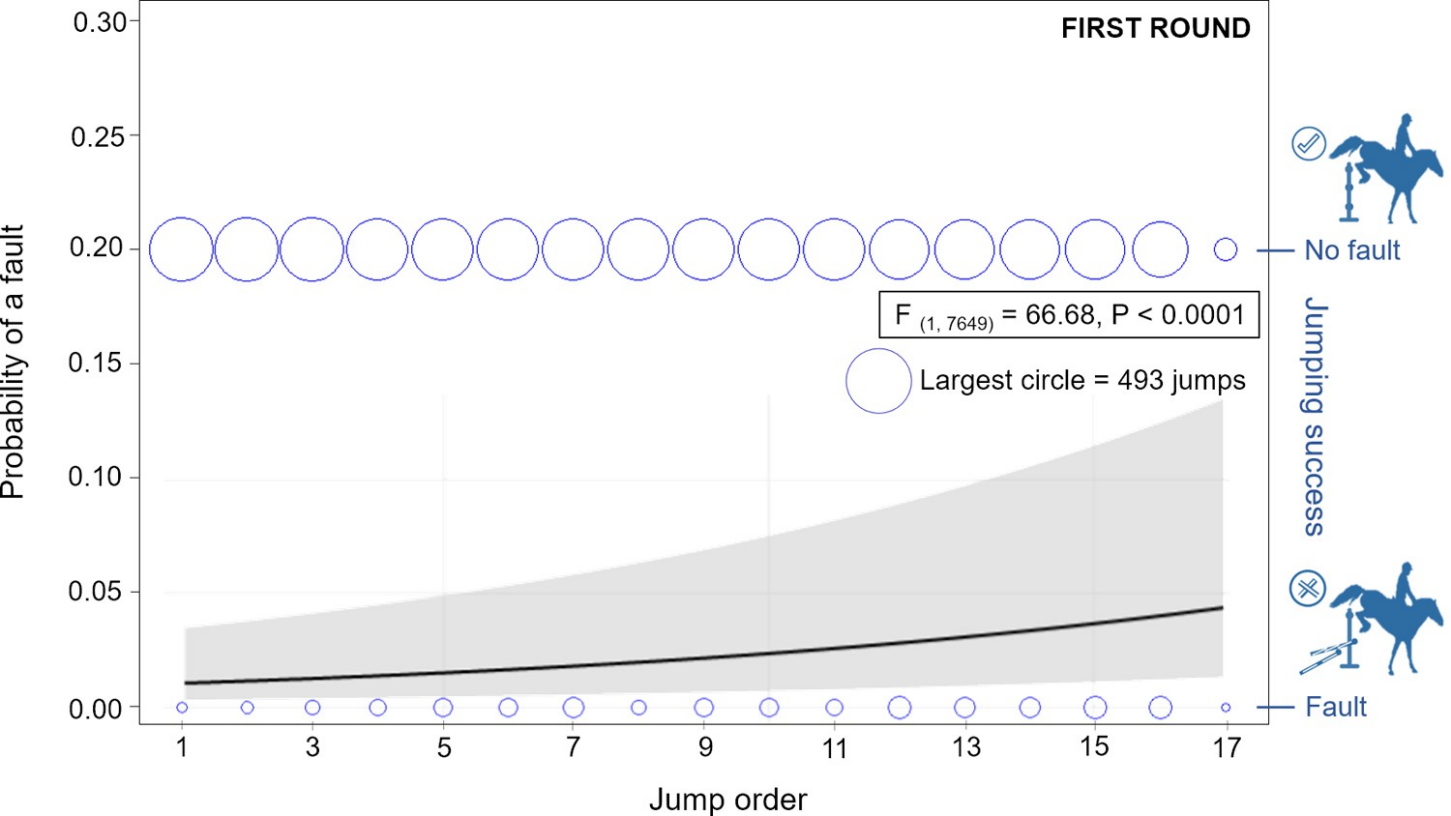
Predicted probability of a fault according to the jump order within the course of the first round. Predicted probability of a fault according to the jump order within the course of the first round (GzLMM, PROC GLIMMIX, SAS). The line and grey field show the predicted probability of a fault ± 95% confidence limit. Two rows of circles represent raw data of numbers of faults (bottom row) or successful jumps (upper row) on the fences. Circle size reflects the number of observations on a particular fence.

**Fig 2:**
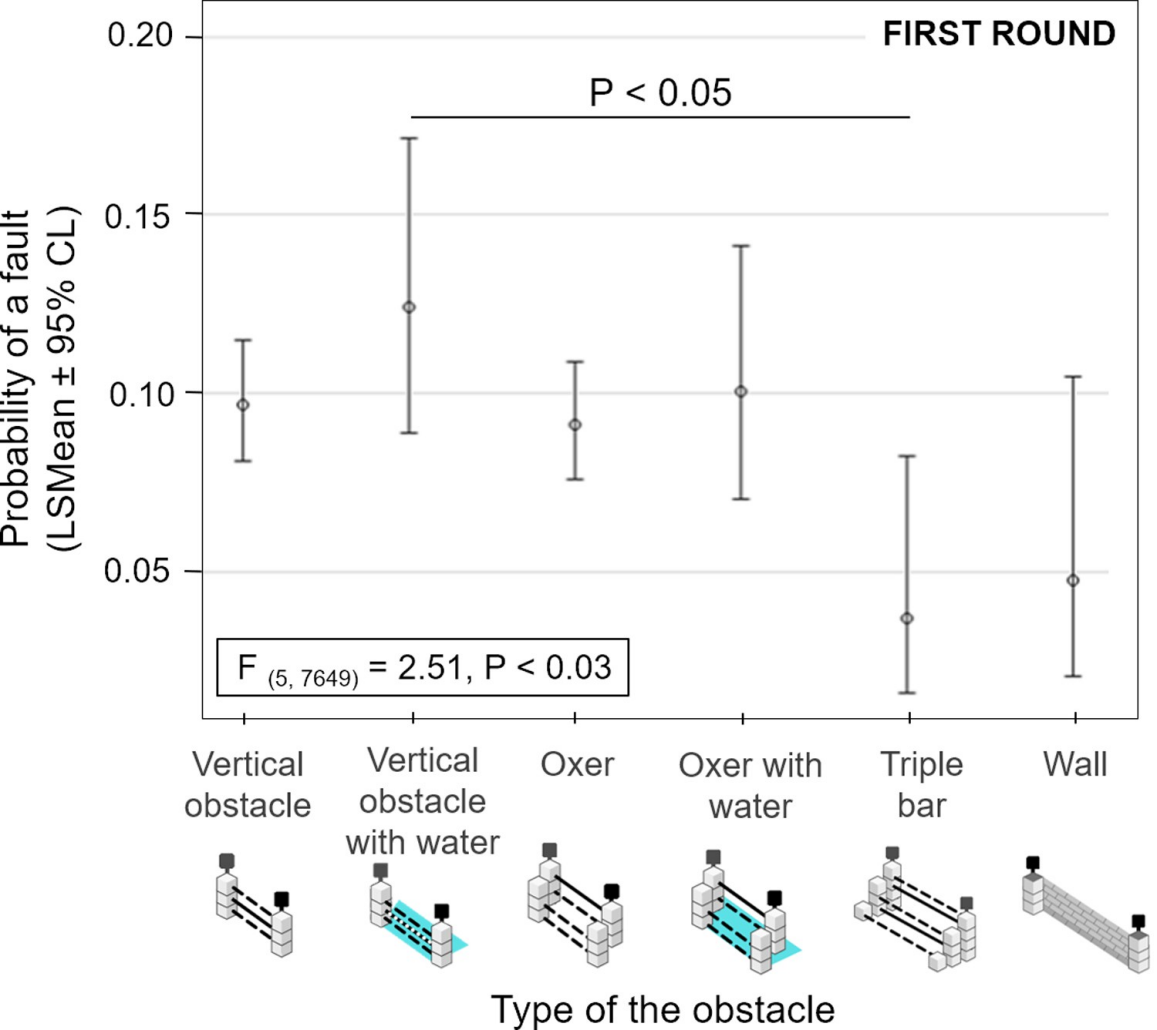
Predicted probability of a fault on different types of obstacles. Predicted probability of a fault on different types of obstacles in the first round (mean values ± 95% confidence limit, GzLMM, PROC GLIMMIX, SAS).

The probability of a fault in the first round of competitions varied based on the rank of the fence within the course (F _(1, 7649)_ = 66.68, P < 0.0001, GzLMM, PROC GLIMMIX, SAS); the later in the course, the higher the probability of failing on the fence (see Fig 1). Certain types of obstacles were associated with a lower probability of a fault (F _(5, 7649)_ = 2.51, P < 0.03). However, only the obstacle with the lowest (triple bar) and highest (vertical with water) predicted probability of fault reached a statistical significance in difference (0.037 ± 0.015 vs. 0.125 ± 0.021, LSMEAN ± standard error; P < 0.05) due to the large individual variability (see Fig 2).

**Fig 3:**
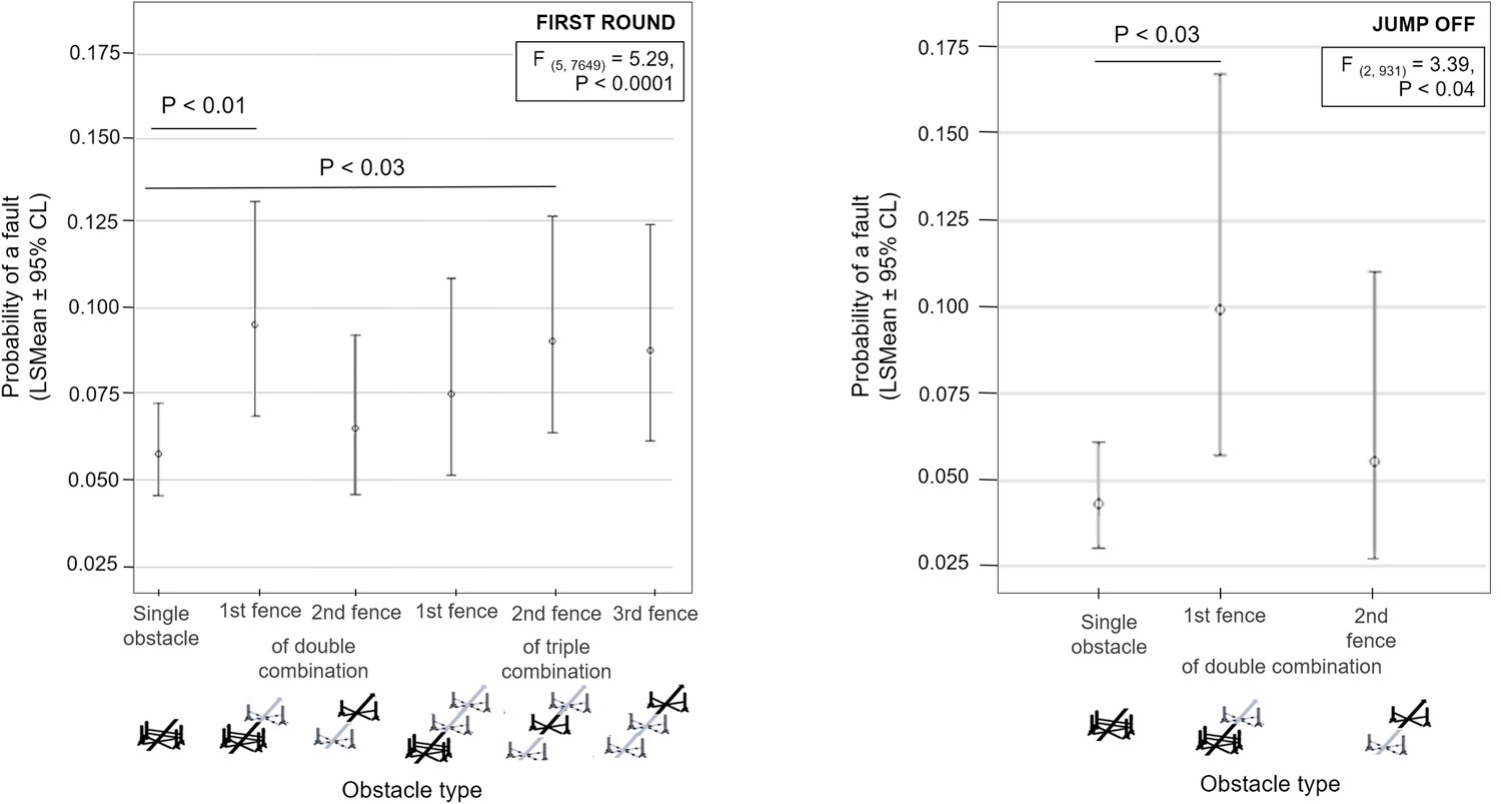
Predicted probability of a fault on single obstacles vs. obstacles in combitation. Predicted probability of a fault on single obstacles vs. obstacles in combitation in the first round (left) and jump off (right, mean values ± 95% confidence limit, GzLMM, PROC GLIMMIX, SAS).

**Fig 4:**
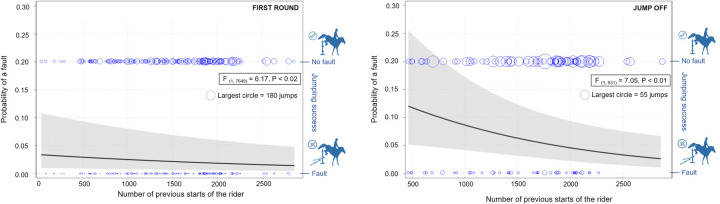
Predicted probability of a fault according to the experience of the rider. Predicted probability of a fault according to the experience of the rider in the first round (left) and jump off (right). The line and grey field show the predicted probability of a fault ± 95% confidence limit. Two rows of circles represent raw data of numbers of faults (bottom row) or successful jumps (upper row) corresponding to the number of previous starts of the rider. Circle size reflects the number of observations on a particular number of the riders’ previous starts.

**Fig 5:**
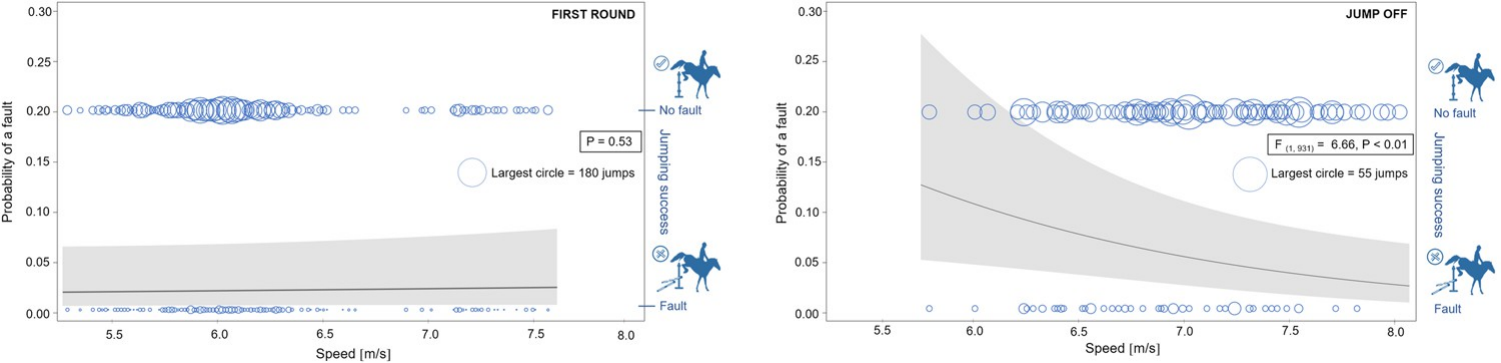
The probability of a fault in relation to the speed. The probability of a fault in relation to the speed in the first round (left) and jump off (right). The line and grey field show the predicted probability of a fault ± 95% confidence limit. Two rows of circles represent raw data of the numbers of faults (bottom row) or successful jumps (upper row). Circle size reflects the number of observations corresponding to the speed.

The jumping success also differed according to the rank of the fence within the obstacle (F _(5, 7649)_ = 5.29, P < 0.0001, see Fig 3 (left). The least probability of a fault was found on single obstacles (0.057 ± 0.007) while the highest was found on the first fence of double combinations (0.095 ± 0.016). The experience of the rider was also significant (F _(1, 7649)_ = 6.17, P < 0.02) in the first round of competitions. The more starts the rider absolved in his/her carrier led to a decrease of the probability of a fault (see Fig 4, left). Other factors like sex of the horse and the rider, as well as horse experience, did not have an impact on show-jumping performance. In the first rounds, the probability of a fault was not significantly influenced by the speed (P = 0.53, see Fig 5, left) or the direction of approach to the obstacle (P = 0.14) (S2 Fig).

### Jump offs

The type of the obstacle, the direction of the approach and the number of previous starts of the horse had an influence on the probability of a fault in the jump off. However, the number of previous starts of the rider (F _(1, 931)_ = 7.05, P < 0.01), speed (F _(1, 931)_ = 6.66, P < 0.01) and the rank of the fence within obstacle (F _(2, 931)_ = 3.39, P < 0.04) were influential for this portion of the competition. The rank of the fence within the course was not a significant factor for the jump offs, but it did improve the quality of the statistical model (r = 0.1, F _(1, 931)_ = 3.12, P = 0.08). In jump offs, t probability of a fault was higher on the first fence within a double combination (0.043 ± 0.008) or second fence within a double combination (0.055 ± 0.020) compared to the single fence (0.100 ± 0.027; see Fig 3, right). The probability of a fault decreased as the experience of the rider increased (Fig 4, right) as well as the probability of a fault decreased with higher speed (Fig 5, right). As expected, the horses ran faster in jump offs (7.10 ± 0.08 m/s; range 5.76–8.08 m/s) compared to first rounds (6.12 ± 0.08 m/s; 4.52–7.59 m/s; F [26] = 546.10, P < 0.0001). No significant correlation was found between the speed in the jump off and the first round in individual horses (Pearson correlation coefficient: r = 0.26, P < 0.01). The speed reflected different competition rules and/or tactics of the rider in first rounds and jump offs. It did not reflect a general characteristic of a horse.

The speed in the jump off differed according to the sex of the horse (F _(2, 25)_ = 4.98, P < 0.02). It subtly increased with the number of previous starts of the horse (r = 0.002, F _(1, 25)_ = 12.99, P < 0.001). The geldings ran slightly slower (6.98 ± 0.12 m/s) compared to mares (7.24 ± 0.12 m/s) or stallions (7.14 ± 0.14 m/s) but the difference to mares was the only speed variation that reached statistical significance (P < 0.02). There were also large individual differences among horses. Neither gender (P = 0.92) nor experience of the rider (P = 0.29) had an effect on the speed.

## Discussion

The results of the study show that faults in the first round and jump off of elite show-jumping competitions were not randomly distributed. They were driven by different factors in each of the two rounds of a show-jumping competition. Factors of general influence were the experience of the rider and whether the fence was a single obstacle or part of the combination. In both, first round as well as in jump off, more faults were found on fences within combinations than on single obstacles, especially on the first fence of the combination. The obstacle type was only relevant in the first round. The highest probability of a fault was found on vertical obstacles including water trays, while the lowest was found on triple bars or walls. In the jump off, the probability of a fault decreased with the higher overall speed.

The riders that had previously passed more competitions reached fewer faults in both first rounds jump offs. Riders’ experience could be associated with a better estimation of jump effort and easier adaption to various courses of competitions [22], as well as with a better-balanced position during the jump compared to the less experienced riders [27]. More studies focused on the effect of the rider´s experience on show-jumping performance are needed to deeply understand the development of the skills of the rider. Experience and education also reduce the risk of rider injury [28] which is especially important in the higher level of equestrian competition associated with increased risk of injury [29].

Other factors, such as the height, number and type of the obstacles, arrangement of fences, approach line, horse age and breed or rider impact were associated earlier with faults in show-jumping competitions [21, 22]. This suggests that faults are driven by various factors in different kinds and difficulty of competition, including different lengths of the course, the surface of the arena, the location of competitions (outdoors/indoor) or psychological factors such as rivalry of the riders.

In the Western European League, the portion of world championship that was analysed in the present study, horses failed (dropped the pole or refused to jump) in 7.85% of the 9114 jumps at heights ranging from 1.40 m to 1.60 m. Higher fault rates (11.22% up to 18.69%) occurred during the 609 rounds of competition at the regional level up to 1.40 m in 5639 jumps [22]. Another study reported 6.4% of faults but only analysed 2^nd^ rounds of European FEI Nations Cup 2017 and jumps within combinations [21].

In our study, the later order of the fence within the course of the competition increased the probability of a fault in the first round. This probability also tended to increase in the jump off which corresponds to the results of a knocked-down fence 2.8 times more often during the second half of the course during the 2^nd^ round of European FEI Nations Cup [21]. On the other hand, in the regional competitions, the 3^rd^ and 4^th^ fences within the course elicited the most (over 20%, frequency not specified) knock-downs and run-outs while the 1^st^, 10^th^ and other later fences caused the least faults [22]. These findings may reflect the aim of the course builders to put easier and more comfortable jumps at the end of the lower level competition courses to motivate horses to perform further.

The aim of course designers in the show-jumping series is to ensure that the competitions are on the same technical level, but contain a specific arrangement of decorations and types of obstacles [2]. This is to ensure that the attention of the audience is on the horse-rider pair, however, it is also important that the course is aesthetically pleasing for the public. The vertical obstacle with water trays has often been reported as being the most susceptible to faults [23, 29]. The water element was still challenging even for the top sport horses in our study, either when accompanying vertical obstacles or oxers. Various reasons could cause the increase in faults on the obstacles with water trays, such as the fear elicited by the shadow of water, complicated jump estimation [23, 29], the obstacle´s contrast with the surface of the arena, or the colour of fences [22].

A crucial question among trainers and riders has been, what type of obstacle is the most difficult for horses? Vertical obstacles generally seem to be the most prone to failure on the regional level [22], in the European FEI Nations Cup [21] and in our study. It was discovered that the higher number of knock-downs was on vertical obstacles and oxers [22], according to the FEI rules [2], oxers can either be ascending, descending or parallel and the width varies as well as height, these factors could also be influential to performance. However the most common run-outs (but not knock-downs) were on the walls [22]. This is not necessarily contradictory to our results that the least penalty points were scored at walls (and triple bars). Walls may draw attention and/or make the jump estimation easier in well trained top horses while less experienced horses may fear and refuse to jump them. Walls and triple bars were suggested to be easier for jumping but they require a good trajectory and higher speed for the successful jump approach compared to the other types of obstacles [30]. Only one run-out on the triple bar (from 5639 jumps) was recorded during the regional competitions in the previously mentioned study by Stachurska et al. [22].

Among studies, failure occurrence also differs on jumps over single or combined obstacles. In the present study observing the world class competition, the most faults appeared on the first fence within the double combination (13.02% jumps over fences of this order failed) while the easiest was a single obstacle (6.65%). Higher fault rate on fences within a combination (41%) was also recorded in the European FEI Nations Cup [21]. On the regional level, however, the least faults were recorded on the 2^nd^ fence in combinations while the most (over 20%) occurred on the 3^rd^ fence in combination [22]. Single obstacles and the 1^st^ fences within combinations were almost similarly difficult in the latter study. Higher competition levels include more jumps in combinations, which probably require higher muscular effort and a higher rapid rate of energy from a horse to finish clear [30].

There was no link between the overall speed and the probability to make a fault in the first round. Meanwhile, the probability of faults decreased with increasing speed in the jump off. Thus, high speed thus likely did not compromise precise jump estimation, and/or even enabled bigger jumps in the decisive phase of the competition. The relationship between overall speed and faults in jump offs is inherent to competition so that a better time results in a better final placing in the competition. Nevertheless, the speed during the competition is not constant, therefore, the overall speed does not necessarily correspond to the actual speed at the moment of approaching the obstacle. The speed may also differ in certain parts of the course due to the riding tactic or because of how the course of the ride goes. Detailed investigation of the speed throughout the ride should be addressed in the future.

Horses ran on average 1 m/s faster in jump offs compared to the first rounds (7.03 ± 0.49 m/s vs. 6.09 ± 0.43 m/s, respectively). The speed in the first round was slightly higher than recommended time for the first round of indoor competitions which was 5.83 m/s (350 m/min) [2]. The higher speed can be due to the physical predisposition of the horse and the readiness of the horse to compete in the elite level of show-jumping. Higher speed can also result from a desire to win competitions and a good quality of training [31], as well as the mental state which is closely connected to communication between the rider and the horse [17, 27].

The present study did not find a significant correlation between the speed in the jump off and the first round that would be expected when the certain pace was a general trait in a horse. In the jump offs, the mares ran slightly faster than geldings and stallions. The mares’ performance was suggested to be strongly dependent on the season, especially in March [32], when a mare´s hormone profile can affect the overall level of focus and cooperation with rider [33]. March, as springtime in general, did not alter performance of the mares in our study either way as the competition ran from October to February. Possible sex differences and their interaction with the jumping success should be an area of focus in further studies.

There were no seasonal differences between the events of the Western European League (October 2017—February 2018). We expected more failures at the beginning, when horses are not yet accustomed to various indoor stimuli, as well as at the end of the series due to the increased exhaustion and energy depletion of both horses and riders. Nevertheless, all rounds of qualifiers were found to be equally challenging for the rider-horse pairs. This result most likely reflects the even level among the competitors and a great condition that they can keep throughout the whole league. Horses at this level of competition are also well prepared to cope with possible seasonal climatic effects as well as various jumping environments. It is likely a well-developed system of qualifiers combined with experience of the course designers that deliver courses of similar difficulty.

A special focus of this study was devoted to the effects of lateralization but it brought disappointing results. On the population level, there was no link between failures and the direction of an approach to the obstacle. Our expectation that horses will fail more often on jumps coming from the right than the left arc because of the lateralization [24], was not proven. The horses should be cautious and vigilant, given that they enter the state connected with the “alert” right hemisphere activation (left eye) in order to actively cope with the environment of competition. Meanwhile left brain hemisphere is more connected with “routine”. However, experience and level of training of the horses participating in the Western European League could explain the lack of faults related to laterality. A horse rider pair that is in side preference balance would appear to have an advantage over one-sided pairs in competitions [34]. It is known that a horse´s inherent laterality preference is commonly overridden by the rider. Experienced riders can reduce the asymmetrical manner in horses by equally strengthening muscles on both sides, same as the horse´s reactions. Meanwhile experienced riders might unintentionally enhance the horse’s laterality [35]. The fault rate at this level of competition seems to be more related to angles and biomechanics [21, 35].

Therefore, the ability to jump successfully from all directions was likely part of the complex traits that qualified that observed pairs for the best competition containing several dozen from 35 563 registered show jumpers (FEI database 2021). Top level athletes should be trained according to sport specific requirements [36], so that some behavioural tendencies, such as physical or brain lateralization, maybe overridden by training. Also, current literature provides sparse and opposing results. The fewer knock-downs were found on obstacles with a straight line approach (13.65 ± 1.88; LSM ± S.E.) compared to the ones in the corner (16.29 ± 2.14; LSM ± S.E.) on the regional competition level [22], while there were more faults on the straight lines of approach (7.9%) compared to the jumps approached from the side (3.8% to 6.2% according to the angle of the approach) during the European FEI Nations Cup competition [21]. Further investigation of lateralization as well as body asymmetry assessment should be done.

Other variables such as the age of the horse and rider, breed, sex of the horse or gender of the rider, horse experience or direction of approach to the obstacle did not affect the performance and success of the horse-rider pairs in first rounds or jump offs. The identification of main course-based factors influencing the show-jumping performance of top sport horses may help to improve the performance and safety when applied to the appropriate training focus and an adequate training plan for sport horses.

### Highlights and limitations of study

The present study identified 4 key factors that significantly affected the fault rate during one series of show-jumping competitions for top sport horses. Faults were driven by different factors in the first rounds compared to jump offs and they were not randomly distributed. It is beyond the scope of this study to address the question of behaviour such as conflict behaviour of the horse and the behaviour of the rider towards the horse’s reactions, especially during challenging situations within the show-jumping course. Due to the lack of information about motor lateralization, body asymmetry of each elite horse and the handedness in riders, the results cannot fully assess the effects of lateralization. The lack of evidence of lateralization might have resulted from the overemphasized selection of the kind of competition driven by the minimisation of hardly tangible confounding effect. In this study, we only analysed overall speed for the rider and the horse in the round, however, a closer look into the speed in particular parts of course could be interesting. For example, an investigation of how the speed differs at the first quarter and the last quarter of the course. Several questions remain unanswered at present. However, the identification of the main course-based factors influencing the show-jumping performance of top sport horses can help trainers set an adequate training plan for sport horses by focusing on special obstacles to lower the fault rate as well as to increase safety of the horse-rider pair.

## Conclusion

Show-jumping is one of the most popular equestrian disciplines where the general aim of competitors is to achieve the fewest faults in a limited time within a course. Therefore, the data of 645 performances (144 riders, 222 horses) during the Western European League (2017/2018) were analysed. The results showed that faults were not randomly distributed, and that performance success depended on different factors according to the type of round. This new information can help the riders and trainers focus on more complicated jumps during training. The results can also help the course designers create competitions of varying difficulties.

## Supporting information

S1 Table Characteristics of competitions. (DOCX)

S2 Table Data set. (PDF)

S1 Fig Distribution of the mean score of penalty points. Distribution of the mean score of penalty points acquired by a rider-horse pair in particular qualifiers throughout the league. The central line in a box plot indicates the median, while the edges of the box indicate the first and third quartiles. Little circles beyond the whiskers are the outliers.
(TIF)

S2 Fig Predicted probability of a fault according to the direction of approach. Predicted probability of a fault according to the direction of approach to the obstacle (LS MEANS ± 95% confidence limit).
(TIF)
