# Supplementary material for: Still beyond a chance: Distribution of faults in elite show-jumping horses
Source: PLoS One. 2022 Mar 16;17(3):e0264615. doi: 10.1371/journal.pone.0264615 (PMC8926200; doi:10.1371/journal.pone.0264615)
Supplement: S1 Table — (DOCX) [file pone.0264615.s001.docx]

Supplementary material

**S1 Table Characteristics of competitions.**

| Date | City | First round | | | Second round (Jump off round) | | | |
| --- | --- | --- | --- | --- | --- | --- | --- | --- |
|  |  | Competitors | Course length (m) | Obstacle number | Competitors | Course length (m) | | Obstacle  number |
| 12.-15.10.2017 | Oslo | 38 | 410 | 16 | 7 | 295 | 9 | |
| 19.-22.10.2017 | Helsinki | 40 | 450 | 17 | 9 | 270 | 8 | |
| 26.-29.10. 2017 | Verona | 38 | 465 | 16 | 11 | 290 | 8 | |
| 1.-5.11.2017 | Lyon | 40 | 420 | 16 | 11 | 280 | 8 | |
| 15.-19.11.2017 | Stuttgart | 40 | 410 | 16 | 16 | 380 | 10 | |
| 23.-26.11.2017 | Madrid | 40 | 430 | 16 | 18 | 300 | 9 | |
| 8.-10.12. 2017 | La Coruna | 40 | 490 | 17 | 8 | 285 | 8 | |
| 12.-18.12.2017 | London | 36 | 400 | 16 | 4 | 280 | 8 | |
| 26.-30.12.2017 | Mechelen | 40 | 380 | 16 | 7 | 290 | 9 | |
| 18.-21.1. 2018 | Leipzig | 40 | 390 | 15 | 15 | 300 | 9 | |
| 26.-28.1. 2018 | Zurich | 39 | 430 | 16 | 14 | 300 | 9 | |
| 2.-4.2.2018 | Bordeaux | 40 | 440 | 16 | 10 | 280 | 8 | |
| 21.-25.2. 2018 | Göteborg | 33 | 420 | 16 | 11 | 300 | 8 | |
| Sum | x | 504 | x | 209 | 141 | x | 111 | |
