# Supplementary material for: Still beyond a chance: Distribution of faults in elite show-jumping horses
Source: PLoS One. 2022 Mar 16;17(3):e0264615. doi: 10.1371/journal.pone.0264615 (PMC8926200; doi:10.1371/journal.pone.0264615)
Supplement: S2 Table — (PDF) [file pone.0264615.s002.pdf]

City	Competition	Date	Class	Level	Height (m)	Course distance (m)	Horse	Horse age	Birth date	Horse sex	Breed	Num_starts	Num_wins	Rider	Longines Ranking	Age	Number of starts in career	Number of wins in career	Sex	Number of jump in course	Number of obstacles in course	Combination	Type	Type specified	Jump approach	Fault	Penalty for jump	Time penalty	Time	Excluded
Oslo	Western European League CSI-W5* round A	15.10.2017	CSI-W5*	4	1.40 - 1.60	410	77	14	04.04.2004	S	BWP	245	19	1	19	38	2803	179	M	1	1	no	spread	oxer	right	yes	4	0	0,00	yes
Oslo	Western European League CSI-W5* round A	15.10.2017	CSI-W5*	4	1.40 - 1.60	410	77	14	04.04.2004	S	BWP	245	19	1	19	38	2803	179	M	2	2	no	vertical	vertical	right	yes	4	0	0,00	yes
Oslo	Western European League CSI-W5* round A	15.10.2017	CSI-W5*	4	1.40 - 1.60	410	77	14	04.04.2004	S	BWP	245	19	1	19	38	2803	179	M	3	3	no	vertical	vertical	left	yes	4	0	0,00	yes
Oslo	Western European League CSI-W5* round A	15.10.2017	CSI-W5*	4	1.40 - 1.60	410	77	14	04.04.2004	S	BWP	245	19	1	19	38	2803	179	M	4	4a	yes	vertical	vertical	straight	yes	4	0	0,00	yes
Oslo	Western European League CSI-W5* round A	15.10.2017	CSI-W5*	4	1.40 - 1.60	410	77	14	04.04.2004	S	BWP	245	19	1	19	38	2803	179	M	5	4b	yes	spread	oxer	straight	no	0	0	0,00	yes
Oslo	Western European League CSI-W5* round A	15.10.2017	CSI-W5*	4	1.40 - 1.60	410	77	14	04.04.2004	S	BWP	245	19	1	19	38	2803	179	M	6	5	no	vertical	vertical with water	straight	yes	4	0	0,00	yes
Oslo	Western European League CSI-W5* round A	15.10.2017	CSI-W5*	4	1.40 - 1.60	410	77	14	04.04.2004	S	BWP	245	19	1	19	38	2803	179	M	7	6a	yes	spread	oxer	right	no	0	0	0,00	yes
Oslo	Western European League CSI-W5* round A	15.10.2017	CSI-W5*	4	1.40 - 1.60	410	77	14	04.04.2004	S	BWP	245	19	1	19	38	2803	179	M	8	6b	yes	vertical	vertical	right	no	0	0	0,00	yes
Oslo	Western European League CSI-W5* round A	15.10.2017	CSI-W5*	4	1.40 - 1.60	410	77	14	04.04.2004	S	BWP	245	19	1	19	38	2803	179	M	9	6c	yes	spread	oxer	right	no	0	0	0,00	yes
Oslo	Western European League CSI-W5* round A	15.10.2017	CSI-W5*	4	1.40 - 1.60	410	77	14	04.04.2004	S	BWP	245	19	1	19	38	2803	179	M	10	7	no	vertical	vertical	right	no	0	0	0,00	yes
Oslo	Western European League CSI-W5* round A	15.10.2017	CSI-W5*	4	1.40 - 1.60	410	77	14	04.04.2004	S	BWP	245	19	1	19	38	2803	179	M	11	8	no	spread	oxer	left	no	0	0	0,00	yes
Oslo	Western European League CSI-W5* round A	15.10.2017	CSI-W5*	4	1.40 - 1.60	410	77	14	04.04.2004	S	BWP	245	19	1	19	38	2803	179	M	12	9	no	vertical	vertical	straight	no	0	0	0,00	yes
Oslo	Western European League CSI-W5* round A	15.10.2017	CSI-W5*	4	1.40 - 1.60	410	77	14	04.04.2004	S	BWP	245	19	1	19	38	2803	179	M	13	10	no	spread	oxer	right	no	0	0	0,00	yes
Oslo	Western European League CSI-W5* round A	15.10.2017	CSI-W5*	4	1.40 - 1.60	410	77	14	04.04.2004	S	BWP	245	19	1	19	38	2803	179	M	14	11	no	vertical	vertical	right	no	0	0	0,00	yes
Oslo	Western European League CSI-W5* round A	15.10.2017	CSI-W5*	4	1.40 - 1.60	410	77	14	04.04.2004	S	BWP	245	19	1	19	38	2803	179	M	15	12	no	spread	oxer	left	no	0	0	0,00	yes
Oslo	Western European League CSI-W5* round A	15.10.2017	CSI-W5*	4	1.40 - 1.60	410	77	14	04.04.2004	S	BWP	245	19	1	19	38	2803	179	M	16	13	no	spread	oxer	straight	no	0	0	0,00	yes
Oslo	Western European League CSI-W5* round A	15.10.2017	CSI-W5*	4	1.40 - 1.60	410	107	11	25.02.2007	S	BWP	196	13	2	7	31	2168	92	M	1	1	no	spread	oxer	right	no	0	0	0,00	yes
Oslo	Western European League CSI-W5* round A	15.10.2017	CSI-W5*	4	1.40 - 1.60	410	107	11	25.02.2007	S	BWP	196	13	2	7	31	2168	92	M	2	2	no	vertical	vertical	right	no	0	0	0,00	yes
Oslo	Western European League CSI-W5* round A	15.10.2017	CSI-W5*	4	1.40 - 1.60	410	107	11	25.02.2007	S	BWP	196	13	2	7	31	2168	92	M	3	3	no	vertical	vertical	left	yes	4	0	0,00	yes
Oslo	Western European League CSI-W5* round A	15.10.2017	CSI-W5*	4	1.40 - 1.60	410	107	11	25.02.2007	S	BWP	196	13	2	7	31	2168	92	M	4	4a	yes	vertical	vertical	straight	no	0	0	0,00	yes
Oslo	Western European League CSI-W5* round A	15.10.2017	CSI-W5*	4	1.40 - 1.60	410	107	11	25.02.2007	S	BWP	196	13	2	7	31	2168	92	M	5	4b	yes	spread	oxer	straight	yes	4	0	0,00	yes
Oslo	Western European League CSI-W5* round A	15.10.2017	CSI-W5*	4	1.40 - 1.60	410	107	11	25.02.2007	S	BWP	196	13	2	7	31	2168	92	M	6	5	no	vertical	vertical with water	straight	no	0	0	0,00	yes
Oslo	Western European League CSI-W5* round A	15.10.2017	CSI-W5*	4	1.40 - 1.60	410	107	11	25.02.2007	S	BWP	196	13	2	7	31	2168	92	M	7	6a	yes	spread	oxer	right	no	0	0	0,00	yes
Oslo	Western European League CSI-W5* round A	15.10.2017	CSI-W5*	4	1.40 - 1.60	410	107	11	25.02.2007	S	BWP	196	13	2	7	31	2168	92	M	8	6b	yes	vertical	vertical	right	no	0	0	0,00	yes
Oslo	Western European League CSI-W5* round A	15.10.2017	CSI-W5*	4	1.40 - 1.60	410	107	11	25.02.2007	S	BWP	196	13	2	7	31	2168	92	M	9	6c	yes	spread	oxer	right	no	0	0	0,00	yes
Oslo	Western European League CSI-W5* round A	15.10.2017	CSI-W5*	4	1.40 - 1.60	410	107	11	25.02.2007	S	BWP	196	13	2	7	31	2168	92	M	10	7	no	vertical	vertical	right	no	0	0	0,00	yes
Oslo	Western European League CSI-W5* round A	15.10.2017	CSI-W5*	4	1.40 - 1.60	410	107	11	25.02.2007	S	BWP	196	13	2	7	31	2168	92	M	11	8	no	spread	oxer	left	no	0	0	0,00	yes
Oslo	Western European League CSI-W5* round A	15.10.2017	CSI-W5*	4	1.40 - 1.60	410	107	11	25.02.2007	S	BWP	196	13	2	7	31	2168	92	M	12	9	no	vertical	vertical	straight	no	0	0	0,00	yes
Oslo	Western European League CSI-W5* round A	15.10.2017	CSI-W5*	4	1.40 - 1.60	410	107	11	25.02.2007	S	BWP	196	13	2	7	31	2168	92	M	13	10	no	spread	oxer	right	no	0	0	0,00	yes
Oslo	Western European League CSI-W5* round A	15.10.2017	CSI-W5*	4	1.40 - 1.60	410	107	11	25.02.2007	S	BWP	196	13	2	7	31	2168	92	M	14	11	no	vertical	vertical	right	no	0	0	0,00	yes
Oslo	Western European League CSI-W5* round A	15.10.2017	CSI-W5*	4	1.40 - 1.60	410	107	11	25.02.2007	S	BWP	196	13	2	7	31	2168	92	M	15	12	no	spread	oxer	left	yes	4	0	0,00	yes
Oslo	Western European League CSI-W5* round A	15.10.2017	CSI-W5*	4	1.40 - 1.60	410	107	11	25.02.2007	S	BWP	196	13	2	7	31	2168	92	M	16	13	no	spread	oxer	straight	no	0	0	0,00	yes
Oslo	Western European League CSI-W5* round A	15.10.2017	CSI-W5*	4	1.40 - 1.60	410	177	12	26.06.2006	S	SF	139	1	3	97	43	1901	45	M	1	1	no	spread	oxer	right	no	0	0	0,00	yes
Oslo	Western European League CSI-W5* round A	15.10.2017	CSI-W5*	4	1.40 - 1.60	410	177	12	26.06.2006	S	SF	139	1	3	97	43	1901	45	M	2	2	no	vertical	vertical	right	no	0	0	0,00	yes
Oslo	Western European League CSI-W5* round A	15.10.2017	CSI-W5*	4	1.40 - 1.60	410	177	12	26.06.2006	S	SF	139	1	3	97	43	1901	45	M	3	3	no	vertical	vertical	left	yes	4	0	0,00	yes
Oslo	Western European League CSI-W5* round A	15.10.2017	CSI-W5*	4	1.40 - 1.60	410	177	12	26.06.2006	S	SF	139	1	3	97	43	1901	45	M	4	4a	yes	vertical	vertical	straight	no	0	0	0,00	yes
Oslo	Western European League CSI-W5* round A	15.10.2017	CSI-W5*	4	1.40 - 1.60	410	177	12	26.06.2006	S	SF	139	1	3	97	43	1901	45	M	5	4b	yes	spread	oxer	straight	yes	4	0	0,00	yes
Oslo	Western European League CSI-W5* round A	15.10.2017	CSI-W5*	4	1.40 - 1.60	410	177	12	26.06.2006	S	SF	139	1	3	97	43	1901	45	M	6	5	no	vertical	vertical with water	straight	yes	4	0	0,00	yes
Oslo	Western European League CSI-W5* round A	15.10.2017	CSI-W5*	4	1.40 - 1.60	410	177	12	26.06.2006	S	SF	139	1	3	97	43	1901	45	M	7	6a	yes	spread	oxer	right	no	0	0	0,00	yes
Oslo	Western European League CSI-W5* round A	15.10.2017	CSI-W5*	4	1.40 - 1.60	410	177	12	26.06.2006	S	SF	139	1	3	97	43	1901	45	M	8	6b	yes	vertical	vertical	right	no	0	0	0,00	yes
Oslo	Western European League CSI-W5* round A	15.10.2017	CSI-W5*	4	1.40 - 1.60	410	177	12	26.06.2006	S	SF	139	1	3	97	43	1901	45	M	9	6c	yes	spread	oxer	right	no	0	0	0,00	yes
Oslo	Western European League CSI-W5* round A	15.10.2017	CSI-W5*	4	1.40 - 1.60	410	177	12	26.06.2006	S	SF	139	1	3	97	43	1901	45	M	10	7	no	vertical	vertical	right	no	0	0	0,00	yes
Oslo	Western European League CSI-W5* round A	15.10.2017	CSI-W5*	4	1.40 - 1.60	410	177	12	26.06.2006	S	SF	139	1	3	97	43	1901	45	M	11	8	no	spread	oxer	left	no	0	0	0,00	yes
Oslo	Western European League CSI-W5* round A	15.10.2017	CSI-W5*	4	1.40 - 1.60	410	177	12	26.06.2006	S	SF	139	1	3	97	43	1901	45	M	12	9	no	vertical	vertical	straight	no	0	0	0,00	yes
Oslo	Western European League CSI-W5* round A	15.10.2017	CSI-W5*	4	1.40 - 1.60	410	177	12	26.06.2006	S	SF	139	1	3	97	43	1901	45	M	13	10	no	spread	oxer	right	no	0	0	0,00	yes
Oslo	Western European League CSI-W5* round A	15.10.2017	CSI-W5*	4	1.40 - 1.60	410	177	12	26.06.2006	S	SF	139	1	3	97	43	1901	45	M	14	11	no	vertical	vertical	right	no	0	0	0,00	yes
Oslo	Western European League CSI-W5* round A	15.10.2017	CSI-W5*	4	1.40 - 1.60	410	177	12	26.06.2006	S	SF	139	1	3	97	43	1901	45	M	15	12	no	spread	oxer	left	no	0	0	0,00	yes
Oslo	Western European League CSI-W5* round A	15.10.2017	CSI-W5*	4	1.40 - 1.60	410	177	12	26.06.2006	S	SF	139	1	3	97	43	1901	45	M	16	13	no	spread	oxer	straight	no	0	0	0,00	yes
Oslo	Western European League CSI-W5* round A	15.10.2017	CSI-W5*	4	1.40 - 1.60	410	16	10	06.05.2008	G	AES	109	0	4	107	52	1652	70	M	1	1	no	spread	oxer	right	no	0	0	0,00	yes
Oslo	Western European League CSI-W5* round A	15.10.2017	CSI-W5*	4	1.40 - 1.60	410	16	10	06.05.2008	G	AES	109	0	4	107	52	1652	70	M	2	2	no	vertical	vertical	right	no	0	0	0,00	yes
Oslo	Western European League CSI-W5* round A	15.10.2017	CSI-W5*	4	1.40 - 1.60	410	16	10	06.05.2008	G	AES	109	0	4	107	52	1652	70	M	3	3	no	vertical	vertical	left	no	0	0	0,00	yes
Oslo	Western European League CSI-W5* round A	15.10.2017	CSI-W5*	4	1.40 - 1.60	410	16	10	06.05.2008	G	AES	109	0	4	107	52	1652	70	M	4	4a	yes	vertical	vertical	straight	no	0	0	0,00	yes
Oslo	Western European League CSI-W5* round A	15.10.2017	CSI-W5*	4	1.40 - 1.60	410	16	10	06.05.2008	G	AES	109	0	4	107	52	1652	70	M	5	4b	yes	spread	oxer	straight	yes	4	0	0,00	yes
Oslo	Western European League CSI-W5* round A	15.10.2017	CSI-W5*	4	1.40 - 1.60	410	16	10	06.05.2008	G	AES	109	0	4	107	52	1652	70	M	6	5	no	vertical	vertical with water	straight	no	0	0	0,00	yes
Oslo	Western European League CSI-W5* round A	15.10.2017	CSI-W5*	4	1.40 - 1.60	410	16	10	06.05.2008	G	AES	109	0	4	107	52	1652	70	M	7	6a	yes	spread	oxer	right	no	0	0	0,00	yes
Oslo	Western European League CSI-W5* round A	15.10.2017	CSI-W5*	4	1.40 - 1.60	410	16	10	06.05.2008	G	AES	109	0	4	107	52	1652	70	M	8	6b	yes	vertical	vertical	right	no	0	0	0,00	yes
Oslo	Western European League CSI-W5* round A	15.10.2017	CSI-W5*	4	1.40 - 1.60	410	16	10	06.05.2008	G	AES	109	0	4	107	52	1652	70	M	9	6c	yes	spread	oxer	right	no	0	0	0,00	yes
Oslo	Western European League CSI-W5* round A	15.10.2017	CSI-W5*	4	1.40 - 1.60	410	16	10	06.05.2008	G	AES	109	0	4	107	52	1652	70	M	10	7	no	vertical	vertical	right	no	0	0	0,00	yes
Oslo	Western European League CSI-W5* round A	15.10.2017	CSI-W5*	4	1.40 - 1.60	410	16	10	06.05.2008	G	AES	109	0	4	107	52	1652	70	M	11	8	no	spread	oxer	left	no	0	0	0,00	yes
Oslo	Western European League CSI-W5* round A	15.10.2017	CSI-W5*	4	1.40 - 1.60	410	16	10	06.05.2008	G	AES	109	0	4	107	52	1652	70	M	12	9	no	vertical	vertical	straight	yes	4	0	0,00	yes
Oslo	Western European League CSI-W5* round A	15.10.2017	CSI-W5*	4	1.40 - 1.60	410	16	10	06.05.2008	G	AES	109	0	4	107	52	1652	70	M	13	10	no	spread	oxer	right	no	0	0	0,00	yes
Oslo	Western European League CSI-W5* round A	15.10.2017	CSI-W5*	4	1.40 - 1.60	410	16	10	06.05.2008	G	AES	109	0	4	107	52	1652	70	M	14	11	no	vertical	vertical	right	no	0	0	0,00	yes
Oslo	Western European League CSI-W5* round A	15.10.2017	CSI-W5*	4	1.40 - 1.60	410	16	10	06.05.2008	G	AES	109	0	4	107	52	1652	70	M	15	12	no	spread	oxer	left	no	0	0	0,00	yes
Oslo	Western European League CSI-W5* round A	15.10.2017	CSI-W5*	4	1.40 - 1.60	410	16	10	06.05.2008	G	AES	109	0	4	107	52	1652	70	M	16	13	no	spread	oxer	straight	no	0	0	0,00	yes
Oslo	Western European League CSI-W5* round A	15.10.2017	CSI-W5*	4	1.40 - 1.60	410	8	13	15.07.2005	G	KWPN	241	7	5	15	32	2092	63	M	1	1	no	spread	oxer	right	no	0	0	90,74	no
Oslo	Western European League CSI-W5* round A	15.10.2017	CSI-W5*	4	1.40 - 1.60	410	8	13	15.07.2005	G	KWPN	241	7	5	15	32	2092	63	M	2	2	no	vertical	vertical	right	no	0	0	90,74	no
Oslo	Western European League CSI-W5* round A	15.10.2017	CSI-W5*	4	1.40 - 1.60	410	8	13	15.07.2005	G	KWPN	241	7	5	15	32	2092	63	M	3	3	no	vertical	vertical	left	yes	4	0	90,74	no
Oslo	Western European League CSI-W5* round A	15.10.2017	CSI-W5*	4	1.40 - 1.60	410	8	13	15.07.2005	G	KWPN	241	7	5	15	32	2092	63	M	4	4a	yes	vertical	vertical	straight	no	0	0	90,74	no
Oslo	Western European League CSI-W5* round A	15.10.2017	CSI-W5*	4	1.40 - 1.60	410	8	13	15.07.2005	G	KWPN	241	7	5	15	32	2092	63	M	5	4b	yes	spread	oxer	straight	yes	4	0	90,74	no
Oslo	Western European League CSI-W5* round A	15.10.2017	CSI-W5*	4	1.40 - 1.60	410	8	13	15.07.2005	G	KWPN	241	7	5	15	32	2092	63	M	6	5	no	vertical	vertical with water	straight	no	0	0	90,74	no
Oslo	Western European League CSI-W5* round A	15.10.2017	CSI-W5*	4	1.40 - 1.60	410	8	13	15.07.2005	G	KWPN	241	7	5	15	32	2092	63	M	7	6a	yes	spread	oxer	right	no	0	0	90,74	no
Oslo	Western European League CSI-W5* round A	15.10.2017	CSI-W5*	4	1.40 - 1.60	410	8	13	15.07.2005	G	KWPN	241	7	5	15	32	2092	63	M	8	6b	yes	vertical	vertical	right	no	0	0	90,74	no
Oslo	Western European League CSI-W5* round A	15.10.2017	CSI-W5*	4	1.40 - 1.60	410	8	13	15.07.2005	G	KWPN	241	7	5	15	32	2092	63	M	9	6c	yes	spread	oxer	right	no	0	0	90,74	no
Oslo	Western European League CSI-W5* round A	15.10.2017	CSI-W5*	4	1.40 - 1.60	410	8	13	15.07.2005	G	KWPN	241	7	5	15	32	2092	63	M	10	7	no	vertical	vertical	right	no	0	0	90,74	no
Oslo	Western European League CSI-W5* round A	15.10.2017	CSI-W5*	4	1.40 - 1.60	410	8	13	15.07.2005	G	KWPN	241	7	5	15	32	2092	63	M	11	8	no	spread	oxer	left	no	0	0	90,74	no
Oslo	Western European League CSI-W5* round A	15.10.2017	CSI-W5*	4	1.40 - 1.60	410	8	13	15.07.2005	G	KWPN	241	7	5	15	32	2092	63	M	12	9	no	vertical	vertical	straight	no	0	0	90,74	no
Oslo	Western European League CSI-W5* round A	15.10.2017	CSI-W5*	4	1.40 - 1.60	410	8	13	15.07.2005	G	KWPN	241	7	5	15	32	2092	63	M	13	10	no	spread	oxer	right	no	0	0	90,74	no
Oslo	Western European League CSI-W5* round A	15.10.2017	CSI-W5*	4	1.40 - 1.60	410	8	13	15.07.2005	G	KWPN	241	7	5	15	32	2092	63	M	14	11	no	vertical	vertical	right	no	0	0	90,74	no
Oslo	Western European League CSI-W5* round A	15.10.2017	CSI-W5*	4	1.40 - 1.60	410	8	13	15.07.2005	G	KWPN	241	7	5	15	32	2092	63	M	15	12	no	spread	oxer	left	no	0	0	90,74	no
Oslo	Western European League CSI-W5* round A	15.10.2017	CSI-W5*	4	1.40 - 1.60	410	8	13	15.07.2005	G	KWPN	241	7	5	15	32	2092	63	M	16	13	no	spread	oxer	straight	no	0	5	90,74	no
Oslo	Western European League CSI-W5* round A	15.10.2017	CSI-W5*	4	1.40 - 1.60	410	88	13	19.04.2005	S	BWP	131	0	6	421	45	483	7	F	1	1	no	spread	oxer	right	no	0	0	89,42	no
Oslo	Western European League CSI-W5* round A	15.10.2017	CSI-W5*	4	1.40 - 1.60	410	88	13	19.04.2005	S	BWP	131	0	6	421	45	483	7	F	2	2	no	vertical	vertical	right	no	0	0	89,42	no
Oslo	Western European League CSI-W5* round A	15.10.2017	CSI-W5*	4	1.40 - 1.60	410	88	13	19.04.2005	S	BWP	131	0	6	421	45	483	7	F	3	3	no	vertical	vertical	left	no	0	0	89,42	no
Oslo	Western European League CSI-W5* round A	15.10.2017	CSI-W5*	4	1.40 - 1.60	410	88	13	19.04.2005	S	BWP	131	0	6	421	45	483	7	F	4	4a	yes	vertical	vertical	straight	no	0	0	89,42	no
Oslo	Western European League CSI-W5* round A	15.10.2017	CSI-W5*	4	1.40 - 1.60	410	88	13	19.04.2005	S	BWP	131	0	6	421	45	483	7	F	5	4b	yes	spread	oxer	straight	no	0	0	89,42	no
Oslo	Western European League CSI-W5* round A	15.10.2017	CSI-W5*	4	1.40 - 1.60	410	88	13	19.04.2005	S	BWP	131	0	6	421	45	483	7	F	6	5	no	vertical	vertical with water	straight	no	0	0	89,42	no
Oslo	Western European League CSI-W5* round A	15.10.2017	CSI-W5*	4	1.40 - 1.60	410	88	13	19.04.2005	S	BWP	131	0	6	421	45	483	7	F	7	6a	yes	spread	oxer	right	no	0	0	89,42	no
Oslo	Western European League CSI-W5* round A	15.10.2017	CSI-W5*	4	1.40 - 1.60	410	88	13	19.04.2005	S	BWP	131	0	6	421	45	483	7	F	8	6b	yes	vertical	vertical	right	no	0	0	89,42	no
Oslo	Western European League CSI-W5* round A	15.10.2017	CSI-W5*	4	1.40 - 1.60	410	88	13	19.04.2005	S	BWP	131	0	6	421	45	483	7	F	9	6c	yes	spread	oxer	right	no	0	0	89,42	no
Oslo	Western European League CSI-W5* round A	15.10.2017	CSI-W5*	4	1.40 - 1.60	410	88	13	19.04.2005	S	BWP	131	0	6	421	45	483	7	F	10	7	no	vertical	vertical	right	no	0	0	89,42	no
Oslo	Western European League CSI-W5* round A	15.10.2017	CSI-W5*	4	1.40 - 1.60	410	88	13	19.04.2005	S	BWP	131	0	6	421	45	483	7	F	11	8	no	spread	oxer	left	no	0	0	89,42	no
Oslo	Western European League CSI-W5* round A	15.10.2017	CSI-W5*	4	1.40 - 1.60	410	88	13	19.04.2005	S	BWP	131	0	6	421	45	483	7	F	12	9	no	vertical	vertical	straight	yes	4	0	89,42	no
Oslo	Western European League CSI-W5* round A	15.10.2017	CSI-W5*	4	1.40 - 1.60	410	88	13	19.04.2005	S	BWP	131	0	6	421	45	483	7	F	13	10	no	spread	oxer	right	yes	4	0	89,42	no
Oslo	Western European League CSI-W5* round A	15.10.2017	CSI-W5*	4	1.40 - 1.60	410	88	13	19.04.2005	S	BWP	131	0	6	421	45	483	7	F	14	11	no	vertical	vertical	right	yes	4	0	89,42	no
Oslo	Western European League CSI-W5* round A	15.10.2017	CSI-W5*	4	1.40 - 1.60	410	88	13	19.04.2005	S	BWP	131	0	6	421	45	483	7	F	15	12	no	spread	oxer	left	no	0	0	89,42	no
Oslo	Western European League CSI-W5* round A	15.10.2017	CSI-W5*	4	1.40 - 1.60	410	88	13	19.04.2005	S	BWP	131	0	6	421	45	483	7	F	16	13	no	spread	oxer	straight	yes	4	5	89,42	no
Oslo	Western European League CSI-W5* round A	15.10.2017	CSI-W5*	4	1.40 - 1.60	410	20	11	16.04.2007	G	OS	121	0	7	48	58	1857	49	M	1	1	no	spread	oxer	right	no	0	0	72,04	no
Oslo	Western European League CSI-W5* round A	15.10.2017	CSI-W5*	4	1.40 - 1.60	410	20	11	16.04.2007	G	OS	121	0	7	48	58	1857	49	M	2	2	no	vertical	vertical	right	no	0	0	72,04	no
Oslo	Western European League CSI-W5* round A	15.10.2017	CSI-W5*	4	1.40 - 1.60	410	20	11	16.04.2007	G	OS	121	0	7	48	58	1857	49	M	3	3	no	vertical	vertical	left	no	0	0	72,04	no
Oslo	Western European League CSI-W5* round A	15.10.2017	CSI-W5*	4	1.40 - 1.60	410	20	11	16.04.2007	G	OS	121	0	7	48	58	1857	49	M	4	4a	yes	vertical	vertical	straight	no	0	0	72,04	no
Oslo	Western European League CSI-W5* round A	15.10.2017	CSI-W5*	4	1.40 - 1.60	410	20	11	16.04.2007	G	OS	121	0	7	48	58	1857	49	M	5	4b	yes	spread	oxer	straight	no	0	0	72,04	no
Oslo	Western European League CSI-W5* round A	15.10.2017	CSI-W5*	4	1.40 - 1.60	410	20	11	16.04.2007	G	OS	121	0	7	48	58	1857	49	M	6	5	no	vertical	vertical with water	straight	no	0	0	72,04	no
Oslo	Western European League CSI-W5* round A	15.10.2017	CSI-W5*	4	1.40 - 1.60	410	20	11	16.04.2007	G	OS	121	0	7	48	58	1857	49	M	7	6a	yes	spread	oxer	right	no	0	0	72,04	no
Oslo	Western European League CSI-W5* round A	15.10.2017	CSI-W5*	4	1.40 - 1.60	410	20	11	16.04.2007	G	OS	121	0	7	48	58	1857	49	M	8	6b	yes	vertical	vertical	right	no	0	0	72,04	no
Oslo	Western European League CSI-W5* round A	15.10.2017	CSI-W5*	4	1.40 - 1.60	410	20	11	16.04.2007	G	OS	121	0	7	48	58	1857	49	M	9	6c	yes	spread	oxer	right	no	0	0	72,04	no
Oslo	Western European League CSI-W5* round A	15.10.2017	CSI-W5*	4	1.40 - 1.60	410	20	11	16.04.2007	G	OS	121	0	7	48	58	1857	49	M	10	7	no	vertical	vertical	right	no	0	0	72,04	no
Oslo	Western European League CSI-W5* round A	15.10.2017	CSI-W5*	4	1.40 - 1.60	410	20	11	16.04.2007	G	OS	121	0	7	48	58	1857	49	M	11	8	no	spread	oxer	left	no	0	0	72,04	no
Oslo	Western European League CSI-W5* round A	15.10.2017	CSI-W5*	4	1.40 - 1.60	410	20	11	16.04.2007	G	OS	121	0	7	48	58	1857	49	M	12	9	no	vertical	vertical	straight	no	0	0	72,04	no
Oslo	Western European League CSI-W5* round A	15.10.2017	CSI-W5*	4	1.40 - 1.60	410	20	11	16.04.2007	G	OS	121	0	7	48	58	1857	49	M	13	10	no	spread	oxer	right	no	0	0	72,04	no
Oslo	Western European League CSI-W5* round A	15.10.2017	CSI-W5*	4	1.40 - 1.60	410	20	11	16.04.2007	G	OS	121	0	7	48	58	1857	49	M	14	11	no	vertical	vertical	right	no	0	0	72,04	no
Oslo	Western European League CSI-W5* round A	15.10.2017	CSI-W5*	4	1.40 - 1.60	410	20	11	16.04.2007	G	OS	121	0	7	48	58	1857	49	M	15	12	no	spread	oxer	left	no	0	0	72,04	no
Oslo	Western European League CSI-W5* round A	15.10.2017	CSI-W5*	4	1.40 - 1.60	410	20	11	16.04.2007	G	OS	121	0	7	48	58	1857	49	M	16	13	no	spread	oxer	straight	no	0	1	72,04	no
Oslo	Western European League CSI-W5* round A	15.10.2017	CSI-W5*	4	1.40 - 1.60	410	125	16	07.05.2002	S	BWP	121	1	8	1160	43	175	1	M	1	1	no	spread	oxer	right	no	0	0	70,59	no
Oslo	Western European League CSI-W5* round A	15.10.2017	CSI-W5*	4	1.40 - 1.60	410	125	16	07.05.2002	S	BWP	121	1	8	1160	43	175	1	M	2	2	no	vertical	vertical	right	no	0	0	70,59	no
Oslo	Western European League CSI-W5* round A	15.10.2017	CSI-W5*	4	1.40 - 1.60	410	125	16	07.05.2002	S	BWP	121	1	8	1160	43	175	1	M	3	3	no	vertical	vertical	left	no	0	0	70,59	no
Oslo	Western European League CSI-W5* round A	15.10.2017	CSI-W5*	4	1.40 - 1.60	410	125	16	07.05.2002	S	BWP	121	1	8	1160	43	175	1	M	4	4a	yes	vertical	vertical	straight	no	0	0	70,59	no
Oslo	Western European League CSI-W5* round A	15.10.2017	CSI-W5*	4	1.40 - 1.60	410	125	16	07.05.2002	S	BWP	121	1	8	1160	43	175	1	M	5	4b	yes	spread	oxer	straight	no	0	0	70,59	no
Oslo	Western European League CSI-W5* round A	15.10.2017	CSI-W5*	4	1.40 - 1.60	410	125	16	07.05.2002	S	BWP	121	1	8	1160	43	175	1	M	6	5	no	vertical	vertical with water	straight	yes	4	0	70,59	no
Oslo	Western European League CSI-W5* round A	15.10.2017	CSI-W5*	4	1.40 - 1.60	410	125	16	07.05.2002	S	BWP	121	1	8	1160	43	175	1	M	7	6a	yes	spread	oxer	right	no	0	0	70,59	no
Oslo	Western European League CSI-W5* round A	15.10.2017	CSI-W5*	4	1.40 - 1.60	410	125	16	07.05.2002	S	BWP	121	1	8	1160	43	175	1	M	8	6b	yes	vertical	vertical	right	no	0	0	70,59	no
Oslo	Western European League CSI-W5* round A	15.10.2017	CSI-W5*	4	1.40 - 1.60	410	125	16	07.05.2002	S	BWP	121	1	8	1160	43	175	1	M	9	6c	yes	spread	oxer	right	no	0	0	70,59	no
Oslo	Western European League CSI-W5* round A	15.10.2017	CSI-W5*	4	1.40 - 1.60	410	125	16	07.05.2002	S	BWP	121	1	8	1160	43	175	1	M	10	7	no	vertical	vertical	right	no	0	0	70,59	no
Oslo	Western European League CSI-W5* round A	15.10.2017	CSI-W5*	4	1.40 - 1.60	410	125	16	07.05.2002	S	BWP	121	1	8	1160	43	175	1	M	11	8	no	spread	oxer	left	yes	4	0	70,59	no
Oslo	Western European League CSI-W5* round A	15.10.2017	CSI-W5*	4	1.40 - 1.60	410	125	16	07.05.2002	S	BWP	121	1	8	1160	43	175	1	M	12	9	no	vertical	vertical	straight	no	0	0	70,59	no
Oslo	Western European League CSI-W5* round A	15.10.2017	CSI-W5*	4	1.40 - 1.60	410	125	16	07.05.2002	S	BWP	121	1	8	1160	43	175	1	M	13	10	no	spread	oxer	right	no	0	0	70,59	no
Oslo	Western European League CSI-W5* round A	15.10.2017	CSI-W5*	4	1.40 - 1.60	410	125	16	07.05.2002	S	BWP	121	1	8	1160	43	175	1	M	14	11	no	vertical	vertical	right	no	0	0	70,59	no
Oslo	Western European League CSI-W5* round A	15.10.2017	CSI-W5*	4	1.40 - 1.60	410	125	16	07.05.2002	S	BWP	121	1	8	1160	43	175	1	M	15	12	no	spread	oxer	left	no	0	0	70,59	no
Oslo	Western European League CSI-W5* round A	15.10.2017	CSI-W5*	4	1.40 - 1.60	410	125	16	07.05.2002	S	BWP	121	1	8	1160	43	175	1	M	16	13	no	spread	oxer	straight	no	0	0	70,59	no
Oslo	Western European League CSI-W5* round A	15.10.2017	CSI-W5*	4	1.40 - 1.60	410	195	11	25.04.2007	G	SF	164	16	9	103	48	1417	59	M	1	1	no	spread	oxer	right	no	0	0	70,55	no
Oslo	Western European League CSI-W5* round A	15.10.2017	CSI-W5*	4	1.40 - 1.60	410	195	11	25.04.2007	G	SF	164	16	9	103	48	1417	59	M	2	2	no	vertical	vertical	right	no	0	0	70,55	no
Oslo	Western European League CSI-W5* round A	15.10.2017	CSI-W5*	4	1.40 - 1.60	410	195	11	25.04.2007	G	SF	164	16	9	103	48	1417	59	M	3	3	no	vertical	vertical	left	no	0	0	70,55	no
Oslo	Western European League CSI-W5* round A	15.10.2017	CSI-W5*	4	1.40 - 1.60	410	195	11	25.04.2007	G	SF	164	16	9	103	48	1417	59	M	4	4a	yes	vertical	vertical	straight	no	0	0	70,55	no
Oslo	Western European League CSI-W5* round A	15.10.2017	CSI-W5*	4	1.40 - 1.60	410	195	11	25.04.2007	G	SF	164	16	9	103	48	1417	59	M	5	4b	yes	spread	oxer	straight	no	0	0	70,55	no
Oslo	Western European League CSI-W5* round A	15.10.2017	CSI-W5*	4	1.40 - 1.60	410	195	11	25.04.2007	G	SF	164	16	9	103	48	1417	59	M	6	5	no	vertical	vertical with water	straight	no	0	0	70,55	no
Oslo	Western European League CSI-W5* round A	15.10.2017	CSI-W5*	4	1.40 - 1.60	410	195	11	25.04.2007	G	SF	164	16	9	103	48	1417	59	M	7	6a	yes	spread	oxer	right	no	0	0	70,55	no
Oslo	Western European League CSI-W5* round A	15.10.2017	CSI-W5*	4	1.40 - 1.60	410	195	11	25.04.2007	G	SF	164	16	9	103	48	1417	59	M	8	6b	yes	vertical	vertical	right	no	0	0	70,55	no
Oslo	Western European League CSI-W5* round A	15.10.2017	CSI-W5*	4	1.40 - 1.60	410	195	11	25.04.2007	G	SF	164	16	9	103	48	1417	59	M	9	6c	yes	spread	oxer	right	no	0	0	70,55	no
Oslo	Western European League CSI-W5* round A	15.10.2017	CSI-W5*	4	1.40 - 1.60	410	195	11	25.04.2007	G	SF	164	16	9	103	48	1417	59	M	10	7	no	vertical	vertical	right	no	0	0	70,55	no
Oslo	Western European League CSI-W5* round A	15.10.2017	CSI-W5*	4	1.40 - 1.60	410	195	11	25.04.2007	G	SF	164	16	9	103	48	1417	59	M	11	8	no	spread	oxer	left	no	0	0	70,55	no
Oslo	Western European League CSI-W5* round A	15.10.2017	CSI-W5*	4	1.40 - 1.60	410	195	11	25.04.2007	G	SF	164	16	9	103	48	1417	59	M	12	9	no	vertical	vertical	straight	yes	4	0	70,55	no
Oslo	Western European League CSI-W5* round A	15.10.2017	CSI-W5*	4	1.40 - 1.60	410	195	11	25.04.2007	G	SF	164	16	9	103	48	1417	59	M	13	10	no	spread	oxer	right	no	0	0	70,55	no
Oslo	Western European League CSI-W5* round A	15.10.2017	CSI-W5*	4	1.40 - 1.60	410	195	11	25.04.2007	G	SF	164	16	9	103	48	1417	59	M	14	11	no	vertical	vertical	right	no	0	0	70,55	no
Oslo	Western European League CSI-W5* round A	15.10.2017	CSI-W5*	4	1.40 - 1.60	410	195	11	25.04.2007	G	SF	164	16	9	103	48	1417	59	M	15	12	no	spread	oxer	left	no	0	0	70,55	no
Oslo	Western European League CSI-W5* round A	15.10.2017	CSI-W5*	4	1.40 - 1.60	410	195	11	25.04.2007	G	SF	164	16	9	103	48	1417	59	M	16	13	no	spread	oxer	straight	no	0	0	70,55	no
Oslo	Western European League CSI-W5* round A	15.10.2017	CSI-W5*	4	1.40 - 1.60	410	40	12	09.05.2006	G	OS	205	5	10	94	43	1847	60	M	1	1	no	spread	oxer	right	no	0	0	70,37	no
Oslo	Western European League CSI-W5* round A	15.10.2017	CSI-W5*	4	1.40 - 1.60	410	40	12	09.05.2006	G	OS	205	5	10	94	43	1847	60	M	2	2	no	vertical	vertical	right	no	0	0	70,37	no
Oslo	Western European League CSI-W5* round A	15.10.2017	CSI-W5*	4	1.40 - 1.60	410	40	12	09.05.2006	G	OS	205	5	10	94	43	1847	60	M	3	3	no	vertical	vertical	left	no	0	0	70,37	no
Oslo	Western European League CSI-W5* round A	15.10.2017	CSI-W5*	4	1.40 - 1.60	410	40	12	09.05.2006	G	OS	205	5	10	94	43	1847	60	M	4	4a	yes	vertical	vertical	straight	no	0	0	70,37	no
Oslo	Western European League CSI-W5* round A	15.10.2017	CSI-W5*	4	1.40 - 1.60	410	40	12	09.05.2006	G	OS	205	5	10	94	43	1847	60	M	5	4b	yes	spread	oxer	straight	no	0	0	70,37	no
Oslo	Western European League CSI-W5* round A	15.10.2017	CSI-W5*	4	1.40 - 1.60	410	40	12	09.05.2006	G	OS	205	5	10	94	43	1847	60	M	6	5	no	vertical	vertical with water	straight	no	0	0	70,37	no
Oslo	Western European League CSI-W5* round A	15.10.2017	CSI-W5*	4	1.40 - 1.60	410	40	12	09.05.2006	G	OS	205	5	10	94	43	1847	60	M	7	6a	yes	spread	oxer	right	no	0	0	70,37	no
Oslo	Western European League CSI-W5* round A	15.10.2017	CSI-W5*	4	1.40 - 1.60	410	40	12	09.05.2006	G	OS	205	5	10	94	43	1847	60	M	8	6b	yes	vertical	vertical	right	no	0	0	70,37	no
Oslo	Western European League CSI-W5* round A	15.10.2017	CSI-W5*	4	1.40 - 1.60	410	40	12	09.05.2006	G	OS	205	5	10	94	43	1847	60	M	9	6c	yes	spread	oxer	right	no	0	0	70,37	no
Oslo	Western European League CSI-W5* round A	15.10.2017	CSI-W5*	4	1.40 - 1.60	410	40	12	09.05.2006	G	OS	205	5	10	94	43	1847	60	M	10	7	no	vertical	vertical	right	no	0	0	70,37	no
Oslo	Western European League CSI-W5* round A	15.10.2017	CSI-W5*	4	1.40 - 1.60	410	40	12	09.05.2006	G	OS	205	5	10	94	43	1847	60	M	11	8	no	spread	oxer	left	no	0	0	70,37	no
Oslo	Western European League CSI-W5* round A	15.10.2017	CSI-W5*	4	1.40 - 1.60	410	40	12	09.05.2006	G	OS	205	5	10	94	43	1847	60	M	12	9	no	vertical	vertical	straight	yes	4	0	70,37	no
Oslo	Western European League CSI-W5* round A	15.10.2017	CSI-W5*	4	1.40 - 1.60	410	40	12	09.05.2006	G	OS	205	5	10	94	43	1847	60	M	13	10	no	spread	oxer	right	no	0	0	70,37	no
Oslo	Western European League CSI-W5* round A	15.10.2017	CSI-W5*	4	1.40 - 1.60	410	40	12	09.05.2006	G	OS	205	5	10	94	43	1847	60	M	14	11	no	vertical	vertical	right	no	0	0	70,37	no
Oslo	Western European League CSI-W5* round A	15.10.2017	CSI-W5*	4	1.40 - 1.60	410	40	12	09.05.2006	G	OS	205	5	10	94	43	1847	60	M	15	12	no	spread	oxer	left	yes	4	0	70,37	no
Oslo	Western European League CSI-W5* round A	15.10.2017	CSI-W5*	4	1.40 - 1.60	410	40	12	09.05.2006	G	OS	205	5	10	94	43	1847	60	M	16	13	no	spread	oxer	straight	no	0	0	70,37	no
Oslo	Western European League CSI-W5* round A	15.10.2017	CSI-W5*	4	1.40 - 1.60	410	159	11	15.04.2007	M	PZHK	161	4	11	414	32	1331	18	F	1	1	no	spread	oxer	right	no	0	0	69,95	no
Oslo	Western European League CSI-W5* round A	15.10.2017	CSI-W5*	4	1.40 - 1.60	410	159	11	15.04.2007	M	PZHK	161	4	11	414	32	1331	18	F	2	2	no	vertical	vertical	right	no	0	0	69,95	no
Oslo	Western European League CSI-W5* round A	15.10.2017	CSI-W5*	4	1.40 - 1.60	410	159	11	15.04.2007	M	PZHK	161	4	11	414	32	1331	18	F	3	3	no	vertical	vertical	left	no	0	0	69,95	no
Oslo	Western European League CSI-W5* round A	15.10.2017	CSI-W5*	4	1.40 - 1.60	410	159	11	15.04.2007	M	PZHK	161	4	11	414	32	1331	18	F	4	4a	yes	vertical	vertical	straight	no	0	0	69,95	no
Oslo	Western European League CSI-W5* round A	15.10.2017	CSI-W5*	4	1.40 - 1.60	410	159	11	15.04.2007	M	PZHK	161	4	11	414	32	1331	18	F	5	4b	yes	spread	oxer	straight	yes	4	0	69,95	no
Oslo	Western European League CSI-W5* round A	15.10.2017	CSI-W5*	4	1.40 - 1.60	410	159	11	15.04.2007	M	PZHK	161	4	11	414	32	1331	18	F	6	5	no	vertical	vertical with water	straight	no	0	0	69,95	no
Oslo	Western European League CSI-W5* round A	15.10.2017	CSI-W5*	4	1.40 - 1.60	410	159	11	15.04.2007	M	PZHK	161	4	11	414	32	1331	18	F	7	6a	yes	spread	oxer	right	no	0	0	69,95	no
Oslo	Western European League CSI-W5* round A	15.10.2017	CSI-W5*	4	1.40 - 1.60	410	159	11	15.04.2007	M	PZHK	161	4	11	414	32	1331	18	F	8	6b	yes	vertical	vertical	right	no	0	0	69,95	no
Oslo	Western European League CSI-W5* round A	15.10.2017	CSI-W5*	4	1.40 - 1.60	410	159	11	15.04.2007	M	PZHK	161	4	11	414	32	1331	18	F	9	6c	yes	spread	oxer	right	no	0	0	69,95	no
Oslo	Western European League CSI-W5* round A	15.10.2017	CSI-W5*	4	1.40 - 1.60	410	159	11	15.04.2007	M	PZHK	161	4	11	414	32	1331	18	F	10	7	no	vertical	vertical	right	no	0	0	69,95	no
Oslo	Western European League CSI-W5* round A	15.10.2017	CSI-W5*	4	1.40 - 1.60	410	159	11	15.04.2007	M	PZHK	161	4	11	414	32	1331	18	F	11	8	no	spread	oxer	left	no	0	0	69,95	no
Oslo	Western European League CSI-W5* round A	15.10.2017	CSI-W5*	4	1.40 - 1.60	410	159	11	15.04.2007	M	PZHK	161	4	11	414	32	1331	18	F	12	9	no	vertical	vertical	straight	no	0	0	69,95	no
Oslo	Western European League CSI-W5* round A	15.10.2017	CSI-W5*	4	1.40 - 1.60	410	159	11	15.04.2007	M	PZHK	161	4	11	414	32	1331	18	F	13	10	no	spread	oxer	right	no	0	0	69,95	no
Oslo	Western European League CSI-W5* round A	15.10.2017	CSI-W5*	4	1.40 - 1.60	410	159	11	15.04.2007	M	PZHK	161	4	11	414	32	1331	18	F	14	11	no	vertical	vertical	right	no	0	0	69,95	no
Oslo	Western European League CSI-W5* round A	15.10.2017	CSI-W5*	4	1.40 - 1.60	410	159	11	15.04.2007	M	PZHK	161	4	11	414	32	1331	18	F	15	12	no	spread	oxer	left	no	0	0	69,95	no
Oslo	Western European League CSI-W5* round A	15.10.2017	CSI-W5*	4	1.40 - 1.60	410	159	11	15.04.2007	M	PZHK	161	4	11	414	32	1331	18	F	16	13	no	spread	oxer	straight	no	0	0	69,95	no
Oslo	Western European League CSI-W5* round A	15.10.2017	CSI-W5*	4	1.40 - 1.60	410	73	17	02.05.2001	G	BWP	229	4	12	235	58	1047	25	M	1	1	no	spread	oxer	right	no	0	0	69,62	no
Oslo	Western European League CSI-W5* round A	15.10.2017	CSI-W5*	4	1.40 - 1.60	410	73	17	02.05.2001	G	BWP	229	4	12	235	58	1047	25	M	2	2	no	vertical	vertical	right	no	0	0	69,62	no
Oslo	Western European League CSI-W5* round A	15.10.2017	CSI-W5*	4	1.40 - 1.60	410	73	17	02.05.2001	G	BWP	229	4	12	235	58	1047	25	M	3	3	no	vertical	vertical	left	no	0	0	69,62	no
Oslo	Western European League CSI-W5* round A	15.10.2017	CSI-W5*	4	1.40 - 1.60	410	73	17	02.05.2001	G	BWP	229	4	12	235	58	1047	25	M	4	4a	yes	vertical	vertical	straight	no	0	0	69,62	no
Oslo	Western European League CSI-W5* round A	15.10.2017	CSI-W5*	4	1.40 - 1.60	410	73	17	02.05.2001	G	BWP	229	4	12	235	58	1047	25	M	5	4b	yes	spread	oxer	straight	no	0	0	69,62	no
Oslo	Western European League CSI-W5* round A	15.10.2017	CSI-W5*	4	1.40 - 1.60	410	73	17	02.05.2001	G	BWP	229	4	12	235	58	1047	25	M	6	5	no	vertical	vertical with water	straight	no	0	0	69,62	no
Oslo	Western European League CSI-W5* round A	15.10.2017	CSI-W5*	4	1.40 - 1.60	410	73	17	02.05.2001	G	BWP	229	4	12	235	58	1047	25	M	7	6a	yes	spread	oxer	right	yes	4	0	69,62	no
Oslo	Western European League CSI-W5* round A	15.10.2017	CSI-W5*	4	1.40 - 1.60	410	73	17	02.05.2001	G	BWP	229	4	12	235	58	1047	25	M	8	6b	yes	vertical	vertical	right	no	0	0	69,62	no
Oslo	Western European League CSI-W5* round A	15.10.2017	CSI-W5*	4	1.40 - 1.60	410	73	17	02.05.2001	G	BWP	229	4	12	235	58	1047	25	M	9	6c	yes	spread	oxer	right	no	0	0	69,62	no
Oslo	Western European League CSI-W5* round A	15.10.2017	CSI-W5*	4	1.40 - 1.60	410	73	17	02.05.2001	G	BWP	229	4	12	235	58	1047	25	M	10	7	no	vertical	vertical	right	no	0	0	69,62	no
Oslo	Western European League CSI-W5* round A	15.10.2017	CSI-W5*	4	1.40 - 1.60	410	73	17	02.05.2001	G	BWP	229	4	12	235	58	1047	25	M	11	8	no	spread	oxer	left	no	0	0	69,62	no
Oslo	Western European League CSI-W5* round A	15.10.2017	CSI-W5*	4	1.40 - 1.60	410	73	17	02.05.2001	G	BWP	229	4	12	235	58	1047	25	M	12	9	no	vertical	vertical	straight	yes	4	0	69,62	no
Oslo	Western European League CSI-W5* round A	15.10.2017	CSI-W5*	4	1.40 - 1.60	410	73	17	02.05.2001	G	BWP	229	4	12	235	58	1047	25	M	13	10	no	spread	oxer	right	no	0	0	69,62	no
Oslo	Western European League CSI-W5* round A	15.10.2017	CSI-W5*	4	1.40 - 1.60	410	73	17	02.05.2001	G	BWP	229	4	12	235	58	1047	25	M	14	11	no	vertical	vertical	right	yes	4	0	69,62	no
Oslo	Western European League CSI-W5* round A	15.10.2017	CSI-W5*	4	1.40 - 1.60	410	73	17	02.05.2001	G	BWP	229	4	12	235	58	1047	25	M	15	12	no	spread	oxer	left	no	0	0	69,62	no
Oslo	Western European League CSI-W5* round A	15.10.2017	CSI-W5*	4	1.40 - 1.60	410	73	17	02.05.2001	G	BWP	229	4	12	235	58	1047	25	M	16	13	no	spread	oxer	straight	no	0	0	69,62	no
Oslo	Western European League CSI-W5* round A	15.10.2017	CSI-W5*	4	1.40 - 1.60	410	212	12	10.04.2006	S	BWP	134	11	13	42	49	1996	131	M	1	1	no	spread	oxer	right	no	0	0	69,07	no
Oslo	Western European League CSI-W5* round A	15.10.2017	CSI-W5*	4	1.40 - 1.60	410	212	12	10.04.2006	S	BWP	134	11	13	42	49	1996	131	M	2	2	no	vertical	vertical	right	no	0	0	69,07	no
Oslo	Western European League CSI-W5* round A	15.10.2017	CSI-W5*	4	1.40 - 1.60	410	212	12	10.04.2006	S	BWP	134	11	13	42	49	1996	131	M	3	3	no	vertical	vertical	left	yes	4	0	69,07	no
Oslo	Western European League CSI-W5* round A	15.10.2017	CSI-W5*	4	1.40 - 1.60	410	212	12	10.04.2006	S	BWP	134	11	13	42	49	1996	131	M	4	4a	yes	vertical	vertical	straight	yes	4	0	69,07	no
Oslo	Western European League CSI-W5* round A	15.10.2017	CSI-W5*	4	1.40 - 1.60	410	212	12	10.04.2006	S	BWP	134	11	13	42	49	1996	131	M	5	4b	yes	spread	oxer	straight	no	0	0	69,07	no
Oslo	Western European League CSI-W5* round A	15.10.2017	CSI-W5*	4	1.40 - 1.60	410	212	12	10.04.2006	S	BWP	134	11	13	42	49	1996	131	M	6	5	no	vertical	vertical with water	straight	yes	4	0	69,07	no
Oslo	Western European League CSI-W5* round A	15.10.2017	CSI-W5*	4	1.40 - 1.60	410	212	12	10.04.2006	S	BWP	134	11	13	42	49	1996	131	M	7	6a	yes	spread	oxer	right	no	0	0	69,07	no
Oslo	Western European League CSI-W5* round A	15.10.2017	CSI-W5*	4	1.40 - 1.60	410	212	12	10.04.2006	S	BWP	134	11	13	42	49	1996	131	M	8	6b	yes	vertical	vertical	right	no	0	0	69,07	no
Oslo	Western European League CSI-W5* round A	15.10.2017	CSI-W5*	4	1.40 - 1.60	410	212	12	10.04.2006	S	BWP	134	11	13	42	49	1996	131	M	9	6c	yes	spread	oxer	right	no	0	0	69,07	no
Oslo	Western European League CSI-W5* round A	15.10.2017	CSI-W5*	4	1.40 - 1.60	410	212	12	10.04.2006	S	BWP	134	11	13	42	49	1996	131	M	10	7	no	vertical	vertical	right	no	0	0	69,07	no
Oslo	Western European League CSI-W5* round A	15.10.2017	CSI-W5*	4	1.40 - 1.60	410	212	12	10.04.2006	S	BWP	134	11	13	42	49	1996	131	M	11	8	no	spread	oxer	left	no	0	0	69,07	no
Oslo	Western European League CSI-W5* round A	15.10.2017	CSI-W5*	4	1.40 - 1.60	410	212	12	10.04.2006	S	BWP	134	11	13	42	49	1996	131	M	12	9	no	vertical	vertical	straight	no	0	0	69,07	no
Oslo	Western European League CSI-W5* round A	15.10.2017	CSI-W5*	4	1.40 - 1.60	410	212	12	10.04.2006	S	BWP	134	11	13	42	49	1996	131	M	13	10	no	spread	oxer	right	no	0	0	69,07	no
Oslo	Western European League CSI-W5* round A	15.10.2017	CSI-W5*	4	1.40 - 1.60	410	212	12	10.04.2006	S	BWP	134	11	13	42	49	1996	131	M	14	11	no	vertical	vertical	right	no	0	0	69,07	no
Oslo	Western European League CSI-W5* round A	15.10.2017	CSI-W5*	4	1.40 - 1.60	410	212	12	10.04.2006	S	BWP	134	11	13	42	49	1996	131	M	15	12	no	spread	oxer	left	no	0	0	69,07	no
Oslo	Western European League CSI-W5* round A	15.10.2017	CSI-W5*	4	1.40 - 1.60	410	212	12	10.04.2006	S	BWP	134	11	13	42	49	1996	131	M	16	13	no	spread	oxer	straight	no	0	0	69,07	no
Oslo	Western European League CSI-W5* round A	15.10.2017	CSI-W5*	4	1.40 - 1.60	410	119	13	06.05.2005	S	OS	194	6	14	5	37	1868	96	M	1	1	no	spread	oxer	right	yes	4	0	69,06	no
Oslo	Western European League CSI-W5* round A	15.10.2017	CSI-W5*	4	1.40 - 1.60	410	119	13	06.05.2005	S	OS	194	6	14	5	37	1868	96	M	2	2	no	vertical	vertical	right	no	0	0	69,06	no
Oslo	Western European League CSI-W5* round A	15.10.2017	CSI-W5*	4	1.40 - 1.60	410	119	13	06.05.2005	S	OS	194	6	14	5	37	1868	96	M	3	3	no	vertical	vertical	left	no	0	0	69,06	no
Oslo	Western European League CSI-W5* round A	15.10.2017	CSI-W5*	4	1.40 - 1.60	410	119	13	06.05.2005	S	OS	194	6	14	5	37	1868	96	M	4	4a	yes	vertical	vertical	straight	no	0	0	69,06	no
Oslo	Western European League CSI-W5* round A	15.10.2017	CSI-W5*	4	1.40 - 1.60	410	119	13	06.05.2005	S	OS	194	6	14	5	37	1868	96	M	5	4b	yes	spread	oxer	straight	no	0	0	69,06	no
Oslo	Western European League CSI-W5* round A	15.10.2017	CSI-W5*	4	1.40 - 1.60	410	119	13	06.05.2005	S	OS	194	6	14	5	37	1868	96	M	6	5	no	vertical	vertical with water	straight	yes	4	0	69,06	no
Oslo	Western European League CSI-W5* round A	15.10.2017	CSI-W5*	4	1.40 - 1.60	410	119	13	06.05.2005	S	OS	194	6	14	5	37	1868	96	M	7	6a	yes	spread	oxer	right	no	0	0	69,06	no
Oslo	Western European League CSI-W5* round A	15.10.2017	CSI-W5*	4	1.40 - 1.60	410	119	13	06.05.2005	S	OS	194	6	14	5	37	1868	96	M	8	6b	yes	vertical	vertical	right	yes	4	0	69,06	no
Oslo	Western European League CSI-W5* round A	15.10.2017	CSI-W5*	4	1.40 - 1.60	410	119	13	06.05.2005	S	OS	194	6	14	5	37	1868	96	M	9	6c	yes	spread	oxer	right	no	0	0	69,06	no
Oslo	Western European League CSI-W5* round A	15.10.2017	CSI-W5*	4	1.40 - 1.60	410	119	13	06.05.2005	S	OS	194	6	14	5	37	1868	96	M	10	7	no	vertical	vertical	right	no	0	0	69,06	no
Oslo	Western European League CSI-W5* round A	15.10.2017	CSI-W5*	4	1.40 - 1.60	410	119	13	06.05.2005	S	OS	194	6	14	5	37	1868	96	M	11	8	no	spread	oxer	left	no	0	0	69,06	no
Oslo	Western European League CSI-W5* round A	15.10.2017	CSI-W5*	4	1.40 - 1.60	410	119	13	06.05.2005	S	OS	194	6	14	5	37	1868	96	M	12	9	no	vertical	vertical	straight	no	0	0	69,06	no
Oslo	Western European League CSI-W5* round A	15.10.2017	CSI-W5*	4	1.40 - 1.60	410	119	13	06.05.2005	S	OS	194	6	14	5	37	1868	96	M	13	10	no	spread	oxer	right	no	0	0	69,06	no
Oslo	Western European League CSI-W5* round A	15.10.2017	CSI-W5*	4	1.40 - 1.60	410	119	13	06.05.2005	S	OS	194	6	14	5	37	1868	96	M	14	11	no	vertical	vertical	right	no	0	0	69,06	no
Oslo	Western European League CSI-W5* round A	15.10.2017	CSI-W5*	4	1.40 - 1.60	410	119	13	06.05.2005	S	OS	194	6	14	5	37	1868	96	M	15	12	no	spread	oxer	left	no	0	0	69,06	no
Oslo	Western European League CSI-W5* round A	15.10.2017	CSI-W5*	4	1.40 - 1.60	410	119	13	06.05.2005	S	OS	194	6	14	5	37	1868	96	M	16	13	no	spread	oxer	straight	no	0	0	69,06	no
Oslo	Western European League CSI-W5* round A	15.10.2017	CSI-W5*	4	1.40 - 1.60	410	66	10	02.04.2008	M	KWPN	107	2	15	138	49	1209	30	F	1	1	no	spread	oxer	right	no	0	0	69,03	no
Oslo	Western European League CSI-W5* round A	15.10.2017	CSI-W5*	4	1.40 - 1.60	410	66	10	02.04.2008	M	KWPN	107	2	15	138	49	1209	30	F	2	2	no	vertical	vertical	right	no	0	0	69,03	no
Oslo	Western European League CSI-W5* round A	15.10.2017	CSI-W5*	4	1.40 - 1.60	410	66	10	02.04.2008	M	KWPN	107	2	15	138	49	1209	30	F	3	3	no	vertical	vertical	left	no	0	0	69,03	no
Oslo	Western European League CSI-W5* round A	15.10.2017	CSI-W5*	4	1.40 - 1.60	410	66	10	02.04.2008	M	KWPN	107	2	15	138	49	1209	30	F	4	4a	yes	vertical	vertical	straight	no	0	0	69,03	no
Oslo	Western European League CSI-W5* round A	15.10.2017	CSI-W5*	4	1.40 - 1.60	410	66	10	02.04.2008	M	KWPN	107	2	15	138	49	1209	30	F	5	4b	yes	spread	oxer	straight	no	0	0	69,03	no
Oslo	Western European League CSI-W5* round A	15.10.2017	CSI-W5*	4	1.40 - 1.60	410	66	10	02.04.2008	M	KWPN	107	2	15	138	49	1209	30	F	6	5	no	vertical	vertical with water	straight	no	0	0	69,03	no
Oslo	Western European League CSI-W5* round A	15.10.2017	CSI-W5*	4	1.40 - 1.60	410	66	10	02.04.2008	M	KWPN	107	2	15	138	49	1209	30	F	7	6a	yes	spread	oxer	right	no	0	0	69,03	no
Oslo	Western European League CSI-W5* round A	15.10.2017	CSI-W5*	4	1.40 - 1.60	410	66	10	02.04.2008	M	KWPN	107	2	15	138	49	1209	30	F	8	6b	yes	vertical	vertical	right	yes	4	0	69,03	no
Oslo	Western European League CSI-W5* round A	15.10.2017	CSI-W5*	4	1.40 - 1.60	410	66	10	02.04.2008	M	KWPN	107	2	15	138	49	1209	30	F	9	6c	yes	spread	oxer	right	no	0	0	69,03	no
Oslo	Western European League CSI-W5* round A	15.10.2017	CSI-W5*	4	1.40 - 1.60	410	66	10	02.04.2008	M	KWPN	107	2	15	138	49	1209	30	F	10	7	no	vertical	vertical	right	no	0	0	69,03	no
Oslo	Western European League CSI-W5* round A	15.10.2017	CSI-W5*	4	1.40 - 1.60	410	66	10	02.04.2008	M	KWPN	107	2	15	138	49	1209	30	F	11	8	no	spread	oxer	left	no	0	0	69,03	no
Oslo	Western European League CSI-W5* round A	15.10.2017	CSI-W5*	4	1.40 - 1.60	410	66	10	02.04.2008	M	KWPN	107	2	15	138	49	1209	30	F	12	9	no	vertical	vertical	straight	no	0	0	69,03	no
Oslo	Western European League CSI-W5* round A	15.10.2017	CSI-W5*	4	1.40 - 1.60	410	66	10	02.04.2008	M	KWPN	107	2	15	138	49	1209	30	F	13	10	no	spread	oxer	right	no	0	0	69,03	no
Oslo	Western European League CSI-W5* round A	15.10.2017	CSI-W5*	4	1.40 - 1.60	410	66	10	02.04.2008	M	KWPN	107	2	15	138	49	1209	30	F	14	11	no	vertical	vertical	right	no	0	0	69,03	no
Oslo	Western European League CSI-W5* round A	15.10.2017	CSI-W5*	4	1.40 - 1.60	410	66	10	02.04.2008	M	KWPN	107	2	15	138	49	1209	30	F	15	12	no	spread	oxer	left	no	0	0	69,03	no
Oslo	Western European League CSI-W5* round A	15.10.2017	CSI-W5*	4	1.40 - 1.60	410	66	10	02.04.2008	M	KWPN	107	2	15	138	49	1209	30	F	16	13	no	spread	oxer	straight	no	0	0	69,03	no
Oslo	Western European League CSI-W5* round A	15.10.2017	CSI-W5*	4	1.40 - 1.60	410	56	14	12.03.2004	G	OLDBG	203	7	16	17	37	2215	106	M	1	1	no	spread	oxer	right	no	0	0	68,86	no
Oslo	Western European League CSI-W5* round A	15.10.2017	CSI-W5*	4	1.40 - 1.60	410	56	14	12.03.2004	G	OLDBG	203	7	16	17	37	2215	106	M	2	2	no	vertical	vertical	right	no	0	0	68,86	no
Oslo	Western European League CSI-W5* round A	15.10.2017	CSI-W5*	4	1.40 - 1.60	410	56	14	12.03.2004	G	OLDBG	203	7	16	17	37	2215	106	M	3	3	no	vertical	vertical	left	no	0	0	68,86	no
Oslo	Western European League CSI-W5* round A	15.10.2017	CSI-W5*	4	1.40 - 1.60	410	56	14	12.03.2004	G	OLDBG	203	7	16	17	37	2215	106	M	4	4a	yes	vertical	vertical	straight	no	0	0	68,86	no
Oslo	Western European League CSI-W5* round A	15.10.2017	CSI-W5*	4	1.40 - 1.60	410	56	14	12.03.2004	G	OLDBG	203	7	16	17	37	2215	106	M	5	4b	yes	spread	oxer	straight	no	0	0	68,86	no
Oslo	Western European League CSI-W5* round A	15.10.2017	CSI-W5*	4	1.40 - 1.60	410	56	14	12.03.2004	G	OLDBG	203	7	16	17	37	2215	106	M	6	5	no	vertical	vertical with water	straight	no	0	0	68,86	no
Oslo	Western European League CSI-W5* round A	15.10.2017	CSI-W5*	4	1.40 - 1.60	410	56	14	12.03.2004	G	OLDBG	203	7	16	17	37	2215	106	M	7	6a	yes	spread	oxer	right	no	0	0	68,86	no
Oslo	Western European League CSI-W5* round A	15.10.2017	CSI-W5*	4	1.40 - 1.60	410	56	14	12.03.2004	G	OLDBG	203	7	16	17	37	2215	106	M	8	6b	yes	vertical	vertical	right	no	0	0	68,86	no
Oslo	Western European League CSI-W5* round A	15.10.2017	CSI-W5*	4	1.40 - 1.60	410	56	14	12.03.2004	G	OLDBG	203	7	16	17	37	2215	106	M	9	6c	yes	spread	oxer	right	no	0	0	68,86	no
Oslo	Western European League CSI-W5* round A	15.10.2017	CSI-W5*	4	1.40 - 1.60	410	56	14	12.03.2004	G	OLDBG	203	7	16	17	37	2215	106	M	10	7	no	vertical	vertical	right	no	0	0	68,86	no
Oslo	Western European League CSI-W5* round A	15.10.2017	CSI-W5*	4	1.40 - 1.60	410	56	14	12.03.2004	G	OLDBG	203	7	16	17	37	2215	106	M	11	8	no	spread	oxer	left	no	0	0	68,86	no
Oslo	Western European League CSI-W5* round A	15.10.2017	CSI-W5*	4	1.40 - 1.60	410	56	14	12.03.2004	G	OLDBG	203	7	16	17	37	2215	106	M	12	9	no	vertical	vertical	straight	no	0	0	68,86	no
Oslo	Western European League CSI-W5* round A	15.10.2017	CSI-W5*	4	1.40 - 1.60	410	56	14	12.03.2004	G	OLDBG	203	7	16	17	37	2215	106	M	13	10	no	spread	oxer	right	no	0	0	68,86	no
Oslo	Western European League CSI-W5* round A	15.10.2017	CSI-W5*	4	1.40 - 1.60	410	56	14	12.03.2004	G	OLDBG	203	7	16	17	37	2215	106	M	14	11	no	vertical	vertical	right	no	0	0	68,86	no
Oslo	Western European League CSI-W5* round A	15.10.2017	CSI-W5*	4	1.40 - 1.60	410	56	14	12.03.2004	G	OLDBG	203	7	16	17	37	2215	106	M	15	12	no	spread	oxer	left	no	0	0	68,86	no
Oslo	Western European League CSI-W5* round A	15.10.2017	CSI-W5*	4	1.40 - 1.60	410	56	14	12.03.2004	G	OLDBG	203	7	16	17	37	2215	106	M	16	13	no	spread	oxer	straight	no	0	0	68,86	no
Oslo	Western European League CSI-W5* round B	15.10.2017	CSI-W5*	4	1.40 - 1.60	295	56	14	12.03.2004	G	OLDBG	203	7	16	17	37	2215	106	M	1	1	no	vertical	vertical	right	no	0	0	36,83	no
Oslo	Western European League CSI-W5* round B	15.10.2017	CSI-W5*	4	1.40 - 1.60	295	56	14	12.03.2004	G	OLDBG	203	7	16	17	37	2215	106	M	2	2	no	spread	oxer	right	no	0	0	36,83	no
Oslo	Western European League CSI-W5* round B	15.10.2017	CSI-W5*	4	1.40 - 1.60	295	56	14	12.03.2004	G	OLDBG	203	7	16	17	37	2215	106	M	3	3	no	vertical	vertical	right	no	0	0	36,83	no
Oslo	Western European League CSI-W5* round B	15.10.2017	CSI-W5*	4	1.40 - 1.60	295	56	14	12.03.2004	G	OLDBG	203	7	16	17	37	2215	106	M	4	4	no	spread	oxer	left	no	0	0	36,83	no

Oslo	Western European League CSI-W5* round B	15.10.2017	CSI-W5*	4	1.40 - 1.60	295	56	14	12.03.2004	G	OLDBG	203	7	16	17	37	2215	106	M	5	5	no	vertical	vertical	left	no	0	0	36,83	no
Oslo	Western European League CSI-W5* round B	15.10.2017	CSI-W5*	4	1.40 - 1.60	295	56	14	12.03.2004	G	OLDBG	203	7	16	17	37	2215	106	M	6	6a	yes	vertical	vertical	straight	no	0	0	36,83	no
Oslo	Western European League CSI-W5* round B	15.10.2017	CSI-W5*	4	1.40 - 1.60	295	56	14	12.03.2004	G	OLDBG	203	7	16	17	37	2215	106	M	7	6b	yes	spread	oxer	straight	no	0	0	36,83	no
Oslo	Western European League CSI-W5* round B	15.10.2017	CSI-W5*	4	1.40 - 1.60	295	56	14	12.03.2004	G	OLDBG	203	7	16	17	37	2215	106	M	8	7	no	spread	oxer	right	no	0	0	36,83	no
Oslo	Western European League CSI-W5* round B	15.10.2017	CSI-W5*	4	1.40 - 1.60	295	56	14	12.03.2004	G	OLDBG	203	7	16	17	37	2215	106	M	9	8	no	vertical	vertical	right	no	0	0	36,83	no
Oslo	Western European League CSI-W5* round A	15.10.2017	CSI-W5*	4	1.40 - 1.60	410	104	11	23.03.2007	G	KWPN	95	0	17	3	46	1005	51	M	1	1	no	spread	oxer	right	no	0	0	68,76	no
Oslo	Western European League CSI-W5* round A	15.10.2017	CSI-W5*	4	1.40 - 1.60	410	104	11	23.03.2007	G	KWPN	95	0	17	3	46	1005	51	M	2	2	no	vertical	vertical	right	no	0	0	68,76	no
Oslo	Western European League CSI-W5* round A	15.10.2017	CSI-W5*	4	1.40 - 1.60	410	104	11	23.03.2007	G	KWPN	95	0	17	3	46	1005	51	M	3	3	no	vertical	vertical	left	no	0	0	68,76	no
Oslo	Western European League CSI-W5* round A	15.10.2017	CSI-W5*	4	1.40 - 1.60	410	104	11	23.03.2007	G	KWPN	95	0	17	3	46	1005	51	M	4	4a	yes	vertical	vertical	straight	no	0	0	68,76	no
Oslo	Western European League CSI-W5* round A	15.10.2017	CSI-W5*	4	1.40 - 1.60	410	104	11	23.03.2007	G	KWPN	95	0	17	3	46	1005	51	M	5	4b	yes	spread	oxer	straight	no	0	0	68,76	no
Oslo	Western European League CSI-W5* round A	15.10.2017	CSI-W5*	4	1.40 - 1.60	410	104	11	23.03.2007	G	KWPN	95	0	17	3	46	1005	51	M	6	5	no	vertical	vertical with water	straight	no	0	0	68,76	no
Oslo	Western European League CSI-W5* round A	15.10.2017	CSI-W5*	4	1.40 - 1.60	410	104	11	23.03.2007	G	KWPN	95	0	17	3	46	1005	51	M	7	6a	yes	spread	oxer	right	no	0	0	68,76	no
Oslo	Western European League CSI-W5* round A	15.10.2017	CSI-W5*	4	1.40 - 1.60	410	104	11	23.03.2007	G	KWPN	95	0	17	3	46	1005	51	M	8	6b	yes	vertical	vertical	right	no	0	0	68,76	no
Oslo	Western European League CSI-W5* round A	15.10.2017	CSI-W5*	4	1.40 - 1.60	410	104	11	23.03.2007	G	KWPN	95	0	17	3	46	1005	51	M	9	6c	yes	spread	oxer	right	no	0	0	68,76	no
Oslo	Western European League CSI-W5* round A	15.10.2017	CSI-W5*	4	1.40 - 1.60	410	104	11	23.03.2007	G	KWPN	95	0	17	3	46	1005	51	M	10	7	no	vertical	vertical	right	no	0	0	68,76	no
Oslo	Western European League CSI-W5* round A	15.10.2017	CSI-W5*	4	1.40 - 1.60	410	104	11	23.03.2007	G	KWPN	95	0	17	3	46	1005	51	M	11	8	no	spread	oxer	left	no	0	0	68,76	no
Oslo	Western European League CSI-W5* round A	15.10.2017	CSI-W5*	4	1.40 - 1.60	410	104	11	23.03.2007	G	KWPN	95	0	17	3	46	1005	51	M	12	9	no	vertical	vertical	straight	no	0	0	68,76	no
Oslo	Western European League CSI-W5* round A	15.10.2017	CSI-W5*	4	1.40 - 1.60	410	104	11	23.03.2007	G	KWPN	95	0	17	3	46	1005	51	M	13	10	no	spread	oxer	right	no	0	0	68,76	no
Oslo	Western European League CSI-W5* round A	15.10.2017	CSI-W5*	4	1.40 - 1.60	410	104	11	23.03.2007	G	KWPN	95	0	17	3	46	1005	51	M	14	11	no	vertical	vertical	right	no	0	0	68,76	no
Oslo	Western European League CSI-W5* round A	15.10.2017	CSI-W5*	4	1.40 - 1.60	410	104	11	23.03.2007	G	KWPN	95	0	17	3	46	1005	51	M	15	12	no	spread	oxer	left	no	0	0	68,76	no
Oslo	Western European League CSI-W5* round A	15.10.2017	CSI-W5*	4	1.40 - 1.60	410	104	11	23.03.2007	G	KWPN	95	0	17	3	46	1005	51	M	16	13	no	spread	oxer	straight	no	0	0	68,76	no
Oslo	Western European League CSI-W5* round B	15.10.2017	CSI-W5*	4	1.40 - 1.60	295	104	11	23.03.2007	G	KWPN	95	0	17	3	46	1005	51	M	1	1	no	vertical	vertical	right	no	0	0	38,44	no
Oslo	Western European League CSI-W5* round B	15.10.2017	CSI-W5*	4	1.40 - 1.60	295	104	11	23.03.2007	G	KWPN	95	0	17	3	46	1005	51	M	2	2	no	spread	oxer	right	no	0	0	38,44	no
Oslo	Western European League CSI-W5* round B	15.10.2017	CSI-W5*	4	1.40 - 1.60	295	104	11	23.03.2007	G	KWPN	95	0	17	3	46	1005	51	M	3	3	no	vertical	vertical	right	no	0	0	38,44	no
Oslo	Western European League CSI-W5* round B	15.10.2017	CSI-W5*	4	1.40 - 1.60	295	104	11	23.03.2007	G	KWPN	95	0	17	3	46	1005	51	M	4	4	no	spread	oxer	left	no	0	0	38,44	no
Oslo	Western European League CSI-W5* round B	15.10.2017	CSI-W5*	4	1.40 - 1.60	295	104	11	23.03.2007	G	KWPN	95	0	17	3	46	1005	51	M	5	5	no	vertical	vertical	left	no	0	0	38,44	no
Oslo	Western European League CSI-W5* round B	15.10.2017	CSI-W5*	4	1.40 - 1.60	295	104	11	23.03.2007	G	KWPN	95	0	17	3	46	1005	51	M	6	6a	yes	vertical	vertical	straight	no	0	0	38,44	no
Oslo	Western European League CSI-W5* round B	15.10.2017	CSI-W5*	4	1.40 - 1.60	295	104	11	23.03.2007	G	KWPN	95	0	17	3	46	1005	51	M	7	6b	yes	spread	oxer	straight	no	0	0	38,44	no
Oslo	Western European League CSI-W5* round B	15.10.2017	CSI-W5*	4	1.40 - 1.60	295	104	11	23.03.2007	G	KWPN	95	0	17	3	46	1005	51	M	8	7	no	spread	oxer	right	no	0	0	38,44	no
Oslo	Western European League CSI-W5* round B	15.10.2017	CSI-W5*	4	1.40 - 1.60	295	104	11	23.03.2007	G	KWPN	95	0	17	3	46	1005	51	M	9	8	no	vertical	vertical	right	no	0	0	38,44	no
Oslo	Western European League CSI-W5* round A	15.10.2017	CSI-W5*	4	1.40 - 1.60	410	154	14	29.04.2004	G	SWB	175	3	18	81	31	1327	0	M	1	1	no	spread	oxer	right	no	0	0	68,42	no
Oslo	Western European League CSI-W5* round A	15.10.2017	CSI-W5*	4	1.40 - 1.60	410	154	14	29.04.2004	G	SWB	175	3	18	81	31	1327	0	M	2	2	no	vertical	vertical	right	no	0	0	68,42	no
Oslo	Western European League CSI-W5* round A	15.10.2017	CSI-W5*	4	1.40 - 1.60	410	154	14	29.04.2004	G	SWB	175	3	18	81	31	1327	0	M	3	3	no	vertical	vertical	left	no	0	0	68,42	no
Oslo	Western European League CSI-W5* round A	15.10.2017	CSI-W5*	4	1.40 - 1.60	410	154	14	29.04.2004	G	SWB	175	3	18	81	31	1327	0	M	4	4a	yes	vertical	vertical	straight	no	0	0	68,42	no
Oslo	Western European League CSI-W5* round A	15.10.2017	CSI-W5*	4	1.40 - 1.60	410	154	14	29.04.2004	G	SWB	175	3	18	81	31	1327	0	M	5	4b	yes	spread	oxer	straight	no	0	0	68,42	no
Oslo	Western European League CSI-W5* round A	15.10.2017	CSI-W5*	4	1.40 - 1.60	410	154	14	29.04.2004	G	SWB	175	3	18	81	31	1327	0	M	6	5	no	vertical	vertical with water	straight	no	0	0	68,42	no
Oslo	Western European League CSI-W5* round A	15.10.2017	CSI-W5*	4	1.40 - 1.60	410	154	14	29.04.2004	G	SWB	175	3	18	81	31	1327	0	M	7	6a	yes	spread	oxer	right	no	0	0	68,42	no
Oslo	Western European League CSI-W5* round A	15.10.2017	CSI-W5*	4	1.40 - 1.60	410	154	14	29.04.2004	G	SWB	175	3	18	81	31	1327	0	M	8	6b	yes	vertical	vertical	right	no	0	0	68,42	no
Oslo	Western European League CSI-W5* round A	15.10.2017	CSI-W5*	4	1.40 - 1.60	410	154	14	29.04.2004	G	SWB	175	3	18	81	31	1327	0	M	9	6c	yes	spread	oxer	right	no	0	0	68,42	no
Oslo	Western European League CSI-W5* round A	15.10.2017	CSI-W5*	4	1.40 - 1.60	410	154	14	29.04.2004	G	SWB	175	3	18	81	31	1327	0	M	10	7	no	vertical	vertical	right	no	0	0	68,42	no
Oslo	Western European League CSI-W5* round A	15.10.2017	CSI-W5*	4	1.40 - 1.60	410	154	14	29.04.2004	G	SWB	175	3	18	81	31	1327	0	M	11	8	no	spread	oxer	left	no	0	0	68,42	no
Oslo	Western European League CSI-W5* round A	15.10.2017	CSI-W5*	4	1.40 - 1.60	410	154	14	29.04.2004	G	SWB	175	3	18	81	31	1327	0	M	12	9	no	vertical	vertical	straight	no	0	0	68,42	no
Oslo	Western European League CSI-W5* round A	15.10.2017	CSI-W5*	4	1.40 - 1.60	410	154	14	29.04.2004	G	SWB	175	3	18	81	31	1327	0	M	13	10	no	spread	oxer	right	no	0	0	68,42	no
Oslo	Western European League CSI-W5* round A	15.10.2017	CSI-W5*	4	1.40 - 1.60	410	154	14	29.04.2004	G	SWB	175	3	18	81	31	1327	0	M	14	11	no	vertical	vertical	right	no	0	0	68,42	no
Oslo	Western European League CSI-W5* round A	15.10.2017	CSI-W5*	4	1.40 - 1.60	410	154	14	29.04.2004	G	SWB	175	3	18	81	31	1327	0	M	15	12	no	spread	oxer	left	no	0	0	68,42	no
Oslo	Western European League CSI-W5* round A	15.10.2017	CSI-W5*	4	1.40 - 1.60	410	154	14	29.04.2004	G	SWB	175	3	18	81	31	1327	0	M	16	13	no	spread	oxer	straight	no	0	0	68,42	no
Oslo	Western European League CSI-W5* round B	15.10.2017	CSI-W5*	4	1.40 - 1.60	295	154	14	29.04.2004	G	SWB	175	3	18	81	31	1327	0	M	1	1	no	vertical	vertical	right	no	0	0	39,24	no
Oslo	Western European League CSI-W5* round B	15.10.2017	CSI-W5*	4	1.40 - 1.60	295	154	14	29.04.2004	G	SWB	175	3	18	81	31	1327	0	M	2	2	no	spread	oxer	right	no	0	0	39,24	no
Oslo	Western European League CSI-W5* round B	15.10.2017	CSI-W5*	4	1.40 - 1.60	295	154	14	29.04.2004	G	SWB	175	3	18	81	31	1327	0	M	3	3	no	vertical	vertical	right	no	0	0	39,24	no
Oslo	Western European League CSI-W5* round B	15.10.2017	CSI-W5*	4	1.40 - 1.60	295	154	14	29.04.2004	G	SWB	175	3	18	81	31	1327	0	M	4	4	no	spread	oxer	left	no	0	0	39,24	no
Oslo	Western European League CSI-W5* round B	15.10.2017	CSI-W5*	4	1.40 - 1.60	295	154	14	29.04.2004	G	SWB	175	3	18	81	31	1327	0	M	5	5	no	vertical	vertical	left	no	0	0	39,24	no
Oslo	Western European League CSI-W5* round B	15.10.2017	CSI-W5*	4	1.40 - 1.60	295	154	14	29.04.2004	G	SWB	175	3	18	81	31	1327	0	M	6	6a	yes	vertical	vertical	straight	no	0	0	39,24	no
Oslo	Western European League CSI-W5* round B	15.10.2017	CSI-W5*	4	1.40 - 1.60	295	154	14	29.04.2004	G	SWB	175	3	18	81	31	1327	0	M	7	6b	yes	spread	oxer	straight	no	0	0	39,24	no
Oslo	Western European League CSI-W5* round B	15.10.2017	CSI-W5*	4	1.40 - 1.60	295	154	14	29.04.2004	G	SWB	175	3	18	81	31	1327	0	M	8	7	no	spread	oxer	right	yes	4	0	39,24	no
Oslo	Western European League CSI-W5* round B	15.10.2017	CSI-W5*	4	1.40 - 1.60	295	154	14	29.04.2004	G	SWB	175	3	18	81	31	1327	0	M	9	8	no	vertical	vertical	right	no	0	0	39,24	no
Oslo	Western European League CSI-W5* round A	15.10.2017	CSI-W5*	4	1.40 - 1.60	410	213	11	29.04.2007	M	OLDBG	177	10	19	54	42	2041	67	M	1	1	no	spread	oxer	right	no	0	0	68,38	no
Oslo	Western European League CSI-W5* round A	15.10.2017	CSI-W5*	4	1.40 - 1.60	410	213	11	29.04.2007	M	OLDBG	177	10	19	54	42	2041	67	M	2	2	no	vertical	vertical	right	no	0	0	68,38	no
Oslo	Western European League CSI-W5* round A	15.10.2017	CSI-W5*	4	1.40 - 1.60	410	213	11	29.04.2007	M	OLDBG	177	10	19	54	42	2041	67	M	3	3	no	vertical	vertical	left	no	0	0	68,38	no
Oslo	Western European League CSI-W5* round A	15.10.2017	CSI-W5*	4	1.40 - 1.60	410	213	11	29.04.2007	M	OLDBG	177	10	19	54	42	2041	67	M	4	4a	yes	vertical	vertical	straight	no	0	0	68,38	no
Oslo	Western European League CSI-W5* round A	15.10.2017	CSI-W5*	4	1.40 - 1.60	410	213	11	29.04.2007	M	OLDBG	177	10	19	54	42	2041	67	M	5	4b	yes	spread	oxer	straight	no	0	0	68,38	no
Oslo	Western European League CSI-W5* round A	15.10.2017	CSI-W5*	4	1.40 - 1.60	410	213	11	29.04.2007	M	OLDBG	177	10	19	54	42	2041	67	M	6	5	no	vertical	vertical with water	straight	no	0	0	68,38	no
Oslo	Western European League CSI-W5* round A	15.10.2017	CSI-W5*	4	1.40 - 1.60	410	213	11	29.04.2007	M	OLDBG	177	10	19	54	42	2041	67	M	7	6a	yes	spread	oxer	right	no	0	0	68,38	no
Oslo	Western European League CSI-W5* round A	15.10.2017	CSI-W5*	4	1.40 - 1.60	410	213	11	29.04.2007	M	OLDBG	177	10	19	54	42	2041	67	M	8	6b	yes	vertical	vertical	right	no	0	0	68,38	no
Oslo	Western European League CSI-W5* round A	15.10.2017	CSI-W5*	4	1.40 - 1.60	410	213	11	29.04.2007	M	OLDBG	177	10	19	54	42	2041	67	M	9	6c	yes	spread	oxer	right	no	0	0	68,38	no
Oslo	Western European League CSI-W5* round A	15.10.2017	CSI-W5*	4	1.40 - 1.60	410	213	11	29.04.2007	M	OLDBG	177	10	19	54	42	2041	67	M	10	7	no	vertical	vertical	right	no	0	0	68,38	no
Oslo	Western European League CSI-W5* round A	15.10.2017	CSI-W5*	4	1.40 - 1.60	410	213	11	29.04.2007	M	OLDBG	177	10	19	54	42	2041	67	M	11	8	no	spread	oxer	left	no	0	0	68,38	no
Oslo	Western European League CSI-W5* round A	15.10.2017	CSI-W5*	4	1.40 - 1.60	410	213	11	29.04.2007	M	OLDBG	177	10	19	54	42	2041	67	M	12	9	no	vertical	vertical	straight	yes	4	0	68,38	no
Oslo	Western European League CSI-W5* round A	15.10.2017	CSI-W5*	4	1.40 - 1.60	410	213	11	29.04.2007	M	OLDBG	177	10	19	54	42	2041	67	M	13	10	no	spread	oxer	right	no	0	0	68,38	no
Oslo	Western European League CSI-W5* round A	15.10.2017	CSI-W5*	4	1.40 - 1.60	410	213	11	29.04.2007	M	OLDBG	177	10	19	54	42	2041	67	M	14	11	no	vertical	vertical	right	no	0	0	68,38	no
Oslo	Western European League CSI-W5* round A	15.10.2017	CSI-W5*	4	1.40 - 1.60	410	213	11	29.04.2007	M	OLDBG	177	10	19	54	42	2041	67	M	15	12	no	spread	oxer	left	no	0	0	68,38	no
Oslo	Western European League CSI-W5* round A	15.10.2017	CSI-W5*	4	1.40 - 1.60	410	213	11	29.04.2007	M	OLDBG	177	10	19	54	42	2041	67	M	16	13	no	spread	oxer	straight	no	0	0	68,38	no
Oslo	Western European League CSI-W5* round A	15.10.2017	CSI-W5*	4	1.40 - 1.60	410	183	12	10.06.2006	M	AA	243	8	20	73	38	2030	91	M	1	1	no	spread	oxer	right	no	0	0	68,09	no
Oslo	Western European League CSI-W5* round A	15.10.2017	CSI-W5*	4	1.40 - 1.60	410	183	12	10.06.2006	M	AA	243	8	20	73	38	2030	91	M	2	2	no	vertical	vertical	right	no	0	0	68,09	no
Oslo	Western European League CSI-W5* round A	15.10.2017	CSI-W5*	4	1.40 - 1.60	410	183	12	10.06.2006	M	AA	243	8	20	73	38	2030	91	M	3	3	no	vertical	vertical	left	no	0	0	68,09	no
Oslo	Western European League CSI-W5* round A	15.10.2017	CSI-W5*	4	1.40 - 1.60	410	183	12	10.06.2006	M	AA	243	8	20	73	38	2030	91	M	4	4a	yes	vertical	vertical	straight	no	0	0	68,09	no
Oslo	Western European League CSI-W5* round A	15.10.2017	CSI-W5*	4	1.40 - 1.60	410	183	12	10.06.2006	M	AA	243	8	20	73	38	2030	91	M	5	4b	yes	spread	oxer	straight	no	0	0	68,09	no
Oslo	Western European League CSI-W5* round A	15.10.2017	CSI-W5*	4	1.40 - 1.60	410	183	12	10.06.2006	M	AA	243	8	20	73	38	2030	91	M	6	5	no	vertical	vertical with water	straight	no	0	0	68,09	no
Oslo	Western European League CSI-W5* round A	15.10.2017	CSI-W5*	4	1.40 - 1.60	410	183	12	10.06.2006	M	AA	243	8	20	73	38	2030	91	M	7	6a	yes	spread	oxer	right	no	0	0	68,09	no
Oslo	Western European League CSI-W5* round A	15.10.2017	CSI-W5*	4	1.40 - 1.60	410	183	12	10.06.2006	M	AA	243	8	20	73	38	2030	91	M	8	6b	yes	vertical	vertical	right	no	0	0	68,09	no
Oslo	Western European League CSI-W5* round A	15.10.2017	CSI-W5*	4	1.40 - 1.60	410	183	12	10.06.2006	M	AA	243	8	20	73	38	2030	91	M	9	6c	yes	spread	oxer	right	yes	4	0	68,09	no
Oslo	Western European League CSI-W5* round A	15.10.2017	CSI-W5*	4	1.40 - 1.60	410	183	12	10.06.2006	M	AA	243	8	20	73	38	2030	91	M	10	7	no	vertical	vertical	right	no	0	0	68,09	no
Oslo	Western European League CSI-W5* round A	15.10.2017	CSI-W5*	4	1.40 - 1.60	410	183	12	10.06.2006	M	AA	243	8	20	73	38	2030	91	M	11	8	no	spread	oxer	left	no	0	0	68,09	no
Oslo	Western European League CSI-W5* round A	15.10.2017	CSI-W5*	4	1.40 - 1.60	410	183	12	10.06.2006	M	AA	243	8	20	73	38	2030	91	M	12	9	no	vertical	vertical	straight	no	0	0	68,09	no
Oslo	Western European League CSI-W5* round A	15.10.2017	CSI-W5*	4	1.40 - 1.60	410	183	12	10.06.2006	M	AA	243	8	20	73	38	2030	91	M	13	10	no	spread	oxer	right	no	0	0	68,09	no
Oslo	Western European League CSI-W5* round A	15.10.2017	CSI-W5*	4	1.40 - 1.60	410	183	12	10.06.2006	M	AA	243	8	20	73	38	2030	91	M	14	11	no	vertical	vertical	right	no	0	0	68,09	no
Oslo	Western European League CSI-W5* round A	15.10.2017	CSI-W5*	4	1.40 - 1.60	410	183	12	10.06.2006	M	AA	243	8	20	73	38	2030	91	M	15	12	no	spread	oxer	left	no	0	0	68,09	no
Oslo	Western European League CSI-W5* round A	15.10.2017	CSI-W5*	4	1.40 - 1.60	410	183	12	10.06.2006	M	AA	243	8	20	73	38	2030	91	M	16	13	no	spread	oxer	straight	no	0	0	68,09	no
Oslo	Western European League CSI-W5* round A	15.10.2017	CSI-W5*	4	1.40 - 1.60	410	219	13	13.05.2005	G	SWB	110	2	21	99	28	1384	56	F	1	1	no	spread	oxer	right	no	0	0	68,04	no
Oslo	Western European League CSI-W5* round A	15.10.2017	CSI-W5*	4	1.40 - 1.60	410	219	13	13.05.2005	G	SWB	110	2	21	99	28	1384	56	F	2	2	no	vertical	vertical	right	no	0	0	68,04	no
Oslo	Western European League CSI-W5* round A	15.10.2017	CSI-W5*	4	1.40 - 1.60	410	219	13	13.05.2005	G	SWB	110	2	21	99	28	1384	56	F	3	3	no	vertical	vertical	left	no	0	0	68,04	no
Oslo	Western European League CSI-W5* round A	15.10.2017	CSI-W5*	4	1.40 - 1.60	410	219	13	13.05.2005	G	SWB	110	2	21	99	28	1384	56	F	4	4a	yes	vertical	vertical	straight	no	0	0	68,04	no
Oslo	Western European League CSI-W5* round A	15.10.2017	CSI-W5*	4	1.40 - 1.60	410	219	13	13.05.2005	G	SWB	110	2	21	99	28	1384	56	F	5	4b	yes	spread	oxer	straight	no	0	0	68,04	no
Oslo	Western European League CSI-W5* round A	15.10.2017	CSI-W5*	4	1.40 - 1.60	410	219	13	13.05.2005	G	SWB	110	2	21	99	28	1384	56	F	6	5	no	vertical	vertical with water	straight	no	0	0	68,04	no
Oslo	Western European League CSI-W5* round A	15.10.2017	CSI-W5*	4	1.40 - 1.60	410	219	13	13.05.2005	G	SWB	110	2	21	99	28	1384	56	F	7	6a	yes	spread	oxer	right	no	0	0	68,04	no
Oslo	Western European League CSI-W5* round A	15.10.2017	CSI-W5*	4	1.40 - 1.60	410	219	13	13.05.2005	G	SWB	110	2	21	99	28	1384	56	F	8	6b	yes	vertical	vertical	right	no	0	0	68,04	no
Oslo	Western European League CSI-W5* round A	15.10.2017	CSI-W5*	4	1.40 - 1.60	410	219	13	13.05.2005	G	SWB	110	2	21	99	28	1384	56	F	9	6c	yes	spread	oxer	right	no	0	0	68,04	no
Oslo	Western European League CSI-W5* round A	15.10.2017	CSI-W5*	4	1.40 - 1.60	410	219	13	13.05.2005	G	SWB	110	2	21	99	28	1384	56	F	10	7	no	vertical	vertical	right	no	0	0	68,04	no
Oslo	Western European League CSI-W5* round A	15.10.2017	CSI-W5*	4	1.40 - 1.60	410	219	13	13.05.2005	G	SWB	110	2	21	99	28	1384	56	F	11	8	no	spread	oxer	left	no	0	0	68,04	no
Oslo	Western European League CSI-W5* round A	15.10.2017	CSI-W5*	4	1.40 - 1.60	410	219	13	13.05.2005	G	SWB	110	2	21	99	28	1384	56	F	12	9	no	vertical	vertical	straight	no	0	0	68,04	no
Oslo	Western European League CSI-W5* round A	15.10.2017	CSI-W5*	4	1.40 - 1.60	410	219	13	13.05.2005	G	SWB	110	2	21	99	28	1384	56	F	13	10	no	spread	oxer	right	no	0	0	68,04	no
Oslo	Western European League CSI-W5* round A	15.10.2017	CSI-W5*	4	1.40 - 1.60	410	219	13	13.05.2005	G	SWB	110	2	21	99	28	1384	56	F	14	11	no	vertical	vertical	right	no	0	0	68,04	no
Oslo	Western European League CSI-W5* round A	15.10.2017	CSI-W5*	4	1.40 - 1.60	410	219	13	13.05.2005	G	SWB	110	2	21	99	28	1384	56	F	15	12	no	spread	oxer	left	no	0	0	68,04	no
Oslo	Western European League CSI-W5* round A	15.10.2017	CSI-W5*	4	1.40 - 1.60	410	219	13	13.05.2005	G	SWB	110	2	21	99	28	1384	56	F	16	13	no	spread	oxer	straight	no	0	0	68,04	no
Oslo	Western European League CSI-W5* round B	15.10.2017	CSI-W5*	4	1.40 - 1.60	295	219	13	13.05.2005	G	SWB	110	2	21	99	28	1384	56	F	1	1	no	vertical	vertical	right	no	0	0	39,27	no
Oslo	Western European League CSI-W5* round B	15.10.2017	CSI-W5*	4	1.40 - 1.60	295	219	13	13.05.2005	G	SWB	110	2	21	99	28	1384	56	F	2	2	no	spread	oxer	right	no	0	0	39,27	no
Oslo	Western European League CSI-W5* round B	15.10.2017	CSI-W5*	4	1.40 - 1.60	295	219	13	13.05.2005	G	SWB	110	2	21	99	28	1384	56	F	3	3	no	vertical	vertical	right	no	0	0	39,27	no
Oslo	Western European League CSI-W5* round B	15.10.2017	CSI-W5*	4	1.40 - 1.60	295	219	13	13.05.2005	G	SWB	110	2	21	99	28	1384	56	F	4	4	no	spread	oxer	left	no	0	0	39,27	no
Oslo	Western European League CSI-W5* round B	15.10.2017	CSI-W5*	4	1.40 - 1.60	295	219	13	13.05.2005	G	SWB	110	2	21	99	28	1384	56	F	5	5	no	vertical	vertical	left	no	0	0	39,27	no
Oslo	Western European League CSI-W5* round B	15.10.2017	CSI-W5*	4	1.40 - 1.60	295	219	13	13.05.2005	G	SWB	110	2	21	99	28	1384	56	F	6	6a	yes	vertical	vertical	straight	no	0	0	39,27	no
Oslo	Western European League CSI-W5* round B	15.10.2017	CSI-W5*	4	1.40 - 1.60	295	219	13	13.05.2005	G	SWB	110	2	21	99	28	1384	56	F	7	6b	yes	spread	oxer	straight	no	0	0	39,27	no
Oslo	Western European League CSI-W5* round B	15.10.2017	CSI-W5*	4	1.40 - 1.60	295	219	13	13.05.2005	G	SWB	110	2	21	99	28	1384	56	F	8	7	no	spread	oxer	right	no	0	0	39,27	no
Oslo	Western European League CSI-W5* round B	15.10.2017	CSI-W5*	4	1.40 - 1.60	295	219	13	13.05.2005	G	SWB	110	2	21	99	28	1384	56	F	9	8	no	vertical	vertical	right	no	0	0	39,27	no
Oslo	Western European League CSI-W5* round A	15.10.2017	CSI-W5*	4	1.40 - 1.60	410	218	12	24.05.2006	M	HOLST	115	0	22	144	55	798	31	M	1	1	no	spread	oxer	right	no	0	0	67,93	no
Oslo	Western European League CSI-W5* round A	15.10.2017	CSI-W5*	4	1.40 - 1.60	410	218	12	24.05.2006	M	HOLST	115	0	22	144	55	798	31	M	2	2	no	vertical	vertical	right	no	0	0	67,93	no
Oslo	Western European League CSI-W5* round A	15.10.2017	CSI-W5*	4	1.40 - 1.60	410	218	12	24.05.2006	M	HOLST	115	0	22	144	55	798	31	M	3	3	no	vertical	vertical	left	yes	4	0	67,93	no
Oslo	Western European League CSI-W5* round A	15.10.2017	CSI-W5*	4	1.40 - 1.60	410	218	12	24.05.2006	M	HOLST	115	0	22	144	55	798	31	M	4	4a	yes	vertical	vertical	straight	no	0	0	67,93	no
Oslo	Western European League CSI-W5* round A	15.10.2017	CSI-W5*	4	1.40 - 1.60	410	218	12	24.05.2006	M	HOLST	115	0	22	144	55	798	31	M	5	4b	yes	spread	oxer	straight	no	0	0	67,93	no
Oslo	Western European League CSI-W5* round A	15.10.2017	CSI-W5*	4	1.40 - 1.60	410	218	12	24.05.2006	M	HOLST	115	0	22	144	55	798	31	M	6	5	no	vertical	vertical with water	straight	no	0	0	67,93	no
Oslo	Western European League CSI-W5* round A	15.10.2017	CSI-W5*	4	1.40 - 1.60	410	218	12	24.05.2006	M	HOLST	115	0	22	144	55	798	31	M	7	6a	yes	spread	oxer	right	no	0	0	67,93	no
Oslo	Western European League CSI-W5* round A	15.10.2017	CSI-W5*	4	1.40 - 1.60	410	218	12	24.05.2006	M	HOLST	115	0	22	144	55	798	31	M	8	6b	yes	vertical	vertical	right	no	0	0	67,93	no
Oslo	Western European League CSI-W5* round A	15.10.2017	CSI-W5*	4	1.40 - 1.60	410	218	12	24.05.2006	M	HOLST	115	0	22	144	55	798	31	M	9	6c	yes	spread	oxer	right	no	0	0	67,93	no
Oslo	Western European League CSI-W5* round A	15.10.2017	CSI-W5*	4	1.40 - 1.60	410	218	12	24.05.2006	M	HOLST	115	0	22	144	55	798	31	M	10	7	no	vertical	vertical	right	no	0	0	67,93	no
Oslo	Western European League CSI-W5* round A	15.10.2017	CSI-W5*	4	1.40 - 1.60	410	218	12	24.05.2006	M	HOLST	115	0	22	144	55	798	31	M	11	8	no	spread	oxer	left	no	0	0	67,93	no
Oslo	Western European League CSI-W5* round A	15.10.2017	CSI-W5*	4	1.40 - 1.60	410	218	12	24.05.2006	M	HOLST	115	0	22	144	55	798	31	M	12	9	no	vertical	vertical	straight	no	0	0	67,93	no
Oslo	Western European League CSI-W5* round A	15.10.2017	CSI-W5*	4	1.40 - 1.60	410	218	12	24.05.2006	M	HOLST	115	0	22	144	55	798	31	M	13	10	no	spread	oxer	right	no	0	0	67,93	no
Oslo	Western European League CSI-W5* round A	15.10.2017	CSI-W5*	4	1.40 - 1.60	410	218	12	24.05.2006	M	HOLST	115	0	22	144	55	798	31	M	14	11	no	vertical	vertical	right	no	0	0	67,93	no
Oslo	Western European League CSI-W5* round A	15.10.2017	CSI-W5*	4	1.40 - 1.60	410	218	12	24.05.2006	M	HOLST	115	0	22	144	55	798	31	M	15	12	no	spread	oxer	left	no	0	0	67,93	no
Oslo	Western European League CSI-W5* round A	15.10.2017	CSI-W5*	4	1.40 - 1.60	410	218	12	24.05.2006	M	HOLST	115	0	22	144	55	798	31	M	16	13	no	spread	oxer	straight	no	0	0	67,93	no
Oslo	Western European League CSI-W5* round A	15.10.2017	CSI-W5*	4	1.40 - 1.60	410	96	12	24.06.2006	M	BWP	140	16	23	11	35	1800	65	M	1	1	no	spread	oxer	right	no	0	0	67,92	no
Oslo	Western European League CSI-W5* round A	15.10.2017	CSI-W5*	4	1.40 - 1.60	410	96	12	24.06.2006	M	BWP	140	16	23	11	35	1800	65	M	2	2	no	vertical	vertical	right	no	0	0	67,92	no
Oslo	Western European League CSI-W5* round A	15.10.2017	CSI-W5*	4	1.40 - 1.60	410	96	12	24.06.2006	M	BWP	140	16	23	11	35	1800	65	M	3	3	no	vertical	vertical	left	no	0	0	67,92	no
Oslo	Western European League CSI-W5* round A	15.10.2017	CSI-W5*	4	1.40 - 1.60	410	96	12	24.06.2006	M	BWP	140	16	23	11	35	1800	65	M	4	4a	yes	vertical	vertical	straight	no	0	0	67,92	no
Oslo	Western European League CSI-W5* round A	15.10.2017	CSI-W5*	4	1.40 - 1.60	410	96	12	24.06.2006	M	BWP	140	16	23	11	35	1800	65	M	5	4b	yes	spread	oxer	straight	no	0	0	67,92	no
Oslo	Western European League CSI-W5* round A	15.10.2017	CSI-W5*	4	1.40 - 1.60	410	96	12	24.06.2006	M	BWP	140	16	23	11	35	1800	65	M	6	5	no	vertical	vertical with water	straight	no	0	0	67,92	no
Oslo	Western European League CSI-W5* round A	15.10.2017	CSI-W5*	4	1.40 - 1.60	410	96	12	24.06.2006	M	BWP	140	16	23	11	35	1800	65	M	7	6a	yes	spread	oxer	right	no	0	0	67,92	no
Oslo	Western European League CSI-W5* round A	15.10.2017	CSI-W5*	4	1.40 - 1.60	410	96	12	24.06.2006	M	BWP	140	16	23	11	35	1800	65	M	8	6b	yes	vertical	vertical	right	no	0	0	67,92	no
Oslo	Western European League CSI-W5* round A	15.10.2017	CSI-W5*	4	1.40 - 1.60	410	96	12	24.06.2006	M	BWP	140	16	23	11	35	1800	65	M	9	6c	yes	spread	oxer	right	no	0	0	67,92	no
Oslo	Western European League CSI-W5* round A	15.10.2017	CSI-W5*	4	1.40 - 1.60	410	96	12	24.06.2006	M	BWP	140	16	23	11	35	1800	65	M	10	7	no	vertical	vertical	right	yes	4	0	67,92	no
Oslo	Western European League CSI-W5* round A	15.10.2017	CSI-W5*	4	1.40 - 1.60	410	96	12	24.06.2006	M	BWP	140	16	23	11	35	1800	65	M	11	8	no	spread	oxer	left	no	0	0	67,92	no
Oslo	Western European League CSI-W5* round A	15.10.2017	CSI-W5*	4	1.40 - 1.60	410	96	12	24.06.2006	M	BWP	140	16	23	11	35	1800	65	M	12	9	no	vertical	vertical	straight	no	0	0	67,92	no
Oslo	Western European League CSI-W5* round A	15.10.2017	CSI-W5*	4	1.40 - 1.60	410	96	12	24.06.2006	M	BWP	140	16	23	11	35	1800	65	M	13	10	no	spread	oxer	right	no	0	0	67,92	no
Oslo	Western European League CSI-W5* round A	15.10.2017	CSI-W5*	4	1.40 - 1.60	410	96	12	24.06.2006	M	BWP	140	16	23	11	35	1800	65	M	14	11	no	vertical	vertical	right	no	0	0	67,92	no
Oslo	Western European League CSI-W5* round A	15.10.2017	CSI-W5*	4	1.40 - 1.60	410	96	12	24.06.2006	M	BWP	140	16	23	11	35	1800	65	M	15	12	no	spread	oxer	left	no	0	0	67,92	no
Oslo	Western European League CSI-W5* round A	15.10.2017	CSI-W5*	4	1.40 - 1.60	410	96	12	24.06.2006	M	BWP	140	16	23	11	35	1800	65	M	16	13	no	spread	oxer	straight	no	0	0	67,92	no
Oslo	Western European League CSI-W5* round A	15.10.2017	CSI-W5*	4	1.40 - 1.60	410	162	12	01.01.2006	M	MIPAAF	146	3	24	287	49	641	15	M	1	1	no	spread	oxer	right	no	0	0	67,88	no
Oslo	Western European League CSI-W5* round A	15.10.2017	CSI-W5*	4	1.40 - 1.60	410	162	12	01.01.2006	M	MIPAAF	146	3	24	287	49	641	15	M	2	2	no	vertical	vertical	right	no	0	0	67,88	no
Oslo	Western European League CSI-W5* round A	15.10.2017	CSI-W5*	4	1.40 - 1.60	410	162	12	01.01.2006	M	MIPAAF	146	3	24	287	49	641	15	M	3	3	no	vertical	vertical	left	no	0	0	67,88	no
Oslo	Western European League CSI-W5* round A	15.10.2017	CSI-W5*	4	1.40 - 1.60	410	162	12	01.01.2006	M	MIPAAF	146	3	24	287	49	641	15	M	4	4a	yes	vertical	vertical	straight	yes	4	0	67,88	no
Oslo	Western European League CSI-W5* round A	15.10.2017	CSI-W5*	4	1.40 - 1.60	410	162	12	01.01.2006	M	MIPAAF	146	3	24	287	49	641	15	M	5	4b	yes	spread	oxer	straight	no	0	0	67,88	no
Oslo	Western European League CSI-W5* round A	15.10.2017	CSI-W5*	4	1.40 - 1.60	410	162	12	01.01.2006	M	MIPAAF	146	3	24	287	49	641	15	M	6	5	no	vertical	vertical with water	straight	no	0	0	67,88	no
Oslo	Western European League CSI-W5* round A	15.10.2017	CSI-W5*	4	1.40 - 1.60	410	162	12	01.01.2006	M	MIPAAF	146	3	24	287	49	641	15	M	7	6a	yes	spread	oxer	right	no	0	0	67,88	no
Oslo	Western European League CSI-W5* round A	15.10.2017	CSI-W5*	4	1.40 - 1.60	410	162	12	01.01.2006	M	MIPAAF	146	3	24	287	49	641	15	M	8	6b	yes	vertical	vertical	right	no	0	0	67,88	no
Oslo	Western European League CSI-W5* round A	15.10.2017	CSI-W5*	4	1.40 - 1.60	410	162	12	01.01.2006	M	MIPAAF	146	3	24	287	49	641	15	M	9	6c	yes	spread	oxer	right	no	0	0	67,88	no
Oslo	Western European League CSI-W5* round A	15.10.2017	CSI-W5*	4	1.40 - 1.60	410	162	12	01.01.2006	M	MIPAAF	146	3	24	287	49	641	15	M	10	7	no	vertical	vertical	right	no	0	0	67,88	no
Oslo	Western European League CSI-W5* round A	15.10.2017	CSI-W5*	4	1.40 - 1.60	410	162	12	01.01.2006	M	MIPAAF	146	3	24	287	49	641	15	M	11	8	no	spread	oxer	left	no	0	0	67,88	no
Oslo	Western European League CSI-W5* round A	15.10.2017	CSI-W5*	4	1.40 - 1.60	410	162	12	01.01.2006	M	MIPAAF	146	3	24	287	49	641	15	M	12	9	no	vertical	vertical	straight	yes	4	0	67,88	no
Oslo	Western European League CSI-W5* round A	15.10.2017	CSI-W5*	4	1.40 - 1.60	410	162	12	01.01.2006	M	MIPAAF	146	3	24	287	49	641	15	M	13	10	no	spread	oxer	right	no	0	0	67,88	no
Oslo	Western European League CSI-W5* round A	15.10.2017	CSI-W5*	4	1.40 - 1.60	410	162	12	01.01.2006	M	MIPAAF	146	3	24	287	49	641	15	M	14	11	no	vertical	vertical	right	no	0	0	67,88	no
Oslo	Western European League CSI-W5* round A	15.10.2017	CSI-W5*	4	1.40 - 1.60	410	162	12	01.01.2006	M	MIPAAF	146	3	24	287	49	641	15	M	15	12	no	spread	oxer	left	no	0	0	67,88	no
Oslo	Western European League CSI-W5* round A	15.10.2017	CSI-W5*	4	1.40 - 1.60	410	162	12	01.01.2006	M	MIPAAF	146	3	24	287	49	641	15	M	16	13	no	spread	oxer	straight	no	0	0	67,88	no
Oslo	Western European League CSI-W5* round A	15.10.2017	CSI-W5*	4	1.40 - 1.60	410	5	13	31.03.2005	G	KWPN	238	4	25	558	26	820	16	F	1	1	no	spread	oxer	right	no	0	0	67,8	no
Oslo	Western European League CSI-W5* round A	15.10.2017	CSI-W5*	4	1.40 - 1.60	410	5	13	31.03.2005	G	KWPN	238	4	25	558	26	820	16	F	2	2	no	vertical	vertical	right	yes	4	0	67,8	no
Oslo	Western European League CSI-W5* round A	15.10.2017	CSI-W5*	4	1.40 - 1.60	410	5	13	31.03.2005	G	KWPN	238	4	25	558	26	820	16	F	3	3	no	vertical	vertical	left	no	0	0	67,8	no
Oslo	Western European League CSI-W5* round A	15.10.2017	CSI-W5*	4	1.40 - 1.60	410	5	13	31.03.2005	G	KWPN	238	4	25	558	26	820	16	F	4	4a	yes	vertical	vertical	straight	no	0	0	67,8	no
Oslo	Western European League CSI-W5* round A	15.10.2017	CSI-W5*	4	1.40 - 1.60	410	5	13	31.03.2005	G	KWPN	238	4	25	558	26	820	16	F	5	4b	yes	spread	oxer	straight	no	0	0	67,8	no
Oslo	Western European League CSI-W5* round A	15.10.2017	CSI-W5*	4	1.40 - 1.60	410	5	13	31.03.2005	G	KWPN	238	4	25	558	26	820	16	F	6	5	no	vertical	vertical with water	straight	no	0	0	67,8	no
Oslo	Western European League CSI-W5* round A	15.10.2017	CSI-W5*	4	1.40 - 1.60	410	5	13	31.03.2005	G	KWPN	238	4	25	558	26	820	16	F	7	6a	yes	spread	oxer	right	no	0	0	67,8	no
Oslo	Western European League CSI-W5* round A	15.10.2017	CSI-W5*	4	1.40 - 1.60	410	5	13	31.03.2005	G	KWPN	238	4	25	558	26	820	16	F	8	6b	yes	vertical	vertical	right	no	0	0	67,8	no
Oslo	Western European League CSI-W5* round A	15.10.2017	CSI-W5*	4	1.40 - 1.60	410	5	13	31.03.2005	G	KWPN	238	4	25	558	26	820	16	F	9	6c	yes	spread	oxer	right	no	0	0	67,8	no
Oslo	Western European League CSI-W5* round A	15.10.2017	CSI-W5*	4	1.40 - 1.60	410	5	13	31.03.2005	G	KWPN	238	4	25	558	26	820	16	F	10	7	no	vertical	vertical	right	no	0	0	67,8	no
Oslo	Western European League CSI-W5* round A	15.10.2017	CSI-W5*	4	1.40 - 1.60	410	5	13	31.03.2005	G	KWPN	238	4	25	558	26	820	16	F	11	8	no	spread	oxer	left	no	0	0	67,8	no
Oslo	Western European League CSI-W5* round A	15.10.2017	CSI-W5*	4	1.40 - 1.60	410	5	13	31.03.2005	G	KWPN	238	4	25	558	26	820	16	F	12	9	no	vertical	vertical	straight	no	0	0	67,8	no
Oslo	Western European League CSI-W5* round A	15.10.2017	CSI-W5*	4	1.40 - 1.60	410	5	13	31.03.2005	G	KWPN	238	4	25	558	26	820	16	F	13	10	no	spread	oxer	right	no	0	0	67,8	no
Oslo	Western European League CSI-W5* round A	15.10.2017	CSI-W5*	4	1.40 - 1.60	410	5	13	31.03.2005	G	KWPN	238	4	25	558	26	820	16	F	14	11	no	vertical	vertical	right	no	0	0	67,8	no
Oslo	Western European League CSI-W5* round A	15.10.2017	CSI-W5*	4	1.40 - 1.60	410	5	13	31.03.2005	G	KWPN	238	4	25	558	26	820	16	F	15	12	no	spread	oxer	left	no	0	0	67,8	no
Oslo	Western European League CSI-W5* round A	15.10.2017	CSI-W5*	4	1.40 - 1.60	410	5	13	31.03.2005	G	KWPN	238	4	25	558	26	820	16	F	16	13	no	spread	oxer	straight	no	0	0	67,8	no
Oslo	Western European League CSI-W5* round A	15.10.2017	CSI-W5*	4	1.40 - 1.60	410	127	10	03.07.2008	M	BWP	131	3	26	14	30	1957	97	M	1	1	no	spread	oxer	right	no	0	0	67,59	no
Oslo	Western European League CSI-W5* round A	15.10.2017	CSI-W5*	4	1.40 - 1.60	410	127	10	03.07.2008	M	BWP	131	3	26	14	30	1957	97	M	2	2	no	vertical	vertical	right	yes	4	0	67,59	no
Oslo	Western European League CSI-W5* round A	15.10.2017	CSI-W5*	4	1.40 - 1.60	410	127	10	03.07.2008	M	BWP	131	3	26	14	30	1957	97	M	3	3	no	vertical	vertical	left	no	0	0	67,59	no
Oslo	Western European League CSI-W5* round A	15.10.2017	CSI-W5*	4	1.40 - 1.60	410	127	10	03.07.2008	M	BWP	131	3	26	14	30	1957	97	M	4	4a	yes	vertical	vertical	straight	no	0	0	67,59	no
Oslo	Western European League CSI-W5* round A	15.10.2017	CSI-W5*	4	1.40 - 1.60	410	127	10	03.07.2008	M	BWP	131	3	26	14	30	1957	97	M	5	4b	yes	spread	oxer	straight	no	0	0	67,59	no
Oslo	Western European League CSI-W5* round A	15.10.2017	CSI-W5*	4	1.40 - 1.60	410	127	10	03.07.2008	M	BWP	131	3	26	14	30	1957	97	M	6	5	no	vertical	vertical with water	straight	no	0	0	67,59	no
Oslo	Western European League CSI-W5* round A	15.10.2017	CSI-W5*	4	1.40 - 1.60	410	127	10	03.07.2008	M	BWP	131	3	26	14	30	1957	97	M	7	6a	yes	spread	oxer	right	no	0	0	67,59	no
Oslo	Western European League CSI-W5* round A	15.10.2017	CSI-W5*	4	1.40 - 1.60	410	127	10	03.07.2008	M	BWP	131	3	26	14	30	1957	97	M	8	6b	yes	vertical	vertical	right	no	0	0	67,59	no
Oslo	Western European League CSI-W5* round A	15.10.2017	CSI-W5*	4	1.40 - 1.60	410	127	10	03.07.2008	M	BWP	131	3	26	14	30	1957	97	M	9	6c	yes	spread	oxer	right	no	0	0	67,59	no
Oslo	Western European League CSI-W5* round A	15.10.2017	CSI-W5*	4	1.40 - 1.60	410	127	10	03.07.2008	M	BWP	131	3	26	14	30	1957	97	M	10	7	no	vertical	vertical	right	no	0	0	67,59	no
Oslo	Western European League CSI-W5* round A	15.10.2017	CSI-W5*	4	1.40 - 1.60	410	127	10	03.07.2008	M	BWP	131	3	26	14	30	1957	97	M	11	8	no	spread	oxer	left	no	0	0	67,59	no
Oslo	Western European League CSI-W5* round A	15.10.2017	CSI-W5*	4	1.40 - 1.60	410	127	10	03.07.2008	M	BWP	131	3	26	14	30	1957	97	M	12	9	no	vertical	vertical	straight	no	0	0	67,59	no
Oslo	Western European League CSI-W5* round A	15.10.2017	CSI-W5*	4	1.40 - 1.60	410	127	10	03.07.2008	M	BWP	131	3	26	14	30	1957	97	M	13	10	no	spread	oxer	right	no	0	0	67,59	no
Oslo	Western European League CSI-W5* round A	15.10.2017	CSI-W5*	4	1.40 - 1.60	410	127	10	03.07.2008	M	BWP	131	3	26	14	30	1957	97	M	14	11	no	vertical	vertical	right	no	0	0	67,59	no
Oslo	Western European League CSI-W5* round A	15.10.2017	CSI-W5*	4	1.40 - 1.60	410	127	10	03.07.2008	M	BWP	131	3	26	14	30	1957	97	M	15	12	no	spread	oxer	left	no	0	0	67,59	no
Oslo	Western European League CSI-W5* round A	15.10.2017	CSI-W5*	4	1.40 - 1.60	410	127	10	03.07.2008	M	BWP	131	3	26	14	30	1957	97	M	16	13	no	spread	oxer	straight	no	0	0	67,59	no
Oslo	Western European League CSI-W5* round A	15.10.2017	CSI-W5*	4	1.40 - 1.60	410	220	13	01.01.2005	M	MIPAAF	153	14	27	269	20	1104	42	F	1	1	no	spread	oxer	right	no	0	0	67,52	no
Oslo	Western European League CSI-W5* round A	15.10.2017	CSI-W5*	4	1.40 - 1.60	410	220	13	01.01.2005	M	MIPAAF	153	14	27	269	20	1104	42	F	2	2	no	vertical	vertical	right	no	0	0	67,52	no
Oslo	Western European League CSI-W5* round A	15.10.2017	CSI-W5*	4	1.40 - 1.60	410	220	13	01.01.2005	M	MIPAAF	153	14	27	269	20	1104	42	F	3	3	no	vertical	vertical	left	no	0	0	67,52	no
Oslo	Western European League CSI-W5* round A	15.10.2017	CSI-W5*	4	1.40 - 1.60	410	220	13	01.01.2005	M	MIPAAF	153	14	27	269	20	1104	42	F	4	4a	yes	vertical	vertical	straight	no	0	0	67,52	no
Oslo	Western European League CSI-W5* round A	15.10.2017	CSI-W5*	4	1.40 - 1.60	410	220	13	01.01.2005	M	MIPAAF	153	14	27	269	20	1104	42	F	5	4b	yes	spread	oxer	straight	no	0	0	67,52	no
Oslo	Western European League CSI-W5* round A	15.10.2017	CSI-W5*	4	1.40 - 1.60	410	220	13	01.01.2005	M	MIPAAF	153	14	27	269	20	1104	42	F	6	5	no	vertical	vertical with water	straight	no	0	0	67,52	no
Oslo	Western European League CSI-W5* round A	15.10.2017	CSI-W5*	4	1.40 - 1.60	410	220	13	01.01.2005	M	MIPAAF	153	14	27	269	20	1104	42	F	7	6a	yes	spread	oxer	right	no	0	0	67,52	no
Oslo	Western European League CSI-W5* round A	15.10.2017	CSI-W5*	4	1.40 - 1.60	410	220	13	01.01.2005	M	MIPAAF	153	14	27	269	20	1104	42	F	8	6b	yes	vertical	vertical	right	no	0	0	67,52	no
Oslo	Western European League CSI-W5* round A	15.10.2017	CSI-W5*	4	1.40 - 1.60	410	220	13	01.01.2005	M	MIPAAF	153	14	27	269	20	1104	42	F	9	6c	yes	spread	oxer	right	no	0	0	67,52	no
Oslo	Western European League CSI-W5* round A	15.10.2017	CSI-W5*	4	1.40 - 1.60	410	220	13	01.01.2005	M	MIPAAF	153	14	27	269	20	1104	42	F	10	7	no	vertical	vertical	right	no	0	0	67,52	no
Oslo	Western European League CSI-W5* round A	15.10.2017	CSI-W5*	4	1.40 - 1.60	410	220	13	01.01.2005	M	MIPAAF	153	14	27	269	20	1104	42	F	11	8	no	spread	oxer	left	no	0	0	67,52	no
Oslo	Western European League CSI-W5* round A	15.10.2017	CSI-W5*	4	1.40 - 1.60	410	220	13	01.01.2005	M	MIPAAF	153	14	27	269	20	1104	42	F	12	9	no	vertical	vertical	straight	yes	4	0	67,52	no
Oslo	Western European League CSI-W5* round A	15.10.2017	CSI-W5*	4	1.40 - 1.60	410	220	13	01.01.2005	M	MIPAAF	153	14	27	269	20	1104	42	F	13	10	no	spread	oxer	right	no	0	0	67,52	no
Oslo	Western European League CSI-W5* round A	15.10.2017	CSI-W5*	4	1.40 - 1.60	410	220	13	01.01.2005	M	MIPAAF	153	14	27	269	20	1104	42	F	14	11	no	vertical	vertical	right	no	0	0	67,52	no
Oslo	Western European League CSI-W5* round A	15.10.2017	CSI-W5*	4	1.40 - 1.60	410	220	13	01.01.2005	M	MIPAAF	153	14	27	269	20	1104	42	F	15	12	no	spread	oxer	left	no	0	0	67,52	no
Oslo	Western European League CSI-W5* round A	15.10.2017	CSI-W5*	4	1.40 - 1.60	410	220	13	01.01.2005	M	MIPAAF	153	14	27	269	20	1104	42	F	16	13	no	spread	oxer	straight	no	0	0	67,52	no
Oslo	Western European League CSI-W5* round A	15.10.2017	CSI-W5*	4	1.40 - 1.60	410	92	13	20.03.2005	G	BWP	140	4	28	4	38	2170	90	M	1	1	no	spread	oxer	right	no	0	0	67,02	no
Oslo	Western European League CSI-W5* round A	15.10.2017	CSI-W5*	4	1.40 - 1.60	410	92	13	20.03.2005	G	BWP	140	4	28	4	38	2170	90	M	2	2	no	vertical	vertical	right	no	0	0	67,02	no
Oslo	Western European League CSI-W5* round A	15.10.2017	CSI-W5*	4	1.40 - 1.60	410	92	13	20.03.2005	G	BWP	140	4	28	4	38	2170	90	M	3	3	no	vertical	vertical	left	no	0	0	67,02	no
Oslo	Western European League CSI-W5* round A	15.10.2017	CSI-W5*	4	1.40 - 1.60	410	92	13	20.03.2005	G	BWP	140	4	28	4	38	2170	90	M	4	4a	yes	vertical	vertical	straight	no	0	0	67,02	no
Oslo	Western European League CSI-W5* round A	15.10.2017	CSI-W5*	4	1.40 - 1.60	410	92	13	20.03.2005	G	BWP	140	4	28	4	38	2170	90	M	5	4b	yes	spread	oxer	straight	no	0	0	67,02	no
Oslo	Western European League CSI-W5* round A	15.10.2017	CSI-W5*	4	1.40 - 1.60	410	92	13	20.03.2005	G	BWP	140	4	28	4	38	2170	90	M	6	5	no	vertical	vertical with water	straight	no	0	0	67,02	no
Oslo	Western European League CSI-W5* round A	15.10.2017	CSI-W5*	4	1.40 - 1.60	410	92	13	20.03.2005	G	BWP	140	4	28	4	38	2170	90	M	7	6a	yes	spread	oxer	right	no	0	0	67,02	no
Oslo	Western European League CSI-W5* round A	15.10.2017	CSI-W5*	4	1.40 - 1.60	410	92	13	20.03.2005	G	BWP	140	4	28	4	38	2170	90	M	8	6b	yes	vertical	vertical	right	no	0	0	67,02	no
Oslo	Western European League CSI-W5* round A	15.10.2017	CSI-W5*	4	1.40 - 1.60	410	92	13	20.03.2005	G	BWP	140	4	28	4	38	2170	90	M	9	6c	yes	spread	oxer	right	no	0	0	67,02	no
Oslo	Western European League CSI-W5* round A	15.10.2017	CSI-W5*	4	1.40 - 1.60	410	92	13	20.03.2005	G	BWP	140	4	28	4	38	2170	90	M	10	7	no	vertical	vertical	right	no	0	0	67,02	no
Oslo	Western European League CSI-W5* round A	15.10.2017	CSI-W5*	4	1.40 - 1.60	410	92	13	20.03.2005	G	BWP	140	4	28	4	38	2170	90	M	11	8	no	spread	oxer	left	no	0	0	67,02	no
Oslo	Western European League CSI-W5* round A	15.10.2017	CSI-W5*	4	1.40 - 1.60	410	92	13	20.03.2005	G	BWP	140	4	28	4	38	2170	90	M	12	9	no	vertical	vertical	straight	no	0	0	67,02	no
Oslo	Western European League CSI-W5* round A	15.10.2017	CSI-W5*	4	1.40 - 1.60	410	92	13	20.03.2005	G	BWP	140	4	28	4	38	2170	90	M	13	10	no	spread	oxer	right	no	0	0	67,02	no
Oslo	Western European League CSI-W5* round A	15.10.2017	CSI-W5*	4	1.40 - 1.60	410	92	13	20.03.2005	G	BWP	140	4	28	4	38	2170	90	M	14	11	no	vertical	vertical	right	no	0	0	67,02	no
Oslo	Western European League CSI-W5* round A	15.10.2017	CSI-W5*	4	1.40 - 1.60	410	92	13	20.03.2005	G	BWP	140	4	28	4	38	2170	90	M	15	12	no	spread	oxer	left	no	0	0	67,02	no
Oslo	Western European League CSI-W5* round A	15.10.2017	CSI-W5*	4	1.40 - 1.60	410	92	13	20.03.2005	G	BWP	140	4	28	4	38	2170	90	M	16	13	no	spread	oxer	straight	no	0	0	67,02	no
Oslo	Western European League CSI-W5* round B	15.10.2017	CSI-W5*	4	1.40 - 1.60	295	92	13	20.03.2005	G	BWP	140	4	28	4	38	2170	90	M	1	1	no	vertical	vertical	right	no	0	0	36,96	no
Oslo	Western European League CSI-W5* round B	15.10.2017	CSI-W5*	4	1.40 - 1.60	295	92	13	20.03.2005	G	BWP	140	4	28	4	38	2170	90	M	2	2	no	spread	oxer	right	no	0	0	36,96	no
Oslo	Western European League CSI-W5* round B	15.10.2017	CSI-W5*	4	1.40 - 1.60	295	92	13	20.03.2005	G	BWP	140	4	28	4	38	2170	90	M	3	3	no	vertical	vertical	right	no	0	0	36,96	no
Oslo	Western European League CSI-W5* round B	15.10.2017	CSI-W5*	4	1.40 - 1.60	295	92	13	20.03.2005	G	BWP	140	4	28	4	38	2170	90	M	4	4	no	spread	oxer	left	no	0	0	36,96	no
Oslo	Western European League CSI-W5* round B	15.10.2017	CSI-W5*	4	1.40 - 1.60	295	92	13	20.03.2005	G	BWP	140	4	28	4	38	2170	90	M	5	5	no	vertical	vertical	left	no	0	0	36,96	no
Oslo	Western European League CSI-W5* round B	15.10.2017	CSI-W5*	4	1.40 - 1.60	295	92	13	20.03.2005	G	BWP	140	4	28	4	38	2170	90	M	6	6a	yes	vertical	vertical	straight	no	0	0	36,96	no
Oslo	Western European League CSI-W5* round B	15.10.2017	CSI-W5*	4	1.40 - 1.60	295	92	13	20.03.2005	G	BWP	140	4	28	4	38	2170	90	M	7	6b	yes	spread	oxer	straight	no	0	0	36,96	no
Oslo	Western European League CSI-W5* round B	15.10.2017	CSI-W5*	4	1.40 - 1.60	295	92	13	20.03.2005	G	BWP	140	4	28	4	38	2170	90	M	8	7	no	spread	oxer	right	no	0	0	36,96	no
Oslo	Western European League CSI-W5* round B	15.10.2017	CSI-W5*	4	1.40 - 1.60	295	92	13	20.03.2005	G	BWP	140	4	28	4	38	2170	90	M	9	8	no	vertical	vertical	right	no	0	0	36,96	no
Oslo	Western European League CSI-W5* round A	15.10.2017	CSI-W5*	4	1.40 - 1.60	410	35	15	22.05.2003	G	HOLST	368	19	29	89	35	1502	55	M	1	1	no	spread	oxer	right	no	0	0	67,02	no
Oslo	Western European League CSI-W5* round A	15.10.2017	CSI-W5*	4	1.40 - 1.60	410	35	15	22.05.2003	G	HOLST	368	19	29	89	35	1502	55	M	2	2	no	vertical	vertical	right	no	0	0	67,02	no
Oslo	Western European League CSI-W5* round A	15.10.2017	CSI-W5*	4	1.40 - 1.60	410	35	15	22.05.2003	G	HOLST	368	19	29	89	35	1502	55	M	3	3	no	vertical	vertical	left	yes	4	0	67,02	no
Oslo	Western European League CSI-W5* round A	15.10.2017	CSI-W5*	4	1.40 - 1.60	410	35	15	22.05.2003	G	HOLST	368	19	29	89	35	1502	55	M	4	4a	yes	vertical	vertical	straight	no	0	0	67,02	no
Oslo	Western European League CSI-W5* round A	15.10.2017	CSI-W5*	4	1.40 - 1.60	410	35	15	22.05.2003	G	HOLST	368	19	29	89	35	1502	55	M	5	4b	yes	spread	oxer	straight	no	0	0	67,02	no
Oslo	Western European League CSI-W5* round A	15.10.2017	CSI-W5*	4	1.40 - 1.60	410	35	15	22.05.2003	G	HOLST	368	19	29	89	35	1502	55	M	6	5	no	vertical	vertical with water	straight	no	0	0	67,02	no
Oslo	Western European League CSI-W5* round A	15.10.2017	CSI-W5*	4	1.40 - 1.60	410	35	15	22.05.2003	G	HOLST	368	19	29	89	35	1502	55	M	7	6a	yes	spread	oxer	right	no	0	0	67,02	no
Oslo	Western European League CSI-W5* round A	15.10.2017	CSI-W5*	4	1.40 - 1.60	410	35	15	22.05.2003	G	HOLST	368	19	29	89	35	1502	55	M	8	6b	yes	vertical	vertical	right	no	0	0	67,02	no
Oslo	Western European League CSI-W5* round A	15.10.2017	CSI-W5*	4	1.40 - 1.60	410	35	15	22.05.2003	G	HOLST	368	19	29	89	35	1502	55	M	9	6c	yes	spread	oxer	right	no	0	0	67,02	no
Oslo	Western European League CSI-W5* round A	15.10.2017	CSI-W5*	4	1.40 - 1.60	410	35	15	22.05.2003	G	HOLST	368	19	29	89	35	1502	55	M	10	7	no	vertical	vertical	right	no	0	0	67,02	no
Oslo	Western European League CSI-W5* round A	15.10.2017	CSI-W5*	4	1.40 - 1.60	410	35	15	22.05.2003	G	HOLST	368	19	29	89	35	1502	55	M	11	8	no	spread	oxer	left	no	0	0	67,02	no
Oslo	Western European League CSI-W5* round A	15.10.2017	CSI-W5*	4	1.40 - 1.60	410	35	15	22.05.2003	G	HOLST	368	19	29	89	35	1502	55	M	12	9	no	vertical	vertical	straight	no	0	0	67,02	no
Oslo	Western European League CSI-W5* round A	15.10.2017	CSI-W5*	4	1.40 - 1.60	410	35	15	22.05.2003	G	HOLST	368	19	29	89	35	1502	55	M	13	10	no	spread	oxer	right	no	0	0	67,02	no
Oslo	Western European League CSI-W5* round A	15.10.2017	CSI-W5*	4	1.40 - 1.60	410	35	15	22.05.2003	G	HOLST	368	19	29	89	35	1502	55	M	14	11	no	vertical	vertical	right	no	0	0	67,02	no
Oslo	Western European League CSI-W5* round A	15.10.2017	CSI-W5*	4	1.40 - 1.60	410	35	15	22.05.2003	G	HOLST	368	19	29	89	35	1502	55	M	15	12	no	spread	oxer	left	no	0	0	67,02	no
Oslo	Western European League CSI-W5* round A	15.10.2017	CSI-W5*	4	1.40 - 1.60	410	35	15	22.05.2003	G	HOLST	368	19	29	89	35	1502	55	M	16	13	no	spread	oxer	straight	no	0	0	67,02	no
Oslo	Western European League CSI-W5* round A	15.10.2017	CSI-W5*	4	1.40 - 1.60	410	108	11	24.03.2007	M	BWP	91	5	30	9	36	2556	124	M	1	1	no	spread	oxer	right	no	0	0	66,9	no
Oslo	Western European League CSI-W5* round A	15.10.2017	CSI-W5*	4	1.40 - 1.60	410	108	11	24.03.2007	M	BWP	91	5	30	9	36	2556	124	M	2	2	no	vertical	vertical	right	no	0	0	66,9	no
Oslo	Western European League CSI-W5* round A	15.10.2017	CSI-W5*	4	1.40 - 1.60	410	108	11	24.03.2007	M	BWP	91	5	30	9	36	2556	124	M	3	3	no	vertical	vertical	left	no	0	0	66,9	no
Oslo	Western European League CSI-W5* round A	15.10.2017	CSI-W5*	4	1.40 - 1.60	410	108	11	24.03.2007	M	BWP	91	5	30	9	36	2556	124	M	4	4a	yes	vertical	vertical	straight	no	0	0	66,9	no
Oslo	Western European League CSI-W5* round A	15.10.2017	CSI-W5*	4	1.40 - 1.60	410	108	11	24.03.2007	M	BWP	91	5	30	9	36	2556	124	M	5	4b	yes	spread	oxer	straight	no	0	0	66,9	no
Oslo	Western European League CSI-W5* round A	15.10.2017	CSI-W5*	4	1.40 - 1.60	410	108	11	24.03.2007	M	BWP	91	5	30	9	36	2556	124	M	6	5	no	vertical	vertical with water	straight	no	0	0	66,9	no
Oslo	Western European League CSI-W5* round A	15.10.2017	CSI-W5*	4	1.40 - 1.60	410	108	11	24.03.2007	M	BWP	91	5	30	9	36	2556	124	M	7	6a	yes	spread	oxer	right	no	0	0	66,9	no
Oslo	Western European League CSI-W5* round A	15.10.2017	CSI-W5*	4	1.40 - 1.60	410	108	11	24.03.2007	M	BWP	91	5	30	9	36	2556	124	M	8	6b	yes	vertical	vertical	right	no	0	0	66,9	no
Oslo	Western European League CSI-W5* round A	15.10.2017	CSI-W5*	4	1.40 - 1.60	410	108	11	24.03.2007	M	BWP	91	5	30	9	36	2556	124	M	9	6c	yes	spread	oxer	right	no	0	0	66,9	no
Oslo	Western European League CSI-W5* round A	15.10.2017	CSI-W5*	4	1.40 - 1.60	410	108	11	24.03.2007	M	BWP	91	5	30	9	36	2556	124	M	10	7	no	vertical	vertical	right	no	0	0	66,9	no
Oslo	Western European League CSI-W5* round A	15.10.2017	CSI-W5*	4	1.40 - 1.60	410	108	11	24.03.2007	M	BWP	91	5	30	9	36	2556	124	M	11	8	no	spread	oxer	left	no	0	0	66,9	no
Oslo	Western European League CSI-W5* round A	15.10.2017	CSI-W5*	4	1.40 - 1.60	410	108	11	24.03.2007	M	BWP	91	5	30	9	36	2556	124	M	12	9	no	vertical	vertical	straight	no	0	0	66,9	no
Oslo	Western European League CSI-W5* round A	15.10.2017	CSI-W5*	4	1.40 - 1.60	410	108	11	24.03.2007	M	BWP	91	5	30	9	36	2556	124	M	13	10	no	spread	oxer	right	no	0	0	66,9	no
Oslo	Western European League CSI-W5* round A	15.10.2017	CSI-W5*	4	1.40 - 1.60	410	108	11	24.03.2007	M	BWP	91	5	30	9	36	2556	124	M	14	11	no	vertical	vertical	right	yes	4	0	66,9	no

Oslo	Western European League CSI-W5* round A	15.10.2017	CSI-W5*	4	1.40 - 1.60	410	108	11	24.03.2007	M	BWP	91	5	30	9	36	2556	124	M	15	12	no	spread	oxer	left	no	0	0	66,9	no
Oslo	Western European League CSI-W5* round A	15.10.2017	CSI-W5*	4	1.40 - 1.60	410	108	11	24.03.2007	M	BWP	91	5	30	9	36	2556	124	M	16	13	no	spread	oxer	straight	no	0	0	66,9	no
Oslo	Western European League CSI-W5* round A	15.10.2017	CSI-W5*	4	1.40 - 1.60	410	178	12	31.05.2006	G	SF	218	3	31	167	28	855	19	M	1	1	no	spread	oxer	right	no	0	0	66,83	no
Oslo	Western European League CSI-W5* round A	15.10.2017	CSI-W5*	4	1.40 - 1.60	410	178	12	31.05.2006	G	SF	218	3	31	167	28	855	19	M	2	2	no	vertical	vertical	right	no	0	0	66,83	no
Oslo	Western European League CSI-W5* round A	15.10.2017	CSI-W5*	4	1.40 - 1.60	410	178	12	31.05.2006	G	SF	218	3	31	167	28	855	19	M	3	3	no	vertical	vertical	left	no	0	0	66,83	no
Oslo	Western European League CSI-W5* round A	15.10.2017	CSI-W5*	4	1.40 - 1.60	410	178	12	31.05.2006	G	SF	218	3	31	167	28	855	19	M	4	4a	yes	vertical	vertical	straight	no	0	0	66,83	no
Oslo	Western European League CSI-W5* round A	15.10.2017	CSI-W5*	4	1.40 - 1.60	410	178	12	31.05.2006	G	SF	218	3	31	167	28	855	19	M	5	4b	yes	spread	oxer	straight	no	0	0	66,83	no
Oslo	Western European League CSI-W5* round A	15.10.2017	CSI-W5*	4	1.40 - 1.60	410	178	12	31.05.2006	G	SF	218	3	31	167	28	855	19	M	6	5	no	vertical	vertical with water	straight	no	0	0	66,83	no
Oslo	Western European League CSI-W5* round A	15.10.2017	CSI-W5*	4	1.40 - 1.60	410	178	12	31.05.2006	G	SF	218	3	31	167	28	855	19	M	7	6a	yes	spread	oxer	right	no	0	0	66,83	no
Oslo	Western European League CSI-W5* round A	15.10.2017	CSI-W5*	4	1.40 - 1.60	410	178	12	31.05.2006	G	SF	218	3	31	167	28	855	19	M	8	6b	yes	vertical	vertical	right	no	0	0	66,83	no
Oslo	Western European League CSI-W5* round A	15.10.2017	CSI-W5*	4	1.40 - 1.60	410	178	12	31.05.2006	G	SF	218	3	31	167	28	855	19	M	9	6c	yes	spread	oxer	right	yes	4	0	66,83	no
Oslo	Western European League CSI-W5* round A	15.10.2017	CSI-W5*	4	1.40 - 1.60	410	178	12	31.05.2006	G	SF	218	3	31	167	28	855	19	M	10	7	no	vertical	vertical	right	no	0	0	66,83	no
Oslo	Western European League CSI-W5* round A	15.10.2017	CSI-W5*	4	1.40 - 1.60	410	178	12	31.05.2006	G	SF	218	3	31	167	28	855	19	M	11	8	no	spread	oxer	left	no	0	0	66,83	no
Oslo	Western European League CSI-W5* round A	15.10.2017	CSI-W5*	4	1.40 - 1.60	410	178	12	31.05.2006	G	SF	218	3	31	167	28	855	19	M	12	9	no	vertical	vertical	straight	no	0	0	66,83	no
Oslo	Western European League CSI-W5* round A	15.10.2017	CSI-W5*	4	1.40 - 1.60	410	178	12	31.05.2006	G	SF	218	3	31	167	28	855	19	M	13	10	no	spread	oxer	right	no	0	0	66,83	no
Oslo	Western European League CSI-W5* round A	15.10.2017	CSI-W5*	4	1.40 - 1.60	410	178	12	31.05.2006	G	SF	218	3	31	167	28	855	19	M	14	11	no	vertical	vertical	right	no	0	0	66,83	no
Oslo	Western European League CSI-W5* round A	15.10.2017	CSI-W5*	4	1.40 - 1.60	410	178	12	31.05.2006	G	SF	218	3	31	167	28	855	19	M	15	12	no	spread	oxer	left	no	0	0	66,83	no
Oslo	Western European League CSI-W5* round A	15.10.2017	CSI-W5*	4	1.40 - 1.60	410	178	12	31.05.2006	G	SF	218	3	31	167	28	855	19	M	16	13	no	spread	oxer	straight	no	0	0	66,83	no
Oslo	Western European League CSI-W5* round A	15.10.2017	CSI-W5*	4	1.40 - 1.60	410	23	13	25.02.2005	S	Bezplemenné	158	2	32	53	23	1832	77	M	1	1	no	spread	oxer	right	yes	4	0	66,74	no
Oslo	Western European League CSI-W5* round A	15.10.2017	CSI-W5*	4	1.40 - 1.60	410	23	13	25.02.2005	S	Bezplemenné	158	2	32	53	23	1832	77	M	2	2	no	vertical	vertical	right	no	0	0	66,74	no
Oslo	Western European League CSI-W5* round A	15.10.2017	CSI-W5*	4	1.40 - 1.60	410	23	13	25.02.2005	S	Bezplemenné	158	2	32	53	23	1832	77	M	3	3	no	vertical	vertical	left	no	0	0	66,74	no
Oslo	Western European League CSI-W5* round A	15.10.2017	CSI-W5*	4	1.40 - 1.60	410	23	13	25.02.2005	S	Bezplemenné	158	2	32	53	23	1832	77	M	4	4a	yes	vertical	vertical	straight	no	0	0	66,74	no
Oslo	Western European League CSI-W5* round A	15.10.2017	CSI-W5*	4	1.40 - 1.60	410	23	13	25.02.2005	S	Bezplemenné	158	2	32	53	23	1832	77	M	5	4b	yes	spread	oxer	straight	no	0	0	66,74	no
Oslo	Western European League CSI-W5* round A	15.10.2017	CSI-W5*	4	1.40 - 1.60	410	23	13	25.02.2005	S	Bezplemenné	158	2	32	53	23	1832	77	M	6	5	no	vertical	vertical with water	straight	no	0	0	66,74	no
Oslo	Western European League CSI-W5* round A	15.10.2017	CSI-W5*	4	1.40 - 1.60	410	23	13	25.02.2005	S	Bezplemenné	158	2	32	53	23	1832	77	M	7	6a	yes	spread	oxer	right	no	0	0	66,74	no
Oslo	Western European League CSI-W5* round A	15.10.2017	CSI-W5*	4	1.40 - 1.60	410	23	13	25.02.2005	S	Bezplemenné	158	2	32	53	23	1832	77	M	8	6b	yes	vertical	vertical	right	no	0	0	66,74	no
Oslo	Western European League CSI-W5* round A	15.10.2017	CSI-W5*	4	1.40 - 1.60	410	23	13	25.02.2005	S	Bezplemenné	158	2	32	53	23	1832	77	M	9	6c	yes	spread	oxer	right	yes	4	0	66,74	no
Oslo	Western European League CSI-W5* round A	15.10.2017	CSI-W5*	4	1.40 - 1.60	410	23	13	25.02.2005	S	Bezplemenné	158	2	32	53	23	1832	77	M	10	7	no	vertical	vertical	right	no	0	0	66,74	no
Oslo	Western European League CSI-W5* round A	15.10.2017	CSI-W5*	4	1.40 - 1.60	410	23	13	25.02.2005	S	Bezplemenné	158	2	32	53	23	1832	77	M	11	8	no	spread	oxer	left	no	0	0	66,74	no
Oslo	Western European League CSI-W5* round A	15.10.2017	CSI-W5*	4	1.40 - 1.60	410	23	13	25.02.2005	S	Bezplemenné	158	2	32	53	23	1832	77	M	12	9	no	vertical	vertical	straight	no	0	0	66,74	no
Oslo	Western European League CSI-W5* round A	15.10.2017	CSI-W5*	4	1.40 - 1.60	410	23	13	25.02.2005	S	Bezplemenné	158	2	32	53	23	1832	77	M	13	10	no	spread	oxer	right	no	0	0	66,74	no
Oslo	Western European League CSI-W5* round A	15.10.2017	CSI-W5*	4	1.40 - 1.60	410	23	13	25.02.2005	S	Bezplemenné	158	2	32	53	23	1832	77	M	14	11	no	vertical	vertical	right	no	0	0	66,74	no
Oslo	Western European League CSI-W5* round A	15.10.2017	CSI-W5*	4	1.40 - 1.60	410	23	13	25.02.2005	S	Bezplemenné	158	2	32	53	23	1832	77	M	15	12	no	spread	oxer	left	no	0	0	66,74	no
Oslo	Western European League CSI-W5* round A	15.10.2017	CSI-W5*	4	1.40 - 1.60	410	23	13	25.02.2005	S	Bezplemenné	158	2	32	53	23	1832	77	M	16	13	no	spread	oxer	straight	no	0	0	66,74	no
Oslo	Western European League CSI-W5* round A	15.10.2017	CSI-W5*	4	1.40 - 1.60	410	134	10	28.04.2006	G	BWP	174	6	33	23	23	1854	199	M	1	1	no	spread	oxer	right	no	0	0	66,66	no
Oslo	Western European League CSI-W5* round A	15.10.2017	CSI-W5*	4	1.40 - 1.60	410	134	10	28.04.2006	G	BWP	174	6	33	23	23	1854	199	M	2	2	no	vertical	vertical	right	no	0	0	66,66	no
Oslo	Western European League CSI-W5* round A	15.10.2017	CSI-W5*	4	1.40 - 1.60	410	134	10	28.04.2006	G	BWP	174	6	33	23	23	1854	199	M	3	3	no	vertical	vertical	left	no	0	0	66,66	no
Oslo	Western European League CSI-W5* round A	15.10.2017	CSI-W5*	4	1.40 - 1.60	410	134	10	28.04.2006	G	BWP	174	6	33	23	23	1854	199	M	4	4a	yes	vertical	vertical	straight	no	0	0	66,66	no
Oslo	Western European League CSI-W5* round A	15.10.2017	CSI-W5*	4	1.40 - 1.60	410	134	10	28.04.2006	G	BWP	174	6	33	23	23	1854	199	M	5	4b	yes	spread	oxer	straight	no	0	0	66,66	no
Oslo	Western European League CSI-W5* round A	15.10.2017	CSI-W5*	4	1.40 - 1.60	410	134	10	28.04.2006	G	BWP	174	6	33	23	23	1854	199	M	6	5	no	vertical	vertical with water	straight	no	0	0	66,66	no
Oslo	Western European League CSI-W5* round A	15.10.2017	CSI-W5*	4	1.40 - 1.60	410	134	10	28.04.2006	G	BWP	174	6	33	23	23	1854	199	M	7	6a	yes	spread	oxer	right	no	0	0	66,66	no
Oslo	Western European League CSI-W5* round A	15.10.2017	CSI-W5*	4	1.40 - 1.60	410	134	10	28.04.2006	G	BWP	174	6	33	23	23	1854	199	M	8	6b	yes	vertical	vertical	right	no	0	0	66,66	no
Oslo	Western European League CSI-W5* round A	15.10.2017	CSI-W5*	4	1.40 - 1.60	410	134	10	28.04.2006	G	BWP	174	6	33	23	23	1854	199	M	9	6c	yes	spread	oxer	right	no	0	0	66,66	no
Oslo	Western European League CSI-W5* round A	15.10.2017	CSI-W5*	4	1.40 - 1.60	410	134	10	28.04.2006	G	BWP	174	6	33	23	23	1854	199	M	10	7	no	vertical	vertical	right	no	0	0	66,66	no
Oslo	Western European League CSI-W5* round A	15.10.2017	CSI-W5*	4	1.40 - 1.60	410	134	10	28.04.2006	G	BWP	174	6	33	23	23	1854	199	M	11	8	no	spread	oxer	left	no	0	0	66,66	no
Oslo	Western European League CSI-W5* round A	15.10.2017	CSI-W5*	4	1.40 - 1.60	410	134	10	28.04.2006	G	BWP	174	6	33	23	23	1854	199	M	12	9	no	vertical	vertical	straight	no	0	0	66,66	no
Oslo	Western European League CSI-W5* round A	15.10.2017	CSI-W5*	4	1.40 - 1.60	410	134	10	28.04.2006	G	BWP	174	6	33	23	23	1854	199	M	13	10	no	spread	oxer	right	no	0	0	66,66	no
Oslo	Western European League CSI-W5* round A	15.10.2017	CSI-W5*	4	1.40 - 1.60	410	134	10	28.04.2006	G	BWP	174	6	33	23	23	1854	199	M	14	11	no	vertical	vertical	right	no	0	0	66,66	no
Oslo	Western European League CSI-W5* round A	15.10.2017	CSI-W5*	4	1.40 - 1.60	410	134	10	28.04.2006	G	BWP	174	6	33	23	23	1854	199	M	15	12	no	spread	oxer	left	no	0	0	66,66	no
Oslo	Western European League CSI-W5* round A	15.10.2017	CSI-W5*	4	1.40 - 1.60	410	134	10	28.04.2006	G	BWP	174	6	33	23	23	1854	199	M	16	13	no	spread	oxer	straight	no	0	0	66,66	no
Oslo	Western European League CSI-W5* round B	15.10.2017	CSI-W5*	4	1.40 - 1.60	295	134	10	28.04.2006	G	BWP	174	6	33	23	23	1854	199	M	1	1	no	vertical	vertical	right	no	0	0	37,5	no
Oslo	Western European League CSI-W5* round B	15.10.2017	CSI-W5*	4	1.40 - 1.60	295	134	10	28.04.2006	G	BWP	174	6	33	23	23	1854	199	M	2	2	no	spread	oxer	right	no	0	0	37,5	no
Oslo	Western European League CSI-W5* round B	15.10.2017	CSI-W5*	4	1.40 - 1.60	295	134	10	28.04.2006	G	BWP	174	6	33	23	23	1854	199	M	3	3	no	vertical	vertical	right	no	0	0	37,5	no
Oslo	Western European League CSI-W5* round B	15.10.2017	CSI-W5*	4	1.40 - 1.60	295	134	10	28.04.2006	G	BWP	174	6	33	23	23	1854	199	M	4	4	no	spread	oxer	left	no	0	0	37,5	no
Oslo	Western European League CSI-W5* round B	15.10.2017	CSI-W5*	4	1.40 - 1.60	295	134	10	28.04.2006	G	BWP	174	6	33	23	23	1854	199	M	5	5	no	vertical	vertical	left	no	0	0	37,5	no
Oslo	Western European League CSI-W5* round B	15.10.2017	CSI-W5*	4	1.40 - 1.60	295	134	10	28.04.2006	G	BWP	174	6	33	23	23	1854	199	M	6	6a	yes	vertical	vertical	straight	no	0	0	37,5	no
Oslo	Western European League CSI-W5* round B	15.10.2017	CSI-W5*	4	1.40 - 1.60	295	134	10	28.04.2006	G	BWP	174	6	33	23	23	1854	199	M	7	6b	yes	spread	oxer	straight	no	0	0	37,5	no
Oslo	Western European League CSI-W5* round B	15.10.2017	CSI-W5*	4	1.40 - 1.60	295	134	10	28.04.2006	G	BWP	174	6	33	23	23	1854	199	M	8	7	no	spread	oxer	right	yes	4	0	37,5	no
Oslo	Western European League CSI-W5* round B	15.10.2017	CSI-W5*	4	1.40 - 1.60	295	134	10	28.04.2006	G	BWP	174	6	33	23	23	1854	199	M	9	8	no	vertical	vertical	right	no	0	0	37,5	no
Oslo	Western European League CSI-W5* round A	15.10.2017	CSI-W5*	4	1.40 - 1.60	410	113	11	16.06.2007	M	BWP	158	13	34	85	29	1646	66	M	1	1	no	spread	oxer	right	no	0	0	66,33	no
Oslo	Western European League CSI-W5* round A	15.10.2017	CSI-W5*	4	1.40 - 1.60	410	113	11	16.06.2007	M	BWP	158	13	34	85	29	1646	66	M	2	2	no	vertical	vertical	right	yes	4	0	66,33	no
Oslo	Western European League CSI-W5* round A	15.10.2017	CSI-W5*	4	1.40 - 1.60	410	113	11	16.06.2007	M	BWP	158	13	34	85	29	1646	66	M	3	3	no	vertical	vertical	left	no	0	0	66,33	no
Oslo	Western European League CSI-W5* round A	15.10.2017	CSI-W5*	4	1.40 - 1.60	410	113	11	16.06.2007	M	BWP	158	13	34	85	29	1646	66	M	4	4a	yes	vertical	vertical	straight	no	0	0	66,33	no
Oslo	Western European League CSI-W5* round A	15.10.2017	CSI-W5*	4	1.40 - 1.60	410	113	11	16.06.2007	M	BWP	158	13	34	85	29	1646	66	M	5	4b	yes	spread	oxer	straight	no	0	0	66,33	no
Oslo	Western European League CSI-W5* round A	15.10.2017	CSI-W5*	4	1.40 - 1.60	410	113	11	16.06.2007	M	BWP	158	13	34	85	29	1646	66	M	6	5	no	vertical	vertical with water	straight	no	0	0	66,33	no
Oslo	Western European League CSI-W5* round A	15.10.2017	CSI-W5*	4	1.40 - 1.60	410	113	11	16.06.2007	M	BWP	158	13	34	85	29	1646	66	M	7	6a	yes	spread	oxer	right	no	0	0	66,33	no
Oslo	Western European League CSI-W5* round A	15.10.2017	CSI-W5*	4	1.40 - 1.60	410	113	11	16.06.2007	M	BWP	158	13	34	85	29	1646	66	M	8	6b	yes	vertical	vertical	right	no	0	0	66,33	no
Oslo	Western European League CSI-W5* round A	15.10.2017	CSI-W5*	4	1.40 - 1.60	410	113	11	16.06.2007	M	BWP	158	13	34	85	29	1646	66	M	9	6c	yes	spread	oxer	right	no	0	0	66,33	no
Oslo	Western European League CSI-W5* round A	15.10.2017	CSI-W5*	4	1.40 - 1.60	410	113	11	16.06.2007	M	BWP	158	13	34	85	29	1646	66	M	10	7	no	vertical	vertical	right	no	0	0	66,33	no
Oslo	Western European League CSI-W5* round A	15.10.2017	CSI-W5*	4	1.40 - 1.60	410	113	11	16.06.2007	M	BWP	158	13	34	85	29	1646	66	M	11	8	no	spread	oxer	left	no	0	0	66,33	no
Oslo	Western European League CSI-W5* round A	15.10.2017	CSI-W5*	4	1.40 - 1.60	410	113	11	16.06.2007	M	BWP	158	13	34	85	29	1646	66	M	12	9	no	vertical	vertical	straight	no	0	0	66,33	no
Oslo	Western European League CSI-W5* round A	15.10.2017	CSI-W5*	4	1.40 - 1.60	410	113	11	16.06.2007	M	BWP	158	13	34	85	29	1646	66	M	13	10	no	spread	oxer	right	yes	4	0	66,33	no
Oslo	Western European League CSI-W5* round A	15.10.2017	CSI-W5*	4	1.40 - 1.60	410	113	11	16.06.2007	M	BWP	158	13	34	85	29	1646	66	M	14	11	no	vertical	vertical	right	no	0	0	66,33	no
Oslo	Western European League CSI-W5* round A	15.10.2017	CSI-W5*	4	1.40 - 1.60	410	113	11	16.06.2007	M	BWP	158	13	34	85	29	1646	66	M	15	12	no	spread	oxer	left	no	0	0	66,33	no
Oslo	Western European League CSI-W5* round A	15.10.2017	CSI-W5*	4	1.40 - 1.60	410	113	11	16.06.2007	M	BWP	158	13	34	85	29	1646	66	M	16	13	no	spread	oxer	straight	no	0	0	66,33	no
Oslo	Western European League CSI-W5* round A	15.10.2017	CSI-W5*	4	1.40 - 1.60	410	37	11	02.03.2007	M	KWPN	133	11	35	44	30	2234	138	M	1	1	no	spread	oxer	right	no	0	0	66,3	no
Oslo	Western European League CSI-W5* round A	15.10.2017	CSI-W5*	4	1.40 - 1.60	410	37	11	02.03.2007	M	KWPN	133	11	35	44	30	2234	138	M	2	2	no	vertical	vertical	right	no	0	0	66,3	no
Oslo	Western European League CSI-W5* round A	15.10.2017	CSI-W5*	4	1.40 - 1.60	410	37	11	02.03.2007	M	KWPN	133	11	35	44	30	2234	138	M	3	3	no	vertical	vertical	left	no	0	0	66,3	no
Oslo	Western European League CSI-W5* round A	15.10.2017	CSI-W5*	4	1.40 - 1.60	410	37	11	02.03.2007	M	KWPN	133	11	35	44	30	2234	138	M	4	4a	yes	vertical	vertical	straight	no	0	0	66,3	no
Oslo	Western European League CSI-W5* round A	15.10.2017	CSI-W5*	4	1.40 - 1.60	410	37	11	02.03.2007	M	KWPN	133	11	35	44	30	2234	138	M	5	4b	yes	spread	oxer	straight	no	0	0	66,3	no
Oslo	Western European League CSI-W5* round A	15.10.2017	CSI-W5*	4	1.40 - 1.60	410	37	11	02.03.2007	M	KWPN	133	11	35	44	30	2234	138	M	6	5	no	vertical	vertical with water	straight	yes	4	0	66,3	no
Oslo	Western European League CSI-W5* round A	15.10.2017	CSI-W5*	4	1.40 - 1.60	410	37	11	02.03.2007	M	KWPN	133	11	35	44	30	2234	138	M	7	6a	yes	spread	oxer	right	no	0	0	66,3	no
Oslo	Western European League CSI-W5* round A	15.10.2017	CSI-W5*	4	1.40 - 1.60	410	37	11	02.03.2007	M	KWPN	133	11	35	44	30	2234	138	M	8	6b	yes	vertical	vertical	right	no	0	0	66,3	no
Oslo	Western European League CSI-W5* round A	15.10.2017	CSI-W5*	4	1.40 - 1.60	410	37	11	02.03.2007	M	KWPN	133	11	35	44	30	2234	138	M	9	6c	yes	spread	oxer	right	no	0	0	66,3	no
Oslo	Western European League CSI-W5* round A	15.10.2017	CSI-W5*	4	1.40 - 1.60	410	37	11	02.03.2007	M	KWPN	133	11	35	44	30	2234	138	M	10	7	no	vertical	vertical	right	no	0	0	66,3	no
Oslo	Western European League CSI-W5* round A	15.10.2017	CSI-W5*	4	1.40 - 1.60	410	37	11	02.03.2007	M	KWPN	133	11	35	44	30	2234	138	M	11	8	no	spread	oxer	left	no	0	0	66,3	no
Oslo	Western European League CSI-W5* round A	15.10.2017	CSI-W5*	4	1.40 - 1.60	410	37	11	02.03.2007	M	KWPN	133	11	35	44	30	2234	138	M	12	9	no	vertical	vertical	straight	no	0	0	66,3	no
Oslo	Western European League CSI-W5* round A	15.10.2017	CSI-W5*	4	1.40 - 1.60	410	37	11	02.03.2007	M	KWPN	133	11	35	44	30	2234	138	M	13	10	no	spread	oxer	right	no	0	0	66,3	no
Oslo	Western European League CSI-W5* round A	15.10.2017	CSI-W5*	4	1.40 - 1.60	410	37	11	02.03.2007	M	KWPN	133	11	35	44	30	2234	138	M	14	11	no	vertical	vertical	right	yes	4	0	66,3	no
Oslo	Western European League CSI-W5* round A	15.10.2017	CSI-W5*	4	1.40 - 1.60	410	37	11	02.03.2007	M	KWPN	133	11	35	44	30	2234	138	M	15	12	no	spread	oxer	left	no	0	0	66,3	no
Oslo	Western European League CSI-W5* round A	15.10.2017	CSI-W5*	4	1.40 - 1.60	410	37	11	02.03.2007	M	KWPN	133	11	35	44	30	2234	138	M	16	13	no	spread	oxer	straight	no	0	0	66,3	no
Oslo	Western European League CSI-W5* round A	15.10.2017	CSI-W5*	4	1.40 - 1.60	410	19	11	15.05.2007	M	KWPN	97	5	36	47	44	1871	37	F	1	1	no	spread	oxer	right	no	0	0	66,14	no
Oslo	Western European League CSI-W5* round A	15.10.2017	CSI-W5*	4	1.40 - 1.60	410	19	11	15.05.2007	M	KWPN	97	5	36	47	44	1871	37	F	2	2	no	vertical	vertical	right	no	0	0	66,14	no
Oslo	Western European League CSI-W5* round A	15.10.2017	CSI-W5*	4	1.40 - 1.60	410	19	11	15.05.2007	M	KWPN	97	5	36	47	44	1871	37	F	3	3	no	vertical	vertical	left	no	0	0	66,14	no
Oslo	Western European League CSI-W5* round A	15.10.2017	CSI-W5*	4	1.40 - 1.60	410	19	11	15.05.2007	M	KWPN	97	5	36	47	44	1871	37	F	4	4a	yes	vertical	vertical	straight	no	0	0	66,14	no
Oslo	Western European League CSI-W5* round A	15.10.2017	CSI-W5*	4	1.40 - 1.60	410	19	11	15.05.2007	M	KWPN	97	5	36	47	44	1871	37	F	5	4b	yes	spread	oxer	straight	no	0	0	66,14	no
Oslo	Western European League CSI-W5* round A	15.10.2017	CSI-W5*	4	1.40 - 1.60	410	19	11	15.05.2007	M	KWPN	97	5	36	47	44	1871	37	F	6	5	no	vertical	vertical with water	straight	no	0	0	66,14	no
Oslo	Western European League CSI-W5* round A	15.10.2017	CSI-W5*	4	1.40 - 1.60	410	19	11	15.05.2007	M	KWPN	97	5	36	47	44	1871	37	F	7	6a	yes	spread	oxer	right	no	0	0	66,14	no
Oslo	Western European League CSI-W5* round A	15.10.2017	CSI-W5*	4	1.40 - 1.60	410	19	11	15.05.2007	M	KWPN	97	5	36	47	44	1871	37	F	8	6b	yes	vertical	vertical	right	no	0	0	66,14	no
Oslo	Western European League CSI-W5* round A	15.10.2017	CSI-W5*	4	1.40 - 1.60	410	19	11	15.05.2007	M	KWPN	97	5	36	47	44	1871	37	F	9	6c	yes	spread	oxer	right	no	0	0	66,14	no
Oslo	Western European League CSI-W5* round A	15.10.2017	CSI-W5*	4	1.40 - 1.60	410	19	11	15.05.2007	M	KWPN	97	5	36	47	44	1871	37	F	10	7	no	vertical	vertical	right	no	0	0	66,14	no
Oslo	Western European League CSI-W5* round A	15.10.2017	CSI-W5*	4	1.40 - 1.60	410	19	11	15.05.2007	M	KWPN	97	5	36	47	44	1871	37	F	11	8	no	spread	oxer	left	no	0	0	66,14	no
Oslo	Western European League CSI-W5* round A	15.10.2017	CSI-W5*	4	1.40 - 1.60	410	19	11	15.05.2007	M	KWPN	97	5	36	47	44	1871	37	F	12	9	no	vertical	vertical	straight	no	0	0	66,14	no
Oslo	Western European League CSI-W5* round A	15.10.2017	CSI-W5*	4	1.40 - 1.60	410	19	11	15.05.2007	M	KWPN	97	5	36	47	44	1871	37	F	13	10	no	spread	oxer	right	no	0	0	66,14	no
Oslo	Western European League CSI-W5* round A	15.10.2017	CSI-W5*	4	1.40 - 1.60	410	19	11	15.05.2007	M	KWPN	97	5	36	47	44	1871	37	F	14	11	no	vertical	vertical	right	no	0	0	66,14	no
Oslo	Western European League CSI-W5* round A	15.10.2017	CSI-W5*	4	1.40 - 1.60	410	19	11	15.05.2007	M	KWPN	97	5	36	47	44	1871	37	F	15	12	no	spread	oxer	left	no	0	0	66,14	no
Oslo	Western European League CSI-W5* round A	15.10.2017	CSI-W5*	4	1.40 - 1.60	410	19	11	15.05.2007	M	KWPN	97	5	36	47	44	1871	37	F	16	13	no	spread	oxer	straight	no	0	0	66,14	no
Oslo	Western European League CSI-W5* round B	15.10.2017	CSI-W5*	4	1.40 - 1.60	295	19	11	15.05.2007	M	KWPN	97	5	36	47	44	1871	37	F	1	1	no	vertical	vertical	right	no	0	0	38,22	no
Oslo	Western European League CSI-W5* round B	15.10.2017	CSI-W5*	4	1.40 - 1.60	295	19	11	15.05.2007	M	KWPN	97	5	36	47	44	1871	37	F	2	2	no	spread	oxer	right	no	0	0	38,22	no
Oslo	Western European League CSI-W5* round B	15.10.2017	CSI-W5*	4	1.40 - 1.60	295	19	11	15.05.2007	M	KWPN	97	5	36	47	44	1871	37	F	3	3	no	vertical	vertical	right	no	0	0	38,22	no
Oslo	Western European League CSI-W5* round B	15.10.2017	CSI-W5*	4	1.40 - 1.60	295	19	11	15.05.2007	M	KWPN	97	5	36	47	44	1871	37	F	4	4	no	spread	oxer	left	no	0	0	38,22	no
Oslo	Western European League CSI-W5* round B	15.10.2017	CSI-W5*	4	1.40 - 1.60	295	19	11	15.05.2007	M	KWPN	97	5	36	47	44	1871	37	F	5	5	no	vertical	vertical	left	no	0	0	38,22	no
Oslo	Western European League CSI-W5* round B	15.10.2017	CSI-W5*	4	1.40 - 1.60	295	19	11	15.05.2007	M	KWPN	97	5	36	47	44	1871	37	F	6	6a	yes	vertical	vertical	straight	no	0	0	38,22	no
Oslo	Western European League CSI-W5* round B	15.10.2017	CSI-W5*	4	1.40 - 1.60	295	19	11	15.05.2007	M	KWPN	97	5	36	47	44	1871	37	F	7	6b	yes	spread	oxer	straight	no	0	0	38,22	no
Oslo	Western European League CSI-W5* round B	15.10.2017	CSI-W5*	4	1.40 - 1.60	295	19	11	15.05.2007	M	KWPN	97	5	36	47	44	1871	37	F	8	7	no	spread	oxer	right	no	0	0	38,22	no
Oslo	Western European League CSI-W5* round B	15.10.2017	CSI-W5*	4	1.40 - 1.60	295	19	11	15.05.2007	M	KWPN	97	5	36	47	44	1871	37	F	9	8	no	vertical	vertical	right	no	0	0	38,22	no
Oslo	Western European League CSI-W5* round A	15.10.2017	CSI-W5*	4	1.40 - 1.60	410	166	14	01.03.2004	M	AA	187	5	37	59	42	1314	68	M	1	1	no	spread	oxer	right	no	0	0	65,2	no
Oslo	Western European League CSI-W5* round A	15.10.2017	CSI-W5*	4	1.40 - 1.60	410	166	14	01.03.2004	M	AA	187	5	37	59	42	1314	68	M	2	2	no	vertical	vertical	right	no	0	0	65,2	no
Oslo	Western European League CSI-W5* round A	15.10.2017	CSI-W5*	4	1.40 - 1.60	410	166	14	01.03.2004	M	AA	187	5	37	59	42	1314	68	M	3	3	no	vertical	vertical	left	yes	4	0	65,2	no
Oslo	Western European League CSI-W5* round A	15.10.2017	CSI-W5*	4	1.40 - 1.60	410	166	14	01.03.2004	M	AA	187	5	37	59	42	1314	68	M	4	4a	yes	vertical	vertical	straight	no	0	0	65,2	no
Oslo	Western European League CSI-W5* round A	15.10.2017	CSI-W5*	4	1.40 - 1.60	410	166	14	01.03.2004	M	AA	187	5	37	59	42	1314	68	M	5	4b	yes	spread	oxer	straight	no	0	0	65,2	no
Oslo	Western European League CSI-W5* round A	15.10.2017	CSI-W5*	4	1.40 - 1.60	410	166	14	01.03.2004	M	AA	187	5	37	59	42	1314	68	M	6	5	no	vertical	vertical with water	straight	no	0	0	65,2	no
Oslo	Western European League CSI-W5* round A	15.10.2017	CSI-W5*	4	1.40 - 1.60	410	166	14	01.03.2004	M	AA	187	5	37	59	42	1314	68	M	7	6a	yes	spread	oxer	right	no	0	0	65,2	no
Oslo	Western European League CSI-W5* round A	15.10.2017	CSI-W5*	4	1.40 - 1.60	410	166	14	01.03.2004	M	AA	187	5	37	59	42	1314	68	M	8	6b	yes	vertical	vertical	right	no	0	0	65,2	no
Oslo	Western European League CSI-W5* round A	15.10.2017	CSI-W5*	4	1.40 - 1.60	410	166	14	01.03.2004	M	AA	187	5	37	59	42	1314	68	M	9	6c	yes	spread	oxer	right	no	0	0	65,2	no
Oslo	Western European League CSI-W5* round A	15.10.2017	CSI-W5*	4	1.40 - 1.60	410	166	14	01.03.2004	M	AA	187	5	37	59	42	1314	68	M	10	7	no	vertical	vertical	right	no	0	0	65,2	no
Oslo	Western European League CSI-W5* round A	15.10.2017	CSI-W5*	4	1.40 - 1.60	410	166	14	01.03.2004	M	AA	187	5	37	59	42	1314	68	M	11	8	no	spread	oxer	left	no	0	0	65,2	no
Oslo	Western European League CSI-W5* round A	15.10.2017	CSI-W5*	4	1.40 - 1.60	410	166	14	01.03.2004	M	AA	187	5	37	59	42	1314	68	M	12	9	no	vertical	vertical	straight	no	0	0	65,2	no
Oslo	Western European League CSI-W5* round A	15.10.2017	CSI-W5*	4	1.40 - 1.60	410	166	14	01.03.2004	M	AA	187	5	37	59	42	1314	68	M	13	10	no	spread	oxer	right	no	0	0	65,2	no
Oslo	Western European League CSI-W5* round A	15.10.2017	CSI-W5*	4	1.40 - 1.60	410	166	14	01.03.2004	M	AA	187	5	37	59	42	1314	68	M	14	11	no	vertical	vertical	right	no	0	0	65,2	no
Oslo	Western European League CSI-W5* round A	15.10.2017	CSI-W5*	4	1.40 - 1.60	410	166	14	01.03.2004	M	AA	187	5	37	59	42	1314	68	M	15	12	no	spread	oxer	left	no	0	0	65,2	no
Oslo	Western European League CSI-W5* round A	15.10.2017	CSI-W5*	4	1.40 - 1.60	410	166	14	01.03.2004	M	AA	187	5	37	59	42	1314	68	M	16	13	no	spread	oxer	straight	yes	4	0	65,2	no
Oslo	Western European League CSI-W5* round A	15.10.2017	CSI-W5*	4	1.40 - 1.60	410	85	13	08.05.2005	M	BWP	270	4	38	10	29	1642	46	M	1	1	no	spread	oxer	right	no	0	0	64,99	no
Oslo	Western European League CSI-W5* round A	15.10.2017	CSI-W5*	4	1.40 - 1.60	410	85	13	08.05.2005	M	BWP	270	4	38	10	29	1642	46	M	2	2	no	vertical	vertical	right	no	0	0	64,99	no
Oslo	Western European League CSI-W5* round A	15.10.2017	CSI-W5*	4	1.40 - 1.60	410	85	13	08.05.2005	M	BWP	270	4	38	10	29	1642	46	M	3	3	no	vertical	vertical	left	no	0	0	64,99	no
Oslo	Western European League CSI-W5* round A	15.10.2017	CSI-W5*	4	1.40 - 1.60	410	85	13	08.05.2005	M	BWP	270	4	38	10	29	1642	46	M	4	4a	yes	vertical	vertical	straight	no	0	0	64,99	no
Oslo	Western European League CSI-W5* round A	15.10.2017	CSI-W5*	4	1.40 - 1.60	410	85	13	08.05.2005	M	BWP	270	4	38	10	29	1642	46	M	5	4b	yes	spread	oxer	straight	yes	4	0	64,99	no
Oslo	Western European League CSI-W5* round A	15.10.2017	CSI-W5*	4	1.40 - 1.60	410	85	13	08.05.2005	M	BWP	270	4	38	10	29	1642	46	M	6	5	no	vertical	vertical with water	straight	no	0	0	64,99	no
Oslo	Western European League CSI-W5* round A	15.10.2017	CSI-W5*	4	1.40 - 1.60	410	85	13	08.05.2005	M	BWP	270	4	38	10	29	1642	46	M	7	6a	yes	spread	oxer	right	no	0	0	64,99	no
Oslo	Western European League CSI-W5* round A	15.10.2017	CSI-W5*	4	1.40 - 1.60	410	85	13	08.05.2005	M	BWP	270	4	38	10	29	1642	46	M	8	6b	yes	vertical	vertical	right	no	0	0	64,99	no
Oslo	Western European League CSI-W5* round A	15.10.2017	CSI-W5*	4	1.40 - 1.60	410	85	13	08.05.2005	M	BWP	270	4	38	10	29	1642	46	M	9	6c	yes	spread	oxer	right	no	0	0	64,99	no
Oslo	Western European League CSI-W5* round A	15.10.2017	CSI-W5*	4	1.40 - 1.60	410	85	13	08.05.2005	M	BWP	270	4	38	10	29	1642	46	M	10	7	no	vertical	vertical	right	no	0	0	64,99	no
Oslo	Western European League CSI-W5* round A	15.10.2017	CSI-W5*	4	1.40 - 1.60	410	85	13	08.05.2005	M	BWP	270	4	38	10	29	1642	46	M	11	8	no	spread	oxer	left	no	0	0	64,99	no
Oslo	Western European League CSI-W5* round A	15.10.2017	CSI-W5*	4	1.40 - 1.60	410	85	13	08.05.2005	M	BWP	270	4	38	10	29	1642	46	M	12	9	no	vertical	vertical	straight	no	0	0	64,99	no
Oslo	Western European League CSI-W5* round A	15.10.2017	CSI-W5*	4	1.40 - 1.60	410	85	13	08.05.2005	M	BWP	270	4	38	10	29	1642	46	M	13	10	no	spread	oxer	right	no	0	0	64,99	no
Oslo	Western European League CSI-W5* round A	15.10.2017	CSI-W5*	4	1.40 - 1.60	410	85	13	08.05.2005	M	BWP	270	4	38	10	29	1642	46	M	14	11	no	vertical	vertical	right	no	0	0	64,99	no
Oslo	Western European League CSI-W5* round A	15.10.2017	CSI-W5*	4	1.40 - 1.60	410	85	13	08.05.2005	M	BWP	270	4	38	10	29	1642	46	M	15	12	no	spread	oxer	left	no	0	0	64,99	no
Oslo	Western European League CSI-W5* round A	15.10.2017	CSI-W5*	4	1.40 - 1.60	410	85	13	08.05.2005	M	BWP	270	4	38	10	29	1642	46	M	16	13	no	spread	oxer	straight	no	0	0	64,99	no
Helsinki	The Longines FEI World Cup Jumping - Helsinki round A	22.10.2017	CSI-W5*	4	1.40 - 1.60	450	93	13	01.06.2005	G	BWP	169	5	1	19	38	2803	179	M	1	1	no	vertical	vertical	right	no	0	0	73,2	no
Helsinki	The Longines FEI World Cup Jumping - Helsinki round A	22.10.2017	CSI-W5*	4	1.40 - 1.60	450	93	13	01.06.2005	G	BWP	169	5	1	19	38	2803	179	M	2	2	no	vertical	vertical	left	no	0	0	73,2	no
Helsinki	The Longines FEI World Cup Jumping - Helsinki round A	22.10.2017	CSI-W5*	4	1.40 - 1.60	450	93	13	01.06.2005	G	BWP	169	5	1	19	38	2803	179	M	3	3	no	spread	oxer	left	no	0	0	73,2	no
Helsinki	The Longines FEI World Cup Jumping - Helsinki round A	22.10.2017	CSI-W5*	4	1.40 - 1.60	450	93	13	01.06.2005	G	BWP	169	5	1	19	38	2803	179	M	4	4a	yes	spread	oxer	left	no	0	0	73,2	no
Helsinki	The Longines FEI World Cup Jumping - Helsinki round A	22.10.2017	CSI-W5*	4	1.40 - 1.60	450	93	13	01.06.2005	G	BWP	169	5	1	19	38	2803	179	M	5	4b	yes	vertical	vertical	left	no	0	0	73,2	no
Helsinki	The Longines FEI World Cup Jumping - Helsinki round A	22.10.2017	CSI-W5*	4	1.40 - 1.60	450	93	13	01.06.2005	G	BWP	169	5	1	19	38	2803	179	M	6	4c	yes	spread	oxer	left	no	0	0	73,2	no
Helsinki	The Longines FEI World Cup Jumping - Helsinki round A	22.10.2017	CSI-W5*	4	1.40 - 1.60	450	93	13	01.06.2005	G	BWP	169	5	1	19	38	2803	179	M	7	5	no	spread	oxer	right	no	0	0	73,2	no
Helsinki	The Longines FEI World Cup Jumping - Helsinki round A	22.10.2017	CSI-W5*	4	1.40 - 1.60	450	93	13	01.06.2005	G	BWP	169	5	1	19	38	2803	179	M	8	6a	yes	vertical	vertical	right	no	0	0	73,2	no
Helsinki	The Longines FEI World Cup Jumping - Helsinki round A	22.10.2017	CSI-W5*	4	1.40 - 1.60	450	93	13	01.06.2005	G	BWP	169	5	1	19	38	2803	179	M	9	6b	yes	vertical	vertical	right	no	0	0	73,2	no
Helsinki	The Longines FEI World Cup Jumping - Helsinki round A	22.10.2017	CSI-W5*	4	1.40 - 1.60	450	93	13	01.06.2005	G	BWP	169	5	1	19	38	2803	179	M	10	7	no	spread	oxer	left	no	0	0	73,2	no
Helsinki	The Longines FEI World Cup Jumping - Helsinki round A	22.10.2017	CSI-W5*	4	1.40 - 1.60	450	93	13	01.06.2005	G	BWP	169	5	1	19	38	2803	179	M	11	8	no	vertical	wall	left	no	0	0	73,2	no
Helsinki	The Longines FEI World Cup Jumping - Helsinki round A	22.10.2017	CSI-W5*	4	1.40 - 1.60	450	93	13	01.06.2005	G	BWP	169	5	1	19	38	2803	179	M	12	9	no	spread	oxer	straight	no	0	0	73,2	no
Helsinki	The Longines FEI World Cup Jumping - Helsinki round A	22.10.2017	CSI-W5*	4	1.40 - 1.60	450	93	13	01.06.2005	G	BWP	169	5	1	19	38	2803	179	M	13	10	no	spread	triple bar	left	no	0	0	73,2	no
Helsinki	The Longines FEI World Cup Jumping - Helsinki round A	22.10.2017	CSI-W5*	4	1.40 - 1.60	450	93	13	01.06.2005	G	BWP	169	5	1	19	38	2803	179	M	14	11	no	vertical	vertical with water	straight	yes	4	0	73,2	no
Helsinki	The Longines FEI World Cup Jumping - Helsinki round A	22.10.2017	CSI-W5*	4	1.40 - 1.60	450	93	13	01.06.2005	G	BWP	169	5	1	19	38	2803	179	M	15	12a	yes	spread	oxer	right	no	0	0	73,2	no
Helsinki	The Longines FEI World Cup Jumping - Helsinki round A	22.10.2017	CSI-W5*	4	1.40 - 1.60	450	93	13	01.06.2005	G	BWP	169	5	1	19	38	2803	179	M	16	12b	yes	vertical	vertical	right	no	0	0	73,2	no
Helsinki	The Longines FEI World Cup Jumping - Helsinki round A	22.10.2017	CSI-W5*	4	1.40 - 1.60	450	93	13	01.06.2005	G	BWP	169	5	1	19	38	2803	179	M	17	13	no	spread	oxer	right	no	0	0	73,2	no
Helsinki	The Longines FEI World Cup Jumping - Helsinki round A	22.10.2017	CSI-W5*	4	1.40 - 1.60	450	63	11	22.03.2007	G	KWPN	175	2	3	97	43	1901	45	M	1	1	no	vertical	vertical	right	no	0	0	75,44	no
Helsinki	The Longines FEI World Cup Jumping - Helsinki round A	22.10.2017	CSI-W5*	4	1.40 - 1.60	450	63	11	22.03.2007	G	KWPN	175	2	3	97	43	1901	45	M	2	2	no	vertical	vertical	left	no	0	0	75,44	no
Helsinki	The Longines FEI World Cup Jumping - Helsinki round A	22.10.2017	CSI-W5*	4	1.40 - 1.60	450	63	11	22.03.2007	G	KWPN	175	2	3	97	43	1901	45	M	3	3	no	spread	oxer	left	no	0	0	75,44	no
Helsinki	The Longines FEI World Cup Jumping - Helsinki round A	22.10.2017	CSI-W5*	4	1.40 - 1.60	450	63	11	22.03.2007	G	KWPN	175	2	3	97	43	1901	45	M	4	4a	yes	spread	oxer	left	no	0	0	75,44	no
Helsinki	The Longines FEI World Cup Jumping - Helsinki round A	22.10.2017	CSI-W5*	4	1.40 - 1.60	450	63	11	22.03.2007	G	KWPN	175	2	3	97	43	1901	45	M	5	4b	yes	vertical	vertical	left	no	0	0	75,44	no
Helsinki	The Longines FEI World Cup Jumping - Helsinki round A	22.10.2017	CSI-W5*	4	1.40 - 1.60	450	63	11	22.03.2007	G	KWPN	175	2	3	97	43	1901	45	M	6	4c	yes	spread	oxer	left	no	0	0	75,44	no
Helsinki	The Longines FEI World Cup Jumping - Helsinki round A	22.10.2017	CSI-W5*	4	1.40 - 1.60	450	63	11	22.03.2007	G	KWPN	175	2	3	97	43	1901	45	M	7	5	no	spread	oxer	right	no	0	0	75,44	no
Helsinki	The Longines FEI World Cup Jumping - Helsinki round A	22.10.2017	CSI-W5*	4	1.40 - 1.60	450	63	11	22.03.2007	G	KWPN	175	2	3	97	43	1901	45	M	8	6a	yes	vertical	vertical	right	no	0	0	75,44	no
Helsinki	The Longines FEI World Cup Jumping - Helsinki round A	22.10.2017	CSI-W5*	4	1.40 - 1.60	450	63	11	22.03.2007	G	KWPN	175	2	3	97	43	1901	45	M	9	6b	yes	vertical	vertical	right	no	0	0	75,44	no
Helsinki	The Longines FEI World Cup Jumping - Helsinki round A	22.10.2017	CSI-W5*	4	1.40 - 1.60	450	63	11	22.03.2007	G	KWPN	175	2	3	97	43	1901	45	M	10	7	no	spread	oxer	left	no	0	0	75,44	no
Helsinki	The Longines FEI World Cup Jumping - Helsinki round A	22.10.2017	CSI-W5*	4	1.40 - 1.60	450	63	11	22.03.2007	G	KWPN	175	2	3	97	43	1901	45	M	11	8	no	vertical	wall	left	yes	4	0	75,44	no
Helsinki	The Longines FEI World Cup Jumping - Helsinki round A	22.10.2017	CSI-W5*	4	1.40 - 1.60	450	63	11	22.03.2007	G	KWPN	175	2	3	97	43	1901	45	M	12	9	no	spread	oxer	straight	no	0	0	75,44	no
Helsinki	The Longines FEI World Cup Jumping - Helsinki round A	22.10.2017	CSI-W5*	4	1.40 - 1.60	450	63	11	22.03.2007	G	KWPN	175	2	3	97	43	1901	45	M	13	10	no	spread	triple bar	left	no	0	0	75,44	no
Helsinki	The Longines FEI World Cup Jumping - Helsinki round A	22.10.2017	CSI-W5*	4	1.40 - 1.60	450	63	11	22.03.2007	G	KWPN	175	2	3	97	43	1901	45	M	14	11	no	vertical	vertical with water	straight	no	0	0	75,44	no
Helsinki	The Longines FEI World Cup Jumping - Helsinki round A	22.10.2017	CSI-W5*	4	1.40 - 1.60	450	63	11	22.03.2007	G	KWPN	175	2	3	97	43	1901	45	M	15	12a	yes	spread	oxer	right	no	0	0	75,44	no
Helsinki	The Longines FEI World Cup Jumping - Helsinki round A	22.10.2017	CSI-W5*	4	1.40 - 1.60	450	63	11	22.03.2007	G	KWPN	175	2	3	97	43	1901	45	M	16	12b	yes	vertical	vertical	right	yes	4	0	75,44	no
Helsinki	The Longines FEI World Cup Jumping - Helsinki round A	22.10.2017	CSI-W5*	4	1.40 - 1.60	450	63	11	22.03.2007	G	KWPN	175	2	3	97	43	1901	45	M	17	13	no	spread	oxer	right	no	0	0	75,44	no
Helsinki	The Longines FEI World Cup Jumping - Helsinki round A	22.10.2017	CSI-W5*	4	1.40 - 1.60	450	61	10	12.04.2008	M	ZANG	82	0	6	421	45	483	7	F	1	1	no	vertical	vertical	right	no	0	0	73,72	no
Helsinki	The Longines FEI World Cup Jumping - Helsinki round A	22.10.2017	CSI-W5*	4	1.40 - 1.60	450	61	10	12.04.2008	M	ZANG	82	0	6	421	45	483	7	F	2	2	no	vertical	vertical	left	no	0	0	73,72	no
Helsinki	The Longines FEI World Cup Jumping - Helsinki round A	22.10.2017	CSI-W5*	4	1.40 - 1.60	450	61	10	12.04.2008	M	ZANG	82	0	6	421	45	483	7	F	3	3	no	spread	oxer	left	no	0	0	73,72	no
Helsinki	The Longines FEI World Cup Jumping - Helsinki round A	22.10.2017	CSI-W5*	4	1.40 - 1.60	450	61	10	12.04.2008	M	ZANG	82	0	6	421	45	483	7	F	4	4a	yes	spread	oxer	left	no	0	0	73,72	no
Helsinki	The Longines FEI World Cup Jumping - Helsinki round A	22.10.2017	CSI-W5*	4	1.40 - 1.60	450	61	10	12.04.2008	M	ZANG	82	0	6	421	45	483	7	F	5	4b	yes	vertical	vertical	left	no	0	0	73,72	no
Helsinki	The Longines FEI World Cup Jumping - Helsinki round A	22.10.2017	CSI-W5*	4	1.40 - 1.60	450	61	10	12.04.2008	M	ZANG	82	0	6	421	45	483	7	F	6	4c	yes	spread	oxer	left	no	0	0	73,72	no
Helsinki	The Longines FEI World Cup Jumping - Helsinki round A	22.10.2017	CSI-W5*	4	1.40 - 1.60	450	61	10	12.04.2008	M	ZANG	82	0	6	421	45	483	7	F	7	5	no	spread	oxer	right	no	0	0	73,72	no
Helsinki	The Longines FEI World Cup Jumping - Helsinki round A	22.10.2017	CSI-W5*	4	1.40 - 1.60	450	61	10	12.04.2008	M	ZANG	82	0	6	421	45	483	7	F	8	6a	yes	vertical	vertical	right	no	0	0	73,72	no
Helsinki	The Longines FEI World Cup Jumping - Helsinki round A	22.10.2017	CSI-W5*	4	1.40 - 1.60	450	61	10	12.04.2008	M	ZANG	82	0	6	421	45	483	7	F	9	6b	yes	vertical	vertical	right	no	0	0	73,72	no
Helsinki	The Longines FEI World Cup Jumping - Helsinki round A	22.10.2017	CSI-W5*	4	1.40 - 1.60	450	61	10	12.04.2008	M	ZANG	82	0	6	421	45	483	7	F	10	7	no	spread	oxer	left	no	0	0	73,72	no
Helsinki	The Longines FEI World Cup Jumping - Helsinki round A	22.10.2017	CSI-W5*	4	1.40 - 1.60	450	61	10	12.04.2008	M	ZANG	82	0	6	421	45	483	7	F	11	8	no	vertical	wall	left	no	0	0	73,72	no
Helsinki	The Longines FEI World Cup Jumping - Helsinki round A	22.10.2017	CSI-W5*	4	1.40 - 1.60	450	61	10	12.04.2008	M	ZANG	82	0	6	421	45	483	7	F	12	9	no	spread	oxer	straight	no	0	0	73,72	no
Helsinki	The Longines FEI World Cup Jumping - Helsinki round A	22.10.2017	CSI-W5*	4	1.40 - 1.60	450	61	10	12.04.2008	M	ZANG	82	0	6	421	45	483	7	F	13	10	no	spread	triple bar	left	no	0	0	73,72	no
Helsinki	The Longines FEI World Cup Jumping - Helsinki round A	22.10.2017	CSI-W5*	4	1.40 - 1.60	450	61	10	12.04.2008	M	ZANG	82	0	6	421	45	483	7	F	14	11	no	vertical	vertical with water	straight	no	0	0	73,72	no
Helsinki	The Longines FEI World Cup Jumping - Helsinki round A	22.10.2017	CSI-W5*	4	1.40 - 1.60	450	61	10	12.04.2008	M	ZANG	82	0	6	421	45	483	7	F	15	12a	yes	spread	oxer	right	yes	4	0	73,72	no
Helsinki	The Longines FEI World Cup Jumping - Helsinki round A	22.10.2017	CSI-W5*	4	1.40 - 1.60	450	61	10	12.04.2008	M	ZANG	82	0	6	421	45	483	7	F	16	12b	yes	vertical	vertical	right	no	0	0	73,72	no
Helsinki	The Longines FEI World Cup Jumping - Helsinki round A	22.10.2017	CSI-W5*	4	1.40 - 1.60	450	61	10	12.04.2008	M	ZANG	82	0	6	421	45	483	7	F	17	13	no	spread	oxer	right	yes	4	0	73,72	no
Helsinki	The Longines FEI World Cup Jumping - Helsinki round A	22.10.2017	CSI-W5*	4	1.40 - 1.60	450	20	11	16.04.2007	G	OS	121	0	7	48	58	1857	49	M	1	1	no	vertical	vertical	right	no	0	0	0,00	yes
Helsinki	The Longines FEI World Cup Jumping - Helsinki round A	22.10.2017	CSI-W5*	4	1.40 - 1.60	450	20	11	16.04.2007	G	OS	121	0	7	48	58	1857	49	M	2	2	no	vertical	vertical	left	no	0	0	0,00	yes
Helsinki	The Longines FEI World Cup Jumping - Helsinki round A	22.10.2017	CSI-W5*	4	1.40 - 1.60	450	20	11	16.04.2007	G	OS	121	0	7	48	58	1857	49	M	3	3	no	spread	oxer	left	no	0	0	0,00	yes
Helsinki	The Longines FEI World Cup Jumping - Helsinki round A	22.10.2017	CSI-W5*	4	1.40 - 1.60	450	20	11	16.04.2007	G	OS	121	0	7	48	58	1857	49	M	4	4a	yes	spread	oxer	left	no	0	0	0,00	yes
Helsinki	The Longines FEI World Cup Jumping - Helsinki round A	22.10.2017	CSI-W5*	4	1.40 - 1.60	450	20	11	16.04.2007	G	OS	121	0	7	48	58	1857	49	M	5	4b	yes	vertical	vertical	left	yes	4	0	0,00	yes
Helsinki	The Longines FEI World Cup Jumping - Helsinki round A	22.10.2017	CSI-W5*	4	1.40 - 1.60	450	20	11	16.04.2007	G	OS	121	0	7	48	58	1857	49	M	6	4c	yes	spread	oxer	left	no	0	0	0,00	yes
Helsinki	The Longines FEI World Cup Jumping - Helsinki round A	22.10.2017	CSI-W5*	4	1.40 - 1.60	450	20	11	16.04.2007	G	OS	121	0	7	48	58	1857	49	M	7	5	no	spread	oxer	right	no	0	0	0,00	yes
Helsinki	The Longines FEI World Cup Jumping - Helsinki round A	22.10.2017	CSI-W5*	4	1.40 - 1.60	450	20	11	16.04.2007	G	OS	121	0	7	48	58	1857	49	M	8	6a	yes	vertical	vertical	right	no	0	0	0,00	yes
Helsinki	The Longines FEI World Cup Jumping - Helsinki round A	22.10.2017	CSI-W5*	4	1.40 - 1.60	450	20	11	16.04.2007	G	OS	121	0	7	48	58	1857	49	M	9	6b	yes	vertical	vertical	right	yes	4	0	0,00	yes
Helsinki	The Longines FEI World Cup Jumping - Helsinki round A	22.10.2017	CSI-W5*	4	1.40 - 1.60	450	20	11	16.04.2007	G	OS	121	0	7	48	58	1857	49	M	10	7	no	spread	oxer	left	no	0	0	0,00	yes
Helsinki	The Longines FEI World Cup Jumping - Helsinki round A	22.10.2017	CSI-W5*	4	1.40 - 1.60	450	20	11	16.04.2007	G	OS	121	0	7	48	58	1857	49	M	11	8	no	vertical	wall	left	no	0	0	0,00	yes
Helsinki	The Longines FEI World Cup Jumping - Helsinki round A	22.10.2017	CSI-W5*	4	1.40 - 1.60	450	20	11	16.04.2007	G	OS	121	0	7	48	58	1857	49	M	12	9	no	spread	oxer	straight	yes	4	0	0,00	yes
Helsinki	The Longines FEI World Cup Jumping - Helsinki round A	22.10.2017	CSI-W5*	4	1.40 - 1.60	450	20	11	16.04.2007	G	OS	121	0	7	48	58	1857	49	M	13	10	no	spread	triple bar	left	no	0	0	0,00	yes
Helsinki	The Longines FEI World Cup Jumping - Helsinki round A	22.10.2017	CSI-W5*	4	1.40 - 1.60	450	20	11	16.04.2007	G	OS	121	0	7	48	58	1857	49	M	14	11	no	vertical	vertical with water	straight	no	0	0	0,00	yes
Helsinki	The Longines FEI World Cup Jumping - Helsinki round A	22.10.2017	CSI-W5*	4	1.40 - 1.60	450	20	11	16.04.2007	G	OS	121	0	7	48	58	1857	49	M	15	12a	yes	spread	oxer	right	no	0	0	0,00	yes
Helsinki	The Longines FEI World Cup Jumping - Helsinki round A	22.10.2017	CSI-W5*	4	1.40 - 1.60	450	20	11	16.04.2007	G	OS	121	0	7	48	58	1857	49	M	16	12b	yes	vertical	vertical	right	no	0	0	0,00	yes
Helsinki	The Longines FEI World Cup Jumping - Helsinki round A	22.10.2017	CSI-W5*	4	1.40 - 1.60	450	20	11	16.04.2007	G	OS	121	0	7	48	58	1857	49	M	17	13	no	spread	oxer	right	no	0	0	0,00	yes
Helsinki	The Longines FEI World Cup Jumping - Helsinki round A	22.10.2017	CSI-W5*	4	1.40 - 1.60	450	125	16	07.05.2002	S	BWP	121	1	8	1160	43	175	1	M	1	1	no	vertical	vertical	right	no	0	0	0,00	yes
Helsinki	The Longines FEI World Cup Jumping - Helsinki round A	22.10.2017	CSI-W5*	4	1.40 - 1.60	450	125	16	07.05.2002	S	BWP	121	1	8	1160	43	175	1	M	2	2	no	vertical	vertical	left	no	0	0	0,00	yes
Helsinki	The Longines FEI World Cup Jumping - Helsinki round A	22.10.2017	CSI-W5*	4	1.40 - 1.60	450	125	16	07.05.2002	S	BWP	121	1	8	1160	43	175	1	M	3	3	no	spread	oxer	left	yes	4	0	0,00	yes
Helsinki	The Longines FEI World Cup Jumping - Helsinki round A	22.10.2017	CSI-W5*	4	1.40 - 1.60	450	125	16	07.05.2002	S	BWP	121	1	8	1160	43	175	1	M	4	4a	yes	spread	oxer	left	no	0	0	0,00	yes
Helsinki	The Longines FEI World Cup Jumping - Helsinki round A	22.10.2017	CSI-W5*	4	1.40 - 1.60	450	125	16	07.05.2002	S	BWP	121	1	8	1160	43	175	1	M	5	4b	yes	vertical	vertical	left	no	0	0	0,00	yes
Helsinki	The Longines FEI World Cup Jumping - Helsinki round A	22.10.2017	CSI-W5*	4	1.40 - 1.60	450	125	16	07.05.2002	S	BWP	121	1	8	1160	43	175	1	M	6	4c	yes	spread	oxer	left	yes	4	0	0,00	yes
Helsinki	The Longines FEI World Cup Jumping - Helsinki round A	22.10.2017	CSI-W5*	4	1.40 - 1.60	450	125	16	07.05.2002	S	BWP	121	1	8	1160	43	175	1	M	7	5	no	spread	oxer	right	no	0	0	0,00	yes
Helsinki	The Longines FEI World Cup Jumping - Helsinki round A	22.10.2017	CSI-W5*	4	1.40 - 1.60	450	125	16	07.05.2002	S	BWP	121	1	8	1160	43	175	1	M	8	6a	yes	vertical	vertical	right	no	0	0	0,00	yes
Helsinki	The Longines FEI World Cup Jumping - Helsinki round A	22.10.2017	CSI-W5*	4	1.40 - 1.60	450	125	16	07.05.2002	S	BWP	121	1	8	1160	43	175	1	M	9	6b	yes	vertical	vertical	right	yes	4	0	0,00	yes
Helsinki	The Longines FEI World Cup Jumping - Helsinki round A	22.10.2017	CSI-W5*	4	1.40 - 1.60	450	125	16	07.05.2002	S	BWP	121	1	8	1160	43	175	1	M	10	7	no	spread	oxer	left	yes	4	0	0,00	yes
Helsinki	The Longines FEI World Cup Jumping - Helsinki round A	22.10.2017	CSI-W5*	4	1.40 - 1.60	450	125	16	07.05.2002	S	BWP	121	1	8	1160	43	175	1	M	11	8	no	vertical	wall	left	no	0	0	0,00	yes
Helsinki	The Longines FEI World Cup Jumping - Helsinki round A	22.10.2017	CSI-W5*	4	1.40 - 1.60	450	125	16	07.05.2002	S	BWP	121	1	8	1160	43	175	1	M	12	9	no	spread	oxer	straight	no	0	0	0,00	yes
Helsinki	The Longines FEI World Cup Jumping - Helsinki round A	22.10.2017	CSI-W5*	4	1.40 - 1.60	450	125	16	07.05.2002	S	BWP	121	1	8	1160	43	175	1	M	13	10	no	spread	triple bar	left	no	0	0	0,00	yes
Helsinki	The Longines FEI World Cup Jumping - Helsinki round A	22.10.2017	CSI-W5*	4	1.40 - 1.60	450	125	16	07.05.2002	S	BWP	121	1	8	1160	43	175	1	M	14	11	no	vertical	vertical with water	straight	no	0	0	0,00	yes
Helsinki	The Longines FEI World Cup Jumping - Helsinki round A	22.10.2017	CSI-W5*	4	1.40 - 1.60	450	125	16	07.05.2002	S	BWP	121	1	8	1160	43	175	1	M	15	12a	yes	spread	oxer	right	no	0	0	0,00	yes
Helsinki	The Longines FEI World Cup Jumping - Helsinki round A	22.10.2017	CSI-W5*	4	1.40 - 1.60	450	125	16	07.05.2002	S	BWP	121	1	8	1160	43	175	1	M	16	12b	yes	vertical	vertical	right	no	0	0	0,00	yes
Helsinki	The Longines FEI World Cup Jumping - Helsinki round A	22.10.2017	CSI-W5*	4	1.40 - 1.60	450	125	16	07.05.2002	S	BWP	121	1	8	1160	43	175	1	M	17	13	no	spread	oxer	right	no	0	0	0,00	yes
Helsinki	The Longines FEI World Cup Jumping - Helsinki round A	22.10.2017	CSI-W5*	4	1.40 - 1.60	450	195	11	25.04.2007	G	SF	164	16	9	103	48	1417	59	M	1	1	no	vertical	vertical	right	no	0	0	73,2	no
Helsinki	The Longines FEI World Cup Jumping - Helsinki round A	22.10.2017	CSI-W5*	4	1.40 - 1.60	450	195	11	25.04.2007	G	SF	164	16	9	103	48	1417	59	M	2	2	no	vertical	vertical	left	no	0	0	73,2	no
Helsinki	The Longines FEI World Cup Jumping - Helsinki round A	22.10.2017	CSI-W5*	4	1.40 - 1.60	450	195	11	25.04.2007	G	SF	164	16	9	103	48	1417	59	M	3	3	no	spread	oxer	left	no	0	0	73,2	no
Helsinki	The Longines FEI World Cup Jumping - Helsinki round A	22.10.2017	CSI-W5*	4	1.40 - 1.60	450	195	11	25.04.2007	G	SF	164	16	9	103	48	1417	59	M	4	4a	yes	spread	oxer	left	no	0	0	73,2	no
Helsinki	The Longines FEI World Cup Jumping - Helsinki round A	22.10.2017	CSI-W5*	4	1.40 - 1.60	450	195	11	25.04.2007	G	SF	164	16	9	103	48	1417	59	M	5	4b	yes	vertical	vertical	left	no	0	0	73,2	no
Helsinki	The Longines FEI World Cup Jumping - Helsinki round A	22.10.2017	CSI-W5*	4	1.40 - 1.60	450	195	11	25.04.2007	G	SF	164	16	9	103	48	1417	59	M	6	4c	yes	spread	oxer	left	no	0	0	73,2	no
Helsinki	The Longines FEI World Cup Jumping - Helsinki round A	22.10.2017	CSI-W5*	4	1.40 - 1.60	450	195	11	25.04.2007	G	SF	164	16	9	103	48	1417	59	M	7	5	no	spread	oxer	right	no	0	0	73,2	no
Helsinki	The Longines FEI World Cup Jumping - Helsinki round A	22.10.2017	CSI-W5*	4	1.40 - 1.60	450	195	11	25.04.2007	G	SF	164	16	9	103	48	1417	59	M	8	6a	yes	vertical	vertical	right	no	0	0	73,2	no
Helsinki	The Longines FEI World Cup Jumping - Helsinki round A	22.10.2017	CSI-W5*	4	1.40 - 1.60	450	195	11	25.04.2007	G	SF	164	16	9	103	48	1417	59	M	9	6b	yes	vertical	vertical	right	no	0	0	73,2	no
Helsinki	The Longines FEI World Cup Jumping - Helsinki round A	22.10.2017	CSI-W5*	4	1.40 - 1.60	450	195	11	25.04.2007	G	SF	164	16	9	103	48	1417	59	M	10	7	no	spread	oxer	left	no	0	0	73,2	no
Helsinki	The Longines FEI World Cup Jumping - Helsinki round A	22.10.2017	CSI-W5*	4	1.40 - 1.60	450	195	11	25.04.2007	G	SF	164	16	9	103	48	1417	59	M	11	8	no	vertical	wall	left	no	0	0	73,2	no
Helsinki	The Longines FEI World Cup Jumping - Helsinki round A	22.10.2017	CSI-W5*	4	1.40 - 1.60	450	195	11	25.04.2007	G	SF	164	16	9	103	48	1417	59	M	12	9	no	spread	oxer	straight	no	0	0	73,2	no
Helsinki	The Longines FEI World Cup Jumping - Helsinki round A	22.10.2017	CSI-W5*	4	1.40 - 1.60	450	195	11	25.04.2007	G	SF	164	16	9	103	48	1417	59	M	13	10	no	spread	triple bar	left	no	0	0	73,2	no
Helsinki	The Longines FEI World Cup Jumping - Helsinki round A	22.10.2017	CSI-W5*	4	1.40 - 1.60	450	195	11	25.04.2007	G	SF	164	16	9	103	48	1417	59	M	14	11	no	vertical	vertical with water	straight	no	0	0	73,2	no
Helsinki	The Longines FEI World Cup Jumping - Helsinki round A	22.10.2017	CSI-W5*	4	1.40 - 1.60	450	195	11	25.04.2007	G	SF	164	16	9	103	48	1417	59	M	15	12a	yes	spread	oxer	right	no	0	0	73,2	no
Helsinki	The Longines FEI World Cup Jumping - Helsinki round A	22.10.2017	CSI-W5*	4	1.40 - 1.60	450	195	11	25.04.2007	G	SF	164	16	9	103	48	1417	59	M	16	12b	yes	vertical	vertical	right	no	0	0	73,2	no
Helsinki	The Longines FEI World Cup Jumping - Helsinki round A	22.10.2017	CSI-W5*	4	1.40 - 1.60	450	195	11	25.04.2007	G	SF	164	16	9	103	48	1417	59	M	17	13	no	spread	oxer	right	no	0	0	73,2	no
Helsinki	The Longines FEI World Cup Jumping - Helsinki round B	22.10.2017	CSI-W5*	4	1.40 - 1.60	270	195	11	25.04.2007	G	SF	164	16	9	103	48	1417	59	M	1	1	no	spread	oxer	right	no	0	0	39,11	no
Helsinki	The Longines FEI World Cup Jumping - Helsinki round B	22.10.2017	CSI-W5*	4	1.40 - 1.60	270	195	11	25.04.2007	G	SF	164	16	9	103	48	1417	59	M	2	2	no	vertical	vertical	right	no	0	0	39,11	no
Helsinki	The Longines FEI World Cup Jumping - Helsinki round B	22.10.2017	CSI-W5*	4	1.40 - 1.60	270	195	11	25.04.2007	G	SF	164	16	9	103	48	1417	59	M	3	3	no	spread	oxer	left	no	0	0	39,11	no
Helsinki	The Longines FEI World Cup Jumping - Helsinki round B	22.10.2017	CSI-W5*	4	1.40 - 1.60	270	195	11	25.04.2007	G	SF	164	16	9	103	48	1417	59	M	4	4a	yes	vertical	vertical	left	no	0	0	39,11	no
Helsinki	The Longines FEI World Cup Jumping - Helsinki round B	22.10.2017	CSI-W5*	4	1.40 - 1.60	270	195	11	25.04.2007	G	SF	164	16	9	103	48	1417	59	M	5	4b	yes	spread	oxer	left	no	0	0	39,11	no
Helsinki	The Longines FEI World Cup Jumping - Helsinki round B	22.10.2017	CSI-W5*	4	1.40 - 1.60	270	195	11	25.04.2007	G	SF	164	16	9	103	48	1417	59	M	6	5	no	spread	oxer	right	no	0	0	39,11	no
Helsinki	The Longines FEI World Cup Jumping - Helsinki round B	22.10.2017	CSI-W5*	4	1.40 - 1.60	270	195	11	25.04.2007	G	SF	164	16	9	103	48	1417	59	M	7	6	no	vertical	vertical	right	no	0	0	39,11	no
Helsinki	The Longines FEI World Cup Jumping - Helsinki round B	22.10.2017	CSI-W5*	4	1.40 - 1.60	270	195	11	25.04.2007	G	SF	164	16	9	103	48	1417	59	M	8	7	no	spread	oxer	left	yes	4	0	39,11	no
Helsinki	The Longines FEI World Cup Jumping - Helsinki round A	22.10.2017	CSI-W5*	4	1.40 - 1.60	450	73	17	02.05.2001	G	BWP	229	4	12	235	58	1047	25	M	1	1	no	vertical	vertical	right	no	0	0	74,82	no
Helsinki	The Longines FEI World Cup Jumping - Helsinki round A	22.10.2017	CSI-W5*	4	1.40 - 1.60	450	73	17	02.05.2001	G	BWP	229	4	12	235	58	1047	25	M	2	2	no	vertical	vertical	left	no	0	0	74,82	no
Helsinki	The Longines FEI World Cup Jumping - Helsinki round A	22.10.2017	CSI-W5*	4	1.40 - 1.60	450	73	17	02.05.2001	G	BWP	229	4	12	235	58	1047	25	M	3	3	no	spread	oxer	left	no	0	0	74,82	no
Helsinki	The Longines FEI World Cup Jumping - Helsinki round A	22.10.2017	CSI-W5*	4	1.40 - 1.60	450	73	17	02.05.2001	G	BWP	229	4	12	235	58	1047	25	M	4	4a	yes	spread	oxer	left	no	0	0	74,82	no
Helsinki	The Longines FEI World Cup Jumping - Helsinki round A	22.10.2017	CSI-W5*	4	1.40 - 1.60	450	73	17	02.05.2001	G	BWP	229	4	12	235	58	1047	25	M	5	4b	yes	vertical	vertical	left	no	0	0	74,82	no

Helsinki	The Longines FEI World Cup Jumping - Helsinki round A	22.10.2017	CSI-W5*	4	1.40 - 1.60	450	73	17	02.05.2001	G	BWP	229	4	12	235	58	1047	25	M	6	4c	yes	spread	oxer	left	no	0	0	74,82	no
Helsinki	The Longines FEI World Cup Jumping - Helsinki round A	22.10.2017	CSI-W5*	4	1.40 - 1.60	450	73	17	02.05.2001	G	BWP	229	4	12	235	58	1047	25	M	7	5	no	spread	oxer	right	no	0	0	74,82	no
Helsinki	The Longines FEI World Cup Jumping - Helsinki round A	22.10.2017	CSI-W5*	4	1.40 - 1.60	450	73	17	02.05.2001	G	BWP	229	4	12	235	58	1047	25	M	8	6a	yes	vertical	vertical	right	no	0	0	74,82	no
Helsinki	The Longines FEI World Cup Jumping - Helsinki round A	22.10.2017	CSI-W5*	4	1.40 - 1.60	450	73	17	02.05.2001	G	BWP	229	4	12	235	58	1047	25	M	9	6b	yes	vertical	vertical	right	no	0	0	74,82	no
Helsinki	The Longines FEI World Cup Jumping - Helsinki round A	22.10.2017	CSI-W5*	4	1.40 - 1.60	450	73	17	02.05.2001	G	BWP	229	4	12	235	58	1047	25	M	10	7	no	spread	oxer	left	no	0	0	74,82	no
Helsinki	The Longines FEI World Cup Jumping - Helsinki round A	22.10.2017	CSI-W5*	4	1.40 - 1.60	450	73	17	02.05.2001	G	BWP	229	4	12	235	58	1047	25	M	11	8	no	vertical	wall	left	no	0	0	74,82	no
Helsinki	The Longines FEI World Cup Jumping - Helsinki round A	22.10.2017	CSI-W5*	4	1.40 - 1.60	450	73	17	02.05.2001	G	BWP	229	4	12	235	58	1047	25	M	12	9	no	spread	oxer	straight	no	0	0	74,82	no
Helsinki	The Longines FEI World Cup Jumping - Helsinki round A	22.10.2017	CSI-W5*	4	1.40 - 1.60	450	73	17	02.05.2001	G	BWP	229	4	12	235	58	1047	25	M	13	10	no	spread	triple bar	left	no	0	0	74,82	no
Helsinki	The Longines FEI World Cup Jumping - Helsinki round A	22.10.2017	CSI-W5*	4	1.40 - 1.60	450	73	17	02.05.2001	G	BWP	229	4	12	235	58	1047	25	M	14	11	no	vertical	vertical with water	straight	no	0	0	74,82	no
Helsinki	The Longines FEI World Cup Jumping - Helsinki round A	22.10.2017	CSI-W5*	4	1.40 - 1.60	450	73	17	02.05.2001	G	BWP	229	4	12	235	58	1047	25	M	15	12a	yes	spread	oxer	right	no	0	0	74,82	no
Helsinki	The Longines FEI World Cup Jumping - Helsinki round A	22.10.2017	CSI-W5*	4	1.40 - 1.60	450	73	17	02.05.2001	G	BWP	229	4	12	235	58	1047	25	M	16	12b	yes	vertical	vertical	right	no	0	0	74,82	no
Helsinki	The Longines FEI World Cup Jumping - Helsinki round A	22.10.2017	CSI-W5*	4	1.40 - 1.60	450	73	17	02.05.2001	G	BWP	229	4	12	235	58	1047	25	M	17	13	no	spread	oxer	right	no	0	0	74,82	no
Helsinki	The Longines FEI World Cup Jumping - Helsinki round B	22.10.2017	CSI-W5*	4	1.40 - 1.60	270	73	17	02.05.2001	G	BWP	229	4	12	235	58	1047	25	M	1	1	no	spread	oxer	right	yes	4	0	43,37	no
Helsinki	The Longines FEI World Cup Jumping - Helsinki round B	22.10.2017	CSI-W5*	4	1.40 - 1.60	270	73	17	02.05.2001	G	BWP	229	4	12	235	58	1047	25	M	2	2	no	vertical	vertical	right	no	0	0	43,37	no
Helsinki	The Longines FEI World Cup Jumping - Helsinki round B	22.10.2017	CSI-W5*	4	1.40 - 1.60	270	73	17	02.05.2001	G	BWP	229	4	12	235	58	1047	25	M	3	3	no	spread	oxer	left	yes	4	0	43,37	no
Helsinki	The Longines FEI World Cup Jumping - Helsinki round B	22.10.2017	CSI-W5*	4	1.40 - 1.60	270	73	17	02.05.2001	G	BWP	229	4	12	235	58	1047	25	M	4	4a	yes	vertical	vertical	left	no	0	0	43,37	no
Helsinki	The Longines FEI World Cup Jumping - Helsinki round B	22.10.2017	CSI-W5*	4	1.40 - 1.60	270	73	17	02.05.2001	G	BWP	229	4	12	235	58	1047	25	M	5	4b	yes	spread	oxer	left	no	0	0	43,37	no
Helsinki	The Longines FEI World Cup Jumping - Helsinki round B	22.10.2017	CSI-W5*	4	1.40 - 1.60	270	73	17	02.05.2001	G	BWP	229	4	12	235	58	1047	25	M	6	5	no	spread	oxer	right	no	0	0	43,37	no
Helsinki	The Longines FEI World Cup Jumping - Helsinki round B	22.10.2017	CSI-W5*	4	1.40 - 1.60	270	73	17	02.05.2001	G	BWP	229	4	12	235	58	1047	25	M	7	6	no	vertical	vertical	right	no	0	0	43,37	no
Helsinki	The Longines FEI World Cup Jumping - Helsinki round B	22.10.2017	CSI-W5*	4	1.40 - 1.60	270	73	17	02.05.2001	G	BWP	229	4	12	235	58	1047	25	M	8	7	no	spread	oxer	left	no	0	0	43,37	no
Helsinki	The Longines FEI World Cup Jumping - Helsinki round A	22.10.2017	CSI-W5*	4	1.40 - 1.60	450	212	12	10.04.2006	S	BWP	134	11	13	42	49	1996	131	M	1	1	no	vertical	vertical	right	no	0	0	74,41	no
Helsinki	The Longines FEI World Cup Jumping - Helsinki round A	22.10.2017	CSI-W5*	4	1.40 - 1.60	450	212	12	10.04.2006	S	BWP	134	11	13	42	49	1996	131	M	2	2	no	vertical	vertical	left	no	0	0	74,41	no
Helsinki	The Longines FEI World Cup Jumping - Helsinki round A	22.10.2017	CSI-W5*	4	1.40 - 1.60	450	212	12	10.04.2006	S	BWP	134	11	13	42	49	1996	131	M	3	3	no	spread	oxer	left	no	0	0	74,41	no
Helsinki	The Longines FEI World Cup Jumping - Helsinki round A	22.10.2017	CSI-W5*	4	1.40 - 1.60	450	212	12	10.04.2006	S	BWP	134	11	13	42	49	1996	131	M	4	4a	yes	spread	oxer	left	no	0	0	74,41	no
Helsinki	The Longines FEI World Cup Jumping - Helsinki round A	22.10.2017	CSI-W5*	4	1.40 - 1.60	450	212	12	10.04.2006	S	BWP	134	11	13	42	49	1996	131	M	5	4b	yes	vertical	vertical	left	no	0	0	74,41	no
Helsinki	The Longines FEI World Cup Jumping - Helsinki round A	22.10.2017	CSI-W5*	4	1.40 - 1.60	450	212	12	10.04.2006	S	BWP	134	11	13	42	49	1996	131	M	6	4c	yes	spread	oxer	left	no	0	0	74,41	no
Helsinki	The Longines FEI World Cup Jumping - Helsinki round A	22.10.2017	CSI-W5*	4	1.40 - 1.60	450	212	12	10.04.2006	S	BWP	134	11	13	42	49	1996	131	M	7	5	no	spread	oxer	right	no	0	0	74,41	no
Helsinki	The Longines FEI World Cup Jumping - Helsinki round A	22.10.2017	CSI-W5*	4	1.40 - 1.60	450	212	12	10.04.2006	S	BWP	134	11	13	42	49	1996	131	M	8	6a	yes	vertical	vertical	right	no	0	0	74,41	no
Helsinki	The Longines FEI World Cup Jumping - Helsinki round A	22.10.2017	CSI-W5*	4	1.40 - 1.60	450	212	12	10.04.2006	S	BWP	134	11	13	42	49	1996	131	M	9	6b	yes	vertical	vertical	right	no	0	0	74,41	no
Helsinki	The Longines FEI World Cup Jumping - Helsinki round A	22.10.2017	CSI-W5*	4	1.40 - 1.60	450	212	12	10.04.2006	S	BWP	134	11	13	42	49	1996	131	M	10	7	no	spread	oxer	left	no	0	0	74,41	no
Helsinki	The Longines FEI World Cup Jumping - Helsinki round A	22.10.2017	CSI-W5*	4	1.40 - 1.60	450	212	12	10.04.2006	S	BWP	134	11	13	42	49	1996	131	M	11	8	no	vertical	wall	left	no	0	0	74,41	no
Helsinki	The Longines FEI World Cup Jumping - Helsinki round A	22.10.2017	CSI-W5*	4	1.40 - 1.60	450	212	12	10.04.2006	S	BWP	134	11	13	42	49	1996	131	M	12	9	no	spread	oxer	straight	no	0	0	74,41	no
Helsinki	The Longines FEI World Cup Jumping - Helsinki round A	22.10.2017	CSI-W5*	4	1.40 - 1.60	450	212	12	10.04.2006	S	BWP	134	11	13	42	49	1996	131	M	13	10	no	spread	triple bar	left	no	0	0	74,41	no
Helsinki	The Longines FEI World Cup Jumping - Helsinki round A	22.10.2017	CSI-W5*	4	1.40 - 1.60	450	212	12	10.04.2006	S	BWP	134	11	13	42	49	1996	131	M	14	11	no	vertical	vertical with water	straight	no	0	0	74,41	no
Helsinki	The Longines FEI World Cup Jumping - Helsinki round A	22.10.2017	CSI-W5*	4	1.40 - 1.60	450	212	12	10.04.2006	S	BWP	134	11	13	42	49	1996	131	M	15	12a	yes	spread	oxer	right	no	0	0	74,41	no
Helsinki	The Longines FEI World Cup Jumping - Helsinki round A	22.10.2017	CSI-W5*	4	1.40 - 1.60	450	212	12	10.04.2006	S	BWP	134	11	13	42	49	1996	131	M	16	12b	yes	vertical	vertical	right	no	0	0	74,41	no
Helsinki	The Longines FEI World Cup Jumping - Helsinki round A	22.10.2017	CSI-W5*	4	1.40 - 1.60	450	212	12	10.04.2006	S	BWP	134	11	13	42	49	1996	131	M	17	13	no	spread	oxer	right	no	0	0	74,41	no
Helsinki	The Longines FEI World Cup Jumping - Helsinki round B	22.10.2017	CSI-W5*	4	1.40 - 1.60	270	212	12	10.04.2006	S	BWP	134	11	13	42	49	1996	131	M	1	1	no	spread	oxer	right	no	0	0	36,85	no
Helsinki	The Longines FEI World Cup Jumping - Helsinki round B	22.10.2017	CSI-W5*	4	1.40 - 1.60	270	212	12	10.04.2006	S	BWP	134	11	13	42	49	1996	131	M	2	2	no	vertical	vertical	right	no	0	0	36,85	no
Helsinki	The Longines FEI World Cup Jumping - Helsinki round B	22.10.2017	CSI-W5*	4	1.40 - 1.60	270	212	12	10.04.2006	S	BWP	134	11	13	42	49	1996	131	M	3	3	no	spread	oxer	left	no	0	0	36,85	no
Helsinki	The Longines FEI World Cup Jumping - Helsinki round B	22.10.2017	CSI-W5*	4	1.40 - 1.60	270	212	12	10.04.2006	S	BWP	134	11	13	42	49	1996	131	M	4	4a	yes	vertical	vertical	left	no	0	0	36,85	no
Helsinki	The Longines FEI World Cup Jumping - Helsinki round B	22.10.2017	CSI-W5*	4	1.40 - 1.60	270	212	12	10.04.2006	S	BWP	134	11	13	42	49	1996	131	M	5	4b	yes	spread	oxer	left	no	0	0	36,85	no
Helsinki	The Longines FEI World Cup Jumping - Helsinki round B	22.10.2017	CSI-W5*	4	1.40 - 1.60	270	212	12	10.04.2006	S	BWP	134	11	13	42	49	1996	131	M	6	5	no	spread	oxer	right	no	0	0	36,85	no
Helsinki	The Longines FEI World Cup Jumping - Helsinki round B	22.10.2017	CSI-W5*	4	1.40 - 1.60	270	212	12	10.04.2006	S	BWP	134	11	13	42	49	1996	131	M	7	6	no	vertical	vertical	right	no	0	0	36,85	no
Helsinki	The Longines FEI World Cup Jumping - Helsinki round B	22.10.2017	CSI-W5*	4	1.40 - 1.60	270	212	12	10.04.2006	S	BWP	134	11	13	42	49	1996	131	M	8	7	no	spread	oxer	left	no	0	0	36,85	no
Helsinki	The Longines FEI World Cup Jumping - Helsinki round A	22.10.2017	CSI-W5*	4	1.40 - 1.60	450	66	10	02.04.2008	M	KWPN	107	2	15	138	49	1209	30	F	1	1	no	vertical	vertical	right	no	0	0	0,00	yes
Helsinki	The Longines FEI World Cup Jumping - Helsinki round A	22.10.2017	CSI-W5*	4	1.40 - 1.60	450	66	10	02.04.2008	M	KWPN	107	2	15	138	49	1209	30	F	2	2	no	vertical	vertical	left	no	0	0	0,00	yes
Helsinki	The Longines FEI World Cup Jumping - Helsinki round A	22.10.2017	CSI-W5*	4	1.40 - 1.60	450	66	10	02.04.2008	M	KWPN	107	2	15	138	49	1209	30	F	3	3	no	spread	oxer	left	no	0	0	0,00	yes
Helsinki	The Longines FEI World Cup Jumping - Helsinki round A	22.10.2017	CSI-W5*	4	1.40 - 1.60	450	66	10	02.04.2008	M	KWPN	107	2	15	138	49	1209	30	F	4	4a	yes	spread	oxer	left	no	0	0	0,00	yes
Helsinki	The Longines FEI World Cup Jumping - Helsinki round A	22.10.2017	CSI-W5*	4	1.40 - 1.60	450	66	10	02.04.2008	M	KWPN	107	2	15	138	49	1209	30	F	5	4b	yes	vertical	vertical	left	no	0	0	0,00	yes
Helsinki	The Longines FEI World Cup Jumping - Helsinki round A	22.10.2017	CSI-W5*	4	1.40 - 1.60	450	66	10	02.04.2008	M	KWPN	107	2	15	138	49	1209	30	F	6	4c	yes	spread	oxer	left	yes	4	0	0,00	yes
Helsinki	The Longines FEI World Cup Jumping - Helsinki round A	22.10.2017	CSI-W5*	4	1.40 - 1.60	450	66	10	02.04.2008	M	KWPN	107	2	15	138	49	1209	30	F	7	5	no	spread	oxer	right	no	0	0	0,00	yes
Helsinki	The Longines FEI World Cup Jumping - Helsinki round A	22.10.2017	CSI-W5*	4	1.40 - 1.60	450	66	10	02.04.2008	M	KWPN	107	2	15	138	49	1209	30	F	8	6a	yes	vertical	vertical	right	yes	4	0	0,00	yes
Helsinki	The Longines FEI World Cup Jumping - Helsinki round A	22.10.2017	CSI-W5*	4	1.40 - 1.60	450	66	10	02.04.2008	M	KWPN	107	2	15	138	49	1209	30	F	9	6b	yes	vertical	vertical	right	no	0	0	0,00	yes
Helsinki	The Longines FEI World Cup Jumping - Helsinki round A	22.10.2017	CSI-W5*	4	1.40 - 1.60	450	66	10	02.04.2008	M	KWPN	107	2	15	138	49	1209	30	F	10	7	no	spread	oxer	left	no	0	0	0,00	yes
Helsinki	The Longines FEI World Cup Jumping - Helsinki round A	22.10.2017	CSI-W5*	4	1.40 - 1.60	450	66	10	02.04.2008	M	KWPN	107	2	15	138	49	1209	30	F	11	8	no	vertical	wall	left	yes	4	0	0,00	yes
Helsinki	The Longines FEI World Cup Jumping - Helsinki round A	22.10.2017	CSI-W5*	4	1.40 - 1.60	450	66	10	02.04.2008	M	KWPN	107	2	15	138	49	1209	30	F	12	9	no	spread	oxer	straight	no	0	0	0,00	yes
Helsinki	The Longines FEI World Cup Jumping - Helsinki round A	22.10.2017	CSI-W5*	4	1.40 - 1.60	450	66	10	02.04.2008	M	KWPN	107	2	15	138	49	1209	30	F	13	10	no	spread	triple bar	left	no	0	0	0,00	yes
Helsinki	The Longines FEI World Cup Jumping - Helsinki round A	22.10.2017	CSI-W5*	4	1.40 - 1.60	450	66	10	02.04.2008	M	KWPN	107	2	15	138	49	1209	30	F	14	11	no	vertical	vertical with water	straight	no	0	0	0,00	yes
Helsinki	The Longines FEI World Cup Jumping - Helsinki round A	22.10.2017	CSI-W5*	4	1.40 - 1.60	450	66	10	02.04.2008	M	KWPN	107	2	15	138	49	1209	30	F	15	12a	yes	spread	oxer	right	no	0	0	0,00	yes
Helsinki	The Longines FEI World Cup Jumping - Helsinki round A	22.10.2017	CSI-W5*	4	1.40 - 1.60	450	66	10	02.04.2008	M	KWPN	107	2	15	138	49	1209	30	F	16	12b	yes	vertical	vertical	right	no	0	0	0,00	yes
Helsinki	The Longines FEI World Cup Jumping - Helsinki round A	22.10.2017	CSI-W5*	4	1.40 - 1.60	450	66	10	02.04.2008	M	KWPN	107	2	15	138	49	1209	30	F	17	13	no	spread	oxer	right	no	0	5	0,00	yes
Helsinki	The Longines FEI World Cup Jumping - Helsinki round A	22.10.2017	CSI-W5*	4	1.40 - 1.60	450	56	14	12.03.2004	G	OLDBG	203	7	16	17	37	2215	106	M	1	1	no	vertical	vertical	right	no	0	0	73	no
Helsinki	The Longines FEI World Cup Jumping - Helsinki round A	22.10.2017	CSI-W5*	4	1.40 - 1.60	450	56	14	12.03.2004	G	OLDBG	203	7	16	17	37	2215	106	M	2	2	no	vertical	vertical	left	no	0	0	73	no
Helsinki	The Longines FEI World Cup Jumping - Helsinki round A	22.10.2017	CSI-W5*	4	1.40 - 1.60	450	56	14	12.03.2004	G	OLDBG	203	7	16	17	37	2215	106	M	3	3	no	spread	oxer	left	no	0	0	73	no
Helsinki	The Longines FEI World Cup Jumping - Helsinki round A	22.10.2017	CSI-W5*	4	1.40 - 1.60	450	56	14	12.03.2004	G	OLDBG	203	7	16	17	37	2215	106	M	4	4a	yes	spread	oxer	left	no	0	0	73	no
Helsinki	The Longines FEI World Cup Jumping - Helsinki round A	22.10.2017	CSI-W5*	4	1.40 - 1.60	450	56	14	12.03.2004	G	OLDBG	203	7	16	17	37	2215	106	M	5	4b	yes	vertical	vertical	left	no	0	0	73	no
Helsinki	The Longines FEI World Cup Jumping - Helsinki round A	22.10.2017	CSI-W5*	4	1.40 - 1.60	450	56	14	12.03.2004	G	OLDBG	203	7	16	17	37	2215	106	M	6	4c	yes	spread	oxer	left	yes	4	0	73	no
Helsinki	The Longines FEI World Cup Jumping - Helsinki round A	22.10.2017	CSI-W5*	4	1.40 - 1.60	450	56	14	12.03.2004	G	OLDBG	203	7	16	17	37	2215	106	M	7	5	no	spread	oxer	right	no	0	0	73	no
Helsinki	The Longines FEI World Cup Jumping - Helsinki round A	22.10.2017	CSI-W5*	4	1.40 - 1.60	450	56	14	12.03.2004	G	OLDBG	203	7	16	17	37	2215	106	M	8	6a	yes	vertical	vertical	right	no	0	0	73	no
Helsinki	The Longines FEI World Cup Jumping - Helsinki round A	22.10.2017	CSI-W5*	4	1.40 - 1.60	450	56	14	12.03.2004	G	OLDBG	203	7	16	17	37	2215	106	M	9	6b	yes	vertical	vertical	right	no	0	0	73	no
Helsinki	The Longines FEI World Cup Jumping - Helsinki round A	22.10.2017	CSI-W5*	4	1.40 - 1.60	450	56	14	12.03.2004	G	OLDBG	203	7	16	17	37	2215	106	M	10	7	no	spread	oxer	left	yes	4	0	73	no
Helsinki	The Longines FEI World Cup Jumping - Helsinki round A	22.10.2017	CSI-W5*	4	1.40 - 1.60	450	56	14	12.03.2004	G	OLDBG	203	7	16	17	37	2215	106	M	11	8	no	vertical	wall	left	no	0	0	73	no
Helsinki	The Longines FEI World Cup Jumping - Helsinki round A	22.10.2017	CSI-W5*	4	1.40 - 1.60	450	56	14	12.03.2004	G	OLDBG	203	7	16	17	37	2215	106	M	12	9	no	spread	oxer	straight	no	0	0	73	no
Helsinki	The Longines FEI World Cup Jumping - Helsinki round A	22.10.2017	CSI-W5*	4	1.40 - 1.60	450	56	14	12.03.2004	G	OLDBG	203	7	16	17	37	2215	106	M	13	10	no	spread	triple bar	left	no	0	0	73	no
Helsinki	The Longines FEI World Cup Jumping - Helsinki round A	22.10.2017	CSI-W5*	4	1.40 - 1.60	450	56	14	12.03.2004	G	OLDBG	203	7	16	17	37	2215	106	M	14	11	no	vertical	vertical with water	straight	no	0	0	73	no
Helsinki	The Longines FEI World Cup Jumping - Helsinki round A	22.10.2017	CSI-W5*	4	1.40 - 1.60	450	56	14	12.03.2004	G	OLDBG	203	7	16	17	37	2215	106	M	15	12a	yes	spread	oxer	right	no	0	0	73	no
Helsinki	The Longines FEI World Cup Jumping - Helsinki round A	22.10.2017	CSI-W5*	4	1.40 - 1.60	450	56	14	12.03.2004	G	OLDBG	203	7	16	17	37	2215	106	M	16	12b	yes	vertical	vertical	right	no	0	0	73	no
Helsinki	The Longines FEI World Cup Jumping - Helsinki round A	22.10.2017	CSI-W5*	4	1.40 - 1.60	450	56	14	12.03.2004	G	OLDBG	203	7	16	17	37	2215	106	M	17	13	no	spread	oxer	right	no	0	0	73	no
Helsinki	The Longines FEI World Cup Jumping - Helsinki round A	22.10.2017	CSI-W5*	4	1.40 - 1.60	450	104	11	23.03.2007	G	KWPN	95	0	17	3	46	1005	51	M	1	1	no	vertical	vertical	right	no	0	0	76,79	no
Helsinki	The Longines FEI World Cup Jumping - Helsinki round A	22.10.2017	CSI-W5*	4	1.40 - 1.60	450	104	11	23.03.2007	G	KWPN	95	0	17	3	46	1005	51	M	2	2	no	vertical	vertical	left	no	0	0	76,79	no
Helsinki	The Longines FEI World Cup Jumping - Helsinki round A	22.10.2017	CSI-W5*	4	1.40 - 1.60	450	104	11	23.03.2007	G	KWPN	95	0	17	3	46	1005	51	M	3	3	no	spread	oxer	left	no	0	0	76,79	no
Helsinki	The Longines FEI World Cup Jumping - Helsinki round A	22.10.2017	CSI-W5*	4	1.40 - 1.60	450	104	11	23.03.2007	G	KWPN	95	0	17	3	46	1005	51	M	4	4a	yes	spread	oxer	left	no	0	0	76,79	no
Helsinki	The Longines FEI World Cup Jumping - Helsinki round A	22.10.2017	CSI-W5*	4	1.40 - 1.60	450	104	11	23.03.2007	G	KWPN	95	0	17	3	46	1005	51	M	5	4b	yes	vertical	vertical	left	no	0	0	76,79	no
Helsinki	The Longines FEI World Cup Jumping - Helsinki round A	22.10.2017	CSI-W5*	4	1.40 - 1.60	450	104	11	23.03.2007	G	KWPN	95	0	17	3	46	1005	51	M	6	4c	yes	spread	oxer	left	no	0	0	76,79	no
Helsinki	The Longines FEI World Cup Jumping - Helsinki round A	22.10.2017	CSI-W5*	4	1.40 - 1.60	450	104	11	23.03.2007	G	KWPN	95	0	17	3	46	1005	51	M	7	5	no	spread	oxer	right	no	0	0	76,79	no
Helsinki	The Longines FEI World Cup Jumping - Helsinki round A	22.10.2017	CSI-W5*	4	1.40 - 1.60	450	104	11	23.03.2007	G	KWPN	95	0	17	3	46	1005	51	M	8	6a	yes	vertical	vertical	right	no	0	0	76,79	no
Helsinki	The Longines FEI World Cup Jumping - Helsinki round A	22.10.2017	CSI-W5*	4	1.40 - 1.60	450	104	11	23.03.2007	G	KWPN	95	0	17	3	46	1005	51	M	9	6b	yes	vertical	vertical	right	no	0	0	76,79	no
Helsinki	The Longines FEI World Cup Jumping - Helsinki round A	22.10.2017	CSI-W5*	4	1.40 - 1.60	450	104	11	23.03.2007	G	KWPN	95	0	17	3	46	1005	51	M	10	7	no	spread	oxer	left	no	0	0	76,79	no
Helsinki	The Longines FEI World Cup Jumping - Helsinki round A	22.10.2017	CSI-W5*	4	1.40 - 1.60	450	104	11	23.03.2007	G	KWPN	95	0	17	3	46	1005	51	M	11	8	no	vertical	wall	left	no	0	0	76,79	no
Helsinki	The Longines FEI World Cup Jumping - Helsinki round A	22.10.2017	CSI-W5*	4	1.40 - 1.60	450	104	11	23.03.2007	G	KWPN	95	0	17	3	46	1005	51	M	12	9	no	spread	oxer	straight	no	0	0	76,79	no
Helsinki	The Longines FEI World Cup Jumping - Helsinki round A	22.10.2017	CSI-W5*	4	1.40 - 1.60	450	104	11	23.03.2007	G	KWPN	95	0	17	3	46	1005	51	M	13	10	no	spread	triple bar	left	yes	4	0	76,79	no
Helsinki	The Longines FEI World Cup Jumping - Helsinki round A	22.10.2017	CSI-W5*	4	1.40 - 1.60	450	104	11	23.03.2007	G	KWPN	95	0	17	3	46	1005	51	M	14	11	no	vertical	vertical with water	straight	yes	4	0	76,79	no
Helsinki	The Longines FEI World Cup Jumping - Helsinki round A	22.10.2017	CSI-W5*	4	1.40 - 1.60	450	104	11	23.03.2007	G	KWPN	95	0	17	3	46	1005	51	M	15	12a	yes	spread	oxer	right	no	0	0	76,79	no
Helsinki	The Longines FEI World Cup Jumping - Helsinki round A	22.10.2017	CSI-W5*	4	1.40 - 1.60	450	104	11	23.03.2007	G	KWPN	95	0	17	3	46	1005	51	M	16	12b	yes	vertical	vertical	right	no	0	0	76,79	no
Helsinki	The Longines FEI World Cup Jumping - Helsinki round A	22.10.2017	CSI-W5*	4	1.40 - 1.60	450	104	11	23.03.2007	G	KWPN	95	0	17	3	46	1005	51	M	17	13	no	spread	oxer	right	no	0	0	76,79	no
Helsinki	The Longines FEI World Cup Jumping - Helsinki round A	22.10.2017	CSI-W5*	4	1.40 - 1.60	450	154	14	29.04.2004	G	SWB	175	3	18	81	31	1327	0	M	1	1	no	vertical	vertical	right	no	0	0	75,48	no
Helsinki	The Longines FEI World Cup Jumping - Helsinki round A	22.10.2017	CSI-W5*	4	1.40 - 1.60	450	154	14	29.04.2004	G	SWB	175	3	18	81	31	1327	0	M	2	2	no	vertical	vertical	left	no	0	0	75,48	no
Helsinki	The Longines FEI World Cup Jumping - Helsinki round A	22.10.2017	CSI-W5*	4	1.40 - 1.60	450	154	14	29.04.2004	G	SWB	175	3	18	81	31	1327	0	M	3	3	no	spread	oxer	left	no	0	0	75,48	no
Helsinki	The Longines FEI World Cup Jumping - Helsinki round A	22.10.2017	CSI-W5*	4	1.40 - 1.60	450	154	14	29.04.2004	G	SWB	175	3	18	81	31	1327	0	M	4	4a	yes	spread	oxer	left	no	0	0	75,48	no
Helsinki	The Longines FEI World Cup Jumping - Helsinki round A	22.10.2017	CSI-W5*	4	1.40 - 1.60	450	154	14	29.04.2004	G	SWB	175	3	18	81	31	1327	0	M	5	4b	yes	vertical	vertical	left	no	0	0	75,48	no
Helsinki	The Longines FEI World Cup Jumping - Helsinki round A	22.10.2017	CSI-W5*	4	1.40 - 1.60	450	154	14	29.04.2004	G	SWB	175	3	18	81	31	1327	0	M	6	4c	yes	spread	oxer	left	no	0	0	75,48	no
Helsinki	The Longines FEI World Cup Jumping - Helsinki round A	22.10.2017	CSI-W5*	4	1.40 - 1.60	450	154	14	29.04.2004	G	SWB	175	3	18	81	31	1327	0	M	7	5	no	spread	oxer	right	no	0	0	75,48	no
Helsinki	The Longines FEI World Cup Jumping - Helsinki round A	22.10.2017	CSI-W5*	4	1.40 - 1.60	450	154	14	29.04.2004	G	SWB	175	3	18	81	31	1327	0	M	8	6a	yes	vertical	vertical	right	no	0	0	75,48	no
Helsinki	The Longines FEI World Cup Jumping - Helsinki round A	22.10.2017	CSI-W5*	4	1.40 - 1.60	450	154	14	29.04.2004	G	SWB	175	3	18	81	31	1327	0	M	9	6b	yes	vertical	vertical	right	no	0	0	75,48	no
Helsinki	The Longines FEI World Cup Jumping - Helsinki round A	22.10.2017	CSI-W5*	4	1.40 - 1.60	450	154	14	29.04.2004	G	SWB	175	3	18	81	31	1327	0	M	10	7	no	spread	oxer	left	no	0	0	75,48	no
Helsinki	The Longines FEI World Cup Jumping - Helsinki round A	22.10.2017	CSI-W5*	4	1.40 - 1.60	450	154	14	29.04.2004	G	SWB	175	3	18	81	31	1327	0	M	11	8	no	vertical	wall	left	no	0	0	75,48	no
Helsinki	The Longines FEI World Cup Jumping - Helsinki round A	22.10.2017	CSI-W5*	4	1.40 - 1.60	450	154	14	29.04.2004	G	SWB	175	3	18	81	31	1327	0	M	12	9	no	spread	oxer	straight	no	0	0	75,48	no
Helsinki	The Longines FEI World Cup Jumping - Helsinki round A	22.10.2017	CSI-W5*	4	1.40 - 1.60	450	154	14	29.04.2004	G	SWB	175	3	18	81	31	1327	0	M	13	10	no	spread	triple bar	left	no	0	0	75,48	no
Helsinki	The Longines FEI World Cup Jumping - Helsinki round A	22.10.2017	CSI-W5*	4	1.40 - 1.60	450	154	14	29.04.2004	G	SWB	175	3	18	81	31	1327	0	M	14	11	no	vertical	vertical with water	straight	yes	4	0	75,48	no
Helsinki	The Longines FEI World Cup Jumping - Helsinki round A	22.10.2017	CSI-W5*	4	1.40 - 1.60	450	154	14	29.04.2004	G	SWB	175	3	18	81	31	1327	0	M	15	12a	yes	spread	oxer	right	yes	4	0	75,48	no
Helsinki	The Longines FEI World Cup Jumping - Helsinki round A	22.10.2017	CSI-W5*	4	1.40 - 1.60	450	154	14	29.04.2004	G	SWB	175	3	18	81	31	1327	0	M	16	12b	yes	vertical	vertical	right	no	0	0	75,48	no
Helsinki	The Longines FEI World Cup Jumping - Helsinki round A	22.10.2017	CSI-W5*	4	1.40 - 1.60	450	154	14	29.04.2004	G	SWB	175	3	18	81	31	1327	0	M	17	13	no	spread	oxer	right	no	0	0	75,48	no
Helsinki	The Longines FEI World Cup Jumping - Helsinki round A	22.10.2017	CSI-W5*	4	1.40 - 1.60	450	213	11	29.04.2007	M	OLDBG	177	10	19	54	42	2041	67	M	1	1	no	vertical	vertical	right	no	0	0	74	no
Helsinki	The Longines FEI World Cup Jumping - Helsinki round A	22.10.2017	CSI-W5*	4	1.40 - 1.60	450	213	11	29.04.2007	M	OLDBG	177	10	19	54	42	2041	67	M	2	2	no	vertical	vertical	left	no	0	0	74	no
Helsinki	The Longines FEI World Cup Jumping - Helsinki round A	22.10.2017	CSI-W5*	4	1.40 - 1.60	450	213	11	29.04.2007	M	OLDBG	177	10	19	54	42	2041	67	M	3	3	no	spread	oxer	left	no	0	0	74	no
Helsinki	The Longines FEI World Cup Jumping - Helsinki round A	22.10.2017	CSI-W5*	4	1.40 - 1.60	450	213	11	29.04.2007	M	OLDBG	177	10	19	54	42	2041	67	M	4	4a	yes	spread	oxer	left	no	0	0	74	no
Helsinki	The Longines FEI World Cup Jumping - Helsinki round A	22.10.2017	CSI-W5*	4	1.40 - 1.60	450	213	11	29.04.2007	M	OLDBG	177	10	19	54	42	2041	67	M	5	4b	yes	vertical	vertical	left	no	0	0	74	no
Helsinki	The Longines FEI World Cup Jumping - Helsinki round A	22.10.2017	CSI-W5*	4	1.40 - 1.60	450	213	11	29.04.2007	M	OLDBG	177	10	19	54	42	2041	67	M	6	4c	yes	spread	oxer	left	no	0	0	74	no
Helsinki	The Longines FEI World Cup Jumping - Helsinki round A	22.10.2017	CSI-W5*	4	1.40 - 1.60	450	213	11	29.04.2007	M	OLDBG	177	10	19	54	42	2041	67	M	7	5	no	spread	oxer	right	yes	4	0	74	no
Helsinki	The Longines FEI World Cup Jumping - Helsinki round A	22.10.2017	CSI-W5*	4	1.40 - 1.60	450	213	11	29.04.2007	M	OLDBG	177	10	19	54	42	2041	67	M	8	6a	yes	vertical	vertical	right	no	0	0	74	no
Helsinki	The Longines FEI World Cup Jumping - Helsinki round A	22.10.2017	CSI-W5*	4	1.40 - 1.60	450	213	11	29.04.2007	M	OLDBG	177	10	19	54	42	2041	67	M	9	6b	yes	vertical	vertical	right	yes	4	0	74	no
Helsinki	The Longines FEI World Cup Jumping - Helsinki round A	22.10.2017	CSI-W5*	4	1.40 - 1.60	450	213	11	29.04.2007	M	OLDBG	177	10	19	54	42	2041	67	M	10	7	no	spread	oxer	left	no	0	0	74	no
Helsinki	The Longines FEI World Cup Jumping - Helsinki round A	22.10.2017	CSI-W5*	4	1.40 - 1.60	450	213	11	29.04.2007	M	OLDBG	177	10	19	54	42	2041	67	M	11	8	no	vertical	wall	left	no	0	0	74	no
Helsinki	The Longines FEI World Cup Jumping - Helsinki round A	22.10.2017	CSI-W5*	4	1.40 - 1.60	450	213	11	29.04.2007	M	OLDBG	177	10	19	54	42	2041	67	M	12	9	no	spread	oxer	straight	no	0	0	74	no
Helsinki	The Longines FEI World Cup Jumping - Helsinki round A	22.10.2017	CSI-W5*	4	1.40 - 1.60	450	213	11	29.04.2007	M	OLDBG	177	10	19	54	42	2041	67	M	13	10	no	spread	triple bar	left	no	0	0	74	no
Helsinki	The Longines FEI World Cup Jumping - Helsinki round A	22.10.2017	CSI-W5*	4	1.40 - 1.60	450	213	11	29.04.2007	M	OLDBG	177	10	19	54	42	2041	67	M	14	11	no	vertical	vertical with water	straight	no	0	0	74	no
Helsinki	The Longines FEI World Cup Jumping - Helsinki round A	22.10.2017	CSI-W5*	4	1.40 - 1.60	450	213	11	29.04.2007	M	OLDBG	177	10	19	54	42	2041	67	M	15	12a	yes	spread	oxer	right	no	0	0	74	no
Helsinki	The Longines FEI World Cup Jumping - Helsinki round A	22.10.2017	CSI-W5*	4	1.40 - 1.60	450	213	11	29.04.2007	M	OLDBG	177	10	19	54	42	2041	67	M	16	12b	yes	vertical	vertical	right	no	0	0	74	no
Helsinki	The Longines FEI World Cup Jumping - Helsinki round A	22.10.2017	CSI-W5*	4	1.40 - 1.60	450	213	11	29.04.2007	M	OLDBG	177	10	19	54	42	2041	67	M	17	13	no	spread	oxer	right	no	0	0	74	no
Helsinki	The Longines FEI World Cup Jumping - Helsinki round A	22.10.2017	CSI-W5*	4	1.40 - 1.60	450	183	12	10.06.2006	M	AA	243	8	20	73	38	2030	91	M	1	1	no	vertical	vertical	right	no	0	0	0,00	yes
Helsinki	The Longines FEI World Cup Jumping - Helsinki round A	22.10.2017	CSI-W5*	4	1.40 - 1.60	450	183	12	10.06.2006	M	AA	243	8	20	73	38	2030	91	M	2	2	no	vertical	vertical	left	no	0	0	0,00	yes
Helsinki	The Longines FEI World Cup Jumping - Helsinki round A	22.10.2017	CSI-W5*	4	1.40 - 1.60	450	183	12	10.06.2006	M	AA	243	8	20	73	38	2030	91	M	3	3	no	spread	oxer	left	no	0	0	0,00	yes
Helsinki	The Longines FEI World Cup Jumping - Helsinki round A	22.10.2017	CSI-W5*	4	1.40 - 1.60	450	183	12	10.06.2006	M	AA	243	8	20	73	38	2030	91	M	4	4a	yes	spread	oxer	left	no	0	0	0,00	yes
Helsinki	The Longines FEI World Cup Jumping - Helsinki round A	22.10.2017	CSI-W5*	4	1.40 - 1.60	450	183	12	10.06.2006	M	AA	243	8	20	73	38	2030	91	M	5	4b	yes	vertical	vertical	left	yes	4	0	0,00	yes
Helsinki	The Longines FEI World Cup Jumping - Helsinki round A	22.10.2017	CSI-W5*	4	1.40 - 1.60	450	183	12	10.06.2006	M	AA	243	8	20	73	38	2030	91	M	6	4c	yes	spread	oxer	left	no	0	0	0,00	yes
Helsinki	The Longines FEI World Cup Jumping - Helsinki round A	22.10.2017	CSI-W5*	4	1.40 - 1.60	450	183	12	10.06.2006	M	AA	243	8	20	73	38	2030	91	M	7	5	no	spread	oxer	right	no	0	0	0,00	yes
Helsinki	The Longines FEI World Cup Jumping - Helsinki round A	22.10.2017	CSI-W5*	4	1.40 - 1.60	450	183	12	10.06.2006	M	AA	243	8	20	73	38	2030	91	M	8	6a	yes	vertical	vertical	right	no	0	0	0,00	yes
Helsinki	The Longines FEI World Cup Jumping - Helsinki round A	22.10.2017	CSI-W5*	4	1.40 - 1.60	450	183	12	10.06.2006	M	AA	243	8	20	73	38	2030	91	M	9	6b	yes	vertical	vertical	right	yes	4	0	0,00	yes
Helsinki	The Longines FEI World Cup Jumping - Helsinki round A	22.10.2017	CSI-W5*	4	1.40 - 1.60	450	183	12	10.06.2006	M	AA	243	8	20	73	38	2030	91	M	10	7	no	spread	oxer	left	no	0	0	0,00	yes
Helsinki	The Longines FEI World Cup Jumping - Helsinki round A	22.10.2017	CSI-W5*	4	1.40 - 1.60	450	183	12	10.06.2006	M	AA	243	8	20	73	38	2030	91	M	11	8	no	vertical	wall	left	no	0	0	0,00	yes
Helsinki	The Longines FEI World Cup Jumping - Helsinki round A	22.10.2017	CSI-W5*	4	1.40 - 1.60	450	183	12	10.06.2006	M	AA	243	8	20	73	38	2030	91	M	12	9	no	spread	oxer	straight	no	0	0	0,00	yes
Helsinki	The Longines FEI World Cup Jumping - Helsinki round A	22.10.2017	CSI-W5*	4	1.40 - 1.60	450	183	12	10.06.2006	M	AA	243	8	20	73	38	2030	91	M	13	10	no	spread	triple bar	left	no	0	0	0,00	yes
Helsinki	The Longines FEI World Cup Jumping - Helsinki round A	22.10.2017	CSI-W5*	4	1.40 - 1.60	450	183	12	10.06.2006	M	AA	243	8	20	73	38	2030	91	M	14	11	no	vertical	vertical with water	straight	no	0	0	0,00	yes
Helsinki	The Longines FEI World Cup Jumping - Helsinki round A	22.10.2017	CSI-W5*	4	1.40 - 1.60	450	183	12	10.06.2006	M	AA	243	8	20	73	38	2030	91	M	15	12a	yes	spread	oxer	right	no	0	0	0,00	yes
Helsinki	The Longines FEI World Cup Jumping - Helsinki round A	22.10.2017	CSI-W5*	4	1.40 - 1.60	450	183	12	10.06.2006	M	AA	243	8	20	73	38	2030	91	M	16	12b	yes	vertical	vertical	right	no	0	0	0,00	yes
Helsinki	The Longines FEI World Cup Jumping - Helsinki round A	22.10.2017	CSI-W5*	4	1.40 - 1.60	450	183	12	10.06.2006	M	AA	243	8	20	73	38	2030	91	M	17	13	no	spread	oxer	right	no	0	0	0,00	yes
Helsinki	The Longines FEI World Cup Jumping - Helsinki round A	22.10.2017	CSI-W5*	4	1.40 - 1.60	450	218	12	24.05.2006	M	HOLST	115	0	22	144	55	798	31	M	1	1	no	vertical	vertical	right	no	0	0	73,47	no
Helsinki	The Longines FEI World Cup Jumping - Helsinki round A	22.10.2017	CSI-W5*	4	1.40 - 1.60	450	218	12	24.05.2006	M	HOLST	115	0	22	144	55	798	31	M	2	2	no	vertical	vertical	left	no	0	0	73,47	no
Helsinki	The Longines FEI World Cup Jumping - Helsinki round A	22.10.2017	CSI-W5*	4	1.40 - 1.60	450	218	12	24.05.2006	M	HOLST	115	0	22	144	55	798	31	M	3	3	no	spread	oxer	left	no	0	0	73,47	no
Helsinki	The Longines FEI World Cup Jumping - Helsinki round A	22.10.2017	CSI-W5*	4	1.40 - 1.60	450	218	12	24.05.2006	M	HOLST	115	0	22	144	55	798	31	M	4	4a	yes	spread	oxer	left	no	0	0	73,47	no
Helsinki	The Longines FEI World Cup Jumping - Helsinki round A	22.10.2017	CSI-W5*	4	1.40 - 1.60	450	218	12	24.05.2006	M	HOLST	115	0	22	144	55	798	31	M	5	4b	yes	vertical	vertical	left	no	0	0	73,47	no
Helsinki	The Longines FEI World Cup Jumping - Helsinki round A	22.10.2017	CSI-W5*	4	1.40 - 1.60	450	218	12	24.05.2006	M	HOLST	115	0	22	144	55	798	31	M	6	4c	yes	spread	oxer	left	no	0	0	73,47	no
Helsinki	The Longines FEI World Cup Jumping - Helsinki round A	22.10.2017	CSI-W5*	4	1.40 - 1.60	450	218	12	24.05.2006	M	HOLST	115	0	22	144	55	798	31	M	7	5	no	spread	oxer	right	no	0	0	73,47	no
Helsinki	The Longines FEI World Cup Jumping - Helsinki round A	22.10.2017	CSI-W5*	4	1.40 - 1.60	450	218	12	24.05.2006	M	HOLST	115	0	22	144	55	798	31	M	8	6a	yes	vertical	vertical	right	no	0	0	73,47	no
Helsinki	The Longines FEI World Cup Jumping - Helsinki round A	22.10.2017	CSI-W5*	4	1.40 - 1.60	450	218	12	24.05.2006	M	HOLST	115	0	22	144	55	798	31	M	9	6b	yes	vertical	vertical	right	no	0	0	73,47	no
Helsinki	The Longines FEI World Cup Jumping - Helsinki round A	22.10.2017	CSI-W5*	4	1.40 - 1.60	450	218	12	24.05.2006	M	HOLST	115	0	22	144	55	798	31	M	10	7	no	spread	oxer	left	no	0	0	73,47	no
Helsinki	The Longines FEI World Cup Jumping - Helsinki round A	22.10.2017	CSI-W5*	4	1.40 - 1.60	450	218	12	24.05.2006	M	HOLST	115	0	22	144	55	798	31	M	11	8	no	vertical	wall	left	no	0	0	73,47	no
Helsinki	The Longines FEI World Cup Jumping - Helsinki round A	22.10.2017	CSI-W5*	4	1.40 - 1.60	450	218	12	24.05.2006	M	HOLST	115	0	22	144	55	798	31	M	12	9	no	spread	oxer	straight	no	0	0	73,47	no
Helsinki	The Longines FEI World Cup Jumping - Helsinki round A	22.10.2017	CSI-W5*	4	1.40 - 1.60	450	218	12	24.05.2006	M	HOLST	115	0	22	144	55	798	31	M	13	10	no	spread	triple bar	left	yes	4	0	73,47	no
Helsinki	The Longines FEI World Cup Jumping - Helsinki round A	22.10.2017	CSI-W5*	4	1.40 - 1.60	450	218	12	24.05.2006	M	HOLST	115	0	22	144	55	798	31	M	14	11	no	vertical	vertical with water	straight	yes	4	0	73,47	no
Helsinki	The Longines FEI World Cup Jumping - Helsinki round A	22.10.2017	CSI-W5*	4	1.40 - 1.60	450	218	12	24.05.2006	M	HOLST	115	0	22	144	55	798	31	M	15	12a	yes	spread	oxer	right	yes	4	0	73,47	no
Helsinki	The Longines FEI World Cup Jumping - Helsinki round A	22.10.2017	CSI-W5*	4	1.40 - 1.60	450	218	12	24.05.2006	M	HOLST	115	0	22	144	55	798	31	M	16	12b	yes	vertical	vertical	right	no	0	0	73,47	no
Helsinki	The Longines FEI World Cup Jumping - Helsinki round A	22.10.2017	CSI-W5*	4	1.40 - 1.60	450	218	12	24.05.2006	M	HOLST	115	0	22	144	55	798	31	M	17	13	no	spread	oxer	right	no	0	0	73,47	no
Helsinki	The Longines FEI World Cup Jumping - Helsinki round A	22.10.2017	CSI-W5*	4	1.40 - 1.60	450	162	12	01.01.2006	M	MIPAAF	146	3	24	287	49	641	15	M	1	1	no	vertical	vertical	right	no	0	0	73,68	no
Helsinki	The Longines FEI World Cup Jumping - Helsinki round A	22.10.2017	CSI-W5*	4	1.40 - 1.60	450	162	12	01.01.2006	M	MIPAAF	146	3	24	287	49	641	15	M	2	2	no	vertical	vertical	left	no	0	0	73,68	no
Helsinki	The Longines FEI World Cup Jumping - Helsinki round A	22.10.2017	CSI-W5*	4	1.40 - 1.60	450	162	12	01.01.2006	M	MIPAAF	146	3	24	287	49	641	15	M	3	3	no	spread	oxer	left	no	0	0	73,68	no
Helsinki	The Longines FEI World Cup Jumping - Helsinki round A	22.10.2017	CSI-W5*	4	1.40 - 1.60	450	162	12	01.01.2006	M	MIPAAF	146	3	24	287	49	641	15	M	4	4a	yes	spread	oxer	left	yes	4	0	73,68	no
Helsinki	The Longines FEI World Cup Jumping - Helsinki round A	22.10.2017	CSI-W5*	4	1.40 - 1.60	450	162	12	01.01.2006	M	MIPAAF	146	3	24	287	49	641	15	M	5	4b	yes	vertical	vertical	left	no	0	0	73,68	no
Helsinki	The Longines FEI World Cup Jumping - Helsinki round A	22.10.2017	CSI-W5*	4	1.40 - 1.60	450	162	12	01.01.2006	M	MIPAAF	146	3	24	287	49	641	15	M	6	4c	yes	spread	oxer	left	no	0	0	73,68	no
Helsinki	The Longines FEI World Cup Jumping - Helsinki round A	22.10.2017	CSI-W5*	4	1.40 - 1.60	450	162	12	01.01.2006	M	MIPAAF	146	3	24	287	49	641	15	M	7	5	no	spread	oxer	right	no	0	0	73,68	no
Helsinki	The Longines FEI World Cup Jumping - Helsinki round A	22.10.2017	CSI-W5*	4	1.40 - 1.60	450	162	12	01.01.2006	M	MIPAAF	146	3	24	287	49	641	15	M	8	6a	yes	vertical	vertical	right	no	0	0	73,68	no
Helsinki	The Longines FEI World Cup Jumping - Helsinki round A	22.10.2017	CSI-W5*	4	1.40 - 1.60	450	162	12	01.01.2006	M	MIPAAF	146	3	24	287	49	641	15	M	9	6b	yes	vertical	vertical	right	no	0	0	73,68	no
Helsinki	The Longines FEI World Cup Jumping - Helsinki round A	22.10.2017	CSI-W5*	4	1.40 - 1.60	450	162	12	01.01.2006	M	MIPAAF	146	3	24	287	49	641	15	M	10	7	no	spread	oxer	left	yes	4	0	73,68	no
Helsinki	The Longines FEI World Cup Jumping - Helsinki round A	22.10.2017	CSI-W5*	4	1.40 - 1.60	450	162	12	01.01.2006	M	MIPAAF	146	3	24	287	49	641	15	M	11	8	no	vertical	wall	left	no	0	0	73,68	no
Helsinki	The Longines FEI World Cup Jumping - Helsinki round A	22.10.2017	CSI-W5*	4	1.40 - 1.60	450	162	12	01.01.2006	M	MIPAAF	146	3	24	287	49	641	15	M	12	9	no	spread	oxer	straight	no	0	0	73,68	no
Helsinki	The Longines FEI World Cup Jumping - Helsinki round A	22.10.2017	CSI-W5*	4	1.40 - 1.60	450	162	12	01.01.2006	M	MIPAAF	146	3	24	287	49	641	15	M	13	10	no	spread	triple bar	left	no	0	0	73,68	no
Helsinki	The Longines FEI World Cup Jumping - Helsinki round A	22.10.2017	CSI-W5*	4	1.40 - 1.60	450	162	12	01.01.2006	M	MIPAAF	146	3	24	287	49	641	15	M	14	11	no	vertical	vertical with water	straight	no	0	0	73,68	no
Helsinki	The Longines FEI World Cup Jumping - Helsinki round A	22.10.2017	CSI-W5*	4	1.40 - 1.60	450	162	12	01.01.2006	M	MIPAAF	146	3	24	287	49	641	15	M	15	12a	yes	spread	oxer	right	no	0	0	73,68	no
Helsinki	The Longines FEI World Cup Jumping - Helsinki round A	22.10.2017	CSI-W5*	4	1.40 - 1.60	450	162	12	01.01.2006	M	MIPAAF	146	3	24	287	49	641	15	M	16	12b	yes	vertical	vertical	right	yes	4	0	73,68	no
Helsinki	The Longines FEI World Cup Jumping - Helsinki round A	22.10.2017	CSI-W5*	4	1.40 - 1.60	450	162	12	01.01.2006	M	MIPAAF	146	3	24	287	49	641	15	M	17	13	no	spread	oxer	right	no	0	0	73,68	no
Helsinki	The Longines FEI World Cup Jumping - Helsinki round A	22.10.2017	CSI-W5*	4	1.40 - 1.60	450	127	10	03.07.2008	M	BWP	131	3	26	14	30	1957	97	M	1	1	no	vertical	vertical	right	no	0	0	76,08	no
Helsinki	The Longines FEI World Cup Jumping - Helsinki round A	22.10.2017	CSI-W5*	4	1.40 - 1.60	450	127	10	03.07.2008	M	BWP	131	3	26	14	30	1957	97	M	2	2	no	vertical	vertical	left	no	0	0	76,08	no
Helsinki	The Longines FEI World Cup Jumping - Helsinki round A	22.10.2017	CSI-W5*	4	1.40 - 1.60	450	127	10	03.07.2008	M	BWP	131	3	26	14	30	1957	97	M	3	3	no	spread	oxer	left	no	0	0	76,08	no
Helsinki	The Longines FEI World Cup Jumping - Helsinki round A	22.10.2017	CSI-W5*	4	1.40 - 1.60	450	127	10	03.07.2008	M	BWP	131	3	26	14	30	1957	97	M	4	4a	yes	spread	oxer	left	no	0	0	76,08	no
Helsinki	The Longines FEI World Cup Jumping - Helsinki round A	22.10.2017	CSI-W5*	4	1.40 - 1.60	450	127	10	03.07.2008	M	BWP	131	3	26	14	30	1957	97	M	5	4b	yes	vertical	vertical	left	no	0	0	76,08	no
Helsinki	The Longines FEI World Cup Jumping - Helsinki round A	22.10.2017	CSI-W5*	4	1.40 - 1.60	450	127	10	03.07.2008	M	BWP	131	3	26	14	30	1957	97	M	6	4c	yes	spread	oxer	left	no	0	0	76,08	no
Helsinki	The Longines FEI World Cup Jumping - Helsinki round A	22.10.2017	CSI-W5*	4	1.40 - 1.60	450	127	10	03.07.2008	M	BWP	131	3	26	14	30	1957	97	M	7	5	no	spread	oxer	right	no	0	0	76,08	no
Helsinki	The Longines FEI World Cup Jumping - Helsinki round A	22.10.2017	CSI-W5*	4	1.40 - 1.60	450	127	10	03.07.2008	M	BWP	131	3	26	14	30	1957	97	M	8	6a	yes	vertical	vertical	right	no	0	0	76,08	no
Helsinki	The Longines FEI World Cup Jumping - Helsinki round A	22.10.2017	CSI-W5*	4	1.40 - 1.60	450	127	10	03.07.2008	M	BWP	131	3	26	14	30	1957	97	M	9	6b	yes	vertical	vertical	right	no	0	0	76,08	no
Helsinki	The Longines FEI World Cup Jumping - Helsinki round A	22.10.2017	CSI-W5*	4	1.40 - 1.60	450	127	10	03.07.2008	M	BWP	131	3	26	14	30	1957	97	M	10	7	no	spread	oxer	left	no	0	0	76,08	no
Helsinki	The Longines FEI World Cup Jumping - Helsinki round A	22.10.2017	CSI-W5*	4	1.40 - 1.60	450	127	10	03.07.2008	M	BWP	131	3	26	14	30	1957	97	M	11	8	no	vertical	wall	left	no	0	0	76,08	no
Helsinki	The Longines FEI World Cup Jumping - Helsinki round A	22.10.2017	CSI-W5*	4	1.40 - 1.60	450	127	10	03.07.2008	M	BWP	131	3	26	14	30	1957	97	M	12	9	no	spread	oxer	straight	no	0	0	76,08	no
Helsinki	The Longines FEI World Cup Jumping - Helsinki round A	22.10.2017	CSI-W5*	4	1.40 - 1.60	450	127	10	03.07.2008	M	BWP	131	3	26	14	30	1957	97	M	13	10	no	spread	triple bar	left	no	0	0	76,08	no
Helsinki	The Longines FEI World Cup Jumping - Helsinki round A	22.10.2017	CSI-W5*	4	1.40 - 1.60	450	127	10	03.07.2008	M	BWP	131	3	26	14	30	1957	97	M	14	11	no	vertical	vertical with water	straight	no	0	0	76,08	no
Helsinki	The Longines FEI World Cup Jumping - Helsinki round A	22.10.2017	CSI-W5*	4	1.40 - 1.60	450	127	10	03.07.2008	M	BWP	131	3	26	14	30	1957	97	M	15	12a	yes	spread	oxer	right	no	0	0	76,08	no
Helsinki	The Longines FEI World Cup Jumping - Helsinki round A	22.10.2017	CSI-W5*	4	1.40 - 1.60	450	127	10	03.07.2008	M	BWP	131	3	26	14	30	1957	97	M	16	12b	yes	vertical	vertical	right	yes	4	0	76,08	no
Helsinki	The Longines FEI World Cup Jumping - Helsinki round A	22.10.2017	CSI-W5*	4	1.40 - 1.60	450	127	10	03.07.2008	M	BWP	131	3	26	14	30	1957	97	M	17	13	no	spread	oxer	right	yes	4	0	76,08	no
Helsinki	The Longines FEI World Cup Jumping - Helsinki round A	22.10.2017	CSI-W5*	4	1.40 - 1.60	450	92	13	20.03.2005	G	BWP	140	4	28	4	38	2170	90	M	1	1	no	vertical	vertical	right	no	0	0	68,84	no
Helsinki	The Longines FEI World Cup Jumping - Helsinki round A	22.10.2017	CSI-W5*	4	1.40 - 1.60	450	92	13	20.03.2005	G	BWP	140	4	28	4	38	2170	90	M	2	2	no	vertical	vertical	left	no	0	0	68,84	no
Helsinki	The Longines FEI World Cup Jumping - Helsinki round A	22.10.2017	CSI-W5*	4	1.40 - 1.60	450	92	13	20.03.2005	G	BWP	140	4	28	4	38	2170	90	M	3	3	no	spread	oxer	left	yes	4	0	68,84	no
Helsinki	The Longines FEI World Cup Jumping - Helsinki round A	22.10.2017	CSI-W5*	4	1.40 - 1.60	450	92	13	20.03.2005	G	BWP	140	4	28	4	38	2170	90	M	4	4a	yes	spread	oxer	left	no	0	0	68,84	no
Helsinki	The Longines FEI World Cup Jumping - Helsinki round A	22.10.2017	CSI-W5*	4	1.40 - 1.60	450	92	13	20.03.2005	G	BWP	140	4	28	4	38	2170	90	M	5	4b	yes	vertical	vertical	left	no	0	0	68,84	no
Helsinki	The Longines FEI World Cup Jumping - Helsinki round A	22.10.2017	CSI-W5*	4	1.40 - 1.60	450	92	13	20.03.2005	G	BWP	140	4	28	4	38	2170	90	M	6	4c	yes	spread	oxer	left	no	0	0	68,84	no
Helsinki	The Longines FEI World Cup Jumping - Helsinki round A	22.10.2017	CSI-W5*	4	1.40 - 1.60	450	92	13	20.03.2005	G	BWP	140	4	28	4	38	2170	90	M	7	5	no	spread	oxer	right	no	0	0	68,84	no
Helsinki	The Longines FEI World Cup Jumping - Helsinki round A	22.10.2017	CSI-W5*	4	1.40 - 1.60	450	92	13	20.03.2005	G	BWP	140	4	28	4	38	2170	90	M	8	6a	yes	vertical	vertical	right	no	0	0	68,84	no
Helsinki	The Longines FEI World Cup Jumping - Helsinki round A	22.10.2017	CSI-W5*	4	1.40 - 1.60	450	92	13	20.03.2005	G	BWP	140	4	28	4	38	2170	90	M	9	6b	yes	vertical	vertical	right	no	0	0	68,84	no
Helsinki	The Longines FEI World Cup Jumping - Helsinki round A	22.10.2017	CSI-W5*	4	1.40 - 1.60	450	92	13	20.03.2005	G	BWP	140	4	28	4	38	2170	90	M	10	7	no	spread	oxer	left	no	0	0	68,84	no
Helsinki	The Longines FEI World Cup Jumping - Helsinki round A	22.10.2017	CSI-W5*	4	1.40 - 1.60	450	92	13	20.03.2005	G	BWP	140	4	28	4	38	2170	90	M	11	8	no	vertical	wall	left	no	0	0	68,84	no
Helsinki	The Longines FEI World Cup Jumping - Helsinki round A	22.10.2017	CSI-W5*	4	1.40 - 1.60	450	92	13	20.03.2005	G	BWP	140	4	28	4	38	2170	90	M	12	9	no	spread	oxer	straight	no	0	0	68,84	no
Helsinki	The Longines FEI World Cup Jumping - Helsinki round A	22.10.2017	CSI-W5*	4	1.40 - 1.60	450	92	13	20.03.2005	G	BWP	140	4	28	4	38	2170	90	M	13	10	no	spread	triple bar	left	no	0	0	68,84	no
Helsinki	The Longines FEI World Cup Jumping - Helsinki round A	22.10.2017	CSI-W5*	4	1.40 - 1.60	450	92	13	20.03.2005	G	BWP	140	4	28	4	38	2170	90	M	14	11	no	vertical	vertical with water	straight	no	0	0	68,84	no
Helsinki	The Longines FEI World Cup Jumping - Helsinki round A	22.10.2017	CSI-W5*	4	1.40 - 1.60	450	92	13	20.03.2005	G	BWP	140	4	28	4	38	2170	90	M	15	12a	yes	spread	oxer	right	no	0	0	68,84	no
Helsinki	The Longines FEI World Cup Jumping - Helsinki round A	22.10.2017	CSI-W5*	4	1.40 - 1.60	450	92	13	20.03.2005	G	BWP	140	4	28	4	38	2170	90	M	16	12b	yes	vertical	vertical	right	no	0	0	68,84	no
Helsinki	The Longines FEI World Cup Jumping - Helsinki round A	22.10.2017	CSI-W5*	4	1.40 - 1.60	450	92	13	20.03.2005	G	BWP	140	4	28	4	38	2170	90	M	17	13	no	spread	oxer	right	no	0	0	68,84	no
Helsinki	The Longines FEI World Cup Jumping - Helsinki round A	22.10.2017	CSI-W5*	4	1.40 - 1.60	450	35	15	22.05.2003	G	HOLST	368	19	29	89	35	1502	55	M	1	1	no	vertical	vertical	right	no	0	0	73,54	no
Helsinki	The Longines FEI World Cup Jumping - Helsinki round A	22.10.2017	CSI-W5*	4	1.40 - 1.60	450	35	15	22.05.2003	G	HOLST	368	19	29	89	35	1502	55	M	2	2	no	vertical	vertical	left	no	0	0	73,54	no
Helsinki	The Longines FEI World Cup Jumping - Helsinki round A	22.10.2017	CSI-W5*	4	1.40 - 1.60	450	35	15	22.05.2003	G	HOLST	368	19	29	89	35	1502	55	M	3	3	no	spread	oxer	left	no	0	0	73,54	no
Helsinki	The Longines FEI World Cup Jumping - Helsinki round A	22.10.2017	CSI-W5*	4	1.40 - 1.60	450	35	15	22.05.2003	G	HOLST	368	19	29	89	35	1502	55	M	4	4a	yes	spread	oxer	left	no	0	0	73,54	no
Helsinki	The Longines FEI World Cup Jumping - Helsinki round A	22.10.2017	CSI-W5*	4	1.40 - 1.60	450	35	15	22.05.2003	G	HOLST	368	19	29	89	35	1502	55	M	5	4b	yes	vertical	vertical	left	no	0	0	73,54	no
Helsinki	The Longines FEI World Cup Jumping - Helsinki round A	22.10.2017	CSI-W5*	4	1.40 - 1.60	450	35	15	22.05.2003	G	HOLST	368	19	29	89	35	1502	55	M	6	4c	yes	spread	oxer	left	no	0	0	73,54	no
Helsinki	The Longines FEI World Cup Jumping - Helsinki round A	22.10.2017	CSI-W5*	4	1.40 - 1.60	450	35	15	22.05.2003	G	HOLST	368	19	29	89	35	1502	55	M	7	5	no	spread	oxer	right	no	0	0	73,54	no
Helsinki	The Longines FEI World Cup Jumping - Helsinki round A	22.10.2017	CSI-W5*	4	1.40 - 1.60	450	35	15	22.05.2003	G	HOLST	368	19	29	89	35	1502	55	M	8	6a	yes	vertical	vertical	right	no	0	0	73,54	no
Helsinki	The Longines FEI World Cup Jumping - Helsinki round A	22.10.2017	CSI-W5*	4	1.40 - 1.60	450	35	15	22.05.2003	G	HOLST	368	19	29	89	35	1502	55	M	9	6b	yes	vertical	vertical	right	no	0	0	73,54	no
Helsinki	The Longines FEI World Cup Jumping - Helsinki round A	22.10.2017	CSI-W5*	4	1.40 - 1.60	450	35	15	22.05.2003	G	HOLST	368	19	29	89	35	1502	55	M	10	7	no	spread	oxer	left	no	0	0	73,54	no
Helsinki	The Longines FEI World Cup Jumping - Helsinki round A	22.10.2017	CSI-W5*	4	1.40 - 1.60	450	35	15	22.05.2003	G	HOLST	368	19	29	89	35	1502	55	M	11	8	no	vertical	wall	left	no	0	0	73,54	no
Helsinki	The Longines FEI World Cup Jumping - Helsinki round A	22.10.2017	CSI-W5*	4	1.40 - 1.60	450	35	15	22.05.2003	G	HOLST	368	19	29	89	35	1502	55	M	12	9	no	spread	oxer	straight	no	0	0	73,54	no
Helsinki	The Longines FEI World Cup Jumping - Helsinki round A	22.10.2017	CSI-W5*	4	1.40 - 1.60	450	35	15	22.05.2003	G	HOLST	368	19	29	89	35	1502	55	M	13	10	no	spread	triple bar	left	no	0	0	73,54	no
Helsinki	The Longines FEI World Cup Jumping - Helsinki round A	22.10.2017	CSI-W5*	4	1.40 - 1.60	450	35	15	22.05.2003	G	HOLST	368	19	29	89	35	1502	55	M	14	11	no	vertical	vertical with water	straight	no	0	0	73,54	no
Helsinki	The Longines FEI World Cup Jumping - Helsinki round A	22.10.2017	CSI-W5*	4	1.40 - 1.60	450	35	15	22.05.2003	G	HOLST	368	19	29	89	35	1502	55	M	15	12a	yes	spread	oxer	right	no	0	0	73,54	no
Helsinki	The Longines FEI World Cup Jumping - Helsinki round A	22.10.2017	CSI-W5*	4	1.40 - 1.60	450	35	15	22.05.2003	G	HOLST	368	19	29	89	35	1502	55	M	16	12b	yes	vertical	vertical	right	no	0	0	73,54	no
Helsinki	The Longines FEI World Cup Jumping - Helsinki round A	22.10.2017	CSI-W5*	4	1.40 - 1.60	450	35	15	22.05.2003	G	HOLST	368	19	29	89	35	1502	55	M	17	13	no	spread	oxer	right	no	0	0	73,54	no
Helsinki	The Longines FEI World Cup Jumping - Helsinki round B	22.10.2017	CSI-W5*	4	1.40 - 1.60	270	35	15	22.05.2003	G	HOLST	368	19	29	89	35	1502	55	M	1	1	no	spread	oxer	right	no	0	0	39,27	no
Helsinki	The Longines FEI World Cup Jumping - Helsinki round B	22.10.2017	CSI-W5*	4	1.40 - 1.60	270	35	15	22.05.2003	G	HOLST	368	19	29	89	35	1502	55	M	2	2	no	vertical	vertical	right	no	0	0	39,27	no
Helsinki	The Longines FEI World Cup Jumping - Helsinki round B	22.10.2017	CSI-W5*	4	1.40 - 1.60	270	35	15	22.05.2003	G	HOLST	368	19	29	89	35	1502	55	M	3	3	no	spread	oxer	left	no	0	0	39,27	no
Helsinki	The Longines FEI World Cup Jumping - Helsinki round B	22.10.2017	CSI-W5*	4	1.40 - 1.60	270	35	15	22.05.2003	G	HOLST	368	19	29	89	35	1502	55	M	4	4a	yes	vertical	vertical	left	no	0	0	39,27	no
Helsinki	The Longines FEI World Cup Jumping - Helsinki round B	22.10.2017	CSI-W5*	4	1.40 - 1.60	270	35	15	22.05.2003	G	HOLST	368	19	29	89	35	1502	55	M	5	4b	yes	spread	oxer	left	no	0	0	39,27	no
Helsinki	The Longines FEI World Cup Jumping - Helsinki round B	22.10.2017	CSI-W5*	4	1.40 - 1.60	270	35	15	22.05.2003	G	HOLST	368	19	29	89	35	1502	55	M	6	5	no	spread	oxer	right	no	0	0	39,27	no
Helsinki	The Longines FEI World Cup Jumping - Helsinki round B	22.10.2017	CSI-W5*	4	1.40 - 1.60	270	35	15	22.05.2003	G	HOLST	368	19	29	89	35	1502	55	M	7	6	no	vertical	vertical	right	no	0	0	39,27	no
Helsinki	The Longines FEI World Cup Jumping - Helsinki round B	22.10.2017	CSI-W5*	4	1.40 - 1.60	270	35	15	22.05.2003	G	HOLST	368	19	29	89	35	1502	55	M	8	7	no	spread	oxer	left	no	0	0	39,27	no
Helsinki	The Longines FEI World Cup Jumping - Helsinki round A	22.10.2017	CSI-W5*	4	1.40 - 1.60	450	108	11	24.03.2007	M	BWP	91	5	30	9	36	2556	124	M	1	1	no	vertical	vertical	right	no	0	0	72,71	no
Helsinki	The Longines FEI World Cup Jumping - Helsinki round A	22.10.2017	CSI-W5*	4	1.40 - 1.60	450	108	11	24.03.2007	M	BWP	91	5	30	9	36	2556	124	M	2	2	no	vertical	vertical	left	no	0	0	72,71	no
Helsinki	The Longines FEI World Cup Jumping - Helsinki round A	22.10.2017	CSI-W5*	4	1.40 - 1.60	450	108	11	24.03.2007	M	BWP	91	5	30	9	36	2556	124	M	3	3	no	spread	oxer	left	no	0	0	72,71	no
Helsinki	The Longines FEI World Cup Jumping - Helsinki round A	22.10.2017	CSI-W5*	4	1.40 - 1.60	450	108	11	24.03.2007	M	BWP	91	5	30	9	36	2556	124	M	4	4a	yes	spread	oxer	left	no	0	0	72,71	no
Helsinki	The Longines FEI World Cup Jumping - Helsinki round A	22.10.2017	CSI-W5*	4	1.40 - 1.60	450	108	11	24.03.2007	M	BWP	91	5	30	9	36	2556	124	M	5	4b	yes	vertical	vertical	left	no	0	0	72,71	no
Helsinki	The Longines FEI World Cup Jumping - Helsinki round A	22.10.2017	CSI-W5*	4	1.40 - 1.60	450	108	11	24.03.2007	M	BWP	91	5	30	9	36	2556	124	M	6	4c	yes	spread	oxer	left	no	0	0	72,71	no
Helsinki	The Longines FEI World Cup Jumping - Helsinki round A	22.10.2017	CSI-W5*	4	1.40 - 1.60	450	108	11	24.03.2007	M	BWP	91	5	30	9	36	2556	124	M	7	5	no	spread	oxer	right	no	0	0	72,71	no
Helsinki	The Longines FEI World Cup Jumping - Helsinki round A	22.10.2017	CSI-W5*	4	1.40 - 1.60	450	108	11	24.03.2007	M	BWP	91	5	30	9	36	2556	124	M	8	6a	yes	vertical	vertical	right	no	0	0	72,71	no
Helsinki	The Longines FEI World Cup Jumping - Helsinki round A	22.10.2017	CSI-W5*	4	1.40 - 1.60	450	108	11	24.03.2007	M	BWP	91	5	30	9	36	2556	124	M	9	6b	yes	vertical	vertical	right	no	0	0	72,71	no
Helsinki	The Longines FEI World Cup Jumping - Helsinki round A	22.10.2017	CSI-W5*	4	1.40 - 1.60	450	108	11	24.03.2007	M	BWP	91	5	30	9	36	2556	124	M	10	7	no	spread	oxer	left	no	0	0	72,71	no
Helsinki	The Longines FEI World Cup Jumping - Helsinki round A	22.10.2017	CSI-W5*	4	1.40 - 1.60	450	108	11	24.03.2007	M	BWP	91	5	30	9	36	2556	124	M	11	8	no	vertical	wall	left	no	0	0	72,71	no
Helsinki	The Longines FEI World Cup Jumping - Helsinki round A	22.10.2017	CSI-W5*	4	1.40 - 1.60	450	108	11	24.03.2007	M	BWP	91	5	30	9	36	2556	124	M	12	9	no	spread	oxer	straight	no	0	0	72,71	no
Helsinki	The Longines FEI World Cup Jumping - Helsinki round A	22.10.2017	CSI-W5*	4	1.40 - 1.60	450	108	11	24.03.2007	M	BWP	91	5	30	9	36	2556	124	M	13	10	no	spread	triple bar	left	no	0	0	72,71	no
Helsinki	The Longines FEI World Cup Jumping - Helsinki round A	22.10.2017	CSI-W5*	4	1.40 - 1.60	450	108	11	24.03.2007	M	BWP	91	5	30	9	36	2556	124	M	14	11	no	vertical	vertical with water	straight	no	0	0	72,71	no
Helsinki	The Longines FEI World Cup Jumping - Helsinki round A	22.10.2017	CSI-W5*	4	1.40 - 1.60	450	108	11	24.03.2007	M	BWP	91	5	30	9	36	2556	124	M	15	12a	yes	spread	oxer	right	no	0	0	72,71	no
Helsinki	The Longines FEI World Cup Jumping - Helsinki round A	22.10.2017	CSI-W5*	4	1.40 - 1.60	450	108	11	24.03.2007	M	BWP	91	5	30	9	36	2556	124	M	16	12b	yes	vertical	vertical	right	yes	4	0	72,71	no
Helsinki	The Longines FEI World Cup Jumping - Helsinki round A	22.10.2017	CSI-W5*	4	1.40 - 1.60	450	108	11	24.03.2007	M	BWP	91	5	30	9	36	2556	124	M	17	13	no	spread	oxer	right	no	0	0	72,71	no
Helsinki	The Longines FEI World Cup Jumping - Helsinki round A	22.10.2017	CSI-W5*	4	1.40 - 1.60	450	178	12	31.05.2006	G	SF	218	3	31	167	28	855	19	M	1	1	no	vertical	vertical	right	no	0	0	72,22	no
Helsinki	The Longines FEI World Cup Jumping - Helsinki round A	22.10.2017	CSI-W5*	4	1.40 - 1.60	450	178	12	31.05.2006	G	SF	218	3	31	167	28	855	19	M	2	2	no	vertical	vertical	left	no	0	0	72,22	no
Helsinki	The Longines FEI World Cup Jumping - Helsinki round A	22.10.2017	CSI-W5*	4	1.40 - 1.60	450	178	12	31.05.2006	G	SF	218	3	31	167	28	855	19	M	3	3	no	spread	oxer	left	no	0	0	72,22	no
Helsinki	The Longines FEI World Cup Jumping - Helsinki round A	22.10.2017	CSI-W5*	4	1.40 - 1.60	450	178	12	31.05.2006	G	SF	218	3	31	167	28	855	19	M	4	4a	yes	spread	oxer	left	no	0	0	72,22	no
Helsinki	The Longines FEI World Cup Jumping - Helsinki round A	22.10.2017	CSI-W5*	4	1.40 - 1.60	450	178	12	31.05.2006	G	SF	218	3	31	167	28	855	19	M	5	4b	yes	vertical	vertical	left	no	0	0	72,22	no
Helsinki	The Longines FEI World Cup Jumping - Helsinki round A	22.10.2017	CSI-W5*	4	1.40 - 1.60	450	178	12	31.05.2006	G	SF	218	3	31	167	28	855	19	M	6	4c	yes	spread	oxer	left	no	0	0	72,22	no

Helsinki	The Longines FEI World Cup Jumping - Helsinki round A	22.10.2017	CSI-W5*	4	1.40 - 1.60	450	178	12	31.05.2006	G	SF	218	3	31	167	28	855	19	M	7	5	no	spread	oxer	right	no	0	0	72,22	no
Helsinki	The Longines FEI World Cup Jumping - Helsinki round A	22.10.2017	CSI-W5*	4	1.40 - 1.60	450	178	12	31.05.2006	G	SF	218	3	31	167	28	855	19	M	8	6a	yes	vertical	vertical	right	no	0	0	72,22	no
Helsinki	The Longines FEI World Cup Jumping - Helsinki round A	22.10.2017	CSI-W5*	4	1.40 - 1.60	450	178	12	31.05.2006	G	SF	218	3	31	167	28	855	19	M	9	6b	yes	vertical	vertical	right	yes	4	0	72,22	no
Helsinki	The Longines FEI World Cup Jumping - Helsinki round A	22.10.2017	CSI-W5*	4	1.40 - 1.60	450	178	12	31.05.2006	G	SF	218	3	31	167	28	855	19	M	10	7	no	spread	oxer	left	no	0	0	72,22	no
Helsinki	The Longines FEI World Cup Jumping - Helsinki round A	22.10.2017	CSI-W5*	4	1.40 - 1.60	450	178	12	31.05.2006	G	SF	218	3	31	167	28	855	19	M	11	8	no	vertical	wall	left	no	0	0	72,22	no
Helsinki	The Longines FEI World Cup Jumping - Helsinki round A	22.10.2017	CSI-W5*	4	1.40 - 1.60	450	178	12	31.05.2006	G	SF	218	3	31	167	28	855	19	M	12	9	no	spread	oxer	straight	no	0	0	72,22	no
Helsinki	The Longines FEI World Cup Jumping - Helsinki round A	22.10.2017	CSI-W5*	4	1.40 - 1.60	450	178	12	31.05.2006	G	SF	218	3	31	167	28	855	19	M	13	10	no	spread	triple bar	left	no	0	0	72,22	no
Helsinki	The Longines FEI World Cup Jumping - Helsinki round A	22.10.2017	CSI-W5*	4	1.40 - 1.60	450	178	12	31.05.2006	G	SF	218	3	31	167	28	855	19	M	14	11	no	vertical	vertical with water	straight	no	0	0	72,22	no
Helsinki	The Longines FEI World Cup Jumping - Helsinki round A	22.10.2017	CSI-W5*	4	1.40 - 1.60	450	178	12	31.05.2006	G	SF	218	3	31	167	28	855	19	M	15	12a	yes	spread	oxer	right	no	0	0	72,22	no
Helsinki	The Longines FEI World Cup Jumping - Helsinki round A	22.10.2017	CSI-W5*	4	1.40 - 1.60	450	178	12	31.05.2006	G	SF	218	3	31	167	28	855	19	M	16	12b	yes	vertical	vertical	right	no	0	0	72,22	no
Helsinki	The Longines FEI World Cup Jumping - Helsinki round A	22.10.2017	CSI-W5*	4	1.40 - 1.60	450	178	12	31.05.2006	G	SF	218	3	31	167	28	855	19	M	17	13	no	spread	oxer	right	no	0	0	72,22	no
Helsinki	The Longines FEI World Cup Jumping - Helsinki round A	22.10.2017	CSI-W5*	4	1.40 - 1.60	450	23	13	25.02.2005	S	Bezplemenné	158	2	32	53	23	1832	77	M	1	1	no	vertical	vertical	right	no	0	0	72,49	no
Helsinki	The Longines FEI World Cup Jumping - Helsinki round A	22.10.2017	CSI-W5*	4	1.40 - 1.60	450	23	13	25.02.2005	S	Bezplemenné	158	2	32	53	23	1832	77	M	2	2	no	vertical	vertical	left	no	0	0	72,49	no
Helsinki	The Longines FEI World Cup Jumping - Helsinki round A	22.10.2017	CSI-W5*	4	1.40 - 1.60	450	23	13	25.02.2005	S	Bezplemenné	158	2	32	53	23	1832	77	M	3	3	no	spread	oxer	left	no	0	0	72,49	no
Helsinki	The Longines FEI World Cup Jumping - Helsinki round A	22.10.2017	CSI-W5*	4	1.40 - 1.60	450	23	13	25.02.2005	S	Bezplemenné	158	2	32	53	23	1832	77	M	4	4a	yes	spread	oxer	left	no	0	0	72,49	no
Helsinki	The Longines FEI World Cup Jumping - Helsinki round A	22.10.2017	CSI-W5*	4	1.40 - 1.60	450	23	13	25.02.2005	S	Bezplemenné	158	2	32	53	23	1832	77	M	5	4b	yes	vertical	vertical	left	no	0	0	72,49	no
Helsinki	The Longines FEI World Cup Jumping - Helsinki round A	22.10.2017	CSI-W5*	4	1.40 - 1.60	450	23	13	25.02.2005	S	Bezplemenné	158	2	32	53	23	1832	77	M	6	4c	yes	spread	oxer	left	no	0	0	72,49	no
Helsinki	The Longines FEI World Cup Jumping - Helsinki round A	22.10.2017	CSI-W5*	4	1.40 - 1.60	450	23	13	25.02.2005	S	Bezplemenné	158	2	32	53	23	1832	77	M	7	5	no	spread	oxer	right	no	0	0	72,49	no
Helsinki	The Longines FEI World Cup Jumping - Helsinki round A	22.10.2017	CSI-W5*	4	1.40 - 1.60	450	23	13	25.02.2005	S	Bezplemenné	158	2	32	53	23	1832	77	M	8	6a	yes	vertical	vertical	right	no	0	0	72,49	no
Helsinki	The Longines FEI World Cup Jumping - Helsinki round A	22.10.2017	CSI-W5*	4	1.40 - 1.60	450	23	13	25.02.2005	S	Bezplemenné	158	2	32	53	23	1832	77	M	9	6b	yes	vertical	vertical	right	no	0	0	72,49	no
Helsinki	The Longines FEI World Cup Jumping - Helsinki round A	22.10.2017	CSI-W5*	4	1.40 - 1.60	450	23	13	25.02.2005	S	Bezplemenné	158	2	32	53	23	1832	77	M	10	7	no	spread	oxer	left	yes	4	0	72,49	no
Helsinki	The Longines FEI World Cup Jumping - Helsinki round A	22.10.2017	CSI-W5*	4	1.40 - 1.60	450	23	13	25.02.2005	S	Bezplemenné	158	2	32	53	23	1832	77	M	11	8	no	vertical	wall	left	no	0	0	72,49	no
Helsinki	The Longines FEI World Cup Jumping - Helsinki round A	22.10.2017	CSI-W5*	4	1.40 - 1.60	450	23	13	25.02.2005	S	Bezplemenné	158	2	32	53	23	1832	77	M	12	9	no	spread	oxer	straight	no	0	0	72,49	no
Helsinki	The Longines FEI World Cup Jumping - Helsinki round A	22.10.2017	CSI-W5*	4	1.40 - 1.60	450	23	13	25.02.2005	S	Bezplemenné	158	2	32	53	23	1832	77	M	13	10	no	spread	triple bar	left	no	0	0	72,49	no
Helsinki	The Longines FEI World Cup Jumping - Helsinki round A	22.10.2017	CSI-W5*	4	1.40 - 1.60	450	23	13	25.02.2005	S	Bezplemenné	158	2	32	53	23	1832	77	M	14	11	no	vertical	vertical with water	straight	no	0	0	72,49	no
Helsinki	The Longines FEI World Cup Jumping - Helsinki round A	22.10.2017	CSI-W5*	4	1.40 - 1.60	450	23	13	25.02.2005	S	Bezplemenné	158	2	32	53	23	1832	77	M	15	12a	yes	spread	oxer	right	no	0	0	72,49	no
Helsinki	The Longines FEI World Cup Jumping - Helsinki round A	22.10.2017	CSI-W5*	4	1.40 - 1.60	450	23	13	25.02.2005	S	Bezplemenné	158	2	32	53	23	1832	77	M	16	12b	yes	vertical	vertical	right	no	0	0	72,49	no
Helsinki	The Longines FEI World Cup Jumping - Helsinki round A	22.10.2017	CSI-W5*	4	1.40 - 1.60	450	23	13	25.02.2005	S	Bezplemenné	158	2	32	53	23	1832	77	M	17	13	no	spread	oxer	right	no	0	0	72,49	no
Helsinki	The Longines FEI World Cup Jumping - Helsinki round A	22.10.2017	CSI-W5*	4	1.40 - 1.60	450	134	10	28.04.2008	G	BWP	174	6	33	23	23	1854	199	M	1	1	no	vertical	vertical	right	no	0	0	72,35	no
Helsinki	The Longines FEI World Cup Jumping - Helsinki round A	22.10.2017	CSI-W5*	4	1.40 - 1.60	450	134	10	28.04.2008	G	BWP	174	6	33	23	23	1854	199	M	2	2	no	vertical	vertical	left	no	0	0	72,35	no
Helsinki	The Longines FEI World Cup Jumping - Helsinki round A	22.10.2017	CSI-W5*	4	1.40 - 1.60	450	134	10	28.04.2008	G	BWP	174	6	33	23	23	1854	199	M	3	3	no	spread	oxer	left	no	0	0	72,35	no
Helsinki	The Longines FEI World Cup Jumping - Helsinki round A	22.10.2017	CSI-W5*	4	1.40 - 1.60	450	134	10	28.04.2008	G	BWP	174	6	33	23	23	1854	199	M	4	4a	yes	spread	oxer	left	no	0	0	72,35	no
Helsinki	The Longines FEI World Cup Jumping - Helsinki round A	22.10.2017	CSI-W5*	4	1.40 - 1.60	450	134	10	28.04.2008	G	BWP	174	6	33	23	23	1854	199	M	5	4b	yes	vertical	vertical	left	no	0	0	72,35	no
Helsinki	The Longines FEI World Cup Jumping - Helsinki round A	22.10.2017	CSI-W5*	4	1.40 - 1.60	450	134	10	28.04.2008	G	BWP	174	6	33	23	23	1854	199	M	6	4c	yes	spread	oxer	left	no	0	0	72,35	no
Helsinki	The Longines FEI World Cup Jumping - Helsinki round A	22.10.2017	CSI-W5*	4	1.40 - 1.60	450	134	10	28.04.2008	G	BWP	174	6	33	23	23	1854	199	M	7	5	no	spread	oxer	right	no	0	0	72,35	no
Helsinki	The Longines FEI World Cup Jumping - Helsinki round A	22.10.2017	CSI-W5*	4	1.40 - 1.60	450	134	10	28.04.2008	G	BWP	174	6	33	23	23	1854	199	M	8	6a	yes	vertical	vertical	right	no	0	0	72,35	no
Helsinki	The Longines FEI World Cup Jumping - Helsinki round A	22.10.2017	CSI-W5*	4	1.40 - 1.60	450	134	10	28.04.2008	G	BWP	174	6	33	23	23	1854	199	M	9	6b	yes	vertical	vertical	right	no	0	0	72,35	no
Helsinki	The Longines FEI World Cup Jumping - Helsinki round A	22.10.2017	CSI-W5*	4	1.40 - 1.60	450	134	10	28.04.2008	G	BWP	174	6	33	23	23	1854	199	M	10	7	no	spread	oxer	left	no	0	0	72,35	no
Helsinki	The Longines FEI World Cup Jumping - Helsinki round A	22.10.2017	CSI-W5*	4	1.40 - 1.60	450	134	10	28.04.2008	G	BWP	174	6	33	23	23	1854	199	M	11	8	no	vertical	wall	left	no	0	0	72,35	no
Helsinki	The Longines FEI World Cup Jumping - Helsinki round A	22.10.2017	CSI-W5*	4	1.40 - 1.60	450	134	10	28.04.2008	G	BWP	174	6	33	23	23	1854	199	M	12	9	no	spread	oxer	straight	no	0	0	72,35	no
Helsinki	The Longines FEI World Cup Jumping - Helsinki round A	22.10.2017	CSI-W5*	4	1.40 - 1.60	450	134	10	28.04.2008	G	BWP	174	6	33	23	23	1854	199	M	13	10	no	spread	triple bar	left	no	0	0	72,35	no
Helsinki	The Longines FEI World Cup Jumping - Helsinki round A	22.10.2017	CSI-W5*	4	1.40 - 1.60	450	134	10	28.04.2008	G	BWP	174	6	33	23	23	1854	199	M	14	11	no	vertical	vertical with water	straight	yes	4	0	72,35	no
Helsinki	The Longines FEI World Cup Jumping - Helsinki round A	22.10.2017	CSI-W5*	4	1.40 - 1.60	450	134	10	28.04.2008	G	BWP	174	6	33	23	23	1854	199	M	15	12a	yes	spread	oxer	right	yes	4	0	72,35	no
Helsinki	The Longines FEI World Cup Jumping - Helsinki round A	22.10.2017	CSI-W5*	4	1.40 - 1.60	450	134	10	28.04.2008	G	BWP	174	6	33	23	23	1854	199	M	16	12b	yes	vertical	vertical	right	yes	4	0	72,35	no
Helsinki	The Longines FEI World Cup Jumping - Helsinki round A	22.10.2017	CSI-W5*	4	1.40 - 1.60	450	134	10	28.04.2008	G	BWP	174	6	33	23	23	1854	199	M	17	13	no	spread	oxer	right	no	0	0	72,35	no
Helsinki	The Longines FEI World Cup Jumping - Helsinki round A	22.10.2017	CSI-W5*	4	1.40 - 1.60	450	37	11	02.03.2007	M	KWPN	133	11	35	44	30	2234	138	M	1	1	no	vertical	vertical	right	no	0	0	75,05	no
Helsinki	The Longines FEI World Cup Jumping - Helsinki round A	22.10.2017	CSI-W5*	4	1.40 - 1.60	450	37	11	02.03.2007	M	KWPN	133	11	35	44	30	2234	138	M	2	2	no	vertical	vertical	left	no	0	0	75,05	no
Helsinki	The Longines FEI World Cup Jumping - Helsinki round A	22.10.2017	CSI-W5*	4	1.40 - 1.60	450	37	11	02.03.2007	M	KWPN	133	11	35	44	30	2234	138	M	3	3	no	spread	oxer	left	no	0	0	75,05	no
Helsinki	The Longines FEI World Cup Jumping - Helsinki round A	22.10.2017	CSI-W5*	4	1.40 - 1.60	450	37	11	02.03.2007	M	KWPN	133	11	35	44	30	2234	138	M	4	4a	yes	spread	oxer	left	no	0	0	75,05	no
Helsinki	The Longines FEI World Cup Jumping - Helsinki round A	22.10.2017	CSI-W5*	4	1.40 - 1.60	450	37	11	02.03.2007	M	KWPN	133	11	35	44	30	2234	138	M	5	4b	yes	vertical	vertical	left	no	0	0	75,05	no
Helsinki	The Longines FEI World Cup Jumping - Helsinki round A	22.10.2017	CSI-W5*	4	1.40 - 1.60	450	37	11	02.03.2007	M	KWPN	133	11	35	44	30	2234	138	M	6	4c	yes	spread	oxer	left	no	0	0	75,05	no
Helsinki	The Longines FEI World Cup Jumping - Helsinki round A	22.10.2017	CSI-W5*	4	1.40 - 1.60	450	37	11	02.03.2007	M	KWPN	133	11	35	44	30	2234	138	M	7	5	no	spread	oxer	right	no	0	0	75,05	no
Helsinki	The Longines FEI World Cup Jumping - Helsinki round A	22.10.2017	CSI-W5*	4	1.40 - 1.60	450	37	11	02.03.2007	M	KWPN	133	11	35	44	30	2234	138	M	8	6a	yes	vertical	vertical	right	no	0	0	75,05	no
Helsinki	The Longines FEI World Cup Jumping - Helsinki round A	22.10.2017	CSI-W5*	4	1.40 - 1.60	450	37	11	02.03.2007	M	KWPN	133	11	35	44	30	2234	138	M	9	6b	yes	vertical	vertical	right	no	0	0	75,05	no
Helsinki	The Longines FEI World Cup Jumping - Helsinki round A	22.10.2017	CSI-W5*	4	1.40 - 1.60	450	37	11	02.03.2007	M	KWPN	133	11	35	44	30	2234	138	M	10	7	no	spread	oxer	left	no	0	0	75,05	no
Helsinki	The Longines FEI World Cup Jumping - Helsinki round A	22.10.2017	CSI-W5*	4	1.40 - 1.60	450	37	11	02.03.2007	M	KWPN	133	11	35	44	30	2234	138	M	11	8	no	vertical	wall	left	no	0	0	75,05	no
Helsinki	The Longines FEI World Cup Jumping - Helsinki round A	22.10.2017	CSI-W5*	4	1.40 - 1.60	450	37	11	02.03.2007	M	KWPN	133	11	35	44	30	2234	138	M	12	9	no	spread	oxer	straight	no	0	0	75,05	no
Helsinki	The Longines FEI World Cup Jumping - Helsinki round A	22.10.2017	CSI-W5*	4	1.40 - 1.60	450	37	11	02.03.2007	M	KWPN	133	11	35	44	30	2234	138	M	13	10	no	spread	triple bar	left	no	0	0	75,05	no
Helsinki	The Longines FEI World Cup Jumping - Helsinki round A	22.10.2017	CSI-W5*	4	1.40 - 1.60	450	37	11	02.03.2007	M	KWPN	133	11	35	44	30	2234	138	M	14	11	no	vertical	vertical with water	straight	no	0	0	75,05	no
Helsinki	The Longines FEI World Cup Jumping - Helsinki round A	22.10.2017	CSI-W5*	4	1.40 - 1.60	450	37	11	02.03.2007	M	KWPN	133	11	35	44	30	2234	138	M	15	12a	yes	spread	oxer	right	no	0	0	75,05	no
Helsinki	The Longines FEI World Cup Jumping - Helsinki round A	22.10.2017	CSI-W5*	4	1.40 - 1.60	450	37	11	02.03.2007	M	KWPN	133	11	35	44	30	2234	138	M	16	12b	yes	vertical	vertical	right	no	0	0	75,05	no
Helsinki	The Longines FEI World Cup Jumping - Helsinki round A	22.10.2017	CSI-W5*	4	1.40 - 1.60	450	37	11	02.03.2007	M	KWPN	133	11	35	44	30	2234	138	M	17	13	no	spread	oxer	right	no	0	0	75,05	no
Helsinki	The Longines FEI World Cup Jumping - Helsinki round B	22.10.2017	CSI-W5*	4	1.40 - 1.60	270	37	11	02.03.2007	M	KWPN	133	11	35	44	30	2234	138	M	1	1	no	spread	oxer	right	no	0	0	38,6	no
Helsinki	The Longines FEI World Cup Jumping - Helsinki round B	22.10.2017	CSI-W5*	4	1.40 - 1.60	270	37	11	02.03.2007	M	KWPN	133	11	35	44	30	2234	138	M	2	2	no	vertical	vertical	right	no	0	0	38,6	no
Helsinki	The Longines FEI World Cup Jumping - Helsinki round B	22.10.2017	CSI-W5*	4	1.40 - 1.60	270	37	11	02.03.2007	M	KWPN	133	11	35	44	30	2234	138	M	3	3	no	spread	oxer	left	no	0	0	38,6	no
Helsinki	The Longines FEI World Cup Jumping - Helsinki round B	22.10.2017	CSI-W5*	4	1.40 - 1.60	270	37	11	02.03.2007	M	KWPN	133	11	35	44	30	2234	138	M	4	4a	yes	vertical	vertical	left	no	0	0	38,6	no
Helsinki	The Longines FEI World Cup Jumping - Helsinki round B	22.10.2017	CSI-W5*	4	1.40 - 1.60	270	37	11	02.03.2007	M	KWPN	133	11	35	44	30	2234	138	M	5	4b	yes	spread	oxer	left	no	0	0	38,6	no
Helsinki	The Longines FEI World Cup Jumping - Helsinki round B	22.10.2017	CSI-W5*	4	1.40 - 1.60	270	37	11	02.03.2007	M	KWPN	133	11	35	44	30	2234	138	M	6	5	no	spread	oxer	right	no	0	0	38,6	no
Helsinki	The Longines FEI World Cup Jumping - Helsinki round B	22.10.2017	CSI-W5*	4	1.40 - 1.60	270	37	11	02.03.2007	M	KWPN	133	11	35	44	30	2234	138	M	7	6	no	vertical	vertical	right	no	0	0	38,6	no
Helsinki	The Longines FEI World Cup Jumping - Helsinki round B	22.10.2017	CSI-W5*	4	1.40 - 1.60	270	37	11	02.03.2007	M	KWPN	133	11	35	44	30	2234	138	M	8	7	no	spread	oxer	left	no	0	0	38,6	no
Helsinki	The Longines FEI World Cup Jumping - Helsinki round A	22.10.2017	CSI-W5*	4	1.40 - 1.60	450	166	14	01.03.2004	M	AA	187	5	37	59	42	1314	68	M	2	2	no	vertical	vertical	left	no	0	0	74,31	no
Helsinki	The Longines FEI World Cup Jumping - Helsinki round A	22.10.2017	CSI-W5*	4	1.40 - 1.60	450	166	14	01.03.2004	M	AA	187	5	37	59	42	1314	68	M	3	3	no	spread	oxer	left	no	0	0	74,31	no
Helsinki	The Longines FEI World Cup Jumping - Helsinki round A	22.10.2017	CSI-W5*	4	1.40 - 1.60	450	166	14	01.03.2004	M	AA	187	5	37	59	42	1314	68	M	4	4a	yes	spread	oxer	left	no	0	0	74,31	no
Helsinki	The Longines FEI World Cup Jumping - Helsinki round A	22.10.2017	CSI-W5*	4	1.40 - 1.60	450	166	14	01.03.2004	M	AA	187	5	37	59	42	1314	68	M	5	4b	yes	vertical	vertical	left	no	0	0	74,31	no
Helsinki	The Longines FEI World Cup Jumping - Helsinki round A	22.10.2017	CSI-W5*	4	1.40 - 1.60	450	166	14	01.03.2004	M	AA	187	5	37	59	42	1314	68	M	6	4c	yes	spread	oxer	left	no	0	0	74,31	no
Helsinki	The Longines FEI World Cup Jumping - Helsinki round A	22.10.2017	CSI-W5*	4	1.40 - 1.60	450	166	14	01.03.2004	M	AA	187	5	37	59	42	1314	68	M	7	5	no	spread	oxer	right	no	0	0	74,31	no
Helsinki	The Longines FEI World Cup Jumping - Helsinki round A	22.10.2017	CSI-W5*	4	1.40 - 1.60	450	166	14	01.03.2004	M	AA	187	5	37	59	42	1314	68	M	8	6a	yes	vertical	vertical	right	no	0	0	74,31	no
Helsinki	The Longines FEI World Cup Jumping - Helsinki round A	22.10.2017	CSI-W5*	4	1.40 - 1.60	450	166	14	01.03.2004	M	AA	187	5	37	59	42	1314	68	M	9	6b	yes	vertical	vertical	right	no	0	0	74,31	no
Helsinki	The Longines FEI World Cup Jumping - Helsinki round A	22.10.2017	CSI-W5*	4	1.40 - 1.60	450	166	14	01.03.2004	M	AA	187	5	37	59	42	1314	68	M	10	7	no	spread	oxer	left	no	0	0	74,31	no
Helsinki	The Longines FEI World Cup Jumping - Helsinki round A	22.10.2017	CSI-W5*	4	1.40 - 1.60	450	166	14	01.03.2004	M	AA	187	5	37	59	42	1314	68	M	11	8	no	vertical	wall	left	no	0	0	74,31	no
Helsinki	The Longines FEI World Cup Jumping - Helsinki round A	22.10.2017	CSI-W5*	4	1.40 - 1.60	450	166	14	01.03.2004	M	AA	187	5	37	59	42	1314	68	M	12	9	no	spread	oxer	straight	no	0	0	74,31	no
Helsinki	The Longines FEI World Cup Jumping - Helsinki round A	22.10.2017	CSI-W5*	4	1.40 - 1.60	450	166	14	01.03.2004	M	AA	187	5	37	59	42	1314	68	M	13	10	no	spread	triple bar	left	no	0	0	74,31	no
Helsinki	The Longines FEI World Cup Jumping - Helsinki round A	22.10.2017	CSI-W5*	4	1.40 - 1.60	450	166	14	01.03.2004	M	AA	187	5	37	59	42	1314	68	M	14	11	no	vertical	vertical with water	straight	no	0	0	74,31	no
Helsinki	The Longines FEI World Cup Jumping - Helsinki round A	22.10.2017	CSI-W5*	4	1.40 - 1.60	450	166	14	01.03.2004	M	AA	187	5	37	59	42	1314	68	M	15	12a	yes	spread	oxer	right	no	0	0	74,31	no
Helsinki	The Longines FEI World Cup Jumping - Helsinki round A	22.10.2017	CSI-W5*	4	1.40 - 1.60	450	166	14	01.03.2004	M	AA	187	5	37	59	42	1314	68	M	16	12b	yes	vertical	vertical	right	yes	4	0	74,31	no
Helsinki	The Longines FEI World Cup Jumping - Helsinki round A	22.10.2017	CSI-W5*	4	1.40 - 1.60	450	166	14	01.03.2004	M	AA	187	5	37	59	42	1314	68	M	17	13	no	spread	oxer	right	no	0	0	74,31	no
Helsinki	The Longines FEI World Cup Jumping - Helsinki round A	22.10.2017	CSI-W5*	4	1.40 - 1.60	450	166	14	01.03.2004	M	AA	187	5	37	59	42	1314	68	M	1	1	no	vertical	vertical	right	no	0	0	39,11	no
Helsinki	The Longines FEI World Cup Jumping - Helsinki round A	22.10.2017	CSI-W5*	4	1.40 - 1.60	450	48	17	01.01.2001	G	WESTF	290	9	39	71	51	2525	123	M	1	1	no	vertical	vertical	right	no	0	0	0,00	yes
Helsinki	The Longines FEI World Cup Jumping - Helsinki round A	22.10.2017	CSI-W5*	4	1.40 - 1.60	450	48	17	01.01.2001	G	WESTF	290	9	39	71	51	2525	123	M	2	2	no	vertical	vertical	left	no	0	0	0,00	yes
Helsinki	The Longines FEI World Cup Jumping - Helsinki round A	22.10.2017	CSI-W5*	4	1.40 - 1.60	450	48	17	01.01.2001	G	WESTF	290	9	39	71	51	2525	123	M	3	3	no	spread	oxer	left	no	0	0	0,00	yes
Helsinki	The Longines FEI World Cup Jumping - Helsinki round A	22.10.2017	CSI-W5*	4	1.40 - 1.60	450	48	17	01.01.2001	G	WESTF	290	9	39	71	51	2525	123	M	4	4a	yes	spread	oxer	left	no	0	0	0,00	yes
Helsinki	The Longines FEI World Cup Jumping - Helsinki round A	22.10.2017	CSI-W5*	4	1.40 - 1.60	450	48	17	01.01.2001	G	WESTF	290	9	39	71	51	2525	123	M	5	4b	yes	vertical	vertical	left	yes	4	0	0,00	yes
Helsinki	The Longines FEI World Cup Jumping - Helsinki round A	22.10.2017	CSI-W5*	4	1.40 - 1.60	450	48	17	01.01.2001	G	WESTF	290	9	39	71	51	2525	123	M	6	4c	yes	spread	oxer	left	yes	4	0	0,00	yes
Helsinki	The Longines FEI World Cup Jumping - Helsinki round A	22.10.2017	CSI-W5*	4	1.40 - 1.60	450	48	17	01.01.2001	G	WESTF	290	9	39	71	51	2525	123	M	7	5	no	spread	oxer	right	no	0	0	0,00	yes
Helsinki	The Longines FEI World Cup Jumping - Helsinki round A	22.10.2017	CSI-W5*	4	1.40 - 1.60	450	48	17	01.01.2001	G	WESTF	290	9	39	71	51	2525	123	M	8	6a	yes	vertical	vertical	right	no	0	0	0,00	yes
Helsinki	The Longines FEI World Cup Jumping - Helsinki round A	22.10.2017	CSI-W5*	4	1.40 - 1.60	450	48	17	01.01.2001	G	WESTF	290	9	39	71	51	2525	123	M	9	6b	yes	vertical	vertical	right	yes	4	0	0,00	yes
Helsinki	The Longines FEI World Cup Jumping - Helsinki round A	22.10.2017	CSI-W5*	4	1.40 - 1.60	450	48	17	01.01.2001	G	WESTF	290	9	39	71	51	2525	123	M	10	7	no	spread	oxer	left	no	0	0	0,00	yes
Helsinki	The Longines FEI World Cup Jumping - Helsinki round A	22.10.2017	CSI-W5*	4	1.40 - 1.60	450	48	17	01.01.2001	G	WESTF	290	9	39	71	51	2525	123	M	11	8	no	vertical	wall	left	no	0	0	0,00	yes
Helsinki	The Longines FEI World Cup Jumping - Helsinki round A	22.10.2017	CSI-W5*	4	1.40 - 1.60	450	48	17	01.01.2001	G	WESTF	290	9	39	71	51	2525	123	M	12	9	no	spread	oxer	straight	no	0	0	0,00	yes
Helsinki	The Longines FEI World Cup Jumping - Helsinki round A	22.10.2017	CSI-W5*	4	1.40 - 1.60	450	48	17	01.01.2001	G	WESTF	290	9	39	71	51	2525	123	M	13	10	no	spread	triple bar	left	no	0	0	0,00	yes
Helsinki	The Longines FEI World Cup Jumping - Helsinki round A	22.10.2017	CSI-W5*	4	1.40 - 1.60	450	48	17	01.01.2001	G	WESTF	290	9	39	71	51	2525	123	M	14	11	no	vertical	vertical with water	straight	no	0	0	0,00	yes
Helsinki	The Longines FEI World Cup Jumping - Helsinki round A	22.10.2017	CSI-W5*	4	1.40 - 1.60	450	48	17	01.01.2001	G	WESTF	290	9	39	71	51	2525	123	M	15	12a	yes	spread	oxer	right	no	0	0	0,00	yes
Helsinki	The Longines FEI World Cup Jumping - Helsinki round A	22.10.2017	CSI-W5*	4	1.40 - 1.60	450	48	17	01.01.2001	G	WESTF	290	9	39	71	51	2525	123	M	16	12b	yes	vertical	vertical	right	no	0	0	0,00	yes
Helsinki	The Longines FEI World Cup Jumping - Helsinki round A	22.10.2017	CSI-W5*	4	1.40 - 1.60	450	48	17	01.01.2001	G	WESTF	290	9	39	71	51	2525	123	M	17	13	no	spread	oxer	right	no	0	0	0,00	yes
Helsinki	The Longines FEI World Cup Jumping - Helsinki round A	22.10.2017	CSI-W5*	4	1.40 - 1.60	450	170	13	06.04.2005	M	SF	135	10	40	137	40	883	76	M	1	1	no	vertical	vertical	right	no	0	0	0,00	yes
Helsinki	The Longines FEI World Cup Jumping - Helsinki round A	22.10.2017	CSI-W5*	4	1.40 - 1.60	450	170	13	06.04.2005	M	SF	135	10	40	137	40	883	76	M	2	2	no	vertical	vertical	left	yes	4	0	0,00	yes
Helsinki	The Longines FEI World Cup Jumping - Helsinki round A	22.10.2017	CSI-W5*	4	1.40 - 1.60	450	170	13	06.04.2005	M	SF	135	10	40	137	40	883	76	M	3	3	no	spread	oxer	left	yes	4	0	0,00	yes
Helsinki	The Longines FEI World Cup Jumping - Helsinki round A	22.10.2017	CSI-W5*	4	1.40 - 1.60	450	170	13	06.04.2005	M	SF	135	10	40	137	40	883	76	M	4	4a	yes	spread	oxer	left	no	0	0	0,00	yes
Helsinki	The Longines FEI World Cup Jumping - Helsinki round A	22.10.2017	CSI-W5*	4	1.40 - 1.60	450	170	13	06.04.2005	M	SF	135	10	40	137	40	883	76	M	5	4b	yes	vertical	vertical	left	no	0	0	0,00	yes
Helsinki	The Longines FEI World Cup Jumping - Helsinki round A	22.10.2017	CSI-W5*	4	1.40 - 1.60	450	170	13	06.04.2005	M	SF	135	10	40	137	40	883	76	M	6	4c	yes	spread	oxer	left	no	0	0	0,00	yes
Helsinki	The Longines FEI World Cup Jumping - Helsinki round A	22.10.2017	CSI-W5*	4	1.40 - 1.60	450	170	13	06.04.2005	M	SF	135	10	40	137	40	883	76	M	7	5	no	spread	oxer	right	no	0	0	0,00	yes
Helsinki	The Longines FEI World Cup Jumping - Helsinki round A	22.10.2017	CSI-W5*	4	1.40 - 1.60	450	170	13	06.04.2005	M	SF	135	10	40	137	40	883	76	M	8	6a	yes	vertical	vertical	right	no	0	0	0,00	yes
Helsinki	The Longines FEI World Cup Jumping - Helsinki round A	22.10.2017	CSI-W5*	4	1.40 - 1.60	450	170	13	06.04.2005	M	SF	135	10	40	137	40	883	76	M	9	6b	yes	vertical	vertical	right	no	0	0	0,00	yes
Helsinki	The Longines FEI World Cup Jumping - Helsinki round A	22.10.2017	CSI-W5*	4	1.40 - 1.60	450	170	13	06.04.2005	M	SF	135	10	40	137	40	883	76	M	10	7	no	spread	oxer	left	no	0	0	0,00	yes
Helsinki	The Longines FEI World Cup Jumping - Helsinki round A	22.10.2017	CSI-W5*	4	1.40 - 1.60	450	170	13	06.04.2005	M	SF	135	10	40	137	40	883	76	M	11	8	no	vertical	wall	left	no	0	0	0,00	yes
Helsinki	The Longines FEI World Cup Jumping - Helsinki round A	22.10.2017	CSI-W5*	4	1.40 - 1.60	450	170	13	06.04.2005	M	SF	135	10	40	137	40	883	76	M	12	9	no	spread	oxer	straight	no	0	0	0,00	yes
Helsinki	The Longines FEI World Cup Jumping - Helsinki round A	22.10.2017	CSI-W5*	4	1.40 - 1.60	450	170	13	06.04.2005	M	SF	135	10	40	137	40	883	76	M	13	10	no	spread	triple bar	left	no	0	0	0,00	yes
Helsinki	The Longines FEI World Cup Jumping - Helsinki round A	22.10.2017	CSI-W5*	4	1.40 - 1.60	450	170	13	06.04.2005	M	SF	135	10	40	137	40	883	76	M	14	11	no	vertical	vertical with water	straight	no	0	0	0,00	yes
Helsinki	The Longines FEI World Cup Jumping - Helsinki round A	22.10.2017	CSI-W5*	4	1.40 - 1.60	450	170	13	06.04.2005	M	SF	135	10	40	137	40	883	76	M	15	12a	yes	spread	oxer	right	no	0	0	0,00	yes
Helsinki	The Longines FEI World Cup Jumping - Helsinki round A	22.10.2017	CSI-W5*	4	1.40 - 1.60	450	170	13	06.04.2005	M	SF	135	10	40	137	40	883	76	M	16	12b	yes	vertical	vertical	right	no	0	0	0,00	yes
Helsinki	The Longines FEI World Cup Jumping - Helsinki round A	22.10.2017	CSI-W5*	4	1.40 - 1.60	450	170	13	06.04.2005	M	SF	135	10	40	137	40	883	76	M	17	13	no	spread	oxer	right	no	0	0	0,00	yes
Helsinki	The Longines FEI World Cup Jumping - Helsinki round A	22.10.2017	CSI-W5*	4	1.40 - 1.60	450	53	12	01.05.2006	S	KWPN	132	2	41	122	28	897	18	F	1	1	no	vertical	vertical	right	no	0	0	93,45	no
Helsinki	The Longines FEI World Cup Jumping - Helsinki round A	22.10.2017	CSI-W5*	4	1.40 - 1.60	450	53	12	01.05.2006	S	KWPN	132	2	41	122	28	897	18	F	2	2	no	vertical	vertical	left	yes	4	0	93,45	no
Helsinki	The Longines FEI World Cup Jumping - Helsinki round A	22.10.2017	CSI-W5*	4	1.40 - 1.60	450	53	12	01.05.2006	S	KWPN	132	2	41	122	28	897	18	F	3	3	no	spread	oxer	left	no	0	0	93,45	no
Helsinki	The Longines FEI World Cup Jumping - Helsinki round A	22.10.2017	CSI-W5*	4	1.40 - 1.60	450	53	12	01.05.2006	S	KWPN	132	2	41	122	28	897	18	F	4	4a	yes	spread	oxer	left	no	0	0	93,45	no
Helsinki	The Longines FEI World Cup Jumping - Helsinki round A	22.10.2017	CSI-W5*	4	1.40 - 1.60	450	53	12	01.05.2006	S	KWPN	132	2	41	122	28	897	18	F	5	4b	yes	vertical	vertical	left	no	0	0	93,45	no
Helsinki	The Longines FEI World Cup Jumping - Helsinki round A	22.10.2017	CSI-W5*	4	1.40 - 1.60	450	53	12	01.05.2006	S	KWPN	132	2	41	122	28	897	18	F	6	4c	yes	spread	oxer	left	no	0	0	93,45	no
Helsinki	The Longines FEI World Cup Jumping - Helsinki round A	22.10.2017	CSI-W5*	4	1.40 - 1.60	450	53	12	01.05.2006	S	KWPN	132	2	41	122	28	897	18	F	7	5	no	spread	oxer	right	no	0	0	93,45	no
Helsinki	The Longines FEI World Cup Jumping - Helsinki round A	22.10.2017	CSI-W5*	4	1.40 - 1.60	450	53	12	01.05.2006	S	KWPN	132	2	41	122	28	897	18	F	8	6a	yes	vertical	vertical	right	no	0	0	93,45	no
Helsinki	The Longines FEI World Cup Jumping - Helsinki round A	22.10.2017	CSI-W5*	4	1.40 - 1.60	450	53	12	01.05.2006	S	KWPN	132	2	41	122	28	897	18	F	9	6b	yes	vertical	vertical	right	no	0	0	93,45	no
Helsinki	The Longines FEI World Cup Jumping - Helsinki round A	22.10.2017	CSI-W5*	4	1.40 - 1.60	450	53	12	01.05.2006	S	KWPN	132	2	41	122	28	897	18	F	10	7	no	spread	oxer	left	no	0	0	93,45	no
Helsinki	The Longines FEI World Cup Jumping - Helsinki round A	22.10.2017	CSI-W5*	4	1.40 - 1.60	450	53	12	01.05.2006	S	KWPN	132	2	41	122	28	897	18	F	11	8	no	vertical	wall	left	no	0	0	93,45	no
Helsinki	The Longines FEI World Cup Jumping - Helsinki round A	22.10.2017	CSI-W5*	4	1.40 - 1.60	450	53	12	01.05.2006	S	KWPN	132	2	41	122	28	897	18	F	12	9	no	spread	oxer	straight	no	0	0	93,45	no
Helsinki	The Longines FEI World Cup Jumping - Helsinki round A	22.10.2017	CSI-W5*	4	1.40 - 1.60	450	53	12	01.05.2006	S	KWPN	132	2	41	122	28	897	18	F	13	10	no	spread	triple bar	left	no	0	0	93,45	no
Helsinki	The Longines FEI World Cup Jumping - Helsinki round A	22.10.2017	CSI-W5*	4	1.40 - 1.60	450	53	12	01.05.2006	S	KWPN	132	2	41	122	28	897	18	F	14	11	no	vertical	vertical with water	straight	no	0	0	93,45	no
Helsinki	The Longines FEI World Cup Jumping - Helsinki round A	22.10.2017	CSI-W5*	4	1.40 - 1.60	450	53	12	01.05.2006	S	KWPN	132	2	41	122	28	897	18	F	15	12a	yes	spread	oxer	right	no	0	0	93,45	no
Helsinki	The Longines FEI World Cup Jumping - Helsinki round A	22.10.2017	CSI-W5*	4	1.40 - 1.60	450	53	12	01.05.2006	S	KWPN	132	2	41	122	28	897	18	F	16	12b	yes	vertical	vertical	right	no	0	0	93,45	no
Helsinki	The Longines FEI World Cup Jumping - Helsinki round A	22.10.2017	CSI-W5*	4	1.40 - 1.60	450	53	12	01.05.2006	S	KWPN	132	2	41	122	28	897	18	F	17	13	no	spread	oxer	right	no	0	4	93,45	no
Helsinki	The Longines FEI World Cup Jumping - Helsinki round A	22.10.2017	CSI-W5*	4	1.40 - 1.60	450	216	13	21.06.2005	G	SBS	196	3	42	542	26	1197	49	F	1	1	no	vertical	vertical	right	no	0	0	77,22	no
Helsinki	The Longines FEI World Cup Jumping - Helsinki round A	22.10.2017	CSI-W5*	4	1.40 - 1.60	450	216	13	21.06.2005	G	SBS	196	3	42	542	26	1197	49	F	2	2	no	vertical	vertical	left	no	0	0	77,22	no
Helsinki	The Longines FEI World Cup Jumping - Helsinki round A	22.10.2017	CSI-W5*	4	1.40 - 1.60	450	216	13	21.06.2005	G	SBS	196	3	42	542	26	1197	49	F	3	3	no	spread	oxer	left	no	0	0	77,22	no
Helsinki	The Longines FEI World Cup Jumping - Helsinki round A	22.10.2017	CSI-W5*	4	1.40 - 1.60	450	216	13	21.06.2005	G	SBS	196	3	42	542	26	1197	49	F	4	4a	yes	spread	oxer	left	no	0	0	77,22	no
Helsinki	The Longines FEI World Cup Jumping - Helsinki round A	22.10.2017	CSI-W5*	4	1.40 - 1.60	450	216	13	21.06.2005	G	SBS	196	3	42	542	26	1197	49	F	5	4b	yes	vertical	vertical	left	no	0	0	77,22	no
Helsinki	The Longines FEI World Cup Jumping - Helsinki round A	22.10.2017	CSI-W5*	4	1.40 - 1.60	450	216	13	21.06.2005	G	SBS	196	3	42	542	26	1197	49	F	6	4c	yes	spread	oxer	left	no	0	0	77,22	no
Helsinki	The Longines FEI World Cup Jumping - Helsinki round A	22.10.2017	CSI-W5*	4	1.40 - 1.60	450	216	13	21.06.2005	G	SBS	196	3	42	542	26	1197	49	F	7	5	no	spread	oxer	right	no	0	0	77,22	no
Helsinki	The Longines FEI World Cup Jumping - Helsinki round A	22.10.2017	CSI-W5*	4	1.40 - 1.60	450	216	13	21.06.2005	G	SBS	196	3	42	542	26	1197	49	F	8	6a	yes	vertical	vertical	right	no	0	0	77,22	no
Helsinki	The Longines FEI World Cup Jumping - Helsinki round A	22.10.2017	CSI-W5*	4	1.40 - 1.60	450	216	13	21.06.2005	G	SBS	196	3	42	542	26	1197	49	F	9	6b	yes	vertical	vertical	right	no	0	0	77,22	no
Helsinki	The Longines FEI World Cup Jumping - Helsinki round A	22.10.2017	CSI-W5*	4	1.40 - 1.60	450	216	13	21.06.2005	G	SBS	196	3	42	542	26	1197	49	F	10	7	no	spread	oxer	left	no	0	0	77,22	no
Helsinki	The Longines FEI World Cup Jumping - Helsinki round A	22.10.2017	CSI-W5*	4	1.40 - 1.60	450	216	13	21.06.2005	G	SBS	196	3	42	542	26	1197	49	F	11	8	no	vertical	wall	left	no	0	0	77,22	no
Helsinki	The Longines FEI World Cup Jumping - Helsinki round A	22.10.2017	CSI-W5*	4	1.40 - 1.60	450	216	13	21.06.2005	G	SBS	196	3	42	542	26	1197	49	F	12	9	no	spread	oxer	straight	yes	4	0	77,22	no
Helsinki	The Longines FEI World Cup Jumping - Helsinki round A	22.10.2017	CSI-W5*	4	1.40 - 1.60	450	216	13	21.06.2005	G	SBS	196	3	42	542	26	1197	49	F	13	10	no	spread	triple bar	left	no	0	0	77,22	no
Helsinki	The Longines FEI World Cup Jumping - Helsinki round A	22.10.2017	CSI-W5*	4	1.40 - 1.60	450	216	13	21.06.2005	G	SBS	196	3	42	542	26	1197	49	F	14	11	no	vertical	vertical with water	straight	yes	4	0	77,22	no
Helsinki	The Longines FEI World Cup Jumping - Helsinki round A	22.10.2017	CSI-W5*	4	1.40 - 1.60	450	216	13	21.06.2005	G	SBS	196	3	42	542	26	1197	49	F	15	12a	yes	spread	oxer	right	no	0	0	77,22	no
Helsinki	The Longines FEI World Cup Jumping - Helsinki round A	22.10.2017	CSI-W5*	4	1.40 - 1.60	450	216	13	21.06.2005	G	SBS	196	3	42	542	26	1197	49	F	16	12b	yes	vertical	vertical	right	no	0	0	77,22	no
Helsinki	The Longines FEI World Cup Jumping - Helsinki round A	22.10.2017	CSI-W5*	4	1.40 - 1.60	450	216	13	21.06.2005	G	SBS	196	3	42	542	26	1197	49	F	17	13	no	spread	oxer	right	no	0	0	77,22	no
Helsinki	The Longines FEI World Cup Jumping - Helsinki round A	22.10.2017	CSI-W5*	4	1.40 - 1.60	450	208	10	18.05.2008	G	SF	126	1	43	134	48	1808	64	M	1	1	no	vertical	vertical	right	no	0	0	77,1	no
Helsinki	The Longines FEI World Cup Jumping - Helsinki round A	22.10.2017	CSI-W5*	4	1.40 - 1.60	450	208	10	18.05.2008	G	SF	126	1	43	134	48	1808	64	M	2	2	no	vertical	vertical	left	no	0	0	77,1	no
Helsinki	The Longines FEI World Cup Jumping - Helsinki round A	22.10.2017	CSI-W5*	4	1.40 - 1.60	450	208	10	18.05.2008	G	SF	126	1	43	134	48	1808	64	M	3	3	no	spread	oxer	left	no	0	0	77,1	no
Helsinki	The Longines FEI World Cup Jumping - Helsinki round A	22.10.2017	CSI-W5*	4	1.40 - 1.60	450	208	10	18.05.2008	G	SF	126	1	43	134	48	1808	64	M	4	4a	yes	spread	oxer	left	no	0	0	77,1	no
Helsinki	The Longines FEI World Cup Jumping - Helsinki round A	22.10.2017	CSI-W5*	4	1.40 - 1.60	450	208	10	18.05.2008	G	SF	126	1	43	134	48	1808	64	M	5	4b	yes	vertical	vertical	left	no	0	0	77,1	no
Helsinki	The Longines FEI World Cup Jumping - Helsinki round A	22.10.2017	CSI-W5*	4	1.40 - 1.60	450	208	10	18.05.2008	G	SF	126	1	43	134	48	1808	64	M	6	4c	yes	spread	oxer	left	no	0	0	77,1	no
Helsinki	The Longines FEI World Cup Jumping - Helsinki round A	22.10.2017	CSI-W5*	4	1.40 - 1.60	450	208	10	18.05.2008	G	SF	126	1	43	134	48	1808	64	M	7	5	no	spread	oxer	right	no	0	0	77,1	no
Helsinki	The Longines FEI World Cup Jumping - Helsinki round A	22.10.2017	CSI-W5*	4	1.40 - 1.60	450	208	10	18.05.2008	G	SF	126	1	43	134	48	1808	64	M	8	6a	yes	vertical	vertical	right	no	0	0	77,1	no
Helsinki	The Longines FEI World Cup Jumping - Helsinki round A	22.10.2017	CSI-W5*	4	1.40 - 1.60	450	208	10	18.05.2008	G	SF	126	1	43	134	48	1808	64	M	9	6b	yes	vertical	vertical	right	no	0	0	77,1	no
Helsinki	The Longines FEI World Cup Jumping - Helsinki round A	22.10.2017	CSI-W5*	4	1.40 - 1.60	450	208	10	18.05.2008	G	SF	126	1	43	134	48	1808	64	M	10	7	no	spread	oxer	left	no	0	0	77,1	no
Helsinki	The Longines FEI World Cup Jumping - Helsinki round A	22.10.2017	CSI-W5*	4	1.40 - 1.60	450	208	10	18.05.2008	G	SF	126	1	43	134	48	1808	64	M	11	8	no	vertical	wall	left	no	0	0	77,1	no
Helsinki	The Longines FEI World Cup Jumping - Helsinki round A	22.10.2017	CSI-W5*	4	1.40 - 1.60	450	208	10	18.05.2008	G	SF	126	1	43	134	48	1808	64	M	12	9	no	spread	oxer	straight	no	0	0	77,1	no
Helsinki	The Longines FEI World Cup Jumping - Helsinki round A	22.10.2017	CSI-W5*	4	1.40 - 1.60	450	208	10	18.05.2008	G	SF	126	1	43	134	48	1808	64	M	13	10	no	spread	triple bar	left	no	0	0	77,1	no
Helsinki	The Longines FEI World Cup Jumping - Helsinki round A	22.10.2017	CSI-W5*	4	1.40 - 1.60	450	208	10	18.05.2008	G	SF	126	1	43	134	48	1808	64	M	14	11	no	vertical	vertical with water	straight	no	0	0	77,1	no
Helsinki	The Longines FEI World Cup Jumping - Helsinki round A	22.10.2017	CSI-W5*	4	1.40 - 1.60	450	208	10	18.05.2008	G	SF	126	1	43	134	48	1808	64	M	15	12a	yes	spread	oxer	right	no	0	0	77,1	no
Helsinki	The Longines FEI World Cup Jumping - Helsinki round A	22.10.2017	CSI-W5*	4	1.40 - 1.60	450	208	10	18.05.2008	G	SF	126	1	43	134	48	1808	64	M	16	12b	yes	vertical	vertical	right	no	0	0	77,1	no
Helsinki	The Longines FEI World Cup Jumping - Helsinki round A	22.10.2017	CSI-W5*	4	1.40 - 1.60	450	208	10	18.05.2008	G	SF	126	1	43	134	48	1808	64	M	17	13	no	spread	oxer	right	no	0	0	77,1	no
Helsinki	The Longines FEI World Cup Jumping - Helsinki round B	22.10.2017	CSI-W5*	4	1.40 - 1.60	270	208	10	18.05.2008	G	SF	126	1	43	134	48	1808	64	M	1	1	no	spread	oxer	right	no	0	0	42,71	no
Helsinki	The Longines FEI World Cup Jumping - Helsinki round B	22.10.2017	CSI-W5*	4	1.40 - 1.60	270	208	10	18.05.2008	G	SF	126	1	43	134	48	1808	64	M	2	2	no	vertical	vertical	right	yes	4	0	42,71	no
Helsinki	The Longines FEI World Cup Jumping - Helsinki round B	22.10.2017	CSI-W5*	4	1.40 - 1.60	270	208	10	18.05.2008	G	SF	126	1	43	134	48	1808	64	M	3	3	no	spread	oxer	left	no	0	0	42,71	no
Helsinki	The Longines FEI World Cup Jumping - Helsinki round B	22.10.2017	CSI-W5*	4	1.40 - 1.60	270	208	10	18.05.2008	G	SF	126	1	43	134	48	1808	64	M	4	4a	yes	vertical	vertical	left	yes	4	0	42,71	no
Helsinki	The Longines FEI World Cup Jumping - Helsinki round B	22.10.2017	CSI-W5*	4	1.40 - 1.60	270	208	10	18.05.2008	G	SF	126	1	43	134	48	1808	64	M	5	4b	yes	spread	oxer	left	no	0	0	42,71	no
Helsinki	The Longines FEI World Cup Jumping - Helsinki round B	22.10.2017	CSI-W5*	4	1.40 - 1.60	270	208	10	18.05.2008	G	SF	126	1	43	134	48	1808	64	M	6	5	no	spread	oxer	right	no	0	0	42,71	no
Helsinki	The Longines FEI World Cup Jumping - Helsinki round B	22.10.2017	CSI-W5*	4	1.40 - 1.60	270	208	10	18.05.2008	G	SF	126	1	43	134	48	1808	64	M	7	6	no	vertical	vertical	right	no	0	0	42,71	no
Helsinki	The Longines FEI World Cup Jumping - Helsinki round B	22.10.2017	CSI-W5*	4	1.40 - 1.60	270	208	10	18.05.2008	G	SF	126	1	43	134	48	1808	64	M	8	7	no	spread	oxer	left	no	0	0	42,71	no
Helsinki	The Longines FEI World Cup Jumping - Helsinki round A	22.10.2017	CSI-W5*	4	1.40 - 1.60	450	42	11	01.01.2007	S	ISH	143	5	44	135	22	458	12	M	1	1	no	vertical	vertical	right	no	0	0	75,03	no
Helsinki	The Longines FEI World Cup Jumping - Helsinki round A	22.10.2017	CSI-W5*	4	1.40 - 1.60	450	42	11	01.01.2007	S	ISH	143	5	44	135	22	458	12	M	2	2	no	vertical	vertical	left	no	0	0	75,03	no
Helsinki	The Longines FEI World Cup Jumping - Helsinki round A	22.10.2017	CSI-W5*	4	1.40 - 1.60	450	42	11	01.01.2007	S	ISH	143	5	44	135	22	458	12	M	3	3	no	spread	oxer	left	no	0	0	75,03	no
Helsinki	The Longines FEI World Cup Jumping - Helsinki round A	22.10.2017	CSI-W5*	4	1.40 - 1.60	450	42	11	01.01.2007	S	ISH	143	5	44	135	22	458	12	M	4	4a	yes	spread	oxer	left	no	0	0	75,03	no
Helsinki	The Longines FEI World Cup Jumping - Helsinki round A	22.10.2017	CSI-W5*	4	1.40 - 1.60	450	42	11	01.01.2007	S	ISH	143	5	44	135	22	458	12	M	5	4b	yes	vertical	vertical	left	no	0	0	75,03	no
Helsinki	The Longines FEI World Cup Jumping - Helsinki round A	22.10.2017	CSI-W5*	4	1.40 - 1.60	450	42	11	01.01.2007	S	ISH	143	5	44	135	22	458	12	M	6	4c	yes	spread	oxer	left	no	0	0	75,03	no
Helsinki	The Longines FEI World Cup Jumping - Helsinki round A	22.10.2017	CSI-W5*	4	1.40 - 1.60	450	42	11	01.01.2007	S	ISH	143	5	44	135	22	458	12	M	7	5	no	spread	oxer	right	no	0	0	75,03	no
Helsinki	The Longines FEI World Cup Jumping - Helsinki round A	22.10.2017	CSI-W5*	4	1.40 - 1.60	450	42	11	01.01.2007	S	ISH	143	5	44	135	22	458	12	M	8	6a	yes	vertical	vertical	right	no	0	0	75,03	no
Helsinki	The Longines FEI World Cup Jumping - Helsinki round A	22.10.2017	CSI-W5*	4	1.40 - 1.60	450	42	11	01.01.2007	S	ISH	143	5	44	135	22	458	12	M	9	6b	yes	vertical	vertical	right	no	0	0	75,03	no
Helsinki	The Longines FEI World Cup Jumping - Helsinki round A	22.10.2017	CSI-W5*	4	1.40 - 1.60	450	42	11	01.01.2007	S	ISH	143	5	44	135	22	458	12	M	10	7	no	spread	oxer	left	no	0	0	75,03	no
Helsinki	The Longines FEI World Cup Jumping - Helsinki round A	22.10.2017	CSI-W5*	4	1.40 - 1.60	450	42	11	01.01.2007	S	ISH	143	5	44	135	22	458	12	M	11	8	no	vertical	wall	left	no	0	0	75,03	no
Helsinki	The Longines FEI World Cup Jumping - Helsinki round A	22.10.2017	CSI-W5*	4	1.40 - 1.60	450	42	11	01.01.2007	S	ISH	143	5	44	135	22	458	12	M	12	9	no	spread	oxer	straight	no	0	0	75,03	no
Helsinki	The Longines FEI World Cup Jumping - Helsinki round A	22.10.2017	CSI-W5*	4	1.40 - 1.60	450	42	11	01.01.2007	S	ISH	143	5	44	135	22	458	12	M	13	10	no	spread	triple bar	left	no	0	0	75,03	no
Helsinki	The Longines FEI World Cup Jumping - Helsinki round A	22.10.2017	CSI-W5*	4	1.40 - 1.60	450	42	11	01.01.2007	S	ISH	143	5	44	135	22	458	12	M	14	11	no	vertical	vertical with water	straight	no	0	0	75,03	no
Helsinki	The Longines FEI World Cup Jumping - Helsinki round A	22.10.2017	CSI-W5*	4	1.40 - 1.60	450	42	11	01.01.2007	S	ISH	143	5	44	135	22	458	12	M	15	12a	yes	spread	oxer	right	no	0	0	75,03	no
Helsinki	The Longines FEI World Cup Jumping - Helsinki round A	22.10.2017	CSI-W5*	4	1.40 - 1.60	450	42	11	01.01.2007	S	ISH	143	5	44	135	22	458	12	M	16	12b	yes	vertical	vertical	right	no	0	0	75,03	no
Helsinki	The Longines FEI World Cup Jumping - Helsinki round A	22.10.2017	CSI-W5*	4	1.40 - 1.60	450	42	11	01.01.2007	S	ISH	143	5	44	135	22	458	12	M	17	13	no	spread	oxer	right	no	0	0	75,03	no
Helsinki	The Longines FEI World Cup Jumping - Helsinki round B	22.10.2017	CSI-W5*	4	1.40 - 1.60	270	42	11	01.01.2007	S	ISH	143	5	44	135	22	458	12	M	1	1	no	spread	oxer	right	no	0	0	40,16	no
Helsinki	The Longines FEI World Cup Jumping - Helsinki round B	22.10.2017	CSI-W5*	4	1.40 - 1.60	270	42	11	01.01.2007	S	ISH	143	5	44	135	22	458	12	M	2	2	no	vertical	vertical	right	yes	4	0	40,16	no
Helsinki	The Longines FEI World Cup Jumping - Helsinki round B	22.10.2017	CSI-W5*	4	1.40 - 1.60	270	42	11	01.01.2007	S	ISH	143	5	44	135	22	458	12	M	3	3	no	spread	oxer	left	no	0	0	40,16	no
Helsinki	The Longines FEI World Cup Jumping - Helsinki round B	22.10.2017	CSI-W5*	4	1.40 - 1.60	270	42	11	01.01.2007	S	ISH	143	5	44	135	22	458	12	M	4	4a	yes	vertical	vertical	left	yes	4	0	40,16	no
Helsinki	The Longines FEI World Cup Jumping - Helsinki round B	22.10.2017	CSI-W5*	4	1.40 - 1.60	270	42	11	01.01.2007	S	ISH	143	5	44	135	22	458	12	M	5	4b	yes	spread	oxer	left	no	0	0	40,16	no
Helsinki	The Longines FEI World Cup Jumping - Helsinki round B	22.10.2017	CSI-W5*	4	1.40 - 1.60	270	42	11	01.01.2007	S	ISH	143	5	44	135	22	458	12	M	6	5	no	spread	oxer	right	no	0	0	40,16	no
Helsinki	The Longines FEI World Cup Jumping - Helsinki round B	22.10.2017	CSI-W5*	4	1.40 - 1.60	270	42	11	01.01.2007	S	ISH	143	5	44	135	22	458	12	M	7	6	no	vertical	vertical	right	no	0	0	40,16	no
Helsinki	The Longines FEI World Cup Jumping - Helsinki round B	22.10.2017	CSI-W5*	4	1.40 - 1.60	270	42	11	01.01.2007	S	ISH	143	5	44	135	22	458	12	M	8	7	no	spread	oxer	left	no	0	0	40,16	no
Helsinki	The Longines FEI World Cup Jumping - Helsinki round A	22.10.2017	CSI-W5*	4	1.40 - 1.60	450	143	12	25.06.2006	G	Bezplemenné	93	4	45	335	31	964	37	F	1	1	no	vertical	vertical	right	no	0	0	74,86	no
Helsinki	The Longines FEI World Cup Jumping - Helsinki round A	22.10.2017	CSI-W5*	4	1.40 - 1.60	450	143	12	25.06.2006	G	Bezplemenné	93	4	45	335	31	964	37	F	2	2	no	vertical	vertical	left	no	0	0	74,86	no
Helsinki	The Longines FEI World Cup Jumping - Helsinki round A	22.10.2017	CSI-W5*	4	1.40 - 1.60	450	143	12	25.06.2006	G	Bezplemenné	93	4	45	335	31	964	37	F	3	3	no	spread	oxer	left	no	0	0	74,86	no
Helsinki	The Longines FEI World Cup Jumping - Helsinki round A	22.10.2017	CSI-W5*	4	1.40 - 1.60	450	143	12	25.06.2006	G	Bezplemenné	93	4	45	335	31	964	37	F	4	4a	yes	spread	oxer	left	no	0	0	74,86	no
Helsinki	The Longines FEI World Cup Jumping - Helsinki round A	22.10.2017	CSI-W5*	4	1.40 - 1.60	450	143	12	25.06.2006	G	Bezplemenné	93	4	45	335	31	964	37	F	5	4b	yes	vertical	vertical	left	no	0	0	74,86	no
Helsinki	The Longines FEI World Cup Jumping - Helsinki round A	22.10.2017	CSI-W5*	4	1.40 - 1.60	450	143	12	25.06.2006	G	Bezplemenné	93	4	45	335	31	964	37	F	6	4c	yes	spread	oxer	left	no	0	0	74,86	no
Helsinki	The Longines FEI World Cup Jumping - Helsinki round A	22.10.2017	CSI-W5*	4	1.40 - 1.60	450	143	12	25.06.2006	G	Bezplemenné	93	4	45	335	31	964	37	F	7	5	no	spread	oxer	right	no	0	0	74,86	no
Helsinki	The Longines FEI World Cup Jumping - Helsinki round A	22.10.2017	CSI-W5*	4	1.40 - 1.60	450	143	12	25.06.2006	G	Bezplemenné	93	4	45	335	31	964	37	F	8	6a	yes	vertical	vertical	right	no	0	0	74,86	no
Helsinki	The Longines FEI World Cup Jumping - Helsinki round A	22.10.2017	CSI-W5*	4	1.40 - 1.60	450	143	12	25.06.2006	G	Bezplemenné	93	4	45	335	31	964	37	F	9	6b	yes	vertical	vertical	right	no	0	0	74,86	no
Helsinki	The Longines FEI World Cup Jumping - Helsinki round A	22.10.2017	CSI-W5*	4	1.40 - 1.60	450	143	12	25.06.2006	G	Bezplemenné	93	4	45	335	31	964	37	F	10	7	no	spread	oxer	left	no	0	0	74,86	no
Helsinki	The Longines FEI World Cup Jumping - Helsinki round A	22.10.2017	CSI-W5*	4	1.40 - 1.60	450	143	12	25.06.2006	G	Bezplemenné	93	4	45	335	31	964	37	F	11	8	no	vertical	wall	left	no	0	0	74,86	no
Helsinki	The Longines FEI World Cup Jumping - Helsinki round A	22.10.2017	CSI-W5*	4	1.40 - 1.60	450	143	12	25.06.2006	G	Bezplemenné	93	4	45	335	31	964	37	F	12	9	no	spread	oxer	straight	yes	4	0	74,86	no
Helsinki	The Longines FEI World Cup Jumping - Helsinki round A	22.10.2017	CSI-W5*	4	1.40 - 1.60	450	143	12	25.06.2006	G	Bezplemenné	93	4	45	335	31	964	37	F	13	10	no	spread	triple bar	left	no	0	0	74,86	no
Helsinki	The Longines FEI World Cup Jumping - Helsinki round A	22.10.2017	CSI-W5*	4	1.40 - 1.60	450	143	12	25.06.2006	G	Bezplemenné	93	4	45	335	31	964	37	F	14	11	no	vertical	vertical with water	straight	no	0	0	74,86	no
Helsinki	The Longines FEI World Cup Jumping - Helsinki round A	22.10.2017	CSI-W5*	4	1.40 - 1.60	450	143	12	25.06.2006	G	Bezplemenné	93	4	45	335	31	964	37	F	15	12a	yes	spread	oxer	right	no	0	0	74,86	no
Helsinki	The Longines FEI World Cup Jumping - Helsinki round A	22.10.2017	CSI-W5*	4	1.40 - 1.60	450	143	12	25.06.2006	G	Bezplemenné	93	4	45	335	31	964	37	F	16	12b	yes	vertical	vertical	right	no	0	0	74,86	no
Helsinki	The Longines FEI World Cup Jumping - Helsinki round A	22.10.2017	CSI-W5*	4	1.40 - 1.60	450	143	12	25.06.2006	G	Bezplemenné	93	4	45	335	31	964	37	F	17	13	no	spread	oxer	right	no	0	0	74,86	no
Helsinki	The Longines FEI World Cup Jumping - Helsinki round A	22.10.2017	CSI-W5*	4	1.40 - 1.60	450	124	11	31.05.2007	G	KWPN	5	0	46	148	26	1060	109	M	1	1	no	vertical	vertical	right	no	0	0	74,58	no
Helsinki	The Longines FEI World Cup Jumping - Helsinki round A	22.10.2017	CSI-W5*	4	1.40 - 1.60	450	124	11	31.05.2007	G	KWPN	5	0	46	148	26	1060	109	M	2	2	no	vertical	vertical	left	no	0	0	74,58	no
Helsinki	The Longines FEI World Cup Jumping - Helsinki round A	22.10.2017	CSI-W5*	4	1.40 - 1.60	450	124	11	31.05.2007	G	KWPN	5	0	46	148	26	1060	109	M	3	3	no	spread	oxer	left	no	0	0	74,58	no
Helsinki	The Longines FEI World Cup Jumping - Helsinki round A	22.10.2017	CSI-W5*	4	1.40 - 1.60	450	124	11	31.05.2007	G	KWPN	5	0	46	148	26	1060	109	M	4	4a	yes	spread	oxer	left	no	0	0	74,58	no
Helsinki	The Longines FEI World Cup Jumping - Helsinki round A	22.10.2017	CSI-W5*	4	1.40 - 1.60	450	124	11	31.05.2007	G	KWPN	5	0	46	148	26	1060	109	M	5	4b	yes	vertical	vertical	left	no	0	0	74,58	no
Helsinki	The Longines FEI World Cup Jumping - Helsinki round A	22.10.2017	CSI-W5*	4	1.40 - 1.60	450	124	11	31.05.2007	G	KWPN	5	0	46	148	26	1060	109	M	6	4c	yes	spread	oxer	left	no	0	0	74,58	no
Helsinki	The Longines FEI World Cup Jumping - Helsinki round A	22.10.2017	CSI-W5*	4	1.40 - 1.60	450	124	11	31.05.2007	G	KWPN	5	0	46	148	26	1060	109	M	7	5	no	spread	oxer	right	no	0	0	74,58	no
Helsinki	The Longines FEI World Cup Jumping - Helsinki round A	22.10.2017	CSI-W5*	4	1.40 - 1.60	450	124	11	31.05.2007	G	KWPN	5	0	46	148	26	1060	109	M	8	6a	yes	vertical	vertical	right	no	0	0	74,58	no
Helsinki	The Longines FEI World Cup Jumping - Helsinki round A	22.10.2017	CSI-W5*	4	1.40 - 1.60	450	124	11	31.05.2007	G	KWPN	5	0	46	148	26	1060	109	M	9	6b	yes	vertical	vertical	right	no	0	0	74,58	no
Helsinki	The Longines FEI World Cup Jumping - Helsinki round A	22.10.2017	CSI-W5*	4	1.40 - 1.60	450	124	11	31.05.2007	G	KWPN	5	0	46	148	26	1060	109	M	10	7	no	spread	oxer	left	no	0	0	74,58	no
Helsinki	The Longines FEI World Cup Jumping - Helsinki round A	22.10.2017	CSI-W5*	4	1.40 - 1.60	450	124	11	31.05.2007	G	KWPN	5	0	46	148	26	1060	109	M	11	8	no	vertical	wall	left	no	0	0	74,58	no
Helsinki	The Longines FEI World Cup Jumping - Helsinki round A	22.10.2017	CSI-W5*	4	1.40 - 1.60	450	124	11	31.05.2007	G	KWPN	5	0	46	148	26	1060	109	M	12	9	no	spread	oxer	straight	no	0	0	74,58	no
Helsinki	The Longines FEI World Cup Jumping - Helsinki round A	22.10.2017	CSI-W5*	4	1.40 - 1.60	450	124	11	31.05.2007	G	KWPN	5	0	46	148	26	1060	109	M	13	10	no	spread	triple bar	left	no	0	0	74,58	no
Helsinki	The Longines FEI World Cup Jumping - Helsinki round A	22.10.2017	CSI-W5*	4	1.40 - 1.60	450	124	11	31.05.2007	G	KWPN	5	0	46	148	26	1060	109	M	14	11	no	vertical	vertical with water	straight	no	0	0	74,58	no
Helsinki	The Longines FEI World Cup Jumping - Helsinki round A	22.10.2017	CSI-W5*	4	1.40 - 1.60	450	124	11	31.05.2007	G	KWPN	5	0	46	148	26	1060	109	M	15	12a	yes	spread	oxer	right	no	0	0	74,58	no
Helsinki	The Longines FEI World Cup Jumping - Helsinki round A	22.10.2017	CSI-W5*	4	1.40 - 1.60	450	124	11	31.05.2007	G	KWPN	5	0	46	148	26	1060	109	M	16	12b	yes	vertical	vertical	right	yes	4	0	74,58	no
Helsinki	The Longines FEI World Cup Jumping - Helsinki round A	22.10.2017	CSI-W5*	4	1.40 - 1.60	450	124	11	31.05.2007	G	KWPN	5	0	46	148	26	1060	109	M	17	13	no	spread	oxer	right	yes	4	0	74,58	no
Helsinki	The Longines FEI World Cup Jumping - Helsinki round A	22.10.2017	CSI-W5*	4	1.40 - 1.60	450	140	11	19.03.2007	S	HANN	162	14	47	44	44	1933	114	M	1	1	no	vertical	vertical	right	no	0	0	74,4	no
Helsinki	The Longines FEI World Cup Jumping - Helsinki round A	22.10.2017	CSI-W5*	4	1.40 - 1.60	450	140	11	19.03.2007	S	HANN	162	14	47	44	44	1933	114	M	2	2	no	vertical	vertical	left	no	0	0	74,4	no
Helsinki	The Longines FEI World Cup Jumping - Helsinki round A	22.10.2017	CSI-W5*	4	1.40 - 1.60	450	140	11	19.03.2007	S	HANN	162	14	47	44	44	1933	114	M	3	3	no	spread	oxer	left	no	0	0	74,4	no
Helsinki	The Longines FEI World Cup Jumping - Helsinki round A	22.10.2017	CSI-W5*	4	1.40 - 1.60	450	140	11	19.03.2007	S	HANN	162	14	47	44	44	1933	114	M	4	4a	yes	spread	oxer	left	no	0	0	74,4	no
Helsinki	The Longines FEI World Cup Jumping - Helsinki round A	22.10.2017	CSI-W5*	4	1.40 - 1.60	450	140	11	19.03.2007	S	HANN	162	14	47	44	44	1933	114	M	5	4b	yes	vertical	vertical	left	no	0	0	74,4	no
Helsinki	The Longines FEI World Cup Jumping - Helsinki round A	22.10.2017	CSI-W5*	4	1.40 - 1.60	450	140	11	19.03.2007	S	HANN	162	14	47	44	44	1933	114	M	6	4c	yes	spread	oxer	left	no	0	0	74,4	no
Helsinki	The Longines FEI World Cup Jumping - Helsinki round A	22.10.2017	CSI-W5*	4	1.40 - 1.60	450	140	11	19.03.2007	S	HANN	162	14	47	44	44	1933	114	M	7	5	no	spread	oxer	right	no	0	0	74,4	no
Helsinki	The Longines FEI World Cup Jumping - Helsinki round A	22.10.2017	CSI-W5*	4	1.40 - 1.60	450	140	11	19.03.2007	S	HANN	162	14	47	44	44	1933	114	M	8	6a	yes	vertical	vertical	right	no	0	0	74,4	no
Helsinki	The Longines FEI World Cup Jumping - Helsinki round A	22.10.2017	CSI-W5*	4	1.40 - 1.60	450	140	11	19.03.2007	S	HANN	162	14	47	44	44	1933	114	M	9	6b	yes	vertical	vertical	right	no	0	0	74,4	no
Helsinki	The Longines FEI World Cup Jumping - Helsinki round A	22.10.2017	CSI-W5*	4	1.40 - 1.60	450	140	11	19.03.2007	S	HANN	162	14	47	44	44	1933	114	M	10	7	no	spread	oxer	left	no	0	0	74,4	no
Helsinki	The Longines FEI World Cup Jumping - Helsinki round A	22.10.2017	CSI-W5*	4	1.40 - 1.60	450	140	11	19.03.2007	S	HANN	162	14	47	44	44	1933	114	M	11	8	no	vertical	wall	left	no	0	0	74,4	no
Helsinki	The Longines FEI World Cup Jumping - Helsinki round A	22.10.2017	CSI-W5*	4	1.40 - 1.60	450	140	11	19.03.2007	S	HANN	162	14	47	44	44	1933	114	M	12	9	no	spread	oxer	straight	no	0	0	74,4	no
Helsinki	The Longines FEI World Cup Jumping - Helsinki round A	22.10.2017	CSI-W5*	4	1.40 - 1.60	450	140	11	19.03.2007	S	HANN	162	14	47	44	44	1933	114	M	13	10	no	spread	triple bar	left	no	0	0	74,4	no
Helsinki	The Longines FEI World Cup Jumping - Helsinki round A	22.10.2017	CSI-W5*	4	1.40 - 1.60	450	140	11	19.03.2007	S	HANN	162	14	47	44	44	1933	114	M	14	11	no	vertical	vertical with water	straight	no	0	0	74,4	no
Helsinki	The Longines FEI World Cup Jumping - Helsinki round A	22.10.2017	CSI-W5*	4	1.40 - 1.60	450	140	11	19.03.2007	S	HANN	162	14	47	44	44	1933	114	M	15	12a	yes	spread	oxer	right	yes	4	0	74,4	no
Helsinki	The Longines FEI World Cup Jumping - Helsinki round A	22.10.2017	CSI-W5*	4	1.40 - 1.60	450	140	11	19.03.2007	S	HANN	162	14	47	44	44	1933	114	M	16	12b	yes	vertical	vertical	right	no	0	0	74,4	no
Helsinki	The Longines FEI World Cup Jumping - Helsinki round A	22.10.2017	CSI-W5*	4	1.40 - 1.60	450	140	11	19.03.2007	S	HANN	162	14	47	44	44	1933	114	M	17	13	no	spread	oxer	right	yes	4	0	74,4	no
Helsinki	The Longines FEI World Cup Jumping - Helsinki round A	22.10.2017	CSI-W5*	4	1.40 - 1.60	450	11	12	13.03.2006	M	KWPN	211	7	48	40	39	1040	33	M	1	1	no	vertical	vertical	right	no	0	0	73,54	no
Helsinki	The Longines FEI World Cup Jumping - Helsinki round A	22.10.2017	CSI-W5*	4	1.40 - 1.60	450	11	12	13.03.2006	M	KWPN	211	7	48	40	39	1040	33	M	2	2	no	vertical	vertical	left	no	0	0	73,54	no
Helsinki	The Longines FEI World Cup Jumping - Helsinki round A	22.10.2017	CSI-W5*	4	1.40 - 1.60	450	11	12	13.03.2006	M	KWPN	211	7	48	40	39	1040	33	M	3	3	no	spread	oxer	left	no	0	0	73,54	no
Helsinki	The Longines FEI World Cup Jumping - Helsinki round A	22.10.2017	CSI-W5*	4	1.40 - 1.60	450	11	12	13.03.2006	M	KWPN	211	7	48	40	39	1040	33	M	4	4a	yes	spread	oxer	left	no	0	0	73,54	no
Helsinki	The Longines FEI World Cup Jumping - Helsinki round A	22.10.2017	CSI-W5*	4	1.40 - 1.60	450	11	12	13.03.2006	M	KWPN	211	7	48	40	39	1040	33	M	5	4b	yes	vertical	vertical	left	yes	4	0	73,54	no
Helsinki	The Longines FEI World Cup Jumping - Helsinki round A	22.10.2017	CSI-W5*	4	1.40 - 1.60	450	11	12	13.03.2006	M	KWPN	211	7	48	40	39	1040	33	M	6	4c	yes	spread	oxer	left	no	0	0	73,54	no
Helsinki	The Longines FEI World Cup Jumping - Helsinki round A	22.10.2017	CSI-W5*	4	1.40 - 1.60	450	11	12	13.03.2006	M	KWPN	211	7	48	40	39	1040	33	M	7	5	no	spread	oxer	right	no	0	0	73,54	no

Helsinki	The Longines FEI World Cup Jumping - Helsinki round A	22.10.2017	CSI-W5*	4	1.40 - 1.60	450	11	12	13.03.2006	M	KWPN	211	7	48	40	39	1040	33	M	8	6a	yes	vertical	vertical	right	no	0	0	73,54	no
Helsinki	The Longines FEI World Cup Jumping - Helsinki round A	22.10.2017	CSI-W5*	4	1.40 - 1.60	450	11	12	13.03.2006	M	KWPN	211	7	48	40	39	1040	33	M	9	6b	yes	vertical	vertical	right	no	0	0	73,54	no
Helsinki	The Longines FEI World Cup Jumping - Helsinki round A	22.10.2017	CSI-W5*	4	1.40 - 1.60	450	11	12	13.03.2006	M	KWPN	211	7	48	40	39	1040	33	M	10	7	no	spread	oxer	left	yes	4	0	73,54	no
Helsinki	The Longines FEI World Cup Jumping - Helsinki round A	22.10.2017	CSI-W5*	4	1.40 - 1.60	450	11	12	13.03.2006	M	KWPN	211	7	48	40	39	1040	33	M	11	8	no	vertical	wall	left	no	0	0	73,54	no
Helsinki	The Longines FEI World Cup Jumping - Helsinki round A	22.10.2017	CSI-W5*	4	1.40 - 1.60	450	11	12	13.03.2006	M	KWPN	211	7	48	40	39	1040	33	M	12	9	no	spread	oxer	straight	yes	4	0	73,54	no
Helsinki	The Longines FEI World Cup Jumping - Helsinki round A	22.10.2017	CSI-W5*	4	1.40 - 1.60	450	11	12	13.03.2006	M	KWPN	211	7	48	40	39	1040	33	M	13	10	no	spread	triple bar	left	no	0	0	73,54	no
Helsinki	The Longines FEI World Cup Jumping - Helsinki round A	22.10.2017	CSI-W5*	4	1.40 - 1.60	450	11	12	13.03.2006	M	KWPN	211	7	48	40	39	1040	33	M	14	11	no	vertical	vertical with water	straight	no	0	0	73,54	no
Helsinki	The Longines FEI World Cup Jumping - Helsinki round A	22.10.2017	CSI-W5*	4	1.40 - 1.60	450	11	12	13.03.2006	M	KWPN	211	7	48	40	39	1040	33	M	15	12a	yes	spread	oxer	right	no	0	0	73,54	no
Helsinki	The Longines FEI World Cup Jumping - Helsinki round A	22.10.2017	CSI-W5*	4	1.40 - 1.60	450	11	12	13.03.2006	M	KWPN	211	7	48	40	39	1040	33	M	16	12b	yes	vertical	vertical	right	no	0	0	73,54	no
Helsinki	The Longines FEI World Cup Jumping - Helsinki round A	22.10.2017	CSI-W5*	4	1.40 - 1.60	450	11	12	13.03.2006	M	KWPN	211	7	48	40	39	1040	33	M	17	13	no	spread	oxer	right	no	0	0	73,54	no
Helsinki	The Longines FEI World Cup Jumping - Helsinki round A	22.10.2017	CSI-W5*	4	1.40 - 1.60	450	25	12	25.05.2006	G	WESTF	85	0	51	220	30	1567	79	M	1	1	no	vertical	vertical	right	no	0	0	73,46	no
Helsinki	The Longines FEI World Cup Jumping - Helsinki round A	22.10.2017	CSI-W5*	4	1.40 - 1.60	450	25	12	25.05.2006	G	WESTF	85	0	51	220	30	1567	79	M	2	2	no	vertical	vertical	left	no	0	0	73,46	no
Helsinki	The Longines FEI World Cup Jumping - Helsinki round A	22.10.2017	CSI-W5*	4	1.40 - 1.60	450	25	12	25.05.2006	G	WESTF	85	0	51	220	30	1567	79	M	3	3	no	spread	oxer	left	no	0	0	73,46	no
Helsinki	The Longines FEI World Cup Jumping - Helsinki round A	22.10.2017	CSI-W5*	4	1.40 - 1.60	450	25	12	25.05.2006	G	WESTF	85	0	51	220	30	1567	79	M	4	4a	yes	spread	oxer	left	no	0	0	73,46	no
Helsinki	The Longines FEI World Cup Jumping - Helsinki round A	22.10.2017	CSI-W5*	4	1.40 - 1.60	450	25	12	25.05.2006	G	WESTF	85	0	51	220	30	1567	79	M	5	4b	yes	vertical	vertical	left	no	0	0	73,46	no
Helsinki	The Longines FEI World Cup Jumping - Helsinki round A	22.10.2017	CSI-W5*	4	1.40 - 1.60	450	25	12	25.05.2006	G	WESTF	85	0	51	220	30	1567	79	M	6	4c	yes	spread	oxer	left	no	0	0	73,46	no
Helsinki	The Longines FEI World Cup Jumping - Helsinki round A	22.10.2017	CSI-W5*	4	1.40 - 1.60	450	25	12	25.05.2006	G	WESTF	85	0	51	220	30	1567	79	M	7	5	no	spread	oxer	right	no	0	0	73,46	no
Helsinki	The Longines FEI World Cup Jumping - Helsinki round A	22.10.2017	CSI-W5*	4	1.40 - 1.60	450	25	12	25.05.2006	G	WESTF	85	0	51	220	30	1567	79	M	8	6a	yes	vertical	vertical	right	no	0	0	73,46	no
Helsinki	The Longines FEI World Cup Jumping - Helsinki round A	22.10.2017	CSI-W5*	4	1.40 - 1.60	450	25	12	25.05.2006	G	WESTF	85	0	51	220	30	1567	79	M	9	6b	yes	vertical	vertical	right	no	0	0	73,46	no
Helsinki	The Longines FEI World Cup Jumping - Helsinki round A	22.10.2017	CSI-W5*	4	1.40 - 1.60	450	25	12	25.05.2006	G	WESTF	85	0	51	220	30	1567	79	M	10	7	no	spread	oxer	left	no	0	0	73,46	no
Helsinki	The Longines FEI World Cup Jumping - Helsinki round A	22.10.2017	CSI-W5*	4	1.40 - 1.60	450	25	12	25.05.2006	G	WESTF	85	0	51	220	30	1567	79	M	11	8	no	vertical	wall	left	no	0	0	73,46	no
Helsinki	The Longines FEI World Cup Jumping - Helsinki round A	22.10.2017	CSI-W5*	4	1.40 - 1.60	450	25	12	25.05.2006	G	WESTF	85	0	51	220	30	1567	79	M	12	9	no	spread	oxer	straight	yes	4	0	73,46	no
Helsinki	The Longines FEI World Cup Jumping - Helsinki round A	22.10.2017	CSI-W5*	4	1.40 - 1.60	450	25	12	25.05.2006	G	WESTF	85	0	51	220	30	1567	79	M	13	10	no	spread	triple bar	left	no	0	0	73,46	no
Helsinki	The Longines FEI World Cup Jumping - Helsinki round A	22.10.2017	CSI-W5*	4	1.40 - 1.60	450	25	12	25.05.2006	G	WESTF	85	0	51	220	30	1567	79	M	14	11	no	vertical	vertical with water	straight	no	0	0	73,46	no
Helsinki	The Longines FEI World Cup Jumping - Helsinki round A	22.10.2017	CSI-W5*	4	1.40 - 1.60	450	25	12	25.05.2006	G	WESTF	85	0	51	220	30	1567	79	M	15	12a	yes	spread	oxer	right	no	0	0	73,46	no
Helsinki	The Longines FEI World Cup Jumping - Helsinki round A	22.10.2017	CSI-W5*	4	1.40 - 1.60	450	25	12	25.05.2006	G	WESTF	85	0	51	220	30	1567	79	M	16	12b	yes	vertical	vertical	right	yes	4	0	73,46	no
Helsinki	The Longines FEI World Cup Jumping - Helsinki round A	22.10.2017	CSI-W5*	4	1.40 - 1.60	450	25	12	25.05.2006	G	WESTF	85	0	51	220	30	1567	79	M	17	13	no	spread	oxer	right	no	0	0	73,46	no
Helsinki	The Longines FEI World Cup Jumping - Helsinki round A	22.10.2017	CSI-W5*	4	1.40 - 1.60	450	36	13	30.05.2005	S	HOLST	223	29	52	52	32	2035	202	M	1	1	no	vertical	vertical	right	no	0	0	73,27	no
Helsinki	The Longines FEI World Cup Jumping - Helsinki round A	22.10.2017	CSI-W5*	4	1.40 - 1.60	450	36	13	30.05.2005	S	HOLST	223	29	52	52	32	2035	202	M	2	2	no	vertical	vertical	left	no	0	0	73,27	no
Helsinki	The Longines FEI World Cup Jumping - Helsinki round A	22.10.2017	CSI-W5*	4	1.40 - 1.60	450	36	13	30.05.2005	S	HOLST	223	29	52	52	32	2035	202	M	3	3	no	spread	oxer	left	no	0	0	73,27	no
Helsinki	The Longines FEI World Cup Jumping - Helsinki round A	22.10.2017	CSI-W5*	4	1.40 - 1.60	450	36	13	30.05.2005	S	HOLST	223	29	52	52	32	2035	202	M	4	4a	yes	spread	oxer	left	no	0	0	73,27	no
Helsinki	The Longines FEI World Cup Jumping - Helsinki round A	22.10.2017	CSI-W5*	4	1.40 - 1.60	450	36	13	30.05.2005	S	HOLST	223	29	52	52	32	2035	202	M	5	4b	yes	vertical	vertical	left	no	0	0	73,27	no
Helsinki	The Longines FEI World Cup Jumping - Helsinki round A	22.10.2017	CSI-W5*	4	1.40 - 1.60	450	36	13	30.05.2005	S	HOLST	223	29	52	52	32	2035	202	M	6	4c	yes	spread	oxer	left	no	0	0	73,27	no
Helsinki	The Longines FEI World Cup Jumping - Helsinki round A	22.10.2017	CSI-W5*	4	1.40 - 1.60	450	36	13	30.05.2005	S	HOLST	223	29	52	52	32	2035	202	M	7	5	no	spread	oxer	right	no	0	0	73,27	no
Helsinki	The Longines FEI World Cup Jumping - Helsinki round A	22.10.2017	CSI-W5*	4	1.40 - 1.60	450	36	13	30.05.2005	S	HOLST	223	29	52	52	32	2035	202	M	8	6a	yes	vertical	vertical	right	no	0	0	73,27	no
Helsinki	The Longines FEI World Cup Jumping - Helsinki round A	22.10.2017	CSI-W5*	4	1.40 - 1.60	450	36	13	30.05.2005	S	HOLST	223	29	52	52	32	2035	202	M	9	6b	yes	vertical	vertical	right	no	0	0	73,27	no
Helsinki	The Longines FEI World Cup Jumping - Helsinki round A	22.10.2017	CSI-W5*	4	1.40 - 1.60	450	36	13	30.05.2005	S	HOLST	223	29	52	52	32	2035	202	M	10	7	no	spread	oxer	left	no	0	0	73,27	no
Helsinki	The Longines FEI World Cup Jumping - Helsinki round A	22.10.2017	CSI-W5*	4	1.40 - 1.60	450	36	13	30.05.2005	S	HOLST	223	29	52	52	32	2035	202	M	11	8	no	vertical	wall	left	no	0	0	73,27	no
Helsinki	The Longines FEI World Cup Jumping - Helsinki round A	22.10.2017	CSI-W5*	4	1.40 - 1.60	450	36	13	30.05.2005	S	HOLST	223	29	52	52	32	2035	202	M	12	9	no	spread	oxer	straight	no	0	0	73,27	no
Helsinki	The Longines FEI World Cup Jumping - Helsinki round A	22.10.2017	CSI-W5*	4	1.40 - 1.60	450	36	13	30.05.2005	S	HOLST	223	29	52	52	32	2035	202	M	13	10	no	spread	triple bar	left	no	0	0	73,27	no
Helsinki	The Longines FEI World Cup Jumping - Helsinki round A	22.10.2017	CSI-W5*	4	1.40 - 1.60	450	36	13	30.05.2005	S	HOLST	223	29	52	52	32	2035	202	M	14	11	no	vertical	vertical with water	straight	no	0	0	73,27	no
Helsinki	The Longines FEI World Cup Jumping - Helsinki round A	22.10.2017	CSI-W5*	4	1.40 - 1.60	450	36	13	30.05.2005	S	HOLST	223	29	52	52	32	2035	202	M	15	12a	yes	spread	oxer	right	no	0	0	73,27	no
Helsinki	The Longines FEI World Cup Jumping - Helsinki round A	22.10.2017	CSI-W5*	4	1.40 - 1.60	450	36	13	30.05.2005	S	HOLST	223	29	52	52	32	2035	202	M	16	12b	yes	vertical	vertical	right	no	0	0	73,27	no
Helsinki	The Longines FEI World Cup Jumping - Helsinki round A	22.10.2017	CSI-W5*	4	1.40 - 1.60	450	36	13	30.05.2005	S	HOLST	223	29	52	52	32	2035	202	M	17	13	no	spread	oxer	right	no	0	0	73,27	no
Helsinki	The Longines FEI World Cup Jumping - Helsinki round B	22.10.2017	CSI-W5*	4	1.40 - 1.60	270	36	13	30.05.2005	S	HOLST	223	29	52	52	32	2035	202	M	1	1	no	spread	oxer	right	no	0	0	38,83	no
Helsinki	The Longines FEI World Cup Jumping - Helsinki round B	22.10.2017	CSI-W5*	4	1.40 - 1.60	270	36	13	30.05.2005	S	HOLST	223	29	52	52	32	2035	202	M	2	2	no	vertical	vertical	right	no	0	0	38,83	no
Helsinki	The Longines FEI World Cup Jumping - Helsinki round B	22.10.2017	CSI-W5*	4	1.40 - 1.60	270	36	13	30.05.2005	S	HOLST	223	29	52	52	32	2035	202	M	3	3	no	spread	oxer	left	no	0	0	38,83	no
Helsinki	The Longines FEI World Cup Jumping - Helsinki round B	22.10.2017	CSI-W5*	4	1.40 - 1.60	270	36	13	30.05.2005	S	HOLST	223	29	52	52	32	2035	202	M	4	4a	yes	vertical	vertical	left	no	0	0	38,83	no
Helsinki	The Longines FEI World Cup Jumping - Helsinki round B	22.10.2017	CSI-W5*	4	1.40 - 1.60	270	36	13	30.05.2005	S	HOLST	223	29	52	52	32	2035	202	M	5	4b	yes	spread	oxer	left	no	0	0	38,83	no
Helsinki	The Longines FEI World Cup Jumping - Helsinki round B	22.10.2017	CSI-W5*	4	1.40 - 1.60	270	36	13	30.05.2005	S	HOLST	223	29	52	52	32	2035	202	M	6	5	no	spread	oxer	right	yes	4	0	38,83	no
Helsinki	The Longines FEI World Cup Jumping - Helsinki round B	22.10.2017	CSI-W5*	4	1.40 - 1.60	270	36	13	30.05.2005	S	HOLST	223	29	52	52	32	2035	202	M	7	6	no	vertical	vertical	right	no	0	0	38,83	no
Helsinki	The Longines FEI World Cup Jumping - Helsinki round B	22.10.2017	CSI-W5*	4	1.40 - 1.60	270	36	13	30.05.2005	S	HOLST	223	29	52	52	32	2035	202	M	8	7	no	spread	oxer	left	no	0	0	38,83	no
Helsinki	The Longines FEI World Cup Jumping - Helsinki round A	22.10.2017	CSI-W5*	4	1.40 - 1.60	450	10	11	07.05.2007	M	Bezplemenné	103	1	53	32	33	1848	55	M	1	1	no	vertical	vertical	right	no	0	0	72,51	no
Helsinki	The Longines FEI World Cup Jumping - Helsinki round A	22.10.2017	CSI-W5*	4	1.40 - 1.60	450	10	11	07.05.2007	M	Bezplemenné	103	1	53	32	33	1848	55	M	2	2	no	vertical	vertical	left	no	0	0	72,51	no
Helsinki	The Longines FEI World Cup Jumping - Helsinki round A	22.10.2017	CSI-W5*	4	1.40 - 1.60	450	10	11	07.05.2007	M	Bezplemenné	103	1	53	32	33	1848	55	M	3	3	no	spread	oxer	left	no	0	0	72,51	no
Helsinki	The Longines FEI World Cup Jumping - Helsinki round A	22.10.2017	CSI-W5*	4	1.40 - 1.60	450	10	11	07.05.2007	M	Bezplemenné	103	1	53	32	33	1848	55	M	4	4a	yes	spread	oxer	left	no	0	0	72,51	no
Helsinki	The Longines FEI World Cup Jumping - Helsinki round A	22.10.2017	CSI-W5*	4	1.40 - 1.60	450	10	11	07.05.2007	M	Bezplemenné	103	1	53	32	33	1848	55	M	5	4b	yes	vertical	vertical	left	no	0	0	72,51	no
Helsinki	The Longines FEI World Cup Jumping - Helsinki round A	22.10.2017	CSI-W5*	4	1.40 - 1.60	450	10	11	07.05.2007	M	Bezplemenné	103	1	53	32	33	1848	55	M	6	4c	yes	spread	oxer	left	no	0	0	72,51	no
Helsinki	The Longines FEI World Cup Jumping - Helsinki round A	22.10.2017	CSI-W5*	4	1.40 - 1.60	450	10	11	07.05.2007	M	Bezplemenné	103	1	53	32	33	1848	55	M	7	5	no	spread	oxer	right	no	0	0	72,51	no
Helsinki	The Longines FEI World Cup Jumping - Helsinki round A	22.10.2017	CSI-W5*	4	1.40 - 1.60	450	10	11	07.05.2007	M	Bezplemenné	103	1	53	32	33	1848	55	M	8	6a	yes	vertical	vertical	right	no	0	0	72,51	no
Helsinki	The Longines FEI World Cup Jumping - Helsinki round A	22.10.2017	CSI-W5*	4	1.40 - 1.60	450	10	11	07.05.2007	M	Bezplemenné	103	1	53	32	33	1848	55	M	9	6b	yes	vertical	vertical	right	no	0	0	72,51	no
Helsinki	The Longines FEI World Cup Jumping - Helsinki round A	22.10.2017	CSI-W5*	4	1.40 - 1.60	450	10	11	07.05.2007	M	Bezplemenné	103	1	53	32	33	1848	55	M	10	7	no	spread	oxer	left	no	0	0	72,51	no
Helsinki	The Longines FEI World Cup Jumping - Helsinki round A	22.10.2017	CSI-W5*	4	1.40 - 1.60	450	10	11	07.05.2007	M	Bezplemenné	103	1	53	32	33	1848	55	M	11	8	no	vertical	wall	left	no	0	0	72,51	no
Helsinki	The Longines FEI World Cup Jumping - Helsinki round A	22.10.2017	CSI-W5*	4	1.40 - 1.60	450	10	11	07.05.2007	M	Bezplemenné	103	1	53	32	33	1848	55	M	12	9	no	spread	oxer	straight	no	0	0	72,51	no
Helsinki	The Longines FEI World Cup Jumping - Helsinki round A	22.10.2017	CSI-W5*	4	1.40 - 1.60	450	10	11	07.05.2007	M	Bezplemenné	103	1	53	32	33	1848	55	M	13	10	no	spread	triple bar	left	no	0	0	72,51	no
Helsinki	The Longines FEI World Cup Jumping - Helsinki round A	22.10.2017	CSI-W5*	4	1.40 - 1.60	450	10	11	07.05.2007	M	Bezplemenné	103	1	53	32	33	1848	55	M	14	11	no	vertical	vertical with water	straight	no	0	0	72,51	no
Helsinki	The Longines FEI World Cup Jumping - Helsinki round A	22.10.2017	CSI-W5*	4	1.40 - 1.60	450	10	11	07.05.2007	M	Bezplemenné	103	1	53	32	33	1848	55	M	15	12a	yes	spread	oxer	right	no	0	0	72,51	no
Helsinki	The Longines FEI World Cup Jumping - Helsinki round A	22.10.2017	CSI-W5*	4	1.40 - 1.60	450	10	11	07.05.2007	M	Bezplemenné	103	1	53	32	33	1848	55	M	16	12b	yes	vertical	vertical	right	yes	4	0	72,51	no
Helsinki	The Longines FEI World Cup Jumping - Helsinki round A	22.10.2017	CSI-W5*	4	1.40 - 1.60	450	10	11	07.05.2007	M	Bezplemenné	103	1	53	32	33	1848	55	M	17	13	no	spread	oxer	right	no	0	0	72,51	no
Helsinki	The Longines FEI World Cup Jumping - Helsinki round A	22.10.2017	CSI-W5*	4	1.40 - 1.60	450	34	10	24.03.2008	G	WESTF	81	1	54	92	25	487	7	F	1	1	no	vertical	vertical	right	no	0	0	72,45	no
Helsinki	The Longines FEI World Cup Jumping - Helsinki round A	22.10.2017	CSI-W5*	4	1.40 - 1.60	450	34	10	24.03.2008	G	WESTF	81	1	54	92	25	487	7	F	2	2	no	vertical	vertical	left	no	0	0	72,45	no
Helsinki	The Longines FEI World Cup Jumping - Helsinki round A	22.10.2017	CSI-W5*	4	1.40 - 1.60	450	34	10	24.03.2008	G	WESTF	81	1	54	92	25	487	7	F	3	3	no	spread	oxer	left	no	0	0	72,45	no
Helsinki	The Longines FEI World Cup Jumping - Helsinki round A	22.10.2017	CSI-W5*	4	1.40 - 1.60	450	34	10	24.03.2008	G	WESTF	81	1	54	92	25	487	7	F	4	4a	yes	spread	oxer	left	yes	4	0	72,45	no
Helsinki	The Longines FEI World Cup Jumping - Helsinki round A	22.10.2017	CSI-W5*	4	1.40 - 1.60	450	34	10	24.03.2008	G	WESTF	81	1	54	92	25	487	7	F	5	4b	yes	vertical	vertical	left	no	0	0	72,45	no
Helsinki	The Longines FEI World Cup Jumping - Helsinki round A	22.10.2017	CSI-W5*	4	1.40 - 1.60	450	34	10	24.03.2008	G	WESTF	81	1	54	92	25	487	7	F	6	4c	yes	spread	oxer	left	no	0	0	72,45	no
Helsinki	The Longines FEI World Cup Jumping - Helsinki round A	22.10.2017	CSI-W5*	4	1.40 - 1.60	450	34	10	24.03.2008	G	WESTF	81	1	54	92	25	487	7	F	7	5	no	spread	oxer	right	no	0	0	72,45	no
Helsinki	The Longines FEI World Cup Jumping - Helsinki round A	22.10.2017	CSI-W5*	4	1.40 - 1.60	450	34	10	24.03.2008	G	WESTF	81	1	54	92	25	487	7	F	8	6a	yes	vertical	vertical	right	no	0	0	72,45	no
Helsinki	The Longines FEI World Cup Jumping - Helsinki round A	22.10.2017	CSI-W5*	4	1.40 - 1.60	450	34	10	24.03.2008	G	WESTF	81	1	54	92	25	487	7	F	9	6b	yes	vertical	vertical	right	no	0	0	72,45	no
Helsinki	The Longines FEI World Cup Jumping - Helsinki round A	22.10.2017	CSI-W5*	4	1.40 - 1.60	450	34	10	24.03.2008	G	WESTF	81	1	54	92	25	487	7	F	10	7	no	spread	oxer	left	no	0	0	72,45	no
Helsinki	The Longines FEI World Cup Jumping - Helsinki round A	22.10.2017	CSI-W5*	4	1.40 - 1.60	450	34	10	24.03.2008	G	WESTF	81	1	54	92	25	487	7	F	11	8	no	vertical	wall	left	no	0	0	72,45	no
Helsinki	The Longines FEI World Cup Jumping - Helsinki round A	22.10.2017	CSI-W5*	4	1.40 - 1.60	450	34	10	24.03.2008	G	WESTF	81	1	54	92	25	487	7	F	12	9	no	spread	oxer	straight	no	0	0	72,45	no
Helsinki	The Longines FEI World Cup Jumping - Helsinki round A	22.10.2017	CSI-W5*	4	1.40 - 1.60	450	34	10	24.03.2008	G	WESTF	81	1	54	92	25	487	7	F	13	10	no	spread	triple bar	left	no	0	0	72,45	no
Helsinki	The Longines FEI World Cup Jumping - Helsinki round A	22.10.2017	CSI-W5*	4	1.40 - 1.60	450	34	10	24.03.2008	G	WESTF	81	1	54	92	25	487	7	F	14	11	no	vertical	vertical with water	straight	no	0	0	72,45	no
Helsinki	The Longines FEI World Cup Jumping - Helsinki round A	22.10.2017	CSI-W5*	4	1.40 - 1.60	450	34	10	24.03.2008	G	WESTF	81	1	54	92	25	487	7	F	15	12a	yes	spread	oxer	right	no	0	0	72,45	no
Helsinki	The Longines FEI World Cup Jumping - Helsinki round A	22.10.2017	CSI-W5*	4	1.40 - 1.60	450	34	10	24.03.2008	G	WESTF	81	1	54	92	25	487	7	F	16	12b	yes	vertical	vertical	right	no	0	0	72,45	no
Helsinki	The Longines FEI World Cup Jumping - Helsinki round A	22.10.2017	CSI-W5*	4	1.40 - 1.60	450	34	10	24.03.2008	G	WESTF	81	1	54	92	25	487	7	F	17	13	no	spread	oxer	right	no	0	0	72,45	no
Helsinki	The Longines FEI World Cup Jumping - Helsinki round A	22.10.2017	CSI-W5*	4	1.40 - 1.60	450	152	12	20.05.2006	M	WESTF	94	7	55	6	37	1750	61	M	1	1	no	vertical	vertical	right	no	0	0	72,01	no
Helsinki	The Longines FEI World Cup Jumping - Helsinki round A	22.10.2017	CSI-W5*	4	1.40 - 1.60	450	152	12	20.05.2006	M	WESTF	94	7	55	6	37	1750	61	M	2	2	no	vertical	vertical	left	no	0	0	72,01	no
Helsinki	The Longines FEI World Cup Jumping - Helsinki round A	22.10.2017	CSI-W5*	4	1.40 - 1.60	450	152	12	20.05.2006	M	WESTF	94	7	55	6	37	1750	61	M	3	3	no	spread	oxer	left	no	0	0	72,01	no
Helsinki	The Longines FEI World Cup Jumping - Helsinki round A	22.10.2017	CSI-W5*	4	1.40 - 1.60	450	152	12	20.05.2006	M	WESTF	94	7	55	6	37	1750	61	M	4	4a	yes	spread	oxer	left	no	0	0	72,01	no
Helsinki	The Longines FEI World Cup Jumping - Helsinki round A	22.10.2017	CSI-W5*	4	1.40 - 1.60	450	152	12	20.05.2006	M	WESTF	94	7	55	6	37	1750	61	M	5	4b	yes	vertical	vertical	left	no	0	0	72,01	no
Helsinki	The Longines FEI World Cup Jumping - Helsinki round A	22.10.2017	CSI-W5*	4	1.40 - 1.60	450	152	12	20.05.2006	M	WESTF	94	7	55	6	37	1750	61	M	6	4c	yes	spread	oxer	left	no	0	0	72,01	no
Helsinki	The Longines FEI World Cup Jumping - Helsinki round A	22.10.2017	CSI-W5*	4	1.40 - 1.60	450	152	12	20.05.2006	M	WESTF	94	7	55	6	37	1750	61	M	7	5	no	spread	oxer	right	no	0	0	72,01	no
Helsinki	The Longines FEI World Cup Jumping - Helsinki round A	22.10.2017	CSI-W5*	4	1.40 - 1.60	450	152	12	20.05.2006	M	WESTF	94	7	55	6	37	1750	61	M	8	6a	yes	vertical	vertical	right	no	0	0	72,01	no
Helsinki	The Longines FEI World Cup Jumping - Helsinki round A	22.10.2017	CSI-W5*	4	1.40 - 1.60	450	152	12	20.05.2006	M	WESTF	94	7	55	6	37	1750	61	M	9	6b	yes	vertical	vertical	right	no	0	0	72,01	no
Helsinki	The Longines FEI World Cup Jumping - Helsinki round A	22.10.2017	CSI-W5*	4	1.40 - 1.60	450	152	12	20.05.2006	M	WESTF	94	7	55	6	37	1750	61	M	10	7	no	spread	oxer	left	no	0	0	72,01	no
Helsinki	The Longines FEI World Cup Jumping - Helsinki round A	22.10.2017	CSI-W5*	4	1.40 - 1.60	450	152	12	20.05.2006	M	WESTF	94	7	55	6	37	1750	61	M	11	8	no	vertical	wall	left	no	0	0	72,01	no
Helsinki	The Longines FEI World Cup Jumping - Helsinki round A	22.10.2017	CSI-W5*	4	1.40 - 1.60	450	152	12	20.05.2006	M	WESTF	94	7	55	6	37	1750	61	M	12	9	no	spread	oxer	straight	no	0	0	72,01	no
Helsinki	The Longines FEI World Cup Jumping - Helsinki round A	22.10.2017	CSI-W5*	4	1.40 - 1.60	450	152	12	20.05.2006	M	WESTF	94	7	55	6	37	1750	61	M	13	10	no	spread	triple bar	left	no	0	0	72,01	no
Helsinki	The Longines FEI World Cup Jumping - Helsinki round A	22.10.2017	CSI-W5*	4	1.40 - 1.60	450	152	12	20.05.2006	M	WESTF	94	7	55	6	37	1750	61	M	14	11	no	vertical	vertical with water	straight	no	0	0	72,01	no
Helsinki	The Longines FEI World Cup Jumping - Helsinki round A	22.10.2017	CSI-W5*	4	1.40 - 1.60	450	152	12	20.05.2006	M	WESTF	94	7	55	6	37	1750	61	M	15	12a	yes	spread	oxer	right	no	0	0	72,01	no
Helsinki	The Longines FEI World Cup Jumping - Helsinki round A	22.10.2017	CSI-W5*	4	1.40 - 1.60	450	152	12	20.05.2006	M	WESTF	94	7	55	6	37	1750	61	M	16	12b	yes	vertical	vertical	right	no	0	0	72,01	no
Helsinki	The Longines FEI World Cup Jumping - Helsinki round A	22.10.2017	CSI-W5*	4	1.40 - 1.60	450	152	12	20.05.2006	M	WESTF	94	7	55	6	37	1750	61	M	17	13	no	spread	oxer	right	no	0	0	72,01	no
Helsinki	The Longines FEI World Cup Jumping - Helsinki round B	22.10.2017	CSI-W5*	4	1.40 - 1.60	270	152	12	20.05.2006	M	WESTF	94	7	55	6	37	1750	61	M	1	1	no	spread	oxer	right	no	0	0	38,41	no
Helsinki	The Longines FEI World Cup Jumping - Helsinki round B	22.10.2017	CSI-W5*	4	1.40 - 1.60	270	152	12	20.05.2006	M	WESTF	94	7	55	6	37	1750	61	M	2	2	no	vertical	vertical	right	no	0	0	38,41	no
Helsinki	The Longines FEI World Cup Jumping - Helsinki round B	22.10.2017	CSI-W5*	4	1.40 - 1.60	270	152	12	20.05.2006	M	WESTF	94	7	55	6	37	1750	61	M	3	3	no	spread	oxer	left	no	0	0	38,41	no
Helsinki	The Longines FEI World Cup Jumping - Helsinki round B	22.10.2017	CSI-W5*	4	1.40 - 1.60	270	152	12	20.05.2006	M	WESTF	94	7	55	6	37	1750	61	M	4	4a	yes	vertical	vertical	left	no	0	0	38,41	no
Helsinki	The Longines FEI World Cup Jumping - Helsinki round B	22.10.2017	CSI-W5*	4	1.40 - 1.60	270	152	12	20.05.2006	M	WESTF	94	7	55	6	37	1750	61	M	5	4b	yes	spread	oxer	left	no	0	0	38,41	no
Helsinki	The Longines FEI World Cup Jumping - Helsinki round B	22.10.2017	CSI-W5*	4	1.40 - 1.60	270	152	12	20.05.2006	M	WESTF	94	7	55	6	37	1750	61	M	6	5	no	spread	oxer	right	no	0	0	38,41	no
Helsinki	The Longines FEI World Cup Jumping - Helsinki round B	22.10.2017	CSI-W5*	4	1.40 - 1.60	270	152	12	20.05.2006	M	WESTF	94	7	55	6	37	1750	61	M	7	6	no	vertical	vertical	right	no	0	0	38,41	no
Helsinki	The Longines FEI World Cup Jumping - Helsinki round B	22.10.2017	CSI-W5*	4	1.40 - 1.60	270	152	12	20.05.2006	M	WESTF	94	7	55	6	37	1750	61	M	8	7	no	spread	oxer	left	no	0	0	38,41	no
Verona	The Longines FEI World Cup Jumping - Verona round A	29.10.2017	CSI-W5*	4	1.40 - 1.60	465	155	10	23.02.2008	S	PZHK	130	6	1	19	38	2803	179	M	1	1	no	vertical	vertical	left	no	0	0	73,13	no
Verona	The Longines FEI World Cup Jumping - Verona round A	29.10.2017	CSI-W5*	4	1.40 - 1.60	465	155	10	23.02.2008	S	PZHK	130	6	1	19	38	2803	179	M	2	2	no	spread	oxer	left	no	0	0	73,13	no
Verona	The Longines FEI World Cup Jumping - Verona round A	29.10.2017	CSI-W5*	4	1.40 - 1.60	465	155	10	23.02.2008	S	PZHK	130	6	1	19	38	2803	179	M	3	3	no	vertical	vertical	right	no	0	0	73,13	no
Verona	The Longines FEI World Cup Jumping - Verona round A	29.10.2017	CSI-W5*	4	1.40 - 1.60	465	155	10	23.02.2008	S	PZHK	130	6	1	19	38	2803	179	M	4	4	no	spread	oxer	right	no	0	0	73,13	no
Verona	The Longines FEI World Cup Jumping - Verona round A	29.10.2017	CSI-W5*	4	1.40 - 1.60	465	155	10	23.02.2008	S	PZHK	130	6	1	19	38	2803	179	M	5	5	no	vertical	vertical with water	straight	no	0	0	73,13	no
Verona	The Longines FEI World Cup Jumping - Verona round A	29.10.2017	CSI-W5*	4	1.40 - 1.60	465	155	10	23.02.2008	S	PZHK	130	6	1	19	38	2803	179	M	6	6	no	spread	oxer with water	left	yes	4	0	73,13	no
Verona	The Longines FEI World Cup Jumping - Verona round A	29.10.2017	CSI-W5*	4	1.40 - 1.60	465	155	10	23.02.2008	S	PZHK	130	6	1	19	38	2803	179	M	7	7a	yes	spread	oxer	straight	no	0	0	73,13	no
Verona	The Longines FEI World Cup Jumping - Verona round A	29.10.2017	CSI-W5*	4	1.40 - 1.60	465	155	10	23.02.2008	S	PZHK	130	6	1	19	38	2803	179	M	8	7b	yes	vertical	vertical	straight	no	0	0	73,13	no
Verona	The Longines FEI World Cup Jumping - Verona round A	29.10.2017	CSI-W5*	4	1.40 - 1.60	465	155	10	23.02.2008	S	PZHK	130	6	1	19	38	2803	179	M	9	8	no	vertical	vertical	left	yes	4	0	73,13	no
Verona	The Longines FEI World Cup Jumping - Verona round A	29.10.2017	CSI-W5*	4	1.40 - 1.60	465	155	10	23.02.2008	S	PZHK	130	6	1	19	38	2803	179	M	10	9	no	spread	oxer	left	yes	4	0	73,13	no
Verona	The Longines FEI World Cup Jumping - Verona round A	29.10.2017	CSI-W5*	4	1.40 - 1.60	465	155	10	23.02.2008	S	PZHK	130	6	1	19	38	2803	179	M	11	10	no	vertical	vertical	right	no	0	0	73,13	no
Verona	The Longines FEI World Cup Jumping - Verona round A	29.10.2017	CSI-W5*	4	1.40 - 1.60	465	155	10	23.02.2008	S	PZHK	130	6	1	19	38	2803	179	M	12	11a	yes	vertical	vertical	right	no	0	0	73,13	no
Verona	The Longines FEI World Cup Jumping - Verona round A	29.10.2017	CSI-W5*	4	1.40 - 1.60	465	155	10	23.02.2008	S	PZHK	130	6	1	19	38	2803	179	M	13	11b	yes	spread	oxer	right	no	0	0	73,13	no
Verona	The Longines FEI World Cup Jumping - Verona round A	29.10.2017	CSI-W5*	4	1.40 - 1.60	465	155	10	23.02.2008	S	PZHK	130	6	1	19	38	2803	179	M	14	11c	yes	spread	oxer	right	no	0	0	73,13	no
Verona	The Longines FEI World Cup Jumping - Verona round A	29.10.2017	CSI-W5*	4	1.40 - 1.60	465	155	10	23.02.2008	S	PZHK	130	6	1	19	38	2803	179	M	15	12	no	vertical	vertical	left	yes	4	0	73,13	no
Verona	The Longines FEI World Cup Jumping - Verona round A	29.10.2017	CSI-W5*	4	1.40 - 1.60	465	155	10	23.02.2008	S	PZHK	130	6	1	19	38	2803	179	M	16	13	no	spread	oxer	straight	no	0	0	73,13	no
Verona	The Longines FEI World Cup Jumping - Verona round A	29.10.2017	CSI-W5*	4	1.40 - 1.60	465	107	11	25.02.2007	S	BWP	196	13	2	7	31	2168	92	M	1	1	no	vertical	vertical	left	no	0	0	77,48	no
Verona	The Longines FEI World Cup Jumping - Verona round A	29.10.2017	CSI-W5*	4	1.40 - 1.60	465	107	11	25.02.2007	S	BWP	196	13	2	7	31	2168	92	M	2	2	no	spread	oxer	left	no	0	0	77,48	no
Verona	The Longines FEI World Cup Jumping - Verona round A	29.10.2017	CSI-W5*	4	1.40 - 1.60	465	107	11	25.02.2007	S	BWP	196	13	2	7	31	2168	92	M	3	3	no	vertical	vertical	right	no	0	0	77,48	no
Verona	The Longines FEI World Cup Jumping - Verona round A	29.10.2017	CSI-W5*	4	1.40 - 1.60	465	107	11	25.02.2007	S	BWP	196	13	2	7	31	2168	92	M	4	4	no	spread	oxer	right	no	0	0	77,48	no
Verona	The Longines FEI World Cup Jumping - Verona round A	29.10.2017	CSI-W5*	4	1.40 - 1.60	465	107	11	25.02.2007	S	BWP	196	13	2	7	31	2168	92	M	5	5	no	vertical	vertical with water	straight	no	0	0	77,48	no
Verona	The Longines FEI World Cup Jumping - Verona round A	29.10.2017	CSI-W5*	4	1.40 - 1.60	465	107	11	25.02.2007	S	BWP	196	13	2	7	31	2168	92	M	6	6	no	spread	oxer with water	left	no	0	0	77,48	no
Verona	The Longines FEI World Cup Jumping - Verona round A	29.10.2017	CSI-W5*	4	1.40 - 1.60	465	107	11	25.02.2007	S	BWP	196	13	2	7	31	2168	92	M	7	7a	yes	spread	oxer	straight	no	0	0	77,48	no
Verona	The Longines FEI World Cup Jumping - Verona round A	29.10.2017	CSI-W5*	4	1.40 - 1.60	465	107	11	25.02.2007	S	BWP	196	13	2	7	31	2168	92	M	8	7b	yes	vertical	vertical	straight	no	0	0	77,48	no
Verona	The Longines FEI World Cup Jumping - Verona round A	29.10.2017	CSI-W5*	4	1.40 - 1.60	465	107	11	25.02.2007	S	BWP	196	13	2	7	31	2168	92	M	9	8	no	vertical	vertical	left	no	0	0	77,48	no
Verona	The Longines FEI World Cup Jumping - Verona round A	29.10.2017	CSI-W5*	4	1.40 - 1.60	465	107	11	25.02.2007	S	BWP	196	13	2	7	31	2168	92	M	10	9	no	spread	oxer	left	no	0	0	77,48	no
Verona	The Longines FEI World Cup Jumping - Verona round A	29.10.2017	CSI-W5*	4	1.40 - 1.60	465	107	11	25.02.2007	S	BWP	196	13	2	7	31	2168	92	M	11	10	no	vertical	vertical	right	no	0	0	77,48	no
Verona	The Longines FEI World Cup Jumping - Verona round A	29.10.2017	CSI-W5*	4	1.40 - 1.60	465	107	11	25.02.2007	S	BWP	196	13	2	7	31	2168	92	M	12	11a	yes	vertical	vertical	right	no	0	0	77,48	no
Verona	The Longines FEI World Cup Jumping - Verona round A	29.10.2017	CSI-W5*	4	1.40 - 1.60	465	107	11	25.02.2007	S	BWP	196	13	2	7	31	2168	92	M	13	11b	yes	spread	oxer	right	no	0	0	77,48	no
Verona	The Longines FEI World Cup Jumping - Verona round A	29.10.2017	CSI-W5*	4	1.40 - 1.60	465	107	11	25.02.2007	S	BWP	196	13	2	7	31	2168	92	M	14	11c	yes	spread	oxer	right	no	0	0	77,48	no
Verona	The Longines FEI World Cup Jumping - Verona round A	29.10.2017	CSI-W5*	4	1.40 - 1.60	465	107	11	25.02.2007	S	BWP	196	13	2	7	31	2168	92	M	15	12	no	vertical	vertical	left	no	0	0	77,48	no
Verona	The Longines FEI World Cup Jumping - Verona round A	29.10.2017	CSI-W5*	4	1.40 - 1.60	465	107	11	25.02.2007	S	BWP	196	13	2	7	31	2168	92	M	16	13	no	spread	oxer	straight	no	0	0	77,48	no
Verona	The Longines FEI World Cup Jumping - Verona round B	29.10.2017	CSI-W5*	4	1.40 - 1.60	290	107	11	25.02.2007	S	BWP	196	13	2	7	31	2168	92	M	1	1	no	vertical	vertical	right	no	0	0	38,00	no
Verona	The Longines FEI World Cup Jumping - Verona round B	29.10.2017	CSI-W5*	4	1.40 - 1.60	290	107	11	25.02.2007	S	BWP	196	13	2	7	31	2168	92	M	2	2	no	spread	oxer	right	no	0	0	38,00	no
Verona	The Longines FEI World Cup Jumping - Verona round B	29.10.2017	CSI-W5*	4	1.40 - 1.60	290	107	11	25.02.2007	S	BWP	196	13	2	7	31	2168	92	M	3	4a	yes	vertical	vertical with water	straight	no	0	0	38,00	no
Verona	The Longines FEI World Cup Jumping - Verona round B	29.10.2017	CSI-W5*	4	1.40 - 1.60	290	107	11	25.02.2007	S	BWP	196	13	2	7	31	2168	92	M	4	4b	yes	spread	oxer	straight	no	0	0	38,00	no
Verona	The Longines FEI World Cup Jumping - Verona round B	29.10.2017	CSI-W5*	4	1.40 - 1.60	290	107	11	25.02.2007	S	BWP	196	13	2	7	31	2168	92	M	5	5	no	vertical	vertical	left	no	0	0	38,00	no
Verona	The Longines FEI World Cup Jumping - Verona round B	29.10.2017	CSI-W5*	4	1.40 - 1.60	290	107	11	25.02.2007	S	BWP	196	13	2	7	31	2168	92	M	6	6	no	spread	oxer	left	no	0	0	38,00	no
Verona	The Longines FEI World Cup Jumping - Verona round B	29.10.2017	CSI-W5*	4	1.40 - 1.60	290	107	11	25.02.2007	S	BWP	196	13	2	7	31	2168	92	M	7	7	no	vertical	vertical	right	no	0	0	38,00	no
Verona	The Longines FEI World Cup Jumping - Verona round B	29.10.2017	CSI-W5*	4	1.40 - 1.60	290	107	11	25.02.2007	S	BWP	196	13	2	7	31	2168	92	M	8	7	no	vertical	vertical	right	no	0	0	38,00	no
Verona	The Longines FEI World Cup Jumping - Verona round A	29.10.2017	CSI-W5*	4	1.40 - 1.60	465	8	13	15.07.2005	G	KWPN	241	7	5	15	32	2092	63	M	1	1	no	vertical	vertical	left	no	0	0	76,09	no
Verona	The Longines FEI World Cup Jumping - Verona round A	29.10.2017	CSI-W5*	4	1.40 - 1.60	465	8	13	15.07.2005	G	KWPN	241	7	5	15	32	2092	63	M	2	2	no	spread	oxer	left	no	0	0	76,09	no
Verona	The Longines FEI World Cup Jumping - Verona round A	29.10.2017	CSI-W5*	4	1.40 - 1.60	465	8	13	15.07.2005	G	KWPN	241	7	5	15	32	2092	63	M	3	3	no	vertical	vertical	right	no	0	0	76,09	no
Verona	The Longines FEI World Cup Jumping - Verona round A	29.10.2017	CSI-W5*	4	1.40 - 1.60	465	8	13	15.07.2005	G	KWPN	241	7	5	15	32	2092	63	M	4	4	no	spread	oxer	right	no	0	0	76,09	no
Verona	The Longines FEI World Cup Jumping - Verona round A	29.10.2017	CSI-W5*	4	1.40 - 1.60	465	8	13	15.07.2005	G	KWPN	241	7	5	15	32	2092	63	M	5	5	no	vertical	vertical with water	straight	no	0	0	76,09	no
Verona	The Longines FEI World Cup Jumping - Verona round A	29.10.2017	CSI-W5*	4	1.40 - 1.60	465	8	13	15.07.2005	G	KWPN	241	7	5	15	32	2092	63	M	6	6	no	spread	oxer with water	left	no	0	0	76,09	no
Verona	The Longines FEI World Cup Jumping - Verona round A	29.10.2017	CSI-W5*	4	1.40 - 1.60	465	8	13	15.07.2005	G	KWPN	241	7	5	15	32	2092	63	M	7	7a	yes	spread	oxer	straight	no	0	0	76,09	no
Verona	The Longines FEI World Cup Jumping - Verona round A	29.10.2017	CSI-W5*	4	1.40 - 1.60	465	8	13	15.07.2005	G	KWPN	241	7	5	15	32	2092	63	M	8	7b	yes	vertical	vertical	straight	no	0	0	76,09	no
Verona	The Longines FEI World Cup Jumping - Verona round A	29.10.2017	CSI-W5*	4	1.40 - 1.60	465	8	13	15.07.2005	G	KWPN	241	7	5	15	32	2092	63	M	9	8	no	vertical	vertical	left	no	0	0	76,09	no
Verona	The Longines FEI World Cup Jumping - Verona round A	29.10.2017	CSI-W5*	4	1.40 - 1.60	465	8	13	15.07.2005	G	KWPN	241	7	5	15	32	2092	63	M	10	9	no	spread	oxer	left	no	0	0	76,09	no
Verona	The Longines FEI World Cup Jumping - Verona round A	29.10.2017	CSI-W5*	4	1.40 - 1.60	465	8	13	15.07.2005	G	KWPN	241	7	5	15	32	2092	63	M	11	10	no	vertical	vertical	right	no	0	0	76,09	no
Verona	The Longines FEI World Cup Jumping - Verona round A	29.10.2017	CSI-W5*	4	1.40 - 1.60	465	8	13	15.07.2005	G	KWPN	241	7	5	15	32	2092	63	M	12	11a	yes	vertical	vertical	right	no	0	0	76,09	no
Verona	The Longines FEI World Cup Jumping - Verona round A	29.10.2017	CSI-W5*	4	1.40 - 1.60	465	8	13	15.07.2005	G	KWPN	241	7	5	15	32	2092	63	M	13	11b	yes	spread	oxer	right	no	0	0	76,09	no
Verona	The Longines FEI World Cup Jumping - Verona round A	29.10.2017	CSI-W5*	4	1.40 - 1.60	465	8	13	15.07.2005	G	KWPN	241	7	5	15	32	2092	63	M	14	11c	yes	spread	oxer	right	no	0	0	76,09	no
Verona	The Longines FEI World Cup Jumping - Verona round A	29.10.2017	CSI-W5*	4	1.40 - 1.60	465	8	13	15.07.2005	G	KWPN	241	7	5	15	32	2092	63	M	15	12	no	vertical	vertical	left	no	0	0	76,09	no
Verona	The Longines FEI World Cup Jumping - Verona round A	29.10.2017	CSI-W5*	4	1.40 - 1.60	465	8	13	15.07.2005	G	KWPN	241	7	5	15	32	2092	63	M	16	13	no	spread	oxer	straight	no	0	0	76,09	no
Verona	The Longines FEI World Cup Jumping - Verona round B	29.10.2017	CSI-W5*	4	1.40 - 1.60	290	8	13	15.07.2005	G	KWPN	241	7	5	15	32	2092	63	M	1	1	no	vertical	vertical	right	no	0	0	37,03	no
Verona	The Longines FEI World Cup Jumping - Verona round B	29.10.2017	CSI-W5*	4	1.40 - 1.60	290	8	13	15.07.2005	G	KWPN	241	7	5	15	32	2092	63	M	2	2	no	spread	oxer	right	no	0	0	37,03	no
Verona	The Longines FEI World Cup Jumping - Verona round B	29.10.2017	CSI-W5*	4	1.40 - 1.60	290	8	13	15.07.2005	G	KWPN	241	7	5	15	32	2092	63	M	3	4a	yes	vertical	vertical with water	straight	no	0	0	37,03	no
Verona	The Longines FEI World Cup Jumping - Verona round B	29.10.2017	CSI-W5*	4	1.40 - 1.60	290	8	13	15.07.2005	G	KWPN	241	7	5	15	32	2092	63	M	4	4b	yes	spread	oxer	straight	no	0	0	37,03	no
Verona	The Longines FEI World Cup Jumping - Verona round B	29.10.2017	CSI-W5*	4	1.40 - 1.60	290	8	13	15.07.2005	G	KWPN	241	7	5	15	32	2092	63	M	5	5	no	vertical	vertical	left	no	0	0	37,03	no
Verona	The Longines FEI World Cup Jumping - Verona round B	29.10.2017	CSI-W5*	4	1.40 - 1.60	290	8	13	15.07.2005	G	KWPN	241	7	5	15	32	2092	63	M	6	6	no	spread	oxer	left	no	0	0	37,03	no
Verona	The Longines FEI World Cup Jumping - Verona round B	29.10.2017	CSI-W5*	4	1.40 - 1.60	290	8	13	15.07.2005	G	KWPN	241	7	5	15	32	2092	63	M	7	7	no	vertical	vertical	right	no	0	0	37,03	no
Verona	The Longines FEI World Cup Jumping - Verona round B	29.10.2017	CSI-W5*	4	1.40 - 1.60	290	8	13	15.07.2005	G	KWPN	241	7	5	15	32	2092	63	M	8	7	no	vertical	vertical	right	no	0	0	37,03	no
Verona	The Longines FEI World Cup Jumping - Verona round A	29.10.2017	CSI-W5*	4	1.40 - 1.60	465	159	11	15.04.2007	M	PZHK	161	4	11	414	32	1331	18	F	1	1	no	vertical	vertical	left	no	0	0	76,96	no
Verona	The Longines FEI World Cup Jumping - Verona round A	29.10.2017	CSI-W5*	4	1.40 - 1.60	465	159	11	15.04.2007	M	PZHK	161	4	11	414	32	1331	18	F	2	2	no	spread	oxer	left	no	0	0	76,96	no
Verona	The Longines FEI World Cup Jumping - Verona round A	29.10.2017	CSI-W5*	4	1.40 - 1.60	465	159	11	15.04.2007	M	PZHK	161	4	11	414	32	1331	18	F	3	3	no	vertical	vertical	right	no	0	0	76,96	no
Verona	The Longines FEI World Cup Jumping - Verona round A	29.10.2017	CSI-W5*	4	1.40 - 1.60	465	159	11	15.04.2007	M	PZHK	161	4	11	414	32	1331	18	F	4	4	no	spread	oxer	right	no	0	0	76,96	no
Verona	The Longines FEI World Cup Jumping - Verona round A	29.10.2017	CSI-W5*	4	1.40 - 1.60	465	159	11	15.04.2007	M	PZHK	161	4	11	414	32	1331	18	F	5	5	no	vertical	vertical with water	straight	no	0	0	76,96	no
Verona	The Longines FEI World Cup Jumping - Verona round A	29.10.2017	CSI-W5*	4	1.40 - 1.60	465	159	11	15.04.2007	M	PZHK	161	4	11	414	32	1331	18	F	6	6	no	spread	oxer with water	left	no	0	0	76,96	no
Verona	The Longines FEI World Cup Jumping - Verona round A	29.10.2017	CSI-W5*	4	1.40 - 1.60	465	159	11	15.04.2007	M	PZHK	161	4	11	414	32	1331	18	F	7	7a	yes	spread	oxer	straight	no	0	0	76,96	no
Verona	The Longines FEI World Cup Jumping - Verona round A	29.10.2017	CSI-W5*	4	1.40 - 1.60	465	159	11	15.04.2007	M	PZHK	161	4	11	414	32	1331	18	F	8	7b	yes	vertical	vertical	straight	no	0	0	76,96	no
Verona	The Longines FEI World Cup Jumping - Verona round A	29.10.2017	CSI-W5*	4	1.40 - 1.60	465	159	11	15.04.2007	M	PZHK	161	4	11	414	32	1331	18	F	9	8	no	vertical	vertical	left	no	0	0	76,96	no
Verona	The Longines FEI World Cup Jumping - Verona round A	29.10.2017	CSI-W5*	4	1.40 - 1.60	465	159	11	15.04.2007	M	PZHK	161	4	11	414	32	1331	18	F	10	9	no	spread	oxer	left	no	0	0	76,96	no
Verona	The Longines FEI World Cup Jumping - Verona round A	29.10.2017	CSI-W5*	4	1.40 - 1.60	465	159	11	15.04.2007	M	PZHK	161	4	11	414	32	1331	18	F	11	10	no	vertical	vertical	right	no	0	0	76,96	no
Verona	The Longines FEI World Cup Jumping - Verona round A	29.10.2017	CSI-W5*	4	1.40 - 1.60	465	159	11	15.04.2007	M	PZHK	161	4	11	414	32	1331	18	F	12	11a	yes	vertical	vertical	right	no	0	0	76,96	no
Verona	The Longines FEI World Cup Jumping - Verona round A	29.10.2017	CSI-W5*	4	1.40 - 1.60	465	159	11	15.04.2007	M	PZHK	161	4	11	414	32	1331	18	F	13	11b	yes	spread	oxer	right	no	0	0	76,96	no
Verona	The Longines FEI World Cup Jumping - Verona round A	29.10.2017	CSI-W5*	4	1.40 - 1.60	465	159	11	15.04.2007	M	PZHK	161	4	11	414	32	1331	18	F	14	11c	yes	spread	oxer	right	no	0	0	76,96	no
Verona	The Longines FEI World Cup Jumping - Verona round A	29.10.2017	CSI-W5*	4	1.40 - 1.60	465	159	11	15.04.2007	M	PZHK	161	4	11	414	32	1331	18	F	15	12	no	vertical	vertical	left	no	0	0	76,96	no
Verona	The Longines FEI World Cup Jumping - Verona round A	29.10.2017	CSI-W5*	4	1.40 - 1.60	465	159	11	15.04.2007	M	PZHK	161	4	11	414	32	1331	18	F	16	13	no	spread	oxer	straight	no	0	0	76,96	no
Verona	The Longines FEI World Cup Jumping - Verona round A	29.10.2017	CSI-W5*	4	1.40 - 1.60	465	112	13	01.06.2005	G	SF	182	8	14	5	37	1868	96	M	1	1	no	vertical	vertical	left	no	0	0	74,48	no
Verona	The Longines FEI World Cup Jumping - Verona round A	29.10.2017	CSI-W5*	4	1.40 - 1.60	465	112	13	01.06.2005	G	SF	182	8	14	5	37	1868	96	M	2	2	no	spread	oxer	left	no	0	0	74,48	no
Verona	The Longines FEI World Cup Jumping - Verona round A	29.10.2017	CSI-W5*	4	1.40 - 1.60	465	112	13	01.06.2005	G	SF	182	8	14	5	37	1868	96	M	3	3	no	vertical	vertical	right	no	0	0	74,48	no
Verona	The Longines FEI World Cup Jumping - Verona round A	29.10.2017	CSI-W5*	4	1.40 - 1.60	465	112	13	01.06.2005	G	SF	182	8	14	5	37	1868	96	M	4	4	no	spread	oxer	right	no	0	0	74,48	no
Verona	The Longines FEI World Cup Jumping - Verona round A	29.10.2017	CSI-W5*	4	1.40 - 1.60	465	112	13	01.06.2005	G	SF	182	8	14	5	37	1868	96	M	5	5	no	vertical	vertical with water	straight	no	0	0	74,48	no
Verona	The Longines FEI World Cup Jumping - Verona round A	29.10.2017	CSI-W5*	4	1.40 - 1.60	465	112	13	01.06.2005	G	SF	182	8	14	5	37	1868	96	M	6	6	no	spread	oxer with water	left	no	0	0	74,48	no
Verona	The Longines FEI World Cup Jumping - Verona round A	29.10.2017	CSI-W5*	4	1.40 - 1.60	465	112	13	01.06.2005	G	SF	182	8	14	5	37	1868	96	M	7	7a	yes	spread	oxer	straight	no	0	0	74,48	no
Verona	The Longines FEI World Cup Jumping - Verona round A	29.10.2017	CSI-W5*	4	1.40 - 1.60	465	112	13	01.06.2005	G	SF	182	8	14	5	37	1868	96	M	8	7b	yes	vertical	vertical	straight	no	0	0	74,48	no
Verona	The Longines FEI World Cup Jumping - Verona round A	29.10.2017	CSI-W5*	4	1.40 - 1.60	465	112	13	01.06.2005	G	SF	182	8	14	5	37	1868	96	M	9	8	no	vertical	vertical	left	no	0	0	74,48	no
Verona	The Longines FEI World Cup Jumping - Verona round A	29.10.2017	CSI-W5*	4	1.40 - 1.60	465	112	13	01.06.2005	G	SF	182	8	14	5	37	1868	96	M	10	9	no	spread	oxer	left	no	0	0	74,48	no
Verona	The Longines FEI World Cup Jumping - Verona round A	29.10.2017	CSI-W5*	4	1.40 - 1.60	465	112	13	01.06.2005	G	SF	182	8	14	5	37	1868	96	M	11	10	no	vertical	vertical	right	no	0	0	74,48	no
Verona	The Longines FEI World Cup Jumping - Verona round A	29.10.2017	CSI-W5*	4	1.40 - 1.60	465	112	13	01.06.2005	G	SF	182	8	14	5	37	1868	96	M	12	11a	yes	vertical	vertical	right	no	0	0	74,48	no
Verona	The Longines FEI World Cup Jumping - Verona round A	29.10.2017	CSI-W5*	4	1.40 - 1.60	465	112	13	01.06.2005	G	SF	182	8	14	5	37	1868	96	M	13	11b	yes	spread	oxer	right	no	0	0	74,48	no
Verona	The Longines FEI World Cup Jumping - Verona round A	29.10.2017	CSI-W5*	4	1.40 - 1.60	465	112	13	01.06.2005	G	SF	182	8	14	5	37	1868	96	M	14	11c	yes	spread	oxer	right	no	0	0	74,48	no
Verona	The Longines FEI World Cup Jumping - Verona round A	29.10.2017	CSI-W5*	4	1.40 - 1.60	465	112	13	01.06.2005	G	SF	182	8	14	5	37	1868	96	M	15	12	no	vertical	vertical	left	no	0	0	74,48	no
Verona	The Longines FEI World Cup Jumping - Verona round A	29.10.2017	CSI-W5*	4	1.40 - 1.60	465	112	13	01.06.2005	G	SF	182	8	14	5	37	1868	96	M	16	13	no	spread	oxer	straight	no	0	0	74,48	no
Verona	The Longines FEI World Cup Jumping - Verona round B	29.10.2017	CSI-W5*	4	1.40 - 1.60	290	112	13	01.06.2005	G	SF	182	8	14	5	37	1868	96	M	1	1	no	vertical	vertical	right	no	0	0	35,49	no
Verona	The Longines FEI World Cup Jumping - Verona round B	29.10.2017	CSI-W5*	4	1.40 - 1.60	290	112	13	01.06.2005	G	SF	182	8	14	5	37	1868	96	M	2	2	no	spread	oxer	right	no	0	0	35,49	no
Verona	The Longines FEI World Cup Jumping - Verona round B	29.10.2017	CSI-W5*	4	1.40 - 1.60	290	112	13	01.06.2005	G	SF	182	8	14	5	37	1868	96	M	3	4a	yes	vertical	vertical with water	straight	no	0	0	35,49	no
Verona	The Longines FEI World Cup Jumping - Verona round B	29.10.2017	CSI-W5*	4	1.40 - 1.60	290	112	13	01.06.2005	G	SF	182	8	14	5	37	1868	96	M	4	4b	yes	spread	oxer	straight	no	0	0	35,49	no
Verona	The Longines FEI World Cup Jumping - Verona round B	29.10.2017	CSI-W5*	4	1.40 - 1.60	290	112	13	01.06.2005	G	SF	182	8	14	5	37	1868	96	M	5	5	no	vertical	vertical	left	no	0	0	35,49	no
Verona	The Longines FEI World Cup Jumping - Verona round B	29.10.2017	CSI-W5*	4	1.40 - 1.60	290	112	13	01.06.2005	G	SF	182	8	14	5	37	1868	96	M	6	6	no	spread	oxer	left	no	0	0	35,49	no
Verona	The Longines FEI World Cup Jumping - Verona round B	29.10.2017	CSI-W5*	4	1.40 - 1.60	290	112	13	01.06.2005	G	SF	182	8	14	5	37	1868	96	M	7	7	no	vertical	vertical	right	yes	4	0	35,49	no
Verona	The Longines FEI World Cup Jumping - Verona round B	29.10.2017	CSI-W5*	4	1.40 - 1.60	290	112	13	01.06.2005	G	SF	182	8	14	5	37	1868	96	M	8	7	no	vertical	vertical	right	no	0	0	35,49	no
Verona	The Longines FEI World Cup Jumping - Verona round A	29.10.2017	CSI-W5*	4	1.40 - 1.60	465	191	11	08.06.2007	G	BWP	169	6	16	17	37	2215	106	M	1	1	no	vertical	vertical	left	no	0	0	0,00	yes
Verona	The Longines FEI World Cup Jumping - Verona round A	29.10.2017	CSI-W5*	4	1.40 - 1.60	465	191	11	08.06.2007	G	BWP	169	6	16	17	37	2215	106	M	2	2	no	spread	oxer	left	yes	4	0	0,00	yes
Verona	The Longines FEI World Cup Jumping - Verona round A	29.10.2017	CSI-W5*	4	1.40 - 1.60	465	191	11	08.06.2007	G	BWP	169	6	16	17	37	2215	106	M	3	3	no	vertical	vertical	right	no	0	0	0,00	yes
Verona	The Longines FEI World Cup Jumping - Verona round A	29.10.2017	CSI-W5*	4	1.40 - 1.60	465	191	11	08.06.2007	G	BWP	169	6	16	17	37	2215	106	M	4	4	no	spread	oxer	right	yes	4	0	0,00	yes
Verona	The Longines FEI World Cup Jumping - Verona round A	29.10.2017	CSI-W5*	4	1.40 - 1.60	465	191	11	08.06.2007	G	BWP	169	6	16	17	37	2215	106	M	5	5	no	vertical	vertical with water	straight	no	0	0	0,00	yes
Verona	The Longines FEI World Cup Jumping - Verona round A	29.10.2017	CSI-W5*	4	1.40 - 1.60	465	191	11	08.06.2007	G	BWP	169	6	16	17	37	2215	106	M	6	6	no	spread	oxer with water	left	no	0	0	0,00	yes
Verona	The Longines FEI World Cup Jumping - Verona round A	29.10.2017	CSI-W5*	4	1.40 - 1.60	465	191	11	08.06.2007	G	BWP	169	6	16	17	37	2215	106	M	7	7a	yes	spread	oxer	straight	no	0	0	0,00	yes
Verona	The Longines FEI World Cup Jumping - Verona round A	29.10.2017	CSI-W5*	4	1.40 - 1.60	465	191	11	08.06.2007	G	BWP	169	6	16	17	37	2215	106	M	8	7b	yes	vertical	vertical	straight	no	0	0	0,00	yes
Verona	The Longines FEI World Cup Jumping - Verona round A	29.10.2017	CSI-W5*	4	1.40 - 1.60	465	191	11	08.06.2007	G	BWP	169	6	16	17	37	2215	106	M	9	8	no	vertical	vertical	left	no	0	0	0,00	yes
Verona	The Longines FEI World Cup Jumping - Verona round A	29.10.2017	CSI-W5*	4	1.40 - 1.60	465	191	11	08.06.2007	G	BWP	169	6	16	17	37	2215	106	M	10	9	no	spread	oxer	left	no	0	0	0,00	yes
Verona	The Longines FEI World Cup Jumping - Verona round A	29.10.2017	CSI-W5*	4	1.40 - 1.60	465	191	11	08.06.2007	G	BWP	169	6	16	17	37	2215	106	M	11	10	no	vertical	vertical	right	no	0	0	0,00	yes
Verona	The Longines FEI World Cup Jumping - Verona round A	29.10.2017	CSI-W5*	4	1.40 - 1.60	465	191	11	08.06.2007	G	BWP	169	6	16	17	37	2215	106	M	12	11a	yes	vertical	vertical	right	no	0	0	0,00	yes
Verona	The Longines FEI World Cup Jumping - Verona round A	29.10.2017	CSI-W5*	4	1.40 - 1.60	465	191	11	08.06.2007	G	BWP	169	6	16	17	37	2215	106	M	13	11b	yes	spread	oxer	right	no	0	0	0,00	yes
Verona	The Longines FEI World Cup Jumping - Verona round A	29.10.2017	CSI-W5*	4	1.40 - 1.60	465	191	11	08.06.2007	G	BWP	169	6	16	17	37	2215	106	M	14	11c	yes	spread	oxer	right	no	0	0	0,00	yes
Verona	The Longines FEI World Cup Jumping - Verona round A	29.10.2017	CSI-W5*	4	1.40 - 1.60	465	191	11	08.06.2007	G	BWP	169	6	16	17	37	2215	106	M	15	12	no	vertical	vertical	left	no	0	0	0,00	yes
Verona	The Longines FEI World Cup Jumping - Verona round A	29.10.2017	CSI-W5*	4	1.40 - 1.60	465	191	11	08.06.2007	G	BWP	169	6	16	17	37	2215	106	M	16	13	no	spread	oxer	straight	no	0	0	0,00	yes
Verona	The Longines FEI World Cup Jumping - Verona round A	29.10.2017	CSI-W5*	4	1.40 - 1.60	465	219	13	13.05.2005	G	SWB	110	2	21	99	28	1384	56	F	1	1	no	vertical	vertical	left	no	0	0	75,27	no
Verona	The Longines FEI World Cup Jumping - Verona round A	29.10.2017	CSI-W5*	4	1.40 - 1.60	465	219	13	13.05.2005	G	SWB	110	2	21	99	28	1384	56	F	2	2	no	spread	oxer	left	no	0	0	75,27	no
Verona	The Longines FEI World Cup Jumping - Verona round A	29.10.2017	CSI-W5*	4	1.40 - 1.60	465	219	13	13.05.2005	G	SWB	110	2	21	99	28	1384	56	F	3	3	no	vertical	vertical	right	no	0	0	75,27	no
Verona	The Longines FEI World Cup Jumping - Verona round A	29.10.2017	CSI-W5*	4	1.40 - 1.60	465	219	13	13.05.2005	G	SWB	110	2	21	99	28	1384	56	F	4	4	no	spread	oxer	right	no	0	0	75,27	no
Verona	The Longines FEI World Cup Jumping - Verona round A	29.10.2017	CSI-W5*	4	1.40 - 1.60	465	219	13	13.05.2005	G	SWB	110	2	21	99	28	1384	56	F	5	5	no	vertical	vertical with water	straight	no	0	0	75,27	no
Verona	The Longines FEI World Cup Jumping - Verona round A	29.10.2017	CSI-W5*	4	1.40 - 1.60	465	219	13	13.05.2005	G	SWB	110	2	21	99	28	1384	56	F	6	6	no	spread	oxer with water	left	no	0	0	75,27	no
Verona	The Longines FEI World Cup Jumping - Verona round A	29.10.2017	CSI-W5*	4	1.40 - 1.60	465	219	13	13.05.2005	G	SWB	110	2	21	99	28	1384	56	F	7	7a	yes	spread	oxer	straight	no	0	0	75,27	no
Verona	The Longines FEI World Cup Jumping - Verona round A	29.10.2017	CSI-W5*	4	1.40 - 1.60	465	219	13	13.05.2005	G	SWB	110	2	21	99	28	1384	56	F	8	7b	yes	vertical	vertical	straight	no	0	0	75,27	no
Verona	The Longines FEI World Cup Jumping - Verona round A	29.10.2017	CSI-W5*	4	1.40 - 1.60	465	219	13	13.05.2005	G	SWB	110	2	21	99	28	1384	56	F	9	8	no	vertical	vertical	left	no	0	0	75,27	no
Verona	The Longines FEI World Cup Jumping - Verona round A	29.10.2017	CSI-W5*	4	1.40 - 1.60	465	219	13	13.05.2005	G	SWB	110	2	21	99	28	1384	56	F	10	9	no	spread	oxer	left	no	0	0	75,27	no
Verona	The Longines FEI World Cup Jumping - Verona round A	29.10.2017	CSI-W5*	4	1.40 - 1.60	465	219	13	13.05.2005	G	SWB	110	2	21	99	28	1384	56	F	11	10	no	vertical	vertical	right	no	0	0	75,27	no
Verona	The Longines FEI World Cup Jumping - Verona round A	29.10.2017	CSI-W5*	4	1.40 - 1.60	465	219	13	13.05.2005	G	SWB	110	2	21	99	28	1384	56	F	12	11a	yes	vertical	vertical	right	no	0	0	75,27	no
Verona	The Longines FEI World Cup Jumping - Verona round A	29.10.2017	CSI-W5*	4	1.40 - 1.60	465	219	13	13.05.2005	G	SWB	110	2	21	99	28	1384	56	F	13	11b	yes	spread	oxer	right	no	0	0	75,27	no
Verona	The Longines FEI World Cup Jumping - Verona round A	29.10.2017	CSI-W5*	4	1.40 - 1.60	465	219	13	13.05.2005	G	SWB	110	2	21	99	28	1384	56	F	14	11c	yes	spread	oxer	right	no	0	0	75,27	no
Verona	The Longines FEI World Cup Jumping - Verona round A	29.10.2017	CSI-W5*	4	1.40 - 1.60	465	219	13	13.05.2005	G	SWB	110	2	21	99	28	1384	56	F	15	12	no	vertical	vertical	left	no	0	0	75,27	no
Verona	The Longines FEI World Cup Jumping - Verona round A	29.10.2017	CSI-W5*	4	1.40 - 1.60	465	219	13	13.05.2005	G	SWB	110	2	21	99	28	1384	56	F	16	13	no	spread	oxer	straight	no	0	0	75,27	no
Verona	The Longines FEI World Cup Jumping - Verona round A	29.10.2017	CSI-W5*	4	1.40 - 1.60	465	28	12	26.03.2006	M	ZANG	193	9	23	11	35	1800	65	M	1	1	no	vertical	vertical	left	no	0	0	75,51	no
Verona	The Longines FEI World Cup Jumping - Verona round A	29.10.2017	CSI-W5*	4	1.40 - 1.60	465	28	12	26.03.2006	M	ZANG	193	9	23	11	35	1800	65	M	2	2	no	spread	oxer	left	no	0	0	75,51	no
Verona	The Longines FEI World Cup Jumping - Verona round A	29.10.2017	CSI-W5*	4	1.40 - 1.60	465	28	12	26.03.2006	M	ZANG	193	9	23	11	35	1800	65	M	3	3	no	vertical	vertical	right	no	0	0	75,51	no
Verona	The Longines FEI World Cup Jumping - Verona round A	29.10.2017	CSI-W5*	4	1.40 - 1.60	465	28	12	26.03.2006	M	ZANG	193	9	23	11	35	1800	65	M	4	4	no	spread	oxer	right	yes	4	0	75,51	no
Verona	The Longines FEI World Cup Jumping - Verona round A	29.10.2017	CSI-W5*	4	1.40 - 1.60	465	28	12	26.03.2006	M	ZANG	193	9	23	11	35	1800	65	M	5	5	no	vertical	vertical with water	straight	no	0	0	75,51	no
Verona	The Longines FEI World Cup Jumping - Verona round A	29.10.2017	CSI-W5*	4	1.40 - 1.60	465	28	12	26.03.2006	M	ZANG	193	9	23	11	35	1800	65	M	6	6	no	spread	oxer with water	left	no	0	0	75,51	no
Verona	The Longines FEI World Cup Jumping - Verona round A	29.10.2017	CSI-W5*	4	1.40 - 1.60	465	28	12	26.03.2006	M	ZANG	193	9	23	11	35	1800	65	M	7	7a	yes	spread	oxer	straight	no	0	0	75,51	no
Verona	The Longines FEI World Cup Jumping - Verona round A	29.10.2017	CSI-W5*	4	1.40 - 1.60	465	28	12	26.03.2006	M	ZANG	193	9	23	11	35	1800	65	M	8	7b	yes	vertical	vertical	straight	no	0	0	75,51	no
Verona	The Longines FEI World Cup Jumping - Verona round A	29.10.2017	CSI-W5*	4	1.40 - 1.60	465	28	12	26.03.2006	M	ZANG	193	9	23	11	35	1800	65	M	9	8	no	vertical	vertical	left	no	0	0	75,51	no
Verona	The Longines FEI World Cup Jumping - Verona round A	29.10.2017	CSI-W5*	4	1.40 - 1.60	465	28	12	26.03.2006	M	ZANG	193	9	23	11	35	1800	65	M	10	9	no	spread	oxer	left	no	0	0	75,51	no
Verona	The Longines FEI World Cup Jumping - Verona round A	29.10.2017	CSI-W5*	4	1.40 - 1.60	465	28	12	26.03.2006	M	ZANG	193	9	23	11	35	1800	65	M	11	10	no	vertical	vertical	right	no	0	0	75,51	no
Verona	The Longines FEI World Cup Jumping - Verona round A	29.10.2017	CSI-W5*	4	1.40 - 1.60	465	28	12	26.03.2006	M	ZANG	193	9	23	11	35	1800	65	M	12	11a	yes	vertical	vertical	right	no	0	0	75,51	no
Verona	The Longines FEI World Cup Jumping - Verona round A	29.10.2017	CSI-W5*	4	1.40 - 1.60	465	28	12	26.03.2006	M	ZANG	193	9	23	11	35	1800	65	M	13	11b	yes	spread	oxer	right	no	0	0	75,51	no
Verona	The Longines FEI World Cup Jumping - Verona round A	29.10.2017	CSI-W5*	4	1.40 - 1.60	465	28	12	26.03.2006	M	ZANG	193	9	23	11	35	1800	65	M	14	11c	yes	spread	oxer	right	no	0	0	75,51	no
Verona	The Longines FEI World Cup Jumping - Verona round A	29.10.2017	CSI-W5*	4	1.40 - 1.60	465	28	12	26.03.2006	M	ZANG	193	9	23	11	35	1800	65	M	15	12	no	vertical	vertical	left	no	0	0	75,51	no
Verona	The Longines FEI World Cup Jumping - Verona round A	29.10.2017	CSI-W5*	4	1.40 - 1.60	465	28	12	26.03.2006	M	ZANG	193	9	23	11	35	1800	65	M	16	13	no	spread	oxer	straight	no	0	0	75,51	no

Verona	The Longines FEI World Cup Jumping - Verona round A	29.10.2017	CSI-W5*	4	1.40 - 1.60	465	214	16	09.05.2002	S	KWPN	324	24	26	14	30	1957	97	M	1	1	no	vertical	vertical	left	no	0	0	77,32	no
Verona	The Longines FEI World Cup Jumping - Verona round A	29.10.2017	CSI-W5*	4	1.40 - 1.60	465	214	16	09.05.2002	S	KWPN	324	24	26	14	30	1957	97	M	2	2	no	spread	oxer	left	no	0	0	77,32	no
Verona	The Longines FEI World Cup Jumping - Verona round A	29.10.2017	CSI-W5*	4	1.40 - 1.60	465	214	16	09.05.2002	S	KWPN	324	24	26	14	30	1957	97	M	3	3	no	vertical	vertical	right	no	0	0	77,32	no
Verona	The Longines FEI World Cup Jumping - Verona round A	29.10.2017	CSI-W5*	4	1.40 - 1.60	465	214	16	09.05.2002	S	KWPN	324	24	26	14	30	1957	97	M	4	4	no	spread	oxer	right	no	0	0	77,32	no
Verona	The Longines FEI World Cup Jumping - Verona round A	29.10.2017	CSI-W5*	4	1.40 - 1.60	465	214	16	09.05.2002	S	KWPN	324	24	26	14	30	1957	97	M	5	5	no	vertical	vertical with water	straight	no	0	0	77,32	no
Verona	The Longines FEI World Cup Jumping - Verona round A	29.10.2017	CSI-W5*	4	1.40 - 1.60	465	214	16	09.05.2002	S	KWPN	324	24	26	14	30	1957	97	M	6	6	no	spread	oxer with water	left	no	0	0	77,32	no
Verona	The Longines FEI World Cup Jumping - Verona round A	29.10.2017	CSI-W5*	4	1.40 - 1.60	465	214	16	09.05.2002	S	KWPN	324	24	26	14	30	1957	97	M	7	7a	yes	spread	oxer	straight	no	0	0	77,32	no
Verona	The Longines FEI World Cup Jumping - Verona round A	29.10.2017	CSI-W5*	4	1.40 - 1.60	465	214	16	09.05.2002	S	KWPN	324	24	26	14	30	1957	97	M	8	7b	yes	vertical	vertical	straight	no	0	0	77,32	no
Verona	The Longines FEI World Cup Jumping - Verona round A	29.10.2017	CSI-W5*	4	1.40 - 1.60	465	214	16	09.05.2002	S	KWPN	324	24	26	14	30	1957	97	M	9	8	no	vertical	vertical	left	no	0	0	77,32	no
Verona	The Longines FEI World Cup Jumping - Verona round A	29.10.2017	CSI-W5*	4	1.40 - 1.60	465	214	16	09.05.2002	S	KWPN	324	24	26	14	30	1957	97	M	10	9	no	spread	oxer	left	no	0	0	77,32	no
Verona	The Longines FEI World Cup Jumping - Verona round A	29.10.2017	CSI-W5*	4	1.40 - 1.60	465	214	16	09.05.2002	S	KWPN	324	24	26	14	30	1957	97	M	11	10	no	vertical	vertical	right	no	0	0	77,32	no
Verona	The Longines FEI World Cup Jumping - Verona round A	29.10.2017	CSI-W5*	4	1.40 - 1.60	465	214	16	09.05.2002	S	KWPN	324	24	26	14	30	1957	97	M	12	11a	yes	vertical	vertical	right	no	0	0	77,32	no
Verona	The Longines FEI World Cup Jumping - Verona round A	29.10.2017	CSI-W5*	4	1.40 - 1.60	465	214	16	09.05.2002	S	KWPN	324	24	26	14	30	1957	97	M	13	11b	yes	spread	oxer	right	no	0	0	77,32	no
Verona	The Longines FEI World Cup Jumping - Verona round A	29.10.2017	CSI-W5*	4	1.40 - 1.60	465	214	16	09.05.2002	S	KWPN	324	24	26	14	30	1957	97	M	14	11c	yes	spread	oxer	right	no	0	0	77,32	no
Verona	The Longines FEI World Cup Jumping - Verona round A	29.10.2017	CSI-W5*	4	1.40 - 1.60	465	214	16	09.05.2002	S	KWPN	324	24	26	14	30	1957	97	M	15	12	no	vertical	vertical	left	no	0	0	77,32	no
Verona	The Longines FEI World Cup Jumping - Verona round A	29.10.2017	CSI-W5*	4	1.40 - 1.60	465	214	16	09.05.2002	S	KWPN	324	24	26	14	30	1957	97	M	16	13	no	spread	oxer	straight	no	0	0	77,32	no
Verona	The Longines FEI World Cup Jumping - Verona round B	29.10.2017	CSI-W5*	4	1.40 - 1.60	290	214	16	09.05.2002	S	KWPN	324	24	26	14	30	1957	97	M	1	1	no	vertical	vertical	right	no	0	0	35,91	no
Verona	The Longines FEI World Cup Jumping - Verona round B	29.10.2017	CSI-W5*	4	1.40 - 1.60	290	214	16	09.05.2002	S	KWPN	324	24	26	14	30	1957	97	M	2	2	no	spread	oxer	right	no	0	0	35,91	no
Verona	The Longines FEI World Cup Jumping - Verona round B	29.10.2017	CSI-W5*	4	1.40 - 1.60	290	214	16	09.05.2002	S	KWPN	324	24	26	14	30	1957	97	M	3	4a	yes	vertical	vertical with water	straight	no	0	0	35,91	no
Verona	The Longines FEI World Cup Jumping - Verona round B	29.10.2017	CSI-W5*	4	1.40 - 1.60	290	214	16	09.05.2002	S	KWPN	324	24	26	14	30	1957	97	M	4	4b	yes	spread	oxer	straight	no	0	0	35,91	no
Verona	The Longines FEI World Cup Jumping - Verona round B	29.10.2017	CSI-W5*	4	1.40 - 1.60	290	214	16	09.05.2002	S	KWPN	324	24	26	14	30	1957	97	M	5	5	no	vertical	vertical	left	no	0	0	35,91	no
Verona	The Longines FEI World Cup Jumping - Verona round B	29.10.2017	CSI-W5*	4	1.40 - 1.60	290	214	16	09.05.2002	S	KWPN	324	24	26	14	30	1957	97	M	6	6	no	spread	oxer	left	no	0	0	35,91	no
Verona	The Longines FEI World Cup Jumping - Verona round B	29.10.2017	CSI-W5*	4	1.40 - 1.60	290	214	16	09.05.2002	S	KWPN	324	24	26	14	30	1957	97	M	7	7	no	vertical	vertical	right	no	0	0	35,91	no
Verona	The Longines FEI World Cup Jumping - Verona round B	29.10.2017	CSI-W5*	4	1.40 - 1.60	290	214	16	09.05.2002	S	KWPN	324	24	26	14	30	1957	97	M	8	7	no	vertical	vertical	right	no	0	0	35,91	no
Verona	The Longines FEI World Cup Jumping - Verona round A	29.10.2017	CSI-W5*	4	1.40 - 1.60	465	220	13	01.01.2005	M	MIPAAF	153	14	27	269	20	1104	42	F	1	1	no	vertical	vertical	left	no	0	0	75,44	no
Verona	The Longines FEI World Cup Jumping - Verona round A	29.10.2017	CSI-W5*	4	1.40 - 1.60	465	220	13	01.01.2005	M	MIPAAF	153	14	27	269	20	1104	42	F	2	2	no	spread	oxer	left	no	0	0	75,44	no
Verona	The Longines FEI World Cup Jumping - Verona round A	29.10.2017	CSI-W5*	4	1.40 - 1.60	465	220	13	01.01.2005	M	MIPAAF	153	14	27	269	20	1104	42	F	3	3	no	vertical	vertical	right	no	0	0	75,44	no
Verona	The Longines FEI World Cup Jumping - Verona round A	29.10.2017	CSI-W5*	4	1.40 - 1.60	465	220	13	01.01.2005	M	MIPAAF	153	14	27	269	20	1104	42	F	4	4	no	spread	oxer	right	yes	4	0	75,44	no
Verona	The Longines FEI World Cup Jumping - Verona round A	29.10.2017	CSI-W5*	4	1.40 - 1.60	465	220	13	01.01.2005	M	MIPAAF	153	14	27	269	20	1104	42	F	5	5	no	vertical	vertical with water	straight	no	0	0	75,44	no
Verona	The Longines FEI World Cup Jumping - Verona round A	29.10.2017	CSI-W5*	4	1.40 - 1.60	465	220	13	01.01.2005	M	MIPAAF	153	14	27	269	20	1104	42	F	6	6	no	spread	oxer with water	left	no	0	0	75,44	no
Verona	The Longines FEI World Cup Jumping - Verona round A	29.10.2017	CSI-W5*	4	1.40 - 1.60	465	220	13	01.01.2005	M	MIPAAF	153	14	27	269	20	1104	42	F	7	7a	yes	spread	oxer	straight	no	0	0	75,44	no
Verona	The Longines FEI World Cup Jumping - Verona round A	29.10.2017	CSI-W5*	4	1.40 - 1.60	465	220	13	01.01.2005	M	MIPAAF	153	14	27	269	20	1104	42	F	8	7b	yes	vertical	vertical	straight	no	0	0	75,44	no
Verona	The Longines FEI World Cup Jumping - Verona round A	29.10.2017	CSI-W5*	4	1.40 - 1.60	465	220	13	01.01.2005	M	MIPAAF	153	14	27	269	20	1104	42	F	9	8	no	vertical	vertical	left	no	0	0	75,44	no
Verona	The Longines FEI World Cup Jumping - Verona round A	29.10.2017	CSI-W5*	4	1.40 - 1.60	465	220	13	01.01.2005	M	MIPAAF	153	14	27	269	20	1104	42	F	10	9	no	spread	oxer	left	no	0	0	75,44	no
Verona	The Longines FEI World Cup Jumping - Verona round A	29.10.2017	CSI-W5*	4	1.40 - 1.60	465	220	13	01.01.2005	M	MIPAAF	153	14	27	269	20	1104	42	F	11	10	no	vertical	vertical	right	no	0	0	75,44	no
Verona	The Longines FEI World Cup Jumping - Verona round A	29.10.2017	CSI-W5*	4	1.40 - 1.60	465	220	13	01.01.2005	M	MIPAAF	153	14	27	269	20	1104	42	F	12	11a	yes	vertical	vertical	right	no	0	0	75,44	no
Verona	The Longines FEI World Cup Jumping - Verona round A	29.10.2017	CSI-W5*	4	1.40 - 1.60	465	220	13	01.01.2005	M	MIPAAF	153	14	27	269	20	1104	42	F	13	11b	yes	spread	oxer	right	no	0	0	75,44	no
Verona	The Longines FEI World Cup Jumping - Verona round A	29.10.2017	CSI-W5*	4	1.40 - 1.60	465	220	13	01.01.2005	M	MIPAAF	153	14	27	269	20	1104	42	F	14	11c	yes	spread	oxer	right	no	0	0	75,44	no
Verona	The Longines FEI World Cup Jumping - Verona round A	29.10.2017	CSI-W5*	4	1.40 - 1.60	465	220	13	01.01.2005	M	MIPAAF	153	14	27	269	20	1104	42	F	15	12	no	vertical	vertical	left	no	0	0	75,44	no
Verona	The Longines FEI World Cup Jumping - Verona round A	29.10.2017	CSI-W5*	4	1.40 - 1.60	465	220	13	01.01.2005	M	MIPAAF	153	14	27	269	20	1104	42	F	16	13	no	spread	oxer	straight	yes	4	0	75,44	no
Verona	The Longines FEI World Cup Jumping - Verona round A	29.10.2017	CSI-W5*	4	1.40 - 1.60	465	171	17	10.06.2001	G	SBS	295	13	28	4	38	2170	90	M	1	1	no	vertical	vertical	left	no	0	0	73,85	no
Verona	The Longines FEI World Cup Jumping - Verona round A	29.10.2017	CSI-W5*	4	1.40 - 1.60	465	171	17	10.06.2001	G	SBS	295	13	28	4	38	2170	90	M	2	2	no	spread	oxer	left	no	0	0	73,85	no
Verona	The Longines FEI World Cup Jumping - Verona round A	29.10.2017	CSI-W5*	4	1.40 - 1.60	465	171	17	10.06.2001	G	SBS	295	13	28	4	38	2170	90	M	3	3	no	vertical	vertical	right	no	0	0	73,85	no
Verona	The Longines FEI World Cup Jumping - Verona round A	29.10.2017	CSI-W5*	4	1.40 - 1.60	465	171	17	10.06.2001	G	SBS	295	13	28	4	38	2170	90	M	4	4	no	spread	oxer	right	no	0	0	73,85	no
Verona	The Longines FEI World Cup Jumping - Verona round A	29.10.2017	CSI-W5*	4	1.40 - 1.60	465	171	17	10.06.2001	G	SBS	295	13	28	4	38	2170	90	M	5	5	no	vertical	vertical with water	straight	no	0	0	73,85	no
Verona	The Longines FEI World Cup Jumping - Verona round A	29.10.2017	CSI-W5*	4	1.40 - 1.60	465	171	17	10.06.2001	G	SBS	295	13	28	4	38	2170	90	M	6	6	no	spread	oxer with water	left	yes	4	0	73,85	no
Verona	The Longines FEI World Cup Jumping - Verona round A	29.10.2017	CSI-W5*	4	1.40 - 1.60	465	171	17	10.06.2001	G	SBS	295	13	28	4	38	2170	90	M	7	7a	yes	spread	oxer	straight	yes	4	0	73,85	no
Verona	The Longines FEI World Cup Jumping - Verona round A	29.10.2017	CSI-W5*	4	1.40 - 1.60	465	171	17	10.06.2001	G	SBS	295	13	28	4	38	2170	90	M	8	7b	yes	vertical	vertical	straight	no	0	0	73,85	no
Verona	The Longines FEI World Cup Jumping - Verona round A	29.10.2017	CSI-W5*	4	1.40 - 1.60	465	171	17	10.06.2001	G	SBS	295	13	28	4	38	2170	90	M	9	8	no	vertical	vertical	left	no	0	0	73,85	no
Verona	The Longines FEI World Cup Jumping - Verona round A	29.10.2017	CSI-W5*	4	1.40 - 1.60	465	171	17	10.06.2001	G	SBS	295	13	28	4	38	2170	90	M	10	9	no	spread	oxer	left	no	0	0	73,85	no
Verona	The Longines FEI World Cup Jumping - Verona round A	29.10.2017	CSI-W5*	4	1.40 - 1.60	465	171	17	10.06.2001	G	SBS	295	13	28	4	38	2170	90	M	11	10	no	vertical	vertical	right	no	0	0	73,85	no
Verona	The Longines FEI World Cup Jumping - Verona round A	29.10.2017	CSI-W5*	4	1.40 - 1.60	465	171	17	10.06.2001	G	SBS	295	13	28	4	38	2170	90	M	12	11a	yes	vertical	vertical	right	no	0	0	73,85	no
Verona	The Longines FEI World Cup Jumping - Verona round A	29.10.2017	CSI-W5*	4	1.40 - 1.60	465	171	17	10.06.2001	G	SBS	295	13	28	4	38	2170	90	M	13	11b	yes	spread	oxer	right	no	0	0	73,85	no
Verona	The Longines FEI World Cup Jumping - Verona round A	29.10.2017	CSI-W5*	4	1.40 - 1.60	465	171	17	10.06.2001	G	SBS	295	13	28	4	38	2170	90	M	14	11c	yes	spread	oxer	right	no	0	0	73,85	no
Verona	The Longines FEI World Cup Jumping - Verona round A	29.10.2017	CSI-W5*	4	1.40 - 1.60	465	171	17	10.06.2001	G	SBS	295	13	28	4	38	2170	90	M	15	12	no	vertical	vertical	left	yes	4	0	73,85	no
Verona	The Longines FEI World Cup Jumping - Verona round A	29.10.2017	CSI-W5*	4	1.40 - 1.60	465	171	17	10.06.2001	G	SBS	295	13	28	4	38	2170	90	M	16	13	no	spread	oxer	straight	no	0	0	73,85	no
Verona	The Longines FEI World Cup Jumping - Verona round A	29.10.2017	CSI-W5*	4	1.40 - 1.60	465	15	12	26.07.2006	M	SWB	159	0	30	9	36	2556	124	M	1	1	no	vertical	vertical	left	no	0	0	76,14	no
Verona	The Longines FEI World Cup Jumping - Verona round A	29.10.2017	CSI-W5*	4	1.40 - 1.60	465	15	12	26.07.2006	M	SWB	159	0	30	9	36	2556	124	M	2	2	no	spread	oxer	left	no	0	0	76,14	no
Verona	The Longines FEI World Cup Jumping - Verona round A	29.10.2017	CSI-W5*	4	1.40 - 1.60	465	15	12	26.07.2006	M	SWB	159	0	30	9	36	2556	124	M	3	3	no	vertical	vertical	right	no	0	0	76,14	no
Verona	The Longines FEI World Cup Jumping - Verona round A	29.10.2017	CSI-W5*	4	1.40 - 1.60	465	15	12	26.07.2006	M	SWB	159	0	30	9	36	2556	124	M	4	4	no	spread	oxer	right	no	0	0	76,14	no
Verona	The Longines FEI World Cup Jumping - Verona round A	29.10.2017	CSI-W5*	4	1.40 - 1.60	465	15	12	26.07.2006	M	SWB	159	0	30	9	36	2556	124	M	5	5	no	vertical	vertical with water	straight	no	0	0	76,14	no
Verona	The Longines FEI World Cup Jumping - Verona round A	29.10.2017	CSI-W5*	4	1.40 - 1.60	465	15	12	26.07.2006	M	SWB	159	0	30	9	36	2556	124	M	6	6	no	spread	oxer with water	left	no	0	0	76,14	no
Verona	The Longines FEI World Cup Jumping - Verona round A	29.10.2017	CSI-W5*	4	1.40 - 1.60	465	15	12	26.07.2006	M	SWB	159	0	30	9	36	2556	124	M	7	7a	yes	spread	oxer	straight	no	0	0	76,14	no
Verona	The Longines FEI World Cup Jumping - Verona round A	29.10.2017	CSI-W5*	4	1.40 - 1.60	465	15	12	26.07.2006	M	SWB	159	0	30	9	36	2556	124	M	8	7b	yes	vertical	vertical	straight	no	0	0	76,14	no
Verona	The Longines FEI World Cup Jumping - Verona round A	29.10.2017	CSI-W5*	4	1.40 - 1.60	465	15	12	26.07.2006	M	SWB	159	0	30	9	36	2556	124	M	9	8	no	vertical	vertical	left	no	0	0	76,14	no
Verona	The Longines FEI World Cup Jumping - Verona round A	29.10.2017	CSI-W5*	4	1.40 - 1.60	465	15	12	26.07.2006	M	SWB	159	0	30	9	36	2556	124	M	10	9	no	spread	oxer	left	no	0	0	76,14	no
Verona	The Longines FEI World Cup Jumping - Verona round A	29.10.2017	CSI-W5*	4	1.40 - 1.60	465	15	12	26.07.2006	M	SWB	159	0	30	9	36	2556	124	M	11	10	no	vertical	vertical	right	no	0	0	76,14	no
Verona	The Longines FEI World Cup Jumping - Verona round A	29.10.2017	CSI-W5*	4	1.40 - 1.60	465	15	12	26.07.2006	M	SWB	159	0	30	9	36	2556	124	M	12	11a	yes	vertical	vertical	right	yes	4	0	76,14	no
Verona	The Longines FEI World Cup Jumping - Verona round A	29.10.2017	CSI-W5*	4	1.40 - 1.60	465	15	12	26.07.2006	M	SWB	159	0	30	9	36	2556	124	M	13	11b	yes	spread	oxer	right	no	0	0	76,14	no
Verona	The Longines FEI World Cup Jumping - Verona round A	29.10.2017	CSI-W5*	4	1.40 - 1.60	465	15	12	26.07.2006	M	SWB	159	0	30	9	36	2556	124	M	14	11c	yes	spread	oxer	right	no	0	0	76,14	no
Verona	The Longines FEI World Cup Jumping - Verona round A	29.10.2017	CSI-W5*	4	1.40 - 1.60	465	15	12	26.07.2006	M	SWB	159	0	30	9	36	2556	124	M	15	12	no	vertical	vertical	left	no	0	0	76,14	no
Verona	The Longines FEI World Cup Jumping - Verona round A	29.10.2017	CSI-W5*	4	1.40 - 1.60	465	15	12	26.07.2006	M	SWB	159	0	30	9	36	2556	124	M	16	13	no	spread	oxer	straight	no	0	0	76,14	no
Verona	The Longines FEI World Cup Jumping - Verona round A	29.10.2017	CSI-W5*	4	1.40 - 1.60	465	111	11	15.05.2007	S	BWP	140	3	33	23	23	1854	199	M	1	1	no	vertical	vertical	left	no	0	0	73,17	no
Verona	The Longines FEI World Cup Jumping - Verona round A	29.10.2017	CSI-W5*	4	1.40 - 1.60	465	111	11	15.05.2007	S	BWP	140	3	33	23	23	1854	199	M	2	2	no	spread	oxer	left	no	0	0	73,17	no
Verona	The Longines FEI World Cup Jumping - Verona round A	29.10.2017	CSI-W5*	4	1.40 - 1.60	465	111	11	15.05.2007	S	BWP	140	3	33	23	23	1854	199	M	3	3	no	vertical	vertical	right	no	0	0	73,17	no
Verona	The Longines FEI World Cup Jumping - Verona round A	29.10.2017	CSI-W5*	4	1.40 - 1.60	465	111	11	15.05.2007	S	BWP	140	3	33	23	23	1854	199	M	4	4	no	spread	oxer	right	no	0	0	73,17	no
Verona	The Longines FEI World Cup Jumping - Verona round A	29.10.2017	CSI-W5*	4	1.40 - 1.60	465	111	11	15.05.2007	S	BWP	140	3	33	23	23	1854	199	M	5	5	no	vertical	vertical with water	straight	no	0	0	73,17	no
Verona	The Longines FEI World Cup Jumping - Verona round A	29.10.2017	CSI-W5*	4	1.40 - 1.60	465	111	11	15.05.2007	S	BWP	140	3	33	23	23	1854	199	M	6	6	no	spread	oxer with water	left	no	0	0	73,17	no
Verona	The Longines FEI World Cup Jumping - Verona round A	29.10.2017	CSI-W5*	4	1.40 - 1.60	465	111	11	15.05.2007	S	BWP	140	3	33	23	23	1854	199	M	7	7a	yes	spread	oxer	straight	no	0	0	73,17	no
Verona	The Longines FEI World Cup Jumping - Verona round A	29.10.2017	CSI-W5*	4	1.40 - 1.60	465	111	11	15.05.2007	S	BWP	140	3	33	23	23	1854	199	M	8	7b	yes	vertical	vertical	straight	no	0	0	73,17	no
Verona	The Longines FEI World Cup Jumping - Verona round A	29.10.2017	CSI-W5*	4	1.40 - 1.60	465	111	11	15.05.2007	S	BWP	140	3	33	23	23	1854	199	M	9	8	no	vertical	vertical	left	no	0	0	73,17	no
Verona	The Longines FEI World Cup Jumping - Verona round A	29.10.2017	CSI-W5*	4	1.40 - 1.60	465	111	11	15.05.2007	S	BWP	140	3	33	23	23	1854	199	M	10	9	no	spread	oxer	left	no	0	0	73,17	no
Verona	The Longines FEI World Cup Jumping - Verona round A	29.10.2017	CSI-W5*	4	1.40 - 1.60	465	111	11	15.05.2007	S	BWP	140	3	33	23	23	1854	199	M	11	10	no	vertical	vertical	right	yes	4	0	73,17	no
Verona	The Longines FEI World Cup Jumping - Verona round A	29.10.2017	CSI-W5*	4	1.40 - 1.60	465	111	11	15.05.2007	S	BWP	140	3	33	23	23	1854	199	M	12	11a	yes	vertical	vertical	right	no	0	0	73,17	no
Verona	The Longines FEI World Cup Jumping - Verona round A	29.10.2017	CSI-W5*	4	1.40 - 1.60	465	111	11	15.05.2007	S	BWP	140	3	33	23	23	1854	199	M	13	11b	yes	spread	oxer	right	no	0	0	73,17	no
Verona	The Longines FEI World Cup Jumping - Verona round A	29.10.2017	CSI-W5*	4	1.40 - 1.60	465	111	11	15.05.2007	S	BWP	140	3	33	23	23	1854	199	M	14	11c	yes	spread	oxer	right	no	0	0	73,17	no
Verona	The Longines FEI World Cup Jumping - Verona round A	29.10.2017	CSI-W5*	4	1.40 - 1.60	465	111	11	15.05.2007	S	BWP	140	3	33	23	23	1854	199	M	15	12	no	vertical	vertical	left	no	0	0	73,17	no
Verona	The Longines FEI World Cup Jumping - Verona round A	29.10.2017	CSI-W5*	4	1.40 - 1.60	465	111	11	15.05.2007	S	BWP	140	3	33	23	23	1854	199	M	16	13	no	spread	oxer	straight	yes	4	0	73,17	no
Verona	The Longines FEI World Cup Jumping - Verona round A	29.10.2017	CSI-W5*	4	1.40 - 1.60	465	138	9	15.03.2009	G	BWP	74	0	34	85	29	1646	66	M	1	1	no	vertical	vertical	left	no	0	0	78,95	no
Verona	The Longines FEI World Cup Jumping - Verona round A	29.10.2017	CSI-W5*	4	1.40 - 1.60	465	138	9	15.03.2009	G	BWP	74	0	34	85	29	1646	66	M	2	2	no	spread	oxer	left	no	0	0	78,95	no
Verona	The Longines FEI World Cup Jumping - Verona round A	29.10.2017	CSI-W5*	4	1.40 - 1.60	465	138	9	15.03.2009	G	BWP	74	0	34	85	29	1646	66	M	3	3	no	vertical	vertical	right	no	0	0	78,95	no
Verona	The Longines FEI World Cup Jumping - Verona round A	29.10.2017	CSI-W5*	4	1.40 - 1.60	465	138	9	15.03.2009	G	BWP	74	0	34	85	29	1646	66	M	4	4	no	spread	oxer	right	no	0	0	78,95	no
Verona	The Longines FEI World Cup Jumping - Verona round A	29.10.2017	CSI-W5*	4	1.40 - 1.60	465	138	9	15.03.2009	G	BWP	74	0	34	85	29	1646	66	M	5	5	no	vertical	vertical with water	straight	no	0	0	78,95	no
Verona	The Longines FEI World Cup Jumping - Verona round A	29.10.2017	CSI-W5*	4	1.40 - 1.60	465	138	9	15.03.2009	G	BWP	74	0	34	85	29	1646	66	M	6	6	no	spread	oxer with water	left	no	0	0	78,95	no
Verona	The Longines FEI World Cup Jumping - Verona round A	29.10.2017	CSI-W5*	4	1.40 - 1.60	465	138	9	15.03.2009	G	BWP	74	0	34	85	29	1646	66	M	7	7a	yes	spread	oxer	straight	no	0	0	78,95	no
Verona	The Longines FEI World Cup Jumping - Verona round A	29.10.2017	CSI-W5*	4	1.40 - 1.60	465	138	9	15.03.2009	G	BWP	74	0	34	85	29	1646	66	M	8	7b	yes	vertical	vertical	straight	no	0	0	78,95	no
Verona	The Longines FEI World Cup Jumping - Verona round A	29.10.2017	CSI-W5*	4	1.40 - 1.60	465	138	9	15.03.2009	G	BWP	74	0	34	85	29	1646	66	M	9	8	no	vertical	vertical	left	no	0	0	78,95	no
Verona	The Longines FEI World Cup Jumping - Verona round A	29.10.2017	CSI-W5*	4	1.40 - 1.60	465	138	9	15.03.2009	G	BWP	74	0	34	85	29	1646	66	M	11	10	no	vertical	vertical	right	no	0	0	78,95	no
Verona	The Longines FEI World Cup Jumping - Verona round A	29.10.2017	CSI-W5*	4	1.40 - 1.60	465	138	9	15.03.2009	G	BWP	74	0	34	85	29	1646	66	M	12	11a	yes	vertical	vertical	right	no	0	0	78,95	no
Verona	The Longines FEI World Cup Jumping - Verona round A	29.10.2017	CSI-W5*	4	1.40 - 1.60	465	138	9	15.03.2009	G	BWP	74	0	34	85	29	1646	66	M	13	11b	yes	spread	oxer	right	no	0	0	78,95	no
Verona	The Longines FEI World Cup Jumping - Verona round A	29.10.2017	CSI-W5*	4	1.40 - 1.60	465	138	9	15.03.2009	G	BWP	74	0	34	85	29	1646	66	M	14	11c	yes	spread	oxer	right	no	0	0	78,95	no
Verona	The Longines FEI World Cup Jumping - Verona round A	29.10.2017	CSI-W5*	4	1.40 - 1.60	465	138	9	15.03.2009	G	BWP	74	0	34	85	29	1646	66	M	15	12	no	vertical	vertical	left	no	0	0	78,95	no
Verona	The Longines FEI World Cup Jumping - Verona round A	29.10.2017	CSI-W5*	4	1.40 - 1.60	465	138	9	15.03.2009	G	BWP	74	0	34	85	29	1646	66	M	16	13	no	spread	oxer	straight	no	0	0	78,95	no
Verona	The Longines FEI World Cup Jumping - Verona round A	29.10.2017	CSI-W5*	4	1.40 - 1.60	465	138	9	15.03.2009	G	BWP	74	0	34	85	29	1646	66	M	10	9	no	spread	oxer	left	no	0	0	78,95	no
Verona	The Longines FEI World Cup Jumping - Verona round A	29.10.2017	CSI-W5*	4	1.40 - 1.60	465	19	11	15.05.2007	M	KWPN	97	5	36	47	44	1871	37	F	1	1	no	vertical	vertical	left	no	0	0	77,86	no
Verona	The Longines FEI World Cup Jumping - Verona round A	29.10.2017	CSI-W5*	4	1.40 - 1.60	465	19	11	15.05.2007	M	KWPN	97	5	36	47	44	1871	37	F	2	2	no	spread	oxer	left	no	0	0	77,86	no
Verona	The Longines FEI World Cup Jumping - Verona round A	29.10.2017	CSI-W5*	4	1.40 - 1.60	465	19	11	15.05.2007	M	KWPN	97	5	36	47	44	1871	37	F	3	3	no	vertical	vertical	right	no	0	0	77,86	no
Verona	The Longines FEI World Cup Jumping - Verona round A	29.10.2017	CSI-W5*	4	1.40 - 1.60	465	19	11	15.05.2007	M	KWPN	97	5	36	47	44	1871	37	F	4	4	no	spread	oxer	right	no	0	0	77,86	no
Verona	The Longines FEI World Cup Jumping - Verona round A	29.10.2017	CSI-W5*	4	1.40 - 1.60	465	19	11	15.05.2007	M	KWPN	97	5	36	47	44	1871	37	F	5	5	no	vertical	vertical with water	straight	no	0	0	77,86	no
Verona	The Longines FEI World Cup Jumping - Verona round A	29.10.2017	CSI-W5*	4	1.40 - 1.60	465	19	11	15.05.2007	M	KWPN	97	5	36	47	44	1871	37	F	6	6	no	spread	oxer with water	left	no	0	0	77,86	no
Verona	The Longines FEI World Cup Jumping - Verona round A	29.10.2017	CSI-W5*	4	1.40 - 1.60	465	19	11	15.05.2007	M	KWPN	97	5	36	47	44	1871	37	F	7	7a	yes	spread	oxer	straight	no	0	0	77,86	no
Verona	The Longines FEI World Cup Jumping - Verona round A	29.10.2017	CSI-W5*	4	1.40 - 1.60	465	19	11	15.05.2007	M	KWPN	97	5	36	47	44	1871	37	F	8	7b	yes	vertical	vertical	straight	no	0	0	77,86	no
Verona	The Longines FEI World Cup Jumping - Verona round A	29.10.2017	CSI-W5*	4	1.40 - 1.60	465	19	11	15.05.2007	M	KWPN	97	5	36	47	44	1871	37	F	9	8	no	vertical	vertical	left	no	0	0	77,86	no
Verona	The Longines FEI World Cup Jumping - Verona round A	29.10.2017	CSI-W5*	4	1.40 - 1.60	465	19	11	15.05.2007	M	KWPN	97	5	36	47	44	1871	37	F	10	9	no	spread	oxer	left	no	0	0	77,86	no
Verona	The Longines FEI World Cup Jumping - Verona round A	29.10.2017	CSI-W5*	4	1.40 - 1.60	465	19	11	15.05.2007	M	KWPN	97	5	36	47	44	1871	37	F	11	10	no	vertical	vertical	right	no	0	0	77,86	no
Verona	The Longines FEI World Cup Jumping - Verona round A	29.10.2017	CSI-W5*	4	1.40 - 1.60	465	19	11	15.05.2007	M	KWPN	97	5	36	47	44	1871	37	F	12	11a	yes	vertical	vertical	right	no	0	0	77,86	no
Verona	The Longines FEI World Cup Jumping - Verona round A	29.10.2017	CSI-W5*	4	1.40 - 1.60	465	19	11	15.05.2007	M	KWPN	97	5	36	47	44	1871	37	F	13	11b	yes	spread	oxer	right	yes	4	0	77,86	no
Verona	The Longines FEI World Cup Jumping - Verona round A	29.10.2017	CSI-W5*	4	1.40 - 1.60	465	19	11	15.05.2007	M	KWPN	97	5	36	47	44	1871	37	F	14	11c	yes	spread	oxer	right	no	0	0	77,86	no
Verona	The Longines FEI World Cup Jumping - Verona round A	29.10.2017	CSI-W5*	4	1.40 - 1.60	465	19	11	15.05.2007	M	KWPN	97	5	36	47	44	1871	37	F	15	12	no	vertical	vertical	left	no	0	0	77,86	no
Verona	The Longines FEI World Cup Jumping - Verona round A	29.10.2017	CSI-W5*	4	1.40 - 1.60	465	19	11	15.05.2007	M	KWPN	97	5	36	47	44	1871	37	F	16	13	no	spread	oxer	straight	no	0	0	77,86	no
Verona	The Longines FEI World Cup Jumping - Verona round A	29.10.2017	CSI-W5*	4	1.40 - 1.60	465	85	13	08.05.2005	M	BWP	270	4	38	10	29	1642	46	M	1	1	no	vertical	vertical	left	no	0	0	75,73	no
Verona	The Longines FEI World Cup Jumping - Verona round A	29.10.2017	CSI-W5*	4	1.40 - 1.60	465	85	13	08.05.2005	M	BWP	270	4	38	10	29	1642	46	M	2	2	no	spread	oxer	left	no	0	0	75,73	no
Verona	The Longines FEI World Cup Jumping - Verona round A	29.10.2017	CSI-W5*	4	1.40 - 1.60	465	85	13	08.05.2005	M	BWP	270	4	38	10	29	1642	46	M	3	3	no	vertical	vertical	right	no	0	0	75,73	no
Verona	The Longines FEI World Cup Jumping - Verona round A	29.10.2017	CSI-W5*	4	1.40 - 1.60	465	85	13	08.05.2005	M	BWP	270	4	38	10	29	1642	46	M	4	4	no	spread	oxer	right	no	0	0	75,73	no
Verona	The Longines FEI World Cup Jumping - Verona round A	29.10.2017	CSI-W5*	4	1.40 - 1.60	465	85	13	08.05.2005	M	BWP	270	4	38	10	29	1642	46	M	5	5	no	vertical	vertical with water	straight	no	0	0	75,73	no
Verona	The Longines FEI World Cup Jumping - Verona round A	29.10.2017	CSI-W5*	4	1.40 - 1.60	465	85	13	08.05.2005	M	BWP	270	4	38	10	29	1642	46	M	6	6	no	spread	oxer with water	left	no	0	0	75,73	no
Verona	The Longines FEI World Cup Jumping - Verona round A	29.10.2017	CSI-W5*	4	1.40 - 1.60	465	85	13	08.05.2005	M	BWP	270	4	38	10	29	1642	46	M	7	7a	yes	spread	oxer	straight	no	0	0	75,73	no
Verona	The Longines FEI World Cup Jumping - Verona round A	29.10.2017	CSI-W5*	4	1.40 - 1.60	465	85	13	08.05.2005	M	BWP	270	4	38	10	29	1642	46	M	8	7b	yes	vertical	vertical	straight	no	0	0	75,73	no
Verona	The Longines FEI World Cup Jumping - Verona round A	29.10.2017	CSI-W5*	4	1.40 - 1.60	465	85	13	08.05.2005	M	BWP	270	4	38	10	29	1642	46	M	9	8	no	vertical	vertical	left	no	0	0	75,73	no
Verona	The Longines FEI World Cup Jumping - Verona round A	29.10.2017	CSI-W5*	4	1.40 - 1.60	465	85	13	08.05.2005	M	BWP	270	4	38	10	29	1642	46	M	10	9	no	spread	oxer	left	no	0	0	75,73	no
Verona	The Longines FEI World Cup Jumping - Verona round A	29.10.2017	CSI-W5*	4	1.40 - 1.60	465	85	13	08.05.2005	M	BWP	270	4	38	10	29	1642	46	M	11	10	no	vertical	vertical	right	no	0	0	75,73	no
Verona	The Longines FEI World Cup Jumping - Verona round A	29.10.2017	CSI-W5*	4	1.40 - 1.60	465	85	13	08.05.2005	M	BWP	270	4	38	10	29	1642	46	M	12	11a	yes	vertical	vertical	right	no	0	0	75,73	no
Verona	The Longines FEI World Cup Jumping - Verona round A	29.10.2017	CSI-W5*	4	1.40 - 1.60	465	85	13	08.05.2005	M	BWP	270	4	38	10	29	1642	46	M	13	11b	yes	spread	oxer	right	no	0	0	75,73	no
Verona	The Longines FEI World Cup Jumping - Verona round A	29.10.2017	CSI-W5*	4	1.40 - 1.60	465	85	13	08.05.2005	M	BWP	270	4	38	10	29	1642	46	M	14	11c	yes	spread	oxer	right	no	0	0	75,73	no
Verona	The Longines FEI World Cup Jumping - Verona round A	29.10.2017	CSI-W5*	4	1.40 - 1.60	465	85	13	08.05.2005	M	BWP	270	4	38	10	29	1642	46	M	15	12	no	vertical	vertical	left	no	0	0	75,73	no
Verona	The Longines FEI World Cup Jumping - Verona round A	29.10.2017	CSI-W5*	4	1.40 - 1.60	465	85	13	08.05.2005	M	BWP	270	4	38	10	29	1642	46	M	16	13	no	spread	oxer	straight	no	0	0	75,73	no
Verona	The Longines FEI World Cup Jumping - Verona round B	29.10.2017	CSI-W5*	4	1.40 - 1.60	290	85	13	08.05.2005	M	BWP	270	4	38	10	29	1642	46	M	1	1	no	vertical	vertical	right	no	0	0	36,17	no
Verona	The Longines FEI World Cup Jumping - Verona round B	29.10.2017	CSI-W5*	4	1.40 - 1.60	290	85	13	08.05.2005	M	BWP	270	4	38	10	29	1642	46	M	2	2	no	spread	oxer	right	no	0	0	36,17	no
Verona	The Longines FEI World Cup Jumping - Verona round B	29.10.2017	CSI-W5*	4	1.40 - 1.60	290	85	13	08.05.2005	M	BWP	270	4	38	10	29	1642	46	M	3	4a	yes	vertical	vertical with water	straight	no	0	0	36,17	no
Verona	The Longines FEI World Cup Jumping - Verona round B	29.10.2017	CSI-W5*	4	1.40 - 1.60	290	85	13	08.05.2005	M	BWP	270	4	38	10	29	1642	46	M	4	4b	yes	spread	oxer	straight	no	0	0	36,17	no
Verona	The Longines FEI World Cup Jumping - Verona round B	29.10.2017	CSI-W5*	4	1.40 - 1.60	290	85	13	08.05.2005	M	BWP	270	4	38	10	29	1642	46	M	5	5	no	vertical	vertical	left	no	0	0	36,17	no
Verona	The Longines FEI World Cup Jumping - Verona round B	29.10.2017	CSI-W5*	4	1.40 - 1.60	290	85	13	08.05.2005	M	BWP	270	4	38	10	29	1642	46	M	6	6	no	spread	oxer	left	no	0	0	36,17	no
Verona	The Longines FEI World Cup Jumping - Verona round B	29.10.2017	CSI-W5*	4	1.40 - 1.60	290	85	13	08.05.2005	M	BWP	270	4	38	10	29	1642	46	M	7	7	no	vertical	vertical	right	no	0	0	36,17	no
Verona	The Longines FEI World Cup Jumping - Verona round B	29.10.2017	CSI-W5*	4	1.40 - 1.60	290	85	13	08.05.2005	M	BWP	270	4	38	10	29	1642	46	M	8	7	no	vertical	vertical	right	no	0	0	36,17	no
Verona	The Longines FEI World Cup Jumping - Verona round A	29.10.2017	CSI-W5*	4	1.40 - 1.60	465	44	11	19.04.2007	S	KWPN	118	1	57	87	63	1783	78	M	1	1	no	vertical	vertical	left	no	0	0	0,00	yes
Verona	The Longines FEI World Cup Jumping - Verona round A	29.10.2017	CSI-W5*	4	1.40 - 1.60	465	44	11	19.04.2007	S	KWPN	118	1	57	87	63	1783	78	M	2	2	no	spread	oxer	left	no	0	0	0,00	yes
Verona	The Longines FEI World Cup Jumping - Verona round A	29.10.2017	CSI-W5*	4	1.40 - 1.60	465	44	11	19.04.2007	S	KWPN	118	1	57	87	63	1783	78	M	3	3	no	vertical	vertical	right	no	0	0	0,00	yes
Verona	The Longines FEI World Cup Jumping - Verona round A	29.10.2017	CSI-W5*	4	1.40 - 1.60	465	44	11	19.04.2007	S	KWPN	118	1	57	87	63	1783	78	M	4	4	no	spread	oxer	right	no	0	0	0,00	yes
Verona	The Longines FEI World Cup Jumping - Verona round A	29.10.2017	CSI-W5*	4	1.40 - 1.60	465	44	11	19.04.2007	S	KWPN	118	1	57	87	63	1783	78	M	5	5	no	vertical	vertical with water	straight	no	0	0	0,00	yes
Verona	The Longines FEI World Cup Jumping - Verona round A	29.10.2017	CSI-W5*	4	1.40 - 1.60	465	44	11	19.04.2007	S	KWPN	118	1	57	87	63	1783	78	M	6	6	no	spread	oxer with water	left	no	0	0	0,00	yes
Verona	The Longines FEI World Cup Jumping - Verona round A	29.10.2017	CSI-W5*	4	1.40 - 1.60	465	44	11	19.04.2007	S	KWPN	118	1	57	87	63	1783	78	M	7	7a	yes	spread	oxer	straight	no	0	0	0,00	yes
Verona	The Longines FEI World Cup Jumping - Verona round A	29.10.2017	CSI-W5*	4	1.40 - 1.60	465	44	11	19.04.2007	S	KWPN	118	1	57	87	63	1783	78	M	8	7b	yes	vertical	vertical	straight	no	0	0	0,00	yes
Verona	The Longines FEI World Cup Jumping - Verona round A	29.10.2017	CSI-W5*	4	1.40 - 1.60	465	44	11	19.04.2007	S	KWPN	118	1	57	87	63	1783	78	M	9	8	no	vertical	vertical	left	no	0	0	0,00	yes
Verona	The Longines FEI World Cup Jumping - Verona round A	29.10.2017	CSI-W5*	4	1.40 - 1.60	465	44	11	19.04.2007	S	KWPN	118	1	57	87	63	1783	78	M	10	9	no	spread	oxer	left	no	0	0	0,00	yes
Verona	The Longines FEI World Cup Jumping - Verona round A	29.10.2017	CSI-W5*	4	1.40 - 1.60	465	44	11	19.04.2007	S	KWPN	118	1	57	87	63	1783	78	M	11	10	no	vertical	vertical	right	no	0	0	0,00	yes
Verona	The Longines FEI World Cup Jumping - Verona round A	29.10.2017	CSI-W5*	4	1.40 - 1.60	465	44	11	19.04.2007	S	KWPN	118	1	57	87	63	1783	78	M	12	11a	yes	vertical	vertical	right	no	0	0	0,00	yes
Verona	The Longines FEI World Cup Jumping - Verona round A	29.10.2017	CSI-W5*	4	1.40 - 1.60	465	44	11	19.04.2007	S	KWPN	118	1	57	87	63	1783	78	M	13	11b	yes	spread	oxer	right	no	0	0	0,00	yes
Verona	The Longines FEI World Cup Jumping - Verona round A	29.10.2017	CSI-W5*	4	1.40 - 1.60	465	44	11	19.04.2007	S	KWPN	118	1	57	87	63	1783	78	M	14	11c	yes	spread	oxer	right	no	0	0	0,00	yes
Verona	The Longines FEI World Cup Jumping - Verona round A	29.10.2017	CSI-W5*	4	1.40 - 1.60	465	44	11	19.04.2007	S	KWPN	118	1	57	87	63	1783	78	M	15	12	no	vertical	vertical	left	no	0	0	0,00	yes
Verona	The Longines FEI World Cup Jumping - Verona round A	29.10.2017	CSI-W5*	4	1.40 - 1.60	465	44	11	19.04.2007	S	KWPN	118	1	57	87	63	1783	78	M	16	13	no	spread	oxer	straight	no	0	0	0,00	yes
Verona	The Longines FEI World Cup Jumping - Verona round A	29.10.2017	CSI-W5*	4	1.40 - 1.60	465	165	14	01.06.2004	S	SF	184	9	58	37	35	1522	63	M	1	1	no	vertical	vertical	left	no	0	0	0,00	yes
Verona	The Longines FEI World Cup Jumping - Verona round A	29.10.2017	CSI-W5*	4	1.40 - 1.60	465	165	14	01.06.2004	S	SF	184	9	58	37	35	1522	63	M	2	2	no	spread	oxer	left	no	0	0	0,00	yes
Verona	The Longines FEI World Cup Jumping - Verona round A	29.10.2017	CSI-W5*	4	1.40 - 1.60	465	165	14	01.06.2004	S	SF	184	9	58	37	35	1522	63	M	3	3	no	vertical	vertical	right	no	0	0	0,00	yes
Verona	The Longines FEI World Cup Jumping - Verona round A	29.10.2017	CSI-W5*	4	1.40 - 1.60	465	165	14	01.06.2004	S	SF	184	9	58	37	35	1522	63	M	4	4	no	spread	oxer	right	no	0	0	0,00	yes
Verona	The Longines FEI World Cup Jumping - Verona round A	29.10.2017	CSI-W5*	4	1.40 - 1.60	465	165	14	01.06.2004	S	SF	184	9	58	37	35	1522	63	M	5	5	no	vertical	vertical with water	straight	no	0	0	0,00	yes
Verona	The Longines FEI World Cup Jumping - Verona round A	29.10.2017	CSI-W5*	4	1.40 - 1.60	465	165	14	01.06.2004	S	SF	184	9	58	37	35	1522	63	M	6	6	no	spread	oxer with water	left	no	0	0	0,00	yes
Verona	The Longines FEI World Cup Jumping - Verona round A	29.10.2017	CSI-W5*	4	1.40 - 1.60	465	165	14	01.06.2004	S	SF	184	9	58	37	35	1522	63	M	7	7a	yes	spread	oxer	straight	no	0	0	0,00	yes
Verona	The Longines FEI World Cup Jumping - Verona round A	29.10.2017	CSI-W5*	4	1.40 - 1.60	465	165	14	01.06.2004	S	SF	184	9	58	37	35	1522	63	M	8	7b	yes	vertical	vertical	straight	no	0	0	0,00	yes
Verona	The Longines FEI World Cup Jumping - Verona round A	29.10.2017	CSI-W5*	4	1.40 - 1.60	465	165	14	01.06.2004	S	SF	184	9	58	37	35	1522	63	M	9	8	no	vertical	vertical	left	no	0	0	0,00	yes
Verona	The Longines FEI World Cup Jumping - Verona round A	29.10.2017	CSI-W5*	4	1.40 - 1.60	465	165	14	01.06.2004	S	SF	184	9	58	37	35	1522	63	M	10	9	no	spread	oxer	left	no	0	0	0,00	yes
Verona	The Longines FEI World Cup Jumping - Verona round A	29.10.2017	CSI-W5*	4	1.40 - 1.60	465	165	14	01.06.2004	S	SF	184	9	58	37	35	1522	63	M	11	10	no	vertical	vertical	right	no	0	0	0,00	yes
Verona	The Longines FEI World Cup Jumping - Verona round A	29.10.2017	CSI-W5*	4	1.40 - 1.60	465	165	14	01.06.2004	S	SF	184	9	58	37	35	1522	63	M	12	11a	yes	vertical	vertical	right	no	0	0	0,00	yes
Verona	The Longines FEI World Cup Jumping - Verona round A	29.10.2017	CSI-W5*	4	1.40 - 1.60	465	165	14	01.06.2004	S	SF	184	9	58	37	35	1522	63	M	13	11b	yes	spread	oxer	right	no	0	0	0,00	yes
Verona	The Longines FEI World Cup Jumping - Verona round A	29.10.2017	CSI-W5*	4	1.40 - 1.60	465	165	14	01.06.2004	S	SF	184	9	58	37	35	1522	63	M	14	11c	yes	spread	oxer	right	no	0	0	0,00	yes
Verona	The Longines FEI World Cup Jumping - Verona round A	29.10.2017	CSI-W5*	4	1.40 - 1.60	465	165	14	01.06.2004	S	SF	184	9	58	37	35	1522	63	M	15	12	no	vertical	vertical	left	no	0	0	0,00	yes
Verona	The Longines FEI World Cup Jumping - Verona round A	29.10.2017	CSI-W5*	4	1.40 - 1.60	465	165	14	01.06.2004	S	SF	184	9	58	37	35	1522	63	M	16	13	no	spread	oxer	straight	no	0	0	0,00	yes
Verona	The Longines FEI World Cup Jumping - Verona round A	29.10.2017	CSI-W5*	4	1.40 - 1.60	465	31	12	15.04.2006	S	OS	292	11	59	57	32	2641	229	M	1	1	no	vertical	vertical	left	no	0	0	87,68	no
Verona	The Longines FEI World Cup Jumping - Verona round A	29.10.2017	CSI-W5*	4	1.40 - 1.60	465	31	12	15.04.2006	S	OS	292	11	59	57	32	2641	229	M	2	2	no	spread	oxer	left	no	0	0	87,68	no
Verona	The Longines FEI World Cup Jumping - Verona round A	29.10.2017	CSI-W5*	4	1.40 - 1.60	465	31	12	15.04.2006	S	OS	292	11	59	57	32	2641	229	M	3	3	no	vertical	vertical	right	no	0	0	87,68	no
Verona	The Longines FEI World Cup Jumping - Verona round A	29.10.2017	CSI-W5*	4	1.40 - 1.60	465	31	12	15.04.2006	S	OS	292	11	59	57	32	2641	229	M	4	4	no	spread	oxer	right	no	0	0	87,68	no
Verona	The Longines FEI World Cup Jumping - Verona round A	29.10.2017	CSI-W5*	4	1.40 - 1.60	465	31	12	15.04.2006	S	OS	292	11	59	57	32	2641	229	M	5	5	no	vertical	vertical with water	straight	no	0	0	87,68	no
Verona	The Longines FEI World Cup Jumping - Verona round A	29.10.2017	CSI-W5*	4	1.40 - 1.60	465	31	12	15.04.2006	S	OS	292	11	59	57	32	2641	229	M	6	6	no	spread	oxer with water	left	no	0	0	87,68	no
Verona	The Longines FEI World Cup Jumping - Verona round A	29.10.2017	CSI-W5*	4	1.40 - 1.60	465	31	12	15.04.2006	S	OS	292	11	59	57	32	2641	229	M	7	7a	yes	spread	oxer	straight	no	0	0	87,68	no
Verona	The Longines FEI World Cup Jumping - Verona round A	29.10.2017	CSI-W5*	4	1.40 - 1.60	465	31	12	15.04.2006	S	OS	292	11	59	57	32	2641	229	M	8	7b	yes	vertical	vertical	straight	no	0	0	87,68	no
Verona	The Longines FEI World Cup Jumping - Verona round A	29.10.2017	CSI-W5*	4	1.40 - 1.60	465	31	12	15.04.2006	S	OS	292	11	59	57	32	2641	229	M	9	8	no	vertical	vertical	left	no	0	0	87,68	no
Verona	The Longines FEI World Cup Jumping - Verona round A	29.10.2017	CSI-W5*	4	1.40 - 1.60	465	31	12	15.04.2006	S	OS	292	11	59	57	32	2641	229	M	10	9	no	spread	oxer	left	no	0	0	87,68	no
Verona	The Longines FEI World Cup Jumping - Verona round A	29.10.2017	CSI-W5*	4	1.40 - 1.60	465	31	12	15.04.2006	S	OS	292	11	59	57	32	2641	229	M	11	10	no	vertical	vertical	right	no	0	0	87,68	no
Verona	The Longines FEI World Cup Jumping - Verona round A	29.10.2017	CSI-W5*	4	1.40 - 1.60	465	31	12	15.04.2006	S	OS	292	11	59	57	32	2641	229	M	12	11a	yes	vertical	vertical	right	no	0	0	87,68	no
Verona	The Longines FEI World Cup Jumping - Verona round A	29.10.2017	CSI-W5*	4	1.40 - 1.60	465	31	12	15.04.2006	S	OS	292	11	59	57	32	2641	229	M	13	11b	yes	spread	oxer	right	no	0	0	87,68	no
Verona	The Longines FEI World Cup Jumping - Verona round A	29.10.2017	CSI-W5*	4	1.40 - 1.60	465	31	12	15.04.2006	S	OS	292	11	59	57	32	2641	229	M	14	11c	yes	spread	oxer	right	no	0	0	87,68	no
Verona	The Longines FEI World Cup Jumping - Verona round A	29.10.2017	CSI-W5*	4	1.40 - 1.60	465	31	12	15.04.2006	S	OS	292	11	59	57	32	2641	229	M	15	12	no	vertical	vertical	left	no	0	0	87,68	no
Verona	The Longines FEI World Cup Jumping - Verona round A	29.10.2017	CSI-W5*	4	1.40 - 1.60	465	31	12	15.04.2006	S	OS	292	11	59	57	32	2641	229	M	16	13	no	spread	oxer	straight	no	0	0	87,68	no
Verona	The Longines FEI World Cup Jumping - Verona round A	29.10.2017	CSI-W5*	4	1.40 - 1.60	465	153	12	01.01.2006	G	ISH	145	4	60	502	33	802	0	F	1	1	no	vertical	vertical	left	no	0	0	82,39	no
Verona	The Longines FEI World Cup Jumping - Verona round A	29.10.2017	CSI-W5*	4	1.40 - 1.60	465	153	12	01.01.2006	G	ISH	145	4	60	502	33	802	0	F	2	2	no	spread	oxer	left	no	0	0	82,39	no
Verona	The Longines FEI World Cup Jumping - Verona round A	29.10.2017	CSI-W5*	4	1.40 - 1.60	465	153	12	01.01.2006	G	ISH	145	4	60	502	33	802	0	F	3	3	no	vertical	vertical	right	no	0	0	82,39	no
Verona	The Longines FEI World Cup Jumping - Verona round A	29.10.2017	CSI-W5*	4	1.40 - 1.60	465	153	12	01.01.2006	G	ISH	145	4	60	502	33	802	0	F	4	4	no	spread	oxer	right	no	0	0	82,39	no
Verona	The Longines FEI World Cup Jumping - Verona round A	29.10.2017	CSI-W5*	4	1.40 - 1.60	465	153	12	01.01.2006	G	ISH	145	4	60	502	33	802	0	F	5	5	no	vertical	vertical with water	straight	no	0	0	82,39	no
Verona	The Longines FEI World Cup Jumping - Verona round A	29.10.2017	CSI-W5*	4	1.40 - 1.60	465	153	12	01.01.2006	G	ISH	145	4	60	502	33	802	0	F	6	6	no	spread	oxer with water	left	no	0	0	82,39	no
Verona	The Longines FEI World Cup Jumping - Verona round A	29.10.2017	CSI-W5*	4	1.40 - 1.60	465	153	12	01.01.2006	G	ISH	145	4	60	502	33	802	0	F	7	7a	yes	spread	oxer	straight	no	0	0	82,39	no
Verona	The Longines FEI World Cup Jumping - Verona round A	29.10.2017	CSI-W5*	4	1.40 - 1.60	465	153	12	01.01.2006	G	ISH	145	4	60	502	33	802	0	F	8	7b	yes	vertical	vertical	straight	no	0	0	82,39	no
Verona	The Longines FEI World Cup Jumping - Verona round A	29.10.2017	CSI-W5*	4	1.40 - 1.60	465	153	12	01.01.2006	G	ISH	145	4	60	502	33	802	0	F	9	8	no	vertical	vertical	left	no	0	0	82,39	no
Verona	The Longines FEI World Cup Jumping - Verona round A	29.10.2017	CSI-W5*	4	1.40 - 1.60	465	153	12	01.01.2006	G	ISH	145	4	60	502	33	802	0	F	10	9	no	spread	oxer	left	no	0	0	82,39	no
Verona	The Longines FEI World Cup Jumping - Verona round A	29.10.2017	CSI-W5*	4	1.40 - 1.60	465	153	12	01.01.2006	G	ISH	145	4	60	502	33	802	0	F	11	10	no	vertical	vertical	right	no	0	0	82,39	no
Verona	The Longines FEI World Cup Jumping - Verona round A	29.10.2017	CSI-W5*	4	1.40 - 1.60	465	153	12	01.01.2006	G	ISH	145	4	60	502	33	802	0	F	12	11a	yes	vertical	vertical	right	no	0	0	82,39	no
Verona	The Longines FEI World Cup Jumping - Verona round A	29.10.2017	CSI-W5*	4	1.40 - 1.60	465	153	12	01.01.2006	G	ISH	145	4	60	502	33	802	0	F	13	11b	yes	spread	oxer	right	no	0	0	82,39	no
Verona	The Longines FEI World Cup Jumping - Verona round A	29.10.2017	CSI-W5*	4	1.40 - 1.60	465	153	12	01.01.2006	G	ISH	145	4	60	502	33	802	0	F	14	11c	yes	spread	oxer	right	no	0	0	82,39	no
Verona	The Longines FEI World Cup Jumping - Verona round A	29.10.2017	CSI-W5*	4	1.40 - 1.60	465	153	12	01.01.2006	G	ISH	145	4	60	502	33	802	0	F	15	12	no	vertical	vertical	left	no	0	0	82,39	no
Verona	The Longines FEI World Cup Jumping - Verona round A	29.10.2017	CSI-W5*	4	1.40 - 1.60	465	153	12	01.01.2006	G	ISH	145	4	60	502	33	802	0	F	16	13	no	spread	oxer	straight	no	0	1	82,39	no
Verona	The Longines FEI World Cup Jumping - Verona round A	29.10.2017	CSI-W5*	4	1.40 - 1.60	465	68	15	30.06.2003	G	BWP	209	4	61	297	35	1222	40	F	1	1	no	vertical	vertical	left	no	0	0	79,96	no
Verona	The Longines FEI World Cup Jumping - Verona round A	29.10.2017	CSI-W5*	4	1.40 - 1.60	465	68	15	30.06.2003	G	BWP	209	4	61	297	35	1222	40	F	2	2	no	spread	oxer	left	no	0	0	79,96	no
Verona	The Longines FEI World Cup Jumping - Verona round A	29.10.2017	CSI-W5*	4	1.40 - 1.60	465	68	15	30.06.2003	G	BWP	209	4	61	297	35	1222	40	F	3	3	no	vertical	vertical	right	no	0	0	79,96	no
Verona	The Longines FEI World Cup Jumping - Verona round A	29.10.2017	CSI-W5*	4	1.40 - 1.60	465	68	15	30.06.2003	G	BWP	209	4	61	297	35	1222	40	F	4	4	no	spread	oxer	right	no	0	0	79,96	no
Verona	The Longines FEI World Cup Jumping - Verona round A	29.10.2017	CSI-W5*	4	1.40 - 1.60	465	68	15	30.06.2003	G	BWP	209	4	61	297	35	1222	40	F	5	5	no	vertical	vertical with water	straight	no	0	0	79,96	no
Verona	The Longines FEI World Cup Jumping - Verona round A	29.10.2017	CSI-W5*	4	1.40 - 1.60	465	68	15	30.06.2003	G	BWP	209	4	61	297	35	1222	40	F	6	6	no	spread	oxer with water	left	yes	4	0	79,96	no
Verona	The Longines FEI World Cup Jumping - Verona round A	29.10.2017	CSI-W5*	4	1.40 - 1.60	465	68	15	30.06.2003	G	BWP	209	4	61	297	35	1222	40	F	7	7a	yes	spread	oxer	straight	no	0	0	79,96	no
Verona	The Longines FEI World Cup Jumping - Verona round A	29.10.2017	CSI-W5*	4	1.40 - 1.60	465	68	15	30.06.2003	G	BWP	209	4	61	297	35	1222	40	F	8	7b	yes	vertical	vertical	straight	no	0	0	79,96	no
Verona	The Longines FEI World Cup Jumping - Verona round A	29.10.2017	CSI-W5*	4	1.40 - 1.60	465	68	15	30.06.2003	G	BWP	209	4	61	297	35	1222	40	F	9	8	no	vertical	vertical	left	no	0	0	79,96	no
Verona	The Longines FEI World Cup Jumping - Verona round A	29.10.2017	CSI-W5*	4	1.40 - 1.60	465	68	15	30.06.2003	G	BWP	209	4	61	297	35	1222	40	F	10	9	no	spread	oxer	left	no	0	0	79,96	no
Verona	The Longines FEI World Cup Jumping - Verona round A	29.10.2017	CSI-W5*	4	1.40 - 1.60	465	68	15	30.06.2003	G	BWP	209	4	61	297	35	1222	40	F	11	10	no	vertical	vertical	right	no	0	0	79,96	no
Verona	The Longines FEI World Cup Jumping - Verona round A	29.10.2017	CSI-W5*	4	1.40 - 1.60	465	68	15	30.06.2003	G	BWP	209	4	61	297	35	1222	40	F	12	11a	yes	vertical	vertical	right	yes	4	0	79,96	no
Verona	The Longines FEI World Cup Jumping - Verona round A	29.10.2017	CSI-W5*	4	1.40 - 1.60	465	68	15	30.06.2003	G	BWP	209	4	61	297	35	1222	40	F	13	11b	yes	spread	oxer	right	no	0	0	79,96	no
Verona	The Longines FEI World Cup Jumping - Verona round A	29.10.2017	CSI-W5*	4	1.40 - 1.60	465	68	15	30.06.2003	G	BWP	209	4	61	297	35	1222	40	F	14	11c	yes	spread	oxer	right	yes	4	0	79,96	no
Verona	The Longines FEI World Cup Jumping - Verona round A	29.10.2017	CSI-W5*	4	1.40 - 1.60	465	68	15	30.06.2003	G	BWP	209	4	61	297	35	1222	40	F	15	12	no	vertical	vertical	left	no	0	0	79,96	no
Verona	The Longines FEI World Cup Jumping - Verona round A	29.10.2017	CSI-W5*	4	1.40 - 1.60	465	68	15	30.06.2003	G	BWP	209	4	61	297	35	1222	40	F	16	13	no	spread	oxer	straight	no	0	0	79,96	no
Verona	The Longines FEI World Cup Jumping - Verona round A	29.10.2017	CSI-W5*	4	1.40 - 1.60	465	97	12	25.04.2006	S	BWP	176	11	62	146	51	33	0	M	1	1	no	vertical	vertical	left	no	0	0	79,63	no
Verona	The Longines FEI World Cup Jumping - Verona round A	29.10.2017	CSI-W5*	4	1.40 - 1.60	465	97	12	25.04.2006	S	BWP	176	11	62	146	51	33	0	M	2	2	no	spread	oxer	left	no	0	0	79,63	no
Verona	The Longines FEI World Cup Jumping - Verona round A	29.10.2017	CSI-W5*	4	1.40 - 1.60	465	97	12	25.04.2006	S	BWP	176	11	62	146	51	33	0	M	3	3	no	vertical	vertical	right	no	0	0	79,63	no
Verona	The Longines FEI World Cup Jumping - Verona round A	29.10.2017	CSI-W5*	4	1.40 - 1.60	465	97	12	25.04.2006	S	BWP	176	11	62	146	51	33	0	M	4	4	no	spread	oxer	right	no	0	0	79,63	no
Verona	The Longines FEI World Cup Jumping - Verona round A	29.10.2017	CSI-W5*	4	1.40 - 1.60	465	97	12	25.04.2006	S	BWP	176	11	62	146	51	33	0	M	5	5	no	vertical	vertical with water	straight	no	0	0	79,63	no
Verona	The Longines FEI World Cup Jumping - Verona round A	29.10.2017	CSI-W5*	4	1.40 - 1.60	465	97	12	25.04.2006	S	BWP	176	11	62	146	51	33	0	M	6	6	no	spread	oxer with water	left	no	0	0	79,63	no
Verona	The Longines FEI World Cup Jumping - Verona round A	29.10.2017	CSI-W5*	4	1.40 - 1.60	465	97	12	25.04.2006	S	BWP	176	11	62	146	51	33	0	M	7	7a	yes	spread	oxer	straight	no	0	0	79,63	no
Verona	The Longines FEI World Cup Jumping - Verona round A	29.10.2017	CSI-W5*	4	1.40 - 1.60	465	97	12	25.04.2006	S	BWP	176	11	62	146	51	33	0	M	8	7b	yes	vertical	vertical	straight	no	0	0	79,63	no
Verona	The Longines FEI World Cup Jumping - Verona round A	29.10.2017	CSI-W5*	4	1.40 - 1.60	465	97	12	25.04.2006	S	BWP	176	11	62	146	51	33	0	M	9	8	no	vertical	vertical	left	no	0	0	79,63	no
Verona	The Longines FEI World Cup Jumping - Verona round A	29.10.2017	CSI-W5*	4	1.40 - 1.60	465	97	12	25.04.2006	S	BWP	176	11	62	146	51	33	0	M	10	9	no	spread	oxer	left	no	0	0	79,63	no
Verona	The Longines FEI World Cup Jumping - Verona round A	29.10.2017	CSI-W5*	4	1.40 - 1.60	465	97	12	25.04.2006	S	BWP	176	11	62	146	51	33	0	M	11	10	no	vertical	vertical	right	no	0	0	79,63	no
Verona	The Longines FEI World Cup Jumping - Verona round A	29.10.2017	CSI-W5*	4	1.40 - 1.60	465	97	12	25.04.2006	S	BWP	176	11	62	146	51	33	0	M	12	11a	yes	vertical	vertical	right	no	0	0	79,63	no
Verona	The Longines FEI World Cup Jumping - Verona round A	29.10.2017	CSI-W5*	4	1.40 - 1.60	465	97	12	25.04.2006	S	BWP	176	11	62	146	51	33	0	M	13	11b	yes	spread	oxer	right	no	0	0	79,63	no
Verona	The Longines FEI World Cup Jumping - Verona round A	29.10.2017	CSI-W5*	4	1.40 - 1.60	465	97	12	25.04.2006	S	BWP	176	11	62	146	51	33	0	M	14	11c	yes	spread	oxer	right	no	0	0	79,63	no
Verona	The Longines FEI World Cup Jumping - Verona round A	29.10.2017	CSI-W5*	4	1.40 - 1.60	465	97	12	25.04.2006	S	BWP	176	11	62	146	51	33	0	M	15	12	no	vertical	vertical	left	no	0	0	79,63	no
Verona	The Longines FEI World Cup Jumping - Verona round A	29.10.2017	CSI-W5*	4	1.40 - 1.60	465	97	12	25.04.2006	S	BWP	176	11	62	146	51	33	0	M	16	13	no	spread	oxer	straight	no	0	0	79,63	no
Verona	The Longines FEI World Cup Jumping - Verona round A	29.10.2017	CSI-W5*	4	1.40 - 1.60	465	186	11	04.06.2007	G	BWP	123	2	63	25	39	1079	61	M	1	1	no	vertical	vertical	left	no	0	0	79,20	no
Verona	The Longines FEI World Cup Jumping - Verona round A	29.10.2017	CSI-W5*	4	1.40 - 1.60	465	186	11	04.06.2007	G	BWP	123	2	63	25	39	1079	61	M	2	2	no	spread	oxer	left	no	0	0	79,20	no
Verona	The Longines FEI World Cup Jumping - Verona round A	29.10.2017	CSI-W5*	4	1.40 - 1.60	465	186	11	04.06.2007	G	BWP	123	2	63	25	39	1079	61	M	3	3	no	vertical	vertical	right	no	0	0	79,20	no
Verona	The Longines FEI World Cup Jumping - Verona round A	29.10.2017	CSI-W5*	4	1.40 - 1.60	465	186	11	04.06.2007	G	BWP	123	2	63	25	39	1079	61	M	4	4	no	spread	oxer	right	no	0	0	79,20	no
Verona	The Longines FEI World Cup Jumping - Verona round A	29.10.2017	CSI-W5*	4	1.40 - 1.60	465	186	11	04.06.2007	G	BWP	123	2	63	25	39	1079	61	M	5	5	no	vertical	vertical with water	straight	no	0	0	79,20	no
Verona	The Longines FEI World Cup Jumping - Verona round A	29.10.2017	CSI-W5*	4	1.40 - 1.60	465	186	11	04.06.2007	G	BWP	123	2	63	25	39	1079	61	M	6	6	no	spread	oxer with water	left	no	0	0	79,20	no
Verona	The Longines FEI World Cup Jumping - Verona round A	29.10.2017	CSI-W5*	4	1.40 - 1.60	465	186	11	04.06.2007	G	BWP	123	2	63	25	39	1079	61	M	7	7a	yes	spread	oxer	straight	no	0	0	79,20	no
Verona	The Longines FEI World Cup Jumping - Verona round A	29.10.2017	CSI-W5*	4	1.40 - 1.60	465	186	11	04.06.2007	G	BWP	123	2	63	25	39	1079	61	M	8	7b	yes	vertical	vertical	straight	no	0	0	79,20	no
Verona	The Longines FEI World Cup Jumping - Verona round A	29.10.2017	CSI-W5*	4	1.40 - 1.60	465	186	11	04.06.2007	G	BWP	123	2	63	25	39	1079	61	M	9	8	no	vertical	vertical	left	no	0	0	79,20	no
Verona	The Longines FEI World Cup Jumping - Verona round A	29.10.2017	CSI-W5*	4	1.40 - 1.60	465	186	11	04.06.2007	G	BWP	123	2	63	25	39	1079	61	M	10	9	no	spread	oxer	left	no	0	0	79,20	no
Verona	The Longines FEI World Cup Jumping - Verona round A	29.10.2017	CSI-W5*	4	1.40 - 1.60	465	186	11	04.06.2007	G	BWP	123	2	63	25	39	1079	61	M	11	10	no	vertical	vertical	right	no	0	0	79,20	no
Verona	The Longines FEI World Cup Jumping - Verona round A	29.10.2017	CSI-W5*	4	1.40 - 1.60	465	186	11	04.06.2007	G	BWP	123	2	63	25	39	1079	61	M	12	11a	yes	vertical	vertical	right	no	0	0	79,20	no
Verona	The Longines FEI World Cup Jumping - Verona round A	29.10.2017	CSI-W5*	4	1.40 - 1.60	465	186	11	04.06.2007	G	BWP	123	2	63	25	39	1079	61	M	13	11b	yes	spread	oxer	right	no	0	0	79,20	no
Verona	The Longines FEI World Cup Jumping - Verona round A	29.10.2017	CSI-W5*	4	1.40 - 1.60	465	186	11	04.06.2007	G	BWP	123	2	63	25	39	1079	61	M	14	11c	yes	spread	oxer	right	no	0	0	79,20	no
Verona	The Longines FEI World Cup Jumping - Verona round A	29.10.2017	CSI-W5*	4	1.40 - 1.60	465	186	11	04.06.2007	G	BWP	123	2	63	25	39	1079	61	M	15	12	no	vertical	vertical	left	no	0	0	79,20	no
Verona	The Longines FEI World Cup Jumping - Verona round A	29.10.2017	CSI-W5*	4	1.40 - 1.60	465	186	11	04.06.2007	G	BWP	123	2	63	25	39	1079	61	M	16	13	no	spread	oxer	straight	no	0	0	79,20	no
Verona	The Longines FEI World Cup Jumping - Verona round B	29.10.2017	CSI-W5*	4	1.40 - 1.60	290	186	11	04.06.2007	G	BWP	123	2	63	25	39	1079	61	M	1	1	no	vertical	vertical	right	no	0	0	55,58	no
Verona	The Longines FEI World Cup Jumping - Verona round B	29.10.2017	CSI-W5*	4	1.40 - 1.60	290	186	11	04.06.2007	G	BWP	123	2	63	25	39	1079	61	M	2	2	no	spread	oxer	right	yes	4	0	55,58	no
Verona	The Longines FEI World Cup Jumping - Verona round B	29.10.2017	CSI-W5*	4	1.40 - 1.60	290	186	11	04.06.2007	G	BWP	123	2	63	25	39	1079	61	M	3	4a	yes	vertical	vertical with water	straight	no	0	0	55,58	no
Verona	The Longines FEI World Cup Jumping - Verona round B	29.10.2017	CSI-W5*	4	1.40 - 1.60	290	186	11	04.06.2007	G	BWP	123	2	63	25	39	1079	61	M	4	4b	yes	spread	oxer	straight	no	0	0	55,58	no
Verona	The Longines FEI World Cup Jumping - Verona round B	29.10.2017	CSI-W5*	4	1.40 - 1.60	290	186	11	04.06.2007	G	BWP	123	2	63	25	39	1079	61	M	5	5	no	vertical	vertical	left	no	0	0	55,58	no
Verona	The Longines FEI World Cup Jumping - Verona round B	29.10.2017	CSI-W5*	4	1.40 - 1.60	290	186	11	04.06.2007	G	BWP	123	2	63	25	39	1079	61	M	6	6	no	spread	oxer	left	no	0	0	55,58	no
Verona	The Longines FEI World Cup Jumping - Verona round B	29.10.2017	CSI-W5*	4	1.40 - 1.60	290	186	11	04.06.2007	G	BWP	123	2	63	25	39	1079	61	M	7	7	no	vertical	vertical	right	no	0	0	55,58	no

Verona	The Longines FEI World Cup Jumping - Verona round B	29.10.2017	CSI-W5*	4	1.40 - 1.60	290	186	11	04.06.2007	G	BWP	123	2	63	25	39	1079	61	M	8	7	no	vertical	vertical	right	no	0	6	55,58	no
Verona	The Longines FEI World Cup Jumping - Verona round A	29.10.2017	CSI-W5*	4	1.40 - 1.60	465	43	15	20.05.2003	S	DSP	218	12	64	20	44	2014	118	M	1	1	no	vertical	vertical	left	no	0	0	78,32	no
Verona	The Longines FEI World Cup Jumping - Verona round A	29.10.2017	CSI-W5*	4	1.40 - 1.60	465	43	15	20.05.2003	S	DSP	218	12	64	20	44	2014	118	M	2	2	no	spread	oxer	left	no	0	0	78,32	no
Verona	The Longines FEI World Cup Jumping - Verona round A	29.10.2017	CSI-W5*	4	1.40 - 1.60	465	43	15	20.05.2003	S	DSP	218	12	64	20	44	2014	118	M	3	3	no	vertical	vertical	right	no	0	0	78,32	no
Verona	The Longines FEI World Cup Jumping - Verona round A	29.10.2017	CSI-W5*	4	1.40 - 1.60	465	43	15	20.05.2003	S	DSP	218	12	64	20	44	2014	118	M	4	4	no	spread	oxer	right	no	0	0	78,32	no
Verona	The Longines FEI World Cup Jumping - Verona round A	29.10.2017	CSI-W5*	4	1.40 - 1.60	465	43	15	20.05.2003	S	DSP	218	12	64	20	44	2014	118	M	5	5	no	vertical	vertical with water	straight	no	0	0	78,32	no
Verona	The Longines FEI World Cup Jumping - Verona round A	29.10.2017	CSI-W5*	4	1.40 - 1.60	465	43	15	20.05.2003	S	DSP	218	12	64	20	44	2014	118	M	6	6	no	spread	oxer with water	left	no	0	0	78,32	no
Verona	The Longines FEI World Cup Jumping - Verona round A	29.10.2017	CSI-W5*	4	1.40 - 1.60	465	43	15	20.05.2003	S	DSP	218	12	64	20	44	2014	118	M	7	7a	yes	spread	oxer	straight	no	0	0	78,32	no
Verona	The Longines FEI World Cup Jumping - Verona round A	29.10.2017	CSI-W5*	4	1.40 - 1.60	465	43	15	20.05.2003	S	DSP	218	12	64	20	44	2014	118	M	8	7b	yes	vertical	vertical	straight	no	0	0	78,32	no
Verona	The Longines FEI World Cup Jumping - Verona round A	29.10.2017	CSI-W5*	4	1.40 - 1.60	465	43	15	20.05.2003	S	DSP	218	12	64	20	44	2014	118	M	9	8	no	vertical	vertical	left	no	0	0	78,32	no
Verona	The Longines FEI World Cup Jumping - Verona round A	29.10.2017	CSI-W5*	4	1.40 - 1.60	465	43	15	20.05.2003	S	DSP	218	12	64	20	44	2014	118	M	10	9	no	spread	oxer	left	yes	4	0	78,32	no
Verona	The Longines FEI World Cup Jumping - Verona round A	29.10.2017	CSI-W5*	4	1.40 - 1.60	465	43	15	20.05.2003	S	DSP	218	12	64	20	44	2014	118	M	11	10	no	vertical	vertical	right	no	0	0	78,32	no
Verona	The Longines FEI World Cup Jumping - Verona round A	29.10.2017	CSI-W5*	4	1.40 - 1.60	465	43	15	20.05.2003	S	DSP	218	12	64	20	44	2014	118	M	12	11a	yes	vertical	vertical	right	no	0	0	78,32	no
Verona	The Longines FEI World Cup Jumping - Verona round A	29.10.2017	CSI-W5*	4	1.40 - 1.60	465	43	15	20.05.2003	S	DSP	218	12	64	20	44	2014	118	M	13	11b	yes	spread	oxer	right	no	0	0	78,32	no
Verona	The Longines FEI World Cup Jumping - Verona round A	29.10.2017	CSI-W5*	4	1.40 - 1.60	465	43	15	20.05.2003	S	DSP	218	12	64	20	44	2014	118	M	14	11c	yes	spread	oxer	right	no	0	0	78,32	no
Verona	The Longines FEI World Cup Jumping - Verona round A	29.10.2017	CSI-W5*	4	1.40 - 1.60	465	43	15	20.05.2003	S	DSP	218	12	64	20	44	2014	118	M	15	12	no	vertical	vertical	left	no	0	0	78,32	no
Verona	The Longines FEI World Cup Jumping - Verona round A	29.10.2017	CSI-W5*	4	1.40 - 1.60	465	43	15	20.05.2003	S	DSP	218	12	64	20	44	2014	118	M	16	13	no	spread	oxer	straight	no	0	0	78,32	no
Verona	The Longines FEI World Cup Jumping - Verona round A	29.10.2017	CSI-W5*	4	1.40 - 1.60	465	71	10	25.05.2008	M	KWPN	131	2	65	70	43	1682	26	M	1	1	no	vertical	vertical	left	no	0	0	76,16	no
Verona	The Longines FEI World Cup Jumping - Verona round A	29.10.2017	CSI-W5*	4	1.40 - 1.60	465	71	10	25.05.2008	M	KWPN	131	2	65	70	43	1682	26	M	2	2	no	spread	oxer	left	no	0	0	76,16	no
Verona	The Longines FEI World Cup Jumping - Verona round A	29.10.2017	CSI-W5*	4	1.40 - 1.60	465	71	10	25.05.2008	M	KWPN	131	2	65	70	43	1682	26	M	3	3	no	vertical	vertical	right	no	0	0	76,16	no
Verona	The Longines FEI World Cup Jumping - Verona round A	29.10.2017	CSI-W5*	4	1.40 - 1.60	465	71	10	25.05.2008	M	KWPN	131	2	65	70	43	1682	26	M	4	4	no	spread	oxer	right	no	0	0	76,16	no
Verona	The Longines FEI World Cup Jumping - Verona round A	29.10.2017	CSI-W5*	4	1.40 - 1.60	465	71	10	25.05.2008	M	KWPN	131	2	65	70	43	1682	26	M	5	5	no	vertical	vertical with water	straight	no	0	0	76,16	no
Verona	The Longines FEI World Cup Jumping - Verona round A	29.10.2017	CSI-W5*	4	1.40 - 1.60	465	71	10	25.05.2008	M	KWPN	131	2	65	70	43	1682	26	M	6	6	no	spread	oxer with water	left	no	0	0	76,16	no
Verona	The Longines FEI World Cup Jumping - Verona round A	29.10.2017	CSI-W5*	4	1.40 - 1.60	465	71	10	25.05.2008	M	KWPN	131	2	65	70	43	1682	26	M	7	7a	yes	spread	oxer	straight	no	0	0	76,16	no
Verona	The Longines FEI World Cup Jumping - Verona round A	29.10.2017	CSI-W5*	4	1.40 - 1.60	465	71	10	25.05.2008	M	KWPN	131	2	65	70	43	1682	26	M	8	7b	yes	vertical	vertical	straight	no	0	0	76,16	no
Verona	The Longines FEI World Cup Jumping - Verona round A	29.10.2017	CSI-W5*	4	1.40 - 1.60	465	71	10	25.05.2008	M	KWPN	131	2	65	70	43	1682	26	M	9	8	no	vertical	vertical	left	no	0	0	76,16	no
Verona	The Longines FEI World Cup Jumping - Verona round A	29.10.2017	CSI-W5*	4	1.40 - 1.60	465	71	10	25.05.2008	M	KWPN	131	2	65	70	43	1682	26	M	10	9	no	spread	oxer	left	no	0	0	76,16	no
Verona	The Longines FEI World Cup Jumping - Verona round A	29.10.2017	CSI-W5*	4	1.40 - 1.60	465	71	10	25.05.2008	M	KWPN	131	2	65	70	43	1682	26	M	11	10	no	vertical	vertical	right	no	0	0	76,16	no
Verona	The Longines FEI World Cup Jumping - Verona round A	29.10.2017	CSI-W5*	4	1.40 - 1.60	465	71	10	25.05.2008	M	KWPN	131	2	65	70	43	1682	26	M	12	11a	yes	vertical	vertical	right	no	0	0	76,16	no
Verona	The Longines FEI World Cup Jumping - Verona round A	29.10.2017	CSI-W5*	4	1.40 - 1.60	465	71	10	25.05.2008	M	KWPN	131	2	65	70	43	1682	26	M	13	11b	yes	spread	oxer	right	no	0	0	76,16	no
Verona	The Longines FEI World Cup Jumping - Verona round A	29.10.2017	CSI-W5*	4	1.40 - 1.60	465	71	10	25.05.2008	M	KWPN	131	2	65	70	43	1682	26	M	14	11c	yes	spread	oxer	right	no	0	0	76,16	no
Verona	The Longines FEI World Cup Jumping - Verona round A	29.10.2017	CSI-W5*	4	1.40 - 1.60	465	71	10	25.05.2008	M	KWPN	131	2	65	70	43	1682	26	M	15	12	no	vertical	vertical	left	no	0	0	76,16	no
Verona	The Longines FEI World Cup Jumping - Verona round A	29.10.2017	CSI-W5*	4	1.40 - 1.60	465	71	10	25.05.2008	M	KWPN	131	2	65	70	43	1682	26	M	16	13	no	spread	oxer	straight	no	0	0	76,16	no
Verona	The Longines FEI World Cup Jumping - Verona round A	29.10.2017	CSI-W5*	4	1.40 - 1.60	465	201	11	22.03.2007	S	SF	102	4	66	243	39	595	18	M	1	1	no	vertical	vertical	left	no	0	0	78,23	no
Verona	The Longines FEI World Cup Jumping - Verona round A	29.10.2017	CSI-W5*	4	1.40 - 1.60	465	201	11	22.03.2007	S	SF	102	4	66	243	39	595	18	M	2	2	no	spread	oxer	left	no	0	0	78,23	no
Verona	The Longines FEI World Cup Jumping - Verona round A	29.10.2017	CSI-W5*	4	1.40 - 1.60	465	201	11	22.03.2007	S	SF	102	4	66	243	39	595	18	M	3	3	no	vertical	vertical	right	no	0	0	78,23	no
Verona	The Longines FEI World Cup Jumping - Verona round A	29.10.2017	CSI-W5*	4	1.40 - 1.60	465	201	11	22.03.2007	S	SF	102	4	66	243	39	595	18	M	4	4	no	spread	oxer	right	no	0	0	78,23	no
Verona	The Longines FEI World Cup Jumping - Verona round A	29.10.2017	CSI-W5*	4	1.40 - 1.60	465	201	11	22.03.2007	S	SF	102	4	66	243	39	595	18	M	5	5	no	vertical	vertical with water	straight	no	0	0	78,23	no
Verona	The Longines FEI World Cup Jumping - Verona round A	29.10.2017	CSI-W5*	4	1.40 - 1.60	465	201	11	22.03.2007	S	SF	102	4	66	243	39	595	18	M	6	6	no	spread	oxer with water	left	no	0	0	78,23	no
Verona	The Longines FEI World Cup Jumping - Verona round A	29.10.2017	CSI-W5*	4	1.40 - 1.60	465	201	11	22.03.2007	S	SF	102	4	66	243	39	595	18	M	7	7a	yes	spread	oxer	straight	no	0	0	78,23	no
Verona	The Longines FEI World Cup Jumping - Verona round A	29.10.2017	CSI-W5*	4	1.40 - 1.60	465	201	11	22.03.2007	S	SF	102	4	66	243	39	595	18	M	8	7b	yes	vertical	vertical	straight	no	0	0	78,23	no
Verona	The Longines FEI World Cup Jumping - Verona round A	29.10.2017	CSI-W5*	4	1.40 - 1.60	465	201	11	22.03.2007	S	SF	102	4	66	243	39	595	18	M	9	8	no	vertical	vertical	left	no	0	0	78,23	no
Verona	The Longines FEI World Cup Jumping - Verona round A	29.10.2017	CSI-W5*	4	1.40 - 1.60	465	201	11	22.03.2007	S	SF	102	4	66	243	39	595	18	M	10	9	no	spread	oxer	left	no	0	0	78,23	no
Verona	The Longines FEI World Cup Jumping - Verona round A	29.10.2017	CSI-W5*	4	1.40 - 1.60	465	201	11	22.03.2007	S	SF	102	4	66	243	39	595	18	M	11	10	no	vertical	vertical	right	no	0	0	78,23	no
Verona	The Longines FEI World Cup Jumping - Verona round A	29.10.2017	CSI-W5*	4	1.40 - 1.60	465	201	11	22.03.2007	S	SF	102	4	66	243	39	595	18	M	12	11a	yes	vertical	vertical	right	no	0	0	78,23	no
Verona	The Longines FEI World Cup Jumping - Verona round A	29.10.2017	CSI-W5*	4	1.40 - 1.60	465	201	11	22.03.2007	S	SF	102	4	66	243	39	595	18	M	13	11b	yes	spread	oxer	right	no	0	0	78,23	no
Verona	The Longines FEI World Cup Jumping - Verona round A	29.10.2017	CSI-W5*	4	1.40 - 1.60	465	201	11	22.03.2007	S	SF	102	4	66	243	39	595	18	M	14	11c	yes	spread	oxer	right	no	0	0	78,23	no
Verona	The Longines FEI World Cup Jumping - Verona round A	29.10.2017	CSI-W5*	4	1.40 - 1.60	465	201	11	22.03.2007	S	SF	102	4	66	243	39	595	18	M	15	12	no	vertical	vertical	left	no	0	0	78,23	no
Verona	The Longines FEI World Cup Jumping - Verona round A	29.10.2017	CSI-W5*	4	1.40 - 1.60	465	201	11	22.03.2007	S	SF	102	4	66	243	39	595	18	M	16	13	no	spread	oxer	straight	no	0	0	78,23	no
Verona	The Longines FEI World Cup Jumping - Verona round A	29.10.2017	CSI-W5*	4	1.40 - 1.60	465	188	11	06.06.2007	G	KWPN	209	10	67	60	47	2033	59	M	1	1	no	vertical	vertical	left	no	0	0	78,07	no
Verona	The Longines FEI World Cup Jumping - Verona round A	29.10.2017	CSI-W5*	4	1.40 - 1.60	465	188	11	06.06.2007	G	KWPN	209	10	67	60	47	2033	59	M	2	2	no	spread	oxer	left	yes	4	0	78,07	no
Verona	The Longines FEI World Cup Jumping - Verona round A	29.10.2017	CSI-W5*	4	1.40 - 1.60	465	188	11	06.06.2007	G	KWPN	209	10	67	60	47	2033	59	M	3	3	no	vertical	vertical	right	no	0	0	78,07	no
Verona	The Longines FEI World Cup Jumping - Verona round A	29.10.2017	CSI-W5*	4	1.40 - 1.60	465	188	11	06.06.2007	G	KWPN	209	10	67	60	47	2033	59	M	4	4	no	spread	oxer	right	no	0	0	78,07	no
Verona	The Longines FEI World Cup Jumping - Verona round A	29.10.2017	CSI-W5*	4	1.40 - 1.60	465	188	11	06.06.2007	G	KWPN	209	10	67	60	47	2033	59	M	5	5	no	vertical	vertical with water	straight	no	0	0	78,07	no
Verona	The Longines FEI World Cup Jumping - Verona round A	29.10.2017	CSI-W5*	4	1.40 - 1.60	465	188	11	06.06.2007	G	KWPN	209	10	67	60	47	2033	59	M	6	6	no	spread	oxer with water	left	no	0	0	78,07	no
Verona	The Longines FEI World Cup Jumping - Verona round A	29.10.2017	CSI-W5*	4	1.40 - 1.60	465	188	11	06.06.2007	G	KWPN	209	10	67	60	47	2033	59	M	7	7a	yes	spread	oxer	straight	no	0	0	78,07	no
Verona	The Longines FEI World Cup Jumping - Verona round A	29.10.2017	CSI-W5*	4	1.40 - 1.60	465	188	11	06.06.2007	G	KWPN	209	10	67	60	47	2033	59	M	8	7b	yes	vertical	vertical	straight	no	0	0	78,07	no
Verona	The Longines FEI World Cup Jumping - Verona round A	29.10.2017	CSI-W5*	4	1.40 - 1.60	465	188	11	06.06.2007	G	KWPN	209	10	67	60	47	2033	59	M	9	8	no	vertical	vertical	left	no	0	0	78,07	no
Verona	The Longines FEI World Cup Jumping - Verona round A	29.10.2017	CSI-W5*	4	1.40 - 1.60	465	188	11	06.06.2007	G	KWPN	209	10	67	60	47	2033	59	M	10	9	no	spread	oxer	left	no	0	0	78,07	no
Verona	The Longines FEI World Cup Jumping - Verona round A	29.10.2017	CSI-W5*	4	1.40 - 1.60	465	188	11	06.06.2007	G	KWPN	209	10	67	60	47	2033	59	M	11	10	no	vertical	vertical	right	yes	4	0	78,07	no
Verona	The Longines FEI World Cup Jumping - Verona round A	29.10.2017	CSI-W5*	4	1.40 - 1.60	465	188	11	06.06.2007	G	KWPN	209	10	67	60	47	2033	59	M	12	11a	yes	vertical	vertical	right	yes	4	0	78,07	no
Verona	The Longines FEI World Cup Jumping - Verona round A	29.10.2017	CSI-W5*	4	1.40 - 1.60	465	188	11	06.06.2007	G	KWPN	209	10	67	60	47	2033	59	M	13	11b	yes	spread	oxer	right	no	0	0	78,07	no
Verona	The Longines FEI World Cup Jumping - Verona round A	29.10.2017	CSI-W5*	4	1.40 - 1.60	465	188	11	06.06.2007	G	KWPN	209	10	67	60	47	2033	59	M	14	11c	yes	spread	oxer	right	no	0	0	78,07	no
Verona	The Longines FEI World Cup Jumping - Verona round A	29.10.2017	CSI-W5*	4	1.40 - 1.60	465	188	11	06.06.2007	G	KWPN	209	10	67	60	47	2033	59	M	15	12	no	vertical	vertical	left	no	0	0	78,07	no
Verona	The Longines FEI World Cup Jumping - Verona round A	29.10.2017	CSI-W5*	4	1.40 - 1.60	465	188	11	06.06.2007	G	KWPN	209	10	67	60	47	2033	59	M	16	13	no	spread	oxer	straight	no	0	0	78,07	no
Verona	The Longines FEI World Cup Jumping - Verona round A	29.10.2017	CSI-W5*	4	1.40 - 1.60	465	12	12	01.04.2006	S	KWPN	105	2	68	67	31	1737	39	F	1	1	no	vertical	vertical	left	no	0	0	78,06	no
Verona	The Longines FEI World Cup Jumping - Verona round A	29.10.2017	CSI-W5*	4	1.40 - 1.60	465	12	12	01.04.2006	S	KWPN	105	2	68	67	31	1737	39	F	2	2	no	spread	oxer	left	no	0	0	78,06	no
Verona	The Longines FEI World Cup Jumping - Verona round A	29.10.2017	CSI-W5*	4	1.40 - 1.60	465	12	12	01.04.2006	S	KWPN	105	2	68	67	31	1737	39	F	3	3	no	vertical	vertical	right	no	0	0	78,06	no
Verona	The Longines FEI World Cup Jumping - Verona round A	29.10.2017	CSI-W5*	4	1.40 - 1.60	465	12	12	01.04.2006	S	KWPN	105	2	68	67	31	1737	39	F	4	4	no	spread	oxer	right	no	0	0	78,06	no
Verona	The Longines FEI World Cup Jumping - Verona round A	29.10.2017	CSI-W5*	4	1.40 - 1.60	465	12	12	01.04.2006	S	KWPN	105	2	68	67	31	1737	39	F	5	5	no	vertical	vertical with water	straight	no	0	0	78,06	no
Verona	The Longines FEI World Cup Jumping - Verona round A	29.10.2017	CSI-W5*	4	1.40 - 1.60	465	12	12	01.04.2006	S	KWPN	105	2	68	67	31	1737	39	F	6	6	no	spread	oxer with water	left	no	0	0	78,06	no
Verona	The Longines FEI World Cup Jumping - Verona round A	29.10.2017	CSI-W5*	4	1.40 - 1.60	465	12	12	01.04.2006	S	KWPN	105	2	68	67	31	1737	39	F	7	7a	yes	spread	oxer	straight	no	0	0	78,06	no
Verona	The Longines FEI World Cup Jumping - Verona round A	29.10.2017	CSI-W5*	4	1.40 - 1.60	465	12	12	01.04.2006	S	KWPN	105	2	68	67	31	1737	39	F	8	7b	yes	vertical	vertical	straight	no	0	0	78,06	no
Verona	The Longines FEI World Cup Jumping - Verona round A	29.10.2017	CSI-W5*	4	1.40 - 1.60	465	12	12	01.04.2006	S	KWPN	105	2	68	67	31	1737	39	F	9	8	no	vertical	vertical	left	no	0	0	78,06	no
Verona	The Longines FEI World Cup Jumping - Verona round A	29.10.2017	CSI-W5*	4	1.40 - 1.60	465	12	12	01.04.2006	S	KWPN	105	2	68	67	31	1737	39	F	10	9	no	spread	oxer	left	no	0	0	78,06	no
Verona	The Longines FEI World Cup Jumping - Verona round A	29.10.2017	CSI-W5*	4	1.40 - 1.60	465	12	12	01.04.2006	S	KWPN	105	2	68	67	31	1737	39	F	11	10	no	vertical	vertical	right	no	0	0	78,06	no
Verona	The Longines FEI World Cup Jumping - Verona round A	29.10.2017	CSI-W5*	4	1.40 - 1.60	465	12	12	01.04.2006	S	KWPN	105	2	68	67	31	1737	39	F	12	11a	yes	vertical	vertical	right	no	0	0	78,06	no
Verona	The Longines FEI World Cup Jumping - Verona round A	29.10.2017	CSI-W5*	4	1.40 - 1.60	465	12	12	01.04.2006	S	KWPN	105	2	68	67	31	1737	39	F	13	11b	yes	spread	oxer	right	no	0	0	78,06	no
Verona	The Longines FEI World Cup Jumping - Verona round A	29.10.2017	CSI-W5*	4	1.40 - 1.60	465	12	12	01.04.2006	S	KWPN	105	2	68	67	31	1737	39	F	14	11c	yes	spread	oxer	right	no	0	0	78,06	no
Verona	The Longines FEI World Cup Jumping - Verona round A	29.10.2017	CSI-W5*	4	1.40 - 1.60	465	12	12	01.04.2006	S	KWPN	105	2	68	67	31	1737	39	F	15	12	no	vertical	vertical	left	no	0	0	78,06	no
Verona	The Longines FEI World Cup Jumping - Verona round A	29.10.2017	CSI-W5*	4	1.40 - 1.60	465	12	12	01.04.2006	S	KWPN	105	2	68	67	31	1737	39	F	16	13	no	spread	oxer	straight	no	0	0	78,06	no
Verona	The Longines FEI World Cup Jumping - Verona round B	29.10.2017	CSI-W5*	4	1.40 - 1.60	290	12	12	01.04.2006	S	KWPN	105	2	68	67	31	1737	39	F	1	1	no	vertical	vertical	right	no	0	0	39,21	no
Verona	The Longines FEI World Cup Jumping - Verona round B	29.10.2017	CSI-W5*	4	1.40 - 1.60	290	12	12	01.04.2006	S	KWPN	105	2	68	67	31	1737	39	F	2	2	no	spread	oxer	right	no	0	0	39,21	no
Verona	The Longines FEI World Cup Jumping - Verona round B	29.10.2017	CSI-W5*	4	1.40 - 1.60	290	12	12	01.04.2006	S	KWPN	105	2	68	67	31	1737	39	F	3	4a	yes	vertical	vertical with water	straight	no	0	0	39,21	no
Verona	The Longines FEI World Cup Jumping - Verona round B	29.10.2017	CSI-W5*	4	1.40 - 1.60	290	12	12	01.04.2006	S	KWPN	105	2	68	67	31	1737	39	F	4	4b	yes	spread	oxer	straight	no	0	0	39,21	no
Verona	The Longines FEI World Cup Jumping - Verona round B	29.10.2017	CSI-W5*	4	1.40 - 1.60	290	12	12	01.04.2006	S	KWPN	105	2	68	67	31	1737	39	F	5	5	no	vertical	vertical	left	no	0	0	39,21	no
Verona	The Longines FEI World Cup Jumping - Verona round B	29.10.2017	CSI-W5*	4	1.40 - 1.60	290	12	12	01.04.2006	S	KWPN	105	2	68	67	31	1737	39	F	6	6	no	spread	oxer	left	no	0	0	39,21	no
Verona	The Longines FEI World Cup Jumping - Verona round B	29.10.2017	CSI-W5*	4	1.40 - 1.60	290	12	12	01.04.2006	S	KWPN	105	2	68	67	31	1737	39	F	7	7	no	vertical	vertical	right	no	0	0	39,21	no
Verona	The Longines FEI World Cup Jumping - Verona round B	29.10.2017	CSI-W5*	4	1.40 - 1.60	290	12	12	01.04.2006	S	KWPN	105	2	68	67	31	1737	39	F	8	7	no	vertical	vertical	right	no	0	0	39,21	no
Verona	The Longines FEI World Cup Jumping - Verona round A	29.10.2017	CSI-W5*	4	1.40 - 1.60	465	179	12	28.05.2006	G	HOLST	170	7	69	147	42	785	73	M	1	1	no	vertical	vertical	left	no	0	0	76,97	no
Verona	The Longines FEI World Cup Jumping - Verona round A	29.10.2017	CSI-W5*	4	1.40 - 1.60	465	179	12	28.05.2006	G	HOLST	170	7	69	147	42	785	73	M	2	2	no	spread	oxer	left	no	0	0	76,97	no
Verona	The Longines FEI World Cup Jumping - Verona round A	29.10.2017	CSI-W5*	4	1.40 - 1.60	465	179	12	28.05.2006	G	HOLST	170	7	69	147	42	785	73	M	3	3	no	vertical	vertical	right	no	0	0	76,97	no
Verona	The Longines FEI World Cup Jumping - Verona round A	29.10.2017	CSI-W5*	4	1.40 - 1.60	465	179	12	28.05.2006	G	HOLST	170	7	69	147	42	785	73	M	4	4	no	spread	oxer	right	no	0	0	76,97	no
Verona	The Longines FEI World Cup Jumping - Verona round A	29.10.2017	CSI-W5*	4	1.40 - 1.60	465	179	12	28.05.2006	G	HOLST	170	7	69	147	42	785	73	M	5	5	no	vertical	vertical with water	straight	no	0	0	76,97	no
Verona	The Longines FEI World Cup Jumping - Verona round A	29.10.2017	CSI-W5*	4	1.40 - 1.60	465	179	12	28.05.2006	G	HOLST	170	7	69	147	42	785	73	M	6	6	no	spread	oxer with water	left	no	0	0	76,97	no
Verona	The Longines FEI World Cup Jumping - Verona round A	29.10.2017	CSI-W5*	4	1.40 - 1.60	465	179	12	28.05.2006	G	HOLST	170	7	69	147	42	785	73	M	7	7a	yes	spread	oxer	straight	no	0	0	76,97	no
Verona	The Longines FEI World Cup Jumping - Verona round A	29.10.2017	CSI-W5*	4	1.40 - 1.60	465	179	12	28.05.2006	G	HOLST	170	7	69	147	42	785	73	M	8	7b	yes	vertical	vertical	straight	no	0	0	76,97	no
Verona	The Longines FEI World Cup Jumping - Verona round A	29.10.2017	CSI-W5*	4	1.40 - 1.60	465	179	12	28.05.2006	G	HOLST	170	7	69	147	42	785	73	M	9	8	no	vertical	vertical	left	no	0	0	76,97	no
Verona	The Longines FEI World Cup Jumping - Verona round A	29.10.2017	CSI-W5*	4	1.40 - 1.60	465	179	12	28.05.2006	G	HOLST	170	7	69	147	42	785	73	M	10	9	no	spread	oxer	left	no	0	0	76,97	no
Verona	The Longines FEI World Cup Jumping - Verona round A	29.10.2017	CSI-W5*	4	1.40 - 1.60	465	179	12	28.05.2006	G	HOLST	170	7	69	147	42	785	73	M	11	10	no	vertical	vertical	right	no	0	0	76,97	no
Verona	The Longines FEI World Cup Jumping - Verona round A	29.10.2017	CSI-W5*	4	1.40 - 1.60	465	179	12	28.05.2006	G	HOLST	170	7	69	147	42	785	73	M	12	11a	yes	vertical	vertical	right	no	0	0	76,97	no
Verona	The Longines FEI World Cup Jumping - Verona round A	29.10.2017	CSI-W5*	4	1.40 - 1.60	465	179	12	28.05.2006	G	HOLST	170	7	69	147	42	785	73	M	13	11b	yes	spread	oxer	right	no	0	0	76,97	no
Verona	The Longines FEI World Cup Jumping - Verona round A	29.10.2017	CSI-W5*	4	1.40 - 1.60	465	179	12	28.05.2006	G	HOLST	170	7	69	147	42	785	73	M	14	11c	yes	spread	oxer	right	no	0	0	76,97	no
Verona	The Longines FEI World Cup Jumping - Verona round A	29.10.2017	CSI-W5*	4	1.40 - 1.60	465	179	12	28.05.2006	G	HOLST	170	7	69	147	42	785	73	M	15	12	no	vertical	vertical	left	no	0	0	76,97	no
Verona	The Longines FEI World Cup Jumping - Verona round A	29.10.2017	CSI-W5*	4	1.40 - 1.60	465	179	12	28.05.2006	G	HOLST	170	7	69	147	42	785	73	M	16	13	no	spread	oxer	straight	no	0	0	76,97	no
Verona	The Longines FEI World Cup Jumping - Verona round B	29.10.2017	CSI-W5*	4	1.40 - 1.60	290	179	12	28.05.2006	G	HOLST	170	7	69	147	42	785	73	M	1	1	no	vertical	vertical	right	no	0	0	36,93	no
Verona	The Longines FEI World Cup Jumping - Verona round B	29.10.2017	CSI-W5*	4	1.40 - 1.60	290	179	12	28.05.2006	G	HOLST	170	7	69	147	42	785	73	M	2	2	no	spread	oxer	right	no	0	0	36,93	no
Verona	The Longines FEI World Cup Jumping - Verona round B	29.10.2017	CSI-W5*	4	1.40 - 1.60	290	179	12	28.05.2006	G	HOLST	170	7	69	147	42	785	73	M	3	4a	yes	vertical	vertical with water	straight	yes	4	0	36,93	no
Verona	The Longines FEI World Cup Jumping - Verona round B	29.10.2017	CSI-W5*	4	1.40 - 1.60	290	179	12	28.05.2006	G	HOLST	170	7	69	147	42	785	73	M	4	4b	yes	spread	oxer	straight	no	0	0	36,93	no
Verona	The Longines FEI World Cup Jumping - Verona round B	29.10.2017	CSI-W5*	4	1.40 - 1.60	290	179	12	28.05.2006	G	HOLST	170	7	69	147	42	785	73	M	5	5	no	vertical	vertical	left	no	0	0	36,93	no
Verona	The Longines FEI World Cup Jumping - Verona round B	29.10.2017	CSI-W5*	4	1.40 - 1.60	290	179	12	28.05.2006	G	HOLST	170	7	69	147	42	785	73	M	6	6	no	spread	oxer	left	no	0	0	36,93	no
Verona	The Longines FEI World Cup Jumping - Verona round B	29.10.2017	CSI-W5*	4	1.40 - 1.60	290	179	12	28.05.2006	G	HOLST	170	7	69	147	42	785	73	M	7	7	no	vertical	vertical	right	yes	4	0	36,93	no
Verona	The Longines FEI World Cup Jumping - Verona round B	29.10.2017	CSI-W5*	4	1.40 - 1.60	290	179	12	28.05.2006	G	HOLST	170	7	69	147	42	785	73	M	8	7	no	vertical	vertical	right	no	0	0	36,93	no
Verona	The Longines FEI World Cup Jumping - Verona round A	29.10.2017	CSI-W5*	4	1.40 - 1.60	465	211	13	11.05.2005	S	SBS	150	4	70	43	38	1891	85	F	2	2	no	spread	oxer	left	no	0	0	76,77	no
Verona	The Longines FEI World Cup Jumping - Verona round A	29.10.2017	CSI-W5*	4	1.40 - 1.60	465	211	13	11.05.2005	S	SBS	150	4	70	43	38	1891	85	F	3	3	no	vertical	vertical	right	no	0	0	76,77	no
Verona	The Longines FEI World Cup Jumping - Verona round A	29.10.2017	CSI-W5*	4	1.40 - 1.60	465	211	13	11.05.2005	S	SBS	150	4	70	43	38	1891	85	F	4	4	no	spread	oxer	right	no	0	0	76,77	no
Verona	The Longines FEI World Cup Jumping - Verona round A	29.10.2017	CSI-W5*	4	1.40 - 1.60	465	211	13	11.05.2005	S	SBS	150	4	70	43	38	1891	85	F	5	5	no	vertical	vertical with water	straight	no	0	0	76,77	no
Verona	The Longines FEI World Cup Jumping - Verona round A	29.10.2017	CSI-W5*	4	1.40 - 1.60	465	211	13	11.05.2005	S	SBS	150	4	70	43	38	1891	85	F	6	6	no	spread	oxer with water	left	no	0	0	76,77	no
Verona	The Longines FEI World Cup Jumping - Verona round A	29.10.2017	CSI-W5*	4	1.40 - 1.60	465	211	13	11.05.2005	S	SBS	150	4	70	43	38	1891	85	F	7	7a	yes	spread	oxer	straight	no	0	0	76,77	no
Verona	The Longines FEI World Cup Jumping - Verona round A	29.10.2017	CSI-W5*	4	1.40 - 1.60	465	211	13	11.05.2005	S	SBS	150	4	70	43	38	1891	85	F	8	7b	yes	vertical	vertical	straight	no	0	0	76,77	no
Verona	The Longines FEI World Cup Jumping - Verona round A	29.10.2017	CSI-W5*	4	1.40 - 1.60	465	211	13	11.05.2005	S	SBS	150	4	70	43	38	1891	85	F	9	8	no	vertical	vertical	left	no	0	0	76,77	no
Verona	The Longines FEI World Cup Jumping - Verona round A	29.10.2017	CSI-W5*	4	1.40 - 1.60	465	211	13	11.05.2005	S	SBS	150	4	70	43	38	1891	85	F	10	9	no	spread	oxer	left	no	0	0	76,77	no
Verona	The Longines FEI World Cup Jumping - Verona round A	29.10.2017	CSI-W5*	4	1.40 - 1.60	465	211	13	11.05.2005	S	SBS	150	4	70	43	38	1891	85	F	11	10	no	vertical	vertical	right	no	0	0	76,77	no
Verona	The Longines FEI World Cup Jumping - Verona round A	29.10.2017	CSI-W5*	4	1.40 - 1.60	465	211	13	11.05.2005	S	SBS	150	4	70	43	38	1891	85	F	12	11a	yes	vertical	vertical	right	no	0	0	76,77	no
Verona	The Longines FEI World Cup Jumping - Verona round A	29.10.2017	CSI-W5*	4	1.40 - 1.60	465	211	13	11.05.2005	S	SBS	150	4	70	43	38	1891	85	F	13	11b	yes	spread	oxer	right	no	0	0	76,77	no
Verona	The Longines FEI World Cup Jumping - Verona round A	29.10.2017	CSI-W5*	4	1.40 - 1.60	465	211	13	11.05.2005	S	SBS	150	4	70	43	38	1891	85	F	14	11c	yes	spread	oxer	right	no	0	0	76,77	no
Verona	The Longines FEI World Cup Jumping - Verona round A	29.10.2017	CSI-W5*	4	1.40 - 1.60	465	211	13	11.05.2005	S	SBS	150	4	70	43	38	1891	85	F	15	12	no	vertical	vertical	left	no	0	0	76,77	no
Verona	The Longines FEI World Cup Jumping - Verona round A	29.10.2017	CSI-W5*	4	1.40 - 1.60	465	211	13	11.05.2005	S	SBS	150	4	70	43	38	1891	85	F	16	13	no	spread	oxer	straight	no	0	0	76,77	no
Verona	The Longines FEI World Cup Jumping - Verona round A	29.10.2017	CSI-W5*	4	1.40 - 1.60	465	211	13	11.05.2005	S	SBS	150	4	70	43	38	1891	85	F	1	1	no	vertical	vertical	left	no	0	0	76,77	no
Verona	The Longines FEI World Cup Jumping - Verona round B	29.10.2017	CSI-W5*	4	1.40 - 1.60	290	211	13	11.05.2005	S	SBS	150	4	70	43	38	1891	85	F	1	1	no	vertical	vertical	right	no	0	0	38,34	no
Verona	The Longines FEI World Cup Jumping - Verona round B	29.10.2017	CSI-W5*	4	1.40 - 1.60	290	211	13	11.05.2005	S	SBS	150	4	70	43	38	1891	85	F	2	2	no	spread	oxer	right	no	0	0	38,34	no
Verona	The Longines FEI World Cup Jumping - Verona round B	29.10.2017	CSI-W5*	4	1.40 - 1.60	290	211	13	11.05.2005	S	SBS	150	4	70	43	38	1891	85	F	3	4a	yes	vertical	vertical with water	straight	yes	4	0	38,34	no
Verona	The Longines FEI World Cup Jumping - Verona round B	29.10.2017	CSI-W5*	4	1.40 - 1.60	290	211	13	11.05.2005	S	SBS	150	4	70	43	38	1891	85	F	4	4b	yes	spread	oxer	straight	no	0	0	38,34	no
Verona	The Longines FEI World Cup Jumping - Verona round B	29.10.2017	CSI-W5*	4	1.40 - 1.60	290	211	13	11.05.2005	S	SBS	150	4	70	43	38	1891	85	F	5	5	no	vertical	vertical	left	no	0	0	38,34	no
Verona	The Longines FEI World Cup Jumping - Verona round B	29.10.2017	CSI-W5*	4	1.40 - 1.60	290	211	13	11.05.2005	S	SBS	150	4	70	43	38	1891	85	F	6	6	no	spread	oxer	left	no	0	0	38,34	no
Verona	The Longines FEI World Cup Jumping - Verona round B	29.10.2017	CSI-W5*	4	1.40 - 1.60	290	211	13	11.05.2005	S	SBS	150	4	70	43	38	1891	85	F	7	7	no	vertical	vertical	right	no	0	0	38,34	no
Verona	The Longines FEI World Cup Jumping - Verona round B	29.10.2017	CSI-W5*	4	1.40 - 1.60	290	211	13	11.05.2005	S	SBS	150	4	70	43	38	1891	85	F	8	7	no	vertical	vertical	right	no	0	0	38,34	no
Verona	The Longines FEI World Cup Jumping - Verona round A	29.10.2017	CSI-W5*	4	1.40 - 1.60	465	94	13	23.02.2005	S	WESTF	178	6	71	12	44	2177	93	M	1	1	no	vertical	vertical	left	no	0	0	76,66	no
Verona	The Longines FEI World Cup Jumping - Verona round A	29.10.2017	CSI-W5*	4	1.40 - 1.60	465	94	13	23.02.2005	S	WESTF	178	6	71	12	44	2177	93	M	2	2	no	spread	oxer	left	no	0	0	76,66	no
Verona	The Longines FEI World Cup Jumping - Verona round A	29.10.2017	CSI-W5*	4	1.40 - 1.60	465	94	13	23.02.2005	S	WESTF	178	6	71	12	44	2177	93	M	3	3	no	vertical	vertical	right	no	0	0	76,66	no
Verona	The Longines FEI World Cup Jumping - Verona round A	29.10.2017	CSI-W5*	4	1.40 - 1.60	465	94	13	23.02.2005	S	WESTF	178	6	71	12	44	2177	93	M	4	4	no	spread	oxer	right	no	0	0	76,66	no
Verona	The Longines FEI World Cup Jumping - Verona round A	29.10.2017	CSI-W5*	4	1.40 - 1.60	465	94	13	23.02.2005	S	WESTF	178	6	71	12	44	2177	93	M	5	5	no	vertical	vertical with water	straight	no	0	0	76,66	no
Verona	The Longines FEI World Cup Jumping - Verona round A	29.10.2017	CSI-W5*	4	1.40 - 1.60	465	94	13	23.02.2005	S	WESTF	178	6	71	12	44	2177	93	M	6	6	no	spread	oxer with water	left	no	0	0	76,66	no
Verona	The Longines FEI World Cup Jumping - Verona round A	29.10.2017	CSI-W5*	4	1.40 - 1.60	465	94	13	23.02.2005	S	WESTF	178	6	71	12	44	2177	93	M	7	7a	yes	spread	oxer	straight	no	0	0	76,66	no
Verona	The Longines FEI World Cup Jumping - Verona round A	29.10.2017	CSI-W5*	4	1.40 - 1.60	465	94	13	23.02.2005	S	WESTF	178	6	71	12	44	2177	93	M	8	7b	yes	vertical	vertical	straight	no	0	0	76,66	no
Verona	The Longines FEI World Cup Jumping - Verona round A	29.10.2017	CSI-W5*	4	1.40 - 1.60	465	94	13	23.02.2005	S	WESTF	178	6	71	12	44	2177	93	M	9	8	no	vertical	vertical	left	yes	4	0	76,66	no
Verona	The Longines FEI World Cup Jumping - Verona round A	29.10.2017	CSI-W5*	4	1.40 - 1.60	465	94	13	23.02.2005	S	WESTF	178	6	71	12	44	2177	93	M	10	9	no	spread	oxer	left	no	0	0	76,66	no
Verona	The Longines FEI World Cup Jumping - Verona round A	29.10.2017	CSI-W5*	4	1.40 - 1.60	465	94	13	23.02.2005	S	WESTF	178	6	71	12	44	2177	93	M	11	10	no	vertical	vertical	right	yes	4	0	76,66	no
Verona	The Longines FEI World Cup Jumping - Verona round A	29.10.2017	CSI-W5*	4	1.40 - 1.60	465	94	13	23.02.2005	S	WESTF	178	6	71	12	44	2177	93	M	12	11a	yes	vertical	vertical	right	no	0	0	76,66	no
Verona	The Longines FEI World Cup Jumping - Verona round A	29.10.2017	CSI-W5*	4	1.40 - 1.60	465	94	13	23.02.2005	S	WESTF	178	6	71	12	44	2177	93	M	13	11b	yes	spread	oxer	right	no	0	0	76,66	no
Verona	The Longines FEI World Cup Jumping - Verona round A	29.10.2017	CSI-W5*	4	1.40 - 1.60	465	94	13	23.02.2005	S	WESTF	178	6	71	12	44	2177	93	M	14	11c	yes	spread	oxer	right	no	0	0	76,66	no
Verona	The Longines FEI World Cup Jumping - Verona round A	29.10.2017	CSI-W5*	4	1.40 - 1.60	465	94	13	23.02.2005	S	WESTF	178	6	71	12	44	2177	93	M	15	12	no	vertical	vertical	left	no	0	0	76,66	no
Verona	The Longines FEI World Cup Jumping - Verona round A	29.10.2017	CSI-W5*	4	1.40 - 1.60	465	94	13	23.02.2005	S	WESTF	178	6	71	12	44	2177	93	M	16	13	no	spread	oxer	straight	no	0	0	76,66	no
Verona	The Longines FEI World Cup Jumping - Verona round A	29.10.2017	CSI-W5*	4	1.40 - 1.60	465	99	12	08.06.2006	G	BWP	164	10	72	26	38	2171	177	M	1	1	no	vertical	vertical	left	no	0	0	76,64	no
Verona	The Longines FEI World Cup Jumping - Verona round A	29.10.2017	CSI-W5*	4	1.40 - 1.60	465	99	12	08.06.2006	G	BWP	164	10	72	26	38	2171	177	M	2	2	no	spread	oxer	left	no	0	0	76,64	no
Verona	The Longines FEI World Cup Jumping - Verona round A	29.10.2017	CSI-W5*	4	1.40 - 1.60	465	99	12	08.06.2006	G	BWP	164	10	72	26	38	2171	177	M	3	3	no	vertical	vertical	right	no	0	0	76,64	no
Verona	The Longines FEI World Cup Jumping - Verona round A	29.10.2017	CSI-W5*	4	1.40 - 1.60	465	99	12	08.06.2006	G	BWP	164	10	72	26	38	2171	177	M	4	4	no	spread	oxer	right	no	0	0	76,64	no
Verona	The Longines FEI World Cup Jumping - Verona round A	29.10.2017	CSI-W5*	4	1.40 - 1.60	465	99	12	08.06.2006	G	BWP	164	10	72	26	38	2171	177	M	5	5	no	vertical	vertical with water	straight	no	0	0	76,64	no
Verona	The Longines FEI World Cup Jumping - Verona round A	29.10.2017	CSI-W5*	4	1.40 - 1.60	465	99	12	08.06.2006	G	BWP	164	10	72	26	38	2171	177	M	6	6	no	spread	oxer with water	left	no	0	0	76,64	no
Verona	The Longines FEI World Cup Jumping - Verona round A	29.10.2017	CSI-W5*	4	1.40 - 1.60	465	99	12	08.06.2006	G	BWP	164	10	72	26	38	2171	177	M	7	7a	yes	spread	oxer	straight	no	0	0	76,64	no
Verona	The Longines FEI World Cup Jumping - Verona round A	29.10.2017	CSI-W5*	4	1.40 - 1.60	465	99	12	08.06.2006	G	BWP	164	10	72	26	38	2171	177	M	8	7b	yes	vertical	vertical	straight	no	0	0	76,64	no
Verona	The Longines FEI World Cup Jumping - Verona round A	29.10.2017	CSI-W5*	4	1.40 - 1.60	465	99	12	08.06.2006	G	BWP	164	10	72	26	38	2171	177	M	9	8	no	vertical	vertical	left	no	0	0	76,64	no
Verona	The Longines FEI World Cup Jumping - Verona round A	29.10.2017	CSI-W5*	4	1.40 - 1.60	465	99	12	08.06.2006	G	BWP	164	10	72	26	38	2171	177	M	10	9	no	spread	oxer	left	no	0	0	76,64	no
Verona	The Longines FEI World Cup Jumping - Verona round A	29.10.2017	CSI-W5*	4	1.40 - 1.60	465	99	12	08.06.2006	G	BWP	164	10	72	26	38	2171	177	M	11	10	no	vertical	vertical	right	no	0	0	76,64	no
Verona	The Longines FEI World Cup Jumping - Verona round A	29.10.2017	CSI-W5*	4	1.40 - 1.60	465	99	12	08.06.2006	G	BWP	164	10	72	26	38	2171	177	M	12	11a	yes	vertical	vertical	right	no	0	0	76,64	no
Verona	The Longines FEI World Cup Jumping - Verona round A	29.10.2017	CSI-W5*	4	1.40 - 1.60	465	99	12	08.06.2006	G	BWP	164	10	72	26	38	2171	177	M	13	11b	yes	spread	oxer	right	no	0	0	76,64	no
Verona	The Longines FEI World Cup Jumping - Verona round A	29.10.2017	CSI-W5*	4	1.40 - 1.60	465	99	12	08.06.2006	G	BWP	164	10	72	26	38	2171	177	M	14	11c	yes	spread	oxer	right	yes	4	0	76,64	no
Verona	The Longines FEI World Cup Jumping - Verona round A	29.10.2017	CSI-W5*	4	1.40 - 1.60	465	99	12	08.06.2006	G	BWP	164	10	72	26	38	2171	177	M	15	12	no	vertical	vertical	left	no	0	0	76,64	no
Verona	The Longines FEI World Cup Jumping - Verona round A	29.10.2017	CSI-W5*	4	1.40 - 1.60	465	99	12	08.06.2006	G	BWP	164	10	72	26	38	2171	177	M	16	13	no	spread	oxer	straight	no	0	0	76,64	no
Verona	The Longines FEI World Cup Jumping - Verona round A	29.10.2017	CSI-W5*	4	1.40 - 1.60	465	163	15	10.05.2003	S	SF	158	2	73	527	24	494	5	M	1	1	no	vertical	vertical	left	no	0	0	76,43	no
Verona	The Longines FEI World Cup Jumping - Verona round A	29.10.2017	CSI-W5*	4	1.40 - 1.60	465	163	15	10.05.2003	S	SF	158	2	73	527	24	494	5	M	2	2	no	spread	oxer	left	no	0	0	76,43	no
Verona	The Longines FEI World Cup Jumping - Verona round A	29.10.2017	CSI-W5*	4	1.40 - 1.60	465	163	15	10.05.2003	S	SF	158	2	73	527	24	494	5	M	3	3	no	vertical	vertical	right	no	0	0	76,43	no
Verona	The Longines FEI World Cup Jumping - Verona round A	29.10.2017	CSI-W5*	4	1.40 - 1.60	465	163	15	10.05.2003	S	SF	158	2	73	527	24	494	5	M	4	4	no	spread	oxer	right	no	0	0	76,43	no
Verona	The Longines FEI World Cup Jumping - Verona round A	29.10.2017	CSI-W5*	4	1.40 - 1.60	465	163	15	10.05.2003	S	SF	158	2	73	527	24	494	5	M	5	5	no	vertical	vertical with water	straight	no	0	0	76,43	no
Verona	The Longines FEI World Cup Jumping - Verona round A	29.10.2017	CSI-W5*	4	1.40 - 1.60	465	163	15	10.05.2003	S	SF	158	2	73	527	24	494	5	M	6	6	no	spread	oxer with water	left	no	0	0	76,43	no
Verona	The Longines FEI World Cup Jumping - Verona round A	29.10.2017	CSI-W5*	4	1.40 - 1.60	465	163	15	10.05.2003	S	SF	158	2	73	527	24	494	5	M	7	7a	yes	spread	oxer	straight	no	0	0	76,43	no
Verona	The Longines FEI World Cup Jumping - Verona round A	29.10.2017	CSI-W5*	4	1.40 - 1.60	465	163	15	10.05.2003	S	SF	158	2	73	527	24	494	5	M	8	7b	yes	vertical	vertical	straight	no	0	0	76,43	no
Verona	The Longines FEI World Cup Jumping - Verona round A	29.10.2017	CSI-W5*	4	1.40 - 1.60	465	163	15	10.05.2003	S	SF	158	2	73	527	24	494	5	M	9	8	no	vertical	vertical	left	no	0	0	76,43	no
Verona	The Longines FEI World Cup Jumping - Verona round A	29.10.2017	CSI-W5*	4	1.40 - 1.60	465	163	15	10.05.2003	S	SF	158	2	73	527	24	494	5	M	10	9	no	spread	oxer	left	no	0	0	76,43	no
Verona	The Longines FEI World Cup Jumping - Verona round A	29.10.2017	CSI-W5*	4	1.40 - 1.60	465	163	15	10.05.2003	S	SF	158	2	73	527	24	494	5	M	11	10	no	vertical	vertical	right	no	0	0	76,43	no
Verona	The Longines FEI World Cup Jumping - Verona round A	29.10.2017	CSI-W5*	4	1.40 - 1.60	465	163	15	10.05.2003	S	SF	158	2	73	527	24	494	5	M	12	11a	yes	vertical	vertical	right	no	0	0	76,43	no
Verona	The Longines FEI World Cup Jumping - Verona round A	29.10.2017	CSI-W5*	4	1.40 - 1.60	465	163	15	10.05.2003	S	SF	158	2	73	527	24	494	5	M	13	11b	yes	spread	oxer	right	no	0	0	76,43	no
Verona	The Longines FEI World Cup Jumping - Verona round A	29.10.2017	CSI-W5*	4	1.40 - 1.60	465	163	15	10.05.2003	S	SF	158	2	73	527	24	494	5	M	14	11c	yes	spread	oxer	right	no	0	0	76,43	no
Verona	The Longines FEI World Cup Jumping - Verona round A	29.10.2017	CSI-W5*	4	1.40 - 1.60	465	163	15	10.05.2003	S	SF	158	2	73	527	24	494	5	M	15	12	no	vertical	vertical	left	no	0	0	76,43	no
Verona	The Longines FEI World Cup Jumping - Verona round A	29.10.2017	CSI-W5*	4	1.40 - 1.60	465	163	15	10.05.2003	S	SF	158	2	73	527	24	494	5	M	16	13	no	spread	oxer	straight	no	0	0	76,43	no
Verona	The Longines FEI World Cup Jumping - Verona round B	29.10.2017	CSI-W5*	4	1.40 - 1.60	290	163	15	10.05.2003	S	SF	158	2	73	527	24	494	5	M	1	1	no	vertical	vertical	right	no	0	0	42,04	no
Verona	The Longines FEI World Cup Jumping - Verona round B	29.10.2017	CSI-W5*	4	1.40 - 1.60	290	163	15	10.05.2003	S	SF	158	2	73	527	24	494	5	M	2	2	no	spread	oxer	right	no	0	0	42,04	no
Verona	The Longines FEI World Cup Jumping - Verona round B	29.10.2017	CSI-W5*	4	1.40 - 1.60	290	163	15	10.05.2003	S	SF	158	2	73	527	24	494	5	M	3	4a	yes	vertical	vertical with water	straight	yes	4	0	42,04	no
Verona	The Longines FEI World Cup Jumping - Verona round B	29.10.2017	CSI-W5*	4	1.40 - 1.60	290	163	15	10.05.2003	S	SF	158	2	73	527	24	494	5	M	4	4b	yes	spread	oxer	straight	no	0	0	42,04	no
Verona	The Longines FEI World Cup Jumping - Verona round B	29.10.2017	CSI-W5*	4	1.40 - 1.60	290	163	15	10.05.2003	S	SF	158	2	73	527	24	494	5	M	5	5	no	vertical	vertical	left	yes	4	0	42,04	no
Verona	The Longines FEI World Cup Jumping - Verona round B	29.10.2017	CSI-W5*	4	1.40 - 1.60	290	163	15	10.05.2003	S	SF	158	2	73	527	24	494	5	M	6	6	no	spread	oxer	left	no	0	0	42,04	no
Verona	The Longines FEI World Cup Jumping - Verona round B	29.10.2017	CSI-W5*	4	1.40 - 1.60	290	163	15	10.05.2003	S	SF	158	2	73	527	24	494	5	M	7	7	no	vertical	vertical	right	no	0	0	42,04	no
Verona	The Longines FEI World Cup Jumping - Verona round B	29.10.2017	CSI-W5*	4	1.40 - 1.60	290	163	15	10.05.2003	S	SF	158	2	73	527	24	494	5	M	8	7	no	vertical	vertical	right	no	0	0	42,04	no
Verona	The Longines FEI World Cup Jumping - Verona round A	29.10.2017	CSI-W5*	4	1.40 - 1.60	465	173	13	14.07.2005	G	SF	229	2	74	65	34	1260	61	M	1	1	no	vertical	vertical	left	no	0	0	76,17	no
Verona	The Longines FEI World Cup Jumping - Verona round A	29.10.2017	CSI-W5*	4	1.40 - 1.60	465	173	13	14.07.2005	G	SF	229	2	74	65	34	1260	61	M	2	2	no	spread	oxer	left	no	0	0	76,17	no
Verona	The Longines FEI World Cup Jumping - Verona round A	29.10.2017	CSI-W5*	4	1.40 - 1.60	465	173	13	14.07.2005	G	SF	229	2	74	65	34	1260	61	M	3	3	no	vertical	vertical	right	no	0	0	76,17	no
Verona	The Longines FEI World Cup Jumping - Verona round A	29.10.2017	CSI-W5*	4	1.40 - 1.60	465	173	13	14.07.2005	G	SF	229	2	74	65	34	1260	61	M	4	4	no	spread	oxer	right	no	0	0	76,17	no
Verona	The Longines FEI World Cup Jumping - Verona round A	29.10.2017	CSI-W5*	4	1.40 - 1.60	465	173	13	14.07.2005	G	SF	229	2	74	65	34	1260	61	M	5	5	no	vertical	vertical with water	straight	no	0	0	76,17	no
Verona	The Longines FEI World Cup Jumping - Verona round A	29.10.2017	CSI-W5*	4	1.40 - 1.60	465	173	13	14.07.2005	G	SF	229	2	74	65	34	1260	61	M	6	6	no	spread	oxer with water	left	no	0	0	76,17	no
Verona	The Longines FEI World Cup Jumping - Verona round A	29.10.2017	CSI-W5*	4	1.40 - 1.60	465	173	13	14.07.2005	G	SF	229	2	74	65	34	1260	61	M	7	7a	yes	spread	oxer	straight	no	0	0	76,17	no
Verona	The Longines FEI World Cup Jumping - Verona round A	29.10.2017	CSI-W5*	4	1.40 - 1.60	465	173	13	14.07.2005	G	SF	229	2	74	65	34	1260	61	M	8	7b	yes	vertical	vertical	straight	no	0	0	76,17	no
Verona	The Longines FEI World Cup Jumping - Verona round A	29.10.2017	CSI-W5*	4	1.40 - 1.60	465	173	13	14.07.2005	G	SF	229	2	74	65	34	1260	61	M	9	8	no	vertical	vertical	left	no	0	0	76,17	no
Verona	The Longines FEI World Cup Jumping - Verona round A	29.10.2017	CSI-W5*	4	1.40 - 1.60	465	173	13	14.07.2005	G	SF	229	2	74	65	34	1260	61	M	10	9	no	spread	oxer	left	no	0	0	76,17	no
Verona	The Longines FEI World Cup Jumping - Verona round A	29.10.2017	CSI-W5*	4	1.40 - 1.60	465	173	13	14.07.2005	G	SF	229	2	74	65	34	1260	61	M	11	10	no	vertical	vertical	right	no	0	0	76,17	no
Verona	The Longines FEI World Cup Jumping - Verona round A	29.10.2017	CSI-W5*	4	1.40 - 1.60	465	173	13	14.07.2005	G	SF	229	2	74	65	34	1260	61	M	12	11a	yes	vertical	vertical	right	no	0	0	76,17	no
Verona	The Longines FEI World Cup Jumping - Verona round A	29.10.2017	CSI-W5*	4	1.40 - 1.60	465	173	13	14.07.2005	G	SF	229	2	74	65	34	1260	61	M	13	11b	yes	spread	oxer	right	no	0	0	76,17	no
Verona	The Longines FEI World Cup Jumping - Verona round A	29.10.2017	CSI-W5*	4	1.40 - 1.60	465	173	13	14.07.2005	G	SF	229	2	74	65	34	1260	61	M	14	11c	yes	spread	oxer	right	no	0	0	76,17	no
Verona	The Longines FEI World Cup Jumping - Verona round A	29.10.2017	CSI-W5*	4	1.40 - 1.60	465	173	13	14.07.2005	G	SF	229	2	74	65	34	1260	61	M	15	12	no	vertical	vertical	left	no	0	0	76,17	no
Verona	The Longines FEI World Cup Jumping - Verona round A	29.10.2017	CSI-W5*	4	1.40 - 1.60	465	173	13	14.07.2005	G	SF	229	2	74	65	34	1260	61	M	16	13	no	spread	oxer	straight	no	0	0	76,17	no
Verona	The Longines FEI World Cup Jumping - Verona round B	29.10.2017	CSI-W5*	4	1.40 - 1.60	290	173	13	14.07.2005	G	SF	229	2	74	65	34	1260	61	M	1	1	no	vertical	vertical	right	no	0	0	38,43	no
Verona	The Longines FEI World Cup Jumping - Verona round B	29.10.2017	CSI-W5*	4	1.40 - 1.60	290	173	13	14.07.2005	G	SF	229	2	74	65	34	1260	61	M	2	2	no	spread	oxer	right	no	0	0	38,43	no
Verona	The Longines FEI World Cup Jumping - Verona round B	29.10.2017	CSI-W5*	4	1.40 - 1.60	290	173	13	14.07.2005	G	SF	229	2	74	65	34	1260	61	M	3	4a	yes	vertical	vertical with water	straight	no	0	0	38,43	no
Verona	The Longines FEI World Cup Jumping - Verona round B	29.10.2017	CSI-W5*	4	1.40 - 1.60	290	173	13	14.07.2005	G	SF	229	2	74	65	34	1260	61	M	4	4b	yes	spread	oxer	straight	no	0	0	38,43	no
Verona	The Longines FEI World Cup Jumping - Verona round B	29.10.2017	CSI-W5*	4	1.40 - 1.60	290	173	13	14.07.2005	G	SF	229	2	74	65	34	1260	61	M	5	5	no	vertical	vertical	left	no	0	0	38,43	no
Verona	The Longines FEI World Cup Jumping - Verona round B	29.10.2017	CSI-W5*	4	1.40 - 1.60	290	173	13	14.07.2005	G	SF	229	2	74	65	34	1260	61	M	6	6	no	spread	oxer	left	no	0	0	38,43	no
Verona	The Longines FEI World Cup Jumping - Verona round B	29.10.2017	CSI-W5*	4	1.40 - 1.60	290	173	13	14.07.2005	G	SF	229	2	74	65	34	1260	61	M	7	7	no	vertical	vertical	right	yes	4	0	38,43	no
Verona	The Longines FEI World Cup Jumping - Verona round B	29.10.2017	CSI-W5*	4	1.40 - 1.60	290	173	13	14.07.2005	G	SF	229	2	74	65	34	1260	61	M	8	7	no	vertical	vertical	right	no	0	0	38,43	no
Verona	The Longines FEI World Cup Jumping - Verona round A	29.10.2017	CSI-W5*	4	1.40 - 1.60	465	192	12	27.04.2006	M	SF	164	14	75	22	53	2104	161	M	1	1	no	vertical	vertical	left	no	0	0	75, 96	no
Verona	The Longines FEI World Cup Jumping - Verona round A	29.10.2017	CSI-W5*	4	1.40 - 1.60	465	192	12	27.04.2006	M	SF	164	14	75	22	53	2104	161	M	2	2	no	spread	oxer	left	no	0	0	75, 96	no
Verona	The Longines FEI World Cup Jumping - Verona round A	29.10.2017	CSI-W5*	4	1.40 - 1.60	465	192	12	27.04.2006	M	SF	164	14	75	22	53	2104	161	M	3	3	no	vertical	vertical	right	no	0	0	75, 96	no
Verona	The Longines FEI World Cup Jumping - Verona round A	29.10.2017	CSI-W5*	4	1.40 - 1.60	465	192	12	27.04.2006	M	SF	164	14	75	22	53	2104	161	M	4	4	no	spread	oxer	right	no	0	0	75, 96	no
Verona	The Longines FEI World Cup Jumping - Verona round A	29.10.2017	CSI-W5*	4	1.40 - 1.60	465	192	12	27.04.2006	M	SF	164	14	75	22	53	2104	161	M	5	5	no	vertical	vertical with water	straight	no	0	0	75, 96	no
Verona	The Longines FEI World Cup Jumping - Verona round A	29.10.2017	CSI-W5*	4	1.40 - 1.60	465	192	12	27.04.2006	M	SF	164	14	75	22	53	2104	161	M	6	6	no	spread	oxer with water	left	no	0	0	75, 96	no
Verona	The Longines FEI World Cup Jumping - Verona round A	29.10.2017	CSI-W5*	4	1.40 - 1.60	465	192	12	27.04.2006	M	SF	164	14	75	22	53	2104	161	M	7	7a	yes	spread	oxer	straight	no	0	0	75, 96	no
Verona	The Longines FEI World Cup Jumping - Verona round A	29.10.2017	CSI-W5*	4	1.40 - 1.60	465	192	12	27.04.2006	M	SF	164	14	75	22	53	2104	161	M	8	7b	yes	vertical	vertical	straight	no	0	0	75, 96	no
Verona	The Longines FEI World Cup Jumping - Verona round A	29.10.2017	CSI-W5*	4	1.40 - 1.60	465	192	12	27.04.2006	M	SF	164	14	75	22	53	2104	161	M	9	8	no	vertical	vertical	left	no	0	0	75, 96	no
Verona	The Longines FEI World Cup Jumping - Verona round A	29.10.2017	CSI-W5*	4	1.40 - 1.60	465	192	12	27.04.2006	M	SF	164	14	75	22	53	2104	161	M	10	9	no	spread	oxer	left	no	0	0	75, 96	no
Verona	The Longines FEI World Cup Jumping - Verona round A	29.10.2017	CSI-W5*	4	1.40 - 1.60	465	192	12	27.04.2006	M	SF	164	14	75	22	53	2104	161	M	11	10	no	vertical	vertical	right	no	0	0	75, 96	no
Verona	The Longines FEI World Cup Jumping - Verona round A	29.10.2017	CSI-W5*	4	1.40 - 1.60	465	192	12	27.04.2006	M	SF	164	14	75	22	53	2104	161	M	12	11a	yes	vertical	vertical	right	no	0	0	75, 96	no
Verona	The Longines FEI World Cup Jumping - Verona round A	29.10.2017	CSI-W5*	4	1.40 - 1.60	465	192	12	27.04.2006	M	SF	164	14	75	22	53	2104	161	M	13	11b	yes	spread	oxer	right	no	0	0	75, 96	no
Verona	The Longines FEI World Cup Jumping - Verona round A	29.10.2017	CSI-W5*	4	1.40 - 1.60	465	192	12	27.04.2006	M	SF	164	14	75	22	53	2104	161	M	14	11c	yes	spread	oxer	right	yes	4	0	75, 96	no
Verona	The Longines FEI World Cup Jumping - Verona round A	29.10.2017	CSI-W5*	4	1.40 - 1.60	465	192	12	27.04.2006	M	SF	164	14	75	22	53	2104	161	M	15	12	no	vertical	vertical	left	no	0	0	75, 96	no
Verona	The Longines FEI World Cup Jumping - Verona round A	29.10.2017	CSI-W5*	4	1.40 - 1.60	465	192	12	27.04.2006	M	SF	164	14	75	22	53	2104	161	M	16	13	no	spread	oxer	straight	no	0	0	75, 96	no
Verona	The Longines FEI World Cup Jumping - Verona round A	29.10.2017	CSI-W5*	4	1.40 - 1.60	465	58	13	04.02.2005	G	DSP	255	4	76	55	44	1886	48	M	1	1	no	vertical	vertical	left	no	0	0	75,14	no
Verona	The Longines FEI World Cup Jumping - Verona round A	29.10.2017	CSI-W5*	4	1.40 - 1.60	465	58	13	04.02.2005	G	DSP	255	4	76	55	44	1886	48	M	2	2	no	spread	oxer	left	no	0	0	75,14	no
Verona	The Longines FEI World Cup Jumping - Verona round A	29.10.2017	CSI-W5*	4	1.40 - 1.60	465	58	13	04.02.2005	G	DSP	255	4	76	55	44	1886	48	M	3	3	no	vertical	vertical	right	no	0	0	75,14	no
Verona	The Longines FEI World Cup Jumping - Verona round A	29.10.2017	CSI-W5*	4	1.40 - 1.60	465	58	13	04.02.2005	G	DSP	255	4	76	55	44	1886	48	M	4	4	no	spread	oxer	right	no	0	0	75,14	no
Verona	The Longines FEI World Cup Jumping - Verona round A	29.10.2017	CSI-W5*	4	1.40 - 1.60	465	58	13	04.02.2005	G	DSP	255	4	76	55	44	1886	48	M	5	5	no	vertical	vertical with water	straight	no	0	0	75,14	no
Verona	The Longines FEI World Cup Jumping - Verona round A	29.10.2017	CSI-W5*	4	1.40 - 1.60	465	58	13	04.02.2005	G	DSP	255	4	76	55	44	1886	48	M	6	6	no	spread	oxer with water	left	no	0	0	75,14	no
Verona	The Longines FEI World Cup Jumping - Verona round A	29.10.2017	CSI-W5*	4	1.40 - 1.60	465	58	13	04.02.2005	G	DSP	255	4	76	55	44	1886	48	M	7	7a	yes	spread	oxer	straight	no	0	0	75,14	no
Verona	The Longines FEI World Cup Jumping - Verona round A	29.10.2017	CSI-W5*	4	1.40 - 1.60	465	58	13	04.02.2005	G	DSP	255	4	76	55	44	1886	48	M	8	7b	yes	vertical	vertical	straight	no	0	0	75,14	no
Verona	The Longines FEI World Cup Jumping - Verona round A	29.10.2017	CSI-W5*	4	1.40 - 1.60	465	58	13	04.02.2005	G	DSP	255	4	76	55	44	1886	48	M	9	8	no	vertical	vertical	left	no	0	0	75,14	no
Verona	The Longines FEI World Cup Jumping - Verona round A	29.10.2017	CSI-W5*	4	1.40 - 1.60	465	58	13	04.02.2005	G	DSP	255	4	76	55	44	1886	48	M	10	9	no	spread	oxer	left	no	0	0	75,14	no
Verona	The Longines FEI World Cup Jumping - Verona round A	29.10.2017	CSI-W5*	4	1.40 - 1.60	465	58	13	04.02.2005	G	DSP	255	4	76	55	44	1886	48	M	11	10	no	vertical	vertical	right	no	0	0	75,14	no
Verona	The Longines FEI World Cup Jumping - Verona round A	29.10.2017	CSI-W5*	4	1.40 - 1.60	465	58	13	04.02.2005	G	DSP	255	4	76	55	44	1886	48	M	12	11a	yes	vertical	vertical	right	no	0	0	75,14	no
Verona	The Longines FEI World Cup Jumping - Verona round A	29.10.2017	CSI-W5*	4	1.40 - 1.60	465	58	13	04.02.2005	G	DSP	255	4	76	55	44	1886	48	M	13	11b	yes	spread	oxer	right	no	0	0	75,14	no
Verona	The Longines FEI World Cup Jumping - Verona round A	29.10.2017	CSI-W5*	4	1.40 - 1.60	465	58	13	04.02.2005	G	DSP	255	4	76	55	44	1886	48	M	14	11c	yes	spread	oxer	right	no	0	0	75,14	no
Verona	The Longines FEI World Cup Jumping - Verona round A	29.10.2017	CSI-W5*	4	1.40 - 1.60	465	58	13	04.02.2005	G	DSP	255	4	76	55	44	1886	48	M	15	12	no	vertical	vertical	left	no	0	0	75,14	no
Verona	The Longines FEI World Cup Jumping - Verona round A	29.10.2017	CSI-W5*	4	1.40 - 1.60	465	58	13	04.02.2005	G	DSP	255	4	76	55	44	1886	48	M	16	13	no	spread	oxer	straight	no	0	0	75,14	no
Verona	The Longines FEI World Cup Jumping - Verona round A	29.10.2017	CSI-W5*	4	1.40 - 1.60	465	95	13	17.04.2005	G	KWPN	146	6	77	27	33	1671	77	M	1	1	no	vertical	vertical	left	no	0	0	74,19	no
Verona	The Longines FEI World Cup Jumping - Verona round A	29.10.2017	CSI-W5*	4	1.40 - 1.60	465	95	13	17.04.2005	G	KWPN	146	6	77	27	33	1671	77	M	2	2	no	spread	oxer	left	no	0	0	74,19	no
Verona	The Longines FEI World Cup Jumping - Verona round A	29.10.2017	CSI-W5*	4	1.40 - 1.60	465	95	13	17.04.2005	G	KWPN	146	6	77	27	33	1671	77	M	3	3	no	vertical	vertical	right	no	0	0	74,19	no
Verona	The Longines FEI World Cup Jumping - Verona round A	29.10.2017	CSI-W5*	4	1.40 - 1.60	465	95	13	17.04.2005	G	KWPN	146	6	77	27	33	1671	77	M	4	4	no	spread	oxer	right	no	0	0	74,19	no
Verona	The Longines FEI World Cup Jumping - Verona round A	29.10.2017	CSI-W5*	4	1.40 - 1.60	465	95	13	17.04.2005	G	KWPN	146	6	77	27	33	1671	77	M	5	5	no	vertical	vertical with water	straight	no	0	0	74,19	no
Verona	The Longines FEI World Cup Jumping - Verona round A	29.10.2017	CSI-W5*	4	1.40 - 1.60	465	95	13	17.04.2005	G	KWPN	146	6	77	27	33	1671	77	M	6	6	no	spread	oxer with water	left	no	0	0	74,19	no
Verona	The Longines FEI World Cup Jumping - Verona round A	29.10.2017	CSI-W5*	4	1.40 - 1.60	465	95	13	17.04.2005	G	KWPN	146	6	77	27	33	1671	77	M	7	7a	yes	spread	oxer	straight	yes	4	0	74,19	no
Verona	The Longines FEI World Cup Jumping - Verona round A	29.10.2017	CSI-W5*	4	1.40 - 1.60	465	95	13	17.04.2005	G	KWPN	146	6	77	27	33	1671	77	M	8	7b	yes	vertical	vertical	straight	no	0	0	74,19	no
Verona	The Longines FEI World Cup Jumping - Verona round A	29.10.2017	CSI-W5*	4	1.40 - 1.60	465	95	13	17.04.2005	G	KWPN	146	6	77	27	33	1671	77	M	9	8	no	vertical	vertical	left	no	0	0	74,19	no
Verona	The Longines FEI World Cup Jumping - Verona round A	29.10.2017	CSI-W5*	4	1.40 - 1.60	465	95	13	17.04.2005	G	KWPN	146	6	77	27	33	1671	77	M	10	9	no	spread	oxer	left	no	0	0	74,19	no
Verona	The Longines FEI World Cup Jumping - Verona round A	29.10.2017	CSI-W5*	4	1.40 - 1.60	465	95	13	17.04.2005	G	KWPN	146	6	77	27	33	1671	77	M	11	10	no	vertical	vertical	right	no	0	0	74,19	no
Verona	The Longines FEI World Cup Jumping - Verona round A	29.10.2017	CSI-W5*	4	1.40 - 1.60	465	95	13	17.04.2005	G	KWPN	146	6	77	27	33	1671	77	M	12	11a	yes	vertical	vertical	right	no	0	0	74,19	no
Verona	The Longines FEI World Cup Jumping - Verona round A	29.10.2017	CSI-W5*	4	1.40 - 1.60	465	95	13	17.04.2005	G	KWPN	146	6	77	27	33	1671	77	M	13	11b	yes	spread	oxer	right	no	0	0	74,19	no
Verona	The Longines FEI World Cup Jumping - Verona round A	29.10.2017	CSI-W5*	4	1.40 - 1.60	465	95	13	17.04.2005	G	KWPN	146	6	77	27	33	1671	77	M	14	11c	yes	spread	oxer	right	no	0	0	74,19	no

Verona	The Longines FEI World Cup Jumping - Verona round A	29.10.2017	CSI-W5*	4	1.40 - 1.60	465	95	13	17.04.2005	G	KWPN	146	6	77	27	33	1671	77	M	15	12	no	vertical	vertical	left	no	0	0	74,19	no
Verona	The Longines FEI World Cup Jumping - Verona round A	29.10.2017	CSI-W5*	4	1.40 - 1.60	465	95	13	17.04.2005	G	KWPN	146	6	77	27	33	1671	77	M	16	13	no	spread	oxer	straight	no	0	0	74,19	no
Verona	The Longines FEI World Cup Jumping - Verona round A	29.10.2017	CSI-W5*	4	1.40 - 1.60	465	46	14	09.06.2004	G	HOLST	151	7	78	303	54	1104	33	M	1	1	no	vertical	vertical	left	no	0	0	73,08	no
Verona	The Longines FEI World Cup Jumping - Verona round A	29.10.2017	CSI-W5*	4	1.40 - 1.60	465	46	14	09.06.2004	G	HOLST	151	7	78	303	54	1104	33	M	2	2	no	spread	oxer	left	no	0	0	73,08	no
Verona	The Longines FEI World Cup Jumping - Verona round A	29.10.2017	CSI-W5*	4	1.40 - 1.60	465	46	14	09.06.2004	G	HOLST	151	7	78	303	54	1104	33	M	3	3	no	vertical	vertical	right	no	0	0	73,08	no
Verona	The Longines FEI World Cup Jumping - Verona round A	29.10.2017	CSI-W5*	4	1.40 - 1.60	465	46	14	09.06.2004	G	HOLST	151	7	78	303	54	1104	33	M	4	4	no	spread	oxer	right	no	0	0	73,08	no
Verona	The Longines FEI World Cup Jumping - Verona round A	29.10.2017	CSI-W5*	4	1.40 - 1.60	465	46	14	09.06.2004	G	HOLST	151	7	78	303	54	1104	33	M	5	5	no	vertical	vertical with water	straight	no	0	0	73,08	no
Verona	The Longines FEI World Cup Jumping - Verona round A	29.10.2017	CSI-W5*	4	1.40 - 1.60	465	46	14	09.06.2004	G	HOLST	151	7	78	303	54	1104	33	M	6	6	no	spread	oxer with water	left	no	0	0	73,08	no
Verona	The Longines FEI World Cup Jumping - Verona round A	29.10.2017	CSI-W5*	4	1.40 - 1.60	465	46	14	09.06.2004	G	HOLST	151	7	78	303	54	1104	33	M	7	7a	yes	spread	oxer	straight	no	0	0	73,08	no
Verona	The Longines FEI World Cup Jumping - Verona round A	29.10.2017	CSI-W5*	4	1.40 - 1.60	465	46	14	09.06.2004	G	HOLST	151	7	78	303	54	1104	33	M	8	7b	yes	vertical	vertical	straight	no	0	0	73,08	no
Verona	The Longines FEI World Cup Jumping - Verona round A	29.10.2017	CSI-W5*	4	1.40 - 1.60	465	46	14	09.06.2004	G	HOLST	151	7	78	303	54	1104	33	M	9	8	no	vertical	vertical	left	yes	4	0	73,08	no
Verona	The Longines FEI World Cup Jumping - Verona round A	29.10.2017	CSI-W5*	4	1.40 - 1.60	465	46	14	09.06.2004	G	HOLST	151	7	78	303	54	1104	33	M	10	9	no	spread	oxer	left	no	0	0	73,08	no
Verona	The Longines FEI World Cup Jumping - Verona round A	29.10.2017	CSI-W5*	4	1.40 - 1.60	465	46	14	09.06.2004	G	HOLST	151	7	78	303	54	1104	33	M	11	10	no	vertical	vertical	right	no	0	0	73,08	no
Verona	The Longines FEI World Cup Jumping - Verona round A	29.10.2017	CSI-W5*	4	1.40 - 1.60	465	46	14	09.06.2004	G	HOLST	151	7	78	303	54	1104	33	M	12	11a	yes	vertical	vertical	right	no	0	0	73,08	no
Verona	The Longines FEI World Cup Jumping - Verona round A	29.10.2017	CSI-W5*	4	1.40 - 1.60	465	46	14	09.06.2004	G	HOLST	151	7	78	303	54	1104	33	M	13	11b	yes	spread	oxer	right	yes	4	0	73,08	no
Verona	The Longines FEI World Cup Jumping - Verona round A	29.10.2017	CSI-W5*	4	1.40 - 1.60	465	46	14	09.06.2004	G	HOLST	151	7	78	303	54	1104	33	M	14	11c	yes	spread	oxer	right	no	0	0	73,08	no
Verona	The Longines FEI World Cup Jumping - Verona round A	29.10.2017	CSI-W5*	4	1.40 - 1.60	465	46	14	09.06.2004	G	HOLST	151	7	78	303	54	1104	33	M	15	12	no	vertical	vertical	left	no	0	0	73,08	no
Verona	The Longines FEI World Cup Jumping - Verona round A	29.10.2017	CSI-W5*	4	1.40 - 1.60	465	46	14	09.06.2004	G	HOLST	151	7	78	303	54	1104	33	M	16	13	no	spread	oxer	straight	no	0	0	73,08	no
Lyon	CSI5*-W presented by GL EVENTS round A	05.11.2017	CSI-W5*	3	1.40 - 1.60	420	39	10	23.05.2008	M	ZANG	150	1	5	15	32	2092	63	M	1	1	no	spread	oxer	right	no	0	0	68,86	no
Lyon	CSI5*-W presented by GL EVENTS round A	05.11.2017	CSI-W5*	3	1.40 - 1.60	420	39	10	23.05.2008	M	ZANG	150	1	5	15	32	2092	63	M	2	2	no	vertical	vertical	right	no	0	0	68,86	no
Lyon	CSI5*-W presented by GL EVENTS round A	05.11.2017	CSI-W5*	3	1.40 - 1.60	420	39	10	23.05.2008	M	ZANG	150	1	5	15	32	2092	63	M	3	3	no	spread	oxer	right	no	0	0	68,86	no
Lyon	CSI5*-W presented by GL EVENTS round A	05.11.2017	CSI-W5*	3	1.40 - 1.60	420	39	10	23.05.2008	M	ZANG	150	1	5	15	32	2092	63	M	4	4	no	spread	oxer	left	no	0	0	68,86	no
Lyon	CSI5*-W presented by GL EVENTS round A	05.11.2017	CSI-W5*	3	1.40 - 1.60	420	39	10	23.05.2008	M	ZANG	150	1	5	15	32	2092	63	M	5	5	no	vertical	vertical	straight	no	0	0	68,86	no
Lyon	CSI5*-W presented by GL EVENTS round A	05.11.2017	CSI-W5*	3	1.40 - 1.60	420	39	10	23.05.2008	M	ZANG	150	1	5	15	32	2092	63	M	6	6	no	vertical	vertical	left	no	0	0	68,86	no
Lyon	CSI5*-W presented by GL EVENTS round A	05.11.2017	CSI-W5*	3	1.40 - 1.60	420	39	10	23.05.2008	M	ZANG	150	1	5	15	32	2092	63	M	7	7a	yes	spread	oxer	straight	no	0	0	68,86	no
Lyon	CSI5*-W presented by GL EVENTS round A	05.11.2017	CSI-W5*	3	1.40 - 1.60	420	39	10	23.05.2008	M	ZANG	150	1	5	15	32	2092	63	M	8	7b	yes	vertical	vertical	straight	no	0	0	68,86	no
Lyon	CSI5*-W presented by GL EVENTS round A	05.11.2017	CSI-W5*	3	1.40 - 1.60	420	39	10	23.05.2008	M	ZANG	150	1	5	15	32	2092	63	M	9	7c	yes	spread	oxer	straight	no	0	0	68,86	no
Lyon	CSI5*-W presented by GL EVENTS round A	05.11.2017	CSI-W5*	3	1.40 - 1.60	420	39	10	23.05.2008	M	ZANG	150	1	5	15	32	2092	63	M	10	8	no	vertical	wall	right	no	0	0	68,86	no
Lyon	CSI5*-W presented by GL EVENTS round A	05.11.2017	CSI-W5*	3	1.40 - 1.60	420	39	10	23.05.2008	M	ZANG	150	1	5	15	32	2092	63	M	11	9	no	vertical	vertical	right	no	0	0	68,86	no
Lyon	CSI5*-W presented by GL EVENTS round A	05.11.2017	CSI-W5*	3	1.40 - 1.60	420	39	10	23.05.2008	M	ZANG	150	1	5	15	32	2092	63	M	12	10	no	spread	oxer	right	no	0	0	68,86	no
Lyon	CSI5*-W presented by GL EVENTS round A	05.11.2017	CSI-W5*	3	1.40 - 1.60	420	39	10	23.05.2008	M	ZANG	150	1	5	15	32	2092	63	M	13	11	no	spread	oxer	left	no	0	0	68,86	no
Lyon	CSI5*-W presented by GL EVENTS round A	05.11.2017	CSI-W5*	3	1.40 - 1.60	420	39	10	23.05.2008	M	ZANG	150	1	5	15	32	2092	63	M	14	12a	yes	vertical	vertical	left	no	0	0	68,86	no
Lyon	CSI5*-W presented by GL EVENTS round A	05.11.2017	CSI-W5*	3	1.40 - 1.60	420	39	10	23.05.2008	M	ZANG	150	1	5	15	32	2092	63	M	15	12b	yes	vertical	vertical with water	left	no	0	0	68,86	no
Lyon	CSI5*-W presented by GL EVENTS round A	05.11.2017	CSI-W5*	3	1.40 - 1.60	420	39	10	23.05.2008	M	ZANG	150	1	5	15	32	2092	63	M	16	13	no	spread	oxer with water	straight	no	0	0	68,86	no
Lyon	CSI5*-W presented by GL EVENTS round B	05.11.2017	CSI-W5*	3	1.40 - 1.60	280	39	10	23.05.2008	M	ZANG	150	1	5	15	32	2092	63	M	1	1	no	vertical	vertical	left	no	0	0	39,28	no
Lyon	CSI5*-W presented by GL EVENTS round B	05.11.2017	CSI-W5*	3	1.40 - 1.60	280	39	10	23.05.2008	M	ZANG	150	1	5	15	32	2092	63	M	2	2	no	vertical	vertical	left	no	0	0	39,28	no
Lyon	CSI5*-W presented by GL EVENTS round B	05.11.2017	CSI-W5*	3	1.40 - 1.60	280	39	10	23.05.2008	M	ZANG	150	1	5	15	32	2092	63	M	3	3	no	spread	oxer	right	no	0	0	39,28	no
Lyon	CSI5*-W presented by GL EVENTS round B	05.11.2017	CSI-W5*	3	1.40 - 1.60	280	39	10	23.05.2008	M	ZANG	150	1	5	15	32	2092	63	M	4	4	no	spread	oxer	left	no	0	0	39,28	no
Lyon	CSI5*-W presented by GL EVENTS round B	05.11.2017	CSI-W5*	3	1.40 - 1.60	280	39	10	23.05.2008	M	ZANG	150	1	5	15	32	2092	63	M	5	5a	yes	vertical	vertical	straight	no	0	0	39,28	no
Lyon	CSI5*-W presented by GL EVENTS round B	05.11.2017	CSI-W5*	3	1.40 - 1.60	280	39	10	23.05.2008	M	ZANG	150	1	5	15	32	2092	63	M	6	5b	yes	vertical	vertical with water	straight	no	0	0	39,28	no
Lyon	CSI5*-W presented by GL EVENTS round B	05.11.2017	CSI-W5*	3	1.40 - 1.60	280	39	10	23.05.2008	M	ZANG	150	1	5	15	32	2092	63	M	7	6	no	spread	oxer	left	no	0	0	39,28	no
Lyon	CSI5*-W presented by GL EVENTS round B	05.11.2017	CSI-W5*	3	1.40 - 1.60	280	39	10	23.05.2008	M	ZANG	150	1	5	15	32	2092	63	M	8	7	no	spread	oxer	right	no	0	0	39,28	no
Lyon	CSI5*-W presented by GL EVENTS round A	05.11.2017	CSI-W5*	3	1.40 - 1.60	420	88	13	19.04.2005	S	BWP	131	0	6	421	45	483	7	F	1	1	no	spread	oxer	right	no	0	0	69,73	no
Lyon	CSI5*-W presented by GL EVENTS round A	05.11.2017	CSI-W5*	3	1.40 - 1.60	420	88	13	19.04.2005	S	BWP	131	0	6	421	45	483	7	F	2	2	no	vertical	vertical	right	no	0	0	69,73	no
Lyon	CSI5*-W presented by GL EVENTS round A	05.11.2017	CSI-W5*	3	1.40 - 1.60	420	88	13	19.04.2005	S	BWP	131	0	6	421	45	483	7	F	3	3	no	spread	oxer	right	no	0	0	69,73	no
Lyon	CSI5*-W presented by GL EVENTS round A	05.11.2017	CSI-W5*	3	1.40 - 1.60	420	88	13	19.04.2005	S	BWP	131	0	6	421	45	483	7	F	4	4	no	spread	oxer	left	no	0	0	69,73	no
Lyon	CSI5*-W presented by GL EVENTS round A	05.11.2017	CSI-W5*	3	1.40 - 1.60	420	88	13	19.04.2005	S	BWP	131	0	6	421	45	483	7	F	5	5	no	vertical	vertical	straight	no	0	0	69,73	no
Lyon	CSI5*-W presented by GL EVENTS round A	05.11.2017	CSI-W5*	3	1.40 - 1.60	420	88	13	19.04.2005	S	BWP	131	0	6	421	45	483	7	F	6	6	no	vertical	vertical	left	no	0	0	69,73	no
Lyon	CSI5*-W presented by GL EVENTS round A	05.11.2017	CSI-W5*	3	1.40 - 1.60	420	88	13	19.04.2005	S	BWP	131	0	6	421	45	483	7	F	7	7a	yes	spread	oxer	straight	no	0	0	69,73	no
Lyon	CSI5*-W presented by GL EVENTS round A	05.11.2017	CSI-W5*	3	1.40 - 1.60	420	88	13	19.04.2005	S	BWP	131	0	6	421	45	483	7	F	8	7b	yes	vertical	vertical	straight	no	0	0	69,73	no
Lyon	CSI5*-W presented by GL EVENTS round A	05.11.2017	CSI-W5*	3	1.40 - 1.60	420	88	13	19.04.2005	S	BWP	131	0	6	421	45	483	7	F	9	7c	yes	spread	oxer	straight	no	0	0	69,73	no
Lyon	CSI5*-W presented by GL EVENTS round A	05.11.2017	CSI-W5*	3	1.40 - 1.60	420	88	13	19.04.2005	S	BWP	131	0	6	421	45	483	7	F	10	8	no	vertical	wall	right	no	0	0	69,73	no
Lyon	CSI5*-W presented by GL EVENTS round A	05.11.2017	CSI-W5*	3	1.40 - 1.60	420	88	13	19.04.2005	S	BWP	131	0	6	421	45	483	7	F	11	9	no	vertical	vertical	right	no	0	0	69,73	no
Lyon	CSI5*-W presented by GL EVENTS round A	05.11.2017	CSI-W5*	3	1.40 - 1.60	420	88	13	19.04.2005	S	BWP	131	0	6	421	45	483	7	F	12	10	no	spread	oxer	right	no	0	0	69,73	no
Lyon	CSI5*-W presented by GL EVENTS round A	05.11.2017	CSI-W5*	3	1.40 - 1.60	420	88	13	19.04.2005	S	BWP	131	0	6	421	45	483	7	F	13	11	no	spread	oxer	left	no	0	0	69,73	no
Lyon	CSI5*-W presented by GL EVENTS round A	05.11.2017	CSI-W5*	3	1.40 - 1.60	420	88	13	19.04.2005	S	BWP	131	0	6	421	45	483	7	F	14	12a	yes	vertical	vertical	left	no	0	0	69,73	no
Lyon	CSI5*-W presented by GL EVENTS round A	05.11.2017	CSI-W5*	3	1.40 - 1.60	420	88	13	19.04.2005	S	BWP	131	0	6	421	45	483	7	F	15	12b	yes	vertical	vertical with water	left	yes	4	0	69,73	no
Lyon	CSI5*-W presented by GL EVENTS round A	05.11.2017	CSI-W5*	3	1.40 - 1.60	420	88	13	19.04.2005	S	BWP	131	0	6	421	45	483	7	F	16	13	no	spread	oxer with water	straight	yes	4	0	69,73	no
Lyon	CSI5*-W presented by GL EVENTS round A	05.11.2017	CSI-W5*	3	1.40 - 1.60	420	20	11	16.04.2007	G	OS	121	0	7	48	58	1857	49	M	1	1	no	spread	oxer	right	no	0	0	67,93	no
Lyon	CSI5*-W presented by GL EVENTS round A	05.11.2017	CSI-W5*	3	1.40 - 1.60	420	20	11	16.04.2007	G	OS	121	0	7	48	58	1857	49	M	2	2	no	vertical	vertical	right	no	0	0	67,93	no
Lyon	CSI5*-W presented by GL EVENTS round A	05.11.2017	CSI-W5*	3	1.40 - 1.60	420	20	11	16.04.2007	G	OS	121	0	7	48	58	1857	49	M	3	3	no	spread	oxer	right	no	0	0	67,93	no
Lyon	CSI5*-W presented by GL EVENTS round A	05.11.2017	CSI-W5*	3	1.40 - 1.60	420	20	11	16.04.2007	G	OS	121	0	7	48	58	1857	49	M	4	4	no	spread	oxer	left	no	0	0	67,93	no
Lyon	CSI5*-W presented by GL EVENTS round A	05.11.2017	CSI-W5*	3	1.40 - 1.60	420	20	11	16.04.2007	G	OS	121	0	7	48	58	1857	49	M	5	5	no	vertical	vertical	straight	no	0	0	67,93	no
Lyon	CSI5*-W presented by GL EVENTS round A	05.11.2017	CSI-W5*	3	1.40 - 1.60	420	20	11	16.04.2007	G	OS	121	0	7	48	58	1857	49	M	6	6	no	vertical	vertical	left	no	0	0	67,93	no
Lyon	CSI5*-W presented by GL EVENTS round A	05.11.2017	CSI-W5*	3	1.40 - 1.60	420	20	11	16.04.2007	G	OS	121	0	7	48	58	1857	49	M	7	7a	yes	spread	oxer	straight	no	0	0	67,93	no
Lyon	CSI5*-W presented by GL EVENTS round A	05.11.2017	CSI-W5*	3	1.40 - 1.60	420	20	11	16.04.2007	G	OS	121	0	7	48	58	1857	49	M	8	7b	yes	vertical	vertical	straight	no	0	0	67,93	no
Lyon	CSI5*-W presented by GL EVENTS round A	05.11.2017	CSI-W5*	3	1.40 - 1.60	420	20	11	16.04.2007	G	OS	121	0	7	48	58	1857	49	M	9	7c	yes	spread	oxer	straight	no	0	0	67,93	no
Lyon	CSI5*-W presented by GL EVENTS round A	05.11.2017	CSI-W5*	3	1.40 - 1.60	420	20	11	16.04.2007	G	OS	121	0	7	48	58	1857	49	M	10	8	no	vertical	wall	right	no	0	0	67,93	no
Lyon	CSI5*-W presented by GL EVENTS round A	05.11.2017	CSI-W5*	3	1.40 - 1.60	420	20	11	16.04.2007	G	OS	121	0	7	48	58	1857	49	M	11	9	no	vertical	vertical	right	no	0	0	67,93	no
Lyon	CSI5*-W presented by GL EVENTS round A	05.11.2017	CSI-W5*	3	1.40 - 1.60	420	20	11	16.04.2007	G	OS	121	0	7	48	58	1857	49	M	12	10	no	spread	oxer	right	no	0	0	67,93	no
Lyon	CSI5*-W presented by GL EVENTS round A	05.11.2017	CSI-W5*	3	1.40 - 1.60	420	20	11	16.04.2007	G	OS	121	0	7	48	58	1857	49	M	13	11	no	spread	oxer	left	no	0	0	67,93	no
Lyon	CSI5*-W presented by GL EVENTS round A	05.11.2017	CSI-W5*	3	1.40 - 1.60	420	20	11	16.04.2007	G	OS	121	0	7	48	58	1857	49	M	14	12a	yes	vertical	vertical	left	no	0	0	67,93	no
Lyon	CSI5*-W presented by GL EVENTS round A	05.11.2017	CSI-W5*	3	1.40 - 1.60	420	20	11	16.04.2007	G	OS	121	0	7	48	58	1857	49	M	15	12b	yes	vertical	vertical with water	left	no	0	0	67,93	no
Lyon	CSI5*-W presented by GL EVENTS round A	05.11.2017	CSI-W5*	3	1.40 - 1.60	420	20	11	16.04.2007	G	OS	121	0	7	48	58	1857	49	M	16	13	no	spread	oxer with water	straight	no	0	0	67,93	no
Lyon	CSI5*-W presented by GL EVENTS round B	05.11.2017	CSI-W5*	3	1.40 - 1.60	280	20	11	16.04.2007	G	OS	121	0	7	48	58	1857	49	M	1	1	no	vertical	vertical	left	no	0	0	40,01	no
Lyon	CSI5*-W presented by GL EVENTS round B	05.11.2017	CSI-W5*	3	1.40 - 1.60	280	20	11	16.04.2007	G	OS	121	0	7	48	58	1857	49	M	2	2	no	vertical	vertical	left	no	0	0	40,01	no
Lyon	CSI5*-W presented by GL EVENTS round B	05.11.2017	CSI-W5*	3	1.40 - 1.60	280	20	11	16.04.2007	G	OS	121	0	7	48	58	1857	49	M	3	3	no	spread	oxer	right	no	0	0	40,01	no
Lyon	CSI5*-W presented by GL EVENTS round B	05.11.2017	CSI-W5*	3	1.40 - 1.60	280	20	11	16.04.2007	G	OS	121	0	7	48	58	1857	49	M	4	4	no	spread	oxer	left	no	0	0	40,01	no
Lyon	CSI5*-W presented by GL EVENTS round B	05.11.2017	CSI-W5*	3	1.40 - 1.60	280	20	11	16.04.2007	G	OS	121	0	7	48	58	1857	49	M	5	5a	yes	vertical	vertical	straight	no	0	0	40,01	no
Lyon	CSI5*-W presented by GL EVENTS round B	05.11.2017	CSI-W5*	3	1.40 - 1.60	280	20	11	16.04.2007	G	OS	121	0	7	48	58	1857	49	M	6	5b	yes	vertical	vertical with water	straight	no	0	0	40,01	no
Lyon	CSI5*-W presented by GL EVENTS round B	05.11.2017	CSI-W5*	3	1.40 - 1.60	280	20	11	16.04.2007	G	OS	121	0	7	48	58	1857	49	M	7	6	no	spread	oxer	left	no	0	0	40,01	no
Lyon	CSI5*-W presented by GL EVENTS round B	05.11.2017	CSI-W5*	3	1.40 - 1.60	280	20	11	16.04.2007	G	OS	121	0	7	48	58	1857	49	M	8	7	no	spread	oxer	right	no	0	0	40,01	no
Lyon	CSI5*-W presented by GL EVENTS round A	05.11.2017	CSI-W5*	3	1.40 - 1.60	420	195	11	25.04.2007	G	SF	164	16	9	103	48	1417	59	M	1	1	no	spread	oxer	right	no	0	0	69,32	no
Lyon	CSI5*-W presented by GL EVENTS round A	05.11.2017	CSI-W5*	3	1.40 - 1.60	420	195	11	25.04.2007	G	SF	164	16	9	103	48	1417	59	M	2	2	no	vertical	vertical	right	no	0	0	69,32	no
Lyon	CSI5*-W presented by GL EVENTS round A	05.11.2017	CSI-W5*	3	1.40 - 1.60	420	195	11	25.04.2007	G	SF	164	16	9	103	48	1417	59	M	3	3	no	spread	oxer	right	no	0	0	69,32	no
Lyon	CSI5*-W presented by GL EVENTS round A	05.11.2017	CSI-W5*	3	1.40 - 1.60	420	195	11	25.04.2007	G	SF	164	16	9	103	48	1417	59	M	4	4	no	spread	oxer	left	no	0	0	69,32	no
Lyon	CSI5*-W presented by GL EVENTS round A	05.11.2017	CSI-W5*	3	1.40 - 1.60	420	195	11	25.04.2007	G	SF	164	16	9	103	48	1417	59	M	5	5	no	vertical	vertical	straight	yes	4	0	69,32	no
Lyon	CSI5*-W presented by GL EVENTS round A	05.11.2017	CSI-W5*	3	1.40 - 1.60	420	195	11	25.04.2007	G	SF	164	16	9	103	48	1417	59	M	6	6	no	vertical	vertical	left	no	0	0	69,32	no
Lyon	CSI5*-W presented by GL EVENTS round A	05.11.2017	CSI-W5*	3	1.40 - 1.60	420	195	11	25.04.2007	G	SF	164	16	9	103	48	1417	59	M	7	7a	yes	spread	oxer	straight	no	0	0	69,32	no
Lyon	CSI5*-W presented by GL EVENTS round A	05.11.2017	CSI-W5*	3	1.40 - 1.60	420	195	11	25.04.2007	G	SF	164	16	9	103	48	1417	59	M	8	7b	yes	vertical	vertical	straight	yes	4	0	69,32	no
Lyon	CSI5*-W presented by GL EVENTS round A	05.11.2017	CSI-W5*	3	1.40 - 1.60	420	195	11	25.04.2007	G	SF	164	16	9	103	48	1417	59	M	9	7c	yes	spread	oxer	straight	no	0	0	69,32	no
Lyon	CSI5*-W presented by GL EVENTS round A	05.11.2017	CSI-W5*	3	1.40 - 1.60	420	195	11	25.04.2007	G	SF	164	16	9	103	48	1417	59	M	10	8	no	vertical	wall	right	no	0	0	69,32	no
Lyon	CSI5*-W presented by GL EVENTS round A	05.11.2017	CSI-W5*	3	1.40 - 1.60	420	195	11	25.04.2007	G	SF	164	16	9	103	48	1417	59	M	11	9	no	vertical	vertical	right	no	0	0	69,32	no
Lyon	CSI5*-W presented by GL EVENTS round A	05.11.2017	CSI-W5*	3	1.40 - 1.60	420	195	11	25.04.2007	G	SF	164	16	9	103	48	1417	59	M	12	10	no	spread	oxer	right	no	0	0	69,32	no
Lyon	CSI5*-W presented by GL EVENTS round A	05.11.2017	CSI-W5*	3	1.40 - 1.60	420	195	11	25.04.2007	G	SF	164	16	9	103	48	1417	59	M	13	11	no	spread	oxer	left	no	0	0	69,32	no
Lyon	CSI5*-W presented by GL EVENTS round A	05.11.2017	CSI-W5*	3	1.40 - 1.60	420	195	11	25.04.2007	G	SF	164	16	9	103	48	1417	59	M	14	12a	yes	vertical	vertical	left	no	0	0	69,32	no
Lyon	CSI5*-W presented by GL EVENTS round A	05.11.2017	CSI-W5*	3	1.40 - 1.60	420	195	11	25.04.2007	G	SF	164	16	9	103	48	1417	59	M	15	12b	yes	vertical	vertical with water	left	no	0	0	69,32	no
Lyon	CSI5*-W presented by GL EVENTS round A	05.11.2017	CSI-W5*	3	1.40 - 1.60	420	195	11	25.04.2007	G	SF	164	16	9	103	48	1417	59	M	16	13	no	spread	oxer with water	straight	no	0	0	69,32	no
Lyon	CSI5*-W presented by GL EVENTS round A	05.11.2017	CSI-W5*	3	1.40 - 1.60	420	112	13	01.06.2005	G	SF	182	8	14	5	37	1868	96	M	1	1	no	spread	oxer	right	no	0	0	67,4	no
Lyon	CSI5*-W presented by GL EVENTS round A	05.11.2017	CSI-W5*	3	1.40 - 1.60	420	112	13	01.06.2005	G	SF	182	8	14	5	37	1868	96	M	2	2	no	vertical	vertical	right	no	0	0	67,4	no
Lyon	CSI5*-W presented by GL EVENTS round A	05.11.2017	CSI-W5*	3	1.40 - 1.60	420	112	13	01.06.2005	G	SF	182	8	14	5	37	1868	96	M	3	3	no	spread	oxer	right	no	0	0	67,4	no
Lyon	CSI5*-W presented by GL EVENTS round A	05.11.2017	CSI-W5*	3	1.40 - 1.60	420	112	13	01.06.2005	G	SF	182	8	14	5	37	1868	96	M	4	4	no	spread	oxer	left	no	0	0	67,4	no
Lyon	CSI5*-W presented by GL EVENTS round A	05.11.2017	CSI-W5*	3	1.40 - 1.60	420	112	13	01.06.2005	G	SF	182	8	14	5	37	1868	96	M	5	5	no	vertical	vertical	straight	no	0	0	67,4	no
Lyon	CSI5*-W presented by GL EVENTS round A	05.11.2017	CSI-W5*	3	1.40 - 1.60	420	112	13	01.06.2005	G	SF	182	8	14	5	37	1868	96	M	6	6	no	vertical	vertical	left	no	0	0	67,4	no
Lyon	CSI5*-W presented by GL EVENTS round A	05.11.2017	CSI-W5*	3	1.40 - 1.60	420	112	13	01.06.2005	G	SF	182	8	14	5	37	1868	96	M	7	7a	yes	spread	oxer	straight	no	0	0	67,4	no
Lyon	CSI5*-W presented by GL EVENTS round A	05.11.2017	CSI-W5*	3	1.40 - 1.60	420	112	13	01.06.2005	G	SF	182	8	14	5	37	1868	96	M	8	7b	yes	vertical	vertical	straight	no	0	0	67,4	no
Lyon	CSI5*-W presented by GL EVENTS round A	05.11.2017	CSI-W5*	3	1.40 - 1.60	420	112	13	01.06.2005	G	SF	182	8	14	5	37	1868	96	M	9	7c	yes	spread	oxer	straight	no	0	0	67,4	no
Lyon	CSI5*-W presented by GL EVENTS round A	05.11.2017	CSI-W5*	3	1.40 - 1.60	420	112	13	01.06.2005	G	SF	182	8	14	5	37	1868	96	M	10	8	no	vertical	wall	right	no	0	0	67,4	no
Lyon	CSI5*-W presented by GL EVENTS round A	05.11.2017	CSI-W5*	3	1.40 - 1.60	420	112	13	01.06.2005	G	SF	182	8	14	5	37	1868	96	M	11	9	no	vertical	vertical	right	no	0	0	67,4	no
Lyon	CSI5*-W presented by GL EVENTS round A	05.11.2017	CSI-W5*	3	1.40 - 1.60	420	112	13	01.06.2005	G	SF	182	8	14	5	37	1868	96	M	12	10	no	spread	oxer	right	no	0	0	67,4	no
Lyon	CSI5*-W presented by GL EVENTS round A	05.11.2017	CSI-W5*	3	1.40 - 1.60	420	112	13	01.06.2005	G	SF	182	8	14	5	37	1868	96	M	13	11	no	spread	oxer	left	no	0	0	67,4	no
Lyon	CSI5*-W presented by GL EVENTS round A	05.11.2017	CSI-W5*	3	1.40 - 1.60	420	112	13	01.06.2005	G	SF	182	8	14	5	37	1868	96	M	14	12a	yes	vertical	vertical	left	no	0	0	67,4	no
Lyon	CSI5*-W presented by GL EVENTS round A	05.11.2017	CSI-W5*	3	1.40 - 1.60	420	112	13	01.06.2005	G	SF	182	8	14	5	37	1868	96	M	15	12b	yes	vertical	vertical with water	left	no	0	0	67,4	no
Lyon	CSI5*-W presented by GL EVENTS round A	05.11.2017	CSI-W5*	3	1.40 - 1.60	420	112	13	01.06.2005	G	SF	182	8	14	5	37	1868	96	M	16	13	no	spread	oxer with water	straight	no	0	0	67,4	no
Lyon	CSI5*-W presented by GL EVENTS round B	05.11.2017	CSI-W5*	3	1.40 - 1.60	280	112	13	01.06.2005	G	SF	182	8	14	5	37	1868	96	M	1	1	no	vertical	vertical	left	no	0	0	37,72	no
Lyon	CSI5*-W presented by GL EVENTS round B	05.11.2017	CSI-W5*	3	1.40 - 1.60	280	112	13	01.06.2005	G	SF	182	8	14	5	37	1868	96	M	2	2	no	vertical	vertical	left	no	0	0	37,72	no
Lyon	CSI5*-W presented by GL EVENTS round B	05.11.2017	CSI-W5*	3	1.40 - 1.60	280	112	13	01.06.2005	G	SF	182	8	14	5	37	1868	96	M	3	3	no	spread	oxer	right	no	0	0	37,72	no
Lyon	CSI5*-W presented by GL EVENTS round B	05.11.2017	CSI-W5*	3	1.40 - 1.60	280	112	13	01.06.2005	G	SF	182	8	14	5	37	1868	96	M	4	4	no	spread	oxer	left	no	0	0	37,72	no
Lyon	CSI5*-W presented by GL EVENTS round B	05.11.2017	CSI-W5*	3	1.40 - 1.60	280	112	13	01.06.2005	G	SF	182	8	14	5	37	1868	96	M	5	5a	yes	vertical	vertical	straight	no	0	0	37,72	no
Lyon	CSI5*-W presented by GL EVENTS round B	05.11.2017	CSI-W5*	3	1.40 - 1.60	280	112	13	01.06.2005	G	SF	182	8	14	5	37	1868	96	M	6	5b	yes	vertical	vertical with water	straight	no	0	0	37,72	no
Lyon	CSI5*-W presented by GL EVENTS round B	05.11.2017	CSI-W5*	3	1.40 - 1.60	280	112	13	01.06.2005	G	SF	182	8	14	5	37	1868	96	M	7	6	no	spread	oxer	left	no	0	0	37,72	no
Lyon	CSI5*-W presented by GL EVENTS round B	05.11.2017	CSI-W5*	3	1.40 - 1.60	280	112	13	01.06.2005	G	SF	182	8	14	5	37	1868	96	M	8	7	no	spread	oxer	right	no	0	0	37,72	no
Lyon	CSI5*-W presented by GL EVENTS round A	05.11.2017	CSI-W5*	3	1.40 - 1.60	420	101	10	07.04.2008	G	HOLST	94	4	17	3	46	1005	51	M	1	1	no	spread	oxer	right	no	0	0	69,87	no
Lyon	CSI5*-W presented by GL EVENTS round A	05.11.2017	CSI-W5*	3	1.40 - 1.60	420	101	10	07.04.2008	G	HOLST	94	4	17	3	46	1005	51	M	2	2	no	vertical	vertical	right	no	0	0	69,87	no
Lyon	CSI5*-W presented by GL EVENTS round A	05.11.2017	CSI-W5*	3	1.40 - 1.60	420	101	10	07.04.2008	G	HOLST	94	4	17	3	46	1005	51	M	3	3	no	spread	oxer	right	no	0	0	69,87	no
Lyon	CSI5*-W presented by GL EVENTS round A	05.11.2017	CSI-W5*	3	1.40 - 1.60	420	101	10	07.04.2008	G	HOLST	94	4	17	3	46	1005	51	M	4	4	no	spread	oxer	left	no	0	0	69,87	no
Lyon	CSI5*-W presented by GL EVENTS round A	05.11.2017	CSI-W5*	3	1.40 - 1.60	420	101	10	07.04.2008	G	HOLST	94	4	17	3	46	1005	51	M	5	5	no	vertical	vertical	straight	no	0	0	69,87	no
Lyon	CSI5*-W presented by GL EVENTS round A	05.11.2017	CSI-W5*	3	1.40 - 1.60	420	101	10	07.04.2008	G	HOLST	94	4	17	3	46	1005	51	M	6	6	no	vertical	vertical	left	yes	4	0	69,87	no
Lyon	CSI5*-W presented by GL EVENTS round A	05.11.2017	CSI-W5*	3	1.40 - 1.60	420	101	10	07.04.2008	G	HOLST	94	4	17	3	46	1005	51	M	7	7a	yes	spread	oxer	straight	no	0	0	69,87	no
Lyon	CSI5*-W presented by GL EVENTS round A	05.11.2017	CSI-W5*	3	1.40 - 1.60	420	101	10	07.04.2008	G	HOLST	94	4	17	3	46	1005	51	M	8	7b	yes	vertical	vertical	straight	yes	4	0	69,87	no
Lyon	CSI5*-W presented by GL EVENTS round A	05.11.2017	CSI-W5*	3	1.40 - 1.60	420	101	10	07.04.2008	G	HOLST	94	4	17	3	46	1005	51	M	9	7c	yes	spread	oxer	straight	no	0	0	69,87	no
Lyon	CSI5*-W presented by GL EVENTS round A	05.11.2017	CSI-W5*	3	1.40 - 1.60	420	101	10	07.04.2008	G	HOLST	94	4	17	3	46	1005	51	M	10	8	no	vertical	wall	right	no	0	0	69,87	no
Lyon	CSI5*-W presented by GL EVENTS round A	05.11.2017	CSI-W5*	3	1.40 - 1.60	420	101	10	07.04.2008	G	HOLST	94	4	17	3	46	1005	51	M	11	9	no	vertical	vertical	right	no	0	0	69,87	no
Lyon	CSI5*-W presented by GL EVENTS round A	05.11.2017	CSI-W5*	3	1.40 - 1.60	420	101	10	07.04.2008	G	HOLST	94	4	17	3	46	1005	51	M	12	10	no	spread	oxer	right	no	0	0	69,87	no
Lyon	CSI5*-W presented by GL EVENTS round A	05.11.2017	CSI-W5*	3	1.40 - 1.60	420	101	10	07.04.2008	G	HOLST	94	4	17	3	46	1005	51	M	13	11	no	spread	oxer	left	no	0	0	69,87	no
Lyon	CSI5*-W presented by GL EVENTS round A	05.11.2017	CSI-W5*	3	1.40 - 1.60	420	101	10	07.04.2008	G	HOLST	94	4	17	3	46	1005	51	M	14	12a	yes	vertical	vertical	left	no	0	0	69,87	no
Lyon	CSI5*-W presented by GL EVENTS round A	05.11.2017	CSI-W5*	3	1.40 - 1.60	420	101	10	07.04.2008	G	HOLST	94	4	17	3	46	1005	51	M	15	12b	yes	vertical	vertical with water	left	no	0	0	69,87	no
Lyon	CSI5*-W presented by GL EVENTS round A	05.11.2017	CSI-W5*	3	1.40 - 1.60	420	101	10	07.04.2008	G	HOLST	94	4	17	3	46	1005	51	M	16	13	no	spread	oxer with water	straight	no	0	0	69,87	no
Lyon	CSI5*-W presented by GL EVENTS round A	05.11.2017	CSI-W5*	3	1.40 - 1.60	420	154	14	29.04.2004	G	SWB	175	3	18	81	31	1327	0	M	1	1	no	spread	oxer	right	no	0	0	68,92	no
Lyon	CSI5*-W presented by GL EVENTS round A	05.11.2017	CSI-W5*	3	1.40 - 1.60	420	154	14	29.04.2004	G	SWB	175	3	18	81	31	1327	0	M	2	2	no	vertical	vertical	right	no	0	0	68,92	no
Lyon	CSI5*-W presented by GL EVENTS round A	05.11.2017	CSI-W5*	3	1.40 - 1.60	420	154	14	29.04.2004	G	SWB	175	3	18	81	31	1327	0	M	3	3	no	spread	oxer	right	no	0	0	68,92	no
Lyon	CSI5*-W presented by GL EVENTS round A	05.11.2017	CSI-W5*	3	1.40 - 1.60	420	154	14	29.04.2004	G	SWB	175	3	18	81	31	1327	0	M	4	4	no	spread	oxer	left	no	0	0	68,92	no
Lyon	CSI5*-W presented by GL EVENTS round A	05.11.2017	CSI-W5*	3	1.40 - 1.60	420	154	14	29.04.2004	G	SWB	175	3	18	81	31	1327	0	M	5	5	no	vertical	vertical	straight	no	0	0	68,92	no
Lyon	CSI5*-W presented by GL EVENTS round A	05.11.2017	CSI-W5*	3	1.40 - 1.60	420	154	14	29.04.2004	G	SWB	175	3	18	81	31	1327	0	M	6	6	no	vertical	vertical	left	no	0	0	68,92	no
Lyon	CSI5*-W presented by GL EVENTS round A	05.11.2017	CSI-W5*	3	1.40 - 1.60	420	154	14	29.04.2004	G	SWB	175	3	18	81	31	1327	0	M	7	7a	yes	spread	oxer	straight	no	0	0	68,92	no
Lyon	CSI5*-W presented by GL EVENTS round A	05.11.2017	CSI-W5*	3	1.40 - 1.60	420	154	14	29.04.2004	G	SWB	175	3	18	81	31	1327	0	M	8	7b	yes	vertical	vertical	straight	no	0	0	68,92	no
Lyon	CSI5*-W presented by GL EVENTS round A	05.11.2017	CSI-W5*	3	1.40 - 1.60	420	154	14	29.04.2004	G	SWB	175	3	18	81	31	1327	0	M	9	7c	yes	spread	oxer	straight	no	0	0	68,92	no
Lyon	CSI5*-W presented by GL EVENTS round A	05.11.2017	CSI-W5*	3	1.40 - 1.60	420	154	14	29.04.2004	G	SWB	175	3	18	81	31	1327	0	M	10	8	no	vertical	wall	right	no	0	0	68,92	no
Lyon	CSI5*-W presented by GL EVENTS round A	05.11.2017	CSI-W5*	3	1.40 - 1.60	420	154	14	29.04.2004	G	SWB	175	3	18	81	31	1327	0	M	11	9	no	vertical	vertical	right	no	0	0	68,92	no
Lyon	CSI5*-W presented by GL EVENTS round A	05.11.2017	CSI-W5*	3	1.40 - 1.60	420	154	14	29.04.2004	G	SWB	175	3	18	81	31	1327	0	M	12	10	no	spread	oxer	right	no	0	0	68,92	no
Lyon	CSI5*-W presented by GL EVENTS round A	05.11.2017	CSI-W5*	3	1.40 - 1.60	420	154	14	29.04.2004	G	SWB	175	3	18	81	31	1327	0	M	13	11	no	spread	oxer	left	no	0	0	68,92	no
Lyon	CSI5*-W presented by GL EVENTS round A	05.11.2017	CSI-W5*	3	1.40 - 1.60	420	154	14	29.04.2004	G	SWB	175	3	18	81	31	1327	0	M	14	12a	yes	vertical	vertical	left	no	0	0	68,92	no
Lyon	CSI5*-W presented by GL EVENTS round A	05.11.2017	CSI-W5*	3	1.40 - 1.60	420	154	14	29.04.2004	G	SWB	175	3	18	81	31	1327	0	M	15	12b	yes	vertical	vertical with water	left	no	0	0	68,92	no
Lyon	CSI5*-W presented by GL EVENTS round A	05.11.2017	CSI-W5*	3	1.40 - 1.60	420	154	14	29.04.2004	G	SWB	175	3	18	81	31	1327	0	M	16	13	no	spread	oxer with water	straight	no	0	0	68,92	no
Lyon	CSI5*-W presented by GL EVENTS round B	05.11.2017	CSI-W5*	3	1.40 - 1.60	280	154	14	29.04.2004	G	SWB	175	3	18	81	31	1327	0	M	1	1	no	vertical	vertical	left	no	0	0	40	no
Lyon	CSI5*-W presented by GL EVENTS round B	05.11.2017	CSI-W5*	3	1.40 - 1.60	280	154	14	29.04.2004	G	SWB	175	3	18	81	31	1327	0	M	2	2	no	vertical	vertical	left	no	0	0	40	no
Lyon	CSI5*-W presented by GL EVENTS round B	05.11.2017	CSI-W5*	3	1.40 - 1.60	280	154	14	29.04.2004	G	SWB	175	3	18	81	31	1327	0	M	3	3	no	spread	oxer	right	no	0	0	40	no
Lyon	CSI5*-W presented by GL EVENTS round B	05.11.2017	CSI-W5*	3	1.40 - 1.60	280	154	14	29.04.2004	G	SWB	175	3	18	81	31	1327	0	M	4	4	no	spread	oxer	left	no	0	0	40	no
Lyon	CSI5*-W presented by GL EVENTS round B	05.11.2017	CSI-W5*	3	1.40 - 1.60	280	154	14	29.04.2004	G	SWB	175	3	18	81	31	1327	0	M	5	5a	yes	vertical	vertical	straight	no	0	0	40	no
Lyon	CSI5*-W presented by GL EVENTS round B	05.11.2017	CSI-W5*	3	1.40 - 1.60	280	154	14	29.04.2004	G	SWB	175	3	18	81	31	1327	0	M	6	5b	yes	vertical	vertical with water	straight	no	0	0	40	no
Lyon	CSI5*-W presented by GL EVENTS round B	05.11.2017	CSI-W5*	3	1.40 - 1.60	280	154	14	29.04.2004	G	SWB	175	3	18	81	31	1327	0	M	7	6	no	spread	oxer	left	no	0	0	40	no
Lyon	CSI5*-W presented by GL EVENTS round B	05.11.2017	CSI-W5*	3	1.40 - 1.60	280	154	14	29.04.2004	G	SWB	175	3	18	81	31	1327	0	M	8	7	no	spread	oxer	right	no	0	0	40	no
Lyon	CSI5*-W presented by GL EVENTS round A	05.11.2017	CSI-W5*	3	1.40 - 1.60	420	183	12	10.06.2006	M	AA	243	8	20	73	38	2030	91	M	1	1	no	spread	oxer	right	yes	4	0	69,41	no
Lyon	CSI5*-W presented by GL EVENTS round A	05.11.2017	CSI-W5*	3	1.40 - 1.60	420	183	12	10.06.2006	M	AA	243	8	20	73	38	2030	91	M	2	2	no	vertical	vertical	right	no	0	0	69,41	no
Lyon	CSI5*-W presented by GL EVENTS round A	05.11.2017	CSI-W5*	3	1.40 - 1.60	420	183	12	10.06.2006	M	AA	243	8	20	73	38	2030	91	M	3	3	no	spread	oxer	right	no	0	0	69,41	no
Lyon	CSI5*-W presented by GL EVENTS round A	05.11.2017	CSI-W5*	3	1.40 - 1.60	420	183	12	10.06.2006	M	AA	243	8	20	73	38	2030	91	M	4	4	no	spread	oxer	left	no	0	0	69,41	no
Lyon	CSI5*-W presented by GL EVENTS round A	05.11.2017	CSI-W5*	3	1.40 - 1.60	420	183	12	10.06.2006	M	AA	243	8	20	73	38	2030	91	M	5	5	no	vertical	vertical	straight	no	0	0	69,41	no
Lyon	CSI5*-W presented by GL EVENTS round A	05.11.2017	CSI-W5*	3	1.40 - 1.60	420	183	12	10.06.2006	M	AA	243	8	20	73	38	2030	91	M	6	6	no	vertical	vertical	left	no	0	0	69,41	no
Lyon	CSI5*-W presented by GL EVENTS round A	05.11.2017	CSI-W5*	3	1.40 - 1.60	420	183	12	10.06.2006	M	AA	243	8	20	73	38	2030	91	M	7	7a	yes	spread	oxer	straight	no	0	0	69,41	no
Lyon	CSI5*-W presented by GL EVENTS round A	05.11.2017	CSI-W5*	3	1.40 - 1.60	420	183	12	10.06.2006	M	AA	243	8	20	73	38	2030	91	M	8	7b	yes	vertical	vertical	straight	yes	4	0	69,41	no
Lyon	CSI5*-W presented by GL EVENTS round A	05.11.2017	CSI-W5*	3	1.40 - 1.60	420	183	12	10.06.2006	M	AA	243	8	20	73	38	2030	91	M	9	7c	yes	spread	oxer	straight	no	0	0	69,41	no
Lyon	CSI5*-W presented by GL EVENTS round A	05.11.2017	CSI-W5*	3	1.40 - 1.60	420	183	12	10.06.2006	M	AA	243	8	20	73	38	2030	91	M	10	8	no	vertical	wall	right	no	0	0	69,41	no
Lyon	CSI5*-W presented by GL EVENTS round A	05.11.2017	CSI-W5*	3	1.40 - 1.60	420	183	12	10.06.2006	M	AA	243	8	20	73	38	2030	91	M	11	9	no	vertical	vertical	right	no	0	0	69,41	no
Lyon	CSI5*-W presented by GL EVENTS round A	05.11.2017	CSI-W5*	3	1.40 - 1.60	420	183	12	10.06.2006	M	AA	243	8	20	73	38	2030	91	M	12	10	no	spread	oxer	right	no	0	0	69,41	no
Lyon	CSI5*-W presented by GL EVENTS round A	05.11.2017	CSI-W5*	3	1.40 - 1.60	420	183	12	10.06.2006	M	AA	243	8	20	73	38	2030	91	M	13	11	no	spread	oxer	left	no	0	0	69,41	no
Lyon	CSI5*-W presented by GL EVENTS round A	05.11.2017	CSI-W5*	3	1.40 - 1.60	420	183	12	10.06.2006	M	AA	243	8	20	73	38	2030	91	M	14	12a	yes	vertical	vertical	left	no	0	0	69,41	no
Lyon	CSI5*-W presented by GL EVENTS round A	05.11.2017	CSI-W5*	3	1.40 - 1.60	420	183	12	10.06.2006	M	AA	243	8	20	73	38	2030	91	M	15	12b	yes	vertical	vertical with water	left	no	0	0	69,41	no
Lyon	CSI5*-W presented by GL EVENTS round A	05.11.2017	CSI-W5*	3	1.40 - 1.60	420	183	12	10.06.2006	M	AA	243	8	20	73	38	2030	91	M	16	13	no	spread	oxer with water	straight	no	0	0	69,41	no
Lyon	CSI5*-W presented by GL EVENTS round A	05.11.2017	CSI-W5*	3	1.40 - 1.60	420	5	13	31.03.2005	G	KWPN	238	4	25	558	26	820	16	F	1	1	no	spread	oxer	right	no	0	0	70,25	no
Lyon	CSI5*-W presented by GL EVENTS round A	05.11.2017	CSI-W5*	3	1.40 - 1.60	420	5	13	31.03.2005	G	KWPN	238	4	25	558	26	820	16	F	2	2	no	vertical	vertical	right	no	0	0	70,25	no
Lyon	CSI5*-W presented by GL EVENTS round A	05.11.2017	CSI-W5*	3	1.40 - 1.60	420	5	13	31.03.2005	G	KWPN	238	4	25	558	26	820	16	F	3	3	no	spread	oxer	right	no	0	0	70,25	no
Lyon	CSI5*-W presented by GL EVENTS round A	05.11.2017	CSI-W5*	3	1.40 - 1.60	420	5	13	31.03.2005	G	KWPN	238	4	25	558	26	820	16	F	4	4	no	spread	oxer	left	no	0	0	70,25	no
Lyon	CSI5*-W presented by GL EVENTS round A	05.11.2017	CSI-W5*	3	1.40 - 1.60	420	5	13	31.03.2005	G	KWPN	238	4	25	558	26	820	16	F	5	5	no	vertical	vertical	straight	no	0	0	70,25	no
Lyon	CSI5*-W presented by GL EVENTS round A	05.11.2017	CSI-W5*	3	1.40 - 1.60	420	5	13	31.03.2005	G	KWPN	238	4	25	558	26	820	16	F	6	6	no	vertical	vertical	left	no	0	0	70,25	no
Lyon	CSI5*-W presented by GL EVENTS round A	05.11.2017	CSI-W5*	3	1.40 - 1.60	420	5	13	31.03.2005	G	KWPN	238	4	25	558	26	820	16	F	7	7a	yes	spread	oxer	straight	no	0	0	70,25	no
Lyon	CSI5*-W presented by GL EVENTS round A	05.11.2017	CSI-W5*	3	1.40 - 1.60	420	5	13	31.03.2005	G	KWPN	238	4	25	558	26	820	16	F	8	7b	yes	vertical	vertical	straight	no	0	0	70,25	no
Lyon	CSI5*-W presented by GL EVENTS round A	05.11.2017	CSI-W5*	3	1.40 - 1.60	420	5	13	31.03.2005	G	KWPN	238	4	25	558	26	820	16	F	9	7c	yes	spread	oxer	straight	yes	4	0	70,25	no
Lyon	CSI5*-W presented by GL EVENTS round A	05.11.2017	CSI-W5*	3	1.40 - 1.60	420	5	13	31.03.2005	G	KWPN	238	4	25	558	26	820	16	F	10	8	no	vertical	wall	right	no	0	0	70,25	no
Lyon	CSI5*-W presented by GL EVENTS round A	05.11.2017	CSI-W5*	3	1.40 - 1.60	420	5	13	31.03.2005	G	KWPN	238	4	25	558	26	820	16	F	11	9	no	vertical	vertical	right	no	0	0	70,25	no
Lyon	CSI5*-W presented by GL EVENTS round A	05.11.2017	CSI-W5*	3	1.40 - 1.60	420	5	13	31.03.2005	G	KWPN	238	4	25	558	26	820	16	F	12	10	no	spread	oxer	right	no	0	0	70,25	no
Lyon	CSI5*-W presented by GL EVENTS round A	05.11.2017	CSI-W5*	3	1.40 - 1.60	420	5	13	31.03.2005	G	KWPN	238	4	25	558	26	820	16	F	13	11	no	spread	oxer	left	no	0	0	70,25	no
Lyon	CSI5*-W presented by GL EVENTS round A	05.11.2017	CSI-W5*	3	1.40 - 1.60	420	5	13	31.03.2005	G	KWPN	238	4	25	558	26	820	16	F	14	12a	yes	vertical	vertical	left	no	0	0	70,25	no
Lyon	CSI5*-W presented by GL EVENTS round A	05.11.2017	CSI-W5*	3	1.40 - 1.60	420	5	13	31.03.2005	G	KWPN	238	4	25	558	26	820	16	F	15	12b	yes	vertical	vertical with water	left	no	0	0	70,25	no
Lyon	CSI5*-W presented by GL EVENTS round A	05.11.2017	CSI-W5*	3	1.40 - 1.60	420	5	13	31.03.2005	G	KWPN	238	4	25	558	26	820	16	F	16	13	no	spread	oxer with water	straight	yes	4	1	70,25	no
Lyon	CSI5*-W presented by GL EVENTS round A	05.11.2017	CSI-W5*	3	1.40 - 1.60	420	127	10	03.07.2008	M	BWP	131	3	26	14	30	1957	97	M	1	1	no	spread	oxer	right	no	0	0	69,99	no
Lyon	CSI5*-W presented by GL EVENTS round A	05.11.2017	CSI-W5*	3	1.40 - 1.60	420	127	10	03.07.2008	M	BWP	131	3	26	14	30	1957	97	M	2	2	no	vertical	vertical	right	no	0	0	69,99	no
Lyon	CSI5*-W presented by GL EVENTS round A	05.11.2017	CSI-W5*	3	1.40 - 1.60	420	127	10	03.07.2008	M	BWP	131	3	26	14	30	1957	97	M	3	3	no	spread	oxer	right	no	0	0	69,99	no
Lyon	CSI5*-W presented by GL EVENTS round A	05.11.2017	CSI-W5*	3	1.40 - 1.60	420	127	10	03.07.2008	M	BWP	131	3	26	14	30	1957	97	M	4	4	no	spread	oxer	left	no	0	0	69,99	no
Lyon	CSI5*-W presented by GL EVENTS round A	05.11.2017	CSI-W5*	3	1.40 - 1.60	420	127	10	03.07.2008	M	BWP	131	3	26	14	30	1957	97	M	5	5	no	vertical	vertical	straight	no	0	0	69,99	no
Lyon	CSI5*-W presented by GL EVENTS round A	05.11.2017	CSI-W5*	3	1.40 - 1.60	420	127	10	03.07.2008	M	BWP	131	3	26	14	30	1957	97	M	6	6	no	vertical	vertical	left	no	0	0	69,99	no
Lyon	CSI5*-W presented by GL EVENTS round A	05.11.2017	CSI-W5*	3	1.40 - 1.60	420	127	10	03.07.2008	M	BWP	131	3	26	14	30	1957	97	M	7	7a	yes	spread	oxer	straight	no	0	0	69,99	no
Lyon	CSI5*-W presented by GL EVENTS round A	05.11.2017	CSI-W5*	3	1.40 - 1.60	420	127	10	03.07.2008	M	BWP	131	3	26	14	30	1957	97	M	8	7b	yes	vertical	vertical	straight	no	0	0	69,99	no
Lyon	CSI5*-W presented by GL EVENTS round A	05.11.2017	CSI-W5*	3	1.40 - 1.60	420	127	10	03.07.2008	M	BWP	131	3	26	14	30	1957	97	M	9	7c	yes	spread	oxer	straight	yes	4	0	69,99	no
Lyon	CSI5*-W presented by GL EVENTS round A	05.11.2017	CSI-W5*	3	1.40 - 1.60	420	127	10	03.07.2008	M	BWP	131	3	26	14	30	1957	97	M	10	8	no	vertical	wall	right	no	0	0	69,99	no
Lyon	CSI5*-W presented by GL EVENTS round A	05.11.2017	CSI-W5*	3	1.40 - 1.60	420	127	10	03.07.2008	M	BWP	131	3	26	14	30	1957	97	M	11	9	no	vertical	vertical	right	no	0	0	69,99	no
Lyon	CSI5*-W presented by GL EVENTS round A	05.11.2017	CSI-W5*	3	1.40 - 1.60	420	127	10	03.07.2008	M	BWP	131	3	26	14	30	1957	97	M	12	10	no	spread	oxer	right	no	0	0	69,99	no
Lyon	CSI5*-W presented by GL EVENTS round A	05.11.2017	CSI-W5*	3	1.40 - 1.60	420	127	10	03.07.2008	M	BWP	131	3	26	14	30	1957	97	M	13	11	no	spread	oxer	left	no	0	0	69,99	no
Lyon	CSI5*-W presented by GL EVENTS round A	05.11.2017	CSI-W5*	3	1.40 - 1.60	420	127	10	03.07.2008	M	BWP	131	3	26	14	30	1957	97	M	14	12a	yes	vertical	vertical	left	no	0	0	69,99	no
Lyon	CSI5*-W presented by GL EVENTS round A	05.11.2017	CSI-W5*	3	1.40 - 1.60	420	127	10	03.07.2008	M	BWP	131	3	26	14	30	1957	97	M	15	12b	yes	vertical	vertical with water	left	no	0	0	69,99	no
Lyon	CSI5*-W presented by GL EVENTS round A	05.11.2017	CSI-W5*	3	1.40 - 1.60	420	127	10	03.07.2008	M	BWP	131	3	26	14	30	1957	97	M	16	13	no	spread	oxer with water	straight	no	0	0	69,99	no
Lyon	CSI5*-W presented by GL EVENTS round A	05.11.2017	CSI-W5*	3	1.40 - 1.60	420	92	13	20.03.2005	G	BWP	140	4	28	4	38	2170	90	M	1	1	no	spread	oxer	right	no	0	0	66,35	no
Lyon	CSI5*-W presented by GL EVENTS round A	05.11.2017	CSI-W5*	3	1.40 - 1.60	420	92	13	20.03.2005	G	BWP	140	4	28	4	38	2170	90	M	2	2	no	vertical	vertical	right	no	0	0	66,35	no
Lyon	CSI5*-W presented by GL EVENTS round A	05.11.2017	CSI-W5*	3	1.40 - 1.60	420	92	13	20.03.2005	G	BWP	140	4	28	4	38	2170	90	M	3	3	no	spread	oxer	right	no	0	0	66,35	no
Lyon	CSI5*-W presented by GL EVENTS round A	05.11.2017	CSI-W5*	3	1.40 - 1.60	420	92	13	20.03.2005	G	BWP	140	4	28	4	38	2170	90	M	4	4	no	spread	oxer	left	no	0	0	66,35	no
Lyon	CSI5*-W presented by GL EVENTS round A	05.11.2017	CSI-W5*	3	1.40 - 1.60	420	92	13	20.03.2005	G	BWP	140	4	28	4	38	2170	90	M	5	5	no	vertical	vertical	straight	no	0	0	66,35	no
Lyon	CSI5*-W presented by GL EVENTS round A	05.11.2017	CSI-W5*	3	1.40 - 1.60	420	92	13	20.03.2005	G	BWP	140	4	28	4	38	2170	90	M	6	6	no	vertical	vertical	left	no	0	0	66,35	no
Lyon	CSI5*-W presented by GL EVENTS round A	05.11.2017	CSI-W5*	3	1.40 - 1.60	420	92	13	20.03.2005	G	BWP	140	4	28	4	38	2170	90	M	7	7a	yes	spread	oxer	straight	no	0	0	66,35	no
Lyon	CSI5*-W presented by GL EVENTS round A	05.11.2017	CSI-W5*	3	1.40 - 1.60	420	92	13	20.03.2005	G	BWP	140	4	28	4	38	2170	90	M	8	7b	yes	vertical	vertical	straight	no	0	0	66,35	no
Lyon	CSI5*-W presented by GL EVENTS round A	05.11.2017	CSI-W5*	3	1.40 - 1.60	420	92	13	20.03.2005	G	BWP	140	4	28	4	38	2170	90	M	9	7c	yes	spread	oxer	straight	no	0	0	66,35	no
Lyon	CSI5*-W presented by GL EVENTS round A	05.11.2017	CSI-W5*	3	1.40 - 1.60	420	92	13	20.03.2005	G	BWP	140	4	28	4	38	2170	90	M	10	8	no	vertical	wall	right	no	0	0	66,35	no
Lyon	CSI5*-W presented by GL EVENTS round A	05.11.2017	CSI-W5*	3	1.40 - 1.60	420	92	13	20.03.2005	G	BWP	140	4	28	4	38	2170	90	M	11	9	no	vertical	vertical	right	no	0	0	66,35	no
Lyon	CSI5*-W presented by GL EVENTS round A	05.11.2017	CSI-W5*	3	1.40 - 1.60	420	92	13	20.03.2005	G	BWP	140	4	28	4	38	2170	90	M	12	10	no	spread	oxer	right	no	0	0	66,35	no
Lyon	CSI5*-W presented by GL EVENTS round A	05.11.2017	CSI-W5*	3	1.40 - 1.60	420	92	13	20.03.2005	G	BWP	140	4	28	4	38	2170	90	M	13	11	no	spread	oxer	left	no	0	0	66,35	no
Lyon	CSI5*-W presented by GL EVENTS round A	05.11.2017	CSI-W5*	3	1.40 - 1.60	420	92	13	20.03.2005	G	BWP	140	4	28	4	38	2170	90	M	14	12a	yes	vertical	vertical	left	no	0	0	66,35	no
Lyon	CSI5*-W presented by GL EVENTS round A	05.11.2017	CSI-W5*	3	1.40 - 1.60	420	92	13	20.03.2005	G	BWP	140	4	28	4	38	2170	90	M	15	12b	yes	vertical	vertical with water	left	no	0	0	66,35	no
Lyon	CSI5*-W presented by GL EVENTS round A	05.11.2017	CSI-W5*	3	1.40 - 1.60	420	92	13	20.03.2005	G	BWP	140	4	28	4	38	2170	90	M	16	13	no	spread	oxer with water	straight	yes	4	0	66,35	no
Lyon	CSI5*-W presented by GL EVENTS round A	05.11.2017	CSI-W5*	3	1.40 - 1.60	420	15	12	26.07.2006	M	SWB	159	0	30	9	36	2556	124	M	1	1	no	spread	oxer	right	no	0	0	68,23	no
Lyon	CSI5*-W presented by GL EVENTS round A	05.11.2017	CSI-W5*	3	1.40 - 1.60	420	15	12	26.07.2006	M	SWB	159	0	30	9	36	2556	124	M	2	2	no	vertical	vertical	right	no	0	0	68,23	no
Lyon	CSI5*-W presented by GL EVENTS round A	05.11.2017	CSI-W5*	3	1.40 - 1.60	420	15	12	26.07.2006	M	SWB	159	0	30	9	36	2556	124	M	3	3	no	spread	oxer	right	no	0	0	68,23	no
Lyon	CSI5*-W presented by GL EVENTS round A	05.11.2017	CSI-W5*	3	1.40 - 1.60	420	15	12	26.07.2006	M	SWB	159	0	30	9	36	2556	124	M	4	4	no	spread	oxer	left	no	0	0	68,23	no
Lyon	CSI5*-W presented by GL EVENTS round A	05.11.2017	CSI-W5*	3	1.40 - 1.60	420	15	12	26.07.2006	M	SWB	159	0	30	9	36	2556	124	M	5	5	no	vertical	vertical	straight	no	0	0	68,23	no
Lyon	CSI5*-W presented by GL EVENTS round A	05.11.2017	CSI-W5*	3	1.40 - 1.60	420	15	12	26.07.2006	M	SWB	159	0	30	9	36	2556	124	M	6	6	no	vertical	vertical	left	no	0	0	68,23	no
Lyon	CSI5*-W presented by GL EVENTS round A	05.11.2017	CSI-W5*	3	1.40 - 1.60	420	15	12	26.07.2006	M	SWB	159	0	30	9	36	2556	124	M	7	7a	yes	spread	oxer	straight	no	0	0	68,23	no
Lyon	CSI5*-W presented by GL EVENTS round A	05.11.2017	CSI-W5*	3	1.40 - 1.60	420	15	12	26.07.2006	M	SWB	159	0	30	9	36	2556	124	M	8	7b	yes	vertical	vertical	straight	no	0	0	68,23	no
Lyon	CSI5*-W presented by GL EVENTS round A	05.11.2017	CSI-W5*	3	1.40 - 1.60	420	15	12	26.07.2006	M	SWB	159	0	30	9	36	2556	124	M	9	7c	yes	spread	oxer	straight	no	0	0	68,23	no
Lyon	CSI5*-W presented by GL EVENTS round A	05.11.2017	CSI-W5*	3	1.40 - 1.60	420	15	12	26.07.2006	M	SWB	159	0	30	9	36	2556	124	M	10	8	no	vertical	wall	right	no	0	0	68,23	no
Lyon	CSI5*-W presented by GL EVENTS round A	05.11.2017	CSI-W5*	3	1.40 - 1.60	420	15	12	26.07.2006	M	SWB	159	0	30	9	36	2556	124	M	11	9	no	vertical	vertical	right	no	0	0	68,23	no
Lyon	CSI5*-W presented by GL EVENTS round A	05.11.2017	CSI-W5*	3	1.40 - 1.60	420	15	12	26.07.2006	M	SWB	159	0	30	9	36	2556	124	M	12	10	no	spread	oxer	right	no	0	0	68,23	no
Lyon	CSI5*-W presented by GL EVENTS round A	05.11.2017	CSI-W5*	3	1.40 - 1.60	420	15	12	26.07.2006	M	SWB	159	0	30	9	36	2556	124	M	13	11	no	spread	oxer	left	no	0	0	68,23	no
Lyon	CSI5*-W presented by GL EVENTS round A	05.11.2017	CSI-W5*	3	1.40 - 1.60	420	15	12	26.07.2006	M	SWB	159	0	30	9	36	2556	124	M	14	12a	yes	vertical	vertical	left	no	0	0	68,23	no
Lyon	CSI5*-W presented by GL EVENTS round A	05.11.2017	CSI-W5*	3	1.40 - 1.60	420	15	12	26.07.2006	M	SWB	159	0	30	9	36	2556	124	M	15	12b	yes	vertical	vertical with water	left	no	0	0	68,23	no
Lyon	CSI5*-W presented by GL EVENTS round A	05.11.2017	CSI-W5*	3	1.40 - 1.60	420	15	12	26.07.2006	M	SWB	159	0	30	9	36	2556	124	M	16	13	no	spread	oxer with water	straight	no	0	0	68,23	no
Lyon	CSI5*-W presented by GL EVENTS round B	05.11.2017	CSI-W5*	3	1.40 - 1.60	280	15	12	26.07.2006	M	SWB	159	0	30	9	36	2556	124	M	1	1	no	vertical	vertical	left	no	0	0	39,05	no
Lyon	CSI5*-W presented by GL EVENTS round B	05.11.2017	CSI-W5*	3	1.40 - 1.60	280	15	12	26.07.2006	M	SWB	159	0	30	9	36	2556	124	M	2	2	no	vertical	vertical	left	no	0	0	39,05	no
Lyon	CSI5*-W presented by GL EVENTS round B	05.11.2017	CSI-W5*	3	1.40 - 1.60	280	15	12	26.07.2006	M	SWB	159	0	30	9	36	2556	124	M	3	3	no	spread	oxer	right	no	0	0	39,05	no
Lyon	CSI5*-W presented by GL EVENTS round B	05.11.2017	CSI-W5*	3	1.40 - 1.60	280	15	12	26.07.2006	M	SWB	159	0	30	9	36	2556	124	M	4	4	no	spread	oxer	left	no	0	0	39,05	no
Lyon	CSI5*-W presented by GL EVENTS round B	05.11.2017	CSI-W5*	3	1.40 - 1.60	280	15	12	26.07.2006	M	SWB	159	0	30	9	36	2556	124	M	5	5a	yes	vertical	vertical	straight	no	0	0	39,05	no
Lyon	CSI5*-W presented by GL EVENTS round B	05.11.2017	CSI-W5*	3	1.40 - 1.60	280	15	12	26.07.2006	M	SWB	159	0	30	9	36	2556	124	M	6	5b	yes	vertical	vertical with water	straight	no	0	0	39,05	no
Lyon	CSI5*-W presented by GL EVENTS round B	05.11.2017	CSI-W5*	3	1.40 - 1.60	280	15	12	26.07.2006	M	SWB	159	0	30	9	36	2556	124	M	7	6	no	spread	oxer	left	no	0	0	39,05	no
Lyon	CSI5*-W presented by GL EVENTS round B	05.11.2017	CSI-W5*	3	1.40 - 1.60	280	15	12	26.07.2006	M	SWB	159	0	30	9	36	2556	124	M	8	7	no	spread	oxer	right	no	0	0	39,05	no
Lyon	CSI5*-W presented by GL EVENTS round A	05.11.2017	CSI-W5*	3	1.40 - 1.60	420	111	11	15.05.2007	S	BWP	140	3	33	23	23	1854	199	M	1	1	no	spread	oxer	right	no	0	0	67,52	no
Lyon	CSI5*-W presented by GL EVENTS round A	05.11.2017	CSI-W5*	3	1.40 - 1.60	420	111	11	15.05.2007	S	BWP	140	3	33	23	23	1854	199	M	2	2	no	vertical	vertical	right	yes	4	0	67,52	no
Lyon	CSI5*-W presented by GL EVENTS round A	05.11.2017	CSI-W5*	3	1.40 - 1.60	420	111	11	15.05.2007	S	BWP	140	3	33	23	23	1854	199	M	3	3	no	spread	oxer	right	no	0	0	67,52	no
Lyon	CSI5*-W presented by GL EVENTS round A	05.11.2017	CSI-W5*	3	1.40 - 1.60	420	111	11	15.05.2007	S	BWP	140	3	33	23	23	1854	199	M	4	4	no	spread	oxer	left	yes	4	0	67,52	no
Lyon	CSI5*-W presented by GL EVENTS round A	05.11.2017	CSI-W5*	3	1.40 - 1.60	420	111	11	15.05.2007	S	BWP	140	3	33	23	23	1854	199	M	5	5	no	vertical	vertical	straight	no	0	0	67,52	no
Lyon	CSI5*-W presented by GL EVENTS round A	05.11.2017	CSI-W5*	3	1.40 - 1.60	420	111	11	15.05.2007	S	BWP	140	3	33	23	23	1854	199	M	6	6	no	vertical	vertical	left	no	0	0	67,52	no
Lyon	CSI5*-W presented by GL EVENTS round A	05.11.2017	CSI-W5*	3	1.40 - 1.60	420	111	11	15.05.2007	S	BWP	140	3	33	23	23	1854	199	M	7	7a	yes	spread	oxer	straight	no	0	0	67,52	no
Lyon	CSI5*-W presented by GL EVENTS round A	05.11.2017	CSI-W5*	3	1.40 - 1.60	420	111	11	15.05.2007	S	BWP	140	3	33	23	23	1854	199	M	8	7b	yes	vertical	vertical	straight	no	0	0	67,52	no
Lyon	CSI5*-W presented by GL EVENTS round A	05.11.2017	CSI-W5*	3	1.40 - 1.60	420	111	11	15.05.2007	S	BWP	140	3	33	23	23	1854	199	M	9	7c	yes	spread	oxer	straight	no	0	0	67,52	no
Lyon	CSI5*-W presented by GL EVENTS round A	05.11.2017	CSI-W5*	3	1.40 - 1.60	420	111	11	15.05.2007	S	BWP	140	3	33	23	23	1854	199	M	10	8	no	vertical	wall	right	no	0	0	67,52	no
Lyon	CSI5*-W presented by GL EVENTS round A	05.11.2017	CSI-W5*	3	1.40 - 1.60	420	111	11	15.05.2007	S	BWP	140	3	33	23	23	1854	199	M	11	9	no	vertical	vertical	right	no	0	0	67,52	no
Lyon	CSI5*-W presented by GL EVENTS round A	05.11.2017	CSI-W5*	3	1.40 - 1.60	420	111	11	15.05.2007	S	BWP	140	3	33	23	23	1854	199	M	12	10	no	spread	oxer	right	no	0	0	67,52	no
Lyon	CSI5*-W presented by GL EVENTS round A	05.11.2017	CSI-W5*	3	1.40 - 1.60	420	111	11	15.05.2007	S	BWP	140	3	33	23	23	1854	199	M	13	11	no	spread	oxer	left	no	0	0	67,52	no

Lyon	CSI5*-W presented by GL EVENTS round A	05.11.2017	CSI-W5*	3	1.40 - 1.60	420	111	11	15.05.2007	S	BWP	140	3	33	23	23	1854	199	M	14	12a	yes	vertical	vertical	left	no	0	0	67,52	no
Lyon	CSI5*-W presented by GL EVENTS round A	05.11.2017	CSI-W5*	3	1.40 - 1.60	420	111	11	15.05.2007	S	BWP	140	3	33	23	23	1854	199	M	15	12b	yes	vertical	vertical with water	left	no	0	0	67,52	no
Lyon	CSI5*-W presented by GL EVENTS round A	05.11.2017	CSI-W5*	3	1.40 - 1.60	420	111	11	15.05.2007	S	BWP	140	3	33	23	23	1854	199	M	16	13	no	spread	oxer with water	straight	no	0	0	67,52	no
Lyon	CSI5*-W presented by GL EVENTS round A	05.11.2017	CSI-W5*	3	1.40 - 1.60	420	166	14	01.03.2004	M	AA	187	5	37	59	42	1314	68	M	1	1	no	spread	oxer	right	no	0	0	66,68	no
Lyon	CSI5*-W presented by GL EVENTS round A	05.11.2017	CSI-W5*	3	1.40 - 1.60	420	166	14	01.03.2004	M	AA	187	5	37	59	42	1314	68	M	2	2	no	vertical	vertical	right	no	0	0	66,68	no
Lyon	CSI5*-W presented by GL EVENTS round A	05.11.2017	CSI-W5*	3	1.40 - 1.60	420	166	14	01.03.2004	M	AA	187	5	37	59	42	1314	68	M	3	3	no	spread	oxer	right	no	0	0	66,68	no
Lyon	CSI5*-W presented by GL EVENTS round A	05.11.2017	CSI-W5*	3	1.40 - 1.60	420	166	14	01.03.2004	M	AA	187	5	37	59	42	1314	68	M	4	4	no	spread	oxer	left	no	0	0	66,68	no
Lyon	CSI5*-W presented by GL EVENTS round A	05.11.2017	CSI-W5*	3	1.40 - 1.60	420	166	14	01.03.2004	M	AA	187	5	37	59	42	1314	68	M	5	5	no	vertical	vertical	straight	no	0	0	66,68	no
Lyon	CSI5*-W presented by GL EVENTS round A	05.11.2017	CSI-W5*	3	1.40 - 1.60	420	166	14	01.03.2004	M	AA	187	5	37	59	42	1314	68	M	6	6	no	vertical	vertical	left	yes	4	0	66,68	no
Lyon	CSI5*-W presented by GL EVENTS round A	05.11.2017	CSI-W5*	3	1.40 - 1.60	420	166	14	01.03.2004	M	AA	187	5	37	59	42	1314	68	M	7	7a	yes	spread	oxer	straight	no	0	0	66,68	no
Lyon	CSI5*-W presented by GL EVENTS round A	05.11.2017	CSI-W5*	3	1.40 - 1.60	420	166	14	01.03.2004	M	AA	187	5	37	59	42	1314	68	M	8	7b	yes	vertical	vertical	straight	no	0	0	66,68	no
Lyon	CSI5*-W presented by GL EVENTS round A	05.11.2017	CSI-W5*	3	1.40 - 1.60	420	166	14	01.03.2004	M	AA	187	5	37	59	42	1314	68	M	9	7c	yes	spread	oxer	straight	no	0	0	66,68	no
Lyon	CSI5*-W presented by GL EVENTS round A	05.11.2017	CSI-W5*	3	1.40 - 1.60	420	166	14	01.03.2004	M	AA	187	5	37	59	42	1314	68	M	10	8	no	vertical	wall	right	yes	4	0	66,68	no
Lyon	CSI5*-W presented by GL EVENTS round A	05.11.2017	CSI-W5*	3	1.40 - 1.60	420	166	14	01.03.2004	M	AA	187	5	37	59	42	1314	68	M	11	9	no	vertical	vertical	right	no	0	0	66,68	no
Lyon	CSI5*-W presented by GL EVENTS round A	05.11.2017	CSI-W5*	3	1.40 - 1.60	420	166	14	01.03.2004	M	AA	187	5	37	59	42	1314	68	M	12	10	no	spread	oxer	right	no	0	0	66,68	no
Lyon	CSI5*-W presented by GL EVENTS round A	05.11.2017	CSI-W5*	3	1.40 - 1.60	420	166	14	01.03.2004	M	AA	187	5	37	59	42	1314	68	M	13	11	no	spread	oxer	left	no	0	0	66,68	no
Lyon	CSI5*-W presented by GL EVENTS round A	05.11.2017	CSI-W5*	3	1.40 - 1.60	420	166	14	01.03.2004	M	AA	187	5	37	59	42	1314	68	M	14	12a	yes	vertical	vertical	left	no	0	0	66,68	no
Lyon	CSI5*-W presented by GL EVENTS round A	05.11.2017	CSI-W5*	3	1.40 - 1.60	420	166	14	01.03.2004	M	AA	187	5	37	59	42	1314	68	M	15	12b	yes	vertical	vertical with water	left	no	0	0	66,68	no
Lyon	CSI5*-W presented by GL EVENTS round A	05.11.2017	CSI-W5*	3	1.40 - 1.60	420	166	14	01.03.2004	M	AA	187	5	37	59	42	1314	68	M	16	13	no	spread	oxer with water	straight	no	0	0	66,68	no
Lyon	CSI5*-W presented by GL EVENTS round A	05.11.2017	CSI-W5*	3	1.40 - 1.60	420	67	10	04.05.2008	M	KWPN	87	1	38	10	29	1642	46	M	1	1	no	spread	oxer	right	no	0	0	66,43	no
Lyon	CSI5*-W presented by GL EVENTS round A	05.11.2017	CSI-W5*	3	1.40 - 1.60	420	67	10	04.05.2008	M	KWPN	87	1	38	10	29	1642	46	M	2	2	no	vertical	vertical	right	no	0	0	66,43	no
Lyon	CSI5*-W presented by GL EVENTS round A	05.11.2017	CSI-W5*	3	1.40 - 1.60	420	67	10	04.05.2008	M	KWPN	87	1	38	10	29	1642	46	M	3	3	no	spread	oxer	right	no	0	0	66,43	no
Lyon	CSI5*-W presented by GL EVENTS round A	05.11.2017	CSI-W5*	3	1.40 - 1.60	420	67	10	04.05.2008	M	KWPN	87	1	38	10	29	1642	46	M	4	4	no	spread	oxer	left	no	0	0	66,43	no
Lyon	CSI5*-W presented by GL EVENTS round A	05.11.2017	CSI-W5*	3	1.40 - 1.60	420	67	10	04.05.2008	M	KWPN	87	1	38	10	29	1642	46	M	5	5	no	vertical	vertical	straight	yes	4	0	66,43	no
Lyon	CSI5*-W presented by GL EVENTS round A	05.11.2017	CSI-W5*	3	1.40 - 1.60	420	67	10	04.05.2008	M	KWPN	87	1	38	10	29	1642	46	M	6	6	no	vertical	vertical	left	no	0	0	66,43	no
Lyon	CSI5*-W presented by GL EVENTS round A	05.11.2017	CSI-W5*	3	1.40 - 1.60	420	67	10	04.05.2008	M	KWPN	87	1	38	10	29	1642	46	M	7	7a	yes	spread	oxer	straight	no	0	0	66,43	no
Lyon	CSI5*-W presented by GL EVENTS round A	05.11.2017	CSI-W5*	3	1.40 - 1.60	420	67	10	04.05.2008	M	KWPN	87	1	38	10	29	1642	46	M	8	7b	yes	vertical	vertical	straight	no	0	0	66,43	no
Lyon	CSI5*-W presented by GL EVENTS round A	05.11.2017	CSI-W5*	3	1.40 - 1.60	420	67	10	04.05.2008	M	KWPN	87	1	38	10	29	1642	46	M	9	7c	yes	spread	oxer	straight	no	0	0	66,43	no
Lyon	CSI5*-W presented by GL EVENTS round A	05.11.2017	CSI-W5*	3	1.40 - 1.60	420	67	10	04.05.2008	M	KWPN	87	1	38	10	29	1642	46	M	10	8	no	vertical	wall	right	no	0	0	66,43	no
Lyon	CSI5*-W presented by GL EVENTS round A	05.11.2017	CSI-W5*	3	1.40 - 1.60	420	67	10	04.05.2008	M	KWPN	87	1	38	10	29	1642	46	M	11	9	no	vertical	vertical	right	no	0	0	66,43	no
Lyon	CSI5*-W presented by GL EVENTS round A	05.11.2017	CSI-W5*	3	1.40 - 1.60	420	67	10	04.05.2008	M	KWPN	87	1	38	10	29	1642	46	M	12	10	no	spread	oxer	right	no	0	0	66,43	no
Lyon	CSI5*-W presented by GL EVENTS round A	05.11.2017	CSI-W5*	3	1.40 - 1.60	420	67	10	04.05.2008	M	KWPN	87	1	38	10	29	1642	46	M	13	11	no	spread	oxer	left	no	0	0	66,43	no
Lyon	CSI5*-W presented by GL EVENTS round A	05.11.2017	CSI-W5*	3	1.40 - 1.60	420	67	10	04.05.2008	M	KWPN	87	1	38	10	29	1642	46	M	14	12a	yes	vertical	vertical	left	no	0	0	66,43	no
Lyon	CSI5*-W presented by GL EVENTS round A	05.11.2017	CSI-W5*	3	1.40 - 1.60	420	67	10	04.05.2008	M	KWPN	87	1	38	10	29	1642	46	M	15	12b	yes	vertical	vertical with water	left	no	0	0	66,43	no
Lyon	CSI5*-W presented by GL EVENTS round A	05.11.2017	CSI-W5*	3	1.40 - 1.60	420	67	10	04.05.2008	M	KWPN	87	1	38	10	29	1642	46	M	16	13	no	spread	oxer with water	straight	yes	4	0	66,43	no
Lyon	CSI5*-W presented by GL EVENTS round A	05.11.2017	CSI-W5*	3	1.40 - 1.60	420	208	10	18.05.2008	G	SF	126	1	43	134	48	1808	64	M	1	1	no	spread	oxer	right	no	0	0	69	no
Lyon	CSI5*-W presented by GL EVENTS round A	05.11.2017	CSI-W5*	3	1.40 - 1.60	420	208	10	18.05.2008	G	SF	126	1	43	134	48	1808	64	M	2	2	no	vertical	vertical	right	no	0	0	69	no
Lyon	CSI5*-W presented by GL EVENTS round A	05.11.2017	CSI-W5*	3	1.40 - 1.60	420	208	10	18.05.2008	G	SF	126	1	43	134	48	1808	64	M	3	3	no	spread	oxer	right	no	0	0	69	no
Lyon	CSI5*-W presented by GL EVENTS round A	05.11.2017	CSI-W5*	3	1.40 - 1.60	420	208	10	18.05.2008	G	SF	126	1	43	134	48	1808	64	M	4	4	no	spread	oxer	left	yes	4	0	69	no
Lyon	CSI5*-W presented by GL EVENTS round A	05.11.2017	CSI-W5*	3	1.40 - 1.60	420	208	10	18.05.2008	G	SF	126	1	43	134	48	1808	64	M	5	5	no	vertical	vertical	straight	no	0	0	69	no
Lyon	CSI5*-W presented by GL EVENTS round A	05.11.2017	CSI-W5*	3	1.40 - 1.60	420	208	10	18.05.2008	G	SF	126	1	43	134	48	1808	64	M	6	6	no	vertical	vertical	left	no	0	0	69	no
Lyon	CSI5*-W presented by GL EVENTS round A	05.11.2017	CSI-W5*	3	1.40 - 1.60	420	208	10	18.05.2008	G	SF	126	1	43	134	48	1808	64	M	7	7a	yes	spread	oxer	straight	no	0	0	69	no
Lyon	CSI5*-W presented by GL EVENTS round A	05.11.2017	CSI-W5*	3	1.40 - 1.60	420	208	10	18.05.2008	G	SF	126	1	43	134	48	1808	64	M	8	7b	yes	vertical	vertical	straight	no	0	0	69	no
Lyon	CSI5*-W presented by GL EVENTS round A	05.11.2017	CSI-W5*	3	1.40 - 1.60	420	208	10	18.05.2008	G	SF	126	1	43	134	48	1808	64	M	9	7c	yes	spread	oxer	straight	no	0	0	69	no
Lyon	CSI5*-W presented by GL EVENTS round A	05.11.2017	CSI-W5*	3	1.40 - 1.60	420	208	10	18.05.2008	G	SF	126	1	43	134	48	1808	64	M	10	8	no	vertical	wall	right	no	0	0	69	no
Lyon	CSI5*-W presented by GL EVENTS round A	05.11.2017	CSI-W5*	3	1.40 - 1.60	420	208	10	18.05.2008	G	SF	126	1	43	134	48	1808	64	M	11	9	no	vertical	vertical	right	no	0	0	69	no
Lyon	CSI5*-W presented by GL EVENTS round A	05.11.2017	CSI-W5*	3	1.40 - 1.60	420	208	10	18.05.2008	G	SF	126	1	43	134	48	1808	64	M	12	10	no	spread	oxer	right	no	0	0	69	no
Lyon	CSI5*-W presented by GL EVENTS round A	05.11.2017	CSI-W5*	3	1.40 - 1.60	420	208	10	18.05.2008	G	SF	126	1	43	134	48	1808	64	M	13	11	no	spread	oxer	left	no	0	0	69	no
Lyon	CSI5*-W presented by GL EVENTS round A	05.11.2017	CSI-W5*	3	1.40 - 1.60	420	208	10	18.05.2008	G	SF	126	1	43	134	48	1808	64	M	14	12a	yes	vertical	vertical	left	no	0	0	69	no
Lyon	CSI5*-W presented by GL EVENTS round A	05.11.2017	CSI-W5*	3	1.40 - 1.60	420	208	10	18.05.2008	G	SF	126	1	43	134	48	1808	64	M	15	12b	yes	vertical	vertical with water	left	no	0	0	69	no
Lyon	CSI5*-W presented by GL EVENTS round A	05.11.2017	CSI-W5*	3	1.40 - 1.60	420	208	10	18.05.2008	G	SF	126	1	43	134	48	1808	64	M	16	13	no	spread	oxer with water	straight	no	0	0	69	no
Lyon	CSI5*-W presented by GL EVENTS round A	05.11.2017	CSI-W5*	3	1.40 - 1.60	420	11	12	13.03.2006	M	KWPN	211	7	48	40	39	1040	33	M	1	1	no	spread	oxer	right	no	0	0	68,96	no
Lyon	CSI5*-W presented by GL EVENTS round A	05.11.2017	CSI-W5*	3	1.40 - 1.60	420	11	12	13.03.2006	M	KWPN	211	7	48	40	39	1040	33	M	2	2	no	vertical	vertical	right	no	0	0	68,96	no
Lyon	CSI5*-W presented by GL EVENTS round A	05.11.2017	CSI-W5*	3	1.40 - 1.60	420	11	12	13.03.2006	M	KWPN	211	7	48	40	39	1040	33	M	3	3	no	spread	oxer	right	no	0	0	68,96	no
Lyon	CSI5*-W presented by GL EVENTS round A	05.11.2017	CSI-W5*	3	1.40 - 1.60	420	11	12	13.03.2006	M	KWPN	211	7	48	40	39	1040	33	M	4	4	no	spread	oxer	left	no	0	0	68,96	no
Lyon	CSI5*-W presented by GL EVENTS round A	05.11.2017	CSI-W5*	3	1.40 - 1.60	420	11	12	13.03.2006	M	KWPN	211	7	48	40	39	1040	33	M	5	5	no	vertical	vertical	straight	no	0	0	68,96	no
Lyon	CSI5*-W presented by GL EVENTS round A	05.11.2017	CSI-W5*	3	1.40 - 1.60	420	11	12	13.03.2006	M	KWPN	211	7	48	40	39	1040	33	M	6	6	no	vertical	vertical	left	no	0	0	68,96	no
Lyon	CSI5*-W presented by GL EVENTS round A	05.11.2017	CSI-W5*	3	1.40 - 1.60	420	11	12	13.03.2006	M	KWPN	211	7	48	40	39	1040	33	M	7	7a	yes	spread	oxer	straight	no	0	0	68,96	no
Lyon	CSI5*-W presented by GL EVENTS round A	05.11.2017	CSI-W5*	3	1.40 - 1.60	420	11	12	13.03.2006	M	KWPN	211	7	48	40	39	1040	33	M	8	7b	yes	vertical	vertical	straight	no	0	0	68,96	no
Lyon	CSI5*-W presented by GL EVENTS round A	05.11.2017	CSI-W5*	3	1.40 - 1.60	420	11	12	13.03.2006	M	KWPN	211	7	48	40	39	1040	33	M	9	7c	yes	spread	oxer	straight	no	0	0	68,96	no
Lyon	CSI5*-W presented by GL EVENTS round A	05.11.2017	CSI-W5*	3	1.40 - 1.60	420	11	12	13.03.2006	M	KWPN	211	7	48	40	39	1040	33	M	10	8	no	vertical	wall	right	no	0	0	68,96	no
Lyon	CSI5*-W presented by GL EVENTS round A	05.11.2017	CSI-W5*	3	1.40 - 1.60	420	11	12	13.03.2006	M	KWPN	211	7	48	40	39	1040	33	M	11	9	no	vertical	vertical	right	no	0	0	68,96	no
Lyon	CSI5*-W presented by GL EVENTS round A	05.11.2017	CSI-W5*	3	1.40 - 1.60	420	11	12	13.03.2006	M	KWPN	211	7	48	40	39	1040	33	M	12	10	no	spread	oxer	right	no	0	0	68,96	no
Lyon	CSI5*-W presented by GL EVENTS round A	05.11.2017	CSI-W5*	3	1.40 - 1.60	420	11	12	13.03.2006	M	KWPN	211	7	48	40	39	1040	33	M	13	11	no	spread	oxer	left	no	0	0	68,96	no
Lyon	CSI5*-W presented by GL EVENTS round A	05.11.2017	CSI-W5*	3	1.40 - 1.60	420	11	12	13.03.2006	M	KWPN	211	7	48	40	39	1040	33	M	14	12a	yes	vertical	vertical	left	yes	4	0	68,96	no
Lyon	CSI5*-W presented by GL EVENTS round A	05.11.2017	CSI-W5*	3	1.40 - 1.60	420	11	12	13.03.2006	M	KWPN	211	7	48	40	39	1040	33	M	15	12b	yes	vertical	vertical with water	left	no	0	0	68,96	no
Lyon	CSI5*-W presented by GL EVENTS round A	05.11.2017	CSI-W5*	3	1.40 - 1.60	420	11	12	13.03.2006	M	KWPN	211	7	48	40	39	1040	33	M	16	13	no	spread	oxer with water	straight	no	0	0	68,96	no
Lyon	CSI5*-W presented by GL EVENTS round A	05.11.2017	CSI-W5*	3	1.40 - 1.60	420	25	12	25.05.2006	G	WESTF	85	0	51	220	30	1567	79	M	1	1	no	spread	oxer	right	no	0	0	71,33	no
Lyon	CSI5*-W presented by GL EVENTS round A	05.11.2017	CSI-W5*	3	1.40 - 1.60	420	25	12	25.05.2006	G	WESTF	85	0	51	220	30	1567	79	M	2	2	no	vertical	vertical	right	no	0	0	71,33	no
Lyon	CSI5*-W presented by GL EVENTS round A	05.11.2017	CSI-W5*	3	1.40 - 1.60	420	25	12	25.05.2006	G	WESTF	85	0	51	220	30	1567	79	M	3	3	no	spread	oxer	right	no	0	0	71,33	no
Lyon	CSI5*-W presented by GL EVENTS round A	05.11.2017	CSI-W5*	3	1.40 - 1.60	420	25	12	25.05.2006	G	WESTF	85	0	51	220	30	1567	79	M	4	4	no	spread	oxer	left	no	0	0	71,33	no
Lyon	CSI5*-W presented by GL EVENTS round A	05.11.2017	CSI-W5*	3	1.40 - 1.60	420	25	12	25.05.2006	G	WESTF	85	0	51	220	30	1567	79	M	5	5	no	vertical	vertical	straight	no	0	0	71,33	no
Lyon	CSI5*-W presented by GL EVENTS round A	05.11.2017	CSI-W5*	3	1.40 - 1.60	420	25	12	25.05.2006	G	WESTF	85	0	51	220	30	1567	79	M	6	6	no	vertical	vertical	left	no	0	0	71,33	no
Lyon	CSI5*-W presented by GL EVENTS round A	05.11.2017	CSI-W5*	3	1.40 - 1.60	420	25	12	25.05.2006	G	WESTF	85	0	51	220	30	1567	79	M	7	7a	yes	spread	oxer	straight	no	0	0	71,33	no
Lyon	CSI5*-W presented by GL EVENTS round A	05.11.2017	CSI-W5*	3	1.40 - 1.60	420	25	12	25.05.2006	G	WESTF	85	0	51	220	30	1567	79	M	8	7b	yes	vertical	vertical	straight	no	0	0	71,33	no
Lyon	CSI5*-W presented by GL EVENTS round A	05.11.2017	CSI-W5*	3	1.40 - 1.60	420	25	12	25.05.2006	G	WESTF	85	0	51	220	30	1567	79	M	9	7c	yes	spread	oxer	straight	no	0	0	71,33	no
Lyon	CSI5*-W presented by GL EVENTS round A	05.11.2017	CSI-W5*	3	1.40 - 1.60	420	25	12	25.05.2006	G	WESTF	85	0	51	220	30	1567	79	M	10	8	no	vertical	wall	right	no	0	0	71,33	no
Lyon	CSI5*-W presented by GL EVENTS round A	05.11.2017	CSI-W5*	3	1.40 - 1.60	420	25	12	25.05.2006	G	WESTF	85	0	51	220	30	1567	79	M	11	9	no	vertical	vertical	right	no	0	0	71,33	no
Lyon	CSI5*-W presented by GL EVENTS round A	05.11.2017	CSI-W5*	3	1.40 - 1.60	420	25	12	25.05.2006	G	WESTF	85	0	51	220	30	1567	79	M	12	10	no	spread	oxer	right	no	0	0	71,33	no
Lyon	CSI5*-W presented by GL EVENTS round A	05.11.2017	CSI-W5*	3	1.40 - 1.60	420	25	12	25.05.2006	G	WESTF	85	0	51	220	30	1567	79	M	13	11	no	spread	oxer	left	no	0	0	71,33	no
Lyon	CSI5*-W presented by GL EVENTS round A	05.11.2017	CSI-W5*	3	1.40 - 1.60	420	25	12	25.05.2006	G	WESTF	85	0	51	220	30	1567	79	M	14	12a	yes	vertical	vertical	left	yes	4	0	71,33	no
Lyon	CSI5*-W presented by GL EVENTS round A	05.11.2017	CSI-W5*	3	1.40 - 1.60	420	25	12	25.05.2006	G	WESTF	85	0	51	220	30	1567	79	M	15	12b	yes	vertical	vertical with water	left	no	0	0	71,33	no
Lyon	CSI5*-W presented by GL EVENTS round A	05.11.2017	CSI-W5*	3	1.40 - 1.60	420	25	12	25.05.2006	G	WESTF	85	0	51	220	30	1567	79	M	16	13	no	spread	oxer with water	straight	no	0	1	71,33	no
Lyon	CSI5*-W presented by GL EVENTS round A	05.11.2017	CSI-W5*	3	1.40 - 1.60	420	10	11	07.05.2007	M	Bezplemenné	103	1	53	32	33	1848	55	M	1	1	no	spread	oxer	right	no	0	0	68,52	no
Lyon	CSI5*-W presented by GL EVENTS round A	05.11.2017	CSI-W5*	3	1.40 - 1.60	420	10	11	07.05.2007	M	Bezplemenné	103	1	53	32	33	1848	55	M	2	2	no	vertical	vertical	right	no	0	0	68,52	no
Lyon	CSI5*-W presented by GL EVENTS round A	05.11.2017	CSI-W5*	3	1.40 - 1.60	420	10	11	07.05.2007	M	Bezplemenné	103	1	53	32	33	1848	55	M	3	3	no	spread	oxer	right	no	0	0	68,52	no
Lyon	CSI5*-W presented by GL EVENTS round A	05.11.2017	CSI-W5*	3	1.40 - 1.60	420	10	11	07.05.2007	M	Bezplemenné	103	1	53	32	33	1848	55	M	4	4	no	spread	oxer	left	no	0	0	68,52	no
Lyon	CSI5*-W presented by GL EVENTS round A	05.11.2017	CSI-W5*	3	1.40 - 1.60	420	10	11	07.05.2007	M	Bezplemenné	103	1	53	32	33	1848	55	M	5	5	no	vertical	vertical	straight	no	0	0	68,52	no
Lyon	CSI5*-W presented by GL EVENTS round A	05.11.2017	CSI-W5*	3	1.40 - 1.60	420	10	11	07.05.2007	M	Bezplemenné	103	1	53	32	33	1848	55	M	6	6	no	vertical	vertical	left	no	0	0	68,52	no
Lyon	CSI5*-W presented by GL EVENTS round A	05.11.2017	CSI-W5*	3	1.40 - 1.60	420	10	11	07.05.2007	M	Bezplemenné	103	1	53	32	33	1848	55	M	7	7a	yes	spread	oxer	straight	no	0	0	68,52	no
Lyon	CSI5*-W presented by GL EVENTS round A	05.11.2017	CSI-W5*	3	1.40 - 1.60	420	10	11	07.05.2007	M	Bezplemenné	103	1	53	32	33	1848	55	M	8	7b	yes	vertical	vertical	straight	no	0	0	68,52	no
Lyon	CSI5*-W presented by GL EVENTS round A	05.11.2017	CSI-W5*	3	1.40 - 1.60	420	10	11	07.05.2007	M	Bezplemenné	103	1	53	32	33	1848	55	M	9	7c	yes	spread	oxer	straight	no	0	0	68,52	no
Lyon	CSI5*-W presented by GL EVENTS round A	05.11.2017	CSI-W5*	3	1.40 - 1.60	420	10	11	07.05.2007	M	Bezplemenné	103	1	53	32	33	1848	55	M	10	8	no	vertical	wall	right	no	0	0	68,52	no
Lyon	CSI5*-W presented by GL EVENTS round A	05.11.2017	CSI-W5*	3	1.40 - 1.60	420	10	11	07.05.2007	M	Bezplemenné	103	1	53	32	33	1848	55	M	11	9	no	vertical	vertical	right	no	0	0	68,52	no
Lyon	CSI5*-W presented by GL EVENTS round A	05.11.2017	CSI-W5*	3	1.40 - 1.60	420	10	11	07.05.2007	M	Bezplemenné	103	1	53	32	33	1848	55	M	12	10	no	spread	oxer	right	no	0	0	68,52	no
Lyon	CSI5*-W presented by GL EVENTS round A	05.11.2017	CSI-W5*	3	1.40 - 1.60	420	10	11	07.05.2007	M	Bezplemenné	103	1	53	32	33	1848	55	M	13	11	no	spread	oxer	left	no	0	0	68,52	no
Lyon	CSI5*-W presented by GL EVENTS round A	05.11.2017	CSI-W5*	3	1.40 - 1.60	420	10	11	07.05.2007	M	Bezplemenné	103	1	53	32	33	1848	55	M	14	12a	yes	vertical	vertical	left	no	0	0	68,52	no
Lyon	CSI5*-W presented by GL EVENTS round A	05.11.2017	CSI-W5*	3	1.40 - 1.60	420	10	11	07.05.2007	M	Bezplemenné	103	1	53	32	33	1848	55	M	15	12b	yes	vertical	vertical with water	left	yes	4	0	68,52	no
Lyon	CSI5*-W presented by GL EVENTS round A	05.11.2017	CSI-W5*	3	1.40 - 1.60	420	10	11	07.05.2007	M	Bezplemenné	103	1	53	32	33	1848	55	M	16	13	no	spread	oxer with water	straight	no	0	0	68,52	no
Lyon	CSI5*-W presented by GL EVENTS round A	05.11.2017	CSI-W5*	3	1.40 - 1.60	420	152	12	20.05.2006	M	WESTF	94	7	55	6	37	1750	61	M	1	1	no	spread	oxer	right	no	0	0	68,37	no
Lyon	CSI5*-W presented by GL EVENTS round A	05.11.2017	CSI-W5*	3	1.40 - 1.60	420	152	12	20.05.2006	M	WESTF	94	7	55	6	37	1750	61	M	2	2	no	vertical	vertical	right	no	0	0	68,37	no
Lyon	CSI5*-W presented by GL EVENTS round A	05.11.2017	CSI-W5*	3	1.40 - 1.60	420	152	12	20.05.2006	M	WESTF	94	7	55	6	37	1750	61	M	3	3	no	spread	oxer	right	no	0	0	68,37	no
Lyon	CSI5*-W presented by GL EVENTS round A	05.11.2017	CSI-W5*	3	1.40 - 1.60	420	152	12	20.05.2006	M	WESTF	94	7	55	6	37	1750	61	M	4	4	no	spread	oxer	left	no	0	0	68,37	no
Lyon	CSI5*-W presented by GL EVENTS round A	05.11.2017	CSI-W5*	3	1.40 - 1.60	420	152	12	20.05.2006	M	WESTF	94	7	55	6	37	1750	61	M	5	5	no	vertical	vertical	straight	no	0	0	68,37	no
Lyon	CSI5*-W presented by GL EVENTS round A	05.11.2017	CSI-W5*	3	1.40 - 1.60	420	152	12	20.05.2006	M	WESTF	94	7	55	6	37	1750	61	M	6	6	no	vertical	vertical	left	no	0	0	68,37	no
Lyon	CSI5*-W presented by GL EVENTS round A	05.11.2017	CSI-W5*	3	1.40 - 1.60	420	152	12	20.05.2006	M	WESTF	94	7	55	6	37	1750	61	M	7	7a	yes	spread	oxer	straight	no	0	0	68,37	no
Lyon	CSI5*-W presented by GL EVENTS round A	05.11.2017	CSI-W5*	3	1.40 - 1.60	420	152	12	20.05.2006	M	WESTF	94	7	55	6	37	1750	61	M	8	7b	yes	vertical	vertical	straight	no	0	0	68,37	no
Lyon	CSI5*-W presented by GL EVENTS round A	05.11.2017	CSI-W5*	3	1.40 - 1.60	420	152	12	20.05.2006	M	WESTF	94	7	55	6	37	1750	61	M	9	7c	yes	spread	oxer	straight	no	0	0	68,37	no
Lyon	CSI5*-W presented by GL EVENTS round A	05.11.2017	CSI-W5*	3	1.40 - 1.60	420	152	12	20.05.2006	M	WESTF	94	7	55	6	37	1750	61	M	10	8	no	vertical	wall	right	no	0	0	68,37	no
Lyon	CSI5*-W presented by GL EVENTS round A	05.11.2017	CSI-W5*	3	1.40 - 1.60	420	152	12	20.05.2006	M	WESTF	94	7	55	6	37	1750	61	M	11	9	no	vertical	vertical	right	no	0	0	68,37	no
Lyon	CSI5*-W presented by GL EVENTS round A	05.11.2017	CSI-W5*	3	1.40 - 1.60	420	152	12	20.05.2006	M	WESTF	94	7	55	6	37	1750	61	M	12	10	no	spread	oxer	right	no	0	0	68,37	no
Lyon	CSI5*-W presented by GL EVENTS round A	05.11.2017	CSI-W5*	3	1.40 - 1.60	420	152	12	20.05.2006	M	WESTF	94	7	55	6	37	1750	61	M	13	11	no	spread	oxer	left	no	0	0	68,37	no
Lyon	CSI5*-W presented by GL EVENTS round A	05.11.2017	CSI-W5*	3	1.40 - 1.60	420	152	12	20.05.2006	M	WESTF	94	7	55	6	37	1750	61	M	14	12a	yes	vertical	vertical	left	no	0	0	68,37	no
Lyon	CSI5*-W presented by GL EVENTS round A	05.11.2017	CSI-W5*	3	1.40 - 1.60	420	152	12	20.05.2006	M	WESTF	94	7	55	6	37	1750	61	M	15	12b	yes	vertical	vertical with water	left	no	0	0	68,37	no
Lyon	CSI5*-W presented by GL EVENTS round A	05.11.2017	CSI-W5*	3	1.40 - 1.60	420	152	12	20.05.2006	M	WESTF	94	7	55	6	37	1750	61	M	16	13	no	spread	oxer with water	straight	no	0	0	68,37	no
Lyon	CSI5*-W presented by GL EVENTS round B	05.11.2017	CSI-W5*	3	1.40 - 1.60	280	152	12	20.05.2006	M	WESTF	94	7	55	6	37	1750	61	M	1	1	no	vertical	vertical	left	no	0	0	38,35	no
Lyon	CSI5*-W presented by GL EVENTS round B	05.11.2017	CSI-W5*	3	1.40 - 1.60	280	152	12	20.05.2006	M	WESTF	94	7	55	6	37	1750	61	M	2	2	no	vertical	vertical	left	no	0	0	38,35	no
Lyon	CSI5*-W presented by GL EVENTS round B	05.11.2017	CSI-W5*	3	1.40 - 1.60	280	152	12	20.05.2006	M	WESTF	94	7	55	6	37	1750	61	M	3	3	no	spread	oxer	right	no	0	0	38,35	no
Lyon	CSI5*-W presented by GL EVENTS round B	05.11.2017	CSI-W5*	3	1.40 - 1.60	280	152	12	20.05.2006	M	WESTF	94	7	55	6	37	1750	61	M	4	4	no	spread	oxer	left	no	0	0	38,35	no
Lyon	CSI5*-W presented by GL EVENTS round B	05.11.2017	CSI-W5*	3	1.40 - 1.60	280	152	12	20.05.2006	M	WESTF	94	7	55	6	37	1750	61	M	5	5a	yes	vertical	vertical	straight	no	0	0	38,35	no
Lyon	CSI5*-W presented by GL EVENTS round B	05.11.2017	CSI-W5*	3	1.40 - 1.60	280	152	12	20.05.2006	M	WESTF	94	7	55	6	37	1750	61	M	6	5b	yes	vertical	vertical with water	straight	no	0	0	38,35	no
Lyon	CSI5*-W presented by GL EVENTS round B	05.11.2017	CSI-W5*	3	1.40 - 1.60	280	152	12	20.05.2006	M	WESTF	94	7	55	6	37	1750	61	M	7	6	no	spread	oxer	left	no	0	0	38,35	no
Lyon	CSI5*-W presented by GL EVENTS round B	05.11.2017	CSI-W5*	3	1.40 - 1.60	280	152	12	20.05.2006	M	WESTF	94	7	55	6	37	1750	61	M	8	7	no	spread	oxer	right	no	0	0	38,35	no
Lyon	CSI5*-W presented by GL EVENTS round A	05.11.2017	CSI-W5*	3	1.40 - 1.60	420	31	12	15.04.2006	S	OS	292	11	59	57	32	2641	229	M	1	1	no	spread	oxer	right	no	0	0	68,63	no
Lyon	CSI5*-W presented by GL EVENTS round A	05.11.2017	CSI-W5*	3	1.40 - 1.60	420	31	12	15.04.2006	S	OS	292	11	59	57	32	2641	229	M	2	2	no	vertical	vertical	right	no	0	0	68,63	no
Lyon	CSI5*-W presented by GL EVENTS round A	05.11.2017	CSI-W5*	3	1.40 - 1.60	420	31	12	15.04.2006	S	OS	292	11	59	57	32	2641	229	M	3	3	no	spread	oxer	right	no	0	0	68,63	no
Lyon	CSI5*-W presented by GL EVENTS round A	05.11.2017	CSI-W5*	3	1.40 - 1.60	420	31	12	15.04.2006	S	OS	292	11	59	57	32	2641	229	M	4	4	no	spread	oxer	left	no	0	0	68,63	no
Lyon	CSI5*-W presented by GL EVENTS round A	05.11.2017	CSI-W5*	3	1.40 - 1.60	420	31	12	15.04.2006	S	OS	292	11	59	57	32	2641	229	M	5	5	no	vertical	vertical	straight	no	0	0	68,63	no
Lyon	CSI5*-W presented by GL EVENTS round A	05.11.2017	CSI-W5*	3	1.40 - 1.60	420	31	12	15.04.2006	S	OS	292	11	59	57	32	2641	229	M	6	6	no	vertical	vertical	left	no	0	0	68,63	no
Lyon	CSI5*-W presented by GL EVENTS round A	05.11.2017	CSI-W5*	3	1.40 - 1.60	420	31	12	15.04.2006	S	OS	292	11	59	57	32	2641	229	M	7	7a	yes	spread	oxer	straight	no	0	0	68,63	no
Lyon	CSI5*-W presented by GL EVENTS round A	05.11.2017	CSI-W5*	3	1.40 - 1.60	420	31	12	15.04.2006	S	OS	292	11	59	57	32	2641	229	M	8	7b	yes	vertical	vertical	straight	no	0	0	68,63	no
Lyon	CSI5*-W presented by GL EVENTS round A	05.11.2017	CSI-W5*	3	1.40 - 1.60	420	31	12	15.04.2006	S	OS	292	11	59	57	32	2641	229	M	9	7c	yes	spread	oxer	straight	no	0	0	68,63	no
Lyon	CSI5*-W presented by GL EVENTS round A	05.11.2017	CSI-W5*	3	1.40 - 1.60	420	31	12	15.04.2006	S	OS	292	11	59	57	32	2641	229	M	10	8	no	vertical	wall	right	no	0	0	68,63	no
Lyon	CSI5*-W presented by GL EVENTS round A	05.11.2017	CSI-W5*	3	1.40 - 1.60	420	31	12	15.04.2006	S	OS	292	11	59	57	32	2641	229	M	11	9	no	vertical	vertical	right	no	0	0	68,63	no
Lyon	CSI5*-W presented by GL EVENTS round A	05.11.2017	CSI-W5*	3	1.40 - 1.60	420	31	12	15.04.2006	S	OS	292	11	59	57	32	2641	229	M	12	10	no	spread	oxer	right	no	0	0	68,63	no
Lyon	CSI5*-W presented by GL EVENTS round A	05.11.2017	CSI-W5*	3	1.40 - 1.60	420	31	12	15.04.2006	S	OS	292	11	59	57	32	2641	229	M	13	11	no	spread	oxer	left	no	0	0	68,63	no
Lyon	CSI5*-W presented by GL EVENTS round A	05.11.2017	CSI-W5*	3	1.40 - 1.60	420	31	12	15.04.2006	S	OS	292	11	59	57	32	2641	229	M	14	12a	yes	vertical	vertical	left	no	0	0	68,63	no
Lyon	CSI5*-W presented by GL EVENTS round A	05.11.2017	CSI-W5*	3	1.40 - 1.60	420	31	12	15.04.2006	S	OS	292	11	59	57	32	2641	229	M	15	12b	yes	vertical	vertical with water	left	no	0	0	68,63	no
Lyon	CSI5*-W presented by GL EVENTS round A	05.11.2017	CSI-W5*	3	1.40 - 1.60	420	31	12	15.04.2006	S	OS	292	11	59	57	32	2641	229	M	16	13	no	spread	oxer with water	straight	yes	4	0	68,63	no
Lyon	CSI5*-W presented by GL EVENTS round A	05.11.2017	CSI-W5*	3	1.40 - 1.60	420	80	14	24.07.2004	S	BWP	179	5	64	20	44	2014	118	M	1	1	no	spread	oxer	right	no	0	0	67,46	no
Lyon	CSI5*-W presented by GL EVENTS round A	05.11.2017	CSI-W5*	3	1.40 - 1.60	420	80	14	24.07.2004	S	BWP	179	5	64	20	44	2014	118	M	2	2	no	vertical	vertical	right	no	0	0	67,46	no
Lyon	CSI5*-W presented by GL EVENTS round A	05.11.2017	CSI-W5*	3	1.40 - 1.60	420	80	14	24.07.2004	S	BWP	179	5	64	20	44	2014	118	M	3	3	no	spread	oxer	right	no	0	0	67,46	no
Lyon	CSI5*-W presented by GL EVENTS round A	05.11.2017	CSI-W5*	3	1.40 - 1.60	420	80	14	24.07.2004	S	BWP	179	5	64	20	44	2014	118	M	4	4	no	spread	oxer	left	no	0	0	67,46	no
Lyon	CSI5*-W presented by GL EVENTS round A	05.11.2017	CSI-W5*	3	1.40 - 1.60	420	80	14	24.07.2004	S	BWP	179	5	64	20	44	2014	118	M	5	5	no	vertical	vertical	straight	no	0	0	67,46	no
Lyon	CSI5*-W presented by GL EVENTS round A	05.11.2017	CSI-W5*	3	1.40 - 1.60	420	80	14	24.07.2004	S	BWP	179	5	64	20	44	2014	118	M	6	6	no	vertical	vertical	left	yes	4	0	67,46	no
Lyon	CSI5*-W presented by GL EVENTS round A	05.11.2017	CSI-W5*	3	1.40 - 1.60	420	80	14	24.07.2004	S	BWP	179	5	64	20	44	2014	118	M	7	7a	yes	spread	oxer	straight	no	0	0	67,46	no
Lyon	CSI5*-W presented by GL EVENTS round A	05.11.2017	CSI-W5*	3	1.40 - 1.60	420	80	14	24.07.2004	S	BWP	179	5	64	20	44	2014	118	M	8	7b	yes	vertical	vertical	straight	no	0	0	67,46	no
Lyon	CSI5*-W presented by GL EVENTS round A	05.11.2017	CSI-W5*	3	1.40 - 1.60	420	80	14	24.07.2004	S	BWP	179	5	64	20	44	2014	118	M	9	7c	yes	spread	oxer	straight	no	0	0	67,46	no
Lyon	CSI5*-W presented by GL EVENTS round A	05.11.2017	CSI-W5*	3	1.40 - 1.60	420	80	14	24.07.2004	S	BWP	179	5	64	20	44	2014	118	M	10	8	no	vertical	wall	right	no	0	0	67,46	no
Lyon	CSI5*-W presented by GL EVENTS round A	05.11.2017	CSI-W5*	3	1.40 - 1.60	420	80	14	24.07.2004	S	BWP	179	5	64	20	44	2014	118	M	11	9	no	vertical	vertical	right	no	0	0	67,46	no
Lyon	CSI5*-W presented by GL EVENTS round A	05.11.2017	CSI-W5*	3	1.40 - 1.60	420	80	14	24.07.2004	S	BWP	179	5	64	20	44	2014	118	M	12	10	no	spread	oxer	right	no	0	0	67,46	no
Lyon	CSI5*-W presented by GL EVENTS round A	05.11.2017	CSI-W5*	3	1.40 - 1.60	420	80	14	24.07.2004	S	BWP	179	5	64	20	44	2014	118	M	13	11	no	spread	oxer	left	no	0	0	67,46	no
Lyon	CSI5*-W presented by GL EVENTS round A	05.11.2017	CSI-W5*	3	1.40 - 1.60	420	80	14	24.07.2004	S	BWP	179	5	64	20	44	2014	118	M	14	12a	yes	vertical	vertical	left	no	0	0	67,46	no
Lyon	CSI5*-W presented by GL EVENTS round A	05.11.2017	CSI-W5*	3	1.40 - 1.60	420	80	14	24.07.2004	S	BWP	179	5	64	20	44	2014	118	M	15	12b	yes	vertical	vertical with water	left	no	0	0	67,46	no
Lyon	CSI5*-W presented by GL EVENTS round A	05.11.2017	CSI-W5*	3	1.40 - 1.60	420	80	14	24.07.2004	S	BWP	179	5	64	20	44	2014	118	M	16	13	no	spread	oxer with water	straight	no	0	0	67,46	no
Lyon	CSI5*-W presented by GL EVENTS round A	05.11.2017	CSI-W5*	3	1.40 - 1.60	420	71	10	25.05.2008	M	KWPN	131	2	65	70	43	1682	26	M	1	1	no	spread	oxer	right	no	0	0	69,9	no
Lyon	CSI5*-W presented by GL EVENTS round A	05.11.2017	CSI-W5*	3	1.40 - 1.60	420	71	10	25.05.2008	M	KWPN	131	2	65	70	43	1682	26	M	2	2	no	vertical	vertical	right	no	0	0	69,9	no
Lyon	CSI5*-W presented by GL EVENTS round A	05.11.2017	CSI-W5*	3	1.40 - 1.60	420	71	10	25.05.2008	M	KWPN	131	2	65	70	43	1682	26	M	3	3	no	spread	oxer	right	no	0	0	69,9	no
Lyon	CSI5*-W presented by GL EVENTS round A	05.11.2017	CSI-W5*	3	1.40 - 1.60	420	71	10	25.05.2008	M	KWPN	131	2	65	70	43	1682	26	M	4	4	no	spread	oxer	left	no	0	0	69,9	no
Lyon	CSI5*-W presented by GL EVENTS round A	05.11.2017	CSI-W5*	3	1.40 - 1.60	420	71	10	25.05.2008	M	KWPN	131	2	65	70	43	1682	26	M	5	5	no	vertical	vertical	straight	no	0	0	69,9	no
Lyon	CSI5*-W presented by GL EVENTS round A	05.11.2017	CSI-W5*	3	1.40 - 1.60	420	71	10	25.05.2008	M	KWPN	131	2	65	70	43	1682	26	M	6	6	no	vertical	vertical	left	no	0	0	69,9	no
Lyon	CSI5*-W presented by GL EVENTS round A	05.11.2017	CSI-W5*	3	1.40 - 1.60	420	71	10	25.05.2008	M	KWPN	131	2	65	70	43	1682	26	M	7	7a	yes	spread	oxer	straight	no	0	0	69,9	no
Lyon	CSI5*-W presented by GL EVENTS round A	05.11.2017	CSI-W5*	3	1.40 - 1.60	420	71	10	25.05.2008	M	KWPN	131	2	65	70	43	1682	26	M	8	7b	yes	vertical	vertical	straight	no	0	0	69,9	no
Lyon	CSI5*-W presented by GL EVENTS round A	05.11.2017	CSI-W5*	3	1.40 - 1.60	420	71	10	25.05.2008	M	KWPN	131	2	65	70	43	1682	26	M	9	7c	yes	spread	oxer	straight	no	0	0	69,9	no
Lyon	CSI5*-W presented by GL EVENTS round A	05.11.2017	CSI-W5*	3	1.40 - 1.60	420	71	10	25.05.2008	M	KWPN	131	2	65	70	43	1682	26	M	10	8	no	vertical	wall	right	no	0	0	69,9	no
Lyon	CSI5*-W presented by GL EVENTS round A	05.11.2017	CSI-W5*	3	1.40 - 1.60	420	71	10	25.05.2008	M	KWPN	131	2	65	70	43	1682	26	M	11	9	no	vertical	vertical	right	no	0	0	69,9	no
Lyon	CSI5*-W presented by GL EVENTS round A	05.11.2017	CSI-W5*	3	1.40 - 1.60	420	71	10	25.05.2008	M	KWPN	131	2	65	70	43	1682	26	M	12	10	no	spread	oxer	right	no	0	0	69,9	no
Lyon	CSI5*-W presented by GL EVENTS round A	05.11.2017	CSI-W5*	3	1.40 - 1.60	420	71	10	25.05.2008	M	KWPN	131	2	65	70	43	1682	26	M	13	11	no	spread	oxer	left	no	0	0	69,9	no
Lyon	CSI5*-W presented by GL EVENTS round A	05.11.2017	CSI-W5*	3	1.40 - 1.60	420	71	10	25.05.2008	M	KWPN	131	2	65	70	43	1682	26	M	14	12a	yes	vertical	vertical	left	no	0	0	69,9	no
Lyon	CSI5*-W presented by GL EVENTS round A	05.11.2017	CSI-W5*	3	1.40 - 1.60	420	71	10	25.05.2008	M	KWPN	131	2	65	70	43	1682	26	M	15	12b	yes	vertical	vertical with water	left	no	0	0	69,9	no
Lyon	CSI5*-W presented by GL EVENTS round A	05.11.2017	CSI-W5*	3	1.40 - 1.60	420	71	10	25.05.2008	M	KWPN	131	2	65	70	43	1682	26	M	16	13	no	spread	oxer with water	straight	no	0	0	69,9	no
Lyon	CSI5*-W presented by GL EVENTS round B	05.11.2017	CSI-W5*	3	1.40 - 1.60	280	71	10	25.05.2008	M	KWPN	131	2	65	70	43	1682	26	M	1	1	no	vertical	vertical	left	no	0	0	45,2	no
Lyon	CSI5*-W presented by GL EVENTS round B	05.11.2017	CSI-W5*	3	1.40 - 1.60	280	71	10	25.05.2008	M	KWPN	131	2	65	70	43	1682	26	M	2	2	no	vertical	vertical	left	no	0	0	45,2	no
Lyon	CSI5*-W presented by GL EVENTS round B	05.11.2017	CSI-W5*	3	1.40 - 1.60	280	71	10	25.05.2008	M	KWPN	131	2	65	70	43	1682	26	M	3	3	no	spread	oxer	right	no	0	0	45,2	no
Lyon	CSI5*-W presented by GL EVENTS round B	05.11.2017	CSI-W5*	3	1.40 - 1.60	280	71	10	25.05.2008	M	KWPN	131	2	65	70	43	1682	26	M	4	4	no	spread	oxer	left	no	0	0	45,2	no
Lyon	CSI5*-W presented by GL EVENTS round B	05.11.2017	CSI-W5*	3	1.40 - 1.60	280	71	10	25.05.2008	M	KWPN	131	2	65	70	43	1682	26	M	5	5a	yes	vertical	vertical	straight	no	0	0	45,2	no
Lyon	CSI5*-W presented by GL EVENTS round B	05.11.2017	CSI-W5*	3	1.40 - 1.60	280	71	10	25.05.2008	M	KWPN	131	2	65	70	43	1682	26	M	6	5b	yes	vertical	vertical with water	straight	no	0	0	45,2	no
Lyon	CSI5*-W presented by GL EVENTS round B	05.11.2017	CSI-W5*	3	1.40 - 1.60	280	71	10	25.05.2008	M	KWPN	131	2	65	70	43	1682	26	M	7	6	no	spread	oxer	left	no	0	0	45,2	no
Lyon	CSI5*-W presented by GL EVENTS round B	05.11.2017	CSI-W5*	3	1.40 - 1.60	280	71	10	25.05.2008	M	KWPN	131	2	65	70	43	1682	26	M	8	7	no	spread	oxer	right	no	0	0	45,2	no
Lyon	CSI5*-W presented by GL EVENTS round A	05.11.2017	CSI-W5*	3	1.40 - 1.60	420	211	13	11.05.2005	S	SBS	150	4	70	43	38	1891	85	F	1	1	no	spread	oxer	right	no	0	0	68,99	no
Lyon	CSI5*-W presented by GL EVENTS round A	05.11.2017	CSI-W5*	3	1.40 - 1.60	420	211	13	11.05.2005	S	SBS	150	4	70	43	38	1891	85	F	2	2	no	vertical	vertical	right	no	0	0	68,99	no
Lyon	CSI5*-W presented by GL EVENTS round A	05.11.2017	CSI-W5*	3	1.40 - 1.60	420	211	13	11.05.2005	S	SBS	150	4	70	43	38	1891	85	F	3	3	no	spread	oxer	right	no	0	0	68,99	no
Lyon	CSI5*-W presented by GL EVENTS round A	05.11.2017	CSI-W5*	3	1.40 - 1.60	420	211	13	11.05.2005	S	SBS	150	4	70	43	38	1891	85	F	4	4	no	spread	oxer	left	yes	4	0	68,99	no
Lyon	CSI5*-W presented by GL EVENTS round A	05.11.2017	CSI-W5*	3	1.40 - 1.60	420	211	13	11.05.2005	S	SBS	150	4	70	43	38	1891	85	F	5	5	no	vertical	vertical	straight	no	0	0	68,99	no
Lyon	CSI5*-W presented by GL EVENTS round A	05.11.2017	CSI-W5*	3	1.40 - 1.60	420	211	13	11.05.2005	S	SBS	150	4	70	43	38	1891	85	F	6	6	no	vertical	vertical	left	no	0	0	68,99	no
Lyon	CSI5*-W presented by GL EVENTS round A	05.11.2017	CSI-W5*	3	1.40 - 1.60	420	211	13	11.05.2005	S	SBS	150	4	70	43	38	1891	85	F	7	7a	yes	spread	oxer	straight	no	0	0	68,99	no
Lyon	CSI5*-W presented by GL EVENTS round A	05.11.2017	CSI-W5*	3	1.40 - 1.60	420	211	13	11.05.2005	S	SBS	150	4	70	43	38	1891	85	F	8	7b	yes	vertical	vertical	straight	no	0	0	68,99	no
Lyon	CSI5*-W presented by GL EVENTS round A	05.11.2017	CSI-W5*	3	1.40 - 1.60	420	211	13	11.05.2005	S	SBS	150	4	70	43	38	1891	85	F	9	7c	yes	spread	oxer	straight	no	0	0	68,99	no
Lyon	CSI5*-W presented by GL EVENTS round A	05.11.2017	CSI-W5*	3	1.40 - 1.60	420	211	13	11.05.2005	S	SBS	150	4	70	43	38	1891	85	F	10	8	no	vertical	wall	right	no	0	0	68,99	no
Lyon	CSI5*-W presented by GL EVENTS round A	05.11.2017	CSI-W5*	3	1.40 - 1.60	420	211	13	11.05.2005	S	SBS	150	4	70	43	38	1891	85	F	11	9	no	vertical	vertical	right	no	0	0	68,99	no
Lyon	CSI5*-W presented by GL EVENTS round A	05.11.2017	CSI-W5*	3	1.40 - 1.60	420	211	13	11.05.2005	S	SBS	150	4	70	43	38	1891	85	F	12	10	no	spread	oxer	right	no	0	0	68,99	no
Lyon	CSI5*-W presented by GL EVENTS round A	05.11.2017	CSI-W5*	3	1.40 - 1.60	420	211	13	11.05.2005	S	SBS	150	4	70	43	38	1891	85	F	13	11	no	spread	oxer	left	no	0	0	68,99	no
Lyon	CSI5*-W presented by GL EVENTS round A	05.11.2017	CSI-W5*	3	1.40 - 1.60	420	211	13	11.05.2005	S	SBS	150	4	70	43	38	1891	85	F	14	12a	yes	vertical	vertical	left	no	0	0	68,99	no
Lyon	CSI5*-W presented by GL EVENTS round A	05.11.2017	CSI-W5*	3	1.40 - 1.60	420	211	13	11.05.2005	S	SBS	150	4	70	43	38	1891	85	F	15	12b	yes	vertical	vertical with water	left	no	0	0	68,99	no
Lyon	CSI5*-W presented by GL EVENTS round A	05.11.2017	CSI-W5*	3	1.40 - 1.60	420	211	13	11.05.2005	S	SBS	150	4	70	43	38	1891	85	F	16	13	no	spread	oxer with water	straight	yes	4	0	68,99	no
Lyon	CSI5*-W presented by GL EVENTS round A	05.11.2017	CSI-W5*	3	1.40 - 1.60	420	94	13	23.02.2005	S	WESTF	178	6	71	12	44	2177	93	M	1	1	no	spread	oxer	right	no	0	0	0,00	yes
Lyon	CSI5*-W presented by GL EVENTS round A	05.11.2017	CSI-W5*	3	1.40 - 1.60	420	94	13	23.02.2005	S	WESTF	178	6	71	12	44	2177	93	M	2	2	no	vertical	vertical	right	no	0	0	0,00	yes
Lyon	CSI5*-W presented by GL EVENTS round A	05.11.2017	CSI-W5*	3	1.40 - 1.60	420	94	13	23.02.2005	S	WESTF	178	6	71	12	44	2177	93	M	3	3	no	spread	oxer	right	no	0	0	0,00	yes
Lyon	CSI5*-W presented by GL EVENTS round A	05.11.2017	CSI-W5*	3	1.40 - 1.60	420	94	13	23.02.2005	S	WESTF	178	6	71	12	44	2177	93	M	4	4	no	spread	oxer	left	no	0	0	0,00	yes
Lyon	CSI5*-W presented by GL EVENTS round A	05.11.2017	CSI-W5*	3	1.40 - 1.60	420	94	13	23.02.2005	S	WESTF	178	6	71	12	44	2177	93	M	5	5	no	vertical	vertical	straight	no	0	0	0,00	yes
Lyon	CSI5*-W presented by GL EVENTS round A	05.11.2017	CSI-W5*	3	1.40 - 1.60	420	94	13	23.02.2005	S	WESTF	178	6	71	12	44	2177	93	M	6	6	no	vertical	vertical	left	yes	4	0	0,00	yes
Lyon	CSI5*-W presented by GL EVENTS round A	05.11.2017	CSI-W5*	3	1.40 - 1.60	420	94	13	23.02.2005	S	WESTF	178	6	71	12	44	2177	93	M	7	7a	yes	spread	oxer	straight	no	0	0	0,00	yes
Lyon	CSI5*-W presented by GL EVENTS round A	05.11.2017	CSI-W5*	3	1.40 - 1.60	420	94	13	23.02.2005	S	WESTF	178	6	71	12	44	2177	93	M	8	7b	yes	vertical	vertical	straight	no	0	0	0,00	yes
Lyon	CSI5*-W presented by GL EVENTS round A	05.11.2017	CSI-W5*	3	1.40 - 1.60	420	94	13	23.02.2005	S	WESTF	178	6	71	12	44	2177	93	M	9	7c	yes	spread	oxer	straight	no	0	0	0,00	yes
Lyon	CSI5*-W presented by GL EVENTS round A	05.11.2017	CSI-W5*	3	1.40 - 1.60	420	94	13	23.02.2005	S	WESTF	178	6	71	12	44	2177	93	M	10	8	no	vertical	wall	right	yes	4	0	0,00	yes
Lyon	CSI5*-W presented by GL EVENTS round A	05.11.2017	CSI-W5*	3	1.40 - 1.60	420	94	13	23.02.2005	S	WESTF	178	6	71	12	44	2177	93	M	11	9	no	vertical	vertical	right	no	0	0	0,00	yes
Lyon	CSI5*-W presented by GL EVENTS round A	05.11.2017	CSI-W5*	3	1.40 - 1.60	420	94	13	23.02.2005	S	WESTF	178	6	71	12	44	2177	93	M	12	10	no	spread	oxer	right	no	0	0	0,00	yes
Lyon	CSI5*-W presented by GL EVENTS round A	05.11.2017	CSI-W5*	3	1.40 - 1.60	420	94	13	23.02.2005	S	WESTF	178	6	71	12	44	2177	93	M	13	11	no	spread	oxer	left	no	0	0	0,00	yes
Lyon	CSI5*-W presented by GL EVENTS round A	05.11.2017	CSI-W5*	3	1.40 - 1.60	420	94	13	23.02.2005	S	WESTF	178	6	71	12	44	2177	93	M	14	12a	yes	vertical	vertical	left	no	0	0	0,00	yes
Lyon	CSI5*-W presented by GL EVENTS round A	05.11.2017	CSI-W5*	3	1.40 - 1.60	420	94	13	23.02.2005	S	WESTF	178	6	71	12	44	2177	93	M	15	12b	yes	vertical	vertical with water	left	no	0	0	0,00	yes
Lyon	CSI5*-W presented by GL EVENTS round A	05.11.2017	CSI-W5*	3	1.40 - 1.60	420	94	13	23.02.2005	S	WESTF	178	6	71	12	44	2177	93	M	16	13	no	spread	oxer with water	straight	no	0	0	0,00	yes
Lyon	CSI5*-W presented by GL EVENTS round A	05.11.2017	CSI-W5*	3	1.40 - 1.60	420	173	13	14.07.2005	G	SF	229	2	74	65	34	1260	61	M	1	1	no	spread	oxer	right	no	0	0	67,55	no
Lyon	CSI5*-W presented by GL EVENTS round A	05.11.2017	CSI-W5*	3	1.40 - 1.60	420	173	13	14.07.2005	G	SF	229	2	74	65	34	1260	61	M	2	2	no	vertical	vertical	right	no	0	0	67,55	no
Lyon	CSI5*-W presented by GL EVENTS round A	05.11.2017	CSI-W5*	3	1.40 - 1.60	420	173	13	14.07.2005	G	SF	229	2	74	65	34	1260	61	M	3	3	no	spread	oxer	right	no	0	0	67,55	no
Lyon	CSI5*-W presented by GL EVENTS round A	05.11.2017	CSI-W5*	3	1.40 - 1.60	420	173	13	14.07.2005	G	SF	229	2	74	65	34	1260	61	M	4	4	no	spread	oxer	left	no	0	0	67,55	no
Lyon	CSI5*-W presented by GL EVENTS round A	05.11.2017	CSI-W5*	3	1.40 - 1.60	420	173	13	14.07.2005	G	SF	229	2	74	65	34	1260	61	M	5	5	no	vertical	vertical	straight	no	0	0	67,55	no
Lyon	CSI5*-W presented by GL EVENTS round A	05.11.2017	CSI-W5*	3	1.40 - 1.60	420	173	13	14.07.2005	G	SF	229	2	74	65	34	1260	61	M	6	6	no	vertical	vertical	left	yes	4	0	67,55	no
Lyon	CSI5*-W presented by GL EVENTS round A	05.11.2017	CSI-W5*	3	1.40 - 1.60	420	173	13	14.07.2005	G	SF	229	2	74	65	34	1260	61	M	7	7a	yes	spread	oxer	straight	no	0	0	67,55	no
Lyon	CSI5*-W presented by GL EVENTS round A	05.11.2017	CSI-W5*	3	1.40 - 1.60	420	173	13	14.07.2005	G	SF	229	2	74	65	34	1260	61	M	8	7b	yes	vertical	vertical	straight	no	0	0	67,55	no
Lyon	CSI5*-W presented by GL EVENTS round A	05.11.2017	CSI-W5*	3	1.40 - 1.60	420	173	13	14.07.2005	G	SF	229	2	74	65	34	1260	61	M	9	7c	yes	spread	oxer	straight	no	0	0	67,55	no
Lyon	CSI5*-W presented by GL EVENTS round A	05.11.2017	CSI-W5*	3	1.40 - 1.60	420	173	13	14.07.2005	G	SF	229	2	74	65	34	1260	61	M	10	8	no	vertical	wall	right	no	0	0	67,55	no
Lyon	CSI5*-W presented by GL EVENTS round A	05.11.2017	CSI-W5*	3	1.40 - 1.60	420	173	13	14.07.2005	G	SF	229	2	74	65	34	1260	61	M	11	9	no	vertical	vertical	right	no	0	0	67,55	no
Lyon	CSI5*-W presented by GL EVENTS round A	05.11.2017	CSI-W5*	3	1.40 - 1.60	420	173	13	14.07.2005	G	SF	229	2	74	65	34	1260	61	M	12	10	no	spread	oxer	right	no	0	0	67,55	no
Lyon	CSI5*-W presented by GL EVENTS round A	05.11.2017	CSI-W5*	3	1.40 - 1.60	420	173	13	14.07.2005	G	SF	229	2	74	65	34	1260	61	M	13	11	no	spread	oxer	left	no	0	0	67,55	no
Lyon	CSI5*-W presented by GL EVENTS round A	05.11.2017	CSI-W5*	3	1.40 - 1.60	420	173	13	14.07.2005	G	SF	229	2	74	65	34	1260	61	M	14	12a	yes	vertical	vertical	left	no	0	0	67,55	no
Lyon	CSI5*-W presented by GL EVENTS round A	05.11.2017	CSI-W5*	3	1.40 - 1.60	420	173	13	14.07.2005	G	SF	229	2	74	65	34	1260	61	M	15	12b	yes	vertical	vertical with water	left	no	0	0	67,55	no
Lyon	CSI5*-W presented by GL EVENTS round A	05.11.2017	CSI-W5*	3	1.40 - 1.60	420	173	13	14.07.2005	G	SF	229	2	74	65	34	1260	61	M	16	13	no	spread	oxer with water	straight	yes	4	0	67,55	no
Lyon	CSI5*-W presented by GL EVENTS round A	05.11.2017	CSI-W5*	3	1.40 - 1.60	420	192	12	27.04.2006	M	SF	164	14	75	22	53	2104	161	M	1	1	no	spread	oxer	right	no	0	0	67,25	no
Lyon	CSI5*-W presented by GL EVENTS round A	05.11.2017	CSI-W5*	3	1.40 - 1.60	420	192	12	27.04.2006	M	SF	164	14	75	22	53	2104	161	M	2	2	no	vertical	vertical	right	no	0	0	67,25	no
Lyon	CSI5*-W presented by GL EVENTS round A	05.11.2017	CSI-W5*	3	1.40 - 1.60	420	192	12	27.04.2006	M	SF	164	14	75	22	53	2104	161	M	3	3	no	spread	oxer	right	no	0	0	67,25	no
Lyon	CSI5*-W presented by GL EVENTS round A	05.11.2017	CSI-W5*	3	1.40 - 1.60	420	192	12	27.04.2006	M	SF	164	14	75	22	53	2104	161	M	4	4	no	spread	oxer	left	no	0	0	67,25	no
Lyon	CSI5*-W presented by GL EVENTS round A	05.11.2017	CSI-W5*	3	1.40 - 1.60	420	192	12	27.04.2006	M	SF	164	14	75	22	53	2104	161	M	5	5	no	vertical	vertical	straight	no	0	0	67,25	no
Lyon	CSI5*-W presented by GL EVENTS round A	05.11.2017	CSI-W5*	3	1.40 - 1.60	420	192	12	27.04.2006	M	SF	164	14	75	22	53	2104	161	M	6	6	no	vertical	vertical	left	no	0	0	67,25	no
Lyon	CSI5*-W presented by GL EVENTS round A	05.11.2017	CSI-W5*	3	1.40 - 1.60	420	192	12	27.04.2006	M	SF	164	14	75	22	53	2104	161	M	7	7a	yes	spread	oxer	straight	no	0	0	67,25	no
Lyon	CSI5*-W presented by GL EVENTS round A	05.11.2017	CSI-W5*	3	1.40 - 1.60	420	192	12	27.04.2006	M	SF	164	14	75	22	53	2104	161	M	8	7b	yes	vertical	vertical	straight	no	0	0	67,25	no
Lyon	CSI5*-W presented by GL EVENTS round A	05.11.2017	CSI-W5*	3	1.40 - 1.60	420	192	12	27.04.2006	M	SF	164	14	75	22	53	2104	161	M	9	7c	yes	spread	oxer	straight	no	0	0	67,25	no
Lyon	CSI5*-W presented by GL EVENTS round A	05.11.2017	CSI-W5*	3	1.40 - 1.60	420	192	12	27.04.2006	M	SF	164	14	75	22	53	2104	161	M	10	8	no	vertical	wall	right	no	0	0	67,25	no
Lyon	CSI5*-W presented by GL EVENTS round A	05.11.2017	CSI-W5*	3	1.40 - 1.60	420	192	12	27.04.2006	M	SF	164	14	75	22	53	2104	161	M	11	9	no	vertical	vertical	right	no	0	0	67,25	no
Lyon	CSI5*-W presented by GL EVENTS round A	05.11.2017	CSI-W5*	3	1.40 - 1.60	420	192	12	27.04.2006	M	SF	164	14	75	22	53	2104	161	M	12	10	no	spread	oxer	right	no	0	0	67,25	no
Lyon	CSI5*-W presented by GL EVENTS round A	05.11.2017	CSI-W5*	3	1.40 - 1.60	420	192	12	27.04.2006	M	SF	164	14	75	22	53	2104	161	M	13	11	no	spread	oxer	left	no	0	0	67,25	no
Lyon	CSI5*-W presented by GL EVENTS round A	05.11.2017	CSI-W5*	3	1.40 - 1.60	420	192	12	27.04.2006	M	SF	164	14	75	22	53	2104	161	M	14	12a	yes	vertical	vertical	left	no	0	0	67,25	no
Lyon	CSI5*-W presented by GL EVENTS round A	05.11.2017	CSI-W5*	3	1.40 - 1.60	420	192	12	27.04.2006	M	SF	164	14	75	22	53	2104	161	M	15	12b	yes	vertical	vertical with water	left	no	0	0	67,25	no
Lyon	CSI5*-W presented by GL EVENTS round A	05.11.2017	CSI-W5*	3	1.40 - 1.60	420	192	12	27.04.2006	M	SF	164	14	75	22	53	2104	161	M	16	13	no	spread	oxer with water	straight	no	0	0	67,25	no
Lyon	CSI5*-W presented by GL EVENTS round B	05.11.2017	CSI-W5*	3	1.40 - 1.60	280	192	12	27.04.2006	M	SF	164	14	75	22	53	2104	161	M	1	1	no	vertical	vertical	left	no	0	0	40,25	no
Lyon	CSI5*-W presented by GL EVENTS round B	05.11.2017	CSI-W5*	3	1.40 - 1.60	280	192	12	27.04.2006	M	SF	164	14	75	22	53	2104	161	M	2	2	no	vertical	vertical	left	no	0	0	40,25	no
Lyon	CSI5*-W presented by GL EVENTS round B	05.11.2017	CSI-W5*	3	1.40 - 1.60	280	192	12	27.04.2006	M	SF	164	14	75	22	53	2104	161	M	3	3	no	spread	oxer	right	no	0	0	40,25	no
Lyon	CSI5*-W presented by GL EVENTS round B	05.11.2017	CSI-W5*	3	1.40 - 1.60	280	192	12	27.04.2006	M	SF	164	14	75	22	53	2104	161	M	4	4	no	spread	oxer	left	yes	4	0	40,25	no
Lyon	CSI5*-W presented by GL EVENTS round B	05.11.2017	CSI-W5*	3	1.40 - 1.60	280	192	12	27.04.2006	M	SF	164	14	75	22	53	2104	161	M	5	5a	yes	vertical	vertical	straight	yes	4	0	40,25	no
Lyon	CSI5*-W presented by GL EVENTS round B	05.11.2017	CSI-W5*	3	1.40 - 1.60	280	192	12	27.04.2006	M	SF	164	14	75	22	53	2104	161	M	6	5b	yes	vertical	vertical with water	straight	no	0	0	40,25	no
Lyon	CSI5*-W presented by GL EVENTS round B	05.11.2017	CSI-W5*	3	1.40 - 1.60	280	192	12	27.04.2006	M	SF	164	14	75	22	53	2104	161	M	7	6	no	spread	oxer	left	no	0	0	40,25	no
Lyon	CSI5*-W presented by GL EVENTS round B	05.11.2017	CSI-W5*	3	1.40 - 1.60	280	192	12	27.04.2006	M	SF	164	14	75	22	53	2104	161	M	8	7	no	spread	oxer	right	no	0	0	40,25	no
Lyon	CSI5*-W presented by GL EVENTS round A	05.11.2017	CSI-W5*	3	1.40 - 1.60	420	58	13	04.02.2005	G	DSP	255	4	76	55	44	1886	48	M	1	1	no	spread	oxer	right	no	0	0	69,67	no
Lyon	CSI5*-W presented by GL EVENTS round A	05.11.2017	CSI-W5*	3	1.40 - 1.60	420	58	13	04.02.2005	G	DSP	255	4	76	55	44	1886	48	M	2	2	no	vertical	vertical	right	no	0	0	69,67	no
Lyon	CSI5*-W presented by GL EVENTS round A	05.11.2017	CSI-W5*	3	1.40 - 1.60	420	58	13	04.02.2005	G	DSP	255	4	76	55	44	1886	48	M	3	3	no	spread	oxer	right	no	0	0	69,67	no
Lyon	CSI5*-W presented by GL EVENTS round A	05.11.2017	CSI-W5*	3	1.40 - 1.60	420	58	13	04.02.2005	G	DSP	255	4	76	55	44	1886	48	M	4	4	no	spread	oxer	left	no	0	0	69,67	no
Lyon	CSI5*-W presented by GL EVENTS round A	05.11.2017	CSI-W5*	3	1.40 - 1.60	420	58	13	04.02.2005	G	DSP	255	4	76	55	44	1886	48	M	5	5	no	vertical	vertical	straight	no	0	0	69,67	no
Lyon	CSI5*-W presented by GL EVENTS round A	05.11.2017	CSI-W5*	3	1.40 - 1.60	420	58	13	04.02.2005	G	DSP	255	4	76	55	44	1886	48	M	6	6	no	vertical	vertical	left	yes	4	0	69,67	no
Lyon	CSI5*-W presented by GL EVENTS round A	05.11.2017	CSI-W5*	3	1.40 - 1.60	420	58	13	04.02.2005	G	DSP	255	4	76	55	44	1886	48	M	7	7a	yes	spread	oxer	straight	no	0	0	69,67	no
Lyon	CSI5*-W presented by GL EVENTS round A	05.11.2017	CSI-W5*	3	1.40 - 1.60	420	58	13	04.02.2005	G	DSP	255	4	76	55	44	1886	48	M	8	7b	yes	vertical	vertical	straight	no	0	0	69,67	no
Lyon	CSI5*-W presented by GL EVENTS round A	05.11.2017	CSI-W5*	3	1.40 - 1.60	420	58	13	04.02.2005	G	DSP	255	4	76	55	44	1886	48	M	9	7c	yes	spread	oxer	straight	yes	4	0	69,67	no
Lyon	CSI5*-W presented by GL EVENTS round A	05.11.2017	CSI-W5*	3	1.40 - 1.60	420	58	13	04.02.2005	G	DSP	255	4	76	55	44	1886	48	M	10	8	no	vertical	wall	right	no	0	0	69,67	no
Lyon	CSI5*-W presented by GL EVENTS round A	05.11.2017	CSI-W5*	3	1.40 - 1.60	420	58	13	04.02.2005	G	DSP	255	4	76	55	44	1886	48	M	11	9	no	vertical	vertical	right	no	0	0	69,67	no
Lyon	CSI5*-W presented by GL EVENTS round A	05.11.2017	CSI-W5*	3	1.40 - 1.60	420	58	13	04.02.2005	G	DSP	255	4	76	55	44	1886	48	M	12	10	no	spread	oxer	right	no	0	0	69,67	no

Lyon	CSI5*-W presented by GL EVENTS round A	05.11.2017	CSI-W5*	3	1.40 - 1.60	420	58	13	04.02.2005	G	DSP	255	4	76	55	44	1886	48	M	13	11	no	spread	oxer	left	no	0	0	69,67	no
Lyon	CSI5*-W presented by GL EVENTS round A	05.11.2017	CSI-W5*	3	1.40 - 1.60	420	58	13	04.02.2005	G	DSP	255	4	76	55	44	1886	48	M	14	12a	yes	vertical	vertical	left	no	0	0	69,67	no
Lyon	CSI5*-W presented by GL EVENTS round A	05.11.2017	CSI-W5*	3	1.40 - 1.60	420	58	13	04.02.2005	G	DSP	255	4	76	55	44	1886	48	M	15	12b	yes	vertical	vertical with water	left	no	0	0	69,67	no
Lyon	CSI5*-W presented by GL EVENTS round A	05.11.2017	CSI-W5*	3	1.40 - 1.60	420	58	13	04.02.2005	G	DSP	255	4	76	55	44	1886	48	M	16	13	no	spread	oxer with water	straight	no	0	0	69,67	no
Lyon	CSI5*-W presented by GL EVENTS round A	05.11.2017	CSI-W5*	3	1.40 - 1.60	420	95	13	17.04.2005	G	KWPN	146	6	77	27	33	1671	77	M	1	1	no	spread	oxer	right	no	0	0	68,51	no
Lyon	CSI5*-W presented by GL EVENTS round A	05.11.2017	CSI-W5*	3	1.40 - 1.60	420	95	13	17.04.2005	G	KWPN	146	6	77	27	33	1671	77	M	2	2	no	vertical	vertical	right	no	0	0	68,51	no
Lyon	CSI5*-W presented by GL EVENTS round A	05.11.2017	CSI-W5*	3	1.40 - 1.60	420	95	13	17.04.2005	G	KWPN	146	6	77	27	33	1671	77	M	3	3	no	spread	oxer	right	no	0	0	68,51	no
Lyon	CSI5*-W presented by GL EVENTS round A	05.11.2017	CSI-W5*	3	1.40 - 1.60	420	95	13	17.04.2005	G	KWPN	146	6	77	27	33	1671	77	M	4	4	no	spread	oxer	left	no	0	0	68,51	no
Lyon	CSI5*-W presented by GL EVENTS round A	05.11.2017	CSI-W5*	3	1.40 - 1.60	420	95	13	17.04.2005	G	KWPN	146	6	77	27	33	1671	77	M	5	5	no	vertical	vertical	straight	no	0	0	68,51	no
Lyon	CSI5*-W presented by GL EVENTS round A	05.11.2017	CSI-W5*	3	1.40 - 1.60	420	95	13	17.04.2005	G	KWPN	146	6	77	27	33	1671	77	M	6	6	no	vertical	vertical	left	no	0	0	68,51	no
Lyon	CSI5*-W presented by GL EVENTS round A	05.11.2017	CSI-W5*	3	1.40 - 1.60	420	95	13	17.04.2005	G	KWPN	146	6	77	27	33	1671	77	M	7	7a	yes	spread	oxer	straight	no	0	0	68,51	no
Lyon	CSI5*-W presented by GL EVENTS round A	05.11.2017	CSI-W5*	3	1.40 - 1.60	420	95	13	17.04.2005	G	KWPN	146	6	77	27	33	1671	77	M	8	7b	yes	vertical	vertical	straight	no	0	0	68,51	no
Lyon	CSI5*-W presented by GL EVENTS round A	05.11.2017	CSI-W5*	3	1.40 - 1.60	420	95	13	17.04.2005	G	KWPN	146	6	77	27	33	1671	77	M	9	7c	yes	spread	oxer	straight	no	0	0	68,51	no
Lyon	CSI5*-W presented by GL EVENTS round A	05.11.2017	CSI-W5*	3	1.40 - 1.60	420	95	13	17.04.2005	G	KWPN	146	6	77	27	33	1671	77	M	10	8	no	vertical	wall	right	no	0	0	68,51	no
Lyon	CSI5*-W presented by GL EVENTS round A	05.11.2017	CSI-W5*	3	1.40 - 1.60	420	95	13	17.04.2005	G	KWPN	146	6	77	27	33	1671	77	M	11	9	no	vertical	vertical	right	no	0	0	68,51	no
Lyon	CSI5*-W presented by GL EVENTS round A	05.11.2017	CSI-W5*	3	1.40 - 1.60	420	95	13	17.04.2005	G	KWPN	146	6	77	27	33	1671	77	M	12	10	no	spread	oxer	right	no	0	0	68,51	no
Lyon	CSI5*-W presented by GL EVENTS round A	05.11.2017	CSI-W5*	3	1.40 - 1.60	420	95	13	17.04.2005	G	KWPN	146	6	77	27	33	1671	77	M	13	11	no	spread	oxer	left	no	0	0	68,51	no
Lyon	CSI5*-W presented by GL EVENTS round A	05.11.2017	CSI-W5*	3	1.40 - 1.60	420	95	13	17.04.2005	G	KWPN	146	6	77	27	33	1671	77	M	14	12a	yes	vertical	vertical	left	no	0	0	68,51	no
Lyon	CSI5*-W presented by GL EVENTS round A	05.11.2017	CSI-W5*	3	1.40 - 1.60	420	95	13	17.04.2005	G	KWPN	146	6	77	27	33	1671	77	M	15	12b	yes	vertical	vertical with water	left	no	0	0	68,51	no
Lyon	CSI5*-W presented by GL EVENTS round A	05.11.2017	CSI-W5*	3	1.40 - 1.60	420	95	13	17.04.2005	G	KWPN	146	6	77	27	33	1671	77	M	16	13	no	spread	oxer with water	straight	no	0	0	68,51	no
Lyon	CSI5*-W presented by GL EVENTS round B	05.11.2017	CSI-W5*	3	1.40 - 1.60	280	95	13	17.04.2005	G	KWPN	146	6	77	27	33	1671	77	M	1	1	no	vertical	vertical	left	no	0	0	38,12	no
Lyon	CSI5*-W presented by GL EVENTS round B	05.11.2017	CSI-W5*	3	1.40 - 1.60	280	95	13	17.04.2005	G	KWPN	146	6	77	27	33	1671	77	M	2	2	no	vertical	vertical	left	no	0	0	38,12	no
Lyon	CSI5*-W presented by GL EVENTS round B	05.11.2017	CSI-W5*	3	1.40 - 1.60	280	95	13	17.04.2005	G	KWPN	146	6	77	27	33	1671	77	M	3	3	no	spread	oxer	right	no	0	0	38,12	no
Lyon	CSI5*-W presented by GL EVENTS round B	05.11.2017	CSI-W5*	3	1.40 - 1.60	280	95	13	17.04.2005	G	KWPN	146	6	77	27	33	1671	77	M	4	4	no	spread	oxer	left	no	0	0	38,12	no
Lyon	CSI5*-W presented by GL EVENTS round B	05.11.2017	CSI-W5*	3	1.40 - 1.60	280	95	13	17.04.2005	G	KWPN	146	6	77	27	33	1671	77	M	5	5a	yes	vertical	vertical	straight	yes	4	0	38,12	no
Lyon	CSI5*-W presented by GL EVENTS round B	05.11.2017	CSI-W5*	3	1.40 - 1.60	280	95	13	17.04.2005	G	KWPN	146	6	77	27	33	1671	77	M	6	5b	yes	vertical	vertical with water	straight	no	0	0	38,12	no
Lyon	CSI5*-W presented by GL EVENTS round B	05.11.2017	CSI-W5*	3	1.40 - 1.60	280	95	13	17.04.2005	G	KWPN	146	6	77	27	33	1671	77	M	7	6	no	spread	oxer	left	no	0	0	38,12	no
Lyon	CSI5*-W presented by GL EVENTS round B	05.11.2017	CSI-W5*	3	1.40 - 1.60	280	95	13	17.04.2005	G	KWPN	146	6	77	27	33	1671	77	M	8	7	no	spread	oxer	right	no	0	0	38,12	no
Lyon	CSI5*-W presented by GL EVENTS round A	05.11.2017	CSI-W5*	3	1.40 - 1.60	420	123	10	05.04.2008	S	WESTF	82	0	79	166	25	1567	29	M	1	1	no	spread	oxer	right	no	0	0	70,56	no
Lyon	CSI5*-W presented by GL EVENTS round A	05.11.2017	CSI-W5*	3	1.40 - 1.60	420	123	10	05.04.2008	S	WESTF	82	0	79	166	25	1567	29	M	2	2	no	vertical	vertical	right	no	0	0	70,56	no
Lyon	CSI5*-W presented by GL EVENTS round A	05.11.2017	CSI-W5*	3	1.40 - 1.60	420	123	10	05.04.2008	S	WESTF	82	0	79	166	25	1567	29	M	3	3	no	spread	oxer	right	no	0	0	70,56	no
Lyon	CSI5*-W presented by GL EVENTS round A	05.11.2017	CSI-W5*	3	1.40 - 1.60	420	123	10	05.04.2008	S	WESTF	82	0	79	166	25	1567	29	M	4	4	no	spread	oxer	left	no	0	0	70,56	no
Lyon	CSI5*-W presented by GL EVENTS round A	05.11.2017	CSI-W5*	3	1.40 - 1.60	420	123	10	05.04.2008	S	WESTF	82	0	79	166	25	1567	29	M	5	5	no	vertical	vertical	straight	no	0	0	70,56	no
Lyon	CSI5*-W presented by GL EVENTS round A	05.11.2017	CSI-W5*	3	1.40 - 1.60	420	123	10	05.04.2008	S	WESTF	82	0	79	166	25	1567	29	M	6	6	no	vertical	vertical	left	no	0	0	70,56	no
Lyon	CSI5*-W presented by GL EVENTS round A	05.11.2017	CSI-W5*	3	1.40 - 1.60	420	123	10	05.04.2008	S	WESTF	82	0	79	166	25	1567	29	M	7	7a	yes	spread	oxer	straight	no	0	0	70,56	no
Lyon	CSI5*-W presented by GL EVENTS round A	05.11.2017	CSI-W5*	3	1.40 - 1.60	420	123	10	05.04.2008	S	WESTF	82	0	79	166	25	1567	29	M	8	7b	yes	vertical	vertical	straight	no	0	0	70,56	no
Lyon	CSI5*-W presented by GL EVENTS round A	05.11.2017	CSI-W5*	3	1.40 - 1.60	420	123	10	05.04.2008	S	WESTF	82	0	79	166	25	1567	29	M	9	7c	yes	spread	oxer	straight	no	0	0	70,56	no
Lyon	CSI5*-W presented by GL EVENTS round A	05.11.2017	CSI-W5*	3	1.40 - 1.60	420	123	10	05.04.2008	S	WESTF	82	0	79	166	25	1567	29	M	10	8	no	vertical	wall	right	no	0	0	70,56	no
Lyon	CSI5*-W presented by GL EVENTS round A	05.11.2017	CSI-W5*	3	1.40 - 1.60	420	123	10	05.04.2008	S	WESTF	82	0	79	166	25	1567	29	M	11	9	no	vertical	vertical	right	no	0	0	70,56	no
Lyon	CSI5*-W presented by GL EVENTS round A	05.11.2017	CSI-W5*	3	1.40 - 1.60	420	123	10	05.04.2008	S	WESTF	82	0	79	166	25	1567	29	M	12	10	no	spread	oxer	right	no	0	0	70,56	no
Lyon	CSI5*-W presented by GL EVENTS round A	05.11.2017	CSI-W5*	3	1.40 - 1.60	420	123	10	05.04.2008	S	WESTF	82	0	79	166	25	1567	29	M	13	11	no	spread	oxer	left	no	0	0	70,56	no
Lyon	CSI5*-W presented by GL EVENTS round A	05.11.2017	CSI-W5*	3	1.40 - 1.60	420	123	10	05.04.2008	S	WESTF	82	0	79	166	25	1567	29	M	14	12a	yes	vertical	vertical	left	no	0	0	70,56	no
Lyon	CSI5*-W presented by GL EVENTS round A	05.11.2017	CSI-W5*	3	1.40 - 1.60	420	123	10	05.04.2008	S	WESTF	82	0	79	166	25	1567	29	M	15	12b	yes	vertical	vertical with water	left	no	0	0	70,56	no
Lyon	CSI5*-W presented by GL EVENTS round A	05.11.2017	CSI-W5*	3	1.40 - 1.60	420	123	10	05.04.2008	S	WESTF	82	0	79	166	25	1567	29	M	16	13	no	spread	oxer with water	straight	yes	4	1	70,56	no
Lyon	CSI5*-W presented by GL EVENTS round A	05.11.2017	CSI-W5*	3	1.40 - 1.60	420	185	12	06.05.2006	S	SF	74	2	80	117	24	877	59	M	1	1	no	spread	oxer	right	no	0	0	69,49	no
Lyon	CSI5*-W presented by GL EVENTS round A	05.11.2017	CSI-W5*	3	1.40 - 1.60	420	185	12	06.05.2006	S	SF	74	2	80	117	24	877	59	M	2	2	no	vertical	vertical	right	no	0	0	69,49	no
Lyon	CSI5*-W presented by GL EVENTS round A	05.11.2017	CSI-W5*	3	1.40 - 1.60	420	185	12	06.05.2006	S	SF	74	2	80	117	24	877	59	M	3	3	no	spread	oxer	right	no	0	0	69,49	no
Lyon	CSI5*-W presented by GL EVENTS round A	05.11.2017	CSI-W5*	3	1.40 - 1.60	420	185	12	06.05.2006	S	SF	74	2	80	117	24	877	59	M	4	4	no	spread	oxer	left	no	0	0	69,49	no
Lyon	CSI5*-W presented by GL EVENTS round A	05.11.2017	CSI-W5*	3	1.40 - 1.60	420	185	12	06.05.2006	S	SF	74	2	80	117	24	877	59	M	5	5	no	vertical	vertical	straight	yes	4	0	69,49	no
Lyon	CSI5*-W presented by GL EVENTS round A	05.11.2017	CSI-W5*	3	1.40 - 1.60	420	185	12	06.05.2006	S	SF	74	2	80	117	24	877	59	M	6	6	no	vertical	vertical	left	no	0	0	69,49	no
Lyon	CSI5*-W presented by GL EVENTS round A	05.11.2017	CSI-W5*	3	1.40 - 1.60	420	185	12	06.05.2006	S	SF	74	2	80	117	24	877	59	M	7	7a	yes	spread	oxer	straight	no	0	0	69,49	no
Lyon	CSI5*-W presented by GL EVENTS round A	05.11.2017	CSI-W5*	3	1.40 - 1.60	420	185	12	06.05.2006	S	SF	74	2	80	117	24	877	59	M	8	7b	yes	vertical	vertical	straight	no	0	0	69,49	no
Lyon	CSI5*-W presented by GL EVENTS round A	05.11.2017	CSI-W5*	3	1.40 - 1.60	420	185	12	06.05.2006	S	SF	74	2	80	117	24	877	59	M	9	7c	yes	spread	oxer	straight	no	0	0	69,49	no
Lyon	CSI5*-W presented by GL EVENTS round A	05.11.2017	CSI-W5*	3	1.40 - 1.60	420	185	12	06.05.2006	S	SF	74	2	80	117	24	877	59	M	10	8	no	vertical	wall	right	no	0	0	69,49	no
Lyon	CSI5*-W presented by GL EVENTS round A	05.11.2017	CSI-W5*	3	1.40 - 1.60	420	185	12	06.05.2006	S	SF	74	2	80	117	24	877	59	M	11	9	no	vertical	vertical	right	no	0	0	69,49	no
Lyon	CSI5*-W presented by GL EVENTS round A	05.11.2017	CSI-W5*	3	1.40 - 1.60	420	185	12	06.05.2006	S	SF	74	2	80	117	24	877	59	M	12	10	no	spread	oxer	right	no	0	0	69,49	no
Lyon	CSI5*-W presented by GL EVENTS round A	05.11.2017	CSI-W5*	3	1.40 - 1.60	420	185	12	06.05.2006	S	SF	74	2	80	117	24	877	59	M	13	11	no	spread	oxer	left	no	0	0	69,49	no
Lyon	CSI5*-W presented by GL EVENTS round A	05.11.2017	CSI-W5*	3	1.40 - 1.60	420	185	12	06.05.2006	S	SF	74	2	80	117	24	877	59	M	14	12a	yes	vertical	vertical	left	no	0	0	69,49	no
Lyon	CSI5*-W presented by GL EVENTS round A	05.11.2017	CSI-W5*	3	1.40 - 1.60	420	185	12	06.05.2006	S	SF	74	2	80	117	24	877	59	M	15	12b	yes	vertical	vertical with water	left	no	0	0	69,49	no
Lyon	CSI5*-W presented by GL EVENTS round A	05.11.2017	CSI-W5*	3	1.40 - 1.60	420	185	12	06.05.2006	S	SF	74	2	80	117	24	877	59	M	16	13	no	spread	oxer with water	straight	no	0	0	69,49	no
Lyon	CSI5*-W presented by GL EVENTS round A	05.11.2017	CSI-W5*	3	1.40 - 1.60	420	181	12	20.03.2006	G	ZANG	214	6	81	76	33	1923	71	M	1	1	no	spread	oxer	right	no	0	0	69,25	no
Lyon	CSI5*-W presented by GL EVENTS round A	05.11.2017	CSI-W5*	3	1.40 - 1.60	420	181	12	20.03.2006	G	ZANG	214	6	81	76	33	1923	71	M	2	2	no	vertical	vertical	right	no	0	0	69,25	no
Lyon	CSI5*-W presented by GL EVENTS round A	05.11.2017	CSI-W5*	3	1.40 - 1.60	420	181	12	20.03.2006	G	ZANG	214	6	81	76	33	1923	71	M	3	3	no	spread	oxer	right	no	0	0	69,25	no
Lyon	CSI5*-W presented by GL EVENTS round A	05.11.2017	CSI-W5*	3	1.40 - 1.60	420	181	12	20.03.2006	G	ZANG	214	6	81	76	33	1923	71	M	4	4	no	spread	oxer	left	no	0	0	69,25	no
Lyon	CSI5*-W presented by GL EVENTS round A	05.11.2017	CSI-W5*	3	1.40 - 1.60	420	181	12	20.03.2006	G	ZANG	214	6	81	76	33	1923	71	M	5	5	no	vertical	vertical	straight	yes	4	0	69,25	no
Lyon	CSI5*-W presented by GL EVENTS round A	05.11.2017	CSI-W5*	3	1.40 - 1.60	420	181	12	20.03.2006	G	ZANG	214	6	81	76	33	1923	71	M	6	6	no	vertical	vertical	left	no	0	0	69,25	no
Lyon	CSI5*-W presented by GL EVENTS round A	05.11.2017	CSI-W5*	3	1.40 - 1.60	420	181	12	20.03.2006	G	ZANG	214	6	81	76	33	1923	71	M	7	7a	yes	spread	oxer	straight	no	0	0	69,25	no
Lyon	CSI5*-W presented by GL EVENTS round A	05.11.2017	CSI-W5*	3	1.40 - 1.60	420	181	12	20.03.2006	G	ZANG	214	6	81	76	33	1923	71	M	8	7b	yes	vertical	vertical	straight	no	0	0	69,25	no
Lyon	CSI5*-W presented by GL EVENTS round A	05.11.2017	CSI-W5*	3	1.40 - 1.60	420	181	12	20.03.2006	G	ZANG	214	6	81	76	33	1923	71	M	9	7c	yes	spread	oxer	straight	no	0	0	69,25	no
Lyon	CSI5*-W presented by GL EVENTS round A	05.11.2017	CSI-W5*	3	1.40 - 1.60	420	181	12	20.03.2006	G	ZANG	214	6	81	76	33	1923	71	M	10	8	no	vertical	wall	right	no	0	0	69,25	no
Lyon	CSI5*-W presented by GL EVENTS round A	05.11.2017	CSI-W5*	3	1.40 - 1.60	420	181	12	20.03.2006	G	ZANG	214	6	81	76	33	1923	71	M	11	9	no	vertical	vertical	right	no	0	0	69,25	no
Lyon	CSI5*-W presented by GL EVENTS round A	05.11.2017	CSI-W5*	3	1.40 - 1.60	420	181	12	20.03.2006	G	ZANG	214	6	81	76	33	1923	71	M	12	10	no	spread	oxer	right	no	0	0	69,25	no
Lyon	CSI5*-W presented by GL EVENTS round A	05.11.2017	CSI-W5*	3	1.40 - 1.60	420	181	12	20.03.2006	G	ZANG	214	6	81	76	33	1923	71	M	13	11	no	spread	oxer	left	yes	4	0	69,25	no
Lyon	CSI5*-W presented by GL EVENTS round A	05.11.2017	CSI-W5*	3	1.40 - 1.60	420	181	12	20.03.2006	G	ZANG	214	6	81	76	33	1923	71	M	14	12a	yes	vertical	vertical	left	no	0	0	69,25	no
Lyon	CSI5*-W presented by GL EVENTS round A	05.11.2017	CSI-W5*	3	1.40 - 1.60	420	181	12	20.03.2006	G	ZANG	214	6	81	76	33	1923	71	M	15	12b	yes	vertical	vertical with water	left	no	0	0	69,25	no
Lyon	CSI5*-W presented by GL EVENTS round A	05.11.2017	CSI-W5*	3	1.40 - 1.60	420	181	12	20.03.2006	G	ZANG	214	6	81	76	33	1923	71	M	16	13	no	spread	oxer with water	straight	no	0	0	69,25	no
Lyon	CSI5*-W presented by GL EVENTS round A	05.11.2017	CSI-W5*	3	1.40 - 1.60	420	151	10	08.05.2008	M	WESTF	71	1	82	192	30	587	10	F	1	1	no	spread	oxer	right	no	0	0	68,98	no
Lyon	CSI5*-W presented by GL EVENTS round A	05.11.2017	CSI-W5*	3	1.40 - 1.60	420	151	10	08.05.2008	M	WESTF	71	1	82	192	30	587	10	F	2	2	no	vertical	vertical	right	no	0	0	68,98	no
Lyon	CSI5*-W presented by GL EVENTS round A	05.11.2017	CSI-W5*	3	1.40 - 1.60	420	151	10	08.05.2008	M	WESTF	71	1	82	192	30	587	10	F	3	3	no	spread	oxer	right	yes	4	0	68,98	no
Lyon	CSI5*-W presented by GL EVENTS round A	05.11.2017	CSI-W5*	3	1.40 - 1.60	420	151	10	08.05.2008	M	WESTF	71	1	82	192	30	587	10	F	4	4	no	spread	oxer	left	no	0	0	68,98	no
Lyon	CSI5*-W presented by GL EVENTS round A	05.11.2017	CSI-W5*	3	1.40 - 1.60	420	151	10	08.05.2008	M	WESTF	71	1	82	192	30	587	10	F	5	5	no	vertical	vertical	straight	no	0	0	68,98	no
Lyon	CSI5*-W presented by GL EVENTS round A	05.11.2017	CSI-W5*	3	1.40 - 1.60	420	151	10	08.05.2008	M	WESTF	71	1	82	192	30	587	10	F	6	6	no	vertical	vertical	left	no	0	0	68,98	no
Lyon	CSI5*-W presented by GL EVENTS round A	05.11.2017	CSI-W5*	3	1.40 - 1.60	420	151	10	08.05.2008	M	WESTF	71	1	82	192	30	587	10	F	7	7a	yes	spread	oxer	straight	no	0	0	68,98	no
Lyon	CSI5*-W presented by GL EVENTS round A	05.11.2017	CSI-W5*	3	1.40 - 1.60	420	151	10	08.05.2008	M	WESTF	71	1	82	192	30	587	10	F	8	7b	yes	vertical	vertical	straight	yes	4	0	68,98	no
Lyon	CSI5*-W presented by GL EVENTS round A	05.11.2017	CSI-W5*	3	1.40 - 1.60	420	151	10	08.05.2008	M	WESTF	71	1	82	192	30	587	10	F	9	7c	yes	spread	oxer	straight	no	0	0	68,98	no
Lyon	CSI5*-W presented by GL EVENTS round A	05.11.2017	CSI-W5*	3	1.40 - 1.60	420	151	10	08.05.2008	M	WESTF	71	1	82	192	30	587	10	F	10	8	no	vertical	wall	right	no	0	0	68,98	no
Lyon	CSI5*-W presented by GL EVENTS round A	05.11.2017	CSI-W5*	3	1.40 - 1.60	420	151	10	08.05.2008	M	WESTF	71	1	82	192	30	587	10	F	11	9	no	vertical	vertical	right	no	0	0	68,98	no
Lyon	CSI5*-W presented by GL EVENTS round A	05.11.2017	CSI-W5*	3	1.40 - 1.60	420	151	10	08.05.2008	M	WESTF	71	1	82	192	30	587	10	F	12	10	no	spread	oxer	right	no	0	0	68,98	no
Lyon	CSI5*-W presented by GL EVENTS round A	05.11.2017	CSI-W5*	3	1.40 - 1.60	420	151	10	08.05.2008	M	WESTF	71	1	82	192	30	587	10	F	13	11	no	spread	oxer	left	no	0	0	68,98	no
Lyon	CSI5*-W presented by GL EVENTS round A	05.11.2017	CSI-W5*	3	1.40 - 1.60	420	151	10	08.05.2008	M	WESTF	71	1	82	192	30	587	10	F	14	12a	yes	vertical	vertical	left	no	0	0	68,98	no
Lyon	CSI5*-W presented by GL EVENTS round A	05.11.2017	CSI-W5*	3	1.40 - 1.60	420	151	10	08.05.2008	M	WESTF	71	1	82	192	30	587	10	F	15	12b	yes	vertical	vertical with water	left	no	0	0	68,98	no
Lyon	CSI5*-W presented by GL EVENTS round A	05.11.2017	CSI-W5*	3	1.40 - 1.60	420	151	10	08.05.2008	M	WESTF	71	1	82	192	30	587	10	F	16	13	no	spread	oxer with water	straight	no	0	0	68,98	no
Lyon	CSI5*-W presented by GL EVENTS round A	05.11.2017	CSI-W5*	3	1.40 - 1.60	420	27	12	01.01.2006	M	ISH	146	2	83	395	25	483	10	M	1	1	no	spread	oxer	right	no	0	0	67,67	no
Lyon	CSI5*-W presented by GL EVENTS round A	05.11.2017	CSI-W5*	3	1.40 - 1.60	420	27	12	01.01.2006	M	ISH	146	2	83	395	25	483	10	M	2	2	no	vertical	vertical	right	no	0	0	67,67	no
Lyon	CSI5*-W presented by GL EVENTS round A	05.11.2017	CSI-W5*	3	1.40 - 1.60	420	27	12	01.01.2006	M	ISH	146	2	83	395	25	483	10	M	3	3	no	spread	oxer	right	yes	4	0	67,67	no
Lyon	CSI5*-W presented by GL EVENTS round A	05.11.2017	CSI-W5*	3	1.40 - 1.60	420	27	12	01.01.2006	M	ISH	146	2	83	395	25	483	10	M	4	4	no	spread	oxer	left	no	0	0	67,67	no
Lyon	CSI5*-W presented by GL EVENTS round A	05.11.2017	CSI-W5*	3	1.40 - 1.60	420	27	12	01.01.2006	M	ISH	146	2	83	395	25	483	10	M	5	5	no	vertical	vertical	straight	no	0	0	67,67	no
Lyon	CSI5*-W presented by GL EVENTS round A	05.11.2017	CSI-W5*	3	1.40 - 1.60	420	27	12	01.01.2006	M	ISH	146	2	83	395	25	483	10	M	6	6	no	vertical	vertical	left	no	0	0	67,67	no
Lyon	CSI5*-W presented by GL EVENTS round A	05.11.2017	CSI-W5*	3	1.40 - 1.60	420	27	12	01.01.2006	M	ISH	146	2	83	395	25	483	10	M	7	7a	yes	spread	oxer	straight	no	0	0	67,67	no
Lyon	CSI5*-W presented by GL EVENTS round A	05.11.2017	CSI-W5*	3	1.40 - 1.60	420	27	12	01.01.2006	M	ISH	146	2	83	395	25	483	10	M	8	7b	yes	vertical	vertical	straight	no	0	0	67,67	no
Lyon	CSI5*-W presented by GL EVENTS round A	05.11.2017	CSI-W5*	3	1.40 - 1.60	420	27	12	01.01.2006	M	ISH	146	2	83	395	25	483	10	M	9	7c	yes	spread	oxer	straight	no	0	0	67,67	no
Lyon	CSI5*-W presented by GL EVENTS round A	05.11.2017	CSI-W5*	3	1.40 - 1.60	420	27	12	01.01.2006	M	ISH	146	2	83	395	25	483	10	M	10	8	no	vertical	wall	right	no	0	0	67,67	no
Lyon	CSI5*-W presented by GL EVENTS round A	05.11.2017	CSI-W5*	3	1.40 - 1.60	420	27	12	01.01.2006	M	ISH	146	2	83	395	25	483	10	M	11	9	no	vertical	vertical	right	yes	4	0	67,67	no
Lyon	CSI5*-W presented by GL EVENTS round A	05.11.2017	CSI-W5*	3	1.40 - 1.60	420	27	12	01.01.2006	M	ISH	146	2	83	395	25	483	10	M	12	10	no	spread	oxer	right	no	0	0	67,67	no
Lyon	CSI5*-W presented by GL EVENTS round A	05.11.2017	CSI-W5*	3	1.40 - 1.60	420	27	12	01.01.2006	M	ISH	146	2	83	395	25	483	10	M	13	11	no	spread	oxer	left	no	0	0	67,67	no
Lyon	CSI5*-W presented by GL EVENTS round A	05.11.2017	CSI-W5*	3	1.40 - 1.60	420	27	12	01.01.2006	M	ISH	146	2	83	395	25	483	10	M	14	12a	yes	vertical	vertical	left	yes	4	0	67,67	no
Lyon	CSI5*-W presented by GL EVENTS round A	05.11.2017	CSI-W5*	3	1.40 - 1.60	420	27	12	01.01.2006	M	ISH	146	2	83	395	25	483	10	M	15	12b	yes	vertical	vertical with water	left	no	0	0	67,67	no
Lyon	CSI5*-W presented by GL EVENTS round A	05.11.2017	CSI-W5*	3	1.40 - 1.60	420	27	12	01.01.2006	M	ISH	146	2	83	395	25	483	10	M	16	13	no	spread	oxer with water	straight	no	0	0	67,67	no
Lyon	CSI5*-W presented by GL EVENTS round A	05.11.2017	CSI-W5*	3	1.40 - 1.60	420	187	12	04.06.2006	G	CH	180	7	84	158	36	1809	142	M	1	1	no	spread	oxer	right	no	0	0	67,44	no
Lyon	CSI5*-W presented by GL EVENTS round A	05.11.2017	CSI-W5*	3	1.40 - 1.60	420	187	12	04.06.2006	G	CH	180	7	84	158	36	1809	142	M	2	2	no	vertical	vertical	right	no	0	0	67,44	no
Lyon	CSI5*-W presented by GL EVENTS round A	05.11.2017	CSI-W5*	3	1.40 - 1.60	420	187	12	04.06.2006	G	CH	180	7	84	158	36	1809	142	M	3	3	no	spread	oxer	right	no	0	0	67,44	no
Lyon	CSI5*-W presented by GL EVENTS round A	05.11.2017	CSI-W5*	3	1.40 - 1.60	420	187	12	04.06.2006	G	CH	180	7	84	158	36	1809	142	M	4	4	no	spread	oxer	left	yes	4	0	67,44	no
Lyon	CSI5*-W presented by GL EVENTS round A	05.11.2017	CSI-W5*	3	1.40 - 1.60	420	187	12	04.06.2006	G	CH	180	7	84	158	36	1809	142	M	5	5	no	vertical	vertical	straight	no	0	0	67,44	no
Lyon	CSI5*-W presented by GL EVENTS round A	05.11.2017	CSI-W5*	3	1.40 - 1.60	420	187	12	04.06.2006	G	CH	180	7	84	158	36	1809	142	M	6	6	no	vertical	vertical	left	no	0	0	67,44	no
Lyon	CSI5*-W presented by GL EVENTS round A	05.11.2017	CSI-W5*	3	1.40 - 1.60	420	187	12	04.06.2006	G	CH	180	7	84	158	36	1809	142	M	7	7a	yes	spread	oxer	straight	no	0	0	67,44	no
Lyon	CSI5*-W presented by GL EVENTS round A	05.11.2017	CSI-W5*	3	1.40 - 1.60	420	187	12	04.06.2006	G	CH	180	7	84	158	36	1809	142	M	8	7b	yes	vertical	vertical	straight	no	0	0	67,44	no
Lyon	CSI5*-W presented by GL EVENTS round A	05.11.2017	CSI-W5*	3	1.40 - 1.60	420	187	12	04.06.2006	G	CH	180	7	84	158	36	1809	142	M	9	7c	yes	spread	oxer	straight	yes	4	0	67,44	no
Lyon	CSI5*-W presented by GL EVENTS round A	05.11.2017	CSI-W5*	3	1.40 - 1.60	420	187	12	04.06.2006	G	CH	180	7	84	158	36	1809	142	M	10	8	no	vertical	wall	right	no	0	0	67,44	no
Lyon	CSI5*-W presented by GL EVENTS round A	05.11.2017	CSI-W5*	3	1.40 - 1.60	420	187	12	04.06.2006	G	CH	180	7	84	158	36	1809	142	M	11	9	no	vertical	vertical	right	no	0	0	67,44	no
Lyon	CSI5*-W presented by GL EVENTS round A	05.11.2017	CSI-W5*	3	1.40 - 1.60	420	187	12	04.06.2006	G	CH	180	7	84	158	36	1809	142	M	12	10	no	spread	oxer	right	no	0	0	67,44	no
Lyon	CSI5*-W presented by GL EVENTS round A	05.11.2017	CSI-W5*	3	1.40 - 1.60	420	187	12	04.06.2006	G	CH	180	7	84	158	36	1809	142	M	13	11	no	spread	oxer	left	no	0	0	67,44	no
Lyon	CSI5*-W presented by GL EVENTS round A	05.11.2017	CSI-W5*	3	1.40 - 1.60	420	187	12	04.06.2006	G	CH	180	7	84	158	36	1809	142	M	14	12a	yes	vertical	vertical	left	no	0	0	67,44	no
Lyon	CSI5*-W presented by GL EVENTS round A	05.11.2017	CSI-W5*	3	1.40 - 1.60	420	187	12	04.06.2006	G	CH	180	7	84	158	36	1809	142	M	15	12b	yes	vertical	vertical with water	left	no	0	0	67,44	no
Lyon	CSI5*-W presented by GL EVENTS round A	05.11.2017	CSI-W5*	3	1.40 - 1.60	420	187	12	04.06.2006	G	CH	180	7	84	158	36	1809	142	M	16	13	no	spread	oxer with water	straight	no	0	0	67,44	no
Lyon	CSI5*-W presented by GL EVENTS round A	05.11.2017	CSI-W5*	3	1.40 - 1.60	420	131	10	28.03.2008	G	BWP	151	17	85	41	41	2052	191	M	1	1	no	spread	oxer	right	no	0	0	67,17	no
Lyon	CSI5*-W presented by GL EVENTS round A	05.11.2017	CSI-W5*	3	1.40 - 1.60	420	131	10	28.03.2008	G	BWP	151	17	85	41	41	2052	191	M	2	2	no	vertical	vertical	right	no	0	0	67,17	no
Lyon	CSI5*-W presented by GL EVENTS round A	05.11.2017	CSI-W5*	3	1.40 - 1.60	420	131	10	28.03.2008	G	BWP	151	17	85	41	41	2052	191	M	3	3	no	spread	oxer	right	no	0	0	67,17	no
Lyon	CSI5*-W presented by GL EVENTS round A	05.11.2017	CSI-W5*	3	1.40 - 1.60	420	131	10	28.03.2008	G	BWP	151	17	85	41	41	2052	191	M	4	4	no	spread	oxer	left	no	0	0	67,17	no
Lyon	CSI5*-W presented by GL EVENTS round A	05.11.2017	CSI-W5*	3	1.40 - 1.60	420	131	10	28.03.2008	G	BWP	151	17	85	41	41	2052	191	M	5	5	no	vertical	vertical	straight	no	0	0	67,17	no
Lyon	CSI5*-W presented by GL EVENTS round A	05.11.2017	CSI-W5*	3	1.40 - 1.60	420	131	10	28.03.2008	G	BWP	151	17	85	41	41	2052	191	M	6	6	no	vertical	vertical	left	no	0	0	67,17	no
Lyon	CSI5*-W presented by GL EVENTS round A	05.11.2017	CSI-W5*	3	1.40 - 1.60	420	131	10	28.03.2008	G	BWP	151	17	85	41	41	2052	191	M	7	7a	yes	spread	oxer	straight	no	0	0	67,17	no
Lyon	CSI5*-W presented by GL EVENTS round A	05.11.2017	CSI-W5*	3	1.40 - 1.60	420	131	10	28.03.2008	G	BWP	151	17	85	41	41	2052	191	M	8	7b	yes	vertical	vertical	straight	no	0	0	67,17	no
Lyon	CSI5*-W presented by GL EVENTS round A	05.11.2017	CSI-W5*	3	1.40 - 1.60	420	131	10	28.03.2008	G	BWP	151	17	85	41	41	2052	191	M	9	7c	yes	spread	oxer	straight	no	0	0	67,17	no
Lyon	CSI5*-W presented by GL EVENTS round A	05.11.2017	CSI-W5*	3	1.40 - 1.60	420	131	10	28.03.2008	G	BWP	151	17	85	41	41	2052	191	M	10	8	no	vertical	wall	right	no	0	0	67,17	no
Lyon	CSI5*-W presented by GL EVENTS round A	05.11.2017	CSI-W5*	3	1.40 - 1.60	420	131	10	28.03.2008	G	BWP	151	17	85	41	41	2052	191	M	11	9	no	vertical	vertical	right	no	0	0	67,17	no
Lyon	CSI5*-W presented by GL EVENTS round A	05.11.2017	CSI-W5*	3	1.40 - 1.60	420	131	10	28.03.2008	G	BWP	151	17	85	41	41	2052	191	M	12	10	no	spread	oxer	right	no	0	0	67,17	no
Lyon	CSI5*-W presented by GL EVENTS round A	05.11.2017	CSI-W5*	3	1.40 - 1.60	420	131	10	28.03.2008	G	BWP	151	17	85	41	41	2052	191	M	13	11	no	spread	oxer	left	no	0	0	67,17	no
Lyon	CSI5*-W presented by GL EVENTS round A	05.11.2017	CSI-W5*	3	1.40 - 1.60	420	131	10	28.03.2008	G	BWP	151	17	85	41	41	2052	191	M	14	12a	yes	vertical	vertical	left	no	0	0	67,17	no
Lyon	CSI5*-W presented by GL EVENTS round A	05.11.2017	CSI-W5*	3	1.40 - 1.60	420	131	10	28.03.2008	G	BWP	151	17	85	41	41	2052	191	M	15	12b	yes	vertical	vertical with water	left	no	0	0	67,17	no
Lyon	CSI5*-W presented by GL EVENTS round A	05.11.2017	CSI-W5*	3	1.40 - 1.60	420	131	10	28.03.2008	G	BWP	151	17	85	41	41	2052	191	M	16	13	no	spread	oxer with water	straight	no	0	0	67,17	no
Lyon	CSI5*-W presented by GL EVENTS round B	05.11.2017	CSI-W5*	3	1.40 - 1.60	280	131	10	28.03.2008	G	BWP	151	17	85	41	41	2052	191	M	1	1	no	vertical	vertical	left	no	0	0	38,78	no
Lyon	CSI5*-W presented by GL EVENTS round B	05.11.2017	CSI-W5*	3	1.40 - 1.60	280	131	10	28.03.2008	G	BWP	151	17	85	41	41	2052	191	M	2	2	no	vertical	vertical	left	no	0	0	38,78	no
Lyon	CSI5*-W presented by GL EVENTS round B	05.11.2017	CSI-W5*	3	1.40 - 1.60	280	131	10	28.03.2008	G	BWP	151	17	85	41	41	2052	191	M	3	3	no	spread	oxer	right	no	0	0	38,78	no
Lyon	CSI5*-W presented by GL EVENTS round B	05.11.2017	CSI-W5*	3	1.40 - 1.60	280	131	10	28.03.2008	G	BWP	151	17	85	41	41	2052	191	M	4	4	no	spread	oxer	left	no	0	0	38,78	no
Lyon	CSI5*-W presented by GL EVENTS round B	05.11.2017	CSI-W5*	3	1.40 - 1.60	280	131	10	28.03.2008	G	BWP	151	17	85	41	41	2052	191	M	5	5a	yes	vertical	vertical	straight	no	0	0	38,78	no
Lyon	CSI5*-W presented by GL EVENTS round B	05.11.2017	CSI-W5*	3	1.40 - 1.60	280	131	10	28.03.2008	G	BWP	151	17	85	41	41	2052	191	M	6	5b	yes	vertical	vertical with water	straight	no	0	0	38,78	no
Lyon	CSI5*-W presented by GL EVENTS round B	05.11.2017	CSI-W5*	3	1.40 - 1.60	280	131	10	28.03.2008	G	BWP	151	17	85	41	41	2052	191	M	7	6	no	spread	oxer	left	yes	4	0	38,78	no
Lyon	CSI5*-W presented by GL EVENTS round B	05.11.2017	CSI-W5*	3	1.40 - 1.60	280	131	10	28.03.2008	G	BWP	151	17	85	41	41	2052	191	M	8	7	no	spread	oxer	right	no	0	0	38,78	no
Lyon	CSI5*-W presented by GL EVENTS round A	05.11.2017	CSI-W5*	3	1.40 - 1.60	420	90	14	01.03.2004	M	HANN	185	6	86	36	48	1157	25	F	1	1	no	spread	oxer	right	no	0	0	67,11	no
Lyon	CSI5*-W presented by GL EVENTS round A	05.11.2017	CSI-W5*	3	1.40 - 1.60	420	90	14	01.03.2004	M	HANN	185	6	86	36	48	1157	25	F	2	2	no	vertical	vertical	right	no	0	0	67,11	no
Lyon	CSI5*-W presented by GL EVENTS round A	05.11.2017	CSI-W5*	3	1.40 - 1.60	420	90	14	01.03.2004	M	HANN	185	6	86	36	48	1157	25	F	3	3	no	spread	oxer	right	no	0	0	67,11	no
Lyon	CSI5*-W presented by GL EVENTS round A	05.11.2017	CSI-W5*	3	1.40 - 1.60	420	90	14	01.03.2004	M	HANN	185	6	86	36	48	1157	25	F	4	4	no	spread	oxer	left	no	0	0	67,11	no
Lyon	CSI5*-W presented by GL EVENTS round A	05.11.2017	CSI-W5*	3	1.40 - 1.60	420	90	14	01.03.2004	M	HANN	185	6	86	36	48	1157	25	F	5	5	no	vertical	vertical	straight	no	0	0	67,11	no
Lyon	CSI5*-W presented by GL EVENTS round A	05.11.2017	CSI-W5*	3	1.40 - 1.60	420	90	14	01.03.2004	M	HANN	185	6	86	36	48	1157	25	F	6	6	no	vertical	vertical	left	no	0	0	67,11	no
Lyon	CSI5*-W presented by GL EVENTS round A	05.11.2017	CSI-W5*	3	1.40 - 1.60	420	90	14	01.03.2004	M	HANN	185	6	86	36	48	1157	25	F	7	7a	yes	spread	oxer	straight	no	0	0	67,11	no
Lyon	CSI5*-W presented by GL EVENTS round A	05.11.2017	CSI-W5*	3	1.40 - 1.60	420	90	14	01.03.2004	M	HANN	185	6	86	36	48	1157	25	F	8	7b	yes	vertical	vertical	straight	no	0	0	67,11	no
Lyon	CSI5*-W presented by GL EVENTS round A	05.11.2017	CSI-W5*	3	1.40 - 1.60	420	90	14	01.03.2004	M	HANN	185	6	86	36	48	1157	25	F	9	7c	yes	spread	oxer	straight	no	0	0	67,11	no
Lyon	CSI5*-W presented by GL EVENTS round A	05.11.2017	CSI-W5*	3	1.40 - 1.60	420	90	14	01.03.2004	M	HANN	185	6	86	36	48	1157	25	F	10	8	no	vertical	wall	right	no	0	0	67,11	no
Lyon	CSI5*-W presented by GL EVENTS round A	05.11.2017	CSI-W5*	3	1.40 - 1.60	420	90	14	01.03.2004	M	HANN	185	6	86	36	48	1157	25	F	11	9	no	vertical	vertical	right	no	0	0	67,11	no
Lyon	CSI5*-W presented by GL EVENTS round A	05.11.2017	CSI-W5*	3	1.40 - 1.60	420	90	14	01.03.2004	M	HANN	185	6	86	36	48	1157	25	F	12	10	no	spread	oxer	right	no	0	0	67,11	no
Lyon	CSI5*-W presented by GL EVENTS round A	05.11.2017	CSI-W5*	3	1.40 - 1.60	420	90	14	01.03.2004	M	HANN	185	6	86	36	48	1157	25	F	13	11	no	spread	oxer	left	no	0	0	67,11	no
Lyon	CSI5*-W presented by GL EVENTS round A	05.11.2017	CSI-W5*	3	1.40 - 1.60	420	90	14	01.03.2004	M	HANN	185	6	86	36	48	1157	25	F	14	12a	yes	vertical	vertical	left	no	0	0	67,11	no
Lyon	CSI5*-W presented by GL EVENTS round A	05.11.2017	CSI-W5*	3	1.40 - 1.60	420	90	14	01.03.2004	M	HANN	185	6	86	36	48	1157	25	F	15	12b	yes	vertical	vertical with water	left	no	0	0	67,11	no
Lyon	CSI5*-W presented by GL EVENTS round A	05.11.2017	CSI-W5*	3	1.40 - 1.60	420	90	14	01.03.2004	M	HANN	185	6	86	36	48	1157	25	F	16	13	no	spread	oxer with water	straight	yes	4	0	67,11	no
Lyon	CSI5*-W presented by GL EVENTS round A	05.11.2017	CSI-W5*	3	1.40 - 1.60	420	18	14	27.04.2004	G	HANN	154	4	87	199	55	1406	43	M	1	1	no	spread	oxer	right	no	0	0	66,77	no
Lyon	CSI5*-W presented by GL EVENTS round A	05.11.2017	CSI-W5*	3	1.40 - 1.60	420	18	14	27.04.2004	G	HANN	154	4	87	199	55	1406	43	M	2	2	no	vertical	vertical	right	no	0	0	66,77	no
Lyon	CSI5*-W presented by GL EVENTS round A	05.11.2017	CSI-W5*	3	1.40 - 1.60	420	18	14	27.04.2004	G	HANN	154	4	87	199	55	1406	43	M	3	3	no	spread	oxer	right	no	0	0	66,77	no
Lyon	CSI5*-W presented by GL EVENTS round A	05.11.2017	CSI-W5*	3	1.40 - 1.60	420	18	14	27.04.2004	G	HANN	154	4	87	199	55	1406	43	M	4	4	no	spread	oxer	left	no	0	0	66,77	no
Lyon	CSI5*-W presented by GL EVENTS round A	05.11.2017	CSI-W5*	3	1.40 - 1.60	420	18	14	27.04.2004	G	HANN	154	4	87	199	55	1406	43	M	5	5	no	vertical	vertical	straight	no	0	0	66,77	no
Lyon	CSI5*-W presented by GL EVENTS round A	05.11.2017	CSI-W5*	3	1.40 - 1.60	420	18	14	27.04.2004	G	HANN	154	4	87	199	55	1406	43	M	6	6	no	vertical	vertical	left	no	0	0	66,77	no
Lyon	CSI5*-W presented by GL EVENTS round A	05.11.2017	CSI-W5*	3	1.40 - 1.60	420	18	14	27.04.2004	G	HANN	154	4	87	199	55	1406	43	M	7	7a	yes	spread	oxer	straight	no	0	0	66,77	no
Lyon	CSI5*-W presented by GL EVENTS round A	05.11.2017	CSI-W5*	3	1.40 - 1.60	420	18	14	27.04.2004	G	HANN	154	4	87	199	55	1406	43	M	8	7b	yes	vertical	vertical	straight	no	0	0	66,77	no
Lyon	CSI5*-W presented by GL EVENTS round A	05.11.2017	CSI-W5*	3	1.40 - 1.60	420	18	14	27.04.2004	G	HANN	154	4	87	199	55	1406	43	M	9	7c	yes	spread	oxer	straight	no	0	0	66,77	no
Lyon	CSI5*-W presented by GL EVENTS round A	05.11.2017	CSI-W5*	3	1.40 - 1.60	420	18	14	27.04.2004	G	HANN	154	4	87	199	55	1406	43	M	10	8	no	vertical	wall	right	no	0	0	66,77	no
Lyon	CSI5*-W presented by GL EVENTS round A	05.11.2017	CSI-W5*	3	1.40 - 1.60	420	18	14	27.04.2004	G	HANN	154	4	87	199	55	1406	43	M	11	9	no	vertical	vertical	right	no	0	0	66,77	no
Lyon	CSI5*-W presented by GL EVENTS round A	05.11.2017	CSI-W5*	3	1.40 - 1.60	420	18	14	27.04.2004	G	HANN	154	4	87	199	55	1406	43	M	12	10	no	spread	oxer	right	no	0	0	66,77	no
Lyon	CSI5*-W presented by GL EVENTS round A	05.11.2017	CSI-W5*	3	1.40 - 1.60	420	18	14	27.04.2004	G	HANN	154	4	87	199	55	1406	43	M	13	11	no	spread	oxer	left	no	0	0	66,77	no
Lyon	CSI5*-W presented by GL EVENTS round A	05.11.2017	CSI-W5*	3	1.40 - 1.60	420	18	14	27.04.2004	G	HANN	154	4	87	199	55	1406	43	M	14	12a	yes	vertical	vertical	left	no	0	0	66,77	no
Lyon	CSI5*-W presented by GL EVENTS round A	05.11.2017	CSI-W5*	3	1.40 - 1.60	420	18	14	27.04.2004	G	HANN	154	4	87	199	55	1406	43	M	15	12b	yes	vertical	vertical with water	left	no	0	0	66,77	no
Lyon	CSI5*-W presented by GL EVENTS round A	05.11.2017	CSI-W5*	3	1.40 - 1.60	420	18	14	27.04.2004	G	HANN	154	4	87	199	55	1406	43	M	16	13	no	spread	oxer with water	straight	no	0	0	66,77	no
Lyon	CSI5*-W presented by GL EVENTS round B	05.11.2017	CSI-W5*	3	1.40 - 1.60	280	18	14	27.04.2004	G	HANN	154	4	87	199	55	1406	43	M	1	1	no	vertical	vertical	left	no	0	0	42,33	no
Lyon	CSI5*-W presented by GL EVENTS round B	05.11.2017	CSI-W5*	3	1.40 - 1.60	280	18	14	27.04.2004	G	HANN	154	4	87	199	55	1406	43	M	2	2	no	vertical	vertical	left	no	0	0	42,33	no
Lyon	CSI5*-W presented by GL EVENTS round B	05.11.2017	CSI-W5*	3	1.40 - 1.60	280	18	14	27.04.2004	G	HANN	154	4	87	199	55	1406	43	M	3	3	no	spread	oxer	right	no	0	0	42,33	no
Lyon	CSI5*-W presented by GL EVENTS round B	05.11.2017	CSI-W5*	3	1.40 - 1.60	280	18	14	27.04.2004	G	HANN	154	4	87	199	55	1406	43	M	4	4	no	spread	oxer	left	no	0	0	42,33	no
Lyon	CSI5*-W presented by GL EVENTS round B	05.11.2017	CSI-W5*	3	1.40 - 1.60	280	18	14	27.04.2004	G	HANN	154	4	87	199	55	1406	43	M	5	5a	yes	vertical	vertical	straight	yes	4	0	42,33	no
Lyon	CSI5*-W presented by GL EVENTS round B	05.11.2017	CSI-W5*	3	1.40 - 1.60	280	18	14	27.04.2004	G	HANN	154	4	87	199	55	1406	43	M	6	5b	yes	vertical	vertical with water	straight	no	0	0	42,33	no
Lyon	CSI5*-W presented by GL EVENTS round B	05.11.2017	CSI-W5*	3	1.40 - 1.60	280	18	14	27.04.2004	G	HANN	154	4	87	199	55	1406	43	M	7	6	no	spread	oxer	left	no	0	0	42,33	no
Lyon	CSI5*-W presented by GL EVENTS round B	05.11.2017	CSI-W5*	3	1.40 - 1.60	280	18	14	27.04.2004	G	HANN	154	4	87	199	55	1406	43	M	8	7	no	spread	oxer	right	no	0	0	42,33	no
Lyon	CSI5*-W presented by GL EVENTS round A	05.11.2017	CSI-W5*	3	1.40 - 1.60	420	161	14	05.04.2004	M	SWB	265	26	88	33	41	1434	57	F	1	1	no	spread	oxer	right	no	0	0	65,38	no
Lyon	CSI5*-W presented by GL EVENTS round A	05.11.2017	CSI-W5*	3	1.40 - 1.60	420	161	14	05.04.2004	M	SWB	265	26	88	33	41	1434	57	F	2	2	no	vertical	vertical	right	no	0	0	65,38	no
Lyon	CSI5*-W presented by GL EVENTS round A	05.11.2017	CSI-W5*	3	1.40 - 1.60	420	161	14	05.04.2004	M	SWB	265	26	88	33	41	1434	57	F	3	3	no	spread	oxer	right	no	0	0	65,38	no
Lyon	CSI5*-W presented by GL EVENTS round A	05.11.2017	CSI-W5*	3	1.40 - 1.60	420	161	14	05.04.2004	M	SWB	265	26	88	33	41	1434	57	F	4	4	no	spread	oxer	left	no	0	0	65,38	no
Lyon	CSI5*-W presented by GL EVENTS round A	05.11.2017	CSI-W5*	3	1.40 - 1.60	420	161	14	05.04.2004	M	SWB	265	26	88	33	41	1434	57	F	5	5	no	vertical	vertical	straight	no	0	0	65,38	no
Lyon	CSI5*-W presented by GL EVENTS round A	05.11.2017	CSI-W5*	3	1.40 - 1.60	420	161	14	05.04.2004	M	SWB	265	26	88	33	41	1434	57	F	6	6	no	vertical	vertical	left	no	0	0	65,38	no
Lyon	CSI5*-W presented by GL EVENTS round A	05.11.2017	CSI-W5*	3	1.40 - 1.60	420	161	14	05.04.2004	M	SWB	265	26	88	33	41	1434	57	F	7	7a	yes	spread	oxer	straight	no	0	0	65,38	no
Lyon	CSI5*-W presented by GL EVENTS round A	05.11.2017	CSI-W5*	3	1.40 - 1.60	420	161	14	05.04.2004	M	SWB	265	26	88	33	41	1434	57	F	8	7b	yes	vertical	vertical	straight	no	0	0	65,38	no
Lyon	CSI5*-W presented by GL EVENTS round A	05.11.2017	CSI-W5*	3	1.40 - 1.60	420	161	14	05.04.2004	M	SWB	265	26	88	33	41	1434	57	F	9	7c	yes	spread	oxer	straight	yes	4	0	65,38	no
Lyon	CSI5*-W presented by GL EVENTS round A	05.11.2017	CSI-W5*	3	1.40 - 1.60	420	161	14	05.04.2004	M	SWB	265	26	88	33	41	1434	57	F	10	8	no	vertical	wall	right	no	0	0	65,38	no
Lyon	CSI5*-W presented by GL EVENTS round A	05.11.2017	CSI-W5*	3	1.40 - 1.60	420	161	14	05.04.2004	M	SWB	265	26	88	33	41	1434	57	F	11	9	no	vertical	vertical	right	no	0	0	65,38	no
Lyon	CSI5*-W presented by GL EVENTS round A	05.11.2017	CSI-W5*	3	1.40 - 1.60	420	161	14	05.04.2004	M	SWB	265	26	88	33	41	1434	57	F	12	10	no	spread	oxer	right	no	0	0	65,38	no
Lyon	CSI5*-W presented by GL EVENTS round A	05.11.2017	CSI-W5*	3	1.40 - 1.60	420	161	14	05.04.2004	M	SWB	265	26	88	33	41	1434	57	F	13	11	no	spread	oxer	left	no	0	0	65,38	no
Lyon	CSI5*-W presented by GL EVENTS round A	05.11.2017	CSI-W5*	3	1.40 - 1.60	420	161	14	05.04.2004	M	SWB	265	26	88	33	41	1434	57	F	14	12a	yes	vertical	vertical	left	no	0	0	65,38	no
Lyon	CSI5*-W presented by GL EVENTS round A	05.11.2017	CSI-W5*	3	1.40 - 1.60	420	161	14	05.04.2004	M	SWB	265	26	88	33	41	1434	57	F	15	12b	yes	vertical	vertical with water	left	no	0	0	65,38	no
Lyon	CSI5*-W presented by GL EVENTS round A	05.11.2017	CSI-W5*	3	1.40 - 1.60	420	161	14	05.04.2004	M	SWB	265	26	88	33	41	1434	57	F	16	13	no	spread	oxer with water	straight	no	0	0	65,38	no
Lyon	CSI5*-W presented by GL EVENTS round A	05.11.2017	CSI-W5*	3	1.40 - 1.60	420	149	16	28.03.2002	M	ISH	246	21	89	245	49	1356	59	M	1	1	no	spread	oxer	right	no	0	0	64,7	no
Lyon	CSI5*-W presented by GL EVENTS round A	05.11.2017	CSI-W5*	3	1.40 - 1.60	420	149	16	28.03.2002	M	ISH	246	21	89	245	49	1356	59	M	2	2	no	vertical	vertical	right	no	0	0	64,7	no
Lyon	CSI5*-W presented by GL EVENTS round A	05.11.2017	CSI-W5*	3	1.40 - 1.60	420	149	16	28.03.2002	M	ISH	246	21	89	245	49	1356	59	M	3	3	no	spread	oxer	right	no	0	0	64,7	no
Lyon	CSI5*-W presented by GL EVENTS round A	05.11.2017	CSI-W5*	3	1.40 - 1.60	420	149	16	28.03.2002	M	ISH	246	21	89	245	49	1356	59	M	4	4	no	spread	oxer	left	no	0	0	64,7	no
Lyon	CSI5*-W presented by GL EVENTS round A	05.11.2017	CSI-W5*	3	1.40 - 1.60	420	149	16	28.03.2002	M	ISH	246	21	89	245	49	1356	59	M	5	5	no	vertical	vertical	straight	no	0	0	64,7	no
Lyon	CSI5*-W presented by GL EVENTS round A	05.11.2017	CSI-W5*	3	1.40 - 1.60	420	149	16	28.03.2002	M	ISH	246	21	89	245	49	1356	59	M	6	6	no	vertical	vertical	left	no	0	0	64,7	no
Lyon	CSI5*-W presented by GL EVENTS round A	05.11.2017	CSI-W5*	3	1.40 - 1.60	420	149	16	28.03.2002	M	ISH	246	21	89	245	49	1356	59	M	7	7a	yes	spread	oxer	straight	yes	4	0	64,7	no
Lyon	CSI5*-W presented by GL EVENTS round A	05.11.2017	CSI-W5*	3	1.40 - 1.60	420	149	16	28.03.2002	M	ISH	246	21	89	245	49	1356	59	M	8	7b	yes	vertical	vertical	straight	yes	4	0	64,7	no
Lyon	CSI5*-W presented by GL EVENTS round A	05.11.2017	CSI-W5*	3	1.40 - 1.60	420	149	16	28.03.2002	M	ISH	246	21	89	245	49	1356	59	M	9	7c	yes	spread	oxer	straight	no	0	0	64,7	no
Lyon	CSI5*-W presented by GL EVENTS round A	05.11.2017	CSI-W5*	3	1.40 - 1.60	420	149	16	28.03.2002	M	ISH	246	21	89	245	49	1356	59	M	10	8	no	vertical	wall	right	no	0	0	64,7	no
Lyon	CSI5*-W presented by GL EVENTS round A	05.11.2017	CSI-W5*	3	1.40 - 1.60	420	149	16	28.03.2002	M	ISH	246	21	89	245	49	1356	59	M	11	9	no	vertical	vertical	right	no	0	0	64,7	no
Lyon	CSI5*-W presented by GL EVENTS round A	05.11.2017	CSI-W5*	3	1.40 - 1.60	420	149	16	28.03.2002	M	ISH	246	21	89	245	49	1356	59	M	12	10	no	spread	oxer	right	no	0	0	64,7	no
Lyon	CSI5*-W presented by GL EVENTS round A	05.11.2017	CSI-W5*	3	1.40 - 1.60	420	149	16	28.03.2002	M	ISH	246	21	89	245	49	1356	59	M	13	11	no	spread	oxer	left	no	0	0	64,7	no
Lyon	CSI5*-W presented by GL EVENTS round A	05.11.2017	CSI-W5*	3	1.40 - 1.60	420	149	16	28.03.2002	M	ISH	246	21	89	245	49	1356	59	M	14	12a	yes	vertical	vertical	left	no	0	0	64,7	no
Lyon	CSI5*-W presented by GL EVENTS round A	05.11.2017	CSI-W5*	3	1.40 - 1.60	420	149	16	28.03.2002	M	ISH	246	21	89	245	49	1356	59	M	15	12b	yes	vertical	vertical with water	left	no	0	0	64,7	no
Lyon	CSI5*-W presented by GL EVENTS round A	05.11.2017	CSI-W5*	3	1.40 - 1.60	420	149	16	28.03.2002	M	ISH	246	21	89	245	49	1356	59	M	16	13	no	spread	oxer with water	straight	no	0	0	64,7	no
Stuttgart	Grand Prix of Stuttgart presented by Mercedes-Benz, WALTER solar	19.11.2017	CSI-W5*	4	1.60	410	159	11	15.04.2007	M	PZHK	161	4	11	414	32	1331	18	F	1	1	no	vertical	wall	right	no	0	0	66,79	no
Stuttgart	Grand Prix of Stuttgart presented by Mercedes-Benz, WALTER solar	19.11.2017	CSI-W5*	4	1.60	410	159	11	15.04.2007	M	PZHK	161	4	11	414	32	1331	18	F	2	2	no	spread	oxer	right	no	0	0	66,79	no
Stuttgart	Grand Prix of Stuttgart presented by Mercedes-Benz, WALTER solar	19.11.2017	CSI-W5*	4	1.60	410	159	11	15.04.2007	M	PZHK	161	4	11	414	32	1331	18	F	3	3	no	vertical	vertical	right	no	0	0	66,79	no
Stuttgart	Grand Prix of Stuttgart presented by Mercedes-Benz, WALTER solar	19.11.2017	CSI-W5*	4	1.60	410	159	11	15.04.2007	M	PZHK	161	4	11	414	32	1331	18	F	4	4	no	spread	oxer	straight	no	0	0	66,79	no
Stuttgart	Grand Prix of Stuttgart presented by Mercedes-Benz, WALTER solar	19.11.2017	CSI-W5*	4	1.60	410	159	11	15.04.2007	M	PZHK	161	4	11	414	32	1331	18	F	5	5	no	spread	oxer	straight	no	0	0	66,79	no
Stuttgart	Grand Prix of Stuttgart presented by Mercedes-Benz, WALTER solar	19.11.2017	CSI-W5*	4	1.60	410	159	11	15.04.2007	M	PZHK	161	4	11	414	32	1331	18	F	6	6	no	vertical	vertical	left	no	0	0	66,79	no
Stuttgart	Grand Prix of Stuttgart presented by Mercedes-Benz, WALTER solar	19.11.2017	CSI-W5*	4	1.60	410	159	11	15.04.2007	M	PZHK	161	4	11	414	32	1331	18	F	7	7a	yes	spread	oxer	left	yes	4	0	66,79	no
Stuttgart	Grand Prix of Stuttgart presented by Mercedes-Benz, WALTER solar	19.11.2017	CSI-W5*	4	1.60	410	159	11	15.04.2007	M	PZHK	161	4	11	414	32	1331	18	F	8	7b	yes	vertical	vertical	left	no	0	0	66,79	no
Stuttgart	Grand Prix of Stuttgart presented by Mercedes-Benz, WALTER solar	19.11.2017	CSI-W5*	4	1.60	410	159	11	15.04.2007	M	PZHK	161	4	11	414	32	1331	18	F	9	7c	yes	spread	oxer	left	yes	4	0	66,79	no
Stuttgart	Grand Prix of Stuttgart presented by Mercedes-Benz, WALTER solar	19.11.2017	CSI-W5*	4	1.60	410	159	11	15.04.2007	M	PZHK	161	4	11	414	32	1331	18	F	10	8	no	spread	oxer	left	no	0	0	66,79	no
Stuttgart	Grand Prix of Stuttgart presented by Mercedes-Benz, WALTER solar	19.11.2017	CSI-W5*	4	1.60	410	159	11	15.04.2007	M	PZHK	161	4	11	414	32	1331	18	F	11	9	no	vertical	vertical	left	no	0	0	66,79	no
Stuttgart	Grand Prix of Stuttgart presented by Mercedes-Benz, WALTER solar	19.11.2017	CSI-W5*	4	1.60	410	159	11	15.04.2007	M	PZHK	161	4	11	414	32	1331	18	F	12	10	no	spread	oxer	right	no	0	0	66,79	no
Stuttgart	Grand Prix of Stuttgart presented by Mercedes-Benz, WALTER solar	19.11.2017	CSI-W5*	4	1.60	410	159	11	15.04.2007	M	PZHK	161	4	11	414	32	1331	18	F	13	11	no	vertical	vertical	right	yes	4	0	66,79	no
Stuttgart	Grand Prix of Stuttgart presented by Mercedes-Benz, WALTER solar	19.11.2017	CSI-W5*	4	1.60	410	159	11	15.04.2007	M	PZHK	161	4	11	414	32	1331	18	F	14	12a	yes	vertical	vertical	right	no	0	0	66,79	no
Stuttgart	Grand Prix of Stuttgart presented by Mercedes-Benz, WALTER solar	19.11.2017	CSI-W5*	4	1.60	410	159	11	15.04.2007	M	PZHK	161	4	11	414	32	1331	18	F	15	12b	yes	spread	oxer with water	right	no	0	0	66,79	no
Stuttgart	Grand Prix of Stuttgart presented by Mercedes-Benz, WALTER solar	19.11.2017	CSI-W5*	4	1.60	410	159	11	15.04.2007	M	PZHK	161	4	11	414	32	1331	18	F	16	13	no	spread	oxer with water	straight	no	0	0	66,79	no
Stuttgart	Grand Prix of Stuttgart presented by Mercedes-Benz, WALTER solar	19.11.2017	CSI-W5*	4	1.60	410	212	12	10.04.2006	S	BWP	134	11	13	42	49	1996	131	M	1	1	no	vertical	wall	right	no	0	0	69,62	no
Stuttgart	Grand Prix of Stuttgart presented by Mercedes-Benz, WALTER solar	19.11.2017	CSI-W5*	4	1.60	410	212	12	10.04.2006	S	BWP	134	11	13	42	49	1996	131	M	2	2	no	spread	oxer	right	no	0	0	69,62	no
Stuttgart	Grand Prix of Stuttgart presented by Mercedes-Benz, WALTER solar	19.11.2017	CSI-W5*	4	1.60	410	212	12	10.04.2006	S	BWP	134	11	13	42	49	1996	131	M	3	3	no	vertical	vertical	right	no	0	0	69,62	no
Stuttgart	Grand Prix of Stuttgart presented by Mercedes-Benz, WALTER solar	19.11.2017	CSI-W5*	4	1.60	410	212	12	10.04.2006	S	BWP	134	11	13	42	49	1996	131	M	4	4	no	spread	oxer	straight	no	0	0	69,62	no
Stuttgart	Grand Prix of Stuttgart presented by Mercedes-Benz, WALTER solar	19.11.2017	CSI-W5*	4	1.60	410	212	12	10.04.2006	S	BWP	134	11	13	42	49	1996	131	M	5	5	no	spread	oxer	straight	no	0	0	69,62	no
Stuttgart	Grand Prix of Stuttgart presented by Mercedes-Benz, WALTER solar	19.11.2017	CSI-W5*	4	1.60	410	212	12	10.04.2006	S	BWP	134	11	13	42	49	1996	131	M	6	6	no	vertical	vertical	left	no	0	0	69,62	no
Stuttgart	Grand Prix of Stuttgart presented by Mercedes-Benz, WALTER solar	19.11.2017	CSI-W5*	4	1.60	410	212	12	10.04.2006	S	BWP	134	11	13	42	49	1996	131	M	7	7a	yes	spread	oxer	left	no	0	0	69,62	no
Stuttgart	Grand Prix of Stuttgart presented by Mercedes-Benz, WALTER solar	19.11.2017	CSI-W5*	4	1.60	410	212	12	10.04.2006	S	BWP	134	11	13	42	49	1996	131	M	8	7b	yes	vertical	vertical	left	no	0	0	69,62	no
Stuttgart	Grand Prix of Stuttgart presented by Mercedes-Benz, WALTER solar	19.11.2017	CSI-W5*	4	1.60	410	212	12	10.04.2006	S	BWP	134	11	13	42	49	1996	131	M	9	7c	yes	spread	oxer	left	no	0	0	69,62	no
Stuttgart	Grand Prix of Stuttgart presented by Mercedes-Benz, WALTER solar	19.11.2017	CSI-W5*	4	1.60	410	212	12	10.04.2006	S	BWP	134	11	13	42	49	1996	131	M	10	8	no	spread	oxer	left	no	0	0	69,62	no
Stuttgart	Grand Prix of Stuttgart presented by Mercedes-Benz, WALTER solar	19.11.2017	CSI-W5*	4	1.60	410	212	12	10.04.2006	S	BWP	134	11	13	42	49	1996	131	M	11	9	no	vertical	vertical	left	yes	4	0	69,62	no
Stuttgart	Grand Prix of Stuttgart presented by Mercedes-Benz, WALTER solar	19.11.2017	CSI-W5*	4	1.60	410	212	12	10.04.2006	S	BWP	134	11	13	42	49	1996	131	M	12	10	no	spread	oxer	right	no	0	0	69,62	no
Stuttgart	Grand Prix of Stuttgart presented by Mercedes-Benz, WALTER solar	19.11.2017	CSI-W5*	4	1.60	410	212	12	10.04.2006	S	BWP	134	11	13	42	49	1996	131	M	13	11	no	vertical	vertical	right	no	0	0	69,62	no
Stuttgart	Grand Prix of Stuttgart presented by Mercedes-Benz, WALTER solar	19.11.2017	CSI-W5*	4	1.60	410	212	12	10.04.2006	S	BWP	134	11	13	42	49	1996	131	M	14	12a	yes	vertical	vertical	right	yes	4	0	69,62	no
Stuttgart	Grand Prix of Stuttgart presented by Mercedes-Benz, WALTER solar	19.11.2017	CSI-W5*	4	1.60	410	212	12	10.04.2006	S	BWP	134	11	13	42	49	1996	131	M	15	12b	yes	spread	oxer with water	right	no	0	0	69,62	no
Stuttgart	Grand Prix of Stuttgart presented by Mercedes-Benz, WALTER solar	19.11.2017	CSI-W5*	4	1.60	410	212	12	10.04.2006	S	BWP	134	11	13	42	49	1996	131	M	16	13	no	spread	oxer with water	straight	yes	4	0	69,62	no
Stuttgart	Grand Prix of Stuttgart presented by Mercedes-Benz, WALTER solar	19.11.2017	CSI-W5*	4	1.60	410	119	13	06.05.2005	S	OS	194	6	14	5	37	1868	96	M	1	1	no	vertical	wall	right	no	0	0	67,78	no
Stuttgart	Grand Prix of Stuttgart presented by Mercedes-Benz, WALTER solar	19.11.2017	CSI-W5*	4	1.60	410	119	13	06.05.2005	S	OS	194	6	14	5	37	1868	96	M	2	2	no	spread	oxer	right	no	0	0	67,78	no
Stuttgart	Grand Prix of Stuttgart presented by Mercedes-Benz, WALTER solar	19.11.2017	CSI-W5*	4	1.60	410	119	13	06.05.2005	S	OS	194	6	14	5	37	1868	96	M	3	3	no	vertical	vertical	right	no	0	0	67,78	no
Stuttgart	Grand Prix of Stuttgart presented by Mercedes-Benz, WALTER solar	19.11.2017	CSI-W5*	4	1.60	410	119	13	06.05.2005	S	OS	194	6	14	5	37	1868	96	M	4	4	no	spread	oxer	straight	no	0	0	67,78	no
Stuttgart	Grand Prix of Stuttgart presented by Mercedes-Benz, WALTER solar	19.11.2017	CSI-W5*	4	1.60	410	119	13	06.05.2005	S	OS	194	6	14	5	37	1868	96	M	5	5	no	spread	oxer	straight	no	0	0	67,78	no
Stuttgart	Grand Prix of Stuttgart presented by Mercedes-Benz, WALTER solar	19.11.2017	CSI-W5*	4	1.60	410	119	13	06.05.2005	S	OS	194	6	14	5	37	1868	96	M	6	6	no	vertical	vertical	left	no	0	0	67,78	no
Stuttgart	Grand Prix of Stuttgart presented by Mercedes-Benz, WALTER solar	19.11.2017	CSI-W5*	4	1.60	410	119	13	06.05.2005	S	OS	194	6	14	5	37	1868	96	M	7	7a	yes	spread	oxer	left	no	0	0	67,78	no
Stuttgart	Grand Prix of Stuttgart presented by Mercedes-Benz, WALTER solar	19.11.2017	CSI-W5*	4	1.60	410	119	13	06.05.2005	S	OS	194	6	14	5	37	1868	96	M	8	7b	yes	vertical	vertical	left	no	0	0	67,78	no
Stuttgart	Grand Prix of Stuttgart presented by Mercedes-Benz, WALTER solar	19.11.2017	CSI-W5*	4	1.60	410	119	13	06.05.2005	S	OS	194	6	14	5	37	1868	96	M	9	7c	yes	spread	oxer	left	no	0	0	67,78	no
Stuttgart	Grand Prix of Stuttgart presented by Mercedes-Benz, WALTER solar	19.11.2017	CSI-W5*	4	1.60	410	119	13	06.05.2005	S	OS	194	6	14	5	37	1868	96	M	10	8	no	spread	oxer	left	no	0	0	67,78	no
Stuttgart	Grand Prix of Stuttgart presented by Mercedes-Benz, WALTER solar	19.11.2017	CSI-W5*	4	1.60	410	119	13	06.05.2005	S	OS	194	6	14	5	37	1868	96	M	11	9	no	vertical	vertical	left	no	0	0	67,78	no

Stuttgart	Grand Prix of Stuttgart presented by Mercedes-Benz, WALTER solar	19.11.2017	CSI-W5*	4	1.60	410	119	13	06.05.2005	S	OS	194	6	14	5	37	1868	96	M	12	10	no	spread	oxer	right	no	0	0	67,78	no
Stuttgart	Grand Prix of Stuttgart presented by Mercedes-Benz, WALTER solar	19.11.2017	CSI-W5*	4	1.60	410	119	13	06.05.2005	S	OS	194	6	14	5	37	1868	96	M	13	11	no	vertical	vertical	right	yes	4	0	67,78	no
Stuttgart	Grand Prix of Stuttgart presented by Mercedes-Benz, WALTER solar	19.11.2017	CSI-W5*	4	1.60	410	119	13	06.05.2005	S	OS	194	6	14	5	37	1868	96	M	14	12a	yes	vertical	vertical	right	no	0	0	67,78	no
Stuttgart	Grand Prix of Stuttgart presented by Mercedes-Benz, WALTER solar	19.11.2017	CSI-W5*	4	1.60	410	119	13	06.05.2005	S	OS	194	6	14	5	37	1868	96	M	15	12b	yes	spread	oxer with water	right	no	0	0	67,78	no
Stuttgart	Grand Prix of Stuttgart presented by Mercedes-Benz, WALTER solar	19.11.2017	CSI-W5*	4	1.60	410	119	13	06.05.2005	S	OS	194	6	14	5	37	1868	96	M	16	13	no	spread	oxer with water	straight	no	0	0	67,78	no
Stuttgart	Grand Prix of Stuttgart presented by Mercedes-Benz, WALTER solar	19.11.2017	CSI-W5*	4	1.60	410	174	13	05.05.2005	G	SF	236	4	16	17	37	2215	106	M	1	1	no	vertical	wall	right	no	0	0	65,44	no
Stuttgart	Grand Prix of Stuttgart presented by Mercedes-Benz, WALTER solar	19.11.2017	CSI-W5*	4	1.60	410	174	13	05.05.2005	G	SF	236	4	16	17	37	2215	106	M	2	2	no	spread	oxer	right	no	0	0	65,44	no
Stuttgart	Grand Prix of Stuttgart presented by Mercedes-Benz, WALTER solar	19.11.2017	CSI-W5*	4	1.60	410	174	13	05.05.2005	G	SF	236	4	16	17	37	2215	106	M	3	3	no	vertical	vertical	right	no	0	0	65,44	no
Stuttgart	Grand Prix of Stuttgart presented by Mercedes-Benz, WALTER solar	19.11.2017	CSI-W5*	4	1.60	410	174	13	05.05.2005	G	SF	236	4	16	17	37	2215	106	M	4	4	no	spread	oxer	straight	no	0	0	65,44	no
Stuttgart	Grand Prix of Stuttgart presented by Mercedes-Benz, WALTER solar	19.11.2017	CSI-W5*	4	1.60	410	174	13	05.05.2005	G	SF	236	4	16	17	37	2215	106	M	5	5	no	spread	oxer	straight	no	0	0	65,44	no
Stuttgart	Grand Prix of Stuttgart presented by Mercedes-Benz, WALTER solar	19.11.2017	CSI-W5*	4	1.60	410	174	13	05.05.2005	G	SF	236	4	16	17	37	2215	106	M	6	6	no	vertical	vertical	left	no	0	0	65,44	no
Stuttgart	Grand Prix of Stuttgart presented by Mercedes-Benz, WALTER solar	19.11.2017	CSI-W5*	4	1.60	410	174	13	05.05.2005	G	SF	236	4	16	17	37	2215	106	M	7	7a	yes	spread	oxer	left	yes	4	0	65,44	no
Stuttgart	Grand Prix of Stuttgart presented by Mercedes-Benz, WALTER solar	19.11.2017	CSI-W5*	4	1.60	410	174	13	05.05.2005	G	SF	236	4	16	17	37	2215	106	M	8	7b	yes	vertical	vertical	left	no	0	0	65,44	no
Stuttgart	Grand Prix of Stuttgart presented by Mercedes-Benz, WALTER solar	19.11.2017	CSI-W5*	4	1.60	410	174	13	05.05.2005	G	SF	236	4	16	17	37	2215	106	M	9	7c	yes	spread	oxer	left	no	0	0	65,44	no
Stuttgart	Grand Prix of Stuttgart presented by Mercedes-Benz, WALTER solar	19.11.2017	CSI-W5*	4	1.60	410	174	13	05.05.2005	G	SF	236	4	16	17	37	2215	106	M	10	8	no	spread	oxer	left	no	0	0	65,44	no
Stuttgart	Grand Prix of Stuttgart presented by Mercedes-Benz, WALTER solar	19.11.2017	CSI-W5*	4	1.60	410	174	13	05.05.2005	G	SF	236	4	16	17	37	2215	106	M	11	9	no	vertical	vertical	left	no	0	0	65,44	no
Stuttgart	Grand Prix of Stuttgart presented by Mercedes-Benz, WALTER solar	19.11.2017	CSI-W5*	4	1.60	410	174	13	05.05.2005	G	SF	236	4	16	17	37	2215	106	M	12	10	no	spread	oxer	right	no	0	0	65,44	no
Stuttgart	Grand Prix of Stuttgart presented by Mercedes-Benz, WALTER solar	19.11.2017	CSI-W5*	4	1.60	410	174	13	05.05.2005	G	SF	236	4	16	17	37	2215	106	M	13	11	no	vertical	vertical	right	no	0	0	65,44	no
Stuttgart	Grand Prix of Stuttgart presented by Mercedes-Benz, WALTER solar	19.11.2017	CSI-W5*	4	1.60	410	174	13	05.05.2005	G	SF	236	4	16	17	37	2215	106	M	14	12a	yes	vertical	vertical	right	no	0	0	65,44	no
Stuttgart	Grand Prix of Stuttgart presented by Mercedes-Benz, WALTER solar	19.11.2017	CSI-W5*	4	1.60	410	174	13	05.05.2005	G	SF	236	4	16	17	37	2215	106	M	15	12b	yes	spread	oxer with water	right	no	0	0	65,44	no
Stuttgart	Grand Prix of Stuttgart presented by Mercedes-Benz, WALTER solar	19.11.2017	CSI-W5*	4	1.60	410	174	13	05.05.2005	G	SF	236	4	16	17	37	2215	106	M	16	13	no	spread	oxer with water	straight	no	0	0	65,44	no
Stuttgart	Grand Prix of Stuttgart presented by Mercedes-Benz, WALTER solar	19.11.2017	CSI-W5*	4	1.60	410	100	12	17.03.2006	G	SBS	137	20	17	3	46	1005	51	M	1	1	no	vertical	wall	right	no	0	0	65,5	no
Stuttgart	Grand Prix of Stuttgart presented by Mercedes-Benz, WALTER solar	19.11.2017	CSI-W5*	4	1.60	410	100	12	17.03.2006	G	SBS	137	20	17	3	46	1005	51	M	2	2	no	spread	oxer	right	no	0	0	65,5	no
Stuttgart	Grand Prix of Stuttgart presented by Mercedes-Benz, WALTER solar	19.11.2017	CSI-W5*	4	1.60	410	100	12	17.03.2006	G	SBS	137	20	17	3	46	1005	51	M	3	3	no	vertical	vertical	right	no	0	0	65,5	no
Stuttgart	Grand Prix of Stuttgart presented by Mercedes-Benz, WALTER solar	19.11.2017	CSI-W5*	4	1.60	410	100	12	17.03.2006	G	SBS	137	20	17	3	46	1005	51	M	4	4	no	spread	oxer	straight	no	0	0	65,5	no
Stuttgart	Grand Prix of Stuttgart presented by Mercedes-Benz, WALTER solar	19.11.2017	CSI-W5*	4	1.60	410	100	12	17.03.2006	G	SBS	137	20	17	3	46	1005	51	M	5	5	no	spread	oxer	straight	no	0	0	65,5	no
Stuttgart	Grand Prix of Stuttgart presented by Mercedes-Benz, WALTER solar	19.11.2017	CSI-W5*	4	1.60	410	100	12	17.03.2006	G	SBS	137	20	17	3	46	1005	51	M	6	6	no	vertical	vertical	left	no	0	0	65,5	no
Stuttgart	Grand Prix of Stuttgart presented by Mercedes-Benz, WALTER solar	19.11.2017	CSI-W5*	4	1.60	410	100	12	17.03.2006	G	SBS	137	20	17	3	46	1005	51	M	7	7a	yes	spread	oxer	left	yes	4	0	65,5	no
Stuttgart	Grand Prix of Stuttgart presented by Mercedes-Benz, WALTER solar	19.11.2017	CSI-W5*	4	1.60	410	100	12	17.03.2006	G	SBS	137	20	17	3	46	1005	51	M	8	7b	yes	vertical	vertical	left	yes	4	0	65,5	no
Stuttgart	Grand Prix of Stuttgart presented by Mercedes-Benz, WALTER solar	19.11.2017	CSI-W5*	4	1.60	410	100	12	17.03.2006	G	SBS	137	20	17	3	46	1005	51	M	9	7c	yes	spread	oxer	left	no	0	0	65,5	no
Stuttgart	Grand Prix of Stuttgart presented by Mercedes-Benz, WALTER solar	19.11.2017	CSI-W5*	4	1.60	410	100	12	17.03.2006	G	SBS	137	20	17	3	46	1005	51	M	10	8	no	spread	oxer	left	no	0	0	65,5	no
Stuttgart	Grand Prix of Stuttgart presented by Mercedes-Benz, WALTER solar	19.11.2017	CSI-W5*	4	1.60	410	100	12	17.03.2006	G	SBS	137	20	17	3	46	1005	51	M	11	9	no	vertical	vertical	left	no	0	0	65,5	no
Stuttgart	Grand Prix of Stuttgart presented by Mercedes-Benz, WALTER solar	19.11.2017	CSI-W5*	4	1.60	410	100	12	17.03.2006	G	SBS	137	20	17	3	46	1005	51	M	12	10	no	spread	oxer	right	no	0	0	65,5	no
Stuttgart	Grand Prix of Stuttgart presented by Mercedes-Benz, WALTER solar	19.11.2017	CSI-W5*	4	1.60	410	100	12	17.03.2006	G	SBS	137	20	17	3	46	1005	51	M	13	11	no	vertical	vertical	right	no	0	0	65,5	no
Stuttgart	Grand Prix of Stuttgart presented by Mercedes-Benz, WALTER solar	19.11.2017	CSI-W5*	4	1.60	410	100	12	17.03.2006	G	SBS	137	20	17	3	46	1005	51	M	14	12a	yes	vertical	vertical	right	no	0	0	65,5	no
Stuttgart	Grand Prix of Stuttgart presented by Mercedes-Benz, WALTER solar	19.11.2017	CSI-W5*	4	1.60	410	100	12	17.03.2006	G	SBS	137	20	17	3	46	1005	51	M	15	12b	yes	spread	oxer with water	right	no	0	0	65,5	no
Stuttgart	Grand Prix of Stuttgart presented by Mercedes-Benz, WALTER solar	19.11.2017	CSI-W5*	4	1.60	410	100	12	17.03.2006	G	SBS	137	20	17	3	46	1005	51	M	16	13	no	spread	oxer with water	straight	no	0	0	65,5	no
Stuttgart	Grand Prix of Stuttgart presented by Mercedes-Benz, WALTER solar	19.11.2017	CSI-W5*	4	1.60	410	219	13	13.05.2005	G	SWB	110	2	21	99	28	1384	56	F	1	1	no	vertical	wall	right	no	0	0	68,88	no
Stuttgart	Grand Prix of Stuttgart presented by Mercedes-Benz, WALTER solar	19.11.2017	CSI-W5*	4	1.60	410	219	13	13.05.2005	G	SWB	110	2	21	99	28	1384	56	F	2	2	no	spread	oxer	right	no	0	0	68,88	no
Stuttgart	Grand Prix of Stuttgart presented by Mercedes-Benz, WALTER solar	19.11.2017	CSI-W5*	4	1.60	410	219	13	13.05.2005	G	SWB	110	2	21	99	28	1384	56	F	3	3	no	vertical	vertical	right	no	0	0	68,88	no
Stuttgart	Grand Prix of Stuttgart presented by Mercedes-Benz, WALTER solar	19.11.2017	CSI-W5*	4	1.60	410	219	13	13.05.2005	G	SWB	110	2	21	99	28	1384	56	F	4	4	no	spread	oxer	straight	no	0	0	68,88	no
Stuttgart	Grand Prix of Stuttgart presented by Mercedes-Benz, WALTER solar	19.11.2017	CSI-W5*	4	1.60	410	219	13	13.05.2005	G	SWB	110	2	21	99	28	1384	56	F	5	5	no	spread	oxer	straight	no	0	0	68,88	no
Stuttgart	Grand Prix of Stuttgart presented by Mercedes-Benz, WALTER solar	19.11.2017	CSI-W5*	4	1.60	410	219	13	13.05.2005	G	SWB	110	2	21	99	28	1384	56	F	6	6	no	vertical	vertical	left	no	0	0	68,88	no
Stuttgart	Grand Prix of Stuttgart presented by Mercedes-Benz, WALTER solar	19.11.2017	CSI-W5*	4	1.60	410	219	13	13.05.2005	G	SWB	110	2	21	99	28	1384	56	F	7	7a	yes	spread	oxer	left	no	0	0	68,88	no
Stuttgart	Grand Prix of Stuttgart presented by Mercedes-Benz, WALTER solar	19.11.2017	CSI-W5*	4	1.60	410	219	13	13.05.2005	G	SWB	110	2	21	99	28	1384	56	F	8	7b	yes	vertical	vertical	left	no	0	0	68,88	no
Stuttgart	Grand Prix of Stuttgart presented by Mercedes-Benz, WALTER solar	19.11.2017	CSI-W5*	4	1.60	410	219	13	13.05.2005	G	SWB	110	2	21	99	28	1384	56	F	9	7c	yes	spread	oxer	left	no	0	0	68,88	no
Stuttgart	Grand Prix of Stuttgart presented by Mercedes-Benz, WALTER solar	19.11.2017	CSI-W5*	4	1.60	410	219	13	13.05.2005	G	SWB	110	2	21	99	28	1384	56	F	10	8	no	spread	oxer	left	no	0	0	68,88	no
Stuttgart	Grand Prix of Stuttgart presented by Mercedes-Benz, WALTER solar	19.11.2017	CSI-W5*	4	1.60	410	219	13	13.05.2005	G	SWB	110	2	21	99	28	1384	56	F	11	9	no	vertical	vertical	left	no	0	0	68,88	no
Stuttgart	Grand Prix of Stuttgart presented by Mercedes-Benz, WALTER solar	19.11.2017	CSI-W5*	4	1.60	410	219	13	13.05.2005	G	SWB	110	2	21	99	28	1384	56	F	12	10	no	spread	oxer	right	no	0	0	68,88	no
Stuttgart	Grand Prix of Stuttgart presented by Mercedes-Benz, WALTER solar	19.11.2017	CSI-W5*	4	1.60	410	219	13	13.05.2005	G	SWB	110	2	21	99	28	1384	56	F	13	11	no	vertical	vertical	right	no	0	0	68,88	no
Stuttgart	Grand Prix of Stuttgart presented by Mercedes-Benz, WALTER solar	19.11.2017	CSI-W5*	4	1.60	410	219	13	13.05.2005	G	SWB	110	2	21	99	28	1384	56	F	14	12a	yes	vertical	vertical	right	no	0	0	68,88	no
Stuttgart	Grand Prix of Stuttgart presented by Mercedes-Benz, WALTER solar	19.11.2017	CSI-W5*	4	1.60	410	219	13	13.05.2005	G	SWB	110	2	21	99	28	1384	56	F	15	12b	yes	spread	oxer with water	right	no	0	0	68,88	no
Stuttgart	Grand Prix of Stuttgart presented by Mercedes-Benz, WALTER solar	19.11.2017	CSI-W5*	4	1.60	410	219	13	13.05.2005	G	SWB	110	2	21	99	28	1384	56	F	16	13	no	spread	oxer with water	straight	no	0	0	68,88	no
Stuttgart	Grand Prix of Stuttgart presented by Mercedes-Benz, WALTER solar	19.11.2017	CSI-W5*	4	1.60	380	219	13	13.05.2005	G	SWB	110	2	21	99	28	1384	56	F	1	1	no	spread	oxer	left	no	0	0	51,44	no
Stuttgart	Grand Prix of Stuttgart presented by Mercedes-Benz, WALTER solar	19.11.2017	CSI-W5*	4	1.60	380	219	13	13.05.2005	G	SWB	110	2	21	99	28	1384	56	F	2	2	no	vertical	vertical	left	no	0	0	51,44	no
Stuttgart	Grand Prix of Stuttgart presented by Mercedes-Benz, WALTER solar	19.11.2017	CSI-W5*	4	1.60	380	219	13	13.05.2005	G	SWB	110	2	21	99	28	1384	56	F	3	3	no	vertical	vertical	right	no	0	0	51,44	no
Stuttgart	Grand Prix of Stuttgart presented by Mercedes-Benz, WALTER solar	19.11.2017	CSI-W5*	4	1.60	380	219	13	13.05.2005	G	SWB	110	2	21	99	28	1384	56	F	4	4	no	spread	oxer with water	right	no	0	0	51,44	no
Stuttgart	Grand Prix of Stuttgart presented by Mercedes-Benz, WALTER solar	19.11.2017	CSI-W5*	4	1.60	380	219	13	13.05.2005	G	SWB	110	2	21	99	28	1384	56	F	5	5	no	vertical	vertical	left	no	0	0	51,44	no
Stuttgart	Grand Prix of Stuttgart presented by Mercedes-Benz, WALTER solar	19.11.2017	CSI-W5*	4	1.60	380	219	13	13.05.2005	G	SWB	110	2	21	99	28	1384	56	F	6	6	no	vertical	wall	right	no	0	0	51,44	no
Stuttgart	Grand Prix of Stuttgart presented by Mercedes-Benz, WALTER solar	19.11.2017	CSI-W5*	4	1.60	380	219	13	13.05.2005	G	SWB	110	2	21	99	28	1384	56	F	7	7	no	spread	oxer	right	no	0	0	51,44	no
Stuttgart	Grand Prix of Stuttgart presented by Mercedes-Benz, WALTER solar	19.11.2017	CSI-W5*	4	1.60	380	219	13	13.05.2005	G	SWB	110	2	21	99	28	1384	56	F	8	8a	yes	spread	oxer	left	no	0	0	51,44	no
Stuttgart	Grand Prix of Stuttgart presented by Mercedes-Benz, WALTER solar	19.11.2017	CSI-W5*	4	1.60	380	219	13	13.05.2005	G	SWB	110	2	21	99	28	1384	56	F	9	8b	yes	vertical	vertical	left	no	0	0	51,44	no
Stuttgart	Grand Prix of Stuttgart presented by Mercedes-Benz, WALTER solar	19.11.2017	CSI-W5*	4	1.60	380	219	13	13.05.2005	G	SWB	110	2	21	99	28	1384	56	F	10	9	no	spread	oxer	left	no	0	0	51,44	no
Stuttgart	Grand Prix of Stuttgart presented by Mercedes-Benz, WALTER solar	19.11.2017	CSI-W5*	4	1.60	410	5	13	31.03.2005	G	KWPN	238	4	25	558	26	820	16	F	1	1	no	vertical	wall	right	no	0	0	68,34	no
Stuttgart	Grand Prix of Stuttgart presented by Mercedes-Benz, WALTER solar	19.11.2017	CSI-W5*	4	1.60	410	5	13	31.03.2005	G	KWPN	238	4	25	558	26	820	16	F	2	2	no	spread	oxer	right	no	0	0	68,34	no
Stuttgart	Grand Prix of Stuttgart presented by Mercedes-Benz, WALTER solar	19.11.2017	CSI-W5*	4	1.60	410	5	13	31.03.2005	G	KWPN	238	4	25	558	26	820	16	F	3	3	no	vertical	vertical	right	no	0	0	68,34	no
Stuttgart	Grand Prix of Stuttgart presented by Mercedes-Benz, WALTER solar	19.11.2017	CSI-W5*	4	1.60	410	5	13	31.03.2005	G	KWPN	238	4	25	558	26	820	16	F	4	4	no	spread	oxer	straight	no	0	0	68,34	no
Stuttgart	Grand Prix of Stuttgart presented by Mercedes-Benz, WALTER solar	19.11.2017	CSI-W5*	4	1.60	410	5	13	31.03.2005	G	KWPN	238	4	25	558	26	820	16	F	5	5	no	spread	oxer	straight	no	0	0	68,34	no
Stuttgart	Grand Prix of Stuttgart presented by Mercedes-Benz, WALTER solar	19.11.2017	CSI-W5*	4	1.60	410	5	13	31.03.2005	G	KWPN	238	4	25	558	26	820	16	F	6	6	no	vertical	vertical	left	no	0	0	68,34	no
Stuttgart	Grand Prix of Stuttgart presented by Mercedes-Benz, WALTER solar	19.11.2017	CSI-W5*	4	1.60	410	5	13	31.03.2005	G	KWPN	238	4	25	558	26	820	16	F	7	7a	yes	spread	oxer	left	yes	4	0	68,34	no
Stuttgart	Grand Prix of Stuttgart presented by Mercedes-Benz, WALTER solar	19.11.2017	CSI-W5*	4	1.60	410	5	13	31.03.2005	G	KWPN	238	4	25	558	26	820	16	F	8	7b	yes	vertical	vertical	left	no	0	0	68,34	no
Stuttgart	Grand Prix of Stuttgart presented by Mercedes-Benz, WALTER solar	19.11.2017	CSI-W5*	4	1.60	410	5	13	31.03.2005	G	KWPN	238	4	25	558	26	820	16	F	9	7c	yes	spread	oxer	left	no	0	0	68,34	no
Stuttgart	Grand Prix of Stuttgart presented by Mercedes-Benz, WALTER solar	19.11.2017	CSI-W5*	4	1.60	410	5	13	31.03.2005	G	KWPN	238	4	25	558	26	820	16	F	10	8	no	spread	oxer	left	no	0	0	68,34	no
Stuttgart	Grand Prix of Stuttgart presented by Mercedes-Benz, WALTER solar	19.11.2017	CSI-W5*	4	1.60	410	5	13	31.03.2005	G	KWPN	238	4	25	558	26	820	16	F	11	9	no	vertical	vertical	left	no	0	0	68,34	no
Stuttgart	Grand Prix of Stuttgart presented by Mercedes-Benz, WALTER solar	19.11.2017	CSI-W5*	4	1.60	410	5	13	31.03.2005	G	KWPN	238	4	25	558	26	820	16	F	12	10	no	spread	oxer	right	no	0	0	68,34	no
Stuttgart	Grand Prix of Stuttgart presented by Mercedes-Benz, WALTER solar	19.11.2017	CSI-W5*	4	1.60	410	5	13	31.03.2005	G	KWPN	238	4	25	558	26	820	16	F	13	11	no	vertical	vertical	right	no	0	0	68,34	no
Stuttgart	Grand Prix of Stuttgart presented by Mercedes-Benz, WALTER solar	19.11.2017	CSI-W5*	4	1.60	410	5	13	31.03.2005	G	KWPN	238	4	25	558	26	820	16	F	14	12a	yes	vertical	vertical	right	no	0	0	68,34	no
Stuttgart	Grand Prix of Stuttgart presented by Mercedes-Benz, WALTER solar	19.11.2017	CSI-W5*	4	1.60	410	5	13	31.03.2005	G	KWPN	238	4	25	558	26	820	16	F	15	12b	yes	spread	oxer with water	right	no	0	0	68,34	no
Stuttgart	Grand Prix of Stuttgart presented by Mercedes-Benz, WALTER solar	19.11.2017	CSI-W5*	4	1.60	410	5	13	31.03.2005	G	KWPN	238	4	25	558	26	820	16	F	16	13	no	spread	oxer with water	straight	no	0	0	68,34	no
Stuttgart	Grand Prix of Stuttgart presented by Mercedes-Benz, WALTER solar	19.11.2017	CSI-W5*	4	1.60	410	184	12	18.06.2006	S	SF	154	4	28	4	38	2170	90	M	1	1	no	vertical	wall	right	no	0	0	66,23	no
Stuttgart	Grand Prix of Stuttgart presented by Mercedes-Benz, WALTER solar	19.11.2017	CSI-W5*	4	1.60	410	184	12	18.06.2006	S	SF	154	4	28	4	38	2170	90	M	2	2	no	spread	oxer	right	no	0	0	66,23	no
Stuttgart	Grand Prix of Stuttgart presented by Mercedes-Benz, WALTER solar	19.11.2017	CSI-W5*	4	1.60	410	184	12	18.06.2006	S	SF	154	4	28	4	38	2170	90	M	3	3	no	vertical	vertical	right	no	0	0	66,23	no
Stuttgart	Grand Prix of Stuttgart presented by Mercedes-Benz, WALTER solar	19.11.2017	CSI-W5*	4	1.60	410	184	12	18.06.2006	S	SF	154	4	28	4	38	2170	90	M	4	4	no	spread	oxer	straight	no	0	0	66,23	no
Stuttgart	Grand Prix of Stuttgart presented by Mercedes-Benz, WALTER solar	19.11.2017	CSI-W5*	4	1.60	410	184	12	18.06.2006	S	SF	154	4	28	4	38	2170	90	M	5	5	no	spread	oxer	straight	no	0	0	66,23	no
Stuttgart	Grand Prix of Stuttgart presented by Mercedes-Benz, WALTER solar	19.11.2017	CSI-W5*	4	1.60	410	184	12	18.06.2006	S	SF	154	4	28	4	38	2170	90	M	6	6	no	vertical	vertical	left	no	0	0	66,23	no
Stuttgart	Grand Prix of Stuttgart presented by Mercedes-Benz, WALTER solar	19.11.2017	CSI-W5*	4	1.60	410	184	12	18.06.2006	S	SF	154	4	28	4	38	2170	90	M	7	7a	yes	spread	oxer	left	no	0	0	66,23	no
Stuttgart	Grand Prix of Stuttgart presented by Mercedes-Benz, WALTER solar	19.11.2017	CSI-W5*	4	1.60	410	184	12	18.06.2006	S	SF	154	4	28	4	38	2170	90	M	8	7b	yes	vertical	vertical	left	no	0	0	66,23	no
Stuttgart	Grand Prix of Stuttgart presented by Mercedes-Benz, WALTER solar	19.11.2017	CSI-W5*	4	1.60	410	184	12	18.06.2006	S	SF	154	4	28	4	38	2170	90	M	9	7c	yes	spread	oxer	left	no	0	0	66,23	no
Stuttgart	Grand Prix of Stuttgart presented by Mercedes-Benz, WALTER solar	19.11.2017	CSI-W5*	4	1.60	410	184	12	18.06.2006	S	SF	154	4	28	4	38	2170	90	M	10	8	no	spread	oxer	left	no	0	0	66,23	no
Stuttgart	Grand Prix of Stuttgart presented by Mercedes-Benz, WALTER solar	19.11.2017	CSI-W5*	4	1.60	410	184	12	18.06.2006	S	SF	154	4	28	4	38	2170	90	M	11	9	no	vertical	vertical	left	no	0	0	66,23	no
Stuttgart	Grand Prix of Stuttgart presented by Mercedes-Benz, WALTER solar	19.11.2017	CSI-W5*	4	1.60	410	184	12	18.06.2006	S	SF	154	4	28	4	38	2170	90	M	12	10	no	spread	oxer	right	no	0	0	66,23	no
Stuttgart	Grand Prix of Stuttgart presented by Mercedes-Benz, WALTER solar	19.11.2017	CSI-W5*	4	1.60	410	184	12	18.06.2006	S	SF	154	4	28	4	38	2170	90	M	13	11	no	vertical	vertical	right	no	0	0	66,23	no
Stuttgart	Grand Prix of Stuttgart presented by Mercedes-Benz, WALTER solar	19.11.2017	CSI-W5*	4	1.60	410	184	12	18.06.2006	S	SF	154	4	28	4	38	2170	90	M	14	12a	yes	vertical	vertical	right	no	0	0	66,23	no
Stuttgart	Grand Prix of Stuttgart presented by Mercedes-Benz, WALTER solar	19.11.2017	CSI-W5*	4	1.60	410	184	12	18.06.2006	S	SF	154	4	28	4	38	2170	90	M	15	12b	yes	spread	oxer with water	right	no	0	0	66,23	no
Stuttgart	Grand Prix of Stuttgart presented by Mercedes-Benz, WALTER solar	19.11.2017	CSI-W5*	4	1.60	410	184	12	18.06.2006	S	SF	154	4	28	4	38	2170	90	M	16	13	no	spread	oxer with water	straight	no	0	0	66,23	no
Stuttgart	Grand Prix of Stuttgart presented by Mercedes-Benz, WALTER solar	19.11.2017	CSI-W5*	4	1.60	380	184	12	18.06.2006	S	SF	154	4	28	4	38	2170	90	M	1	1	no	spread	oxer	left	no	0	0	51,01	no
Stuttgart	Grand Prix of Stuttgart presented by Mercedes-Benz, WALTER solar	19.11.2017	CSI-W5*	4	1.60	380	184	12	18.06.2006	S	SF	154	4	28	4	38	2170	90	M	2	2	no	vertical	vertical	left	no	0	0	51,01	no
Stuttgart	Grand Prix of Stuttgart presented by Mercedes-Benz, WALTER solar	19.11.2017	CSI-W5*	4	1.60	380	184	12	18.06.2006	S	SF	154	4	28	4	38	2170	90	M	3	3	no	vertical	vertical	right	no	0	0	51,01	no
Stuttgart	Grand Prix of Stuttgart presented by Mercedes-Benz, WALTER solar	19.11.2017	CSI-W5*	4	1.60	380	184	12	18.06.2006	S	SF	154	4	28	4	38	2170	90	M	4	4	no	spread	oxer with water	right	no	0	0	51,01	no
Stuttgart	Grand Prix of Stuttgart presented by Mercedes-Benz, WALTER solar	19.11.2017	CSI-W5*	4	1.60	380	184	12	18.06.2006	S	SF	154	4	28	4	38	2170	90	M	5	5	no	vertical	vertical	left	no	0	0	51,01	no
Stuttgart	Grand Prix of Stuttgart presented by Mercedes-Benz, WALTER solar	19.11.2017	CSI-W5*	4	1.60	380	184	12	18.06.2006	S	SF	154	4	28	4	38	2170	90	M	6	6	no	vertical	wall	right	no	0	0	51,01	no
Stuttgart	Grand Prix of Stuttgart presented by Mercedes-Benz, WALTER solar	19.11.2017	CSI-W5*	4	1.60	380	184	12	18.06.2006	S	SF	154	4	28	4	38	2170	90	M	7	7	no	spread	oxer	right	no	0	0	51,01	no
Stuttgart	Grand Prix of Stuttgart presented by Mercedes-Benz, WALTER solar	19.11.2017	CSI-W5*	4	1.60	380	184	12	18.06.2006	S	SF	154	4	28	4	38	2170	90	M	8	8a	yes	spread	oxer	left	no	0	0	51,01	no
Stuttgart	Grand Prix of Stuttgart presented by Mercedes-Benz, WALTER solar	19.11.2017	CSI-W5*	4	1.60	380	184	12	18.06.2006	S	SF	154	4	28	4	38	2170	90	M	9	8b	yes	vertical	vertical	left	no	0	0	51,01	no
Stuttgart	Grand Prix of Stuttgart presented by Mercedes-Benz, WALTER solar	19.11.2017	CSI-W5*	4	1.60	380	184	12	18.06.2006	S	SF	154	4	28	4	38	2170	90	M	10	9	no	spread	oxer	left	no	0	0	51,01	no
Stuttgart	Grand Prix of Stuttgart presented by Mercedes-Benz, WALTER solar	19.11.2017	CSI-W5*	4	1.60	410	35	15	22.05.2003	G	HOLST	368	19	29	89	35	1502	55	M	1	1	no	vertical	wall	right	no	0	0	68,81	no
Stuttgart	Grand Prix of Stuttgart presented by Mercedes-Benz, WALTER solar	19.11.2017	CSI-W5*	4	1.60	410	35	15	22.05.2003	G	HOLST	368	19	29	89	35	1502	55	M	2	2	no	spread	oxer	right	no	0	0	68,81	no
Stuttgart	Grand Prix of Stuttgart presented by Mercedes-Benz, WALTER solar	19.11.2017	CSI-W5*	4	1.60	410	35	15	22.05.2003	G	HOLST	368	19	29	89	35	1502	55	M	3	3	no	vertical	vertical	right	no	0	0	68,81	no
Stuttgart	Grand Prix of Stuttgart presented by Mercedes-Benz, WALTER solar	19.11.2017	CSI-W5*	4	1.60	410	35	15	22.05.2003	G	HOLST	368	19	29	89	35	1502	55	M	4	4	no	spread	oxer	straight	no	0	0	68,81	no
Stuttgart	Grand Prix of Stuttgart presented by Mercedes-Benz, WALTER solar	19.11.2017	CSI-W5*	4	1.60	410	35	15	22.05.2003	G	HOLST	368	19	29	89	35	1502	55	M	5	5	no	spread	oxer	straight	no	0	0	68,81	no
Stuttgart	Grand Prix of Stuttgart presented by Mercedes-Benz, WALTER solar	19.11.2017	CSI-W5*	4	1.60	410	35	15	22.05.2003	G	HOLST	368	19	29	89	35	1502	55	M	6	6	no	vertical	vertical	left	no	0	0	68,81	no
Stuttgart	Grand Prix of Stuttgart presented by Mercedes-Benz, WALTER solar	19.11.2017	CSI-W5*	4	1.60	410	35	15	22.05.2003	G	HOLST	368	19	29	89	35	1502	55	M	7	7a	yes	spread	oxer	left	no	0	0	68,81	no
Stuttgart	Grand Prix of Stuttgart presented by Mercedes-Benz, WALTER solar	19.11.2017	CSI-W5*	4	1.60	410	35	15	22.05.2003	G	HOLST	368	19	29	89	35	1502	55	M	8	7b	yes	vertical	vertical	left	no	0	0	68,81	no
Stuttgart	Grand Prix of Stuttgart presented by Mercedes-Benz, WALTER solar	19.11.2017	CSI-W5*	4	1.60	410	35	15	22.05.2003	G	HOLST	368	19	29	89	35	1502	55	M	9	7c	yes	spread	oxer	left	yes	4	0	68,81	no
Stuttgart	Grand Prix of Stuttgart presented by Mercedes-Benz, WALTER solar	19.11.2017	CSI-W5*	4	1.60	410	35	15	22.05.2003	G	HOLST	368	19	29	89	35	1502	55	M	10	8	no	spread	oxer	left	no	0	0	68,81	no
Stuttgart	Grand Prix of Stuttgart presented by Mercedes-Benz, WALTER solar	19.11.2017	CSI-W5*	4	1.60	410	35	15	22.05.2003	G	HOLST	368	19	29	89	35	1502	55	M	11	9	no	vertical	vertical	left	no	0	0	68,81	no
Stuttgart	Grand Prix of Stuttgart presented by Mercedes-Benz, WALTER solar	19.11.2017	CSI-W5*	4	1.60	410	35	15	22.05.2003	G	HOLST	368	19	29	89	35	1502	55	M	12	10	no	spread	oxer	right	no	0	0	68,81	no
Stuttgart	Grand Prix of Stuttgart presented by Mercedes-Benz, WALTER solar	19.11.2017	CSI-W5*	4	1.60	410	35	15	22.05.2003	G	HOLST	368	19	29	89	35	1502	55	M	13	11	no	vertical	vertical	right	no	0	0	68,81	no
Stuttgart	Grand Prix of Stuttgart presented by Mercedes-Benz, WALTER solar	19.11.2017	CSI-W5*	4	1.60	410	35	15	22.05.2003	G	HOLST	368	19	29	89	35	1502	55	M	14	12a	yes	vertical	vertical	right	yes	4	0	68,81	no
Stuttgart	Grand Prix of Stuttgart presented by Mercedes-Benz, WALTER solar	19.11.2017	CSI-W5*	4	1.60	410	35	15	22.05.2003	G	HOLST	368	19	29	89	35	1502	55	M	15	12b	yes	spread	oxer with water	right	no	0	0	68,81	no
Stuttgart	Grand Prix of Stuttgart presented by Mercedes-Benz, WALTER solar	19.11.2017	CSI-W5*	4	1.60	410	35	15	22.05.2003	G	HOLST	368	19	29	89	35	1502	55	M	16	13	no	spread	oxer with water	straight	no	0	0	68,81	no
Stuttgart	Grand Prix of Stuttgart presented by Mercedes-Benz, WALTER solar	19.11.2017	CSI-W5*	4	1.60	410	108	11	24.03.2007	M	BWP	91	5	30	9	36	2556	124	M	1	1	no	vertical	wall	right	no	0	0	65,93	no
Stuttgart	Grand Prix of Stuttgart presented by Mercedes-Benz, WALTER solar	19.11.2017	CSI-W5*	4	1.60	410	108	11	24.03.2007	M	BWP	91	5	30	9	36	2556	124	M	2	2	no	spread	oxer	right	no	0	0	65,93	no
Stuttgart	Grand Prix of Stuttgart presented by Mercedes-Benz, WALTER solar	19.11.2017	CSI-W5*	4	1.60	410	108	11	24.03.2007	M	BWP	91	5	30	9	36	2556	124	M	3	3	no	vertical	vertical	right	no	0	0	65,93	no
Stuttgart	Grand Prix of Stuttgart presented by Mercedes-Benz, WALTER solar	19.11.2017	CSI-W5*	4	1.60	410	108	11	24.03.2007	M	BWP	91	5	30	9	36	2556	124	M	4	4	no	spread	oxer	straight	no	0	0	65,93	no
Stuttgart	Grand Prix of Stuttgart presented by Mercedes-Benz, WALTER solar	19.11.2017	CSI-W5*	4	1.60	410	108	11	24.03.2007	M	BWP	91	5	30	9	36	2556	124	M	5	5	no	spread	oxer	straight	no	0	0	65,93	no
Stuttgart	Grand Prix of Stuttgart presented by Mercedes-Benz, WALTER solar	19.11.2017	CSI-W5*	4	1.60	410	108	11	24.03.2007	M	BWP	91	5	30	9	36	2556	124	M	6	6	no	vertical	vertical	left	no	0	0	65,93	no
Stuttgart	Grand Prix of Stuttgart presented by Mercedes-Benz, WALTER solar	19.11.2017	CSI-W5*	4	1.60	410	108	11	24.03.2007	M	BWP	91	5	30	9	36	2556	124	M	7	7a	yes	spread	oxer	left	no	0	0	65,93	no
Stuttgart	Grand Prix of Stuttgart presented by Mercedes-Benz, WALTER solar	19.11.2017	CSI-W5*	4	1.60	410	108	11	24.03.2007	M	BWP	91	5	30	9	36	2556	124	M	8	7b	yes	vertical	vertical	left	no	0	0	65,93	no
Stuttgart	Grand Prix of Stuttgart presented by Mercedes-Benz, WALTER solar	19.11.2017	CSI-W5*	4	1.60	410	108	11	24.03.2007	M	BWP	91	5	30	9	36	2556	124	M	9	7c	yes	spread	oxer	left	no	0	0	65,93	no
Stuttgart	Grand Prix of Stuttgart presented by Mercedes-Benz, WALTER solar	19.11.2017	CSI-W5*	4	1.60	410	108	11	24.03.2007	M	BWP	91	5	30	9	36	2556	124	M	10	8	no	spread	oxer	left	no	0	0	65,93	no
Stuttgart	Grand Prix of Stuttgart presented by Mercedes-Benz, WALTER solar	19.11.2017	CSI-W5*	4	1.60	410	108	11	24.03.2007	M	BWP	91	5	30	9	36	2556	124	M	11	9	no	vertical	vertical	left	no	0	0	65,93	no
Stuttgart	Grand Prix of Stuttgart presented by Mercedes-Benz, WALTER solar	19.11.2017	CSI-W5*	4	1.60	410	108	11	24.03.2007	M	BWP	91	5	30	9	36	2556	124	M	12	10	no	spread	oxer	right	no	0	0	65,93	no
Stuttgart	Grand Prix of Stuttgart presented by Mercedes-Benz, WALTER solar	19.11.2017	CSI-W5*	4	1.60	410	108	11	24.03.2007	M	BWP	91	5	30	9	36	2556	124	M	13	11	no	vertical	vertical	right	no	0	0	65,93	no
Stuttgart	Grand Prix of Stuttgart presented by Mercedes-Benz, WALTER solar	19.11.2017	CSI-W5*	4	1.60	410	108	11	24.03.2007	M	BWP	91	5	30	9	36	2556	124	M	14	12a	yes	vertical	vertical	right	no	0	0	65,93	no
Stuttgart	Grand Prix of Stuttgart presented by Mercedes-Benz, WALTER solar	19.11.2017	CSI-W5*	4	1.60	410	108	11	24.03.2007	M	BWP	91	5	30	9	36	2556	124	M	15	12b	yes	spread	oxer with water	right	no	0	0	65,93	no
Stuttgart	Grand Prix of Stuttgart presented by Mercedes-Benz, WALTER solar	19.11.2017	CSI-W5*	4	1.60	410	108	11	24.03.2007	M	BWP	91	5	30	9	36	2556	124	M	16	13	no	spread	oxer with water	straight	no	0	0	65,93	no
Stuttgart	Grand Prix of Stuttgart presented by Mercedes-Benz, WALTER solar	19.11.2017	CSI-W5*	4	1.60	380	108	11	24.03.2007	M	BWP	91	5	30	9	36	2556	124	M	1	1	no	spread	oxer	left	no	0	0	48,19	no
Stuttgart	Grand Prix of Stuttgart presented by Mercedes-Benz, WALTER solar	19.11.2017	CSI-W5*	4	1.60	380	108	11	24.03.2007	M	BWP	91	5	30	9	36	2556	124	M	2	2	no	vertical	vertical	left	no	0	0	48,19	no
Stuttgart	Grand Prix of Stuttgart presented by Mercedes-Benz, WALTER solar	19.11.2017	CSI-W5*	4	1.60	380	108	11	24.03.2007	M	BWP	91	5	30	9	36	2556	124	M	3	3	no	vertical	vertical	right	no	0	0	48,19	no
Stuttgart	Grand Prix of Stuttgart presented by Mercedes-Benz, WALTER solar	19.11.2017	CSI-W5*	4	1.60	380	108	11	24.03.2007	M	BWP	91	5	30	9	36	2556	124	M	4	4	no	spread	oxer with water	right	no	0	0	48,19	no
Stuttgart	Grand Prix of Stuttgart presented by Mercedes-Benz, WALTER solar	19.11.2017	CSI-W5*	4	1.60	380	108	11	24.03.2007	M	BWP	91	5	30	9	36	2556	124	M	5	5	no	vertical	vertical	left	no	0	0	48,19	no
Stuttgart	Grand Prix of Stuttgart presented by Mercedes-Benz, WALTER solar	19.11.2017	CSI-W5*	4	1.60	380	108	11	24.03.2007	M	BWP	91	5	30	9	36	2556	124	M	6	6	no	vertical	wall	right	no	0	0	48,19	no
Stuttgart	Grand Prix of Stuttgart presented by Mercedes-Benz, WALTER solar	19.11.2017	CSI-W5*	4	1.60	380	108	11	24.03.2007	M	BWP	91	5	30	9	36	2556	124	M	7	7	no	spread	oxer	right	no	0	0	48,19	no
Stuttgart	Grand Prix of Stuttgart presented by Mercedes-Benz, WALTER solar	19.11.2017	CSI-W5*	4	1.60	380	108	11	24.03.2007	M	BWP	91	5	30	9	36	2556	124	M	8	8a	yes	spread	oxer	left	no	0	0	48,19	no
Stuttgart	Grand Prix of Stuttgart presented by Mercedes-Benz, WALTER solar	19.11.2017	CSI-W5*	4	1.60	380	108	11	24.03.2007	M	BWP	91	5	30	9	36	2556	124	M	9	8b	yes	vertical	vertical	left	no	0	0	48,19	no
Stuttgart	Grand Prix of Stuttgart presented by Mercedes-Benz, WALTER solar	19.11.2017	CSI-W5*	4	1.60	380	108	11	24.03.2007	M	BWP	91	5	30	9	36	2556	124	M	10	9	no	spread	oxer	left	no	0	0	48,19	no
Stuttgart	Grand Prix of Stuttgart presented by Mercedes-Benz, WALTER solar	19.11.2017	CSI-W5*	4	1.60	410	134	10	28.04.2008	G	BWP	174	6	33	23	23	1854	199	M	1	1	no	vertical	wall	right	no	0	0	67,12	no
Stuttgart	Grand Prix of Stuttgart presented by Mercedes-Benz, WALTER solar	19.11.2017	CSI-W5*	4	1.60	410	134	10	28.04.2008	G	BWP	174	6	33	23	23	1854	199	M	2	2	no	spread	oxer	right	no	0	0	67,12	no
Stuttgart	Grand Prix of Stuttgart presented by Mercedes-Benz, WALTER solar	19.11.2017	CSI-W5*	4	1.60	410	134	10	28.04.2008	G	BWP	174	6	33	23	23	1854	199	M	3	3	no	vertical	vertical	right	no	0	0	67,12	no
Stuttgart	Grand Prix of Stuttgart presented by Mercedes-Benz, WALTER solar	19.11.2017	CSI-W5*	4	1.60	410	134	10	28.04.2008	G	BWP	174	6	33	23	23	1854	199	M	4	4	no	spread	oxer	straight	no	0	0	67,12	no
Stuttgart	Grand Prix of Stuttgart presented by Mercedes-Benz, WALTER solar	19.11.2017	CSI-W5*	4	1.60	410	134	10	28.04.2008	G	BWP	174	6	33	23	23	1854	199	M	5	5	no	spread	oxer	straight	no	0	0	67,12	no
Stuttgart	Grand Prix of Stuttgart presented by Mercedes-Benz, WALTER solar	19.11.2017	CSI-W5*	4	1.60	410	134	10	28.04.2008	G	BWP	174	6	33	23	23	1854	199	M	6	6	no	vertical	vertical	left	no	0	0	67,12	no
Stuttgart	Grand Prix of Stuttgart presented by Mercedes-Benz, WALTER solar	19.11.2017	CSI-W5*	4	1.60	410	134	10	28.04.2008	G	BWP	174	6	33	23	23	1854	199	M	7	7a	yes	spread	oxer	left	no	0	0	67,12	no
Stuttgart	Grand Prix of Stuttgart presented by Mercedes-Benz, WALTER solar	19.11.2017	CSI-W5*	4	1.60	410	134	10	28.04.2008	G	BWP	174	6	33	23	23	1854	199	M	8	7b	yes	vertical	vertical	left	no	0	0	67,12	no
Stuttgart	Grand Prix of Stuttgart presented by Mercedes-Benz, WALTER solar	19.11.2017	CSI-W5*	4	1.60	410	134	10	28.04.2008	G	BWP	174	6	33	23	23	1854	199	M	9	7c	yes	spread	oxer	left	no	0	0	67,12	no
Stuttgart	Grand Prix of Stuttgart presented by Mercedes-Benz, WALTER solar	19.11.2017	CSI-W5*	4	1.60	410	134	10	28.04.2008	G	BWP	174	6	33	23	23	1854	199	M	10	8	no	spread	oxer	left	no	0	0	67,12	no
Stuttgart	Grand Prix of Stuttgart presented by Mercedes-Benz, WALTER solar	19.11.2017	CSI-W5*	4	1.60	410	134	10	28.04.2008	G	BWP	174	6	33	23	23	1854	199	M	11	9	no	vertical	vertical	left	no	0	0	67,12	no
Stuttgart	Grand Prix of Stuttgart presented by Mercedes-Benz, WALTER solar	19.11.2017	CSI-W5*	4	1.60	410	134	10	28.04.2008	G	BWP	174	6	33	23	23	1854	199	M	12	10	no	spread	oxer	right	no	0	0	67,12	no
Stuttgart	Grand Prix of Stuttgart presented by Mercedes-Benz, WALTER solar	19.11.2017	CSI-W5*	4	1.60	410	134	10	28.04.2008	G	BWP	174	6	33	23	23	1854	199	M	13	11	no	vertical	vertical	right	no	0	0	67,12	no
Stuttgart	Grand Prix of Stuttgart presented by Mercedes-Benz, WALTER solar	19.11.2017	CSI-W5*	4	1.60	410	134	10	28.04.2008	G	BWP	174	6	33	23	23	1854	199	M	14	12a	yes	vertical	vertical	right	yes	4	0	67,12	no
Stuttgart	Grand Prix of Stuttgart presented by Mercedes-Benz, WALTER solar	19.11.2017	CSI-W5*	4	1.60	410	134	10	28.04.2008	G	BWP	174	6	33	23	23	1854	199	M	15	12b	yes	spread	oxer with water	right	no	0	0	67,12	no
Stuttgart	Grand Prix of Stuttgart presented by Mercedes-Benz, WALTER solar	19.11.2017	CSI-W5*	4	1.60	410	134	10	28.04.2008	G	BWP	174	6	33	23	23	1854	199	M	16	13	no	spread	oxer with water	straight	no	0	0	67,12	no
Stuttgart	Grand Prix of Stuttgart presented by Mercedes-Benz, WALTER solar	19.11.2017	CSI-W5*	4	1.60	410	49	14	10.06.2004	M	OS	150	2	38	10	29	1642	46	M	1	1	no	vertical	wall	right	no	0	0	68,17	no
Stuttgart	Grand Prix of Stuttgart presented by Mercedes-Benz, WALTER solar	19.11.2017	CSI-W5*	4	1.60	410	49	14	10.06.2004	M	OS	150	2	38	10	29	1642	46	M	2	2	no	spread	oxer	right	no	0	0	68,17	no
Stuttgart	Grand Prix of Stuttgart presented by Mercedes-Benz, WALTER solar	19.11.2017	CSI-W5*	4	1.60	410	49	14	10.06.2004	M	OS	150	2	38	10	29	1642	46	M	3	3	no	vertical	vertical	right	no	0	0	68,17	no
Stuttgart	Grand Prix of Stuttgart presented by Mercedes-Benz, WALTER solar	19.11.2017	CSI-W5*	4	1.60	410	49	14	10.06.2004	M	OS	150	2	38	10	29	1642	46	M	4	4	no	spread	oxer	straight	no	0	0	68,17	no
Stuttgart	Grand Prix of Stuttgart presented by Mercedes-Benz, WALTER solar	19.11.2017	CSI-W5*	4	1.60	410	49	14	10.06.2004	M	OS	150	2	38	10	29	1642	46	M	5	5	no	spread	oxer	straight	no	0	0	68,17	no
Stuttgart	Grand Prix of Stuttgart presented by Mercedes-Benz, WALTER solar	19.11.2017	CSI-W5*	4	1.60	410	49	14	10.06.2004	M	OS	150	2	38	10	29	1642	46	M	6	6	no	vertical	vertical	left	no	0	0	68,17	no
Stuttgart	Grand Prix of Stuttgart presented by Mercedes-Benz, WALTER solar	19.11.2017	CSI-W5*	4	1.60	410	49	14	10.06.2004	M	OS	150	2	38	10	29	1642	46	M	7	7a	yes	spread	oxer	left	no	0	0	68,17	no
Stuttgart	Grand Prix of Stuttgart presented by Mercedes-Benz, WALTER solar	19.11.2017	CSI-W5*	4	1.60	410	49	14	10.06.2004	M	OS	150	2	38	10	29	1642	46	M	8	7b	yes	vertical	vertical	left	no	0	0	68,17	no
Stuttgart	Grand Prix of Stuttgart presented by Mercedes-Benz, WALTER solar	19.11.2017	CSI-W5*	4	1.60	410	49	14	10.06.2004	M	OS	150	2	38	10	29	1642	46	M	9	7c	yes	spread	oxer	left	no	0	0	68,17	no
Stuttgart	Grand Prix of Stuttgart presented by Mercedes-Benz, WALTER solar	19.11.2017	CSI-W5*	4	1.60	410	49	14	10.06.2004	M	OS	150	2	38	10	29	1642	46	M	10	8	no	spread	oxer	left	no	0	0	68,17	no
Stuttgart	Grand Prix of Stuttgart presented by Mercedes-Benz, WALTER solar	19.11.2017	CSI-W5*	4	1.60	410	49	14	10.06.2004	M	OS	150	2	38	10	29	1642	46	M	11	9	no	vertical	vertical	left	no	0	0	68,17	no
Stuttgart	Grand Prix of Stuttgart presented by Mercedes-Benz, WALTER solar	19.11.2017	CSI-W5*	4	1.60	410	49	14	10.06.2004	M	OS	150	2	38	10	29	1642	46	M	12	10	no	spread	oxer	right	no	0	0	68,17	no
Stuttgart	Grand Prix of Stuttgart presented by Mercedes-Benz, WALTER solar	19.11.2017	CSI-W5*	4	1.60	410	49	14	10.06.2004	M	OS	150	2	38	10	29	1642	46	M	13	11	no	vertical	vertical	right	no	0	0	68,17	no
Stuttgart	Grand Prix of Stuttgart presented by Mercedes-Benz, WALTER solar	19.11.2017	CSI-W5*	4	1.60	410	49	14	10.06.2004	M	OS	150	2	38	10	29	1642	46	M	14	12a	yes	vertical	vertical	right	no	0	0	68,17	no
Stuttgart	Grand Prix of Stuttgart presented by Mercedes-Benz, WALTER solar	19.11.2017	CSI-W5*	4	1.60	410	49	14	10.06.2004	M	OS	150	2	38	10	29	1642	46	M	15	12b	yes	spread	oxer with water	right	no	0	0	68,17	no
Stuttgart	Grand Prix of Stuttgart presented by Mercedes-Benz, WALTER solar	19.11.2017	CSI-W5*	4	1.60	410	49	14	10.06.2004	M	OS	150	2	38	10	29	1642	46	M	16	13	no	spread	oxer with water	straight	no	0	0	68,17	no
Stuttgart	Grand Prix of Stuttgart presented by Mercedes-Benz, WALTER solar	19.11.2017	CSI-W5*	4	1.60	380	49	14	10.06.2004	M	OS	150	2	38	10	29	1642	46	M	1	1	no	spread	oxer	left	no	0	0	56,54	no
Stuttgart	Grand Prix of Stuttgart presented by Mercedes-Benz, WALTER solar	19.11.2017	CSI-W5*	4	1.60	380	49	14	10.06.2004	M	OS	150	2	38	10	29	1642	46	M	2	2	no	vertical	vertical	left	no	0	0	56,54	no
Stuttgart	Grand Prix of Stuttgart presented by Mercedes-Benz, WALTER solar	19.11.2017	CSI-W5*	4	1.60	380	49	14	10.06.2004	M	OS	150	2	38	10	29	1642	46	M	3	3	no	vertical	vertical	right	no	0	0	56,54	no
Stuttgart	Grand Prix of Stuttgart presented by Mercedes-Benz, WALTER solar	19.11.2017	CSI-W5*	4	1.60	380	49	14	10.06.2004	M	OS	150	2	38	10	29	1642	46	M	4	4	no	spread	oxer with water	right	no	0	0	56,54	no
Stuttgart	Grand Prix of Stuttgart presented by Mercedes-Benz, WALTER solar	19.11.2017	CSI-W5*	4	1.60	380	49	14	10.06.2004	M	OS	150	2	38	10	29	1642	46	M	5	5	no	vertical	vertical	left	no	0	0	56,54	no
Stuttgart	Grand Prix of Stuttgart presented by Mercedes-Benz, WALTER solar	19.11.2017	CSI-W5*	4	1.60	380	49	14	10.06.2004	M	OS	150	2	38	10	29	1642	46	M	6	6	no	vertical	wall	right	no	0	0	56,54	no
Stuttgart	Grand Prix of Stuttgart presented by Mercedes-Benz, WALTER solar	19.11.2017	CSI-W5*	4	1.60	380	49	14	10.06.2004	M	OS	150	2	38	10	29	1642	46	M	7	7	no	spread	oxer	right	no	0	0	56,54	no
Stuttgart	Grand Prix of Stuttgart presented by Mercedes-Benz, WALTER solar	19.11.2017	CSI-W5*	4	1.60	380	49	14	10.06.2004	M	OS	150	2	38	10	29	1642	46	M	8	8a	yes	spread	oxer	left	no	0	0	56,54	no
Stuttgart	Grand Prix of Stuttgart presented by Mercedes-Benz, WALTER solar	19.11.2017	CSI-W5*	4	1.60	380	49	14	10.06.2004	M	OS	150	2	38	10	29	1642	46	M	9	8b	yes	vertical	vertical	left	no	0	0	56,54	no
Stuttgart	Grand Prix of Stuttgart presented by Mercedes-Benz, WALTER solar	19.11.2017	CSI-W5*	4	1.60	380	49	14	10.06.2004	M	OS	150	2	38	10	29	1642	46	M	10	9	no	spread	oxer	left	no	0	0	56,54	no
Stuttgart	Grand Prix of Stuttgart presented by Mercedes-Benz, WALTER solar	19.11.2017	CSI-W5*	4	1.60	410	208	10	18.05.2008	G	SF	126	1	43	134	48	1808	64	M	1	1	no	vertical	wall	right	no	0	0	69,36	no
Stuttgart	Grand Prix of Stuttgart presented by Mercedes-Benz, WALTER solar	19.11.2017	CSI-W5*	4	1.60	410	208	10	18.05.2008	G	SF	126	1	43	134	48	1808	64	M	2	2	no	spread	oxer	right	no	0	0	69,36	no
Stuttgart	Grand Prix of Stuttgart presented by Mercedes-Benz, WALTER solar	19.11.2017	CSI-W5*	4	1.60	410	208	10	18.05.2008	G	SF	126	1	43	134	48	1808	64	M	3	3	no	vertical	vertical	right	no	0	0	69,36	no
Stuttgart	Grand Prix of Stuttgart presented by Mercedes-Benz, WALTER solar	19.11.2017	CSI-W5*	4	1.60	410	208	10	18.05.2008	G	SF	126	1	43	134	48	1808	64	M	4	4	no	spread	oxer	straight	no	0	0	69,36	no
Stuttgart	Grand Prix of Stuttgart presented by Mercedes-Benz, WALTER solar	19.11.2017	CSI-W5*	4	1.60	410	208	10	18.05.2008	G	SF	126	1	43	134	48	1808	64	M	5	5	no	spread	oxer	straight	no	0	0	69,36	no
Stuttgart	Grand Prix of Stuttgart presented by Mercedes-Benz, WALTER solar	19.11.2017	CSI-W5*	4	1.60	410	208	10	18.05.2008	G	SF	126	1	43	134	48	1808	64	M	6	6	no	vertical	vertical	left	no	0	0	69,36	no
Stuttgart	Grand Prix of Stuttgart presented by Mercedes-Benz, WALTER solar	19.11.2017	CSI-W5*	4	1.60	410	208	10	18.05.2008	G	SF	126	1	43	134	48	1808	64	M	7	7a	yes	spread	oxer	left	no	0	0	69,36	no
Stuttgart	Grand Prix of Stuttgart presented by Mercedes-Benz, WALTER solar	19.11.2017	CSI-W5*	4	1.60	410	208	10	18.05.2008	G	SF	126	1	43	134	48	1808	64	M	8	7b	yes	vertical	vertical	left	no	0	0	69,36	no
Stuttgart	Grand Prix of Stuttgart presented by Mercedes-Benz, WALTER solar	19.11.2017	CSI-W5*	4	1.60	410	208	10	18.05.2008	G	SF	126	1	43	134	48	1808	64	M	9	7c	yes	spread	oxer	left	no	0	0	69,36	no
Stuttgart	Grand Prix of Stuttgart presented by Mercedes-Benz, WALTER solar	19.11.2017	CSI-W5*	4	1.60	410	208	10	18.05.2008	G	SF	126	1	43	134	48	1808	64	M	10	8	no	spread	oxer	left	no	0	0	69,36	no
Stuttgart	Grand Prix of Stuttgart presented by Mercedes-Benz, WALTER solar	19.11.2017	CSI-W5*	4	1.60	410	208	10	18.05.2008	G	SF	126	1	43	134	48	1808	64	M	11	9	no	vertical	vertical	left	no	0	0	69,36	no
Stuttgart	Grand Prix of Stuttgart presented by Mercedes-Benz, WALTER solar	19.11.2017	CSI-W5*	4	1.60	410	208	10	18.05.2008	G	SF	126	1	43	134	48	1808	64	M	12	10	no	spread	oxer	right	no	0	0	69,36	no
Stuttgart	Grand Prix of Stuttgart presented by Mercedes-Benz, WALTER solar	19.11.2017	CSI-W5*	4	1.60	410	208	10	18.05.2008	G	SF	126	1	43	134	48	1808	64	M	13	11	no	vertical	vertical	right	no	0	0	69,36	no
Stuttgart	Grand Prix of Stuttgart presented by Mercedes-Benz, WALTER solar	19.11.2017	CSI-W5*	4	1.60	410	208	10	18.05.2008	G	SF	126	1	43	134	48	1808	64	M	14	12a	yes	vertical	vertical	right	no	0	0	69,36	no
Stuttgart	Grand Prix of Stuttgart presented by Mercedes-Benz, WALTER solar	19.11.2017	CSI-W5*	4	1.60	410	208	10	18.05.2008	G	SF	126	1	43	134	48	1808	64	M	15	12b	yes	spread	oxer with water	right	no	0	0	69,36	no
Stuttgart	Grand Prix of Stuttgart presented by Mercedes-Benz, WALTER solar	19.11.2017	CSI-W5*	4	1.60	410	208	10	18.05.2008	G	SF	126	1	43	134	48	1808	64	M	16	13	no	spread	oxer with water	straight	no	0	0	69,36	no
Stuttgart	Grand Prix of Stuttgart presented by Mercedes-Benz, WALTER solar	19.11.2017	CSI-W5*	4	1.60	380	208	10	18.05.2008	G	SF	126	1	43	134	48	1808	64	M	1	1	no	spread	oxer	left	no	0	0	62,92	no
Stuttgart	Grand Prix of Stuttgart presented by Mercedes-Benz, WALTER solar	19.11.2017	CSI-W5*	4	1.60	380	208	10	18.05.2008	G	SF	126	1	43	134	48	1808	64	M	2	2	no	vertical	vertical	left	no	0	0	62,92	no
Stuttgart	Grand Prix of Stuttgart presented by Mercedes-Benz, WALTER solar	19.11.2017	CSI-W5*	4	1.60	380	208	10	18.05.2008	G	SF	126	1	43	134	48	1808	64	M	3	3	no	vertical	vertical	right	no	0	0	62,92	no
Stuttgart	Grand Prix of Stuttgart presented by Mercedes-Benz, WALTER solar	19.11.2017	CSI-W5*	4	1.60	380	208	10	18.05.2008	G	SF	126	1	43	134	48	1808	64	M	4	4	no	spread	oxer with water	right	no	0	0	62,92	no
Stuttgart	Grand Prix of Stuttgart presented by Mercedes-Benz, WALTER solar	19.11.2017	CSI-W5*	4	1.60	380	208	10	18.05.2008	G	SF	126	1	43	134	48	1808	64	M	5	5	no	vertical	vertical	left	no	0	0	62,92	no
Stuttgart	Grand Prix of Stuttgart presented by Mercedes-Benz, WALTER solar	19.11.2017	CSI-W5*	4	1.60	380	208	10	18.05.2008	G	SF	126	1	43	134	48	1808	64	M	6	6	no	vertical	wall	right	no	0	0	62,92	no
Stuttgart	Grand Prix of Stuttgart presented by Mercedes-Benz, WALTER solar	19.11.2017	CSI-W5*	4	1.60	380	208	10	18.05.2008	G	SF	126	1	43	134	48	1808	64	M	7	7	no	spread	oxer	right	no	0	0	62,92	no
Stuttgart	Grand Prix of Stuttgart presented by Mercedes-Benz, WALTER solar	19.11.2017	CSI-W5*	4	1.60	380	208	10	18.05.2008	G	SF	126	1	43	134	48	1808	64	M	8	8a	yes	spread	oxer	left	no	0	0	62,92	no
Stuttgart	Grand Prix of Stuttgart presented by Mercedes-Benz, WALTER solar	19.11.2017	CSI-W5*	4	1.60	380	208	10	18.05.2008	G	SF	126	1	43	134	48	1808	64	M	9	8b	yes	vertical	vertical	left	no	0	0	62,92	no
Stuttgart	Grand Prix of Stuttgart presented by Mercedes-Benz, WALTER solar	19.11.2017	CSI-W5*	4	1.60	380	208	10	18.05.2008	G	SF	126	1	43	134	48	1808	64	M	10	9	no	spread	oxer	left	no	0	0	62,92	no
Stuttgart	Grand Prix of Stuttgart presented by Mercedes-Benz, WALTER solar	19.11.2017	CSI-W5*	4	1.60	410	42	11	01.01.2007	S	ISH	143	5	44	135	22	458	12	M	1	1	no	vertical	wall	right	no	0	0	68,03	no
Stuttgart	Grand Prix of Stuttgart presented by Mercedes-Benz, WALTER solar	19.11.2017	CSI-W5*	4	1.60	410	42	11	01.01.2007	S	ISH	143	5	44	135	22	458	12	M	2	2	no	spread	oxer	right	no	0	0	68,03	no
Stuttgart	Grand Prix of Stuttgart presented by Mercedes-Benz, WALTER solar	19.11.2017	CSI-W5*	4	1.60	410	42	11	01.01.2007	S	ISH	143	5	44	135	22	458	12	M	3	3	no	vertical	vertical	right	no	0	0	68,03	no
Stuttgart	Grand Prix of Stuttgart presented by Mercedes-Benz, WALTER solar	19.11.2017	CSI-W5*	4	1.60	410	42	11	01.01.2007	S	ISH	143	5	44	135	22	458	12	M	4	4	no	spread	oxer	straight	no	0	0	68,03	no
Stuttgart	Grand Prix of Stuttgart presented by Mercedes-Benz, WALTER solar	19.11.2017	CSI-W5*	4	1.60	410	42	11	01.01.2007	S	ISH	143	5	44	135	22	458	12	M	5	5	no	spread	oxer	straight	no	0	0	68,03	no
Stuttgart	Grand Prix of Stuttgart presented by Mercedes-Benz, WALTER solar	19.11.2017	CSI-W5*	4	1.60	410	42	11	01.01.2007	S	ISH	143	5	44	135	22	458	12	M	6	6	no	vertical	vertical	left	no	0	0	68,03	no
Stuttgart	Grand Prix of Stuttgart presented by Mercedes-Benz, WALTER solar	19.11.2017	CSI-W5*	4	1.60	410	42	11	01.01.2007	S	ISH	143	5	44	135	22	458	12	M	7	7a	yes	spread	oxer	left	no	0	0	68,03	no
Stuttgart	Grand Prix of Stuttgart presented by Mercedes-Benz, WALTER solar	19.11.2017	CSI-W5*	4	1.60	410	42	11	01.01.2007	S	ISH	143	5	44	135	22	458	12	M	8	7b	yes	vertical	vertical	left	no	0	0	68,03	no
Stuttgart	Grand Prix of Stuttgart presented by Mercedes-Benz, WALTER solar	19.11.2017	CSI-W5*	4	1.60	410	42	11	01.01.2007	S	ISH	143	5	44	135	22	458	12	M	9	7c	yes	spread	oxer	left	no	0	0	68,03	no
Stuttgart	Grand Prix of Stuttgart presented by Mercedes-Benz, WALTER solar	19.11.2017	CSI-W5*	4	1.60	410	42	11	01.01.2007	S	ISH	143	5	44	135	22	458	12	M	10	8	no	spread	oxer	left	no	0	0	68,03	no
Stuttgart	Grand Prix of Stuttgart presented by Mercedes-Benz, WALTER solar	19.11.2017	CSI-W5*	4	1.60	410	42	11	01.01.2007	S	ISH	143	5	44	135	22	458	12	M	11	9	no	vertical	vertical	left	no	0	0	68,03	no
Stuttgart	Grand Prix of Stuttgart presented by Mercedes-Benz, WALTER solar	19.11.2017	CSI-W5*	4	1.60	410	42	11	01.01.2007	S	ISH	143	5	44	135	22	458	12	M	12	10	no	spread	oxer	right	no	0	0	68,03	no
Stuttgart	Grand Prix of Stuttgart presented by Mercedes-Benz, WALTER solar	19.11.2017	CSI-W5*	4	1.60	410	42	11	01.01.2007	S	ISH	143	5	44	135	22	458	12	M	13	11	no	vertical	vertical	right	no	0	0	68,03	no
Stuttgart	Grand Prix of Stuttgart presented by Mercedes-Benz, WALTER solar	19.11.2017	CSI-W5*	4	1.60	410	42	11	01.01.2007	S	ISH	143	5	44	135	22	458	12	M	14	12a	yes	vertical	vertical	right	yes	4	0	68,03	no
Stuttgart	Grand Prix of Stuttgart presented by Mercedes-Benz, WALTER solar	19.11.2017	CSI-W5*	4	1.60	410	42	11	01.01.2007	S	ISH	143	5	44	135	22	458	12	M	15	12b	yes	spread	oxer with water	right	no	0	0	68,03	no
Stuttgart	Grand Prix of Stuttgart presented by Mercedes-Benz, WALTER solar	19.11.2017	CSI-W5*	4	1.60	410	42	11	01.01.2007	S	ISH	143	5	44	135	22	458	12	M	16	13	no	spread	oxer with water	straight	no	0	0	68,03	no
Stuttgart	Grand Prix of Stuttgart presented by Mercedes-Benz, WALTER solar	19.11.2017	CSI-W5*	4	1.60	410	140	11	19.03.2007	S	HANN	162	14	47	44	44	1933	114	M	1	1	no	vertical	wall	right	no	0	0	68,52	no
Stuttgart	Grand Prix of Stuttgart presented by Mercedes-Benz, WALTER solar	19.11.2017	CSI-W5*	4	1.60	410	140	11	19.03.2007	S	HANN	162	14	47	44	44	1933	114	M	2	2	no	spread	oxer	right	no	0	0	68,52	no
Stuttgart	Grand Prix of Stuttgart presented by Mercedes-Benz, WALTER solar	19.11.2017	CSI-W5*	4	1.60	410	140	11	19.03.2007	S	HANN	162	14	47	44	44	1933	114	M	3	3	no	vertical	vertical	right	no	0	0	68,52	no
Stuttgart	Grand Prix of Stuttgart presented by Mercedes-Benz, WALTER solar	19.11.2017	CSI-W5*	4	1.60	410	140	11	19.03.2007	S	HANN	162	14	47	44	44	1933	114	M	4	4	no	spread	oxer	straight	yes	4	0	68,52	no
Stuttgart	Grand Prix of Stuttgart presented by Mercedes-Benz, WALTER solar	19.11.2017	CSI-W5*	4	1.60	410	140	11	19.03.2007	S	HANN	162	14	47	44	44	1933	114	M	5	5	no	spread	oxer	straight	no	0	0	68,52	no
Stuttgart	Grand Prix of Stuttgart presented by Mercedes-Benz, WALTER solar	19.11.2017	CSI-W5*	4	1.60	410	140	11	19.03.2007	S	HANN	162	14	47	44	44	1933	114	M	6	6	no	vertical	vertical	left	no	0	0	68,52	no
Stuttgart	Grand Prix of Stuttgart presented by Mercedes-Benz, WALTER solar	19.11.2017	CSI-W5*	4	1.60	410	140	11	19.03.2007	S	HANN	162	14	47	44	44	1933	114	M	7	7a	yes	spread	oxer	left	no	0	0	68,52	no
Stuttgart	Grand Prix of Stuttgart presented by Mercedes-Benz, WALTER solar	19.11.2017	CSI-W5*	4	1.60	410	140	11	19.03.2007	S	HANN	162	14	47	44	44	1933	114	M	8	7b	yes	vertical	vertical	left	no	0	0	68,52	no
Stuttgart	Grand Prix of Stuttgart presented by Mercedes-Benz, WALTER solar	19.11.2017	CSI-W5*	4	1.60	410	140	11	19.03.2007	S	HANN	162	14	47	44	44	1933	114	M	9	7c	yes	spread	oxer	left	no	0	0	68,52	no
Stuttgart	Grand Prix of Stuttgart presented by Mercedes-Benz, WALTER solar	19.11.2017	CSI-W5*	4	1.60	410	140	11	19.03.2007	S	HANN	162	14	47	44	44	1933	114	M	10	8	no	spread	oxer	left	no	0	0	68,52	no
Stuttgart	Grand Prix of Stuttgart presented by Mercedes-Benz, WALTER solar	19.11.2017	CSI-W5*	4	1.60	410	140	11	19.03.2007	S	HANN	162	14	47	44	44	1933	114	M	11	9	no	vertical	vertical	left	no	0	0	68,52	no
Stuttgart	Grand Prix of Stuttgart presented by Mercedes-Benz, WALTER solar	19.11.2017	CSI-W5*	4	1.60	410	140	11	19.03.2007	S	HANN	162	14	47	44	44	1933	114	M	12	10	no	spread	oxer	right	no	0	0	68,52	no
Stuttgart	Grand Prix of Stuttgart presented by Mercedes-Benz, WALTER solar	19.11.2017	CSI-W5*	4	1.60	410	140	11	19.03.2007	S	HANN	162	14	47	44	44	1933	114	M	13	11	no	vertical	vertical	right	no	0	0	68,52	no
Stuttgart	Grand Prix of Stuttgart presented by Mercedes-Benz, WALTER solar	19.11.2017	CSI-W5*	4	1.60	410	140	11	19.03.2007	S	HANN	162	14	47	44	44	1933	114	M	14	12a	yes	vertical	vertical	right	no	0	0	68,52	no
Stuttgart	Grand Prix of Stuttgart presented by Mercedes-Benz, WALTER solar	19.11.2017	CSI-W5*	4	1.60	410	140	11	19.03.2007	S	HANN	162	14	47	44	44	1933	114	M	15	12b	yes	spread	oxer with water	right	no	0	0	68,52	no
Stuttgart	Grand Prix of Stuttgart presented by Mercedes-Benz, WALTER solar	19.11.2017	CSI-W5*	4	1.60	410	140	11	19.03.2007	S	HANN	162	14	47	44	44	1933	114	M	16	13	no	spread	oxer with water	straight	no	0	0	68,52	no
Stuttgart	Grand Prix of Stuttgart presented by Mercedes-Benz, WALTER solar	19.11.2017	CSI-W5*	4	1.60	410	11	12	13.03.2006	M	KWPN	211	7	48	40	39	1040	33	M	1	1	no	vertical	wall	right	no	0	0	67,21	no
Stuttgart	Grand Prix of Stuttgart presented by Mercedes-Benz, WALTER solar	19.11.2017	CSI-W5*	4	1.60	410	11	12	13.03.2006	M	KWPN	211	7	48	40	39	1040	33	M	2	2	no	spread	oxer	right	no	0	0	67,21	no
Stuttgart	Grand Prix of Stuttgart presented by Mercedes-Benz, WALTER solar	19.11.2017	CSI-W5*	4	1.60	410	11	12	13.03.2006	M	KWPN	211	7	48	40	39	1040	33	M	3	3	no	vertical	vertical	right	no	0	0	67,21	no
Stuttgart	Grand Prix of Stuttgart presented by Mercedes-Benz, WALTER solar	19.11.2017	CSI-W5*	4	1.60	410	11	12	13.03.2006	M	KWPN	211	7	48	40	39	1040	33	M	4	4	no	spread	oxer	straight	no	0	0	67,21	no
Stuttgart	Grand Prix of Stuttgart presented by Mercedes-Benz, WALTER solar	19.11.2017	CSI-W5*	4	1.60	410	11	12	13.03.2006	M	KWPN	211	7	48	40	39	1040	33	M	5	5	no	spread	oxer	straight	no	0	0	67,21	no
Stuttgart	Grand Prix of Stuttgart presented by Mercedes-Benz, WALTER solar	19.11.2017	CSI-W5*	4	1.60	410	11	12	13.03.2006	M	KWPN	211	7	48	40	39	1040	33	M	6	6	no	vertical	vertical	left	no	0	0	67,21	no
Stuttgart	Grand Prix of Stuttgart presented by Mercedes-Benz, WALTER solar	19.11.2017	CSI-W5*	4	1.60	410	11	12	13.03.2006	M	KWPN	211	7	48	40	39	1040	33	M	7	7a	yes	spread	oxer	left	no	0	0	67,21	no
Stuttgart	Grand Prix of Stuttgart presented by Mercedes-Benz, WALTER solar	19.11.2017	CSI-W5*	4	1.60	410	11	12	13.03.2006	M	KWPN	211	7	48	40	39	1040	33	M	8	7b	yes	vertical	vertical	left	no	0	0	67,21	no
Stuttgart	Grand Prix of Stuttgart presented by Mercedes-Benz, WALTER solar	19.11.2017	CSI-W5*	4	1.60	410	11	12	13.03.2006	M	KWPN	211	7	48	40	39	1040	33	M	9	7c	yes	spread	oxer	left	no	0	0	67,21	no
Stuttgart	Grand Prix of Stuttgart presented by Mercedes-Benz, WALTER solar	19.11.2017	CSI-W5*	4	1.60	410	11	12	13.03.2006	M	KWPN	211	7	48	40	39	1040	33	M	10	8	no	spread	oxer	left	no	0	0	67,21	no
Stuttgart	Grand Prix of Stuttgart presented by Mercedes-Benz, WALTER solar	19.11.2017	CSI-W5*	4	1.60	410	11	12	13.03.2006	M	KWPN	211	7	48	40	39	1040	33	M	11	9	no	vertical	vertical	left	no	0	0	67,21	no
Stuttgart	Grand Prix of Stuttgart presented by Mercedes-Benz, WALTER solar	19.11.2017	CSI-W5*	4	1.60	410	11	12	13.03.2006	M	KWPN	211	7	48	40	39	1040	33	M	12	10	no	spread	oxer	right	no	0	0	67,21	no
Stuttgart	Grand Prix of Stuttgart presented by Mercedes-Benz, WALTER solar	19.11.2017	CSI-W5*	4	1.60	410	11	12	13.03.2006	M	KWPN	211	7	48	40	39	1040	33	M	13	11	no	vertical	vertical	right	no	0	0	67,21	no
Stuttgart	Grand Prix of Stuttgart presented by Mercedes-Benz, WALTER solar	19.11.2017	CSI-W5*	4	1.60	410	11	12	13.03.2006	M	KWPN	211	7	48	40	39	1040	33	M	14	12a	yes	vertical	vertical	right	no	0	0	67,21	no
Stuttgart	Grand Prix of Stuttgart presented by Mercedes-Benz, WALTER solar	19.11.2017	CSI-W5*	4	1.60	410	11	12	13.03.2006	M	KWPN	211	7	48	40	39	1040	33	M	15	12b	yes	spread	oxer with water	right	no	0	0	67,21	no
Stuttgart	Grand Prix of Stuttgart presented by Mercedes-Benz, WALTER solar	19.11.2017	CSI-W5*	4	1.60	410	11	12	13.03.2006	M	KWPN	211	7	48	40	39	1040	33	M	16	13	no	spread	oxer with water	straight	no	0	0	67,21	no

Stuttgart	Grand Prix of Stuttgart presented by Mercedes-Benz, WALTER solar	19.11.2017	CSI-W5*	4	1.60	380	11	12	13.03.2006	M	KWPN	211	7	48	40	39	1040	33	M	1	1	no	spread	oxer	left	no	0	0	50,86	no
Stuttgart	Grand Prix of Stuttgart presented by Mercedes-Benz, WALTER solar	19.11.2017	CSI-W5*	4	1.60	380	11	12	13.03.2006	M	KWPN	211	7	48	40	39	1040	33	M	2	2	no	vertical	vertical	left	no	0	0	50,86	no
Stuttgart	Grand Prix of Stuttgart presented by Mercedes-Benz, WALTER solar	19.11.2017	CSI-W5*	4	1.60	380	11	12	13.03.2006	M	KWPN	211	7	48	40	39	1040	33	M	3	3	no	vertical	vertical	right	no	0	0	50,86	no
Stuttgart	Grand Prix of Stuttgart presented by Mercedes-Benz, WALTER solar	19.11.2017	CSI-W5*	4	1.60	380	11	12	13.03.2006	M	KWPN	211	7	48	40	39	1040	33	M	4	4	no	spread	oxer with water	right	no	0	0	50,86	no
Stuttgart	Grand Prix of Stuttgart presented by Mercedes-Benz, WALTER solar	19.11.2017	CSI-W5*	4	1.60	380	11	12	13.03.2006	M	KWPN	211	7	48	40	39	1040	33	M	5	5	no	vertical	vertical	left	no	0	0	50,86	no
Stuttgart	Grand Prix of Stuttgart presented by Mercedes-Benz, WALTER solar	19.11.2017	CSI-W5*	4	1.60	380	11	12	13.03.2006	M	KWPN	211	7	48	40	39	1040	33	M	6	6	no	vertical	wall	right	no	0	0	50,86	no
Stuttgart	Grand Prix of Stuttgart presented by Mercedes-Benz, WALTER solar	19.11.2017	CSI-W5*	4	1.60	380	11	12	13.03.2006	M	KWPN	211	7	48	40	39	1040	33	M	7	7	no	spread	oxer	right	no	0	0	50,86	no
Stuttgart	Grand Prix of Stuttgart presented by Mercedes-Benz, WALTER solar	19.11.2017	CSI-W5*	4	1.60	380	11	12	13.03.2006	M	KWPN	211	7	48	40	39	1040	33	M	8	8a	yes	spread	oxer	left	no	0	0	50,86	no
Stuttgart	Grand Prix of Stuttgart presented by Mercedes-Benz, WALTER solar	19.11.2017	CSI-W5*	4	1.60	380	11	12	13.03.2006	M	KWPN	211	7	48	40	39	1040	33	M	9	8b	yes	vertical	vertical	left	no	0	0	50,86	no
Stuttgart	Grand Prix of Stuttgart presented by Mercedes-Benz, WALTER solar	19.11.2017	CSI-W5*	4	1.60	380	11	12	13.03.2006	M	KWPN	211	7	48	40	39	1040	33	M	10	9	no	spread	oxer	left	no	0	0	50,86	no
Stuttgart	Grand Prix of Stuttgart presented by Mercedes-Benz, WALTER solar	19.11.2017	CSI-W5*	4	1.60	410	72	11	15.02.2007	M	DSP	109	11	49	109	29	577	25	F	1	1	no	vertical	wall	right	no	0	0	66,26	no
Stuttgart	Grand Prix of Stuttgart presented by Mercedes-Benz, WALTER solar	19.11.2017	CSI-W5*	4	1.60	410	72	11	15.02.2007	M	DSP	109	11	49	109	29	577	25	F	2	2	no	spread	oxer	right	no	0	0	66,26	no
Stuttgart	Grand Prix of Stuttgart presented by Mercedes-Benz, WALTER solar	19.11.2017	CSI-W5*	4	1.60	410	72	11	15.02.2007	M	DSP	109	11	49	109	29	577	25	F	3	3	no	vertical	vertical	right	no	0	0	66,26	no
Stuttgart	Grand Prix of Stuttgart presented by Mercedes-Benz, WALTER solar	19.11.2017	CSI-W5*	4	1.60	410	72	11	15.02.2007	M	DSP	109	11	49	109	29	577	25	F	4	4	no	spread	oxer	straight	no	0	0	66,26	no
Stuttgart	Grand Prix of Stuttgart presented by Mercedes-Benz, WALTER solar	19.11.2017	CSI-W5*	4	1.60	410	72	11	15.02.2007	M	DSP	109	11	49	109	29	577	25	F	5	5	no	spread	oxer	straight	no	0	0	66,26	no
Stuttgart	Grand Prix of Stuttgart presented by Mercedes-Benz, WALTER solar	19.11.2017	CSI-W5*	4	1.60	410	72	11	15.02.2007	M	DSP	109	11	49	109	29	577	25	F	6	6	no	vertical	vertical	left	no	0	0	66,26	no
Stuttgart	Grand Prix of Stuttgart presented by Mercedes-Benz, WALTER solar	19.11.2017	CSI-W5*	4	1.60	410	72	11	15.02.2007	M	DSP	109	11	49	109	29	577	25	F	7	7a	yes	spread	oxer	left	no	0	0	66,26	no
Stuttgart	Grand Prix of Stuttgart presented by Mercedes-Benz, WALTER solar	19.11.2017	CSI-W5*	4	1.60	410	72	11	15.02.2007	M	DSP	109	11	49	109	29	577	25	F	8	7b	yes	vertical	vertical	left	no	0	0	66,26	no
Stuttgart	Grand Prix of Stuttgart presented by Mercedes-Benz, WALTER solar	19.11.2017	CSI-W5*	4	1.60	410	72	11	15.02.2007	M	DSP	109	11	49	109	29	577	25	F	9	7c	yes	spread	oxer	left	no	0	0	66,26	no
Stuttgart	Grand Prix of Stuttgart presented by Mercedes-Benz, WALTER solar	19.11.2017	CSI-W5*	4	1.60	410	72	11	15.02.2007	M	DSP	109	11	49	109	29	577	25	F	10	8	no	spread	oxer	left	no	0	0	66,26	no
Stuttgart	Grand Prix of Stuttgart presented by Mercedes-Benz, WALTER solar	19.11.2017	CSI-W5*	4	1.60	410	72	11	15.02.2007	M	DSP	109	11	49	109	29	577	25	F	11	9	no	vertical	vertical	left	no	0	0	66,26	no
Stuttgart	Grand Prix of Stuttgart presented by Mercedes-Benz, WALTER solar	19.11.2017	CSI-W5*	4	1.60	410	72	11	15.02.2007	M	DSP	109	11	49	109	29	577	25	F	12	10	no	spread	oxer	right	no	0	0	66,26	no
Stuttgart	Grand Prix of Stuttgart presented by Mercedes-Benz, WALTER solar	19.11.2017	CSI-W5*	4	1.60	410	72	11	15.02.2007	M	DSP	109	11	49	109	29	577	25	F	13	11	no	vertical	vertical	right	no	0	0	66,26	no
Stuttgart	Grand Prix of Stuttgart presented by Mercedes-Benz, WALTER solar	19.11.2017	CSI-W5*	4	1.60	410	72	11	15.02.2007	M	DSP	109	11	49	109	29	577	25	F	14	12a	yes	vertical	vertical	right	no	0	0	66,26	no
Stuttgart	Grand Prix of Stuttgart presented by Mercedes-Benz, WALTER solar	19.11.2017	CSI-W5*	4	1.60	410	72	11	15.02.2007	M	DSP	109	11	49	109	29	577	25	F	15	12b	yes	spread	oxer with water	right	no	0	0	66,26	no
Stuttgart	Grand Prix of Stuttgart presented by Mercedes-Benz, WALTER solar	19.11.2017	CSI-W5*	4	1.60	410	72	11	15.02.2007	M	DSP	109	11	49	109	29	577	25	F	16	13	no	spread	oxer with water	straight	no	0	0	66,26	no
Stuttgart	Grand Prix of Stuttgart presented by Mercedes-Benz, WALTER solar	19.11.2017	CSI-W5*	4	1.60	380	72	11	15.02.2007	M	DSP	109	11	49	109	29	577	25	F	1	1	no	spread	oxer	left	no	0	0	50,14	no
Stuttgart	Grand Prix of Stuttgart presented by Mercedes-Benz, WALTER solar	19.11.2017	CSI-W5*	4	1.60	380	72	11	15.02.2007	M	DSP	109	11	49	109	29	577	25	F	2	2	no	vertical	vertical	left	no	0	0	50,14	no
Stuttgart	Grand Prix of Stuttgart presented by Mercedes-Benz, WALTER solar	19.11.2017	CSI-W5*	4	1.60	380	72	11	15.02.2007	M	DSP	109	11	49	109	29	577	25	F	3	3	no	vertical	vertical	right	no	0	0	50,14	no
Stuttgart	Grand Prix of Stuttgart presented by Mercedes-Benz, WALTER solar	19.11.2017	CSI-W5*	4	1.60	380	72	11	15.02.2007	M	DSP	109	11	49	109	29	577	25	F	4	4	no	spread	oxer with water	right	no	0	0	50,14	no
Stuttgart	Grand Prix of Stuttgart presented by Mercedes-Benz, WALTER solar	19.11.2017	CSI-W5*	4	1.60	380	72	11	15.02.2007	M	DSP	109	11	49	109	29	577	25	F	5	5	no	vertical	vertical	left	no	0	0	50,14	no
Stuttgart	Grand Prix of Stuttgart presented by Mercedes-Benz, WALTER solar	19.11.2017	CSI-W5*	4	1.60	380	72	11	15.02.2007	M	DSP	109	11	49	109	29	577	25	F	6	6	no	vertical	wall	right	no	0	0	50,14	no
Stuttgart	Grand Prix of Stuttgart presented by Mercedes-Benz, WALTER solar	19.11.2017	CSI-W5*	4	1.60	380	72	11	15.02.2007	M	DSP	109	11	49	109	29	577	25	F	7	7	no	spread	oxer	right	no	0	0	50,14	no
Stuttgart	Grand Prix of Stuttgart presented by Mercedes-Benz, WALTER solar	19.11.2017	CSI-W5*	4	1.60	380	72	11	15.02.2007	M	DSP	109	11	49	109	29	577	25	F	8	8a	yes	spread	oxer	left	no	0	0	50,14	no
Stuttgart	Grand Prix of Stuttgart presented by Mercedes-Benz, WALTER solar	19.11.2017	CSI-W5*	4	1.60	380	72	11	15.02.2007	M	DSP	109	11	49	109	29	577	25	F	9	8b	yes	vertical	vertical	left	no	0	0	50,14	no
Stuttgart	Grand Prix of Stuttgart presented by Mercedes-Benz, WALTER solar	19.11.2017	CSI-W5*	4	1.60	380	72	11	15.02.2007	M	DSP	109	11	49	109	29	577	25	F	10	9	no	spread	oxer	left	no	0	0	50,14	no
Stuttgart	Grand Prix of Stuttgart presented by Mercedes-Benz, WALTER solar	19.11.2017	CSI-W5*	4	1.60	410	105	12	02.04.2006	M	DSP	186	8	50	30	25	1981	78	M	1	1	no	vertical	wall	right	no	0	0	65,02	no
Stuttgart	Grand Prix of Stuttgart presented by Mercedes-Benz, WALTER solar	19.11.2017	CSI-W5*	4	1.60	410	105	12	02.04.2006	M	DSP	186	8	50	30	25	1981	78	M	2	2	no	spread	oxer	right	no	0	0	65,02	no
Stuttgart	Grand Prix of Stuttgart presented by Mercedes-Benz, WALTER solar	19.11.2017	CSI-W5*	4	1.60	410	105	12	02.04.2006	M	DSP	186	8	50	30	25	1981	78	M	3	3	no	vertical	vertical	right	no	0	0	65,02	no
Stuttgart	Grand Prix of Stuttgart presented by Mercedes-Benz, WALTER solar	19.11.2017	CSI-W5*	4	1.60	410	105	12	02.04.2006	M	DSP	186	8	50	30	25	1981	78	M	4	4	no	spread	oxer	straight	no	0	0	65,02	no
Stuttgart	Grand Prix of Stuttgart presented by Mercedes-Benz, WALTER solar	19.11.2017	CSI-W5*	4	1.60	410	105	12	02.04.2006	M	DSP	186	8	50	30	25	1981	78	M	5	5	no	spread	oxer	straight	no	0	0	65,02	no
Stuttgart	Grand Prix of Stuttgart presented by Mercedes-Benz, WALTER solar	19.11.2017	CSI-W5*	4	1.60	410	105	12	02.04.2006	M	DSP	186	8	50	30	25	1981	78	M	6	6	no	vertical	vertical	left	no	0	0	65,02	no
Stuttgart	Grand Prix of Stuttgart presented by Mercedes-Benz, WALTER solar	19.11.2017	CSI-W5*	4	1.60	410	105	12	02.04.2006	M	DSP	186	8	50	30	25	1981	78	M	7	7a	yes	spread	oxer	left	no	0	0	65,02	no
Stuttgart	Grand Prix of Stuttgart presented by Mercedes-Benz, WALTER solar	19.11.2017	CSI-W5*	4	1.60	410	105	12	02.04.2006	M	DSP	186	8	50	30	25	1981	78	M	8	7b	yes	vertical	vertical	left	yes	4	0	65,02	no
Stuttgart	Grand Prix of Stuttgart presented by Mercedes-Benz, WALTER solar	19.11.2017	CSI-W5*	4	1.60	410	105	12	02.04.2006	M	DSP	186	8	50	30	25	1981	78	M	9	7c	yes	spread	oxer	left	no	0	0	65,02	no
Stuttgart	Grand Prix of Stuttgart presented by Mercedes-Benz, WALTER solar	19.11.2017	CSI-W5*	4	1.60	410	105	12	02.04.2006	M	DSP	186	8	50	30	25	1981	78	M	10	8	no	spread	oxer	left	no	0	0	65,02	no
Stuttgart	Grand Prix of Stuttgart presented by Mercedes-Benz, WALTER solar	19.11.2017	CSI-W5*	4	1.60	410	105	12	02.04.2006	M	DSP	186	8	50	30	25	1981	78	M	11	9	no	vertical	vertical	left	no	0	0	65,02	no
Stuttgart	Grand Prix of Stuttgart presented by Mercedes-Benz, WALTER solar	19.11.2017	CSI-W5*	4	1.60	410	105	12	02.04.2006	M	DSP	186	8	50	30	25	1981	78	M	12	10	no	spread	oxer	right	no	0	0	65,02	no
Stuttgart	Grand Prix of Stuttgart presented by Mercedes-Benz, WALTER solar	19.11.2017	CSI-W5*	4	1.60	410	105	12	02.04.2006	M	DSP	186	8	50	30	25	1981	78	M	13	11	no	vertical	vertical	right	no	0	0	65,02	no
Stuttgart	Grand Prix of Stuttgart presented by Mercedes-Benz, WALTER solar	19.11.2017	CSI-W5*	4	1.60	410	105	12	02.04.2006	M	DSP	186	8	50	30	25	1981	78	M	14	12a	yes	vertical	vertical	right	no	0	0	65,02	no
Stuttgart	Grand Prix of Stuttgart presented by Mercedes-Benz, WALTER solar	19.11.2017	CSI-W5*	4	1.60	410	105	12	02.04.2006	M	DSP	186	8	50	30	25	1981	78	M	15	12b	yes	spread	oxer with water	right	no	0	0	65,02	no
Stuttgart	Grand Prix of Stuttgart presented by Mercedes-Benz, WALTER solar	19.11.2017	CSI-W5*	4	1.60	410	105	12	02.04.2006	M	DSP	186	8	50	30	25	1981	78	M	16	13	no	spread	oxer with water	straight	no	0	0	65,02	no
Stuttgart	Grand Prix of Stuttgart presented by Mercedes-Benz, WALTER solar	19.11.2017	CSI-W5*	4	1.60	410	36	13	30.05.2005	S	HOLST	223	29	52	52	32	2035	202	M	1	1	no	vertical	wall	right	no	0	0	67,43	no
Stuttgart	Grand Prix of Stuttgart presented by Mercedes-Benz, WALTER solar	19.11.2017	CSI-W5*	4	1.60	410	36	13	30.05.2005	S	HOLST	223	29	52	52	32	2035	202	M	2	2	no	spread	oxer	right	no	0	0	67,43	no
Stuttgart	Grand Prix of Stuttgart presented by Mercedes-Benz, WALTER solar	19.11.2017	CSI-W5*	4	1.60	410	36	13	30.05.2005	S	HOLST	223	29	52	52	32	2035	202	M	3	3	no	vertical	vertical	right	no	0	0	67,43	no
Stuttgart	Grand Prix of Stuttgart presented by Mercedes-Benz, WALTER solar	19.11.2017	CSI-W5*	4	1.60	410	36	13	30.05.2005	S	HOLST	223	29	52	52	32	2035	202	M	4	4	no	spread	oxer	straight	no	0	0	67,43	no
Stuttgart	Grand Prix of Stuttgart presented by Mercedes-Benz, WALTER solar	19.11.2017	CSI-W5*	4	1.60	410	36	13	30.05.2005	S	HOLST	223	29	52	52	32	2035	202	M	5	5	no	spread	oxer	straight	no	0	0	67,43	no
Stuttgart	Grand Prix of Stuttgart presented by Mercedes-Benz, WALTER solar	19.11.2017	CSI-W5*	4	1.60	410	36	13	30.05.2005	S	HOLST	223	29	52	52	32	2035	202	M	6	6	no	vertical	vertical	left	no	0	0	67,43	no
Stuttgart	Grand Prix of Stuttgart presented by Mercedes-Benz, WALTER solar	19.11.2017	CSI-W5*	4	1.60	410	36	13	30.05.2005	S	HOLST	223	29	52	52	32	2035	202	M	7	7a	yes	spread	oxer	left	no	0	0	67,43	no
Stuttgart	Grand Prix of Stuttgart presented by Mercedes-Benz, WALTER solar	19.11.2017	CSI-W5*	4	1.60	410	36	13	30.05.2005	S	HOLST	223	29	52	52	32	2035	202	M	8	7b	yes	vertical	vertical	left	no	0	0	67,43	no
Stuttgart	Grand Prix of Stuttgart presented by Mercedes-Benz, WALTER solar	19.11.2017	CSI-W5*	4	1.60	410	36	13	30.05.2005	S	HOLST	223	29	52	52	32	2035	202	M	9	7c	yes	spread	oxer	left	no	0	0	67,43	no
Stuttgart	Grand Prix of Stuttgart presented by Mercedes-Benz, WALTER solar	19.11.2017	CSI-W5*	4	1.60	410	36	13	30.05.2005	S	HOLST	223	29	52	52	32	2035	202	M	10	8	no	spread	oxer	left	no	0	0	67,43	no
Stuttgart	Grand Prix of Stuttgart presented by Mercedes-Benz, WALTER solar	19.11.2017	CSI-W5*	4	1.60	410	36	13	30.05.2005	S	HOLST	223	29	52	52	32	2035	202	M	11	9	no	vertical	vertical	left	no	0	0	67,43	no
Stuttgart	Grand Prix of Stuttgart presented by Mercedes-Benz, WALTER solar	19.11.2017	CSI-W5*	4	1.60	410	36	13	30.05.2005	S	HOLST	223	29	52	52	32	2035	202	M	12	10	no	spread	oxer	right	no	0	0	67,43	no
Stuttgart	Grand Prix of Stuttgart presented by Mercedes-Benz, WALTER solar	19.11.2017	CSI-W5*	4	1.60	410	36	13	30.05.2005	S	HOLST	223	29	52	52	32	2035	202	M	13	11	no	vertical	vertical	right	no	0	0	67,43	no
Stuttgart	Grand Prix of Stuttgart presented by Mercedes-Benz, WALTER solar	19.11.2017	CSI-W5*	4	1.60	410	36	13	30.05.2005	S	HOLST	223	29	52	52	32	2035	202	M	14	12a	yes	vertical	vertical	right	no	0	0	67,43	no
Stuttgart	Grand Prix of Stuttgart presented by Mercedes-Benz, WALTER solar	19.11.2017	CSI-W5*	4	1.60	410	36	13	30.05.2005	S	HOLST	223	29	52	52	32	2035	202	M	15	12b	yes	spread	oxer with water	right	yes	4	0	67,43	no
Stuttgart	Grand Prix of Stuttgart presented by Mercedes-Benz, WALTER solar	19.11.2017	CSI-W5*	4	1.60	410	36	13	30.05.2005	S	HOLST	223	29	52	52	32	2035	202	M	16	13	no	spread	oxer with water	straight	no	0	0	67,43	no
Stuttgart	Grand Prix of Stuttgart presented by Mercedes-Benz, WALTER solar	19.11.2017	CSI-W5*	4	1.60	410	10	11	07.05.2007	M	Bezplemenné	103	1	53	32	33	1848	55	M	1	1	no	vertical	wall	right	no	0	0	67,77	no
Stuttgart	Grand Prix of Stuttgart presented by Mercedes-Benz, WALTER solar	19.11.2017	CSI-W5*	4	1.60	410	10	11	07.05.2007	M	Bezplemenné	103	1	53	32	33	1848	55	M	2	2	no	spread	oxer	right	no	0	0	67,77	no
Stuttgart	Grand Prix of Stuttgart presented by Mercedes-Benz, WALTER solar	19.11.2017	CSI-W5*	4	1.60	410	10	11	07.05.2007	M	Bezplemenné	103	1	53	32	33	1848	55	M	3	3	no	vertical	vertical	right	no	0	0	67,77	no
Stuttgart	Grand Prix of Stuttgart presented by Mercedes-Benz, WALTER solar	19.11.2017	CSI-W5*	4	1.60	410	10	11	07.05.2007	M	Bezplemenné	103	1	53	32	33	1848	55	M	4	4	no	spread	oxer	straight	no	0	0	67,77	no
Stuttgart	Grand Prix of Stuttgart presented by Mercedes-Benz, WALTER solar	19.11.2017	CSI-W5*	4	1.60	410	10	11	07.05.2007	M	Bezplemenné	103	1	53	32	33	1848	55	M	5	5	no	spread	oxer	straight	no	0	0	67,77	no
Stuttgart	Grand Prix of Stuttgart presented by Mercedes-Benz, WALTER solar	19.11.2017	CSI-W5*	4	1.60	410	10	11	07.05.2007	M	Bezplemenné	103	1	53	32	33	1848	55	M	6	6	no	vertical	vertical	left	no	0	0	67,77	no
Stuttgart	Grand Prix of Stuttgart presented by Mercedes-Benz, WALTER solar	19.11.2017	CSI-W5*	4	1.60	410	10	11	07.05.2007	M	Bezplemenné	103	1	53	32	33	1848	55	M	7	7a	yes	spread	oxer	left	no	0	0	67,77	no
Stuttgart	Grand Prix of Stuttgart presented by Mercedes-Benz, WALTER solar	19.11.2017	CSI-W5*	4	1.60	410	10	11	07.05.2007	M	Bezplemenné	103	1	53	32	33	1848	55	M	8	7b	yes	vertical	vertical	left	no	0	0	67,77	no
Stuttgart	Grand Prix of Stuttgart presented by Mercedes-Benz, WALTER solar	19.11.2017	CSI-W5*	4	1.60	410	10	11	07.05.2007	M	Bezplemenné	103	1	53	32	33	1848	55	M	9	7c	yes	spread	oxer	left	no	0	0	67,77	no
Stuttgart	Grand Prix of Stuttgart presented by Mercedes-Benz, WALTER solar	19.11.2017	CSI-W5*	4	1.60	410	10	11	07.05.2007	M	Bezplemenné	103	1	53	32	33	1848	55	M	10	8	no	spread	oxer	left	no	0	0	67,77	no
Stuttgart	Grand Prix of Stuttgart presented by Mercedes-Benz, WALTER solar	19.11.2017	CSI-W5*	4	1.60	410	10	11	07.05.2007	M	Bezplemenné	103	1	53	32	33	1848	55	M	11	9	no	vertical	vertical	left	no	0	0	67,77	no
Stuttgart	Grand Prix of Stuttgart presented by Mercedes-Benz, WALTER solar	19.11.2017	CSI-W5*	4	1.60	410	10	11	07.05.2007	M	Bezplemenné	103	1	53	32	33	1848	55	M	12	10	no	spread	oxer	right	no	0	0	67,77	no
Stuttgart	Grand Prix of Stuttgart presented by Mercedes-Benz, WALTER solar	19.11.2017	CSI-W5*	4	1.60	410	10	11	07.05.2007	M	Bezplemenné	103	1	53	32	33	1848	55	M	13	11	no	vertical	vertical	right	no	0	0	67,77	no
Stuttgart	Grand Prix of Stuttgart presented by Mercedes-Benz, WALTER solar	19.11.2017	CSI-W5*	4	1.60	410	10	11	07.05.2007	M	Bezplemenné	103	1	53	32	33	1848	55	M	14	12a	yes	vertical	vertical	right	no	0	0	67,77	no
Stuttgart	Grand Prix of Stuttgart presented by Mercedes-Benz, WALTER solar	19.11.2017	CSI-W5*	4	1.60	410	10	11	07.05.2007	M	Bezplemenné	103	1	53	32	33	1848	55	M	15	12b	yes	spread	oxer with water	right	no	0	0	67,77	no
Stuttgart	Grand Prix of Stuttgart presented by Mercedes-Benz, WALTER solar	19.11.2017	CSI-W5*	4	1.60	410	10	11	07.05.2007	M	Bezplemenné	103	1	53	32	33	1848	55	M	16	13	no	spread	oxer with water	straight	no	0	0	67,77	no
Stuttgart	Grand Prix of Stuttgart presented by Mercedes-Benz, WALTER solar	19.11.2017	CSI-W5*	4	1.60	380	10	11	07.05.2007	M	Bezplemenné	103	1	53	32	33	1848	55	M	1	1	no	spread	oxer	left	no	0	0	48,76	no
Stuttgart	Grand Prix of Stuttgart presented by Mercedes-Benz, WALTER solar	19.11.2017	CSI-W5*	4	1.60	380	10	11	07.05.2007	M	Bezplemenné	103	1	53	32	33	1848	55	M	2	2	no	vertical	vertical	left	no	0	0	48,76	no
Stuttgart	Grand Prix of Stuttgart presented by Mercedes-Benz, WALTER solar	19.11.2017	CSI-W5*	4	1.60	380	10	11	07.05.2007	M	Bezplemenné	103	1	53	32	33	1848	55	M	3	3	no	vertical	vertical	right	no	0	0	48,76	no
Stuttgart	Grand Prix of Stuttgart presented by Mercedes-Benz, WALTER solar	19.11.2017	CSI-W5*	4	1.60	380	10	11	07.05.2007	M	Bezplemenné	103	1	53	32	33	1848	55	M	4	4	no	spread	oxer with water	right	no	0	0	48,76	no
Stuttgart	Grand Prix of Stuttgart presented by Mercedes-Benz, WALTER solar	19.11.2017	CSI-W5*	4	1.60	380	10	11	07.05.2007	M	Bezplemenné	103	1	53	32	33	1848	55	M	5	5	no	vertical	vertical	left	no	0	0	48,76	no
Stuttgart	Grand Prix of Stuttgart presented by Mercedes-Benz, WALTER solar	19.11.2017	CSI-W5*	4	1.60	380	10	11	07.05.2007	M	Bezplemenné	103	1	53	32	33	1848	55	M	6	6	no	vertical	wall	right	no	0	0	48,76	no
Stuttgart	Grand Prix of Stuttgart presented by Mercedes-Benz, WALTER solar	19.11.2017	CSI-W5*	4	1.60	380	10	11	07.05.2007	M	Bezplemenné	103	1	53	32	33	1848	55	M	7	7	no	spread	oxer	right	no	0	0	48,76	no
Stuttgart	Grand Prix of Stuttgart presented by Mercedes-Benz, WALTER solar	19.11.2017	CSI-W5*	4	1.60	380	10	11	07.05.2007	M	Bezplemenné	103	1	53	32	33	1848	55	M	8	8a	yes	spread	oxer	left	no	0	0	48,76	no
Stuttgart	Grand Prix of Stuttgart presented by Mercedes-Benz, WALTER solar	19.11.2017	CSI-W5*	4	1.60	380	10	11	07.05.2007	M	Bezplemenné	103	1	53	32	33	1848	55	M	9	8b	yes	vertical	vertical	left	no	0	0	48,76	no
Stuttgart	Grand Prix of Stuttgart presented by Mercedes-Benz, WALTER solar	19.11.2017	CSI-W5*	4	1.60	380	10	11	07.05.2007	M	Bezplemenné	103	1	53	32	33	1848	55	M	10	9	no	spread	oxer	left	no	0	0	48,76	no
Stuttgart	Grand Prix of Stuttgart presented by Mercedes-Benz, WALTER solar	19.11.2017	CSI-W5*	4	1.60	410	158	12	12.06.2006	G	AES	146	1	55	6	37	1750	61	M	1	1	no	vertical	wall	right	no	0	0	67,32	no
Stuttgart	Grand Prix of Stuttgart presented by Mercedes-Benz, WALTER solar	19.11.2017	CSI-W5*	4	1.60	410	158	12	12.06.2006	G	AES	146	1	55	6	37	1750	61	M	2	2	no	spread	oxer	right	no	0	0	67,32	no
Stuttgart	Grand Prix of Stuttgart presented by Mercedes-Benz, WALTER solar	19.11.2017	CSI-W5*	4	1.60	410	158	12	12.06.2006	G	AES	146	1	55	6	37	1750	61	M	3	3	no	vertical	vertical	right	no	0	0	67,32	no
Stuttgart	Grand Prix of Stuttgart presented by Mercedes-Benz, WALTER solar	19.11.2017	CSI-W5*	4	1.60	410	158	12	12.06.2006	G	AES	146	1	55	6	37	1750	61	M	4	4	no	spread	oxer	straight	no	0	0	67,32	no
Stuttgart	Grand Prix of Stuttgart presented by Mercedes-Benz, WALTER solar	19.11.2017	CSI-W5*	4	1.60	410	158	12	12.06.2006	G	AES	146	1	55	6	37	1750	61	M	5	5	no	spread	oxer	straight	no	0	0	67,32	no
Stuttgart	Grand Prix of Stuttgart presented by Mercedes-Benz, WALTER solar	19.11.2017	CSI-W5*	4	1.60	410	158	12	12.06.2006	G	AES	146	1	55	6	37	1750	61	M	6	6	no	vertical	vertical	left	no	0	0	67,32	no
Stuttgart	Grand Prix of Stuttgart presented by Mercedes-Benz, WALTER solar	19.11.2017	CSI-W5*	4	1.60	410	158	12	12.06.2006	G	AES	146	1	55	6	37	1750	61	M	7	7a	yes	spread	oxer	left	no	0	0	67,32	no
Stuttgart	Grand Prix of Stuttgart presented by Mercedes-Benz, WALTER solar	19.11.2017	CSI-W5*	4	1.60	410	158	12	12.06.2006	G	AES	146	1	55	6	37	1750	61	M	8	7b	yes	vertical	vertical	left	no	0	0	67,32	no
Stuttgart	Grand Prix of Stuttgart presented by Mercedes-Benz, WALTER solar	19.11.2017	CSI-W5*	4	1.60	410	158	12	12.06.2006	G	AES	146	1	55	6	37	1750	61	M	9	7c	yes	spread	oxer	left	no	0	0	67,32	no
Stuttgart	Grand Prix of Stuttgart presented by Mercedes-Benz, WALTER solar	19.11.2017	CSI-W5*	4	1.60	410	158	12	12.06.2006	G	AES	146	1	55	6	37	1750	61	M	10	8	no	spread	oxer	left	no	0	0	67,32	no
Stuttgart	Grand Prix of Stuttgart presented by Mercedes-Benz, WALTER solar	19.11.2017	CSI-W5*	4	1.60	410	158	12	12.06.2006	G	AES	146	1	55	6	37	1750	61	M	11	9	no	vertical	vertical	left	no	0	0	67,32	no
Stuttgart	Grand Prix of Stuttgart presented by Mercedes-Benz, WALTER solar	19.11.2017	CSI-W5*	4	1.60	410	158	12	12.06.2006	G	AES	146	1	55	6	37	1750	61	M	12	10	no	spread	oxer	right	no	0	0	67,32	no
Stuttgart	Grand Prix of Stuttgart presented by Mercedes-Benz, WALTER solar	19.11.2017	CSI-W5*	4	1.60	410	158	12	12.06.2006	G	AES	146	1	55	6	37	1750	61	M	13	11	no	vertical	vertical	right	no	0	0	67,32	no
Stuttgart	Grand Prix of Stuttgart presented by Mercedes-Benz, WALTER solar	19.11.2017	CSI-W5*	4	1.60	410	158	12	12.06.2006	G	AES	146	1	55	6	37	1750	61	M	14	12a	yes	vertical	vertical	right	no	0	0	67,32	no
Stuttgart	Grand Prix of Stuttgart presented by Mercedes-Benz, WALTER solar	19.11.2017	CSI-W5*	4	1.60	410	158	12	12.06.2006	G	AES	146	1	55	6	37	1750	61	M	15	12b	yes	spread	oxer with water	right	no	0	0	67,32	no
Stuttgart	Grand Prix of Stuttgart presented by Mercedes-Benz, WALTER solar	19.11.2017	CSI-W5*	4	1.60	410	158	12	12.06.2006	G	AES	146	1	55	6	37	1750	61	M	16	13	no	spread	oxer with water	straight	no	0	0	67,32	no
Stuttgart	Grand Prix of Stuttgart presented by Mercedes-Benz, WALTER solar	19.11.2017	CSI-W5*	4	1.60	380	158	12	12.06.2006	G	AES	146	1	55	6	37	1750	61	M	1	1	no	spread	oxer	left	no	0	0	52,34	no
Stuttgart	Grand Prix of Stuttgart presented by Mercedes-Benz, WALTER solar	19.11.2017	CSI-W5*	4	1.60	380	158	12	12.06.2006	G	AES	146	1	55	6	37	1750	61	M	2	2	no	vertical	vertical	left	no	0	0	52,34	no
Stuttgart	Grand Prix of Stuttgart presented by Mercedes-Benz, WALTER solar	19.11.2017	CSI-W5*	4	1.60	380	158	12	12.06.2006	G	AES	146	1	55	6	37	1750	61	M	3	3	no	vertical	vertical	right	no	0	0	52,34	no
Stuttgart	Grand Prix of Stuttgart presented by Mercedes-Benz, WALTER solar	19.11.2017	CSI-W5*	4	1.60	380	158	12	12.06.2006	G	AES	146	1	55	6	37	1750	61	M	4	4	no	spread	oxer with water	right	no	0	0	52,34	no
Stuttgart	Grand Prix of Stuttgart presented by Mercedes-Benz, WALTER solar	19.11.2017	CSI-W5*	4	1.60	380	158	12	12.06.2006	G	AES	146	1	55	6	37	1750	61	M	5	5	no	vertical	vertical	left	no	0	0	52,34	no
Stuttgart	Grand Prix of Stuttgart presented by Mercedes-Benz, WALTER solar	19.11.2017	CSI-W5*	4	1.60	380	158	12	12.06.2006	G	AES	146	1	55	6	37	1750	61	M	6	6	no	vertical	wall	right	yes	4	0	52,34	no
Stuttgart	Grand Prix of Stuttgart presented by Mercedes-Benz, WALTER solar	19.11.2017	CSI-W5*	4	1.60	380	158	12	12.06.2006	G	AES	146	1	55	6	37	1750	61	M	7	7	no	spread	oxer	right	yes	4	0	52,34	no
Stuttgart	Grand Prix of Stuttgart presented by Mercedes-Benz, WALTER solar	19.11.2017	CSI-W5*	4	1.60	380	158	12	12.06.2006	G	AES	146	1	55	6	37	1750	61	M	8	8a	yes	spread	oxer	left	yes	4	0	52,34	no
Stuttgart	Grand Prix of Stuttgart presented by Mercedes-Benz, WALTER solar	19.11.2017	CSI-W5*	4	1.60	380	158	12	12.06.2006	G	AES	146	1	55	6	37	1750	61	M	9	8b	yes	vertical	vertical	left	no	0	0	52,34	no
Stuttgart	Grand Prix of Stuttgart presented by Mercedes-Benz, WALTER solar	19.11.2017	CSI-W5*	4	1.60	380	158	12	12.06.2006	G	AES	146	1	55	6	37	1750	61	M	10	9	no	spread	oxer	left	no	0	0	52,34	no
Stuttgart	Grand Prix of Stuttgart presented by Mercedes-Benz, WALTER solar	19.11.2017	CSI-W5*	4	1.60	410	165	14	01.06.2004	S	SF	184	9	58	37	35	1522	63	M	1	1	no	vertical	wall	right	no	0	0	68,87	no
Stuttgart	Grand Prix of Stuttgart presented by Mercedes-Benz, WALTER solar	19.11.2017	CSI-W5*	4	1.60	410	165	14	01.06.2004	S	SF	184	9	58	37	35	1522	63	M	2	2	no	spread	oxer	right	no	0	0	68,87	no
Stuttgart	Grand Prix of Stuttgart presented by Mercedes-Benz, WALTER solar	19.11.2017	CSI-W5*	4	1.60	410	165	14	01.06.2004	S	SF	184	9	58	37	35	1522	63	M	3	3	no	vertical	vertical	right	no	0	0	68,87	no
Stuttgart	Grand Prix of Stuttgart presented by Mercedes-Benz, WALTER solar	19.11.2017	CSI-W5*	4	1.60	410	165	14	01.06.2004	S	SF	184	9	58	37	35	1522	63	M	4	4	no	spread	oxer	straight	no	0	0	68,87	no
Stuttgart	Grand Prix of Stuttgart presented by Mercedes-Benz, WALTER solar	19.11.2017	CSI-W5*	4	1.60	410	165	14	01.06.2004	S	SF	184	9	58	37	35	1522	63	M	5	5	no	spread	oxer	straight	no	0	0	68,87	no
Stuttgart	Grand Prix of Stuttgart presented by Mercedes-Benz, WALTER solar	19.11.2017	CSI-W5*	4	1.60	410	165	14	01.06.2004	S	SF	184	9	58	37	35	1522	63	M	6	6	no	vertical	vertical	left	no	0	0	68,87	no
Stuttgart	Grand Prix of Stuttgart presented by Mercedes-Benz, WALTER solar	19.11.2017	CSI-W5*	4	1.60	410	165	14	01.06.2004	S	SF	184	9	58	37	35	1522	63	M	7	7a	yes	spread	oxer	left	yes	4	0	68,87	no
Stuttgart	Grand Prix of Stuttgart presented by Mercedes-Benz, WALTER solar	19.11.2017	CSI-W5*	4	1.60	410	165	14	01.06.2004	S	SF	184	9	58	37	35	1522	63	M	8	7b	yes	vertical	vertical	left	no	0	0	68,87	no
Stuttgart	Grand Prix of Stuttgart presented by Mercedes-Benz, WALTER solar	19.11.2017	CSI-W5*	4	1.60	410	165	14	01.06.2004	S	SF	184	9	58	37	35	1522	63	M	9	7c	yes	spread	oxer	left	no	0	0	68,87	no
Stuttgart	Grand Prix of Stuttgart presented by Mercedes-Benz, WALTER solar	19.11.2017	CSI-W5*	4	1.60	410	165	14	01.06.2004	S	SF	184	9	58	37	35	1522	63	M	10	8	no	spread	oxer	left	no	0	0	68,87	no
Stuttgart	Grand Prix of Stuttgart presented by Mercedes-Benz, WALTER solar	19.11.2017	CSI-W5*	4	1.60	410	165	14	01.06.2004	S	SF	184	9	58	37	35	1522	63	M	11	9	no	vertical	vertical	left	no	0	0	68,87	no
Stuttgart	Grand Prix of Stuttgart presented by Mercedes-Benz, WALTER solar	19.11.2017	CSI-W5*	4	1.60	410	165	14	01.06.2004	S	SF	184	9	58	37	35	1522	63	M	12	10	no	spread	oxer	right	no	0	0	68,87	no
Stuttgart	Grand Prix of Stuttgart presented by Mercedes-Benz, WALTER solar	19.11.2017	CSI-W5*	4	1.60	410	165	14	01.06.2004	S	SF	184	9	58	37	35	1522	63	M	13	11	no	vertical	vertical	right	no	0	0	68,87	no
Stuttgart	Grand Prix of Stuttgart presented by Mercedes-Benz, WALTER solar	19.11.2017	CSI-W5*	4	1.60	410	165	14	01.06.2004	S	SF	184	9	58	37	35	1522	63	M	14	12a	yes	vertical	vertical	right	no	0	0	68,87	no
Stuttgart	Grand Prix of Stuttgart presented by Mercedes-Benz, WALTER solar	19.11.2017	CSI-W5*	4	1.60	410	165	14	01.06.2004	S	SF	184	9	58	37	35	1522	63	M	15	12b	yes	spread	oxer with water	right	no	0	0	68,87	no
Stuttgart	Grand Prix of Stuttgart presented by Mercedes-Benz, WALTER solar	19.11.2017	CSI-W5*	4	1.60	410	165	14	01.06.2004	S	SF	184	9	58	37	35	1522	63	M	16	13	no	spread	oxer with water	straight	no	0	0	68,87	no
Stuttgart	Grand Prix of Stuttgart presented by Mercedes-Benz, WALTER solar	19.11.2017	CSI-W5*	4	1.60	410	80	14	24.07.2004	S	BWP	179	5	64	20	44	2014	118	M	1	1	no	vertical	wall	right	no	0	0	68,43	no
Stuttgart	Grand Prix of Stuttgart presented by Mercedes-Benz, WALTER solar	19.11.2017	CSI-W5*	4	1.60	410	80	14	24.07.2004	S	BWP	179	5	64	20	44	2014	118	M	2	2	no	spread	oxer	right	no	0	0	68,43	no
Stuttgart	Grand Prix of Stuttgart presented by Mercedes-Benz, WALTER solar	19.11.2017	CSI-W5*	4	1.60	410	80	14	24.07.2004	S	BWP	179	5	64	20	44	2014	118	M	3	3	no	vertical	vertical	right	no	0	0	68,43	no
Stuttgart	Grand Prix of Stuttgart presented by Mercedes-Benz, WALTER solar	19.11.2017	CSI-W5*	4	1.60	410	80	14	24.07.2004	S	BWP	179	5	64	20	44	2014	118	M	4	4	no	spread	oxer	straight	no	0	0	68,43	no
Stuttgart	Grand Prix of Stuttgart presented by Mercedes-Benz, WALTER solar	19.11.2017	CSI-W5*	4	1.60	410	80	14	24.07.2004	S	BWP	179	5	64	20	44	2014	118	M	5	5	no	spread	oxer	straight	no	0	0	68,43	no
Stuttgart	Grand Prix of Stuttgart presented by Mercedes-Benz, WALTER solar	19.11.2017	CSI-W5*	4	1.60	410	80	14	24.07.2004	S	BWP	179	5	64	20	44	2014	118	M	6	6	no	vertical	vertical	left	no	0	0	68,43	no
Stuttgart	Grand Prix of Stuttgart presented by Mercedes-Benz, WALTER solar	19.11.2017	CSI-W5*	4	1.60	410	80	14	24.07.2004	S	BWP	179	5	64	20	44	2014	118	M	7	7a	yes	spread	oxer	left	no	0	0	68,43	no
Stuttgart	Grand Prix of Stuttgart presented by Mercedes-Benz, WALTER solar	19.11.2017	CSI-W5*	4	1.60	410	80	14	24.07.2004	S	BWP	179	5	64	20	44	2014	118	M	8	7b	yes	vertical	vertical	left	no	0	0	68,43	no
Stuttgart	Grand Prix of Stuttgart presented by Mercedes-Benz, WALTER solar	19.11.2017	CSI-W5*	4	1.60	410	80	14	24.07.2004	S	BWP	179	5	64	20	44	2014	118	M	9	7c	yes	spread	oxer	left	no	0	0	68,43	no
Stuttgart	Grand Prix of Stuttgart presented by Mercedes-Benz, WALTER solar	19.11.2017	CSI-W5*	4	1.60	410	80	14	24.07.2004	S	BWP	179	5	64	20	44	2014	118	M	10	8	no	spread	oxer	left	no	0	0	68,43	no
Stuttgart	Grand Prix of Stuttgart presented by Mercedes-Benz, WALTER solar	19.11.2017	CSI-W5*	4	1.60	410	80	14	24.07.2004	S	BWP	179	5	64	20	44	2014	118	M	11	9	no	vertical	vertical	left	no	0	0	68,43	no
Stuttgart	Grand Prix of Stuttgart presented by Mercedes-Benz, WALTER solar	19.11.2017	CSI-W5*	4	1.60	410	80	14	24.07.2004	S	BWP	179	5	64	20	44	2014	118	M	12	10	no	spread	oxer	right	no	0	0	68,43	no
Stuttgart	Grand Prix of Stuttgart presented by Mercedes-Benz, WALTER solar	19.11.2017	CSI-W5*	4	1.60	410	80	14	24.07.2004	S	BWP	179	5	64	20	44	2014	118	M	13	11	no	vertical	vertical	right	no	0	0	68,43	no
Stuttgart	Grand Prix of Stuttgart presented by Mercedes-Benz, WALTER solar	19.11.2017	CSI-W5*	4	1.60	410	80	14	24.07.2004	S	BWP	179	5	64	20	44	2014	118	M	14	12a	yes	vertical	vertical	right	no	0	0	68,43	no
Stuttgart	Grand Prix of Stuttgart presented by Mercedes-Benz, WALTER solar	19.11.2017	CSI-W5*	4	1.60	410	80	14	24.07.2004	S	BWP	179	5	64	20	44	2014	118	M	15	12b	yes	spread	oxer with water	right	no	0	0	68,43	no
Stuttgart	Grand Prix of Stuttgart presented by Mercedes-Benz, WALTER solar	19.11.2017	CSI-W5*	4	1.60	410	80	14	24.07.2004	S	BWP	179	5	64	20	44	2014	118	M	16	13	no	spread	oxer with water	straight	no	0	0	68,43	no
Stuttgart	Grand Prix of Stuttgart presented by Mercedes-Benz, WALTER solar	19.11.2017	CSI-W5*	4	1.60	380	80	14	24.07.2004	S	BWP	179	5	64	20	44	2014	118	M	1	1	no	spread	oxer	left	no	0	0	49,13	no
Stuttgart	Grand Prix of Stuttgart presented by Mercedes-Benz, WALTER solar	19.11.2017	CSI-W5*	4	1.60	380	80	14	24.07.2004	S	BWP	179	5	64	20	44	2014	118	M	2	2	no	vertical	vertical	left	no	0	0	49,13	no
Stuttgart	Grand Prix of Stuttgart presented by Mercedes-Benz, WALTER solar	19.11.2017	CSI-W5*	4	1.60	380	80	14	24.07.2004	S	BWP	179	5	64	20	44	2014	118	M	3	3	no	vertical	vertical	right	no	0	0	49,13	no
Stuttgart	Grand Prix of Stuttgart presented by Mercedes-Benz, WALTER solar	19.11.2017	CSI-W5*	4	1.60	380	80	14	24.07.2004	S	BWP	179	5	64	20	44	2014	118	M	4	4	no	spread	oxer with water	right	no	0	0	49,13	no
Stuttgart	Grand Prix of Stuttgart presented by Mercedes-Benz, WALTER solar	19.11.2017	CSI-W5*	4	1.60	380	80	14	24.07.2004	S	BWP	179	5	64	20	44	2014	118	M	5	5	no	vertical	vertical	left	no	0	0	49,13	no
Stuttgart	Grand Prix of Stuttgart presented by Mercedes-Benz, WALTER solar	19.11.2017	CSI-W5*	4	1.60	380	80	14	24.07.2004	S	BWP	179	5	64	20	44	2014	118	M	6	6	no	vertical	wall	right	no	0	0	49,13	no
Stuttgart	Grand Prix of Stuttgart presented by Mercedes-Benz, WALTER solar	19.11.2017	CSI-W5*	4	1.60	380	80	14	24.07.2004	S	BWP	179	5	64	20	44	2014	118	M	7	7	no	spread	oxer	right	no	0	0	49,13	no
Stuttgart	Grand Prix of Stuttgart presented by Mercedes-Benz, WALTER solar	19.11.2017	CSI-W5*	4	1.60	380	80	14	24.07.2004	S	BWP	179	5	64	20	44	2014	118	M	8	8a	yes	spread	oxer	left	no	0	0	49,13	no
Stuttgart	Grand Prix of Stuttgart presented by Mercedes-Benz, WALTER solar	19.11.2017	CSI-W5*	4	1.60	380	80	14	24.07.2004	S	BWP	179	5	64	20	44	2014	118	M	9	8b	yes	vertical	vertical	left	no	0	0	49,13	no
Stuttgart	Grand Prix of Stuttgart presented by Mercedes-Benz, WALTER solar	19.11.2017	CSI-W5*	4	1.60	380	80	14	24.07.2004	S	BWP	179	5	64	20	44	2014	118	M	10	9	no	spread	oxer	left	no	0	0	49,13	no
Stuttgart	Grand Prix of Stuttgart presented by Mercedes-Benz, WALTER solar	19.11.2017	CSI-W5*	4	1.60	410	179	12	28.05.2006	G	HOLST	170	7	69	147	42	785	73	M	1	1	no	vertical	wall	right	no	0	0	69,23	no
Stuttgart	Grand Prix of Stuttgart presented by Mercedes-Benz, WALTER solar	19.11.2017	CSI-W5*	4	1.60	410	179	12	28.05.2006	G	HOLST	170	7	69	147	42	785	73	M	2	2	no	spread	oxer	right	no	0	0	69,23	no
Stuttgart	Grand Prix of Stuttgart presented by Mercedes-Benz, WALTER solar	19.11.2017	CSI-W5*	4	1.60	410	179	12	28.05.2006	G	HOLST	170	7	69	147	42	785	73	M	3	3	no	vertical	vertical	right	no	0	0	69,23	no
Stuttgart	Grand Prix of Stuttgart presented by Mercedes-Benz, WALTER solar	19.11.2017	CSI-W5*	4	1.60	410	179	12	28.05.2006	G	HOLST	170	7	69	147	42	785	73	M	4	4	no	spread	oxer	straight	no	0	0	69,23	no
Stuttgart	Grand Prix of Stuttgart presented by Mercedes-Benz, WALTER solar	19.11.2017	CSI-W5*	4	1.60	410	179	12	28.05.2006	G	HOLST	170	7	69	147	42	785	73	M	5	5	no	spread	oxer	straight	no	0	0	69,23	no
Stuttgart	Grand Prix of Stuttgart presented by Mercedes-Benz, WALTER solar	19.11.2017	CSI-W5*	4	1.60	410	179	12	28.05.2006	G	HOLST	170	7	69	147	42	785	73	M	6	6	no	vertical	vertical	left	no	0	0	69,23	no
Stuttgart	Grand Prix of Stuttgart presented by Mercedes-Benz, WALTER solar	19.11.2017	CSI-W5*	4	1.60	410	179	12	28.05.2006	G	HOLST	170	7	69	147	42	785	73	M	7	7a	yes	spread	oxer	left	no	0	0	69,23	no
Stuttgart	Grand Prix of Stuttgart presented by Mercedes-Benz, WALTER solar	19.11.2017	CSI-W5*	4	1.60	410	179	12	28.05.2006	G	HOLST	170	7	69	147	42	785	73	M	8	7b	yes	vertical	vertical	left	no	0	0	69,23	no
Stuttgart	Grand Prix of Stuttgart presented by Mercedes-Benz, WALTER solar	19.11.2017	CSI-W5*	4	1.60	410	179	12	28.05.2006	G	HOLST	170	7	69	147	42	785	73	M	9	7c	yes	spread	oxer	left	no	0	0	69,23	no
Stuttgart	Grand Prix of Stuttgart presented by Mercedes-Benz, WALTER solar	19.11.2017	CSI-W5*	4	1.60	410	179	12	28.05.2006	G	HOLST	170	7	69	147	42	785	73	M	10	8	no	spread	oxer	left	no	0	0	69,23	no
Stuttgart	Grand Prix of Stuttgart presented by Mercedes-Benz, WALTER solar	19.11.2017	CSI-W5*	4	1.60	410	179	12	28.05.2006	G	HOLST	170	7	69	147	42	785	73	M	11	9	no	vertical	vertical	left	no	0	0	69,23	no
Stuttgart	Grand Prix of Stuttgart presented by Mercedes-Benz, WALTER solar	19.11.2017	CSI-W5*	4	1.60	410	179	12	28.05.2006	G	HOLST	170	7	69	147	42	785	73	M	12	10	no	spread	oxer	right	no	0	0	69,23	no
Stuttgart	Grand Prix of Stuttgart presented by Mercedes-Benz, WALTER solar	19.11.2017	CSI-W5*	4	1.60	410	179	12	28.05.2006	G	HOLST	170	7	69	147	42	785	73	M	13	11	no	vertical	vertical	right	no	0	0	69,23	no
Stuttgart	Grand Prix of Stuttgart presented by Mercedes-Benz, WALTER solar	19.11.2017	CSI-W5*	4	1.60	410	179	12	28.05.2006	G	HOLST	170	7	69	147	42	785	73	M	14	12a	yes	vertical	vertical	right	no	0	0	69,23	no
Stuttgart	Grand Prix of Stuttgart presented by Mercedes-Benz, WALTER solar	19.11.2017	CSI-W5*	4	1.60	410	179	12	28.05.2006	G	HOLST	170	7	69	147	42	785	73	M	15	12b	yes	spread	oxer with water	right	no	0	0	69,23	no
Stuttgart	Grand Prix of Stuttgart presented by Mercedes-Benz, WALTER solar	19.11.2017	CSI-W5*	4	1.60	410	179	12	28.05.2006	G	HOLST	170	7	69	147	42	785	73	M	16	13	no	spread	oxer with water	straight	no	0	0	69,23	no
Stuttgart	Grand Prix of Stuttgart presented by Mercedes-Benz, WALTER solar	19.11.2017	CSI-W5*	4	1.60	380	179	12	28.05.2006	G	HOLST	170	7	69	147	42	785	73	M	1	1	no	spread	oxer	left	no	0	0	51,83	no
Stuttgart	Grand Prix of Stuttgart presented by Mercedes-Benz, WALTER solar	19.11.2017	CSI-W5*	4	1.60	380	179	12	28.05.2006	G	HOLST	170	7	69	147	42	785	73	M	2	2	no	vertical	vertical	left	no	0	0	51,83	no
Stuttgart	Grand Prix of Stuttgart presented by Mercedes-Benz, WALTER solar	19.11.2017	CSI-W5*	4	1.60	380	179	12	28.05.2006	G	HOLST	170	7	69	147	42	785	73	M	3	3	no	vertical	vertical	right	no	0	0	51,83	no
Stuttgart	Grand Prix of Stuttgart presented by Mercedes-Benz, WALTER solar	19.11.2017	CSI-W5*	4	1.60	380	179	12	28.05.2006	G	HOLST	170	7	69	147	42	785	73	M	4	4	no	spread	oxer with water	right	no	0	0	51,83	no
Stuttgart	Grand Prix of Stuttgart presented by Mercedes-Benz, WALTER solar	19.11.2017	CSI-W5*	4	1.60	380	179	12	28.05.2006	G	HOLST	170	7	69	147	42	785	73	M	5	5	no	vertical	vertical	left	no	0	0	51,83	no
Stuttgart	Grand Prix of Stuttgart presented by Mercedes-Benz, WALTER solar	19.11.2017	CSI-W5*	4	1.60	380	179	12	28.05.2006	G	HOLST	170	7	69	147	42	785	73	M	6	6	no	vertical	wall	right	no	0	0	51,83	no
Stuttgart	Grand Prix of Stuttgart presented by Mercedes-Benz, WALTER solar	19.11.2017	CSI-W5*	4	1.60	380	179	12	28.05.2006	G	HOLST	170	7	69	147	42	785	73	M	7	7	no	spread	oxer	right	no	0	0	51,83	no
Stuttgart	Grand Prix of Stuttgart presented by Mercedes-Benz, WALTER solar	19.11.2017	CSI-W5*	4	1.60	380	179	12	28.05.2006	G	HOLST	170	7	69	147	42	785	73	M	8	8a	yes	spread	oxer	left	yes	4	0	51,83	no
Stuttgart	Grand Prix of Stuttgart presented by Mercedes-Benz, WALTER solar	19.11.2017	CSI-W5*	4	1.60	380	179	12	28.05.2006	G	HOLST	170	7	69	147	42	785	73	M	9	8b	yes	vertical	vertical	left	yes	4	0	51,83	no
Stuttgart	Grand Prix of Stuttgart presented by Mercedes-Benz, WALTER solar	19.11.2017	CSI-W5*	4	1.60	380	179	12	28.05.2006	G	HOLST	170	7	69	147	42	785	73	M	10	9	no	spread	oxer	left	no	0	0	51,83	no
Stuttgart	Grand Prix of Stuttgart presented by Mercedes-Benz, WALTER solar	19.11.2017	CSI-W5*	4	1.60	410	211	13	11.05.2005	S	SBS	150	4	70	43	38	1891	85	F	1	1	no	vertical	wall	right	no	0	0	69,14	no
Stuttgart	Grand Prix of Stuttgart presented by Mercedes-Benz, WALTER solar	19.11.2017	CSI-W5*	4	1.60	410	211	13	11.05.2005	S	SBS	150	4	70	43	38	1891	85	F	2	2	no	spread	oxer	right	no	0	0	69,14	no
Stuttgart	Grand Prix of Stuttgart presented by Mercedes-Benz, WALTER solar	19.11.2017	CSI-W5*	4	1.60	410	211	13	11.05.2005	S	SBS	150	4	70	43	38	1891	85	F	3	3	no	vertical	vertical	right	yes	4	0	69,14	no
Stuttgart	Grand Prix of Stuttgart presented by Mercedes-Benz, WALTER solar	19.11.2017	CSI-W5*	4	1.60	410	211	13	11.05.2005	S	SBS	150	4	70	43	38	1891	85	F	4	4	no	spread	oxer	straight	no	0	0	69,14	no
Stuttgart	Grand Prix of Stuttgart presented by Mercedes-Benz, WALTER solar	19.11.2017	CSI-W5*	4	1.60	410	211	13	11.05.2005	S	SBS	150	4	70	43	38	1891	85	F	5	5	no	spread	oxer	straight	no	0	0	69,14	no
Stuttgart	Grand Prix of Stuttgart presented by Mercedes-Benz, WALTER solar	19.11.2017	CSI-W5*	4	1.60	410	211	13	11.05.2005	S	SBS	150	4	70	43	38	1891	85	F	6	6	no	vertical	vertical	left	no	0	0	69,14	no
Stuttgart	Grand Prix of Stuttgart presented by Mercedes-Benz, WALTER solar	19.11.2017	CSI-W5*	4	1.60	410	211	13	11.05.2005	S	SBS	150	4	70	43	38	1891	85	F	7	7a	yes	spread	oxer	left	no	0	0	69,14	no
Stuttgart	Grand Prix of Stuttgart presented by Mercedes-Benz, WALTER solar	19.11.2017	CSI-W5*	4	1.60	410	211	13	11.05.2005	S	SBS	150	4	70	43	38	1891	85	F	8	7b	yes	vertical	vertical	left	no	0	0	69,14	no
Stuttgart	Grand Prix of Stuttgart presented by Mercedes-Benz, WALTER solar	19.11.2017	CSI-W5*	4	1.60	410	211	13	11.05.2005	S	SBS	150	4	70	43	38	1891	85	F	9	7c	yes	spread	oxer	left	no	0	0	69,14	no
Stuttgart	Grand Prix of Stuttgart presented by Mercedes-Benz, WALTER solar	19.11.2017	CSI-W5*	4	1.60	410	211	13	11.05.2005	S	SBS	150	4	70	43	38	1891	85	F	10	8	no	spread	oxer	left	no	0	0	69,14	no
Stuttgart	Grand Prix of Stuttgart presented by Mercedes-Benz, WALTER solar	19.11.2017	CSI-W5*	4	1.60	410	211	13	11.05.2005	S	SBS	150	4	70	43	38	1891	85	F	11	9	no	vertical	vertical	left	no	0	0	69,14	no
Stuttgart	Grand Prix of Stuttgart presented by Mercedes-Benz, WALTER solar	19.11.2017	CSI-W5*	4	1.60	410	211	13	11.05.2005	S	SBS	150	4	70	43	38	1891	85	F	12	10	no	spread	oxer	right	no	0	0	69,14	no
Stuttgart	Grand Prix of Stuttgart presented by Mercedes-Benz, WALTER solar	19.11.2017	CSI-W5*	4	1.60	410	211	13	11.05.2005	S	SBS	150	4	70	43	38	1891	85	F	13	11	no	vertical	vertical	right	no	0	0	69,14	no
Stuttgart	Grand Prix of Stuttgart presented by Mercedes-Benz, WALTER solar	19.11.2017	CSI-W5*	4	1.60	410	211	13	11.05.2005	S	SBS	150	4	70	43	38	1891	85	F	14	12a	yes	vertical	vertical	right	no	0	0	69,14	no
Stuttgart	Grand Prix of Stuttgart presented by Mercedes-Benz, WALTER solar	19.11.2017	CSI-W5*	4	1.60	410	211	13	11.05.2005	S	SBS	150	4	70	43	38	1891	85	F	15	12b	yes	spread	oxer with water	right	no	0	0	69,14	no
Stuttgart	Grand Prix of Stuttgart presented by Mercedes-Benz, WALTER solar	19.11.2017	CSI-W5*	4	1.60	410	211	13	11.05.2005	S	SBS	150	4	70	43	38	1891	85	F	16	13	no	spread	oxer with water	straight	no	0	0	69,14	no
Stuttgart	Grand Prix of Stuttgart presented by Mercedes-Benz, WALTER solar	19.11.2017	CSI-W5*	4	1.60	410	164	15	15.03.2003	G	SF	197	13	71	12	44	2177	93	M	1	1	no	vertical	wall	right	no	0	0	67,9	no
Stuttgart	Grand Prix of Stuttgart presented by Mercedes-Benz, WALTER solar	19.11.2017	CSI-W5*	4	1.60	410	164	15	15.03.2003	G	SF	197	13	71	12	44	2177	93	M	2	2	no	spread	oxer	right	no	0	0	67,9	no
Stuttgart	Grand Prix of Stuttgart presented by Mercedes-Benz, WALTER solar	19.11.2017	CSI-W5*	4	1.60	410	164	15	15.03.2003	G	SF	197	13	71	12	44	2177	93	M	3	3	no	vertical	vertical	right	no	0	0	67,9	no
Stuttgart	Grand Prix of Stuttgart presented by Mercedes-Benz, WALTER solar	19.11.2017	CSI-W5*	4	1.60	410	164	15	15.03.2003	G	SF	197	13	71	12	44	2177	93	M	4	4	no	spread	oxer	straight	no	0	0	67,9	no
Stuttgart	Grand Prix of Stuttgart presented by Mercedes-Benz, WALTER solar	19.11.2017	CSI-W5*	4	1.60	410	164	15	15.03.2003	G	SF	197	13	71	12	44	2177	93	M	5	5	no	spread	oxer	straight	no	0	0	67,9	no
Stuttgart	Grand Prix of Stuttgart presented by Mercedes-Benz, WALTER solar	19.11.2017	CSI-W5*	4	1.60	410	164	15	15.03.2003	G	SF	197	13	71	12	44	2177	93	M	6	6	no	vertical	vertical	left	no	0	0	67,9	no
Stuttgart	Grand Prix of Stuttgart presented by Mercedes-Benz, WALTER solar	19.11.2017	CSI-W5*	4	1.60	410	164	15	15.03.2003	G	SF	197	13	71	12	44	2177	93	M	7	7a	yes	spread	oxer	left	no	0	0	67,9	no
Stuttgart	Grand Prix of Stuttgart presented by Mercedes-Benz, WALTER solar	19.11.2017	CSI-W5*	4	1.60	410	164	15	15.03.2003	G	SF	197	13	71	12	44	2177	93	M	8	7b	yes	vertical	vertical	left	no	0	0	67,9	no
Stuttgart	Grand Prix of Stuttgart presented by Mercedes-Benz, WALTER solar	19.11.2017	CSI-W5*	4	1.60	410	164	15	15.03.2003	G	SF	197	13	71	12	44	2177	93	M	9	7c	yes	spread	oxer	left	no	0	0	67,9	no
Stuttgart	Grand Prix of Stuttgart presented by Mercedes-Benz, WALTER solar	19.11.2017	CSI-W5*	4	1.60	410	164	15	15.03.2003	G	SF	197	13	71	12	44	2177	93	M	10	8	no	spread	oxer	left	no	0	0	67,9	no
Stuttgart	Grand Prix of Stuttgart presented by Mercedes-Benz, WALTER solar	19.11.2017	CSI-W5*	4	1.60	410	164	15	15.03.2003	G	SF	197	13	71	12	44	2177	93	M	11	9	no	vertical	vertical	left	no	0	0	67,9	no
Stuttgart	Grand Prix of Stuttgart presented by Mercedes-Benz, WALTER solar	19.11.2017	CSI-W5*	4	1.60	410	164	15	15.03.2003	G	SF	197	13	71	12	44	2177	93	M	12	10	no	spread	oxer	right	no	0	0	67,9	no
Stuttgart	Grand Prix of Stuttgart presented by Mercedes-Benz, WALTER solar	19.11.2017	CSI-W5*	4	1.60	410	164	15	15.03.2003	G	SF	197	13	71	12	44	2177	93	M	13	11	no	vertical	vertical	right	no	0	0	67,9	no
Stuttgart	Grand Prix of Stuttgart presented by Mercedes-Benz, WALTER solar	19.11.2017	CSI-W5*	4	1.60	410	164	15	15.03.2003	G	SF	197	13	71	12	44	2177	93	M	14	12a	yes	vertical	vertical	right	yes	4	0	67,9	no
Stuttgart	Grand Prix of Stuttgart presented by Mercedes-Benz, WALTER solar	19.11.2017	CSI-W5*	4	1.60	410	164	15	15.03.2003	G	SF	197	13	71	12	44	2177	93	M	15	12b	yes	spread	oxer with water	right	no	0	0	67,9	no
Stuttgart	Grand Prix of Stuttgart presented by Mercedes-Benz, WALTER solar	19.11.2017	CSI-W5*	4	1.60	410	164	15	15.03.2003	G	SF	197	13	71	12	44	2177	93	M	16	13	no	spread	oxer with water	straight	no	0	0	67,9	no
Stuttgart	Grand Prix of Stuttgart presented by Mercedes-Benz, WALTER solar	19.11.2017	CSI-W5*	4	1.60	410	173	13	14.07.2005	G	SF	229	2	74	65	34	1260	61	M	1	1	no	vertical	wall	right	no	0	0	69,1	no
Stuttgart	Grand Prix of Stuttgart presented by Mercedes-Benz, WALTER solar	19.11.2017	CSI-W5*	4	1.60	410	173	13	14.07.2005	G	SF	229	2	74	65	34	1260	61	M	2	2	no	spread	oxer	right	no	0	0	69,1	no
Stuttgart	Grand Prix of Stuttgart presented by Mercedes-Benz, WALTER solar	19.11.2017	CSI-W5*	4	1.60	410	173	13	14.07.2005	G	SF	229	2	74	65	34	1260	61	M	3	3	no	vertical	vertical	right	no	0	0	69,1	no
Stuttgart	Grand Prix of Stuttgart presented by Mercedes-Benz, WALTER solar	19.11.2017	CSI-W5*	4	1.60	410	173	13	14.07.2005	G	SF	229	2	74	65	34	1260	61	M	4	4	no	spread	oxer	straight	no	0	0	69,1	no
Stuttgart	Grand Prix of Stuttgart presented by Mercedes-Benz, WALTER solar	19.11.2017	CSI-W5*	4	1.60	410	173	13	14.07.2005	G	SF	229	2	74	65	34	1260	61	M	5	5	no	spread	oxer	straight	no	0	0	69,1	no
Stuttgart	Grand Prix of Stuttgart presented by Mercedes-Benz, WALTER solar	19.11.2017	CSI-W5*	4	1.60	410	173	13	14.07.2005	G	SF	229	2	74	65	34	1260	61	M	6	6	no	vertical	vertical	left	no	0	0	69,1	no
Stuttgart	Grand Prix of Stuttgart presented by Mercedes-Benz, WALTER solar	19.11.2017	CSI-W5*	4	1.60	410	173	13	14.07.2005	G	SF	229	2	74	65	34	1260	61	M	7	7a	yes	spread	oxer	left	no	0	0	69,1	no
Stuttgart	Grand Prix of Stuttgart presented by Mercedes-Benz, WALTER solar	19.11.2017	CSI-W5*	4	1.60	410	173	13	14.07.2005	G	SF	229	2	74	65	34	1260	61	M	8	7b	yes	vertical	vertical	left	no	0	0	69,1	no
Stuttgart	Grand Prix of Stuttgart presented by Mercedes-Benz, WALTER solar	19.11.2017	CSI-W5*	4	1.60	410	173	13	14.07.2005	G	SF	229	2	74	65	34	1260	61	M	9	7c	yes	spread	oxer	left	no	0	0	69,1	no
Stuttgart	Grand Prix of Stuttgart presented by Mercedes-Benz, WALTER solar	19.11.2017	CSI-W5*	4	1.60	410	173	13	14.07.2005	G	SF	229	2	74	65	34	1260	61	M	10	8	no	spread	oxer	left	no	0	0	69,1	no
Stuttgart	Grand Prix of Stuttgart presented by Mercedes-Benz, WALTER solar	19.11.2017	CSI-W5*	4	1.60	410	173	13	14.07.2005	G	SF	229	2	74	65	34	1260	61	M	11	9	no	vertical	vertical	left	no	0	0	69,1	no
Stuttgart	Grand Prix of Stuttgart presented by Mercedes-Benz, WALTER solar	19.11.2017	CSI-W5*	4	1.60	410	173	13	14.07.2005	G	SF	229	2	74	65	34	1260	61	M	12	10	no	spread	oxer	right	no	0	0	69,1	no
Stuttgart	Grand Prix of Stuttgart presented by Mercedes-Benz, WALTER solar	19.11.2017	CSI-W5*	4	1.60	410	173	13	14.07.2005	G	SF	229	2	74	65	34	1260	61	M	13	11	no	vertical	vertical	right	no	0	0	69,1	no
Stuttgart	Grand Prix of Stuttgart presented by Mercedes-Benz, WALTER solar	19.11.2017	CSI-W5*	4	1.60	410	173	13	14.07.2005	G	SF	229	2	74	65	34	1260	61	M	14	12a	yes	vertical	vertical	right	no	0	0	69,1	no
Stuttgart	Grand Prix of Stuttgart presented by Mercedes-Benz, WALTER solar	19.11.2017	CSI-W5*	4	1.60	410	173	13	14.07.2005	G	SF	229	2	74	65	34	1260	61	M	15	12b	yes	spread	oxer with water	right	no	0	0	69,1	no
Stuttgart	Grand Prix of Stuttgart presented by Mercedes-Benz, WALTER solar	19.11.2017	CSI-W5*	4	1.60	410	173	13	14.07.2005	G	SF	229	2	74	65	34	1260	61	M	16	13	no	spread	oxer with water	straight	no	0	0	69,1	no
Stuttgart	Grand Prix of Stuttgart presented by Mercedes-Benz, WALTER solar	19.11.2017	CSI-W5*	4	1.60	380	173	13	14.07.2005	G	SF	229	2	74	65	34	1260	61	M	1	1	no	spread	oxer	left	no	0	0	50,22	no
Stuttgart	Grand Prix of Stuttgart presented by Mercedes-Benz, WALTER solar	19.11.2017	CSI-W5*	4	1.60	380	173	13	14.07.2005	G	SF	229	2	74	65	34	1260	61	M	2	2	no	vertical	vertical	left	no	0	0	50,22	no
Stuttgart	Grand Prix of Stuttgart presented by Mercedes-Benz, WALTER solar	19.11.2017	CSI-W5*	4	1.60	380	173	13	14.07.2005	G	SF	229	2	74	65	34	1260	61	M	3	3	no	vertical	vertical	right	no	0	0	50,22	no
Stuttgart	Grand Prix of Stuttgart presented by Mercedes-Benz, WALTER solar	19.11.2017	CSI-W5*	4	1.60	380	173	13	14.07.2005	G	SF	229	2	74	65	34	1260	61	M	4	4	no	spread	oxer with water	right	no	0	0	50,22	no
Stuttgart	Grand Prix of Stuttgart presented by Mercedes-Benz, WALTER solar	19.11.2017	CSI-W5*	4	1.60	380	173	13	14.07.2005	G	SF	229	2	74	65	34	1260	61	M	5	5	no	vertical	vertical	left	no	0	0	50,22	no
Stuttgart	Grand Prix of Stuttgart presented by Mercedes-Benz, WALTER solar	19.11.2017	CSI-W5*	4	1.60	380	173	13	14.07.2005	G	SF	229	2	74	65	34	1260	61	M	6	6	no	vertical	wall	right	no	0	0	50,22	no
Stuttgart	Grand Prix of Stuttgart presented by Mercedes-Benz, WALTER solar	19.11.2017	CSI-W5*	4	1.60	380	173	13	14.07.2005	G	SF	229	2	74	65	34	1260	61	M	7	7	no	spread	oxer	right	no	0	0	50,22	no
Stuttgart	Grand Prix of Stuttgart presented by Mercedes-Benz, WALTER solar	19.11.2017	CSI-W5*	4	1.60	380	173	13	14.07.2005	G	SF	229	2	74	65	34	1260	61	M	8	8a	yes	spread	oxer	left	no	0	0	50,22	no
Stuttgart	Grand Prix of Stuttgart presented by Mercedes-Benz, WALTER solar	19.11.2017	CSI-W5*	4	1.60	380	173	13	14.07.2005	G	SF	229	2	74	65	34	1260	61	M	9	8b	yes	vertical	vertical	left	no	0	0	50,22	no
Stuttgart	Grand Prix of Stuttgart presented by Mercedes-Benz, WALTER solar	19.11.2017	CSI-W5*	4	1.60	380	173	13	14.07.2005	G	SF	229	2	74	65	34	1260	61	M	10	9	no	spread	oxer	left	no	0	0	50,22	no
Stuttgart	Grand Prix of Stuttgart presented by Mercedes-Benz, WALTER solar	19.11.2017	CSI-W5*	4	1.60	410	176	12	21.05.2006	M	SF	110	2	75	22	53	2104	161	M	1	1	no	vertical	wall	right	no	0	0	68,45	no
Stuttgart	Grand Prix of Stuttgart presented by Mercedes-Benz, WALTER solar	19.11.2017	CSI-W5*	4	1.60	410	176	12	21.05.2006	M	SF	110	2	75	22	53	2104	161	M	2	2	no	spread	oxer	right	no	0	0	68,45	no
Stuttgart	Grand Prix of Stuttgart presented by Mercedes-Benz, WALTER solar	19.11.2017	CSI-W5*	4	1.60	410	176	12	21.05.2006	M	SF	110	2	75	22	53	2104	161	M	3	3	no	vertical	vertical	right	no	0	0	68,45	no
Stuttgart	Grand Prix of Stuttgart presented by Mercedes-Benz, WALTER solar	19.11.2017	CSI-W5*	4	1.60	410	176	12	21.05.2006	M	SF	110	2	75	22	53	2104	161	M	4	4	no	spread	oxer	straight	no	0	0	68,45	no
Stuttgart	Grand Prix of Stuttgart presented by Mercedes-Benz, WALTER solar	19.11.2017	CSI-W5*	4	1.60	410	176	12	21.05.2006	M	SF	110	2	75	22	53	2104	161	M	5	5	no	spread	oxer	straight	no	0	0	68,45	no
Stuttgart	Grand Prix of Stuttgart presented by Mercedes-Benz, WALTER solar	19.11.2017	CSI-W5*	4	1.60	410	176	12	21.05.2006	M	SF	110	2	75	22	53	2104	161	M	6	6	no	vertical	vertical	left	no	0	0	68,45	no
Stuttgart	Grand Prix of Stuttgart presented by Mercedes-Benz, WALTER solar	19.11.2017	CSI-W5*	4	1.60	410	176	12	21.05.2006	M	SF	110	2	75	22	53	2104	161	M	7	7a	yes	spread	oxer	left	no	0	0	68,45	no
Stuttgart	Grand Prix of Stuttgart presented by Mercedes-Benz, WALTER solar	19.11.2017	CSI-W5*	4	1.60	410	176	12	21.05.2006	M	SF	110	2	75	22	53	2104	161	M	8	7b	yes	vertical	vertical	left	no	0	0	68,45	no
Stuttgart	Grand Prix of Stuttgart presented by Mercedes-Benz, WALTER solar	19.11.2017	CSI-W5*	4	1.60	410	176	12	21.05.2006	M	SF	110	2	75	22	53	2104	161	M	9	7c	yes	spread	oxer	left	no	0	0	68,45	no
Stuttgart	Grand Prix of Stuttgart presented by Mercedes-Benz, WALTER solar	19.11.2017	CSI-W5*	4	1.60	410	176	12	21.05.2006	M	SF	110	2	75	22	53	2104	161	M	10	8	no	spread	oxer	left	no	0	0	68,45	no
Stuttgart	Grand Prix of Stuttgart presented by Mercedes-Benz, WALTER solar	19.11.2017	CSI-W5*	4	1.60	410	176	12	21.05.2006	M	SF	110	2	75	22	53	2104	161	M	11	9	no	vertical	vertical	left	no	0	0	68,45	no
Stuttgart	Grand Prix of Stuttgart presented by Mercedes-Benz, WALTER solar	19.11.2017	CSI-W5*	4	1.60	410	176	12	21.05.2006	M	SF	110	2	75	22	53	2104	161	M	12	10	no	spread	oxer	right	no	0	0	68,45	no
Stuttgart	Grand Prix of Stuttgart presented by Mercedes-Benz, WALTER solar	19.11.2017	CSI-W5*	4	1.60	410	176	12	21.05.2006	M	SF	110	2	75	22	53	2104	161	M	13	11	no	vertical	vertical	right	no	0	0	68,45	no
Stuttgart	Grand Prix of Stuttgart presented by Mercedes-Benz, WALTER solar	19.11.2017	CSI-W5*	4	1.60	410	176	12	21.05.2006	M	SF	110	2	75	22	53	2104	161	M	14	12a	yes	vertical	vertical	right	no	0	0	68,45	no
Stuttgart	Grand Prix of Stuttgart presented by Mercedes-Benz, WALTER solar	19.11.2017	CSI-W5*	4	1.60	410	176	12	21.05.2006	M	SF	110	2	75	22	53	2104	161	M	15	12b	yes	spread	oxer with water	right	no	0	0	68,45	no
Stuttgart	Grand Prix of Stuttgart presented by Mercedes-Benz, WALTER solar	19.11.2017	CSI-W5*	4	1.60	410	176	12	21.05.2006	M	SF	110	2	75	22	53	2104	161	M	16	13	no	spread	oxer with water	straight	no	0	0	68,45	no
Stuttgart	Grand Prix of Stuttgart presented by Mercedes-Benz, WALTER solar	19.11.2017	CSI-W5*	4	1.60	380	176	12	21.05.2006	M	SF	110	2	75	22	53	2104	161	M	1	1	no	spread	oxer	left	no	0	0	48,99	no

Stuttgart	Grand Prix of Stuttgart presented by Mercedes-Benz, WALTER solar	19.11.2017	CSI-W5*	4	1.60	380	176	12	21.05.2006	M	SF	110	2	75	22	53	2104	161	M	2	2	no	vertical	vertical	left	no	0	0	48,99	no
Stuttgart	Grand Prix of Stuttgart presented by Mercedes-Benz, WALTER solar	19.11.2017	CSI-W5*	4	1.60	380	176	12	21.05.2006	M	SF	110	2	75	22	53	2104	161	M	3	3	no	vertical	vertical	right	no	0	0	48,99	no
Stuttgart	Grand Prix of Stuttgart presented by Mercedes-Benz, WALTER solar	19.11.2017	CSI-W5*	4	1.60	380	176	12	21.05.2006	M	SF	110	2	75	22	53	2104	161	M	4	4	no	spread	oxer with water	right	no	0	0	48,99	no
Stuttgart	Grand Prix of Stuttgart presented by Mercedes-Benz, WALTER solar	19.11.2017	CSI-W5*	4	1.60	380	176	12	21.05.2006	M	SF	110	2	75	22	53	2104	161	M	5	5	no	vertical	vertical	left	no	0	0	48,99	no
Stuttgart	Grand Prix of Stuttgart presented by Mercedes-Benz, WALTER solar	19.11.2017	CSI-W5*	4	1.60	380	176	12	21.05.2006	M	SF	110	2	75	22	53	2104	161	M	6	6	no	vertical	wall	right	no	0	0	48,99	no
Stuttgart	Grand Prix of Stuttgart presented by Mercedes-Benz, WALTER solar	19.11.2017	CSI-W5*	4	1.60	380	176	12	21.05.2006	M	SF	110	2	75	22	53	2104	161	M	7	7	no	spread	oxer	right	no	0	0	48,99	no
Stuttgart	Grand Prix of Stuttgart presented by Mercedes-Benz, WALTER solar	19.11.2017	CSI-W5*	4	1.60	380	176	12	21.05.2006	M	SF	110	2	75	22	53	2104	161	M	8	8a	yes	spread	oxer	left	no	0	0	48,99	no
Stuttgart	Grand Prix of Stuttgart presented by Mercedes-Benz, WALTER solar	19.11.2017	CSI-W5*	4	1.60	380	176	12	21.05.2006	M	SF	110	2	75	22	53	2104	161	M	9	8b	yes	vertical	vertical	left	yes	4	0	48,99	no
Stuttgart	Grand Prix of Stuttgart presented by Mercedes-Benz, WALTER solar	19.11.2017	CSI-W5*	4	1.60	380	176	12	21.05.2006	M	SF	110	2	75	22	53	2104	161	M	10	9	no	spread	oxer	left	no	0	0	48,99	no
Stuttgart	Grand Prix of Stuttgart presented by Mercedes-Benz, WALTER solar	19.11.2017	CSI-W5*	4	1.60	410	120	11	28.04.2007	S	HOLST	147	1	76	55	44	1886	48	M	1	1	no	vertical	wall	right	no	0	0	67,71	no
Stuttgart	Grand Prix of Stuttgart presented by Mercedes-Benz, WALTER solar	19.11.2017	CSI-W5*	4	1.60	410	120	11	28.04.2007	S	HOLST	147	1	76	55	44	1886	48	M	2	2	no	spread	oxer	right	no	0	0	67,71	no
Stuttgart	Grand Prix of Stuttgart presented by Mercedes-Benz, WALTER solar	19.11.2017	CSI-W5*	4	1.60	410	120	11	28.04.2007	S	HOLST	147	1	76	55	44	1886	48	M	3	3	no	vertical	vertical	right	no	0	0	67,71	no
Stuttgart	Grand Prix of Stuttgart presented by Mercedes-Benz, WALTER solar	19.11.2017	CSI-W5*	4	1.60	410	120	11	28.04.2007	S	HOLST	147	1	76	55	44	1886	48	M	4	4	no	spread	oxer	straight	no	0	0	67,71	no
Stuttgart	Grand Prix of Stuttgart presented by Mercedes-Benz, WALTER solar	19.11.2017	CSI-W5*	4	1.60	410	120	11	28.04.2007	S	HOLST	147	1	76	55	44	1886	48	M	5	5	no	spread	oxer	straight	no	0	0	67,71	no
Stuttgart	Grand Prix of Stuttgart presented by Mercedes-Benz, WALTER solar	19.11.2017	CSI-W5*	4	1.60	410	120	11	28.04.2007	S	HOLST	147	1	76	55	44	1886	48	M	6	6	no	vertical	vertical	left	no	0	0	67,71	no
Stuttgart	Grand Prix of Stuttgart presented by Mercedes-Benz, WALTER solar	19.11.2017	CSI-W5*	4	1.60	410	120	11	28.04.2007	S	HOLST	147	1	76	55	44	1886	48	M	7	7a	yes	spread	oxer	left	no	0	0	67,71	no
Stuttgart	Grand Prix of Stuttgart presented by Mercedes-Benz, WALTER solar	19.11.2017	CSI-W5*	4	1.60	410	120	11	28.04.2007	S	HOLST	147	1	76	55	44	1886	48	M	8	7b	yes	vertical	vertical	left	no	0	0	67,71	no
Stuttgart	Grand Prix of Stuttgart presented by Mercedes-Benz, WALTER solar	19.11.2017	CSI-W5*	4	1.60	410	120	11	28.04.2007	S	HOLST	147	1	76	55	44	1886	48	M	9	7c	yes	spread	oxer	left	no	0	0	67,71	no
Stuttgart	Grand Prix of Stuttgart presented by Mercedes-Benz, WALTER solar	19.11.2017	CSI-W5*	4	1.60	410	120	11	28.04.2007	S	HOLST	147	1	76	55	44	1886	48	M	10	8	no	spread	oxer	left	no	0	0	67,71	no
Stuttgart	Grand Prix of Stuttgart presented by Mercedes-Benz, WALTER solar	19.11.2017	CSI-W5*	4	1.60	410	120	11	28.04.2007	S	HOLST	147	1	76	55	44	1886	48	M	11	9	no	vertical	vertical	left	no	0	0	67,71	no
Stuttgart	Grand Prix of Stuttgart presented by Mercedes-Benz, WALTER solar	19.11.2017	CSI-W5*	4	1.60	410	120	11	28.04.2007	S	HOLST	147	1	76	55	44	1886	48	M	12	10	no	spread	oxer	right	no	0	0	67,71	no
Stuttgart	Grand Prix of Stuttgart presented by Mercedes-Benz, WALTER solar	19.11.2017	CSI-W5*	4	1.60	410	120	11	28.04.2007	S	HOLST	147	1	76	55	44	1886	48	M	13	11	no	vertical	vertical	right	no	0	0	67,71	no
Stuttgart	Grand Prix of Stuttgart presented by Mercedes-Benz, WALTER solar	19.11.2017	CSI-W5*	4	1.60	410	120	11	28.04.2007	S	HOLST	147	1	76	55	44	1886	48	M	14	12a	yes	vertical	vertical	right	no	0	0	67,71	no
Stuttgart	Grand Prix of Stuttgart presented by Mercedes-Benz, WALTER solar	19.11.2017	CSI-W5*	4	1.60	410	120	11	28.04.2007	S	HOLST	147	1	76	55	44	1886	48	M	15	12b	yes	spread	oxer with water	right	no	0	0	67,71	no
Stuttgart	Grand Prix of Stuttgart presented by Mercedes-Benz, WALTER solar	19.11.2017	CSI-W5*	4	1.60	410	120	11	28.04.2007	S	HOLST	147	1	76	55	44	1886	48	M	16	13	no	spread	oxer with water	straight	no	0	0	67,71	no
Stuttgart	Grand Prix of Stuttgart presented by Mercedes-Benz, WALTER solar	19.11.2017	CSI-W5*	4	1.60	380	120	11	28.04.2007	S	HOLST	147	1	76	55	44	1886	48	M	1	1	no	spread	oxer	left	no	0	0	55,91	no
Stuttgart	Grand Prix of Stuttgart presented by Mercedes-Benz, WALTER solar	19.11.2017	CSI-W5*	4	1.60	380	120	11	28.04.2007	S	HOLST	147	1	76	55	44	1886	48	M	2	2	no	vertical	vertical	left	no	0	0	55,91	no
Stuttgart	Grand Prix of Stuttgart presented by Mercedes-Benz, WALTER solar	19.11.2017	CSI-W5*	4	1.60	380	120	11	28.04.2007	S	HOLST	147	1	76	55	44	1886	48	M	3	3	no	vertical	vertical	right	no	0	0	55,91	no
Stuttgart	Grand Prix of Stuttgart presented by Mercedes-Benz, WALTER solar	19.11.2017	CSI-W5*	4	1.60	380	120	11	28.04.2007	S	HOLST	147	1	76	55	44	1886	48	M	4	4	no	spread	oxer with water	right	no	0	0	55,91	no
Stuttgart	Grand Prix of Stuttgart presented by Mercedes-Benz, WALTER solar	19.11.2017	CSI-W5*	4	1.60	380	120	11	28.04.2007	S	HOLST	147	1	76	55	44	1886	48	M	5	5	no	vertical	vertical	left	no	0	0	55,91	no
Stuttgart	Grand Prix of Stuttgart presented by Mercedes-Benz, WALTER solar	19.11.2017	CSI-W5*	4	1.60	380	120	11	28.04.2007	S	HOLST	147	1	76	55	44	1886	48	M	6	6	no	vertical	wall	right	no	0	0	55,91	no
Stuttgart	Grand Prix of Stuttgart presented by Mercedes-Benz, WALTER solar	19.11.2017	CSI-W5*	4	1.60	380	120	11	28.04.2007	S	HOLST	147	1	76	55	44	1886	48	M	7	7	no	spread	oxer	right	no	0	0	55,91	no
Stuttgart	Grand Prix of Stuttgart presented by Mercedes-Benz, WALTER solar	19.11.2017	CSI-W5*	4	1.60	380	120	11	28.04.2007	S	HOLST	147	1	76	55	44	1886	48	M	8	8a	yes	spread	oxer	left	no	0	0	55,91	no
Stuttgart	Grand Prix of Stuttgart presented by Mercedes-Benz, WALTER solar	19.11.2017	CSI-W5*	4	1.60	380	120	11	28.04.2007	S	HOLST	147	1	76	55	44	1886	48	M	9	8b	yes	vertical	vertical	left	no	0	0	55,91	no
Stuttgart	Grand Prix of Stuttgart presented by Mercedes-Benz, WALTER solar	19.11.2017	CSI-W5*	4	1.60	380	120	11	28.04.2007	S	HOLST	147	1	76	55	44	1886	48	M	10	9	no	spread	oxer	left	no	0	0	55,91	no
Stuttgart	Grand Prix of Stuttgart presented by Mercedes-Benz, WALTER solar	19.11.2017	CSI-W5*	4	1.60	410	123	10	05.04.2008	S	WESTF	82	0	79	166	25	1567	29	M	1	1	no	vertical	wall	right	no	0	0	69,02	no
Stuttgart	Grand Prix of Stuttgart presented by Mercedes-Benz, WALTER solar	19.11.2017	CSI-W5*	4	1.60	410	123	10	05.04.2008	S	WESTF	82	0	79	166	25	1567	29	M	2	2	no	spread	oxer	right	no	0	0	69,02	no
Stuttgart	Grand Prix of Stuttgart presented by Mercedes-Benz, WALTER solar	19.11.2017	CSI-W5*	4	1.60	410	123	10	05.04.2008	S	WESTF	82	0	79	166	25	1567	29	M	3	3	no	vertical	vertical	right	no	0	0	69,02	no
Stuttgart	Grand Prix of Stuttgart presented by Mercedes-Benz, WALTER solar	19.11.2017	CSI-W5*	4	1.60	410	123	10	05.04.2008	S	WESTF	82	0	79	166	25	1567	29	M	4	4	no	spread	oxer	straight	no	0	0	69,02	no
Stuttgart	Grand Prix of Stuttgart presented by Mercedes-Benz, WALTER solar	19.11.2017	CSI-W5*	4	1.60	410	123	10	05.04.2008	S	WESTF	82	0	79	166	25	1567	29	M	5	5	no	spread	oxer	straight	no	0	0	69,02	no
Stuttgart	Grand Prix of Stuttgart presented by Mercedes-Benz, WALTER solar	19.11.2017	CSI-W5*	4	1.60	410	123	10	05.04.2008	S	WESTF	82	0	79	166	25	1567	29	M	6	6	no	vertical	vertical	left	no	0	0	69,02	no
Stuttgart	Grand Prix of Stuttgart presented by Mercedes-Benz, WALTER solar	19.11.2017	CSI-W5*	4	1.60	410	123	10	05.04.2008	S	WESTF	82	0	79	166	25	1567	29	M	7	7a	yes	spread	oxer	left	yes	4	0	69,02	no
Stuttgart	Grand Prix of Stuttgart presented by Mercedes-Benz, WALTER solar	19.11.2017	CSI-W5*	4	1.60	410	123	10	05.04.2008	S	WESTF	82	0	79	166	25	1567	29	M	8	7b	yes	vertical	vertical	left	no	0	0	69,02	no
Stuttgart	Grand Prix of Stuttgart presented by Mercedes-Benz, WALTER solar	19.11.2017	CSI-W5*	4	1.60	410	123	10	05.04.2008	S	WESTF	82	0	79	166	25	1567	29	M	9	7c	yes	spread	oxer	left	no	0	0	69,02	no
Stuttgart	Grand Prix of Stuttgart presented by Mercedes-Benz, WALTER solar	19.11.2017	CSI-W5*	4	1.60	410	123	10	05.04.2008	S	WESTF	82	0	79	166	25	1567	29	M	10	8	no	spread	oxer	left	yes	4	0	69,02	no
Stuttgart	Grand Prix of Stuttgart presented by Mercedes-Benz, WALTER solar	19.11.2017	CSI-W5*	4	1.60	410	123	10	05.04.2008	S	WESTF	82	0	79	166	25	1567	29	M	11	9	no	vertical	vertical	left	no	0	0	69,02	no
Stuttgart	Grand Prix of Stuttgart presented by Mercedes-Benz, WALTER solar	19.11.2017	CSI-W5*	4	1.60	410	123	10	05.04.2008	S	WESTF	82	0	79	166	25	1567	29	M	12	10	no	spread	oxer	right	no	0	0	69,02	no
Stuttgart	Grand Prix of Stuttgart presented by Mercedes-Benz, WALTER solar	19.11.2017	CSI-W5*	4	1.60	410	123	10	05.04.2008	S	WESTF	82	0	79	166	25	1567	29	M	13	11	no	vertical	vertical	right	no	0	0	69,02	no
Stuttgart	Grand Prix of Stuttgart presented by Mercedes-Benz, WALTER solar	19.11.2017	CSI-W5*	4	1.60	410	123	10	05.04.2008	S	WESTF	82	0	79	166	25	1567	29	M	14	12a	yes	vertical	vertical	right	no	0	0	69,02	no
Stuttgart	Grand Prix of Stuttgart presented by Mercedes-Benz, WALTER solar	19.11.2017	CSI-W5*	4	1.60	410	123	10	05.04.2008	S	WESTF	82	0	79	166	25	1567	29	M	15	12b	yes	spread	oxer with water	right	no	0	0	69,02	no
Stuttgart	Grand Prix of Stuttgart presented by Mercedes-Benz, WALTER solar	19.11.2017	CSI-W5*	4	1.60	410	123	10	05.04.2008	S	WESTF	82	0	79	166	25	1567	29	M	16	13	no	spread	oxer with water	straight	no	0	0	69,02	no
Stuttgart	Grand Prix of Stuttgart presented by Mercedes-Benz, WALTER solar	19.11.2017	CSI-W5*	4	1.60	410	90	14	01.03.2004	M	HANN	185	6	86	36	48	1157	25	F	1	1	no	vertical	wall	right	no	0	0	0,00	yes
Stuttgart	Grand Prix of Stuttgart presented by Mercedes-Benz, WALTER solar	19.11.2017	CSI-W5*	4	1.60	410	90	14	01.03.2004	M	HANN	185	6	86	36	48	1157	25	F	2	2	no	spread	oxer	right	no	0	0	0,00	yes
Stuttgart	Grand Prix of Stuttgart presented by Mercedes-Benz, WALTER solar	19.11.2017	CSI-W5*	4	1.60	410	90	14	01.03.2004	M	HANN	185	6	86	36	48	1157	25	F	3	3	no	vertical	vertical	right	no	0	0	0,00	yes
Stuttgart	Grand Prix of Stuttgart presented by Mercedes-Benz, WALTER solar	19.11.2017	CSI-W5*	4	1.60	410	90	14	01.03.2004	M	HANN	185	6	86	36	48	1157	25	F	4	4	no	spread	oxer	straight	no	0	0	0,00	yes
Stuttgart	Grand Prix of Stuttgart presented by Mercedes-Benz, WALTER solar	19.11.2017	CSI-W5*	4	1.60	410	90	14	01.03.2004	M	HANN	185	6	86	36	48	1157	25	F	5	5	no	spread	oxer	straight	no	0	0	0,00	yes
Stuttgart	Grand Prix of Stuttgart presented by Mercedes-Benz, WALTER solar	19.11.2017	CSI-W5*	4	1.60	410	90	14	01.03.2004	M	HANN	185	6	86	36	48	1157	25	F	6	6	no	vertical	vertical	left	no	0	0	0,00	yes
Stuttgart	Grand Prix of Stuttgart presented by Mercedes-Benz, WALTER solar	19.11.2017	CSI-W5*	4	1.60	410	90	14	01.03.2004	M	HANN	185	6	86	36	48	1157	25	F	7	7a	yes	spread	oxer	left	no	0	0	0,00	yes
Stuttgart	Grand Prix of Stuttgart presented by Mercedes-Benz, WALTER solar	19.11.2017	CSI-W5*	4	1.60	410	90	14	01.03.2004	M	HANN	185	6	86	36	48	1157	25	F	8	7b	yes	vertical	vertical	left	no	0	0	0,00	yes
Stuttgart	Grand Prix of Stuttgart presented by Mercedes-Benz, WALTER solar	19.11.2017	CSI-W5*	4	1.60	410	90	14	01.03.2004	M	HANN	185	6	86	36	48	1157	25	F	9	7c	yes	spread	oxer	left	no	0	0	0,00	yes
Stuttgart	Grand Prix of Stuttgart presented by Mercedes-Benz, WALTER solar	19.11.2017	CSI-W5*	4	1.60	410	90	14	01.03.2004	M	HANN	185	6	86	36	48	1157	25	F	10	8	no	spread	oxer	left	yes	4	0	0,00	yes
Stuttgart	Grand Prix of Stuttgart presented by Mercedes-Benz, WALTER solar	19.11.2017	CSI-W5*	4	1.60	410	90	14	01.03.2004	M	HANN	185	6	86	36	48	1157	25	F	11	9	no	vertical	vertical	left	no	0	0	0,00	yes
Stuttgart	Grand Prix of Stuttgart presented by Mercedes-Benz, WALTER solar	19.11.2017	CSI-W5*	4	1.60	410	90	14	01.03.2004	M	HANN	185	6	86	36	48	1157	25	F	12	10	no	spread	oxer	right	no	0	0	0,00	yes
Stuttgart	Grand Prix of Stuttgart presented by Mercedes-Benz, WALTER solar	19.11.2017	CSI-W5*	4	1.60	410	90	14	01.03.2004	M	HANN	185	6	86	36	48	1157	25	F	13	11	no	vertical	vertical	right	no	0	0	0,00	yes
Stuttgart	Grand Prix of Stuttgart presented by Mercedes-Benz, WALTER solar	19.11.2017	CSI-W5*	4	1.60	410	90	14	01.03.2004	M	HANN	185	6	86	36	48	1157	25	F	14	12a	yes	vertical	vertical	right	no	0	0	0,00	yes
Stuttgart	Grand Prix of Stuttgart presented by Mercedes-Benz, WALTER solar	19.11.2017	CSI-W5*	4	1.60	410	90	14	01.03.2004	M	HANN	185	6	86	36	48	1157	25	F	15	12b	yes	spread	oxer with water	right	no	0	0	0,00	yes
Stuttgart	Grand Prix of Stuttgart presented by Mercedes-Benz, WALTER solar	19.11.2017	CSI-W5*	4	1.60	410	90	14	01.03.2004	M	HANN	185	6	86	36	48	1157	25	F	16	13	no	spread	oxer with water	straight	no	0	0	0,00	yes
Stuttgart	Grand Prix of Stuttgart presented by Mercedes-Benz, WALTER solar	19.11.2017	CSI-W5*	4	1.60	410	17	13	04.06.2005	S	HOLST	172	4	87	199	55	1406	43	M	1	1	no	vertical	wall	right	no	0	0	0,00	yes
Stuttgart	Grand Prix of Stuttgart presented by Mercedes-Benz, WALTER solar	19.11.2017	CSI-W5*	4	1.60	410	17	13	04.06.2005	S	HOLST	172	4	87	199	55	1406	43	M	2	2	no	spread	oxer	right	no	0	0	0,00	yes
Stuttgart	Grand Prix of Stuttgart presented by Mercedes-Benz, WALTER solar	19.11.2017	CSI-W5*	4	1.60	410	17	13	04.06.2005	S	HOLST	172	4	87	199	55	1406	43	M	3	3	no	vertical	vertical	right	no	0	0	0,00	yes
Stuttgart	Grand Prix of Stuttgart presented by Mercedes-Benz, WALTER solar	19.11.2017	CSI-W5*	4	1.60	410	17	13	04.06.2005	S	HOLST	172	4	87	199	55	1406	43	M	4	4	no	spread	oxer	straight	no	0	0	0,00	yes
Stuttgart	Grand Prix of Stuttgart presented by Mercedes-Benz, WALTER solar	19.11.2017	CSI-W5*	4	1.60	410	17	13	04.06.2005	S	HOLST	172	4	87	199	55	1406	43	M	5	5	no	spread	oxer	straight	no	0	0	0,00	yes
Stuttgart	Grand Prix of Stuttgart presented by Mercedes-Benz, WALTER solar	19.11.2017	CSI-W5*	4	1.60	410	17	13	04.06.2005	S	HOLST	172	4	87	199	55	1406	43	M	6	6	no	vertical	vertical	left	no	0	0	0,00	yes
Stuttgart	Grand Prix of Stuttgart presented by Mercedes-Benz, WALTER solar	19.11.2017	CSI-W5*	4	1.60	410	17	13	04.06.2005	S	HOLST	172	4	87	199	55	1406	43	M	7	7a	yes	spread	oxer	left	yes	4	0	0,00	yes
Stuttgart	Grand Prix of Stuttgart presented by Mercedes-Benz, WALTER solar	19.11.2017	CSI-W5*	4	1.60	410	17	13	04.06.2005	S	HOLST	172	4	87	199	55	1406	43	M	8	7b	yes	vertical	vertical	left	no	0	0	0,00	yes
Stuttgart	Grand Prix of Stuttgart presented by Mercedes-Benz, WALTER solar	19.11.2017	CSI-W5*	4	1.60	410	17	13	04.06.2005	S	HOLST	172	4	87	199	55	1406	43	M	9	7c	yes	spread	oxer	left	yes	4	0	0,00	yes
Stuttgart	Grand Prix of Stuttgart presented by Mercedes-Benz, WALTER solar	19.11.2017	CSI-W5*	4	1.60	410	17	13	04.06.2005	S	HOLST	172	4	87	199	55	1406	43	M	10	8	no	spread	oxer	left	yes	4	0	0,00	yes
Stuttgart	Grand Prix of Stuttgart presented by Mercedes-Benz, WALTER solar	19.11.2017	CSI-W5*	4	1.60	410	17	13	04.06.2005	S	HOLST	172	4	87	199	55	1406	43	M	11	9	no	vertical	vertical	left	no	0	0	0,00	yes
Stuttgart	Grand Prix of Stuttgart presented by Mercedes-Benz, WALTER solar	19.11.2017	CSI-W5*	4	1.60	410	17	13	04.06.2005	S	HOLST	172	4	87	199	55	1406	43	M	12	10	no	spread	oxer	right	yes	4	0	0,00	yes
Stuttgart	Grand Prix of Stuttgart presented by Mercedes-Benz, WALTER solar	19.11.2017	CSI-W5*	4	1.60	410	17	13	04.06.2005	S	HOLST	172	4	87	199	55	1406	43	M	13	11	no	vertical	vertical	right	yes	4	0	0,00	yes
Stuttgart	Grand Prix of Stuttgart presented by Mercedes-Benz, WALTER solar	19.11.2017	CSI-W5*	4	1.60	410	17	13	04.06.2005	S	HOLST	172	4	87	199	55	1406	43	M	14	12a	yes	vertical	vertical	right	no	0	0	0,00	yes
Stuttgart	Grand Prix of Stuttgart presented by Mercedes-Benz, WALTER solar	19.11.2017	CSI-W5*	4	1.60	410	17	13	04.06.2005	S	HOLST	172	4	87	199	55	1406	43	M	15	12b	yes	spread	oxer with water	right	no	0	0	0,00	yes
Stuttgart	Grand Prix of Stuttgart presented by Mercedes-Benz, WALTER solar	19.11.2017	CSI-W5*	4	1.60	410	17	13	04.06.2005	S	HOLST	172	4	87	199	55	1406	43	M	16	13	no	spread	oxer with water	straight	no	0	0	0,00	yes
Stuttgart	Grand Prix of Stuttgart presented by Mercedes-Benz, WALTER solar	19.11.2017	CSI-W5*	4	1.60	410	116	11	25.05.2007	S	WESTF	157	15	90	169	24	632	50	M	1	1	no	vertical	wall	right	no	0	0	0,00	yes
Stuttgart	Grand Prix of Stuttgart presented by Mercedes-Benz, WALTER solar	19.11.2017	CSI-W5*	4	1.60	410	116	11	25.05.2007	S	WESTF	157	15	90	169	24	632	50	M	2	2	no	spread	oxer	right	no	0	0	0,00	yes
Stuttgart	Grand Prix of Stuttgart presented by Mercedes-Benz, WALTER solar	19.11.2017	CSI-W5*	4	1.60	410	116	11	25.05.2007	S	WESTF	157	15	90	169	24	632	50	M	3	3	no	vertical	vertical	right	no	0	0	0,00	yes
Stuttgart	Grand Prix of Stuttgart presented by Mercedes-Benz, WALTER solar	19.11.2017	CSI-W5*	4	1.60	410	116	11	25.05.2007	S	WESTF	157	15	90	169	24	632	50	M	4	4	no	spread	oxer	straight	no	0	0	0,00	yes
Stuttgart	Grand Prix of Stuttgart presented by Mercedes-Benz, WALTER solar	19.11.2017	CSI-W5*	4	1.60	410	116	11	25.05.2007	S	WESTF	157	15	90	169	24	632	50	M	5	5	no	spread	oxer	straight	no	0	0	0,00	yes
Stuttgart	Grand Prix of Stuttgart presented by Mercedes-Benz, WALTER solar	19.11.2017	CSI-W5*	4	1.60	410	116	11	25.05.2007	S	WESTF	157	15	90	169	24	632	50	M	6	6	no	vertical	vertical	left	no	0	0	0,00	yes
Stuttgart	Grand Prix of Stuttgart presented by Mercedes-Benz, WALTER solar	19.11.2017	CSI-W5*	4	1.60	410	116	11	25.05.2007	S	WESTF	157	15	90	169	24	632	50	M	7	7a	yes	spread	oxer	left	no	0	0	0,00	yes
Stuttgart	Grand Prix of Stuttgart presented by Mercedes-Benz, WALTER solar	19.11.2017	CSI-W5*	4	1.60	410	116	11	25.05.2007	S	WESTF	157	15	90	169	24	632	50	M	8	7b	yes	vertical	vertical	left	no	0	0	0,00	yes
Stuttgart	Grand Prix of Stuttgart presented by Mercedes-Benz, WALTER solar	19.11.2017	CSI-W5*	4	1.60	410	116	11	25.05.2007	S	WESTF	157	15	90	169	24	632	50	M	9	7c	yes	spread	oxer	left	yes	4	0	0,00	yes
Stuttgart	Grand Prix of Stuttgart presented by Mercedes-Benz, WALTER solar	19.11.2017	CSI-W5*	4	1.60	410	116	11	25.05.2007	S	WESTF	157	15	90	169	24	632	50	M	10	8	no	spread	oxer	left	no	0	0	0,00	yes
Stuttgart	Grand Prix of Stuttgart presented by Mercedes-Benz, WALTER solar	19.11.2017	CSI-W5*	4	1.60	410	116	11	25.05.2007	S	WESTF	157	15	90	169	24	632	50	M	11	9	no	vertical	vertical	left	no	0	0	0,00	yes
Stuttgart	Grand Prix of Stuttgart presented by Mercedes-Benz, WALTER solar	19.11.2017	CSI-W5*	4	1.60	410	116	11	25.05.2007	S	WESTF	157	15	90	169	24	632	50	M	12	10	no	spread	oxer	right	no	0	0	0,00	yes
Stuttgart	Grand Prix of Stuttgart presented by Mercedes-Benz, WALTER solar	19.11.2017	CSI-W5*	4	1.60	410	116	11	25.05.2007	S	WESTF	157	15	90	169	24	632	50	M	13	11	no	vertical	vertical	right	no	0	0	0,00	yes
Stuttgart	Grand Prix of Stuttgart presented by Mercedes-Benz, WALTER solar	19.11.2017	CSI-W5*	4	1.60	410	116	11	25.05.2007	S	WESTF	157	15	90	169	24	632	50	M	14	12a	yes	vertical	vertical	right	yes	4	0	0,00	yes
Stuttgart	Grand Prix of Stuttgart presented by Mercedes-Benz, WALTER solar	19.11.2017	CSI-W5*	4	1.60	410	116	11	25.05.2007	S	WESTF	157	15	90	169	24	632	50	M	15	12b	yes	spread	oxer with water	right	yes	4	0	0,00	yes
Stuttgart	Grand Prix of Stuttgart presented by Mercedes-Benz, WALTER solar	19.11.2017	CSI-W5*	4	1.60	410	116	11	25.05.2007	S	WESTF	157	15	90	169	24	632	50	M	16	13	no	spread	oxer with water	straight	yes	4	0	0,00	yes
Stuttgart	Grand Prix of Stuttgart presented by Mercedes-Benz, WALTER solar	19.11.2017	CSI-W5*	4	1.60	410	76	10	20.06.2008	M	BWP	145	4	91	93	45	1436	65	M	1	1	no	vertical	wall	right	no	0	0	77,13	no
Stuttgart	Grand Prix of Stuttgart presented by Mercedes-Benz, WALTER solar	19.11.2017	CSI-W5*	4	1.60	410	76	10	20.06.2008	M	BWP	145	4	91	93	45	1436	65	M	2	2	no	spread	oxer	right	no	0	0	77,13	no
Stuttgart	Grand Prix of Stuttgart presented by Mercedes-Benz, WALTER solar	19.11.2017	CSI-W5*	4	1.60	410	76	10	20.06.2008	M	BWP	145	4	91	93	45	1436	65	M	3	3	no	vertical	vertical	right	no	0	0	77,13	no
Stuttgart	Grand Prix of Stuttgart presented by Mercedes-Benz, WALTER solar	19.11.2017	CSI-W5*	4	1.60	410	76	10	20.06.2008	M	BWP	145	4	91	93	45	1436	65	M	4	4	no	spread	oxer	straight	no	0	0	77,13	no
Stuttgart	Grand Prix of Stuttgart presented by Mercedes-Benz, WALTER solar	19.11.2017	CSI-W5*	4	1.60	410	76	10	20.06.2008	M	BWP	145	4	91	93	45	1436	65	M	5	5	no	spread	oxer	straight	no	0	0	77,13	no
Stuttgart	Grand Prix of Stuttgart presented by Mercedes-Benz, WALTER solar	19.11.2017	CSI-W5*	4	1.60	410	76	10	20.06.2008	M	BWP	145	4	91	93	45	1436	65	M	6	6	no	vertical	vertical	left	yes	4	0	77,13	no
Stuttgart	Grand Prix of Stuttgart presented by Mercedes-Benz, WALTER solar	19.11.2017	CSI-W5*	4	1.60	410	76	10	20.06.2008	M	BWP	145	4	91	93	45	1436	65	M	7	7a	yes	spread	oxer	left	no	0	0	77,13	no
Stuttgart	Grand Prix of Stuttgart presented by Mercedes-Benz, WALTER solar	19.11.2017	CSI-W5*	4	1.60	410	76	10	20.06.2008	M	BWP	145	4	91	93	45	1436	65	M	8	7b	yes	vertical	vertical	left	yes	4	0	77,13	no
Stuttgart	Grand Prix of Stuttgart presented by Mercedes-Benz, WALTER solar	19.11.2017	CSI-W5*	4	1.60	410	76	10	20.06.2008	M	BWP	145	4	91	93	45	1436	65	M	9	7c	yes	spread	oxer	left	yes	4	0	77,13	no
Stuttgart	Grand Prix of Stuttgart presented by Mercedes-Benz, WALTER solar	19.11.2017	CSI-W5*	4	1.60	410	76	10	20.06.2008	M	BWP	145	4	91	93	45	1436	65	M	10	8	no	spread	oxer	left	no	0	0	77,13	no
Stuttgart	Grand Prix of Stuttgart presented by Mercedes-Benz, WALTER solar	19.11.2017	CSI-W5*	4	1.60	410	76	10	20.06.2008	M	BWP	145	4	91	93	45	1436	65	M	11	9	no	vertical	vertical	left	no	0	0	77,13	no
Stuttgart	Grand Prix of Stuttgart presented by Mercedes-Benz, WALTER solar	19.11.2017	CSI-W5*	4	1.60	410	76	10	20.06.2008	M	BWP	145	4	91	93	45	1436	65	M	12	10	no	spread	oxer	right	no	0	0	77,13	no
Stuttgart	Grand Prix of Stuttgart presented by Mercedes-Benz, WALTER solar	19.11.2017	CSI-W5*	4	1.60	410	76	10	20.06.2008	M	BWP	145	4	91	93	45	1436	65	M	13	11	no	vertical	vertical	right	no	0	0	77,13	no
Stuttgart	Grand Prix of Stuttgart presented by Mercedes-Benz, WALTER solar	19.11.2017	CSI-W5*	4	1.60	410	76	10	20.06.2008	M	BWP	145	4	91	93	45	1436	65	M	14	12a	yes	vertical	vertical	right	yes	4	0	77,13	no
Stuttgart	Grand Prix of Stuttgart presented by Mercedes-Benz, WALTER solar	19.11.2017	CSI-W5*	4	1.60	410	76	10	20.06.2008	M	BWP	145	4	91	93	45	1436	65	M	15	12b	yes	spread	oxer with water	right	yes	4	0	77,13	no
Stuttgart	Grand Prix of Stuttgart presented by Mercedes-Benz, WALTER solar	19.11.2017	CSI-W5*	4	1.60	410	76	10	20.06.2008	M	BWP	145	4	91	93	45	1436	65	M	16	13	no	spread	oxer with water	straight	no	0	6	77,13	no
Stuttgart	Grand Prix of Stuttgart presented by Mercedes-Benz, WALTER solar	19.11.2017	CSI-W5*	4	1.60	410	103	10	06.03.2008	S	HOLST	118	5	92	38	25	1918	72	M	1	1	no	vertical	wall	right	no	0	0	69,07	no
Stuttgart	Grand Prix of Stuttgart presented by Mercedes-Benz, WALTER solar	19.11.2017	CSI-W5*	4	1.60	410	103	10	06.03.2008	S	HOLST	118	5	92	38	25	1918	72	M	2	2	no	spread	oxer	right	no	0	0	69,07	no
Stuttgart	Grand Prix of Stuttgart presented by Mercedes-Benz, WALTER solar	19.11.2017	CSI-W5*	4	1.60	410	103	10	06.03.2008	S	HOLST	118	5	92	38	25	1918	72	M	3	3	no	vertical	vertical	right	no	0	0	69,07	no
Stuttgart	Grand Prix of Stuttgart presented by Mercedes-Benz, WALTER solar	19.11.2017	CSI-W5*	4	1.60	410	103	10	06.03.2008	S	HOLST	118	5	92	38	25	1918	72	M	4	4	no	spread	oxer	straight	no	0	0	69,07	no
Stuttgart	Grand Prix of Stuttgart presented by Mercedes-Benz, WALTER solar	19.11.2017	CSI-W5*	4	1.60	410	103	10	06.03.2008	S	HOLST	118	5	92	38	25	1918	72	M	5	5	no	spread	oxer	straight	yes	4	0	69,07	no
Stuttgart	Grand Prix of Stuttgart presented by Mercedes-Benz, WALTER solar	19.11.2017	CSI-W5*	4	1.60	410	103	10	06.03.2008	S	HOLST	118	5	92	38	25	1918	72	M	6	6	no	vertical	vertical	left	no	0	0	69,07	no
Stuttgart	Grand Prix of Stuttgart presented by Mercedes-Benz, WALTER solar	19.11.2017	CSI-W5*	4	1.60	410	103	10	06.03.2008	S	HOLST	118	5	92	38	25	1918	72	M	7	7a	yes	spread	oxer	left	no	0	0	69,07	no
Stuttgart	Grand Prix of Stuttgart presented by Mercedes-Benz, WALTER solar	19.11.2017	CSI-W5*	4	1.60	410	103	10	06.03.2008	S	HOLST	118	5	92	38	25	1918	72	M	8	7b	yes	vertical	vertical	left	yes	4	0	69,07	no
Stuttgart	Grand Prix of Stuttgart presented by Mercedes-Benz, WALTER solar	19.11.2017	CSI-W5*	4	1.60	410	103	10	06.03.2008	S	HOLST	118	5	92	38	25	1918	72	M	9	7c	yes	spread	oxer	left	no	0	0	69,07	no
Stuttgart	Grand Prix of Stuttgart presented by Mercedes-Benz, WALTER solar	19.11.2017	CSI-W5*	4	1.60	410	103	10	06.03.2008	S	HOLST	118	5	92	38	25	1918	72	M	10	8	no	spread	oxer	left	no	0	0	69,07	no
Stuttgart	Grand Prix of Stuttgart presented by Mercedes-Benz, WALTER solar	19.11.2017	CSI-W5*	4	1.60	410	103	10	06.03.2008	S	HOLST	118	5	92	38	25	1918	72	M	11	9	no	vertical	vertical	left	no	0	0	69,07	no
Stuttgart	Grand Prix of Stuttgart presented by Mercedes-Benz, WALTER solar	19.11.2017	CSI-W5*	4	1.60	410	103	10	06.03.2008	S	HOLST	118	5	92	38	25	1918	72	M	12	10	no	spread	oxer	right	no	0	0	69,07	no
Stuttgart	Grand Prix of Stuttgart presented by Mercedes-Benz, WALTER solar	19.11.2017	CSI-W5*	4	1.60	410	103	10	06.03.2008	S	HOLST	118	5	92	38	25	1918	72	M	13	11	no	vertical	vertical	right	no	0	0	69,07	no
Stuttgart	Grand Prix of Stuttgart presented by Mercedes-Benz, WALTER solar	19.11.2017	CSI-W5*	4	1.60	410	103	10	06.03.2008	S	HOLST	118	5	92	38	25	1918	72	M	14	12a	yes	vertical	vertical	right	no	0	0	69,07	no
Stuttgart	Grand Prix of Stuttgart presented by Mercedes-Benz, WALTER solar	19.11.2017	CSI-W5*	4	1.60	410	103	10	06.03.2008	S	HOLST	118	5	92	38	25	1918	72	M	15	12b	yes	spread	oxer with water	right	yes	4	0	69,07	no
Stuttgart	Grand Prix of Stuttgart presented by Mercedes-Benz, WALTER solar	19.11.2017	CSI-W5*	4	1.60	410	103	10	06.03.2008	S	HOLST	118	5	92	38	25	1918	72	M	16	13	no	spread	oxer with water	straight	no	0	0	69,07	no
Stuttgart	Grand Prix of Stuttgart presented by Mercedes-Benz, WALTER solar	19.11.2017	CSI-W5*	4	1.60	410	147	12	04.05.2006	G	OLDBG	239	5	93	106	55	1594	47	M	1	1	no	vertical	wall	right	no	0	0	68,99	no
Stuttgart	Grand Prix of Stuttgart presented by Mercedes-Benz, WALTER solar	19.11.2017	CSI-W5*	4	1.60	410	147	12	04.05.2006	G	OLDBG	239	5	93	106	55	1594	47	M	2	2	no	spread	oxer	right	no	0	0	68,99	no
Stuttgart	Grand Prix of Stuttgart presented by Mercedes-Benz, WALTER solar	19.11.2017	CSI-W5*	4	1.60	410	147	12	04.05.2006	G	OLDBG	239	5	93	106	55	1594	47	M	3	3	no	vertical	vertical	right	no	0	0	68,99	no
Stuttgart	Grand Prix of Stuttgart presented by Mercedes-Benz, WALTER solar	19.11.2017	CSI-W5*	4	1.60	410	147	12	04.05.2006	G	OLDBG	239	5	93	106	55	1594	47	M	4	4	no	spread	oxer	straight	no	0	0	68,99	no
Stuttgart	Grand Prix of Stuttgart presented by Mercedes-Benz, WALTER solar	19.11.2017	CSI-W5*	4	1.60	410	147	12	04.05.2006	G	OLDBG	239	5	93	106	55	1594	47	M	5	5	no	spread	oxer	straight	no	0	0	68,99	no
Stuttgart	Grand Prix of Stuttgart presented by Mercedes-Benz, WALTER solar	19.11.2017	CSI-W5*	4	1.60	410	147	12	04.05.2006	G	OLDBG	239	5	93	106	55	1594	47	M	6	6	no	vertical	vertical	left	no	0	0	68,99	no
Stuttgart	Grand Prix of Stuttgart presented by Mercedes-Benz, WALTER solar	19.11.2017	CSI-W5*	4	1.60	410	147	12	04.05.2006	G	OLDBG	239	5	93	106	55	1594	47	M	7	7a	yes	spread	oxer	left	no	0	0	68,99	no
Stuttgart	Grand Prix of Stuttgart presented by Mercedes-Benz, WALTER solar	19.11.2017	CSI-W5*	4	1.60	410	147	12	04.05.2006	G	OLDBG	239	5	93	106	55	1594	47	M	8	7b	yes	vertical	vertical	left	no	0	0	68,99	no
Stuttgart	Grand Prix of Stuttgart presented by Mercedes-Benz, WALTER solar	19.11.2017	CSI-W5*	4	1.60	410	147	12	04.05.2006	G	OLDBG	239	5	93	106	55	1594	47	M	9	7c	yes	spread	oxer	left	no	0	0	68,99	no
Stuttgart	Grand Prix of Stuttgart presented by Mercedes-Benz, WALTER solar	19.11.2017	CSI-W5*	4	1.60	410	147	12	04.05.2006	G	OLDBG	239	5	93	106	55	1594	47	M	10	8	no	spread	oxer	left	no	0	0	68,99	no
Stuttgart	Grand Prix of Stuttgart presented by Mercedes-Benz, WALTER solar	19.11.2017	CSI-W5*	4	1.60	410	147	12	04.05.2006	G	OLDBG	239	5	93	106	55	1594	47	M	11	9	no	vertical	vertical	left	no	0	0	68,99	no
Stuttgart	Grand Prix of Stuttgart presented by Mercedes-Benz, WALTER solar	19.11.2017	CSI-W5*	4	1.60	410	147	12	04.05.2006	G	OLDBG	239	5	93	106	55	1594	47	M	12	10	no	spread	oxer	right	no	0	0	68,99	no
Stuttgart	Grand Prix of Stuttgart presented by Mercedes-Benz, WALTER solar	19.11.2017	CSI-W5*	4	1.60	410	147	12	04.05.2006	G	OLDBG	239	5	93	106	55	1594	47	M	13	11	no	vertical	vertical	right	no	0	0	68,99	no
Stuttgart	Grand Prix of Stuttgart presented by Mercedes-Benz, WALTER solar	19.11.2017	CSI-W5*	4	1.60	410	147	12	04.05.2006	G	OLDBG	239	5	93	106	55	1594	47	M	14	12a	yes	vertical	vertical	right	no	0	0	68,99	no
Stuttgart	Grand Prix of Stuttgart presented by Mercedes-Benz, WALTER solar	19.11.2017	CSI-W5*	4	1.60	410	147	12	04.05.2006	G	OLDBG	239	5	93	106	55	1594	47	M	15	12b	yes	spread	oxer with water	right	no	0	0	68,99	no
Stuttgart	Grand Prix of Stuttgart presented by Mercedes-Benz, WALTER solar	19.11.2017	CSI-W5*	4	1.60	410	147	12	04.05.2006	G	OLDBG	239	5	93	106	55	1594	47	M	16	13	no	spread	oxer with water	straight	no	0	0	68,99	no
Stuttgart	Grand Prix of Stuttgart presented by Mercedes-Benz, WALTER solar	19.11.2017	CSI-W5*	4	1.60	380	147	12	04.05.2006	G	OLDBG	239	5	93	106	55	1594	47	M	1	1	no	spread	oxer	left	no	0	0	53,97	no
Stuttgart	Grand Prix of Stuttgart presented by Mercedes-Benz, WALTER solar	19.11.2017	CSI-W5*	4	1.60	380	147	12	04.05.2006	G	OLDBG	239	5	93	106	55	1594	47	M	2	2	no	vertical	vertical	left	no	0	0	53,97	no
Stuttgart	Grand Prix of Stuttgart presented by Mercedes-Benz, WALTER solar	19.11.2017	CSI-W5*	4	1.60	380	147	12	04.05.2006	G	OLDBG	239	5	93	106	55	1594	47	M	3	3	no	vertical	vertical	right	no	0	0	53,97	no
Stuttgart	Grand Prix of Stuttgart presented by Mercedes-Benz, WALTER solar	19.11.2017	CSI-W5*	4	1.60	380	147	12	04.05.2006	G	OLDBG	239	5	93	106	55	1594	47	M	4	4	no	spread	oxer with water	right	no	0	0	53,97	no
Stuttgart	Grand Prix of Stuttgart presented by Mercedes-Benz, WALTER solar	19.11.2017	CSI-W5*	4	1.60	380	147	12	04.05.2006	G	OLDBG	239	5	93	106	55	1594	47	M	5	5	no	vertical	vertical	left	no	0	0	53,97	no
Stuttgart	Grand Prix of Stuttgart presented by Mercedes-Benz, WALTER solar	19.11.2017	CSI-W5*	4	1.60	380	147	12	04.05.2006	G	OLDBG	239	5	93	106	55	1594	47	M	6	6	no	vertical	wall	right	no	0	0	53,97	no
Stuttgart	Grand Prix of Stuttgart presented by Mercedes-Benz, WALTER solar	19.11.2017	CSI-W5*	4	1.60	380	147	12	04.05.2006	G	OLDBG	239	5	93	106	55	1594	47	M	7	7	no	spread	oxer	right	no	0	0	53,97	no
Stuttgart	Grand Prix of Stuttgart presented by Mercedes-Benz, WALTER solar	19.11.2017	CSI-W5*	4	1.60	380	147	12	04.05.2006	G	OLDBG	239	5	93	106	55	1594	47	M	8	8a	yes	spread	oxer	left	no	0	0	53,97	no
Stuttgart	Grand Prix of Stuttgart presented by Mercedes-Benz, WALTER solar	19.11.2017	CSI-W5*	4	1.60	380	147	12	04.05.2006	G	OLDBG	239	5	93	106	55	1594	47	M	9	8b	yes	vertical	vertical	left	no	0	0	53,97	no
Stuttgart	Grand Prix of Stuttgart presented by Mercedes-Benz, WALTER solar	19.11.2017	CSI-W5*	4	1.60	380	147	12	04.05.2006	G	OLDBG	239	5	93	106	55	1594	47	M	10	9	no	spread	oxer	left	no	0	0	53,97	no
Stuttgart	Grand Prix of Stuttgart presented by Mercedes-Benz, WALTER solar	19.11.2017	CSI-W5*	4	1.60	410	52	11	11.04.2007	G	OLDBG	175	3	94	160	48	520	8	M	1	1	no	vertical	wall	right	no	0	0	68,59	no
Stuttgart	Grand Prix of Stuttgart presented by Mercedes-Benz, WALTER solar	19.11.2017	CSI-W5*	4	1.60	410	52	11	11.04.2007	G	OLDBG	175	3	94	160	48	520	8	M	2	2	no	spread	oxer	right	no	0	0	68,59	no
Stuttgart	Grand Prix of Stuttgart presented by Mercedes-Benz, WALTER solar	19.11.2017	CSI-W5*	4	1.60	410	52	11	11.04.2007	G	OLDBG	175	3	94	160	48	520	8	M	3	3	no	vertical	vertical	right	no	0	0	68,59	no
Stuttgart	Grand Prix of Stuttgart presented by Mercedes-Benz, WALTER solar	19.11.2017	CSI-W5*	4	1.60	410	52	11	11.04.2007	G	OLDBG	175	3	94	160	48	520	8	M	4	4	no	spread	oxer	straight	no	0	0	68,59	no
Stuttgart	Grand Prix of Stuttgart presented by Mercedes-Benz, WALTER solar	19.11.2017	CSI-W5*	4	1.60	410	52	11	11.04.2007	G	OLDBG	175	3	94	160	48	520	8	M	5	5	no	spread	oxer	straight	no	0	0	68,59	no
Stuttgart	Grand Prix of Stuttgart presented by Mercedes-Benz, WALTER solar	19.11.2017	CSI-W5*	4	1.60	410	52	11	11.04.2007	G	OLDBG	175	3	94	160	48	520	8	M	6	6	no	vertical	vertical	left	no	0	0	68,59	no
Stuttgart	Grand Prix of Stuttgart presented by Mercedes-Benz, WALTER solar	19.11.2017	CSI-W5*	4	1.60	410	52	11	11.04.2007	G	OLDBG	175	3	94	160	48	520	8	M	7	7a	yes	spread	oxer	left	yes	4	0	68,59	no
Stuttgart	Grand Prix of Stuttgart presented by Mercedes-Benz, WALTER solar	19.11.2017	CSI-W5*	4	1.60	410	52	11	11.04.2007	G	OLDBG	175	3	94	160	48	520	8	M	8	7b	yes	vertical	vertical	left	no	0	0	68,59	no
Stuttgart	Grand Prix of Stuttgart presented by Mercedes-Benz, WALTER solar	19.11.2017	CSI-W5*	4	1.60	410	52	11	11.04.2007	G	OLDBG	175	3	94	160	48	520	8	M	9	7c	yes	spread	oxer	left	no	0	0	68,59	no
Stuttgart	Grand Prix of Stuttgart presented by Mercedes-Benz, WALTER solar	19.11.2017	CSI-W5*	4	1.60	410	52	11	11.04.2007	G	OLDBG	175	3	94	160	48	520	8	M	10	8	no	spread	oxer	left	no	0	0	68,59	no
Stuttgart	Grand Prix of Stuttgart presented by Mercedes-Benz, WALTER solar	19.11.2017	CSI-W5*	4	1.60	410	52	11	11.04.2007	G	OLDBG	175	3	94	160	48	520	8	M	11	9	no	vertical	vertical	left	no	0	0	68,59	no
Stuttgart	Grand Prix of Stuttgart presented by Mercedes-Benz, WALTER solar	19.11.2017	CSI-W5*	4	1.60	410	52	11	11.04.2007	G	OLDBG	175	3	94	160	48	520	8	M	12	10	no	spread	oxer	right	no	0	0	68,59	no
Stuttgart	Grand Prix of Stuttgart presented by Mercedes-Benz, WALTER solar	19.11.2017	CSI-W5*	4	1.60	410	52	11	11.04.2007	G	OLDBG	175	3	94	160	48	520	8	M	13	11	no	vertical	vertical	right	no	0	0	68,59	no
Stuttgart	Grand Prix of Stuttgart presented by Mercedes-Benz, WALTER solar	19.11.2017	CSI-W5*	4	1.60	410	52	11	11.04.2007	G	OLDBG	175	3	94	160	48	520	8	M	14	12a	yes	vertical	vertical	right	no	0	0	68,59	no
Stuttgart	Grand Prix of Stuttgart presented by Mercedes-Benz, WALTER solar	19.11.2017	CSI-W5*	4	1.60	410	52	11	11.04.2007	G	OLDBG	175	3	94	160	48	520	8	M	15	12b	yes	spread	oxer with water	right	yes	4	0	68,59	no
Stuttgart	Grand Prix of Stuttgart presented by Mercedes-Benz, WALTER solar	19.11.2017	CSI-W5*	4	1.60	410	52	11	11.04.2007	G	OLDBG	175	3	94	160	48	520	8	M	16	13	no	spread	oxer with water	straight	no	0	0	68,59	no
Stuttgart	Grand Prix of Stuttgart presented by Mercedes-Benz, WALTER solar	19.11.2017	CSI-W5*	4	1.60	410	109	11	14.08.2007	S	BWP	136	1	95	449	29	916	16	M	1	1	no	vertical	wall	right	no	0	0	68,42	no
Stuttgart	Grand Prix of Stuttgart presented by Mercedes-Benz, WALTER solar	19.11.2017	CSI-W5*	4	1.60	410	109	11	14.08.2007	S	BWP	136	1	95	449	29	916	16	M	2	2	no	spread	oxer	right	no	0	0	68,42	no
Stuttgart	Grand Prix of Stuttgart presented by Mercedes-Benz, WALTER solar	19.11.2017	CSI-W5*	4	1.60	410	109	11	14.08.2007	S	BWP	136	1	95	449	29	916	16	M	3	3	no	vertical	vertical	right	no	0	0	68,42	no
Stuttgart	Grand Prix of Stuttgart presented by Mercedes-Benz, WALTER solar	19.11.2017	CSI-W5*	4	1.60	410	109	11	14.08.2007	S	BWP	136	1	95	449	29	916	16	M	4	4	no	spread	oxer	straight	no	0	0	68,42	no
Stuttgart	Grand Prix of Stuttgart presented by Mercedes-Benz, WALTER solar	19.11.2017	CSI-W5*	4	1.60	410	109	11	14.08.2007	S	BWP	136	1	95	449	29	916	16	M	5	5	no	spread	oxer	straight	no	0	0	68,42	no
Stuttgart	Grand Prix of Stuttgart presented by Mercedes-Benz, WALTER solar	19.11.2017	CSI-W5*	4	1.60	410	109	11	14.08.2007	S	BWP	136	1	95	449	29	916	16	M	6	6	no	vertical	vertical	left	no	0	0	68,42	no
Stuttgart	Grand Prix of Stuttgart presented by Mercedes-Benz, WALTER solar	19.11.2017	CSI-W5*	4	1.60	410	109	11	14.08.2007	S	BWP	136	1	95	449	29	916	16	M	7	7a	yes	spread	oxer	left	yes	4	0	68,42	no
Stuttgart	Grand Prix of Stuttgart presented by Mercedes-Benz, WALTER solar	19.11.2017	CSI-W5*	4	1.60	410	109	11	14.08.2007	S	BWP	136	1	95	449	29	916	16	M	8	7b	yes	vertical	vertical	left	no	0	0	68,42	no
Stuttgart	Grand Prix of Stuttgart presented by Mercedes-Benz, WALTER solar	19.11.2017	CSI-W5*	4	1.60	410	109	11	14.08.2007	S	BWP	136	1	95	449	29	916	16	M	9	7c	yes	spread	oxer	left	no	0	0	68,42	no
Stuttgart	Grand Prix of Stuttgart presented by Mercedes-Benz, WALTER solar	19.11.2017	CSI-W5*	4	1.60	410	109	11	14.08.2007	S	BWP	136	1	95	449	29	916	16	M	10	8	no	spread	oxer	left	no	0	0	68,42	no
Stuttgart	Grand Prix of Stuttgart presented by Mercedes-Benz, WALTER solar	19.11.2017	CSI-W5*	4	1.60	410	109	11	14.08.2007	S	BWP	136	1	95	449	29	916	16	M	11	9	no	vertical	vertical	left	no	0	0	68,42	no
Stuttgart	Grand Prix of Stuttgart presented by Mercedes-Benz, WALTER solar	19.11.2017	CSI-W5*	4	1.60	410	109	11	14.08.2007	S	BWP	136	1	95	449	29	916	16	M	12	10	no	spread	oxer	right	yes	4	0	68,42	no
Stuttgart	Grand Prix of Stuttgart presented by Mercedes-Benz, WALTER solar	19.11.2017	CSI-W5*	4	1.60	410	109	11	14.08.2007	S	BWP	136	1	95	449	29	916	16	M	13	11	no	vertical	vertical	right	yes	4	0	68,42	no
Stuttgart	Grand Prix of Stuttgart presented by Mercedes-Benz, WALTER solar	19.11.2017	CSI-W5*	4	1.60	410	109	11	14.08.2007	S	BWP	136	1	95	449	29	916	16	M	14	12a	yes	vertical	vertical	right	no	0	0	68,42	no
Stuttgart	Grand Prix of Stuttgart presented by Mercedes-Benz, WALTER solar	19.11.2017	CSI-W5*	4	1.60	410	109	11	14.08.2007	S	BWP	136	1	95	449	29	916	16	M	15	12b	yes	spread	oxer with water	right	no	0	0	68,42	no
Stuttgart	Grand Prix of Stuttgart presented by Mercedes-Benz, WALTER solar	19.11.2017	CSI-W5*	4	1.60	410	109	11	14.08.2007	S	BWP	136	1	95	449	29	916	16	M	16	13	no	spread	oxer with water	straight	no	0	0	68,42	no
Stuttgart	Grand Prix of Stuttgart presented by Mercedes-Benz, WALTER solar	19.11.2017	CSI-W5*	4	1.60	410	118	12	26.01.2006	G	OS	262	11	96	97	55	1508	53	M	1	1	no	vertical	wall	right	no	0	0	68,23	no
Stuttgart	Grand Prix of Stuttgart presented by Mercedes-Benz, WALTER solar	19.11.2017	CSI-W5*	4	1.60	410	118	12	26.01.2006	G	OS	262	11	96	97	55	1508	53	M	2	2	no	spread	oxer	right	no	0	0	68,23	no
Stuttgart	Grand Prix of Stuttgart presented by Mercedes-Benz, WALTER solar	19.11.2017	CSI-W5*	4	1.60	410	118	12	26.01.2006	G	OS	262	11	96	97	55	1508	53	M	3	3	no	vertical	vertical	right	no	0	0	68,23	no
Stuttgart	Grand Prix of Stuttgart presented by Mercedes-Benz, WALTER solar	19.11.2017	CSI-W5*	4	1.60	410	118	12	26.01.2006	G	OS	262	11	96	97	55	1508	53	M	4	4	no	spread	oxer	straight	no	0	0	68,23	no
Stuttgart	Grand Prix of Stuttgart presented by Mercedes-Benz, WALTER solar	19.11.2017	CSI-W5*	4	1.60	410	118	12	26.01.2006	G	OS	262	11	96	97	55	1508	53	M	5	5	no	spread	oxer	straight	no	0	0	68,23	no
Stuttgart	Grand Prix of Stuttgart presented by Mercedes-Benz, WALTER solar	19.11.2017	CSI-W5*	4	1.60	410	118	12	26.01.2006	G	OS	262	11	96	97	55	1508	53	M	6	6	no	vertical	vertical	left	no	0	0	68,23	no
Stuttgart	Grand Prix of Stuttgart presented by Mercedes-Benz, WALTER solar	19.11.2017	CSI-W5*	4	1.60	410	118	12	26.01.2006	G	OS	262	11	96	97	55	1508	53	M	7	7a	yes	spread	oxer	left	no	0	0	68,23	no
Stuttgart	Grand Prix of Stuttgart presented by Mercedes-Benz, WALTER solar	19.11.2017	CSI-W5*	4	1.60	410	118	12	26.01.2006	G	OS	262	11	96	97	55	1508	53	M	8	7b	yes	vertical	vertical	left	no	0	0	68,23	no
Stuttgart	Grand Prix of Stuttgart presented by Mercedes-Benz, WALTER solar	19.11.2017	CSI-W5*	4	1.60	410	118	12	26.01.2006	G	OS	262	11	96	97	55	1508	53	M	9	7c	yes	spread	oxer	left	no	0	0	68,23	no
Stuttgart	Grand Prix of Stuttgart presented by Mercedes-Benz, WALTER solar	19.11.2017	CSI-W5*	4	1.60	410	118	12	26.01.2006	G	OS	262	11	96	97	55	1508	53	M	10	8	no	spread	oxer	left	no	0	0	68,23	no
Stuttgart	Grand Prix of Stuttgart presented by Mercedes-Benz, WALTER solar	19.11.2017	CSI-W5*	4	1.60	410	118	12	26.01.2006	G	OS	262	11	96	97	55	1508	53	M	11	9	no	vertical	vertical	left	no	0	0	68,23	no
Stuttgart	Grand Prix of Stuttgart presented by Mercedes-Benz, WALTER solar	19.11.2017	CSI-W5*	4	1.60	410	118	12	26.01.2006	G	OS	262	11	96	97	55	1508	53	M	12	10	no	spread	oxer	right	yes	4	0	68,23	no
Stuttgart	Grand Prix of Stuttgart presented by Mercedes-Benz, WALTER solar	19.11.2017	CSI-W5*	4	1.60	410	118	12	26.01.2006	G	OS	262	11	96	97	55	1508	53	M	13	11	no	vertical	vertical	right	no	0	0	68,23	no
Stuttgart	Grand Prix of Stuttgart presented by Mercedes-Benz, WALTER solar	19.11.2017	CSI-W5*	4	1.60	410	118	12	26.01.2006	G	OS	262	11	96	97	55	1508	53	M	14	12a	yes	vertical	vertical	right	yes	4	0	68,23	no
Stuttgart	Grand Prix of Stuttgart presented by Mercedes-Benz, WALTER solar	19.11.2017	CSI-W5*	4	1.60	410	118	12	26.01.2006	G	OS	262	11	96	97	55	1508	53	M	15	12b	yes	spread	oxer with water	right	no	0	0	68,23	no
Stuttgart	Grand Prix of Stuttgart presented by Mercedes-Benz, WALTER solar	19.11.2017	CSI-W5*	4	1.60	410	118	12	26.01.2006	G	OS	262	11	96	97	55	1508	53	M	16	13	no	spread	oxer with water	straight	no	0	0	68,23	no
Stuttgart	Grand Prix of Stuttgart presented by Mercedes-Benz, WALTER solar	19.11.2017	CSI-W5*	4	1.60	410	117	11	22.02.2007	S	OS	91	0	97	201	28	1198	35	M	1	1	no	vertical	wall	right	no	0	0	68,03	no
Stuttgart	Grand Prix of Stuttgart presented by Mercedes-Benz, WALTER solar	19.11.2017	CSI-W5*	4	1.60	410	117	11	22.02.2007	S	OS	91	0	97	201	28	1198	35	M	2	2	no	spread	oxer	right	no	0	0	68,03	no
Stuttgart	Grand Prix of Stuttgart presented by Mercedes-Benz, WALTER solar	19.11.2017	CSI-W5*	4	1.60	410	117	11	22.02.2007	S	OS	91	0	97	201	28	1198	35	M	3	3	no	vertical	vertical	right	no	0	0	68,03	no
Stuttgart	Grand Prix of Stuttgart presented by Mercedes-Benz, WALTER solar	19.11.2017	CSI-W5*	4	1.60	410	117	11	22.02.2007	S	OS	91	0	97	201	28	1198	35	M	4	4	no	spread	oxer	straight	no	0	0	68,03	no
Stuttgart	Grand Prix of Stuttgart presented by Mercedes-Benz, WALTER solar	19.11.2017	CSI-W5*	4	1.60	410	117	11	22.02.2007	S	OS	91	0	97	201	28	1198	35	M	5	5	no	spread	oxer	straight	no	0	0	68,03	no
Stuttgart	Grand Prix of Stuttgart presented by Mercedes-Benz, WALTER solar	19.11.2017	CSI-W5*	4	1.60	410	117	11	22.02.2007	S	OS	91	0	97	201	28	1198	35	M	6	6	no	vertical	vertical	left	no	0	0	68,03	no
Stuttgart	Grand Prix of Stuttgart presented by Mercedes-Benz, WALTER solar	19.11.2017	CSI-W5*	4	1.60	410	117	11	22.02.2007	S	OS	91	0	97	201	28	1198	35	M	7	7a	yes	spread	oxer	left	no	0	0	68,03	no
Stuttgart	Grand Prix of Stuttgart presented by Mercedes-Benz, WALTER solar	19.11.2017	CSI-W5*	4	1.60	410	117	11	22.02.2007	S	OS	91	0	97	201	28	1198	35	M	8	7b	yes	vertical	vertical	left	no	0	0	68,03	no
Stuttgart	Grand Prix of Stuttgart presented by Mercedes-Benz, WALTER solar	19.11.2017	CSI-W5*	4	1.60	410	117	11	22.02.2007	S	OS	91	0	97	201	28	1198	35	M	9	7c	yes	spread	oxer	left	no	0	0	68,03	no
Stuttgart	Grand Prix of Stuttgart presented by Mercedes-Benz, WALTER solar	19.11.2017	CSI-W5*	4	1.60	410	117	11	22.02.2007	S	OS	91	0	97	201	28	1198	35	M	10	8	no	spread	oxer	left	no	0	0	68,03	no
Stuttgart	Grand Prix of Stuttgart presented by Mercedes-Benz, WALTER solar	19.11.2017	CSI-W5*	4	1.60	410	117	11	22.02.2007	S	OS	91	0	97	201	28	1198	35	M	11	9	no	vertical	vertical	left	no	0	0	68,03	no
Stuttgart	Grand Prix of Stuttgart presented by Mercedes-Benz, WALTER solar	19.11.2017	CSI-W5*	4	1.60	410	117	11	22.02.2007	S	OS	91	0	97	201	28	1198	35	M	12	10	no	spread	oxer	right	no	0	0	68,03	no
Stuttgart	Grand Prix of Stuttgart presented by Mercedes-Benz, WALTER solar	19.11.2017	CSI-W5*	4	1.60	410	117	11	22.02.2007	S	OS	91	0	97	201	28	1198	35	M	13	11	no	vertical	vertical	right	no	0	0	68,03	no
Stuttgart	Grand Prix of Stuttgart presented by Mercedes-Benz, WALTER solar	19.11.2017	CSI-W5*	4	1.60	410	117	11	22.02.2007	S	OS	91	0	97	201	28	1198	35	M	14	12a	yes	vertical	vertical	right	no	0	0	68,03	no
Stuttgart	Grand Prix of Stuttgart presented by Mercedes-Benz, WALTER solar	19.11.2017	CSI-W5*	4	1.60	410	117	11	22.02.2007	S	OS	91	0	97	201	28	1198	35	M	15	12b	yes	spread	oxer with water	right	no	0	0	68,03	no
Stuttgart	Grand Prix of Stuttgart presented by Mercedes-Benz, WALTER solar	19.11.2017	CSI-W5*	4	1.60	410	117	11	22.02.2007	S	OS	91	0	97	201	28	1198	35	M	16	13	no	spread	oxer with water	straight	no	0	0	68,03	no
Stuttgart	Grand Prix of Stuttgart presented by Mercedes-Benz, WALTER solar	19.11.2017	CSI-W5*	4	1.60	380	117	11	22.02.2007	S	OS	91	0	97	201	28	1198	35	M	1	1	no	spread	oxer	left	no	0	0	60,16	no
Stuttgart	Grand Prix of Stuttgart presented by Mercedes-Benz, WALTER solar	19.11.2017	CSI-W5*	4	1.60	380	117	11	22.02.2007	S	OS	91	0	97	201	28	1198	35	M	2	2	no	vertical	vertical	left	no	0	0	60,16	no
Stuttgart	Grand Prix of Stuttgart presented by Mercedes-Benz, WALTER solar	19.11.2017	CSI-W5*	4	1.60	380	117	11	22.02.2007	S	OS	91	0	97	201	28	1198	35	M	3	3	no	vertical	vertical	right	no	0	0	60,16	no
Stuttgart	Grand Prix of Stuttgart presented by Mercedes-Benz, WALTER solar	19.11.2017	CSI-W5*	4	1.60	380	117	11	22.02.2007	S	OS	91	0	97	201	28	1198	35	M	4	4	no	spread	oxer with water	right	no	0	0	60,16	no
Stuttgart	Grand Prix of Stuttgart presented by Mercedes-Benz, WALTER solar	19.11.2017	CSI-W5*	4	1.60	380	117	11	22.02.2007	S	OS	91	0	97	201	28	1198	35	M	5	5	no	vertical	vertical	left	no	0	0	60,16	no
Stuttgart	Grand Prix of Stuttgart presented by Mercedes-Benz, WALTER solar	19.11.2017	CSI-W5*	4	1.60	380	117	11	22.02.2007	S	OS	91	0	97	201	28	1198	35	M	6	6	no	vertical	wall	right	no	0	0	60,16	no
Stuttgart	Grand Prix of Stuttgart presented by Mercedes-Benz, WALTER solar	19.11.2017	CSI-W5*	4	1.60	380	117	11	22.02.2007	S	OS	91	0	97	201	28	1198	35	M	7	7	no	spread	oxer	right	no	0	0	60,16	no
Stuttgart	Grand Prix of Stuttgart presented by Mercedes-Benz, WALTER solar	19.11.2017	CSI-W5*	4	1.60	380	117	11	22.02.2007	S	OS	91	0	97	201	28	1198	35	M	8	8a	yes	spread	oxer	left	no	0	0	60,16	no
Stuttgart	Grand Prix of Stuttgart presented by Mercedes-Benz, WALTER solar	19.11.2017	CSI-W5*	4	1.60	380	117	11	22.02.2007	S	OS	91	0	97	201	28	1198	35	M	9	8b	yes	vertical	vertical	left	no	0	0	60,16	no
Stuttgart	Grand Prix of Stuttgart presented by Mercedes-Benz, WALTER solar	19.11.2017	CSI-W5*	4	1.60	380	117	11	22.02.2007	S	OS	91	0	97	201	28	1198	35	M	10	9	no	spread	oxer	left	no	0	0	60,16	no
Madrid	MUTUA MADRILEÑA TROPHY GRAND PRIX CSI*5 W round A	25.11.2017	CSI-W5*	3	1.60	430	63	11	22.03.2007	G	KWPN	175	2	3	97	43	1901	45	M	1	1	no	spread	oxer	left	no	0	0	71,41	no
Madrid	MUTUA MADRILEÑA TROPHY GRAND PRIX CSI*5 W round A	25.11.2017	CSI-W5*	3	1.60	430	63	11	22.03.2007	G	KWPN	175	2	3	97	43	1901	45	M	2	2	no	vertical	vertical	straight	no	0	0	71,41	no
Madrid	MUTUA MADRILEÑA TROPHY GRAND PRIX CSI*5 W round A	25.11.2017	CSI-W5*	3	1.60	430	63	11	22.03.2007	G	KWPN	175	2	3	97	43	1901	45	M	3	3	no	spread	oxer	right	no	0	0	71,41	no
Madrid	MUTUA MADRILEÑA TROPHY GRAND PRIX CSI*5 W round A	25.11.2017	CSI-W5*	3	1.60	430	63	11	22.03.2007	G	KWPN	175	2	3	97	43	1901	45	M	4	4	no	vertical	vertical	straight	no	0	0	71,41	no
Madrid	MUTUA MADRILEÑA TROPHY GRAND PRIX CSI*5 W round A	25.11.2017	CSI-W5*	3	1.60	430	63	11	22.03.2007	G	KWPN	175	2	3	97	43	1901	45	M	5	5	no	spread	oxer	left	no	0	0	71,41	no
Madrid	MUTUA MADRILEÑA TROPHY GRAND PRIX CSI*5 W round A	25.11.2017	CSI-W5*	3	1.60	430	63	11	22.03.2007	G	KWPN	175	2	3	97	43	1901	45	M	6	6	no	vertical	vertical	left	no	0	0	71,41	no
Madrid	MUTUA MADRILEÑA TROPHY GRAND PRIX CSI*5 W round A	25.11.2017	CSI-W5*	3	1.60	430	63	11	22.03.2007	G	KWPN	175	2	3	97	43	1901	45	M	7	7a	yes	spread	oxer	right	no	0	0	71,41	no
Madrid	MUTUA MADRILEÑA TROPHY GRAND PRIX CSI*5 W round A	25.11.2017	CSI-W5*	3	1.60	430	63	11	22.03.2007	G	KWPN	175	2	3	97	43	1901	45	M	8	7b	yes	vertical	vertical	right	no	0	0	71,41	no
Madrid	MUTUA MADRILEÑA TROPHY GRAND PRIX CSI*5 W round A	25.11.2017	CSI-W5*	3	1.60	430	63	11	22.03.2007	G	KWPN	175	2	3	97	43	1901	45	M	9	8	no	spread	oxer	right	no	0	0	71,41	no
Madrid	MUTUA MADRILEÑA TROPHY GRAND PRIX CSI*5 W round A	25.11.2017	CSI-W5*	3	1.60	430	63	11	22.03.2007	G	KWPN	175	2	3	97	43	1901	45	M	10	9	no	vertical	vertical	right	no	0	0	71,41	no
Madrid	MUTUA MADRILEÑA TROPHY GRAND PRIX CSI*5 W round A	25.11.2017	CSI-W5*	3	1.60	430	63	11	22.03.2007	G	KWPN	175	2	3	97	43	1901	45	M	11	10	no	spread	oxer	straight	no	0	0	71,41	no
Madrid	MUTUA MADRILEÑA TROPHY GRAND PRIX CSI*5 W round A	25.11.2017	CSI-W5*	3	1.60	430	63	11	22.03.2007	G	KWPN	175	2	3	97	43	1901	45	M	12	11	no	vertical	vertical with water	left	no	0	0	71,41	no
Madrid	MUTUA MADRILEÑA TROPHY GRAND PRIX CSI*5 W round A	25.11.2017	CSI-W5*	3	1.60	430	63	11	22.03.2007	G	KWPN	175	2	3	97	43	1901	45	M	13	12a	yes	vertical	vertical	left	yes	4	0	71,41	no
Madrid	MUTUA MADRILEÑA TROPHY GRAND PRIX CSI*5 W round A	25.11.2017	CSI-W5*	3	1.60	430	63	11	22.03.2007	G	KWPN	175	2	3	97	43	1901	45	M	14	12b	yes	spread	oxer with water	left	no	0	0	71,41	no
Madrid	MUTUA MADRILEÑA TROPHY GRAND PRIX CSI*5 W round A	25.11.2017	CSI-W5*	3	1.60	430	63	11	22.03.2007	G	KWPN	175	2	3	97	43	1901	45	M	15	12c	yes	vertical	vertical	left	no	0	0	71,41	no
Madrid	MUTUA MADRILEÑA TROPHY GRAND PRIX CSI*5 W round A	25.11.2017	CSI-W5*	3	1.60	430	63	11	22.03.2007	G	KWPN	175	2	3	97	43	1901	45	M	16	13	no	spread	oxer	right	no	0	0	71,41	no
Madrid	MUTUA MADRILEÑA TROPHY GRAND PRIX CSI*5 W round A	25.11.2017	CSI-W5*	3	1.60	430	82	14	17.04.2004	G	BWP	214	17	5	15	32	2092	63	M	1	1	no	spread	oxer	left	no	0	0	70,47	no
Madrid	MUTUA MADRILEÑA TROPHY GRAND PRIX CSI*5 W round A	25.11.2017	CSI-W5*	3	1.60	430	82	14	17.04.2004	G	BWP	214	17	5	15	32	2092	63	M	2	2	no	vertical	vertical	straight	no	0	0	70,47	no
Madrid	MUTUA MADRILEÑA TROPHY GRAND PRIX CSI*5 W round A	25.11.2017	CSI-W5*	3	1.60	430	82	14	17.04.2004	G	BWP	214	17	5	15	32	2092	63	M	3	3	no	spread	oxer	right	no	0	0	70,47	no
Madrid	MUTUA MADRILEÑA TROPHY GRAND PRIX CSI*5 W round A	25.11.2017	CSI-W5*	3	1.60	430	82	14	17.04.2004	G	BWP	214	17	5	15	32	2092	63	M	4	4	no	vertical	vertical	straight	no	0	0	70,47	no
Madrid	MUTUA MADRILEÑA TROPHY GRAND PRIX CSI*5 W round A	25.11.2017	CSI-W5*	3	1.60	430	82	14	17.04.2004	G	BWP	214	17	5	15	32	2092	63	M	5	5	no	spread	oxer	left	no	0	0	70,47	no
Madrid	MUTUA MADRILEÑA TROPHY GRAND PRIX CSI*5 W round A	25.11.2017	CSI-W5*	3	1.60	430	82	14	17.04.2004	G	BWP	214	17	5	15	32	2092	63	M	6	6	no	vertical	vertical	left	no	0	0	70,47	no
Madrid	MUTUA MADRILEÑA TROPHY GRAND PRIX CSI*5 W round A	25.11.2017	CSI-W5*	3	1.60	430	82	14	17.04.2004	G	BWP	214	17	5	15	32	2092	63	M	7	7a	yes	spread	oxer	right	no	0	0	70,47	no
Madrid	MUTUA MADRILEÑA TROPHY GRAND PRIX CSI*5 W round A	25.11.2017	CSI-W5*	3	1.60	430	82	14	17.04.2004	G	BWP	214	17	5	15	32	2092	63	M	8	7b	yes	vertical	vertical	right	no	0	0	70,47	no
Madrid	MUTUA MADRILEÑA TROPHY GRAND PRIX CSI*5 W round A	25.11.2017	CSI-W5*	3	1.60	430	82	14	17.04.2004	G	BWP	214	17	5	15	32	2092	63	M	9	8	no	spread	oxer	right	no	0	0	70,47	no
Madrid	MUTUA MADRILEÑA TROPHY GRAND PRIX CSI*5 W round A	25.11.2017	CSI-W5*	3	1.60	430	82	14	17.04.2004	G	BWP	214	17	5	15	32	2092	63	M	10	9	no	vertical	vertical	right	no	0	0	70,47	no
Madrid	MUTUA MADRILEÑA TROPHY GRAND PRIX CSI*5 W round A	25.11.2017	CSI-W5*	3	1.60	430	82	14	17.04.2004	G	BWP	214	17	5	15	32	2092	63	M	11	10	no	spread	oxer	straight	no	0	0	70,47	no
Madrid	MUTUA MADRILEÑA TROPHY GRAND PRIX CSI*5 W round A	25.11.2017	CSI-W5*	3	1.60	430	82	14	17.04.2004	G	BWP	214	17	5	15	32	2092	63	M	12	11	no	vertical	vertical with water	left	no	0	0	70,47	no
Madrid	MUTUA MADRILEÑA TROPHY GRAND PRIX CSI*5 W round A	25.11.2017	CSI-W5*	3	1.60	430	82	14	17.04.2004	G	BWP	214	17	5	15	32	2092	63	M	13	12a	yes	vertical	vertical	left	no	0	0	70,47	no
Madrid	MUTUA MADRILEÑA TROPHY GRAND PRIX CSI*5 W round A	25.11.2017	CSI-W5*	3	1.60	430	82	14	17.04.2004	G	BWP	214	17	5	15	32	2092	63	M	14	12b	yes	spread	oxer with water	left	no	0	0	70,47	no
Madrid	MUTUA MADRILEÑA TROPHY GRAND PRIX CSI*5 W round A	25.11.2017	CSI-W5*	3	1.60	430	82	14	17.04.2004	G	BWP	214	17	5	15	32	2092	63	M	15	12c	yes	vertical	vertical	left	no	0	0	70,47	no
Madrid	MUTUA MADRILEÑA TROPHY GRAND PRIX CSI*5 W round A	25.11.2017	CSI-W5*	3	1.60	430	82	14	17.04.2004	G	BWP	214	17	5	15	32	2092	63	M	16	13	no	spread	oxer	right	no	0	0	70,47	no

Madrid	MUTUA MADRILEÑA TROPHY GRAND PRIX CSI*5 W round B	25.11.2017	CSI-W5*	3	1.60	300	82	14	17.04.2004	G	BWP	214	17	5	15	32	2092	63	M	1	1	no	spread	oxer	right	no	0	0	48,12	no
Madrid	MUTUA MADRILEÑA TROPHY GRAND PRIX CSI*5 W round B	25.11.2017	CSI-W5*	3	1.60	300	82	14	17.04.2004	G	BWP	214	17	5	15	32	2092	63	M	2	2	no	spread	oxer	right	yes	4	0	48,12	no
Madrid	MUTUA MADRILEÑA TROPHY GRAND PRIX CSI*5 W round B	25.11.2017	CSI-W5*	3	1.60	300	82	14	17.04.2004	G	BWP	214	17	5	15	32	2092	63	M	3	3	no	vertical	vertical with water	left	no	0	0	48,12	no
Madrid	MUTUA MADRILEÑA TROPHY GRAND PRIX CSI*5 W round B	25.11.2017	CSI-W5*	3	1.60	300	82	14	17.04.2004	G	BWP	214	17	5	15	32	2092	63	M	4	4a	yes	vertical	vertical	left	no	0	0	48,12	no
Madrid	MUTUA MADRILEÑA TROPHY GRAND PRIX CSI*5 W round B	25.11.2017	CSI-W5*	3	1.60	300	82	14	17.04.2004	G	BWP	214	17	5	15	32	2092	63	M	5	4b	yes	spread	oxer with water	left	no	0	0	48,12	no
Madrid	MUTUA MADRILEÑA TROPHY GRAND PRIX CSI*5 W round B	25.11.2017	CSI-W5*	3	1.60	300	82	14	17.04.2004	G	BWP	214	17	5	15	32	2092	63	M	6	5	no	spread	oxer	right	no	0	0	48,12	no
Madrid	MUTUA MADRILEÑA TROPHY GRAND PRIX CSI*5 W round B	25.11.2017	CSI-W5*	3	1.60	300	82	14	17.04.2004	G	BWP	214	17	5	15	32	2092	63	M	7	6	no	vertical	vertical	right	no	0	0	48,12	no
Madrid	MUTUA MADRILEÑA TROPHY GRAND PRIX CSI*5 W round B	25.11.2017	CSI-W5*	3	1.60	300	82	14	17.04.2004	G	BWP	214	17	5	15	32	2092	63	M	8	7	no	vertical	vertical	left	no	0	0	48,12	no
Madrid	MUTUA MADRILEÑA TROPHY GRAND PRIX CSI*5 W round B	25.11.2017	CSI-W5*	3	1.60	300	82	14	17.04.2004	G	BWP	214	17	5	15	32	2092	63	M	9	8	no	spread	oxer	left	no	0	0	48,12	no
Madrid	MUTUA MADRILEÑA TROPHY GRAND PRIX CSI*5 W round A	25.11.2017	CSI-W5*	3	1.60	430	88	13	19.04.2005	S	BWP	131	0	6	421	45	483	7	F	1	1	no	spread	oxer	left	no	0	0	0,00	yes
Madrid	MUTUA MADRILEÑA TROPHY GRAND PRIX CSI*5 W round A	25.11.2017	CSI-W5*	3	1.60	430	88	13	19.04.2005	S	BWP	131	0	6	421	45	483	7	F	2	2	no	vertical	vertical	straight	no	0	0	0,00	yes
Madrid	MUTUA MADRILEÑA TROPHY GRAND PRIX CSI*5 W round A	25.11.2017	CSI-W5*	3	1.60	430	88	13	19.04.2005	S	BWP	131	0	6	421	45	483	7	F	3	3	no	spread	oxer	right	no	0	0	0,00	yes
Madrid	MUTUA MADRILEÑA TROPHY GRAND PRIX CSI*5 W round A	25.11.2017	CSI-W5*	3	1.60	430	88	13	19.04.2005	S	BWP	131	0	6	421	45	483	7	F	4	4	no	vertical	vertical	straight	no	0	0	0,00	yes
Madrid	MUTUA MADRILEÑA TROPHY GRAND PRIX CSI*5 W round A	25.11.2017	CSI-W5*	3	1.60	430	88	13	19.04.2005	S	BWP	131	0	6	421	45	483	7	F	5	5	no	spread	oxer	left	no	0	0	0,00	yes
Madrid	MUTUA MADRILEÑA TROPHY GRAND PRIX CSI*5 W round A	25.11.2017	CSI-W5*	3	1.60	430	88	13	19.04.2005	S	BWP	131	0	6	421	45	483	7	F	6	6	no	vertical	vertical	left	no	0	0	0,00	yes
Madrid	MUTUA MADRILEÑA TROPHY GRAND PRIX CSI*5 W round A	25.11.2017	CSI-W5*	3	1.60	430	88	13	19.04.2005	S	BWP	131	0	6	421	45	483	7	F	7	7a	yes	spread	oxer	right	no	0	0	0,00	yes
Madrid	MUTUA MADRILEÑA TROPHY GRAND PRIX CSI*5 W round A	25.11.2017	CSI-W5*	3	1.60	430	88	13	19.04.2005	S	BWP	131	0	6	421	45	483	7	F	8	7b	yes	vertical	vertical	right	no	0	0	0,00	yes
Madrid	MUTUA MADRILEÑA TROPHY GRAND PRIX CSI*5 W round A	25.11.2017	CSI-W5*	3	1.60	430	88	13	19.04.2005	S	BWP	131	0	6	421	45	483	7	F	9	8	no	spread	oxer	right	yes	4	0	0,00	yes
Madrid	MUTUA MADRILEÑA TROPHY GRAND PRIX CSI*5 W round A	25.11.2017	CSI-W5*	3	1.60	430	88	13	19.04.2005	S	BWP	131	0	6	421	45	483	7	F	10	9	no	vertical	vertical	right	no	0	0	0,00	yes
Madrid	MUTUA MADRILEÑA TROPHY GRAND PRIX CSI*5 W round A	25.11.2017	CSI-W5*	3	1.60	430	88	13	19.04.2005	S	BWP	131	0	6	421	45	483	7	F	11	10	no	spread	oxer	straight	yes	4	0	0,00	yes
Madrid	MUTUA MADRILEÑA TROPHY GRAND PRIX CSI*5 W round A	25.11.2017	CSI-W5*	3	1.60	430	88	13	19.04.2005	S	BWP	131	0	6	421	45	483	7	F	12	11	no	vertical	vertical with water	left	yes	4	0	0,00	yes
Madrid	MUTUA MADRILEÑA TROPHY GRAND PRIX CSI*5 W round A	25.11.2017	CSI-W5*	3	1.60	430	88	13	19.04.2005	S	BWP	131	0	6	421	45	483	7	F	13	12a	yes	vertical	vertical	left	no	0	0	0,00	yes
Madrid	MUTUA MADRILEÑA TROPHY GRAND PRIX CSI*5 W round A	25.11.2017	CSI-W5*	3	1.60	430	88	13	19.04.2005	S	BWP	131	0	6	421	45	483	7	F	14	12b	yes	spread	oxer with water	left	yes	4	0	0,00	yes
Madrid	MUTUA MADRILEÑA TROPHY GRAND PRIX CSI*5 W round A	25.11.2017	CSI-W5*	3	1.60	430	88	13	19.04.2005	S	BWP	131	0	6	421	45	483	7	F	15	12c	yes	vertical	vertical	left	no	0	0	0,00	yes
Madrid	MUTUA MADRILEÑA TROPHY GRAND PRIX CSI*5 W round A	25.11.2017	CSI-W5*	3	1.60	430	88	13	19.04.2005	S	BWP	131	0	6	421	45	483	7	F	16	13	no	spread	oxer	right	no	0	0	0,00	yes
Madrid	MUTUA MADRILEÑA TROPHY GRAND PRIX CSI*5 W round A	25.11.2017	CSI-W5*	3	1.60	430	20	11	16.04.2007	G	OS	121	0	7	48	58	1857	49	M	1	1	no	spread	oxer	left	no	0	0	73	no
Madrid	MUTUA MADRILEÑA TROPHY GRAND PRIX CSI*5 W round A	25.11.2017	CSI-W5*	3	1.60	430	20	11	16.04.2007	G	OS	121	0	7	48	58	1857	49	M	2	2	no	vertical	vertical	straight	no	0	0	73	no
Madrid	MUTUA MADRILEÑA TROPHY GRAND PRIX CSI*5 W round A	25.11.2017	CSI-W5*	3	1.60	430	20	11	16.04.2007	G	OS	121	0	7	48	58	1857	49	M	3	3	no	spread	oxer	right	no	0	0	73	no
Madrid	MUTUA MADRILEÑA TROPHY GRAND PRIX CSI*5 W round A	25.11.2017	CSI-W5*	3	1.60	430	20	11	16.04.2007	G	OS	121	0	7	48	58	1857	49	M	4	4	no	vertical	vertical	straight	no	0	0	73	no
Madrid	MUTUA MADRILEÑA TROPHY GRAND PRIX CSI*5 W round A	25.11.2017	CSI-W5*	3	1.60	430	20	11	16.04.2007	G	OS	121	0	7	48	58	1857	49	M	5	5	no	spread	oxer	left	no	0	0	73	no
Madrid	MUTUA MADRILEÑA TROPHY GRAND PRIX CSI*5 W round A	25.11.2017	CSI-W5*	3	1.60	430	20	11	16.04.2007	G	OS	121	0	7	48	58	1857	49	M	6	6	no	vertical	vertical	left	no	0	0	73	no
Madrid	MUTUA MADRILEÑA TROPHY GRAND PRIX CSI*5 W round A	25.11.2017	CSI-W5*	3	1.60	430	20	11	16.04.2007	G	OS	121	0	7	48	58	1857	49	M	7	7a	yes	spread	oxer	right	no	0	0	73	no
Madrid	MUTUA MADRILEÑA TROPHY GRAND PRIX CSI*5 W round A	25.11.2017	CSI-W5*	3	1.60	430	20	11	16.04.2007	G	OS	121	0	7	48	58	1857	49	M	8	7b	yes	vertical	vertical	right	no	0	0	73	no
Madrid	MUTUA MADRILEÑA TROPHY GRAND PRIX CSI*5 W round A	25.11.2017	CSI-W5*	3	1.60	430	20	11	16.04.2007	G	OS	121	0	7	48	58	1857	49	M	9	8	no	spread	oxer	right	no	0	0	73	no
Madrid	MUTUA MADRILEÑA TROPHY GRAND PRIX CSI*5 W round A	25.11.2017	CSI-W5*	3	1.60	430	20	11	16.04.2007	G	OS	121	0	7	48	58	1857	49	M	10	9	no	vertical	vertical	right	no	0	0	73	no
Madrid	MUTUA MADRILEÑA TROPHY GRAND PRIX CSI*5 W round A	25.11.2017	CSI-W5*	3	1.60	430	20	11	16.04.2007	G	OS	121	0	7	48	58	1857	49	M	11	10	no	spread	oxer	straight	no	0	0	73	no
Madrid	MUTUA MADRILEÑA TROPHY GRAND PRIX CSI*5 W round A	25.11.2017	CSI-W5*	3	1.60	430	20	11	16.04.2007	G	OS	121	0	7	48	58	1857	49	M	12	11	no	vertical	vertical with water	left	no	0	0	73	no
Madrid	MUTUA MADRILEÑA TROPHY GRAND PRIX CSI*5 W round A	25.11.2017	CSI-W5*	3	1.60	430	20	11	16.04.2007	G	OS	121	0	7	48	58	1857	49	M	13	12a	yes	vertical	vertical	left	no	0	0	73	no
Madrid	MUTUA MADRILEÑA TROPHY GRAND PRIX CSI*5 W round A	25.11.2017	CSI-W5*	3	1.60	430	20	11	16.04.2007	G	OS	121	0	7	48	58	1857	49	M	14	12b	yes	spread	oxer with water	left	no	0	0	73	no
Madrid	MUTUA MADRILEÑA TROPHY GRAND PRIX CSI*5 W round A	25.11.2017	CSI-W5*	3	1.60	430	20	11	16.04.2007	G	OS	121	0	7	48	58	1857	49	M	15	12c	yes	vertical	vertical	left	no	0	0	73	no
Madrid	MUTUA MADRILEÑA TROPHY GRAND PRIX CSI*5 W round A	25.11.2017	CSI-W5*	3	1.60	430	20	11	16.04.2007	G	OS	121	0	7	48	58	1857	49	M	16	13	no	spread	oxer	right	no	0	0	73	no
Madrid	MUTUA MADRILEÑA TROPHY GRAND PRIX CSI*5 W round B	25.11.2017	CSI-W5*	3	1.60	300	20	11	16.04.2007	G	OS	121	0	7	48	58	1857	49	M	1	1	no	spread	oxer	right	no	0	0	43,82	no
Madrid	MUTUA MADRILEÑA TROPHY GRAND PRIX CSI*5 W round B	25.11.2017	CSI-W5*	3	1.60	300	20	11	16.04.2007	G	OS	121	0	7	48	58	1857	49	M	2	2	no	spread	oxer	right	no	0	0	43,82	no
Madrid	MUTUA MADRILEÑA TROPHY GRAND PRIX CSI*5 W round B	25.11.2017	CSI-W5*	3	1.60	300	20	11	16.04.2007	G	OS	121	0	7	48	58	1857	49	M	3	3	no	vertical	vertical with water	left	no	0	0	43,82	no
Madrid	MUTUA MADRILEÑA TROPHY GRAND PRIX CSI*5 W round B	25.11.2017	CSI-W5*	3	1.60	300	20	11	16.04.2007	G	OS	121	0	7	48	58	1857	49	M	4	4a	yes	vertical	vertical	left	no	0	0	43,82	no
Madrid	MUTUA MADRILEÑA TROPHY GRAND PRIX CSI*5 W round B	25.11.2017	CSI-W5*	3	1.60	300	20	11	16.04.2007	G	OS	121	0	7	48	58	1857	49	M	5	4b	yes	spread	oxer with water	left	no	0	0	43,82	no
Madrid	MUTUA MADRILEÑA TROPHY GRAND PRIX CSI*5 W round B	25.11.2017	CSI-W5*	3	1.60	300	20	11	16.04.2007	G	OS	121	0	7	48	58	1857	49	M	6	5	no	spread	oxer	right	no	0	0	43,82	no
Madrid	MUTUA MADRILEÑA TROPHY GRAND PRIX CSI*5 W round B	25.11.2017	CSI-W5*	3	1.60	300	20	11	16.04.2007	G	OS	121	0	7	48	58	1857	49	M	7	6	no	vertical	vertical	right	no	0	0	43,82	no
Madrid	MUTUA MADRILEÑA TROPHY GRAND PRIX CSI*5 W round B	25.11.2017	CSI-W5*	3	1.60	300	20	11	16.04.2007	G	OS	121	0	7	48	58	1857	49	M	8	7	no	vertical	vertical	left	no	0	0	43,82	no
Madrid	MUTUA MADRILEÑA TROPHY GRAND PRIX CSI*5 W round B	25.11.2017	CSI-W5*	3	1.60	300	20	11	16.04.2007	G	OS	121	0	7	48	58	1857	49	M	9	8	no	spread	oxer	left	no	0	0	43,82	no
Madrid	MUTUA MADRILEÑA TROPHY GRAND PRIX CSI*5 W round A	25.11.2017	CSI-W5*	3	1.60	430	195	11	25.04.2007	G	SF	164	16	9	103	48	1417	59	M	1	1	no	spread	oxer	left	no	0	0	68,83	no
Madrid	MUTUA MADRILEÑA TROPHY GRAND PRIX CSI*5 W round A	25.11.2017	CSI-W5*	3	1.60	430	195	11	25.04.2007	G	SF	164	16	9	103	48	1417	59	M	2	2	no	vertical	vertical	straight	no	0	0	68,83	no
Madrid	MUTUA MADRILEÑA TROPHY GRAND PRIX CSI*5 W round A	25.11.2017	CSI-W5*	3	1.60	430	195	11	25.04.2007	G	SF	164	16	9	103	48	1417	59	M	3	3	no	spread	oxer	right	no	0	0	68,83	no
Madrid	MUTUA MADRILEÑA TROPHY GRAND PRIX CSI*5 W round A	25.11.2017	CSI-W5*	3	1.60	430	195	11	25.04.2007	G	SF	164	16	9	103	48	1417	59	M	4	4	no	vertical	vertical	straight	no	0	0	68,83	no
Madrid	MUTUA MADRILEÑA TROPHY GRAND PRIX CSI*5 W round A	25.11.2017	CSI-W5*	3	1.60	430	195	11	25.04.2007	G	SF	164	16	9	103	48	1417	59	M	5	5	no	spread	oxer	left	no	0	0	68,83	no
Madrid	MUTUA MADRILEÑA TROPHY GRAND PRIX CSI*5 W round A	25.11.2017	CSI-W5*	3	1.60	430	195	11	25.04.2007	G	SF	164	16	9	103	48	1417	59	M	6	6	no	vertical	vertical	left	no	0	0	68,83	no
Madrid	MUTUA MADRILEÑA TROPHY GRAND PRIX CSI*5 W round A	25.11.2017	CSI-W5*	3	1.60	430	195	11	25.04.2007	G	SF	164	16	9	103	48	1417	59	M	7	7a	yes	spread	oxer	right	no	0	0	68,83	no
Madrid	MUTUA MADRILEÑA TROPHY GRAND PRIX CSI*5 W round A	25.11.2017	CSI-W5*	3	1.60	430	195	11	25.04.2007	G	SF	164	16	9	103	48	1417	59	M	8	7b	yes	vertical	vertical	right	no	0	0	68,83	no
Madrid	MUTUA MADRILEÑA TROPHY GRAND PRIX CSI*5 W round A	25.11.2017	CSI-W5*	3	1.60	430	195	11	25.04.2007	G	SF	164	16	9	103	48	1417	59	M	9	8	no	spread	oxer	right	no	0	0	68,83	no
Madrid	MUTUA MADRILEÑA TROPHY GRAND PRIX CSI*5 W round A	25.11.2017	CSI-W5*	3	1.60	430	195	11	25.04.2007	G	SF	164	16	9	103	48	1417	59	M	10	9	no	vertical	vertical	right	no	0	0	68,83	no
Madrid	MUTUA MADRILEÑA TROPHY GRAND PRIX CSI*5 W round A	25.11.2017	CSI-W5*	3	1.60	430	195	11	25.04.2007	G	SF	164	16	9	103	48	1417	59	M	11	10	no	spread	oxer	straight	no	0	0	68,83	no
Madrid	MUTUA MADRILEÑA TROPHY GRAND PRIX CSI*5 W round A	25.11.2017	CSI-W5*	3	1.60	430	195	11	25.04.2007	G	SF	164	16	9	103	48	1417	59	M	12	11	no	vertical	vertical with water	left	no	0	0	68,83	no
Madrid	MUTUA MADRILEÑA TROPHY GRAND PRIX CSI*5 W round A	25.11.2017	CSI-W5*	3	1.60	430	195	11	25.04.2007	G	SF	164	16	9	103	48	1417	59	M	13	12a	yes	vertical	vertical	left	no	0	0	68,83	no
Madrid	MUTUA MADRILEÑA TROPHY GRAND PRIX CSI*5 W round A	25.11.2017	CSI-W5*	3	1.60	430	195	11	25.04.2007	G	SF	164	16	9	103	48	1417	59	M	14	12b	yes	spread	oxer with water	left	no	0	0	68,83	no
Madrid	MUTUA MADRILEÑA TROPHY GRAND PRIX CSI*5 W round A	25.11.2017	CSI-W5*	3	1.60	430	195	11	25.04.2007	G	SF	164	16	9	103	48	1417	59	M	15	12c	yes	vertical	vertical	left	no	0	0	68,83	no
Madrid	MUTUA MADRILEÑA TROPHY GRAND PRIX CSI*5 W round A	25.11.2017	CSI-W5*	3	1.60	430	195	11	25.04.2007	G	SF	164	16	9	103	48	1417	59	M	16	13	no	spread	oxer	right	no	0	0	68,83	no
Madrid	MUTUA MADRILEÑA TROPHY GRAND PRIX CSI*5 W round B	25.11.2017	CSI-W5*	3	1.60	300	195	11	25.04.2007	G	SF	164	16	9	103	48	1417	59	M	1	1	no	spread	oxer	right	no	0	0	43,22	no
Madrid	MUTUA MADRILEÑA TROPHY GRAND PRIX CSI*5 W round B	25.11.2017	CSI-W5*	3	1.60	300	195	11	25.04.2007	G	SF	164	16	9	103	48	1417	59	M	2	2	no	spread	oxer	right	no	0	0	43,22	no
Madrid	MUTUA MADRILEÑA TROPHY GRAND PRIX CSI*5 W round B	25.11.2017	CSI-W5*	3	1.60	300	195	11	25.04.2007	G	SF	164	16	9	103	48	1417	59	M	3	3	no	vertical	vertical with water	left	no	0	0	43,22	no
Madrid	MUTUA MADRILEÑA TROPHY GRAND PRIX CSI*5 W round B	25.11.2017	CSI-W5*	3	1.60	300	195	11	25.04.2007	G	SF	164	16	9	103	48	1417	59	M	4	4a	yes	vertical	vertical	left	no	0	0	43,22	no
Madrid	MUTUA MADRILEÑA TROPHY GRAND PRIX CSI*5 W round B	25.11.2017	CSI-W5*	3	1.60	300	195	11	25.04.2007	G	SF	164	16	9	103	48	1417	59	M	5	4b	yes	spread	oxer with water	left	no	0	0	43,22	no
Madrid	MUTUA MADRILEÑA TROPHY GRAND PRIX CSI*5 W round B	25.11.2017	CSI-W5*	3	1.60	300	195	11	25.04.2007	G	SF	164	16	9	103	48	1417	59	M	6	5	no	spread	oxer	right	no	0	0	43,22	no
Madrid	MUTUA MADRILEÑA TROPHY GRAND PRIX CSI*5 W round B	25.11.2017	CSI-W5*	3	1.60	300	195	11	25.04.2007	G	SF	164	16	9	103	48	1417	59	M	7	6	no	vertical	vertical	right	no	0	0	43,22	no
Madrid	MUTUA MADRILEÑA TROPHY GRAND PRIX CSI*5 W round B	25.11.2017	CSI-W5*	3	1.60	300	195	11	25.04.2007	G	SF	164	16	9	103	48	1417	59	M	8	7	no	vertical	vertical	left	no	0	0	43,22	no
Madrid	MUTUA MADRILEÑA TROPHY GRAND PRIX CSI*5 W round B	25.11.2017	CSI-W5*	3	1.60	300	195	11	25.04.2007	G	SF	164	16	9	103	48	1417	59	M	9	8	no	spread	oxer	left	no	0	0	43,22	no
Madrid	MUTUA MADRILEÑA TROPHY GRAND PRIX CSI*5 W round A	25.11.2017	CSI-W5*	3	1.60	430	73	17	02.05.2001	G	BWP	229	4	12	235	58	1047	25	M	1	1	no	spread	oxer	left	no	0	0	74,3	no
Madrid	MUTUA MADRILEÑA TROPHY GRAND PRIX CSI*5 W round A	25.11.2017	CSI-W5*	3	1.60	430	73	17	02.05.2001	G	BWP	229	4	12	235	58	1047	25	M	2	2	no	vertical	vertical	straight	no	0	0	74,3	no
Madrid	MUTUA MADRILEÑA TROPHY GRAND PRIX CSI*5 W round A	25.11.2017	CSI-W5*	3	1.60	430	73	17	02.05.2001	G	BWP	229	4	12	235	58	1047	25	M	3	3	no	spread	oxer	right	no	0	0	74,3	no
Madrid	MUTUA MADRILEÑA TROPHY GRAND PRIX CSI*5 W round A	25.11.2017	CSI-W5*	3	1.60	430	73	17	02.05.2001	G	BWP	229	4	12	235	58	1047	25	M	4	4	no	vertical	vertical	straight	no	0	0	74,3	no
Madrid	MUTUA MADRILEÑA TROPHY GRAND PRIX CSI*5 W round A	25.11.2017	CSI-W5*	3	1.60	430	73	17	02.05.2001	G	BWP	229	4	12	235	58	1047	25	M	5	5	no	spread	oxer	left	no	0	0	74,3	no
Madrid	MUTUA MADRILEÑA TROPHY GRAND PRIX CSI*5 W round A	25.11.2017	CSI-W5*	3	1.60	430	73	17	02.05.2001	G	BWP	229	4	12	235	58	1047	25	M	6	6	no	vertical	vertical	left	no	0	0	74,3	no
Madrid	MUTUA MADRILEÑA TROPHY GRAND PRIX CSI*5 W round A	25.11.2017	CSI-W5*	3	1.60	430	73	17	02.05.2001	G	BWP	229	4	12	235	58	1047	25	M	7	7a	yes	spread	oxer	right	no	0	0	74,3	no
Madrid	MUTUA MADRILEÑA TROPHY GRAND PRIX CSI*5 W round A	25.11.2017	CSI-W5*	3	1.60	430	73	17	02.05.2001	G	BWP	229	4	12	235	58	1047	25	M	8	7b	yes	vertical	vertical	right	no	0	0	74,3	no
Madrid	MUTUA MADRILEÑA TROPHY GRAND PRIX CSI*5 W round A	25.11.2017	CSI-W5*	3	1.60	430	73	17	02.05.2001	G	BWP	229	4	12	235	58	1047	25	M	9	8	no	spread	oxer	right	no	0	0	74,3	no
Madrid	MUTUA MADRILEÑA TROPHY GRAND PRIX CSI*5 W round A	25.11.2017	CSI-W5*	3	1.60	430	73	17	02.05.2001	G	BWP	229	4	12	235	58	1047	25	M	10	9	no	vertical	vertical	right	no	0	0	74,3	no
Madrid	MUTUA MADRILEÑA TROPHY GRAND PRIX CSI*5 W round A	25.11.2017	CSI-W5*	3	1.60	430	73	17	02.05.2001	G	BWP	229	4	12	235	58	1047	25	M	11	10	no	spread	oxer	straight	no	0	0	74,3	no
Madrid	MUTUA MADRILEÑA TROPHY GRAND PRIX CSI*5 W round A	25.11.2017	CSI-W5*	3	1.60	430	73	17	02.05.2001	G	BWP	229	4	12	235	58	1047	25	M	12	11	no	vertical	vertical with water	left	no	0	0	74,3	no
Madrid	MUTUA MADRILEÑA TROPHY GRAND PRIX CSI*5 W round A	25.11.2017	CSI-W5*	3	1.60	430	73	17	02.05.2001	G	BWP	229	4	12	235	58	1047	25	M	13	12a	yes	vertical	vertical	left	no	0	0	74,3	no
Madrid	MUTUA MADRILEÑA TROPHY GRAND PRIX CSI*5 W round A	25.11.2017	CSI-W5*	3	1.60	430	73	17	02.05.2001	G	BWP	229	4	12	235	58	1047	25	M	14	12b	yes	spread	oxer with water	left	no	0	0	74,3	no
Madrid	MUTUA MADRILEÑA TROPHY GRAND PRIX CSI*5 W round A	25.11.2017	CSI-W5*	3	1.60	430	73	17	02.05.2001	G	BWP	229	4	12	235	58	1047	25	M	15	12c	yes	vertical	vertical	left	no	0	0	74,3	no
Madrid	MUTUA MADRILEÑA TROPHY GRAND PRIX CSI*5 W round A	25.11.2017	CSI-W5*	3	1.60	430	73	17	02.05.2001	G	BWP	229	4	12	235	58	1047	25	M	16	13	no	spread	oxer	right	yes	4	1	74,3	no
Madrid	MUTUA MADRILEÑA TROPHY GRAND PRIX CSI*5 W round A	25.11.2017	CSI-W5*	3	1.60	430	196	13	15.05.2005	M	ZANG	166	4	14	5	37	1868	96	M	1	1	no	spread	oxer	left	no	0	0	70,45	no
Madrid	MUTUA MADRILEÑA TROPHY GRAND PRIX CSI*5 W round A	25.11.2017	CSI-W5*	3	1.60	430	196	13	15.05.2005	M	ZANG	166	4	14	5	37	1868	96	M	2	2	no	vertical	vertical	straight	no	0	0	70,45	no
Madrid	MUTUA MADRILEÑA TROPHY GRAND PRIX CSI*5 W round A	25.11.2017	CSI-W5*	3	1.60	430	196	13	15.05.2005	M	ZANG	166	4	14	5	37	1868	96	M	3	3	no	spread	oxer	right	no	0	0	70,45	no
Madrid	MUTUA MADRILEÑA TROPHY GRAND PRIX CSI*5 W round A	25.11.2017	CSI-W5*	3	1.60	430	196	13	15.05.2005	M	ZANG	166	4	14	5	37	1868	96	M	4	4	no	vertical	vertical	straight	no	0	0	70,45	no
Madrid	MUTUA MADRILEÑA TROPHY GRAND PRIX CSI*5 W round A	25.11.2017	CSI-W5*	3	1.60	430	196	13	15.05.2005	M	ZANG	166	4	14	5	37	1868	96	M	5	5	no	spread	oxer	left	no	0	0	70,45	no
Madrid	MUTUA MADRILEÑA TROPHY GRAND PRIX CSI*5 W round A	25.11.2017	CSI-W5*	3	1.60	430	196	13	15.05.2005	M	ZANG	166	4	14	5	37	1868	96	M	6	6	no	vertical	vertical	left	no	0	0	70,45	no
Madrid	MUTUA MADRILEÑA TROPHY GRAND PRIX CSI*5 W round A	25.11.2017	CSI-W5*	3	1.60	430	196	13	15.05.2005	M	ZANG	166	4	14	5	37	1868	96	M	7	7a	yes	spread	oxer	right	no	0	0	70,45	no
Madrid	MUTUA MADRILEÑA TROPHY GRAND PRIX CSI*5 W round A	25.11.2017	CSI-W5*	3	1.60	430	196	13	15.05.2005	M	ZANG	166	4	14	5	37	1868	96	M	8	7b	yes	vertical	vertical	right	no	0	0	70,45	no
Madrid	MUTUA MADRILEÑA TROPHY GRAND PRIX CSI*5 W round A	25.11.2017	CSI-W5*	3	1.60	430	196	13	15.05.2005	M	ZANG	166	4	14	5	37	1868	96	M	9	8	no	spread	oxer	right	no	0	0	70,45	no
Madrid	MUTUA MADRILEÑA TROPHY GRAND PRIX CSI*5 W round A	25.11.2017	CSI-W5*	3	1.60	430	196	13	15.05.2005	M	ZANG	166	4	14	5	37	1868	96	M	10	9	no	vertical	vertical	right	no	0	0	70,45	no
Madrid	MUTUA MADRILEÑA TROPHY GRAND PRIX CSI*5 W round A	25.11.2017	CSI-W5*	3	1.60	430	196	13	15.05.2005	M	ZANG	166	4	14	5	37	1868	96	M	11	10	no	spread	oxer	straight	no	0	0	70,45	no
Madrid	MUTUA MADRILEÑA TROPHY GRAND PRIX CSI*5 W round A	25.11.2017	CSI-W5*	3	1.60	430	196	13	15.05.2005	M	ZANG	166	4	14	5	37	1868	96	M	12	11	no	vertical	vertical with water	left	no	0	0	70,45	no
Madrid	MUTUA MADRILEÑA TROPHY GRAND PRIX CSI*5 W round A	25.11.2017	CSI-W5*	3	1.60	430	196	13	15.05.2005	M	ZANG	166	4	14	5	37	1868	96	M	13	12a	yes	vertical	vertical	left	no	0	0	70,45	no
Madrid	MUTUA MADRILEÑA TROPHY GRAND PRIX CSI*5 W round A	25.11.2017	CSI-W5*	3	1.60	430	196	13	15.05.2005	M	ZANG	166	4	14	5	37	1868	96	M	14	12b	yes	spread	oxer with water	left	no	0	0	70,45	no
Madrid	MUTUA MADRILEÑA TROPHY GRAND PRIX CSI*5 W round A	25.11.2017	CSI-W5*	3	1.60	430	196	13	15.05.2005	M	ZANG	166	4	14	5	37	1868	96	M	15	12c	yes	vertical	vertical	left	no	0	0	70,45	no
Madrid	MUTUA MADRILEÑA TROPHY GRAND PRIX CSI*5 W round A	25.11.2017	CSI-W5*	3	1.60	430	196	13	15.05.2005	M	ZANG	166	4	14	5	37	1868	96	M	16	13	no	spread	oxer	right	no	0	0	70,45	no
Madrid	MUTUA MADRILEÑA TROPHY GRAND PRIX CSI*5 W round B	25.11.2017	CSI-W5*	3	1.60	300	196	13	15.05.2005	M	ZANG	166	4	14	5	37	1868	96	M	1	1	no	spread	oxer	right	no	0	0	45,74	no
Madrid	MUTUA MADRILEÑA TROPHY GRAND PRIX CSI*5 W round B	25.11.2017	CSI-W5*	3	1.60	300	196	13	15.05.2005	M	ZANG	166	4	14	5	37	1868	96	M	2	2	no	spread	oxer	right	yes	4	0	45,74	no
Madrid	MUTUA MADRILEÑA TROPHY GRAND PRIX CSI*5 W round B	25.11.2017	CSI-W5*	3	1.60	300	196	13	15.05.2005	M	ZANG	166	4	14	5	37	1868	96	M	3	3	no	vertical	vertical with water	left	no	0	0	45,74	no
Madrid	MUTUA MADRILEÑA TROPHY GRAND PRIX CSI*5 W round B	25.11.2017	CSI-W5*	3	1.60	300	196	13	15.05.2005	M	ZANG	166	4	14	5	37	1868	96	M	4	4a	yes	vertical	vertical	left	yes	4	0	45,74	no
Madrid	MUTUA MADRILEÑA TROPHY GRAND PRIX CSI*5 W round B	25.11.2017	CSI-W5*	3	1.60	300	196	13	15.05.2005	M	ZANG	166	4	14	5	37	1868	96	M	5	4b	yes	spread	oxer with water	left	no	0	0	45,74	no
Madrid	MUTUA MADRILEÑA TROPHY GRAND PRIX CSI*5 W round B	25.11.2017	CSI-W5*	3	1.60	300	196	13	15.05.2005	M	ZANG	166	4	14	5	37	1868	96	M	6	5	no	spread	oxer	right	no	0	0	45,74	no
Madrid	MUTUA MADRILEÑA TROPHY GRAND PRIX CSI*5 W round B	25.11.2017	CSI-W5*	3	1.60	300	196	13	15.05.2005	M	ZANG	166	4	14	5	37	1868	96	M	7	6	no	vertical	vertical	right	yes	4	0	45,74	no
Madrid	MUTUA MADRILEÑA TROPHY GRAND PRIX CSI*5 W round B	25.11.2017	CSI-W5*	3	1.60	300	196	13	15.05.2005	M	ZANG	166	4	14	5	37	1868	96	M	8	7	no	vertical	vertical	left	no	0	0	45,74	no
Madrid	MUTUA MADRILEÑA TROPHY GRAND PRIX CSI*5 W round B	25.11.2017	CSI-W5*	3	1.60	300	196	13	15.05.2005	M	ZANG	166	4	14	5	37	1868	96	M	9	8	no	spread	oxer	left	no	0	0	45,74	no
Madrid	MUTUA MADRILEÑA TROPHY GRAND PRIX CSI*5 W round A	25.11.2017	CSI-W5*	3	1.60	430	56	14	12.03.2004	G	OLDBG	203	7	16	17	37	2215	106	M	1	1	no	spread	oxer	left	no	0	0	70,97	no
Madrid	MUTUA MADRILEÑA TROPHY GRAND PRIX CSI*5 W round A	25.11.2017	CSI-W5*	3	1.60	430	56	14	12.03.2004	G	OLDBG	203	7	16	17	37	2215	106	M	2	2	no	vertical	vertical	straight	no	0	0	70,97	no
Madrid	MUTUA MADRILEÑA TROPHY GRAND PRIX CSI*5 W round A	25.11.2017	CSI-W5*	3	1.60	430	56	14	12.03.2004	G	OLDBG	203	7	16	17	37	2215	106	M	3	3	no	spread	oxer	right	no	0	0	70,97	no
Madrid	MUTUA MADRILEÑA TROPHY GRAND PRIX CSI*5 W round A	25.11.2017	CSI-W5*	3	1.60	430	56	14	12.03.2004	G	OLDBG	203	7	16	17	37	2215	106	M	4	4	no	vertical	vertical	straight	no	0	0	70,97	no
Madrid	MUTUA MADRILEÑA TROPHY GRAND PRIX CSI*5 W round A	25.11.2017	CSI-W5*	3	1.60	430	56	14	12.03.2004	G	OLDBG	203	7	16	17	37	2215	106	M	5	5	no	spread	oxer	left	no	0	0	70,97	no
Madrid	MUTUA MADRILEÑA TROPHY GRAND PRIX CSI*5 W round A	25.11.2017	CSI-W5*	3	1.60	430	56	14	12.03.2004	G	OLDBG	203	7	16	17	37	2215	106	M	6	6	no	vertical	vertical	left	no	0	0	70,97	no
Madrid	MUTUA MADRILEÑA TROPHY GRAND PRIX CSI*5 W round A	25.11.2017	CSI-W5*	3	1.60	430	56	14	12.03.2004	G	OLDBG	203	7	16	17	37	2215	106	M	7	7a	yes	spread	oxer	right	no	0	0	70,97	no
Madrid	MUTUA MADRILEÑA TROPHY GRAND PRIX CSI*5 W round A	25.11.2017	CSI-W5*	3	1.60	430	56	14	12.03.2004	G	OLDBG	203	7	16	17	37	2215	106	M	8	7b	yes	vertical	vertical	right	no	0	0	70,97	no
Madrid	MUTUA MADRILEÑA TROPHY GRAND PRIX CSI*5 W round A	25.11.2017	CSI-W5*	3	1.60	430	56	14	12.03.2004	G	OLDBG	203	7	16	17	37	2215	106	M	9	8	no	spread	oxer	right	no	0	0	70,97	no
Madrid	MUTUA MADRILEÑA TROPHY GRAND PRIX CSI*5 W round A	25.11.2017	CSI-W5*	3	1.60	430	56	14	12.03.2004	G	OLDBG	203	7	16	17	37	2215	106	M	10	9	no	vertical	vertical	right	no	0	0	70,97	no
Madrid	MUTUA MADRILEÑA TROPHY GRAND PRIX CSI*5 W round A	25.11.2017	CSI-W5*	3	1.60	430	56	14	12.03.2004	G	OLDBG	203	7	16	17	37	2215	106	M	11	10	no	spread	oxer	straight	no	0	0	70,97	no
Madrid	MUTUA MADRILEÑA TROPHY GRAND PRIX CSI*5 W round A	25.11.2017	CSI-W5*	3	1.60	430	56	14	12.03.2004	G	OLDBG	203	7	16	17	37	2215	106	M	12	11	no	vertical	vertical with water	left	no	0	0	70,97	no
Madrid	MUTUA MADRILEÑA TROPHY GRAND PRIX CSI*5 W round A	25.11.2017	CSI-W5*	3	1.60	430	56	14	12.03.2004	G	OLDBG	203	7	16	17	37	2215	106	M	13	12a	yes	vertical	vertical	left	no	0	0	70,97	no
Madrid	MUTUA MADRILEÑA TROPHY GRAND PRIX CSI*5 W round A	25.11.2017	CSI-W5*	3	1.60	430	56	14	12.03.2004	G	OLDBG	203	7	16	17	37	2215	106	M	14	12b	yes	spread	oxer with water	left	no	0	0	70,97	no
Madrid	MUTUA MADRILEÑA TROPHY GRAND PRIX CSI*5 W round A	25.11.2017	CSI-W5*	3	1.60	430	56	14	12.03.2004	G	OLDBG	203	7	16	17	37	2215	106	M	15	12c	yes	vertical	vertical	left	no	0	0	70,97	no
Madrid	MUTUA MADRILEÑA TROPHY GRAND PRIX CSI*5 W round A	25.11.2017	CSI-W5*	3	1.60	430	56	14	12.03.2004	G	OLDBG	203	7	16	17	37	2215	106	M	16	13	no	spread	oxer	right	no	0	0	70,97	no
Madrid	MUTUA MADRILEÑA TROPHY GRAND PRIX CSI*5 W round B	25.11.2017	CSI-W5*	3	1.60	300	56	14	12.03.2004	G	OLDBG	203	7	16	17	37	2215	106	M	1	1	no	spread	oxer	right	no	0	0	43,19	no
Madrid	MUTUA MADRILEÑA TROPHY GRAND PRIX CSI*5 W round B	25.11.2017	CSI-W5*	3	1.60	300	56	14	12.03.2004	G	OLDBG	203	7	16	17	37	2215	106	M	2	2	no	spread	oxer	right	no	0	0	43,19	no
Madrid	MUTUA MADRILEÑA TROPHY GRAND PRIX CSI*5 W round B	25.11.2017	CSI-W5*	3	1.60	300	56	14	12.03.2004	G	OLDBG	203	7	16	17	37	2215	106	M	3	3	no	vertical	vertical with water	left	no	0	0	43,19	no
Madrid	MUTUA MADRILEÑA TROPHY GRAND PRIX CSI*5 W round B	25.11.2017	CSI-W5*	3	1.60	300	56	14	12.03.2004	G	OLDBG	203	7	16	17	37	2215	106	M	4	4a	yes	vertical	vertical	left	no	0	0	43,19	no
Madrid	MUTUA MADRILEÑA TROPHY GRAND PRIX CSI*5 W round B	25.11.2017	CSI-W5*	3	1.60	300	56	14	12.03.2004	G	OLDBG	203	7	16	17	37	2215	106	M	5	4b	yes	spread	oxer with water	left	no	0	0	43,19	no
Madrid	MUTUA MADRILEÑA TROPHY GRAND PRIX CSI*5 W round B	25.11.2017	CSI-W5*	3	1.60	300	56	14	12.03.2004	G	OLDBG	203	7	16	17	37	2215	106	M	6	5	no	spread	oxer	right	no	0	0	43,19	no
Madrid	MUTUA MADRILEÑA TROPHY GRAND PRIX CSI*5 W round B	25.11.2017	CSI-W5*	3	1.60	300	56	14	12.03.2004	G	OLDBG	203	7	16	17	37	2215	106	M	7	6	no	vertical	vertical	right	no	0	0	43,19	no
Madrid	MUTUA MADRILEÑA TROPHY GRAND PRIX CSI*5 W round B	25.11.2017	CSI-W5*	3	1.60	300	56	14	12.03.2004	G	OLDBG	203	7	16	17	37	2215	106	M	8	7	no	vertical	vertical	left	no	0	0	43,19	no
Madrid	MUTUA MADRILEÑA TROPHY GRAND PRIX CSI*5 W round B	25.11.2017	CSI-W5*	3	1.60	300	56	14	12.03.2004	G	OLDBG	203	7	16	17	37	2215	106	M	9	8	no	spread	oxer	left	no	0	0	43,19	no
Madrid	MUTUA MADRILEÑA TROPHY GRAND PRIX CSI*5 W round A	25.11.2017	CSI-W5*	3	1.60	430	154	14	29.04.2004	G	SWB	175	3	18	81	31	1327	0	M	1	1	no	spread	oxer	left	no	0	0	71,08	no
Madrid	MUTUA MADRILEÑA TROPHY GRAND PRIX CSI*5 W round A	25.11.2017	CSI-W5*	3	1.60	430	154	14	29.04.2004	G	SWB	175	3	18	81	31	1327	0	M	2	2	no	vertical	vertical	straight	no	0	0	71,08	no
Madrid	MUTUA MADRILEÑA TROPHY GRAND PRIX CSI*5 W round A	25.11.2017	CSI-W5*	3	1.60	430	154	14	29.04.2004	G	SWB	175	3	18	81	31	1327	0	M	3	3	no	spread	oxer	right	no	0	0	71,08	no
Madrid	MUTUA MADRILEÑA TROPHY GRAND PRIX CSI*5 W round A	25.11.2017	CSI-W5*	3	1.60	430	154	14	29.04.2004	G	SWB	175	3	18	81	31	1327	0	M	4	4	no	vertical	vertical	straight	no	0	0	71,08	no
Madrid	MUTUA MADRILEÑA TROPHY GRAND PRIX CSI*5 W round A	25.11.2017	CSI-W5*	3	1.60	430	154	14	29.04.2004	G	SWB	175	3	18	81	31	1327	0	M	5	5	no	spread	oxer	left	no	0	0	71,08	no
Madrid	MUTUA MADRILEÑA TROPHY GRAND PRIX CSI*5 W round A	25.11.2017	CSI-W5*	3	1.60	430	154	14	29.04.2004	G	SWB	175	3	18	81	31	1327	0	M	6	6	no	vertical	vertical	left	no	0	0	71,08	no
Madrid	MUTUA MADRILEÑA TROPHY GRAND PRIX CSI*5 W round A	25.11.2017	CSI-W5*	3	1.60	430	154	14	29.04.2004	G	SWB	175	3	18	81	31	1327	0	M	7	7a	yes	spread	oxer	right	no	0	0	71,08	no
Madrid	MUTUA MADRILEÑA TROPHY GRAND PRIX CSI*5 W round A	25.11.2017	CSI-W5*	3	1.60	430	154	14	29.04.2004	G	SWB	175	3	18	81	31	1327	0	M	8	7b	yes	vertical	vertical	right	no	0	0	71,08	no
Madrid	MUTUA MADRILEÑA TROPHY GRAND PRIX CSI*5 W round A	25.11.2017	CSI-W5*	3	1.60	430	154	14	29.04.2004	G	SWB	175	3	18	81	31	1327	0	M	9	8	no	spread	oxer	right	no	0	0	71,08	no
Madrid	MUTUA MADRILEÑA TROPHY GRAND PRIX CSI*5 W round A	25.11.2017	CSI-W5*	3	1.60	430	154	14	29.04.2004	G	SWB	175	3	18	81	31	1327	0	M	10	9	no	vertical	vertical	right	no	0	0	71,08	no
Madrid	MUTUA MADRILEÑA TROPHY GRAND PRIX CSI*5 W round A	25.11.2017	CSI-W5*	3	1.60	430	154	14	29.04.2004	G	SWB	175	3	18	81	31	1327	0	M	11	10	no	spread	oxer	straight	no	0	0	71,08	no
Madrid	MUTUA MADRILEÑA TROPHY GRAND PRIX CSI*5 W round A	25.11.2017	CSI-W5*	3	1.60	430	154	14	29.04.2004	G	SWB	175	3	18	81	31	1327	0	M	12	11	no	vertical	vertical with water	left	no	0	0	71,08	no
Madrid	MUTUA MADRILEÑA TROPHY GRAND PRIX CSI*5 W round A	25.11.2017	CSI-W5*	3	1.60	430	154	14	29.04.2004	G	SWB	175	3	18	81	31	1327	0	M	13	12a	yes	vertical	vertical	left	no	0	0	71,08	no
Madrid	MUTUA MADRILEÑA TROPHY GRAND PRIX CSI*5 W round A	25.11.2017	CSI-W5*	3	1.60	430	154	14	29.04.2004	G	SWB	175	3	18	81	31	1327	0	M	14	12b	yes	spread	oxer with water	left	no	0	0	71,08	no
Madrid	MUTUA MADRILEÑA TROPHY GRAND PRIX CSI*5 W round A	25.11.2017	CSI-W5*	3	1.60	430	154	14	29.04.2004	G	SWB	175	3	18	81	31	1327	0	M	15	12c	yes	vertical	vertical	left	no	0	0	71,08	no
Madrid	MUTUA MADRILEÑA TROPHY GRAND PRIX CSI*5 W round A	25.11.2017	CSI-W5*	3	1.60	430	154	14	29.04.2004	G	SWB	175	3	18	81	31	1327	0	M	16	13	no	spread	oxer	right	no	0	0	71,08	no
Madrid	MUTUA MADRILEÑA TROPHY GRAND PRIX CSI*5 W round B	25.11.2017	CSI-W5*	3	1.60	300	154	14	29.04.2004	G	SWB	175	3	18	81	31	1327	0	M	1	1	no	spread	oxer	right	no	0	0	42,68	no
Madrid	MUTUA MADRILEÑA TROPHY GRAND PRIX CSI*5 W round B	25.11.2017	CSI-W5*	3	1.60	300	154	14	29.04.2004	G	SWB	175	3	18	81	31	1327	0	M	2	2	no	spread	oxer	right	no	0	0	42,68	no
Madrid	MUTUA MADRILEÑA TROPHY GRAND PRIX CSI*5 W round B	25.11.2017	CSI-W5*	3	1.60	300	154	14	29.04.2004	G	SWB	175	3	18	81	31	1327	0	M	3	3	no	vertical	vertical with water	left	no	0	0	42,68	no
Madrid	MUTUA MADRILEÑA TROPHY GRAND PRIX CSI*5 W round B	25.11.2017	CSI-W5*	3	1.60	300	154	14	29.04.2004	G	SWB	175	3	18	81	31	1327	0	M	4	4a	yes	vertical	vertical	left	no	0	0	42,68	no
Madrid	MUTUA MADRILEÑA TROPHY GRAND PRIX CSI*5 W round B	25.11.2017	CSI-W5*	3	1.60	300	154	14	29.04.2004	G	SWB	175	3	18	81	31	1327	0	M	5	4b	yes	spread	oxer with water	left	no	0	0	42,68	no
Madrid	MUTUA MADRILEÑA TROPHY GRAND PRIX CSI*5 W round B	25.11.2017	CSI-W5*	3	1.60	300	154	14	29.04.2004	G	SWB	175	3	18	81	31	1327	0	M	6	5	no	spread	oxer	right	no	0	0	42,68	no
Madrid	MUTUA MADRILEÑA TROPHY GRAND PRIX CSI*5 W round B	25.11.2017	CSI-W5*	3	1.60	300	154	14	29.04.2004	G	SWB	175	3	18	81	31	1327	0	M	7	6	no	vertical	vertical	right	no	0	0	42,68	no
Madrid	MUTUA MADRILEÑA TROPHY GRAND PRIX CSI*5 W round B	25.11.2017	CSI-W5*	3	1.60	300	154	14	29.04.2004	G	SWB	175	3	18	81	31	1327	0	M	8	7	no	vertical	vertical	left	no	0	0	42,68	no
Madrid	MUTUA MADRILEÑA TROPHY GRAND PRIX CSI*5 W round B	25.11.2017	CSI-W5*	3	1.60	300	154	14	29.04.2004	G	SWB	175	3	18	81	31	1327	0	M	9	8	no	spread	oxer	left	no	0	0	42,68	no
Madrid	MUTUA MADRILEÑA TROPHY GRAND PRIX CSI*5 W round A	25.11.2017	CSI-W5*	3	1.60	430	218	12	24.05.2006	M	HOLST	115	0	22	144	55	798	31	M	1	1	no	spread	oxer	left	no	0	0	86,37	no
Madrid	MUTUA MADRILEÑA TROPHY GRAND PRIX CSI*5 W round A	25.11.2017	CSI-W5*	3	1.60	430	218	12	24.05.2006	M	HOLST	115	0	22	144	55	798	31	M	2	2	no	vertical	vertical	straight	no	0	0	86,37	no
Madrid	MUTUA MADRILEÑA TROPHY GRAND PRIX CSI*5 W round A	25.11.2017	CSI-W5*	3	1.60	430	218	12	24.05.2006	M	HOLST	115	0	22	144	55	798	31	M	3	3	no	spread	oxer	right	no	0	0	86,37	no
Madrid	MUTUA MADRILEÑA TROPHY GRAND PRIX CSI*5 W round A	25.11.2017	CSI-W5*	3	1.60	430	218	12	24.05.2006	M	HOLST	115	0	22	144	55	798	31	M	4	4	no	vertical	vertical	straight	no	0	0	86,37	no
Madrid	MUTUA MADRILEÑA TROPHY GRAND PRIX CSI*5 W round A	25.11.2017	CSI-W5*	3	1.60	430	218	12	24.05.2006	M	HOLST	115	0	22	144	55	798	31	M	5	5	no	spread	oxer	left	no	0	0	86,37	no
Madrid	MUTUA MADRILEÑA TROPHY GRAND PRIX CSI*5 W round A	25.11.2017	CSI-W5*	3	1.60	430	218	12	24.05.2006	M	HOLST	115	0	22	144	55	798	31	M	6	6	no	vertical	vertical	left	no	0	0	86,37	no
Madrid	MUTUA MADRILEÑA TROPHY GRAND PRIX CSI*5 W round A	25.11.2017	CSI-W5*	3	1.60	430	218	12	24.05.2006	M	HOLST	115	0	22	144	55	798	31	M	7	7a	yes	spread	oxer	right	no	0	0	86,37	no
Madrid	MUTUA MADRILEÑA TROPHY GRAND PRIX CSI*5 W round A	25.11.2017	CSI-W5*	3	1.60	430	218	12	24.05.2006	M	HOLST	115	0	22	144	55	798	31	M	8	7b	yes	vertical	vertical	right	no	0	0	86,37	no
Madrid	MUTUA MADRILEÑA TROPHY GRAND PRIX CSI*5 W round A	25.11.2017	CSI-W5*	3	1.60	430	218	12	24.05.2006	M	HOLST	115	0	22	144	55	798	31	M	9	8	no	spread	oxer	right	no	0	0	86,37	no
Madrid	MUTUA MADRILEÑA TROPHY GRAND PRIX CSI*5 W round A	25.11.2017	CSI-W5*	3	1.60	430	218	12	24.05.2006	M	HOLST	115	0	22	144	55	798	31	M	10	9	no	vertical	vertical	right	no	0	0	86,37	no
Madrid	MUTUA MADRILEÑA TROPHY GRAND PRIX CSI*5 W round A	25.11.2017	CSI-W5*	3	1.60	430	218	12	24.05.2006	M	HOLST	115	0	22	144	55	798	31	M	11	10	no	spread	oxer	straight	no	0	0	86,37	no
Madrid	MUTUA MADRILEÑA TROPHY GRAND PRIX CSI*5 W round A	25.11.2017	CSI-W5*	3	1.60	430	218	12	24.05.2006	M	HOLST	115	0	22	144	55	798	31	M	12	11	no	vertical	vertical with water	left	no	0	0	86,37	no
Madrid	MUTUA MADRILEÑA TROPHY GRAND PRIX CSI*5 W round A	25.11.2017	CSI-W5*	3	1.60	430	218	12	24.05.2006	M	HOLST	115	0	22	144	55	798	31	M	13	12a	yes	vertical	vertical	left	no	0	0	86,37	no
Madrid	MUTUA MADRILEÑA TROPHY GRAND PRIX CSI*5 W round A	25.11.2017	CSI-W5*	3	1.60	430	218	12	24.05.2006	M	HOLST	115	0	22	144	55	798	31	M	14	12b	yes	spread	oxer with water	left	no	0	0	86,37	no
Madrid	MUTUA MADRILEÑA TROPHY GRAND PRIX CSI*5 W round A	25.11.2017	CSI-W5*	3	1.60	430	218	12	24.05.2006	M	HOLST	115	0	22	144	55	798	31	M	15	12c	yes	vertical	vertical	left	no	0	0	86,37	no
Madrid	MUTUA MADRILEÑA TROPHY GRAND PRIX CSI*5 W round A	25.11.2017	CSI-W5*	3	1.60	430	218	12	24.05.2006	M	HOLST	115	0	22	144	55	798	31	M	16	13	no	spread	oxer	right	yes	4	4	86,37	no
Madrid	MUTUA MADRILEÑA TROPHY GRAND PRIX CSI*5 W round A	25.11.2017	CSI-W5*	3	1.60	430	28	12	26.03.2006	M	ZANG	193	9	23	11	35	1800	65	M	1	1	no	spread	oxer	left	no	0	0	72,52	no
Madrid	MUTUA MADRILEÑA TROPHY GRAND PRIX CSI*5 W round A	25.11.2017	CSI-W5*	3	1.60	430	28	12	26.03.2006	M	ZANG	193	9	23	11	35	1800	65	M	2	2	no	vertical	vertical	straight	no	0	0	72,52	no
Madrid	MUTUA MADRILEÑA TROPHY GRAND PRIX CSI*5 W round A	25.11.2017	CSI-W5*	3	1.60	430	28	12	26.03.2006	M	ZANG	193	9	23	11	35	1800	65	M	3	3	no	spread	oxer	right	no	0	0	72,52	no
Madrid	MUTUA MADRILEÑA TROPHY GRAND PRIX CSI*5 W round A	25.11.2017	CSI-W5*	3	1.60	430	28	12	26.03.2006	M	ZANG	193	9	23	11	35	1800	65	M	4	4	no	vertical	vertical	straight	no	0	0	72,52	no
Madrid	MUTUA MADRILEÑA TROPHY GRAND PRIX CSI*5 W round A	25.11.2017	CSI-W5*	3	1.60	430	28	12	26.03.2006	M	ZANG	193	9	23	11	35	1800	65	M	5	5	no	spread	oxer	left	no	0	0	72,52	no
Madrid	MUTUA MADRILEÑA TROPHY GRAND PRIX CSI*5 W round A	25.11.2017	CSI-W5*	3	1.60	430	28	12	26.03.2006	M	ZANG	193	9	23	11	35	1800	65	M	6	6	no	vertical	vertical	left	no	0	0	72,52	no
Madrid	MUTUA MADRILEÑA TROPHY GRAND PRIX CSI*5 W round A	25.11.2017	CSI-W5*	3	1.60	430	28	12	26.03.2006	M	ZANG	193	9	23	11	35	1800	65	M	7	7a	yes	spread	oxer	right	no	0	0	72,52	no
Madrid	MUTUA MADRILEÑA TROPHY GRAND PRIX CSI*5 W round A	25.11.2017	CSI-W5*	3	1.60	430	28	12	26.03.2006	M	ZANG	193	9	23	11	35	1800	65	M	8	7b	yes	vertical	vertical	right	no	0	0	72,52	no
Madrid	MUTUA MADRILEÑA TROPHY GRAND PRIX CSI*5 W round A	25.11.2017	CSI-W5*	3	1.60	430	28	12	26.03.2006	M	ZANG	193	9	23	11	35	1800	65	M	9	8	no	spread	oxer	right	no	0	0	72,52	no
Madrid	MUTUA MADRILEÑA TROPHY GRAND PRIX CSI*5 W round A	25.11.2017	CSI-W5*	3	1.60	430	28	12	26.03.2006	M	ZANG	193	9	23	11	35	1800	65	M	10	9	no	vertical	vertical	right	no	0	0	72,52	no
Madrid	MUTUA MADRILEÑA TROPHY GRAND PRIX CSI*5 W round A	25.11.2017	CSI-W5*	3	1.60	430	28	12	26.03.2006	M	ZANG	193	9	23	11	35	1800	65	M	11	10	no	spread	oxer	straight	no	0	0	72,52	no
Madrid	MUTUA MADRILEÑA TROPHY GRAND PRIX CSI*5 W round A	25.11.2017	CSI-W5*	3	1.60	430	28	12	26.03.2006	M	ZANG	193	9	23	11	35	1800	65	M	12	11	no	vertical	vertical with water	left	no	0	0	72,52	no
Madrid	MUTUA MADRILEÑA TROPHY GRAND PRIX CSI*5 W round A	25.11.2017	CSI-W5*	3	1.60	430	28	12	26.03.2006	M	ZANG	193	9	23	11	35	1800	65	M	13	12a	yes	vertical	vertical	left	no	0	0	72,52	no
Madrid	MUTUA MADRILEÑA TROPHY GRAND PRIX CSI*5 W round A	25.11.2017	CSI-W5*	3	1.60	430	28	12	26.03.2006	M	ZANG	193	9	23	11	35	1800	65	M	14	12b	yes	spread	oxer with water	left	no	0	0	72,52	no
Madrid	MUTUA MADRILEÑA TROPHY GRAND PRIX CSI*5 W round A	25.11.2017	CSI-W5*	3	1.60	430	28	12	26.03.2006	M	ZANG	193	9	23	11	35	1800	65	M	15	12c	yes	vertical	vertical	left	no	0	0	72,52	no
Madrid	MUTUA MADRILEÑA TROPHY GRAND PRIX CSI*5 W round A	25.11.2017	CSI-W5*	3	1.60	430	28	12	26.03.2006	M	ZANG	193	9	23	11	35	1800	65	M	16	13	no	spread	oxer	right	no	0	0	72,52	no
Madrid	MUTUA MADRILEÑA TROPHY GRAND PRIX CSI*5 W round B	25.11.2017	CSI-W5*	3	1.60	300	28	12	26.03.2006	M	ZANG	193	9	23	11	35	1800	65	M	1	1	no	spread	oxer	right	no	0	0	42,67	no
Madrid	MUTUA MADRILEÑA TROPHY GRAND PRIX CSI*5 W round B	25.11.2017	CSI-W5*	3	1.60	300	28	12	26.03.2006	M	ZANG	193	9	23	11	35	1800	65	M	2	2	no	spread	oxer	right	no	0	0	42,67	no
Madrid	MUTUA MADRILEÑA TROPHY GRAND PRIX CSI*5 W round B	25.11.2017	CSI-W5*	3	1.60	300	28	12	26.03.2006	M	ZANG	193	9	23	11	35	1800	65	M	3	3	no	vertical	vertical with water	left	no	0	0	42,67	no
Madrid	MUTUA MADRILEÑA TROPHY GRAND PRIX CSI*5 W round B	25.11.2017	CSI-W5*	3	1.60	300	28	12	26.03.2006	M	ZANG	193	9	23	11	35	1800	65	M	4	4a	yes	vertical	vertical	left	no	0	0	42,67	no
Madrid	MUTUA MADRILEÑA TROPHY GRAND PRIX CSI*5 W round B	25.11.2017	CSI-W5*	3	1.60	300	28	12	26.03.2006	M	ZANG	193	9	23	11	35	1800	65	M	5	4b	yes	spread	oxer with water	left	no	0	0	42,67	no
Madrid	MUTUA MADRILEÑA TROPHY GRAND PRIX CSI*5 W round B	25.11.2017	CSI-W5*	3	1.60	300	28	12	26.03.2006	M	ZANG	193	9	23	11	35	1800	65	M	6	5	no	spread	oxer	right	no	0	0	42,67	no
Madrid	MUTUA MADRILEÑA TROPHY GRAND PRIX CSI*5 W round B	25.11.2017	CSI-W5*	3	1.60	300	28	12	26.03.2006	M	ZANG	193	9	23	11	35	1800	65	M	7	6	no	vertical	vertical	right	no	0	0	42,67	no
Madrid	MUTUA MADRILEÑA TROPHY GRAND PRIX CSI*5 W round B	25.11.2017	CSI-W5*	3	1.60	300	28	12	26.03.2006	M	ZANG	193	9	23	11	35	1800	65	M	8	7	no	vertical	vertical	left	no	0	0	42,67	no
Madrid	MUTUA MADRILEÑA TROPHY GRAND PRIX CSI*5 W round B	25.11.2017	CSI-W5*	3	1.60	300	28	12	26.03.2006	M	ZANG	193	9	23	11	35	1800	65	M	9	8	no	spread	oxer	left	no	0	0	42,67	no
Madrid	MUTUA MADRILEÑA TROPHY GRAND PRIX CSI*5 W round A	25.11.2017	CSI-W5*	3	1.60	430	127	10	03.07.2008	M	BWP	131	3	26	14	30	1957	97	M	1	1	no	spread	oxer	left	no	0	0	72	no
Madrid	MUTUA MADRILEÑA TROPHY GRAND PRIX CSI*5 W round A	25.11.2017	CSI-W5*	3	1.60	430	127	10	03.07.2008	M	BWP	131	3	26	14	30	1957	97	M	2	2	no	vertical	vertical	straight	no	0	0	72	no
Madrid	MUTUA MADRILEÑA TROPHY GRAND PRIX CSI*5 W round A	25.11.2017	CSI-W5*	3	1.60	430	127	10	03.07.2008	M	BWP	131	3	26	14	30	1957	97	M	3	3	no	spread	oxer	right	no	0	0	72	no
Madrid	MUTUA MADRILEÑA TROPHY GRAND PRIX CSI*5 W round A	25.11.2017	CSI-W5*	3	1.60	430	127	10	03.07.2008	M	BWP	131	3	26	14	30	1957	97	M	4	4	no	vertical	vertical	straight	no	0	0	72	no
Madrid	MUTUA MADRILEÑA TROPHY GRAND PRIX CSI*5 W round A	25.11.2017	CSI-W5*	3	1.60	430	127	10	03.07.2008	M	BWP	131	3	26	14	30	1957	97	M	5	5	no	spread	oxer	left	no	0	0	72	no
Madrid	MUTUA MADRILEÑA TROPHY GRAND PRIX CSI*5 W round A	25.11.2017	CSI-W5*	3	1.60	430	127	10	03.07.2008	M	BWP	131	3	26	14	30	1957	97	M	6	6	no	vertical	vertical	left	no	0	0	72	no
Madrid	MUTUA MADRILEÑA TROPHY GRAND PRIX CSI*5 W round A	25.11.2017	CSI-W5*	3	1.60	430	127	10	03.07.2008	M	BWP	131	3	26	14	30	1957	97	M	7	7a	yes	spread	oxer	right	no	0	0	72	no
Madrid	MUTUA MADRILEÑA TROPHY GRAND PRIX CSI*5 W round A	25.11.2017	CSI-W5*	3	1.60	430	127	10	03.07.2008	M	BWP	131	3	26	14	30	1957	97	M	8	7b	yes	vertical	vertical	right	no	0	0	72	no
Madrid	MUTUA MADRILEÑA TROPHY GRAND PRIX CSI*5 W round A	25.11.2017	CSI-W5*	3	1.60	430	127	10	03.07.2008	M	BWP	131	3	26	14	30	1957	97	M	9	8	no	spread	oxer	right	no	0	0	72	no
Madrid	MUTUA MADRILEÑA TROPHY GRAND PRIX CSI*5 W round A	25.11.2017	CSI-W5*	3	1.60	430	127	10	03.07.2008	M	BWP	131	3	26	14	30	1957	97	M	10	9	no	vertical	vertical	right	no	0	0	72	no
Madrid	MUTUA MADRILEÑA TROPHY GRAND PRIX CSI*5 W round A	25.11.2017	CSI-W5*	3	1.60	430	127	10	03.07.2008	M	BWP	131	3	26	14	30	1957	97	M	11	10	no	spread	oxer	straight	no	0	0	72	no
Madrid	MUTUA MADRILEÑA TROPHY GRAND PRIX CSI*5 W round A	25.11.2017	CSI-W5*	3	1.60	430	127	10	03.07.2008	M	BWP	131	3	26	14	30	1957	97	M	12	11	no	vertical	vertical with water	left	yes	4	0	72	no
Madrid	MUTUA MADRILEÑA TROPHY GRAND PRIX CSI*5 W round A	25.11.2017	CSI-W5*	3	1.60	430	127	10	03.07.2008	M	BWP	131	3	26	14	30	1957	97	M	13	12a	yes	vertical	vertical	left	no	0	0	72	no
Madrid	MUTUA MADRILEÑA TROPHY GRAND PRIX CSI*5 W round A	25.11.2017	CSI-W5*	3	1.60	430	127	10	03.07.2008	M	BWP	131	3	26	14	30	1957	97	M	14	12b	yes	spread	oxer with water	left	no	0	0	72	no
Madrid	MUTUA MADRILEÑA TROPHY GRAND PRIX CSI*5 W round A	25.11.2017	CSI-W5*	3	1.60	430	127	10	03.07.2008	M	BWP	131	3	26	14	30	1957	97	M	15	12c	yes	vertical	vertical	left	no	0	0	72	no
Madrid	MUTUA MADRILEÑA TROPHY GRAND PRIX CSI*5 W round A	25.11.2017	CSI-W5*	3	1.60	430	127	10	03.07.2008	M	BWP	131	3	26	14	30	1957	97	M	16	13	no	spread	oxer	right	no	0	0	72	no
Madrid	MUTUA MADRILEÑA TROPHY GRAND PRIX CSI*5 W round A	25.11.2017	CSI-W5*	3	1.60	430	92	13	20.03.2005	G	BWP	140	4	28	4	38	2170	90	M	1	1	no	spread	oxer	left	no	0	0	94,42	no
Madrid	MUTUA MADRILEÑA TROPHY GRAND PRIX CSI*5 W round A	25.11.2017	CSI-W5*	3	1.60	430	92	13	20.03.2005	G	BWP	140	4	28	4	38	2170	90	M	2	2	no	vertical	vertical	straight	no	0	0	94,42	no
Madrid	MUTUA MADRILEÑA TROPHY GRAND PRIX CSI*5 W round A	25.11.2017	CSI-W5*	3	1.60	430	92	13	20.03.2005	G	BWP	140	4	28	4	38	2170	90	M	3	3	no	spread	oxer	right	yes	4	0	94,42	no
Madrid	MUTUA MADRILEÑA TROPHY GRAND PRIX CSI*5 W round A	25.11.2017	CSI-W5*	3	1.60	430	92	13	20.03.2005	G	BWP	140	4	28	4	38	2170	90	M	4	4	no	vertical	vertical	straight	no	0	0	94,42	no
Madrid	MUTUA MADRILEÑA TROPHY GRAND PRIX CSI*5 W round A	25.11.2017	CSI-W5*	3	1.60	430	92	13	20.03.2005	G	BWP	140	4	28	4	38	2170	90	M	5	5	no	spread	oxer	left	no	0	0	94,42	no
Madrid	MUTUA MADRILEÑA TROPHY GRAND PRIX CSI*5 W round A	25.11.2017	CSI-W5*	3	1.60	430	92	13	20.03.2005	G	BWP	140	4	28	4	38	2170	90	M	6	6	no	vertical	vertical	left	no	0	0	94,42	no
Madrid	MUTUA MADRILEÑA TROPHY GRAND PRIX CSI*5 W round A	25.11.2017	CSI-W5*	3	1.60	430	92	13	20.03.2005	G	BWP	140	4	28	4	38	2170	90	M	7	7a	yes	spread	oxer	right	no	0	0	94,42	no
Madrid	MUTUA MADRILEÑA TROPHY GRAND PRIX CSI*5 W round A	25.11.2017	CSI-W5*	3	1.60	430	92	13	20.03.2005	G	BWP	140	4	28	4	38	2170	90	M	8	7b	yes	vertical	vertical	right	no	0	0	94,42	no
Madrid	MUTUA MADRILEÑA TROPHY GRAND PRIX CSI*5 W round A	25.11.2017	CSI-W5*	3	1.60	430	92	13	20.03.2005	G	BWP	140	4	28	4	38	2170	90	M	9	8	no	spread	oxer	right	no	0	0	94,42	no
Madrid	MUTUA MADRILEÑA TROPHY GRAND PRIX CSI*5 W round A	25.11.2017	CSI-W5*	3	1.60	430	92	13	20.03.2005	G	BWP	140	4	28	4	38	2170	90	M	10	9	no	vertical	vertical	right	no	0	0	94,42	no
Madrid	MUTUA MADRILEÑA TROPHY GRAND PRIX CSI*5 W round A	25.11.2017	CSI-W5*	3	1.60	430	92	13	20.03.2005	G	BWP	140	4	28	4	38	2170	90	M	11	10	no	spread	oxer	straight	no	0	0	94,42	no
Madrid	MUTUA MADRILEÑA TROPHY GRAND PRIX CSI*5 W round A	25.11.2017	CSI-W5*	3	1.60	430	92	13	20.03.2005	G	BWP	140	4	28	4	38	2170	90	M	12	11	no	vertical	vertical with water	left	yes	4	0	94,42	no
Madrid	MUTUA MADRILEÑA TROPHY GRAND PRIX CSI*5 W round A	25.11.2017	CSI-W5*	3	1.60	430	92	13	20.03.2005	G	BWP	140	4	28	4	38	2170	90	M	13	12a	yes	vertical	vertical	left	no	0	0	94,42	no
Madrid	MUTUA MADRILEÑA TROPHY GRAND PRIX CSI*5 W round A	25.11.2017	CSI-W5*	3	1.60	430	92	13	20.03.2005	G	BWP	140	4	28	4	38	2170	90	M	14	12b	yes	spread	oxer with water	left	no	0	0	94,42	no
Madrid	MUTUA MADRILEÑA TROPHY GRAND PRIX CSI*5 W round A	25.11.2017	CSI-W5*	3	1.60	430	92	13	20.03.2005	G	BWP	140	4	28	4	38	2170	90	M	15	12c	yes	vertical	vertical	left	no	0	0	94,42	no
Madrid	MUTUA MADRILEÑA TROPHY GRAND PRIX CSI*5 W round A	25.11.2017	CSI-W5*	3	1.60	430	92	13	20.03.2005	G	BWP	140	4	28	4	38	2170	90	M	16	13	no	spread	oxer	right	no	0	0	94,42	no
Madrid	MUTUA MADRILEÑA TROPHY GRAND PRIX CSI*5 W round A	25.11.2017	CSI-W5*	3	1.60	430	15	12	26.07.2006	M	SWB	159	0	30	9	36	2556	124	M	1	1	no	spread	oxer	left	no	0	0	72,45	no
Madrid	MUTUA MADRILEÑA TROPHY GRAND PRIX CSI*5 W round A	25.11.2017	CSI-W5*	3	1.60	430	15	12	26.07.2006	M	SWB	159	0	30	9	36	2556	124	M	2	2	no	vertical	vertical	straight	yes	4	0	72,45	no
Madrid	MUTUA MADRILEÑA TROPHY GRAND PRIX CSI*5 W round A	25.11.2017	CSI-W5*	3	1.60	430	15	12	26.07.2006	M	SWB	159	0	30	9	36	2556	124	M	3	3	no	spread	oxer	right	no	0	0	72,45	no
Madrid	MUTUA MADRILEÑA TROPHY GRAND PRIX CSI*5 W round A	25.11.2017	CSI-W5*	3	1.60	430	15	12	26.07.2006	M	SWB	159	0	30	9	36	2556	124	M	4	4	no	vertical	vertical	straight	no	0	0	72,45	no
Madrid	MUTUA MADRILEÑA TROPHY GRAND PRIX CSI*5 W round A	25.11.2017	CSI-W5*	3	1.60	430	15	12	26.07.2006	M	SWB	159	0	30	9	36	2556	124	M	5	5	no	spread	oxer	left	no	0	0	72,45	no
Madrid	MUTUA MADRILEÑA TROPHY GRAND PRIX CSI*5 W round A	25.11.2017	CSI-W5*	3	1.60	430	15	12	26.07.2006	M	SWB	159	0	30	9	36	2556	124	M	6	6	no	vertical	vertical	left	no	0	0	72,45	no
Madrid	MUTUA MADRILEÑA TROPHY GRAND PRIX CSI*5 W round A	25.11.2017	CSI-W5*	3	1.60	430	15	12	26.07.2006	M	SWB	159	0	30	9	36	2556	124	M	7	7a	yes	spread	oxer	right	no	0	0	72,45	no
Madrid	MUTUA MADRILEÑA TROPHY GRAND PRIX CSI*5 W round A	25.11.2017	CSI-W5*	3	1.60	430	15	12	26.07.2006	M	SWB	159	0	30	9	36	2556	124	M	8	7b	yes	vertical	vertical	right	no	0	0	72,45	no
Madrid	MUTUA MADRILEÑA TROPHY GRAND PRIX CSI*5 W round A	25.11.2017	CSI-W5*	3	1.60	430	15	12	26.07.2006	M	SWB	159	0	30	9	36	2556	124	M	9	8	no	spread	oxer	right	no	0	0	72,45	no
Madrid	MUTUA MADRILEÑA TROPHY GRAND PRIX CSI*5 W round A	25.11.2017	CSI-W5*	3	1.60	430	15	12	26.07.2006	M	SWB	159	0	30	9	36	2556	124	M	10	9	no	vertical	vertical	right	no	0	0	72,45	no
Madrid	MUTUA MADRILEÑA TROPHY GRAND PRIX CSI*5 W round A	25.11.2017	CSI-W5*	3	1.60	430	15	12	26.07.2006	M	SWB	159	0	30	9	36	2556	124	M	11	10	no	spread	oxer	straight	no	0	0	72,45	no
Madrid	MUTUA MADRILEÑA TROPHY GRAND PRIX CSI*5 W round A	25.11.2017	CSI-W5*	3	1.60	430	15	12	26.07.2006	M	SWB	159	0	30	9	36	2556	124	M	12	11	no	vertical	vertical with water	left	no	0	0	72,45	no
Madrid	MUTUA MADRILEÑA TROPHY GRAND PRIX CSI*5 W round A	25.11.2017	CSI-W5*	3	1.60	430	15	12	26.07.2006	M	SWB	159	0	30	9	36	2556	124	M	13	12a	yes	vertical	vertical	left	no	0	0	72,45	no
Madrid	MUTUA MADRILEÑA TROPHY GRAND PRIX CSI*5 W round A	25.11.2017	CSI-W5*	3	1.60	430	15	12	26.07.2006	M	SWB	159	0	30	9	36	2556	124	M	14	12b	yes	spread	oxer with water	left	no	0	0	72,45	no
Madrid	MUTUA MADRILEÑA TROPHY GRAND PRIX CSI*5 W round A	25.11.2017	CSI-W5*	3	1.60	430	15	12	26.07.2006	M	SWB	159	0	30	9	36	2556	124	M	15	12c	yes	vertical	vertical	left	no	0	0	72,45	no
Madrid	MUTUA MADRILEÑA TROPHY GRAND PRIX CSI*5 W round A	25.11.2017	CSI-W5*	3	1.60	430	15	12	26.07.2006	M	SWB	159	0	30	9	36	2556	124	M	16	13	no	spread	oxer	right	no	0	0	72,45	no
Madrid	MUTUA MADRILEÑA TROPHY GRAND PRIX CSI*5 W round A	25.11.2017	CSI-W5*	3	1.60	430	67	10	04.05.2008	M	KWPN	87	1	38	10	29	1642	46	M	1	1	no	spread	oxer	left	no	0	0	74,11	no
Madrid	MUTUA MADRILEÑA TROPHY GRAND PRIX CSI*5 W round A	25.11.2017	CSI-W5*	3	1.60	430	67	10	04.05.2008	M	KWPN	87	1	38	10	29	1642	46	M	2	2	no	vertical	vertical	straight	no	0	0	74,11	no
Madrid	MUTUA MADRILEÑA TROPHY GRAND PRIX CSI*5 W round A	25.11.2017	CSI-W5*	3	1.60	430	67	10	04.05.2008	M	KWPN	87	1	38	10	29	1642	46	M	3	3	no	spread	oxer	right	no	0	0	74,11	no
Madrid	MUTUA MADRILEÑA TROPHY GRAND PRIX CSI*5 W round A	25.11.2017	CSI-W5*	3	1.60	430	67	10	04.05.2008	M	KWPN	87	1	38	10	29	1642	46	M	4	4	no	vertical	vertical	straight	no	0	0	74,11	no
Madrid	MUTUA MADRILEÑA TROPHY GRAND PRIX CSI*5 W round A	25.11.2017	CSI-W5*	3	1.60	430	67	10	04.05.2008	M	KWPN	87	1	38	10	29	1642	46	M	5	5	no	spread	oxer	left	no	0	0	74,11	no
Madrid	MUTUA MADRILEÑA TROPHY GRAND PRIX CSI*5 W round A	25.11.2017	CSI-W5*	3	1.60	430	67	10	04.05.2008	M	KWPN	87	1	38	10	29	1642	46	M	6	6	no	vertical	vertical	left	no	0	0	74,11	no
Madrid	MUTUA MADRILEÑA TROPHY GRAND PRIX CSI*5 W round A	25.11.2017	CSI-W5*	3	1.60	430	67	10	04.05.2008	M	KWPN	87	1	38	10	29	1642	46	M	7	7a	yes	spread	oxer	right	no	0	0	74,11	no
Madrid	MUTUA MADRILEÑA TROPHY GRAND PRIX CSI*5 W round A	25.11.2017	CSI-W5*	3	1.60	430	67	10	04.05.2008	M	KWPN	87	1	38	10	29	1642	46	M	8	7b	yes	vertical	vertical	right	yes	4	0	74,11	no

Madrid	MUTUA MADRILEÑA TROPHY GRAND PRIX CSI*5 W round A	25.11.2017	CSI-W5*	3	1.60	430	67	10	04.05.2008	M	KWPN	87	1	38	10	29	1642	46	M	9	8	no	spread	oxer	right	yes	4	0	74,11	no
Madrid	MUTUA MADRILEÑA TROPHY GRAND PRIX CSI*5 W round A	25.11.2017	CSI-W5*	3	1.60	430	67	10	04.05.2008	M	KWPN	87	1	38	10	29	1642	46	M	10	9	no	vertical	vertical	right	no	0	0	74,11	no
Madrid	MUTUA MADRILEÑA TROPHY GRAND PRIX CSI*5 W round A	25.11.2017	CSI-W5*	3	1.60	430	67	10	04.05.2008	M	KWPN	87	1	38	10	29	1642	46	M	11	10	no	spread	oxer	straight	no	0	0	74,11	no
Madrid	MUTUA MADRILEÑA TROPHY GRAND PRIX CSI*5 W round A	25.11.2017	CSI-W5*	3	1.60	430	67	10	04.05.2008	M	KWPN	87	1	38	10	29	1642	46	M	12	11	no	vertical	vertical with water	left	yes	4	0	74,11	no
Madrid	MUTUA MADRILEÑA TROPHY GRAND PRIX CSI*5 W round A	25.11.2017	CSI-W5*	3	1.60	430	67	10	04.05.2008	M	KWPN	87	1	38	10	29	1642	46	M	13	12a	yes	vertical	vertical	left	no	0	0	74,11	no
Madrid	MUTUA MADRILEÑA TROPHY GRAND PRIX CSI*5 W round A	25.11.2017	CSI-W5*	3	1.60	430	67	10	04.05.2008	M	KWPN	87	1	38	10	29	1642	46	M	14	12b	yes	spread	oxer with water	left	no	0	0	74,11	no
Madrid	MUTUA MADRILEÑA TROPHY GRAND PRIX CSI*5 W round A	25.11.2017	CSI-W5*	3	1.60	430	67	10	04.05.2008	M	KWPN	87	1	38	10	29	1642	46	M	15	12c	yes	vertical	vertical	left	no	0	0	74,11	no
Madrid	MUTUA MADRILEÑA TROPHY GRAND PRIX CSI*5 W round A	25.11.2017	CSI-W5*	3	1.60	430	67	10	04.05.2008	M	KWPN	87	1	38	10	29	1642	46	M	16	13	no	spread	oxer	right	no	0	1	74,11	no
Madrid	MUTUA MADRILEÑA TROPHY GRAND PRIX CSI*5 W round A	25.11.2017	CSI-W5*	3	1.60	430	48	17	01.01.2001	G	WESTF	290	9	39	71	51	2525	123	M	1	1	no	spread	oxer	left	no	0	0	70,64	no
Madrid	MUTUA MADRILEÑA TROPHY GRAND PRIX CSI*5 W round A	25.11.2017	CSI-W5*	3	1.60	430	48	17	01.01.2001	G	WESTF	290	9	39	71	51	2525	123	M	2	2	no	vertical	vertical	straight	no	0	0	70,64	no
Madrid	MUTUA MADRILEÑA TROPHY GRAND PRIX CSI*5 W round A	25.11.2017	CSI-W5*	3	1.60	430	48	17	01.01.2001	G	WESTF	290	9	39	71	51	2525	123	M	3	3	no	spread	oxer	right	no	0	0	70,64	no
Madrid	MUTUA MADRILEÑA TROPHY GRAND PRIX CSI*5 W round A	25.11.2017	CSI-W5*	3	1.60	430	48	17	01.01.2001	G	WESTF	290	9	39	71	51	2525	123	M	4	4	no	vertical	vertical	straight	no	0	0	70,64	no
Madrid	MUTUA MADRILEÑA TROPHY GRAND PRIX CSI*5 W round A	25.11.2017	CSI-W5*	3	1.60	430	48	17	01.01.2001	G	WESTF	290	9	39	71	51	2525	123	M	5	5	no	spread	oxer	left	no	0	0	70,64	no
Madrid	MUTUA MADRILEÑA TROPHY GRAND PRIX CSI*5 W round A	25.11.2017	CSI-W5*	3	1.60	430	48	17	01.01.2001	G	WESTF	290	9	39	71	51	2525	123	M	6	6	no	vertical	vertical	left	no	0	0	70,64	no
Madrid	MUTUA MADRILEÑA TROPHY GRAND PRIX CSI*5 W round A	25.11.2017	CSI-W5*	3	1.60	430	48	17	01.01.2001	G	WESTF	290	9	39	71	51	2525	123	M	7	7a	yes	spread	oxer	right	no	0	0	70,64	no
Madrid	MUTUA MADRILEÑA TROPHY GRAND PRIX CSI*5 W round A	25.11.2017	CSI-W5*	3	1.60	430	48	17	01.01.2001	G	WESTF	290	9	39	71	51	2525	123	M	8	7b	yes	vertical	vertical	right	no	0	0	70,64	no
Madrid	MUTUA MADRILEÑA TROPHY GRAND PRIX CSI*5 W round A	25.11.2017	CSI-W5*	3	1.60	430	48	17	01.01.2001	G	WESTF	290	9	39	71	51	2525	123	M	9	8	no	spread	oxer	right	no	0	0	70,64	no
Madrid	MUTUA MADRILEÑA TROPHY GRAND PRIX CSI*5 W round A	25.11.2017	CSI-W5*	3	1.60	430	48	17	01.01.2001	G	WESTF	290	9	39	71	51	2525	123	M	10	9	no	vertical	vertical	right	no	0	0	70,64	no
Madrid	MUTUA MADRILEÑA TROPHY GRAND PRIX CSI*5 W round A	25.11.2017	CSI-W5*	3	1.60	430	48	17	01.01.2001	G	WESTF	290	9	39	71	51	2525	123	M	11	10	no	spread	oxer	straight	no	0	0	70,64	no
Madrid	MUTUA MADRILEÑA TROPHY GRAND PRIX CSI*5 W round A	25.11.2017	CSI-W5*	3	1.60	430	48	17	01.01.2001	G	WESTF	290	9	39	71	51	2525	123	M	12	11	no	vertical	vertical with water	left	no	0	0	70,64	no
Madrid	MUTUA MADRILEÑA TROPHY GRAND PRIX CSI*5 W round A	25.11.2017	CSI-W5*	3	1.60	430	48	17	01.01.2001	G	WESTF	290	9	39	71	51	2525	123	M	13	12a	yes	vertical	vertical	left	no	0	0	70,64	no
Madrid	MUTUA MADRILEÑA TROPHY GRAND PRIX CSI*5 W round A	25.11.2017	CSI-W5*	3	1.60	430	48	17	01.01.2001	G	WESTF	290	9	39	71	51	2525	123	M	14	12b	yes	spread	oxer with water	left	no	0	0	70,64	no
Madrid	MUTUA MADRILEÑA TROPHY GRAND PRIX CSI*5 W round A	25.11.2017	CSI-W5*	3	1.60	430	48	17	01.01.2001	G	WESTF	290	9	39	71	51	2525	123	M	15	12c	yes	vertical	vertical	left	no	0	0	70,64	no
Madrid	MUTUA MADRILEÑA TROPHY GRAND PRIX CSI*5 W round A	25.11.2017	CSI-W5*	3	1.60	430	48	17	01.01.2001	G	WESTF	290	9	39	71	51	2525	123	M	16	13	no	spread	oxer	right	no	0	0	70,64	no
Madrid	MUTUA MADRILEÑA TROPHY GRAND PRIX CSI*5 W round B	25.11.2017	CSI-W5*	3	1.60	300	48	17	01.01.2001	G	WESTF	290	9	39	71	51	2525	123	M	1	1	no	spread	oxer	right	no	0	0	44,07	no
Madrid	MUTUA MADRILEÑA TROPHY GRAND PRIX CSI*5 W round B	25.11.2017	CSI-W5*	3	1.60	300	48	17	01.01.2001	G	WESTF	290	9	39	71	51	2525	123	M	2	2	no	spread	oxer	right	no	0	0	44,07	no
Madrid	MUTUA MADRILEÑA TROPHY GRAND PRIX CSI*5 W round B	25.11.2017	CSI-W5*	3	1.60	300	48	17	01.01.2001	G	WESTF	290	9	39	71	51	2525	123	M	3	3	no	vertical	vertical with water	left	no	0	0	44,07	no
Madrid	MUTUA MADRILEÑA TROPHY GRAND PRIX CSI*5 W round B	25.11.2017	CSI-W5*	3	1.60	300	48	17	01.01.2001	G	WESTF	290	9	39	71	51	2525	123	M	4	4a	yes	vertical	vertical	left	no	0	0	44,07	no
Madrid	MUTUA MADRILEÑA TROPHY GRAND PRIX CSI*5 W round B	25.11.2017	CSI-W5*	3	1.60	300	48	17	01.01.2001	G	WESTF	290	9	39	71	51	2525	123	M	5	4b	yes	spread	oxer with water	left	no	0	0	44,07	no
Madrid	MUTUA MADRILEÑA TROPHY GRAND PRIX CSI*5 W round B	25.11.2017	CSI-W5*	3	1.60	300	48	17	01.01.2001	G	WESTF	290	9	39	71	51	2525	123	M	6	5	no	spread	oxer	right	no	0	0	44,07	no
Madrid	MUTUA MADRILEÑA TROPHY GRAND PRIX CSI*5 W round B	25.11.2017	CSI-W5*	3	1.60	300	48	17	01.01.2001	G	WESTF	290	9	39	71	51	2525	123	M	7	6	no	vertical	vertical	right	no	0	0	44,07	no
Madrid	MUTUA MADRILEÑA TROPHY GRAND PRIX CSI*5 W round B	25.11.2017	CSI-W5*	3	1.60	300	48	17	01.01.2001	G	WESTF	290	9	39	71	51	2525	123	M	8	7	no	vertical	vertical	left	no	0	0	44,07	no
Madrid	MUTUA MADRILEÑA TROPHY GRAND PRIX CSI*5 W round B	25.11.2017	CSI-W5*	3	1.60	300	48	17	01.01.2001	G	WESTF	290	9	39	71	51	2525	123	M	9	8	no	spread	oxer	left	no	0	0	44,07	no
Madrid	MUTUA MADRILEÑA TROPHY GRAND PRIX CSI*5 W round A	25.11.2017	CSI-W5*	3	1.60	430	216	13	21.06.2005	G	SBS	196	3	42	542	26	1197	49	F	1	1	no	spread	oxer	left	no	0	0	0,00	yes
Madrid	MUTUA MADRILEÑA TROPHY GRAND PRIX CSI*5 W round A	25.11.2017	CSI-W5*	3	1.60	430	216	13	21.06.2005	G	SBS	196	3	42	542	26	1197	49	F	2	2	no	vertical	vertical	straight	no	0	0	0,00	yes
Madrid	MUTUA MADRILEÑA TROPHY GRAND PRIX CSI*5 W round A	25.11.2017	CSI-W5*	3	1.60	430	216	13	21.06.2005	G	SBS	196	3	42	542	26	1197	49	F	3	3	no	spread	oxer	right	no	0	0	0,00	yes
Madrid	MUTUA MADRILEÑA TROPHY GRAND PRIX CSI*5 W round A	25.11.2017	CSI-W5*	3	1.60	430	216	13	21.06.2005	G	SBS	196	3	42	542	26	1197	49	F	4	4	no	vertical	vertical	straight	no	0	0	0,00	yes
Madrid	MUTUA MADRILEÑA TROPHY GRAND PRIX CSI*5 W round A	25.11.2017	CSI-W5*	3	1.60	430	216	13	21.06.2005	G	SBS	196	3	42	542	26	1197	49	F	5	5	no	spread	oxer	left	no	0	0	0,00	yes
Madrid	MUTUA MADRILEÑA TROPHY GRAND PRIX CSI*5 W round A	25.11.2017	CSI-W5*	3	1.60	430	216	13	21.06.2005	G	SBS	196	3	42	542	26	1197	49	F	6	6	no	vertical	vertical	left	yes	4	0	0,00	yes
Madrid	MUTUA MADRILEÑA TROPHY GRAND PRIX CSI*5 W round A	25.11.2017	CSI-W5*	3	1.60	430	216	13	21.06.2005	G	SBS	196	3	42	542	26	1197	49	F	7	7a	yes	spread	oxer	right	no	0	0	0,00	yes
Madrid	MUTUA MADRILEÑA TROPHY GRAND PRIX CSI*5 W round A	25.11.2017	CSI-W5*	3	1.60	430	216	13	21.06.2005	G	SBS	196	3	42	542	26	1197	49	F	8	7b	yes	vertical	vertical	right	no	0	0	0,00	yes
Madrid	MUTUA MADRILEÑA TROPHY GRAND PRIX CSI*5 W round A	25.11.2017	CSI-W5*	3	1.60	430	216	13	21.06.2005	G	SBS	196	3	42	542	26	1197	49	F	9	8	no	spread	oxer	right	no	0	0	0,00	yes
Madrid	MUTUA MADRILEÑA TROPHY GRAND PRIX CSI*5 W round A	25.11.2017	CSI-W5*	3	1.60	430	216	13	21.06.2005	G	SBS	196	3	42	542	26	1197	49	F	10	9	no	vertical	vertical	right	yes	4	0	0,00	yes
Madrid	MUTUA MADRILEÑA TROPHY GRAND PRIX CSI*5 W round A	25.11.2017	CSI-W5*	3	1.60	430	216	13	21.06.2005	G	SBS	196	3	42	542	26	1197	49	F	11	10	no	spread	oxer	straight	yes	4	0	0,00	yes
Madrid	MUTUA MADRILEÑA TROPHY GRAND PRIX CSI*5 W round A	25.11.2017	CSI-W5*	3	1.60	430	216	13	21.06.2005	G	SBS	196	3	42	542	26	1197	49	F	12	11	no	vertical	vertical with water	left	yes	4	0	0,00	yes
Madrid	MUTUA MADRILEÑA TROPHY GRAND PRIX CSI*5 W round A	25.11.2017	CSI-W5*	3	1.60	430	216	13	21.06.2005	G	SBS	196	3	42	542	26	1197	49	F	13	12a	yes	vertical	vertical	left	yes	4	0	0,00	yes
Madrid	MUTUA MADRILEÑA TROPHY GRAND PRIX CSI*5 W round A	25.11.2017	CSI-W5*	3	1.60	430	216	13	21.06.2005	G	SBS	196	3	42	542	26	1197	49	F	14	12b	yes	spread	oxer with water	left	no	0	0	0,00	yes
Madrid	MUTUA MADRILEÑA TROPHY GRAND PRIX CSI*5 W round A	25.11.2017	CSI-W5*	3	1.60	430	216	13	21.06.2005	G	SBS	196	3	42	542	26	1197	49	F	15	12c	yes	vertical	vertical	left	yes	4	0	0,00	yes
Madrid	MUTUA MADRILEÑA TROPHY GRAND PRIX CSI*5 W round A	25.11.2017	CSI-W5*	3	1.60	430	216	13	21.06.2005	G	SBS	196	3	42	542	26	1197	49	F	16	13	no	spread	oxer	right	no	0	0	0,00	yes
Madrid	MUTUA MADRILEÑA TROPHY GRAND PRIX CSI*5 W round A	25.11.2017	CSI-W5*	3	1.60	430	11	12	13.03.2006	M	KWPN	211	7	48	40	39	1040	33	M	1	1	no	spread	oxer	left	no	0	0	71,87	no
Madrid	MUTUA MADRILEÑA TROPHY GRAND PRIX CSI*5 W round A	25.11.2017	CSI-W5*	3	1.60	430	11	12	13.03.2006	M	KWPN	211	7	48	40	39	1040	33	M	2	2	no	vertical	vertical	straight	no	0	0	71,87	no
Madrid	MUTUA MADRILEÑA TROPHY GRAND PRIX CSI*5 W round A	25.11.2017	CSI-W5*	3	1.60	430	11	12	13.03.2006	M	KWPN	211	7	48	40	39	1040	33	M	3	3	no	spread	oxer	right	no	0	0	71,87	no
Madrid	MUTUA MADRILEÑA TROPHY GRAND PRIX CSI*5 W round A	25.11.2017	CSI-W5*	3	1.60	430	11	12	13.03.2006	M	KWPN	211	7	48	40	39	1040	33	M	4	4	no	vertical	vertical	straight	no	0	0	71,87	no
Madrid	MUTUA MADRILEÑA TROPHY GRAND PRIX CSI*5 W round A	25.11.2017	CSI-W5*	3	1.60	430	11	12	13.03.2006	M	KWPN	211	7	48	40	39	1040	33	M	5	5	no	spread	oxer	left	no	0	0	71,87	no
Madrid	MUTUA MADRILEÑA TROPHY GRAND PRIX CSI*5 W round A	25.11.2017	CSI-W5*	3	1.60	430	11	12	13.03.2006	M	KWPN	211	7	48	40	39	1040	33	M	6	6	no	vertical	vertical	left	no	0	0	71,87	no
Madrid	MUTUA MADRILEÑA TROPHY GRAND PRIX CSI*5 W round A	25.11.2017	CSI-W5*	3	1.60	430	11	12	13.03.2006	M	KWPN	211	7	48	40	39	1040	33	M	7	7a	yes	spread	oxer	right	no	0	0	71,87	no
Madrid	MUTUA MADRILEÑA TROPHY GRAND PRIX CSI*5 W round A	25.11.2017	CSI-W5*	3	1.60	430	11	12	13.03.2006	M	KWPN	211	7	48	40	39	1040	33	M	8	7b	yes	vertical	vertical	right	no	0	0	71,87	no
Madrid	MUTUA MADRILEÑA TROPHY GRAND PRIX CSI*5 W round A	25.11.2017	CSI-W5*	3	1.60	430	11	12	13.03.2006	M	KWPN	211	7	48	40	39	1040	33	M	9	8	no	spread	oxer	right	no	0	0	71,87	no
Madrid	MUTUA MADRILEÑA TROPHY GRAND PRIX CSI*5 W round A	25.11.2017	CSI-W5*	3	1.60	430	11	12	13.03.2006	M	KWPN	211	7	48	40	39	1040	33	M	10	9	no	vertical	vertical	right	no	0	0	71,87	no
Madrid	MUTUA MADRILEÑA TROPHY GRAND PRIX CSI*5 W round A	25.11.2017	CSI-W5*	3	1.60	430	11	12	13.03.2006	M	KWPN	211	7	48	40	39	1040	33	M	11	10	no	spread	oxer	straight	no	0	0	71,87	no
Madrid	MUTUA MADRILEÑA TROPHY GRAND PRIX CSI*5 W round A	25.11.2017	CSI-W5*	3	1.60	430	11	12	13.03.2006	M	KWPN	211	7	48	40	39	1040	33	M	12	11	no	vertical	vertical with water	left	no	0	0	71,87	no
Madrid	MUTUA MADRILEÑA TROPHY GRAND PRIX CSI*5 W round A	25.11.2017	CSI-W5*	3	1.60	430	11	12	13.03.2006	M	KWPN	211	7	48	40	39	1040	33	M	13	12a	yes	vertical	vertical	left	no	0	0	71,87	no
Madrid	MUTUA MADRILEÑA TROPHY GRAND PRIX CSI*5 W round A	25.11.2017	CSI-W5*	3	1.60	430	11	12	13.03.2006	M	KWPN	211	7	48	40	39	1040	33	M	14	12b	yes	spread	oxer with water	left	no	0	0	71,87	no
Madrid	MUTUA MADRILEÑA TROPHY GRAND PRIX CSI*5 W round A	25.11.2017	CSI-W5*	3	1.60	430	11	12	13.03.2006	M	KWPN	211	7	48	40	39	1040	33	M	15	12c	yes	vertical	vertical	left	no	0	0	71,87	no
Madrid	MUTUA MADRILEÑA TROPHY GRAND PRIX CSI*5 W round A	25.11.2017	CSI-W5*	3	1.60	430	11	12	13.03.2006	M	KWPN	211	7	48	40	39	1040	33	M	16	13	no	spread	oxer	right	no	0	0	71,87	no
Madrid	MUTUA MADRILEÑA TROPHY GRAND PRIX CSI*5 W round B	25.11.2017	CSI-W5*	3	1.60	300	11	12	13.03.2006	M	KWPN	211	7	48	40	39	1040	33	M	1	1	no	spread	oxer	right	no	0	0	42,84	no
Madrid	MUTUA MADRILEÑA TROPHY GRAND PRIX CSI*5 W round B	25.11.2017	CSI-W5*	3	1.60	300	11	12	13.03.2006	M	KWPN	211	7	48	40	39	1040	33	M	2	2	no	spread	oxer	right	no	0	0	42,84	no
Madrid	MUTUA MADRILEÑA TROPHY GRAND PRIX CSI*5 W round B	25.11.2017	CSI-W5*	3	1.60	300	11	12	13.03.2006	M	KWPN	211	7	48	40	39	1040	33	M	3	3	no	vertical	vertical with water	left	yes	4	0	42,84	no
Madrid	MUTUA MADRILEÑA TROPHY GRAND PRIX CSI*5 W round B	25.11.2017	CSI-W5*	3	1.60	300	11	12	13.03.2006	M	KWPN	211	7	48	40	39	1040	33	M	4	4a	yes	vertical	vertical	left	no	0	0	42,84	no
Madrid	MUTUA MADRILEÑA TROPHY GRAND PRIX CSI*5 W round B	25.11.2017	CSI-W5*	3	1.60	300	11	12	13.03.2006	M	KWPN	211	7	48	40	39	1040	33	M	5	4b	yes	spread	oxer with water	left	no	0	0	42,84	no
Madrid	MUTUA MADRILEÑA TROPHY GRAND PRIX CSI*5 W round B	25.11.2017	CSI-W5*	3	1.60	300	11	12	13.03.2006	M	KWPN	211	7	48	40	39	1040	33	M	6	5	no	spread	oxer	right	no	0	0	42,84	no
Madrid	MUTUA MADRILEÑA TROPHY GRAND PRIX CSI*5 W round B	25.11.2017	CSI-W5*	3	1.60	300	11	12	13.03.2006	M	KWPN	211	7	48	40	39	1040	33	M	7	6	no	vertical	vertical	right	no	0	0	42,84	no
Madrid	MUTUA MADRILEÑA TROPHY GRAND PRIX CSI*5 W round B	25.11.2017	CSI-W5*	3	1.60	300	11	12	13.03.2006	M	KWPN	211	7	48	40	39	1040	33	M	8	7	no	vertical	vertical	left	no	0	0	42,84	no
Madrid	MUTUA MADRILEÑA TROPHY GRAND PRIX CSI*5 W round B	25.11.2017	CSI-W5*	3	1.60	300	11	12	13.03.2006	M	KWPN	211	7	48	40	39	1040	33	M	9	8	no	spread	oxer	left	yes	4	0	42,84	no
Madrid	MUTUA MADRILEÑA TROPHY GRAND PRIX CSI*5 W round A	25.11.2017	CSI-W5*	3	1.60	430	152	12	20.05.2006	M	WESTF	94	7	55	6	37	1750	61	M	1	1	no	spread	oxer	left	no	0	0	71,34	no
Madrid	MUTUA MADRILEÑA TROPHY GRAND PRIX CSI*5 W round A	25.11.2017	CSI-W5*	3	1.60	430	152	12	20.05.2006	M	WESTF	94	7	55	6	37	1750	61	M	2	2	no	vertical	vertical	straight	no	0	0	71,34	no
Madrid	MUTUA MADRILEÑA TROPHY GRAND PRIX CSI*5 W round A	25.11.2017	CSI-W5*	3	1.60	430	152	12	20.05.2006	M	WESTF	94	7	55	6	37	1750	61	M	3	3	no	spread	oxer	right	no	0	0	71,34	no
Madrid	MUTUA MADRILEÑA TROPHY GRAND PRIX CSI*5 W round A	25.11.2017	CSI-W5*	3	1.60	430	152	12	20.05.2006	M	WESTF	94	7	55	6	37	1750	61	M	4	4	no	vertical	vertical	straight	no	0	0	71,34	no
Madrid	MUTUA MADRILEÑA TROPHY GRAND PRIX CSI*5 W round A	25.11.2017	CSI-W5*	3	1.60	430	152	12	20.05.2006	M	WESTF	94	7	55	6	37	1750	61	M	5	5	no	spread	oxer	left	no	0	0	71,34	no
Madrid	MUTUA MADRILEÑA TROPHY GRAND PRIX CSI*5 W round A	25.11.2017	CSI-W5*	3	1.60	430	152	12	20.05.2006	M	WESTF	94	7	55	6	37	1750	61	M	6	6	no	vertical	vertical	left	no	0	0	71,34	no
Madrid	MUTUA MADRILEÑA TROPHY GRAND PRIX CSI*5 W round A	25.11.2017	CSI-W5*	3	1.60	430	152	12	20.05.2006	M	WESTF	94	7	55	6	37	1750	61	M	7	7a	yes	spread	oxer	right	no	0	0	71,34	no
Madrid	MUTUA MADRILEÑA TROPHY GRAND PRIX CSI*5 W round A	25.11.2017	CSI-W5*	3	1.60	430	152	12	20.05.2006	M	WESTF	94	7	55	6	37	1750	61	M	8	7b	yes	vertical	vertical	right	no	0	0	71,34	no
Madrid	MUTUA MADRILEÑA TROPHY GRAND PRIX CSI*5 W round A	25.11.2017	CSI-W5*	3	1.60	430	152	12	20.05.2006	M	WESTF	94	7	55	6	37	1750	61	M	9	8	no	spread	oxer	right	no	0	0	71,34	no
Madrid	MUTUA MADRILEÑA TROPHY GRAND PRIX CSI*5 W round A	25.11.2017	CSI-W5*	3	1.60	430	152	12	20.05.2006	M	WESTF	94	7	55	6	37	1750	61	M	10	9	no	vertical	vertical	right	no	0	0	71,34	no
Madrid	MUTUA MADRILEÑA TROPHY GRAND PRIX CSI*5 W round A	25.11.2017	CSI-W5*	3	1.60	430	152	12	20.05.2006	M	WESTF	94	7	55	6	37	1750	61	M	11	10	no	spread	oxer	straight	no	0	0	71,34	no
Madrid	MUTUA MADRILEÑA TROPHY GRAND PRIX CSI*5 W round A	25.11.2017	CSI-W5*	3	1.60	430	152	12	20.05.2006	M	WESTF	94	7	55	6	37	1750	61	M	12	11	no	vertical	vertical with water	left	no	0	0	71,34	no
Madrid	MUTUA MADRILEÑA TROPHY GRAND PRIX CSI*5 W round A	25.11.2017	CSI-W5*	3	1.60	430	152	12	20.05.2006	M	WESTF	94	7	55	6	37	1750	61	M	13	12a	yes	vertical	vertical	left	no	0	0	71,34	no
Madrid	MUTUA MADRILEÑA TROPHY GRAND PRIX CSI*5 W round A	25.11.2017	CSI-W5*	3	1.60	430	152	12	20.05.2006	M	WESTF	94	7	55	6	37	1750	61	M	14	12b	yes	spread	oxer with water	left	no	0	0	71,34	no
Madrid	MUTUA MADRILEÑA TROPHY GRAND PRIX CSI*5 W round A	25.11.2017	CSI-W5*	3	1.60	430	152	12	20.05.2006	M	WESTF	94	7	55	6	37	1750	61	M	15	12c	yes	vertical	vertical	left	no	0	0	71,34	no
Madrid	MUTUA MADRILEÑA TROPHY GRAND PRIX CSI*5 W round A	25.11.2017	CSI-W5*	3	1.60	430	152	12	20.05.2006	M	WESTF	94	7	55	6	37	1750	61	M	16	13	no	spread	oxer	right	no	0	0	71,34	no
Madrid	MUTUA MADRILEÑA TROPHY GRAND PRIX CSI*5 W round B	25.11.2017	CSI-W5*	3	1.60	300	152	12	20.05.2006	M	WESTF	94	7	55	6	37	1750	61	M	1	1	no	spread	oxer	right	no	0	0	43,58	no
Madrid	MUTUA MADRILEÑA TROPHY GRAND PRIX CSI*5 W round B	25.11.2017	CSI-W5*	3	1.60	300	152	12	20.05.2006	M	WESTF	94	7	55	6	37	1750	61	M	2	2	no	spread	oxer	right	no	0	0	43,58	no
Madrid	MUTUA MADRILEÑA TROPHY GRAND PRIX CSI*5 W round B	25.11.2017	CSI-W5*	3	1.60	300	152	12	20.05.2006	M	WESTF	94	7	55	6	37	1750	61	M	3	3	no	vertical	vertical with water	left	no	0	0	43,58	no
Madrid	MUTUA MADRILEÑA TROPHY GRAND PRIX CSI*5 W round B	25.11.2017	CSI-W5*	3	1.60	300	152	12	20.05.2006	M	WESTF	94	7	55	6	37	1750	61	M	4	4a	yes	vertical	vertical	left	no	0	0	43,58	no
Madrid	MUTUA MADRILEÑA TROPHY GRAND PRIX CSI*5 W round B	25.11.2017	CSI-W5*	3	1.60	300	152	12	20.05.2006	M	WESTF	94	7	55	6	37	1750	61	M	5	4b	yes	spread	oxer with water	left	yes	4	0	43,58	no
Madrid	MUTUA MADRILEÑA TROPHY GRAND PRIX CSI*5 W round B	25.11.2017	CSI-W5*	3	1.60	300	152	12	20.05.2006	M	WESTF	94	7	55	6	37	1750	61	M	6	5	no	spread	oxer	right	no	0	0	43,58	no
Madrid	MUTUA MADRILEÑA TROPHY GRAND PRIX CSI*5 W round B	25.11.2017	CSI-W5*	3	1.60	300	152	12	20.05.2006	M	WESTF	94	7	55	6	37	1750	61	M	7	6	no	vertical	vertical	right	no	0	0	43,58	no
Madrid	MUTUA MADRILEÑA TROPHY GRAND PRIX CSI*5 W round B	25.11.2017	CSI-W5*	3	1.60	300	152	12	20.05.2006	M	WESTF	94	7	55	6	37	1750	61	M	8	7	no	vertical	vertical	left	no	0	0	43,58	no
Madrid	MUTUA MADRILEÑA TROPHY GRAND PRIX CSI*5 W round B	25.11.2017	CSI-W5*	3	1.60	300	152	12	20.05.2006	M	WESTF	94	7	55	6	37	1750	61	M	9	8	no	spread	oxer	left	no	0	0	43,58	no
Madrid	MUTUA MADRILEÑA TROPHY GRAND PRIX CSI*5 W round A	25.11.2017	CSI-W5*	3	1.60	430	31	12	15.04.2006	S	OS	292	11	59	57	32	2641	229	M	1	1	no	spread	oxer	left	no	0	0	71,16	no
Madrid	MUTUA MADRILEÑA TROPHY GRAND PRIX CSI*5 W round A	25.11.2017	CSI-W5*	3	1.60	430	31	12	15.04.2006	S	OS	292	11	59	57	32	2641	229	M	2	2	no	vertical	vertical	straight	no	0	0	71,16	no
Madrid	MUTUA MADRILEÑA TROPHY GRAND PRIX CSI*5 W round A	25.11.2017	CSI-W5*	3	1.60	430	31	12	15.04.2006	S	OS	292	11	59	57	32	2641	229	M	3	3	no	spread	oxer	right	no	0	0	71,16	no
Madrid	MUTUA MADRILEÑA TROPHY GRAND PRIX CSI*5 W round A	25.11.2017	CSI-W5*	3	1.60	430	31	12	15.04.2006	S	OS	292	11	59	57	32	2641	229	M	4	4	no	vertical	vertical	straight	no	0	0	71,16	no
Madrid	MUTUA MADRILEÑA TROPHY GRAND PRIX CSI*5 W round A	25.11.2017	CSI-W5*	3	1.60	430	31	12	15.04.2006	S	OS	292	11	59	57	32	2641	229	M	5	5	no	spread	oxer	left	no	0	0	71,16	no
Madrid	MUTUA MADRILEÑA TROPHY GRAND PRIX CSI*5 W round A	25.11.2017	CSI-W5*	3	1.60	430	31	12	15.04.2006	S	OS	292	11	59	57	32	2641	229	M	6	6	no	vertical	vertical	left	no	0	0	71,16	no
Madrid	MUTUA MADRILEÑA TROPHY GRAND PRIX CSI*5 W round A	25.11.2017	CSI-W5*	3	1.60	430	31	12	15.04.2006	S	OS	292	11	59	57	32	2641	229	M	7	7a	yes	spread	oxer	right	no	0	0	71,16	no
Madrid	MUTUA MADRILEÑA TROPHY GRAND PRIX CSI*5 W round A	25.11.2017	CSI-W5*	3	1.60	430	31	12	15.04.2006	S	OS	292	11	59	57	32	2641	229	M	8	7b	yes	vertical	vertical	right	no	0	0	71,16	no
Madrid	MUTUA MADRILEÑA TROPHY GRAND PRIX CSI*5 W round A	25.11.2017	CSI-W5*	3	1.60	430	31	12	15.04.2006	S	OS	292	11	59	57	32	2641	229	M	9	8	no	spread	oxer	right	no	0	0	71,16	no
Madrid	MUTUA MADRILEÑA TROPHY GRAND PRIX CSI*5 W round A	25.11.2017	CSI-W5*	3	1.60	430	31	12	15.04.2006	S	OS	292	11	59	57	32	2641	229	M	10	9	no	vertical	vertical	right	no	0	0	71,16	no
Madrid	MUTUA MADRILEÑA TROPHY GRAND PRIX CSI*5 W round A	25.11.2017	CSI-W5*	3	1.60	430	31	12	15.04.2006	S	OS	292	11	59	57	32	2641	229	M	11	10	no	spread	oxer	straight	no	0	0	71,16	no
Madrid	MUTUA MADRILEÑA TROPHY GRAND PRIX CSI*5 W round A	25.11.2017	CSI-W5*	3	1.60	430	31	12	15.04.2006	S	OS	292	11	59	57	32	2641	229	M	12	11	no	vertical	vertical with water	left	no	0	0	71,16	no
Madrid	MUTUA MADRILEÑA TROPHY GRAND PRIX CSI*5 W round A	25.11.2017	CSI-W5*	3	1.60	430	31	12	15.04.2006	S	OS	292	11	59	57	32	2641	229	M	13	12a	yes	vertical	vertical	left	no	0	0	71,16	no
Madrid	MUTUA MADRILEÑA TROPHY GRAND PRIX CSI*5 W round A	25.11.2017	CSI-W5*	3	1.60	430	31	12	15.04.2006	S	OS	292	11	59	57	32	2641	229	M	14	12b	yes	spread	oxer with water	left	no	0	0	71,16	no
Madrid	MUTUA MADRILEÑA TROPHY GRAND PRIX CSI*5 W round A	25.11.2017	CSI-W5*	3	1.60	430	31	12	15.04.2006	S	OS	292	11	59	57	32	2641	229	M	15	12c	yes	vertical	vertical	left	yes	4	0	71,16	no
Madrid	MUTUA MADRILEÑA TROPHY GRAND PRIX CSI*5 W round A	25.11.2017	CSI-W5*	3	1.60	430	31	12	15.04.2006	S	OS	292	11	59	57	32	2641	229	M	16	13	no	spread	oxer	right	no	0	0	71,16	no
Madrid	MUTUA MADRILEÑA TROPHY GRAND PRIX CSI*5 W round A	25.11.2017	CSI-W5*	3	1.60	430	43	15	20.05.2003	S	DSP	218	12	64	20	44	2014	118	M	1	1	no	spread	oxer	left	no	0	0	70,72	no
Madrid	MUTUA MADRILEÑA TROPHY GRAND PRIX CSI*5 W round A	25.11.2017	CSI-W5*	3	1.60	430	43	15	20.05.2003	S	DSP	218	12	64	20	44	2014	118	M	2	2	no	vertical	vertical	straight	no	0	0	70,72	no
Madrid	MUTUA MADRILEÑA TROPHY GRAND PRIX CSI*5 W round A	25.11.2017	CSI-W5*	3	1.60	430	43	15	20.05.2003	S	DSP	218	12	64	20	44	2014	118	M	3	3	no	spread	oxer	right	no	0	0	70,72	no
Madrid	MUTUA MADRILEÑA TROPHY GRAND PRIX CSI*5 W round A	25.11.2017	CSI-W5*	3	1.60	430	43	15	20.05.2003	S	DSP	218	12	64	20	44	2014	118	M	4	4	no	vertical	vertical	straight	no	0	0	70,72	no
Madrid	MUTUA MADRILEÑA TROPHY GRAND PRIX CSI*5 W round A	25.11.2017	CSI-W5*	3	1.60	430	43	15	20.05.2003	S	DSP	218	12	64	20	44	2014	118	M	5	5	no	spread	oxer	left	no	0	0	70,72	no
Madrid	MUTUA MADRILEÑA TROPHY GRAND PRIX CSI*5 W round A	25.11.2017	CSI-W5*	3	1.60	430	43	15	20.05.2003	S	DSP	218	12	64	20	44	2014	118	M	6	6	no	vertical	vertical	left	no	0	0	70,72	no
Madrid	MUTUA MADRILEÑA TROPHY GRAND PRIX CSI*5 W round A	25.11.2017	CSI-W5*	3	1.60	430	43	15	20.05.2003	S	DSP	218	12	64	20	44	2014	118	M	7	7a	yes	spread	oxer	right	no	0	0	70,72	no
Madrid	MUTUA MADRILEÑA TROPHY GRAND PRIX CSI*5 W round A	25.11.2017	CSI-W5*	3	1.60	430	43	15	20.05.2003	S	DSP	218	12	64	20	44	2014	118	M	8	7b	yes	vertical	vertical	right	no	0	0	70,72	no
Madrid	MUTUA MADRILEÑA TROPHY GRAND PRIX CSI*5 W round A	25.11.2017	CSI-W5*	3	1.60	430	43	15	20.05.2003	S	DSP	218	12	64	20	44	2014	118	M	9	8	no	spread	oxer	right	no	0	0	70,72	no
Madrid	MUTUA MADRILEÑA TROPHY GRAND PRIX CSI*5 W round A	25.11.2017	CSI-W5*	3	1.60	430	43	15	20.05.2003	S	DSP	218	12	64	20	44	2014	118	M	10	9	no	vertical	vertical	right	no	0	0	70,72	no
Madrid	MUTUA MADRILEÑA TROPHY GRAND PRIX CSI*5 W round A	25.11.2017	CSI-W5*	3	1.60	430	43	15	20.05.2003	S	DSP	218	12	64	20	44	2014	118	M	11	10	no	spread	oxer	straight	no	0	0	70,72	no
Madrid	MUTUA MADRILEÑA TROPHY GRAND PRIX CSI*5 W round A	25.11.2017	CSI-W5*	3	1.60	430	43	15	20.05.2003	S	DSP	218	12	64	20	44	2014	118	M	12	11	no	vertical	vertical with water	left	no	0	0	70,72	no
Madrid	MUTUA MADRILEÑA TROPHY GRAND PRIX CSI*5 W round A	25.11.2017	CSI-W5*	3	1.60	430	43	15	20.05.2003	S	DSP	218	12	64	20	44	2014	118	M	13	12a	yes	vertical	vertical	left	no	0	0	70,72	no
Madrid	MUTUA MADRILEÑA TROPHY GRAND PRIX CSI*5 W round A	25.11.2017	CSI-W5*	3	1.60	430	43	15	20.05.2003	S	DSP	218	12	64	20	44	2014	118	M	14	12b	yes	spread	oxer with water	left	no	0	0	70,72	no
Madrid	MUTUA MADRILEÑA TROPHY GRAND PRIX CSI*5 W round A	25.11.2017	CSI-W5*	3	1.60	430	43	15	20.05.2003	S	DSP	218	12	64	20	44	2014	118	M	15	12c	yes	vertical	vertical	left	yes	4	0	70,72	no
Madrid	MUTUA MADRILEÑA TROPHY GRAND PRIX CSI*5 W round A	25.11.2017	CSI-W5*	3	1.60	430	43	15	20.05.2003	S	DSP	218	12	64	20	44	2014	118	M	16	13	no	spread	oxer	right	no	0	0	70,72	no
Madrid	MUTUA MADRILEÑA TROPHY GRAND PRIX CSI*5 W round A	25.11.2017	CSI-W5*	3	1.60	430	71	10	25.05.2008	M	KWPN	131	2	65	70	43	1682	26	M	1	1	no	spread	oxer	left	no	0	0	71,69	no
Madrid	MUTUA MADRILEÑA TROPHY GRAND PRIX CSI*5 W round A	25.11.2017	CSI-W5*	3	1.60	430	71	10	25.05.2008	M	KWPN	131	2	65	70	43	1682	26	M	2	2	no	vertical	vertical	straight	no	0	0	71,69	no
Madrid	MUTUA MADRILEÑA TROPHY GRAND PRIX CSI*5 W round A	25.11.2017	CSI-W5*	3	1.60	430	71	10	25.05.2008	M	KWPN	131	2	65	70	43	1682	26	M	3	3	no	spread	oxer	right	no	0	0	71,69	no
Madrid	MUTUA MADRILEÑA TROPHY GRAND PRIX CSI*5 W round A	25.11.2017	CSI-W5*	3	1.60	430	71	10	25.05.2008	M	KWPN	131	2	65	70	43	1682	26	M	4	4	no	vertical	vertical	straight	no	0	0	71,69	no
Madrid	MUTUA MADRILEÑA TROPHY GRAND PRIX CSI*5 W round A	25.11.2017	CSI-W5*	3	1.60	430	71	10	25.05.2008	M	KWPN	131	2	65	70	43	1682	26	M	5	5	no	spread	oxer	left	no	0	0	71,69	no
Madrid	MUTUA MADRILEÑA TROPHY GRAND PRIX CSI*5 W round A	25.11.2017	CSI-W5*	3	1.60	430	71	10	25.05.2008	M	KWPN	131	2	65	70	43	1682	26	M	6	6	no	vertical	vertical	left	no	0	0	71,69	no
Madrid	MUTUA MADRILEÑA TROPHY GRAND PRIX CSI*5 W round A	25.11.2017	CSI-W5*	3	1.60	430	71	10	25.05.2008	M	KWPN	131	2	65	70	43	1682	26	M	7	7a	yes	spread	oxer	right	no	0	0	71,69	no
Madrid	MUTUA MADRILEÑA TROPHY GRAND PRIX CSI*5 W round A	25.11.2017	CSI-W5*	3	1.60	430	71	10	25.05.2008	M	KWPN	131	2	65	70	43	1682	26	M	8	7b	yes	vertical	vertical	right	no	0	0	71,69	no
Madrid	MUTUA MADRILEÑA TROPHY GRAND PRIX CSI*5 W round A	25.11.2017	CSI-W5*	3	1.60	430	71	10	25.05.2008	M	KWPN	131	2	65	70	43	1682	26	M	9	8	no	spread	oxer	right	no	0	0	71,69	no
Madrid	MUTUA MADRILEÑA TROPHY GRAND PRIX CSI*5 W round A	25.11.2017	CSI-W5*	3	1.60	430	71	10	25.05.2008	M	KWPN	131	2	65	70	43	1682	26	M	10	9	no	vertical	vertical	right	no	0	0	71,69	no
Madrid	MUTUA MADRILEÑA TROPHY GRAND PRIX CSI*5 W round A	25.11.2017	CSI-W5*	3	1.60	430	71	10	25.05.2008	M	KWPN	131	2	65	70	43	1682	26	M	11	10	no	spread	oxer	straight	no	0	0	71,69	no
Madrid	MUTUA MADRILEÑA TROPHY GRAND PRIX CSI*5 W round A	25.11.2017	CSI-W5*	3	1.60	430	71	10	25.05.2008	M	KWPN	131	2	65	70	43	1682	26	M	12	11	no	vertical	vertical with water	left	no	0	0	71,69	no
Madrid	MUTUA MADRILEÑA TROPHY GRAND PRIX CSI*5 W round A	25.11.2017	CSI-W5*	3	1.60	430	71	10	25.05.2008	M	KWPN	131	2	65	70	43	1682	26	M	13	12a	yes	vertical	vertical	left	no	0	0	71,69	no
Madrid	MUTUA MADRILEÑA TROPHY GRAND PRIX CSI*5 W round A	25.11.2017	CSI-W5*	3	1.60	430	71	10	25.05.2008	M	KWPN	131	2	65	70	43	1682	26	M	14	12b	yes	spread	oxer with water	left	no	0	0	71,69	no
Madrid	MUTUA MADRILEÑA TROPHY GRAND PRIX CSI*5 W round A	25.11.2017	CSI-W5*	3	1.60	430	71	10	25.05.2008	M	KWPN	131	2	65	70	43	1682	26	M	15	12c	yes	vertical	vertical	left	no	0	0	71,69	no
Madrid	MUTUA MADRILEÑA TROPHY GRAND PRIX CSI*5 W round A	25.11.2017	CSI-W5*	3	1.60	430	71	10	25.05.2008	M	KWPN	131	2	65	70	43	1682	26	M	16	13	no	spread	oxer	right	no	0	0	71,69	no
Madrid	MUTUA MADRILEÑA TROPHY GRAND PRIX CSI*5 W round B	25.11.2017	CSI-W5*	3	1.60	300	71	10	25.05.2008	M	KWPN	131	2	65	70	43	1682	26	M	1	1	no	spread	oxer	right	no	0	0	46,93	no
Madrid	MUTUA MADRILEÑA TROPHY GRAND PRIX CSI*5 W round B	25.11.2017	CSI-W5*	3	1.60	300	71	10	25.05.2008	M	KWPN	131	2	65	70	43	1682	26	M	2	2	no	spread	oxer	right	no	0	0	46,93	no
Madrid	MUTUA MADRILEÑA TROPHY GRAND PRIX CSI*5 W round B	25.11.2017	CSI-W5*	3	1.60	300	71	10	25.05.2008	M	KWPN	131	2	65	70	43	1682	26	M	3	3	no	vertical	vertical with water	left	no	0	0	46,93	no
Madrid	MUTUA MADRILEÑA TROPHY GRAND PRIX CSI*5 W round B	25.11.2017	CSI-W5*	3	1.60	300	71	10	25.05.2008	M	KWPN	131	2	65	70	43	1682	26	M	4	4a	yes	vertical	vertical	left	no	0	0	46,93	no
Madrid	MUTUA MADRILEÑA TROPHY GRAND PRIX CSI*5 W round B	25.11.2017	CSI-W5*	3	1.60	300	71	10	25.05.2008	M	KWPN	131	2	65	70	43	1682	26	M	5	4b	yes	spread	oxer with water	left	no	0	0	46,93	no
Madrid	MUTUA MADRILEÑA TROPHY GRAND PRIX CSI*5 W round B	25.11.2017	CSI-W5*	3	1.60	300	71	10	25.05.2008	M	KWPN	131	2	65	70	43	1682	26	M	6	5	no	spread	oxer	right	no	0	0	46,93	no
Madrid	MUTUA MADRILEÑA TROPHY GRAND PRIX CSI*5 W round B	25.11.2017	CSI-W5*	3	1.60	300	71	10	25.05.2008	M	KWPN	131	2	65	70	43	1682	26	M	7	6	no	vertical	vertical	right	no	0	0	46,93	no
Madrid	MUTUA MADRILEÑA TROPHY GRAND PRIX CSI*5 W round B	25.11.2017	CSI-W5*	3	1.60	300	71	10	25.05.2008	M	KWPN	131	2	65	70	43	1682	26	M	8	7	no	vertical	vertical	left	no	0	0	46,93	no
Madrid	MUTUA MADRILEÑA TROPHY GRAND PRIX CSI*5 W round B	25.11.2017	CSI-W5*	3	1.60	300	71	10	25.05.2008	M	KWPN	131	2	65	70	43	1682	26	M	9	8	no	spread	oxer	left	no	0	0	46,93	no
Madrid	MUTUA MADRILEÑA TROPHY GRAND PRIX CSI*5 W round A	25.11.2017	CSI-W5*	3	1.60	430	188	11	06.06.2007	G	KWPN	209	10	67	60	47	2033	59	M	1	1	no	spread	oxer	left	no	0	0	70,8	no
Madrid	MUTUA MADRILEÑA TROPHY GRAND PRIX CSI*5 W round A	25.11.2017	CSI-W5*	3	1.60	430	188	11	06.06.2007	G	KWPN	209	10	67	60	47	2033	59	M	2	2	no	vertical	vertical	straight	no	0	0	70,8	no
Madrid	MUTUA MADRILEÑA TROPHY GRAND PRIX CSI*5 W round A	25.11.2017	CSI-W5*	3	1.60	430	188	11	06.06.2007	G	KWPN	209	10	67	60	47	2033	59	M	3	3	no	spread	oxer	right	no	0	0	70,8	no
Madrid	MUTUA MADRILEÑA TROPHY GRAND PRIX CSI*5 W round A	25.11.2017	CSI-W5*	3	1.60	430	188	11	06.06.2007	G	KWPN	209	10	67	60	47	2033	59	M	4	4	no	vertical	vertical	straight	no	0	0	70,8	no
Madrid	MUTUA MADRILEÑA TROPHY GRAND PRIX CSI*5 W round A	25.11.2017	CSI-W5*	3	1.60	430	188	11	06.06.2007	G	KWPN	209	10	67	60	47	2033	59	M	5	5	no	spread	oxer	left	no	0	0	70,8	no
Madrid	MUTUA MADRILEÑA TROPHY GRAND PRIX CSI*5 W round A	25.11.2017	CSI-W5*	3	1.60	430	188	11	06.06.2007	G	KWPN	209	10	67	60	47	2033	59	M	6	6	no	vertical	vertical	left	no	0	0	70,8	no
Madrid	MUTUA MADRILEÑA TROPHY GRAND PRIX CSI*5 W round A	25.11.2017	CSI-W5*	3	1.60	430	188	11	06.06.2007	G	KWPN	209	10	67	60	47	2033	59	M	7	7a	yes	spread	oxer	right	no	0	0	70,8	no
Madrid	MUTUA MADRILEÑA TROPHY GRAND PRIX CSI*5 W round A	25.11.2017	CSI-W5*	3	1.60	430	188	11	06.06.2007	G	KWPN	209	10	67	60	47	2033	59	M	8	7b	yes	vertical	vertical	right	no	0	0	70,8	no
Madrid	MUTUA MADRILEÑA TROPHY GRAND PRIX CSI*5 W round A	25.11.2017	CSI-W5*	3	1.60	430	188	11	06.06.2007	G	KWPN	209	10	67	60	47	2033	59	M	9	8	no	spread	oxer	right	no	0	0	70,8	no
Madrid	MUTUA MADRILEÑA TROPHY GRAND PRIX CSI*5 W round A	25.11.2017	CSI-W5*	3	1.60	430	188	11	06.06.2007	G	KWPN	209	10	67	60	47	2033	59	M	10	9	no	vertical	vertical	right	no	0	0	70,8	no
Madrid	MUTUA MADRILEÑA TROPHY GRAND PRIX CSI*5 W round A	25.11.2017	CSI-W5*	3	1.60	430	188	11	06.06.2007	G	KWPN	209	10	67	60	47	2033	59	M	11	10	no	spread	oxer	straight	no	0	0	70,8	no
Madrid	MUTUA MADRILEÑA TROPHY GRAND PRIX CSI*5 W round A	25.11.2017	CSI-W5*	3	1.60	430	188	11	06.06.2007	G	KWPN	209	10	67	60	47	2033	59	M	12	11	no	vertical	vertical with water	left	no	0	0	70,8	no
Madrid	MUTUA MADRILEÑA TROPHY GRAND PRIX CSI*5 W round A	25.11.2017	CSI-W5*	3	1.60	430	188	11	06.06.2007	G	KWPN	209	10	67	60	47	2033	59	M	13	12a	yes	vertical	vertical	left	no	0	0	70,8	no
Madrid	MUTUA MADRILEÑA TROPHY GRAND PRIX CSI*5 W round A	25.11.2017	CSI-W5*	3	1.60	430	188	11	06.06.2007	G	KWPN	209	10	67	60	47	2033	59	M	14	12b	yes	spread	oxer with water	left	no	0	0	70,8	no
Madrid	MUTUA MADRILEÑA TROPHY GRAND PRIX CSI*5 W round A	25.11.2017	CSI-W5*	3	1.60	430	188	11	06.06.2007	G	KWPN	209	10	67	60	47	2033	59	M	15	12c	yes	vertical	vertical	left	no	0	0	70,8	no
Madrid	MUTUA MADRILEÑA TROPHY GRAND PRIX CSI*5 W round A	25.11.2017	CSI-W5*	3	1.60	430	188	11	06.06.2007	G	KWPN	209	10	67	60	47	2033	59	M	16	13	no	spread	oxer	right	no	0	0	70,8	no
Madrid	MUTUA MADRILEÑA TROPHY GRAND PRIX CSI*5 W round B	25.11.2017	CSI-W5*	3	1.60	300	188	11	06.06.2007	G	KWPN	209	10	67	60	47	2033	59	M	1	1	no	spread	oxer	right	no	0	0	43,33	no
Madrid	MUTUA MADRILEÑA TROPHY GRAND PRIX CSI*5 W round B	25.11.2017	CSI-W5*	3	1.60	300	188	11	06.06.2007	G	KWPN	209	10	67	60	47	2033	59	M	2	2	no	spread	oxer	right	no	0	0	43,33	no
Madrid	MUTUA MADRILEÑA TROPHY GRAND PRIX CSI*5 W round B	25.11.2017	CSI-W5*	3	1.60	300	188	11	06.06.2007	G	KWPN	209	10	67	60	47	2033	59	M	3	3	no	vertical	vertical with water	left	no	0	0	43,33	no
Madrid	MUTUA MADRILEÑA TROPHY GRAND PRIX CSI*5 W round B	25.11.2017	CSI-W5*	3	1.60	300	188	11	06.06.2007	G	KWPN	209	10	67	60	47	2033	59	M	4	4a	yes	vertical	vertical	left	no	0	0	43,33	no
Madrid	MUTUA MADRILEÑA TROPHY GRAND PRIX CSI*5 W round B	25.11.2017	CSI-W5*	3	1.60	300	188	11	06.06.2007	G	KWPN	209	10	67	60	47	2033	59	M	5	4b	yes	spread	oxer with water	left	no	0	0	43,33	no
Madrid	MUTUA MADRILEÑA TROPHY GRAND PRIX CSI*5 W round B	25.11.2017	CSI-W5*	3	1.60	300	188	11	06.06.2007	G	KWPN	209	10	67	60	47	2033	59	M	6	5	no	spread	oxer	right	no	0	0	43,33	no
Madrid	MUTUA MADRILEÑA TROPHY GRAND PRIX CSI*5 W round B	25.11.2017	CSI-W5*	3	1.60	300	188	11	06.06.2007	G	KWPN	209	10	67	60	47	2033	59	M	7	6	no	vertical	vertical	right	no	0	0	43,33	no
Madrid	MUTUA MADRILEÑA TROPHY GRAND PRIX CSI*5 W round B	25.11.2017	CSI-W5*	3	1.60	300	188	11	06.06.2007	G	KWPN	209	10	67	60	47	2033	59	M	8	7	no	vertical	vertical	left	no	0	0	43,33	no
Madrid	MUTUA MADRILEÑA TROPHY GRAND PRIX CSI*5 W round B	25.11.2017	CSI-W5*	3	1.60	300	188	11	06.06.2007	G	KWPN	209	10	67	60	47	2033	59	M	9	8	no	spread	oxer	left	no	0	0	43,33	no
Madrid	MUTUA MADRILEÑA TROPHY GRAND PRIX CSI*5 W round A	25.11.2017	CSI-W5*	3	1.60	430	12	12	01.04.2006	S	KWPN	105	2	68	67	31	1737	39	F	1	1	no	spread	oxer	left	no	0	0	72,56	no
Madrid	MUTUA MADRILEÑA TROPHY GRAND PRIX CSI*5 W round A	25.11.2017	CSI-W5*	3	1.60	430	12	12	01.04.2006	S	KWPN	105	2	68	67	31	1737	39	F	2	2	no	vertical	vertical	straight	no	0	0	72,56	no
Madrid	MUTUA MADRILEÑA TROPHY GRAND PRIX CSI*5 W round A	25.11.2017	CSI-W5*	3	1.60	430	12	12	01.04.2006	S	KWPN	105	2	68	67	31	1737	39	F	3	3	no	spread	oxer	right	no	0	0	72,56	no
Madrid	MUTUA MADRILEÑA TROPHY GRAND PRIX CSI*5 W round A	25.11.2017	CSI-W5*	3	1.60	430	12	12	01.04.2006	S	KWPN	105	2	68	67	31	1737	39	F	4	4	no	vertical	vertical	straight	no	0	0	72,56	no
Madrid	MUTUA MADRILEÑA TROPHY GRAND PRIX CSI*5 W round A	25.11.2017	CSI-W5*	3	1.60	430	12	12	01.04.2006	S	KWPN	105	2	68	67	31	1737	39	F	5	5	no	spread	oxer	left	no	0	0	72,56	no
Madrid	MUTUA MADRILEÑA TROPHY GRAND PRIX CSI*5 W round A	25.11.2017	CSI-W5*	3	1.60	430	12	12	01.04.2006	S	KWPN	105	2	68	67	31	1737	39	F	6	6	no	vertical	vertical	left	yes	4	0	72,56	no
Madrid	MUTUA MADRILEÑA TROPHY GRAND PRIX CSI*5 W round A	25.11.2017	CSI-W5*	3	1.60	430	12	12	01.04.2006	S	KWPN	105	2	68	67	31	1737	39	F	7	7a	yes	spread	oxer	right	no	0	0	72,56	no
Madrid	MUTUA MADRILEÑA TROPHY GRAND PRIX CSI*5 W round A	25.11.2017	CSI-W5*	3	1.60	430	12	12	01.04.2006	S	KWPN	105	2	68	67	31	1737	39	F	8	7b	yes	vertical	vertical	right	no	0	0	72,56	no
Madrid	MUTUA MADRILEÑA TROPHY GRAND PRIX CSI*5 W round A	25.11.2017	CSI-W5*	3	1.60	430	12	12	01.04.2006	S	KWPN	105	2	68	67	31	1737	39	F	9	8	no	spread	oxer	right	no	0	0	72,56	no
Madrid	MUTUA MADRILEÑA TROPHY GRAND PRIX CSI*5 W round A	25.11.2017	CSI-W5*	3	1.60	430	12	12	01.04.2006	S	KWPN	105	2	68	67	31	1737	39	F	10	9	no	vertical	vertical	right	no	0	0	72,56	no
Madrid	MUTUA MADRILEÑA TROPHY GRAND PRIX CSI*5 W round A	25.11.2017	CSI-W5*	3	1.60	430	12	12	01.04.2006	S	KWPN	105	2	68	67	31	1737	39	F	11	10	no	spread	oxer	straight	no	0	0	72,56	no
Madrid	MUTUA MADRILEÑA TROPHY GRAND PRIX CSI*5 W round A	25.11.2017	CSI-W5*	3	1.60	430	12	12	01.04.2006	S	KWPN	105	2	68	67	31	1737	39	F	12	11	no	vertical	vertical with water	left	no	0	0	72,56	no
Madrid	MUTUA MADRILEÑA TROPHY GRAND PRIX CSI*5 W round A	25.11.2017	CSI-W5*	3	1.60	430	12	12	01.04.2006	S	KWPN	105	2	68	67	31	1737	39	F	13	12a	yes	vertical	vertical	left	no	0	0	72,56	no
Madrid	MUTUA MADRILEÑA TROPHY GRAND PRIX CSI*5 W round A	25.11.2017	CSI-W5*	3	1.60	430	12	12	01.04.2006	S	KWPN	105	2	68	67	31	1737	39	F	14	12b	yes	spread	oxer with water	left	no	0	0	72,56	no
Madrid	MUTUA MADRILEÑA TROPHY GRAND PRIX CSI*5 W round A	25.11.2017	CSI-W5*	3	1.60	430	12	12	01.04.2006	S	KWPN	105	2	68	67	31	1737	39	F	15	12c	yes	vertical	vertical	left	no	0	0	72,56	no
Madrid	MUTUA MADRILEÑA TROPHY GRAND PRIX CSI*5 W round A	25.11.2017	CSI-W5*	3	1.60	430	12	12	01.04.2006	S	KWPN	105	2	68	67	31	1737	39	F	16	13	no	spread	oxer	right	no	0	0	72,56	no
Madrid	MUTUA MADRILEÑA TROPHY GRAND PRIX CSI*5 W round A	25.11.2017	CSI-W5*	3	1.60	430	55	15	05.05.2003	S	WESTF	199	6	71	12	44	2177	93	M	1	1	no	spread	oxer	left	no	0	0	70,59	no
Madrid	MUTUA MADRILEÑA TROPHY GRAND PRIX CSI*5 W round A	25.11.2017	CSI-W5*	3	1.60	430	55	15	05.05.2003	S	WESTF	199	6	71	12	44	2177	93	M	2	2	no	vertical	vertical	straight	no	0	0	70,59	no
Madrid	MUTUA MADRILEÑA TROPHY GRAND PRIX CSI*5 W round A	25.11.2017	CSI-W5*	3	1.60	430	55	15	05.05.2003	S	WESTF	199	6	71	12	44	2177	93	M	3	3	no	spread	oxer	right	no	0	0	70,59	no
Madrid	MUTUA MADRILEÑA TROPHY GRAND PRIX CSI*5 W round A	25.11.2017	CSI-W5*	3	1.60	430	55	15	05.05.2003	S	WESTF	199	6	71	12	44	2177	93	M	4	4	no	vertical	vertical	straight	no	0	0	70,59	no
Madrid	MUTUA MADRILEÑA TROPHY GRAND PRIX CSI*5 W round A	25.11.2017	CSI-W5*	3	1.60	430	55	15	05.05.2003	S	WESTF	199	6	71	12	44	2177	93	M	5	5	no	spread	oxer	left	no	0	0	70,59	no
Madrid	MUTUA MADRILEÑA TROPHY GRAND PRIX CSI*5 W round A	25.11.2017	CSI-W5*	3	1.60	430	55	15	05.05.2003	S	WESTF	199	6	71	12	44	2177	93	M	6	6	no	vertical	vertical	left	no	0	0	70,59	no
Madrid	MUTUA MADRILEÑA TROPHY GRAND PRIX CSI*5 W round A	25.11.2017	CSI-W5*	3	1.60	430	55	15	05.05.2003	S	WESTF	199	6	71	12	44	2177	93	M	7	7a	yes	spread	oxer	right	no	0	0	70,59	no
Madrid	MUTUA MADRILEÑA TROPHY GRAND PRIX CSI*5 W round A	25.11.2017	CSI-W5*	3	1.60	430	55	15	05.05.2003	S	WESTF	199	6	71	12	44	2177	93	M	8	7b	yes	vertical	vertical	right	no	0	0	70,59	no
Madrid	MUTUA MADRILEÑA TROPHY GRAND PRIX CSI*5 W round A	25.11.2017	CSI-W5*	3	1.60	430	55	15	05.05.2003	S	WESTF	199	6	71	12	44	2177	93	M	9	8	no	spread	oxer	right	no	0	0	70,59	no
Madrid	MUTUA MADRILEÑA TROPHY GRAND PRIX CSI*5 W round A	25.11.2017	CSI-W5*	3	1.60	430	55	15	05.05.2003	S	WESTF	199	6	71	12	44	2177	93	M	10	9	no	vertical	vertical	right	no	0	0	70,59	no
Madrid	MUTUA MADRILEÑA TROPHY GRAND PRIX CSI*5 W round A	25.11.2017	CSI-W5*	3	1.60	430	55	15	05.05.2003	S	WESTF	199	6	71	12	44	2177	93	M	11	10	no	spread	oxer	straight	no	0	0	70,59	no
Madrid	MUTUA MADRILEÑA TROPHY GRAND PRIX CSI*5 W round A	25.11.2017	CSI-W5*	3	1.60	430	55	15	05.05.2003	S	WESTF	199	6	71	12	44	2177	93	M	12	11	no	vertical	vertical with water	left	no	0	0	70,59	no
Madrid	MUTUA MADRILEÑA TROPHY GRAND PRIX CSI*5 W round A	25.11.2017	CSI-W5*	3	1.60	430	55	15	05.05.2003	S	WESTF	199	6	71	12	44	2177	93	M	13	12a	yes	vertical	vertical	left	no	0	0	70,59	no
Madrid	MUTUA MADRILEÑA TROPHY GRAND PRIX CSI*5 W round A	25.11.2017	CSI-W5*	3	1.60	430	55	15	05.05.2003	S	WESTF	199	6	71	12	44	2177	93	M	14	12b	yes	spread	oxer with water	left	no	0	0	70,59	no
Madrid	MUTUA MADRILEÑA TROPHY GRAND PRIX CSI*5 W round A	25.11.2017	CSI-W5*	3	1.60	430	55	15	05.05.2003	S	WESTF	199	6	71	12	44	2177	93	M	15	12c	yes	vertical	vertical	left	no	0	0	70,59	no
Madrid	MUTUA MADRILEÑA TROPHY GRAND PRIX CSI*5 W round A	25.11.2017	CSI-W5*	3	1.60	430	55	15	05.05.2003	S	WESTF	199	6	71	12	44	2177	93	M	16	13	no	spread	oxer	right	no	0	0	70,59	no
Madrid	MUTUA MADRILEÑA TROPHY GRAND PRIX CSI*5 W round B	25.11.2017	CSI-W5*	3	1.60	300	55	15	05.05.2003	S	WESTF	199	6	71	12	44	2177	93	M	1	1	no	spread	oxer	right	no	0	0	42,76	no
Madrid	MUTUA MADRILEÑA TROPHY GRAND PRIX CSI*5 W round B	25.11.2017	CSI-W5*	3	1.60	300	55	15	05.05.2003	S	WESTF	199	6	71	12	44	2177	93	M	2	2	no	spread	oxer	right	no	0	0	42,76	no
Madrid	MUTUA MADRILEÑA TROPHY GRAND PRIX CSI*5 W round B	25.11.2017	CSI-W5*	3	1.60	300	55	15	05.05.2003	S	WESTF	199	6	71	12	44	2177	93	M	3	3	no	vertical	vertical with water	left	no	0	0	42,76	no
Madrid	MUTUA MADRILEÑA TROPHY GRAND PRIX CSI*5 W round B	25.11.2017	CSI-W5*	3	1.60	300	55	15	05.05.2003	S	WESTF	199	6	71	12	44	2177	93	M	4	4a	yes	vertical	vertical	left	no	0	0	42,76	no
Madrid	MUTUA MADRILEÑA TROPHY GRAND PRIX CSI*5 W round B	25.11.2017	CSI-W5*	3	1.60	300	55	15	05.05.2003	S	WESTF	199	6	71	12	44	2177	93	M	5	4b	yes	spread	oxer with water	left	no	0	0	42,76	no
Madrid	MUTUA MADRILEÑA TROPHY GRAND PRIX CSI*5 W round B	25.11.2017	CSI-W5*	3	1.60	300	55	15	05.05.2003	S	WESTF	199	6	71	12	44	2177	93	M	6	5	no	spread	oxer	right	no	0	0	42,76	no
Madrid	MUTUA MADRILEÑA TROPHY GRAND PRIX CSI*5 W round B	25.11.2017	CSI-W5*	3	1.60	300	55	15	05.05.2003	S	WESTF	199	6	71	12	44	2177	93	M	7	6	no	vertical	vertical	right	no	0	0	42,76	no
Madrid	MUTUA MADRILEÑA TROPHY GRAND PRIX CSI*5 W round B	25.11.2017	CSI-W5*	3	1.60	300	55	15	05.05.2003	S	WESTF	199	6	71	12	44	2177	93	M	8	7	no	vertical	vertical	left	no	0	0	42,76	no
Madrid	MUTUA MADRILEÑA TROPHY GRAND PRIX CSI*5 W round B	25.11.2017	CSI-W5*	3	1.60	300	55	15	05.05.2003	S	WESTF	199	6	71	12	44	2177	93	M	9	8	no	spread	oxer	left	no	0	0	42,76	no
Madrid	MUTUA MADRILEÑA TROPHY GRAND PRIX CSI*5 W round A	25.11.2017	CSI-W5*	3	1.60	430	173	13	14.07.2005	G	SF	229	2	74	65	34	1260	61	M	1	1	no	spread	oxer	left	no	0	0	74,36	no
Madrid	MUTUA MADRILEÑA TROPHY GRAND PRIX CSI*5 W round A	25.11.2017	CSI-W5*	3	1.60	430	173	13	14.07.2005	G	SF	229	2	74	65	34	1260	61	M	2	2	no	vertical	vertical	straight	no	0	0	74,36	no
Madrid	MUTUA MADRILEÑA TROPHY GRAND PRIX CSI*5 W round A	25.11.2017	CSI-W5*	3	1.60	430	173	13	14.07.2005	G	SF	229	2	74	65	34	1260	61	M	3	3	no	spread	oxer	right	no	0	0	74,36	no
Madrid	MUTUA MADRILEÑA TROPHY GRAND PRIX CSI*5 W round A	25.11.2017	CSI-W5*	3	1.60	430	173	13	14.07.2005	G	SF	229	2	74	65	34	1260	61	M	4	4	no	vertical	vertical	straight	no	0	0	74,36	no
Madrid	MUTUA MADRILEÑA TROPHY GRAND PRIX CSI*5 W round A	25.11.2017	CSI-W5*	3	1.60	430	173	13	14.07.2005	G	SF	229	2	74	65	34	1260	61	M	5	5	no	spread	oxer	left	no	0	0	74,36	no
Madrid	MUTUA MADRILEÑA TROPHY GRAND PRIX CSI*5 W round A	25.11.2017	CSI-W5*	3	1.60	430	173	13	14.07.2005	G	SF	229	2	74	65	34	1260	61	M	6	6	no	vertical	vertical	left	no	0	0	74,36	no
Madrid	MUTUA MADRILEÑA TROPHY GRAND PRIX CSI*5 W round A	25.11.2017	CSI-W5*	3	1.60	430	173	13	14.07.2005	G	SF	229	2	74	65	34	1260	61	M	7	7a	yes	spread	oxer	right	no	0	0	74,36	no
Madrid	MUTUA MADRILEÑA TROPHY GRAND PRIX CSI*5 W round A	25.11.2017	CSI-W5*	3	1.60	430	173	13	14.07.2005	G	SF	229	2	74	65	34	1260	61	M	8	7b	yes	vertical	vertical	right	yes	4	0	74,36	no
Madrid	MUTUA MADRILEÑA TROPHY GRAND PRIX CSI*5 W round A	25.11.2017	CSI-W5*	3	1.60	430	173	13	14.07.2005	G	SF	229	2	74	65	34	1260	61	M	9	8	no	spread	oxer	right	no	0	0	74,36	no
Madrid	MUTUA MADRILEÑA TROPHY GRAND PRIX CSI*5 W round A	25.11.2017	CSI-W5*	3	1.60	430	173	13	14.07.2005	G	SF	229	2	74	65	34	1260	61	M	10	9	no	vertical	vertical	right	no	0	0	74,36	no
Madrid	MUTUA MADRILEÑA TROPHY GRAND PRIX CSI*5 W round A	25.11.2017	CSI-W5*	3	1.60	430	173	13	14.07.2005	G	SF	229	2	74	65	34	1260	61	M	11	10	no	spread	oxer	straight	no	0	0	74,36	no
Madrid	MUTUA MADRILEÑA TROPHY GRAND PRIX CSI*5 W round A	25.11.2017	CSI-W5*	3	1.60	430	173	13	14.07.2005	G	SF	229	2	74	65	34	1260	61	M	12	11	no	vertical	vertical with water	left	yes	4	0	74,36	no
Madrid	MUTUA MADRILEÑA TROPHY GRAND PRIX CSI*5 W round A	25.11.2017	CSI-W5*	3	1.60	430	173	13	14.07.2005	G	SF	229	2	74	65	34	1260	61	M	13	12a	yes	vertical	vertical	left	no	0	0	74,36	no
Madrid	MUTUA MADRILEÑA TROPHY GRAND PRIX CSI*5 W round A	25.11.2017	CSI-W5*	3	1.60	430	173	13	14.07.2005	G	SF	229	2	74	65	34	1260	61	M	14	12b	yes	spread	oxer with water	left	no	0	0	74,36	no
Madrid	MUTUA MADRILEÑA TROPHY GRAND PRIX CSI*5 W round A	25.11.2017	CSI-W5*	3	1.60	430	173	13	14.07.2005	G	SF	229	2	74	65	34	1260	61	M	15	12c	yes	vertical	vertical	left	no	0	0	74,36	no
Madrid	MUTUA MADRILEÑA TROPHY GRAND PRIX CSI*5 W round A	25.11.2017	CSI-W5*	3	1.60	430	173	13	14.07.2005	G	SF	229	2	74	65	34	1260	61	M	16	13	no	spread	oxer	right	no	0	1	74,36	no
Madrid	MUTUA MADRILEÑA TROPHY GRAND PRIX CSI*5 W round A	25.11.2017	CSI-W5*	3	1.60	430	192	12	27.04.2006	M	SF	164	14	75	22	53	2104	161	M	1	1	no	spread	oxer	left	no	0	0	72,23	no
Madrid	MUTUA MADRILEÑA TROPHY GRAND PRIX CSI*5 W round A	25.11.2017	CSI-W5*	3	1.60	430	192	12	27.04.2006	M	SF	164	14	75	22	53	2104	161	M	2	2	no	vertical	vertical	straight	no	0	0	72,23	no
Madrid	MUTUA MADRILEÑA TROPHY GRAND PRIX CSI*5 W round A	25.11.2017	CSI-W5*	3	1.60	430	192	12	27.04.2006	M	SF	164	14	75	22	53	2104	161	M	3	3	no	spread	oxer	right	no	0	0	72,23	no
Madrid	MUTUA MADRILEÑA TROPHY GRAND PRIX CSI*5 W round A	25.11.2017	CSI-W5*	3	1.60	430	192	12	27.04.2006	M	SF	164	14	75	22	53	2104	161	M	4	4	no	vertical	vertical	straight	no	0	0	72,23	no
Madrid	MUTUA MADRILEÑA TROPHY GRAND PRIX CSI*5 W round A	25.11.2017	CSI-W5*	3	1.60	430	192	12	27.04.2006	M	SF	164	14	75	22	53	2104	161	M	5	5	no	spread	oxer	left	no	0	0	72,23	no
Madrid	MUTUA MADRILEÑA TROPHY GRAND PRIX CSI*5 W round A	25.11.2017	CSI-W5*	3	1.60	430	192	12	27.04.2006	M	SF	164	14	75	22	53	2104	161	M	6	6	no	vertical	vertical	left	no	0	0	72,23	no
Madrid	MUTUA MADRILEÑA TROPHY GRAND PRIX CSI*5 W round A	25.11.2017	CSI-W5*	3	1.60	430	192	12	27.04.2006	M	SF	164	14	75	22	53	2104	161	M	7	7a	yes	spread	oxer	right	no	0	0	72,23	no
Madrid	MUTUA MADRILEÑA TROPHY GRAND PRIX CSI*5 W round A	25.11.2017	CSI-W5*	3	1.60	430	192	12	27.04.2006	M	SF	164	14	75	22	53	2104	161	M	8	7b	yes	vertical	vertical	right	no	0	0	72,23	no
Madrid	MUTUA MADRILEÑA TROPHY GRAND PRIX CSI*5 W round A	25.11.2017	CSI-W5*	3	1.60	430	192	12	27.04.2006	M	SF	164	14	75	22	53	2104	161	M	9	8	no	spread	oxer	right	no	0	0	72,23	no
Madrid	MUTUA MADRILEÑA TROPHY GRAND PRIX CSI*5 W round A	25.11.2017	CSI-W5*	3	1.60	430	192	12	27.04.2006	M	SF	164	14	75	22	53	2104	161	M	10	9	no	vertical	vertical	right	no	0	0	72,23	no
Madrid	MUTUA MADRILEÑA TROPHY GRAND PRIX CSI*5 W round A	25.11.2017	CSI-W5*	3	1.60	430	192	12	27.04.2006	M	SF	164	14	75	22	53	2104	161	M	11	10	no	spread	oxer	straight	no	0	0	72,23	no
Madrid	MUTUA MADRILEÑA TROPHY GRAND PRIX CSI*5 W round A	25.11.2017	CSI-W5*	3	1.60	430	192	12	27.04.2006	M	SF	164	14	75	22	53	2104	161	M	12	11	no	vertical	vertical with water	left	no	0	0	72,23	no
Madrid	MUTUA MADRILEÑA TROPHY GRAND PRIX CSI*5 W round A	25.11.2017	CSI-W5*	3	1.60	430	192	12	27.04.2006	M	SF	164	14	75	22	53	2104	161	M	13	12a	yes	vertical	vertical	left	no	0	0	72,23	no
Madrid	MUTUA MADRILEÑA TROPHY GRAND PRIX CSI*5 W round A	25.11.2017	CSI-W5*	3	1.60	430	192	12	27.04.2006	M	SF	164	14	75	22	53	2104	161	M	14	12b	yes	spread	oxer with water	left	no	0	0	72,23	no
Madrid	MUTUA MADRILEÑA TROPHY GRAND PRIX CSI*5 W round A	25.11.2017	CSI-W5*	3	1.60	430	192	12	27.04.2006	M	SF	164	14	75	22	53	2104	161	M	15	12c	yes	vertical	vertical	left	no	0	0	72,23	no
Madrid	MUTUA MADRILEÑA TROPHY GRAND PRIX CSI*5 W round A	25.11.2017	CSI-W5*	3	1.60	430	192	12	27.04.2006	M	SF	164	14	75	22	53	2104	161	M	16	13	no	spread	oxer	right	no	0	0	72,23	no
Madrid	MUTUA MADRILEÑA TROPHY GRAND PRIX CSI*5 W round B	25.11.2017	CSI-W5*	3	1.60	300	192	12	27.04.2006	M	SF	164	14	75	22	53	2104	161	M	1	1	no	spread	oxer	right	no	0	0	42,23	no
Madrid	MUTUA MADRILEÑA TROPHY GRAND PRIX CSI*5 W round B	25.11.2017	CSI-W5*	3	1.60	300	192	12	27.04.2006	M	SF	164	14	75	22	53	2104	161	M	2	2	no	spread	oxer	right	no	0	0	42,23	no
Madrid	MUTUA MADRILEÑA TROPHY GRAND PRIX CSI*5 W round B	25.11.2017	CSI-W5*	3	1.60	300	192	12	27.04.2006	M	SF	164	14	75	22	53	2104	161	M	3	3	no	vertical	vertical with water	left	no	0	0	42,23	no
Madrid	MUTUA MADRILEÑA TROPHY GRAND PRIX CSI*5 W round B	25.11.2017	CSI-W5*	3	1.60	300	192	12	27.04.2006	M	SF	164	14	75	22	53	2104	161	M	4	4a	yes	vertical	vertical	left	no	0	0	42,23	no
Madrid	MUTUA MADRILEÑA TROPHY GRAND PRIX CSI*5 W round B	25.11.2017	CSI-W5*	3	1.60	300	192	12	27.04.2006	M	SF	164	14	75	22	53	2104	161	M	5	4b	yes	spread	oxer with water	left	no	0	0	42,23	no
Madrid	MUTUA MADRILEÑA TROPHY GRAND PRIX CSI*5 W round B	25.11.2017	CSI-W5*	3	1.60	300	192	12	27.04.2006	M	SF	164	14	75	22	53	2104	161	M	6	5	no	spread	oxer	right	no	0	0	42,23	no
Madrid	MUTUA MADRILEÑA TROPHY GRAND PRIX CSI*5 W round B	25.11.2017	CSI-W5*	3	1.60	300	192	12	27.04.2006	M	SF	164	14	75	22	53	2104	161	M	7	6	no	vertical	vertical	right	no	0	0	42,23	no
Madrid	MUTUA MADRILEÑA TROPHY GRAND PRIX CSI*5 W round B	25.11.2017	CSI-W5*	3	1.60	300	192	12	27.04.2006	M	SF	164	14	75	22	53	2104	161	M	8	7	no	vertical	vertical	left	no	0	0	42,23	no
Madrid	MUTUA MADRILEÑA TROPHY GRAND PRIX CSI*5 W round B	25.11.2017	CSI-W5*	3	1.60	300	192	12	27.04.2006	M	SF	164	14	75	22	53	2104	161	M	9	8	no	spread	oxer	left	no	0	0	42,23	no

Madrid	MUTUA MADRILEÑA TROPHY GRAND PRIX CSI*5 W round A	25.11.2017	CSI-W5*	3	1.60	430	26	17	15.05.2001	G	HOLST	292	18	77	27	33	1671	77	M	1	1	no	spread	oxer	left	no	0	0	68,34	no
Madrid	MUTUA MADRILEÑA TROPHY GRAND PRIX CSI*5 W round A	25.11.2017	CSI-W5*	3	1.60	430	26	17	15.05.2001	G	HOLST	292	18	77	27	33	1671	77	M	2	2	no	vertical	vertical	straight	no	0	0	68,34	no
Madrid	MUTUA MADRILEÑA TROPHY GRAND PRIX CSI*5 W round A	25.11.2017	CSI-W5*	3	1.60	430	26	17	15.05.2001	G	HOLST	292	18	77	27	33	1671	77	M	3	3	no	spread	oxer	right	no	0	0	68,34	no
Madrid	MUTUA MADRILEÑA TROPHY GRAND PRIX CSI*5 W round A	25.11.2017	CSI-W5*	3	1.60	430	26	17	15.05.2001	G	HOLST	292	18	77	27	33	1671	77	M	4	4	no	vertical	vertical	straight	no	0	0	68,34	no
Madrid	MUTUA MADRILEÑA TROPHY GRAND PRIX CSI*5 W round A	25.11.2017	CSI-W5*	3	1.60	430	26	17	15.05.2001	G	HOLST	292	18	77	27	33	1671	77	M	5	5	no	spread	oxer	left	no	0	0	68,34	no
Madrid	MUTUA MADRILEÑA TROPHY GRAND PRIX CSI*5 W round A	25.11.2017	CSI-W5*	3	1.60	430	26	17	15.05.2001	G	HOLST	292	18	77	27	33	1671	77	M	6	6	no	vertical	vertical	left	no	0	0	68,34	no
Madrid	MUTUA MADRILEÑA TROPHY GRAND PRIX CSI*5 W round A	25.11.2017	CSI-W5*	3	1.60	430	26	17	15.05.2001	G	HOLST	292	18	77	27	33	1671	77	M	7	7a	yes	spread	oxer	right	no	0	0	68,34	no
Madrid	MUTUA MADRILEÑA TROPHY GRAND PRIX CSI*5 W round A	25.11.2017	CSI-W5*	3	1.60	430	26	17	15.05.2001	G	HOLST	292	18	77	27	33	1671	77	M	8	7b	yes	vertical	vertical	right	no	0	0	68,34	no
Madrid	MUTUA MADRILEÑA TROPHY GRAND PRIX CSI*5 W round A	25.11.2017	CSI-W5*	3	1.60	430	26	17	15.05.2001	G	HOLST	292	18	77	27	33	1671	77	M	9	8	no	spread	oxer	right	yes	4	0	68,34	no
Madrid	MUTUA MADRILEÑA TROPHY GRAND PRIX CSI*5 W round A	25.11.2017	CSI-W5*	3	1.60	430	26	17	15.05.2001	G	HOLST	292	18	77	27	33	1671	77	M	10	9	no	vertical	vertical	right	no	0	0	68,34	no
Madrid	MUTUA MADRILEÑA TROPHY GRAND PRIX CSI*5 W round A	25.11.2017	CSI-W5*	3	1.60	430	26	17	15.05.2001	G	HOLST	292	18	77	27	33	1671	77	M	11	10	no	spread	oxer	straight	no	0	0	68,34	no
Madrid	MUTUA MADRILEÑA TROPHY GRAND PRIX CSI*5 W round A	25.11.2017	CSI-W5*	3	1.60	430	26	17	15.05.2001	G	HOLST	292	18	77	27	33	1671	77	M	12	11	no	vertical	vertical with water	left	no	0	0	68,34	no
Madrid	MUTUA MADRILEÑA TROPHY GRAND PRIX CSI*5 W round A	25.11.2017	CSI-W5*	3	1.60	430	26	17	15.05.2001	G	HOLST	292	18	77	27	33	1671	77	M	13	12a	yes	vertical	vertical	left	no	0	0	68,34	no
Madrid	MUTUA MADRILEÑA TROPHY GRAND PRIX CSI*5 W round A	25.11.2017	CSI-W5*	3	1.60	430	26	17	15.05.2001	G	HOLST	292	18	77	27	33	1671	77	M	14	12b	yes	spread	oxer with water	left	no	0	0	68,34	no
Madrid	MUTUA MADRILEÑA TROPHY GRAND PRIX CSI*5 W round A	25.11.2017	CSI-W5*	3	1.60	430	26	17	15.05.2001	G	HOLST	292	18	77	27	33	1671	77	M	15	12c	yes	vertical	vertical	left	no	0	0	68,34	no
Madrid	MUTUA MADRILEÑA TROPHY GRAND PRIX CSI*5 W round A	25.11.2017	CSI-W5*	3	1.60	430	26	17	15.05.2001	G	HOLST	292	18	77	27	33	1671	77	M	16	13	no	spread	oxer	right	no	0	0	68,34	no
Madrid	MUTUA MADRILEÑA TROPHY GRAND PRIX CSI*5 W round A	25.11.2017	CSI-W5*	3	1.60	430	181	12	20.03.2006	G	ZANG	214	6	81	76	33	1923	71	M	1	1	no	spread	oxer	left	no	0	0	71,13	no
Madrid	MUTUA MADRILEÑA TROPHY GRAND PRIX CSI*5 W round A	25.11.2017	CSI-W5*	3	1.60	430	181	12	20.03.2006	G	ZANG	214	6	81	76	33	1923	71	M	2	2	no	vertical	vertical	straight	no	0	0	71,13	no
Madrid	MUTUA MADRILEÑA TROPHY GRAND PRIX CSI*5 W round A	25.11.2017	CSI-W5*	3	1.60	430	181	12	20.03.2006	G	ZANG	214	6	81	76	33	1923	71	M	3	3	no	spread	oxer	right	no	0	0	71,13	no
Madrid	MUTUA MADRILEÑA TROPHY GRAND PRIX CSI*5 W round A	25.11.2017	CSI-W5*	3	1.60	430	181	12	20.03.2006	G	ZANG	214	6	81	76	33	1923	71	M	4	4	no	vertical	vertical	straight	no	0	0	71,13	no
Madrid	MUTUA MADRILEÑA TROPHY GRAND PRIX CSI*5 W round A	25.11.2017	CSI-W5*	3	1.60	430	181	12	20.03.2006	G	ZANG	214	6	81	76	33	1923	71	M	5	5	no	spread	oxer	left	no	0	0	71,13	no
Madrid	MUTUA MADRILEÑA TROPHY GRAND PRIX CSI*5 W round A	25.11.2017	CSI-W5*	3	1.60	430	181	12	20.03.2006	G	ZANG	214	6	81	76	33	1923	71	M	6	6	no	vertical	vertical	left	no	0	0	71,13	no
Madrid	MUTUA MADRILEÑA TROPHY GRAND PRIX CSI*5 W round A	25.11.2017	CSI-W5*	3	1.60	430	181	12	20.03.2006	G	ZANG	214	6	81	76	33	1923	71	M	7	7a	yes	spread	oxer	right	no	0	0	71,13	no
Madrid	MUTUA MADRILEÑA TROPHY GRAND PRIX CSI*5 W round A	25.11.2017	CSI-W5*	3	1.60	430	181	12	20.03.2006	G	ZANG	214	6	81	76	33	1923	71	M	8	7b	yes	vertical	vertical	right	no	0	0	71,13	no
Madrid	MUTUA MADRILEÑA TROPHY GRAND PRIX CSI*5 W round A	25.11.2017	CSI-W5*	3	1.60	430	181	12	20.03.2006	G	ZANG	214	6	81	76	33	1923	71	M	9	8	no	spread	oxer	right	no	0	0	71,13	no
Madrid	MUTUA MADRILEÑA TROPHY GRAND PRIX CSI*5 W round A	25.11.2017	CSI-W5*	3	1.60	430	181	12	20.03.2006	G	ZANG	214	6	81	76	33	1923	71	M	10	9	no	vertical	vertical	right	no	0	0	71,13	no
Madrid	MUTUA MADRILEÑA TROPHY GRAND PRIX CSI*5 W round A	25.11.2017	CSI-W5*	3	1.60	430	181	12	20.03.2006	G	ZANG	214	6	81	76	33	1923	71	M	11	10	no	spread	oxer	straight	no	0	0	71,13	no
Madrid	MUTUA MADRILEÑA TROPHY GRAND PRIX CSI*5 W round A	25.11.2017	CSI-W5*	3	1.60	430	181	12	20.03.2006	G	ZANG	214	6	81	76	33	1923	71	M	12	11	no	vertical	vertical with water	left	yes	4	0	71,13	no
Madrid	MUTUA MADRILEÑA TROPHY GRAND PRIX CSI*5 W round A	25.11.2017	CSI-W5*	3	1.60	430	181	12	20.03.2006	G	ZANG	214	6	81	76	33	1923	71	M	13	12a	yes	vertical	vertical	left	no	0	0	71,13	no
Madrid	MUTUA MADRILEÑA TROPHY GRAND PRIX CSI*5 W round A	25.11.2017	CSI-W5*	3	1.60	430	181	12	20.03.2006	G	ZANG	214	6	81	76	33	1923	71	M	14	12b	yes	spread	oxer with water	left	no	0	0	71,13	no
Madrid	MUTUA MADRILEÑA TROPHY GRAND PRIX CSI*5 W round A	25.11.2017	CSI-W5*	3	1.60	430	181	12	20.03.2006	G	ZANG	214	6	81	76	33	1923	71	M	15	12c	yes	vertical	vertical	left	no	0	0	71,13	no
Madrid	MUTUA MADRILEÑA TROPHY GRAND PRIX CSI*5 W round A	25.11.2017	CSI-W5*	3	1.60	430	181	12	20.03.2006	G	ZANG	214	6	81	76	33	1923	71	M	16	13	no	spread	oxer	right	no	0	0	71,13	no
Madrid	MUTUA MADRILEÑA TROPHY GRAND PRIX CSI*5 W round A	25.11.2017	CSI-W5*	3	1.60	430	131	10	28.03.2008	G	BWP	151	17	85	41	41	2052	191	M	1	1	no	spread	oxer	left	no	0	0	69,49	no
Madrid	MUTUA MADRILEÑA TROPHY GRAND PRIX CSI*5 W round A	25.11.2017	CSI-W5*	3	1.60	430	131	10	28.03.2008	G	BWP	151	17	85	41	41	2052	191	M	2	2	no	vertical	vertical	straight	no	0	0	69,49	no
Madrid	MUTUA MADRILEÑA TROPHY GRAND PRIX CSI*5 W round A	25.11.2017	CSI-W5*	3	1.60	430	131	10	28.03.2008	G	BWP	151	17	85	41	41	2052	191	M	3	3	no	spread	oxer	right	yes	4	0	69,49	no
Madrid	MUTUA MADRILEÑA TROPHY GRAND PRIX CSI*5 W round A	25.11.2017	CSI-W5*	3	1.60	430	131	10	28.03.2008	G	BWP	151	17	85	41	41	2052	191	M	4	4	no	vertical	vertical	straight	no	0	0	69,49	no
Madrid	MUTUA MADRILEÑA TROPHY GRAND PRIX CSI*5 W round A	25.11.2017	CSI-W5*	3	1.60	430	131	10	28.03.2008	G	BWP	151	17	85	41	41	2052	191	M	5	5	no	spread	oxer	left	no	0	0	69,49	no
Madrid	MUTUA MADRILEÑA TROPHY GRAND PRIX CSI*5 W round A	25.11.2017	CSI-W5*	3	1.60	430	131	10	28.03.2008	G	BWP	151	17	85	41	41	2052	191	M	6	6	no	vertical	vertical	left	no	0	0	69,49	no
Madrid	MUTUA MADRILEÑA TROPHY GRAND PRIX CSI*5 W round A	25.11.2017	CSI-W5*	3	1.60	430	131	10	28.03.2008	G	BWP	151	17	85	41	41	2052	191	M	7	7a	yes	spread	oxer	right	yes	4	0	69,49	no
Madrid	MUTUA MADRILEÑA TROPHY GRAND PRIX CSI*5 W round A	25.11.2017	CSI-W5*	3	1.60	430	131	10	28.03.2008	G	BWP	151	17	85	41	41	2052	191	M	8	7b	yes	vertical	vertical	right	no	0	0	69,49	no
Madrid	MUTUA MADRILEÑA TROPHY GRAND PRIX CSI*5 W round A	25.11.2017	CSI-W5*	3	1.60	430	131	10	28.03.2008	G	BWP	151	17	85	41	41	2052	191	M	9	8	no	spread	oxer	right	no	0	0	69,49	no
Madrid	MUTUA MADRILEÑA TROPHY GRAND PRIX CSI*5 W round A	25.11.2017	CSI-W5*	3	1.60	430	131	10	28.03.2008	G	BWP	151	17	85	41	41	2052	191	M	10	9	no	vertical	vertical	right	no	0	0	69,49	no
Madrid	MUTUA MADRILEÑA TROPHY GRAND PRIX CSI*5 W round A	25.11.2017	CSI-W5*	3	1.60	430	131	10	28.03.2008	G	BWP	151	17	85	41	41	2052	191	M	11	10	no	spread	oxer	straight	no	0	0	69,49	no
Madrid	MUTUA MADRILEÑA TROPHY GRAND PRIX CSI*5 W round A	25.11.2017	CSI-W5*	3	1.60	430	131	10	28.03.2008	G	BWP	151	17	85	41	41	2052	191	M	12	11	no	vertical	vertical with water	left	no	0	0	69,49	no
Madrid	MUTUA MADRILEÑA TROPHY GRAND PRIX CSI*5 W round A	25.11.2017	CSI-W5*	3	1.60	430	131	10	28.03.2008	G	BWP	151	17	85	41	41	2052	191	M	13	12a	yes	vertical	vertical	left	no	0	0	69,49	no
Madrid	MUTUA MADRILEÑA TROPHY GRAND PRIX CSI*5 W round A	25.11.2017	CSI-W5*	3	1.60	430	131	10	28.03.2008	G	BWP	151	17	85	41	41	2052	191	M	14	12b	yes	spread	oxer with water	left	no	0	0	69,49	no
Madrid	MUTUA MADRILEÑA TROPHY GRAND PRIX CSI*5 W round A	25.11.2017	CSI-W5*	3	1.60	430	131	10	28.03.2008	G	BWP	151	17	85	41	41	2052	191	M	15	12c	yes	vertical	vertical	left	no	0	0	69,49	no
Madrid	MUTUA MADRILEÑA TROPHY GRAND PRIX CSI*5 W round A	25.11.2017	CSI-W5*	3	1.60	430	131	10	28.03.2008	G	BWP	151	17	85	41	41	2052	191	M	16	13	no	spread	oxer	right	no	0	0	69,49	no
Madrid	MUTUA MADRILEÑA TROPHY GRAND PRIX CSI*5 W round A	25.11.2017	CSI-W5*	3	1.60	430	168	9	25.03.2009	G	HOLST	89	2	91	93	45	1436	65	M	1	1	no	spread	oxer	left	no	0	0	73,73	no
Madrid	MUTUA MADRILEÑA TROPHY GRAND PRIX CSI*5 W round A	25.11.2017	CSI-W5*	3	1.60	430	168	9	25.03.2009	G	HOLST	89	2	91	93	45	1436	65	M	2	2	no	vertical	vertical	straight	no	0	0	73,73	no
Madrid	MUTUA MADRILEÑA TROPHY GRAND PRIX CSI*5 W round A	25.11.2017	CSI-W5*	3	1.60	430	168	9	25.03.2009	G	HOLST	89	2	91	93	45	1436	65	M	3	3	no	spread	oxer	right	no	0	0	73,73	no
Madrid	MUTUA MADRILEÑA TROPHY GRAND PRIX CSI*5 W round A	25.11.2017	CSI-W5*	3	1.60	430	168	9	25.03.2009	G	HOLST	89	2	91	93	45	1436	65	M	4	4	no	vertical	vertical	straight	no	0	0	73,73	no
Madrid	MUTUA MADRILEÑA TROPHY GRAND PRIX CSI*5 W round A	25.11.2017	CSI-W5*	3	1.60	430	168	9	25.03.2009	G	HOLST	89	2	91	93	45	1436	65	M	5	5	no	spread	oxer	left	no	0	0	73,73	no
Madrid	MUTUA MADRILEÑA TROPHY GRAND PRIX CSI*5 W round A	25.11.2017	CSI-W5*	3	1.60	430	168	9	25.03.2009	G	HOLST	89	2	91	93	45	1436	65	M	6	6	no	vertical	vertical	left	no	0	0	73,73	no
Madrid	MUTUA MADRILEÑA TROPHY GRAND PRIX CSI*5 W round A	25.11.2017	CSI-W5*	3	1.60	430	168	9	25.03.2009	G	HOLST	89	2	91	93	45	1436	65	M	7	7a	yes	spread	oxer	right	no	0	0	73,73	no
Madrid	MUTUA MADRILEÑA TROPHY GRAND PRIX CSI*5 W round A	25.11.2017	CSI-W5*	3	1.60	430	168	9	25.03.2009	G	HOLST	89	2	91	93	45	1436	65	M	8	7b	yes	vertical	vertical	right	no	0	0	73,73	no
Madrid	MUTUA MADRILEÑA TROPHY GRAND PRIX CSI*5 W round A	25.11.2017	CSI-W5*	3	1.60	430	168	9	25.03.2009	G	HOLST	89	2	91	93	45	1436	65	M	9	8	no	spread	oxer	right	no	0	0	73,73	no
Madrid	MUTUA MADRILEÑA TROPHY GRAND PRIX CSI*5 W round A	25.11.2017	CSI-W5*	3	1.60	430	168	9	25.03.2009	G	HOLST	89	2	91	93	45	1436	65	M	10	9	no	vertical	vertical	right	no	0	0	73,73	no
Madrid	MUTUA MADRILEÑA TROPHY GRAND PRIX CSI*5 W round A	25.11.2017	CSI-W5*	3	1.60	430	168	9	25.03.2009	G	HOLST	89	2	91	93	45	1436	65	M	11	10	no	spread	oxer	straight	no	0	0	73,73	no
Madrid	MUTUA MADRILEÑA TROPHY GRAND PRIX CSI*5 W round A	25.11.2017	CSI-W5*	3	1.60	430	168	9	25.03.2009	G	HOLST	89	2	91	93	45	1436	65	M	12	11	no	vertical	vertical with water	left	no	0	0	73,73	no
Madrid	MUTUA MADRILEÑA TROPHY GRAND PRIX CSI*5 W round A	25.11.2017	CSI-W5*	3	1.60	430	168	9	25.03.2009	G	HOLST	89	2	91	93	45	1436	65	M	13	12a	yes	vertical	vertical	left	no	0	0	73,73	no
Madrid	MUTUA MADRILEÑA TROPHY GRAND PRIX CSI*5 W round A	25.11.2017	CSI-W5*	3	1.60	430	168	9	25.03.2009	G	HOLST	89	2	91	93	45	1436	65	M	14	12b	yes	spread	oxer with water	left	no	0	0	73,73	no
Madrid	MUTUA MADRILEÑA TROPHY GRAND PRIX CSI*5 W round A	25.11.2017	CSI-W5*	3	1.60	430	168	9	25.03.2009	G	HOLST	89	2	91	93	45	1436	65	M	15	12c	yes	vertical	vertical	left	no	0	0	73,73	no
Madrid	MUTUA MADRILEÑA TROPHY GRAND PRIX CSI*5 W round A	25.11.2017	CSI-W5*	3	1.60	430	168	9	25.03.2009	G	HOLST	89	2	91	93	45	1436	65	M	16	13	no	spread	oxer	right	no	0	0	73,73	no
Madrid	MUTUA MADRILEÑA TROPHY GRAND PRIX CSI*5 W round B	25.11.2017	CSI-W5*	3	1.60	300	168	9	25.03.2009	G	HOLST	89	2	91	93	45	1436	65	M	1	1	no	spread	oxer	right	no	0	0	47,83	no
Madrid	MUTUA MADRILEÑA TROPHY GRAND PRIX CSI*5 W round B	25.11.2017	CSI-W5*	3	1.60	300	168	9	25.03.2009	G	HOLST	89	2	91	93	45	1436	65	M	2	2	no	spread	oxer	right	no	0	0	47,83	no
Madrid	MUTUA MADRILEÑA TROPHY GRAND PRIX CSI*5 W round B	25.11.2017	CSI-W5*	3	1.60	300	168	9	25.03.2009	G	HOLST	89	2	91	93	45	1436	65	M	3	3	no	vertical	vertical with water	left	yes	4	0	47,83	no
Madrid	MUTUA MADRILEÑA TROPHY GRAND PRIX CSI*5 W round B	25.11.2017	CSI-W5*	3	1.60	300	168	9	25.03.2009	G	HOLST	89	2	91	93	45	1436	65	M	4	4a	yes	vertical	vertical	left	no	0	0	47,83	no
Madrid	MUTUA MADRILEÑA TROPHY GRAND PRIX CSI*5 W round B	25.11.2017	CSI-W5*	3	1.60	300	168	9	25.03.2009	G	HOLST	89	2	91	93	45	1436	65	M	5	4b	yes	spread	oxer with water	left	no	0	0	47,83	no
Madrid	MUTUA MADRILEÑA TROPHY GRAND PRIX CSI*5 W round B	25.11.2017	CSI-W5*	3	1.60	300	168	9	25.03.2009	G	HOLST	89	2	91	93	45	1436	65	M	6	5	no	spread	oxer	right	no	0	0	47,83	no
Madrid	MUTUA MADRILEÑA TROPHY GRAND PRIX CSI*5 W round B	25.11.2017	CSI-W5*	3	1.60	300	168	9	25.03.2009	G	HOLST	89	2	91	93	45	1436	65	M	7	6	no	vertical	vertical	right	no	0	0	47,83	no
Madrid	MUTUA MADRILEÑA TROPHY GRAND PRIX CSI*5 W round B	25.11.2017	CSI-W5*	3	1.60	300	168	9	25.03.2009	G	HOLST	89	2	91	93	45	1436	65	M	8	7	no	vertical	vertical	left	no	0	0	47,83	no
Madrid	MUTUA MADRILEÑA TROPHY GRAND PRIX CSI*5 W round B	25.11.2017	CSI-W5*	3	1.60	300	168	9	25.03.2009	G	HOLST	89	2	91	93	45	1436	65	M	9	8	no	spread	oxer	left	no	0	0	47,83	no
Madrid	MUTUA MADRILEÑA TROPHY GRAND PRIX CSI*5 W round A	25.11.2017	CSI-W5*	3	1.60	430	118	12	26.01.2006	G	OS	262	11	96	97	55	1508	53	M	1	1	no	spread	oxer	left	no	0	0	72,6	no
Madrid	MUTUA MADRILEÑA TROPHY GRAND PRIX CSI*5 W round A	25.11.2017	CSI-W5*	3	1.60	430	118	12	26.01.2006	G	OS	262	11	96	97	55	1508	53	M	2	2	no	vertical	vertical	straight	no	0	0	72,6	no
Madrid	MUTUA MADRILEÑA TROPHY GRAND PRIX CSI*5 W round A	25.11.2017	CSI-W5*	3	1.60	430	118	12	26.01.2006	G	OS	262	11	96	97	55	1508	53	M	3	3	no	spread	oxer	right	no	0	0	72,6	no
Madrid	MUTUA MADRILEÑA TROPHY GRAND PRIX CSI*5 W round A	25.11.2017	CSI-W5*	3	1.60	430	118	12	26.01.2006	G	OS	262	11	96	97	55	1508	53	M	4	4	no	vertical	vertical	straight	no	0	0	72,6	no
Madrid	MUTUA MADRILEÑA TROPHY GRAND PRIX CSI*5 W round A	25.11.2017	CSI-W5*	3	1.60	430	118	12	26.01.2006	G	OS	262	11	96	97	55	1508	53	M	5	5	no	spread	oxer	left	no	0	0	72,6	no
Madrid	MUTUA MADRILEÑA TROPHY GRAND PRIX CSI*5 W round A	25.11.2017	CSI-W5*	3	1.60	430	118	12	26.01.2006	G	OS	262	11	96	97	55	1508	53	M	6	6	no	vertical	vertical	left	no	0	0	72,6	no
Madrid	MUTUA MADRILEÑA TROPHY GRAND PRIX CSI*5 W round A	25.11.2017	CSI-W5*	3	1.60	430	118	12	26.01.2006	G	OS	262	11	96	97	55	1508	53	M	7	7a	yes	spread	oxer	right	no	0	0	72,6	no
Madrid	MUTUA MADRILEÑA TROPHY GRAND PRIX CSI*5 W round A	25.11.2017	CSI-W5*	3	1.60	430	118	12	26.01.2006	G	OS	262	11	96	97	55	1508	53	M	8	7b	yes	vertical	vertical	right	no	0	0	72,6	no
Madrid	MUTUA MADRILEÑA TROPHY GRAND PRIX CSI*5 W round A	25.11.2017	CSI-W5*	3	1.60	430	118	12	26.01.2006	G	OS	262	11	96	97	55	1508	53	M	9	8	no	spread	oxer	right	no	0	0	72,6	no
Madrid	MUTUA MADRILEÑA TROPHY GRAND PRIX CSI*5 W round A	25.11.2017	CSI-W5*	3	1.60	430	118	12	26.01.2006	G	OS	262	11	96	97	55	1508	53	M	10	9	no	vertical	vertical	right	yes	4	0	72,6	no
Madrid	MUTUA MADRILEÑA TROPHY GRAND PRIX CSI*5 W round A	25.11.2017	CSI-W5*	3	1.60	430	118	12	26.01.2006	G	OS	262	11	96	97	55	1508	53	M	11	10	no	spread	oxer	straight	no	0	0	72,6	no
Madrid	MUTUA MADRILEÑA TROPHY GRAND PRIX CSI*5 W round A	25.11.2017	CSI-W5*	3	1.60	430	118	12	26.01.2006	G	OS	262	11	96	97	55	1508	53	M	12	11	no	vertical	vertical with water	left	no	0	0	72,6	no
Madrid	MUTUA MADRILEÑA TROPHY GRAND PRIX CSI*5 W round A	25.11.2017	CSI-W5*	3	1.60	430	118	12	26.01.2006	G	OS	262	11	96	97	55	1508	53	M	13	12a	yes	vertical	vertical	left	no	0	0	72,6	no
Madrid	MUTUA MADRILEÑA TROPHY GRAND PRIX CSI*5 W round A	25.11.2017	CSI-W5*	3	1.60	430	118	12	26.01.2006	G	OS	262	11	96	97	55	1508	53	M	14	12b	yes	spread	oxer with water	left	no	0	0	72,6	no
Madrid	MUTUA MADRILEÑA TROPHY GRAND PRIX CSI*5 W round A	25.11.2017	CSI-W5*	3	1.60	430	118	12	26.01.2006	G	OS	262	11	96	97	55	1508	53	M	15	12c	yes	vertical	vertical	left	no	0	0	72,6	no
Madrid	MUTUA MADRILEÑA TROPHY GRAND PRIX CSI*5 W round A	25.11.2017	CSI-W5*	3	1.60	430	118	12	26.01.2006	G	OS	262	11	96	97	55	1508	53	M	16	13	no	spread	oxer	right	no	0	0	72,6	no
Madrid	MUTUA MADRILEÑA TROPHY GRAND PRIX CSI*5 W round A	25.11.2017	CSI-W5*	3	1.60	430	114	13	22.05.2005	G	OS	124	3	98	187	43	919	24	M	1	1	no	spread	oxer	left	no	0	0	75,64	no
Madrid	MUTUA MADRILEÑA TROPHY GRAND PRIX CSI*5 W round A	25.11.2017	CSI-W5*	3	1.60	430	114	13	22.05.2005	G	OS	124	3	98	187	43	919	24	M	2	2	no	vertical	vertical	straight	no	0	0	75,64	no
Madrid	MUTUA MADRILEÑA TROPHY GRAND PRIX CSI*5 W round A	25.11.2017	CSI-W5*	3	1.60	430	114	13	22.05.2005	G	OS	124	3	98	187	43	919	24	M	3	3	no	spread	oxer	right	no	0	0	75,64	no
Madrid	MUTUA MADRILEÑA TROPHY GRAND PRIX CSI*5 W round A	25.11.2017	CSI-W5*	3	1.60	430	114	13	22.05.2005	G	OS	124	3	98	187	43	919	24	M	4	4	no	vertical	vertical	straight	no	0	0	75,64	no
Madrid	MUTUA MADRILEÑA TROPHY GRAND PRIX CSI*5 W round A	25.11.2017	CSI-W5*	3	1.60	430	114	13	22.05.2005	G	OS	124	3	98	187	43	919	24	M	5	5	no	spread	oxer	left	no	0	0	75,64	no
Madrid	MUTUA MADRILEÑA TROPHY GRAND PRIX CSI*5 W round A	25.11.2017	CSI-W5*	3	1.60	430	114	13	22.05.2005	G	OS	124	3	98	187	43	919	24	M	6	6	no	vertical	vertical	left	no	0	0	75,64	no
Madrid	MUTUA MADRILEÑA TROPHY GRAND PRIX CSI*5 W round A	25.11.2017	CSI-W5*	3	1.60	430	114	13	22.05.2005	G	OS	124	3	98	187	43	919	24	M	7	7a	yes	spread	oxer	right	no	0	0	75,64	no
Madrid	MUTUA MADRILEÑA TROPHY GRAND PRIX CSI*5 W round A	25.11.2017	CSI-W5*	3	1.60	430	114	13	22.05.2005	G	OS	124	3	98	187	43	919	24	M	8	7b	yes	vertical	vertical	right	no	0	0	75,64	no
Madrid	MUTUA MADRILEÑA TROPHY GRAND PRIX CSI*5 W round A	25.11.2017	CSI-W5*	3	1.60	430	114	13	22.05.2005	G	OS	124	3	98	187	43	919	24	M	9	8	no	spread	oxer	right	no	0	0	75,64	no
Madrid	MUTUA MADRILEÑA TROPHY GRAND PRIX CSI*5 W round A	25.11.2017	CSI-W5*	3	1.60	430	114	13	22.05.2005	G	OS	124	3	98	187	43	919	24	M	10	9	no	vertical	vertical	right	no	0	0	75,64	no
Madrid	MUTUA MADRILEÑA TROPHY GRAND PRIX CSI*5 W round A	25.11.2017	CSI-W5*	3	1.60	430	114	13	22.05.2005	G	OS	124	3	98	187	43	919	24	M	11	10	no	spread	oxer	straight	no	0	0	75,64	no
Madrid	MUTUA MADRILEÑA TROPHY GRAND PRIX CSI*5 W round A	25.11.2017	CSI-W5*	3	1.60	430	114	13	22.05.2005	G	OS	124	3	98	187	43	919	24	M	12	11	no	vertical	vertical with water	left	no	0	0	75,64	no
Madrid	MUTUA MADRILEÑA TROPHY GRAND PRIX CSI*5 W round A	25.11.2017	CSI-W5*	3	1.60	430	114	13	22.05.2005	G	OS	124	3	98	187	43	919	24	M	13	12a	yes	vertical	vertical	left	no	0	0	75,64	no
Madrid	MUTUA MADRILEÑA TROPHY GRAND PRIX CSI*5 W round A	25.11.2017	CSI-W5*	3	1.60	430	114	13	22.05.2005	G	OS	124	3	98	187	43	919	24	M	14	12b	yes	spread	oxer with water	left	no	0	0	75,64	no
Madrid	MUTUA MADRILEÑA TROPHY GRAND PRIX CSI*5 W round A	25.11.2017	CSI-W5*	3	1.60	430	114	13	22.05.2005	G	OS	124	3	98	187	43	919	24	M	15	12c	yes	vertical	vertical	left	no	0	0	75,64	no
Madrid	MUTUA MADRILEÑA TROPHY GRAND PRIX CSI*5 W round A	25.11.2017	CSI-W5*	3	1.60	430	114	13	22.05.2005	G	OS	124	3	98	187	43	919	24	M	16	13	no	spread	oxer	right	no	0	1	75,64	no
Madrid	MUTUA MADRILEÑA TROPHY GRAND PRIX CSI*5 W round A	25.11.2017	CSI-W5*	3	1.60	430	81	12	01.01.2006	M	CDE	78	2	99	1060	53	252	5	M	1	1	no	spread	oxer	left	no	0	0	74,4	no
Madrid	MUTUA MADRILEÑA TROPHY GRAND PRIX CSI*5 W round A	25.11.2017	CSI-W5*	3	1.60	430	81	12	01.01.2006	M	CDE	78	2	99	1060	53	252	5	M	2	2	no	vertical	vertical	straight	no	0	0	74,4	no
Madrid	MUTUA MADRILEÑA TROPHY GRAND PRIX CSI*5 W round A	25.11.2017	CSI-W5*	3	1.60	430	81	12	01.01.2006	M	CDE	78	2	99	1060	53	252	5	M	3	3	no	spread	oxer	right	no	0	0	74,4	no
Madrid	MUTUA MADRILEÑA TROPHY GRAND PRIX CSI*5 W round A	25.11.2017	CSI-W5*	3	1.60	430	81	12	01.01.2006	M	CDE	78	2	99	1060	53	252	5	M	4	4	no	vertical	vertical	straight	no	0	0	74,4	no
Madrid	MUTUA MADRILEÑA TROPHY GRAND PRIX CSI*5 W round A	25.11.2017	CSI-W5*	3	1.60	430	81	12	01.01.2006	M	CDE	78	2	99	1060	53	252	5	M	5	5	no	spread	oxer	left	no	0	0	74,4	no
Madrid	MUTUA MADRILEÑA TROPHY GRAND PRIX CSI*5 W round A	25.11.2017	CSI-W5*	3	1.60	430	81	12	01.01.2006	M	CDE	78	2	99	1060	53	252	5	M	6	6	no	vertical	vertical	left	no	0	0	74,4	no
Madrid	MUTUA MADRILEÑA TROPHY GRAND PRIX CSI*5 W round A	25.11.2017	CSI-W5*	3	1.60	430	81	12	01.01.2006	M	CDE	78	2	99	1060	53	252	5	M	7	7a	yes	spread	oxer	right	no	0	0	74,4	no
Madrid	MUTUA MADRILEÑA TROPHY GRAND PRIX CSI*5 W round A	25.11.2017	CSI-W5*	3	1.60	430	81	12	01.01.2006	M	CDE	78	2	99	1060	53	252	5	M	8	7b	yes	vertical	vertical	right	no	0	0	74,4	no
Madrid	MUTUA MADRILEÑA TROPHY GRAND PRIX CSI*5 W round A	25.11.2017	CSI-W5*	3	1.60	430	81	12	01.01.2006	M	CDE	78	2	99	1060	53	252	5	M	9	8	no	spread	oxer	right	no	0	0	74,4	no
Madrid	MUTUA MADRILEÑA TROPHY GRAND PRIX CSI*5 W round A	25.11.2017	CSI-W5*	3	1.60	430	81	12	01.01.2006	M	CDE	78	2	99	1060	53	252	5	M	10	9	no	vertical	vertical	right	yes	4	0	74,4	no
Madrid	MUTUA MADRILEÑA TROPHY GRAND PRIX CSI*5 W round A	25.11.2017	CSI-W5*	3	1.60	430	81	12	01.01.2006	M	CDE	78	2	99	1060	53	252	5	M	11	10	no	spread	oxer	straight	no	0	0	74,4	no
Madrid	MUTUA MADRILEÑA TROPHY GRAND PRIX CSI*5 W round A	25.11.2017	CSI-W5*	3	1.60	430	81	12	01.01.2006	M	CDE	78	2	99	1060	53	252	5	M	12	11	no	vertical	vertical with water	left	no	0	0	74,4	no
Madrid	MUTUA MADRILEÑA TROPHY GRAND PRIX CSI*5 W round A	25.11.2017	CSI-W5*	3	1.60	430	81	12	01.01.2006	M	CDE	78	2	99	1060	53	252	5	M	13	12a	yes	vertical	vertical	left	yes	4	0	74,4	no
Madrid	MUTUA MADRILEÑA TROPHY GRAND PRIX CSI*5 W round A	25.11.2017	CSI-W5*	3	1.60	430	81	12	01.01.2006	M	CDE	78	2	99	1060	53	252	5	M	14	12b	yes	spread	oxer with water	left	yes	4	0	74,4	no
Madrid	MUTUA MADRILEÑA TROPHY GRAND PRIX CSI*5 W round A	25.11.2017	CSI-W5*	3	1.60	430	81	12	01.01.2006	M	CDE	78	2	99	1060	53	252	5	M	15	12c	yes	vertical	vertical	left	no	0	0	74,4	no
Madrid	MUTUA MADRILEÑA TROPHY GRAND PRIX CSI*5 W round A	25.11.2017	CSI-W5*	3	1.60	430	81	12	01.01.2006	M	CDE	78	2	99	1060	53	252	5	M	16	13	no	spread	oxer	right	no	0	1	74,4	no
Madrid	MUTUA MADRILEÑA TROPHY GRAND PRIX CSI*5 W round A	25.11.2017	CSI-W5*	3	1.60	430	167	14	01.01.2004	G	SF	159	4	100	623	28	760	37	F	1	1	no	spread	oxer	left	no	0	0	73,59	no
Madrid	MUTUA MADRILEÑA TROPHY GRAND PRIX CSI*5 W round A	25.11.2017	CSI-W5*	3	1.60	430	167	14	01.01.2004	G	SF	159	4	100	623	28	760	37	F	2	2	no	vertical	vertical	straight	no	0	0	73,59	no
Madrid	MUTUA MADRILEÑA TROPHY GRAND PRIX CSI*5 W round A	25.11.2017	CSI-W5*	3	1.60	430	167	14	01.01.2004	G	SF	159	4	100	623	28	760	37	F	3	3	no	spread	oxer	right	no	0	0	73,59	no
Madrid	MUTUA MADRILEÑA TROPHY GRAND PRIX CSI*5 W round A	25.11.2017	CSI-W5*	3	1.60	430	167	14	01.01.2004	G	SF	159	4	100	623	28	760	37	F	4	4	no	vertical	vertical	straight	no	0	0	73,59	no
Madrid	MUTUA MADRILEÑA TROPHY GRAND PRIX CSI*5 W round A	25.11.2017	CSI-W5*	3	1.60	430	167	14	01.01.2004	G	SF	159	4	100	623	28	760	37	F	5	5	no	spread	oxer	left	no	0	0	73,59	no
Madrid	MUTUA MADRILEÑA TROPHY GRAND PRIX CSI*5 W round A	25.11.2017	CSI-W5*	3	1.60	430	167	14	01.01.2004	G	SF	159	4	100	623	28	760	37	F	6	6	no	vertical	vertical	left	yes	4	0	73,59	no
Madrid	MUTUA MADRILEÑA TROPHY GRAND PRIX CSI*5 W round A	25.11.2017	CSI-W5*	3	1.60	430	167	14	01.01.2004	G	SF	159	4	100	623	28	760	37	F	7	7a	yes	spread	oxer	right	no	0	0	73,59	no
Madrid	MUTUA MADRILEÑA TROPHY GRAND PRIX CSI*5 W round A	25.11.2017	CSI-W5*	3	1.60	430	167	14	01.01.2004	G	SF	159	4	100	623	28	760	37	F	8	7b	yes	vertical	vertical	right	no	0	0	73,59	no
Madrid	MUTUA MADRILEÑA TROPHY GRAND PRIX CSI*5 W round A	25.11.2017	CSI-W5*	3	1.60	430	167	14	01.01.2004	G	SF	159	4	100	623	28	760	37	F	9	8	no	spread	oxer	right	no	0	0	73,59	no
Madrid	MUTUA MADRILEÑA TROPHY GRAND PRIX CSI*5 W round A	25.11.2017	CSI-W5*	3	1.60	430	167	14	01.01.2004	G	SF	159	4	100	623	28	760	37	F	10	9	no	vertical	vertical	right	no	0	0	73,59	no
Madrid	MUTUA MADRILEÑA TROPHY GRAND PRIX CSI*5 W round A	25.11.2017	CSI-W5*	3	1.60	430	167	14	01.01.2004	G	SF	159	4	100	623	28	760	37	F	11	10	no	spread	oxer	straight	yes	4	0	73,59	no
Madrid	MUTUA MADRILEÑA TROPHY GRAND PRIX CSI*5 W round A	25.11.2017	CSI-W5*	3	1.60	430	167	14	01.01.2004	G	SF	159	4	100	623	28	760	37	F	12	11	no	vertical	vertical with water	left	no	0	0	73,59	no
Madrid	MUTUA MADRILEÑA TROPHY GRAND PRIX CSI*5 W round A	25.11.2017	CSI-W5*	3	1.60	430	167	14	01.01.2004	G	SF	159	4	100	623	28	760	37	F	13	12a	yes	vertical	vertical	left	yes	4	0	73,59	no
Madrid	MUTUA MADRILEÑA TROPHY GRAND PRIX CSI*5 W round A	25.11.2017	CSI-W5*	3	1.60	430	167	14	01.01.2004	G	SF	159	4	100	623	28	760	37	F	14	12b	yes	spread	oxer with water	left	no	0	0	73,59	no
Madrid	MUTUA MADRILEÑA TROPHY GRAND PRIX CSI*5 W round A	25.11.2017	CSI-W5*	3	1.60	430	167	14	01.01.2004	G	SF	159	4	100	623	28	760	37	F	15	12c	yes	vertical	vertical	left	no	0	0	73,59	no
Madrid	MUTUA MADRILEÑA TROPHY GRAND PRIX CSI*5 W round A	25.11.2017	CSI-W5*	3	1.60	430	167	14	01.01.2004	G	SF	159	4	100	623	28	760	37	F	16	13	no	spread	oxer	right	no	0	0	73,59	no
Madrid	MUTUA MADRILEÑA TROPHY GRAND PRIX CSI*5 W round A	25.11.2017	CSI-W5*	3	1.60	430	2	13	17.04.2005	G	KWPN	274	23	101	46	53	686	44	M	1	1	no	spread	oxer	left	no	0	0	73,26	no
Madrid	MUTUA MADRILEÑA TROPHY GRAND PRIX CSI*5 W round A	25.11.2017	CSI-W5*	3	1.60	430	2	13	17.04.2005	G	KWPN	274	23	101	46	53	686	44	M	2	2	no	vertical	vertical	straight	no	0	0	73,26	no
Madrid	MUTUA MADRILEÑA TROPHY GRAND PRIX CSI*5 W round A	25.11.2017	CSI-W5*	3	1.60	430	2	13	17.04.2005	G	KWPN	274	23	101	46	53	686	44	M	3	3	no	spread	oxer	right	no	0	0	73,26	no
Madrid	MUTUA MADRILEÑA TROPHY GRAND PRIX CSI*5 W round A	25.11.2017	CSI-W5*	3	1.60	430	2	13	17.04.2005	G	KWPN	274	23	101	46	53	686	44	M	4	4	no	vertical	vertical	straight	no	0	0	73,26	no
Madrid	MUTUA MADRILEÑA TROPHY GRAND PRIX CSI*5 W round A	25.11.2017	CSI-W5*	3	1.60	430	2	13	17.04.2005	G	KWPN	274	23	101	46	53	686	44	M	5	5	no	spread	oxer	left	no	0	0	73,26	no
Madrid	MUTUA MADRILEÑA TROPHY GRAND PRIX CSI*5 W round A	25.11.2017	CSI-W5*	3	1.60	430	2	13	17.04.2005	G	KWPN	274	23	101	46	53	686	44	M	6	6	no	vertical	vertical	left	no	0	0	73,26	no
Madrid	MUTUA MADRILEÑA TROPHY GRAND PRIX CSI*5 W round A	25.11.2017	CSI-W5*	3	1.60	430	2	13	17.04.2005	G	KWPN	274	23	101	46	53	686	44	M	7	7a	yes	spread	oxer	right	no	0	0	73,26	no
Madrid	MUTUA MADRILEÑA TROPHY GRAND PRIX CSI*5 W round A	25.11.2017	CSI-W5*	3	1.60	430	2	13	17.04.2005	G	KWPN	274	23	101	46	53	686	44	M	8	7b	yes	vertical	vertical	right	no	0	0	73,26	no
Madrid	MUTUA MADRILEÑA TROPHY GRAND PRIX CSI*5 W round A	25.11.2017	CSI-W5*	3	1.60	430	2	13	17.04.2005	G	KWPN	274	23	101	46	53	686	44	M	9	8	no	spread	oxer	right	no	0	0	73,26	no
Madrid	MUTUA MADRILEÑA TROPHY GRAND PRIX CSI*5 W round A	25.11.2017	CSI-W5*	3	1.60	430	2	13	17.04.2005	G	KWPN	274	23	101	46	53	686	44	M	10	9	no	vertical	vertical	right	no	0	0	73,26	no
Madrid	MUTUA MADRILEÑA TROPHY GRAND PRIX CSI*5 W round A	25.11.2017	CSI-W5*	3	1.60	430	2	13	17.04.2005	G	KWPN	274	23	101	46	53	686	44	M	11	10	no	spread	oxer	straight	no	0	0	73,26	no
Madrid	MUTUA MADRILEÑA TROPHY GRAND PRIX CSI*5 W round A	25.11.2017	CSI-W5*	3	1.60	430	2	13	17.04.2005	G	KWPN	274	23	101	46	53	686	44	M	12	11	no	vertical	vertical with water	left	no	0	0	73,26	no
Madrid	MUTUA MADRILEÑA TROPHY GRAND PRIX CSI*5 W round A	25.11.2017	CSI-W5*	3	1.60	430	2	13	17.04.2005	G	KWPN	274	23	101	46	53	686	44	M	13	12a	yes	vertical	vertical	left	no	0	0	73,26	no
Madrid	MUTUA MADRILEÑA TROPHY GRAND PRIX CSI*5 W round A	25.11.2017	CSI-W5*	3	1.60	430	2	13	17.04.2005	G	KWPN	274	23	101	46	53	686	44	M	14	12b	yes	spread	oxer with water	left	no	0	0	73,26	no
Madrid	MUTUA MADRILEÑA TROPHY GRAND PRIX CSI*5 W round A	25.11.2017	CSI-W5*	3	1.60	430	2	13	17.04.2005	G	KWPN	274	23	101	46	53	686	44	M	15	12c	yes	vertical	vertical	left	no	0	0	73,26	no
Madrid	MUTUA MADRILEÑA TROPHY GRAND PRIX CSI*5 W round A	25.11.2017	CSI-W5*	3	1.60	430	2	13	17.04.2005	G	KWPN	274	23	101	46	53	686	44	M	16	13	no	spread	oxer	right	no	0	0	73,26	no
Madrid	MUTUA MADRILEÑA TROPHY GRAND PRIX CSI*5 W round B	25.11.2017	CSI-W5*	3	1.60	300	2	13	17.04.2005	G	KWPN	274	23	101	46	53	686	44	M	1	1	no	spread	oxer	right	no	0	0	43,77	no
Madrid	MUTUA MADRILEÑA TROPHY GRAND PRIX CSI*5 W round B	25.11.2017	CSI-W5*	3	1.60	300	2	13	17.04.2005	G	KWPN	274	23	101	46	53	686	44	M	2	2	no	spread	oxer	right	no	0	0	43,77	no
Madrid	MUTUA MADRILEÑA TROPHY GRAND PRIX CSI*5 W round B	25.11.2017	CSI-W5*	3	1.60	300	2	13	17.04.2005	G	KWPN	274	23	101	46	53	686	44	M	3	3	no	vertical	vertical with water	left	no	0	0	43,77	no
Madrid	MUTUA MADRILEÑA TROPHY GRAND PRIX CSI*5 W round B	25.11.2017	CSI-W5*	3	1.60	300	2	13	17.04.2005	G	KWPN	274	23	101	46	53	686	44	M	4	4a	yes	vertical	vertical	left	no	0	0	43,77	no
Madrid	MUTUA MADRILEÑA TROPHY GRAND PRIX CSI*5 W round B	25.11.2017	CSI-W5*	3	1.60	300	2	13	17.04.2005	G	KWPN	274	23	101	46	53	686	44	M	5	4b	yes	spread	oxer with water	left	no	0	0	43,77	no
Madrid	MUTUA MADRILEÑA TROPHY GRAND PRIX CSI*5 W round B	25.11.2017	CSI-W5*	3	1.60	300	2	13	17.04.2005	G	KWPN	274	23	101	46	53	686	44	M	6	5	no	spread	oxer	right	no	0	0	43,77	no
Madrid	MUTUA MADRILEÑA TROPHY GRAND PRIX CSI*5 W round B	25.11.2017	CSI-W5*	3	1.60	300	2	13	17.04.2005	G	KWPN	274	23	101	46	53	686	44	M	7	6	no	vertical	vertical	right	no	0	0	43,77	no
Madrid	MUTUA MADRILEÑA TROPHY GRAND PRIX CSI*5 W round B	25.11.2017	CSI-W5*	3	1.60	300	2	13	17.04.2005	G	KWPN	274	23	101	46	53	686	44	M	8	7	no	vertical	vertical	left	no	0	0	43,77	no
Madrid	MUTUA MADRILEÑA TROPHY GRAND PRIX CSI*5 W round B	25.11.2017	CSI-W5*	3	1.60	300	2	13	17.04.2005	G	KWPN	274	23	101	46	53	686	44	M	9	8	no	spread	oxer	left	no	0	0	43,77	no
Madrid	MUTUA MADRILEÑA TROPHY GRAND PRIX CSI*5 W round A	25.11.2017	CSI-W5*	3	1.60	430	189	14	03.10.2004	S	ZANG	276	0	102	321	44	1305	3	F	1	1	no	spread	oxer	left	no	0	0	73,03	no
Madrid	MUTUA MADRILEÑA TROPHY GRAND PRIX CSI*5 W round A	25.11.2017	CSI-W5*	3	1.60	430	189	14	03.10.2004	S	ZANG	276	0	102	321	44	1305	3	F	2	2	no	vertical	vertical	straight	no	0	0	73,03	no
Madrid	MUTUA MADRILEÑA TROPHY GRAND PRIX CSI*5 W round A	25.11.2017	CSI-W5*	3	1.60	430	189	14	03.10.2004	S	ZANG	276	0	102	321	44	1305	3	F	3	3	no	spread	oxer	right	no	0	0	73,03	no
Madrid	MUTUA MADRILEÑA TROPHY GRAND PRIX CSI*5 W round A	25.11.2017	CSI-W5*	3	1.60	430	189	14	03.10.2004	S	ZANG	276	0	102	321	44	1305	3	F	4	4	no	vertical	vertical	straight	no	0	0	73,03	no
Madrid	MUTUA MADRILEÑA TROPHY GRAND PRIX CSI*5 W round A	25.11.2017	CSI-W5*	3	1.60	430	189	14	03.10.2004	S	ZANG	276	0	102	321	44	1305	3	F	5	5	no	spread	oxer	left	yes	4	0	73,03	no
Madrid	MUTUA MADRILEÑA TROPHY GRAND PRIX CSI*5 W round A	25.11.2017	CSI-W5*	3	1.60	430	189	14	03.10.2004	S	ZANG	276	0	102	321	44	1305	3	F	6	6	no	vertical	vertical	left	no	0	0	73,03	no
Madrid	MUTUA MADRILEÑA TROPHY GRAND PRIX CSI*5 W round A	25.11.2017	CSI-W5*	3	1.60	430	189	14	03.10.2004	S	ZANG	276	0	102	321	44	1305	3	F	7	7a	yes	spread	oxer	right	no	0	0	73,03	no
Madrid	MUTUA MADRILEÑA TROPHY GRAND PRIX CSI*5 W round A	25.11.2017	CSI-W5*	3	1.60	430	189	14	03.10.2004	S	ZANG	276	0	102	321	44	1305	3	F	8	7b	yes	vertical	vertical	right	no	0	0	73,03	no
Madrid	MUTUA MADRILEÑA TROPHY GRAND PRIX CSI*5 W round A	25.11.2017	CSI-W5*	3	1.60	430	189	14	03.10.2004	S	ZANG	276	0	102	321	44	1305	3	F	9	8	no	spread	oxer	right	no	0	0	73,03	no
Madrid	MUTUA MADRILEÑA TROPHY GRAND PRIX CSI*5 W round A	25.11.2017	CSI-W5*	3	1.60	430	189	14	03.10.2004	S	ZANG	276	0	102	321	44	1305	3	F	10	9	no	vertical	vertical	right	no	0	0	73,03	no
Madrid	MUTUA MADRILEÑA TROPHY GRAND PRIX CSI*5 W round A	25.11.2017	CSI-W5*	3	1.60	430	189	14	03.10.2004	S	ZANG	276	0	102	321	44	1305	3	F	11	10	no	spread	oxer	straight	no	0	0	73,03	no
Madrid	MUTUA MADRILEÑA TROPHY GRAND PRIX CSI*5 W round A	25.11.2017	CSI-W5*	3	1.60	430	189	14	03.10.2004	S	ZANG	276	0	102	321	44	1305	3	F	12	11	no	vertical	vertical with water	left	no	0	0	73,03	no
Madrid	MUTUA MADRILEÑA TROPHY GRAND PRIX CSI*5 W round A	25.11.2017	CSI-W5*	3	1.60	430	189	14	03.10.2004	S	ZANG	276	0	102	321	44	1305	3	F	13	12a	yes	vertical	vertical	left	yes	4	0	73,03	no
Madrid	MUTUA MADRILEÑA TROPHY GRAND PRIX CSI*5 W round A	25.11.2017	CSI-W5*	3	1.60	430	189	14	03.10.2004	S	ZANG	276	0	102	321	44	1305	3	F	14	12b	yes	spread	oxer with water	left	yes	4	0	73,03	no
Madrid	MUTUA MADRILEÑA TROPHY GRAND PRIX CSI*5 W round A	25.11.2017	CSI-W5*	3	1.60	430	189	14	03.10.2004	S	ZANG	276	0	102	321	44	1305	3	F	15	12c	yes	vertical	vertical	left	no	0	0	73,03	no
Madrid	MUTUA MADRILEÑA TROPHY GRAND PRIX CSI*5 W round A	25.11.2017	CSI-W5*	3	1.60	430	189	14	03.10.2004	S	ZANG	276	0	102	321	44	1305	3	F	16	13	no	spread	oxer	right	no	0	0	73,03	no
Madrid	MUTUA MADRILEÑA TROPHY GRAND PRIX CSI*5 W round A	25.11.2017	CSI-W5*	3	1.60	430	144	14	28.02.2004	G	DSP	186	2	103	171	32	1274	37	M	1	1	no	spread	oxer	left	no	0	0	72,41	no
Madrid	MUTUA MADRILEÑA TROPHY GRAND PRIX CSI*5 W round A	25.11.2017	CSI-W5*	3	1.60	430	144	14	28.02.2004	G	DSP	186	2	103	171	32	1274	37	M	2	2	no	vertical	vertical	straight	no	0	0	72,41	no
Madrid	MUTUA MADRILEÑA TROPHY GRAND PRIX CSI*5 W round A	25.11.2017	CSI-W5*	3	1.60	430	144	14	28.02.2004	G	DSP	186	2	103	171	32	1274	37	M	3	3	no	spread	oxer	right	no	0	0	72,41	no
Madrid	MUTUA MADRILEÑA TROPHY GRAND PRIX CSI*5 W round A	25.11.2017	CSI-W5*	3	1.60	430	144	14	28.02.2004	G	DSP	186	2	103	171	32	1274	37	M	4	4	no	vertical	vertical	straight	no	0	0	72,41	no
Madrid	MUTUA MADRILEÑA TROPHY GRAND PRIX CSI*5 W round A	25.11.2017	CSI-W5*	3	1.60	430	144	14	28.02.2004	G	DSP	186	2	103	171	32	1274	37	M	5	5	no	spread	oxer	left	no	0	0	72,41	no
Madrid	MUTUA MADRILEÑA TROPHY GRAND PRIX CSI*5 W round A	25.11.2017	CSI-W5*	3	1.60	430	144	14	28.02.2004	G	DSP	186	2	103	171	32	1274	37	M	6	6	no	vertical	vertical	left	no	0	0	72,41	no
Madrid	MUTUA MADRILEÑA TROPHY GRAND PRIX CSI*5 W round A	25.11.2017	CSI-W5*	3	1.60	430	144	14	28.02.2004	G	DSP	186	2	103	171	32	1274	37	M	7	7a	yes	spread	oxer	right	no	0	0	72,41	no
Madrid	MUTUA MADRILEÑA TROPHY GRAND PRIX CSI*5 W round A	25.11.2017	CSI-W5*	3	1.60	430	144	14	28.02.2004	G	DSP	186	2	103	171	32	1274	37	M	8	7b	yes	vertical	vertical	right	no	0	0	72,41	no
Madrid	MUTUA MADRILEÑA TROPHY GRAND PRIX CSI*5 W round A	25.11.2017	CSI-W5*	3	1.60	430	144	14	28.02.2004	G	DSP	186	2	103	171	32	1274	37	M	9	8	no	spread	oxer	right	no	0	0	72,41	no
Madrid	MUTUA MADRILEÑA TROPHY GRAND PRIX CSI*5 W round A	25.11.2017	CSI-W5*	3	1.60	430	144	14	28.02.2004	G	DSP	186	2	103	171	32	1274	37	M	10	9	no	vertical	vertical	right	no	0	0	72,41	no
Madrid	MUTUA MADRILEÑA TROPHY GRAND PRIX CSI*5 W round A	25.11.2017	CSI-W5*	3	1.60	430	144	14	28.02.2004	G	DSP	186	2	103	171	32	1274	37	M	11	10	no	spread	oxer	straight	no	0	0	72,41	no
Madrid	MUTUA MADRILEÑA TROPHY GRAND PRIX CSI*5 W round A	25.11.2017	CSI-W5*	3	1.60	430	144	14	28.02.2004	G	DSP	186	2	103	171	32	1274	37	M	12	11	no	vertical	vertical with water	left	no	0	0	72,41	no
Madrid	MUTUA MADRILEÑA TROPHY GRAND PRIX CSI*5 W round A	25.11.2017	CSI-W5*	3	1.60	430	144	14	28.02.2004	G	DSP	186	2	103	171	32	1274	37	M	13	12a	yes	vertical	vertical	left	no	0	0	72,41	no
Madrid	MUTUA MADRILEÑA TROPHY GRAND PRIX CSI*5 W round A	25.11.2017	CSI-W5*	3	1.60	430	144	14	28.02.2004	G	DSP	186	2	103	171	32	1274	37	M	14	12b	yes	spread	oxer with water	left	no	0	0	72,41	no
Madrid	MUTUA MADRILEÑA TROPHY GRAND PRIX CSI*5 W round A	25.11.2017	CSI-W5*	3	1.60	430	144	14	28.02.2004	G	DSP	186	2	103	171	32	1274	37	M	15	12c	yes	vertical	vertical	left	no	0	0	72,41	no
Madrid	MUTUA MADRILEÑA TROPHY GRAND PRIX CSI*5 W round A	25.11.2017	CSI-W5*	3	1.60	430	144	14	28.02.2004	G	DSP	186	2	103	171	32	1274	37	M	16	13	no	spread	oxer	right	no	0	0	72,41	no
Madrid	MUTUA MADRILEÑA TROPHY GRAND PRIX CSI*5 W round B	25.11.2017	CSI-W5*	3	1.60	300	144	14	28.02.2004	G	DSP	186	2	103	171	32	1274	37	M	1	1	no	spread	oxer	right	no	0	0	45,77	no
Madrid	MUTUA MADRILEÑA TROPHY GRAND PRIX CSI*5 W round B	25.11.2017	CSI-W5*	3	1.60	300	144	14	28.02.2004	G	DSP	186	2	103	171	32	1274	37	M	2	2	no	spread	oxer	right	no	0	0	45,77	no
Madrid	MUTUA MADRILEÑA TROPHY GRAND PRIX CSI*5 W round B	25.11.2017	CSI-W5*	3	1.60	300	144	14	28.02.2004	G	DSP	186	2	103	171	32	1274	37	M	3	3	no	vertical	vertical with water	left	no	0	0	45,77	no
Madrid	MUTUA MADRILEÑA TROPHY GRAND PRIX CSI*5 W round B	25.11.2017	CSI-W5*	3	1.60	300	144	14	28.02.2004	G	DSP	186	2	103	171	32	1274	37	M	4	4a	yes	vertical	vertical	left	no	0	0	45,77	no
Madrid	MUTUA MADRILEÑA TROPHY GRAND PRIX CSI*5 W round B	25.11.2017	CSI-W5*	3	1.60	300	144	14	28.02.2004	G	DSP	186	2	103	171	32	1274	37	M	5	4b	yes	spread	oxer with water	left	no	0	0	45,77	no
Madrid	MUTUA MADRILEÑA TROPHY GRAND PRIX CSI*5 W round B	25.11.2017	CSI-W5*	3	1.60	300	144	14	28.02.2004	G	DSP	186	2	103	171	32	1274	37	M	6	5	no	spread	oxer	right	no	0	0	45,77	no
Madrid	MUTUA MADRILEÑA TROPHY GRAND PRIX CSI*5 W round B	25.11.2017	CSI-W5*	3	1.60	300	144	14	28.02.2004	G	DSP	186	2	103	171	32	1274	37	M	7	6	no	vertical	vertical	right	no	0	0	45,77	no
Madrid	MUTUA MADRILEÑA TROPHY GRAND PRIX CSI*5 W round B	25.11.2017	CSI-W5*	3	1.60	300	144	14	28.02.2004	G	DSP	186	2	103	171	32	1274	37	M	8	7	no	vertical	vertical	left	no	0	0	45,77	no
Madrid	MUTUA MADRILEÑA TROPHY GRAND PRIX CSI*5 W round B	25.11.2017	CSI-W5*	3	1.60	300	144	14	28.02.2004	G	DSP	186	2	103	171	32	1274	37	M	9	8	no	spread	oxer	left	yes	4	0	45,77	no
Madrid	MUTUA MADRILEÑA TROPHY GRAND PRIX CSI*5 W round A	25.11.2017	CSI-W5*	3	1.60	430	6	15	17.04.2003	M	HANN	261	7	104	24	42	2261	97	M	1	1	no	spread	oxer	left	no	0	0	72,1	no
Madrid	MUTUA MADRILEÑA TROPHY GRAND PRIX CSI*5 W round A	25.11.2017	CSI-W5*	3	1.60	430	6	15	17.04.2003	M	HANN	261	7	104	24	42	2261	97	M	2	2	no	vertical	vertical	straight	no	0	0	72,1	no
Madrid	MUTUA MADRILEÑA TROPHY GRAND PRIX CSI*5 W round A	25.11.2017	CSI-W5*	3	1.60	430	6	15	17.04.2003	M	HANN	261	7	104	24	42	2261	97	M	3	3	no	spread	oxer	right	no	0	0	72,1	no
Madrid	MUTUA MADRILEÑA TROPHY GRAND PRIX CSI*5 W round A	25.11.2017	CSI-W5*	3	1.60	430	6	15	17.04.2003	M	HANN	261	7	104	24	42	2261	97	M	4	4	no	vertical	vertical	straight	no	0	0	72,1	no
Madrid	MUTUA MADRILEÑA TROPHY GRAND PRIX CSI*5 W round A	25.11.2017	CSI-W5*	3	1.60	430	6	15	17.04.2003	M	HANN	261	7	104	24	42	2261	97	M	5	5	no	spread	oxer	left	no	0	0	72,1	no
Madrid	MUTUA MADRILEÑA TROPHY GRAND PRIX CSI*5 W round A	25.11.2017	CSI-W5*	3	1.60	430	6	15	17.04.2003	M	HANN	261	7	104	24	42	2261	97	M	6	6	no	vertical	vertical	left	no	0	0	72,1	no
Madrid	MUTUA MADRILEÑA TROPHY GRAND PRIX CSI*5 W round A	25.11.2017	CSI-W5*	3	1.60	430	6	15	17.04.2003	M	HANN	261	7	104	24	42	2261	97	M	7	7a	yes	spread	oxer	right	no	0	0	72,1	no
Madrid	MUTUA MADRILEÑA TROPHY GRAND PRIX CSI*5 W round A	25.11.2017	CSI-W5*	3	1.60	430	6	15	17.04.2003	M	HANN	261	7	104	24	42	2261	97	M	8	7b	yes	vertical	vertical	right	no	0	0	72,1	no
Madrid	MUTUA MADRILEÑA TROPHY GRAND PRIX CSI*5 W round A	25.11.2017	CSI-W5*	3	1.60	430	6	15	17.04.2003	M	HANN	261	7	104	24	42	2261	97	M	9	8	no	spread	oxer	right	no	0	0	72,1	no
Madrid	MUTUA MADRILEÑA TROPHY GRAND PRIX CSI*5 W round A	25.11.2017	CSI-W5*	3	1.60	430	6	15	17.04.2003	M	HANN	261	7	104	24	42	2261	97	M	10	9	no	vertical	vertical	right	no	0	0	72,1	no
Madrid	MUTUA MADRILEÑA TROPHY GRAND PRIX CSI*5 W round A	25.11.2017	CSI-W5*	3	1.60	430	6	15	17.04.2003	M	HANN	261	7	104	24	42	2261	97	M	11	10	no	spread	oxer	straight	no	0	0	72,1	no
Madrid	MUTUA MADRILEÑA TROPHY GRAND PRIX CSI*5 W round A	25.11.2017	CSI-W5*	3	1.60	430	6	15	17.04.2003	M	HANN	261	7	104	24	42	2261	97	M	12	11	no	vertical	vertical with water	left	no	0	0	72,1	no
Madrid	MUTUA MADRILEÑA TROPHY GRAND PRIX CSI*5 W round A	25.11.2017	CSI-W5*	3	1.60	430	6	15	17.04.2003	M	HANN	261	7	104	24	42	2261	97	M	13	12a	yes	vertical	vertical	left	no	0	0	72,1	no
Madrid	MUTUA MADRILEÑA TROPHY GRAND PRIX CSI*5 W round A	25.11.2017	CSI-W5*	3	1.60	430	6	15	17.04.2003	M	HANN	261	7	104	24	42	2261	97	M	14	12b	yes	spread	oxer with water	left	no	0	0	72,1	no
Madrid	MUTUA MADRILEÑA TROPHY GRAND PRIX CSI*5 W round A	25.11.2017	CSI-W5*	3	1.60	430	6	15	17.04.2003	M	HANN	261	7	104	24	42	2261	97	M	15	12c	yes	vertical	vertical	left	no	0	0	72,1	no
Madrid	MUTUA MADRILEÑA TROPHY GRAND PRIX CSI*5 W round A	25.11.2017	CSI-W5*	3	1.60	430	6	15	17.04.2003	M	HANN	261	7	104	24	42	2261	97	M	16	13	no	spread	oxer	right	no	0	0	72,1	no
Madrid	MUTUA MADRILEÑA TROPHY GRAND PRIX CSI*5 W round B	25.11.2017	CSI-W5*	3	1.60	300	6	15	17.04.2003	M	HANN	261	7	104	24	42	2261	97	M	1	1	no	spread	oxer	right	no	0	0	45,09	no
Madrid	MUTUA MADRILEÑA TROPHY GRAND PRIX CSI*5 W round B	25.11.2017	CSI-W5*	3	1.60	300	6	15	17.04.2003	M	HANN	261	7	104	24	42	2261	97	M	2	2	no	spread	oxer	right	no	0	0	45,09	no
Madrid	MUTUA MADRILEÑA TROPHY GRAND PRIX CSI*5 W round B	25.11.2017	CSI-W5*	3	1.60	300	6	15	17.04.2003	M	HANN	261	7	104	24	42	2261	97	M	3	3	no	vertical	vertical with water	left	no	0	0	45,09	no
Madrid	MUTUA MADRILEÑA TROPHY GRAND PRIX CSI*5 W round B	25.11.2017	CSI-W5*	3	1.60	300	6	15	17.04.2003	M	HANN	261	7	104	24	42	2261	97	M	4	4a	yes	vertical	vertical	left	no	0	0	45,09	no
Madrid	MUTUA MADRILEÑA TROPHY GRAND PRIX CSI*5 W round B	25.11.2017	CSI-W5*	3	1.60	300	6	15	17.04.2003	M	HANN	261	7	104	24	42	2261	97	M	5	4b	yes	spread	oxer with water	left	no	0	0	45,09	no
Madrid	MUTUA MADRILEÑA TROPHY GRAND PRIX CSI*5 W round B	25.11.2017	CSI-W5*	3	1.60	300	6	15	17.04.2003	M	HANN	261	7	104	24	42	2261	97	M	6	5	no	spread	oxer	right	no	0	0	45,09	no
Madrid	MUTUA MADRILEÑA TROPHY GRAND PRIX CSI*5 W round B	25.11.2017	CSI-W5*	3	1.60	300	6	15	17.04.2003	M	HANN	261	7	104	24	42	2261	97	M	7	6	no	vertical	vertical	right	no	0	0	45,09	no
Madrid	MUTUA MADRILEÑA TROPHY GRAND PRIX CSI*5 W round B	25.11.2017	CSI-W5*	3	1.60	300	6	15	17.04.2003	M	HANN	261	7	104	24	42	2261	97	M	8	7	no	vertical	vertical	left	no	0	0	45,09	no
Madrid	MUTUA MADRILEÑA TROPHY GRAND PRIX CSI*5 W round B	25.11.2017	CSI-W5*	3	1.60	300	6	15	17.04.2003	M	HANN	261	7	104	24	42	2261	97	M	9	8	no	spread	oxer	left	yes	4	0	45,09	no
Madrid	MUTUA MADRILEÑA TROPHY GRAND PRIX CSI*5 W round A	25.11.2017	CSI-W5*	3	1.60	430	217	15	17.04.2003	M	KWPN	227	2	105	112	37	1565	24	M	1	1	no	spread	oxer	left	no	0	0	71,5	no
Madrid	MUTUA MADRILEÑA TROPHY GRAND PRIX CSI*5 W round A	25.11.2017	CSI-W5*	3	1.60	430	217	15	17.04.2003	M	KWPN	227	2	105	112	37	1565	24	M	2	2	no	vertical	vertical	straight	no	0	0	71,5	no
Madrid	MUTUA MADRILEÑA TROPHY GRAND PRIX CSI*5 W round A	25.11.2017	CSI-W5*	3	1.60	430	217	15	17.04.2003	M	KWPN	227	2	105	112	37	1565	24	M	3	3	no	spread	oxer	right	no	0	0	71,5	no
Madrid	MUTUA MADRILEÑA TROPHY GRAND PRIX CSI*5 W round A	25.11.2017	CSI-W5*	3	1.60	430	217	15	17.04.2003	M	KWPN	227	2	105	112	37	1565	24	M	4	4	no	vertical	vertical	straight	no	0	0	71,5	no
Madrid	MUTUA MADRILEÑA TROPHY GRAND PRIX CSI*5 W round A	25.11.2017	CSI-W5*	3	1.60	430	217	15	17.04.2003	M	KWPN	227	2	105	112	37	1565	24	M	5	5	no	spread	oxer	left	no	0	0	71,5	no
Madrid	MUTUA MADRILEÑA TROPHY GRAND PRIX CSI*5 W round A	25.11.2017	CSI-W5*	3	1.60	430	217	15	17.04.2003	M	KWPN	227	2	105	112	37	1565	24	M	6	6	no	vertical	vertical	left	no	0	0	71,5	no
Madrid	MUTUA MADRILEÑA TROPHY GRAND PRIX CSI*5 W round A	25.11.2017	CSI-W5*	3	1.60	430	217	15	17.04.2003	M	KWPN	227	2	105	112	37	1565	24	M	7	7a	yes	spread	oxer	right	yes	4	0	71,5	no
Madrid	MUTUA MADRILEÑA TROPHY GRAND PRIX CSI*5 W round A	25.11.2017	CSI-W5*	3	1.60	430	217	15	17.04.2003	M	KWPN	227	2	105	112	37	1565	24	M	8	7b	yes	vertical	vertical	right	no	0	0	71,5	no
Madrid	MUTUA MADRILEÑA TROPHY GRAND PRIX CSI*5 W round A	25.11.2017	CSI-W5*	3	1.60	430	217	15	17.04.2003	M	KWPN	227	2	105	112	37	1565	24	M	9	8	no	spread	oxer	right	no	0	0	71,5	no
Madrid	MUTUA MADRILEÑA TROPHY GRAND PRIX CSI*5 W round A	25.11.2017	CSI-W5*	3	1.60	430	217	15	17.04.2003	M	KWPN	227	2	105	112	37	1565	24	M	10	9	no	vertical	vertical	right	no	0	0	71,5	no
Madrid	MUTUA MADRILEÑA TROPHY GRAND PRIX CSI*5 W round A	25.11.2017	CSI-W5*	3	1.60	430	217	15	17.04.2003	M	KWPN	227	2	105	112	37	1565	24	M	11	10	no	spread	oxer	straight	no	0	0	71,5	no
Madrid	MUTUA MADRILEÑA TROPHY GRAND PRIX CSI*5 W round A	25.11.2017	CSI-W5*	3	1.60	430	217	15	17.04.2003	M	KWPN	227	2	105	112	37	1565	24	M	12	11	no	vertical	vertical with water	left	no	0	0	71,5	no
Madrid	MUTUA MADRILEÑA TROPHY GRAND PRIX CSI*5 W round A	25.11.2017	CSI-W5*	3	1.60	430	217	15	17.04.2003	M	KWPN	227	2	105	112	37	1565	24	M	13	12a	yes	vertical	vertical	left	no	0	0	71,5	no
Madrid	MUTUA MADRILEÑA TROPHY GRAND PRIX CSI*5 W round A	25.11.2017	CSI-W5*	3	1.60	430	217	15	17.04.2003	M	KWPN	227	2	105	112	37	1565	24	M	14	12b	yes	spread	oxer with water	left	no	0	0	71,5	no
Madrid	MUTUA MADRILEÑA TROPHY GRAND PRIX CSI*5 W round A	25.11.2017	CSI-W5*	3	1.60	430	217	15	17.04.2003	M	KWPN	227	2	105	112	37	1565	24	M	15	12c	yes	vertical	vertical	left	no	0	0	71,5	no
Madrid	MUTUA MADRILEÑA TROPHY GRAND PRIX CSI*5 W round A	25.11.2017	CSI-W5*	3	1.60	430	217	15	17.04.2003	M	KWPN	227	2	105	112	37	1565	24	M	16	13	no	spread	oxer	right	no	0	0	71,5	no
La Coruna	WORLD CUP GRAND PRIX – CAIXABANK TROPHY CSI W 5 /CSI 2 round	10.12.2017	CSI-W5*	4	1.60	490	63	11	22.03.2007	G	KWPN	175	2	3	97	43	1901	45	M	1	1	no	spread	oxer	left	no	0	0	81,1	no
La Coruna	WORLD CUP GRAND PRIX – CAIXABANK TROPHY CSI W 5 /CSI 2 round	10.12.2017	CSI-W5*	4	1.60	490	63	11	22.03.2007	G	KWPN	175	2	3	97	43	1901	45	M	2	2	no	vertical	vertical	left	no	0	0	81,1	no
La Coruna	WORLD CUP GRAND PRIX – CAIXABANK TROPHY CSI W 5 /CSI 2 round	10.12.2017	CSI-W5*	4	1.60	490	63	11	22.03.2007	G	KWPN	175	2	3	97	43	1901	45	M	3	3	no	spread	oxer	straight	no	0	0	81,1	no
La Coruna	WORLD CUP GRAND PRIX – CAIXABANK TROPHY CSI W 5 /CSI 2 round	10.12.2017	CSI-W5*	4	1.60	490	63	11	22.03.2007	G	KWPN	175	2	3	97	43	1901	45	M	4	4	no	spread	oxer	right	no	0	0	81,1	no
La Coruna	WORLD CUP GRAND PRIX – CAIXABANK TROPHY CSI W 5 /CSI 2 round	10.12.2017	CSI-W5*	4	1.60	490	63	11	22.03.2007	G	KWPN	175	2	3	97	43	1901	45	M	5	5a	yes	vertical	vertical	right	no	0	0	81,1	no
La Coruna	WORLD CUP GRAND PRIX – CAIXABANK TROPHY CSI W 5 /CSI 2 round	10.12.2017	CSI-W5*	4	1.60	490	63	11	22.03.2007	G	KWPN	175	2	3	97	43	1901	45	M	6	5b	yes	vertical	vertical	right	no	0	0	81,1	no
La Coruna	WORLD CUP GRAND PRIX – CAIXABANK TROPHY CSI W 5 /CSI 2 round	10.12.2017	CSI-W5*	4	1.60	490	63	11	22.03.2007	G	KWPN	175	2	3	97	43	1901	45	M	7	5c	yes	spread	oxer	right	no	0	0	81,1	no
La Coruna	WORLD CUP GRAND PRIX – CAIXABANK TROPHY CSI W 5 /CSI 2 round	10.12.2017	CSI-W5*	4	1.60	490	63	11	22.03.2007	G	KWPN	175	2	3	97	43	1901	45	M	8	6	no	vertical	vertical	left	no	0	0	81,1	no
La Coruna	WORLD CUP GRAND PRIX – CAIXABANK TROPHY CSI W 5 /CSI 2 round	10.12.2017	CSI-W5*	4	1.60	490	63	11	22.03.2007	G	KWPN	175	2	3	97	43	1901	45	M	9	7	no	spread	oxer	left	no	0	0	81,1	no
La Coruna	WORLD CUP GRAND PRIX – CAIXABANK TROPHY CSI W 5 /CSI 2 round	10.12.2017	CSI-W5*	4	1.60	490	63	11	22.03.2007	G	KWPN	175	2	3	97	43	1901	45	M	10	8a	yes	spread	oxer	straight	no	0	0	81,1	no
La Coruna	WORLD CUP GRAND PRIX – CAIXABANK TROPHY CSI W 5 /CSI 2 round	10.12.2017	CSI-W5*	4	1.60	490	63	11	22.03.2007	G	KWPN	175	2	3	97	43	1901	45	M	11	8b	yes	vertical	vertical	straight	no	0	0	81,1	no
La Coruna	WORLD CUP GRAND PRIX – CAIXABANK TROPHY CSI W 5 /CSI 2 round	10.12.2017	CSI-W5*	4	1.60	490	63	11	22.03.2007	G	KWPN	175	2	3	97	43	1901	45	M	12	9	no	spread	oxer with water	left	no	0	0	81,1	no
La Coruna	WORLD CUP GRAND PRIX – CAIXABANK TROPHY CSI W 5 /CSI 2 round	10.12.2017	CSI-W5*	4	1.60	490	63	11	22.03.2007	G	KWPN	175	2	3	97	43	1901	45	M	13	10	no	vertical	vertical	right	no	0	0	81,1	no
La Coruna	WORLD CUP GRAND PRIX – CAIXABANK TROPHY CSI W 5 /CSI 2 round	10.12.2017	CSI-W5*	4	1.60	490	63	11	22.03.2007	G	KWPN	175	2	3	97	43	1901	45	M	14	11	no	spread	oxer	right	no	0	0	81,1	no
La Coruna	WORLD CUP GRAND PRIX – CAIXABANK TROPHY CSI W 5 /CSI 2 round	10.12.2017	CSI-W5*	4	1.60	490	63	11	22.03.2007	G	KWPN	175	2	3	97	43	1901	45	M	15	12	no	vertical	vertical	straight	no	0	0	81,1	no
La Coruna	WORLD CUP GRAND PRIX – CAIXABANK TROPHY CSI W 5 /CSI 2 round	10.12.2017	CSI-W5*	4	1.60	490	63	11	22.03.2007	G	KWPN	175	2	3	97	43	1901	45	M	16	13	no	spread	oxer	straight	no	0	0	81,1	no
La Coruna	WORLD CUP GRAND PRIX – CAIXABANK TROPHY CSI W 5 /CSI 2 round	10.12.2017	CSI-W5*	4	1.60	490	63	11	22.03.2007	G	KWPN	175	2	3	97	43	1901	45	M	17	14	no	vertical	vertical with water	left	no	0	0	81,1	no
La Coruna	WORLD CUP GRAND PRIX – CAIXABANK TROPHY CSI W 5 /CSI 2 round	10.12.2017	CSI-W5*	4	1.60	285	63	11	22.03.2007	G	KWPN	175	2	3	97	43	1901	45	M	1	1	no	vertical	vertical	left	no	0	0	43,89	no
La Coruna	WORLD CUP GRAND PRIX – CAIXABANK TROPHY CSI W 5 /CSI 2 round	10.12.2017	CSI-W5*	4	1.60	285	63	11	22.03.2007	G	KWPN	175	2	3	97	43	1901	45	M	2	2	no	spread	oxer	right	no	0	0	43,89	no

La Coruna	WORLD CUP GRAND PRIX – CAIXABANK TROPHY CSI W 5 /CSI 2 roun	10.12.2017	CSI-W5*	4	1.60	285	63	11	22.03.2007	G	KWPN	175	2	3	97	43	1901	45	M	3	3	no	spread	oxer with water	left	no	0	0	43,89	no
La Coruna	WORLD CUP GRAND PRIX – CAIXABANK TROPHY CSI W 5 /CSI 2 roun	10.12.2017	CSI-W5*	4	1.60	285	63	11	22.03.2007	G	KWPN	175	2	3	97	43	1901	45	M	4	4	no	vertical	vertical	right	no	0	0	43,89	no
La Coruna	WORLD CUP GRAND PRIX – CAIXABANK TROPHY CSI W 5 /CSI 2 roun	10.12.2017	CSI-W5*	4	1.60	285	63	11	22.03.2007	G	KWPN	175	2	3	97	43	1901	45	M	5	5	no	spread	oxer	right	no	0	0	43,89	no
La Coruna	WORLD CUP GRAND PRIX – CAIXABANK TROPHY CSI W 5 /CSI 2 roun	10.12.2017	CSI-W5*	4	1.60	285	63	11	22.03.2007	G	KWPN	175	2	3	97	43	1901	45	M	6	6a	yes	vertical	vertical	right	no	0	0	43,89	no
La Coruna	WORLD CUP GRAND PRIX – CAIXABANK TROPHY CSI W 5 /CSI 2 roun	10.12.2017	CSI-W5*	4	1.60	285	63	11	22.03.2007	G	KWPN	175	2	3	97	43	1901	45	M	7	6b	yes	spread	oxer	right	no	0	0	43,89	no
La Coruna	WORLD CUP GRAND PRIX – CAIXABANK TROPHY CSI W 5 /CSI 2 roun	10.12.2017	CSI-W5*	4	1.60	285	63	11	22.03.2007	G	KWPN	175	2	3	97	43	1901	45	M	8	7	no	vertical	vertical	left	no	0	0	43,89	no
La Coruna	WORLD CUP GRAND PRIX – CAIXABANK TROPHY CSI W 5 /CSI 2 roun	10.12.2017	CSI-W5*	4	1.60	490	61	10	12.04.2008	M	ZANG	82	0	6	421	45	483	7	F	1	1	no	spread	oxer	left	no	0	0	0,00	yes
La Coruna	WORLD CUP GRAND PRIX – CAIXABANK TROPHY CSI W 5 /CSI 2 roun	10.12.2017	CSI-W5*	4	1.60	490	61	10	12.04.2008	M	ZANG	82	0	6	421	45	483	7	F	2	2	no	vertical	vertical	left	no	0	0	0,00	yes
La Coruna	WORLD CUP GRAND PRIX – CAIXABANK TROPHY CSI W 5 /CSI 2 roun	10.12.2017	CSI-W5*	4	1.60	490	61	10	12.04.2008	M	ZANG	82	0	6	421	45	483	7	F	3	3	no	spread	oxer	straight	no	0	0	0,00	yes
La Coruna	WORLD CUP GRAND PRIX – CAIXABANK TROPHY CSI W 5 /CSI 2 roun	10.12.2017	CSI-W5*	4	1.60	490	61	10	12.04.2008	M	ZANG	82	0	6	421	45	483	7	F	4	4	no	spread	oxer	right	no	0	0	0,00	yes
La Coruna	WORLD CUP GRAND PRIX – CAIXABANK TROPHY CSI W 5 /CSI 2 roun	10.12.2017	CSI-W5*	4	1.60	490	61	10	12.04.2008	M	ZANG	82	0	6	421	45	483	7	F	5	5a	yes	vertical	vertical	right	no	0	0	0,00	yes
La Coruna	WORLD CUP GRAND PRIX – CAIXABANK TROPHY CSI W 5 /CSI 2 roun	10.12.2017	CSI-W5*	4	1.60	490	61	10	12.04.2008	M	ZANG	82	0	6	421	45	483	7	F	6	5b	yes	vertical	vertical	right	yes	4	0	0,00	yes
La Coruna	WORLD CUP GRAND PRIX – CAIXABANK TROPHY CSI W 5 /CSI 2 roun	10.12.2017	CSI-W5*	4	1.60	490	61	10	12.04.2008	M	ZANG	82	0	6	421	45	483	7	F	7	5c	yes	spread	oxer	right	yes	4	0	0,00	yes
La Coruna	WORLD CUP GRAND PRIX – CAIXABANK TROPHY CSI W 5 /CSI 2 roun	10.12.2017	CSI-W5*	4	1.60	490	61	10	12.04.2008	M	ZANG	82	0	6	421	45	483	7	F	8	6	no	vertical	vertical	left	no	0	0	0,00	yes
La Coruna	WORLD CUP GRAND PRIX – CAIXABANK TROPHY CSI W 5 /CSI 2 roun	10.12.2017	CSI-W5*	4	1.60	490	61	10	12.04.2008	M	ZANG	82	0	6	421	45	483	7	F	9	7	no	spread	oxer	left	no	0	0	0,00	yes
La Coruna	WORLD CUP GRAND PRIX – CAIXABANK TROPHY CSI W 5 /CSI 2 roun	10.12.2017	CSI-W5*	4	1.60	490	61	10	12.04.2008	M	ZANG	82	0	6	421	45	483	7	F	10	8a	yes	spread	oxer	straight	yes	4	0	0,00	yes
La Coruna	WORLD CUP GRAND PRIX – CAIXABANK TROPHY CSI W 5 /CSI 2 roun	10.12.2017	CSI-W5*	4	1.60	490	61	10	12.04.2008	M	ZANG	82	0	6	421	45	483	7	F	11	8b	yes	vertical	vertical	straight	yes	4	0	0,00	yes
La Coruna	WORLD CUP GRAND PRIX – CAIXABANK TROPHY CSI W 5 /CSI 2 roun	10.12.2017	CSI-W5*	4	1.60	490	61	10	12.04.2008	M	ZANG	82	0	6	421	45	483	7	F	12	9	no	spread	oxer with water	left	no	0	0	0,00	yes
La Coruna	WORLD CUP GRAND PRIX – CAIXABANK TROPHY CSI W 5 /CSI 2 roun	10.12.2017	CSI-W5*	4	1.60	490	61	10	12.04.2008	M	ZANG	82	0	6	421	45	483	7	F	13	10	no	vertical	vertical	right	no	0	0	0,00	yes
La Coruna	WORLD CUP GRAND PRIX – CAIXABANK TROPHY CSI W 5 /CSI 2 roun	10.12.2017	CSI-W5*	4	1.60	490	61	10	12.04.2008	M	ZANG	82	0	6	421	45	483	7	F	14	11	no	spread	oxer	right	no	0	0	0,00	yes
La Coruna	WORLD CUP GRAND PRIX – CAIXABANK TROPHY CSI W 5 /CSI 2 roun	10.12.2017	CSI-W5*	4	1.60	490	61	10	12.04.2008	M	ZANG	82	0	6	421	45	483	7	F	15	12	no	vertical	vertical	straight	no	0	0	0,00	yes
La Coruna	WORLD CUP GRAND PRIX – CAIXABANK TROPHY CSI W 5 /CSI 2 roun	10.12.2017	CSI-W5*	4	1.60	490	61	10	12.04.2008	M	ZANG	82	0	6	421	45	483	7	F	16	13	no	spread	oxer	straight	no	0	0	0,00	yes
La Coruna	WORLD CUP GRAND PRIX – CAIXABANK TROPHY CSI W 5 /CSI 2 roun	10.12.2017	CSI-W5*	4	1.60	490	61	10	12.04.2008	M	ZANG	82	0	6	421	45	483	7	F	17	14	no	vertical	vertical with water	left	no	0	0	0,00	yes
La Coruna	WORLD CUP GRAND PRIX – CAIXABANK TROPHY CSI W 5 /CSI 2 roun	10.12.2017	CSI-W5*	4	1.60	490	195	11	25.04.2007	G	SF	164	16	9	103	48	1417	59	M	1	1	no	spread	oxer	left	no	0	0	78,38	no
La Coruna	WORLD CUP GRAND PRIX – CAIXABANK TROPHY CSI W 5 /CSI 2 roun	10.12.2017	CSI-W5*	4	1.60	490	195	11	25.04.2007	G	SF	164	16	9	103	48	1417	59	M	2	2	no	vertical	vertical	left	no	0	0	78,38	no
La Coruna	WORLD CUP GRAND PRIX – CAIXABANK TROPHY CSI W 5 /CSI 2 roun	10.12.2017	CSI-W5*	4	1.60	490	195	11	25.04.2007	G	SF	164	16	9	103	48	1417	59	M	3	3	no	spread	oxer	straight	no	0	0	78,38	no
La Coruna	WORLD CUP GRAND PRIX – CAIXABANK TROPHY CSI W 5 /CSI 2 roun	10.12.2017	CSI-W5*	4	1.60	490	195	11	25.04.2007	G	SF	164	16	9	103	48	1417	59	M	4	4	no	spread	oxer	right	no	0	0	78,38	no
La Coruna	WORLD CUP GRAND PRIX – CAIXABANK TROPHY CSI W 5 /CSI 2 roun	10.12.2017	CSI-W5*	4	1.60	490	195	11	25.04.2007	G	SF	164	16	9	103	48	1417	59	M	5	5a	yes	vertical	vertical	right	no	0	0	78,38	no
La Coruna	WORLD CUP GRAND PRIX – CAIXABANK TROPHY CSI W 5 /CSI 2 roun	10.12.2017	CSI-W5*	4	1.60	490	195	11	25.04.2007	G	SF	164	16	9	103	48	1417	59	M	6	5b	yes	vertical	vertical	right	yes	4	0	78,38	no
La Coruna	WORLD CUP GRAND PRIX – CAIXABANK TROPHY CSI W 5 /CSI 2 roun	10.12.2017	CSI-W5*	4	1.60	490	195	11	25.04.2007	G	SF	164	16	9	103	48	1417	59	M	7	5c	yes	spread	oxer	right	no	0	0	78,38	no
La Coruna	WORLD CUP GRAND PRIX – CAIXABANK TROPHY CSI W 5 /CSI 2 roun	10.12.2017	CSI-W5*	4	1.60	490	195	11	25.04.2007	G	SF	164	16	9	103	48	1417	59	M	8	6	no	vertical	vertical	left	no	0	0	78,38	no
La Coruna	WORLD CUP GRAND PRIX – CAIXABANK TROPHY CSI W 5 /CSI 2 roun	10.12.2017	CSI-W5*	4	1.60	490	195	11	25.04.2007	G	SF	164	16	9	103	48	1417	59	M	9	7	no	spread	oxer	left	no	0	0	78,38	no
La Coruna	WORLD CUP GRAND PRIX – CAIXABANK TROPHY CSI W 5 /CSI 2 roun	10.12.2017	CSI-W5*	4	1.60	490	195	11	25.04.2007	G	SF	164	16	9	103	48	1417	59	M	10	8a	yes	spread	oxer	straight	yes	4	0	78,38	no
La Coruna	WORLD CUP GRAND PRIX – CAIXABANK TROPHY CSI W 5 /CSI 2 roun	10.12.2017	CSI-W5*	4	1.60	490	195	11	25.04.2007	G	SF	164	16	9	103	48	1417	59	M	11	8b	yes	vertical	vertical	straight	no	0	0	78,38	no
La Coruna	WORLD CUP GRAND PRIX – CAIXABANK TROPHY CSI W 5 /CSI 2 roun	10.12.2017	CSI-W5*	4	1.60	490	195	11	25.04.2007	G	SF	164	16	9	103	48	1417	59	M	12	9	no	spread	oxer with water	left	no	0	0	78,38	no
La Coruna	WORLD CUP GRAND PRIX – CAIXABANK TROPHY CSI W 5 /CSI 2 roun	10.12.2017	CSI-W5*	4	1.60	490	195	11	25.04.2007	G	SF	164	16	9	103	48	1417	59	M	13	10	no	vertical	vertical	right	no	0	0	78,38	no
La Coruna	WORLD CUP GRAND PRIX – CAIXABANK TROPHY CSI W 5 /CSI 2 roun	10.12.2017	CSI-W5*	4	1.60	490	195	11	25.04.2007	G	SF	164	16	9	103	48	1417	59	M	14	11	no	spread	oxer	right	no	0	0	78,38	no
La Coruna	WORLD CUP GRAND PRIX – CAIXABANK TROPHY CSI W 5 /CSI 2 roun	10.12.2017	CSI-W5*	4	1.60	490	195	11	25.04.2007	G	SF	164	16	9	103	48	1417	59	M	15	12	no	vertical	vertical	straight	no	0	0	78,38	no
La Coruna	WORLD CUP GRAND PRIX – CAIXABANK TROPHY CSI W 5 /CSI 2 roun	10.12.2017	CSI-W5*	4	1.60	490	195	11	25.04.2007	G	SF	164	16	9	103	48	1417	59	M	16	13	no	spread	oxer	straight	no	0	0	78,38	no
La Coruna	WORLD CUP GRAND PRIX – CAIXABANK TROPHY CSI W 5 /CSI 2 roun	10.12.2017	CSI-W5*	4	1.60	490	195	11	25.04.2007	G	SF	164	16	9	103	48	1417	59	M	17	14	no	vertical	vertical with water	left	no	0	0	78,38	no
La Coruna	WORLD CUP GRAND PRIX – CAIXABANK TROPHY CSI W 5 /CSI 2 roun	10.12.2017	CSI-W5*	4	1.60	490	212	12	10.04.2006	S	BWP	134	11	13	42	49	1996	131	M	1	1	no	spread	oxer	left	no	0	0	82,6	no
La Coruna	WORLD CUP GRAND PRIX – CAIXABANK TROPHY CSI W 5 /CSI 2 roun	10.12.2017	CSI-W5*	4	1.60	490	212	12	10.04.2006	S	BWP	134	11	13	42	49	1996	131	M	2	2	no	vertical	vertical	left	no	0	0	82,6	no
La Coruna	WORLD CUP GRAND PRIX – CAIXABANK TROPHY CSI W 5 /CSI 2 roun	10.12.2017	CSI-W5*	4	1.60	490	212	12	10.04.2006	S	BWP	134	11	13	42	49	1996	131	M	3	3	no	spread	oxer	straight	no	0	0	82,6	no
La Coruna	WORLD CUP GRAND PRIX – CAIXABANK TROPHY CSI W 5 /CSI 2 roun	10.12.2017	CSI-W5*	4	1.60	490	212	12	10.04.2006	S	BWP	134	11	13	42	49	1996	131	M	4	4	no	spread	oxer	right	no	0	0	82,6	no
La Coruna	WORLD CUP GRAND PRIX – CAIXABANK TROPHY CSI W 5 /CSI 2 roun	10.12.2017	CSI-W5*	4	1.60	490	212	12	10.04.2006	S	BWP	134	11	13	42	49	1996	131	M	5	5a	yes	vertical	vertical	right	no	0	0	82,6	no
La Coruna	WORLD CUP GRAND PRIX – CAIXABANK TROPHY CSI W 5 /CSI 2 roun	10.12.2017	CSI-W5*	4	1.60	490	212	12	10.04.2006	S	BWP	134	11	13	42	49	1996	131	M	6	5b	yes	vertical	vertical	right	no	0	0	82,6	no
La Coruna	WORLD CUP GRAND PRIX – CAIXABANK TROPHY CSI W 5 /CSI 2 roun	10.12.2017	CSI-W5*	4	1.60	490	212	12	10.04.2006	S	BWP	134	11	13	42	49	1996	131	M	7	5c	yes	spread	oxer	right	no	0	0	82,6	no
La Coruna	WORLD CUP GRAND PRIX – CAIXABANK TROPHY CSI W 5 /CSI 2 roun	10.12.2017	CSI-W5*	4	1.60	490	212	12	10.04.2006	S	BWP	134	11	13	42	49	1996	131	M	8	6	no	vertical	vertical	left	no	0	0	82,6	no
La Coruna	WORLD CUP GRAND PRIX – CAIXABANK TROPHY CSI W 5 /CSI 2 roun	10.12.2017	CSI-W5*	4	1.60	490	212	12	10.04.2006	S	BWP	134	11	13	42	49	1996	131	M	9	7	no	spread	oxer	left	no	0	0	82,6	no
La Coruna	WORLD CUP GRAND PRIX – CAIXABANK TROPHY CSI W 5 /CSI 2 roun	10.12.2017	CSI-W5*	4	1.60	490	212	12	10.04.2006	S	BWP	134	11	13	42	49	1996	131	M	10	8a	yes	spread	oxer	straight	no	0	0	82,6	no
La Coruna	WORLD CUP GRAND PRIX – CAIXABANK TROPHY CSI W 5 /CSI 2 roun	10.12.2017	CSI-W5*	4	1.60	490	212	12	10.04.2006	S	BWP	134	11	13	42	49	1996	131	M	11	8b	yes	vertical	vertical	straight	no	0	0	82,6	no
La Coruna	WORLD CUP GRAND PRIX – CAIXABANK TROPHY CSI W 5 /CSI 2 roun	10.12.2017	CSI-W5*	4	1.60	490	212	12	10.04.2006	S	BWP	134	11	13	42	49	1996	131	M	12	9	no	spread	oxer with water	left	no	0	0	82,6	no
La Coruna	WORLD CUP GRAND PRIX – CAIXABANK TROPHY CSI W 5 /CSI 2 roun	10.12.2017	CSI-W5*	4	1.60	490	212	12	10.04.2006	S	BWP	134	11	13	42	49	1996	131	M	13	10	no	vertical	vertical	right	no	0	0	82,6	no
La Coruna	WORLD CUP GRAND PRIX – CAIXABANK TROPHY CSI W 5 /CSI 2 roun	10.12.2017	CSI-W5*	4	1.60	490	212	12	10.04.2006	S	BWP	134	11	13	42	49	1996	131	M	14	11	no	spread	oxer	right	no	0	0	82,6	no
La Coruna	WORLD CUP GRAND PRIX – CAIXABANK TROPHY CSI W 5 /CSI 2 roun	10.12.2017	CSI-W5*	4	1.60	490	212	12	10.04.2006	S	BWP	134	11	13	42	49	1996	131	M	15	12	no	vertical	vertical	straight	no	0	0	82,6	no
La Coruna	WORLD CUP GRAND PRIX – CAIXABANK TROPHY CSI W 5 /CSI 2 roun	10.12.2017	CSI-W5*	4	1.60	490	212	12	10.04.2006	S	BWP	134	11	13	42	49	1996	131	M	16	13	no	spread	oxer	straight	no	0	0	82,6	no
La Coruna	WORLD CUP GRAND PRIX – CAIXABANK TROPHY CSI W 5 /CSI 2 roun	10.12.2017	CSI-W5*	4	1.60	490	212	12	10.04.2006	S	BWP	134	11	13	42	49	1996	131	M	17	14	no	vertical	vertical with water	left	yes	4	0	82,6	no
La Coruna	WORLD CUP GRAND PRIX – CAIXABANK TROPHY CSI W 5 /CSI 2 roun	10.12.2017	CSI-W5*	4	1.60	490	56	14	12.03.2004	G	OLDBG	203	7	16	17	37	2215	106	M	1	1	no	spread	oxer	left	no	0	0	80,56	no
La Coruna	WORLD CUP GRAND PRIX – CAIXABANK TROPHY CSI W 5 /CSI 2 roun	10.12.2017	CSI-W5*	4	1.60	490	56	14	12.03.2004	G	OLDBG	203	7	16	17	37	2215	106	M	2	2	no	vertical	vertical	left	no	0	0	80,56	no
La Coruna	WORLD CUP GRAND PRIX – CAIXABANK TROPHY CSI W 5 /CSI 2 roun	10.12.2017	CSI-W5*	4	1.60	490	56	14	12.03.2004	G	OLDBG	203	7	16	17	37	2215	106	M	3	3	no	spread	oxer	straight	no	0	0	80,56	no
La Coruna	WORLD CUP GRAND PRIX – CAIXABANK TROPHY CSI W 5 /CSI 2 roun	10.12.2017	CSI-W5*	4	1.60	490	56	14	12.03.2004	G	OLDBG	203	7	16	17	37	2215	106	M	4	4	no	spread	oxer	right	no	0	0	80,56	no
La Coruna	WORLD CUP GRAND PRIX – CAIXABANK TROPHY CSI W 5 /CSI 2 roun	10.12.2017	CSI-W5*	4	1.60	490	56	14	12.03.2004	G	OLDBG	203	7	16	17	37	2215	106	M	5	5a	yes	vertical	vertical	right	no	0	0	80,56	no
La Coruna	WORLD CUP GRAND PRIX – CAIXABANK TROPHY CSI W 5 /CSI 2 roun	10.12.2017	CSI-W5*	4	1.60	490	56	14	12.03.2004	G	OLDBG	203	7	16	17	37	2215	106	M	6	5b	yes	vertical	vertical	right	no	0	0	80,56	no
La Coruna	WORLD CUP GRAND PRIX – CAIXABANK TROPHY CSI W 5 /CSI 2 roun	10.12.2017	CSI-W5*	4	1.60	490	56	14	12.03.2004	G	OLDBG	203	7	16	17	37	2215	106	M	7	5c	yes	spread	oxer	right	no	0	0	80,56	no
La Coruna	WORLD CUP GRAND PRIX – CAIXABANK TROPHY CSI W 5 /CSI 2 roun	10.12.2017	CSI-W5*	4	1.60	490	56	14	12.03.2004	G	OLDBG	203	7	16	17	37	2215	106	M	8	6	no	vertical	vertical	left	no	0	0	80,56	no
La Coruna	WORLD CUP GRAND PRIX – CAIXABANK TROPHY CSI W 5 /CSI 2 roun	10.12.2017	CSI-W5*	4	1.60	490	56	14	12.03.2004	G	OLDBG	203	7	16	17	37	2215	106	M	9	7	no	spread	oxer	left	no	0	0	80,56	no
La Coruna	WORLD CUP GRAND PRIX – CAIXABANK TROPHY CSI W 5 /CSI 2 roun	10.12.2017	CSI-W5*	4	1.60	490	56	14	12.03.2004	G	OLDBG	203	7	16	17	37	2215	106	M	10	8a	yes	spread	oxer	straight	no	0	0	80,56	no
La Coruna	WORLD CUP GRAND PRIX – CAIXABANK TROPHY CSI W 5 /CSI 2 roun	10.12.2017	CSI-W5*	4	1.60	490	56	14	12.03.2004	G	OLDBG	203	7	16	17	37	2215	106	M	11	8b	yes	vertical	vertical	straight	no	0	0	80,56	no
La Coruna	WORLD CUP GRAND PRIX – CAIXABANK TROPHY CSI W 5 /CSI 2 roun	10.12.2017	CSI-W5*	4	1.60	490	56	14	12.03.2004	G	OLDBG	203	7	16	17	37	2215	106	M	12	9	no	spread	oxer with water	left	yes	4	0	80,56	no
La Coruna	WORLD CUP GRAND PRIX – CAIXABANK TROPHY CSI W 5 /CSI 2 roun	10.12.2017	CSI-W5*	4	1.60	490	56	14	12.03.2004	G	OLDBG	203	7	16	17	37	2215	106	M	13	10	no	vertical	vertical	right	no	0	0	80,56	no
La Coruna	WORLD CUP GRAND PRIX – CAIXABANK TROPHY CSI W 5 /CSI 2 roun	10.12.2017	CSI-W5*	4	1.60	490	56	14	12.03.2004	G	OLDBG	203	7	16	17	37	2215	106	M	14	11	no	spread	oxer	right	no	0	0	80,56	no
La Coruna	WORLD CUP GRAND PRIX – CAIXABANK TROPHY CSI W 5 /CSI 2 roun	10.12.2017	CSI-W5*	4	1.60	490	56	14	12.03.2004	G	OLDBG	203	7	16	17	37	2215	106	M	15	12	no	vertical	vertical	straight	yes	4	0	80,56	no
La Coruna	WORLD CUP GRAND PRIX – CAIXABANK TROPHY CSI W 5 /CSI 2 roun	10.12.2017	CSI-W5*	4	1.60	490	56	14	12.03.2004	G	OLDBG	203	7	16	17	37	2215	106	M	16	13	no	spread	oxer	straight	yes	4	0	80,56	no
La Coruna	WORLD CUP GRAND PRIX – CAIXABANK TROPHY CSI W 5 /CSI 2 roun	10.12.2017	CSI-W5*	4	1.60	490	56	14	12.03.2004	G	OLDBG	203	7	16	17	37	2215	106	M	17	14	no	vertical	vertical with water	left	no	0	0	80,56	no
La Coruna	WORLD CUP GRAND PRIX – CAIXABANK TROPHY CSI W 5 /CSI 2 roun	10.12.2017	CSI-W5*	4	1.60	490	215	14	20.07.2004	G	KWPN	236	9	19	54	42	2041	67	M	1	1	no	spread	oxer	left	no	0	0	79,72	no
La Coruna	WORLD CUP GRAND PRIX – CAIXABANK TROPHY CSI W 5 /CSI 2 roun	10.12.2017	CSI-W5*	4	1.60	490	215	14	20.07.2004	G	KWPN	236	9	19	54	42	2041	67	M	2	2	no	vertical	vertical	left	no	0	0	79,72	no
La Coruna	WORLD CUP GRAND PRIX – CAIXABANK TROPHY CSI W 5 /CSI 2 roun	10.12.2017	CSI-W5*	4	1.60	490	215	14	20.07.2004	G	KWPN	236	9	19	54	42	2041	67	M	3	3	no	spread	oxer	straight	no	0	0	79,72	no
La Coruna	WORLD CUP GRAND PRIX – CAIXABANK TROPHY CSI W 5 /CSI 2 roun	10.12.2017	CSI-W5*	4	1.60	490	215	14	20.07.2004	G	KWPN	236	9	19	54	42	2041	67	M	4	4	no	spread	oxer	right	no	0	0	79,72	no
La Coruna	WORLD CUP GRAND PRIX – CAIXABANK TROPHY CSI W 5 /CSI 2 roun	10.12.2017	CSI-W5*	4	1.60	490	215	14	20.07.2004	G	KWPN	236	9	19	54	42	2041	67	M	5	5a	yes	vertical	vertical	right	no	0	0	79,72	no
La Coruna	WORLD CUP GRAND PRIX – CAIXABANK TROPHY CSI W 5 /CSI 2 roun	10.12.2017	CSI-W5*	4	1.60	490	215	14	20.07.2004	G	KWPN	236	9	19	54	42	2041	67	M	6	5b	yes	vertical	vertical	right	no	0	0	79,72	no
La Coruna	WORLD CUP GRAND PRIX – CAIXABANK TROPHY CSI W 5 /CSI 2 roun	10.12.2017	CSI-W5*	4	1.60	490	215	14	20.07.2004	G	KWPN	236	9	19	54	42	2041	67	M	7	5c	yes	spread	oxer	right	no	0	0	79,72	no
La Coruna	WORLD CUP GRAND PRIX – CAIXABANK TROPHY CSI W 5 /CSI 2 roun	10.12.2017	CSI-W5*	4	1.60	490	215	14	20.07.2004	G	KWPN	236	9	19	54	42	2041	67	M	8	6	no	vertical	vertical	left	no	0	0	79,72	no
La Coruna	WORLD CUP GRAND PRIX – CAIXABANK TROPHY CSI W 5 /CSI 2 roun	10.12.2017	CSI-W5*	4	1.60	490	215	14	20.07.2004	G	KWPN	236	9	19	54	42	2041	67	M	9	7	no	spread	oxer	left	no	0	0	79,72	no
La Coruna	WORLD CUP GRAND PRIX – CAIXABANK TROPHY CSI W 5 /CSI 2 roun	10.12.2017	CSI-W5*	4	1.60	490	215	14	20.07.2004	G	KWPN	236	9	19	54	42	2041	67	M	10	8a	yes	spread	oxer	straight	no	0	0	79,72	no
La Coruna	WORLD CUP GRAND PRIX – CAIXABANK TROPHY CSI W 5 /CSI 2 roun	10.12.2017	CSI-W5*	4	1.60	490	215	14	20.07.2004	G	KWPN	236	9	19	54	42	2041	67	M	11	8b	yes	vertical	vertical	straight	no	0	0	79,72	no
La Coruna	WORLD CUP GRAND PRIX – CAIXABANK TROPHY CSI W 5 /CSI 2 roun	10.12.2017	CSI-W5*	4	1.60	490	215	14	20.07.2004	G	KWPN	236	9	19	54	42	2041	67	M	12	9	no	spread	oxer with water	left	no	0	0	79,72	no
La Coruna	WORLD CUP GRAND PRIX – CAIXABANK TROPHY CSI W 5 /CSI 2 roun	10.12.2017	CSI-W5*	4	1.60	490	215	14	20.07.2004	G	KWPN	236	9	19	54	42	2041	67	M	13	10	no	vertical	vertical	right	no	0	0	79,72	no
La Coruna	WORLD CUP GRAND PRIX – CAIXABANK TROPHY CSI W 5 /CSI 2 roun	10.12.2017	CSI-W5*	4	1.60	490	215	14	20.07.2004	G	KWPN	236	9	19	54	42	2041	67	M	14	11	no	spread	oxer	right	yes	4	0	79,72	no
La Coruna	WORLD CUP GRAND PRIX – CAIXABANK TROPHY CSI W 5 /CSI 2 roun	10.12.2017	CSI-W5*	4	1.60	490	215	14	20.07.2004	G	KWPN	236	9	19	54	42	2041	67	M	15	12	no	vertical	vertical	straight	no	0	0	79,72	no
La Coruna	WORLD CUP GRAND PRIX – CAIXABANK TROPHY CSI W 5 /CSI 2 roun	10.12.2017	CSI-W5*	4	1.60	490	215	14	20.07.2004	G	KWPN	236	9	19	54	42	2041	67	M	16	13	no	spread	oxer	straight	no	0	0	79,72	no
La Coruna	WORLD CUP GRAND PRIX – CAIXABANK TROPHY CSI W 5 /CSI 2 roun	10.12.2017	CSI-W5*	4	1.60	490	215	14	20.07.2004	G	KWPN	236	9	19	54	42	2041	67	M	17	14	no	vertical	vertical with water	left	no	0	0	79,72	no
La Coruna	WORLD CUP GRAND PRIX – CAIXABANK TROPHY CSI W 5 /CSI 2 roun	10.12.2017	CSI-W5*	4	1.60	490	162	12	01.01.2006	M	MIPAAF	146	3	24	287	49	641	15	M	1	1	no	spread	oxer	left	no	0	0	78	no
La Coruna	WORLD CUP GRAND PRIX – CAIXABANK TROPHY CSI W 5 /CSI 2 roun	10.12.2017	CSI-W5*	4	1.60	490	162	12	01.01.2006	M	MIPAAF	146	3	24	287	49	641	15	M	2	2	no	vertical	vertical	left	no	0	0	78	no
La Coruna	WORLD CUP GRAND PRIX – CAIXABANK TROPHY CSI W 5 /CSI 2 roun	10.12.2017	CSI-W5*	4	1.60	490	162	12	01.01.2006	M	MIPAAF	146	3	24	287	49	641	15	M	3	3	no	spread	oxer	straight	no	0	0	78	no
La Coruna	WORLD CUP GRAND PRIX – CAIXABANK TROPHY CSI W 5 /CSI 2 roun	10.12.2017	CSI-W5*	4	1.60	490	162	12	01.01.2006	M	MIPAAF	146	3	24	287	49	641	15	M	4	4	no	spread	oxer	right	no	0	0	78	no
La Coruna	WORLD CUP GRAND PRIX – CAIXABANK TROPHY CSI W 5 /CSI 2 roun	10.12.2017	CSI-W5*	4	1.60	490	162	12	01.01.2006	M	MIPAAF	146	3	24	287	49	641	15	M	5	5a	yes	vertical	vertical	right	no	0	0	78	no
La Coruna	WORLD CUP GRAND PRIX – CAIXABANK TROPHY CSI W 5 /CSI 2 roun	10.12.2017	CSI-W5*	4	1.60	490	162	12	01.01.2006	M	MIPAAF	146	3	24	287	49	641	15	M	6	5b	yes	vertical	vertical	right	no	0	0	78	no
La Coruna	WORLD CUP GRAND PRIX – CAIXABANK TROPHY CSI W 5 /CSI 2 roun	10.12.2017	CSI-W5*	4	1.60	490	162	12	01.01.2006	M	MIPAAF	146	3	24	287	49	641	15	M	7	5c	yes	spread	oxer	right	no	0	0	78	no
La Coruna	WORLD CUP GRAND PRIX – CAIXABANK TROPHY CSI W 5 /CSI 2 roun	10.12.2017	CSI-W5*	4	1.60	490	162	12	01.01.2006	M	MIPAAF	146	3	24	287	49	641	15	M	8	6	no	vertical	vertical	left	no	0	0	78	no
La Coruna	WORLD CUP GRAND PRIX – CAIXABANK TROPHY CSI W 5 /CSI 2 roun	10.12.2017	CSI-W5*	4	1.60	490	162	12	01.01.2006	M	MIPAAF	146	3	24	287	49	641	15	M	9	7	no	spread	oxer	left	no	0	0	78	no
La Coruna	WORLD CUP GRAND PRIX – CAIXABANK TROPHY CSI W 5 /CSI 2 roun	10.12.2017	CSI-W5*	4	1.60	490	162	12	01.01.2006	M	MIPAAF	146	3	24	287	49	641	15	M	10	8a	yes	spread	oxer	straight	no	0	0	78	no
La Coruna	WORLD CUP GRAND PRIX – CAIXABANK TROPHY CSI W 5 /CSI 2 roun	10.12.2017	CSI-W5*	4	1.60	490	162	12	01.01.2006	M	MIPAAF	146	3	24	287	49	641	15	M	11	8b	yes	vertical	vertical	straight	no	0	0	78	no
La Coruna	WORLD CUP GRAND PRIX – CAIXABANK TROPHY CSI W 5 /CSI 2 roun	10.12.2017	CSI-W5*	4	1.60	490	162	12	01.01.2006	M	MIPAAF	146	3	24	287	49	641	15	M	12	9	no	spread	oxer with water	left	no	0	0	78	no
La Coruna	WORLD CUP GRAND PRIX – CAIXABANK TROPHY CSI W 5 /CSI 2 roun	10.12.2017	CSI-W5*	4	1.60	490	162	12	01.01.2006	M	MIPAAF	146	3	24	287	49	641	15	M	13	10	no	vertical	vertical	right	no	0	0	78	no
La Coruna	WORLD CUP GRAND PRIX – CAIXABANK TROPHY CSI W 5 /CSI 2 roun	10.12.2017	CSI-W5*	4	1.60	490	162	12	01.01.2006	M	MIPAAF	146	3	24	287	49	641	15	M	14	11	no	spread	oxer	right	no	0	0	78	no
La Coruna	WORLD CUP GRAND PRIX – CAIXABANK TROPHY CSI W 5 /CSI 2 roun	10.12.2017	CSI-W5*	4	1.60	490	162	12	01.01.2006	M	MIPAAF	146	3	24	287	49	641	15	M	15	12	no	vertical	vertical	straight	no	0	0	78	no
La Coruna	WORLD CUP GRAND PRIX – CAIXABANK TROPHY CSI W 5 /CSI 2 roun	10.12.2017	CSI-W5*	4	1.60	490	162	12	01.01.2006	M	MIPAAF	146	3	24	287	49	641	15	M	16	13	no	spread	oxer	straight	no	0	0	78	no
La Coruna	WORLD CUP GRAND PRIX – CAIXABANK TROPHY CSI W 5 /CSI 2 roun	10.12.2017	CSI-W5*	4	1.60	490	162	12	01.01.2006	M	MIPAAF	146	3	24	287	49	641	15	M	17	14	no	vertical	vertical with water	left	yes	4	0	78	no
La Coruna	WORLD CUP GRAND PRIX – CAIXABANK TROPHY CSI W 5 /CSI 2 roun	10.12.2017	CSI-W5*	4	1.60	490	127	10	03.07.2008	M	BWP	131	3	26	14	30	1957	97	M	1	1	no	spread	oxer	left	no	0	0	81,14	no
La Coruna	WORLD CUP GRAND PRIX – CAIXABANK TROPHY CSI W 5 /CSI 2 roun	10.12.2017	CSI-W5*	4	1.60	490	127	10	03.07.2008	M	BWP	131	3	26	14	30	1957	97	M	2	2	no	vertical	vertical	left	no	0	0	81,14	no
La Coruna	WORLD CUP GRAND PRIX – CAIXABANK TROPHY CSI W 5 /CSI 2 roun	10.12.2017	CSI-W5*	4	1.60	490	127	10	03.07.2008	M	BWP	131	3	26	14	30	1957	97	M	3	3	no	spread	oxer	straight	no	0	0	81,14	no
La Coruna	WORLD CUP GRAND PRIX – CAIXABANK TROPHY CSI W 5 /CSI 2 roun	10.12.2017	CSI-W5*	4	1.60	490	127	10	03.07.2008	M	BWP	131	3	26	14	30	1957	97	M	4	4	no	spread	oxer	right	no	0	0	81,14	no
La Coruna	WORLD CUP GRAND PRIX – CAIXABANK TROPHY CSI W 5 /CSI 2 roun	10.12.2017	CSI-W5*	4	1.60	490	127	10	03.07.2008	M	BWP	131	3	26	14	30	1957	97	M	5	5a	yes	vertical	vertical	right	no	0	0	81,14	no
La Coruna	WORLD CUP GRAND PRIX – CAIXABANK TROPHY CSI W 5 /CSI 2 roun	10.12.2017	CSI-W5*	4	1.60	490	127	10	03.07.2008	M	BWP	131	3	26	14	30	1957	97	M	6	5b	yes	vertical	vertical	right	no	0	0	81,14	no
La Coruna	WORLD CUP GRAND PRIX – CAIXABANK TROPHY CSI W 5 /CSI 2 roun	10.12.2017	CSI-W5*	4	1.60	490	127	10	03.07.2008	M	BWP	131	3	26	14	30	1957	97	M	7	5c	yes	spread	oxer	right	no	0	0	81,14	no
La Coruna	WORLD CUP GRAND PRIX – CAIXABANK TROPHY CSI W 5 /CSI 2 roun	10.12.2017	CSI-W5*	4	1.60	490	127	10	03.07.2008	M	BWP	131	3	26	14	30	1957	97	M	8	6	no	vertical	vertical	left	no	0	0	81,14	no
La Coruna	WORLD CUP GRAND PRIX – CAIXABANK TROPHY CSI W 5 /CSI 2 roun	10.12.2017	CSI-W5*	4	1.60	490	127	10	03.07.2008	M	BWP	131	3	26	14	30	1957	97	M	9	7	no	spread	oxer	left	no	0	0	81,14	no
La Coruna	WORLD CUP GRAND PRIX – CAIXABANK TROPHY CSI W 5 /CSI 2 roun	10.12.2017	CSI-W5*	4	1.60	490	127	10	03.07.2008	M	BWP	131	3	26	14	30	1957	97	M	10	8a	yes	spread	oxer	straight	no	0	0	81,14	no
La Coruna	WORLD CUP GRAND PRIX – CAIXABANK TROPHY CSI W 5 /CSI 2 roun	10.12.2017	CSI-W5*	4	1.60	490	127	10	03.07.2008	M	BWP	131	3	26	14	30	1957	97	M	11	8b	yes	vertical	vertical	straight	no	0	0	81,14	no
La Coruna	WORLD CUP GRAND PRIX – CAIXABANK TROPHY CSI W 5 /CSI 2 roun	10.12.2017	CSI-W5*	4	1.60	490	127	10	03.07.2008	M	BWP	131	3	26	14	30	1957	97	M	12	9	no	spread	oxer with water	left	no	0	0	81,14	no
La Coruna	WORLD CUP GRAND PRIX – CAIXABANK TROPHY CSI W 5 /CSI 2 roun	10.12.2017	CSI-W5*	4	1.60	490	127	10	03.07.2008	M	BWP	131	3	26	14	30	1957	97	M	13	10	no	vertical	vertical	right	no	0	0	81,14	no
La Coruna	WORLD CUP GRAND PRIX – CAIXABANK TROPHY CSI W 5 /CSI 2 roun	10.12.2017	CSI-W5*	4	1.60	490	127	10	03.07.2008	M	BWP	131	3	26	14	30	1957	97	M	14	11	no	spread	oxer	right	no	0	0	81,14	no
La Coruna	WORLD CUP GRAND PRIX – CAIXABANK TROPHY CSI W 5 /CSI 2 roun	10.12.2017	CSI-W5*	4	1.60	490	127	10	03.07.2008	M	BWP	131	3	26	14	30	1957	97	M	15	12	no	vertical	vertical	straight	no	0	0	81,14	no
La Coruna	WORLD CUP GRAND PRIX – CAIXABANK TROPHY CSI W 5 /CSI 2 roun	10.12.2017	CSI-W5*	4	1.60	490	127	10	03.07.2008	M	BWP	131	3	26	14	30	1957	97	M	16	13	no	spread	oxer	straight	no	0	0	81,14	no
La Coruna	WORLD CUP GRAND PRIX – CAIXABANK TROPHY CSI W 5 /CSI 2 roun	10.12.2017	CSI-W5*	4	1.60	490	127	10	03.07.2008	M	BWP	131	3	26	14	30	1957	97	M	17	14	no	vertical	vertical with water	left	no	0	0	81,14	no
La Coruna	WORLD CUP GRAND PRIX – CAIXABANK TROPHY CSI W 5 /CSI 2 roun	10.12.2017	CSI-W5*	4	1.60	285	127	10	03.07.2008	M	BWP	131	3	26	14	30	1957	97	M	1	1	no	vertical	vertical	left	no	0	0	45,82	no
La Coruna	WORLD CUP GRAND PRIX – CAIXABANK TROPHY CSI W 5 /CSI 2 roun	10.12.2017	CSI-W5*	4	1.60	285	127	10	03.07.2008	M	BWP	131	3	26	14	30	1957	97	M	2	2	no	spread	oxer	right	no	0	0	45,82	no
La Coruna	WORLD CUP GRAND PRIX – CAIXABANK TROPHY CSI W 5 /CSI 2 roun	10.12.2017	CSI-W5*	4	1.60	285	127	10	03.07.2008	M	BWP	131	3	26	14	30	1957	97	M	3	3	no	spread	oxer with water	left	no	0	0	45,82	no
La Coruna	WORLD CUP GRAND PRIX – CAIXABANK TROPHY CSI W 5 /CSI 2 roun	10.12.2017	CSI-W5*	4	1.60	285	127	10	03.07.2008	M	BWP	131	3	26	14	30	1957	97	M	4	4	no	vertical	vertical	right	no	0	0	45,82	no
La Coruna	WORLD CUP GRAND PRIX – CAIXABANK TROPHY CSI W 5 /CSI 2 roun	10.12.2017	CSI-W5*	4	1.60	285	127	10	03.07.2008	M	BWP	131	3	26	14	30	1957	97	M	5	5	no	spread	oxer	right	no	0	0	45,82	no
La Coruna	WORLD CUP GRAND PRIX – CAIXABANK TROPHY CSI W 5 /CSI 2 roun	10.12.2017	CSI-W5*	4	1.60	285	127	10	03.07.2008	M	BWP	131	3	26	14	30	1957	97	M	6	6a	yes	vertical	vertical	right	yes	4	0	45,82	no
La Coruna	WORLD CUP GRAND PRIX – CAIXABANK TROPHY CSI W 5 /CSI 2 roun	10.12.2017	CSI-W5*	4	1.60	285	127	10	03.07.2008	M	BWP	131	3	26	14	30	1957	97	M	7	6b	yes	spread	oxer	right	no	0	0	45,82	no
La Coruna	WORLD CUP GRAND PRIX – CAIXABANK TROPHY CSI W 5 /CSI 2 roun	10.12.2017	CSI-W5*	4	1.60	285	127	10	03.07.2008	M	BWP	131	3	26	14	30	1957	97	M	8	7	no	vertical	vertical	left	no	0	0	45,82	no
La Coruna	WORLD CUP GRAND PRIX – CAIXABANK TROPHY CSI W 5 /CSI 2 roun	10.12.2017	CSI-W5*	4	1.60	490	220	13	01.01.2005	M	MIPAAF	153	14	27	269	20	1104	42	F	1	1	no	spread	oxer	left	no	0	0	78,99	no
La Coruna	WORLD CUP GRAND PRIX – CAIXABANK TROPHY CSI W 5 /CSI 2 roun	10.12.2017	CSI-W5*	4	1.60	490	220	13	01.01.2005	M	MIPAAF	153	14	27	269	20	1104	42	F	2	2	no	vertical	vertical	left	no	0	0	78,99	no
La Coruna	WORLD CUP GRAND PRIX – CAIXABANK TROPHY CSI W 5 /CSI 2 roun	10.12.2017	CSI-W5*	4	1.60	490	220	13	01.01.2005	M	MIPAAF	153	14	27	269	20	1104	42	F	3	3	no	spread	oxer	straight	no	0	0	78,99	no
La Coruna	WORLD CUP GRAND PRIX – CAIXABANK TROPHY CSI W 5 /CSI 2 roun	10.12.2017	CSI-W5*	4	1.60	490	220	13	01.01.2005	M	MIPAAF	153	14	27	269	20	1104	42	F	4	4	no	spread	oxer	right	no	0	0	78,99	no
La Coruna	WORLD CUP GRAND PRIX – CAIXABANK TROPHY CSI W 5 /CSI 2 roun	10.12.2017	CSI-W5*	4	1.60	490	220	13	01.01.2005	M	MIPAAF	153	14	27	269	20	1104	42	F	5	5a	yes	vertical	vertical	right	no	0	0	78,99	no
La Coruna	WORLD CUP GRAND PRIX – CAIXABANK TROPHY CSI W 5 /CSI 2 roun	10.12.2017	CSI-W5*	4	1.60	490	220	13	01.01.2005	M	MIPAAF	153	14	27	269	20	1104	42	F	6	5b	yes	vertical	vertical	right	no	0	0	78,99	no
La Coruna	WORLD CUP GRAND PRIX – CAIXABANK TROPHY CSI W 5 /CSI 2 roun	10.12.2017	CSI-W5*	4	1.60	490	220	13	01.01.2005	M	MIPAAF	153	14	27	269	20	1104	42	F	7	5c	yes	spread	oxer	right	yes	4	0	78,99	no
La Coruna	WORLD CUP GRAND PRIX – CAIXABANK TROPHY CSI W 5 /CSI 2 roun	10.12.2017	CSI-W5*	4	1.60	490	220	13	01.01.2005	M	MIPAAF	153	14	27	269	20	1104	42	F	8	6	no	vertical	vertical	left	no	0	0	78,99	no
La Coruna	WORLD CUP GRAND PRIX – CAIXABANK TROPHY CSI W 5 /CSI 2 roun	10.12.2017	CSI-W5*	4	1.60	490	220	13	01.01.2005	M	MIPAAF	153	14	27	269	20	1104	42	F	9	7	no	spread	oxer	left	no	0	0	78,99	no
La Coruna	WORLD CUP GRAND PRIX – CAIXABANK TROPHY CSI W 5 /CSI 2 roun	10.12.2017	CSI-W5*	4	1.60	490	220	13	01.01.2005	M	MIPAAF	153	14	27	269	20	1104	42	F	10	8a	yes	spread	oxer	straight	no	0	0	78,99	no
La Coruna	WORLD CUP GRAND PRIX – CAIXABANK TROPHY CSI W 5 /CSI 2 roun	10.12.2017	CSI-W5*	4	1.60	490	220	13	01.01.2005	M	MIPAAF	153	14	27	269	20	1104	42	F	11	8b	yes	vertical	vertical	straight	no	0	0	78,99	no
La Coruna	WORLD CUP GRAND PRIX – CAIXABANK TROPHY CSI W 5 /CSI 2 roun	10.12.2017	CSI-W5*	4	1.60	490	220	13	01.01.2005	M	MIPAAF	153	14	27	269	20	1104	42	F	12	9	no	spread	oxer with water	left	no	0	0	78,99	no
La Coruna	WORLD CUP GRAND PRIX – CAIXABANK TROPHY CSI W 5 /CSI 2 roun	10.12.2017	CSI-W5*	4	1.60	490	220	13	01.01.2005	M	MIPAAF	153	14	27	269	20	1104	42	F	13	10	no	vertical	vertical	right	no	0	0	78,99	no
La Coruna	WORLD CUP GRAND PRIX – CAIXABANK TROPHY CSI W 5 /CSI 2 roun	10.12.2017	CSI-W5*	4	1.60	490	220	13	01.01.2005	M	MIPAAF	153	14	27	269	20	1104	42	F	14	11	no	spread	oxer	right	no	0	0	78,99	no
La Coruna	WORLD CUP GRAND PRIX – CAIXABANK TROPHY CSI W 5 /CSI 2 roun	10.12.2017	CSI-W5*	4	1.60	490	220	13	01.01.2005	M	MIPAAF	153	14	27	269	20	1104	42	F	15	12	no	vertical	vertical	straight	no	0	0	78,99	no
La Coruna	WORLD CUP GRAND PRIX – CAIXABANK TROPHY CSI W 5 /CSI 2 roun	10.12.2017	CSI-W5*	4	1.60	490	220	13	01.01.2005	M	MIPAAF	153	14	27	269	20	1104	42	F	16	13	no	spread	oxer	straight	no	0	0	78,99	no
La Coruna	WORLD CUP GRAND PRIX – CAIXABANK TROPHY CSI W 5 /CSI 2 roun	10.12.2017	CSI-W5*	4	1.60	490	220	13	01.01.2005	M	MIPAAF	153	14	27	269	20	1104	42	F	17	14	no	vertical	vertical with water	left	yes	4	0	78,99	no
La Coruna	WORLD CUP GRAND PRIX – CAIXABANK TROPHY CSI W 5 /CSI 2 roun	10.12.2017	CSI-W5*	4	1.60	490	35	15	22.05.2003	G	HOLST	368	19	29	89	35	1502	55	M	1	1	no	spread	oxer	left	no	0	0	80,34	no
La Coruna	WORLD CUP GRAND PRIX – CAIXABANK TROPHY CSI W 5 /CSI 2 roun	10.12.2017	CSI-W5*	4	1.60	490	35	15	22.05.2003	G	HOLST	368	19	29	89	35	1502	55	M	2	2	no	vertical	vertical	left	no	0	0	80,34	no
La Coruna	WORLD CUP GRAND PRIX – CAIXABANK TROPHY CSI W 5 /CSI 2 roun	10.12.2017	CSI-W5*	4	1.60	490	35	15	22.05.2003	G	HOLST	368	19	29	89	35	1502	55	M	3	3	no	spread	oxer	straight	no	0	0	80,34	no
La Coruna	WORLD CUP GRAND PRIX – CAIXABANK TROPHY CSI W 5 /CSI 2 roun	10.12.2017	CSI-W5*	4	1.60	490	35	15	22.05.2003	G	HOLST	368	19	29	89	35	1502	55	M	4	4	no	spread	oxer	right	no	0	0	80,34	no
La Coruna	WORLD CUP GRAND PRIX – CAIXABANK TROPHY CSI W 5 /CSI 2 roun	10.12.2017	CSI-W5*	4	1.60	490	35	15	22.05.2003	G	HOLST	368	19	29	89	35	1502	55	M	5	5a	yes	vertical	vertical	right	no	0	0	80,34	no
La Coruna	WORLD CUP GRAND PRIX – CAIXABANK TROPHY CSI W 5 /CSI 2 roun	10.12.2017	CSI-W5*	4	1.60	490	35	15	22.05.2003	G	HOLST	368	19	29	89	35	1502	55	M	6	5b	yes	vertical	vertical	right	no	0	0	80,34	no
La Coruna	WORLD CUP GRAND PRIX – CAIXABANK TROPHY CSI W 5 /CSI 2 roun	10.12.2017	CSI-W5*	4	1.60	490	35	15	22.05.2003	G	HOLST	368	19	29	89	35	1502	55	M	7	5c	yes	spread	oxer	right	no	0	0	80,34	no
La Coruna	WORLD CUP GRAND PRIX – CAIXABANK TROPHY CSI W 5 /CSI 2 roun	10.12.2017	CSI-W5*	4	1.60	490	35	15	22.05.2003	G	HOLST	368	19	29	89	35	1502	55	M	8	6	no	vertical	vertical	left	no	0	0	80,34	no
La Coruna	WORLD CUP GRAND PRIX – CAIXABANK TROPHY CSI W 5 /CSI 2 roun	10.12.2017	CSI-W5*	4	1.60	490	35	15	22.05.2003	G	HOLST	368	19	29	89	35	1502	55	M	9	7	no	spread	oxer	left	no	0	0	80,34	no
La Coruna	WORLD CUP GRAND PRIX – CAIXABANK TROPHY CSI W 5 /CSI 2 roun	10.12.2017	CSI-W5*	4	1.60	490	35	15	22.05.2003	G	HOLST	368	19	29	89	35	1502	55	M	10	8a	yes	spread	oxer	straight	no	0	0	80,34	no
La Coruna	WORLD CUP GRAND PRIX – CAIXABANK TROPHY CSI W 5 /CSI 2 roun	10.12.2017	CSI-W5*	4	1.60	490	35	15	22.05.2003	G	HOLST	368	19	29	89	35	1502	55	M	11	8b	yes	vertical	vertical	straight	no	0	0	80,34	no
La Coruna	WORLD CUP GRAND PRIX – CAIXABANK TROPHY CSI W 5 /CSI 2 roun	10.12.2017	CSI-W5*	4	1.60	490	35	15	22.05.2003	G	HOLST	368	19	29	89	35	1502	55	M	12	9	no	spread	oxer with water	left	no	0	0	80,34	no
La Coruna	WORLD CUP GRAND PRIX – CAIXABANK TROPHY CSI W 5 /CSI 2 roun	10.12.2017	CSI-W5*	4	1.60	490	35	15	22.05.2003	G	HOLST	368	19	29	89	35	1502	55	M	13	10	no	vertical	vertical	right	no	0	0	80,34	no
La Coruna	WORLD CUP GRAND PRIX – CAIXABANK TROPHY CSI W 5 /CSI 2 roun	10.12.2017	CSI-W5*	4	1.60	490	35	15	22.05.2003	G	HOLST	368	19	29	89	35	1502	55	M	14	11	no	spread	oxer	right	no	0	0	80,34	no
La Coruna	WORLD CUP GRAND PRIX – CAIXABANK TROPHY CSI W 5 /CSI 2 roun	10.12.2017	CSI-W5*	4	1.60	490	35	15	22.05.2003	G	HOLST	368	19	29	89	35	1502	55	M	15	12	no	vertical	vertical	straight	no	0	0	80,34	no
La Coruna	WORLD CUP GRAND PRIX – CAIXABANK TROPHY CSI W 5 /CSI 2 roun	10.12.2017	CSI-W5*	4	1.60	490	35	15	22.05.2003	G	HOLST	368	19	29	89	35	1502	55	M	16	13	no	spread	oxer	straight	no	0	0	80,34	no
La Coruna	WORLD CUP GRAND PRIX – CAIXABANK TROPHY CSI W 5 /CSI 2 roun	10.12.2017	CSI-W5*	4	1.60	490	35	15	22.05.2003	G	HOLST	368	19	29	89	35	1502	55	M	17	14	no	vertical	vertical with water	left	no	0	0	80,34	no
La Coruna	WORLD CUP GRAND PRIX – CAIXABANK TROPHY CSI W 5 /CSI 2 roun	10.12.2017	CSI-W5*	4	1.60	285	35	15	22.05.2003	G	HOLST	368	19	29	89	35	1502	55	M	1	1	no	vertical	vertical	left	no	0	0	42,58	no
La Coruna	WORLD CUP GRAND PRIX – CAIXABANK TROPHY CSI W 5 /CSI 2 roun	10.12.2017	CSI-W5*	4	1.60	285	35	15	22.05.2003	G	HOLST	368	19	29	89	35	1502	55	M	2	2	no	spread	oxer	right	no	0	0	42,58	no
La Coruna	WORLD CUP GRAND PRIX – CAIXABANK TROPHY CSI W 5 /CSI 2 roun	10.12.2017	CSI-W5*	4	1.60	285	35	15	22.05.2003	G	HOLST	368	19	29	89	35	1502	55	M	3	3	no	spread	oxer with water	left	no	0	0	42,58	no
La Coruna	WORLD CUP GRAND PRIX – CAIXABANK TROPHY CSI W 5 /CSI 2 roun	10.12.2017	CSI-W5*	4	1.60	285	35	15	22.05.2003	G	HOLST	368	19	29	89	35	1502	55	M	4	4	no	vertical	vertical	right	no	0	0	42,58	no
La Coruna	WORLD CUP GRAND PRIX – CAIXABANK TROPHY CSI W 5 /CSI 2 roun	10.12.2017	CSI-W5*	4	1.60	285	35	15	22.05.2003	G	HOLST	368	19	29	89	35	1502	55	M	5	5	no	spread	oxer	right	yes	4	0	42,58	no
La Coruna	WORLD CUP GRAND PRIX – CAIXABANK TROPHY CSI W 5 /CSI 2 roun	10.12.2017	CSI-W5*	4	1.60	285	35	15	22.05.2003	G	HOLST	368	19	29	89	35	1502	55	M	6	6a	yes	vertical	vertical	right	no	0	0	42,58	no
La Coruna	WORLD CUP GRAND PRIX – CAIXABANK TROPHY CSI W 5 /CSI 2 roun	10.12.2017	CSI-W5*	4	1.60	285	35	15	22.05.2003	G	HOLST	368	19	29	89	35	1502	55	M	7	6b	yes	spread	oxer	right	no	0	0	42,58	no
La Coruna	WORLD CUP GRAND PRIX – CAIXABANK TROPHY CSI W 5 /CSI 2 roun	10.12.2017	CSI-W5*	4	1.60	285	35	15	22.05.2003	G	HOLST	368	19	29	89	35	1502	55	M	8	7	no	vertical	vertical	left	no	0	0	42,58	no
La Coruna	WORLD CUP GRAND PRIX – CAIXABANK TROPHY CSI W 5 /CSI 2 roun	10.12.2017	CSI-W5*	4	1.60	490	23	13	25.02.2005	S	Bezplemenné	158	2	32	53	23	1832	77	M	1	1	no	spread	oxer	left	no	0	0	77,67	no
La Coruna	WORLD CUP GRAND PRIX – CAIXABANK TROPHY CSI W 5 /CSI 2 roun	10.12.2017	CSI-W5*	4	1.60	490	23	13	25.02.2005	S	Bezplemenné	158	2	32	53	23	1832	77	M	2	2	no	vertical	vertical	left	no	0	0	77,67	no
La Coruna	WORLD CUP GRAND PRIX – CAIXABANK TROPHY CSI W 5 /CSI 2 roun	10.12.2017	CSI-W5*	4	1.60	490	23	13	25.02.2005	S	Bezplemenné	158	2	32	53	23	1832	77	M	3	3	no	spread	oxer	straight	no	0	0	77,67	no
La Coruna	WORLD CUP GRAND PRIX – CAIXABANK TROPHY CSI W 5 /CSI 2 roun	10.12.2017	CSI-W5*	4	1.60	490	23	13	25.02.2005	S	Bezplemenné	158	2	32	53	23	1832	77	M	4	4	no	spread	oxer	right	no	0	0	77,67	no
La Coruna	WORLD CUP GRAND PRIX – CAIXABANK TROPHY CSI W 5 /CSI 2 roun	10.12.2017	CSI-W5*	4	1.60	490	23	13	25.02.2005	S	Bezplemenné	158	2	32	53	23	1832	77	M	5	5a	yes	vertical	vertical	right	no	0	0	77,67	no
La Coruna	WORLD CUP GRAND PRIX – CAIXABANK TROPHY CSI W 5 /CSI 2 roun	10.12.2017	CSI-W5*	4	1.60	490	23	13	25.02.2005	S	Bezplemenné	158	2	32	53	23	1832	77	M	6	5b	yes	vertical	vertical	right	no	0	0	77,67	no
La Coruna	WORLD CUP GRAND PRIX – CAIXABANK TROPHY CSI W 5 /CSI 2 roun	10.12.2017	CSI-W5*	4	1.60	490	23	13	25.02.2005	S	Bezplemenné	158	2	32	53	23	1832	77	M	7	5c	yes	spread	oxer	right	yes	4	0	77,67	no
La Coruna	WORLD CUP GRAND PRIX – CAIXABANK TROPHY CSI W 5 /CSI 2 roun	10.12.2017	CSI-W5*	4	1.60	490	23	13	25.02.2005	S	Bezplemenné	158	2	32	53	23	1832	77	M	8	6	no	vertical	vertical	left	no	0	0	77,67	no
La Coruna	WORLD CUP GRAND PRIX – CAIXABANK TROPHY CSI W 5 /CSI 2 roun	10.12.2017	CSI-W5*	4	1.60	490	23	13	25.02.2005	S	Bezplemenné	158	2	32	53	23	1832	77	M	9	7	no	spread	oxer	left	no	0	0	77,67	no
La Coruna	WORLD CUP GRAND PRIX – CAIXABANK TROPHY CSI W 5 /CSI 2 roun	10.12.2017	CSI-W5*	4	1.60	490	23	13	25.02.2005	S	Bezplemenné	158	2	32	53	23	1832	77	M	10	8a	yes	spread	oxer	straight	no	0	0	77,67	no
La Coruna	WORLD CUP GRAND PRIX – CAIXABANK TROPHY CSI W 5 /CSI 2 roun	10.12.2017	CSI-W5*	4	1.60	490	23	13	25.02.2005	S	Bezplemenné	158	2	32	53	23	1832	77	M	11	8b	yes	vertical	vertical	straight	no	0	0	77,67	no
La Coruna	WORLD CUP GRAND PRIX – CAIXABANK TROPHY CSI W 5 /CSI 2 roun	10.12.2017	CSI-W5*	4	1.60	490	23	13	25.02.2005	S	Bezplemenné	158	2	32	53	23	1832	77	M	12	9	no	spread	oxer with water	left	no	0	0	77,67	no
La Coruna	WORLD CUP GRAND PRIX – CAIXABANK TROPHY CSI W 5 /CSI 2 roun	10.12.2017	CSI-W5*	4	1.60	490	23	13	25.02.2005	S	Bezplemenné	158	2	32	53	23	1832	77	M	13	10	no	vertical	vertical	right	no	0	0	77,67	no
La Coruna	WORLD CUP GRAND PRIX – CAIXABANK TROPHY CSI W 5 /CSI 2 roun	10.12.2017	CSI-W5*	4	1.60	490	23	13	25.02.2005	S	Bezplemenné	158	2	32	53	23	1832	77	M	14	11	no	spread	oxer	right	yes	4	0	77,67	no
La Coruna	WORLD CUP GRAND PRIX – CAIXABANK TROPHY CSI W 5 /CSI 2 roun	10.12.2017	CSI-W5*	4	1.60	490	23	13	25.02.2005	S	Bezplemenné	158	2	32	53	23	1832	77	M	15	12	no	vertical	vertical	straight	no	0	0	77,67	no
La Coruna	WORLD CUP GRAND PRIX – CAIXABANK TROPHY CSI W 5 /CSI 2 roun	10.12.2017	CSI-W5*	4	1.60	490	23	13	25.02.2005	S	Bezplemenné	158	2	32	53	23	1832	77	M	16	13	no	spread	oxer	straight	no	0	0	77,67	no
La Coruna	WORLD CUP GRAND PRIX – CAIXABANK TROPHY CSI W 5 /CSI 2 roun	10.12.2017	CSI-W5*	4	1.60	490	23	13	25.02.2005	S	Bezplemenné	158	2	32	53	23	1832	77	M	17	14	no	vertical	vertical with water	left	no	0	0	77,67	no
La Coruna	WORLD CUP GRAND PRIX – CAIXABANK TROPHY CSI W 5 /CSI 2 roun	10.12.2017	CSI-W5*	4	1.60	490	37	11	02.03.2007	M	KWPN	133	11	35	44	30	2234	138	M	1	1	no	spread	oxer	left	yes	4	0	75,86	no
La Coruna	WORLD CUP GRAND PRIX – CAIXABANK TROPHY CSI W 5 /CSI 2 roun	10.12.2017	CSI-W5*	4	1.60	490	37	11	02.03.2007	M	KWPN	133	11	35	44	30	2234	138	M	2	2	no	vertical	vertical	left	no	0	0	75,86	no
La Coruna	WORLD CUP GRAND PRIX – CAIXABANK TROPHY CSI W 5 /CSI 2 roun	10.12.2017	CSI-W5*	4	1.60	490	37	11	02.03.2007	M	KWPN	133	11	35	44	30	2234	138	M	3	3	no	spread	oxer	straight	no	0	0	75,86	no
La Coruna	WORLD CUP GRAND PRIX – CAIXABANK TROPHY CSI W 5 /CSI 2 roun	10.12.2017	CSI-W5*	4	1.60	490	37	11	02.03.2007	M	KWPN	133	11	35	44	30	2234	138	M	4	4	no	spread	oxer	right	no	0	0	75,86	no
La Coruna	WORLD CUP GRAND PRIX – CAIXABANK TROPHY CSI W 5 /CSI 2 roun	10.12.2017	CSI-W5*	4	1.60	490	37	11	02.03.2007	M	KWPN	133	11	35	44	30	2234	138	M	5	5a	yes	vertical	vertical	right	no	0	0	75,86	no
La Coruna	WORLD CUP GRAND PRIX – CAIXABANK TROPHY CSI W 5 /CSI 2 roun	10.12.2017	CSI-W5*	4	1.60	490	37	11	02.03.2007	M	KWPN	133	11	35	44	30	2234	138	M	6	5b	yes	vertical	vertical	right	no	0	0	75,86	no
La Coruna	WORLD CUP GRAND PRIX – CAIXABANK TROPHY CSI W 5 /CSI 2 roun	10.12.2017	CSI-W5*	4	1.60	490	37	11	02.03.2007	M	KWPN	133	11	35	44	30	2234	138	M	7	5c	yes	spread	oxer	right	no	0	0	75,86	no
La Coruna	WORLD CUP GRAND PRIX – CAIXABANK TROPHY CSI W 5 /CSI 2 roun	10.12.2017	CSI-W5*	4	1.60	490	37	11	02.03.2007	M	KWPN	133	11	35	44	30	2234	138	M	8	6	no	vertical	vertical	left	no	0	0	75,86	no
La Coruna	WORLD CUP GRAND PRIX – CAIXABANK TROPHY CSI W 5 /CSI 2 roun	10.12.2017	CSI-W5*	4	1.60	490	37	11	02.03.2007	M	KWPN	133	11	35	44	30	2234	138	M	9	7	no	spread	oxer	left	no	0	0	75,86	no
La Coruna	WORLD CUP GRAND PRIX – CAIXABANK TROPHY CSI W 5 /CSI 2 roun	10.12.2017	CSI-W5*	4	1.60	490	37	11	02.03.2007	M	KWPN	133	11	35	44	30	2234	138	M	10	8a	yes	spread	oxer	straight	no	0	0	75,86	no
La Coruna	WORLD CUP GRAND PRIX – CAIXABANK TROPHY CSI W 5 /CSI 2 roun	10.12.2017	CSI-W5*	4	1.60	490	37	11	02.03.2007	M	KWPN	133	11	35	44	30	2234	138	M	11	8b	yes	vertical	vertical	straight	no	0	0	75,86	no
La Coruna	WORLD CUP GRAND PRIX – CAIXABANK TROPHY CSI W 5 /CSI 2 roun	10.12.2017	CSI-W5*	4	1.60	490	37	11	02.03.2007	M	KWPN	133	11	35	44	30	2234	138	M	12	9	no	spread	oxer with water	left	no	0	0	75,86	no
La Coruna	WORLD CUP GRAND PRIX – CAIXABANK TROPHY CSI W 5 /CSI 2 roun	10.12.2017	CSI-W5*	4	1.60	490	37	11	02.03.2007	M	KWPN	133	11	35	44	30	2234	138	M	13	10	no	vertical	vertical	right	no	0	0	75,86	no
La Coruna	WORLD CUP GRAND PRIX – CAIXABANK TROPHY CSI W 5 /CSI 2 roun	10.12.2017	CSI-W5*	4	1.60	490	37	11	02.03.2007	M	KWPN	133	11	35	44	30	2234	138	M	14	11	no	spread	oxer	right	no	0	0	75,86	no
La Coruna	WORLD CUP GRAND PRIX – CAIXABANK TROPHY CSI W 5 /CSI 2 roun	10.12.2017	CSI-W5*	4	1.60	490	37	11	02.03.2007	M	KWPN	133	11	35	44	30	2234	138	M	15	12	no	vertical	vertical	straight	no	0	0	75,86	no
La Coruna	WORLD CUP GRAND PRIX – CAIXABANK TROPHY CSI W 5 /CSI 2 roun	10.12.2017	CSI-W5*	4	1.60	490	37	11	02.03.2007	M	KWPN	133	11	35	44	30	2234	138	M	16	13	no	spread	oxer	straight	no	0	0	75,86	no
La Coruna	WORLD CUP GRAND PRIX – CAIXABANK TROPHY CSI W 5 /CSI 2 roun	10.12.2017	CSI-W5*	4	1.60	490	37	11	02.03.2007	M	KWPN	133	11	35	44	30	2234	138	M	17	14	no	vertical	vertical with water	left	no	0	0	75,86	no
La Coruna	WORLD CUP GRAND PRIX – CAIXABANK TROPHY CSI W 5 /CSI 2 roun	10.12.2017	CSI-W5*	4	1.60	490	19	11	15.05.2007	M	KWPN	97	5	36	47	44	1871	37	F	1	1	no	spread	oxer	left	no	0	0	79,61	no
La Coruna	WORLD CUP GRAND PRIX – CAIXABANK TROPHY CSI W 5 /CSI 2 roun	10.12.2017	CSI-W5*	4	1.60	490	19	11	15.05.2007	M	KWPN	97	5	36	47	44	1871	37	F	2	2	no	vertical	vertical	left	no	0	0	79,61	no
La Coruna	WORLD CUP GRAND PRIX – CAIXABANK TROPHY CSI W 5 /CSI 2 roun	10.12.2017	CSI-W5*	4	1.60	490	19	11	15.05.2007	M	KWPN	97	5	36	47	44	1871	37	F	3	3	no	spread	oxer	straight	no	0	0	79,61	no
La Coruna	WORLD CUP GRAND PRIX – CAIXABANK TROPHY CSI W 5 /CSI 2 roun	10.12.2017	CSI-W5*	4	1.60	490	19	11	15.05.2007	M	KWPN	97	5	36	47	44	1871	37	F	4	4	no	spread	oxer	right	no	0	0	79,61	no
La Coruna	WORLD CUP GRAND PRIX – CAIXABANK TROPHY CSI W 5 /CSI 2 roun	10.12.2017	CSI-W5*	4	1.60	490	19	11	15.05.2007	M	KWPN	97	5	36	47	44	1871	37	F	5	5a	yes	vertical	vertical	right	no	0	0	79,61	no
La Coruna	WORLD CUP GRAND PRIX – CAIXABANK TROPHY CSI W 5 /CSI 2 roun	10.12.2017	CSI-W5*	4	1.60	490	19	11	15.05.2007	M	KWPN	97	5	36	47	44	1871	37	F	6	5b	yes	vertical	vertical	right	no	0	0	79,61	no
La Coruna	WORLD CUP GRAND PRIX – CAIXABANK TROPHY CSI W 5 /CSI 2 roun	10.12.2017	CSI-W5*	4	1.60	490	19	11	15.05.2007	M	KWPN	97	5	36	47	44	1871	37	F	7	5c	yes	spread	oxer	right	no	0	0	79,61	no
La Coruna	WORLD CUP GRAND PRIX – CAIXABANK TROPHY CSI W 5 /CSI 2 roun	10.12.2017	CSI-W5*	4	1.60	490	19	11	15.05.2007	M	KWPN	97	5	36	47	44	1871	37	F	8	6	no	vertical	vertical	left	no	0	0	79,61	no
La Coruna	WORLD CUP GRAND PRIX – CAIXABANK TROPHY CSI W 5 /CSI 2 roun	10.12.2017	CSI-W5*	4	1.60	490	19	11	15.05.2007	M	KWPN	97	5	36	47	44	1871	37	F	9	7	no	spread	oxer	left	no	0	0	79,61	no
La Coruna	WORLD CUP GRAND PRIX – CAIXABANK TROPHY CSI W 5 /CSI 2 roun	10.12.2017	CSI-W5*	4	1.60	490	19	11	15.05.2007	M	KWPN	97	5	36	47	44	1871	37	F	10	8a	yes	spread	oxer	straight	no	0	0	79,61	no
La Coruna	WORLD CUP GRAND PRIX – CAIXABANK TROPHY CSI W 5 /CSI 2 roun	10.12.2017	CSI-W5*	4	1.60	490	19	11	15.05.2007	M	KWPN	97	5	36	47	44	1871	37	F	11	8b	yes	vertical	vertical	straight	no	0	0	79,61	no
La Coruna	WORLD CUP GRAND PRIX – CAIXABANK TROPHY CSI W 5 /CSI 2 roun	10.12.2017	CSI-W5*	4	1.60	490	19	11	15.05.2007	M	KWPN	97	5	36	47	44	1871	37	F	12	9	no	spread	oxer with water	left	no	0	0	79,61	no
La Coruna	WORLD CUP GRAND PRIX – CAIXABANK TROPHY CSI W 5 /CSI 2 roun	10.12.2017	CSI-W5*	4	1.60	490	19	11	15.05.2007	M	KWPN	97	5	36	47	44	1871	37	F	13	10	no	vertical	vertical	right	no	0	0	79,61	no
La Coruna	WORLD CUP GRAND PRIX – CAIXABANK TROPHY CSI W 5 /CSI 2 roun	10.12.2017	CSI-W5*	4	1.60	490	19	11	15.05.2007	M	KWPN	97	5	36	47	44	1871	37	F	14	11	no	spread	oxer	right	no	0	0	79,61	no
La Coruna	WORLD CUP GRAND PRIX – CAIXABANK TROPHY CSI W 5 /CSI 2 roun	10.12.2017	CSI-W5*	4	1.60	490	19	11	15.05.2007	M	KWPN	97	5	36	47	44	1871	37	F	15	12	no	vertical	vertical	straight	no	0	0	79,61	no
La Coruna	WORLD CUP GRAND PRIX – CAIXABANK TROPHY CSI W 5 /CSI 2 roun	10.12.2017	CSI-W5*	4	1.60	490	19	11	15.05.2007	M	KWPN	97	5	36	47	44	1871	37	F	16	13	no	spread	oxer	straight	no	0	0	79,61	no
La Coruna	WORLD CUP GRAND PRIX – CAIXABANK TROPHY CSI W 5 /CSI 2 roun	10.12.2017	CSI-W5*	4	1.60	490	19	11	15.05.2007	M	KWPN	97	5	36	47	44	1871	37	F	17	14	no	vertical	vertical with water	left	no	0	0	79,61	no
La Coruna	WORLD CUP GRAND PRIX – CAIXABANK TROPHY CSI W 5 /CSI 2 roun	10.12.2017	CSI-W5*	4	1.60	285	19	11	15.05.2007	M	KWPN	97	5	36	47	44	1871	37	F	1	1	no	vertical	vertical	left	no	0	0	40,08	no
La Coruna	WORLD CUP GRAND PRIX – CAIXABANK TROPHY CSI W 5 /CSI 2 roun	10.12.2017	CSI-W5*	4	1.60	285	19	11	15.05.2007	M	KWPN	97	5	36	47	44	1871	37	F	2	2	no	spread	oxer	right	no	0	0	40,08	no
La Coruna	WORLD CUP GRAND PRIX – CAIXABANK TROPHY CSI W 5 /CSI 2 roun	10.12.2017	CSI-W5*	4	1.60	285	19	11	15.05.2007	M	KWPN	97	5	36	47	44	1871	37	F	3	3	no	spread	oxer with water	left	no	0	0	40,08	no
La Coruna	WORLD CUP GRAND PRIX – CAIXABANK TROPHY CSI W 5 /CSI 2 roun	10.12.2017	CSI-W5*	4	1.60	285	19	11	15.05.2007	M	KWPN	97	5	36	47	44	1871	37	F	4	4	no	vertical	vertical	right	no	0	0	40,08	no
La Coruna	WORLD CUP GRAND PRIX – CAIXABANK TROPHY CSI W 5 /CSI 2 roun	10.12.2017	CSI-W5*	4	1.60	285	19	11	15.05.2007	M	KWPN	97	5	36	47	44	1871	37	F	5	5	no	spread	oxer	right	no	0	0	40,08	no
La Coruna	WORLD CUP GRAND PRIX – CAIXABANK TROPHY CSI W 5 /CSI 2 roun	10.12.2017	CSI-W5*	4	1.60	285	19	11	15.05.2007	M	KWPN	97	5	36	47	44	1871	37	F	6	6a	yes	vertical	vertical	right	no	0	0	40,08	no
La Coruna	WORLD CUP GRAND PRIX – CAIXABANK TROPHY CSI W 5 /CSI 2 roun	10.12.2017	CSI-W5*	4	1.60	285	19	11	15.05.2007	M	KWPN	97	5	36	47	44	1871	37	F	7	6b	yes	spread	oxer	right	no	0	0	40,08	no
La Coruna	WORLD CUP GRAND PRIX – CAIXABANK TROPHY CSI W 5 /CSI 2 roun	10.12.2017	CSI-W5*	4	1.60	285	19	11	15.05.2007	M	KWPN	97	5	36	47	44	1871	37	F	8	7	no	vertical	vertical	left	no	0	0	40,08	no
La Coruna	WORLD CUP GRAND PRIX – CAIXABANK TROPHY CSI W 5 /CSI 2 roun	10.12.2017	CSI-W5*	4	1.60	490	197	11	30.04.2007	G	AA	96	0	37	59	42	1314	68	M	1	1	no	spread	oxer	left	no	0	0	80,55	no
La Coruna	WORLD CUP GRAND PRIX – CAIXABANK TROPHY CSI W 5 /CSI 2 roun	10.12.2017	CSI-W5*	4	1.60	490	197	11	30.04.2007	G	AA	96	0	37	59	42	1314	68	M	2	2	no	vertical	vertical	left	no	0	0	80,55	no
La Coruna	WORLD CUP GRAND PRIX – CAIXABANK TROPHY CSI W 5 /CSI 2 roun	10.12.2017	CSI-W5*	4	1.60	490	197	11	30.04.2007	G	AA	96	0	37	59	42	1314	68	M	3	3	no	spread	oxer	straight	no	0	0	80,55	no
La Coruna	WORLD CUP GRAND PRIX – CAIXABANK TROPHY CSI W 5 /CSI 2 roun	10.12.2017	CSI-W5*	4	1.60	490	197	11	30.04.2007	G	AA	96	0	37	59	42	1314	68	M	4	4	no	spread	oxer	right	no	0	0	80,55	no
La Coruna	WORLD CUP GRAND PRIX – CAIXABANK TROPHY CSI W 5 /CSI 2 roun	10.12.2017	CSI-W5*	4	1.60	490	197	11	30.04.2007	G	AA	96	0	37	59	42	1314	68	M	5	5a	yes	vertical	vertical	right	no	0	0	80,55	no
La Coruna	WORLD CUP GRAND PRIX – CAIXABANK TROPHY CSI W 5 /CSI 2 roun	10.12.2017	CSI-W5*	4	1.60	490	197	11	30.04.2007	G	AA	96	0	37	59	42	1314	68	M	6	5b	yes	vertical	vertical	right	yes	4	0	80,55	no
La Coruna	WORLD CUP GRAND PRIX – CAIXABANK TROPHY CSI W 5 /CSI 2 roun	10.12.2017	CSI-W5*	4	1.60	490	197	11	30.04.2007	G	AA	96	0	37	59	42	1314	68	M	7	5c	yes	spread	oxer	right	no	0	0	80,55	no
La Coruna	WORLD CUP GRAND PRIX – CAIXABANK TROPHY CSI W 5 /CSI 2 roun	10.12.2017	CSI-W5*	4	1.60	490	197	11	30.04.2007	G	AA	96	0	37	59	42	1314	68	M	8	6	no	vertical	vertical	left	no	0	0	80,55	no
La Coruna	WORLD CUP GRAND PRIX – CAIXABANK TROPHY CSI W 5 /CSI 2 roun	10.12.2017	CSI-W5*	4	1.60	490	197	11	30.04.2007	G	AA	96	0	37	59	42	1314	68	M	9	7	no	spread	oxer	left	no	0	0	80,55	no
La Coruna	WORLD CUP GRAND PRIX – CAIXABANK TROPHY CSI W 5 /CSI 2 roun	10.12.2017	CSI-W5*	4	1.60	490	197	11	30.04.2007	G	AA	96	0	37	59	42	1314	68	M	10	8a	yes	spread	oxer	straight	no	0	0	80,55	no
La Coruna	WORLD CUP GRAND PRIX – CAIXABANK TROPHY CSI W 5 /CSI 2 roun	10.12.2017	CSI-W5*	4	1.60	490	197	11	30.04.2007	G	AA	96	0	37	59	42	1314	68	M	11	8b	yes	vertical	vertical	straight	no	0	0	80,55	no
La Coruna	WORLD CUP GRAND PRIX – CAIXABANK TROPHY CSI W 5 /CSI 2 roun	10.12.2017	CSI-W5*	4	1.60	490	197	11	30.04.2007	G	AA	96	0	37	59	42	1314	68	M	12	9	no	spread	oxer with water	left	no	0	0	80,55	no
La Coruna	WORLD CUP GRAND PRIX – CAIXABANK TROPHY CSI W 5 /CSI 2 roun	10.12.2017	CSI-W5*	4	1.60	490	197	11	30.04.2007	G	AA	96	0	37	59	42	1314	68	M	13	10	no	vertical	vertical	right	yes	4	0	80,55	no
La Coruna	WORLD CUP GRAND PRIX – CAIXABANK TROPHY CSI W 5 /CSI 2 roun	10.12.2017	CSI-W5*	4	1.60	490	197	11	30.04.2007	G	AA	96	0	37	59	42	1314	68	M	14	11	no	spread	oxer	right	no	0	0	80,55	no
La Coruna	WORLD CUP GRAND PRIX – CAIXABANK TROPHY CSI W 5 /CSI 2 roun	10.12.2017	CSI-W5*	4	1.60	490	197	11	30.04.2007	G	AA	96	0	37	59	42	1314	68	M	15	12	no	vertical	vertical	straight	no	0	0	80,55	no
La Coruna	WORLD CUP GRAND PRIX – CAIXABANK TROPHY CSI W 5 /CSI 2 roun	10.12.2017	CSI-W5*	4	1.60	490	197	11	30.04.2007	G	AA	96	0	37	59	42	1314	68	M	16	13	no	spread	oxer	straight	no	0	0	80,55	no
La Coruna	WORLD CUP GRAND PRIX – CAIXABANK TROPHY CSI W 5 /CSI 2 roun	10.12.2017	CSI-W5*	4	1.60	490	197	11	30.04.2007	G	AA	96	0	37	59	42	1314	68	M	17	14	no	vertical	vertical with water	left	no	0	0	80,55	no
La Coruna	WORLD CUP GRAND PRIX – CAIXABANK TROPHY CSI W 5 /CSI 2 roun	10.12.2017	CSI-W5*	4	1.60	490	216	13	21.06.2005	G	SBS	196	3	42	542	26	1197	49	F	1	1	no	spread	oxer	left	no	0	0	82,82	no
La Coruna	WORLD CUP GRAND PRIX – CAIXABANK TROPHY CSI W 5 /CSI 2 roun	10.12.2017	CSI-W5*	4	1.60	490	216	13	21.06.2005	G	SBS	196	3	42	542	26	1197	49	F	2	2	no	vertical	vertical	left	no	0	0	82,82	no
La Coruna	WORLD CUP GRAND PRIX – CAIXABANK TROPHY CSI W 5 /CSI 2 roun	10.12.2017	CSI-W5*	4	1.60	490	216	13	21.06.2005	G	SBS	196	3	42	542	26	1197	49	F	3	3	no	spread	oxer	straight	no	0	0	82,82	no
La Coruna	WORLD CUP GRAND PRIX – CAIXABANK TROPHY CSI W 5 /CSI 2 roun	10.12.2017	CSI-W5*	4	1.60	490	216	13	21.06.2005	G	SBS	196	3	42	542	26	1197	49	F	4	4	no	spread	oxer	right	no	0	0	82,82	no
La Coruna	WORLD CUP GRAND PRIX – CAIXABANK TROPHY CSI W 5 /CSI 2 roun	10.12.2017	CSI-W5*	4	1.60	490	216	13	21.06.2005	G	SBS	196	3	42	542	26	1197	49	F	5	5a	yes	vertical	vertical	right	no	0	0	82,82	no
La Coruna	WORLD CUP GRAND PRIX – CAIXABANK TROPHY CSI W 5 /CSI 2 roun	10.12.2017	CSI-W5*	4	1.60	490	216	13	21.06.2005	G	SBS	196	3	42	542	26	1197	49	F	6	5b	yes	vertical	vertical	right	no	0	0	82,82	no
La Coruna	WORLD CUP GRAND PRIX – CAIXABANK TROPHY CSI W 5 /CSI 2 roun	10.12.2017	CSI-W5*	4	1.60	490	216	13	21.06.2005	G	SBS	196	3	42	542	26	1197	49	F	7	5c	yes	spread	oxer	right	no	0	0	82,82	no
La Coruna	WORLD CUP GRAND PRIX – CAIXABANK TROPHY CSI W 5 /CSI 2 roun	10.12.2017	CSI-W5*	4	1.60	490	216	13	21.06.2005	G	SBS	196	3	42	542	26	1197	49	F	8	6	no	vertical	vertical	left	no	0	0	82,82	no
La Coruna	WORLD CUP GRAND PRIX – CAIXABANK TROPHY CSI W 5 /CSI 2 roun	10.12.2017	CSI-W5*	4	1.60	490	216	13	21.06.2005	G	SBS	196	3	42	542	26	1197	49	F	9	7	no	spread	oxer	left	no	0	0	82,82	no
La Coruna	WORLD CUP GRAND PRIX – CAIXABANK TROPHY CSI W 5 /CSI 2 roun	10.12.2017	CSI-W5*	4	1.60	490	216	13	21.06.2005	G	SBS	196	3	42	542	26	1197	49	F	10	8a	yes	spread	oxer	straight	yes	4	0	82,82	no
La Coruna	WORLD CUP GRAND PRIX – CAIXABANK TROPHY CSI W 5 /CSI 2 roun	10.12.2017	CSI-W5*	4	1.60	490	216	13	21.06.2005	G	SBS	196	3	42	542	26	1197	49	F	11	8b	yes	vertical	vertical	straight	no	0	0	82,82	no
La Coruna	WORLD CUP GRAND PRIX – CAIXABANK TROPHY CSI W 5 /CSI 2 roun	10.12.2017	CSI-W5*	4	1.60	490	216	13	21.06.2005	G	SBS	196	3	42	542	26	1197	49	F	12	9	no	spread	oxer with water	left	no	0	0	82,82	no

La Coruna	WORLD CUP GRAND PRIX – CAIXABANK TROPHY CSI W 5 /CSI 2 rounc	10.12.2017	CSI-W5*	4	1.60	490	216	13	21.06.2005	G	SBS	196	3	42	542	26	1197	49	F	13	10	no	vertical	vertical	right	no	0	0	82,82	no
La Coruna	WORLD CUP GRAND PRIX – CAIXABANK TROPHY CSI W 5 /CSI 2 rounc	10.12.2017	CSI-W5*	4	1.60	490	216	13	21.06.2005	G	SBS	196	3	42	542	26	1197	49	F	14	11	no	spread	oxer	right	no	0	0	82,82	no
La Coruna	WORLD CUP GRAND PRIX – CAIXABANK TROPHY CSI W 5 /CSI 2 rounc	10.12.2017	CSI-W5*	4	1.60	490	216	13	21.06.2005	G	SBS	196	3	42	542	26	1197	49	F	15	12	no	vertical	vertical	straight	no	0	0	82,82	no
La Coruna	WORLD CUP GRAND PRIX – CAIXABANK TROPHY CSI W 5 /CSI 2 rounc	10.12.2017	CSI-W5*	4	1.60	490	216	13	21.06.2005	G	SBS	196	3	42	542	26	1197	49	F	16	13	no	spread	oxer	straight	no	0	0	82,82	no
La Coruna	WORLD CUP GRAND PRIX – CAIXABANK TROPHY CSI W 5 /CSI 2 rounc	10.12.2017	CSI-W5*	4	1.60	490	216	13	21.06.2005	G	SBS	196	3	42	542	26	1197	49	F	17	14	no	vertical	vertical with water	left	no	0	0	82,82	no
La Coruna	WORLD CUP GRAND PRIX – CAIXABANK TROPHY CSI W 5 /CSI 2 rounc	10.12.2017	CSI-W5*	4	1.60	490	42	11	01.01.2007	S	ISH	143	5	44	135	22	458	12	M	1	1	no	spread	oxer	left	no	0	0	0,00	yes
La Coruna	WORLD CUP GRAND PRIX – CAIXABANK TROPHY CSI W 5 /CSI 2 rounc	10.12.2017	CSI-W5*	4	1.60	490	42	11	01.01.2007	S	ISH	143	5	44	135	22	458	12	M	2	2	no	vertical	vertical	left	no	0	0	0,00	yes
La Coruna	WORLD CUP GRAND PRIX – CAIXABANK TROPHY CSI W 5 /CSI 2 rounc	10.12.2017	CSI-W5*	4	1.60	490	42	11	01.01.2007	S	ISH	143	5	44	135	22	458	12	M	3	3	no	spread	oxer	straight	no	0	0	0,00	yes
La Coruna	WORLD CUP GRAND PRIX – CAIXABANK TROPHY CSI W 5 /CSI 2 rounc	10.12.2017	CSI-W5*	4	1.60	490	42	11	01.01.2007	S	ISH	143	5	44	135	22	458	12	M	4	4	no	spread	oxer	right	no	0	0	0,00	yes
La Coruna	WORLD CUP GRAND PRIX – CAIXABANK TROPHY CSI W 5 /CSI 2 rounc	10.12.2017	CSI-W5*	4	1.60	490	42	11	01.01.2007	S	ISH	143	5	44	135	22	458	12	M	5	5a	yes	vertical	vertical	right	no	0	0	0,00	yes
La Coruna	WORLD CUP GRAND PRIX – CAIXABANK TROPHY CSI W 5 /CSI 2 rounc	10.12.2017	CSI-W5*	4	1.60	490	42	11	01.01.2007	S	ISH	143	5	44	135	22	458	12	M	6	5b	yes	vertical	vertical	right	no	0	0	0,00	yes
La Coruna	WORLD CUP GRAND PRIX – CAIXABANK TROPHY CSI W 5 /CSI 2 rounc	10.12.2017	CSI-W5*	4	1.60	490	42	11	01.01.2007	S	ISH	143	5	44	135	22	458	12	M	7	5c	yes	spread	oxer	right	yes	4	0	0,00	yes
La Coruna	WORLD CUP GRAND PRIX – CAIXABANK TROPHY CSI W 5 /CSI 2 rounc	10.12.2017	CSI-W5*	4	1.60	490	42	11	01.01.2007	S	ISH	143	5	44	135	22	458	12	M	8	6	no	vertical	vertical	left	no	0	0	0,00	yes
La Coruna	WORLD CUP GRAND PRIX – CAIXABANK TROPHY CSI W 5 /CSI 2 rounc	10.12.2017	CSI-W5*	4	1.60	490	42	11	01.01.2007	S	ISH	143	5	44	135	22	458	12	M	9	7	no	spread	oxer	left	no	0	0	0,00	yes
La Coruna	WORLD CUP GRAND PRIX – CAIXABANK TROPHY CSI W 5 /CSI 2 rounc	10.12.2017	CSI-W5*	4	1.60	490	42	11	01.01.2007	S	ISH	143	5	44	135	22	458	12	M	10	8a	yes	spread	oxer	straight	no	0	0	0,00	yes
La Coruna	WORLD CUP GRAND PRIX – CAIXABANK TROPHY CSI W 5 /CSI 2 rounc	10.12.2017	CSI-W5*	4	1.60	490	42	11	01.01.2007	S	ISH	143	5	44	135	22	458	12	M	11	8b	yes	vertical	vertical	straight	no	0	0	0,00	yes
La Coruna	WORLD CUP GRAND PRIX – CAIXABANK TROPHY CSI W 5 /CSI 2 rounc	10.12.2017	CSI-W5*	4	1.60	490	42	11	01.01.2007	S	ISH	143	5	44	135	22	458	12	M	12	9	no	spread	oxer with water	left	no	0	0	0,00	yes
La Coruna	WORLD CUP GRAND PRIX – CAIXABANK TROPHY CSI W 5 /CSI 2 rounc	10.12.2017	CSI-W5*	4	1.60	490	42	11	01.01.2007	S	ISH	143	5	44	135	22	458	12	M	13	10	no	vertical	vertical	right	no	0	0	0,00	yes
La Coruna	WORLD CUP GRAND PRIX – CAIXABANK TROPHY CSI W 5 /CSI 2 rounc	10.12.2017	CSI-W5*	4	1.60	490	42	11	01.01.2007	S	ISH	143	5	44	135	22	458	12	M	14	11	no	spread	oxer	right	no	0	0	0,00	yes
La Coruna	WORLD CUP GRAND PRIX – CAIXABANK TROPHY CSI W 5 /CSI 2 rounc	10.12.2017	CSI-W5*	4	1.60	490	42	11	01.01.2007	S	ISH	143	5	44	135	22	458	12	M	15	12	no	vertical	vertical	straight	no	0	0	0,00	yes
La Coruna	WORLD CUP GRAND PRIX – CAIXABANK TROPHY CSI W 5 /CSI 2 rounc	10.12.2017	CSI-W5*	4	1.60	490	42	11	01.01.2007	S	ISH	143	5	44	135	22	458	12	M	16	13	no	spread	oxer	straight	no	0	0	0,00	yes
La Coruna	WORLD CUP GRAND PRIX – CAIXABANK TROPHY CSI W 5 /CSI 2 rounc	10.12.2017	CSI-W5*	4	1.60	490	42	11	01.01.2007	S	ISH	143	5	44	135	22	458	12	M	17	14	no	vertical	vertical with water	left	no	0	0	0,00	yes
La Coruna	WORLD CUP GRAND PRIX – CAIXABANK TROPHY CSI W 5 /CSI 2 rounc	10.12.2017	CSI-W5*	4	1.60	490	140	11	19.03.2007	S	HANN	162	14	47	44	44	1933	114	M	1	1	no	spread	oxer	left	no	0	0	77,37	no
La Coruna	WORLD CUP GRAND PRIX – CAIXABANK TROPHY CSI W 5 /CSI 2 rounc	10.12.2017	CSI-W5*	4	1.60	490	140	11	19.03.2007	S	HANN	162	14	47	44	44	1933	114	M	2	2	no	vertical	vertical	left	no	0	0	77,37	no
La Coruna	WORLD CUP GRAND PRIX – CAIXABANK TROPHY CSI W 5 /CSI 2 rounc	10.12.2017	CSI-W5*	4	1.60	490	140	11	19.03.2007	S	HANN	162	14	47	44	44	1933	114	M	3	3	no	spread	oxer	straight	no	0	0	77,37	no
La Coruna	WORLD CUP GRAND PRIX – CAIXABANK TROPHY CSI W 5 /CSI 2 rounc	10.12.2017	CSI-W5*	4	1.60	490	140	11	19.03.2007	S	HANN	162	14	47	44	44	1933	114	M	4	4	no	spread	oxer	right	no	0	0	77,37	no
La Coruna	WORLD CUP GRAND PRIX – CAIXABANK TROPHY CSI W 5 /CSI 2 rounc	10.12.2017	CSI-W5*	4	1.60	490	140	11	19.03.2007	S	HANN	162	14	47	44	44	1933	114	M	5	5a	yes	vertical	vertical	right	yes	4	0	77,37	no
La Coruna	WORLD CUP GRAND PRIX – CAIXABANK TROPHY CSI W 5 /CSI 2 rounc	10.12.2017	CSI-W5*	4	1.60	490	140	11	19.03.2007	S	HANN	162	14	47	44	44	1933	114	M	6	5b	yes	vertical	vertical	right	no	0	0	77,37	no
La Coruna	WORLD CUP GRAND PRIX – CAIXABANK TROPHY CSI W 5 /CSI 2 rounc	10.12.2017	CSI-W5*	4	1.60	490	140	11	19.03.2007	S	HANN	162	14	47	44	44	1933	114	M	7	5c	yes	spread	oxer	right	no	0	0	77,37	no
La Coruna	WORLD CUP GRAND PRIX – CAIXABANK TROPHY CSI W 5 /CSI 2 rounc	10.12.2017	CSI-W5*	4	1.60	490	140	11	19.03.2007	S	HANN	162	14	47	44	44	1933	114	M	8	6	no	vertical	vertical	left	no	0	0	77,37	no
La Coruna	WORLD CUP GRAND PRIX – CAIXABANK TROPHY CSI W 5 /CSI 2 rounc	10.12.2017	CSI-W5*	4	1.60	490	140	11	19.03.2007	S	HANN	162	14	47	44	44	1933	114	M	9	7	no	spread	oxer	left	no	0	0	77,37	no
La Coruna	WORLD CUP GRAND PRIX – CAIXABANK TROPHY CSI W 5 /CSI 2 rounc	10.12.2017	CSI-W5*	4	1.60	490	140	11	19.03.2007	S	HANN	162	14	47	44	44	1933	114	M	10	8a	yes	spread	oxer	straight	no	0	0	77,37	no
La Coruna	WORLD CUP GRAND PRIX – CAIXABANK TROPHY CSI W 5 /CSI 2 rounc	10.12.2017	CSI-W5*	4	1.60	490	140	11	19.03.2007	S	HANN	162	14	47	44	44	1933	114	M	11	8b	yes	vertical	vertical	straight	no	0	0	77,37	no
La Coruna	WORLD CUP GRAND PRIX – CAIXABANK TROPHY CSI W 5 /CSI 2 rounc	10.12.2017	CSI-W5*	4	1.60	490	140	11	19.03.2007	S	HANN	162	14	47	44	44	1933	114	M	12	9	no	spread	oxer with water	left	no	0	0	77,37	no
La Coruna	WORLD CUP GRAND PRIX – CAIXABANK TROPHY CSI W 5 /CSI 2 rounc	10.12.2017	CSI-W5*	4	1.60	490	140	11	19.03.2007	S	HANN	162	14	47	44	44	1933	114	M	13	10	no	vertical	vertical	right	no	0	0	77,37	no
La Coruna	WORLD CUP GRAND PRIX – CAIXABANK TROPHY CSI W 5 /CSI 2 rounc	10.12.2017	CSI-W5*	4	1.60	490	140	11	19.03.2007	S	HANN	162	14	47	44	44	1933	114	M	14	11	no	spread	oxer	right	no	0	0	77,37	no
La Coruna	WORLD CUP GRAND PRIX – CAIXABANK TROPHY CSI W 5 /CSI 2 rounc	10.12.2017	CSI-W5*	4	1.60	490	140	11	19.03.2007	S	HANN	162	14	47	44	44	1933	114	M	15	12	no	vertical	vertical	straight	no	0	0	77,37	no
La Coruna	WORLD CUP GRAND PRIX – CAIXABANK TROPHY CSI W 5 /CSI 2 rounc	10.12.2017	CSI-W5*	4	1.60	490	140	11	19.03.2007	S	HANN	162	14	47	44	44	1933	114	M	16	13	no	spread	oxer	straight	no	0	0	77,37	no
La Coruna	WORLD CUP GRAND PRIX – CAIXABANK TROPHY CSI W 5 /CSI 2 rounc	10.12.2017	CSI-W5*	4	1.60	490	140	11	19.03.2007	S	HANN	162	14	47	44	44	1933	114	M	17	14	no	vertical	vertical with water	left	no	0	0	77,37	no
La Coruna	WORLD CUP GRAND PRIX – CAIXABANK TROPHY CSI W 5 /CSI 2 rounc	10.12.2017	CSI-W5*	4	1.60	490	36	13	30.05.2005	S	HOLST	223	29	52	52	32	2035	202	M	1	1	no	spread	oxer	left	no	0	0	0,00	yes
La Coruna	WORLD CUP GRAND PRIX – CAIXABANK TROPHY CSI W 5 /CSI 2 rounc	10.12.2017	CSI-W5*	4	1.60	490	36	13	30.05.2005	S	HOLST	223	29	52	52	32	2035	202	M	2	2	no	vertical	vertical	left	no	0	0	0,00	yes
La Coruna	WORLD CUP GRAND PRIX – CAIXABANK TROPHY CSI W 5 /CSI 2 rounc	10.12.2017	CSI-W5*	4	1.60	490	36	13	30.05.2005	S	HOLST	223	29	52	52	32	2035	202	M	3	3	no	spread	oxer	straight	no	0	0	0,00	yes
La Coruna	WORLD CUP GRAND PRIX – CAIXABANK TROPHY CSI W 5 /CSI 2 rounc	10.12.2017	CSI-W5*	4	1.60	490	36	13	30.05.2005	S	HOLST	223	29	52	52	32	2035	202	M	4	4	no	spread	oxer	right	no	0	0	0,00	yes
La Coruna	WORLD CUP GRAND PRIX – CAIXABANK TROPHY CSI W 5 /CSI 2 rounc	10.12.2017	CSI-W5*	4	1.60	490	36	13	30.05.2005	S	HOLST	223	29	52	52	32	2035	202	M	5	5a	yes	vertical	vertical	right	no	0	0	0,00	yes
La Coruna	WORLD CUP GRAND PRIX – CAIXABANK TROPHY CSI W 5 /CSI 2 rounc	10.12.2017	CSI-W5*	4	1.60	490	36	13	30.05.2005	S	HOLST	223	29	52	52	32	2035	202	M	6	5b	yes	vertical	vertical	right	yes	4	0	0,00	yes
La Coruna	WORLD CUP GRAND PRIX – CAIXABANK TROPHY CSI W 5 /CSI 2 rounc	10.12.2017	CSI-W5*	4	1.60	490	36	13	30.05.2005	S	HOLST	223	29	52	52	32	2035	202	M	7	5c	yes	spread	oxer	right	yes	4	0	0,00	yes
La Coruna	WORLD CUP GRAND PRIX – CAIXABANK TROPHY CSI W 5 /CSI 2 rounc	10.12.2017	CSI-W5*	4	1.60	490	36	13	30.05.2005	S	HOLST	223	29	52	52	32	2035	202	M	8	6	no	vertical	vertical	left	no	0	0	0,00	yes
La Coruna	WORLD CUP GRAND PRIX – CAIXABANK TROPHY CSI W 5 /CSI 2 rounc	10.12.2017	CSI-W5*	4	1.60	490	36	13	30.05.2005	S	HOLST	223	29	52	52	32	2035	202	M	9	7	no	spread	oxer	left	no	0	0	0,00	yes
La Coruna	WORLD CUP GRAND PRIX – CAIXABANK TROPHY CSI W 5 /CSI 2 rounc	10.12.2017	CSI-W5*	4	1.60	490	36	13	30.05.2005	S	HOLST	223	29	52	52	32	2035	202	M	10	8a	yes	spread	oxer	straight	yes	4	0	0,00	yes
La Coruna	WORLD CUP GRAND PRIX – CAIXABANK TROPHY CSI W 5 /CSI 2 rounc	10.12.2017	CSI-W5*	4	1.60	490	36	13	30.05.2005	S	HOLST	223	29	52	52	32	2035	202	M	11	8b	yes	vertical	vertical	straight	yes	4	0	0,00	yes
La Coruna	WORLD CUP GRAND PRIX – CAIXABANK TROPHY CSI W 5 /CSI 2 rounc	10.12.2017	CSI-W5*	4	1.60	490	36	13	30.05.2005	S	HOLST	223	29	52	52	32	2035	202	M	12	9	no	spread	oxer with water	left	no	0	0	0,00	yes
La Coruna	WORLD CUP GRAND PRIX – CAIXABANK TROPHY CSI W 5 /CSI 2 rounc	10.12.2017	CSI-W5*	4	1.60	490	36	13	30.05.2005	S	HOLST	223	29	52	52	32	2035	202	M	13	10	no	vertical	vertical	right	no	0	0	0,00	yes
La Coruna	WORLD CUP GRAND PRIX – CAIXABANK TROPHY CSI W 5 /CSI 2 rounc	10.12.2017	CSI-W5*	4	1.60	490	36	13	30.05.2005	S	HOLST	223	29	52	52	32	2035	202	M	14	11	no	spread	oxer	right	no	0	0	0,00	yes
La Coruna	WORLD CUP GRAND PRIX – CAIXABANK TROPHY CSI W 5 /CSI 2 rounc	10.12.2017	CSI-W5*	4	1.60	490	36	13	30.05.2005	S	HOLST	223	29	52	52	32	2035	202	M	15	12	no	vertical	vertical	straight	no	0	0	0,00	yes
La Coruna	WORLD CUP GRAND PRIX – CAIXABANK TROPHY CSI W 5 /CSI 2 rounc	10.12.2017	CSI-W5*	4	1.60	490	36	13	30.05.2005	S	HOLST	223	29	52	52	32	2035	202	M	16	13	no	spread	oxer	straight	no	0	0	0,00	yes
La Coruna	WORLD CUP GRAND PRIX – CAIXABANK TROPHY CSI W 5 /CSI 2 rounc	10.12.2017	CSI-W5*	4	1.60	490	36	13	30.05.2005	S	HOLST	223	29	52	52	32	2035	202	M	17	14	no	vertical	vertical with water	left	no	0	0	0,00	yes
La Coruna	WORLD CUP GRAND PRIX – CAIXABANK TROPHY CSI W 5 /CSI 2 rounc	10.12.2017	CSI-W5*	4	1.60	490	31	12	15.04.2006	S	OS	292	11	59	57	32	2641	229	M	1	1	no	spread	oxer	left	no	0	0	74,01	no
La Coruna	WORLD CUP GRAND PRIX – CAIXABANK TROPHY CSI W 5 /CSI 2 rounc	10.12.2017	CSI-W5*	4	1.60	490	31	12	15.04.2006	S	OS	292	11	59	57	32	2641	229	M	2	2	no	vertical	vertical	left	no	0	0	74,01	no
La Coruna	WORLD CUP GRAND PRIX – CAIXABANK TROPHY CSI W 5 /CSI 2 rounc	10.12.2017	CSI-W5*	4	1.60	490	31	12	15.04.2006	S	OS	292	11	59	57	32	2641	229	M	3	3	no	spread	oxer	straight	no	0	0	74,01	no
La Coruna	WORLD CUP GRAND PRIX – CAIXABANK TROPHY CSI W 5 /CSI 2 rounc	10.12.2017	CSI-W5*	4	1.60	490	31	12	15.04.2006	S	OS	292	11	59	57	32	2641	229	M	4	4	no	spread	oxer	right	no	0	0	74,01	no
La Coruna	WORLD CUP GRAND PRIX – CAIXABANK TROPHY CSI W 5 /CSI 2 rounc	10.12.2017	CSI-W5*	4	1.60	490	31	12	15.04.2006	S	OS	292	11	59	57	32	2641	229	M	5	5a	yes	vertical	vertical	right	no	0	0	74,01	no
La Coruna	WORLD CUP GRAND PRIX – CAIXABANK TROPHY CSI W 5 /CSI 2 rounc	10.12.2017	CSI-W5*	4	1.60	490	31	12	15.04.2006	S	OS	292	11	59	57	32	2641	229	M	6	5b	yes	vertical	vertical	right	no	0	0	74,01	no
La Coruna	WORLD CUP GRAND PRIX – CAIXABANK TROPHY CSI W 5 /CSI 2 rounc	10.12.2017	CSI-W5*	4	1.60	490	31	12	15.04.2006	S	OS	292	11	59	57	32	2641	229	M	7	5c	yes	spread	oxer	right	no	0	0	74,01	no
La Coruna	WORLD CUP GRAND PRIX – CAIXABANK TROPHY CSI W 5 /CSI 2 rounc	10.12.2017	CSI-W5*	4	1.60	490	31	12	15.04.2006	S	OS	292	11	59	57	32	2641	229	M	8	6	no	vertical	vertical	left	yes	4	0	74,01	no
La Coruna	WORLD CUP GRAND PRIX – CAIXABANK TROPHY CSI W 5 /CSI 2 rounc	10.12.2017	CSI-W5*	4	1.60	490	31	12	15.04.2006	S	OS	292	11	59	57	32	2641	229	M	9	7	no	spread	oxer	left	no	0	0	74,01	no
La Coruna	WORLD CUP GRAND PRIX – CAIXABANK TROPHY CSI W 5 /CSI 2 rounc	10.12.2017	CSI-W5*	4	1.60	490	31	12	15.04.2006	S	OS	292	11	59	57	32	2641	229	M	10	8a	yes	spread	oxer	straight	no	0	0	74,01	no
La Coruna	WORLD CUP GRAND PRIX – CAIXABANK TROPHY CSI W 5 /CSI 2 rounc	10.12.2017	CSI-W5*	4	1.60	490	31	12	15.04.2006	S	OS	292	11	59	57	32	2641	229	M	11	8b	yes	vertical	vertical	straight	no	0	0	74,01	no
La Coruna	WORLD CUP GRAND PRIX – CAIXABANK TROPHY CSI W 5 /CSI 2 rounc	10.12.2017	CSI-W5*	4	1.60	490	31	12	15.04.2006	S	OS	292	11	59	57	32	2641	229	M	12	9	no	spread	oxer with water	left	no	0	0	74,01	no
La Coruna	WORLD CUP GRAND PRIX – CAIXABANK TROPHY CSI W 5 /CSI 2 rounc	10.12.2017	CSI-W5*	4	1.60	490	31	12	15.04.2006	S	OS	292	11	59	57	32	2641	229	M	13	10	no	vertical	vertical	right	no	0	0	74,01	no
La Coruna	WORLD CUP GRAND PRIX – CAIXABANK TROPHY CSI W 5 /CSI 2 rounc	10.12.2017	CSI-W5*	4	1.60	490	31	12	15.04.2006	S	OS	292	11	59	57	32	2641	229	M	14	11	no	spread	oxer	right	no	0	0	74,01	no
La Coruna	WORLD CUP GRAND PRIX – CAIXABANK TROPHY CSI W 5 /CSI 2 rounc	10.12.2017	CSI-W5*	4	1.60	490	31	12	15.04.2006	S	OS	292	11	59	57	32	2641	229	M	15	12	no	vertical	vertical	straight	no	0	0	74,01	no
La Coruna	WORLD CUP GRAND PRIX – CAIXABANK TROPHY CSI W 5 /CSI 2 rounc	10.12.2017	CSI-W5*	4	1.60	490	31	12	15.04.2006	S	OS	292	11	59	57	32	2641	229	M	16	13	no	spread	oxer	straight	no	0	0	74,01	no
La Coruna	WORLD CUP GRAND PRIX – CAIXABANK TROPHY CSI W 5 /CSI 2 rounc	10.12.2017	CSI-W5*	4	1.60	490	31	12	15.04.2006	S	OS	292	11	59	57	32	2641	229	M	17	14	no	vertical	vertical with water	left	no	0	0	74,01	no
La Coruna	WORLD CUP GRAND PRIX – CAIXABANK TROPHY CSI W 5 /CSI 2 rounc	10.12.2017	CSI-W5*	4	1.60	490	68	15	30.06.2003	G	BWP	209	4	61	297	35	1222	40	F	1	1	no	spread	oxer	left	no	0	0	80,27	no
La Coruna	WORLD CUP GRAND PRIX – CAIXABANK TROPHY CSI W 5 /CSI 2 rounc	10.12.2017	CSI-W5*	4	1.60	490	68	15	30.06.2003	G	BWP	209	4	61	297	35	1222	40	F	2	2	no	vertical	vertical	left	no	0	0	80,27	no
La Coruna	WORLD CUP GRAND PRIX – CAIXABANK TROPHY CSI W 5 /CSI 2 rounc	10.12.2017	CSI-W5*	4	1.60	490	68	15	30.06.2003	G	BWP	209	4	61	297	35	1222	40	F	3	3	no	spread	oxer	straight	no	0	0	80,27	no
La Coruna	WORLD CUP GRAND PRIX – CAIXABANK TROPHY CSI W 5 /CSI 2 rounc	10.12.2017	CSI-W5*	4	1.60	490	68	15	30.06.2003	G	BWP	209	4	61	297	35	1222	40	F	4	4	no	spread	oxer	right	no	0	0	80,27	no
La Coruna	WORLD CUP GRAND PRIX – CAIXABANK TROPHY CSI W 5 /CSI 2 rounc	10.12.2017	CSI-W5*	4	1.60	490	68	15	30.06.2003	G	BWP	209	4	61	297	35	1222	40	F	5	5a	yes	vertical	vertical	right	no	0	0	80,27	no
La Coruna	WORLD CUP GRAND PRIX – CAIXABANK TROPHY CSI W 5 /CSI 2 rounc	10.12.2017	CSI-W5*	4	1.60	490	68	15	30.06.2003	G	BWP	209	4	61	297	35	1222	40	F	6	5b	yes	vertical	vertical	right	no	0	0	80,27	no
La Coruna	WORLD CUP GRAND PRIX – CAIXABANK TROPHY CSI W 5 /CSI 2 rounc	10.12.2017	CSI-W5*	4	1.60	490	68	15	30.06.2003	G	BWP	209	4	61	297	35	1222	40	F	7	5c	yes	spread	oxer	right	yes	4	0	80,27	no
La Coruna	WORLD CUP GRAND PRIX – CAIXABANK TROPHY CSI W 5 /CSI 2 rounc	10.12.2017	CSI-W5*	4	1.60	490	68	15	30.06.2003	G	BWP	209	4	61	297	35	1222	40	F	8	6	no	vertical	vertical	left	no	0	0	80,27	no
La Coruna	WORLD CUP GRAND PRIX – CAIXABANK TROPHY CSI W 5 /CSI 2 rounc	10.12.2017	CSI-W5*	4	1.60	490	68	15	30.06.2003	G	BWP	209	4	61	297	35	1222	40	F	9	7	no	spread	oxer	left	no	0	0	80,27	no
La Coruna	WORLD CUP GRAND PRIX – CAIXABANK TROPHY CSI W 5 /CSI 2 rounc	10.12.2017	CSI-W5*	4	1.60	490	68	15	30.06.2003	G	BWP	209	4	61	297	35	1222	40	F	10	8a	yes	spread	oxer	straight	no	0	0	80,27	no
La Coruna	WORLD CUP GRAND PRIX – CAIXABANK TROPHY CSI W 5 /CSI 2 rounc	10.12.2017	CSI-W5*	4	1.60	490	68	15	30.06.2003	G	BWP	209	4	61	297	35	1222	40	F	11	8b	yes	vertical	vertical	straight	no	0	0	80,27	no
La Coruna	WORLD CUP GRAND PRIX – CAIXABANK TROPHY CSI W 5 /CSI 2 rounc	10.12.2017	CSI-W5*	4	1.60	490	68	15	30.06.2003	G	BWP	209	4	61	297	35	1222	40	F	12	9	no	spread	oxer with water	left	no	0	0	80,27	no
La Coruna	WORLD CUP GRAND PRIX – CAIXABANK TROPHY CSI W 5 /CSI 2 rounc	10.12.2017	CSI-W5*	4	1.60	490	68	15	30.06.2003	G	BWP	209	4	61	297	35	1222	40	F	13	10	no	vertical	vertical	right	no	0	0	80,27	no
La Coruna	WORLD CUP GRAND PRIX – CAIXABANK TROPHY CSI W 5 /CSI 2 rounc	10.12.2017	CSI-W5*	4	1.60	490	68	15	30.06.2003	G	BWP	209	4	61	297	35	1222	40	F	14	11	no	spread	oxer	right	no	0	0	80,27	no
La Coruna	WORLD CUP GRAND PRIX – CAIXABANK TROPHY CSI W 5 /CSI 2 rounc	10.12.2017	CSI-W5*	4	1.60	490	68	15	30.06.2003	G	BWP	209	4	61	297	35	1222	40	F	15	12	no	vertical	vertical	straight	no	0	0	80,27	no
La Coruna	WORLD CUP GRAND PRIX – CAIXABANK TROPHY CSI W 5 /CSI 2 rounc	10.12.2017	CSI-W5*	4	1.60	490	68	15	30.06.2003	G	BWP	209	4	61	297	35	1222	40	F	16	13	no	spread	oxer	straight	no	0	0	80,27	no
La Coruna	WORLD CUP GRAND PRIX – CAIXABANK TROPHY CSI W 5 /CSI 2 rounc	10.12.2017	CSI-W5*	4	1.60	490	68	15	30.06.2003	G	BWP	209	4	61	297	35	1222	40	F	17	14	no	vertical	vertical with water	left	no	0	0	80,27	no
La Coruna	WORLD CUP GRAND PRIX – CAIXABANK TROPHY CSI W 5 /CSI 2 rounc	10.12.2017	CSI-W5*	4	1.60	490	55	15	05.05.2003	S	WESTF	199	6	71	12	44	2177	93	M	1	1	no	spread	oxer	left	no	0	0	77,99	no
La Coruna	WORLD CUP GRAND PRIX – CAIXABANK TROPHY CSI W 5 /CSI 2 rounc	10.12.2017	CSI-W5*	4	1.60	490	55	15	05.05.2003	S	WESTF	199	6	71	12	44	2177	93	M	2	2	no	vertical	vertical	left	no	0	0	77,99	no
La Coruna	WORLD CUP GRAND PRIX – CAIXABANK TROPHY CSI W 5 /CSI 2 rounc	10.12.2017	CSI-W5*	4	1.60	490	55	15	05.05.2003	S	WESTF	199	6	71	12	44	2177	93	M	3	3	no	spread	oxer	straight	no	0	0	77,99	no
La Coruna	WORLD CUP GRAND PRIX – CAIXABANK TROPHY CSI W 5 /CSI 2 rounc	10.12.2017	CSI-W5*	4	1.60	490	55	15	05.05.2003	S	WESTF	199	6	71	12	44	2177	93	M	4	4	no	spread	oxer	right	no	0	0	77,99	no
La Coruna	WORLD CUP GRAND PRIX – CAIXABANK TROPHY CSI W 5 /CSI 2 rounc	10.12.2017	CSI-W5*	4	1.60	490	55	15	05.05.2003	S	WESTF	199	6	71	12	44	2177	93	M	5	5a	yes	vertical	vertical	right	no	0	0	77,99	no
La Coruna	WORLD CUP GRAND PRIX – CAIXABANK TROPHY CSI W 5 /CSI 2 rounc	10.12.2017	CSI-W5*	4	1.60	490	55	15	05.05.2003	S	WESTF	199	6	71	12	44	2177	93	M	6	5b	yes	vertical	vertical	right	no	0	0	77,99	no
La Coruna	WORLD CUP GRAND PRIX – CAIXABANK TROPHY CSI W 5 /CSI 2 rounc	10.12.2017	CSI-W5*	4	1.60	490	55	15	05.05.2003	S	WESTF	199	6	71	12	44	2177	93	M	7	5c	yes	spread	oxer	right	no	0	0	77,99	no
La Coruna	WORLD CUP GRAND PRIX – CAIXABANK TROPHY CSI W 5 /CSI 2 rounc	10.12.2017	CSI-W5*	4	1.60	490	55	15	05.05.2003	S	WESTF	199	6	71	12	44	2177	93	M	8	6	no	vertical	vertical	left	no	0	0	77,99	no
La Coruna	WORLD CUP GRAND PRIX – CAIXABANK TROPHY CSI W 5 /CSI 2 rounc	10.12.2017	CSI-W5*	4	1.60	490	55	15	05.05.2003	S	WESTF	199	6	71	12	44	2177	93	M	9	7	no	spread	oxer	left	no	0	0	77,99	no
La Coruna	WORLD CUP GRAND PRIX – CAIXABANK TROPHY CSI W 5 /CSI 2 rounc	10.12.2017	CSI-W5*	4	1.60	490	55	15	05.05.2003	S	WESTF	199	6	71	12	44	2177	93	M	10	8a	yes	spread	oxer	straight	no	0	0	77,99	no
La Coruna	WORLD CUP GRAND PRIX – CAIXABANK TROPHY CSI W 5 /CSI 2 rounc	10.12.2017	CSI-W5*	4	1.60	490	55	15	05.05.2003	S	WESTF	199	6	71	12	44	2177	93	M	11	8b	yes	vertical	vertical	straight	no	0	0	77,99	no
La Coruna	WORLD CUP GRAND PRIX – CAIXABANK TROPHY CSI W 5 /CSI 2 rounc	10.12.2017	CSI-W5*	4	1.60	490	55	15	05.05.2003	S	WESTF	199	6	71	12	44	2177	93	M	12	9	no	spread	oxer with water	left	no	0	0	77,99	no
La Coruna	WORLD CUP GRAND PRIX – CAIXABANK TROPHY CSI W 5 /CSI 2 rounc	10.12.2017	CSI-W5*	4	1.60	490	55	15	05.05.2003	S	WESTF	199	6	71	12	44	2177	93	M	13	10	no	vertical	vertical	right	no	0	0	77,99	no
La Coruna	WORLD CUP GRAND PRIX – CAIXABANK TROPHY CSI W 5 /CSI 2 rounc	10.12.2017	CSI-W5*	4	1.60	490	55	15	05.05.2003	S	WESTF	199	6	71	12	44	2177	93	M	14	11	no	spread	oxer	right	yes	4	0	77,99	no
La Coruna	WORLD CUP GRAND PRIX – CAIXABANK TROPHY CSI W 5 /CSI 2 rounc	10.12.2017	CSI-W5*	4	1.60	490	55	15	05.05.2003	S	WESTF	199	6	71	12	44	2177	93	M	15	12	no	vertical	vertical	straight	no	0	0	77,99	no
La Coruna	WORLD CUP GRAND PRIX – CAIXABANK TROPHY CSI W 5 /CSI 2 rounc	10.12.2017	CSI-W5*	4	1.60	490	55	15	05.05.2003	S	WESTF	199	6	71	12	44	2177	93	M	16	13	no	spread	oxer	straight	no	0	0	77,99	no
La Coruna	WORLD CUP GRAND PRIX – CAIXABANK TROPHY CSI W 5 /CSI 2 rounc	10.12.2017	CSI-W5*	4	1.60	490	55	15	05.05.2003	S	WESTF	199	6	71	12	44	2177	93	M	17	14	no	vertical	vertical with water	left	no	0	0	77,99	no
La Coruna	WORLD CUP GRAND PRIX – CAIXABANK TROPHY CSI W 5 /CSI 2 rounc	10.12.2017	CSI-W5*	4	1.60	490	173	13	14.07.2005	G	SF	229	2	74	65	34	1260	61	M	1	1	no	spread	oxer	left	no	0	0	80,22	no
La Coruna	WORLD CUP GRAND PRIX – CAIXABANK TROPHY CSI W 5 /CSI 2 rounc	10.12.2017	CSI-W5*	4	1.60	490	173	13	14.07.2005	G	SF	229	2	74	65	34	1260	61	M	2	2	no	vertical	vertical	left	no	0	0	80,22	no
La Coruna	WORLD CUP GRAND PRIX – CAIXABANK TROPHY CSI W 5 /CSI 2 rounc	10.12.2017	CSI-W5*	4	1.60	490	173	13	14.07.2005	G	SF	229	2	74	65	34	1260	61	M	3	3	no	spread	oxer	straight	no	0	0	80,22	no
La Coruna	WORLD CUP GRAND PRIX – CAIXABANK TROPHY CSI W 5 /CSI 2 rounc	10.12.2017	CSI-W5*	4	1.60	490	173	13	14.07.2005	G	SF	229	2	74	65	34	1260	61	M	4	4	no	spread	oxer	right	no	0	0	80,22	no
La Coruna	WORLD CUP GRAND PRIX – CAIXABANK TROPHY CSI W 5 /CSI 2 rounc	10.12.2017	CSI-W5*	4	1.60	490	173	13	14.07.2005	G	SF	229	2	74	65	34	1260	61	M	5	5a	yes	vertical	vertical	right	no	0	0	80,22	no
La Coruna	WORLD CUP GRAND PRIX – CAIXABANK TROPHY CSI W 5 /CSI 2 rounc	10.12.2017	CSI-W5*	4	1.60	490	173	13	14.07.2005	G	SF	229	2	74	65	34	1260	61	M	6	5b	yes	vertical	vertical	right	no	0	0	80,22	no
La Coruna	WORLD CUP GRAND PRIX – CAIXABANK TROPHY CSI W 5 /CSI 2 rounc	10.12.2017	CSI-W5*	4	1.60	490	173	13	14.07.2005	G	SF	229	2	74	65	34	1260	61	M	7	5c	yes	spread	oxer	right	no	0	0	80,22	no
La Coruna	WORLD CUP GRAND PRIX – CAIXABANK TROPHY CSI W 5 /CSI 2 rounc	10.12.2017	CSI-W5*	4	1.60	490	173	13	14.07.2005	G	SF	229	2	74	65	34	1260	61	M	8	6	no	vertical	vertical	left	no	0	0	80,22	no
La Coruna	WORLD CUP GRAND PRIX – CAIXABANK TROPHY CSI W 5 /CSI 2 rounc	10.12.2017	CSI-W5*	4	1.60	490	173	13	14.07.2005	G	SF	229	2	74	65	34	1260	61	M	9	7	no	spread	oxer	left	no	0	0	80,22	no
La Coruna	WORLD CUP GRAND PRIX – CAIXABANK TROPHY CSI W 5 /CSI 2 rounc	10.12.2017	CSI-W5*	4	1.60	490	173	13	14.07.2005	G	SF	229	2	74	65	34	1260	61	M	10	8a	yes	spread	oxer	straight	no	0	0	80,22	no
La Coruna	WORLD CUP GRAND PRIX – CAIXABANK TROPHY CSI W 5 /CSI 2 rounc	10.12.2017	CSI-W5*	4	1.60	490	173	13	14.07.2005	G	SF	229	2	74	65	34	1260	61	M	11	8b	yes	vertical	vertical	straight	no	0	0	80,22	no
La Coruna	WORLD CUP GRAND PRIX – CAIXABANK TROPHY CSI W 5 /CSI 2 rounc	10.12.2017	CSI-W5*	4	1.60	490	173	13	14.07.2005	G	SF	229	2	74	65	34	1260	61	M	12	9	no	spread	oxer with water	left	no	0	0	80,22	no
La Coruna	WORLD CUP GRAND PRIX – CAIXABANK TROPHY CSI W 5 /CSI 2 rounc	10.12.2017	CSI-W5*	4	1.60	490	173	13	14.07.2005	G	SF	229	2	74	65	34	1260	61	M	13	10	no	vertical	vertical	right	no	0	0	80,22	no
La Coruna	WORLD CUP GRAND PRIX – CAIXABANK TROPHY CSI W 5 /CSI 2 rounc	10.12.2017	CSI-W5*	4	1.60	490	173	13	14.07.2005	G	SF	229	2	74	65	34	1260	61	M	14	11	no	spread	oxer	right	no	0	0	80,22	no
La Coruna	WORLD CUP GRAND PRIX – CAIXABANK TROPHY CSI W 5 /CSI 2 rounc	10.12.2017	CSI-W5*	4	1.60	490	173	13	14.07.2005	G	SF	229	2	74	65	34	1260	61	M	15	12	no	vertical	vertical	straight	yes	4	0	80,22	no
La Coruna	WORLD CUP GRAND PRIX – CAIXABANK TROPHY CSI W 5 /CSI 2 rounc	10.12.2017	CSI-W5*	4	1.60	490	173	13	14.07.2005	G	SF	229	2	74	65	34	1260	61	M	16	13	no	spread	oxer	straight	no	0	0	80,22	no
La Coruna	WORLD CUP GRAND PRIX – CAIXABANK TROPHY CSI W 5 /CSI 2 rounc	10.12.2017	CSI-W5*	4	1.60	490	173	13	14.07.2005	G	SF	229	2	74	65	34	1260	61	M	17	14	no	vertical	vertical with water	left	no	0	0	80,22	no
La Coruna	WORLD CUP GRAND PRIX – CAIXABANK TROPHY CSI W 5 /CSI 2 rounc	10.12.2017	CSI-W5*	4	1.60	490	120	11	28.04.2007	S	HOLST	147	1	76	55	44	1886	48	M	1	1	no	spread	oxer	left	no	0	0	79,98	no
La Coruna	WORLD CUP GRAND PRIX – CAIXABANK TROPHY CSI W 5 /CSI 2 rounc	10.12.2017	CSI-W5*	4	1.60	490	120	11	28.04.2007	S	HOLST	147	1	76	55	44	1886	48	M	2	2	no	vertical	vertical	left	no	0	0	79,98	no
La Coruna	WORLD CUP GRAND PRIX – CAIXABANK TROPHY CSI W 5 /CSI 2 rounc	10.12.2017	CSI-W5*	4	1.60	490	120	11	28.04.2007	S	HOLST	147	1	76	55	44	1886	48	M	3	3	no	spread	oxer	straight	no	0	0	79,98	no
La Coruna	WORLD CUP GRAND PRIX – CAIXABANK TROPHY CSI W 5 /CSI 2 rounc	10.12.2017	CSI-W5*	4	1.60	490	120	11	28.04.2007	S	HOLST	147	1	76	55	44	1886	48	M	4	4	no	spread	oxer	right	no	0	0	79,98	no
La Coruna	WORLD CUP GRAND PRIX – CAIXABANK TROPHY CSI W 5 /CSI 2 rounc	10.12.2017	CSI-W5*	4	1.60	490	120	11	28.04.2007	S	HOLST	147	1	76	55	44	1886	48	M	5	5a	yes	vertical	vertical	right	no	0	0	79,98	no
La Coruna	WORLD CUP GRAND PRIX – CAIXABANK TROPHY CSI W 5 /CSI 2 rounc	10.12.2017	CSI-W5*	4	1.60	490	120	11	28.04.2007	S	HOLST	147	1	76	55	44	1886	48	M	6	5b	yes	vertical	vertical	right	no	0	0	79,98	no
La Coruna	WORLD CUP GRAND PRIX – CAIXABANK TROPHY CSI W 5 /CSI 2 rounc	10.12.2017	CSI-W5*	4	1.60	490	120	11	28.04.2007	S	HOLST	147	1	76	55	44	1886	48	M	7	5c	yes	spread	oxer	right	no	0	0	79,98	no
La Coruna	WORLD CUP GRAND PRIX – CAIXABANK TROPHY CSI W 5 /CSI 2 rounc	10.12.2017	CSI-W5*	4	1.60	490	120	11	28.04.2007	S	HOLST	147	1	76	55	44	1886	48	M	8	6	no	vertical	vertical	left	no	0	0	79,98	no
La Coruna	WORLD CUP GRAND PRIX – CAIXABANK TROPHY CSI W 5 /CSI 2 rounc	10.12.2017	CSI-W5*	4	1.60	490	120	11	28.04.2007	S	HOLST	147	1	76	55	44	1886	48	M	9	7	no	spread	oxer	left	no	0	0	79,98	no
La Coruna	WORLD CUP GRAND PRIX – CAIXABANK TROPHY CSI W 5 /CSI 2 rounc	10.12.2017	CSI-W5*	4	1.60	490	120	11	28.04.2007	S	HOLST	147	1	76	55	44	1886	48	M	10	8a	yes	spread	oxer	straight	no	0	0	79,98	no
La Coruna	WORLD CUP GRAND PRIX – CAIXABANK TROPHY CSI W 5 /CSI 2 rounc	10.12.2017	CSI-W5*	4	1.60	490	120	11	28.04.2007	S	HOLST	147	1	76	55	44	1886	48	M	11	8b	yes	vertical	vertical	straight	no	0	0	79,98	no
La Coruna	WORLD CUP GRAND PRIX – CAIXABANK TROPHY CSI W 5 /CSI 2 rounc	10.12.2017	CSI-W5*	4	1.60	490	120	11	28.04.2007	S	HOLST	147	1	76	55	44	1886	48	M	12	9	no	spread	oxer with water	left	no	0	0	79,98	no
La Coruna	WORLD CUP GRAND PRIX – CAIXABANK TROPHY CSI W 5 /CSI 2 rounc	10.12.2017	CSI-W5*	4	1.60	490	120	11	28.04.2007	S	HOLST	147	1	76	55	44	1886	48	M	13	10	no	vertical	vertical	right	no	0	0	79,98	no
La Coruna	WORLD CUP GRAND PRIX – CAIXABANK TROPHY CSI W 5 /CSI 2 rounc	10.12.2017	CSI-W5*	4	1.60	490	120	11	28.04.2007	S	HOLST	147	1	76	55	44	1886	48	M	14	11	no	spread	oxer	right	no	0	0	79,98	no
La Coruna	WORLD CUP GRAND PRIX – CAIXABANK TROPHY CSI W 5 /CSI 2 rounc	10.12.2017	CSI-W5*	4	1.60	490	120	11	28.04.2007	S	HOLST	147	1	76	55	44	1886	48	M	15	12	no	vertical	vertical	straight	no	0	0	79,98	no
La Coruna	WORLD CUP GRAND PRIX – CAIXABANK TROPHY CSI W 5 /CSI 2 rounc	10.12.2017	CSI-W5*	4	1.60	490	120	11	28.04.2007	S	HOLST	147	1	76	55	44	1886	48	M	16	13	no	spread	oxer	straight	no	0	0	79,98	no
La Coruna	WORLD CUP GRAND PRIX – CAIXABANK TROPHY CSI W 5 /CSI 2 rounc	10.12.2017	CSI-W5*	4	1.60	490	120	11	28.04.2007	S	HOLST	147	1	76	55	44	1886	48	M	17	14	no	vertical	vertical with water	left	no	0	0	79,98	no
La Coruna	WORLD CUP GRAND PRIX – CAIXABANK TROPHY CSI W 5 /CSI 2 rounc	10.12.2017	CSI-W5*	4	1.60	285	120	11	28.04.2007	S	HOLST	147	1	76	55	44	1886	48	M	1	1	no	vertical	vertical	left	no	0	0	42,33	no
La Coruna	WORLD CUP GRAND PRIX – CAIXABANK TROPHY CSI W 5 /CSI 2 rounc	10.12.2017	CSI-W5*	4	1.60	285	120	11	28.04.2007	S	HOLST	147	1	76	55	44	1886	48	M	2	2	no	spread	oxer	right	no	0	0	42,33	no
La Coruna	WORLD CUP GRAND PRIX – CAIXABANK TROPHY CSI W 5 /CSI 2 rounc	10.12.2017	CSI-W5*	4	1.60	285	120	11	28.04.2007	S	HOLST	147	1	76	55	44	1886	48	M	3	3	no	spread	oxer with water	left	no	0	0	42,33	no
La Coruna	WORLD CUP GRAND PRIX – CAIXABANK TROPHY CSI W 5 /CSI 2 rounc	10.12.2017	CSI-W5*	4	1.60	285	120	11	28.04.2007	S	HOLST	147	1	76	55	44	1886	48	M	4	4	no	vertical	vertical	right	no	0	0	42,33	no
La Coruna	WORLD CUP GRAND PRIX – CAIXABANK TROPHY CSI W 5 /CSI 2 rounc	10.12.2017	CSI-W5*	4	1.60	285	120	11	28.04.2007	S	HOLST	147	1	76	55	44	1886	48	M	5	5	no	spread	oxer	right	no	0	0	42,33	no
La Coruna	WORLD CUP GRAND PRIX – CAIXABANK TROPHY CSI W 5 /CSI 2 rounc	10.12.2017	CSI-W5*	4	1.60	285	120	11	28.04.2007	S	HOLST	147	1	76	55	44	1886	48	M	6	6a	yes	vertical	vertical	right	no	0	0	42,33	no
La Coruna	WORLD CUP GRAND PRIX – CAIXABANK TROPHY CSI W 5 /CSI 2 rounc	10.12.2017	CSI-W5*	4	1.60	285	120	11	28.04.2007	S	HOLST	147	1	76	55	44	1886	48	M	7	6b	yes	spread	oxer	right	no	0	0	42,33	no
La Coruna	WORLD CUP GRAND PRIX – CAIXABANK TROPHY CSI W 5 /CSI 2 rounc	10.12.2017	CSI-W5*	4	1.60	285	120	11	28.04.2007	S	HOLST	147	1	76	55	44	1886	48	M	8	7	no	vertical	vertical	left	no	0	0	42,33	no
La Coruna	WORLD CUP GRAND PRIX – CAIXABANK TROPHY CSI W 5 /CSI 2 rounc	10.12.2017	CSI-W5*	4	1.60	490	26	17	15.05.2001	G	HOLST	292	18	77	27	33	1671	77	M	1	1	no	spread	oxer	left	no	0	0	80,67	no
La Coruna	WORLD CUP GRAND PRIX – CAIXABANK TROPHY CSI W 5 /CSI 2 rounc	10.12.2017	CSI-W5*	4	1.60	490	26	17	15.05.2001	G	HOLST	292	18	77	27	33	1671	77	M	2	2	no	vertical	vertical	left	no	0	0	80,67	no
La Coruna	WORLD CUP GRAND PRIX – CAIXABANK TROPHY CSI W 5 /CSI 2 rounc	10.12.2017	CSI-W5*	4	1.60	490	26	17	15.05.2001	G	HOLST	292	18	77	27	33	1671	77	M	3	3	no	spread	oxer	straight	no	0	0	80,67	no
La Coruna	WORLD CUP GRAND PRIX – CAIXABANK TROPHY CSI W 5 /CSI 2 rounc	10.12.2017	CSI-W5*	4	1.60	490	26	17	15.05.2001	G	HOLST	292	18	77	27	33	1671	77	M	4	4	no	spread	oxer	right	no	0	0	80,67	no
La Coruna	WORLD CUP GRAND PRIX – CAIXABANK TROPHY CSI W 5 /CSI 2 rounc	10.12.2017	CSI-W5*	4	1.60	490	26	17	15.05.2001	G	HOLST	292	18	77	27	33	1671	77	M	5	5a	yes	vertical	vertical	right	no	0	0	80,67	no
La Coruna	WORLD CUP GRAND PRIX – CAIXABANK TROPHY CSI W 5 /CSI 2 rounc	10.12.2017	CSI-W5*	4	1.60	490	26	17	15.05.2001	G	HOLST	292	18	77	27	33	1671	77	M	6	5b	yes	vertical	vertical	right	no	0	0	80,67	no
La Coruna	WORLD CUP GRAND PRIX – CAIXABANK TROPHY CSI W 5 /CSI 2 rounc	10.12.2017	CSI-W5*	4	1.60	490	26	17	15.05.2001	G	HOLST	292	18	77	27	33	1671	77	M	7	5c	yes	spread	oxer	right	no	0	0	80,67	no
La Coruna	WORLD CUP GRAND PRIX – CAIXABANK TROPHY CSI W 5 /CSI 2 rounc	10.12.2017	CSI-W5*	4	1.60	490	26	17	15.05.2001	G	HOLST	292	18	77	27	33	1671	77	M	8	6	no	vertical	vertical	left	no	0	0	80,67	no
La Coruna	WORLD CUP GRAND PRIX – CAIXABANK TROPHY CSI W 5 /CSI 2 rounc	10.12.2017	CSI-W5*	4	1.60	490	26	17	15.05.2001	G	HOLST	292	18	77	27	33	1671	77	M	9	7	no	spread	oxer	left	no	0	0	80,67	no
La Coruna	WORLD CUP GRAND PRIX – CAIXABANK TROPHY CSI W 5 /CSI 2 rounc	10.12.2017	CSI-W5*	4	1.60	490	26	17	15.05.2001	G	HOLST	292	18	77	27	33	1671	77	M	10	8a	yes	spread	oxer	straight	no	0	0	80,67	no
La Coruna	WORLD CUP GRAND PRIX – CAIXABANK TROPHY CSI W 5 /CSI 2 rounc	10.12.2017	CSI-W5*	4	1.60	490	26	17	15.05.2001	G	HOLST	292	18	77	27	33	1671	77	M	11	8b	yes	vertical	vertical	straight	no	0	0	80,67	no
La Coruna	WORLD CUP GRAND PRIX – CAIXABANK TROPHY CSI W 5 /CSI 2 rounc	10.12.2017	CSI-W5*	4	1.60	490	26	17	15.05.2001	G	HOLST	292	18	77	27	33	1671	77	M	12	9	no	spread	oxer with water	left	no	0	0	80,67	no
La Coruna	WORLD CUP GRAND PRIX – CAIXABANK TROPHY CSI W 5 /CSI 2 rounc	10.12.2017	CSI-W5*	4	1.60	490	26	17	15.05.2001	G	HOLST	292	18	77	27	33	1671	77	M	13	10	no	vertical	vertical	right	no	0	0	80,67	no
La Coruna	WORLD CUP GRAND PRIX – CAIXABANK TROPHY CSI W 5 /CSI 2 rounc	10.12.2017	CSI-W5*	4	1.60	490	26	17	15.05.2001	G	HOLST	292	18	77	27	33	1671	77	M	14	11	no	spread	oxer	right	no	0	0	80,67	no
La Coruna	WORLD CUP GRAND PRIX – CAIXABANK TROPHY CSI W 5 /CSI 2 rounc	10.12.2017	CSI-W5*	4	1.60	490	26	17	15.05.2001	G	HOLST	292	18	77	27	33	1671	77	M	15	12	no	vertical	vertical	straight	yes	4	0	80,67	no
La Coruna	WORLD CUP GRAND PRIX – CAIXABANK TROPHY CSI W 5 /CSI 2 rounc	10.12.2017	CSI-W5*	4	1.60	490	26	17	15.05.2001	G	HOLST	292	18	77	27	33	1671	77	M	16	13	no	spread	oxer	straight	no	0	0	80,67	no
La Coruna	WORLD CUP GRAND PRIX – CAIXABANK TROPHY CSI W 5 /CSI 2 rounc	10.12.2017	CSI-W5*	4	1.60	490	26	17	15.05.2001	G	HOLST	292	18	77	27	33	1671	77	M	17	14	no	vertical	vertical with water	left	no	0	0	80,67	no
La Coruna	WORLD CUP GRAND PRIX – CAIXABANK TROPHY CSI W 5 /CSI 2 rounc	10.12.2017	CSI-W5*	4	1.60	490	181	12	20.03.2006	G	ZANG	214	6	81	76	33	1923	71	M	1	1	no	spread	oxer	left	no	0	0	77,9	no
La Coruna	WORLD CUP GRAND PRIX – CAIXABANK TROPHY CSI W 5 /CSI 2 rounc	10.12.2017	CSI-W5*	4	1.60	490	181	12	20.03.2006	G	ZANG	214	6	81	76	33	1923	71	M	2	2	no	vertical	vertical	left	no	0	0	77,9	no
La Coruna	WORLD CUP GRAND PRIX – CAIXABANK TROPHY CSI W 5 /CSI 2 rounc	10.12.2017	CSI-W5*	4	1.60	490	181	12	20.03.2006	G	ZANG	214	6	81	76	33	1923	71	M	3	3	no	spread	oxer	straight	no	0	0	77,9	no
La Coruna	WORLD CUP GRAND PRIX – CAIXABANK TROPHY CSI W 5 /CSI 2 rounc	10.12.2017	CSI-W5*	4	1.60	490	181	12	20.03.2006	G	ZANG	214	6	81	76	33	1923	71	M	4	4	no	spread	oxer	right	no	0	0	77,9	no
La Coruna	WORLD CUP GRAND PRIX – CAIXABANK TROPHY CSI W 5 /CSI 2 rounc	10.12.2017	CSI-W5*	4	1.60	490	181	12	20.03.2006	G	ZANG	214	6	81	76	33	1923	71	M	5	5a	yes	vertical	vertical	right	no	0	0	77,9	no
La Coruna	WORLD CUP GRAND PRIX – CAIXABANK TROPHY CSI W 5 /CSI 2 rounc	10.12.2017	CSI-W5*	4	1.60	490	181	12	20.03.2006	G	ZANG	214	6	81	76	33	1923	71	M	6	5b	yes	vertical	vertical	right	no	0	0	77,9	no
La Coruna	WORLD CUP GRAND PRIX – CAIXABANK TROPHY CSI W 5 /CSI 2 rounc	10.12.2017	CSI-W5*	4	1.60	490	181	12	20.03.2006	G	ZANG	214	6	81	76	33	1923	71	M	7	5c	yes	spread	oxer	right	yes	4	0	77,9	no
La Coruna	WORLD CUP GRAND PRIX – CAIXABANK TROPHY CSI W 5 /CSI 2 rounc	10.12.2017	CSI-W5*	4	1.60	490	181	12	20.03.2006	G	ZANG	214	6	81	76	33	1923	71	M	8	6	no	vertical	vertical	left	no	0	0	77,9	no
La Coruna	WORLD CUP GRAND PRIX – CAIXABANK TROPHY CSI W 5 /CSI 2 rounc	10.12.2017	CSI-W5*	4	1.60	490	181	12	20.03.2006	G	ZANG	214	6	81	76	33	1923	71	M	9	7	no	spread	oxer	left	no	0	0	77,9	no
La Coruna	WORLD CUP GRAND PRIX – CAIXABANK TROPHY CSI W 5 /CSI 2 rounc	10.12.2017	CSI-W5*	4	1.60	490	181	12	20.03.2006	G	ZANG	214	6	81	76	33	1923	71	M	10	8a	yes	spread	oxer	straight	no	0	0	77,9	no
La Coruna	WORLD CUP GRAND PRIX – CAIXABANK TROPHY CSI W 5 /CSI 2 rounc	10.12.2017	CSI-W5*	4	1.60	490	181	12	20.03.2006	G	ZANG	214	6	81	76	33	1923	71	M	11	8b	yes	vertical	vertical	straight	no	0	0	77,9	no
La Coruna	WORLD CUP GRAND PRIX – CAIXABANK TROPHY CSI W 5 /CSI 2 rounc	10.12.2017	CSI-W5*	4	1.60	490	181	12	20.03.2006	G	ZANG	214	6	81	76	33	1923	71	M	12	9	no	spread	oxer with water	left	no	0	0	77,9	no
La Coruna	WORLD CUP GRAND PRIX – CAIXABANK TROPHY CSI W 5 /CSI 2 rounc	10.12.2017	CSI-W5*	4	1.60	490	181	12	20.03.2006	G	ZANG	214	6	81	76	33	1923	71	M	13	10	no	vertical	vertical	right	no	0	0	77,9	no
La Coruna	WORLD CUP GRAND PRIX – CAIXABANK TROPHY CSI W 5 /CSI 2 rounc	10.12.2017	CSI-W5*	4	1.60	490	181	12	20.03.2006	G	ZANG	214	6	81	76	33	1923	71	M	14	11	no	spread	oxer	right	no	0	0	77,9	no
La Coruna	WORLD CUP GRAND PRIX – CAIXABANK TROPHY CSI W 5 /CSI 2 rounc	10.12.2017	CSI-W5*	4	1.60	490	181	12	20.03.2006	G	ZANG	214	6	81	76	33	1923	71	M	15	12	no	vertical	vertical	straight	no	0	0	77,9	no
La Coruna	WORLD CUP GRAND PRIX – CAIXABANK TROPHY CSI W 5 /CSI 2 rounc	10.12.2017	CSI-W5*	4	1.60	490	181	12	20.03.2006	G	ZANG	214	6	81	76	33	1923	71	M	16	13	no	spread	oxer	straight	no	0	0	77,9	no
La Coruna	WORLD CUP GRAND PRIX – CAIXABANK TROPHY CSI W 5 /CSI 2 rounc	10.12.2017	CSI-W5*	4	1.60	490	181	12	20.03.2006	G	ZANG	214	6	81	76	33	1923	71	M	17	14	no	vertical	vertical with water	left	no	0	0	77,9	no
La Coruna	WORLD CUP GRAND PRIX – CAIXABANK TROPHY CSI W 5 /CSI 2 rounc	10.12.2017	CSI-W5*	4	1.60	490	187	12	04.06.2006	G	CH	180	7	84	158	36	1809	142	M	1	1	no	spread	oxer	left	no	0	0	77,08	no
La Coruna	WORLD CUP GRAND PRIX – CAIXABANK TROPHY CSI W 5 /CSI 2 rounc	10.12.2017	CSI-W5*	4	1.60	490	187	12	04.06.2006	G	CH	180	7	84	158	36	1809	142	M	2	2	no	vertical	vertical	left	no	0	0	77,08	no
La Coruna	WORLD CUP GRAND PRIX – CAIXABANK TROPHY CSI W 5 /CSI 2 rounc	10.12.2017	CSI-W5*	4	1.60	490	187	12	04.06.2006	G	CH	180	7	84	158	36	1809	142	M	3	3	no	spread	oxer	straight	no	0	0	77,08	no
La Coruna	WORLD CUP GRAND PRIX – CAIXABANK TROPHY CSI W 5 /CSI 2 rounc	10.12.2017	CSI-W5*	4	1.60	490	187	12	04.06.2006	G	CH	180	7	84	158	36	1809	142	M	4	4	no	spread	oxer	right	no	0	0	77,08	no
La Coruna	WORLD CUP GRAND PRIX – CAIXABANK TROPHY CSI W 5 /CSI 2 rounc	10.12.2017	CSI-W5*	4	1.60	490	187	12	04.06.2006	G	CH	180	7	84	158	36	1809	142	M	5	5a	yes	vertical	vertical	right	no	0	0	77,08	no
La Coruna	WORLD CUP GRAND PRIX – CAIXABANK TROPHY CSI W 5 /CSI 2 rounc	10.12.2017	CSI-W5*	4	1.60	490	187	12	04.06.2006	G	CH	180	7	84	158	36	1809	142	M	6	5b	yes	vertical	vertical	right	yes	4	0	77,08	no
La Coruna	WORLD CUP GRAND PRIX – CAIXABANK TROPHY CSI W 5 /CSI 2 rounc	10.12.2017	CSI-W5*	4	1.60	490	187	12	04.06.2006	G	CH	180	7	84	158	36	1809	142	M	7	5c	yes	spread	oxer	right	no	0	0	77,08	no
La Coruna	WORLD CUP GRAND PRIX – CAIXABANK TROPHY CSI W 5 /CSI 2 rounc	10.12.2017	CSI-W5*	4	1.60	490	187	12	04.06.2006	G	CH	180	7	84	158	36	1809	142	M	8	6	no	vertical	vertical	left	no	0	0	77,08	no
La Coruna	WORLD CUP GRAND PRIX – CAIXABANK TROPHY CSI W 5 /CSI 2 rounc	10.12.2017	CSI-W5*	4	1.60	490	187	12	04.06.2006	G	CH	180	7	84	158	36	1809	142	M	9	7	no	spread	oxer	left	no	0	0	77,08	no
La Coruna	WORLD CUP GRAND PRIX – CAIXABANK TROPHY CSI W 5 /CSI 2 rounc	10.12.2017	CSI-W5*	4	1.60	490	187	12	04.06.2006	G	CH	180	7	84	158	36	1809	142	M	10	8a	yes	spread	oxer	straight	no	0	0	77,08	no
La Coruna	WORLD CUP GRAND PRIX – CAIXABANK TROPHY CSI W 5 /CSI 2 rounc	10.12.2017	CSI-W5*	4	1.60	490	187	12	04.06.2006	G	CH	180	7	84	158	36	1809	142	M	11	8b	yes	vertical	vertical	straight	no	0	0	77,08	no
La Coruna	WORLD CUP GRAND PRIX – CAIXABANK TROPHY CSI W 5 /CSI 2 rounc	10.12.2017	CSI-W5*	4	1.60	490	187	12	04.06.2006	G	CH	180	7	84	158	36	1809	142	M	12	9	no	spread	oxer with water	left	no	0	0	77,08	no
La Coruna	WORLD CUP GRAND PRIX – CAIXABANK TROPHY CSI W 5 /CSI 2 rounc	10.12.2017	CSI-W5*	4	1.60	490	187	12	04.06.2006	G	CH	180	7	84	158	36	1809	142	M	13	10	no	vertical	vertical	right	no	0	0	77,08	no
La Coruna	WORLD CUP GRAND PRIX – CAIXABANK TROPHY CSI W 5 /CSI 2 rounc	10.12.2017	CSI-W5*	4	1.60	490	187	12	04.06.2006	G	CH	180	7	84	158	36	1809	142	M	14	11	no	spread	oxer	right	no	0	0	77,08	no
La Coruna	WORLD CUP GRAND PRIX – CAIXABANK TROPHY CSI W 5 /CSI 2 rounc	10.12.2017	CSI-W5*	4	1.60	490	187	12	04.06.2006	G	CH	180	7	84	158	36	1809	142	M	15	12	no	vertical	vertical	straight	no	0	0	77,08	no
La Coruna	WORLD CUP GRAND PRIX – CAIXABANK TROPHY CSI W 5 /CSI 2 rounc	10.12.2017	CSI-W5*	4	1.60	490	187	12	04.06.2006	G	CH	180	7	84	158	36	1809	142	M	16	13	no	spread	oxer	straight	no	0	0	77,08	no
La Coruna	WORLD CUP GRAND PRIX – CAIXABANK TROPHY CSI W 5 /CSI 2 rounc	10.12.2017	CSI-W5*	4	1.60	490	187	12	04.06.2006	G	CH	180	7	84	158	36	1809	142	M	17	14	no	vertical	vertical with water	left	no	0	0	77,08	no
La Coruna	WORLD CUP GRAND PRIX – CAIXABANK TROPHY CSI W 5 /CSI 2 rounc	10.12.2017	CSI-W5*	4	1.60	490	131	10	28.03.2008	G	BWP	151	17	85	41	41	2052	191	M	1	1	no	spread	oxer	left	no	0	0	77,82	no
La Coruna	WORLD CUP GRAND PRIX – CAIXABANK TROPHY CSI W 5 /CSI 2 rounc	10.12.2017	CSI-W5*	4	1.60	490	131	10	28.03.2008	G	BWP	151	17	85	41	41	2052	191	M	2	2	no	vertical	vertical	left	no	0	0	77,82	no
La Coruna	WORLD CUP GRAND PRIX – CAIXABANK TROPHY CSI W 5 /CSI 2 rounc	10.12.2017	CSI-W5*	4	1.60	490	131	10	28.03.2008	G	BWP	151	17	85	41	41	2052	191	M	3	3	no	spread	oxer	straight	no	0	0	77,82	no
La Coruna	WORLD CUP GRAND PRIX – CAIXABANK TROPHY CSI W 5 /CSI 2 rounc	10.12.2017	CSI-W5*	4	1.60	490	131	10	28.03.2008	G	BWP	151	17	85	41	41	2052	191	M	4	4	no	spread	oxer	right	no	0	0	77,82	no
La Coruna	WORLD CUP GRAND PRIX – CAIXABANK TROPHY CSI W 5 /CSI 2 rounc	10.12.2017	CSI-W5*	4	1.60	490	131	10	28.03.2008	G	BWP	151	17	85	41	41	2052	191	M	5	5a	yes	vertical	vertical	right	no	0	0	77,82	no
La Coruna	WORLD CUP GRAND PRIX – CAIXABANK TROPHY CSI W 5 /CSI 2 rounc	10.12.2017	CSI-W5*	4	1.60	490	131	10	28.03.2008	G	BWP	151	17	85	41	41	2052	191	M	6	5b	yes	vertical	vertical	right	yes	4	0	77,82	no
La Coruna	WORLD CUP GRAND PRIX – CAIXABANK TROPHY CSI W 5 /CSI 2 rounc	10.12.2017	CSI-W5*	4	1.60	490	131	10	28.03.2008	G	BWP	151	17	85	41	41	2052	191	M	7	5c	yes	spread	oxer	right	yes	4	0	77,82	no
La Coruna	WORLD CUP GRAND PRIX – CAIXABANK TROPHY CSI W 5 /CSI 2 rounc	10.12.2017	CSI-W5*	4	1.60	490	131	10	28.03.2008	G	BWP	151	17	85	41	41	2052	191	M	8	6	no	vertical	vertical	left	no	0	0	77,82	no
La Coruna	WORLD CUP GRAND PRIX – CAIXABANK TROPHY CSI W 5 /CSI 2 rounc	10.12.2017	CSI-W5*	4	1.60	490	131	10	28.03.2008	G	BWP	151	17	85	41	41	2052	191	M	9	7	no	spread	oxer	left	no	0	0	77,82	no
La Coruna	WORLD CUP GRAND PRIX – CAIXABANK TROPHY CSI W 5 /CSI 2 rounc	10.12.2017	CSI-W5*	4	1.60	490	131	10	28.03.2008	G	BWP	151	17	85	41	41	2052	191	M	10	8a	yes	spread	oxer	straight	no	0	0	77,82	no
La Coruna	WORLD CUP GRAND PRIX – CAIXABANK TROPHY CSI W 5 /CSI 2 rounc	10.12.2017	CSI-W5*	4	1.60	490	131	10	28.03.2008	G	BWP	151	17	85	41	41	2052	191	M	11	8b	yes	vertical	vertical	straight	no	0	0	77,82	no
La Coruna	WORLD CUP GRAND PRIX – CAIXABANK TROPHY CSI W 5 /CSI 2 rounc	10.12.2017	CSI-W5*	4	1.60	490	131	10	28.03.2008	G	BWP	151	17	85	41	41	2052	191	M	12	9	no	spread	oxer with water	left	no	0	0	77,82	no
La Coruna	WORLD CUP GRAND PRIX – CAIXABANK TROPHY CSI W 5 /CSI 2 rounc	10.12.2017	CSI-W5*	4	1.60	490	131	10	28.03.2008	G	BWP	151	17	85	41	41	2052	191	M	13	10	no	vertical	vertical	right	no	0	0	77,82	no
La Coruna	WORLD CUP GRAND PRIX – CAIXABANK TROPHY CSI W 5 /CSI 2 rounc	10.12.2017	CSI-W5*	4	1.60	490	131	10	28.03.2008	G	BWP	151	17	85	41	41	2052	191	M	14	11	no	spread	oxer	right	no	0	0	77,82	no
La Coruna	WORLD CUP GRAND PRIX – CAIXABANK TROPHY CSI W 5 /CSI 2 rounc	10.12.2017	CSI-W5*	4	1.60	490	131	10	28.03.2008	G	BWP	151	17	85	41	41	2052	191	M	15	12	no	vertical	vertical	straight	no	0	0	77,82	no
La Coruna	WORLD CUP GRAND PRIX – CAIXABANK TROPHY CSI W 5 /CSI 2 rounc	10.12.2017	CSI-W5*	4	1.60	490	131	10	28.03.2008	G	BWP	151	17	85	41	41	2052	191	M	16	13	no	spread	oxer	straight	no	0	0	77,82	no
La Coruna	WORLD CUP GRAND PRIX – CAIXABANK TROPHY CSI W 5 /CSI 2 rounc	10.12.2017	CSI-W5*	4	1.60	490	131	10	28.03.2008	G	BWP	151	17	85	41	41	2052	191	M	17	14	no	vertical	vertical with water	left	no	0	0	77,82	no
La Coruna	WORLD CUP GRAND PRIX – CAIXABANK TROPHY CSI W 5 /CSI 2 rounc	10.12.2017	CSI-W5*	4	1.60	490	103	10	06.03.2008	S	HOLST	118	5	92	38	25	1918	72	M	1	1	no	spread	oxer	left	no	0	0	0,00	yes
La Coruna	WORLD CUP GRAND PRIX – CAIXABANK TROPHY CSI W 5 /CSI 2 rounc	10.12.2017	CSI-W5*	4	1.60	490	103	10	06.03.2008	S	HOLST	118	5	92	38	25	1918	72	M	2	2	no	vertical	vertical	left	no	0	0	0,00	yes
La Coruna	WORLD CUP GRAND PRIX – CAIXABANK TROPHY CSI W 5 /CSI 2 rounc	10.12.2017	CSI-W5*	4	1.60	490	103	10	06.03.2008	S	HOLST	118	5	92	38	25	1918	72	M	3	3	no	spread	oxer	straight	no	0	0	0,00	yes
La Coruna	WORLD CUP GRAND PRIX – CAIXABANK TROPHY CSI W 5 /CSI 2 rounc	10.12.2017	CSI-W5*	4	1.60	490	103	10	06.03.2008	S	HOLST	118	5	92	38	25	1918	72	M	4	4	no	spread	oxer	right	no	0	0	0,00	yes
La Coruna	WORLD CUP GRAND PRIX – CAIXABANK TROPHY CSI W 5 /CSI 2 rounc	10.12.2017	CSI-W5*	4	1.60	490	103	10	06.03.2008	S	HOLST	118	5	92	38	25	1918	72	M	5	5a	yes	vertical	vertical	right	no	0	0	0,00	yes
La Coruna	WORLD CUP GRAND PRIX – CAIXABANK TROPHY CSI W 5 /CSI 2 rounc	10.12.2017	CSI-W5*	4	1.60	490	103	10	06.03.2008	S	HOLST	118	5	92	38	25	1918	72	M	6	5b	yes	vertical	vertical	right	no	0	0	0,00	yes
La Coruna	WORLD CUP GRAND PRIX – CAIXABANK TROPHY CSI W 5 /CSI 2 rounc	10.12.2017	CSI-W5*	4	1.60	490	103	10	06.03.2008	S	HOLST	118	5	92	38	25	1918	72	M	7	5c	yes	spread	oxer	right	yes	4	0	0,00	yes
La Coruna	WORLD CUP GRAND PRIX – CAIXABANK TROPHY CSI W 5 /CSI 2 rounc	10.12.2017	CSI-W5*	4	1.60	490	103	10	06.03.2008	S	HOLST	118	5	92	38	25	1918	72	M	8	6	no	vertical	vertical	left	no	0	0	0,00	yes
La Coruna	WORLD CUP GRAND PRIX – CAIXABANK TROPHY CSI W 5 /CSI 2 rounc	10.12.2017	CSI-W5*	4	1.60	490	103	10	06.03.2008	S	HOLST	118	5	92	38	25	1918	72	M	9	7	no	spread	oxer	left	no	0	0	0,00	yes
La Coruna	WORLD CUP GRAND PRIX – CAIXABANK TROPHY CSI W 5 /CSI 2 rounc	10.12.2017	CSI-W5*	4	1.60	490	103	10	06.03.2008	S	HOLST	118	5	92	38	25	1918	72	M	10	8a	yes	spread	oxer	straight	no	0	0	0,00	yes
La Coruna	WORLD CUP GRAND PRIX – CAIXABANK TROPHY CSI W 5 /CSI 2 rounc	10.12.2017	CSI-W5*	4	1.60	490	103	10	06.03.2008	S	HOLST	118	5	92	38	25	1918	72	M	11	8b	yes	vertical	vertical	straight	no	0	0	0,00	yes
La Coruna	WORLD CUP GRAND PRIX – CAIXABANK TROPHY CSI W 5 /CSI 2 rounc	10.12.2017	CSI-W5*	4	1.60	490	103	10	06.03.2008	S	HOLST	118	5	92	38	25	1918	72	M	12	9	no	spread	oxer with water	left	yes	4	0	0,00	yes
La Coruna	WORLD CUP GRAND PRIX – CAIXABANK TROPHY CSI W 5 /CSI 2 rounc	10.12.2017	CSI-W5*	4	1.60	490	103	10	06.03.2008	S	HOLST	118	5	92	38	25	1918	72	M	13	10	no	vertical	vertical	right	no	0	0	0,00	yes
La Coruna	WORLD CUP GRAND PRIX – CAIXABANK TROPHY CSI W 5 /CSI 2 rounc	10.12.2017	CSI-W5*	4	1.60	490	103	10	06.03.2008	S	HOLST	118	5	92	38	25	1918	72	M	14	11	no	spread	oxer	right	no	0	0	0,00	yes
La Coruna	WORLD CUP GRAND PRIX – CAIXABANK TROPHY CSI W 5 /CSI 2 rounc	10.12.2017	CSI-W5*	4	1.60	490	103	10	06.03.2008	S	HOLST	118	5	92	38	25	1918	72	M	15	12	no	vertical	vertical	straight	no	0	0	0,00	yes
La Coruna	WORLD CUP GRAND PRIX – CAIXABANK TROPHY CSI W 5 /CSI 2 rounc	10.12.2017	CSI-W5*	4	1.60	490	103	10	06.03.2008	S	HOLST	118	5	92	38	25	1918	72	M	16	13	no	spread	oxer	straight	no	0	0	0,00	yes
La Coruna	WORLD CUP GRAND PRIX – CAIXABANK TROPHY CSI W 5 /CSI 2 rounc	10.12.2017	CSI-W5*	4	1.60	490	103	10	06.03.2008	S	HOLST	118	5	92	38	25	1918	72	M	17	14	no	vertical	vertical with water	left	no	0	0	0,00	yes
La Coruna	WORLD CUP GRAND PRIX – CAIXABANK TROPHY CSI W 5 /CSI 2 rounc	10.12.2017	CSI-W5*	4	1.60	490	118	12	26.01.2006	G	OS	262	11	96	97	55	1508	53	M	1	1	no	spread	oxer	left	no	0	0	77,66	no
La Coruna	WORLD CUP GRAND PRIX – CAIXABANK TROPHY CSI W 5 /CSI 2 rounc	10.12.2017	CSI-W5*	4	1.60	490	118	12	26.01.2006	G	OS	262	11	96	97	55	1508	53	M	2	2	no	vertical	vertical	left	no	0	0	77,66	no
La Coruna	WORLD CUP GRAND PRIX – CAIXABANK TROPHY CSI W 5 /CSI 2 rounc	10.12.2017	CSI-W5*	4	1.60	490	118	12	26.01.2006	G	OS	262	11	96	97	55	1508	53	M	3	3	no	spread	oxer	straight	no	0	0	77,66	no
La Coruna	WORLD CUP GRAND PRIX – CAIXABANK TROPHY CSI W 5 /CSI 2 rounc	10.12.2017	CSI-W5*	4	1.60	490	118	12	26.01.2006	G	OS	262	11	96	97	55	1508	53	M	4	4	no	spread	oxer	right	no	0	0	77,66	no
La Coruna	WORLD CUP GRAND PRIX – CAIXABANK TROPHY CSI W 5 /CSI 2 rounc	10.12.2017	CSI-W5*	4	1.60	490	118	12	26.01.2006	G	OS	262	11	96	97	55	1508	53	M	5	5a	yes	vertical	vertical	right	no	0	0	77,66	no
La Coruna	WORLD CUP GRAND PRIX – CAIXABANK TROPHY CSI W 5 /CSI 2 rounc	10.12.2017	CSI-W5*	4	1.60	490	118	12	26.01.2006	G	OS	262	11	96	97	55	1508	53	M	6	5b	yes	vertical	vertical	right	no	0	0	77,66	no
La Coruna	WORLD CUP GRAND PRIX – CAIXABANK TROPHY CSI W 5 /CSI 2 rounc	10.12.2017	CSI-W5*	4	1.60	490	118	12	26.01.2006	G	OS	262	11	96	97	55	1508	53	M	7	5c	yes	spread	oxer	right	no	0	0	77,66	no
La Coruna	WORLD CUP GRAND PRIX – CAIXABANK TROPHY CSI W 5 /CSI 2 rounc	10.12.2017	CSI-W5*	4	1.60	490	118	12	26.01.2006	G	OS	262	11	96	97	55	1508	53	M	8	6	no	vertical	vertical	left	no	0	0	77,66	no
La Coruna	WORLD CUP GRAND PRIX – CAIXABANK TROPHY CSI W 5 /CSI 2 rounc	10.12.2017	CSI-W5*	4	1.60	490	118	12	26.01.2006	G	OS	262	11	96	97	55	1508	53	M	9	7	no	spread	oxer	left	no	0	0	77,66	no
La Coruna	WORLD CUP GRAND PRIX – CAIXABANK TROPHY CSI W 5 /CSI 2 rounc	10.12.2017	CSI-W5*	4	1.60	490	118	12	26.01.2006	G	OS	262	11	96	97	55	1508	53	M	10	8a	yes	spread	oxer	straight	yes	4	0	77,66	no
La Coruna	WORLD CUP GRAND PRIX – CAIXABANK TROPHY CSI W 5 /CSI 2 rounc	10.12.2017	CSI-W5*	4	1.60	490	118	12	26.01.2006	G	OS	262	11	96	97	55	1508	53	M	11	8b	yes	vertical	vertical	straight	no	0	0	77,66	no
La Coruna	WORLD CUP GRAND PRIX – CAIXABANK TROPHY CSI W 5 /CSI 2 rounc	10.12.2017	CSI-W5*	4	1.60	490	118	12	26.01.2006	G	OS	262	11	96	97	55	1508	53	M	12	9	no	spread	oxer with water	left	no	0	0	77,66	no
La Coruna	WORLD CUP GRAND PRIX – CAIXABANK TROPHY CSI W 5 /CSI 2 rounc	10.12.2017	CSI-W5*	4	1.60	490	118	12	26.01.2006	G	OS	262	11	96	97	55	1508	53	M	13	10	no	vertical	vertical	right	no	0	0	77,66	no
La Coruna	WORLD CUP GRAND PRIX – CAIXABANK TROPHY CSI W 5 /CSI 2 rounc	10.12.2017	CSI-W5*	4	1.60	490	118	12	26.01.2006	G	OS	262	11	96	97	55	1508	53	M	14	11	no	spread	oxer	right	no	0	0	77,66	no
La Coruna	WORLD CUP GRAND PRIX – CAIXABANK TROPHY CSI W 5 /CSI 2 rounc	10.12.2017	CSI-W5*	4	1.60	490	118	12	26.01.2006	G	OS	262	11	96	97	55	1508	53	M	15	12	no	vertical	vertical	straight	yes	4	0	77,66	no
La Coruna	WORLD CUP GRAND PRIX – CAIXABANK TROPHY CSI W 5 /CSI 2 rounc	10.12.2017	CSI-W5*	4	1.60	490	118	12	26.01.2006	G	OS	262	11	96	97	55	1508	53	M	16	13	no	spread	oxer	straight	no	0	0	77,66	no
La Coruna	WORLD CUP GRAND PRIX – CAIXABANK TROPHY CSI W 5 /CSI 2 rounc	10.12.2017	CSI-W5*	4	1.60	490	118	12	26.01.2006	G	OS	262	11	96	97	55	1508	53	M	17	14	no	vertical	vertical with water	left	yes	4	0	77,66	no
La Coruna	WORLD CUP GRAND PRIX – CAIXABANK TROPHY CSI W 5 /CSI 2 rounc	10.12.2017	CSI-W5*	4	1.60	490	114	13	22.05.2005	G	OS	124	3	98	187	43	919	24	M	1	1	no	spread	oxer	left	no	0	0	81,95	no
La Coruna	WORLD CUP GRAND PRIX – CAIXABANK TROPHY CSI W 5 /CSI 2 rounc	10.12.2017	CSI-W5*	4	1.60	490	114	13	22.05.2005	G	OS	124	3	98	187	43	919	24	M	2	2	no	vertical	vertical	left	no	0	0	81,95	no
La Coruna	WORLD CUP GRAND PRIX – CAIXABANK TROPHY CSI W 5 /CSI 2 rounc	10.12.2017	CSI-W5*	4	1.60	490	114	13	22.05.2005	G	OS	124	3	98	187	43	919	24	M	3	3	no	spread	oxer	straight	no	0	0	81,95	no
La Coruna	WORLD CUP GRAND PRIX – CAIXABANK TROPHY CSI W 5 /CSI 2 rounc	10.12.2017	CSI-W5*	4	1.60	490	114	13	22.05.2005	G	OS	124	3	98	187	43	919	24	M	4	4	no	spread	oxer	right	no	0	0	81,95	no
La Coruna	WORLD CUP GRAND PRIX – CAIXABANK TROPHY CSI W 5 /CSI 2 rounc	10.12.2017	CSI-W5*	4	1.60	490	114	13	22.05.2005	G	OS	124	3	98	187	43	919	24	M	5	5a	yes	vertical	vertical	right	no	0	0	81,95	no
La Coruna	WORLD CUP GRAND PRIX – CAIXABANK TROPHY CSI W 5 /CSI 2 rounc	10.12.2017	CSI-W5*	4	1.60	490	114	13	22.05.2005	G	OS	124	3	98	187	43	919	24	M	6	5b	yes	vertical	vertical	right	no	0	0	81,95	no
La Coruna	WORLD CUP GRAND PRIX – CAIXABANK TROPHY CSI W 5 /CSI 2 rounc	10.12.2017	CSI-W5*	4	1.60	490	114	13	22.05.2005	G	OS	124	3	98	187	43	919	24	M	7	5c	yes	spread	oxer	right	no	0	0	81,95	no
La Coruna	WORLD CUP GRAND PRIX – CAIXABANK TROPHY CSI W 5 /CSI 2 rounc	10.12.2017	CSI-W5*	4	1.60	490	114	13	22.05.2005	G	OS	124	3	98	187	43	919	24	M	8	6	no	vertical	vertical	left	no	0	0	81,95	no
La Coruna	WORLD CUP GRAND PRIX – CAIXABANK TROPHY CSI W 5 /CSI 2 rounc	10.12.2017	CSI-W5*	4	1.60	490	114	13	22.05.2005	G	OS	124	3	98	187	43	919	24	M	9	7	no	spread	oxer	left	no	0	0	81,95	no
La Coruna	WORLD CUP GRAND PRIX – CAIXABANK TROPHY CSI W 5 /CSI 2 rounc	10.12.2017	CSI-W5*	4	1.60	490	114	13	22.05.2005	G	OS	124	3	98	187	43	919	24	M	10	8a	yes	spread	oxer	straight	no	0	0	81,95	no
La Coruna	WORLD CUP GRAND PRIX – CAIXABANK TROPHY CSI W 5 /CSI 2 rounc	10.12.2017	CSI-W5*	4	1.60	490	114	13	22.05.2005	G	OS	124	3	98	187	43	919	24	M	11	8b	yes	vertical	vertical	straight	no	0	0	81,95	no
La Coruna	WORLD CUP GRAND PRIX – CAIXABANK TROPHY CSI W 5 /CSI 2 rounc	10.12.2017	CSI-W5*	4	1.60	490	114	13	22.05.2005	G	OS	124	3	98	187	43	919	24	M	12	9	no	spread	oxer with water	left	no	0	0	81,95	no

La Coruna	WORLD CUP GRAND PRIX – CAIXABANK TROPHY CSI W 5 /CSI 2 round	10.12.2017	CSI-W5*	4	1.60	490	114	13	22.05.2005	G	OS	124	3	98	187	43	919	24	M	13	10	no	vertical	vertical	right	no	0	0	81,95	no
La Coruna	WORLD CUP GRAND PRIX – CAIXABANK TROPHY CSI W 5 /CSI 2 round	10.12.2017	CSI-W5*	4	1.60	490	114	13	22.05.2005	G	OS	124	3	98	187	43	919	24	M	14	11	no	spread	oxer	right	no	0	0	81,95	no
La Coruna	WORLD CUP GRAND PRIX – CAIXABANK TROPHY CSI W 5 /CSI 2 round	10.12.2017	CSI-W5*	4	1.60	490	114	13	22.05.2005	G	OS	124	3	98	187	43	919	24	M	15	12	no	vertical	vertical	straight	no	0	0	81,95	no
La Coruna	WORLD CUP GRAND PRIX – CAIXABANK TROPHY CSI W 5 /CSI 2 round	10.12.2017	CSI-W5*	4	1.60	490	114	13	22.05.2005	G	OS	124	3	98	187	43	919	24	M	16	13	no	spread	oxer	straight	no	0	0	81,95	no
La Coruna	WORLD CUP GRAND PRIX – CAIXABANK TROPHY CSI W 5 /CSI 2 round	10.12.2017	CSI-W5*	4	1.60	490	114	13	22.05.2005	G	OS	124	3	98	187	43	919	24	M	17	14	no	vertical	vertical with water	left	no	0	0	81,95	no
La Coruna	WORLD CUP GRAND PRIX – CAIXABANK TROPHY CSI W 5 /CSI 2 round	10.12.2017	CSI-W5*	4	1.60	285	114	13	22.05.2005	G	OS	124	3	98	187	43	919	24	M	1	1	no	vertical	vertical	left	no	0	0	44,72	no
La Coruna	WORLD CUP GRAND PRIX – CAIXABANK TROPHY CSI W 5 /CSI 2 round	10.12.2017	CSI-W5*	4	1.60	285	114	13	22.05.2005	G	OS	124	3	98	187	43	919	24	M	2	2	no	spread	oxer	right	no	0	0	44,72	no
La Coruna	WORLD CUP GRAND PRIX – CAIXABANK TROPHY CSI W 5 /CSI 2 round	10.12.2017	CSI-W5*	4	1.60	285	114	13	22.05.2005	G	OS	124	3	98	187	43	919	24	M	3	3	no	spread	oxer with water	left	yes	4	0	44,72	no
La Coruna	WORLD CUP GRAND PRIX – CAIXABANK TROPHY CSI W 5 /CSI 2 round	10.12.2017	CSI-W5*	4	1.60	285	114	13	22.05.2005	G	OS	124	3	98	187	43	919	24	M	4	4	no	vertical	vertical	right	no	0	0	44,72	no
La Coruna	WORLD CUP GRAND PRIX – CAIXABANK TROPHY CSI W 5 /CSI 2 round	10.12.2017	CSI-W5*	4	1.60	285	114	13	22.05.2005	G	OS	124	3	98	187	43	919	24	M	5	5	no	spread	oxer	right	no	0	0	44,72	no
La Coruna	WORLD CUP GRAND PRIX – CAIXABANK TROPHY CSI W 5 /CSI 2 round	10.12.2017	CSI-W5*	4	1.60	285	114	13	22.05.2005	G	OS	124	3	98	187	43	919	24	M	6	6a	yes	vertical	vertical	right	no	0	0	44,72	no
La Coruna	WORLD CUP GRAND PRIX – CAIXABANK TROPHY CSI W 5 /CSI 2 round	10.12.2017	CSI-W5*	4	1.60	285	114	13	22.05.2005	G	OS	124	3	98	187	43	919	24	M	7	6b	yes	spread	oxer	right	no	0	0	44,72	no
La Coruna	WORLD CUP GRAND PRIX – CAIXABANK TROPHY CSI W 5 /CSI 2 round	10.12.2017	CSI-W5*	4	1.60	285	114	13	22.05.2005	G	OS	124	3	98	187	43	919	24	M	8	7	no	vertical	vertical	left	no	0	0	44,72	no
La Coruna	WORLD CUP GRAND PRIX – CAIXABANK TROPHY CSI W 5 /CSI 2 round	10.12.2017	CSI-W5*	4	1.60	490	2	13	17.04.2005	G	KWPN	274	23	101	46	53	686	44	M	1	1	no	spread	oxer	left	no	0	0	80,47	no
La Coruna	WORLD CUP GRAND PRIX – CAIXABANK TROPHY CSI W 5 /CSI 2 round	10.12.2017	CSI-W5*	4	1.60	490	2	13	17.04.2005	G	KWPN	274	23	101	46	53	686	44	M	2	2	no	vertical	vertical	left	no	0	0	80,47	no
La Coruna	WORLD CUP GRAND PRIX – CAIXABANK TROPHY CSI W 5 /CSI 2 round	10.12.2017	CSI-W5*	4	1.60	490	2	13	17.04.2005	G	KWPN	274	23	101	46	53	686	44	M	3	3	no	spread	oxer	straight	no	0	0	80,47	no
La Coruna	WORLD CUP GRAND PRIX – CAIXABANK TROPHY CSI W 5 /CSI 2 round	10.12.2017	CSI-W5*	4	1.60	490	2	13	17.04.2005	G	KWPN	274	23	101	46	53	686	44	M	4	4	no	spread	oxer	right	no	0	0	80,47	no
La Coruna	WORLD CUP GRAND PRIX – CAIXABANK TROPHY CSI W 5 /CSI 2 round	10.12.2017	CSI-W5*	4	1.60	490	2	13	17.04.2005	G	KWPN	274	23	101	46	53	686	44	M	5	5a	yes	vertical	vertical	right	no	0	0	80,47	no
La Coruna	WORLD CUP GRAND PRIX – CAIXABANK TROPHY CSI W 5 /CSI 2 round	10.12.2017	CSI-W5*	4	1.60	490	2	13	17.04.2005	G	KWPN	274	23	101	46	53	686	44	M	6	5b	yes	vertical	vertical	right	no	0	0	80,47	no
La Coruna	WORLD CUP GRAND PRIX – CAIXABANK TROPHY CSI W 5 /CSI 2 round	10.12.2017	CSI-W5*	4	1.60	490	2	13	17.04.2005	G	KWPN	274	23	101	46	53	686	44	M	7	5c	yes	spread	oxer	right	no	0	0	80,47	no
La Coruna	WORLD CUP GRAND PRIX – CAIXABANK TROPHY CSI W 5 /CSI 2 round	10.12.2017	CSI-W5*	4	1.60	490	2	13	17.04.2005	G	KWPN	274	23	101	46	53	686	44	M	8	6	no	vertical	vertical	left	no	0	0	80,47	no
La Coruna	WORLD CUP GRAND PRIX – CAIXABANK TROPHY CSI W 5 /CSI 2 round	10.12.2017	CSI-W5*	4	1.60	490	2	13	17.04.2005	G	KWPN	274	23	101	46	53	686	44	M	9	7	no	spread	oxer	left	no	0	0	80,47	no
La Coruna	WORLD CUP GRAND PRIX – CAIXABANK TROPHY CSI W 5 /CSI 2 round	10.12.2017	CSI-W5*	4	1.60	490	2	13	17.04.2005	G	KWPN	274	23	101	46	53	686	44	M	10	8a	yes	spread	oxer	straight	no	0	0	80,47	no
La Coruna	WORLD CUP GRAND PRIX – CAIXABANK TROPHY CSI W 5 /CSI 2 round	10.12.2017	CSI-W5*	4	1.60	490	2	13	17.04.2005	G	KWPN	274	23	101	46	53	686	44	M	11	8b	yes	vertical	vertical	straight	yes	4	0	80,47	no
La Coruna	WORLD CUP GRAND PRIX – CAIXABANK TROPHY CSI W 5 /CSI 2 round	10.12.2017	CSI-W5*	4	1.60	490	2	13	17.04.2005	G	KWPN	274	23	101	46	53	686	44	M	12	9	no	spread	oxer with water	left	no	0	0	80,47	no
La Coruna	WORLD CUP GRAND PRIX – CAIXABANK TROPHY CSI W 5 /CSI 2 round	10.12.2017	CSI-W5*	4	1.60	490	2	13	17.04.2005	G	KWPN	274	23	101	46	53	686	44	M	13	10	no	vertical	vertical	right	no	0	0	80,47	no
La Coruna	WORLD CUP GRAND PRIX – CAIXABANK TROPHY CSI W 5 /CSI 2 round	10.12.2017	CSI-W5*	4	1.60	490	2	13	17.04.2005	G	KWPN	274	23	101	46	53	686	44	M	14	11	no	spread	oxer	right	no	0	0	80,47	no
La Coruna	WORLD CUP GRAND PRIX – CAIXABANK TROPHY CSI W 5 /CSI 2 round	10.12.2017	CSI-W5*	4	1.60	490	2	13	17.04.2005	G	KWPN	274	23	101	46	53	686	44	M	15	12	no	vertical	vertical	straight	no	0	0	80,47	no
La Coruna	WORLD CUP GRAND PRIX – CAIXABANK TROPHY CSI W 5 /CSI 2 round	10.12.2017	CSI-W5*	4	1.60	490	2	13	17.04.2005	G	KWPN	274	23	101	46	53	686	44	M	16	13	no	spread	oxer	straight	no	0	0	80,47	no
La Coruna	WORLD CUP GRAND PRIX – CAIXABANK TROPHY CSI W 5 /CSI 2 round	10.12.2017	CSI-W5*	4	1.60	490	2	13	17.04.2005	G	KWPN	274	23	101	46	53	686	44	M	17	14	no	vertical	vertical with water	left	no	0	0	80,47	no
La Coruna	WORLD CUP GRAND PRIX – CAIXABANK TROPHY CSI W 5 /CSI 2 round	10.12.2017	CSI-W5*	4	1.60	490	144	14	28.02.2004	G	DSP	186	2	103	171	32	1274	37	M	1	1	no	spread	oxer	left	no	0	0	80,1	no
La Coruna	WORLD CUP GRAND PRIX – CAIXABANK TROPHY CSI W 5 /CSI 2 round	10.12.2017	CSI-W5*	4	1.60	490	144	14	28.02.2004	G	DSP	186	2	103	171	32	1274	37	M	2	2	no	vertical	vertical	left	no	0	0	80,1	no
La Coruna	WORLD CUP GRAND PRIX – CAIXABANK TROPHY CSI W 5 /CSI 2 round	10.12.2017	CSI-W5*	4	1.60	490	144	14	28.02.2004	G	DSP	186	2	103	171	32	1274	37	M	3	3	no	spread	oxer	straight	no	0	0	80,1	no
La Coruna	WORLD CUP GRAND PRIX – CAIXABANK TROPHY CSI W 5 /CSI 2 round	10.12.2017	CSI-W5*	4	1.60	490	144	14	28.02.2004	G	DSP	186	2	103	171	32	1274	37	M	4	4	no	spread	oxer	right	no	0	0	80,1	no
La Coruna	WORLD CUP GRAND PRIX – CAIXABANK TROPHY CSI W 5 /CSI 2 round	10.12.2017	CSI-W5*	4	1.60	490	144	14	28.02.2004	G	DSP	186	2	103	171	32	1274	37	M	5	5a	yes	vertical	vertical	right	no	0	0	80,1	no
La Coruna	WORLD CUP GRAND PRIX – CAIXABANK TROPHY CSI W 5 /CSI 2 round	10.12.2017	CSI-W5*	4	1.60	490	144	14	28.02.2004	G	DSP	186	2	103	171	32	1274	37	M	6	5b	yes	vertical	vertical	right	no	0	0	80,1	no
La Coruna	WORLD CUP GRAND PRIX – CAIXABANK TROPHY CSI W 5 /CSI 2 round	10.12.2017	CSI-W5*	4	1.60	490	144	14	28.02.2004	G	DSP	186	2	103	171	32	1274	37	M	7	5c	yes	spread	oxer	right	no	0	0	80,1	no
La Coruna	WORLD CUP GRAND PRIX – CAIXABANK TROPHY CSI W 5 /CSI 2 round	10.12.2017	CSI-W5*	4	1.60	490	144	14	28.02.2004	G	DSP	186	2	103	171	32	1274	37	M	8	6	no	vertical	vertical	left	no	0	0	80,1	no
La Coruna	WORLD CUP GRAND PRIX – CAIXABANK TROPHY CSI W 5 /CSI 2 round	10.12.2017	CSI-W5*	4	1.60	490	144	14	28.02.2004	G	DSP	186	2	103	171	32	1274	37	M	9	7	no	spread	oxer	left	no	0	0	80,1	no
La Coruna	WORLD CUP GRAND PRIX – CAIXABANK TROPHY CSI W 5 /CSI 2 round	10.12.2017	CSI-W5*	4	1.60	490	144	14	28.02.2004	G	DSP	186	2	103	171	32	1274	37	M	10	8a	yes	spread	oxer	straight	no	0	0	80,1	no
La Coruna	WORLD CUP GRAND PRIX – CAIXABANK TROPHY CSI W 5 /CSI 2 round	10.12.2017	CSI-W5*	4	1.60	490	144	14	28.02.2004	G	DSP	186	2	103	171	32	1274	37	M	11	8b	yes	vertical	vertical	straight	no	0	0	80,1	no
La Coruna	WORLD CUP GRAND PRIX – CAIXABANK TROPHY CSI W 5 /CSI 2 round	10.12.2017	CSI-W5*	4	1.60	490	144	14	28.02.2004	G	DSP	186	2	103	171	32	1274	37	M	12	9	no	spread	oxer with water	left	no	0	0	80,1	no
La Coruna	WORLD CUP GRAND PRIX – CAIXABANK TROPHY CSI W 5 /CSI 2 round	10.12.2017	CSI-W5*	4	1.60	490	144	14	28.02.2004	G	DSP	186	2	103	171	32	1274	37	M	13	10	no	vertical	vertical	right	no	0	0	80,1	no
La Coruna	WORLD CUP GRAND PRIX – CAIXABANK TROPHY CSI W 5 /CSI 2 round	10.12.2017	CSI-W5*	4	1.60	490	144	14	28.02.2004	G	DSP	186	2	103	171	32	1274	37	M	14	11	no	spread	oxer	right	yes	4	0	80,1	no
La Coruna	WORLD CUP GRAND PRIX – CAIXABANK TROPHY CSI W 5 /CSI 2 round	10.12.2017	CSI-W5*	4	1.60	490	144	14	28.02.2004	G	DSP	186	2	103	171	32	1274	37	M	15	12	no	vertical	vertical	straight	no	0	0	80,1	no
La Coruna	WORLD CUP GRAND PRIX – CAIXABANK TROPHY CSI W 5 /CSI 2 round	10.12.2017	CSI-W5*	4	1.60	490	144	14	28.02.2004	G	DSP	186	2	103	171	32	1274	37	M	16	13	no	spread	oxer	straight	no	0	0	80,1	no
La Coruna	WORLD CUP GRAND PRIX – CAIXABANK TROPHY CSI W 5 /CSI 2 round	10.12.2017	CSI-W5*	4	1.60	490	144	14	28.02.2004	G	DSP	186	2	103	171	32	1274	37	M	17	14	no	vertical	vertical with water	left	no	0	0	80,1	no
La Coruna	WORLD CUP GRAND PRIX – CAIXABANK TROPHY CSI W 5 /CSI 2 round	10.12.2017	CSI-W5*	4	1.60	490	6	15	17.04.2003	M	HANN	261	7	104	24	42	2261	97	M	1	1	no	spread	oxer	left	no	0	0	81,72	no
La Coruna	WORLD CUP GRAND PRIX – CAIXABANK TROPHY CSI W 5 /CSI 2 round	10.12.2017	CSI-W5*	4	1.60	490	6	15	17.04.2003	M	HANN	261	7	104	24	42	2261	97	M	2	2	no	vertical	vertical	left	no	0	0	81,72	no
La Coruna	WORLD CUP GRAND PRIX – CAIXABANK TROPHY CSI W 5 /CSI 2 round	10.12.2017	CSI-W5*	4	1.60	490	6	15	17.04.2003	M	HANN	261	7	104	24	42	2261	97	M	3	3	no	spread	oxer	straight	no	0	0	81,72	no
La Coruna	WORLD CUP GRAND PRIX – CAIXABANK TROPHY CSI W 5 /CSI 2 round	10.12.2017	CSI-W5*	4	1.60	490	6	15	17.04.2003	M	HANN	261	7	104	24	42	2261	97	M	4	4	no	spread	oxer	right	no	0	0	81,72	no
La Coruna	WORLD CUP GRAND PRIX – CAIXABANK TROPHY CSI W 5 /CSI 2 round	10.12.2017	CSI-W5*	4	1.60	490	6	15	17.04.2003	M	HANN	261	7	104	24	42	2261	97	M	5	5a	yes	vertical	vertical	right	no	0	0	81,72	no
La Coruna	WORLD CUP GRAND PRIX – CAIXABANK TROPHY CSI W 5 /CSI 2 round	10.12.2017	CSI-W5*	4	1.60	490	6	15	17.04.2003	M	HANN	261	7	104	24	42	2261	97	M	6	5b	yes	vertical	vertical	right	no	0	0	81,72	no
La Coruna	WORLD CUP GRAND PRIX – CAIXABANK TROPHY CSI W 5 /CSI 2 round	10.12.2017	CSI-W5*	4	1.60	490	6	15	17.04.2003	M	HANN	261	7	104	24	42	2261	97	M	7	5c	yes	spread	oxer	right	no	0	0	81,72	no
La Coruna	WORLD CUP GRAND PRIX – CAIXABANK TROPHY CSI W 5 /CSI 2 round	10.12.2017	CSI-W5*	4	1.60	490	6	15	17.04.2003	M	HANN	261	7	104	24	42	2261	97	M	8	6	no	vertical	vertical	left	no	0	0	81,72	no
La Coruna	WORLD CUP GRAND PRIX – CAIXABANK TROPHY CSI W 5 /CSI 2 round	10.12.2017	CSI-W5*	4	1.60	490	6	15	17.04.2003	M	HANN	261	7	104	24	42	2261	97	M	9	7	no	spread	oxer	left	no	0	0	81,72	no
La Coruna	WORLD CUP GRAND PRIX – CAIXABANK TROPHY CSI W 5 /CSI 2 round	10.12.2017	CSI-W5*	4	1.60	490	6	15	17.04.2003	M	HANN	261	7	104	24	42	2261	97	M	10	8a	yes	spread	oxer	straight	no	0	0	81,72	no
La Coruna	WORLD CUP GRAND PRIX – CAIXABANK TROPHY CSI W 5 /CSI 2 round	10.12.2017	CSI-W5*	4	1.60	490	6	15	17.04.2003	M	HANN	261	7	104	24	42	2261	97	M	11	8b	yes	vertical	vertical	straight	no	0	0	81,72	no
La Coruna	WORLD CUP GRAND PRIX – CAIXABANK TROPHY CSI W 5 /CSI 2 round	10.12.2017	CSI-W5*	4	1.60	490	6	15	17.04.2003	M	HANN	261	7	104	24	42	2261	97	M	12	9	no	spread	oxer with water	left	no	0	0	81,72	no
La Coruna	WORLD CUP GRAND PRIX – CAIXABANK TROPHY CSI W 5 /CSI 2 round	10.12.2017	CSI-W5*	4	1.60	490	6	15	17.04.2003	M	HANN	261	7	104	24	42	2261	97	M	13	10	no	vertical	vertical	right	no	0	0	81,72	no
La Coruna	WORLD CUP GRAND PRIX – CAIXABANK TROPHY CSI W 5 /CSI 2 round	10.12.2017	CSI-W5*	4	1.60	490	6	15	17.04.2003	M	HANN	261	7	104	24	42	2261	97	M	14	11	no	spread	oxer	right	no	0	0	81,72	no
La Coruna	WORLD CUP GRAND PRIX – CAIXABANK TROPHY CSI W 5 /CSI 2 round	10.12.2017	CSI-W5*	4	1.60	490	6	15	17.04.2003	M	HANN	261	7	104	24	42	2261	97	M	15	12	no	vertical	vertical	straight	no	0	0	81,72	no
La Coruna	WORLD CUP GRAND PRIX – CAIXABANK TROPHY CSI W 5 /CSI 2 round	10.12.2017	CSI-W5*	4	1.60	490	6	15	17.04.2003	M	HANN	261	7	104	24	42	2261	97	M	16	13	no	spread	oxer	straight	no	0	0	81,72	no
La Coruna	WORLD CUP GRAND PRIX – CAIXABANK TROPHY CSI W 5 /CSI 2 round	10.12.2017	CSI-W5*	4	1.60	490	6	15	17.04.2003	M	HANN	261	7	104	24	42	2261	97	M	17	14	no	vertical	vertical with water	left	no	0	0	81,72	no
La Coruna	WORLD CUP GRAND PRIX – CAIXABANK TROPHY CSI W 5 /CSI 2 round	10.12.2017	CSI-W5*	4	1.60	285	6	15	17.04.2003	M	HANN	261	7	104	24	42	2261	97	M	1	1	no	vertical	vertical	left	no	0	0	45,77	no
La Coruna	WORLD CUP GRAND PRIX – CAIXABANK TROPHY CSI W 5 /CSI 2 round	10.12.2017	CSI-W5*	4	1.60	285	6	15	17.04.2003	M	HANN	261	7	104	24	42	2261	97	M	2	2	no	spread	oxer	right	no	0	0	45,77	no
La Coruna	WORLD CUP GRAND PRIX – CAIXABANK TROPHY CSI W 5 /CSI 2 round	10.12.2017	CSI-W5*	4	1.60	285	6	15	17.04.2003	M	HANN	261	7	104	24	42	2261	97	M	3	3	no	spread	oxer with water	left	no	0	0	45,77	no
La Coruna	WORLD CUP GRAND PRIX – CAIXABANK TROPHY CSI W 5 /CSI 2 round	10.12.2017	CSI-W5*	4	1.60	285	6	15	17.04.2003	M	HANN	261	7	104	24	42	2261	97	M	4	4	no	vertical	vertical	right	no	0	0	45,77	no
La Coruna	WORLD CUP GRAND PRIX – CAIXABANK TROPHY CSI W 5 /CSI 2 round	10.12.2017	CSI-W5*	4	1.60	285	6	15	17.04.2003	M	HANN	261	7	104	24	42	2261	97	M	5	5	no	spread	oxer	right	no	0	0	45,77	no
La Coruna	WORLD CUP GRAND PRIX – CAIXABANK TROPHY CSI W 5 /CSI 2 round	10.12.2017	CSI-W5*	4	1.60	285	6	15	17.04.2003	M	HANN	261	7	104	24	42	2261	97	M	6	6a	yes	vertical	vertical	right	no	0	0	45,77	no
La Coruna	WORLD CUP GRAND PRIX – CAIXABANK TROPHY CSI W 5 /CSI 2 round	10.12.2017	CSI-W5*	4	1.60	285	6	15	17.04.2003	M	HANN	261	7	104	24	42	2261	97	M	7	6b	yes	spread	oxer	right	no	0	0	45,77	no
La Coruna	WORLD CUP GRAND PRIX – CAIXABANK TROPHY CSI W 5 /CSI 2 round	10.12.2017	CSI-W5*	4	1.60	285	6	15	17.04.2003	M	HANN	261	7	104	24	42	2261	97	M	8	7	no	vertical	vertical	left	no	0	0	45,77	no
La Coruna	WORLD CUP GRAND PRIX – CAIXABANK TROPHY CSI W 5 /CSI 2 round	10.12.2017	CSI-W5*	4	1.60	490	139	9	20.05.2009	G	AES	45	2	106	176	41	1364	65	M	1	1	no	spread	oxer	left	no	0	0	0,00	yes
La Coruna	WORLD CUP GRAND PRIX – CAIXABANK TROPHY CSI W 5 /CSI 2 round	10.12.2017	CSI-W5*	4	1.60	490	139	9	20.05.2009	G	AES	45	2	106	176	41	1364	65	M	2	2	no	vertical	vertical	left	no	0	0	0,00	yes
La Coruna	WORLD CUP GRAND PRIX – CAIXABANK TROPHY CSI W 5 /CSI 2 round	10.12.2017	CSI-W5*	4	1.60	490	139	9	20.05.2009	G	AES	45	2	106	176	41	1364	65	M	3	3	no	spread	oxer	straight	no	0	0	0,00	yes
La Coruna	WORLD CUP GRAND PRIX – CAIXABANK TROPHY CSI W 5 /CSI 2 round	10.12.2017	CSI-W5*	4	1.60	490	139	9	20.05.2009	G	AES	45	2	106	176	41	1364	65	M	4	4	no	spread	oxer	right	yes	4	0	0,00	yes
La Coruna	WORLD CUP GRAND PRIX – CAIXABANK TROPHY CSI W 5 /CSI 2 round	10.12.2017	CSI-W5*	4	1.60	490	139	9	20.05.2009	G	AES	45	2	106	176	41	1364	65	M	5	5a	yes	vertical	vertical	right	no	0	0	0,00	yes
La Coruna	WORLD CUP GRAND PRIX – CAIXABANK TROPHY CSI W 5 /CSI 2 round	10.12.2017	CSI-W5*	4	1.60	490	139	9	20.05.2009	G	AES	45	2	106	176	41	1364	65	M	6	5b	yes	vertical	vertical	right	yes	4	0	0,00	yes
La Coruna	WORLD CUP GRAND PRIX – CAIXABANK TROPHY CSI W 5 /CSI 2 round	10.12.2017	CSI-W5*	4	1.60	490	139	9	20.05.2009	G	AES	45	2	106	176	41	1364	65	M	7	5c	yes	spread	oxer	right	no	0	0	0,00	yes
La Coruna	WORLD CUP GRAND PRIX – CAIXABANK TROPHY CSI W 5 /CSI 2 round	10.12.2017	CSI-W5*	4	1.60	490	139	9	20.05.2009	G	AES	45	2	106	176	41	1364	65	M	8	6	no	vertical	vertical	left	no	0	0	0,00	yes
La Coruna	WORLD CUP GRAND PRIX – CAIXABANK TROPHY CSI W 5 /CSI 2 round	10.12.2017	CSI-W5*	4	1.60	490	139	9	20.05.2009	G	AES	45	2	106	176	41	1364	65	M	9	7	no	spread	oxer	left	no	0	0	0,00	yes
La Coruna	WORLD CUP GRAND PRIX – CAIXABANK TROPHY CSI W 5 /CSI 2 round	10.12.2017	CSI-W5*	4	1.60	490	139	9	20.05.2009	G	AES	45	2	106	176	41	1364	65	M	10	8a	yes	spread	oxer	straight	no	0	0	0,00	yes
La Coruna	WORLD CUP GRAND PRIX – CAIXABANK TROPHY CSI W 5 /CSI 2 round	10.12.2017	CSI-W5*	4	1.60	490	139	9	20.05.2009	G	AES	45	2	106	176	41	1364	65	M	11	8b	yes	vertical	vertical	straight	no	0	0	0,00	yes
La Coruna	WORLD CUP GRAND PRIX – CAIXABANK TROPHY CSI W 5 /CSI 2 round	10.12.2017	CSI-W5*	4	1.60	490	139	9	20.05.2009	G	AES	45	2	106	176	41	1364	65	M	12	9	no	spread	oxer with water	left	no	0	0	0,00	yes
La Coruna	WORLD CUP GRAND PRIX – CAIXABANK TROPHY CSI W 5 /CSI 2 round	10.12.2017	CSI-W5*	4	1.60	490	139	9	20.05.2009	G	AES	45	2	106	176	41	1364	65	M	13	10	no	vertical	vertical	right	no	0	0	0,00	yes
La Coruna	WORLD CUP GRAND PRIX – CAIXABANK TROPHY CSI W 5 /CSI 2 round	10.12.2017	CSI-W5*	4	1.60	490	139	9	20.05.2009	G	AES	45	2	106	176	41	1364	65	M	14	11	no	spread	oxer	right	no	0	0	0,00	yes
La Coruna	WORLD CUP GRAND PRIX – CAIXABANK TROPHY CSI W 5 /CSI 2 round	10.12.2017	CSI-W5*	4	1.60	490	139	9	20.05.2009	G	AES	45	2	106	176	41	1364	65	M	15	12	no	vertical	vertical	straight	yes	4	0	0,00	yes
La Coruna	WORLD CUP GRAND PRIX – CAIXABANK TROPHY CSI W 5 /CSI 2 round	10.12.2017	CSI-W5*	4	1.60	490	139	9	20.05.2009	G	AES	45	2	106	176	41	1364	65	M	16	13	no	spread	oxer	straight	no	0	0	0,00	yes
La Coruna	WORLD CUP GRAND PRIX – CAIXABANK TROPHY CSI W 5 /CSI 2 round	10.12.2017	CSI-W5*	4	1.60	490	139	9	20.05.2009	G	AES	45	2	106	176	41	1364	65	M	17	14	no	vertical	vertical with water	left	no	0	0	0,00	yes
La Coruna	WORLD CUP GRAND PRIX – CAIXABANK TROPHY CSI W 5 /CSI 2 round	10.12.2017	CSI-W5*	4	1.60	490	193	12	17.06.2006	M	SF	154	3	107	248	43	1244	64	M	1	1	no	spread	oxer	left	no	0	0	0,00	yes
La Coruna	WORLD CUP GRAND PRIX – CAIXABANK TROPHY CSI W 5 /CSI 2 round	10.12.2017	CSI-W5*	4	1.60	490	193	12	17.06.2006	M	SF	154	3	107	248	43	1244	64	M	2	2	no	vertical	vertical	left	no	0	0	0,00	yes
La Coruna	WORLD CUP GRAND PRIX – CAIXABANK TROPHY CSI W 5 /CSI 2 round	10.12.2017	CSI-W5*	4	1.60	490	193	12	17.06.2006	M	SF	154	3	107	248	43	1244	64	M	3	3	no	spread	oxer	straight	no	0	0	0,00	yes
La Coruna	WORLD CUP GRAND PRIX – CAIXABANK TROPHY CSI W 5 /CSI 2 round	10.12.2017	CSI-W5*	4	1.60	490	193	12	17.06.2006	M	SF	154	3	107	248	43	1244	64	M	4	4	no	spread	oxer	right	no	0	0	0,00	yes
La Coruna	WORLD CUP GRAND PRIX – CAIXABANK TROPHY CSI W 5 /CSI 2 round	10.12.2017	CSI-W5*	4	1.60	490	193	12	17.06.2006	M	SF	154	3	107	248	43	1244	64	M	5	5a	yes	vertical	vertical	right	no	0	0	0,00	yes
La Coruna	WORLD CUP GRAND PRIX – CAIXABANK TROPHY CSI W 5 /CSI 2 round	10.12.2017	CSI-W5*	4	1.60	490	193	12	17.06.2006	M	SF	154	3	107	248	43	1244	64	M	6	5b	yes	vertical	vertical	right	no	0	0	0,00	yes
La Coruna	WORLD CUP GRAND PRIX – CAIXABANK TROPHY CSI W 5 /CSI 2 round	10.12.2017	CSI-W5*	4	1.60	490	193	12	17.06.2006	M	SF	154	3	107	248	43	1244	64	M	7	5c	yes	spread	oxer	right	no	0	0	0,00	yes
La Coruna	WORLD CUP GRAND PRIX – CAIXABANK TROPHY CSI W 5 /CSI 2 round	10.12.2017	CSI-W5*	4	1.60	490	193	12	17.06.2006	M	SF	154	3	107	248	43	1244	64	M	8	6	no	vertical	vertical	left	no	0	0	0,00	yes
La Coruna	WORLD CUP GRAND PRIX – CAIXABANK TROPHY CSI W 5 /CSI 2 round	10.12.2017	CSI-W5*	4	1.60	490	193	12	17.06.2006	M	SF	154	3	107	248	43	1244	64	M	9	7	no	spread	oxer	left	no	0	0	0,00	yes
La Coruna	WORLD CUP GRAND PRIX – CAIXABANK TROPHY CSI W 5 /CSI 2 round	10.12.2017	CSI-W5*	4	1.60	490	193	12	17.06.2006	M	SF	154	3	107	248	43	1244	64	M	10	8a	yes	spread	oxer	straight	no	0	0	0,00	yes
La Coruna	WORLD CUP GRAND PRIX – CAIXABANK TROPHY CSI W 5 /CSI 2 round	10.12.2017	CSI-W5*	4	1.60	490	193	12	17.06.2006	M	SF	154	3	107	248	43	1244	64	M	11	8b	yes	vertical	vertical	straight	no	0	0	0,00	yes
La Coruna	WORLD CUP GRAND PRIX – CAIXABANK TROPHY CSI W 5 /CSI 2 round	10.12.2017	CSI-W5*	4	1.60	490	193	12	17.06.2006	M	SF	154	3	107	248	43	1244	64	M	12	9	no	spread	oxer with water	left	no	0	0	0,00	yes
La Coruna	WORLD CUP GRAND PRIX – CAIXABANK TROPHY CSI W 5 /CSI 2 round	10.12.2017	CSI-W5*	4	1.60	490	193	12	17.06.2006	M	SF	154	3	107	248	43	1244	64	M	13	10	no	vertical	vertical	right	no	0	0	0,00	yes
La Coruna	WORLD CUP GRAND PRIX – CAIXABANK TROPHY CSI W 5 /CSI 2 round	10.12.2017	CSI-W5*	4	1.60	490	193	12	17.06.2006	M	SF	154	3	107	248	43	1244	64	M	14	11	no	spread	oxer	right	yes	4	0	0,00	yes
La Coruna	WORLD CUP GRAND PRIX – CAIXABANK TROPHY CSI W 5 /CSI 2 round	10.12.2017	CSI-W5*	4	1.60	490	193	12	17.06.2006	M	SF	154	3	107	248	43	1244	64	M	15	12	no	vertical	vertical	straight	yes	4	0	0,00	yes
La Coruna	WORLD CUP GRAND PRIX – CAIXABANK TROPHY CSI W 5 /CSI 2 round	10.12.2017	CSI-W5*	4	1.60	490	193	12	17.06.2006	M	SF	154	3	107	248	43	1244	64	M	16	13	no	spread	oxer	straight	no	0	0	0,00	yes
La Coruna	WORLD CUP GRAND PRIX – CAIXABANK TROPHY CSI W 5 /CSI 2 round	10.12.2017	CSI-W5*	4	1.60	490	193	12	17.06.2006	M	SF	154	3	107	248	43	1244	64	M	17	14	no	vertical	vertical with water	left	no	0	0	0,00	yes
La Coruna	WORLD CUP GRAND PRIX – CAIXABANK TROPHY CSI W 5 /CSI 2 round	10.12.2017	CSI-W5*	4	1.60	490	33	13	01.01.2005	S	Bezplemenné	154	4	108	206	42	569	26	M	1	1	no	spread	oxer	left	no	0	0	0,00	yes
La Coruna	WORLD CUP GRAND PRIX – CAIXABANK TROPHY CSI W 5 /CSI 2 round	10.12.2017	CSI-W5*	4	1.60	490	33	13	01.01.2005	S	Bezplemenné	154	4	108	206	42	569	26	M	2	2	no	vertical	vertical	left	no	0	0	0,00	yes
La Coruna	WORLD CUP GRAND PRIX – CAIXABANK TROPHY CSI W 5 /CSI 2 round	10.12.2017	CSI-W5*	4	1.60	490	33	13	01.01.2005	S	Bezplemenné	154	4	108	206	42	569	26	M	3	3	no	spread	oxer	straight	no	0	0	0,00	yes
La Coruna	WORLD CUP GRAND PRIX – CAIXABANK TROPHY CSI W 5 /CSI 2 round	10.12.2017	CSI-W5*	4	1.60	490	33	13	01.01.2005	S	Bezplemenné	154	4	108	206	42	569	26	M	4	4	no	spread	oxer	right	yes	4	0	0,00	yes
La Coruna	WORLD CUP GRAND PRIX – CAIXABANK TROPHY CSI W 5 /CSI 2 round	10.12.2017	CSI-W5*	4	1.60	490	33	13	01.01.2005	S	Bezplemenné	154	4	108	206	42	569	26	M	5	5a	yes	vertical	vertical	right	no	0	0	0,00	yes
La Coruna	WORLD CUP GRAND PRIX – CAIXABANK TROPHY CSI W 5 /CSI 2 round	10.12.2017	CSI-W5*	4	1.60	490	33	13	01.01.2005	S	Bezplemenné	154	4	108	206	42	569	26	M	6	5b	yes	vertical	vertical	right	no	0	0	0,00	yes
La Coruna	WORLD CUP GRAND PRIX – CAIXABANK TROPHY CSI W 5 /CSI 2 round	10.12.2017	CSI-W5*	4	1.60	490	33	13	01.01.2005	S	Bezplemenné	154	4	108	206	42	569	26	M	7	5c	yes	spread	oxer	right	yes	4	0	0,00	yes
La Coruna	WORLD CUP GRAND PRIX – CAIXABANK TROPHY CSI W 5 /CSI 2 round	10.12.2017	CSI-W5*	4	1.60	490	33	13	01.01.2005	S	Bezplemenné	154	4	108	206	42	569	26	M	8	6	no	vertical	vertical	left	no	0	0	0,00	yes
La Coruna	WORLD CUP GRAND PRIX – CAIXABANK TROPHY CSI W 5 /CSI 2 round	10.12.2017	CSI-W5*	4	1.60	490	33	13	01.01.2005	S	Bezplemenné	154	4	108	206	42	569	26	M	9	7	no	spread	oxer	left	no	0	0	0,00	yes
La Coruna	WORLD CUP GRAND PRIX – CAIXABANK TROPHY CSI W 5 /CSI 2 round	10.12.2017	CSI-W5*	4	1.60	490	33	13	01.01.2005	S	Bezplemenné	154	4	108	206	42	569	26	M	10	8a	yes	spread	oxer	straight	no	0	0	0,00	yes
La Coruna	WORLD CUP GRAND PRIX – CAIXABANK TROPHY CSI W 5 /CSI 2 round	10.12.2017	CSI-W5*	4	1.60	490	33	13	01.01.2005	S	Bezplemenné	154	4	108	206	42	569	26	M	11	8b	yes	vertical	vertical	straight	no	0	0	0,00	yes
La Coruna	WORLD CUP GRAND PRIX – CAIXABANK TROPHY CSI W 5 /CSI 2 round	10.12.2017	CSI-W5*	4	1.60	490	33	13	01.01.2005	S	Bezplemenné	154	4	108	206	42	569	26	M	12	9	no	spread	oxer with water	left	no	0	0	0,00	yes
La Coruna	WORLD CUP GRAND PRIX – CAIXABANK TROPHY CSI W 5 /CSI 2 round	10.12.2017	CSI-W5*	4	1.60	490	33	13	01.01.2005	S	Bezplemenné	154	4	108	206	42	569	26	M	13	10	no	vertical	vertical	right	no	0	0	0,00	yes
La Coruna	WORLD CUP GRAND PRIX – CAIXABANK TROPHY CSI W 5 /CSI 2 round	10.12.2017	CSI-W5*	4	1.60	490	33	13	01.01.2005	S	Bezplemenné	154	4	108	206	42	569	26	M	14	11	no	spread	oxer	right	no	0	0	0,00	yes
La Coruna	WORLD CUP GRAND PRIX – CAIXABANK TROPHY CSI W 5 /CSI 2 round	10.12.2017	CSI-W5*	4	1.60	490	33	13	01.01.2005	S	Bezplemenné	154	4	108	206	42	569	26	M	15	12	no	vertical	vertical	straight	no	0	0	0,00	yes
La Coruna	WORLD CUP GRAND PRIX – CAIXABANK TROPHY CSI W 5 /CSI 2 round	10.12.2017	CSI-W5*	4	1.60	490	33	13	01.01.2005	S	Bezplemenné	154	4	108	206	42	569	26	M	16	13	no	spread	oxer	straight	no	0	0	0,00	yes
La Coruna	WORLD CUP GRAND PRIX – CAIXABANK TROPHY CSI W 5 /CSI 2 round	10.12.2017	CSI-W5*	4	1.60	490	33	13	01.01.2005	S	Bezplemenné	154	4	108	206	42	569	26	M	17	14	no	vertical	vertical with water	left	no	0	0	0,00	yes
La Coruna	WORLD CUP GRAND PRIX – CAIXABANK TROPHY CSI W 5 /CSI 2 round	10.12.2017	CSI-W5*	4	1.60	490	62	11	03.05.2007	G	SWB	48	0	109	266	40	1077	15	F	1	1	no	spread	oxer	left	yes	4	0	88,52	no
La Coruna	WORLD CUP GRAND PRIX – CAIXABANK TROPHY CSI W 5 /CSI 2 round	10.12.2017	CSI-W5*	4	1.60	490	62	11	03.05.2007	G	SWB	48	0	109	266	40	1077	15	F	2	2	no	vertical	vertical	left	no	0	0	88,52	no
La Coruna	WORLD CUP GRAND PRIX – CAIXABANK TROPHY CSI W 5 /CSI 2 round	10.12.2017	CSI-W5*	4	1.60	490	62	11	03.05.2007	G	SWB	48	0	109	266	40	1077	15	F	3	3	no	spread	oxer	straight	no	0	0	88,52	no
La Coruna	WORLD CUP GRAND PRIX – CAIXABANK TROPHY CSI W 5 /CSI 2 round	10.12.2017	CSI-W5*	4	1.60	490	62	11	03.05.2007	G	SWB	48	0	109	266	40	1077	15	F	4	4	no	spread	oxer	right	yes	4	0	88,52	no
La Coruna	WORLD CUP GRAND PRIX – CAIXABANK TROPHY CSI W 5 /CSI 2 round	10.12.2017	CSI-W5*	4	1.60	490	62	11	03.05.2007	G	SWB	48	0	109	266	40	1077	15	F	5	5a	yes	vertical	vertical	right	no	0	0	88,52	no
La Coruna	WORLD CUP GRAND PRIX – CAIXABANK TROPHY CSI W 5 /CSI 2 round	10.12.2017	CSI-W5*	4	1.60	490	62	11	03.05.2007	G	SWB	48	0	109	266	40	1077	15	F	6	5b	yes	vertical	vertical	right	no	0	0	88,52	no
La Coruna	WORLD CUP GRAND PRIX – CAIXABANK TROPHY CSI W 5 /CSI 2 round	10.12.2017	CSI-W5*	4	1.60	490	62	11	03.05.2007	G	SWB	48	0	109	266	40	1077	15	F	7	5c	yes	spread	oxer	right	no	0	0	88,52	no
La Coruna	WORLD CUP GRAND PRIX – CAIXABANK TROPHY CSI W 5 /CSI 2 round	10.12.2017	CSI-W5*	4	1.60	490	62	11	03.05.2007	G	SWB	48	0	109	266	40	1077	15	F	8	6	no	vertical	vertical	left	no	0	0	88,52	no
La Coruna	WORLD CUP GRAND PRIX – CAIXABANK TROPHY CSI W 5 /CSI 2 round	10.12.2017	CSI-W5*	4	1.60	490	62	11	03.05.2007	G	SWB	48	0	109	266	40	1077	15	F	9	7	no	spread	oxer	left	no	0	0	88,52	no
La Coruna	WORLD CUP GRAND PRIX – CAIXABANK TROPHY CSI W 5 /CSI 2 round	10.12.2017	CSI-W5*	4	1.60	490	62	11	03.05.2007	G	SWB	48	0	109	266	40	1077	15	F	10	8a	yes	spread	oxer	straight	no	0	0	88,52	no
La Coruna	WORLD CUP GRAND PRIX – CAIXABANK TROPHY CSI W 5 /CSI 2 round	10.12.2017	CSI-W5*	4	1.60	490	62	11	03.05.2007	G	SWB	48	0	109	266	40	1077	15	F	11	8b	yes	vertical	vertical	straight	no	0	0	88,52	no
La Coruna	WORLD CUP GRAND PRIX – CAIXABANK TROPHY CSI W 5 /CSI 2 round	10.12.2017	CSI-W5*	4	1.60	490	62	11	03.05.2007	G	SWB	48	0	109	266	40	1077	15	F	12	9	no	spread	oxer with water	left	yes	4	0	88,52	no
La Coruna	WORLD CUP GRAND PRIX – CAIXABANK TROPHY CSI W 5 /CSI 2 round	10.12.2017	CSI-W5*	4	1.60	490	62	11	03.05.2007	G	SWB	48	0	109	266	40	1077	15	F	13	10	no	vertical	vertical	right	no	0	0	88,52	no
La Coruna	WORLD CUP GRAND PRIX – CAIXABANK TROPHY CSI W 5 /CSI 2 round	10.12.2017	CSI-W5*	4	1.60	490	62	11	03.05.2007	G	SWB	48	0	109	266	40	1077	15	F	14	11	no	spread	oxer	right	no	0	0	88,52	no
La Coruna	WORLD CUP GRAND PRIX – CAIXABANK TROPHY CSI W 5 /CSI 2 round	10.12.2017	CSI-W5*	4	1.60	490	62	11	03.05.2007	G	SWB	48	0	109	266	40	1077	15	F	15	12	no	vertical	vertical	straight	no	0	0	88,52	no
La Coruna	WORLD CUP GRAND PRIX – CAIXABANK TROPHY CSI W 5 /CSI 2 round	10.12.2017	CSI-W5*	4	1.60	490	62	11	03.05.2007	G	SWB	48	0	109	266	40	1077	15	F	16	13	no	spread	oxer	straight	no	0	0	88,52	no
La Coruna	WORLD CUP GRAND PRIX – CAIXABANK TROPHY CSI W 5 /CSI 2 round	10.12.2017	CSI-W5*	4	1.60	490	62	11	03.05.2007	G	SWB	48	0	109	266	40	1077	15	F	17	14	no	vertical	vertical with water	left	no	0	6	88,52	no
La Coruna	WORLD CUP GRAND PRIX – CAIXABANK TROPHY CSI W 5 /CSI 2 round	10.12.2017	CSI-W5*	4	1.60	490	98	12	01.07.2006	M	BWP	122	0	110	542	45	1564	23	F	1	1	no	spread	oxer	left	no	0	0	81,31	no
La Coruna	WORLD CUP GRAND PRIX – CAIXABANK TROPHY CSI W 5 /CSI 2 round	10.12.2017	CSI-W5*	4	1.60	490	98	12	01.07.2006	M	BWP	122	0	110	542	45	1564	23	F	2	2	no	vertical	vertical	left	no	0	0	81,31	no
La Coruna	WORLD CUP GRAND PRIX – CAIXABANK TROPHY CSI W 5 /CSI 2 round	10.12.2017	CSI-W5*	4	1.60	490	98	12	01.07.2006	M	BWP	122	0	110	542	45	1564	23	F	3	3	no	spread	oxer	straight	no	0	0	81,31	no
La Coruna	WORLD CUP GRAND PRIX – CAIXABANK TROPHY CSI W 5 /CSI 2 round	10.12.2017	CSI-W5*	4	1.60	490	98	12	01.07.2006	M	BWP	122	0	110	542	45	1564	23	F	4	4	no	spread	oxer	right	no	0	0	81,31	no
La Coruna	WORLD CUP GRAND PRIX – CAIXABANK TROPHY CSI W 5 /CSI 2 round	10.12.2017	CSI-W5*	4	1.60	490	98	12	01.07.2006	M	BWP	122	0	110	542	45	1564	23	F	5	5a	yes	vertical	vertical	right	no	0	0	81,31	no
La Coruna	WORLD CUP GRAND PRIX – CAIXABANK TROPHY CSI W 5 /CSI 2 round	10.12.2017	CSI-W5*	4	1.60	490	98	12	01.07.2006	M	BWP	122	0	110	542	45	1564	23	F	6	5b	yes	vertical	vertical	right	yes	4	0	81,31	no
La Coruna	WORLD CUP GRAND PRIX – CAIXABANK TROPHY CSI W 5 /CSI 2 round	10.12.2017	CSI-W5*	4	1.60	490	98	12	01.07.2006	M	BWP	122	0	110	542	45	1564	23	F	7	5c	yes	spread	oxer	right	no	0	0	81,31	no
La Coruna	WORLD CUP GRAND PRIX – CAIXABANK TROPHY CSI W 5 /CSI 2 round	10.12.2017	CSI-W5*	4	1.60	490	98	12	01.07.2006	M	BWP	122	0	110	542	45	1564	23	F	8	6	no	vertical	vertical	left	no	0	0	81,31	no
La Coruna	WORLD CUP GRAND PRIX – CAIXABANK TROPHY CSI W 5 /CSI 2 round	10.12.2017	CSI-W5*	4	1.60	490	98	12	01.07.2006	M	BWP	122	0	110	542	45	1564	23	F	9	7	no	spread	oxer	left	no	0	0	81,31	no
La Coruna	WORLD CUP GRAND PRIX – CAIXABANK TROPHY CSI W 5 /CSI 2 round	10.12.2017	CSI-W5*	4	1.60	490	98	12	01.07.2006	M	BWP	122	0	110	542	45	1564	23	F	10	8a	yes	spread	oxer	straight	no	0	0	81,31	no
La Coruna	WORLD CUP GRAND PRIX – CAIXABANK TROPHY CSI W 5 /CSI 2 round	10.12.2017	CSI-W5*	4	1.60	490	98	12	01.07.2006	M	BWP	122	0	110	542	45	1564	23	F	11	8b	yes	vertical	vertical	straight	no	0	0	81,31	no
La Coruna	WORLD CUP GRAND PRIX – CAIXABANK TROPHY CSI W 5 /CSI 2 round	10.12.2017	CSI-W5*	4	1.60	490	98	12	01.07.2006	M	BWP	122	0	110	542	45	1564	23	F	12	9	no	spread	oxer with water	left	yes	4	0	81,31	no
La Coruna	WORLD CUP GRAND PRIX – CAIXABANK TROPHY CSI W 5 /CSI 2 round	10.12.2017	CSI-W5*	4	1.60	490	98	12	01.07.2006	M	BWP	122	0	110	542	45	1564	23	F	13	10	no	vertical	vertical	right	no	0	0	81,31	no
La Coruna	WORLD CUP GRAND PRIX – CAIXABANK TROPHY CSI W 5 /CSI 2 round	10.12.2017	CSI-W5*	4	1.60	490	98	12	01.07.2006	M	BWP	122	0	110	542	45	1564	23	F	14	11	no	spread	oxer	right	no	0	0	81,31	no
La Coruna	WORLD CUP GRAND PRIX – CAIXABANK TROPHY CSI W 5 /CSI 2 round	10.12.2017	CSI-W5*	4	1.60	490	98	12	01.07.2006	M	BWP	122	0	110	542	45	1564	23	F	15	12	no	vertical	vertical	straight	no	0	0	81,31	no
La Coruna	WORLD CUP GRAND PRIX – CAIXABANK TROPHY CSI W 5 /CSI 2 round	10.12.2017	CSI-W5*	4	1.60	490	98	12	01.07.2006	M	BWP	122	0	110	542	45	1564	23	F	16	13	no	spread	oxer	straight	no	0	0	81,31	no
La Coruna	WORLD CUP GRAND PRIX – CAIXABANK TROPHY CSI W 5 /CSI 2 round	10.12.2017	CSI-W5*	4	1.60	490	98	12	01.07.2006	M	BWP	122	0	110	542	45	1564	23	F	17	14	no	vertical	vertical with water	left	no	0	0	81,31	no
La Coruna	WORLD CUP GRAND PRIX – CAIXABANK TROPHY CSI W 5 /CSI 2 round	10.12.2017	CSI-W5*	4	1.60	490	130	10	26.05.2008	S	BWP	116	4	111	532	39	861	51	M	1	1	no	spread	oxer	left	no	0	0	78,13	no
La Coruna	WORLD CUP GRAND PRIX – CAIXABANK TROPHY CSI W 5 /CSI 2 round	10.12.2017	CSI-W5*	4	1.60	490	130	10	26.05.2008	S	BWP	116	4	111	532	39	861	51	M	2	2	no	vertical	vertical	left	no	0	0	78,13	no
La Coruna	WORLD CUP GRAND PRIX – CAIXABANK TROPHY CSI W 5 /CSI 2 round	10.12.2017	CSI-W5*	4	1.60	490	130	10	26.05.2008	S	BWP	116	4	111	532	39	861	51	M	3	3	no	spread	oxer	straight	no	0	0	78,13	no
La Coruna	WORLD CUP GRAND PRIX – CAIXABANK TROPHY CSI W 5 /CSI 2 round	10.12.2017	CSI-W5*	4	1.60	490	130	10	26.05.2008	S	BWP	116	4	111	532	39	861	51	M	4	4	no	spread	oxer	right	no	0	0	78,13	no
La Coruna	WORLD CUP GRAND PRIX – CAIXABANK TROPHY CSI W 5 /CSI 2 round	10.12.2017	CSI-W5*	4	1.60	490	130	10	26.05.2008	S	BWP	116	4	111	532	39	861	51	M	5	5a	yes	vertical	vertical	right	no	0	0	78,13	no
La Coruna	WORLD CUP GRAND PRIX – CAIXABANK TROPHY CSI W 5 /CSI 2 round	10.12.2017	CSI-W5*	4	1.60	490	130	10	26.05.2008	S	BWP	116	4	111	532	39	861	51	M	6	5b	yes	vertical	vertical	right	yes	4	0	78,13	no
La Coruna	WORLD CUP GRAND PRIX – CAIXABANK TROPHY CSI W 5 /CSI 2 round	10.12.2017	CSI-W5*	4	1.60	490	130	10	26.05.2008	S	BWP	116	4	111	532	39	861	51	M	7	5c	yes	spread	oxer	right	no	0	0	78,13	no
La Coruna	WORLD CUP GRAND PRIX – CAIXABANK TROPHY CSI W 5 /CSI 2 round	10.12.2017	CSI-W5*	4	1.60	490	130	10	26.05.2008	S	BWP	116	4	111	532	39	861	51	M	8	6	no	vertical	vertical	left	no	0	0	78,13	no
La Coruna	WORLD CUP GRAND PRIX – CAIXABANK TROPHY CSI W 5 /CSI 2 round	10.12.2017	CSI-W5*	4	1.60	490	130	10	26.05.2008	S	BWP	116	4	111	532	39	861	51	M	9	7	no	spread	oxer	left	no	0	0	78,13	no
La Coruna	WORLD CUP GRAND PRIX – CAIXABANK TROPHY CSI W 5 /CSI 2 round	10.12.2017	CSI-W5*	4	1.60	490	130	10	26.05.2008	S	BWP	116	4	111	532	39	861	51	M	10	8a	yes	spread	oxer	straight	no	0	0	78,13	no
La Coruna	WORLD CUP GRAND PRIX – CAIXABANK TROPHY CSI W 5 /CSI 2 round	10.12.2017	CSI-W5*	4	1.60	490	130	10	26.05.2008	S	BWP	116	4	111	532	39	861	51	M	11	8b	yes	vertical	vertical	straight	no	0	0	78,13	no
La Coruna	WORLD CUP GRAND PRIX – CAIXABANK TROPHY CSI W 5 /CSI 2 round	10.12.2017	CSI-W5*	4	1.60	490	130	10	26.05.2008	S	BWP	116	4	111	532	39	861	51	M	12	9	no	spread	oxer with water	left	no	0	0	78,13	no
La Coruna	WORLD CUP GRAND PRIX – CAIXABANK TROPHY CSI W 5 /CSI 2 round	10.12.2017	CSI-W5*	4	1.60	490	130	10	26.05.2008	S	BWP	116	4	111	532	39	861	51	M	13	10	no	vertical	vertical	right	no	0	0	78,13	no
La Coruna	WORLD CUP GRAND PRIX – CAIXABANK TROPHY CSI W 5 /CSI 2 round	10.12.2017	CSI-W5*	4	1.60	490	130	10	26.05.2008	S	BWP	116	4	111	532	39	861	51	M	14	11	no	spread	oxer	right	no	0	0	78,13	no
La Coruna	WORLD CUP GRAND PRIX – CAIXABANK TROPHY CSI W 5 /CSI 2 round	10.12.2017	CSI-W5*	4	1.60	490	130	10	26.05.2008	S	BWP	116	4	111	532	39	861	51	M	15	12	no	vertical	vertical	straight	no	0	0	78,13	no
La Coruna	WORLD CUP GRAND PRIX – CAIXABANK TROPHY CSI W 5 /CSI 2 round	10.12.2017	CSI-W5*	4	1.60	490	130	10	26.05.2008	S	BWP	116	4	111	532	39	861	51	M	16	13	no	spread	oxer	straight	no	0	0	78,13	no
La Coruna	WORLD CUP GRAND PRIX – CAIXABANK TROPHY CSI W 5 /CSI 2 round	10.12.2017	CSI-W5*	4	1.60	490	130	10	26.05.2008	S	BWP	116	4	111	532	39	861	51	M	17	14	no	vertical	vertical with water	left	no	0	0	78,13	no
La Coruna	WORLD CUP GRAND PRIX – CAIXABANK TROPHY CSI W 5 /CSI 2 round	10.12.2017	CSI-W5*	4	1.60	490	190	14	22.03.2004	M	AES	269	11	112	74	33	1410	55	M	1	1	no	spread	oxer	left	no	0	0	77,62	no
La Coruna	WORLD CUP GRAND PRIX – CAIXABANK TROPHY CSI W 5 /CSI 2 round	10.12.2017	CSI-W5*	4	1.60	490	190	14	22.03.2004	M	AES	269	11	112	74	33	1410	55	M	2	2	no	vertical	vertical	left	no	0	0	77,62	no
La Coruna	WORLD CUP GRAND PRIX – CAIXABANK TROPHY CSI W 5 /CSI 2 round	10.12.2017	CSI-W5*	4	1.60	490	190	14	22.03.2004	M	AES	269	11	112	74	33	1410	55	M	3	3	no	spread	oxer	straight	no	0	0	77,62	no
La Coruna	WORLD CUP GRAND PRIX – CAIXABANK TROPHY CSI W 5 /CSI 2 round	10.12.2017	CSI-W5*	4	1.60	490	190	14	22.03.2004	M	AES	269	11	112	74	33	1410	55	M	4	4	no	spread	oxer	right	no	0	0	77,62	no
La Coruna	WORLD CUP GRAND PRIX – CAIXABANK TROPHY CSI W 5 /CSI 2 round	10.12.2017	CSI-W5*	4	1.60	490	190	14	22.03.2004	M	AES	269	11	112	74	33	1410	55	M	5	5a	yes	vertical	vertical	right	no	0	0	77,62	no
La Coruna	WORLD CUP GRAND PRIX – CAIXABANK TROPHY CSI W 5 /CSI 2 round	10.12.2017	CSI-W5*	4	1.60	490	190	14	22.03.2004	M	AES	269	11	112	74	33	1410	55	M	6	5b	yes	vertical	vertical	right	no	0	0	77,62	no
La Coruna	WORLD CUP GRAND PRIX – CAIXABANK TROPHY CSI W 5 /CSI 2 round	10.12.2017	CSI-W5*	4	1.60	490	190	14	22.03.2004	M	AES	269	11	112	74	33	1410	55	M	7	5c	yes	spread	oxer	right	no	0	0	77,62	no
La Coruna	WORLD CUP GRAND PRIX – CAIXABANK TROPHY CSI W 5 /CSI 2 round	10.12.2017	CSI-W5*	4	1.60	490	190	14	22.03.2004	M	AES	269	11	112	74	33	1410	55	M	8	6	no	vertical	vertical	left	no	0	0	77,62	no
La Coruna	WORLD CUP GRAND PRIX – CAIXABANK TROPHY CSI W 5 /CSI 2 round	10.12.2017	CSI-W5*	4	1.60	490	190	14	22.03.2004	M	AES	269	11	112	74	33	1410	55	M	9	7	no	spread	oxer	left	no	0	0	77,62	no
La Coruna	WORLD CUP GRAND PRIX – CAIXABANK TROPHY CSI W 5 /CSI 2 round	10.12.2017	CSI-W5*	4	1.60	490	190	14	22.03.2004	M	AES	269	11	112	74	33	1410	55	M	10	8a	yes	spread	oxer	straight	no	0	0	77,62	no
La Coruna	WORLD CUP GRAND PRIX – CAIXABANK TROPHY CSI W 5 /CSI 2 round	10.12.2017	CSI-W5*	4	1.60	490	190	14	22.03.2004	M	AES	269	11	112	74	33	1410	55	M	11	8b	yes	vertical	vertical	straight	no	0	0	77,62	no
La Coruna	WORLD CUP GRAND PRIX – CAIXABANK TROPHY CSI W 5 /CSI 2 round	10.12.2017	CSI-W5*	4	1.60	490	190	14	22.03.2004	M	AES	269	11	112	74	33	1410	55	M	12	9	no	spread	oxer with water	left	no	0	0	77,62	no
La Coruna	WORLD CUP GRAND PRIX – CAIXABANK TROPHY CSI W 5 /CSI 2 round	10.12.2017	CSI-W5*	4	1.60	490	190	14	22.03.2004	M	AES	269	11	112	74	33	1410	55	M	13	10	no	vertical	vertical	right	no	0	0	77,62	no
La Coruna	WORLD CUP GRAND PRIX – CAIXABANK TROPHY CSI W 5 /CSI 2 round	10.12.2017	CSI-W5*	4	1.60	490	190	14	22.03.2004	M	AES	269	11	112	74	33	1410	55	M	14	11	no	spread	oxer	right	no	0	0	77,62	no
La Coruna	WORLD CUP GRAND PRIX – CAIXABANK TROPHY CSI W 5 /CSI 2 round	10.12.2017	CSI-W5*	4	1.60	490	190	14	22.03.2004	M	AES	269	11	112	74	33	1410	55	M	15	12	no	vertical	vertical	straight	no	0	0	77,62	no
La Coruna	WORLD CUP GRAND PRIX – CAIXABANK TROPHY CSI W 5 /CSI 2 round	10.12.2017	CSI-W5*	4	1.60	490	190	14	22.03.2004	M	AES	269	11	112	74	33	1410	55	M	16	13	no	spread	oxer	straight	no	0	0	77,62	no
La Coruna	WORLD CUP GRAND PRIX – CAIXABANK TROPHY CSI W 5 /CSI 2 round	10.12.2017	CSI-W5*	4	1.60	490	190	14	22.03.2004	M	AES	269	11	112	74	33	1410	55	M	17	14	no	vertical	vertical with water	left	no	0	0	77,62	no
La Coruna	WORLD CUP GRAND PRIX – CAIXABANK TROPHY CSI W 5 /CSI 2 round	10.12.2017	CSI-W5*	4	1.60	285	190	14	22.03.2004	M	AES	269	11	112	74	33	1410	55	M	1	1	no	vertical	vertical	left	no	0	0	39,97	no
La Coruna	WORLD CUP GRAND PRIX – CAIXABANK TROPHY CSI W 5 /CSI 2 round	10.12.2017	CSI-W5*	4	1.60	285	190	14	22.03.2004	M	AES	269	11	112	74	33	1410	55	M	2	2	no	spread	oxer	right	no	0	0	39,97	no
La Coruna	WORLD CUP GRAND PRIX – CAIXABANK TROPHY CSI W 5 /CSI 2 round	10.12.2017	CSI-W5*	4	1.60	285	190	14	22.03.2004	M	AES	269	11	112	74	33	1410	55	M	3	3	no	spread	oxer with water	left	no	0	0	39,97	no
La Coruna	WORLD CUP GRAND PRIX – CAIXABANK TROPHY CSI W 5 /CSI 2 round	10.12.2017	CSI-W5*	4	1.60	285	190	14	22.03.2004	M	AES	269	11	112	74	33	1410	55	M	4	4	no	vertical	vertical	right	no	0	0	39,97	no
La Coruna	WORLD CUP GRAND PRIX – CAIXABANK TROPHY CSI W 5 /CSI 2 round	10.12.2017	CSI-W5*	4	1.60	285	190	14	22.03.2004	M	AES	269	11	112	74	33	1410	55	M	5	5	no	spread	oxer	right	yes	4	0	39,97	no
La Coruna	WORLD CUP GRAND PRIX – CAIXABANK TROPHY CSI W 5 /CSI 2 round	10.12.2017	CSI-W5*	4	1.60	285	190	14	22.03.2004	M	AES	269	11	112	74	33	1410	55	M	6	6a	yes	vertical	vertical	right	no	0	0	39,97	no
La Coruna	WORLD CUP GRAND PRIX – CAIXABANK TROPHY CSI W 5 /CSI 2 round	10.12.2017	CSI-W5*	4	1.60	285	190	14	22.03.2004	M	AES	269	11	112	74	33	1410	55	M	7	6b	yes	spread	oxer	right	no	0	0	39,97	no
La Coruna	WORLD CUP GRAND PRIX – CAIXABANK TROPHY CSI W 5 /CSI 2 round	10.12.2017	CSI-W5*	4	1.60	285	190	14	22.03.2004	M	AES	269	11	112	74	33	1410	55	M	8	7	no	vertical	vertical	left	no	0	0	39,97	no
London	The Longines FEI World Cup Jumping - London Olympia round A	17.12.2017	CSI-W5*	4	1.60	490	145	12	17.03.2006	G	ISH	210	8	2	7	31	2168	92	M	1	1	no	spread	oxer	left	no	0	0	67,65	no
London	The Longines FEI World Cup Jumping - London Olympia round A	17.12.2017	CSI-W5*	4	1.60	490	145	12	17.03.2006	G	ISH	210	8	2	7	31	2168	92	M	2	2	no	spread	oxer	left	no	0	0	67,65	no
London	The Longines FEI World Cup Jumping - London Olympia round A	17.12.2017	CSI-W5*	4	1.60	490	145	12	17.03.2006	G	ISH	210	8	2	7	31	2168	92	M	3	3	no	vertical	vertical	left	no	0	0	67,65	no
London	The Longines FEI World Cup Jumping - London Olympia round A	17.12.2017	CSI-W5*	4	1.60	490	145	12	17.03.2006	G	ISH	210	8	2	7	31	2168	92	M	4	4a	yes	spread	oxer	right	no	0	0	67,65	no
London	The Longines FEI World Cup Jumping - London Olympia round A	17.12.2017	CSI-W5*	4	1.60	490	145	12	17.03.2006	G	ISH	210	8	2	7	31	2168	92	M	5	4b	yes	vertical	vertical	right	yes	4	0	67,65	no
London	The Longines FEI World Cup Jumping - London Olympia round A	17.12.2017	CSI-W5*	4	1.60	490	145	12	17.03.2006	G	ISH	210	8	2	7	31	2168	92	M	6	4c	yes	spread	oxer	right	no	0	0	67,65	no
London	The Longines FEI World Cup Jumping - London Olympia round A	17.12.2017	CSI-W5*	4	1.60	490	145	12	17.03.2006	G	ISH	210	8	2	7	31	2168	92	M	7	5	no	vertical	vertical	left	yes	4	0	67,65	no
London	The Longines FEI World Cup Jumping - London Olympia round A	17.12.2017	CSI-W5*	4	1.60	490	145	12	17.03.2006	G	ISH	210	8	2	7	31	2168	92	M	8	6	no	vertical	wall	left	no	0	0	67,65	no
London	The Longines FEI World Cup Jumping - London Olympia round A	17.12.2017	CSI-W5*	4	1.60	490	145	12	17.03.2006	G	ISH	210	8	2	7	31	2168	92	M	9	7	no	spread	oxer	right	yes	4	0	67,65	no
London	The Longines FEI World Cup Jumping - London Olympia round A	17.12.2017	CSI-W5*	4	1.60	490	145	12	17.03.2006	G	ISH	210	8	2	7	31	2168	92	M	10	8	no	vertical	vertical	right	no	0	0	67,65	no
London	The Longines FEI World Cup Jumping - London Olympia round A	17.12.2017	CSI-W5*	4	1.60	490	145	12	17.03.2006	G	ISH	210	8	2	7	31	2168	92	M	11	9	no	spread	triple bar	right	no	0	0	67,65	no
London	The Longines FEI World Cup Jumping - London Olympia round A	17.12.2017	CSI-W5*	4	1.60	490	145	12	17.03.2006	G	ISH	210	8	2	7	31	2168	92	M	12	10	no	vertical	vertical	straight	no	0	0	67,65	no
London	The Longines FEI World Cup Jumping - London Olympia round A	17.12.2017	CSI-W5*	4	1.60	490	145	12	17.03.2006	G	ISH	210	8	2	7	31	2168	92	M	13	11a	yes	vertical	vertical	left	no	0	0	67,65	no
London	The Longines FEI World Cup Jumping - London Olympia round A	17.12.2017	CSI-W5*	4	1.60	490	145	12	17.03.2006	G	ISH	210	8	2	7	31	2168	92	M	14	11b	yes	spread	oxer	left	no	0	0	67,65	no
London	The Longines FEI World Cup Jumping - London Olympia round A	17.12.2017	CSI-W5*	4	1.60	490	145	12	17.03.2006	G	ISH	210	8	2	7	31	2168	92	M	15	12	no	vertical	vertical with water	straight	no	0	0	67,65	no
London	The Longines FEI World Cup Jumping - London Olympia round A	17.12.2017	CSI-W5*	4	1.60	490	145	12	17.03.2006	G	ISH	210	8	2	7	31	2168	92	M	16	13	no	spread	oxer	left	no	0	0	67,65	no
London	The Longines FEI World Cup Jumping - London Olympia round A	17.12.2017	CSI-W5*	4	1.60	490	63	11	22.03.2007	G	KWPN	175	2	3	97	43	1901	45	M	1	1	no	spread	oxer	left	no	0	0	69,71	no
London	The Longines FEI World Cup Jumping - London Olympia round A	17.12.2017	CSI-W5*	4	1.60	490	63	11	22.03.2007	G	KWPN	175	2	3	97	43	1901	45	M	2	2	no	spread	oxer	left	no	0	0	69,71	no
London	The Longines FEI World Cup Jumping - London Olympia round A	17.12.2017	CSI-W5*	4	1.60	490	63	11	22.03.2007	G	KWPN	175	2	3	97	43	1901	45	M	3	3	no	vertical	vertical	left	no	0	0	69,71	no
London	The Longines FEI World Cup Jumping - London Olympia round A	17.12.2017	CSI-W5*	4	1.60	490	63	11	22.03.2007	G	KWPN	175	2	3	97	43	1901	45	M	4	4a	yes	spread	oxer	right	no	0	0	69,71	no
London	The Longines FEI World Cup Jumping - London Olympia round A	17.12.2017	CSI-W5*	4	1.60	490	63	11	22.03.2007	G	KWPN	175	2	3	97	43	1901	45	M	5	4b	yes	vertical	vertical	right	no	0	0	69,71	no
London	The Longines FEI World Cup Jumping - London Olympia round A	17.12.2017	CSI-W5*	4	1.60	490	63	11	22.03.2007	G	KWPN	175	2	3	97	43	1901	45	M	6	4c	yes	spread	oxer	right	no	0	0	69,71	no
London	The Longines FEI World Cup Jumping - London Olympia round A	17.12.2017	CSI-W5*	4	1.60	490	63	11	22.03.2007	G	KWPN	175	2	3	97	43	1901	45	M	7	5	no	vertical	vertical	left	no	0	0	69,71	no
London	The Longines FEI World Cup Jumping - London Olympia round A	17.12.2017	CSI-W5*	4	1.60	490	63	11	22.03.2007	G	KWPN	175	2	3	97	43	1901	45	M	8	6	no	vertical	wall	left	no	0	0	69,71	no
London	The Longines FEI World Cup Jumping - London Olympia round A	17.12.2017	CSI-W5*	4	1.60	490	63	11	22.03.2007	G	KWPN	175	2	3	97	43	1901	45	M	9	7	no	spread	oxer	right	no	0	0	69,71	no
London	The Longines FEI World Cup Jumping - London Olympia round A	17.12.2017	CSI-W5*	4	1.60	490	63	11	22.03.2007	G	KWPN	175	2	3	97	43	1901	45	M	10	8	no	vertical	vertical	right	yes	4	0	69,71	no
London	The Longines FEI World Cup Jumping - London Olympia round A	17.12.2017	CSI-W5*	4	1.60	490	63	11	22.03.2007	G	KWPN	175	2	3	97	43	1901	45	M	11	9	no	spread	triple bar	right	no	0	0	69,71	no
London	The Longines FEI World Cup Jumping - London Olympia round A	17.12.2017	CSI-W5*	4	1.60	490	63	11	22.03.2007	G	KWPN	175	2	3	97	43	1901	45	M	12	10	no	vertical	vertical	straight	no	0	0	69,71	no
London	The Longines FEI World Cup Jumping - London Olympia round A	17.12.2017	CSI-W5*	4	1.60	490	63	11	22.03.2007	G	KWPN	175	2	3	97	43	1901	45	M	13	11a	yes	vertical	vertical	left	no	0	0	69,71	no
London	The Longines FEI World Cup Jumping - London Olympia round A	17.12.2017	CSI-W5*	4	1.60	490	63	11	22.03.2007	G	KWPN	175	2	3	97	43	1901	45	M	14	11b	yes	spread	oxer	left	no	0	0	69,71	no
London	The Longines FEI World Cup Jumping - London Olympia round A	17.12.2017	CSI-W5*	4	1.60	490	63	11	22.03.2007	G	KWPN	175	2	3	97	43	1901	45	M	15	12	no	vertical	vertical with water	straight	no	0	0	69,71	no
London	The Longines FEI World Cup Jumping - London Olympia round A	17.12.2017	CSI-W5*	4	1.60	490	63	11	22.03.2007	G	KWPN	175	2	3	97	43	1901	45	M	16	13	no	spread	oxer	left	yes	4	0	69,71	no
London	The Longines FEI World Cup Jumping - London Olympia round A	17.12.2017	CSI-W5*	4	1.60	490	16	10	06.05.2008	G	AES	109	0	4	107	52	1652	70	M	1	1	no	spread	oxer	left	no	0	0	67,02	no
London	The Longines FEI World Cup Jumping - London Olympia round A	17.12.2017	CSI-W5*	4	1.60	490	16	10	06.05.2008	G	AES	109	0	4	107	52	1652	70	M	2	2	no	spread	oxer	left	no	0	0	67,02	no
London	The Longines FEI World Cup Jumping - London Olympia round A	17.12.2017	CSI-W5*	4	1.60	490	16	10	06.05.2008	G	AES	109	0	4	107	52	1652	70	M	3	3	no	vertical	vertical	left	no	0	0	67,02	no
London	The Longines FEI World Cup Jumping - London Olympia round A	17.12.2017	CSI-W5*	4	1.60	490	16	10	06.05.2008	G	AES	109	0	4	107	52	1652	70	M	4	4a	yes	spread	oxer	right	no	0	0	67,02	no
London	The Longines FEI World Cup Jumping - London Olympia round A	17.12.2017	CSI-W5*	4	1.60	490	16	10	06.05.2008	G	AES	109	0	4	107	52	1652	70	M	5	4b	yes	vertical	vertical	right	no	0	0	67,02	no
London	The Longines FEI World Cup Jumping - London Olympia round A	17.12.2017	CSI-W5*	4	1.60	490	16	10	06.05.2008	G	AES	109	0	4	107	52	1652	70	M	6	4c	yes	spread	oxer	right	no	0	0	67,02	no
London	The Longines FEI World Cup Jumping - London Olympia round A	17.12.2017	CSI-W5*	4	1.60	490	16	10	06.05.2008	G	AES	109	0	4	107	52	1652	70	M	7	5	no	vertical	vertical	left	no	0	0	67,02	no
London	The Longines FEI World Cup Jumping - London Olympia round A	17.12.2017	CSI-W5*	4	1.60	490	16	10	06.05.2008	G	AES	109	0	4	107	52	1652	70	M	8	6	no	vertical	wall	left	no	0	0	67,02	no
London	The Longines FEI World Cup Jumping - London Olympia round A	17.12.2017	CSI-W5*	4	1.60	490	16	10	06.05.2008	G	AES	109	0	4	107	52	1652	70	M	9	7	no	spread	oxer	right	no	0	0	67,02	no
London	The Longines FEI World Cup Jumping - London Olympia round A	17.12.2017	CSI-W5*	4	1.60	490	16	10	06.05.2008	G	AES	109	0	4	107	52	1652	70	M	10	8	no	vertical	vertical	right	yes	4	0	67,02	no
London	The Longines FEI World Cup Jumping - London Olympia round A	17.12.2017	CSI-W5*	4	1.60	490	16	10	06.05.2008	G	AES	109	0	4	107	52	1652	70	M	11	9	no	spread	triple bar	right	no	0	0	67,02	no
London	The Longines FEI World Cup Jumping - London Olympia round A	17.12.2017	CSI-W5*	4	1.60	490	16	10	06.05.2008	G	AES	109	0	4	107	52	1652	70	M	12	10	no	vertical	vertical	straight	no	0	0	67,02	no
London	The Longines FEI World Cup Jumping - London Olympia round A	17.12.2017	CSI-W5*	4	1.60	490	16	10	06.05.2008	G	AES	109	0	4	107	52	1652	70	M	13	11a	yes	vertical	vertical	left	yes	4	0	67,02	no
London	The Longines FEI World Cup Jumping - London Olympia round A	17.12.2017	CSI-W5*	4	1.60	490	16	10	06.05.2008	G	AES	109	0	4	107	52	1652	70	M	14	11b	yes	spread	oxer	left	no	0	0	67,02	no
London	The Longines FEI World Cup Jumping - London Olympia round A	17.12.2017	CSI-W5*	4	1.60	490	16	10	06.05.2008	G	AES	109	0	4	107	52	1652	70	M	15	12	no	vertical	vertical with water	straight	yes	4	0	67,02	no
London	The Longines FEI World Cup Jumping - London Olympia round A	17.12.2017	CSI-W5*	4	1.60	490	16	10	06.05.2008	G	AES	109	0	4	107	52	1652	70	M	16	13	no	spread	oxer	left	no	0	0	67,02	no
London	The Longines FEI World Cup Jumping - London Olympia round A	17.12.2017	CSI-W5*	4	1.60	490	20	11	16.04.2007	G	OS	121	0	7	48	58	1857	49	M	1	1	no	spread	oxer	left	no	0	0	82,69	no
London	The Longines FEI World Cup Jumping - London Olympia round A	17.12.2017	CSI-W5*	4	1.60	490	20	11	16.04.2007	G	OS	121	0	7	48	58	1857	49	M	2	2	no	spread	oxer	left	no	0	0	82,69	no
London	The Longines FEI World Cup Jumping - London Olympia round A	17.12.2017	CSI-W5*	4	1.60	490	20	11	16.04.2007	G	OS	121	0	7	48	58	1857	49	M	3	3	no	vertical	vertical	left	no	0	0	82,69	no
London	The Longines FEI World Cup Jumping - London Olympia round A	17.12.2017	CSI-W5*	4	1.60	490	20	11	16.04.2007	G	OS	121	0	7	48	58	1857	49	M	4	4a	yes	spread	oxer	right	no	0	0	82,69	no
London	The Longines FEI World Cup Jumping - London Olympia round A	17.12.2017	CSI-W5*	4	1.60	490	20	11	16.04.2007	G	OS	121	0	7	48	58	1857	49	M	5	4b	yes	vertical	vertical	right	no	0	0	82,69	no
London	The Longines FEI World Cup Jumping - London Olympia round A	17.12.2017	CSI-W5*	4	1.60	490	20	11	16.04.2007	G	OS	121	0	7	48	58	1857	49	M	6	4c	yes	spread	oxer	right	no	0	0	82,69	no
London	The Longines FEI World Cup Jumping - London Olympia round A	17.12.2017	CSI-W5*	4	1.60	490	20	11	16.04.2007	G	OS	121	0	7	48	58	1857	49	M	7	5	no	vertical	vertical	left	yes	4	0	82,69	no
London	The Longines FEI World Cup Jumping - London Olympia round A	17.12.2017	CSI-W5*	4	1.60	490	20	11	16.04.2007	G	OS	121	0	7	48	58	1857	49	M	8	6	no	vertical	wall	left	no	0	0	82,69	no
London	The Longines FEI World Cup Jumping - London Olympia round A	17.12.2017	CSI-W5*	4	1.60	490	20	11	16.04.2007	G	OS	121	0	7	48	58	1857	49	M	9	7	no	spread	oxer	right	no	0	0	82,69	no
London	The Longines FEI World Cup Jumping - London Olympia round A	17.12.2017	CSI-W5*	4	1.60	490	20	11	16.04.2007	G	OS	121	0	7	48	58	1857	49	M	10	8	no	vertical	vertical	right	no	0	0	82,69	no
London	The Longines FEI World Cup Jumping - London Olympia round A	17.12.2017	CSI-W5*	4	1.60	490	20	11	16.04.2007	G	OS	121	0	7	48	58	1857	49	M	11	9	no	spread	triple bar	right	no	0	0	82,69	no
London	The Longines FEI World Cup Jumping - London Olympia round A	17.12.2017	CSI-W5*	4	1.60	490	20	11	16.04.2007	G	OS	121	0	7	48	58	1857	49	M	12	10	no	vertical	vertical	straight	no	0	0	82,69	no
London	The Longines FEI World Cup Jumping - London Olympia round A	17.12.2017	CSI-W5*	4	1.60	490	20	11	16.04.2007	G	OS	121	0	7	48	58	1857	49	M	13	11a	yes	vertical	vertical	left	yes	4	0	82,69	no
London	The Longines FEI World Cup Jumping - London Olympia round A	17.12.2017	CSI-W5*	4	1.60	490	20	11	16.04.2007	G	OS	121	0	7	48	58	1857	49	M	14	11b	yes	spread	oxer	left	no	0	0	82,69	no
London	The Longines FEI World Cup Jumping - London Olympia round A	17.12.2017	CSI-W5*	4	1.60	490	20	11	16.04.2007	G	OS	121	0	7	48	58	1857	49	M	15	12	no	vertical	vertical with water	straight	no	0	0	82,69	no
London	The Longines FEI World Cup Jumping - London Olympia round A	17.12.2017	CSI-W5*	4	1.60	490	20	11	16.04.2007	G	OS	121	0	7	48	58	1857	49	M	16	13	no	spread	oxer	left	no	0	4	82,69	no

London	The Longines FEI World Cup Jumping - London Olympia round A	17.12.2017	CSI-W5*	4	1.60	490	182	12	23.05.2006	G	SF	72	4	12	235	58	1047	25	M	1	1	no	spread	oxer	left	no	0	0	70,16	no
London	The Longines FEI World Cup Jumping - London Olympia round A	17.12.2017	CSI-W5*	4	1.60	490	182	12	23.05.2006	G	SF	72	4	12	235	58	1047	25	M	2	2	no	spread	oxer	left	no	0	0	70,16	no
London	The Longines FEI World Cup Jumping - London Olympia round A	17.12.2017	CSI-W5*	4	1.60	490	182	12	23.05.2006	G	SF	72	4	12	235	58	1047	25	M	3	3	no	vertical	vertical	left	no	0	0	70,16	no
London	The Longines FEI World Cup Jumping - London Olympia round A	17.12.2017	CSI-W5*	4	1.60	490	182	12	23.05.2006	G	SF	72	4	12	235	58	1047	25	M	4	4a	yes	spread	oxer	right	no	0	0	70,16	no
London	The Longines FEI World Cup Jumping - London Olympia round A	17.12.2017	CSI-W5*	4	1.60	490	182	12	23.05.2006	G	SF	72	4	12	235	58	1047	25	M	5	4b	yes	vertical	vertical	right	no	0	0	70,16	no
London	The Longines FEI World Cup Jumping - London Olympia round A	17.12.2017	CSI-W5*	4	1.60	490	182	12	23.05.2006	G	SF	72	4	12	235	58	1047	25	M	6	4c	yes	spread	oxer	right	no	0	0	70,16	no
London	The Longines FEI World Cup Jumping - London Olympia round A	17.12.2017	CSI-W5*	4	1.60	490	182	12	23.05.2006	G	SF	72	4	12	235	58	1047	25	M	7	5	no	vertical	vertical	left	no	0	0	70,16	no
London	The Longines FEI World Cup Jumping - London Olympia round A	17.12.2017	CSI-W5*	4	1.60	490	182	12	23.05.2006	G	SF	72	4	12	235	58	1047	25	M	8	6	no	vertical	wall	left	no	0	0	70,16	no
London	The Longines FEI World Cup Jumping - London Olympia round A	17.12.2017	CSI-W5*	4	1.60	490	182	12	23.05.2006	G	SF	72	4	12	235	58	1047	25	M	9	7	no	spread	oxer	right	no	0	0	70,16	no
London	The Longines FEI World Cup Jumping - London Olympia round A	17.12.2017	CSI-W5*	4	1.60	490	182	12	23.05.2006	G	SF	72	4	12	235	58	1047	25	M	10	8	no	vertical	vertical	right	yes	4	0	70,16	no
London	The Longines FEI World Cup Jumping - London Olympia round A	17.12.2017	CSI-W5*	4	1.60	490	182	12	23.05.2006	G	SF	72	4	12	235	58	1047	25	M	11	9	no	spread	triple bar	right	no	0	0	70,16	no
London	The Longines FEI World Cup Jumping - London Olympia round A	17.12.2017	CSI-W5*	4	1.60	490	182	12	23.05.2006	G	SF	72	4	12	235	58	1047	25	M	12	10	no	vertical	vertical	straight	no	0	0	70,16	no
London	The Longines FEI World Cup Jumping - London Olympia round A	17.12.2017	CSI-W5*	4	1.60	490	182	12	23.05.2006	G	SF	72	4	12	235	58	1047	25	M	13	11a	yes	vertical	vertical	left	yes	4	0	70,16	no
London	The Longines FEI World Cup Jumping - London Olympia round A	17.12.2017	CSI-W5*	4	1.60	490	182	12	23.05.2006	G	SF	72	4	12	235	58	1047	25	M	14	11b	yes	spread	oxer	left	no	0	0	70,16	no
London	The Longines FEI World Cup Jumping - London Olympia round A	17.12.2017	CSI-W5*	4	1.60	490	182	12	23.05.2006	G	SF	72	4	12	235	58	1047	25	M	15	12	no	vertical	vertical with water	straight	yes	4	0	70,16	no
London	The Longines FEI World Cup Jumping - London Olympia round A	17.12.2017	CSI-W5*	4	1.60	490	182	12	23.05.2006	G	SF	72	4	12	235	58	1047	25	M	16	13	no	spread	oxer	left	no	0	1	70,16	no
London	The Longines FEI World Cup Jumping - London Olympia round A	17.12.2017	CSI-W5*	4	1.60	490	196	13	15.05.2005	M	ZANG	166	4	14	5	37	1868	96	M	1	1	no	spread	oxer	left	no	0	0	67,73	no
London	The Longines FEI World Cup Jumping - London Olympia round A	17.12.2017	CSI-W5*	4	1.60	490	196	13	15.05.2005	M	ZANG	166	4	14	5	37	1868	96	M	2	2	no	spread	oxer	left	no	0	0	67,73	no
London	The Longines FEI World Cup Jumping - London Olympia round A	17.12.2017	CSI-W5*	4	1.60	490	196	13	15.05.2005	M	ZANG	166	4	14	5	37	1868	96	M	3	3	no	vertical	vertical	left	no	0	0	67,73	no
London	The Longines FEI World Cup Jumping - London Olympia round A	17.12.2017	CSI-W5*	4	1.60	490	196	13	15.05.2005	M	ZANG	166	4	14	5	37	1868	96	M	4	4a	yes	spread	oxer	right	no	0	0	67,73	no
London	The Longines FEI World Cup Jumping - London Olympia round A	17.12.2017	CSI-W5*	4	1.60	490	196	13	15.05.2005	M	ZANG	166	4	14	5	37	1868	96	M	5	4b	yes	vertical	vertical	right	no	0	0	67,73	no
London	The Longines FEI World Cup Jumping - London Olympia round A	17.12.2017	CSI-W5*	4	1.60	490	196	13	15.05.2005	M	ZANG	166	4	14	5	37	1868	96	M	6	4c	yes	spread	oxer	right	no	0	0	67,73	no
London	The Longines FEI World Cup Jumping - London Olympia round A	17.12.2017	CSI-W5*	4	1.60	490	196	13	15.05.2005	M	ZANG	166	4	14	5	37	1868	96	M	7	5	no	vertical	vertical	left	no	0	0	67,73	no
London	The Longines FEI World Cup Jumping - London Olympia round A	17.12.2017	CSI-W5*	4	1.60	490	196	13	15.05.2005	M	ZANG	166	4	14	5	37	1868	96	M	8	6	no	vertical	wall	left	no	0	0	67,73	no
London	The Longines FEI World Cup Jumping - London Olympia round A	17.12.2017	CSI-W5*	4	1.60	490	196	13	15.05.2005	M	ZANG	166	4	14	5	37	1868	96	M	9	7	no	spread	oxer	right	no	0	0	67,73	no
London	The Longines FEI World Cup Jumping - London Olympia round A	17.12.2017	CSI-W5*	4	1.60	490	196	13	15.05.2005	M	ZANG	166	4	14	5	37	1868	96	M	10	8	no	vertical	vertical	right	no	0	0	67,73	no
London	The Longines FEI World Cup Jumping - London Olympia round A	17.12.2017	CSI-W5*	4	1.60	490	196	13	15.05.2005	M	ZANG	166	4	14	5	37	1868	96	M	11	9	no	spread	triple bar	right	no	0	0	67,73	no
London	The Longines FEI World Cup Jumping - London Olympia round A	17.12.2017	CSI-W5*	4	1.60	490	196	13	15.05.2005	M	ZANG	166	4	14	5	37	1868	96	M	12	10	no	vertical	vertical	straight	no	0	0	67,73	no
London	The Longines FEI World Cup Jumping - London Olympia round A	17.12.2017	CSI-W5*	4	1.60	490	196	13	15.05.2005	M	ZANG	166	4	14	5	37	1868	96	M	13	11a	yes	vertical	vertical	left	no	0	0	67,73	no
London	The Longines FEI World Cup Jumping - London Olympia round A	17.12.2017	CSI-W5*	4	1.60	490	196	13	15.05.2005	M	ZANG	166	4	14	5	37	1868	96	M	14	11b	yes	spread	oxer	left	no	0	0	67,73	no
London	The Longines FEI World Cup Jumping - London Olympia round A	17.12.2017	CSI-W5*	4	1.60	490	196	13	15.05.2005	M	ZANG	166	4	14	5	37	1868	96	M	15	12	no	vertical	vertical with water	straight	no	0	0	67,73	no
London	The Longines FEI World Cup Jumping - London Olympia round A	17.12.2017	CSI-W5*	4	1.60	490	196	13	15.05.2005	M	ZANG	166	4	14	5	37	1868	96	M	16	13	no	spread	oxer	left	yes	4	0	67,73	no
London	The Longines FEI World Cup Jumping - London Olympia round A	17.12.2017	CSI-W5*	4	1.60	490	56	14	12.03.2004	G	OLDBG	203	7	16	17	37	2215	106	M	1	1	no	spread	oxer	left	no	0	0	68,1	no
London	The Longines FEI World Cup Jumping - London Olympia round A	17.12.2017	CSI-W5*	4	1.60	490	56	14	12.03.2004	G	OLDBG	203	7	16	17	37	2215	106	M	2	2	no	spread	oxer	left	no	0	0	68,1	no
London	The Longines FEI World Cup Jumping - London Olympia round A	17.12.2017	CSI-W5*	4	1.60	490	56	14	12.03.2004	G	OLDBG	203	7	16	17	37	2215	106	M	3	3	no	vertical	vertical	left	no	0	0	68,1	no
London	The Longines FEI World Cup Jumping - London Olympia round A	17.12.2017	CSI-W5*	4	1.60	490	56	14	12.03.2004	G	OLDBG	203	7	16	17	37	2215	106	M	4	4a	yes	spread	oxer	right	no	0	0	68,1	no
London	The Longines FEI World Cup Jumping - London Olympia round A	17.12.2017	CSI-W5*	4	1.60	490	56	14	12.03.2004	G	OLDBG	203	7	16	17	37	2215	106	M	5	4b	yes	vertical	vertical	right	no	0	0	68,1	no
London	The Longines FEI World Cup Jumping - London Olympia round A	17.12.2017	CSI-W5*	4	1.60	490	56	14	12.03.2004	G	OLDBG	203	7	16	17	37	2215	106	M	6	4c	yes	spread	oxer	right	no	0	0	68,1	no
London	The Longines FEI World Cup Jumping - London Olympia round A	17.12.2017	CSI-W5*	4	1.60	490	56	14	12.03.2004	G	OLDBG	203	7	16	17	37	2215	106	M	7	5	no	vertical	vertical	left	no	0	0	68,1	no
London	The Longines FEI World Cup Jumping - London Olympia round A	17.12.2017	CSI-W5*	4	1.60	490	56	14	12.03.2004	G	OLDBG	203	7	16	17	37	2215	106	M	8	6	no	vertical	wall	left	no	0	0	68,1	no
London	The Longines FEI World Cup Jumping - London Olympia round A	17.12.2017	CSI-W5*	4	1.60	490	56	14	12.03.2004	G	OLDBG	203	7	16	17	37	2215	106	M	9	7	no	spread	oxer	right	no	0	0	68,1	no
London	The Longines FEI World Cup Jumping - London Olympia round A	17.12.2017	CSI-W5*	4	1.60	490	56	14	12.03.2004	G	OLDBG	203	7	16	17	37	2215	106	M	10	8	no	vertical	vertical	right	no	0	0	68,1	no
London	The Longines FEI World Cup Jumping - London Olympia round A	17.12.2017	CSI-W5*	4	1.60	490	56	14	12.03.2004	G	OLDBG	203	7	16	17	37	2215	106	M	11	9	no	spread	triple bar	right	no	0	0	68,1	no
London	The Longines FEI World Cup Jumping - London Olympia round A	17.12.2017	CSI-W5*	4	1.60	490	56	14	12.03.2004	G	OLDBG	203	7	16	17	37	2215	106	M	12	10	no	vertical	vertical	straight	no	0	0	68,1	no
London	The Longines FEI World Cup Jumping - London Olympia round A	17.12.2017	CSI-W5*	4	1.60	490	56	14	12.03.2004	G	OLDBG	203	7	16	17	37	2215	106	M	13	11a	yes	vertical	vertical	left	yes	4	0	68,1	no
London	The Longines FEI World Cup Jumping - London Olympia round A	17.12.2017	CSI-W5*	4	1.60	490	56	14	12.03.2004	G	OLDBG	203	7	16	17	37	2215	106	M	14	11b	yes	spread	oxer	left	no	0	0	68,1	no
London	The Longines FEI World Cup Jumping - London Olympia round A	17.12.2017	CSI-W5*	4	1.60	490	56	14	12.03.2004	G	OLDBG	203	7	16	17	37	2215	106	M	15	12	no	vertical	vertical with water	straight	no	0	0	68,1	no
London	The Longines FEI World Cup Jumping - London Olympia round A	17.12.2017	CSI-W5*	4	1.60	490	56	14	12.03.2004	G	OLDBG	203	7	16	17	37	2215	106	M	16	13	no	spread	oxer	left	no	0	0	68,1	no
London	The Longines FEI World Cup Jumping - London Olympia round A	17.12.2017	CSI-W5*	4	1.60	490	104	11	23.03.2007	G	KWPN	95	0	17	3	46	1005	51	M	1	1	no	spread	oxer	left	no	0	0	68,29	no
London	The Longines FEI World Cup Jumping - London Olympia round A	17.12.2017	CSI-W5*	4	1.60	490	104	11	23.03.2007	G	KWPN	95	0	17	3	46	1005	51	M	2	2	no	spread	oxer	left	no	0	0	68,29	no
London	The Longines FEI World Cup Jumping - London Olympia round A	17.12.2017	CSI-W5*	4	1.60	490	104	11	23.03.2007	G	KWPN	95	0	17	3	46	1005	51	M	3	3	no	vertical	vertical	left	no	0	0	68,29	no
London	The Longines FEI World Cup Jumping - London Olympia round A	17.12.2017	CSI-W5*	4	1.60	490	104	11	23.03.2007	G	KWPN	95	0	17	3	46	1005	51	M	4	4a	yes	spread	oxer	right	no	0	0	68,29	no
London	The Longines FEI World Cup Jumping - London Olympia round A	17.12.2017	CSI-W5*	4	1.60	490	104	11	23.03.2007	G	KWPN	95	0	17	3	46	1005	51	M	5	4b	yes	vertical	vertical	right	no	0	0	68,29	no
London	The Longines FEI World Cup Jumping - London Olympia round A	17.12.2017	CSI-W5*	4	1.60	490	104	11	23.03.2007	G	KWPN	95	0	17	3	46	1005	51	M	6	4c	yes	spread	oxer	right	no	0	0	68,29	no
London	The Longines FEI World Cup Jumping - London Olympia round A	17.12.2017	CSI-W5*	4	1.60	490	104	11	23.03.2007	G	KWPN	95	0	17	3	46	1005	51	M	7	5	no	vertical	vertical	left	no	0	0	68,29	no
London	The Longines FEI World Cup Jumping - London Olympia round A	17.12.2017	CSI-W5*	4	1.60	490	104	11	23.03.2007	G	KWPN	95	0	17	3	46	1005	51	M	8	6	no	vertical	wall	left	no	0	0	68,29	no
London	The Longines FEI World Cup Jumping - London Olympia round A	17.12.2017	CSI-W5*	4	1.60	490	104	11	23.03.2007	G	KWPN	95	0	17	3	46	1005	51	M	9	7	no	spread	oxer	right	no	0	0	68,29	no
London	The Longines FEI World Cup Jumping - London Olympia round A	17.12.2017	CSI-W5*	4	1.60	490	104	11	23.03.2007	G	KWPN	95	0	17	3	46	1005	51	M	10	8	no	vertical	vertical	right	no	0	0	68,29	no
London	The Longines FEI World Cup Jumping - London Olympia round A	17.12.2017	CSI-W5*	4	1.60	490	104	11	23.03.2007	G	KWPN	95	0	17	3	46	1005	51	M	11	9	no	spread	triple bar	right	no	0	0	68,29	no
London	The Longines FEI World Cup Jumping - London Olympia round A	17.12.2017	CSI-W5*	4	1.60	490	104	11	23.03.2007	G	KWPN	95	0	17	3	46	1005	51	M	12	10	no	vertical	vertical	straight	no	0	0	68,29	no
London	The Longines FEI World Cup Jumping - London Olympia round A	17.12.2017	CSI-W5*	4	1.60	490	104	11	23.03.2007	G	KWPN	95	0	17	3	46	1005	51	M	13	11a	yes	vertical	vertical	left	no	0	0	68,29	no
London	The Longines FEI World Cup Jumping - London Olympia round A	17.12.2017	CSI-W5*	4	1.60	490	104	11	23.03.2007	G	KWPN	95	0	17	3	46	1005	51	M	14	11b	yes	spread	oxer	left	no	0	0	68,29	no
London	The Longines FEI World Cup Jumping - London Olympia round A	17.12.2017	CSI-W5*	4	1.60	490	104	11	23.03.2007	G	KWPN	95	0	17	3	46	1005	51	M	15	12	no	vertical	vertical with water	straight	no	0	0	68,29	no
London	The Longines FEI World Cup Jumping - London Olympia round A	17.12.2017	CSI-W5*	4	1.60	490	104	11	23.03.2007	G	KWPN	95	0	17	3	46	1005	51	M	16	13	no	spread	oxer	left	no	0	0	68,29	no
London	The Longines FEI World Cup Jumping - London Olympia round B	17.12.2017	CSI-W5*	4	1.60	280	104	11	23.03.2007	G	KWPN	95	0	17	3	46	1005	51	M	1	1	no	spread	oxer	left	no	0	0	41,34	no
London	The Longines FEI World Cup Jumping - London Olympia round B	17.12.2017	CSI-W5*	4	1.60	280	104	11	23.03.2007	G	KWPN	95	0	17	3	46	1005	51	M	2	2	no	spread	oxer	left	no	0	0	41,34	no
London	The Longines FEI World Cup Jumping - London Olympia round B	17.12.2017	CSI-W5*	4	1.60	280	104	11	23.03.2007	G	KWPN	95	0	17	3	46	1005	51	M	3	3	no	vertical	vertical	left	no	0	0	41,34	no
London	The Longines FEI World Cup Jumping - London Olympia round B	17.12.2017	CSI-W5*	4	1.60	280	104	11	23.03.2007	G	KWPN	95	0	17	3	46	1005	51	M	4	4a	yes	vertical	vertical	right	no	0	0	41,34	no
London	The Longines FEI World Cup Jumping - London Olympia round B	17.12.2017	CSI-W5*	4	1.60	280	104	11	23.03.2007	G	KWPN	95	0	17	3	46	1005	51	M	5	4b	yes	spread	oxer	right	no	0	0	41,34	no
London	The Longines FEI World Cup Jumping - London Olympia round B	17.12.2017	CSI-W5*	4	1.60	280	104	11	23.03.2007	G	KWPN	95	0	17	3	46	1005	51	M	6	5	no	vertical	wall	left	no	0	0	41,34	no
London	The Longines FEI World Cup Jumping - London Olympia round B	17.12.2017	CSI-W5*	4	1.60	280	104	11	23.03.2007	G	KWPN	95	0	17	3	46	1005	51	M	7	6	no	vertical	vertical	right	no	0	0	41,34	no
London	The Longines FEI World Cup Jumping - London Olympia round B	17.12.2017	CSI-W5*	4	1.60	280	104	11	23.03.2007	G	KWPN	95	0	17	3	46	1005	51	M	8	7	no	spread	oxer	right	no	0	0	41,34	no
London	The Longines FEI World Cup Jumping - London Olympia round A	17.12.2017	CSI-W5*	4	1.60	490	9	13	13.04.2005	M	KWPN	216	6	26	14	30	1957	97	M	1	1	no	spread	oxer	left	no	0	0	67,48	no
London	The Longines FEI World Cup Jumping - London Olympia round A	17.12.2017	CSI-W5*	4	1.60	490	9	13	13.04.2005	M	KWPN	216	6	26	14	30	1957	97	M	2	2	no	spread	oxer	left	no	0	0	67,48	no
London	The Longines FEI World Cup Jumping - London Olympia round A	17.12.2017	CSI-W5*	4	1.60	490	9	13	13.04.2005	M	KWPN	216	6	26	14	30	1957	97	M	3	3	no	vertical	vertical	left	yes	4	0	67,48	no
London	The Longines FEI World Cup Jumping - London Olympia round A	17.12.2017	CSI-W5*	4	1.60	490	9	13	13.04.2005	M	KWPN	216	6	26	14	30	1957	97	M	4	4a	yes	spread	oxer	right	no	0	0	67,48	no
London	The Longines FEI World Cup Jumping - London Olympia round A	17.12.2017	CSI-W5*	4	1.60	490	9	13	13.04.2005	M	KWPN	216	6	26	14	30	1957	97	M	5	4b	yes	vertical	vertical	right	no	0	0	67,48	no
London	The Longines FEI World Cup Jumping - London Olympia round A	17.12.2017	CSI-W5*	4	1.60	490	9	13	13.04.2005	M	KWPN	216	6	26	14	30	1957	97	M	6	4c	yes	spread	oxer	right	no	0	0	67,48	no
London	The Longines FEI World Cup Jumping - London Olympia round A	17.12.2017	CSI-W5*	4	1.60	490	9	13	13.04.2005	M	KWPN	216	6	26	14	30	1957	97	M	7	5	no	vertical	vertical	left	no	0	0	67,48	no
London	The Longines FEI World Cup Jumping - London Olympia round A	17.12.2017	CSI-W5*	4	1.60	490	9	13	13.04.2005	M	KWPN	216	6	26	14	30	1957	97	M	8	6	no	vertical	wall	left	no	0	0	67,48	no
London	The Longines FEI World Cup Jumping - London Olympia round A	17.12.2017	CSI-W5*	4	1.60	490	9	13	13.04.2005	M	KWPN	216	6	26	14	30	1957	97	M	9	7	no	spread	oxer	right	no	0	0	67,48	no
London	The Longines FEI World Cup Jumping - London Olympia round A	17.12.2017	CSI-W5*	4	1.60	490	9	13	13.04.2005	M	KWPN	216	6	26	14	30	1957	97	M	10	8	no	vertical	vertical	right	no	0	0	67,48	no
London	The Longines FEI World Cup Jumping - London Olympia round A	17.12.2017	CSI-W5*	4	1.60	490	9	13	13.04.2005	M	KWPN	216	6	26	14	30	1957	97	M	11	9	no	spread	triple bar	right	no	0	0	67,48	no
London	The Longines FEI World Cup Jumping - London Olympia round A	17.12.2017	CSI-W5*	4	1.60	490	9	13	13.04.2005	M	KWPN	216	6	26	14	30	1957	97	M	12	10	no	vertical	vertical	straight	no	0	0	67,48	no
London	The Longines FEI World Cup Jumping - London Olympia round A	17.12.2017	CSI-W5*	4	1.60	490	9	13	13.04.2005	M	KWPN	216	6	26	14	30	1957	97	M	13	11a	yes	vertical	vertical	left	yes	4	0	67,48	no
London	The Longines FEI World Cup Jumping - London Olympia round A	17.12.2017	CSI-W5*	4	1.60	490	9	13	13.04.2005	M	KWPN	216	6	26	14	30	1957	97	M	14	11b	yes	spread	oxer	left	no	0	0	67,48	no
London	The Longines FEI World Cup Jumping - London Olympia round A	17.12.2017	CSI-W5*	4	1.60	490	9	13	13.04.2005	M	KWPN	216	6	26	14	30	1957	97	M	15	12	no	vertical	vertical with water	straight	no	0	0	67,48	no
London	The Longines FEI World Cup Jumping - London Olympia round A	17.12.2017	CSI-W5*	4	1.60	490	9	13	13.04.2005	M	KWPN	216	6	26	14	30	1957	97	M	16	13	no	spread	oxer	left	no	0	0	67,48	no
London	The Longines FEI World Cup Jumping - London Olympia round A	17.12.2017	CSI-W5*	4	1.60	490	184	12	18.06.2006	S	SF	154	4	28	4	38	2170	90	M	1	1	no	spread	oxer	left	no	0	0	64,53	no
London	The Longines FEI World Cup Jumping - London Olympia round A	17.12.2017	CSI-W5*	4	1.60	490	184	12	18.06.2006	S	SF	154	4	28	4	38	2170	90	M	2	2	no	spread	oxer	left	no	0	0	64,53	no
London	The Longines FEI World Cup Jumping - London Olympia round A	17.12.2017	CSI-W5*	4	1.60	490	184	12	18.06.2006	S	SF	154	4	28	4	38	2170	90	M	3	3	no	vertical	vertical	left	no	0	0	64,53	no
London	The Longines FEI World Cup Jumping - London Olympia round A	17.12.2017	CSI-W5*	4	1.60	490	184	12	18.06.2006	S	SF	154	4	28	4	38	2170	90	M	4	4a	yes	spread	oxer	right	yes	4	0	64,53	no
London	The Longines FEI World Cup Jumping - London Olympia round A	17.12.2017	CSI-W5*	4	1.60	490	184	12	18.06.2006	S	SF	154	4	28	4	38	2170	90	M	5	4b	yes	vertical	vertical	right	no	0	0	64,53	no
London	The Longines FEI World Cup Jumping - London Olympia round A	17.12.2017	CSI-W5*	4	1.60	490	184	12	18.06.2006	S	SF	154	4	28	4	38	2170	90	M	6	4c	yes	spread	oxer	right	no	0	0	64,53	no
London	The Longines FEI World Cup Jumping - London Olympia round A	17.12.2017	CSI-W5*	4	1.60	490	184	12	18.06.2006	S	SF	154	4	28	4	38	2170	90	M	7	5	no	vertical	vertical	left	no	0	0	64,53	no
London	The Longines FEI World Cup Jumping - London Olympia round A	17.12.2017	CSI-W5*	4	1.60	490	184	12	18.06.2006	S	SF	154	4	28	4	38	2170	90	M	8	6	no	vertical	wall	left	no	0	0	64,53	no
London	The Longines FEI World Cup Jumping - London Olympia round A	17.12.2017	CSI-W5*	4	1.60	490	184	12	18.06.2006	S	SF	154	4	28	4	38	2170	90	M	9	7	no	spread	oxer	right	no	0	0	64,53	no
London	The Longines FEI World Cup Jumping - London Olympia round A	17.12.2017	CSI-W5*	4	1.60	490	184	12	18.06.2006	S	SF	154	4	28	4	38	2170	90	M	10	8	no	vertical	vertical	right	no	0	0	64,53	no
London	The Longines FEI World Cup Jumping - London Olympia round A	17.12.2017	CSI-W5*	4	1.60	490	184	12	18.06.2006	S	SF	154	4	28	4	38	2170	90	M	11	9	no	spread	triple bar	right	no	0	0	64,53	no
London	The Longines FEI World Cup Jumping - London Olympia round A	17.12.2017	CSI-W5*	4	1.60	490	184	12	18.06.2006	S	SF	154	4	28	4	38	2170	90	M	12	10	no	vertical	vertical	straight	no	0	0	64,53	no
London	The Longines FEI World Cup Jumping - London Olympia round A	17.12.2017	CSI-W5*	4	1.60	490	184	12	18.06.2006	S	SF	154	4	28	4	38	2170	90	M	13	11a	yes	vertical	vertical	left	no	0	0	64,53	no
London	The Longines FEI World Cup Jumping - London Olympia round A	17.12.2017	CSI-W5*	4	1.60	490	184	12	18.06.2006	S	SF	154	4	28	4	38	2170	90	M	14	11b	yes	spread	oxer	left	no	0	0	64,53	no
London	The Longines FEI World Cup Jumping - London Olympia round A	17.12.2017	CSI-W5*	4	1.60	490	184	12	18.06.2006	S	SF	154	4	28	4	38	2170	90	M	15	12	no	vertical	vertical with water	straight	no	0	0	64,53	no
London	The Longines FEI World Cup Jumping - London Olympia round A	17.12.2017	CSI-W5*	4	1.60	490	184	12	18.06.2006	S	SF	154	4	28	4	38	2170	90	M	16	13	no	spread	oxer	left	no	0	0	64,53	no
London	The Longines FEI World Cup Jumping - London Olympia round A	17.12.2017	CSI-W5*	4	1.60	490	3	10	01.01.2008	G	KWPN	69	1	30	9	36	2556	124	M	1	1	no	spread	oxer	left	no	0	0	78,48	no
London	The Longines FEI World Cup Jumping - London Olympia round A	17.12.2017	CSI-W5*	4	1.60	490	3	10	01.01.2008	G	KWPN	69	1	30	9	36	2556	124	M	2	2	no	spread	oxer	left	no	0	0	78,48	no
London	The Longines FEI World Cup Jumping - London Olympia round A	17.12.2017	CSI-W5*	4	1.60	490	3	10	01.01.2008	G	KWPN	69	1	30	9	36	2556	124	M	3	3	no	vertical	vertical	left	no	0	0	78,48	no
London	The Longines FEI World Cup Jumping - London Olympia round A	17.12.2017	CSI-W5*	4	1.60	490	3	10	01.01.2008	G	KWPN	69	1	30	9	36	2556	124	M	4	4a	yes	spread	oxer	right	no	0	0	78,48	no
London	The Longines FEI World Cup Jumping - London Olympia round A	17.12.2017	CSI-W5*	4	1.60	490	3	10	01.01.2008	G	KWPN	69	1	30	9	36	2556	124	M	5	4b	yes	vertical	vertical	right	no	0	0	78,48	no
London	The Longines FEI World Cup Jumping - London Olympia round A	17.12.2017	CSI-W5*	4	1.60	490	3	10	01.01.2008	G	KWPN	69	1	30	9	36	2556	124	M	6	4c	yes	spread	oxer	right	no	0	0	78,48	no
London	The Longines FEI World Cup Jumping - London Olympia round A	17.12.2017	CSI-W5*	4	1.60	490	3	10	01.01.2008	G	KWPN	69	1	30	9	36	2556	124	M	7	5	no	vertical	vertical	left	no	0	0	78,48	no
London	The Longines FEI World Cup Jumping - London Olympia round A	17.12.2017	CSI-W5*	4	1.60	490	3	10	01.01.2008	G	KWPN	69	1	30	9	36	2556	124	M	8	6	no	vertical	wall	left	no	0	0	78,48	no
London	The Longines FEI World Cup Jumping - London Olympia round A	17.12.2017	CSI-W5*	4	1.60	490	3	10	01.01.2008	G	KWPN	69	1	30	9	36	2556	124	M	9	7	no	spread	oxer	right	no	0	0	78,48	no
London	The Longines FEI World Cup Jumping - London Olympia round A	17.12.2017	CSI-W5*	4	1.60	490	3	10	01.01.2008	G	KWPN	69	1	30	9	36	2556	124	M	10	8	no	vertical	vertical	right	no	0	0	78,48	no
London	The Longines FEI World Cup Jumping - London Olympia round A	17.12.2017	CSI-W5*	4	1.60	490	3	10	01.01.2008	G	KWPN	69	1	30	9	36	2556	124	M	11	9	no	spread	triple bar	right	no	0	0	78,48	no
London	The Longines FEI World Cup Jumping - London Olympia round A	17.12.2017	CSI-W5*	4	1.60	490	3	10	01.01.2008	G	KWPN	69	1	30	9	36	2556	124	M	12	10	no	vertical	vertical	straight	no	0	0	78,48	no
London	The Longines FEI World Cup Jumping - London Olympia round A	17.12.2017	CSI-W5*	4	1.60	490	3	10	01.01.2008	G	KWPN	69	1	30	9	36	2556	124	M	13	11a	yes	vertical	vertical	left	no	0	0	78,48	no
London	The Longines FEI World Cup Jumping - London Olympia round A	17.12.2017	CSI-W5*	4	1.60	490	3	10	01.01.2008	G	KWPN	69	1	30	9	36	2556	124	M	14	11b	yes	spread	oxer	left	no	0	0	78,48	no
London	The Longines FEI World Cup Jumping - London Olympia round A	17.12.2017	CSI-W5*	4	1.60	490	3	10	01.01.2008	G	KWPN	69	1	30	9	36	2556	124	M	15	12	no	vertical	vertical with water	straight	yes	4	0	78,48	no
London	The Longines FEI World Cup Jumping - London Olympia round A	17.12.2017	CSI-W5*	4	1.60	490	3	10	01.01.2008	G	KWPN	69	1	30	9	36	2556	124	M	16	13	no	spread	oxer	left	yes	4	3	78,48	no
London	The Longines FEI World Cup Jumping - London Olympia round A	17.12.2017	CSI-W5*	4	1.60	490	178	12	31.05.2006	G	SF	218	3	31	167	28	855	19	M	1	1	no	spread	oxer	left	no	0	0	84,53	no
London	The Longines FEI World Cup Jumping - London Olympia round A	17.12.2017	CSI-W5*	4	1.60	490	178	12	31.05.2006	G	SF	218	3	31	167	28	855	19	M	2	2	no	spread	oxer	left	no	0	0	84,53	no
London	The Longines FEI World Cup Jumping - London Olympia round A	17.12.2017	CSI-W5*	4	1.60	490	178	12	31.05.2006	G	SF	218	3	31	167	28	855	19	M	3	3	no	vertical	vertical	left	no	0	0	84,53	no
London	The Longines FEI World Cup Jumping - London Olympia round A	17.12.2017	CSI-W5*	4	1.60	490	178	12	31.05.2006	G	SF	218	3	31	167	28	855	19	M	4	4a	yes	spread	oxer	right	yes	4	0	84,53	no
London	The Longines FEI World Cup Jumping - London Olympia round A	17.12.2017	CSI-W5*	4	1.60	490	178	12	31.05.2006	G	SF	218	3	31	167	28	855	19	M	5	4b	yes	vertical	vertical	right	no	0	0	84,53	no
London	The Longines FEI World Cup Jumping - London Olympia round A	17.12.2017	CSI-W5*	4	1.60	490	178	12	31.05.2006	G	SF	218	3	31	167	28	855	19	M	6	4c	yes	spread	oxer	right	no	0	0	84,53	no
London	The Longines FEI World Cup Jumping - London Olympia round A	17.12.2017	CSI-W5*	4	1.60	490	178	12	31.05.2006	G	SF	218	3	31	167	28	855	19	M	7	5	no	vertical	vertical	left	no	0	0	84,53	no
London	The Longines FEI World Cup Jumping - London Olympia round A	17.12.2017	CSI-W5*	4	1.60	490	178	12	31.05.2006	G	SF	218	3	31	167	28	855	19	M	8	6	no	vertical	wall	left	no	0	0	84,53	no
London	The Longines FEI World Cup Jumping - London Olympia round A	17.12.2017	CSI-W5*	4	1.60	490	178	12	31.05.2006	G	SF	218	3	31	167	28	855	19	M	9	7	no	spread	oxer	right	no	0	0	84,53	no
London	The Longines FEI World Cup Jumping - London Olympia round A	17.12.2017	CSI-W5*	4	1.60	490	178	12	31.05.2006	G	SF	218	3	31	167	28	855	19	M	10	8	no	vertical	vertical	right	no	0	0	84,53	no
London	The Longines FEI World Cup Jumping - London Olympia round A	17.12.2017	CSI-W5*	4	1.60	490	178	12	31.05.2006	G	SF	218	3	31	167	28	855	19	M	11	9	no	spread	triple bar	right	yes	4	0	84,53	no
London	The Longines FEI World Cup Jumping - London Olympia round A	17.12.2017	CSI-W5*	4	1.60	490	178	12	31.05.2006	G	SF	218	3	31	167	28	855	19	M	12	10	no	vertical	vertical	straight	no	0	0	84,53	no
London	The Longines FEI World Cup Jumping - London Olympia round A	17.12.2017	CSI-W5*	4	1.60	490	178	12	31.05.2006	G	SF	218	3	31	167	28	855	19	M	13	11a	yes	vertical	vertical	left	no	0	0	84,53	no
London	The Longines FEI World Cup Jumping - London Olympia round A	17.12.2017	CSI-W5*	4	1.60	490	178	12	31.05.2006	G	SF	218	3	31	167	28	855	19	M	14	11b	yes	spread	oxer	left	no	0	0	84,53	no
London	The Longines FEI World Cup Jumping - London Olympia round A	17.12.2017	CSI-W5*	4	1.60	490	178	12	31.05.2006	G	SF	218	3	31	167	28	855	19	M	15	12	no	vertical	vertical with water	straight	no	0	0	84,53	no
London	The Longines FEI World Cup Jumping - London Olympia round A	17.12.2017	CSI-W5*	4	1.60	490	178	12	31.05.2006	G	SF	218	3	31	167	28	855	19	M	16	13	no	spread	oxer	left	no	0	4	84,53	no
London	The Longines FEI World Cup Jumping - London Olympia round A	17.12.2017	CSI-W5*	4	1.60	490	156	14	28.07.2004	M	AES	262	25	33	23	23	1854	199	M	1	1	no	spread	oxer	left	no	0	0	64,6	no
London	The Longines FEI World Cup Jumping - London Olympia round A	17.12.2017	CSI-W5*	4	1.60	490	156	14	28.07.2004	M	AES	262	25	33	23	23	1854	199	M	2	2	no	spread	oxer	left	no	0	0	64,6	no
London	The Longines FEI World Cup Jumping - London Olympia round A	17.12.2017	CSI-W5*	4	1.60	490	156	14	28.07.2004	M	AES	262	25	33	23	23	1854	199	M	3	3	no	vertical	vertical	left	no	0	0	64,6	no
London	The Longines FEI World Cup Jumping - London Olympia round A	17.12.2017	CSI-W5*	4	1.60	490	156	14	28.07.2004	M	AES	262	25	33	23	23	1854	199	M	4	4a	yes	spread	oxer	right	no	0	0	64,6	no
London	The Longines FEI World Cup Jumping - London Olympia round A	17.12.2017	CSI-W5*	4	1.60	490	156	14	28.07.2004	M	AES	262	25	33	23	23	1854	199	M	5	4b	yes	vertical	vertical	right	no	0	0	64,6	no
London	The Longines FEI World Cup Jumping - London Olympia round A	17.12.2017	CSI-W5*	4	1.60	490	156	14	28.07.2004	M	AES	262	25	33	23	23	1854	199	M	6	4c	yes	spread	oxer	right	no	0	0	64,6	no
London	The Longines FEI World Cup Jumping - London Olympia round A	17.12.2017	CSI-W5*	4	1.60	490	156	14	28.07.2004	M	AES	262	25	33	23	23	1854	199	M	7	5	no	vertical	vertical	left	no	0	0	64,6	no
London	The Longines FEI World Cup Jumping - London Olympia round A	17.12.2017	CSI-W5*	4	1.60	490	156	14	28.07.2004	M	AES	262	25	33	23	23	1854	199	M	8	6	no	vertical	wall	left	no	0	0	64,6	no
London	The Longines FEI World Cup Jumping - London Olympia round A	17.12.2017	CSI-W5*	4	1.60	490	156	14	28.07.2004	M	AES	262	25	33	23	23	1854	199	M	9	7	no	spread	oxer	right	no	0	0	64,6	no
London	The Longines FEI World Cup Jumping - London Olympia round A	17.12.2017	CSI-W5*	4	1.60	490	156	14	28.07.2004	M	AES	262	25	33	23	23	1854	199	M	10	8	no	vertical	vertical	right	no	0	0	64,6	no
London	The Longines FEI World Cup Jumping - London Olympia round A	17.12.2017	CSI-W5*	4	1.60	490	156	14	28.07.2004	M	AES	262	25	33	23	23	1854	199	M	11	9	no	spread	triple bar	right	no	0	0	64,6	no
London	The Longines FEI World Cup Jumping - London Olympia round A	17.12.2017	CSI-W5*	4	1.60	490	156	14	28.07.2004	M	AES	262	25	33	23	23	1854	199	M	12	10	no	vertical	vertical	straight	no	0	0	64,6	no
London	The Longines FEI World Cup Jumping - London Olympia round A	17.12.2017	CSI-W5*	4	1.60	490	156	14	28.07.2004	M	AES	262	25	33	23	23	1854	199	M	13	11a	yes	vertical	vertical	left	yes	4	0	64,6	no
London	The Longines FEI World Cup Jumping - London Olympia round A	17.12.2017	CSI-W5*	4	1.60	490	156	14	28.07.2004	M	AES	262	25	33	23	23	1854	199	M	14	11b	yes	spread	oxer	left	no	0	0	64,6	no
London	The Longines FEI World Cup Jumping - London Olympia round A	17.12.2017	CSI-W5*	4	1.60	490	156	14	28.07.2004	M	AES	262	25	33	23	23	1854	199	M	15	12	no	vertical	vertical with water	straight	no	0	0	64,6	no
London	The Longines FEI World Cup Jumping - London Olympia round A	17.12.2017	CSI-W5*	4	1.60	490	156	14	28.07.2004	M	AES	262	25	33	23	23	1854	199	M	16	13	no	spread	oxer	left	no	0	0	64,6	no
London	The Longines FEI World Cup Jumping - London Olympia round A	17.12.2017	CSI-W5*	4	1.60	490	129	10	02.04.2008	G	BWP	123	5	36	47	44	1871	37	F	1	1	no	spread	oxer	left	no	0	0	69,94	no
London	The Longines FEI World Cup Jumping - London Olympia round A	17.12.2017	CSI-W5*	4	1.60	490	129	10	02.04.2008	G	BWP	123	5	36	47	44	1871	37	F	2	2	no	spread	oxer	left	no	0	0	69,94	no
London	The Longines FEI World Cup Jumping - London Olympia round A	17.12.2017	CSI-W5*	4	1.60	490	129	10	02.04.2008	G	BWP	123	5	36	47	44	1871	37	F	3	3	no	vertical	vertical	left	no	0	0	69,94	no
London	The Longines FEI World Cup Jumping - London Olympia round A	17.12.2017	CSI-W5*	4	1.60	490	129	10	02.04.2008	G	BWP	123	5	36	47	44	1871	37	F	4	4a	yes	spread	oxer	right	no	0	0	69,94	no
London	The Longines FEI World Cup Jumping - London Olympia round A	17.12.2017	CSI-W5*	4	1.60	490	129	10	02.04.2008	G	BWP	123	5	36	47	44	1871	37	F	5	4b	yes	vertical	vertical	right	no	0	0	69,94	no
London	The Longines FEI World Cup Jumping - London Olympia round A	17.12.2017	CSI-W5*	4	1.60	490	129	10	02.04.2008	G	BWP	123	5	36	47	44	1871	37	F	6	4c	yes	spread	oxer	right	no	0	0	69,94	no
London	The Longines FEI World Cup Jumping - London Olympia round A	17.12.2017	CSI-W5*	4	1.60	490	129	10	02.04.2008	G	BWP	123	5	36	47	44	1871	37	F	7	5	no	vertical	vertical	left	no	0	0	69,94	no
London	The Longines FEI World Cup Jumping - London Olympia round A	17.12.2017	CSI-W5*	4	1.60	490	129	10	02.04.2008	G	BWP	123	5	36	47	44	1871	37	F	8	6	no	vertical	wall	left	no	0	0	69,94	no
London	The Longines FEI World Cup Jumping - London Olympia round A	17.12.2017	CSI-W5*	4	1.60	490	129	10	02.04.2008	G	BWP	123	5	36	47	44	1871	37	F	9	7	no	spread	oxer	right	no	0	0	69,94	no
London	The Longines FEI World Cup Jumping - London Olympia round A	17.12.2017	CSI-W5*	4	1.60	490	129	10	02.04.2008	G	BWP	123	5	36	47	44	1871	37	F	10	8	no	vertical	vertical	right	no	0	0	69,94	no
London	The Longines FEI World Cup Jumping - London Olympia round A	17.12.2017	CSI-W5*	4	1.60	490	129	10	02.04.2008	G	BWP	123	5	36	47	44	1871	37	F	11	9	no	spread	triple bar	right	no	0	0	69,94	no
London	The Longines FEI World Cup Jumping - London Olympia round A	17.12.2017	CSI-W5*	4	1.60	490	129	10	02.04.2008	G	BWP	123	5	36	47	44	1871	37	F	12	10	no	vertical	vertical	straight	no	0	0	69,94	no
London	The Longines FEI World Cup Jumping - London Olympia round A	17.12.2017	CSI-W5*	4	1.60	490	129	10	02.04.2008	G	BWP	123	5	36	47	44	1871	37	F	13	11a	yes	vertical	vertical	left	no	0	0	69,94	no
London	The Longines FEI World Cup Jumping - London Olympia round A	17.12.2017	CSI-W5*	4	1.60	490	129	10	02.04.2008	G	BWP	123	5	36	47	44	1871	37	F	14	11b	yes	spread	oxer	left	no	0	0	69,94	no
London	The Longines FEI World Cup Jumping - London Olympia round A	17.12.2017	CSI-W5*	4	1.60	490	129	10	02.04.2008	G	BWP	123	5	36	47	44	1871	37	F	15	12	no	vertical	vertical with water	straight	no	0	0	69,94	no
London	The Longines FEI World Cup Jumping - London Olympia round A	17.12.2017	CSI-W5*	4	1.60	490	129	10	02.04.2008	G	BWP	123	5	36	47	44	1871	37	F	16	13	no	spread	oxer	left	no	0	0	69,94	no
London	The Longines FEI World Cup Jumping - London Olympia round B	17.12.2017	CSI-W5*	4	1.60	280	129	10	02.04.2008	G	BWP	123	5	36	47	44	1871	37	F	1	1	no	spread	oxer	left	no	0	0	41,05	no
London	The Longines FEI World Cup Jumping - London Olympia round B	17.12.2017	CSI-W5*	4	1.60	280	129	10	02.04.2008	G	BWP	123	5	36	47	44	1871	37	F	2	2	no	spread	oxer	left	no	0	0	41,05	no
London	The Longines FEI World Cup Jumping - London Olympia round B	17.12.2017	CSI-W5*	4	1.60	280	129	10	02.04.2008	G	BWP	123	5	36	47	44	1871	37	F	3	3	no	vertical	vertical	left	no	0	0	41,05	no
London	The Longines FEI World Cup Jumping - London Olympia round B	17.12.2017	CSI-W5*	4	1.60	280	129	10	02.04.2008	G	BWP	123	5	36	47	44	1871	37	F	4	4a	yes	vertical	vertical	right	no	0	0	41,05	no
London	The Longines FEI World Cup Jumping - London Olympia round B	17.12.2017	CSI-W5*	4	1.60	280	129	10	02.04.2008	G	BWP	123	5	36	47	44	1871	37	F	5	4b	yes	spread	oxer	right	no	0	0	41,05	no
London	The Longines FEI World Cup Jumping - London Olympia round B	17.12.2017	CSI-W5*	4	1.60	280	129	10	02.04.2008	G	BWP	123	5	36	47	44	1871	37	F	6	5	no	vertical	wall	left	no	0	0	41,05	no
London	The Longines FEI World Cup Jumping - London Olympia round B	17.12.2017	CSI-W5*	4	1.60	280	129	10	02.04.2008	G	BWP	123	5	36	47	44	1871	37	F	7	6	no	vertical	vertical	right	no	0	0	41,05	no
London	The Longines FEI World Cup Jumping - London Olympia round B	17.12.2017	CSI-W5*	4	1.60	280	129	10	02.04.2008	G	BWP	123	5	36	47	44	1871	37	F	8	7	no	spread	oxer	right	no	0	0	41,05	no
London	The Longines FEI World Cup Jumping - London Olympia round A	17.12.2017	CSI-W5*	4	1.60	490	197	11	30.04.2007	G	AA	96	0	37	59	42	1314	68	M	1	1	no	spread	oxer	left	no	0	0	67,4	no
London	The Longines FEI World Cup Jumping - London Olympia round A	17.12.2017	CSI-W5*	4	1.60	490	197	11	30.04.2007	G	AA	96	0	37	59	42	1314	68	M	2	2	no	spread	oxer	left	no	0	0	67,4	no
London	The Longines FEI World Cup Jumping - London Olympia round A	17.12.2017	CSI-W5*	4	1.60	490	197	11	30.04.2007	G	AA	96	0	37	59	42	1314	68	M	3	3	no	vertical	vertical	left	no	0	0	67,4	no
London	The Longines FEI World Cup Jumping - London Olympia round A	17.12.2017	CSI-W5*	4	1.60	490	197	11	30.04.2007	G	AA	96	0	37	59	42	1314	68	M	4	4a	yes	spread	oxer	right	no	0	0	67,4	no
London	The Longines FEI World Cup Jumping - London Olympia round A	17.12.2017	CSI-W5*	4	1.60	490	197	11	30.04.2007	G	AA	96	0	37	59	42	1314	68	M	5	4b	yes	vertical	vertical	right	yes	4	0	67,4	no
London	The Longines FEI World Cup Jumping - London Olympia round A	17.12.2017	CSI-W5*	4	1.60	490	197	11	30.04.2007	G	AA	96	0	37	59	42	1314	68	M	6	4c	yes	spread	oxer	right	no	0	0	67,4	no
London	The Longines FEI World Cup Jumping - London Olympia round A	17.12.2017	CSI-W5*	4	1.60	490	197	11	30.04.2007	G	AA	96	0	37	59	42	1314	68	M	7	5	no	vertical	vertical	left	no	0	0	67,4	no
London	The Longines FEI World Cup Jumping - London Olympia round A	17.12.2017	CSI-W5*	4	1.60	490	197	11	30.04.2007	G	AA	96	0	37	59	42	1314	68	M	8	6	no	vertical	wall	left	no	0	0	67,4	no
London	The Longines FEI World Cup Jumping - London Olympia round A	17.12.2017	CSI-W5*	4	1.60	490	197	11	30.04.2007	G	AA	96	0	37	59	42	1314	68	M	9	7	no	spread	oxer	right	no	0	0	67,4	no
London	The Longines FEI World Cup Jumping - London Olympia round A	17.12.2017	CSI-W5*	4	1.60	490	197	11	30.04.2007	G	AA	96	0	37	59	42	1314	68	M	10	8	no	vertical	vertical	right	no	0	0	67,4	no
London	The Longines FEI World Cup Jumping - London Olympia round A	17.12.2017	CSI-W5*	4	1.60	490	197	11	30.04.2007	G	AA	96	0	37	59	42	1314	68	M	11	9	no	spread	triple bar	right	no	0	0	67,4	no
London	The Longines FEI World Cup Jumping - London Olympia round A	17.12.2017	CSI-W5*	4	1.60	490	197	11	30.04.2007	G	AA	96	0	37	59	42	1314	68	M	12	10	no	vertical	vertical	straight	no	0	0	67,4	no
London	The Longines FEI World Cup Jumping - London Olympia round A	17.12.2017	CSI-W5*	4	1.60	490	197	11	30.04.2007	G	AA	96	0	37	59	42	1314	68	M	13	11a	yes	vertical	vertical	left	no	0	0	67,4	no
London	The Longines FEI World Cup Jumping - London Olympia round A	17.12.2017	CSI-W5*	4	1.60	490	197	11	30.04.2007	G	AA	96	0	37	59	42	1314	68	M	14	11b	yes	spread	oxer	left	no	0	0	67,4	no
London	The Longines FEI World Cup Jumping - London Olympia round A	17.12.2017	CSI-W5*	4	1.60	490	197	11	30.04.2007	G	AA	96	0	37	59	42	1314	68	M	15	12	no	vertical	vertical with water	straight	yes	4	0	67,4	no
London	The Longines FEI World Cup Jumping - London Olympia round A	17.12.2017	CSI-W5*	4	1.60	490	197	11	30.04.2007	G	AA	96	0	37	59	42	1314	68	M	16	13	no	spread	oxer	left	no	0	0	67,4	no
London	The Longines FEI World Cup Jumping - London Olympia round A	17.12.2017	CSI-W5*	4	1.60	490	49	14	10.06.2004	M	OS	150	2	38	10	29	1642	46	M	1	1	no	spread	oxer	left	no	0	0	66,65	no
London	The Longines FEI World Cup Jumping - London Olympia round A	17.12.2017	CSI-W5*	4	1.60	490	49	14	10.06.2004	M	OS	150	2	38	10	29	1642	46	M	2	2	no	spread	oxer	left	no	0	0	66,65	no
London	The Longines FEI World Cup Jumping - London Olympia round A	17.12.2017	CSI-W5*	4	1.60	490	49	14	10.06.2004	M	OS	150	2	38	10	29	1642	46	M	3	3	no	vertical	vertical	left	no	0	0	66,65	no
London	The Longines FEI World Cup Jumping - London Olympia round A	17.12.2017	CSI-W5*	4	1.60	490	49	14	10.06.2004	M	OS	150	2	38	10	29	1642	46	M	4	4a	yes	spread	oxer	right	no	0	0	66,65	no
London	The Longines FEI World Cup Jumping - London Olympia round A	17.12.2017	CSI-W5*	4	1.60	490	49	14	10.06.2004	M	OS	150	2	38	10	29	1642	46	M	5	4b	yes	vertical	vertical	right	no	0	0	66,65	no
London	The Longines FEI World Cup Jumping - London Olympia round A	17.12.2017	CSI-W5*	4	1.60	490	49	14	10.06.2004	M	OS	150	2	38	10	29	1642	46	M	6	4c	yes	spread	oxer	right	no	0	0	66,65	no
London	The Longines FEI World Cup Jumping - London Olympia round A	17.12.2017	CSI-W5*	4	1.60	490	49	14	10.06.2004	M	OS	150	2	38	10	29	1642	46	M	7	5	no	vertical	vertical	left	no	0	0	66,65	no
London	The Longines FEI World Cup Jumping - London Olympia round A	17.12.2017	CSI-W5*	4	1.60	490	49	14	10.06.2004	M	OS	150	2	38	10	29	1642	46	M	8	6	no	vertical	wall	left	no	0	0	66,65	no
London	The Longines FEI World Cup Jumping - London Olympia round A	17.12.2017	CSI-W5*	4	1.60	490	49	14	10.06.2004	M	OS	150	2	38	10	29	1642	46	M	9	7	no	spread	oxer	right	no	0	0	66,65	no
London	The Longines FEI World Cup Jumping - London Olympia round A	17.12.2017	CSI-W5*	4	1.60	490	49	14	10.06.2004	M	OS	150	2	38	10	29	1642	46	M	10	8	no	vertical	vertical	right	yes	4	0	66,65	no
London	The Longines FEI World Cup Jumping - London Olympia round A	17.12.2017	CSI-W5*	4	1.60	490	49	14	10.06.2004	M	OS	150	2	38	10	29	1642	46	M	11	9	no	spread	triple bar	right	no	0	0	66,65	no
London	The Longines FEI World Cup Jumping - London Olympia round A	17.12.2017	CSI-W5*	4	1.60	490	49	14	10.06.2004	M	OS	150	2	38	10	29	1642	46	M	12	10	no	vertical	vertical	straight	no	0	0	66,65	no
London	The Longines FEI World Cup Jumping - London Olympia round A	17.12.2017	CSI-W5*	4	1.60	490	49	14	10.06.2004	M	OS	150	2	38	10	29	1642	46	M	13	11a	yes	vertical	vertical	left	no	0	0	66,65	no
London	The Longines FEI World Cup Jumping - London Olympia round A	17.12.2017	CSI-W5*	4	1.60	490	49	14	10.06.2004	M	OS	150	2	38	10	29	1642	46	M	14	11b	yes	spread	oxer	left	no	0	0	66,65	no
London	The Longines FEI World Cup Jumping - London Olympia round A	17.12.2017	CSI-W5*	4	1.60	490	49	14	10.06.2004	M	OS	150	2	38	10	29	1642	46	M	15	12	no	vertical	vertical with water	straight	no	0	0	66,65	no
London	The Longines FEI World Cup Jumping - London Olympia round A	17.12.2017	CSI-W5*	4	1.60	490	49	14	10.06.2004	M	OS	150	2	38	10	29	1642	46	M	16	13	no	spread	oxer	left	no	0	0	66,65	no
London	The Longines FEI World Cup Jumping - London Olympia round A	17.12.2017	CSI-W5*	4	1.60	490	208	10	18.05.2008	G	SF	126	1	43	134	48	1808	64	M	1	1	no	spread	oxer	left	no	0	0	68,27	no
London	The Longines FEI World Cup Jumping - London Olympia round A	17.12.2017	CSI-W5*	4	1.60	490	208	10	18.05.2008	G	SF	126	1	43	134	48	1808	64	M	2	2	no	spread	oxer	left	no	0	0	68,27	no
London	The Longines FEI World Cup Jumping - London Olympia round A	17.12.2017	CSI-W5*	4	1.60	490	208	10	18.05.2008	G	SF	126	1	43	134	48	1808	64	M	3	3	no	vertical	vertical	left	yes	4	0	68,27	no
London	The Longines FEI World Cup Jumping - London Olympia round A	17.12.2017	CSI-W5*	4	1.60	490	208	10	18.05.2008	G	SF	126	1	43	134	48	1808	64	M	4	4a	yes	spread	oxer	right	no	0	0	68,27	no
London	The Longines FEI World Cup Jumping - London Olympia round A	17.12.2017	CSI-W5*	4	1.60	490	208	10	18.05.2008	G	SF	126	1	43	134	48	1808	64	M	5	4b	yes	vertical	vertical	right	no	0	0	68,27	no
London	The Longines FEI World Cup Jumping - London Olympia round A	17.12.2017	CSI-W5*	4	1.60	490	208	10	18.05.2008	G	SF	126	1	43	134	48	1808	64	M	6	4c	yes	spread	oxer	right	no	0	0	68,27	no
London	The Longines FEI World Cup Jumping - London Olympia round A	17.12.2017	CSI-W5*	4	1.60	490	208	10	18.05.2008	G	SF	126	1	43	134	48	1808	64	M	7	5	no	vertical	vertical	left	no	0	0	68,27	no
London	The Longines FEI World Cup Jumping - London Olympia round A	17.12.2017	CSI-W5*	4	1.60	490	208	10	18.05.2008	G	SF	126	1	43	134	48	1808	64	M	8	6	no	vertical	wall	left	no	0	0	68,27	no
London	The Longines FEI World Cup Jumping - London Olympia round A	17.12.2017	CSI-W5*	4	1.60	490	208	10	18.05.2008	G	SF	126	1	43	134	48	1808	64	M	9	7	no	spread	oxer	right	no	0	0	68,27	no
London	The Longines FEI World Cup Jumping - London Olympia round A	17.12.2017	CSI-W5*	4	1.60	490	208	10	18.05.2008	G	SF	126	1	43	134	48	1808	64	M	10	8	no	vertical	vertical	right	no	0	0	68,27	no
London	The Longines FEI World Cup Jumping - London Olympia round A	17.12.2017	CSI-W5*	4	1.60	490	208	10	18.05.2008	G	SF	126	1	43	134	48	1808	64	M	11	9	no	spread	triple bar	right	no	0	0	68,27	no
London	The Longines FEI World Cup Jumping - London Olympia round A	17.12.2017	CSI-W5*	4	1.60	490	208	10	18.05.2008	G	SF	126	1	43	134	48	1808	64	M	12	10	no	vertical	vertical	straight	no	0	0	68,27	no
London	The Longines FEI World Cup Jumping - London Olympia round A	17.12.2017	CSI-W5*	4	1.60	490	208	10	18.05.2008	G	SF	126	1	43	134	48	1808	64	M	13	11a	yes	vertical	vertical	left	no	0	0	68,27	no
London	The Longines FEI World Cup Jumping - London Olympia round A	17.12.2017	CSI-W5*	4	1.60	490	208	10	18.05.2008	G	SF	126	1	43	134	48	1808	64	M	14	11b	yes	spread	oxer	left	no	0	0	68,27	no
London	The Longines FEI World Cup Jumping - London Olympia round A	17.12.2017	CSI-W5*	4	1.60	490	208	10	18.05.2008	G	SF	126	1	43	134	48	1808	64	M	15	12	no	vertical	vertical with water	straight	yes	4	0	68,27	no
London	The Longines FEI World Cup Jumping - London Olympia round A	17.12.2017	CSI-W5*	4	1.60	490	208	10	18.05.2008	G	SF	126	1	43	134	48	1808	64	M	16	13	no	spread	oxer	left	no	0	0	68,27	no
London	The Longines FEI World Cup Jumping - London Olympia round A	17.12.2017	CSI-W5*	4	1.60	490	105	12	02.04.2006	M	DSP	186	8	50	30	25	1981	78	M	1	1	no	spread	oxer	left	no	0	0	68,58	no
London	The Longines FEI World Cup Jumping - London Olympia round A	17.12.2017	CSI-W5*	4	1.60	490	105	12	02.04.2006	M	DSP	186	8	50	30	25	1981	78	M	2	2	no	spread	oxer	left	no	0	0	68,58	no
London	The Longines FEI World Cup Jumping - London Olympia round A	17.12.2017	CSI-W5*	4	1.60	490	105	12	02.04.2006	M	DSP	186	8	50	30	25	1981	78	M	3	3	no	vertical	vertical	left	no	0	0	68,58	no
London	The Longines FEI World Cup Jumping - London Olympia round A	17.12.2017	CSI-W5*	4	1.60	490	105	12	02.04.2006	M	DSP	186	8	50	30	25	1981	78	M	4	4a	yes	spread	oxer	right	no	0	0	68,58	no
London	The Longines FEI World Cup Jumping - London Olympia round A	17.12.2017	CSI-W5*	4	1.60	490	105	12	02.04.2006	M	DSP	186	8	50	30	25	1981	78	M	5	4b	yes	vertical	vertical	right	no	0	0	68,58	no
London	The Longines FEI World Cup Jumping - London Olympia round A	17.12.2017	CSI-W5*	4	1.60	490	105	12	02.04.2006	M	DSP	186	8	50	30	25	1981	78	M	6	4c	yes	spread	oxer	right	no	0	0	68,58	no
London	The Longines FEI World Cup Jumping - London Olympia round A	17.12.2017	CSI-W5*	4	1.60	490	105	12	02.04.2006	M	DSP	186	8	50	30	25	1981	78	M	7	5	no	vertical	vertical	left	no	0	0	68,58	no
London	The Longines FEI World Cup Jumping - London Olympia round A	17.12.2017	CSI-W5*	4	1.60	490	105	12	02.04.2006	M	DSP	186	8	50	30	25	1981	78	M	8	6	no	vertical	wall	left	no	0	0	68,58	no
London	The Longines FEI World Cup Jumping - London Olympia round A	17.12.2017	CSI-W5*	4	1.60	490	105	12	02.04.2006	M	DSP	186	8	50	30	25	1981	78	M	9	7	no	spread	oxer	right	no	0	0	68,58	no
London	The Longines FEI World Cup Jumping - London Olympia round A	17.12.2017	CSI-W5*	4	1.60	490	105	12	02.04.2006	M	DSP	186	8	50	30	25	1981	78	M	10	8	no	vertical	vertical	right	no	0	0	68,58	no
London	The Longines FEI World Cup Jumping - London Olympia round A	17.12.2017	CSI-W5*	4	1.60	490	105	12	02.04.2006	M	DSP	186	8	50	30	25	1981	78	M	11	9	no	spread	triple bar	right	no	0	0	68,58	no
London	The Longines FEI World Cup Jumping - London Olympia round A	17.12.2017	CSI-W5*	4	1.60	490	105	12	02.04.2006	M	DSP	186	8	50	30	25	1981	78	M	12	10	no	vertical	vertical	straight	no	0	0	68,58	no
London	The Longines FEI World Cup Jumping - London Olympia round A	17.12.2017	CSI-W5*	4	1.60	490	105	12	02.04.2006	M	DSP	186	8	50	30	25	1981	78	M	13	11a	yes	vertical	vertical	left	yes	4	0	68,58	no
London	The Longines FEI World Cup Jumping - London Olympia round A	17.12.2017	CSI-W5*	4	1.60	490	105	12	02.04.2006	M	DSP	186	8	50	30	25	1981	78	M	14	11b	yes	spread	oxer	left	no	0	0	68,58	no
London	The Longines FEI World Cup Jumping - London Olympia round A	17.12.2017	CSI-W5*	4	1.60	490	105	12	02.04.2006	M	DSP	186	8	50	30	25	1981	78	M	15	12	no	vertical	vertical with water	straight	no	0	0	68,58	no
London	The Longines FEI World Cup Jumping - London Olympia round A	17.12.2017	CSI-W5*	4	1.60	490	105	12	02.04.2006	M	DSP	186	8	50	30	25	1981	78	M	16	13	no	spread	oxer	left	no	0	0	68,58	no
London	The Longines FEI World Cup Jumping - London Olympia round A	17.12.2017	CSI-W5*	4	1.60	490	31	12	15.04.2006	S	OS	292	11	59	57	32	2641	229	M	1	1	no	spread	oxer	left	no	0	0	65,44	no
London	The Longines FEI World Cup Jumping - London Olympia round A	17.12.2017	CSI-W5*	4	1.60	490	31	12	15.04.2006	S	OS	292	11	59	57	32	2641	229	M	2	2	no	spread	oxer	left	yes	4	0	65,44	no
London	The Longines FEI World Cup Jumping - London Olympia round A	17.12.2017	CSI-W5*	4	1.60	490	31	12	15.04.2006	S	OS	292	11	59	57	32	2641	229	M	3	3	no	vertical	vertical	left	no	0	0	65,44	no
London	The Longines FEI World Cup Jumping - London Olympia round A	17.12.2017	CSI-W5*	4	1.60	490	31	12	15.04.2006	S	OS	292	11	59	57	32	2641	229	M	4	4a	yes	spread	oxer	right	no	0	0	65,44	no
London	The Longines FEI World Cup Jumping - London Olympia round A	17.12.2017	CSI-W5*	4	1.60	490	31	12	15.04.2006	S	OS	292	11	59	57	32	2641	229	M	5	4b	yes	vertical	vertical	right	no	0	0	65,44	no
London	The Longines FEI World Cup Jumping - London Olympia round A	17.12.2017	CSI-W5*	4	1.60	490	31	12	15.04.2006	S	OS	292	11	59	57	32	2641	229	M	6	4c	yes	spread	oxer	right	no	0	0	65,44	no
London	The Longines FEI World Cup Jumping - London Olympia round A	17.12.2017	CSI-W5*	4	1.60	490	31	12	15.04.2006	S	OS	292	11	59	57	32	2641	229	M	7	5	no	vertical	vertical	left	no	0	0	65,44	no
London	The Longines FEI World Cup Jumping - London Olympia round A	17.12.2017	CSI-W5*	4	1.60	490	31	12	15.04.2006	S	OS	292	11	59	57	32	2641	229	M	8	6	no	vertical	wall	left	no	0	0	65,44	no
London	The Longines FEI World Cup Jumping - London Olympia round A	17.12.2017	CSI-W5*	4	1.60	490	31	12	15.04.2006	S	OS	292	11	59	57	32	2641	229	M	9	7	no	spread	oxer	right	no	0	0	65,44	no
London	The Longines FEI World Cup Jumping - London Olympia round A	17.12.2017	CSI-W5*	4	1.60	490	31	12	15.04.2006	S	OS	292	11	59	57	32	2641	229	M	10	8	no	vertical	vertical	right	no	0	0	65,44	no
London	The Longines FEI World Cup Jumping - London Olympia round A	17.12.2017	CSI-W5*	4	1.60	490	31	12	15.04.2006	S	OS	292	11	59	57	32	2641	229	M	11	9	no	spread	triple bar	right	no	0	0	65,44	no
London	The Longines FEI World Cup Jumping - London Olympia round A	17.12.2017	CSI-W5*	4	1.60	490	31	12	15.04.2006	S	OS	292	11	59	57	32	2641	229	M	12	10	no	vertical	vertical	straight	no	0	0	65,44	no
London	The Longines FEI World Cup Jumping - London Olympia round A	17.12.2017	CSI-W5*	4	1.60	490	31	12	15.04.2006	S	OS	292	11	59	57	32	2641	229	M	13	11a	yes	vertical	vertical	left	yes	4	0	65,44	no
London	The Longines FEI World Cup Jumping - London Olympia round A	17.12.2017	CSI-W5*	4	1.60	490	31	12	15.04.2006	S	OS	292	11	59	57	32	2641	229	M	14	11b	yes	spread	oxer	left	yes	4	0	65,44	no
London	The Longines FEI World Cup Jumping - London Olympia round A	17.12.2017	CSI-W5*	4	1.60	490	31	12	15.04.2006	S	OS	292	11	59	57	32	2641	229	M	15	12	no	vertical	vertical with water	straight	no	0	0	65,44	no
London	The Longines FEI World Cup Jumping - London Olympia round A	17.12.2017	CSI-W5*	4	1.60	490	31	12	15.04.2006	S	OS	292	11	59	57	32	2641	229	M	16	13	no	spread	oxer	left	no	0	0	65,44	no
London	The Longines FEI World Cup Jumping - London Olympia round A	17.12.2017	CSI-W5*	4	1.60	490	186	11	04.06.2007	G	BWP	123	2	63	25	39	1079	61	M	1	1	no	spread	oxer	left	no	0	0	68,52	no
London	The Longines FEI World Cup Jumping - London Olympia round A	17.12.2017	CSI-W5*	4	1.60	490	186	11	04.06.2007	G	BWP	123	2	63	25	39	1079	61	M	2	2	no	spread	oxer	left	no	0	0	68,52	no
London	The Longines FEI World Cup Jumping - London Olympia round A	17.12.2017	CSI-W5*	4	1.60	490	186	11	04.06.2007	G	BWP	123	2	63	25	39	1079	61	M	3	3	no	vertical	vertical	left	no	0	0	68,52	no
London	The Longines FEI World Cup Jumping - London Olympia round A	17.12.2017	CSI-W5*	4	1.60	490	186	11	04.06.2007	G	BWP	123	2	63	25	39	1079	61	M	4	4a	yes	spread	oxer	right	no	0	0	68,52	no
London	The Longines FEI World Cup Jumping - London Olympia round A	17.12.2017	CSI-W5*	4	1.60	490	186	11	04.06.2007	G	BWP	123	2	63	25	39	1079	61	M	5	4b	yes	vertical	vertical	right	no	0	0	68,52	no
London	The Longines FEI World Cup Jumping - London Olympia round A	17.12.2017	CSI-W5*	4	1.60	490	186	11	04.06.2007	G	BWP	123	2	63	25	39	1079	61	M	6	4c	yes	spread	oxer	right	no	0	0	68,52	no
London	The Longines FEI World Cup Jumping - London Olympia round A	17.12.2017	CSI-W5*	4	1.60	490	186	11	04.06.2007	G	BWP	123	2	63	25	39	1079	61	M	7	5	no	vertical	vertical	left	no	0	0	68,52	no

London	The Longines FEI World Cup Jumping - London Olympia round A	17.12.2017	CSI-W5*	4	1.60	490	186	11	04.06.2007	G	BWP	123	2	63	25	39	1079	61	M	8	6	no	vertical	wall	left	no	0	0	68,52	no
London	The Longines FEI World Cup Jumping - London Olympia round A	17.12.2017	CSI-W5*	4	1.60	490	186	11	04.06.2007	G	BWP	123	2	63	25	39	1079	61	M	9	7	no	spread	oxer	right	no	0	0	68,52	no
London	The Longines FEI World Cup Jumping - London Olympia round A	17.12.2017	CSI-W5*	4	1.60	490	186	11	04.06.2007	G	BWP	123	2	63	25	39	1079	61	M	10	8	no	vertical	vertical	right	no	0	0	68,52	no
London	The Longines FEI World Cup Jumping - London Olympia round A	17.12.2017	CSI-W5*	4	1.60	490	186	11	04.06.2007	G	BWP	123	2	63	25	39	1079	61	M	11	9	no	spread	triple bar	right	no	0	0	68,52	no
London	The Longines FEI World Cup Jumping - London Olympia round A	17.12.2017	CSI-W5*	4	1.60	490	186	11	04.06.2007	G	BWP	123	2	63	25	39	1079	61	M	12	10	no	vertical	vertical	straight	yes	4	0	68,52	no
London	The Longines FEI World Cup Jumping - London Olympia round A	17.12.2017	CSI-W5*	4	1.60	490	186	11	04.06.2007	G	BWP	123	2	63	25	39	1079	61	M	13	11a	yes	vertical	vertical	left	no	0	0	68,52	no
London	The Longines FEI World Cup Jumping - London Olympia round A	17.12.2017	CSI-W5*	4	1.60	490	186	11	04.06.2007	G	BWP	123	2	63	25	39	1079	61	M	14	11b	yes	spread	oxer	left	no	0	0	68,52	no
London	The Longines FEI World Cup Jumping - London Olympia round A	17.12.2017	CSI-W5*	4	1.60	490	186	11	04.06.2007	G	BWP	123	2	63	25	39	1079	61	M	15	12	no	vertical	vertical with water	straight	yes	4	0	68,52	no
London	The Longines FEI World Cup Jumping - London Olympia round A	17.12.2017	CSI-W5*	4	1.60	490	186	11	04.06.2007	G	BWP	123	2	63	25	39	1079	61	M	16	13	no	spread	oxer	left	no	0	0	68,52	no
London	The Longines FEI World Cup Jumping - London Olympia round A	17.12.2017	CSI-W5*	4	1.60	490	43	15	20.05.2003	S	DSP	218	12	64	20	44	2014	118	M	1	1	no	spread	oxer	left	no	0	0	67,76	no
London	The Longines FEI World Cup Jumping - London Olympia round A	17.12.2017	CSI-W5*	4	1.60	490	43	15	20.05.2003	S	DSP	218	12	64	20	44	2014	118	M	2	2	no	spread	oxer	left	no	0	0	67,76	no
London	The Longines FEI World Cup Jumping - London Olympia round A	17.12.2017	CSI-W5*	4	1.60	490	43	15	20.05.2003	S	DSP	218	12	64	20	44	2014	118	M	3	3	no	vertical	vertical	left	no	0	0	67,76	no
London	The Longines FEI World Cup Jumping - London Olympia round A	17.12.2017	CSI-W5*	4	1.60	490	43	15	20.05.2003	S	DSP	218	12	64	20	44	2014	118	M	4	4a	yes	spread	oxer	right	no	0	0	67,76	no
London	The Longines FEI World Cup Jumping - London Olympia round A	17.12.2017	CSI-W5*	4	1.60	490	43	15	20.05.2003	S	DSP	218	12	64	20	44	2014	118	M	5	4b	yes	vertical	vertical	right	no	0	0	67,76	no
London	The Longines FEI World Cup Jumping - London Olympia round A	17.12.2017	CSI-W5*	4	1.60	490	43	15	20.05.2003	S	DSP	218	12	64	20	44	2014	118	M	6	4c	yes	spread	oxer	right	no	0	0	67,76	no
London	The Longines FEI World Cup Jumping - London Olympia round A	17.12.2017	CSI-W5*	4	1.60	490	43	15	20.05.2003	S	DSP	218	12	64	20	44	2014	118	M	7	5	no	vertical	vertical	left	no	0	0	67,76	no
London	The Longines FEI World Cup Jumping - London Olympia round A	17.12.2017	CSI-W5*	4	1.60	490	43	15	20.05.2003	S	DSP	218	12	64	20	44	2014	118	M	8	6	no	vertical	wall	left	no	0	0	67,76	no
London	The Longines FEI World Cup Jumping - London Olympia round A	17.12.2017	CSI-W5*	4	1.60	490	43	15	20.05.2003	S	DSP	218	12	64	20	44	2014	118	M	9	7	no	spread	oxer	right	no	0	0	67,76	no
London	The Longines FEI World Cup Jumping - London Olympia round A	17.12.2017	CSI-W5*	4	1.60	490	43	15	20.05.2003	S	DSP	218	12	64	20	44	2014	118	M	10	8	no	vertical	vertical	right	no	0	0	67,76	no
London	The Longines FEI World Cup Jumping - London Olympia round A	17.12.2017	CSI-W5*	4	1.60	490	43	15	20.05.2003	S	DSP	218	12	64	20	44	2014	118	M	11	9	no	spread	triple bar	right	yes	4	0	67,76	no
London	The Longines FEI World Cup Jumping - London Olympia round A	17.12.2017	CSI-W5*	4	1.60	490	43	15	20.05.2003	S	DSP	218	12	64	20	44	2014	118	M	12	10	no	vertical	vertical	straight	no	0	0	67,76	no
London	The Longines FEI World Cup Jumping - London Olympia round A	17.12.2017	CSI-W5*	4	1.60	490	43	15	20.05.2003	S	DSP	218	12	64	20	44	2014	118	M	13	11a	yes	vertical	vertical	left	yes	4	0	67,76	no
London	The Longines FEI World Cup Jumping - London Olympia round A	17.12.2017	CSI-W5*	4	1.60	490	43	15	20.05.2003	S	DSP	218	12	64	20	44	2014	118	M	14	11b	yes	spread	oxer	left	no	0	0	67,76	no
London	The Longines FEI World Cup Jumping - London Olympia round A	17.12.2017	CSI-W5*	4	1.60	490	43	15	20.05.2003	S	DSP	218	12	64	20	44	2014	118	M	15	12	no	vertical	vertical with water	straight	no	0	0	67,76	no
London	The Longines FEI World Cup Jumping - London Olympia round A	17.12.2017	CSI-W5*	4	1.60	490	43	15	20.05.2003	S	DSP	218	12	64	20	44	2014	118	M	16	13	no	spread	oxer	left	no	0	0	67,76	no
London	The Longines FEI World Cup Jumping - London Olympia round A	17.12.2017	CSI-W5*	4	1.60	490	45	13	20.04.2005	S	WESTF	126	6	71	12	44	2177	93	M	1	1	no	spread	oxer	left	no	0	0	65,03	no
London	The Longines FEI World Cup Jumping - London Olympia round A	17.12.2017	CSI-W5*	4	1.60	490	45	13	20.04.2005	S	WESTF	126	6	71	12	44	2177	93	M	2	2	no	spread	oxer	left	no	0	0	65,03	no
London	The Longines FEI World Cup Jumping - London Olympia round A	17.12.2017	CSI-W5*	4	1.60	490	45	13	20.04.2005	S	WESTF	126	6	71	12	44	2177	93	M	3	3	no	vertical	vertical	left	no	0	0	65,03	no
London	The Longines FEI World Cup Jumping - London Olympia round A	17.12.2017	CSI-W5*	4	1.60	490	45	13	20.04.2005	S	WESTF	126	6	71	12	44	2177	93	M	4	4a	yes	spread	oxer	right	no	0	0	65,03	no
London	The Longines FEI World Cup Jumping - London Olympia round A	17.12.2017	CSI-W5*	4	1.60	490	45	13	20.04.2005	S	WESTF	126	6	71	12	44	2177	93	M	5	4b	yes	vertical	vertical	right	no	0	0	65,03	no
London	The Longines FEI World Cup Jumping - London Olympia round A	17.12.2017	CSI-W5*	4	1.60	490	45	13	20.04.2005	S	WESTF	126	6	71	12	44	2177	93	M	6	4c	yes	spread	oxer	right	no	0	0	65,03	no
London	The Longines FEI World Cup Jumping - London Olympia round A	17.12.2017	CSI-W5*	4	1.60	490	45	13	20.04.2005	S	WESTF	126	6	71	12	44	2177	93	M	7	5	no	vertical	vertical	left	no	0	0	65,03	no
London	The Longines FEI World Cup Jumping - London Olympia round A	17.12.2017	CSI-W5*	4	1.60	490	45	13	20.04.2005	S	WESTF	126	6	71	12	44	2177	93	M	8	6	no	vertical	wall	left	no	0	0	65,03	no
London	The Longines FEI World Cup Jumping - London Olympia round A	17.12.2017	CSI-W5*	4	1.60	490	45	13	20.04.2005	S	WESTF	126	6	71	12	44	2177	93	M	9	7	no	spread	oxer	right	no	0	0	65,03	no
London	The Longines FEI World Cup Jumping - London Olympia round A	17.12.2017	CSI-W5*	4	1.60	490	45	13	20.04.2005	S	WESTF	126	6	71	12	44	2177	93	M	10	8	no	vertical	vertical	right	no	0	0	65,03	no
London	The Longines FEI World Cup Jumping - London Olympia round A	17.12.2017	CSI-W5*	4	1.60	490	45	13	20.04.2005	S	WESTF	126	6	71	12	44	2177	93	M	11	9	no	spread	triple bar	right	yes	4	0	65,03	no
London	The Longines FEI World Cup Jumping - London Olympia round A	17.12.2017	CSI-W5*	4	1.60	490	45	13	20.04.2005	S	WESTF	126	6	71	12	44	2177	93	M	12	10	no	vertical	vertical	straight	no	0	0	65,03	no
London	The Longines FEI World Cup Jumping - London Olympia round A	17.12.2017	CSI-W5*	4	1.60	490	45	13	20.04.2005	S	WESTF	126	6	71	12	44	2177	93	M	13	11a	yes	vertical	vertical	left	no	0	0	65,03	no
London	The Longines FEI World Cup Jumping - London Olympia round A	17.12.2017	CSI-W5*	4	1.60	490	45	13	20.04.2005	S	WESTF	126	6	71	12	44	2177	93	M	14	11b	yes	spread	oxer	left	no	0	0	65,03	no
London	The Longines FEI World Cup Jumping - London Olympia round A	17.12.2017	CSI-W5*	4	1.60	490	45	13	20.04.2005	S	WESTF	126	6	71	12	44	2177	93	M	15	12	no	vertical	vertical with water	straight	no	0	0	65,03	no
London	The Longines FEI World Cup Jumping - London Olympia round A	17.12.2017	CSI-W5*	4	1.60	490	45	13	20.04.2005	S	WESTF	126	6	71	12	44	2177	93	M	16	13	no	spread	oxer	left	no	0	0	65,03	no
London	The Longines FEI World Cup Jumping - London Olympia round A	17.12.2017	CSI-W5*	4	1.60	490	173	13	14.07.2005	G	SF	229	2	74	65	34	1260	61	M	1	1	no	spread	oxer	left	no	0	0	66,46	no
London	The Longines FEI World Cup Jumping - London Olympia round A	17.12.2017	CSI-W5*	4	1.60	490	173	13	14.07.2005	G	SF	229	2	74	65	34	1260	61	M	2	2	no	spread	oxer	left	no	0	0	66,46	no
London	The Longines FEI World Cup Jumping - London Olympia round A	17.12.2017	CSI-W5*	4	1.60	490	173	13	14.07.2005	G	SF	229	2	74	65	34	1260	61	M	3	3	no	vertical	vertical	left	no	0	0	66,46	no
London	The Longines FEI World Cup Jumping - London Olympia round A	17.12.2017	CSI-W5*	4	1.60	490	173	13	14.07.2005	G	SF	229	2	74	65	34	1260	61	M	4	4a	yes	spread	oxer	right	no	0	0	66,46	no
London	The Longines FEI World Cup Jumping - London Olympia round A	17.12.2017	CSI-W5*	4	1.60	490	173	13	14.07.2005	G	SF	229	2	74	65	34	1260	61	M	5	4b	yes	vertical	vertical	right	no	0	0	66,46	no
London	The Longines FEI World Cup Jumping - London Olympia round A	17.12.2017	CSI-W5*	4	1.60	490	173	13	14.07.2005	G	SF	229	2	74	65	34	1260	61	M	6	4c	yes	spread	oxer	right	no	0	0	66,46	no
London	The Longines FEI World Cup Jumping - London Olympia round A	17.12.2017	CSI-W5*	4	1.60	490	173	13	14.07.2005	G	SF	229	2	74	65	34	1260	61	M	7	5	no	vertical	vertical	left	no	0	0	66,46	no
London	The Longines FEI World Cup Jumping - London Olympia round A	17.12.2017	CSI-W5*	4	1.60	490	173	13	14.07.2005	G	SF	229	2	74	65	34	1260	61	M	8	6	no	vertical	wall	left	no	0	0	66,46	no
London	The Longines FEI World Cup Jumping - London Olympia round A	17.12.2017	CSI-W5*	4	1.60	490	173	13	14.07.2005	G	SF	229	2	74	65	34	1260	61	M	9	7	no	spread	oxer	right	no	0	0	66,46	no
London	The Longines FEI World Cup Jumping - London Olympia round A	17.12.2017	CSI-W5*	4	1.60	490	173	13	14.07.2005	G	SF	229	2	74	65	34	1260	61	M	10	8	no	vertical	vertical	right	yes	4	0	66,46	no
London	The Longines FEI World Cup Jumping - London Olympia round A	17.12.2017	CSI-W5*	4	1.60	490	173	13	14.07.2005	G	SF	229	2	74	65	34	1260	61	M	11	9	no	spread	triple bar	right	no	0	0	66,46	no
London	The Longines FEI World Cup Jumping - London Olympia round A	17.12.2017	CSI-W5*	4	1.60	490	173	13	14.07.2005	G	SF	229	2	74	65	34	1260	61	M	12	10	no	vertical	vertical	straight	no	0	0	66,46	no
London	The Longines FEI World Cup Jumping - London Olympia round A	17.12.2017	CSI-W5*	4	1.60	490	173	13	14.07.2005	G	SF	229	2	74	65	34	1260	61	M	13	11a	yes	vertical	vertical	left	no	0	0	66,46	no
London	The Longines FEI World Cup Jumping - London Olympia round A	17.12.2017	CSI-W5*	4	1.60	490	173	13	14.07.2005	G	SF	229	2	74	65	34	1260	61	M	14	11b	yes	spread	oxer	left	no	0	0	66,46	no
London	The Longines FEI World Cup Jumping - London Olympia round A	17.12.2017	CSI-W5*	4	1.60	490	173	13	14.07.2005	G	SF	229	2	74	65	34	1260	61	M	15	12	no	vertical	vertical with water	straight	no	0	0	66,46	no
London	The Longines FEI World Cup Jumping - London Olympia round A	17.12.2017	CSI-W5*	4	1.60	490	173	13	14.07.2005	G	SF	229	2	74	65	34	1260	61	M	16	13	no	spread	oxer	left	no	0	0	66,46	no
London	The Longines FEI World Cup Jumping - London Olympia round A	17.12.2017	CSI-W5*	4	1.60	490	203	11	11.03.2007	M	SF	211	12	85	41	41	2052	191	M	1	1	no	spread	oxer	left	no	0	0	65,7	no
London	The Longines FEI World Cup Jumping - London Olympia round A	17.12.2017	CSI-W5*	4	1.60	490	203	11	11.03.2007	M	SF	211	12	85	41	41	2052	191	M	2	2	no	spread	oxer	left	no	0	0	65,7	no
London	The Longines FEI World Cup Jumping - London Olympia round A	17.12.2017	CSI-W5*	4	1.60	490	203	11	11.03.2007	M	SF	211	12	85	41	41	2052	191	M	3	3	no	vertical	vertical	left	no	0	0	65,7	no
London	The Longines FEI World Cup Jumping - London Olympia round A	17.12.2017	CSI-W5*	4	1.60	490	203	11	11.03.2007	M	SF	211	12	85	41	41	2052	191	M	4	4a	yes	spread	oxer	right	no	0	0	65,7	no
London	The Longines FEI World Cup Jumping - London Olympia round A	17.12.2017	CSI-W5*	4	1.60	490	203	11	11.03.2007	M	SF	211	12	85	41	41	2052	191	M	5	4b	yes	vertical	vertical	right	no	0	0	65,7	no
London	The Longines FEI World Cup Jumping - London Olympia round A	17.12.2017	CSI-W5*	4	1.60	490	203	11	11.03.2007	M	SF	211	12	85	41	41	2052	191	M	6	4c	yes	spread	oxer	right	no	0	0	65,7	no
London	The Longines FEI World Cup Jumping - London Olympia round A	17.12.2017	CSI-W5*	4	1.60	490	203	11	11.03.2007	M	SF	211	12	85	41	41	2052	191	M	7	5	no	vertical	vertical	left	no	0	0	65,7	no
London	The Longines FEI World Cup Jumping - London Olympia round A	17.12.2017	CSI-W5*	4	1.60	490	203	11	11.03.2007	M	SF	211	12	85	41	41	2052	191	M	8	6	no	vertical	wall	left	no	0	0	65,7	no
London	The Longines FEI World Cup Jumping - London Olympia round A	17.12.2017	CSI-W5*	4	1.60	490	203	11	11.03.2007	M	SF	211	12	85	41	41	2052	191	M	9	7	no	spread	oxer	right	no	0	0	65,7	no
London	The Longines FEI World Cup Jumping - London Olympia round A	17.12.2017	CSI-W5*	4	1.60	490	203	11	11.03.2007	M	SF	211	12	85	41	41	2052	191	M	10	8	no	vertical	vertical	right	no	0	0	65,7	no
London	The Longines FEI World Cup Jumping - London Olympia round A	17.12.2017	CSI-W5*	4	1.60	490	203	11	11.03.2007	M	SF	211	12	85	41	41	2052	191	M	11	9	no	spread	triple bar	right	no	0	0	65,7	no
London	The Longines FEI World Cup Jumping - London Olympia round A	17.12.2017	CSI-W5*	4	1.60	490	203	11	11.03.2007	M	SF	211	12	85	41	41	2052	191	M	12	10	no	vertical	vertical	straight	no	0	0	65,7	no
London	The Longines FEI World Cup Jumping - London Olympia round A	17.12.2017	CSI-W5*	4	1.60	490	203	11	11.03.2007	M	SF	211	12	85	41	41	2052	191	M	13	11a	yes	vertical	vertical	left	no	0	0	65,7	no
London	The Longines FEI World Cup Jumping - London Olympia round A	17.12.2017	CSI-W5*	4	1.60	490	203	11	11.03.2007	M	SF	211	12	85	41	41	2052	191	M	14	11b	yes	spread	oxer	left	no	0	0	65,7	no
London	The Longines FEI World Cup Jumping - London Olympia round A	17.12.2017	CSI-W5*	4	1.60	490	203	11	11.03.2007	M	SF	211	12	85	41	41	2052	191	M	15	12	no	vertical	vertical with water	straight	no	0	0	65,7	no
London	The Longines FEI World Cup Jumping - London Olympia round A	17.12.2017	CSI-W5*	4	1.60	490	203	11	11.03.2007	M	SF	211	12	85	41	41	2052	191	M	16	13	no	spread	oxer	left	no	0	0	65,7	no
London	The Longines FEI World Cup Jumping - London Olympia round B	17.12.2017	CSI-W5*	4	1.60	280	203	11	11.03.2007	M	SF	211	12	85	41	41	2052	191	M	1	1	no	spread	oxer	left	no	0	0	36,91	no
London	The Longines FEI World Cup Jumping - London Olympia round B	17.12.2017	CSI-W5*	4	1.60	280	203	11	11.03.2007	M	SF	211	12	85	41	41	2052	191	M	2	2	no	spread	oxer	left	no	0	0	36,91	no
London	The Longines FEI World Cup Jumping - London Olympia round B	17.12.2017	CSI-W5*	4	1.60	280	203	11	11.03.2007	M	SF	211	12	85	41	41	2052	191	M	3	3	no	vertical	vertical	left	no	0	0	36,91	no
London	The Longines FEI World Cup Jumping - London Olympia round B	17.12.2017	CSI-W5*	4	1.60	280	203	11	11.03.2007	M	SF	211	12	85	41	41	2052	191	M	4	4a	yes	vertical	vertical	right	no	0	0	36,91	no
London	The Longines FEI World Cup Jumping - London Olympia round B	17.12.2017	CSI-W5*	4	1.60	280	203	11	11.03.2007	M	SF	211	12	85	41	41	2052	191	M	5	4b	yes	spread	oxer	right	no	0	0	36,91	no
London	The Longines FEI World Cup Jumping - London Olympia round B	17.12.2017	CSI-W5*	4	1.60	280	203	11	11.03.2007	M	SF	211	12	85	41	41	2052	191	M	6	5	no	vertical	wall	left	no	0	0	36,91	no
London	The Longines FEI World Cup Jumping - London Olympia round B	17.12.2017	CSI-W5*	4	1.60	280	203	11	11.03.2007	M	SF	211	12	85	41	41	2052	191	M	7	6	no	vertical	vertical	right	no	0	0	36,91	no
London	The Longines FEI World Cup Jumping - London Olympia round B	17.12.2017	CSI-W5*	4	1.60	280	203	11	11.03.2007	M	SF	211	12	85	41	41	2052	191	M	8	7	no	spread	oxer	right	no	0	0	36,91	no
London	The Longines FEI World Cup Jumping - London Olympia round A	17.12.2017	CSI-W5*	4	1.60	490	147	12	04.05.2006	G	OLDBG	239	5	93	106	55	1594	47	M	1	1	no	spread	oxer	left	no	0	0	68,17	no
London	The Longines FEI World Cup Jumping - London Olympia round A	17.12.2017	CSI-W5*	4	1.60	490	147	12	04.05.2006	G	OLDBG	239	5	93	106	55	1594	47	M	2	2	no	spread	oxer	left	no	0	0	68,17	no
London	The Longines FEI World Cup Jumping - London Olympia round A	17.12.2017	CSI-W5*	4	1.60	490	147	12	04.05.2006	G	OLDBG	239	5	93	106	55	1594	47	M	3	3	no	vertical	vertical	left	no	0	0	68,17	no
London	The Longines FEI World Cup Jumping - London Olympia round A	17.12.2017	CSI-W5*	4	1.60	490	147	12	04.05.2006	G	OLDBG	239	5	93	106	55	1594	47	M	4	4a	yes	spread	oxer	right	no	0	0	68,17	no
London	The Longines FEI World Cup Jumping - London Olympia round A	17.12.2017	CSI-W5*	4	1.60	490	147	12	04.05.2006	G	OLDBG	239	5	93	106	55	1594	47	M	5	4b	yes	vertical	vertical	right	no	0	0	68,17	no
London	The Longines FEI World Cup Jumping - London Olympia round A	17.12.2017	CSI-W5*	4	1.60	490	147	12	04.05.2006	G	OLDBG	239	5	93	106	55	1594	47	M	6	4c	yes	spread	oxer	right	no	0	0	68,17	no
London	The Longines FEI World Cup Jumping - London Olympia round A	17.12.2017	CSI-W5*	4	1.60	490	147	12	04.05.2006	G	OLDBG	239	5	93	106	55	1594	47	M	7	5	no	vertical	vertical	left	no	0	0	68,17	no
London	The Longines FEI World Cup Jumping - London Olympia round A	17.12.2017	CSI-W5*	4	1.60	490	147	12	04.05.2006	G	OLDBG	239	5	93	106	55	1594	47	M	8	6	no	vertical	wall	left	no	0	0	68,17	no
London	The Longines FEI World Cup Jumping - London Olympia round A	17.12.2017	CSI-W5*	4	1.60	490	147	12	04.05.2006	G	OLDBG	239	5	93	106	55	1594	47	M	9	7	no	spread	oxer	right	no	0	0	68,17	no
London	The Longines FEI World Cup Jumping - London Olympia round A	17.12.2017	CSI-W5*	4	1.60	490	147	12	04.05.2006	G	OLDBG	239	5	93	106	55	1594	47	M	10	8	no	vertical	vertical	right	no	0	0	68,17	no
London	The Longines FEI World Cup Jumping - London Olympia round A	17.12.2017	CSI-W5*	4	1.60	490	147	12	04.05.2006	G	OLDBG	239	5	93	106	55	1594	47	M	11	9	no	spread	triple bar	right	no	0	0	68,17	no
London	The Longines FEI World Cup Jumping - London Olympia round A	17.12.2017	CSI-W5*	4	1.60	490	147	12	04.05.2006	G	OLDBG	239	5	93	106	55	1594	47	M	12	10	no	vertical	vertical	straight	yes	4	0	68,17	no
London	The Longines FEI World Cup Jumping - London Olympia round A	17.12.2017	CSI-W5*	4	1.60	490	147	12	04.05.2006	G	OLDBG	239	5	93	106	55	1594	47	M	13	11a	yes	vertical	vertical	left	yes	4	0	68,17	no
London	The Longines FEI World Cup Jumping - London Olympia round A	17.12.2017	CSI-W5*	4	1.60	490	147	12	04.05.2006	G	OLDBG	239	5	93	106	55	1594	47	M	14	11b	yes	spread	oxer	left	no	0	0	68,17	no
London	The Longines FEI World Cup Jumping - London Olympia round A	17.12.2017	CSI-W5*	4	1.60	490	147	12	04.05.2006	G	OLDBG	239	5	93	106	55	1594	47	M	15	12	no	vertical	vertical with water	straight	no	0	0	68,17	no
London	The Longines FEI World Cup Jumping - London Olympia round A	17.12.2017	CSI-W5*	4	1.60	490	147	12	04.05.2006	G	OLDBG	239	5	93	106	55	1594	47	M	16	13	no	spread	oxer	left	no	0	0	68,17	no
London	The Longines FEI World Cup Jumping - London Olympia round A	17.12.2017	CSI-W5*	4	1.60	490	70	10	05.06.2008	G	KWPN	142	5	113	28	35	1547	94	M	1	1	no	spread	oxer	left	no	0	0	84,77	no
London	The Longines FEI World Cup Jumping - London Olympia round A	17.12.2017	CSI-W5*	4	1.60	490	70	10	05.06.2008	G	KWPN	142	5	113	28	35	1547	94	M	2	2	no	spread	oxer	left	no	0	0	84,77	no
London	The Longines FEI World Cup Jumping - London Olympia round A	17.12.2017	CSI-W5*	4	1.60	490	70	10	05.06.2008	G	KWPN	142	5	113	28	35	1547	94	M	3	3	no	vertical	vertical	left	no	0	0	84,77	no
London	The Longines FEI World Cup Jumping - London Olympia round A	17.12.2017	CSI-W5*	4	1.60	490	70	10	05.06.2008	G	KWPN	142	5	113	28	35	1547	94	M	4	4a	yes	spread	oxer	right	no	0	0	84,77	no
London	The Longines FEI World Cup Jumping - London Olympia round A	17.12.2017	CSI-W5*	4	1.60	490	70	10	05.06.2008	G	KWPN	142	5	113	28	35	1547	94	M	5	4b	yes	vertical	vertical	right	no	0	0	84,77	no
London	The Longines FEI World Cup Jumping - London Olympia round A	17.12.2017	CSI-W5*	4	1.60	490	70	10	05.06.2008	G	KWPN	142	5	113	28	35	1547	94	M	6	4c	yes	spread	oxer	right	no	0	0	84,77	no
London	The Longines FEI World Cup Jumping - London Olympia round A	17.12.2017	CSI-W5*	4	1.60	490	70	10	05.06.2008	G	KWPN	142	5	113	28	35	1547	94	M	7	5	no	vertical	vertical	left	yes	4	0	84,77	no
London	The Longines FEI World Cup Jumping - London Olympia round A	17.12.2017	CSI-W5*	4	1.60	490	70	10	05.06.2008	G	KWPN	142	5	113	28	35	1547	94	M	8	6	no	vertical	wall	left	no	0	0	84,77	no
London	The Longines FEI World Cup Jumping - London Olympia round A	17.12.2017	CSI-W5*	4	1.60	490	70	10	05.06.2008	G	KWPN	142	5	113	28	35	1547	94	M	9	7	no	spread	oxer	right	no	0	0	84,77	no
London	The Longines FEI World Cup Jumping - London Olympia round A	17.12.2017	CSI-W5*	4	1.60	490	70	10	05.06.2008	G	KWPN	142	5	113	28	35	1547	94	M	10	8	no	vertical	vertical	right	no	0	0	84,77	no
London	The Longines FEI World Cup Jumping - London Olympia round A	17.12.2017	CSI-W5*	4	1.60	490	70	10	05.06.2008	G	KWPN	142	5	113	28	35	1547	94	M	11	9	no	spread	triple bar	right	no	0	0	84,77	no
London	The Longines FEI World Cup Jumping - London Olympia round A	17.12.2017	CSI-W5*	4	1.60	490	70	10	05.06.2008	G	KWPN	142	5	113	28	35	1547	94	M	12	10	no	vertical	vertical	straight	no	0	0	84,77	no
London	The Longines FEI World Cup Jumping - London Olympia round A	17.12.2017	CSI-W5*	4	1.60	490	70	10	05.06.2008	G	KWPN	142	5	113	28	35	1547	94	M	13	11a	yes	vertical	vertical	left	yes	4	0	84,77	no
London	The Longines FEI World Cup Jumping - London Olympia round A	17.12.2017	CSI-W5*	4	1.60	490	70	10	05.06.2008	G	KWPN	142	5	113	28	35	1547	94	M	14	11b	yes	spread	oxer	left	no	0	0	84,77	no
London	The Longines FEI World Cup Jumping - London Olympia round A	17.12.2017	CSI-W5*	4	1.60	490	70	10	05.06.2008	G	KWPN	142	5	113	28	35	1547	94	M	15	12	no	vertical	vertical with water	straight	no	0	0	84,77	no
London	The Longines FEI World Cup Jumping - London Olympia round A	17.12.2017	CSI-W5*	4	1.60	490	70	10	05.06.2008	G	KWPN	142	5	113	28	35	1547	94	M	16	13	no	spread	oxer	left	yes	4	8	84,77	no
London	The Longines FEI World Cup Jumping - London Olympia round A	17.12.2017	CSI-W5*	4	1.60	490	128	10	04.06.2008	S	BWP	122	1	114	207	57	835	17	M	1	1	no	spread	oxer	left	no	0	0	70,84	no
London	The Longines FEI World Cup Jumping - London Olympia round A	17.12.2017	CSI-W5*	4	1.60	490	128	10	04.06.2008	S	BWP	122	1	114	207	57	835	17	M	2	2	no	spread	oxer	left	no	0	0	70,84	no
London	The Longines FEI World Cup Jumping - London Olympia round A	17.12.2017	CSI-W5*	4	1.60	490	128	10	04.06.2008	S	BWP	122	1	114	207	57	835	17	M	3	3	no	vertical	vertical	left	yes	4	0	70,84	no
London	The Longines FEI World Cup Jumping - London Olympia round A	17.12.2017	CSI-W5*	4	1.60	490	128	10	04.06.2008	S	BWP	122	1	114	207	57	835	17	M	4	4a	yes	spread	oxer	right	no	0	0	70,84	no
London	The Longines FEI World Cup Jumping - London Olympia round A	17.12.2017	CSI-W5*	4	1.60	490	128	10	04.06.2008	S	BWP	122	1	114	207	57	835	17	M	5	4b	yes	vertical	vertical	right	no	0	0	70,84	no
London	The Longines FEI World Cup Jumping - London Olympia round A	17.12.2017	CSI-W5*	4	1.60	490	128	10	04.06.2008	S	BWP	122	1	114	207	57	835	17	M	6	4c	yes	spread	oxer	right	no	0	0	70,84	no
London	The Longines FEI World Cup Jumping - London Olympia round A	17.12.2017	CSI-W5*	4	1.60	490	128	10	04.06.2008	S	BWP	122	1	114	207	57	835	17	M	7	5	no	vertical	vertical	left	no	0	0	70,84	no
London	The Longines FEI World Cup Jumping - London Olympia round A	17.12.2017	CSI-W5*	4	1.60	490	128	10	04.06.2008	S	BWP	122	1	114	207	57	835	17	M	8	6	no	vertical	wall	left	no	0	0	70,84	no
London	The Longines FEI World Cup Jumping - London Olympia round A	17.12.2017	CSI-W5*	4	1.60	490	128	10	04.06.2008	S	BWP	122	1	114	207	57	835	17	M	9	7	no	spread	oxer	right	yes	4	0	70,84	no
London	The Longines FEI World Cup Jumping - London Olympia round A	17.12.2017	CSI-W5*	4	1.60	490	128	10	04.06.2008	S	BWP	122	1	114	207	57	835	17	M	10	8	no	vertical	vertical	right	no	0	0	70,84	no
London	The Longines FEI World Cup Jumping - London Olympia round A	17.12.2017	CSI-W5*	4	1.60	490	128	10	04.06.2008	S	BWP	122	1	114	207	57	835	17	M	11	9	no	spread	triple bar	right	no	0	0	70,84	no
London	The Longines FEI World Cup Jumping - London Olympia round A	17.12.2017	CSI-W5*	4	1.60	490	128	10	04.06.2008	S	BWP	122	1	114	207	57	835	17	M	12	10	no	vertical	vertical	straight	no	0	0	70,84	no
London	The Longines FEI World Cup Jumping - London Olympia round A	17.12.2017	CSI-W5*	4	1.60	490	128	10	04.06.2008	S	BWP	122	1	114	207	57	835	17	M	13	11a	yes	vertical	vertical	left	yes	4	0	70,84	no
London	The Longines FEI World Cup Jumping - London Olympia round A	17.12.2017	CSI-W5*	4	1.60	490	128	10	04.06.2008	S	BWP	122	1	114	207	57	835	17	M	14	11b	yes	spread	oxer	left	yes	4	0	70,84	no
London	The Longines FEI World Cup Jumping - London Olympia round A	17.12.2017	CSI-W5*	4	1.60	490	128	10	04.06.2008	S	BWP	122	1	114	207	57	835	17	M	15	12	no	vertical	vertical with water	straight	no	0	0	70,84	no
London	The Longines FEI World Cup Jumping - London Olympia round A	17.12.2017	CSI-W5*	4	1.60	490	128	10	04.06.2008	S	BWP	122	1	114	207	57	835	17	M	16	13	no	spread	oxer	left	no	0	5	70,84	no
London	The Longines FEI World Cup Jumping - London Olympia round A	17.12.2017	CSI-W5*	4	1.60	490	132	10	04.05.2008	G	BWP	145	7	115	62	39	1976	81	M	1	1	no	spread	oxer	left	no	0	0	68,5	no
London	The Longines FEI World Cup Jumping - London Olympia round A	17.12.2017	CSI-W5*	4	1.60	490	132	10	04.05.2008	G	BWP	145	7	115	62	39	1976	81	M	2	2	no	spread	oxer	left	no	0	0	68,5	no
London	The Longines FEI World Cup Jumping - London Olympia round A	17.12.2017	CSI-W5*	4	1.60	490	132	10	04.05.2008	G	BWP	145	7	115	62	39	1976	81	M	3	3	no	vertical	vertical	left	no	0	0	68,5	no
London	The Longines FEI World Cup Jumping - London Olympia round A	17.12.2017	CSI-W5*	4	1.60	490	132	10	04.05.2008	G	BWP	145	7	115	62	39	1976	81	M	4	4a	yes	spread	oxer	right	no	0	0	68,5	no
London	The Longines FEI World Cup Jumping - London Olympia round A	17.12.2017	CSI-W5*	4	1.60	490	132	10	04.05.2008	G	BWP	145	7	115	62	39	1976	81	M	5	4b	yes	vertical	vertical	right	no	0	0	68,5	no
London	The Longines FEI World Cup Jumping - London Olympia round A	17.12.2017	CSI-W5*	4	1.60	490	132	10	04.05.2008	G	BWP	145	7	115	62	39	1976	81	M	6	4c	yes	spread	oxer	right	no	0	0	68,5	no
London	The Longines FEI World Cup Jumping - London Olympia round A	17.12.2017	CSI-W5*	4	1.60	490	132	10	04.05.2008	G	BWP	145	7	115	62	39	1976	81	M	7	5	no	vertical	vertical	left	no	0	0	68,5	no
London	The Longines FEI World Cup Jumping - London Olympia round A	17.12.2017	CSI-W5*	4	1.60	490	132	10	04.05.2008	G	BWP	145	7	115	62	39	1976	81	M	8	6	no	vertical	wall	left	no	0	0	68,5	no
London	The Longines FEI World Cup Jumping - London Olympia round A	17.12.2017	CSI-W5*	4	1.60	490	132	10	04.05.2008	G	BWP	145	7	115	62	39	1976	81	M	9	7	no	spread	oxer	right	no	0	0	68,5	no
London	The Longines FEI World Cup Jumping - London Olympia round A	17.12.2017	CSI-W5*	4	1.60	490	132	10	04.05.2008	G	BWP	145	7	115	62	39	1976	81	M	10	8	no	vertical	vertical	right	yes	4	0	68,5	no
London	The Longines FEI World Cup Jumping - London Olympia round A	17.12.2017	CSI-W5*	4	1.60	490	132	10	04.05.2008	G	BWP	145	7	115	62	39	1976	81	M	11	9	no	spread	triple bar	right	no	0	0	68,5	no
London	The Longines FEI World Cup Jumping - London Olympia round A	17.12.2017	CSI-W5*	4	1.60	490	132	10	04.05.2008	G	BWP	145	7	115	62	39	1976	81	M	12	10	no	vertical	vertical	straight	no	0	0	68,5	no
London	The Longines FEI World Cup Jumping - London Olympia round A	17.12.2017	CSI-W5*	4	1.60	490	132	10	04.05.2008	G	BWP	145	7	115	62	39	1976	81	M	13	11a	yes	vertical	vertical	left	yes	4	0	68,5	no
London	The Longines FEI World Cup Jumping - London Olympia round A	17.12.2017	CSI-W5*	4	1.60	490	132	10	04.05.2008	G	BWP	145	7	115	62	39	1976	81	M	14	11b	yes	spread	oxer	left	no	0	0	68,5	no
London	The Longines FEI World Cup Jumping - London Olympia round A	17.12.2017	CSI-W5*	4	1.60	490	132	10	04.05.2008	G	BWP	145	7	115	62	39	1976	81	M	15	12	no	vertical	vertical with water	straight	yes	4	0	68,5	no
London	The Longines FEI World Cup Jumping - London Olympia round A	17.12.2017	CSI-W5*	4	1.60	490	132	10	04.05.2008	G	BWP	145	7	115	62	39	1976	81	M	16	13	no	spread	oxer	left	yes	4	0	68,5	no
London	The Longines FEI World Cup Jumping - London Olympia round A	17.12.2017	CSI-W5*	4	1.60	490	47	11	06.04.2007	G	HOLST	136	3	116	29	53	1736	88	F	1	1	no	spread	oxer	left	no	0	0	68,47	no
London	The Longines FEI World Cup Jumping - London Olympia round A	17.12.2017	CSI-W5*	4	1.60	490	47	11	06.04.2007	G	HOLST	136	3	116	29	53	1736	88	F	2	2	no	spread	oxer	left	yes	4	0	68,47	no
London	The Longines FEI World Cup Jumping - London Olympia round A	17.12.2017	CSI-W5*	4	1.60	490	47	11	06.04.2007	G	HOLST	136	3	116	29	53	1736	88	F	3	3	no	vertical	vertical	left	no	0	0	68,47	no
London	The Longines FEI World Cup Jumping - London Olympia round A	17.12.2017	CSI-W5*	4	1.60	490	47	11	06.04.2007	G	HOLST	136	3	116	29	53	1736	88	F	4	4a	yes	spread	oxer	right	no	0	0	68,47	no
London	The Longines FEI World Cup Jumping - London Olympia round A	17.12.2017	CSI-W5*	4	1.60	490	47	11	06.04.2007	G	HOLST	136	3	116	29	53	1736	88	F	5	4b	yes	vertical	vertical	right	no	0	0	68,47	no
London	The Longines FEI World Cup Jumping - London Olympia round A	17.12.2017	CSI-W5*	4	1.60	490	47	11	06.04.2007	G	HOLST	136	3	116	29	53	1736	88	F	6	4c	yes	spread	oxer	right	no	0	0	68,47	no
London	The Longines FEI World Cup Jumping - London Olympia round A	17.12.2017	CSI-W5*	4	1.60	490	47	11	06.04.2007	G	HOLST	136	3	116	29	53	1736	88	F	7	5	no	vertical	vertical	left	no	0	0	68,47	no
London	The Longines FEI World Cup Jumping - London Olympia round A	17.12.2017	CSI-W5*	4	1.60	490	47	11	06.04.2007	G	HOLST	136	3	116	29	53	1736	88	F	8	6	no	vertical	wall	left	no	0	0	68,47	no
London	The Longines FEI World Cup Jumping - London Olympia round A	17.12.2017	CSI-W5*	4	1.60	490	47	11	06.04.2007	G	HOLST	136	3	116	29	53	1736	88	F	9	7	no	spread	oxer	right	no	0	0	68,47	no
London	The Longines FEI World Cup Jumping - London Olympia round A	17.12.2017	CSI-W5*	4	1.60	490	47	11	06.04.2007	G	HOLST	136	3	116	29	53	1736	88	F	10	8	no	vertical	vertical	right	no	0	0	68,47	no
London	The Longines FEI World Cup Jumping - London Olympia round A	17.12.2017	CSI-W5*	4	1.60	490	47	11	06.04.2007	G	HOLST	136	3	116	29	53	1736	88	F	11	9	no	spread	triple bar	right	no	0	0	68,47	no
London	The Longines FEI World Cup Jumping - London Olympia round A	17.12.2017	CSI-W5*	4	1.60	490	47	11	06.04.2007	G	HOLST	136	3	116	29	53	1736	88	F	12	10	no	vertical	vertical	straight	no	0	0	68,47	no
London	The Longines FEI World Cup Jumping - London Olympia round A	17.12.2017	CSI-W5*	4	1.60	490	47	11	06.04.2007	G	HOLST	136	3	116	29	53	1736	88	F	13	11a	yes	vertical	vertical	left	no	0	0	68,47	no
London	The Longines FEI World Cup Jumping - London Olympia round A	17.12.2017	CSI-W5*	4	1.60	490	47	11	06.04.2007	G	HOLST	136	3	116	29	53	1736	88	F	14	11b	yes	spread	oxer	left	no	0	0	68,47	no
London	The Longines FEI World Cup Jumping - London Olympia round A	17.12.2017	CSI-W5*	4	1.60	490	47	11	06.04.2007	G	HOLST	136	3	116	29	53	1736	88	F	15	12	no	vertical	vertical with water	straight	no	0	0	68,47	no
London	The Longines FEI World Cup Jumping - London Olympia round A	17.12.2017	CSI-W5*	4	1.60	490	47	11	06.04.2007	G	HOLST	136	3	116	29	53	1736	88	F	16	13	no	spread	oxer	left	no	0	0	68,47	no
London	The Longines FEI World Cup Jumping - London Olympia round A	17.12.2017	CSI-W5*	4	1.60	490	150	10	26.05.2008	M	ISH	80	7	117	76	30	253	13	F	1	1	no	spread	oxer	left	no	0	0	68,41	no
London	The Longines FEI World Cup Jumping - London Olympia round A	17.12.2017	CSI-W5*	4	1.60	490	150	10	26.05.2008	M	ISH	80	7	117	76	30	253	13	F	2	2	no	spread	oxer	left	no	0	0	68,41	no
London	The Longines FEI World Cup Jumping - London Olympia round A	17.12.2017	CSI-W5*	4	1.60	490	150	10	26.05.2008	M	ISH	80	7	117	76	30	253	13	F	3	3	no	vertical	vertical	left	no	0	0	68,41	no
London	The Longines FEI World Cup Jumping - London Olympia round A	17.12.2017	CSI-W5*	4	1.60	490	150	10	26.05.2008	M	ISH	80	7	117	76	30	253	13	F	4	4a	yes	spread	oxer	right	no	0	0	68,41	no
London	The Longines FEI World Cup Jumping - London Olympia round A	17.12.2017	CSI-W5*	4	1.60	490	150	10	26.05.2008	M	ISH	80	7	117	76	30	253	13	F	5	4b	yes	vertical	vertical	right	no	0	0	68,41	no
London	The Longines FEI World Cup Jumping - London Olympia round A	17.12.2017	CSI-W5*	4	1.60	490	150	10	26.05.2008	M	ISH	80	7	117	76	30	253	13	F	6	4c	yes	spread	oxer	right	no	0	0	68,41	no
London	The Longines FEI World Cup Jumping - London Olympia round A	17.12.2017	CSI-W5*	4	1.60	490	150	10	26.05.2008	M	ISH	80	7	117	76	30	253	13	F	7	5	no	vertical	vertical	left	no	0	0	68,41	no
London	The Longines FEI World Cup Jumping - London Olympia round A	17.12.2017	CSI-W5*	4	1.60	490	150	10	26.05.2008	M	ISH	80	7	117	76	30	253	13	F	8	6	no	vertical	wall	left	no	0	0	68,41	no
London	The Longines FEI World Cup Jumping - London Olympia round A	17.12.2017	CSI-W5*	4	1.60	490	150	10	26.05.2008	M	ISH	80	7	117	76	30	253	13	F	9	7	no	spread	oxer	right	no	0	0	68,41	no
London	The Longines FEI World Cup Jumping - London Olympia round A	17.12.2017	CSI-W5*	4	1.60	490	150	10	26.05.2008	M	ISH	80	7	117	76	30	253	13	F	10	8	no	vertical	vertical	right	yes	4	0	68,41	no
London	The Longines FEI World Cup Jumping - London Olympia round A	17.12.2017	CSI-W5*	4	1.60	490	150	10	26.05.2008	M	ISH	80	7	117	76	30	253	13	F	11	9	no	spread	triple bar	right	no	0	0	68,41	no
London	The Longines FEI World Cup Jumping - London Olympia round A	17.12.2017	CSI-W5*	4	1.60	490	150	10	26.05.2008	M	ISH	80	7	117	76	30	253	13	F	12	10	no	vertical	vertical	straight	no	0	0	68,41	no
London	The Longines FEI World Cup Jumping - London Olympia round A	17.12.2017	CSI-W5*	4	1.60	490	150	10	26.05.2008	M	ISH	80	7	117	76	30	253	13	F	13	11a	yes	vertical	vertical	left	yes	4	0	68,41	no
London	The Longines FEI World Cup Jumping - London Olympia round A	17.12.2017	CSI-W5*	4	1.60	490	150	10	26.05.2008	M	ISH	80	7	117	76	30	253	13	F	14	11b	yes	spread	oxer	left	yes	4	0	68,41	no
London	The Longines FEI World Cup Jumping - London Olympia round A	17.12.2017	CSI-W5*	4	1.60	490	150	10	26.05.2008	M	ISH	80	7	117	76	30	253	13	F	15	12	no	vertical	vertical with water	straight	no	0	0	68,41	no
London	The Longines FEI World Cup Jumping - London Olympia round A	17.12.2017	CSI-W5*	4	1.60	490	150	10	26.05.2008	M	ISH	80	7	117	76	30	253	13	F	16	13	no	spread	oxer	left	no	0	0	68,41	no
London	The Longines FEI World Cup Jumping - London Olympia round A	17.12.2017	CSI-W5*	4	1.60	490	202	9	09.08.2009	S	AES	64	5	118	69	44	2264	366	F	1	1	no	spread	oxer	left	no	0	0	68,41	no
London	The Longines FEI World Cup Jumping - London Olympia round A	17.12.2017	CSI-W5*	4	1.60	490	202	9	09.08.2009	S	AES	64	5	118	69	44	2264	366	F	2	2	no	spread	oxer	left	no	0	0	68,41	no
London	The Longines FEI World Cup Jumping - London Olympia round A	17.12.2017	CSI-W5*	4	1.60	490	202	9	09.08.2009	S	AES	64	5	118	69	44	2264	366	F	3	3	no	vertical	vertical	left	no	0	0	68,41	no
London	The Longines FEI World Cup Jumping - London Olympia round A	17.12.2017	CSI-W5*	4	1.60	490	202	9	09.08.2009	S	AES	64	5	118	69	44	2264	366	F	4	4a	yes	spread	oxer	right	no	0	0	68,41	no
London	The Longines FEI World Cup Jumping - London Olympia round A	17.12.2017	CSI-W5*	4	1.60	490	202	9	09.08.2009	S	AES	64	5	118	69	44	2264	366	F	5	4b	yes	vertical	vertical	right	no	0	0	68,41	no
London	The Longines FEI World Cup Jumping - London Olympia round A	17.12.2017	CSI-W5*	4	1.60	490	202	9	09.08.2009	S	AES	64	5	118	69	44	2264	366	F	6	4c	yes	spread	oxer	right	no	0	0	68,41	no
London	The Longines FEI World Cup Jumping - London Olympia round A	17.12.2017	CSI-W5*	4	1.60	490	202	9	09.08.2009	S	AES	64	5	118	69	44	2264	366	F	7	5	no	vertical	vertical	left	no	0	0	68,41	no
London	The Longines FEI World Cup Jumping - London Olympia round A	17.12.2017	CSI-W5*	4	1.60	490	202	9	09.08.2009	S	AES	64	5	118	69	44	2264	366	F	8	6	no	vertical	wall	left	no	0	0	68,41	no
London	The Longines FEI World Cup Jumping - London Olympia round A	17.12.2017	CSI-W5*	4	1.60	490	202	9	09.08.2009	S	AES	64	5	118	69	44	2264	366	F	9	7	no	spread	oxer	right	no	0	0	68,41	no
London	The Longines FEI World Cup Jumping - London Olympia round A	17.12.2017	CSI-W5*	4	1.60	490	202	9	09.08.2009	S	AES	64	5	118	69	44	2264	366	F	10	8	no	vertical	vertical	right	no	0	0	68,41	no
London	The Longines FEI World Cup Jumping - London Olympia round A	17.12.2017	CSI-W5*	4	1.60	490	202	9	09.08.2009	S	AES	64	5	118	69	44	2264	366	F	11	9	no	spread	triple bar	right	no	0	0	68,41	no
London	The Longines FEI World Cup Jumping - London Olympia round A	17.12.2017	CSI-W5*	4	1.60	490	202	9	09.08.2009	S	AES	64	5	118	69	44	2264	366	F	12	10	no	vertical	vertical	straight	yes	4	0	68,41	no
London	The Longines FEI World Cup Jumping - London Olympia round A	17.12.2017	CSI-W5*	4	1.60	490	202	9	09.08.2009	S	AES	64	5	118	69	44	2264	366	F	13	11a	yes	vertical	vertical	left	no	0	0	68,41	no
London	The Longines FEI World Cup Jumping - London Olympia round A	17.12.2017	CSI-W5*	4	1.60	490	202	9	09.08.2009	S	AES	64	5	118	69	44	2264	366	F	14	11b	yes	spread	oxer	left	no	0	0	68,41	no
London	The Longines FEI World Cup Jumping - London Olympia round A	17.12.2017	CSI-W5*	4	1.60	490	202	9	09.08.2009	S	AES	64	5	118	69	44	2264	366	F	15	12	no	vertical	vertical with water	straight	no	0	0	68,41	no
London	The Longines FEI World Cup Jumping - London Olympia round A	17.12.2017	CSI-W5*	4	1.60	490	202	9	09.08.2009	S	AES	64	5	118	69	44	2264	366	F	16	13	no	spread	oxer	left	no	0	0	68,41	no
London	The Longines FEI World Cup Jumping - London Olympia round A	17.12.2017	CSI-W5*	4	1.60	490	209	17	28.04.2001	M	SHBGB	209	4	119	18	33	1574	93	M	1	1	no	spread	oxer	left	no	0	0	67,58	no
London	The Longines FEI World Cup Jumping - London Olympia round A	17.12.2017	CSI-W5*	4	1.60	490	209	17	28.04.2001	M	SHBGB	209	4	119	18	33	1574	93	M	2	2	no	spread	oxer	left	no	0	0	67,58	no
London	The Longines FEI World Cup Jumping - London Olympia round A	17.12.2017	CSI-W5*	4	1.60	490	209	17	28.04.2001	M	SHBGB	209	4	119	18	33	1574	93	M	3	3	no	vertical	vertical	left	no	0	0	67,58	no
London	The Longines FEI World Cup Jumping - London Olympia round A	17.12.2017	CSI-W5*	4	1.60	490	209	17	28.04.2001	M	SHBGB	209	4	119	18	33	1574	93	M	4	4a	yes	spread	oxer	right	no	0	0	67,58	no
London	The Longines FEI World Cup Jumping - London Olympia round A	17.12.2017	CSI-W5*	4	1.60	490	209	17	28.04.2001	M	SHBGB	209	4	119	18	33	1574	93	M	5	4b	yes	vertical	vertical	right	no	0	0	67,58	no
London	The Longines FEI World Cup Jumping - London Olympia round A	17.12.2017	CSI-W5*	4	1.60	490	209	17	28.04.2001	M	SHBGB	209	4	119	18	33	1574	93	M	6	4c	yes	spread	oxer	right	no	0	0	67,58	no
London	The Longines FEI World Cup Jumping - London Olympia round A	17.12.2017	CSI-W5*	4	1.60	490	209	17	28.04.2001	M	SHBGB	209	4	119	18	33	1574	93	M	7	5	no	vertical	vertical	left	no	0	0	67,58	no
London	The Longines FEI World Cup Jumping - London Olympia round A	17.12.2017	CSI-W5*	4	1.60	490	209	17	28.04.2001	M	SHBGB	209	4	119	18	33	1574	93	M	8	6	no	vertical	wall	left	no	0	0	67,58	no
London	The Longines FEI World Cup Jumping - London Olympia round A	17.12.2017	CSI-W5*	4	1.60	490	209	17	28.04.2001	M	SHBGB	209	4	119	18	33	1574	93	M	9	7	no	spread	oxer	right	no	0	0	67,58	no
London	The Longines FEI World Cup Jumping - London Olympia round A	17.12.2017	CSI-W5*	4	1.60	490	209	17	28.04.2001	M	SHBGB	209	4	119	18	33	1574	93	M	10	8	no	vertical	vertical	right	no	0	0	67,58	no
London	The Longines FEI World Cup Jumping - London Olympia round A	17.12.2017	CSI-W5*	4	1.60	490	209	17	28.04.2001	M	SHBGB	209	4	119	18	33	1574	93	M	11	9	no	spread	triple bar	right	no	0	0	67,58	no
London	The Longines FEI World Cup Jumping - London Olympia round A	17.12.2017	CSI-W5*	4	1.60	490	209	17	28.04.2001	M	SHBGB	209	4	119	18	33	1574	93	M	12	10	no	vertical	vertical	straight	no	0	0	67,58	no
London	The Longines FEI World Cup Jumping - London Olympia round A	17.12.2017	CSI-W5*	4	1.60	490	209	17	28.04.2001	M	SHBGB	209	4	119	18	33	1574	93	M	13	11a	yes	vertical	vertical	left	no	0	0	67,58	no
London	The Longines FEI World Cup Jumping - London Olympia round A	17.12.2017	CSI-W5*	4	1.60	490	209	17	28.04.2001	M	SHBGB	209	4	119	18	33	1574	93	M	14	11b	yes	spread	oxer	left	no	0	0	67,58	no
London	The Longines FEI World Cup Jumping - London Olympia round A	17.12.2017	CSI-W5*	4	1.60	490	209	17	28.04.2001	M	SHBGB	209	4	119	18	33	1574	93	M	15	12	no	vertical	vertical with water	straight	no	0	0	67,58	no
London	The Longines FEI World Cup Jumping - London Olympia round A	17.12.2017	CSI-W5*	4	1.60	490	209	17	28.04.2001	M	SHBGB	209	4	119	18	33	1574	93	M	16	13	no	spread	oxer	left	no	0	0	67,58	no
London	The Longines FEI World Cup Jumping - London Olympia round B	17.12.2017	CSI-W5*	4	1.60	280	209	17	28.04.2001	M	SHBGB	209	4	119	18	33	1574	93	M	1	1	no	spread	oxer	left	no	0	0	37,34	no
London	The Longines FEI World Cup Jumping - London Olympia round B	17.12.2017	CSI-W5*	4	1.60	280	209	17	28.04.2001	M	SHBGB	209	4	119	18	33	1574	93	M	2	2	no	spread	oxer	left	no	0	0	37,34	no
London	The Longines FEI World Cup Jumping - London Olympia round B	17.12.2017	CSI-W5*	4	1.60	280	209	17	28.04.2001	M	SHBGB	209	4	119	18	33	1574	93	M	3	3	no	vertical	vertical	left	no	0	0	37,34	no
London	The Longines FEI World Cup Jumping - London Olympia round B	17.12.2017	CSI-W5*	4	1.60	280	209	17	28.04.2001	M	SHBGB	209	4	119	18	33	1574	93	M	4	4a	yes	vertical	vertical	right	no	0	0	37,34	no
London	The Longines FEI World Cup Jumping - London Olympia round B	17.12.2017	CSI-W5*	4	1.60	280	209	17	28.04.2001	M	SHBGB	209	4	119	18	33	1574	93	M	5	4b	yes	spread	oxer	right	no	0	0	37,34	no
London	The Longines FEI World Cup Jumping - London Olympia round B	17.12.2017	CSI-W5*	4	1.60	280	209	17	28.04.2001	M	SHBGB	209	4	119	18	33	1574	93	M	6	5	no	vertical	wall	left	no	0	0	37,34	no
London	The Longines FEI World Cup Jumping - London Olympia round B	17.12.2017	CSI-W5*	4	1.60	280	209	17	28.04.2001	M	SHBGB	209	4	119	18	33	1574	93	M	7	6	no	vertical	vertical	right	no	0	0	37,34	no
London	The Longines FEI World Cup Jumping - London Olympia round B	17.12.2017	CSI-W5*	4	1.60	280	209	17	28.04.2001	M	SHBGB	209	4	119	18	33	1574	93	M	8	7	no	spread	oxer	right	no	0	0	37,34	no
London	The Longines FEI World Cup Jumping - London Olympia round A	17.12.2017	CSI-W5*	4	1.60	490	148	10	28.06.2008	G	Bezplemenné	125	0	120	550	27	986	18	F	1	1	no	spread	oxer	left	no	0	0	67,333	no
London	The Longines FEI World Cup Jumping - London Olympia round A	17.12.2017	CSI-W5*	4	1.60	490	148	10	28.06.2008	G	Bezplemenné	125	0	120	550	27	986	18	F	2	2	no	spread	oxer	left	no	0	0	67,333	no
London	The Longines FEI World Cup Jumping - London Olympia round A	17.12.2017	CSI-W5*	4	1.60	490	148	10	28.06.2008	G	Bezplemenné	125	0	120	550	27	986	18	F	3	3	no	vertical	vertical	left	no	0	0	67,333	no
London	The Longines FEI World Cup Jumping - London Olympia round A	17.12.2017	CSI-W5*	4	1.60	490	148	10	28.06.2008	G	Bezplemenné	125	0	120	550	27	986	18	F	4	4a	yes	spread	oxer	right	no	0	0	67,333	no
London	The Longines FEI World Cup Jumping - London Olympia round A	17.12.2017	CSI-W5*	4	1.60	490	148	10	28.06.2008	G	Bezplemenné	125	0	120	550	27	986	18	F	5	4b	yes	vertical	vertical	right	no	0	0	67,333	no
London	The Longines FEI World Cup Jumping - London Olympia round A	17.12.2017	CSI-W5*	4	1.60	490	148	10	28.06.2008	G	Bezplemenné	125	0	120	550	27	986	18	F	6	4c	yes	spread	oxer	right	no	0	0	67,333	no
London	The Longines FEI World Cup Jumping - London Olympia round A	17.12.2017	CSI-W5*	4	1.60	490	148	10	28.06.2008	G	Bezplemenné	125	0	120	550	27	986	18	F	7	5	no	vertical	vertical	left	no	0	0	67,333	no
London	The Longines FEI World Cup Jumping - London Olympia round A	17.12.2017	CSI-W5*	4	1.60	490	148	10	28.06.2008	G	Bezplemenné	125	0	120	550	27	986	18	F	8	6	no	vertical	wall	left	no	0	0	67,333	no
London	The Longines FEI World Cup Jumping - London Olympia round A	17.12.2017	CSI-W5*	4	1.60	490	148	10	28.06.2008	G	Bezplemenné	125	0	120	550	27	986	18	F	9	7	no	spread	oxer	right	no	0	0	67,333	no
London	The Longines FEI World Cup Jumping - London Olympia round A	17.12.2017	CSI-W5*	4	1.60	490	148	10	28.06.2008	G	Bezplemenné	125	0	120	550	27	986	18	F	10	8	no	vertical	vertical	right	no	0	0	67,333	no
London	The Longines FEI World Cup Jumping - London Olympia round A	17.12.2017	CSI-W5*	4	1.60	490	148	10	28.06.2008	G	Bezplemenné	125	0	120	550	27	986	18	F	11	9	no	spread	triple bar	right	no	0	0	67,333	no
London	The Longines FEI World Cup Jumping - London Olympia round A	17.12.2017	CSI-W5*	4	1.60	490	148	10	28.06.2008	G	Bezplemenné	125	0	120	550	27	986	18	F	12	10	no	vertical	vertical	straight	yes	4	0	67,333	no
London	The Longines FEI World Cup Jumping - London Olympia round A	17.12.2017	CSI-W5*	4	1.60	490	148	10	28.06.2008	G	Bezplemenné	125	0	120	550	27	986	18	F	13	11a	yes	vertical	vertical	left	yes	4	0	67,333	no
London	The Longines FEI World Cup Jumping - London Olympia round A	17.12.2017	CSI-W5*	4	1.60	490	148	10	28.06.2008	G	Bezplemenné	125	0	120	550	27	986	18	F	14	11b	yes	spread	oxer	left	yes	4	0	67,333	no
London	The Longines FEI World Cup Jumping - London Olympia round A	17.12.2017	CSI-W5*	4	1.60	490	148	10	28.06.2008	G	Bezplemenné	125	0	120	550	27	986	18	F	15	12	no	vertical	vertical with water	straight	no	0	0	67,333	no
London	The Longines FEI World Cup Jumping - London Olympia round A	17.12.2017	CSI-W5*	4	1.60	490	148	10	28.06.2008	G	Bezplemenné	125	0	120	550	27	986	18	F	16	13	no	spread	oxer	left	no	0	0	67,333	no
London	The Longines FEI World Cup Jumping - London Olympia round A	17.12.2017	CSI-W5*	4	1.60	490	221	14	30.04.2004	G	KWPN	281	20	121	2	38	1958	103	M	1	1	no	spread	oxer	left	no	0	0	66,78	no
London	The Longines FEI World Cup Jumping - London Olympia round A	17.12.2017	CSI-W5*	4	1.60	490	221	14	30.04.2004	G	KWPN	281	20	121	2	38	1958	103	M	2	2	no	spread	oxer	left	no	0	0	66,78	no
London	The Longines FEI World Cup Jumping - London Olympia round A	17.12.2017	CSI-W5*	4	1.60	490	221	14	30.04.2004	G	KWPN	281	20	121	2	38	1958	103	M	3	3	no	vertical	vertical	left	no	0	0	66,78	no
London	The Longines FEI World Cup Jumping - London Olympia round A	17.12.2017	CSI-W5*	4	1.60	490	221	14	30.04.2004	G	KWPN	281	20	121	2	38	1958	103	M	4	4a	yes	spread	oxer	right	no	0	0	66,78	no
London	The Longines FEI World Cup Jumping - London Olympia round A	17.12.2017	CSI-W5*	4	1.60	490	221	14	30.04.2004	G	KWPN	281	20	121	2	38	1958	103	M	5	4b	yes	vertical	vertical	right	no	0	0	66,78	no
London	The Longines FEI World Cup Jumping - London Olympia round A	17.12.2017	CSI-W5*	4	1.60	490	221	14	30.04.2004	G	KWPN	281	20	121	2	38	1958	103	M	6	4c	yes	spread	oxer	right	no	0	0	66,78	no
London	The Longines FEI World Cup Jumping - London Olympia round A	17.12.2017	CSI-W5*	4	1.60	490	221	14	30.04.2004	G	KWPN	281	20	121	2	38	1958	103	M	7	5	no	vertical	vertical	left	no	0	0	66,78	no
London	The Longines FEI World Cup Jumping - London Olympia round A	17.12.2017	CSI-W5*	4	1.60	490	221	14	30.04.2004	G	KWPN	281	20	121	2	38	1958	103	M	8	6	no	vertical	wall	left	no	0	0	66,78	no
London	The Longines FEI World Cup Jumping - London Olympia round A	17.12.2017	CSI-W5*	4	1.60	490	221	14	30.04.2004	G	KWPN	281	20	121	2	38	1958	103	M	9	7	no	spread	oxer	right	yes	4	0	66,78	no
London	The Longines FEI World Cup Jumping - London Olympia round A	17.12.2017	CSI-W5*	4	1.60	490	221	14	30.04.2004	G	KWPN	281	20	121	2	38	1958	103	M	10	8	no	vertical	vertical	right	no	0	0	66,78	no
London	The Longines FEI World Cup Jumping - London Olympia round A	17.12.2017	CSI-W5*	4	1.60	490	221	14	30.04.2004	G	KWPN	281	20	121	2	38	1958	103	M	11	9	no	spread	triple bar	right	no	0	0	66,78	no
London	The Longines FEI World Cup Jumping - London Olympia round A	17.12.2017	CSI-W5*	4	1.60	490	221	14	30.04.2004	G	KWPN	281	20	121	2	38	1958	103	M	12	10	no	vertical	vertical	straight	no	0	0	66,78	no
London	The Longines FEI World Cup Jumping - London Olympia round A	17.12.2017	CSI-W5*	4	1.60	490	221	14	30.04.2004	G	KWPN	281	20	121	2	38	1958	103	M	13	11a	yes	vertical	vertical	left	no	0	0	66,78	no
London	The Longines FEI World Cup Jumping - London Olympia round A	17.12.2017	CSI-W5*	4	1.60	490	221	14	30.04.2004	G	KWPN	281	20	121	2	38	1958	103	M	14	11b	yes	spread	oxer	left	no	0	0	66,78	no
London	The Longines FEI World Cup Jumping - London Olympia round A	17.12.2017	CSI-W5*	4	1.60	490	221	14	30.04.2004	G	KWPN	281	20	121	2	38	1958	103	M	15	12	no	vertical	vertical with water	straight	no	0	0	66,78	no
London	The Longines FEI World Cup Jumping - London Olympia round A	17.12.2017	CSI-W5*	4	1.60	490	221	14	30.04.2004	G	KWPN	281	20	121	2	38	1958	103	M	16	13	no	spread	oxer	left	no	0	0	66,78	no
London	The Longines FEI World Cup Jumping - London Olympia round A	17.12.2017	CSI-W5*	4	1.60	490	86	15	01.04.2003	G	SWB	263	9	122	156	29	1538	43	M	1	1	no	spread	oxer	left	no	0	0	65,87	no
London	The Longines FEI World Cup Jumping - London Olympia round A	17.12.2017	CSI-W5*	4	1.60	490	86	15	01.04.2003	G	SWB	263	9	122	156	29	1538	43	M	2	2	no	spread	oxer	left	no	0	0	65,87	no
London	The Longines FEI World Cup Jumping - London Olympia round A	17.12.2017	CSI-W5*	4	1.60	490	86	15	01.04.2003	G	SWB	263	9	122	156	29	1538	43	M	3	3	no	vertical	vertical	left	no	0	0	65,87	no
London	The Longines FEI World Cup Jumping - London Olympia round A	17.12.2017	CSI-W5*	4	1.60	490	86	15	01.04.2003	G	SWB	263	9	122	156	29	1538	43	M	4	4a	yes	spread	oxer	right	no	0	0	65,87	no
London	The Longines FEI World Cup Jumping - London Olympia round A	17.12.2017	CSI-W5*	4	1.60	490	86	15	01.04.2003	G	SWB	263	9	122	156	29	1538	43	M	5	4b	yes	vertical	vertical	right	no	0	0	65,87	no
London	The Longines FEI World Cup Jumping - London Olympia round A	17.12.2017	CSI-W5*	4	1.60	490	86	15	01.04.2003	G	SWB	263	9	122	156	29	1538	43	M	6	4c	yes	spread	oxer	right	no	0	0	65,87	no
London	The Longines FEI World Cup Jumping - London Olympia round A	17.12.2017	CSI-W5*	4	1.60	490	86	15	01.04.2003	G	SWB	263	9	122	156	29	1538	43	M	7	5	no	vertical	vertical	left	no	0	0	65,87	no
London	The Longines FEI World Cup Jumping - London Olympia round A	17.12.2017	CSI-W5*	4	1.60	490	86	15	01.04.2003	G	SWB	263	9	122	156	29	1538	43	M	8	6	no	vertical	wall	left	no	0	0	65,87	no
London	The Longines FEI World Cup Jumping - London Olympia round A	17.12.2017	CSI-W5*	4	1.60	490	86	15	01.04.2003	G	SWB	263	9	122	156	29	1538	43	M	9	7	no	spread	oxer	right	no	0	0	65,87	no
London	The Longines FEI World Cup Jumping - London Olympia round A	17.12.2017	CSI-W5*	4	1.60	490	86	15	01.04.2003	G	SWB	263	9	122	156	29	1538	43	M	10	8	no	vertical	vertical	right	yes	4	0	65,87	no
London	The Longines FEI World Cup Jumping - London Olympia round A	17.12.2017	CSI-W5*	4	1.60	490	86	15	01.04.2003	G	SWB	263	9	122	156	29	1538	43	M	11	9	no	spread	triple bar	right	no	0	0	65,87	no
London	The Longines FEI World Cup Jumping - London Olympia round A	17.12.2017	CSI-W5*	4	1.60	490	86	15	01.04.2003	G	SWB	263	9	122	156	29	1538	43	M	12	10	no	vertical	vertical	straight	no	0	0	65,87	no
London	The Longines FEI World Cup Jumping - London Olympia round A	17.12.2017	CSI-W5*	4	1.60	490	86	15	01.04.2003	G	SWB	263	9	122	156	29	1538	43	M	13	11a	yes	vertical	vertical	left	no	0	0	65,87	no
London	The Longines FEI World Cup Jumping - London Olympia round A	17.12.2017	CSI-W5*	4	1.60	490	86	15	01.04.2003	G	SWB	263	9	122	156	29	1538	43	M	14	11b	yes	spread	oxer	left	no	0	0	65,87	no

London	The Longines FEI World Cup Jumping - London Olympia round A	17.12.2017	CSI-W5*	4	1.60	490	86	15	01.04.2003	G	SWB	263	9	122	156	29	1538	43	M	15	12	no	vertical	vertical with water	straight	no	0	0	65,87	no
London	The Longines FEI World Cup Jumping - London Olympia round A	17.12.2017	CSI-W5*	4	1.60	490	86	15	01.04.2003	G	SWB	263	9	122	156	29	1538	43	M	16	13	no	spread	oxer	left	no	0	0	65,87	no
London	The Longines FEI World Cup Jumping - London Olympia round A	17.12.2017	CSI-W5*	4	1.60	490	175	13	01.06.2005	S	SF	137	9	123	131	47	1970	153	M	1	1	no	spread	oxer	left	no	0	0	65,24	no
London	The Longines FEI World Cup Jumping - London Olympia round A	17.12.2017	CSI-W5*	4	1.60	490	175	13	01.06.2005	S	SF	137	9	123	131	47	1970	153	M	2	2	no	spread	oxer	left	no	0	0	65,24	no
London	The Longines FEI World Cup Jumping - London Olympia round A	17.12.2017	CSI-W5*	4	1.60	490	175	13	01.06.2005	S	SF	137	9	123	131	47	1970	153	M	3	3	no	vertical	vertical	left	no	0	0	65,24	no
London	The Longines FEI World Cup Jumping - London Olympia round A	17.12.2017	CSI-W5*	4	1.60	490	175	13	01.06.2005	S	SF	137	9	123	131	47	1970	153	M	4	4a	yes	spread	oxer	right	no	0	0	65,24	no
London	The Longines FEI World Cup Jumping - London Olympia round A	17.12.2017	CSI-W5*	4	1.60	490	175	13	01.06.2005	S	SF	137	9	123	131	47	1970	153	M	5	4b	yes	vertical	vertical	right	no	0	0	65,24	no
London	The Longines FEI World Cup Jumping - London Olympia round A	17.12.2017	CSI-W5*	4	1.60	490	175	13	01.06.2005	S	SF	137	9	123	131	47	1970	153	M	6	4c	yes	spread	oxer	right	no	0	0	65,24	no
London	The Longines FEI World Cup Jumping - London Olympia round A	17.12.2017	CSI-W5*	4	1.60	490	175	13	01.06.2005	S	SF	137	9	123	131	47	1970	153	M	7	5	no	vertical	vertical	left	no	0	0	65,24	no
London	The Longines FEI World Cup Jumping - London Olympia round A	17.12.2017	CSI-W5*	4	1.60	490	175	13	01.06.2005	S	SF	137	9	123	131	47	1970	153	M	8	6	no	vertical	wall	left	no	0	0	65,24	no
London	The Longines FEI World Cup Jumping - London Olympia round A	17.12.2017	CSI-W5*	4	1.60	490	175	13	01.06.2005	S	SF	137	9	123	131	47	1970	153	M	9	7	no	spread	oxer	right	no	0	0	65,24	no
London	The Longines FEI World Cup Jumping - London Olympia round A	17.12.2017	CSI-W5*	4	1.60	490	175	13	01.06.2005	S	SF	137	9	123	131	47	1970	153	M	10	8	no	vertical	vertical	right	yes	4	0	65,24	no
London	The Longines FEI World Cup Jumping - London Olympia round A	17.12.2017	CSI-W5*	4	1.60	490	175	13	01.06.2005	S	SF	137	9	123	131	47	1970	153	M	11	9	no	spread	triple bar	right	no	0	0	65,24	no
London	The Longines FEI World Cup Jumping - London Olympia round A	17.12.2017	CSI-W5*	4	1.60	490	175	13	01.06.2005	S	SF	137	9	123	131	47	1970	153	M	12	10	no	vertical	vertical	straight	yes	4	0	65,24	no
London	The Longines FEI World Cup Jumping - London Olympia round A	17.12.2017	CSI-W5*	4	1.60	490	175	13	01.06.2005	S	SF	137	9	123	131	47	1970	153	M	13	11a	yes	vertical	vertical	left	yes	4	0	65,24	no
London	The Longines FEI World Cup Jumping - London Olympia round A	17.12.2017	CSI-W5*	4	1.60	490	175	13	01.06.2005	S	SF	137	9	123	131	47	1970	153	M	14	11b	yes	spread	oxer	left	yes	4	0	65,24	no
London	The Longines FEI World Cup Jumping - London Olympia round A	17.12.2017	CSI-W5*	4	1.60	490	175	13	01.06.2005	S	SF	137	9	123	131	47	1970	153	M	15	12	no	vertical	vertical with water	straight	yes	4	0	65,24	no
London	The Longines FEI World Cup Jumping - London Olympia round A	17.12.2017	CSI-W5*	4	1.60	490	175	13	01.06.2005	S	SF	137	9	123	131	47	1970	153	M	16	13	no	spread	oxer	left	no	0	0	65,24	no
Mechelen	The Longines FEI World Cup Jumping - Mechelen round A	30.12.2017	CSI-W5*	3	1.60	390	54	12	17.03.2006	M	WESTF	171	6	1	19	38	2803	179	M	1	1	no	spread	oxer	left	no	0	0	0,00	yes
Mechelen	The Longines FEI World Cup Jumping - Mechelen round A	30.12.2017	CSI-W5*	3	1.60	390	54	12	17.03.2006	M	WESTF	171	6	1	19	38	2803	179	M	2	2	no	vertical	vertical	straight	no	0	0	0,00	yes
Mechelen	The Longines FEI World Cup Jumping - Mechelen round A	30.12.2017	CSI-W5*	3	1.60	390	54	12	17.03.2006	M	WESTF	171	6	1	19	38	2803	179	M	3	3	no	spread	oxer	right	no	0	0	0,00	yes
Mechelen	The Longines FEI World Cup Jumping - Mechelen round A	30.12.2017	CSI-W5*	3	1.60	390	54	12	17.03.2006	M	WESTF	171	6	1	19	38	2803	179	M	4	4	no	vertical	vertical	right	no	0	0	0,00	yes
Mechelen	The Longines FEI World Cup Jumping - Mechelen round A	30.12.2017	CSI-W5*	3	1.60	390	54	12	17.03.2006	M	WESTF	171	6	1	19	38	2803	179	M	5	5a	yes	spread	oxer	left	yes	4	0	0,00	yes
Mechelen	The Longines FEI World Cup Jumping - Mechelen round A	30.12.2017	CSI-W5*	3	1.60	390	54	12	17.03.2006	M	WESTF	171	6	1	19	38	2803	179	M	6	5b	yes	vertical	vertical	left	no	0	0	0,00	yes
Mechelen	The Longines FEI World Cup Jumping - Mechelen round A	30.12.2017	CSI-W5*	3	1.60	390	54	12	17.03.2006	M	WESTF	171	6	1	19	38	2803	179	M	7	6	no	spread	oxer	straight	no	0	0	0,00	yes
Mechelen	The Longines FEI World Cup Jumping - Mechelen round A	30.12.2017	CSI-W5*	3	1.60	390	54	12	17.03.2006	M	WESTF	171	6	1	19	38	2803	179	M	8	7	no	vertical	vertical with water	left	no	0	0	0,00	yes
Mechelen	The Longines FEI World Cup Jumping - Mechelen round A	30.12.2017	CSI-W5*	3	1.60	390	54	12	17.03.2006	M	WESTF	171	6	1	19	38	2803	179	M	9	8	no	spread	triple bar	left	no	0	0	0,00	yes
Mechelen	The Longines FEI World Cup Jumping - Mechelen round A	30.12.2017	CSI-W5*	3	1.60	390	54	12	17.03.2006	M	WESTF	171	6	1	19	38	2803	179	M	10	9	no	vertical	vertical	left	no	0	0	0,00	yes
Mechelen	The Longines FEI World Cup Jumping - Mechelen round A	30.12.2017	CSI-W5*	3	1.60	390	54	12	17.03.2006	M	WESTF	171	6	1	19	38	2803	179	M	11	10	no	spread	oxer	right	no	0	0	0,00	yes
Mechelen	The Longines FEI World Cup Jumping - Mechelen round A	30.12.2017	CSI-W5*	3	1.60	390	54	12	17.03.2006	M	WESTF	171	6	1	19	38	2803	179	M	12	11a	yes	vertical	vertical	right	no	0	0	0,00	yes
Mechelen	The Longines FEI World Cup Jumping - Mechelen round A	30.12.2017	CSI-W5*	3	1.60	390	54	12	17.03.2006	M	WESTF	171	6	1	19	38	2803	179	M	13	11b	yes	spread	oxer	right	yes	4	0	0,00	yes
Mechelen	The Longines FEI World Cup Jumping - Mechelen round A	30.12.2017	CSI-W5*	3	1.60	390	54	12	17.03.2006	M	WESTF	171	6	1	19	38	2803	179	M	14	11c	yes	spread	oxer	right	no	0	0	0,00	yes
Mechelen	The Longines FEI World Cup Jumping - Mechelen round A	30.12.2017	CSI-W5*	3	1.60	390	54	12	17.03.2006	M	WESTF	171	6	1	19	38	2803	179	M	15	12	no	vertical	vertical	left	no	0	0	0,00	yes
Mechelen	The Longines FEI World Cup Jumping - Mechelen round A	30.12.2017	CSI-W5*	3	1.60	390	54	12	17.03.2006	M	WESTF	171	6	1	19	38	2803	179	M	16	13	no	spread	oxer	left	yes	4	1	0,00	yes
Mechelen	The Longines FEI World Cup Jumping - Mechelen round A	30.12.2017	CSI-W5*	3	1.60	390	107	11	25.02.2007	S	BWP	196	13	2	7	31	2168	92	M	1	1	no	spread	oxer	left	no	0	0	64,58	no
Mechelen	The Longines FEI World Cup Jumping - Mechelen round A	30.12.2017	CSI-W5*	3	1.60	390	107	11	25.02.2007	S	BWP	196	13	2	7	31	2168	92	M	2	2	no	vertical	vertical	straight	no	0	0	64,58	no
Mechelen	The Longines FEI World Cup Jumping - Mechelen round A	30.12.2017	CSI-W5*	3	1.60	390	107	11	25.02.2007	S	BWP	196	13	2	7	31	2168	92	M	3	3	no	spread	oxer	right	no	0	0	64,58	no
Mechelen	The Longines FEI World Cup Jumping - Mechelen round A	30.12.2017	CSI-W5*	3	1.60	390	107	11	25.02.2007	S	BWP	196	13	2	7	31	2168	92	M	4	4	no	vertical	vertical	right	no	0	0	64,58	no
Mechelen	The Longines FEI World Cup Jumping - Mechelen round A	30.12.2017	CSI-W5*	3	1.60	390	107	11	25.02.2007	S	BWP	196	13	2	7	31	2168	92	M	5	5a	yes	spread	oxer	left	no	0	0	64,58	no
Mechelen	The Longines FEI World Cup Jumping - Mechelen round A	30.12.2017	CSI-W5*	3	1.60	390	107	11	25.02.2007	S	BWP	196	13	2	7	31	2168	92	M	6	5b	yes	vertical	vertical	left	no	0	0	64,58	no
Mechelen	The Longines FEI World Cup Jumping - Mechelen round A	30.12.2017	CSI-W5*	3	1.60	390	107	11	25.02.2007	S	BWP	196	13	2	7	31	2168	92	M	7	6	no	spread	oxer	straight	no	0	0	64,58	no
Mechelen	The Longines FEI World Cup Jumping - Mechelen round A	30.12.2017	CSI-W5*	3	1.60	390	107	11	25.02.2007	S	BWP	196	13	2	7	31	2168	92	M	8	7	no	vertical	vertical with water	left	no	0	0	64,58	no
Mechelen	The Longines FEI World Cup Jumping - Mechelen round A	30.12.2017	CSI-W5*	3	1.60	390	107	11	25.02.2007	S	BWP	196	13	2	7	31	2168	92	M	9	8	no	spread	triple bar	left	no	0	0	64,58	no
Mechelen	The Longines FEI World Cup Jumping - Mechelen round A	30.12.2017	CSI-W5*	3	1.60	390	107	11	25.02.2007	S	BWP	196	13	2	7	31	2168	92	M	10	9	no	vertical	vertical	left	no	0	0	64,58	no
Mechelen	The Longines FEI World Cup Jumping - Mechelen round A	30.12.2017	CSI-W5*	3	1.60	390	107	11	25.02.2007	S	BWP	196	13	2	7	31	2168	92	M	11	10	no	spread	oxer	right	no	0	0	64,58	no
Mechelen	The Longines FEI World Cup Jumping - Mechelen round A	30.12.2017	CSI-W5*	3	1.60	390	107	11	25.02.2007	S	BWP	196	13	2	7	31	2168	92	M	12	11a	yes	vertical	vertical	right	no	0	0	64,58	no
Mechelen	The Longines FEI World Cup Jumping - Mechelen round A	30.12.2017	CSI-W5*	3	1.60	390	107	11	25.02.2007	S	BWP	196	13	2	7	31	2168	92	M	13	11b	yes	spread	oxer	right	no	0	0	64,58	no
Mechelen	The Longines FEI World Cup Jumping - Mechelen round A	30.12.2017	CSI-W5*	3	1.60	390	107	11	25.02.2007	S	BWP	196	13	2	7	31	2168	92	M	14	11c	yes	spread	oxer	right	no	0	0	64,58	no
Mechelen	The Longines FEI World Cup Jumping - Mechelen round A	30.12.2017	CSI-W5*	3	1.60	390	107	11	25.02.2007	S	BWP	196	13	2	7	31	2168	92	M	15	12	no	vertical	vertical	left	no	0	0	64,58	no
Mechelen	The Longines FEI World Cup Jumping - Mechelen round A	30.12.2017	CSI-W5*	3	1.60	390	107	11	25.02.2007	S	BWP	196	13	2	7	31	2168	92	M	16	13	no	spread	oxer	left	no	0	0	64,58	no
Mechelen	The Longines FEI World Cup Jumping - Mechelen round B	30.12.2017	CSI-W5*	3	1.60	290	107	11	25.02.2007	S	BWP	196	13	2	7	31	2168	92	M	1	1	no	spread	oxer	right	no	0	0	39,97	no
Mechelen	The Longines FEI World Cup Jumping - Mechelen round B	30.12.2017	CSI-W5*	3	1.60	290	107	11	25.02.2007	S	BWP	196	13	2	7	31	2168	92	M	2	2	no	vertical	vertical	right	no	0	0	39,97	no
Mechelen	The Longines FEI World Cup Jumping - Mechelen round B	30.12.2017	CSI-W5*	3	1.60	290	107	11	25.02.2007	S	BWP	196	13	2	7	31	2168	92	M	3	3	no	spread	oxer	left	no	0	0	39,97	no
Mechelen	The Longines FEI World Cup Jumping - Mechelen round B	30.12.2017	CSI-W5*	3	1.60	290	107	11	25.02.2007	S	BWP	196	13	2	7	31	2168	92	M	4	5	no	vertical	vertical	left	no	0	0	39,97	no
Mechelen	The Longines FEI World Cup Jumping - Mechelen round B	30.12.2017	CSI-W5*	3	1.60	290	107	11	25.02.2007	S	BWP	196	13	2	7	31	2168	92	M	5	6a	yes	vertical	vertical	right	no	0	0	39,97	no
Mechelen	The Longines FEI World Cup Jumping - Mechelen round B	30.12.2017	CSI-W5*	3	1.60	290	107	11	25.02.2007	S	BWP	196	13	2	7	31	2168	92	M	6	6b	yes	spread	oxer	right	no	0	0	39,97	no
Mechelen	The Longines FEI World Cup Jumping - Mechelen round B	30.12.2017	CSI-W5*	3	1.60	290	107	11	25.02.2007	S	BWP	196	13	2	7	31	2168	92	M	7	7	no	vertical	vertical	left	no	0	0	39,97	no
Mechelen	The Longines FEI World Cup Jumping - Mechelen round B	30.12.2017	CSI-W5*	3	1.60	290	107	11	25.02.2007	S	BWP	196	13	2	7	31	2168	92	M	8	8	no	spread	triple bar	left	no	0	0	39,97	no
Mechelen	The Longines FEI World Cup Jumping - Mechelen round B	30.12.2017	CSI-W5*	3	1.60	290	107	11	25.02.2007	S	BWP	196	13	2	7	31	2168	92	M	9	9	no	vertical	vertical	left	no	0	0	39,97	no
Mechelen	The Longines FEI World Cup Jumping - Mechelen round A	30.12.2017	CSI-W5*	3	1.60	390	39	10	23.05.2008	M	ZANG	150	1	5	15	32	2092	63	M	1	1	no	spread	oxer	left	no	0	0	0,00	yes
Mechelen	The Longines FEI World Cup Jumping - Mechelen round A	30.12.2017	CSI-W5*	3	1.60	390	39	10	23.05.2008	M	ZANG	150	1	5	15	32	2092	63	M	2	2	no	vertical	vertical	straight	no	0	0	0,00	yes
Mechelen	The Longines FEI World Cup Jumping - Mechelen round A	30.12.2017	CSI-W5*	3	1.60	390	39	10	23.05.2008	M	ZANG	150	1	5	15	32	2092	63	M	3	3	no	spread	oxer	right	no	0	0	0,00	yes
Mechelen	The Longines FEI World Cup Jumping - Mechelen round A	30.12.2017	CSI-W5*	3	1.60	390	39	10	23.05.2008	M	ZANG	150	1	5	15	32	2092	63	M	4	4	no	vertical	vertical	right	no	0	0	0,00	yes
Mechelen	The Longines FEI World Cup Jumping - Mechelen round A	30.12.2017	CSI-W5*	3	1.60	390	39	10	23.05.2008	M	ZANG	150	1	5	15	32	2092	63	M	5	5a	yes	spread	oxer	left	yes	4	0	0,00	yes
Mechelen	The Longines FEI World Cup Jumping - Mechelen round A	30.12.2017	CSI-W5*	3	1.60	390	39	10	23.05.2008	M	ZANG	150	1	5	15	32	2092	63	M	6	5b	yes	vertical	vertical	left	no	0	0	0,00	yes
Mechelen	The Longines FEI World Cup Jumping - Mechelen round A	30.12.2017	CSI-W5*	3	1.60	390	39	10	23.05.2008	M	ZANG	150	1	5	15	32	2092	63	M	7	6	no	spread	oxer	straight	no	0	0	0,00	yes
Mechelen	The Longines FEI World Cup Jumping - Mechelen round A	30.12.2017	CSI-W5*	3	1.60	390	39	10	23.05.2008	M	ZANG	150	1	5	15	32	2092	63	M	8	7	no	vertical	vertical with water	left	no	0	0	0,00	yes
Mechelen	The Longines FEI World Cup Jumping - Mechelen round A	30.12.2017	CSI-W5*	3	1.60	390	39	10	23.05.2008	M	ZANG	150	1	5	15	32	2092	63	M	9	8	no	spread	triple bar	left	no	0	0	0,00	yes
Mechelen	The Longines FEI World Cup Jumping - Mechelen round A	30.12.2017	CSI-W5*	3	1.60	390	39	10	23.05.2008	M	ZANG	150	1	5	15	32	2092	63	M	10	9	no	vertical	vertical	left	no	0	0	0,00	yes
Mechelen	The Longines FEI World Cup Jumping - Mechelen round A	30.12.2017	CSI-W5*	3	1.60	390	39	10	23.05.2008	M	ZANG	150	1	5	15	32	2092	63	M	11	10	no	spread	oxer	right	yes	4	0	0,00	yes
Mechelen	The Longines FEI World Cup Jumping - Mechelen round A	30.12.2017	CSI-W5*	3	1.60	390	39	10	23.05.2008	M	ZANG	150	1	5	15	32	2092	63	M	12	11a	yes	vertical	vertical	right	no	0	0	0,00	yes
Mechelen	The Longines FEI World Cup Jumping - Mechelen round A	30.12.2017	CSI-W5*	3	1.60	390	39	10	23.05.2008	M	ZANG	150	1	5	15	32	2092	63	M	13	11b	yes	spread	oxer	right	no	0	0	0,00	yes
Mechelen	The Longines FEI World Cup Jumping - Mechelen round A	30.12.2017	CSI-W5*	3	1.60	390	39	10	23.05.2008	M	ZANG	150	1	5	15	32	2092	63	M	14	11c	yes	spread	oxer	right	no	0	0	0,00	yes
Mechelen	The Longines FEI World Cup Jumping - Mechelen round A	30.12.2017	CSI-W5*	3	1.60	390	39	10	23.05.2008	M	ZANG	150	1	5	15	32	2092	63	M	15	12	no	vertical	vertical	left	no	0	0	0,00	yes
Mechelen	The Longines FEI World Cup Jumping - Mechelen round A	30.12.2017	CSI-W5*	3	1.60	390	39	10	23.05.2008	M	ZANG	150	1	5	15	32	2092	63	M	16	13	no	spread	oxer	left	no	0	0	0,00	yes
Mechelen	The Longines FEI World Cup Jumping - Mechelen round A	30.12.2017	CSI-W5*	3	1.60	390	20	11	16.04.2007	G	OS	121	0	7	48	58	1857	49	M	1	1	no	spread	oxer	left	no	0	0	67,79	no
Mechelen	The Longines FEI World Cup Jumping - Mechelen round A	30.12.2017	CSI-W5*	3	1.60	390	20	11	16.04.2007	G	OS	121	0	7	48	58	1857	49	M	2	2	no	vertical	vertical	straight	no	0	0	67,79	no
Mechelen	The Longines FEI World Cup Jumping - Mechelen round A	30.12.2017	CSI-W5*	3	1.60	390	20	11	16.04.2007	G	OS	121	0	7	48	58	1857	49	M	3	3	no	spread	oxer	right	no	0	0	67,79	no
Mechelen	The Longines FEI World Cup Jumping - Mechelen round A	30.12.2017	CSI-W5*	3	1.60	390	20	11	16.04.2007	G	OS	121	0	7	48	58	1857	49	M	4	4	no	vertical	vertical	right	no	0	0	67,79	no
Mechelen	The Longines FEI World Cup Jumping - Mechelen round A	30.12.2017	CSI-W5*	3	1.60	390	20	11	16.04.2007	G	OS	121	0	7	48	58	1857	49	M	5	5a	yes	spread	oxer	left	no	0	0	67,79	no
Mechelen	The Longines FEI World Cup Jumping - Mechelen round A	30.12.2017	CSI-W5*	3	1.60	390	20	11	16.04.2007	G	OS	121	0	7	48	58	1857	49	M	6	5b	yes	vertical	vertical	left	no	0	0	67,79	no
Mechelen	The Longines FEI World Cup Jumping - Mechelen round A	30.12.2017	CSI-W5*	3	1.60	390	20	11	16.04.2007	G	OS	121	0	7	48	58	1857	49	M	7	6	no	spread	oxer	straight	no	0	0	67,79	no
Mechelen	The Longines FEI World Cup Jumping - Mechelen round A	30.12.2017	CSI-W5*	3	1.60	390	20	11	16.04.2007	G	OS	121	0	7	48	58	1857	49	M	8	7	no	vertical	vertical with water	left	no	0	0	67,79	no
Mechelen	The Longines FEI World Cup Jumping - Mechelen round A	30.12.2017	CSI-W5*	3	1.60	390	20	11	16.04.2007	G	OS	121	0	7	48	58	1857	49	M	9	8	no	spread	triple bar	left	no	0	0	67,79	no
Mechelen	The Longines FEI World Cup Jumping - Mechelen round A	30.12.2017	CSI-W5*	3	1.60	390	20	11	16.04.2007	G	OS	121	0	7	48	58	1857	49	M	10	9	no	vertical	vertical	left	no	0	0	67,79	no
Mechelen	The Longines FEI World Cup Jumping - Mechelen round A	30.12.2017	CSI-W5*	3	1.60	390	20	11	16.04.2007	G	OS	121	0	7	48	58	1857	49	M	11	10	no	spread	oxer	right	no	0	0	67,79	no
Mechelen	The Longines FEI World Cup Jumping - Mechelen round A	30.12.2017	CSI-W5*	3	1.60	390	20	11	16.04.2007	G	OS	121	0	7	48	58	1857	49	M	12	11a	yes	vertical	vertical	right	yes	4	0	67,79	no
Mechelen	The Longines FEI World Cup Jumping - Mechelen round A	30.12.2017	CSI-W5*	3	1.60	390	20	11	16.04.2007	G	OS	121	0	7	48	58	1857	49	M	13	11b	yes	spread	oxer	right	yes	4	0	67,79	no
Mechelen	The Longines FEI World Cup Jumping - Mechelen round A	30.12.2017	CSI-W5*	3	1.60	390	20	11	16.04.2007	G	OS	121	0	7	48	58	1857	49	M	14	11c	yes	spread	oxer	right	no	0	0	67,79	no
Mechelen	The Longines FEI World Cup Jumping - Mechelen round A	30.12.2017	CSI-W5*	3	1.60	390	20	11	16.04.2007	G	OS	121	0	7	48	58	1857	49	M	15	12	no	vertical	vertical	left	no	0	0	67,79	no
Mechelen	The Longines FEI World Cup Jumping - Mechelen round A	30.12.2017	CSI-W5*	3	1.60	390	20	11	16.04.2007	G	OS	121	0	7	48	58	1857	49	M	16	13	no	spread	oxer	left	yes	4	1	67,79	no
Mechelen	The Longines FEI World Cup Jumping - Mechelen round A	30.12.2017	CSI-W5*	3	1.60	390	159	11	15.04.2007	M	PZHK	161	4	11	414	32	1331	18	F	1	1	no	spread	oxer	left	no	0	0	66,1	no
Mechelen	The Longines FEI World Cup Jumping - Mechelen round A	30.12.2017	CSI-W5*	3	1.60	390	159	11	15.04.2007	M	PZHK	161	4	11	414	32	1331	18	F	2	2	no	vertical	vertical	straight	no	0	0	66,1	no
Mechelen	The Longines FEI World Cup Jumping - Mechelen round A	30.12.2017	CSI-W5*	3	1.60	390	159	11	15.04.2007	M	PZHK	161	4	11	414	32	1331	18	F	3	3	no	spread	oxer	right	no	0	0	66,1	no
Mechelen	The Longines FEI World Cup Jumping - Mechelen round A	30.12.2017	CSI-W5*	3	1.60	390	159	11	15.04.2007	M	PZHK	161	4	11	414	32	1331	18	F	4	4	no	vertical	vertical	right	no	0	0	66,1	no
Mechelen	The Longines FEI World Cup Jumping - Mechelen round A	30.12.2017	CSI-W5*	3	1.60	390	159	11	15.04.2007	M	PZHK	161	4	11	414	32	1331	18	F	5	5a	yes	spread	oxer	left	no	0	0	66,1	no
Mechelen	The Longines FEI World Cup Jumping - Mechelen round A	30.12.2017	CSI-W5*	3	1.60	390	159	11	15.04.2007	M	PZHK	161	4	11	414	32	1331	18	F	6	5b	yes	vertical	vertical	left	no	0	0	66,1	no
Mechelen	The Longines FEI World Cup Jumping - Mechelen round A	30.12.2017	CSI-W5*	3	1.60	390	159	11	15.04.2007	M	PZHK	161	4	11	414	32	1331	18	F	7	6	no	spread	oxer	straight	no	0	0	66,1	no
Mechelen	The Longines FEI World Cup Jumping - Mechelen round A	30.12.2017	CSI-W5*	3	1.60	390	159	11	15.04.2007	M	PZHK	161	4	11	414	32	1331	18	F	8	7	no	vertical	vertical with water	left	no	0	0	66,1	no
Mechelen	The Longines FEI World Cup Jumping - Mechelen round A	30.12.2017	CSI-W5*	3	1.60	390	159	11	15.04.2007	M	PZHK	161	4	11	414	32	1331	18	F	9	8	no	spread	triple bar	left	no	0	0	66,1	no
Mechelen	The Longines FEI World Cup Jumping - Mechelen round A	30.12.2017	CSI-W5*	3	1.60	390	159	11	15.04.2007	M	PZHK	161	4	11	414	32	1331	18	F	10	9	no	vertical	vertical	left	no	0	0	66,1	no
Mechelen	The Longines FEI World Cup Jumping - Mechelen round A	30.12.2017	CSI-W5*	3	1.60	390	159	11	15.04.2007	M	PZHK	161	4	11	414	32	1331	18	F	11	10	no	spread	oxer	right	yes	4	0	66,1	no
Mechelen	The Longines FEI World Cup Jumping - Mechelen round A	30.12.2017	CSI-W5*	3	1.60	390	159	11	15.04.2007	M	PZHK	161	4	11	414	32	1331	18	F	12	11a	yes	vertical	vertical	right	no	0	0	66,1	no
Mechelen	The Longines FEI World Cup Jumping - Mechelen round A	30.12.2017	CSI-W5*	3	1.60	390	159	11	15.04.2007	M	PZHK	161	4	11	414	32	1331	18	F	13	11b	yes	spread	oxer	right	no	0	0	66,1	no
Mechelen	The Longines FEI World Cup Jumping - Mechelen round A	30.12.2017	CSI-W5*	3	1.60	390	159	11	15.04.2007	M	PZHK	161	4	11	414	32	1331	18	F	14	11c	yes	spread	oxer	right	no	0	0	66,1	no
Mechelen	The Longines FEI World Cup Jumping - Mechelen round A	30.12.2017	CSI-W5*	3	1.60	390	159	11	15.04.2007	M	PZHK	161	4	11	414	32	1331	18	F	15	12	no	vertical	vertical	left	yes	4	0	66,1	no
Mechelen	The Longines FEI World Cup Jumping - Mechelen round A	30.12.2017	CSI-W5*	3	1.60	390	159	11	15.04.2007	M	PZHK	161	4	11	414	32	1331	18	F	16	13	no	spread	oxer	left	yes	4	0	66,1	no
Mechelen	The Longines FEI World Cup Jumping - Mechelen round A	30.12.2017	CSI-W5*	3	1.60	390	196	13	15.05.2005	M	ZANG	166	4	14	5	37	1868	96	M	1	1	no	spread	oxer	left	no	0	0	66,2	no
Mechelen	The Longines FEI World Cup Jumping - Mechelen round A	30.12.2017	CSI-W5*	3	1.60	390	196	13	15.05.2005	M	ZANG	166	4	14	5	37	1868	96	M	2	2	no	vertical	vertical	straight	no	0	0	66,2	no
Mechelen	The Longines FEI World Cup Jumping - Mechelen round A	30.12.2017	CSI-W5*	3	1.60	390	196	13	15.05.2005	M	ZANG	166	4	14	5	37	1868	96	M	3	3	no	spread	oxer	right	no	0	0	66,2	no
Mechelen	The Longines FEI World Cup Jumping - Mechelen round A	30.12.2017	CSI-W5*	3	1.60	390	196	13	15.05.2005	M	ZANG	166	4	14	5	37	1868	96	M	4	4	no	vertical	vertical	right	no	0	0	66,2	no
Mechelen	The Longines FEI World Cup Jumping - Mechelen round A	30.12.2017	CSI-W5*	3	1.60	390	196	13	15.05.2005	M	ZANG	166	4	14	5	37	1868	96	M	5	5a	yes	spread	oxer	left	no	0	0	66,2	no
Mechelen	The Longines FEI World Cup Jumping - Mechelen round A	30.12.2017	CSI-W5*	3	1.60	390	196	13	15.05.2005	M	ZANG	166	4	14	5	37	1868	96	M	6	5b	yes	vertical	vertical	left	no	0	0	66,2	no
Mechelen	The Longines FEI World Cup Jumping - Mechelen round A	30.12.2017	CSI-W5*	3	1.60	390	196	13	15.05.2005	M	ZANG	166	4	14	5	37	1868	96	M	7	6	no	spread	oxer	straight	yes	4	0	66,2	no
Mechelen	The Longines FEI World Cup Jumping - Mechelen round A	30.12.2017	CSI-W5*	3	1.60	390	196	13	15.05.2005	M	ZANG	166	4	14	5	37	1868	96	M	8	7	no	vertical	vertical with water	left	no	0	0	66,2	no
Mechelen	The Longines FEI World Cup Jumping - Mechelen round A	30.12.2017	CSI-W5*	3	1.60	390	196	13	15.05.2005	M	ZANG	166	4	14	5	37	1868	96	M	9	8	no	spread	triple bar	left	no	0	0	66,2	no
Mechelen	The Longines FEI World Cup Jumping - Mechelen round A	30.12.2017	CSI-W5*	3	1.60	390	196	13	15.05.2005	M	ZANG	166	4	14	5	37	1868	96	M	10	9	no	vertical	vertical	left	no	0	0	66,2	no
Mechelen	The Longines FEI World Cup Jumping - Mechelen round A	30.12.2017	CSI-W5*	3	1.60	390	196	13	15.05.2005	M	ZANG	166	4	14	5	37	1868	96	M	11	10	no	spread	oxer	right	no	0	0	66,2	no
Mechelen	The Longines FEI World Cup Jumping - Mechelen round A	30.12.2017	CSI-W5*	3	1.60	390	196	13	15.05.2005	M	ZANG	166	4	14	5	37	1868	96	M	12	11a	yes	vertical	vertical	right	no	0	0	66,2	no
Mechelen	The Longines FEI World Cup Jumping - Mechelen round A	30.12.2017	CSI-W5*	3	1.60	390	196	13	15.05.2005	M	ZANG	166	4	14	5	37	1868	96	M	13	11b	yes	spread	oxer	right	no	0	0	66,2	no
Mechelen	The Longines FEI World Cup Jumping - Mechelen round A	30.12.2017	CSI-W5*	3	1.60	390	196	13	15.05.2005	M	ZANG	166	4	14	5	37	1868	96	M	14	11c	yes	spread	oxer	right	no	0	0	66,2	no
Mechelen	The Longines FEI World Cup Jumping - Mechelen round A	30.12.2017	CSI-W5*	3	1.60	390	196	13	15.05.2005	M	ZANG	166	4	14	5	37	1868	96	M	15	12	no	vertical	vertical	left	no	0	0	66,2	no
Mechelen	The Longines FEI World Cup Jumping - Mechelen round A	30.12.2017	CSI-W5*	3	1.60	390	196	13	15.05.2005	M	ZANG	166	4	14	5	37	1868	96	M	16	13	no	spread	oxer	left	no	0	0	66,2	no
Mechelen	The Longines FEI World Cup Jumping - Mechelen round A	30.12.2017	CSI-W5*	3	1.60	390	57	15	04.04.2003	G	WESTF	230	13	16	17	37	2215	106	M	1	1	no	spread	oxer	left	no	0	0	64,21	no
Mechelen	The Longines FEI World Cup Jumping - Mechelen round A	30.12.2017	CSI-W5*	3	1.60	390	57	15	04.04.2003	G	WESTF	230	13	16	17	37	2215	106	M	2	2	no	vertical	vertical	straight	no	0	0	64,21	no
Mechelen	The Longines FEI World Cup Jumping - Mechelen round A	30.12.2017	CSI-W5*	3	1.60	390	57	15	04.04.2003	G	WESTF	230	13	16	17	37	2215	106	M	3	3	no	spread	oxer	right	no	0	0	64,21	no
Mechelen	The Longines FEI World Cup Jumping - Mechelen round A	30.12.2017	CSI-W5*	3	1.60	390	57	15	04.04.2003	G	WESTF	230	13	16	17	37	2215	106	M	4	4	no	vertical	vertical	right	no	0	0	64,21	no
Mechelen	The Longines FEI World Cup Jumping - Mechelen round A	30.12.2017	CSI-W5*	3	1.60	390	57	15	04.04.2003	G	WESTF	230	13	16	17	37	2215	106	M	5	5a	yes	spread	oxer	left	no	0	0	64,21	no
Mechelen	The Longines FEI World Cup Jumping - Mechelen round A	30.12.2017	CSI-W5*	3	1.60	390	57	15	04.04.2003	G	WESTF	230	13	16	17	37	2215	106	M	6	5b	yes	vertical	vertical	left	no	0	0	64,21	no
Mechelen	The Longines FEI World Cup Jumping - Mechelen round A	30.12.2017	CSI-W5*	3	1.60	390	57	15	04.04.2003	G	WESTF	230	13	16	17	37	2215	106	M	7	6	no	spread	oxer	straight	no	0	0	64,21	no
Mechelen	The Longines FEI World Cup Jumping - Mechelen round A	30.12.2017	CSI-W5*	3	1.60	390	57	15	04.04.2003	G	WESTF	230	13	16	17	37	2215	106	M	8	7	no	vertical	vertical with water	left	no	0	0	64,21	no
Mechelen	The Longines FEI World Cup Jumping - Mechelen round A	30.12.2017	CSI-W5*	3	1.60	390	57	15	04.04.2003	G	WESTF	230	13	16	17	37	2215	106	M	9	8	no	spread	triple bar	left	no	0	0	64,21	no
Mechelen	The Longines FEI World Cup Jumping - Mechelen round A	30.12.2017	CSI-W5*	3	1.60	390	57	15	04.04.2003	G	WESTF	230	13	16	17	37	2215	106	M	10	9	no	vertical	vertical	left	no	0	0	64,21	no
Mechelen	The Longines FEI World Cup Jumping - Mechelen round A	30.12.2017	CSI-W5*	3	1.60	390	57	15	04.04.2003	G	WESTF	230	13	16	17	37	2215	106	M	11	10	no	spread	oxer	right	no	0	0	64,21	no
Mechelen	The Longines FEI World Cup Jumping - Mechelen round A	30.12.2017	CSI-W5*	3	1.60	390	57	15	04.04.2003	G	WESTF	230	13	16	17	37	2215	106	M	12	11a	yes	vertical	vertical	right	no	0	0	64,21	no
Mechelen	The Longines FEI World Cup Jumping - Mechelen round A	30.12.2017	CSI-W5*	3	1.60	390	57	15	04.04.2003	G	WESTF	230	13	16	17	37	2215	106	M	13	11b	yes	spread	oxer	right	no	0	0	64,21	no
Mechelen	The Longines FEI World Cup Jumping - Mechelen round A	30.12.2017	CSI-W5*	3	1.60	390	57	15	04.04.2003	G	WESTF	230	13	16	17	37	2215	106	M	14	11c	yes	spread	oxer	right	no	0	0	64,21	no
Mechelen	The Longines FEI World Cup Jumping - Mechelen round A	30.12.2017	CSI-W5*	3	1.60	390	57	15	04.04.2003	G	WESTF	230	13	16	17	37	2215	106	M	15	12	no	vertical	vertical	left	no	0	0	64,21	no
Mechelen	The Longines FEI World Cup Jumping - Mechelen round A	30.12.2017	CSI-W5*	3	1.60	390	57	15	04.04.2003	G	WESTF	230	13	16	17	37	2215	106	M	16	13	no	spread	oxer	left	no	0	0	64,21	no
Mechelen	The Longines FEI World Cup Jumping - Mechelen round B	30.12.2017	CSI-W5*	3	1.60	290	57	15	04.04.2003	G	WESTF	230	13	16	17	37	2215	106	M	1	1	no	spread	oxer	right	no	0	0	40,51	no
Mechelen	The Longines FEI World Cup Jumping - Mechelen round B	30.12.2017	CSI-W5*	3	1.60	290	57	15	04.04.2003	G	WESTF	230	13	16	17	37	2215	106	M	2	2	no	vertical	vertical	right	no	0	0	40,51	no
Mechelen	The Longines FEI World Cup Jumping - Mechelen round B	30.12.2017	CSI-W5*	3	1.60	290	57	15	04.04.2003	G	WESTF	230	13	16	17	37	2215	106	M	3	3	no	spread	oxer	left	no	0	0	40,51	no
Mechelen	The Longines FEI World Cup Jumping - Mechelen round B	30.12.2017	CSI-W5*	3	1.60	290	57	15	04.04.2003	G	WESTF	230	13	16	17	37	2215	106	M	4	5	no	vertical	vertical	left	no	0	0	40,51	no
Mechelen	The Longines FEI World Cup Jumping - Mechelen round B	30.12.2017	CSI-W5*	3	1.60	290	57	15	04.04.2003	G	WESTF	230	13	16	17	37	2215	106	M	5	6a	yes	vertical	vertical	right	no	0	0	40,51	no
Mechelen	The Longines FEI World Cup Jumping - Mechelen round B	30.12.2017	CSI-W5*	3	1.60	290	57	15	04.04.2003	G	WESTF	230	13	16	17	37	2215	106	M	6	6b	yes	spread	oxer	right	no	0	0	40,51	no
Mechelen	The Longines FEI World Cup Jumping - Mechelen round B	30.12.2017	CSI-W5*	3	1.60	290	57	15	04.04.2003	G	WESTF	230	13	16	17	37	2215	106	M	7	7	no	vertical	vertical	left	no	0	0	40,51	no
Mechelen	The Longines FEI World Cup Jumping - Mechelen round B	30.12.2017	CSI-W5*	3	1.60	290	57	15	04.04.2003	G	WESTF	230	13	16	17	37	2215	106	M	8	8	no	spread	triple bar	left	no	0	0	40,51	no
Mechelen	The Longines FEI World Cup Jumping - Mechelen round B	30.12.2017	CSI-W5*	3	1.60	290	57	15	04.04.2003	G	WESTF	230	13	16	17	37	2215	106	M	9	9	no	vertical	vertical	left	no	0	0	40,51	no
Mechelen	The Longines FEI World Cup Jumping - Mechelen round A	30.12.2017	CSI-W5*	3	1.60	390	154	14	29.04.2004	G	SWB	175	3	18	81	31	1327	0	M	1	1	no	spread	oxer	left	no	0	0	67,29	no
Mechelen	The Longines FEI World Cup Jumping - Mechelen round A	30.12.2017	CSI-W5*	3	1.60	390	154	14	29.04.2004	G	SWB	175	3	18	81	31	1327	0	M	2	2	no	vertical	vertical	straight	no	0	0	67,29	no
Mechelen	The Longines FEI World Cup Jumping - Mechelen round A	30.12.2017	CSI-W5*	3	1.60	390	154	14	29.04.2004	G	SWB	175	3	18	81	31	1327	0	M	3	3	no	spread	oxer	right	no	0	0	67,29	no
Mechelen	The Longines FEI World Cup Jumping - Mechelen round A	30.12.2017	CSI-W5*	3	1.60	390	154	14	29.04.2004	G	SWB	175	3	18	81	31	1327	0	M	4	4	no	vertical	vertical	right	no	0	0	67,29	no
Mechelen	The Longines FEI World Cup Jumping - Mechelen round A	30.12.2017	CSI-W5*	3	1.60	390	154	14	29.04.2004	G	SWB	175	3	18	81	31	1327	0	M	5	5a	yes	spread	oxer	left	yes	4	0	67,29	no
Mechelen	The Longines FEI World Cup Jumping - Mechelen round A	30.12.2017	CSI-W5*	3	1.60	390	154	14	29.04.2004	G	SWB	175	3	18	81	31	1327	0	M	6	5b	yes	vertical	vertical	left	no	0	0	67,29	no
Mechelen	The Longines FEI World Cup Jumping - Mechelen round A	30.12.2017	CSI-W5*	3	1.60	390	154	14	29.04.2004	G	SWB	175	3	18	81	31	1327	0	M	7	6	no	spread	oxer	straight	no	0	0	67,29	no
Mechelen	The Longines FEI World Cup Jumping - Mechelen round A	30.12.2017	CSI-W5*	3	1.60	390	154	14	29.04.2004	G	SWB	175	3	18	81	31	1327	0	M	8	7	no	vertical	vertical with water	left	yes	4	0	67,29	no
Mechelen	The Longines FEI World Cup Jumping - Mechelen round A	30.12.2017	CSI-W5*	3	1.60	390	154	14	29.04.2004	G	SWB	175	3	18	81	31	1327	0	M	9	8	no	spread	triple bar	left	no	0	0	67,29	no
Mechelen	The Longines FEI World Cup Jumping - Mechelen round A	30.12.2017	CSI-W5*	3	1.60	390	154	14	29.04.2004	G	SWB	175	3	18	81	31	1327	0	M	10	9	no	vertical	vertical	left	no	0	0	67,29	no
Mechelen	The Longines FEI World Cup Jumping - Mechelen round A	30.12.2017	CSI-W5*	3	1.60	390	154	14	29.04.2004	G	SWB	175	3	18	81	31	1327	0	M	11	10	no	spread	oxer	right	no	0	0	67,29	no
Mechelen	The Longines FEI World Cup Jumping - Mechelen round A	30.12.2017	CSI-W5*	3	1.60	390	154	14	29.04.2004	G	SWB	175	3	18	81	31	1327	0	M	12	11a	yes	vertical	vertical	right	no	0	0	67,29	no
Mechelen	The Longines FEI World Cup Jumping - Mechelen round A	30.12.2017	CSI-W5*	3	1.60	390	154	14	29.04.2004	G	SWB	175	3	18	81	31	1327	0	M	13	11b	yes	spread	oxer	right	no	0	0	67,29	no
Mechelen	The Longines FEI World Cup Jumping - Mechelen round A	30.12.2017	CSI-W5*	3	1.60	390	154	14	29.04.2004	G	SWB	175	3	18	81	31	1327	0	M	14	11c	yes	spread	oxer	right	no	0	0	67,29	no
Mechelen	The Longines FEI World Cup Jumping - Mechelen round A	30.12.2017	CSI-W5*	3	1.60	390	154	14	29.04.2004	G	SWB	175	3	18	81	31	1327	0	M	15	12	no	vertical	vertical	left	no	0	0	67,29	no
Mechelen	The Longines FEI World Cup Jumping - Mechelen round A	30.12.2017	CSI-W5*	3	1.60	390	154	14	29.04.2004	G	SWB	175	3	18	81	31	1327	0	M	16	13	no	spread	oxer	left	no	0	1	67,29	no
Mechelen	The Longines FEI World Cup Jumping - Mechelen round A	30.12.2017	CSI-W5*	3	1.60	390	215	14	20.07.2004	G	KWPN	236	9	19	54	42	2041	67	M	1	1	no	spread	oxer	left	no	0	0	66,23	no
Mechelen	The Longines FEI World Cup Jumping - Mechelen round A	30.12.2017	CSI-W5*	3	1.60	390	215	14	20.07.2004	G	KWPN	236	9	19	54	42	2041	67	M	2	2	no	vertical	vertical	straight	no	0	0	66,23	no
Mechelen	The Longines FEI World Cup Jumping - Mechelen round A	30.12.2017	CSI-W5*	3	1.60	390	215	14	20.07.2004	G	KWPN	236	9	19	54	42	2041	67	M	3	3	no	spread	oxer	right	no	0	0	66,23	no
Mechelen	The Longines FEI World Cup Jumping - Mechelen round A	30.12.2017	CSI-W5*	3	1.60	390	215	14	20.07.2004	G	KWPN	236	9	19	54	42	2041	67	M	4	4	no	vertical	vertical	right	no	0	0	66,23	no
Mechelen	The Longines FEI World Cup Jumping - Mechelen round A	30.12.2017	CSI-W5*	3	1.60	390	215	14	20.07.2004	G	KWPN	236	9	19	54	42	2041	67	M	5	5a	yes	spread	oxer	left	no	0	0	66,23	no
Mechelen	The Longines FEI World Cup Jumping - Mechelen round A	30.12.2017	CSI-W5*	3	1.60	390	215	14	20.07.2004	G	KWPN	236	9	19	54	42	2041	67	M	6	5b	yes	vertical	vertical	left	no	0	0	66,23	no
Mechelen	The Longines FEI World Cup Jumping - Mechelen round A	30.12.2017	CSI-W5*	3	1.60	390	215	14	20.07.2004	G	KWPN	236	9	19	54	42	2041	67	M	7	6	no	spread	oxer	straight	no	0	0	66,23	no
Mechelen	The Longines FEI World Cup Jumping - Mechelen round A	30.12.2017	CSI-W5*	3	1.60	390	215	14	20.07.2004	G	KWPN	236	9	19	54	42	2041	67	M	8	7	no	vertical	vertical with water	left	no	0	0	66,23	no
Mechelen	The Longines FEI World Cup Jumping - Mechelen round A	30.12.2017	CSI-W5*	3	1.60	390	215	14	20.07.2004	G	KWPN	236	9	19	54	42	2041	67	M	9	8	no	spread	triple bar	left	no	0	0	66,23	no
Mechelen	The Longines FEI World Cup Jumping - Mechelen round A	30.12.2017	CSI-W5*	3	1.60	390	215	14	20.07.2004	G	KWPN	236	9	19	54	42	2041	67	M	10	9	no	vertical	vertical	left	no	0	0	66,23	no
Mechelen	The Longines FEI World Cup Jumping - Mechelen round A	30.12.2017	CSI-W5*	3	1.60	390	215	14	20.07.2004	G	KWPN	236	9	19	54	42	2041	67	M	11	10	no	spread	oxer	right	no	0	0	66,23	no
Mechelen	The Longines FEI World Cup Jumping - Mechelen round A	30.12.2017	CSI-W5*	3	1.60	390	215	14	20.07.2004	G	KWPN	236	9	19	54	42	2041	67	M	12	11a	yes	vertical	vertical	right	no	0	0	66,23	no
Mechelen	The Longines FEI World Cup Jumping - Mechelen round A	30.12.2017	CSI-W5*	3	1.60	390	215	14	20.07.2004	G	KWPN	236	9	19	54	42	2041	67	M	13	11b	yes	spread	oxer	right	no	0	0	66,23	no
Mechelen	The Longines FEI World Cup Jumping - Mechelen round A	30.12.2017	CSI-W5*	3	1.60	390	215	14	20.07.2004	G	KWPN	236	9	19	54	42	2041	67	M	14	11c	yes	spread	oxer	right	no	0	0	66,23	no
Mechelen	The Longines FEI World Cup Jumping - Mechelen round A	30.12.2017	CSI-W5*	3	1.60	390	215	14	20.07.2004	G	KWPN	236	9	19	54	42	2041	67	M	15	12	no	vertical	vertical	left	no	0	0	66,23	no
Mechelen	The Longines FEI World Cup Jumping - Mechelen round A	30.12.2017	CSI-W5*	3	1.60	390	215	14	20.07.2004	G	KWPN	236	9	19	54	42	2041	67	M	16	13	no	spread	oxer	left	no	0	0	66,23	no
Mechelen	The Longines FEI World Cup Jumping - Mechelen round B	30.12.2017	CSI-W5*	3	1.60	290	215	14	20.07.2004	G	KWPN	236	9	19	54	42	2041	67	M	1	1	no	spread	oxer	right	no	0	0	41,71	no
Mechelen	The Longines FEI World Cup Jumping - Mechelen round B	30.12.2017	CSI-W5*	3	1.60	290	215	14	20.07.2004	G	KWPN	236	9	19	54	42	2041	67	M	2	2	no	vertical	vertical	right	no	0	0	41,71	no
Mechelen	The Longines FEI World Cup Jumping - Mechelen round B	30.12.2017	CSI-W5*	3	1.60	290	215	14	20.07.2004	G	KWPN	236	9	19	54	42	2041	67	M	3	3	no	spread	oxer	left	no	0	0	41,71	no
Mechelen	The Longines FEI World Cup Jumping - Mechelen round B	30.12.2017	CSI-W5*	3	1.60	290	215	14	20.07.2004	G	KWPN	236	9	19	54	42	2041	67	M	4	5	no	vertical	vertical	left	no	0	0	41,71	no
Mechelen	The Longines FEI World Cup Jumping - Mechelen round B	30.12.2017	CSI-W5*	3	1.60	290	215	14	20.07.2004	G	KWPN	236	9	19	54	42	2041	67	M	5	6a	yes	vertical	vertical	right	no	0	0	41,71	no
Mechelen	The Longines FEI World Cup Jumping - Mechelen round B	30.12.2017	CSI-W5*	3	1.60	290	215	14	20.07.2004	G	KWPN	236	9	19	54	42	2041	67	M	6	6b	yes	spread	oxer	right	yes	4	0	41,71	no
Mechelen	The Longines FEI World Cup Jumping - Mechelen round B	30.12.2017	CSI-W5*	3	1.60	290	215	14	20.07.2004	G	KWPN	236	9	19	54	42	2041	67	M	7	7	no	vertical	vertical	left	no	0	0	41,71	no
Mechelen	The Longines FEI World Cup Jumping - Mechelen round B	30.12.2017	CSI-W5*	3	1.60	290	215	14	20.07.2004	G	KWPN	236	9	19	54	42	2041	67	M	8	8	no	spread	triple bar	left	no	0	0	41,71	no
Mechelen	The Longines FEI World Cup Jumping - Mechelen round B	30.12.2017	CSI-W5*	3	1.60	290	215	14	20.07.2004	G	KWPN	236	9	19	54	42	2041	67	M	9	9	no	vertical	vertical	left	no	0	0	41,71	no
Mechelen	The Longines FEI World Cup Jumping - Mechelen round A	30.12.2017	CSI-W5*	3	1.60	390	205	11	27.05.2007	M	SF	183	14	20	73	38	2030	91	M	1	1	no	spread	oxer	left	no	0	0	66,05	no
Mechelen	The Longines FEI World Cup Jumping - Mechelen round A	30.12.2017	CSI-W5*	3	1.60	390	205	11	27.05.2007	M	SF	183	14	20	73	38	2030	91	M	2	2	no	vertical	vertical	straight	no	0	0	66,05	no
Mechelen	The Longines FEI World Cup Jumping - Mechelen round A	30.12.2017	CSI-W5*	3	1.60	390	205	11	27.05.2007	M	SF	183	14	20	73	38	2030	91	M	3	3	no	spread	oxer	right	no	0	0	66,05	no
Mechelen	The Longines FEI World Cup Jumping - Mechelen round A	30.12.2017	CSI-W5*	3	1.60	390	205	11	27.05.2007	M	SF	183	14	20	73	38	2030	91	M	4	4	no	vertical	vertical	right	no	0	0	66,05	no
Mechelen	The Longines FEI World Cup Jumping - Mechelen round A	30.12.2017	CSI-W5*	3	1.60	390	205	11	27.05.2007	M	SF	183	14	20	73	38	2030	91	M	5	5a	yes	spread	oxer	left	yes	4	0	66,05	no
Mechelen	The Longines FEI World Cup Jumping - Mechelen round A	30.12.2017	CSI-W5*	3	1.60	390	205	11	27.05.2007	M	SF	183	14	20	73	38	2030	91	M	6	5b	yes	vertical	vertical	left	no	0	0	66,05	no
Mechelen	The Longines FEI World Cup Jumping - Mechelen round A	30.12.2017	CSI-W5*	3	1.60	390	205	11	27.05.2007	M	SF	183	14	20	73	38	2030	91	M	7	6	no	spread	oxer	straight	no	0	0	66,05	no
Mechelen	The Longines FEI World Cup Jumping - Mechelen round A	30.12.2017	CSI-W5*	3	1.60	390	205	11	27.05.2007	M	SF	183	14	20	73	38	2030	91	M	8	7	no	vertical	vertical with water	left	no	0	0	66,05	no
Mechelen	The Longines FEI World Cup Jumping - Mechelen round A	30.12.2017	CSI-W5*	3	1.60	390	205	11	27.05.2007	M	SF	183	14	20	73	38	2030	91	M	9	8	no	spread	triple bar	left	no	0	0	66,05	no
Mechelen	The Longines FEI World Cup Jumping - Mechelen round A	30.12.2017	CSI-W5*	3	1.60	390	205	11	27.05.2007	M	SF	183	14	20	73	38	2030	91	M	10	9	no	vertical	vertical	left	no	0	0	66,05	no
Mechelen	The Longines FEI World Cup Jumping - Mechelen round A	30.12.2017	CSI-W5*	3	1.60	390	205	11	27.05.2007	M	SF	183	14	20	73	38	2030	91	M	11	10	no	spread	oxer	right	no	0	0	66,05	no
Mechelen	The Longines FEI World Cup Jumping - Mechelen round A	30.12.2017	CSI-W5*	3	1.60	390	205	11	27.05.2007	M	SF	183	14	20	73	38	2030	91	M	12	11a	yes	vertical	vertical	right	no	0	0	66,05	no
Mechelen	The Longines FEI World Cup Jumping - Mechelen round A	30.12.2017	CSI-W5*	3	1.60	390	205	11	27.05.2007	M	SF	183	14	20	73	38	2030	91	M	13	11b	yes	spread	oxer	right	no	0	0	66,05	no
Mechelen	The Longines FEI World Cup Jumping - Mechelen round A	30.12.2017	CSI-W5*	3	1.60	390	205	11	27.05.2007	M	SF	183	14	20	73	38	2030	91	M	14	11c	yes	spread	oxer	right	yes	4	0	66,05	no
Mechelen	The Longines FEI World Cup Jumping - Mechelen round A	30.12.2017	CSI-W5*	3	1.60	390	205	11	27.05.2007	M	SF	183	14	20	73	38	2030	91	M	15	12	no	vertical	vertical	left	no	0	0	66,05	no
Mechelen	The Longines FEI World Cup Jumping - Mechelen round A	30.12.2017	CSI-W5*	3	1.60	390	205	11	27.05.2007	M	SF	183	14	20	73	38	2030	91	M	16	13	no	spread	oxer	left	no	0	0	66,05	no
Mechelen	The Longines FEI World Cup Jumping - Mechelen round A	30.12.2017	CSI-W5*	3	1.60	390	219	13	13.05.2005	G	SWB	110	2	21	99	28	1384	56	F	1	1	no	spread	oxer	left	no	0	0	65,58	no
Mechelen	The Longines FEI World Cup Jumping - Mechelen round A	30.12.2017	CSI-W5*	3	1.60	390	219	13	13.05.2005	G	SWB	110	2	21	99	28	1384	56	F	2	2	no	vertical	vertical	straight	no	0	0	65,58	no
Mechelen	The Longines FEI World Cup Jumping - Mechelen round A	30.12.2017	CSI-W5*	3	1.60	390	219	13	13.05.2005	G	SWB	110	2	21	99	28	1384	56	F	3	3	no	spread	oxer	right	no	0	0	65,58	no
Mechelen	The Longines FEI World Cup Jumping - Mechelen round A	30.12.2017	CSI-W5*	3	1.60	390	219	13	13.05.2005	G	SWB	110	2	21	99	28	1384	56	F	4	4	no	vertical	vertical	right	no	0	0	65,58	no
Mechelen	The Longines FEI World Cup Jumping - Mechelen round A	30.12.2017	CSI-W5*	3	1.60	390	219	13	13.05.2005	G	SWB	110	2	21	99	28	1384	56	F	5	5a	yes	spread	oxer	left	no	0	0	65,58	no
Mechelen	The Longines FEI World Cup Jumping - Mechelen round A	30.12.2017	CSI-W5*	3	1.60	390	219	13	13.05.2005	G	SWB	110	2	21	99	28	1384	56	F	6	5b	yes	vertical	vertical	left	no	0	0	65,58	no
Mechelen	The Longines FEI World Cup Jumping - Mechelen round A	30.12.2017	CSI-W5*	3	1.60	390	219	13	13.05.2005	G	SWB	110	2	21	99	28	1384	56	F	7	6	no	spread	oxer	straight	no	0	0	65,58	no
Mechelen	The Longines FEI World Cup Jumping - Mechelen round A	30.12.2017	CSI-W5*	3	1.60	390	219	13	13.05.2005	G	SWB	110	2	21	99	28	1384	56	F	8	7	no	vertical	vertical with water	left	no	0	0	65,58	no
Mechelen	The Longines FEI World Cup Jumping - Mechelen round A	30.12.2017	CSI-W5*	3	1.60	390	219	13	13.05.2005	G	SWB	110	2	21	99	28	1384	56	F	9	8	no	spread	triple bar	left	no	0	0	65,58	no
Mechelen	The Longines FEI World Cup Jumping - Mechelen round A	30.12.2017	CSI-W5*	3	1.60	390	219	13	13.05.2005	G	SWB	110	2	21	99	28	1384	56	F	10	9	no	vertical	vertical	left	no	0	0	65,58	no
Mechelen	The Longines FEI World Cup Jumping - Mechelen round A	30.12.2017	CSI-W5*	3	1.60	390	219	13	13.05.2005	G	SWB	110	2	21	99	28	1384	56	F	11	10	no	spread	oxer	right	no	0	0	65,58	no
Mechelen	The Longines FEI World Cup Jumping - Mechelen round A	30.12.2017	CSI-W5*	3	1.60	390	219	13	13.05.2005	G	SWB	110	2	21	99	28	1384	56	F	12	11a	yes	vertical	vertical	right	no	0	0	65,58	no
Mechelen	The Longines FEI World Cup Jumping - Mechelen round A	30.12.2017	CSI-W5*	3	1.60	390	219	13	13.05.2005	G	SWB	110	2	21	99	28	1384	56	F	13	11b	yes	spread	oxer	right	no	0	0	65,58	no
Mechelen	The Longines FEI World Cup Jumping - Mechelen round A	30.12.2017	CSI-W5*	3	1.60	390	219	13	13.05.2005	G	SWB	110	2	21	99	28	1384	56	F	14	11c	yes	spread	oxer	right	no	0	0	65,58	no
Mechelen	The Longines FEI World Cup Jumping - Mechelen round A	30.12.2017	CSI-W5*	3	1.60	390	219	13	13.05.2005	G	SWB	110	2	21	99	28	1384	56	F	15	12	no	vertical	vertical	left	no	0	0	65,58	no
Mechelen	The Longines FEI World Cup Jumping - Mechelen round A	30.12.2017	CSI-W5*	3	1.60	390	219	13	13.05.2005	G	SWB	110	2	21	99	28	1384	56	F	16	13	no	spread	oxer	left	yes	4	0	65,58	no
Mechelen	The Longines FEI World Cup Jumping - Mechelen round A	30.12.2017	CSI-W5*	3	1.60	390	28	12	26.03.2006	M	ZANG	193	9	23	11	35	1800	65	M	1	1	no	spread	oxer	left	no	0	0	66,41	no
Mechelen	The Longines FEI World Cup Jumping - Mechelen round A	30.12.2017	CSI-W5*	3	1.60	390	28	12	26.03.2006	M	ZANG	193	9	23	11	35	1800	65	M	2	2	no	vertical	vertical	straight	no	0	0	66,41	no
Mechelen	The Longines FEI World Cup Jumping - Mechelen round A	30.12.2017	CSI-W5*	3	1.60	390	28	12	26.03.2006	M	ZANG	193	9	23	11	35	1800	65	M	3	3	no	spread	oxer	right	no	0	0	66,41	no
Mechelen	The Longines FEI World Cup Jumping - Mechelen round A	30.12.2017	CSI-W5*	3	1.60	390	28	12	26.03.2006	M	ZANG	193	9	23	11	35	1800	65	M	4	4	no	vertical	vertical	right	no	0	0	66,41	no
Mechelen	The Longines FEI World Cup Jumping - Mechelen round A	30.12.2017	CSI-W5*	3	1.60	390	28	12	26.03.2006	M	ZANG	193	9	23	11	35	1800	65	M	5	5a	yes	spread	oxer	left	no	0	0	66,41	no
Mechelen	The Longines FEI World Cup Jumping - Mechelen round A	30.12.2017	CSI-W5*	3	1.60	390	28	12	26.03.2006	M	ZANG	193	9	23	11	35	1800	65	M	6	5b	yes	vertical	vertical	left	no	0	0	66,41	no
Mechelen	The Longines FEI World Cup Jumping - Mechelen round A	30.12.2017	CSI-W5*	3	1.60	390	28	12	26.03.2006	M	ZANG	193	9	23	11	35	1800	65	M	7	6	no	spread	oxer	straight	no	0	0	66,41	no
Mechelen	The Longines FEI World Cup Jumping - Mechelen round A	30.12.2017	CSI-W5*	3	1.60	390	28	12	26.03.2006	M	ZANG	193	9	23	11	35	1800	65	M	8	7	no	vertical	vertical with water	left	no	0	0	66,41	no
Mechelen	The Longines FEI World Cup Jumping - Mechelen round A	30.12.2017	CSI-W5*	3	1.60	390	28	12	26.03.2006	M	ZANG	193	9	23	11	35	1800	65	M	9	8	no	spread	triple bar	left	no	0	0	66,41	no
Mechelen	The Longines FEI World Cup Jumping - Mechelen round A	30.12.2017	CSI-W5*	3	1.60	390	28	12	26.03.2006	M	ZANG	193	9	23	11	35	1800	65	M	10	9	no	vertical	vertical	left	no	0	0	66,41	no
Mechelen	The Longines FEI World Cup Jumping - Mechelen round A	30.12.2017	CSI-W5*	3	1.60	390	28	12	26.03.2006	M	ZANG	193	9	23	11	35	1800	65	M	11	10	no	spread	oxer	right	no	0	0	66,41	no
Mechelen	The Longines FEI World Cup Jumping - Mechelen round A	30.12.2017	CSI-W5*	3	1.60	390	28	12	26.03.2006	M	ZANG	193	9	23	11	35	1800	65	M	12	11a	yes	vertical	vertical	right	no	0	0	66,41	no
Mechelen	The Longines FEI World Cup Jumping - Mechelen round A	30.12.2017	CSI-W5*	3	1.60	390	28	12	26.03.2006	M	ZANG	193	9	23	11	35	1800	65	M	13	11b	yes	spread	oxer	right	yes	4	0	66,41	no
Mechelen	The Longines FEI World Cup Jumping - Mechelen round A	30.12.2017	CSI-W5*	3	1.60	390	28	12	26.03.2006	M	ZANG	193	9	23	11	35	1800	65	M	14	11c	yes	spread	oxer	right	no	0	0	66,41	no
Mechelen	The Longines FEI World Cup Jumping - Mechelen round A	30.12.2017	CSI-W5*	3	1.60	390	28	12	26.03.2006	M	ZANG	193	9	23	11	35	1800	65	M	15	12	no	vertical	vertical	left	no	0	0	66,41	no
Mechelen	The Longines FEI World Cup Jumping - Mechelen round A	30.12.2017	CSI-W5*	3	1.60	390	28	12	26.03.2006	M	ZANG	193	9	23	11	35	1800	65	M	16	13	no	spread	oxer	left	no	0	0	66,41	no
Mechelen	The Longines FEI World Cup Jumping - Mechelen round A	30.12.2017	CSI-W5*	3	1.60	390	133	10	23.05.2008	M	BWP	131	1	27	269	20	1104	42	F	1	1	no	spread	oxer	left	no	0	0	69,62	no
Mechelen	The Longines FEI World Cup Jumping - Mechelen round A	30.12.2017	CSI-W5*	3	1.60	390	133	10	23.05.2008	M	BWP	131	1	27	269	20	1104	42	F	2	2	no	vertical	vertical	straight	no	0	0	69,62	no
Mechelen	The Longines FEI World Cup Jumping - Mechelen round A	30.12.2017	CSI-W5*	3	1.60	390	133	10	23.05.2008	M	BWP	131	1	27	269	20	1104	42	F	3	3	no	spread	oxer	right	no	0	0	69,62	no
Mechelen	The Longines FEI World Cup Jumping - Mechelen round A	30.12.2017	CSI-W5*	3	1.60	390	133	10	23.05.2008	M	BWP	131	1	27	269	20	1104	42	F	4	4	no	vertical	vertical	right	no	0	0	69,62	no
Mechelen	The Longines FEI World Cup Jumping - Mechelen round A	30.12.2017	CSI-W5*	3	1.60	390	133	10	23.05.2008	M	BWP	131	1	27	269	20	1104	42	F	5	5a	yes	spread	oxer	left	no	0	0	69,62	no
Mechelen	The Longines FEI World Cup Jumping - Mechelen round A	30.12.2017	CSI-W5*	3	1.60	390	133	10	23.05.2008	M	BWP	131	1	27	269	20	1104	42	F	6	5b	yes	vertical	vertical	left	no	0	0	69,62	no
Mechelen	The Longines FEI World Cup Jumping - Mechelen round A	30.12.2017	CSI-W5*	3	1.60	390	133	10	23.05.2008	M	BWP	131	1	27	269	20	1104	42	F	7	6	no	spread	oxer	straight	no	0	0	69,62	no
Mechelen	The Longines FEI World Cup Jumping - Mechelen round A	30.12.2017	CSI-W5*	3	1.60	390	133	10	23.05.2008	M	BWP	131	1	27	269	20	1104	42	F	8	7	no	vertical	vertical with water	left	no	0	0	69,62	no
Mechelen	The Longines FEI World Cup Jumping - Mechelen round A	30.12.2017	CSI-W5*	3	1.60	390	133	10	23.05.2008	M	BWP	131	1	27	269	20	1104	42	F	9	8	no	spread	triple bar	left	no	0	0	69,62	no
Mechelen	The Longines FEI World Cup Jumping - Mechelen round A	30.12.2017	CSI-W5*	3	1.60	390	133	10	23.05.2008	M	BWP	131	1	27	269	20	1104	42	F	10	9	no	vertical	vertical	left	no	0	0	69,62	no
Mechelen	The Longines FEI World Cup Jumping - Mechelen round A	30.12.2017	CSI-W5*	3	1.60	390	133	10	23.05.2008	M	BWP	131	1	27	269	20	1104	42	F	11	10	no	spread	oxer	right	no	0	0	69,62	no
Mechelen	The Longines FEI World Cup Jumping - Mechelen round A	30.12.2017	CSI-W5*	3	1.60	390	133	10	23.05.2008	M	BWP	131	1	27	269	20	1104	42	F	12	11a	yes	vertical	vertical	right	no	0	0	69,62	no
Mechelen	The Longines FEI World Cup Jumping - Mechelen round A	30.12.2017	CSI-W5*	3	1.60	390	133	10	23.05.2008	M	BWP	131	1	27	269	20	1104	42	F	13	11b	yes	spread	oxer	right	yes	4	0	69,62	no
Mechelen	The Longines FEI World Cup Jumping - Mechelen round A	30.12.2017	CSI-W5*	3	1.60	390	133	10	23.05.2008	M	BWP	131	1	27	269	20	1104	42	F	14	11c	yes	spread	oxer	right	no	0	0	69,62	no
Mechelen	The Longines FEI World Cup Jumping - Mechelen round A	30.12.2017	CSI-W5*	3	1.60	390	133	10	23.05.2008	M	BWP	131	1	27	269	20	1104	42	F	15	12	no	vertical	vertical	left	no	0	0	69,62	no
Mechelen	The Longines FEI World Cup Jumping - Mechelen round A	30.12.2017	CSI-W5*	3	1.60	390	133	10	23.05.2008	M	BWP	131	1	27	269	20	1104	42	F	16	13	no	spread	oxer	left	yes	4	1	69,62	no
Mechelen	The Longines FEI World Cup Jumping - Mechelen round A	30.12.2017	CSI-W5*	3	1.60	390	184	12	18.06.2006	S	SF	154	4	28	4	38	2170	90	M	1	1	no	spread	oxer	left	no	0	0	64,75	no
Mechelen	The Longines FEI World Cup Jumping - Mechelen round A	30.12.2017	CSI-W5*	3	1.60	390	184	12	18.06.2006	S	SF	154	4	28	4	38	2170	90	M	2	2	no	vertical	vertical	straight	no	0	0	64,75	no
Mechelen	The Longines FEI World Cup Jumping - Mechelen round A	30.12.2017	CSI-W5*	3	1.60	390	184	12	18.06.2006	S	SF	154	4	28	4	38	2170	90	M	3	3	no	spread	oxer	right	no	0	0	64,75	no
Mechelen	The Longines FEI World Cup Jumping - Mechelen round A	30.12.2017	CSI-W5*	3	1.60	390	184	12	18.06.2006	S	SF	154	4	28	4	38	2170	90	M	4	4	no	vertical	vertical	right	no	0	0	64,75	no
Mechelen	The Longines FEI World Cup Jumping - Mechelen round A	30.12.2017	CSI-W5*	3	1.60	390	184	12	18.06.2006	S	SF	154	4	28	4	38	2170	90	M	5	5a	yes	spread	oxer	left	no	0	0	64,75	no
Mechelen	The Longines FEI World Cup Jumping - Mechelen round A	30.12.2017	CSI-W5*	3	1.60	390	184	12	18.06.2006	S	SF	154	4	28	4	38	2170	90	M	6	5b	yes	vertical	vertical	left	no	0	0	64,75	no
Mechelen	The Longines FEI World Cup Jumping - Mechelen round A	30.12.2017	CSI-W5*	3	1.60	390	184	12	18.06.2006	S	SF	154	4	28	4	38	2170	90	M	7	6	no	spread	oxer	straight	no	0	0	64,75	no
Mechelen	The Longines FEI World Cup Jumping - Mechelen round A	30.12.2017	CSI-W5*	3	1.60	390	184	12	18.06.2006	S	SF	154	4	28	4	38	2170	90	M	8	7	no	vertical	vertical with water	left	no	0	0	64,75	no
Mechelen	The Longines FEI World Cup Jumping - Mechelen round A	30.12.2017	CSI-W5*	3	1.60	390	184	12	18.06.2006	S	SF	154	4	28	4	38	2170	90	M	9	8	no	spread	triple bar	left	no	0	0	64,75	no
Mechelen	The Longines FEI World Cup Jumping - Mechelen round A	30.12.2017	CSI-W5*	3	1.60	390	184	12	18.06.2006	S	SF	154	4	28	4	38	2170	90	M	10	9	no	vertical	vertical	left	no	0	0	64,75	no

Mechelen	The Longines FEI World Cup Jumping - Mechelen round A	30.12.2017	CSI-W5*	3	1.60	390	184	12	18.06.2006	S	SF	154	4	28	4	38	2170	90	M	11	10	no	spread	oxer	right	no	0	0	64,75	no
Mechelen	The Longines FEI World Cup Jumping - Mechelen round A	30.12.2017	CSI-W5*	3	1.60	390	184	12	18.06.2006	S	SF	154	4	28	4	38	2170	90	M	12	11a	yes	vertical	vertical	right	no	0	0	64,75	no
Mechelen	The Longines FEI World Cup Jumping - Mechelen round A	30.12.2017	CSI-W5*	3	1.60	390	184	12	18.06.2006	S	SF	154	4	28	4	38	2170	90	M	13	11b	yes	spread	oxer	right	no	0	0	64,75	no
Mechelen	The Longines FEI World Cup Jumping - Mechelen round A	30.12.2017	CSI-W5*	3	1.60	390	184	12	18.06.2006	S	SF	154	4	28	4	38	2170	90	M	14	11c	yes	spread	oxer	right	no	0	0	64,75	no
Mechelen	The Longines FEI World Cup Jumping - Mechelen round A	30.12.2017	CSI-W5*	3	1.60	390	184	12	18.06.2006	S	SF	154	4	28	4	38	2170	90	M	15	12	no	vertical	vertical	left	no	0	0	64,75	no
Mechelen	The Longines FEI World Cup Jumping - Mechelen round A	30.12.2017	CSI-W5*	3	1.60	390	184	12	18.06.2006	S	SF	154	4	28	4	38	2170	90	M	16	13	no	spread	oxer	left	yes	4	0	64,75	no
Mechelen	The Longines FEI World Cup Jumping - Mechelen round A	30.12.2017	CSI-W5*	3	1.60	390	23	13	25.02.2005	S	Bezplemenné	158	2	32	53	23	1832	77	M	1	1	no	spread	oxer	left	no	0	0	68,92	no
Mechelen	The Longines FEI World Cup Jumping - Mechelen round A	30.12.2017	CSI-W5*	3	1.60	390	23	13	25.02.2005	S	Bezplemenné	158	2	32	53	23	1832	77	M	2	2	no	vertical	vertical	straight	no	0	0	68,92	no
Mechelen	The Longines FEI World Cup Jumping - Mechelen round A	30.12.2017	CSI-W5*	3	1.60	390	23	13	25.02.2005	S	Bezplemenné	158	2	32	53	23	1832	77	M	3	3	no	spread	oxer	right	no	0	0	68,92	no
Mechelen	The Longines FEI World Cup Jumping - Mechelen round A	30.12.2017	CSI-W5*	3	1.60	390	23	13	25.02.2005	S	Bezplemenné	158	2	32	53	23	1832	77	M	4	4	no	vertical	vertical	right	no	0	0	68,92	no
Mechelen	The Longines FEI World Cup Jumping - Mechelen round A	30.12.2017	CSI-W5*	3	1.60	390	23	13	25.02.2005	S	Bezplemenné	158	2	32	53	23	1832	77	M	5	5a	yes	spread	oxer	left	yes	4	0	68,92	no
Mechelen	The Longines FEI World Cup Jumping - Mechelen round A	30.12.2017	CSI-W5*	3	1.60	390	23	13	25.02.2005	S	Bezplemenné	158	2	32	53	23	1832	77	M	6	5b	yes	vertical	vertical	left	no	0	0	68,92	no
Mechelen	The Longines FEI World Cup Jumping - Mechelen round A	30.12.2017	CSI-W5*	3	1.60	390	23	13	25.02.2005	S	Bezplemenné	158	2	32	53	23	1832	77	M	7	6	no	spread	oxer	straight	no	0	0	68,92	no
Mechelen	The Longines FEI World Cup Jumping - Mechelen round A	30.12.2017	CSI-W5*	3	1.60	390	23	13	25.02.2005	S	Bezplemenné	158	2	32	53	23	1832	77	M	8	7	no	vertical	vertical with water	left	no	0	0	68,92	no
Mechelen	The Longines FEI World Cup Jumping - Mechelen round A	30.12.2017	CSI-W5*	3	1.60	390	23	13	25.02.2005	S	Bezplemenné	158	2	32	53	23	1832	77	M	9	8	no	spread	triple bar	left	no	0	0	68,92	no
Mechelen	The Longines FEI World Cup Jumping - Mechelen round A	30.12.2017	CSI-W5*	3	1.60	390	23	13	25.02.2005	S	Bezplemenné	158	2	32	53	23	1832	77	M	10	9	no	vertical	vertical	left	no	0	0	68,92	no
Mechelen	The Longines FEI World Cup Jumping - Mechelen round A	30.12.2017	CSI-W5*	3	1.60	390	23	13	25.02.2005	S	Bezplemenné	158	2	32	53	23	1832	77	M	11	10	no	spread	oxer	right	yes	4	0	68,92	no
Mechelen	The Longines FEI World Cup Jumping - Mechelen round A	30.12.2017	CSI-W5*	3	1.60	390	23	13	25.02.2005	S	Bezplemenné	158	2	32	53	23	1832	77	M	12	11a	yes	vertical	vertical	right	no	0	0	68,92	no
Mechelen	The Longines FEI World Cup Jumping - Mechelen round A	30.12.2017	CSI-W5*	3	1.60	390	23	13	25.02.2005	S	Bezplemenné	158	2	32	53	23	1832	77	M	13	11b	yes	spread	oxer	right	no	0	0	68,92	no
Mechelen	The Longines FEI World Cup Jumping - Mechelen round A	30.12.2017	CSI-W5*	3	1.60	390	23	13	25.02.2005	S	Bezplemenné	158	2	32	53	23	1832	77	M	14	11c	yes	spread	oxer	right	no	0	0	68,92	no
Mechelen	The Longines FEI World Cup Jumping - Mechelen round A	30.12.2017	CSI-W5*	3	1.60	390	23	13	25.02.2005	S	Bezplemenné	158	2	32	53	23	1832	77	M	15	12	no	vertical	vertical	left	no	0	0	68,92	no
Mechelen	The Longines FEI World Cup Jumping - Mechelen round A	30.12.2017	CSI-W5*	3	1.60	390	23	13	25.02.2005	S	Bezplemenné	158	2	32	53	23	1832	77	M	16	13	no	spread	oxer	left	no	0	1	68,92	no
Mechelen	The Longines FEI World Cup Jumping - Mechelen round A	30.12.2017	CSI-W5*	3	1.60	390	156	14	28.07.2004	M	AES	262	25	33	23	23	1854	199	M	1	1	no	spread	oxer	left	no	0	0	64,18	no
Mechelen	The Longines FEI World Cup Jumping - Mechelen round A	30.12.2017	CSI-W5*	3	1.60	390	156	14	28.07.2004	M	AES	262	25	33	23	23	1854	199	M	2	2	no	vertical	vertical	straight	no	0	0	64,18	no
Mechelen	The Longines FEI World Cup Jumping - Mechelen round A	30.12.2017	CSI-W5*	3	1.60	390	156	14	28.07.2004	M	AES	262	25	33	23	23	1854	199	M	3	3	no	spread	oxer	right	no	0	0	64,18	no
Mechelen	The Longines FEI World Cup Jumping - Mechelen round A	30.12.2017	CSI-W5*	3	1.60	390	156	14	28.07.2004	M	AES	262	25	33	23	23	1854	199	M	4	4	no	vertical	vertical	right	no	0	0	64,18	no
Mechelen	The Longines FEI World Cup Jumping - Mechelen round A	30.12.2017	CSI-W5*	3	1.60	390	156	14	28.07.2004	M	AES	262	25	33	23	23	1854	199	M	5	5a	yes	spread	oxer	left	no	0	0	64,18	no
Mechelen	The Longines FEI World Cup Jumping - Mechelen round A	30.12.2017	CSI-W5*	3	1.60	390	156	14	28.07.2004	M	AES	262	25	33	23	23	1854	199	M	6	5b	yes	vertical	vertical	left	no	0	0	64,18	no
Mechelen	The Longines FEI World Cup Jumping - Mechelen round A	30.12.2017	CSI-W5*	3	1.60	390	156	14	28.07.2004	M	AES	262	25	33	23	23	1854	199	M	7	6	no	spread	oxer	straight	no	0	0	64,18	no
Mechelen	The Longines FEI World Cup Jumping - Mechelen round A	30.12.2017	CSI-W5*	3	1.60	390	156	14	28.07.2004	M	AES	262	25	33	23	23	1854	199	M	8	7	no	vertical	vertical with water	left	no	0	0	64,18	no
Mechelen	The Longines FEI World Cup Jumping - Mechelen round A	30.12.2017	CSI-W5*	3	1.60	390	156	14	28.07.2004	M	AES	262	25	33	23	23	1854	199	M	9	8	no	spread	triple bar	left	no	0	0	64,18	no
Mechelen	The Longines FEI World Cup Jumping - Mechelen round A	30.12.2017	CSI-W5*	3	1.60	390	156	14	28.07.2004	M	AES	262	25	33	23	23	1854	199	M	10	9	no	vertical	vertical	left	no	0	0	64,18	no
Mechelen	The Longines FEI World Cup Jumping - Mechelen round A	30.12.2017	CSI-W5*	3	1.60	390	156	14	28.07.2004	M	AES	262	25	33	23	23	1854	199	M	11	10	no	spread	oxer	right	yes	4	0	64,18	no
Mechelen	The Longines FEI World Cup Jumping - Mechelen round A	30.12.2017	CSI-W5*	3	1.60	390	156	14	28.07.2004	M	AES	262	25	33	23	23	1854	199	M	12	11a	yes	vertical	vertical	right	no	0	0	64,18	no
Mechelen	The Longines FEI World Cup Jumping - Mechelen round A	30.12.2017	CSI-W5*	3	1.60	390	156	14	28.07.2004	M	AES	262	25	33	23	23	1854	199	M	13	11b	yes	spread	oxer	right	no	0	0	64,18	no
Mechelen	The Longines FEI World Cup Jumping - Mechelen round A	30.12.2017	CSI-W5*	3	1.60	390	156	14	28.07.2004	M	AES	262	25	33	23	23	1854	199	M	14	11c	yes	spread	oxer	right	no	0	0	64,18	no
Mechelen	The Longines FEI World Cup Jumping - Mechelen round A	30.12.2017	CSI-W5*	3	1.60	390	156	14	28.07.2004	M	AES	262	25	33	23	23	1854	199	M	15	12	no	vertical	vertical	left	no	0	0	64,18	no
Mechelen	The Longines FEI World Cup Jumping - Mechelen round A	30.12.2017	CSI-W5*	3	1.60	390	156	14	28.07.2004	M	AES	262	25	33	23	23	1854	199	M	16	13	no	spread	oxer	left	no	0	0	64,18	no
Mechelen	The Longines FEI World Cup Jumping - Mechelen round A	30.12.2017	CSI-W5*	3	1.60	390	208	10	18.05.2008	G	SF	126	1	43	134	48	1808	64	M	1	1	no	spread	oxer	left	no	0	0	68,23	no
Mechelen	The Longines FEI World Cup Jumping - Mechelen round A	30.12.2017	CSI-W5*	3	1.60	390	208	10	18.05.2008	G	SF	126	1	43	134	48	1808	64	M	2	2	no	vertical	vertical	straight	no	0	0	68,23	no
Mechelen	The Longines FEI World Cup Jumping - Mechelen round A	30.12.2017	CSI-W5*	3	1.60	390	208	10	18.05.2008	G	SF	126	1	43	134	48	1808	64	M	3	3	no	spread	oxer	right	no	0	0	68,23	no
Mechelen	The Longines FEI World Cup Jumping - Mechelen round A	30.12.2017	CSI-W5*	3	1.60	390	208	10	18.05.2008	G	SF	126	1	43	134	48	1808	64	M	4	4	no	vertical	vertical	right	no	0	0	68,23	no
Mechelen	The Longines FEI World Cup Jumping - Mechelen round A	30.12.2017	CSI-W5*	3	1.60	390	208	10	18.05.2008	G	SF	126	1	43	134	48	1808	64	M	5	5a	yes	spread	oxer	left	no	0	0	68,23	no
Mechelen	The Longines FEI World Cup Jumping - Mechelen round A	30.12.2017	CSI-W5*	3	1.60	390	208	10	18.05.2008	G	SF	126	1	43	134	48	1808	64	M	6	5b	yes	vertical	vertical	left	no	0	0	68,23	no
Mechelen	The Longines FEI World Cup Jumping - Mechelen round A	30.12.2017	CSI-W5*	3	1.60	390	208	10	18.05.2008	G	SF	126	1	43	134	48	1808	64	M	7	6	no	spread	oxer	straight	no	0	0	68,23	no
Mechelen	The Longines FEI World Cup Jumping - Mechelen round A	30.12.2017	CSI-W5*	3	1.60	390	208	10	18.05.2008	G	SF	126	1	43	134	48	1808	64	M	8	7	no	vertical	vertical with water	left	no	0	0	68,23	no
Mechelen	The Longines FEI World Cup Jumping - Mechelen round A	30.12.2017	CSI-W5*	3	1.60	390	208	10	18.05.2008	G	SF	126	1	43	134	48	1808	64	M	9	8	no	spread	triple bar	left	no	0	0	68,23	no
Mechelen	The Longines FEI World Cup Jumping - Mechelen round A	30.12.2017	CSI-W5*	3	1.60	390	208	10	18.05.2008	G	SF	126	1	43	134	48	1808	64	M	10	9	no	vertical	vertical	left	no	0	0	68,23	no
Mechelen	The Longines FEI World Cup Jumping - Mechelen round A	30.12.2017	CSI-W5*	3	1.60	390	208	10	18.05.2008	G	SF	126	1	43	134	48	1808	64	M	11	10	no	spread	oxer	right	no	0	0	68,23	no
Mechelen	The Longines FEI World Cup Jumping - Mechelen round A	30.12.2017	CSI-W5*	3	1.60	390	208	10	18.05.2008	G	SF	126	1	43	134	48	1808	64	M	12	11a	yes	vertical	vertical	right	no	0	0	68,23	no
Mechelen	The Longines FEI World Cup Jumping - Mechelen round A	30.12.2017	CSI-W5*	3	1.60	390	208	10	18.05.2008	G	SF	126	1	43	134	48	1808	64	M	13	11b	yes	spread	oxer	right	no	0	0	68,23	no
Mechelen	The Longines FEI World Cup Jumping - Mechelen round A	30.12.2017	CSI-W5*	3	1.60	390	208	10	18.05.2008	G	SF	126	1	43	134	48	1808	64	M	14	11c	yes	spread	oxer	right	no	0	0	68,23	no
Mechelen	The Longines FEI World Cup Jumping - Mechelen round A	30.12.2017	CSI-W5*	3	1.60	390	208	10	18.05.2008	G	SF	126	1	43	134	48	1808	64	M	15	12	no	vertical	vertical	left	no	0	0	68,23	no
Mechelen	The Longines FEI World Cup Jumping - Mechelen round A	30.12.2017	CSI-W5*	3	1.60	390	208	10	18.05.2008	G	SF	126	1	43	134	48	1808	64	M	16	13	no	spread	oxer	left	no	0	1	68,23	no
Mechelen	The Longines FEI World Cup Jumping - Mechelen round A	30.12.2017	CSI-W5*	3	1.60	390	105	12	02.04.2006	M	DSP	186	8	50	30	25	1981	78	M	1	1	no	spread	oxer	left	no	0	0	65,39	no
Mechelen	The Longines FEI World Cup Jumping - Mechelen round A	30.12.2017	CSI-W5*	3	1.60	390	105	12	02.04.2006	M	DSP	186	8	50	30	25	1981	78	M	2	2	no	vertical	vertical	straight	no	0	0	65,39	no
Mechelen	The Longines FEI World Cup Jumping - Mechelen round A	30.12.2017	CSI-W5*	3	1.60	390	105	12	02.04.2006	M	DSP	186	8	50	30	25	1981	78	M	3	3	no	spread	oxer	right	no	0	0	65,39	no
Mechelen	The Longines FEI World Cup Jumping - Mechelen round A	30.12.2017	CSI-W5*	3	1.60	390	105	12	02.04.2006	M	DSP	186	8	50	30	25	1981	78	M	4	4	no	vertical	vertical	right	no	0	0	65,39	no
Mechelen	The Longines FEI World Cup Jumping - Mechelen round A	30.12.2017	CSI-W5*	3	1.60	390	105	12	02.04.2006	M	DSP	186	8	50	30	25	1981	78	M	5	5a	yes	spread	oxer	left	no	0	0	65,39	no
Mechelen	The Longines FEI World Cup Jumping - Mechelen round A	30.12.2017	CSI-W5*	3	1.60	390	105	12	02.04.2006	M	DSP	186	8	50	30	25	1981	78	M	6	5b	yes	vertical	vertical	left	no	0	0	65,39	no
Mechelen	The Longines FEI World Cup Jumping - Mechelen round A	30.12.2017	CSI-W5*	3	1.60	390	105	12	02.04.2006	M	DSP	186	8	50	30	25	1981	78	M	7	6	no	spread	oxer	straight	no	0	0	65,39	no
Mechelen	The Longines FEI World Cup Jumping - Mechelen round A	30.12.2017	CSI-W5*	3	1.60	390	105	12	02.04.2006	M	DSP	186	8	50	30	25	1981	78	M	8	7	no	vertical	vertical with water	left	no	0	0	65,39	no
Mechelen	The Longines FEI World Cup Jumping - Mechelen round A	30.12.2017	CSI-W5*	3	1.60	390	105	12	02.04.2006	M	DSP	186	8	50	30	25	1981	78	M	9	8	no	spread	triple bar	left	no	0	0	65,39	no
Mechelen	The Longines FEI World Cup Jumping - Mechelen round A	30.12.2017	CSI-W5*	3	1.60	390	105	12	02.04.2006	M	DSP	186	8	50	30	25	1981	78	M	10	9	no	vertical	vertical	left	no	0	0	65,39	no
Mechelen	The Longines FEI World Cup Jumping - Mechelen round A	30.12.2017	CSI-W5*	3	1.60	390	105	12	02.04.2006	M	DSP	186	8	50	30	25	1981	78	M	11	10	no	spread	oxer	right	no	0	0	65,39	no
Mechelen	The Longines FEI World Cup Jumping - Mechelen round A	30.12.2017	CSI-W5*	3	1.60	390	105	12	02.04.2006	M	DSP	186	8	50	30	25	1981	78	M	12	11a	yes	vertical	vertical	right	no	0	0	65,39	no
Mechelen	The Longines FEI World Cup Jumping - Mechelen round A	30.12.2017	CSI-W5*	3	1.60	390	105	12	02.04.2006	M	DSP	186	8	50	30	25	1981	78	M	13	11b	yes	spread	oxer	right	no	0	0	65,39	no
Mechelen	The Longines FEI World Cup Jumping - Mechelen round A	30.12.2017	CSI-W5*	3	1.60	390	105	12	02.04.2006	M	DSP	186	8	50	30	25	1981	78	M	14	11c	yes	spread	oxer	right	no	0	0	65,39	no
Mechelen	The Longines FEI World Cup Jumping - Mechelen round A	30.12.2017	CSI-W5*	3	1.60	390	105	12	02.04.2006	M	DSP	186	8	50	30	25	1981	78	M	15	12	no	vertical	vertical	left	no	0	0	65,39	no
Mechelen	The Longines FEI World Cup Jumping - Mechelen round A	30.12.2017	CSI-W5*	3	1.60	390	105	12	02.04.2006	M	DSP	186	8	50	30	25	1981	78	M	16	13	no	spread	oxer	left	yes	4	0	65,39	no
Mechelen	The Longines FEI World Cup Jumping - Mechelen round A	30.12.2017	CSI-W5*	3	1.60	390	13	15	01.05.2003	S	WESTF	239	19	52	52	32	2035	202	M	1	1	no	spread	oxer	left	no	0	0	65,39	no
Mechelen	The Longines FEI World Cup Jumping - Mechelen round A	30.12.2017	CSI-W5*	3	1.60	390	13	15	01.05.2003	S	WESTF	239	19	52	52	32	2035	202	M	2	2	no	vertical	vertical	straight	no	0	0	65,39	no
Mechelen	The Longines FEI World Cup Jumping - Mechelen round A	30.12.2017	CSI-W5*	3	1.60	390	13	15	01.05.2003	S	WESTF	239	19	52	52	32	2035	202	M	3	3	no	spread	oxer	right	no	0	0	65,39	no
Mechelen	The Longines FEI World Cup Jumping - Mechelen round A	30.12.2017	CSI-W5*	3	1.60	390	13	15	01.05.2003	S	WESTF	239	19	52	52	32	2035	202	M	4	4	no	vertical	vertical	right	no	0	0	65,39	no
Mechelen	The Longines FEI World Cup Jumping - Mechelen round A	30.12.2017	CSI-W5*	3	1.60	390	13	15	01.05.2003	S	WESTF	239	19	52	52	32	2035	202	M	5	5a	yes	spread	oxer	left	yes	4	0	65,39	no
Mechelen	The Longines FEI World Cup Jumping - Mechelen round A	30.12.2017	CSI-W5*	3	1.60	390	13	15	01.05.2003	S	WESTF	239	19	52	52	32	2035	202	M	6	5b	yes	vertical	vertical	left	no	0	0	65,39	no
Mechelen	The Longines FEI World Cup Jumping - Mechelen round A	30.12.2017	CSI-W5*	3	1.60	390	13	15	01.05.2003	S	WESTF	239	19	52	52	32	2035	202	M	7	6	no	spread	oxer	straight	no	0	0	65,39	no
Mechelen	The Longines FEI World Cup Jumping - Mechelen round A	30.12.2017	CSI-W5*	3	1.60	390	13	15	01.05.2003	S	WESTF	239	19	52	52	32	2035	202	M	8	7	no	vertical	vertical with water	left	no	0	0	65,39	no
Mechelen	The Longines FEI World Cup Jumping - Mechelen round A	30.12.2017	CSI-W5*	3	1.60	390	13	15	01.05.2003	S	WESTF	239	19	52	52	32	2035	202	M	9	8	no	spread	triple bar	left	no	0	0	65,39	no
Mechelen	The Longines FEI World Cup Jumping - Mechelen round A	30.12.2017	CSI-W5*	3	1.60	390	13	15	01.05.2003	S	WESTF	239	19	52	52	32	2035	202	M	10	9	no	vertical	vertical	left	no	0	0	65,39	no
Mechelen	The Longines FEI World Cup Jumping - Mechelen round A	30.12.2017	CSI-W5*	3	1.60	390	13	15	01.05.2003	S	WESTF	239	19	52	52	32	2035	202	M	11	10	no	spread	oxer	right	yes	4	0	65,39	no
Mechelen	The Longines FEI World Cup Jumping - Mechelen round A	30.12.2017	CSI-W5*	3	1.60	390	13	15	01.05.2003	S	WESTF	239	19	52	52	32	2035	202	M	12	11a	yes	vertical	vertical	right	no	0	0	65,39	no
Mechelen	The Longines FEI World Cup Jumping - Mechelen round A	30.12.2017	CSI-W5*	3	1.60	390	13	15	01.05.2003	S	WESTF	239	19	52	52	32	2035	202	M	13	11b	yes	spread	oxer	right	no	0	0	65,39	no
Mechelen	The Longines FEI World Cup Jumping - Mechelen round A	30.12.2017	CSI-W5*	3	1.60	390	13	15	01.05.2003	S	WESTF	239	19	52	52	32	2035	202	M	14	11c	yes	spread	oxer	right	no	0	0	65,39	no
Mechelen	The Longines FEI World Cup Jumping - Mechelen round A	30.12.2017	CSI-W5*	3	1.60	390	13	15	01.05.2003	S	WESTF	239	19	52	52	32	2035	202	M	15	12	no	vertical	vertical	left	no	0	0	65,39	no
Mechelen	The Longines FEI World Cup Jumping - Mechelen round A	30.12.2017	CSI-W5*	3	1.60	390	13	15	01.05.2003	S	WESTF	239	19	52	52	32	2035	202	M	16	13	no	spread	oxer	left	no	0	0	65,39	no
Mechelen	The Longines FEI World Cup Jumping - Mechelen round A	30.12.2017	CSI-W5*	3	1.60	390	152	12	20.05.2006	M	WESTF	94	7	55	6	37	1750	61	M	1	1	no	spread	oxer	left	no	0	0	65,12	no
Mechelen	The Longines FEI World Cup Jumping - Mechelen round A	30.12.2017	CSI-W5*	3	1.60	390	152	12	20.05.2006	M	WESTF	94	7	55	6	37	1750	61	M	2	2	no	vertical	vertical	straight	no	0	0	65,12	no
Mechelen	The Longines FEI World Cup Jumping - Mechelen round A	30.12.2017	CSI-W5*	3	1.60	390	152	12	20.05.2006	M	WESTF	94	7	55	6	37	1750	61	M	3	3	no	spread	oxer	right	no	0	0	65,12	no
Mechelen	The Longines FEI World Cup Jumping - Mechelen round A	30.12.2017	CSI-W5*	3	1.60	390	152	12	20.05.2006	M	WESTF	94	7	55	6	37	1750	61	M	4	4	no	vertical	vertical	right	no	0	0	65,12	no
Mechelen	The Longines FEI World Cup Jumping - Mechelen round A	30.12.2017	CSI-W5*	3	1.60	390	152	12	20.05.2006	M	WESTF	94	7	55	6	37	1750	61	M	5	5a	yes	spread	oxer	left	no	0	0	65,12	no
Mechelen	The Longines FEI World Cup Jumping - Mechelen round A	30.12.2017	CSI-W5*	3	1.60	390	152	12	20.05.2006	M	WESTF	94	7	55	6	37	1750	61	M	6	5b	yes	vertical	vertical	left	no	0	0	65,12	no
Mechelen	The Longines FEI World Cup Jumping - Mechelen round A	30.12.2017	CSI-W5*	3	1.60	390	152	12	20.05.2006	M	WESTF	94	7	55	6	37	1750	61	M	7	6	no	spread	oxer	straight	no	0	0	65,12	no
Mechelen	The Longines FEI World Cup Jumping - Mechelen round A	30.12.2017	CSI-W5*	3	1.60	390	152	12	20.05.2006	M	WESTF	94	7	55	6	37	1750	61	M	8	7	no	vertical	vertical with water	left	no	0	0	65,12	no
Mechelen	The Longines FEI World Cup Jumping - Mechelen round A	30.12.2017	CSI-W5*	3	1.60	390	152	12	20.05.2006	M	WESTF	94	7	55	6	37	1750	61	M	9	8	no	spread	triple bar	left	no	0	0	65,12	no
Mechelen	The Longines FEI World Cup Jumping - Mechelen round A	30.12.2017	CSI-W5*	3	1.60	390	152	12	20.05.2006	M	WESTF	94	7	55	6	37	1750	61	M	10	9	no	vertical	vertical	left	no	0	0	65,12	no
Mechelen	The Longines FEI World Cup Jumping - Mechelen round A	30.12.2017	CSI-W5*	3	1.60	390	152	12	20.05.2006	M	WESTF	94	7	55	6	37	1750	61	M	11	10	no	spread	oxer	right	no	0	0	65,12	no
Mechelen	The Longines FEI World Cup Jumping - Mechelen round A	30.12.2017	CSI-W5*	3	1.60	390	152	12	20.05.2006	M	WESTF	94	7	55	6	37	1750	61	M	12	11a	yes	vertical	vertical	right	no	0	0	65,12	no
Mechelen	The Longines FEI World Cup Jumping - Mechelen round A	30.12.2017	CSI-W5*	3	1.60	390	152	12	20.05.2006	M	WESTF	94	7	55	6	37	1750	61	M	13	11b	yes	spread	oxer	right	no	0	0	65,12	no
Mechelen	The Longines FEI World Cup Jumping - Mechelen round A	30.12.2017	CSI-W5*	3	1.60	390	152	12	20.05.2006	M	WESTF	94	7	55	6	37	1750	61	M	14	11c	yes	spread	oxer	right	no	0	0	65,12	no
Mechelen	The Longines FEI World Cup Jumping - Mechelen round A	30.12.2017	CSI-W5*	3	1.60	390	152	12	20.05.2006	M	WESTF	94	7	55	6	37	1750	61	M	15	12	no	vertical	vertical	left	no	0	0	65,12	no
Mechelen	The Longines FEI World Cup Jumping - Mechelen round A	30.12.2017	CSI-W5*	3	1.60	390	152	12	20.05.2006	M	WESTF	94	7	55	6	37	1750	61	M	16	13	no	spread	oxer	left	no	0	0	65,12	no
Mechelen	The Longines FEI World Cup Jumping - Mechelen round B	30.12.2017	CSI-W5*	3	1.60	290	152	12	20.05.2006	M	WESTF	94	7	55	6	37	1750	61	M	1	1	no	spread	oxer	right	no	0	0	38,74	no
Mechelen	The Longines FEI World Cup Jumping - Mechelen round B	30.12.2017	CSI-W5*	3	1.60	290	152	12	20.05.2006	M	WESTF	94	7	55	6	37	1750	61	M	2	2	no	vertical	vertical	right	no	0	0	38,74	no
Mechelen	The Longines FEI World Cup Jumping - Mechelen round B	30.12.2017	CSI-W5*	3	1.60	290	152	12	20.05.2006	M	WESTF	94	7	55	6	37	1750	61	M	3	3	no	spread	oxer	left	no	0	0	38,74	no
Mechelen	The Longines FEI World Cup Jumping - Mechelen round B	30.12.2017	CSI-W5*	3	1.60	290	152	12	20.05.2006	M	WESTF	94	7	55	6	37	1750	61	M	4	5	no	vertical	vertical	left	no	0	0	38,74	no
Mechelen	The Longines FEI World Cup Jumping - Mechelen round B	30.12.2017	CSI-W5*	3	1.60	290	152	12	20.05.2006	M	WESTF	94	7	55	6	37	1750	61	M	5	6a	yes	vertical	vertical	right	no	0	0	38,74	no
Mechelen	The Longines FEI World Cup Jumping - Mechelen round B	30.12.2017	CSI-W5*	3	1.60	290	152	12	20.05.2006	M	WESTF	94	7	55	6	37	1750	61	M	6	6b	yes	spread	oxer	right	no	0	0	38,74	no
Mechelen	The Longines FEI World Cup Jumping - Mechelen round B	30.12.2017	CSI-W5*	3	1.60	290	152	12	20.05.2006	M	WESTF	94	7	55	6	37	1750	61	M	7	7	no	vertical	vertical	left	no	0	0	38,74	no
Mechelen	The Longines FEI World Cup Jumping - Mechelen round B	30.12.2017	CSI-W5*	3	1.60	290	152	12	20.05.2006	M	WESTF	94	7	55	6	37	1750	61	M	8	8	no	spread	triple bar	left	no	0	0	38,74	no
Mechelen	The Longines FEI World Cup Jumping - Mechelen round B	30.12.2017	CSI-W5*	3	1.60	290	152	12	20.05.2006	M	WESTF	94	7	55	6	37	1750	61	M	9	9	no	vertical	vertical	left	no	0	0	38,74	no
Mechelen	The Longines FEI World Cup Jumping - Mechelen round A	30.12.2017	CSI-W5*	3	1.60	390	194	18	07.04.2000	S	KWPN	239	27	64	20	44	2014	118	M	1	1	no	spread	oxer	left	no	0	0	66,54	no
Mechelen	The Longines FEI World Cup Jumping - Mechelen round A	30.12.2017	CSI-W5*	3	1.60	390	194	18	07.04.2000	S	KWPN	239	27	64	20	44	2014	118	M	2	2	no	vertical	vertical	straight	no	0	0	66,54	no
Mechelen	The Longines FEI World Cup Jumping - Mechelen round A	30.12.2017	CSI-W5*	3	1.60	390	194	18	07.04.2000	S	KWPN	239	27	64	20	44	2014	118	M	3	3	no	spread	oxer	right	no	0	0	66,54	no
Mechelen	The Longines FEI World Cup Jumping - Mechelen round A	30.12.2017	CSI-W5*	3	1.60	390	194	18	07.04.2000	S	KWPN	239	27	64	20	44	2014	118	M	4	4	no	vertical	vertical	right	no	0	0	66,54	no
Mechelen	The Longines FEI World Cup Jumping - Mechelen round A	30.12.2017	CSI-W5*	3	1.60	390	194	18	07.04.2000	S	KWPN	239	27	64	20	44	2014	118	M	5	5a	yes	spread	oxer	left	no	0	0	66,54	no
Mechelen	The Longines FEI World Cup Jumping - Mechelen round A	30.12.2017	CSI-W5*	3	1.60	390	194	18	07.04.2000	S	KWPN	239	27	64	20	44	2014	118	M	6	5b	yes	vertical	vertical	left	no	0	0	66,54	no
Mechelen	The Longines FEI World Cup Jumping - Mechelen round A	30.12.2017	CSI-W5*	3	1.60	390	194	18	07.04.2000	S	KWPN	239	27	64	20	44	2014	118	M	7	6	no	spread	oxer	straight	no	0	0	66,54	no
Mechelen	The Longines FEI World Cup Jumping - Mechelen round A	30.12.2017	CSI-W5*	3	1.60	390	194	18	07.04.2000	S	KWPN	239	27	64	20	44	2014	118	M	8	7	no	vertical	vertical with water	left	no	0	0	66,54	no
Mechelen	The Longines FEI World Cup Jumping - Mechelen round A	30.12.2017	CSI-W5*	3	1.60	390	194	18	07.04.2000	S	KWPN	239	27	64	20	44	2014	118	M	9	8	no	spread	triple bar	left	no	0	0	66,54	no
Mechelen	The Longines FEI World Cup Jumping - Mechelen round A	30.12.2017	CSI-W5*	3	1.60	390	194	18	07.04.2000	S	KWPN	239	27	64	20	44	2014	118	M	10	9	no	vertical	vertical	left	no	0	0	66,54	no
Mechelen	The Longines FEI World Cup Jumping - Mechelen round A	30.12.2017	CSI-W5*	3	1.60	390	194	18	07.04.2000	S	KWPN	239	27	64	20	44	2014	118	M	11	10	no	spread	oxer	right	no	0	0	66,54	no
Mechelen	The Longines FEI World Cup Jumping - Mechelen round A	30.12.2017	CSI-W5*	3	1.60	390	194	18	07.04.2000	S	KWPN	239	27	64	20	44	2014	118	M	12	11a	yes	vertical	vertical	right	no	0	0	66,54	no
Mechelen	The Longines FEI World Cup Jumping - Mechelen round A	30.12.2017	CSI-W5*	3	1.60	390	194	18	07.04.2000	S	KWPN	239	27	64	20	44	2014	118	M	13	11b	yes	spread	oxer	right	no	0	0	66,54	no
Mechelen	The Longines FEI World Cup Jumping - Mechelen round A	30.12.2017	CSI-W5*	3	1.60	390	194	18	07.04.2000	S	KWPN	239	27	64	20	44	2014	118	M	14	11c	yes	spread	oxer	right	no	0	0	66,54	no
Mechelen	The Longines FEI World Cup Jumping - Mechelen round A	30.12.2017	CSI-W5*	3	1.60	390	194	18	07.04.2000	S	KWPN	239	27	64	20	44	2014	118	M	15	12	no	vertical	vertical	left	no	0	0	66,54	no
Mechelen	The Longines FEI World Cup Jumping - Mechelen round A	30.12.2017	CSI-W5*	3	1.60	390	194	18	07.04.2000	S	KWPN	239	27	64	20	44	2014	118	M	16	13	no	spread	oxer	left	no	0	0	66,54	no
Mechelen	The Longines FEI World Cup Jumping - Mechelen round B	30.12.2017	CSI-W5*	3	1.60	290	194	18	07.04.2000	S	KWPN	239	27	64	20	44	2014	118	M	1	1	no	spread	oxer	right	no	0	0	38,83	no
Mechelen	The Longines FEI World Cup Jumping - Mechelen round B	30.12.2017	CSI-W5*	3	1.60	290	194	18	07.04.2000	S	KWPN	239	27	64	20	44	2014	118	M	2	2	no	vertical	vertical	right	no	0	0	38,83	no
Mechelen	The Longines FEI World Cup Jumping - Mechelen round B	30.12.2017	CSI-W5*	3	1.60	290	194	18	07.04.2000	S	KWPN	239	27	64	20	44	2014	118	M	3	3	no	spread	oxer	left	no	0	0	38,83	no
Mechelen	The Longines FEI World Cup Jumping - Mechelen round B	30.12.2017	CSI-W5*	3	1.60	290	194	18	07.04.2000	S	KWPN	239	27	64	20	44	2014	118	M	4	5	no	vertical	vertical	left	no	0	0	38,83	no
Mechelen	The Longines FEI World Cup Jumping - Mechelen round B	30.12.2017	CSI-W5*	3	1.60	290	194	18	07.04.2000	S	KWPN	239	27	64	20	44	2014	118	M	5	6a	yes	vertical	vertical	right	yes	4	0	38,83	no
Mechelen	The Longines FEI World Cup Jumping - Mechelen round B	30.12.2017	CSI-W5*	3	1.60	290	194	18	07.04.2000	S	KWPN	239	27	64	20	44	2014	118	M	6	6b	yes	spread	oxer	right	no	0	0	38,83	no
Mechelen	The Longines FEI World Cup Jumping - Mechelen round B	30.12.2017	CSI-W5*	3	1.60	290	194	18	07.04.2000	S	KWPN	239	27	64	20	44	2014	118	M	7	7	no	vertical	vertical	left	no	0	0	38,83	no
Mechelen	The Longines FEI World Cup Jumping - Mechelen round B	30.12.2017	CSI-W5*	3	1.60	290	194	18	07.04.2000	S	KWPN	239	27	64	20	44	2014	118	M	8	8	no	spread	triple bar	left	no	0	0	38,83	no
Mechelen	The Longines FEI World Cup Jumping - Mechelen round B	30.12.2017	CSI-W5*	3	1.60	290	194	18	07.04.2000	S	KWPN	239	27	64	20	44	2014	118	M	9	9	no	vertical	vertical	left	no	0	0	38,83	no
Mechelen	The Longines FEI World Cup Jumping - Mechelen round A	30.12.2017	CSI-W5*	3	1.60	390	71	10	25.05.2008	M	KWPN	131	2	65	70	43	1682	26	M	1	1	no	spread	oxer	left	no	0	0	68,8	no
Mechelen	The Longines FEI World Cup Jumping - Mechelen round A	30.12.2017	CSI-W5*	3	1.60	390	71	10	25.05.2008	M	KWPN	131	2	65	70	43	1682	26	M	2	2	no	vertical	vertical	straight	no	0	0	68,8	no
Mechelen	The Longines FEI World Cup Jumping - Mechelen round A	30.12.2017	CSI-W5*	3	1.60	390	71	10	25.05.2008	M	KWPN	131	2	65	70	43	1682	26	M	3	3	no	spread	oxer	right	no	0	0	68,8	no
Mechelen	The Longines FEI World Cup Jumping - Mechelen round A	30.12.2017	CSI-W5*	3	1.60	390	71	10	25.05.2008	M	KWPN	131	2	65	70	43	1682	26	M	4	4	no	vertical	vertical	right	no	0	0	68,8	no
Mechelen	The Longines FEI World Cup Jumping - Mechelen round A	30.12.2017	CSI-W5*	3	1.60	390	71	10	25.05.2008	M	KWPN	131	2	65	70	43	1682	26	M	5	5a	yes	spread	oxer	left	no	0	0	68,8	no
Mechelen	The Longines FEI World Cup Jumping - Mechelen round A	30.12.2017	CSI-W5*	3	1.60	390	71	10	25.05.2008	M	KWPN	131	2	65	70	43	1682	26	M	6	5b	yes	vertical	vertical	left	no	0	0	68,8	no
Mechelen	The Longines FEI World Cup Jumping - Mechelen round A	30.12.2017	CSI-W5*	3	1.60	390	71	10	25.05.2008	M	KWPN	131	2	65	70	43	1682	26	M	7	6	no	spread	oxer	straight	no	0	0	68,8	no
Mechelen	The Longines FEI World Cup Jumping - Mechelen round A	30.12.2017	CSI-W5*	3	1.60	390	71	10	25.05.2008	M	KWPN	131	2	65	70	43	1682	26	M	8	7	no	vertical	vertical with water	left	no	0	0	68,8	no
Mechelen	The Longines FEI World Cup Jumping - Mechelen round A	30.12.2017	CSI-W5*	3	1.60	390	71	10	25.05.2008	M	KWPN	131	2	65	70	43	1682	26	M	9	8	no	spread	triple bar	left	no	0	0	68,8	no
Mechelen	The Longines FEI World Cup Jumping - Mechelen round A	30.12.2017	CSI-W5*	3	1.60	390	71	10	25.05.2008	M	KWPN	131	2	65	70	43	1682	26	M	10	9	no	vertical	vertical	left	no	0	0	68,8	no
Mechelen	The Longines FEI World Cup Jumping - Mechelen round A	30.12.2017	CSI-W5*	3	1.60	390	71	10	25.05.2008	M	KWPN	131	2	65	70	43	1682	26	M	11	10	no	spread	oxer	right	no	0	0	68,8	no
Mechelen	The Longines FEI World Cup Jumping - Mechelen round A	30.12.2017	CSI-W5*	3	1.60	390	71	10	25.05.2008	M	KWPN	131	2	65	70	43	1682	26	M	12	11a	yes	vertical	vertical	right	no	0	0	68,8	no
Mechelen	The Longines FEI World Cup Jumping - Mechelen round A	30.12.2017	CSI-W5*	3	1.60	390	71	10	25.05.2008	M	KWPN	131	2	65	70	43	1682	26	M	13	11b	yes	spread	oxer	right	no	0	0	68,8	no
Mechelen	The Longines FEI World Cup Jumping - Mechelen round A	30.12.2017	CSI-W5*	3	1.60	390	71	10	25.05.2008	M	KWPN	131	2	65	70	43	1682	26	M	14	11c	yes	spread	oxer	right	no	0	0	68,8	no
Mechelen	The Longines FEI World Cup Jumping - Mechelen round A	30.12.2017	CSI-W5*	3	1.60	390	71	10	25.05.2008	M	KWPN	131	2	65	70	43	1682	26	M	15	12	no	vertical	vertical	left	no	0	0	68,8	no
Mechelen	The Longines FEI World Cup Jumping - Mechelen round A	30.12.2017	CSI-W5*	3	1.60	390	71	10	25.05.2008	M	KWPN	131	2	65	70	43	1682	26	M	16	13	no	spread	oxer	left	no	0	1	68,8	no
Mechelen	The Longines FEI World Cup Jumping - Mechelen round A	30.12.2017	CSI-W5*	3	1.60	390	99	12	08.06.2006	G	BWP	164	10	72	26	38	2171	177	M	1	1	no	spread	oxer	left	no	0	0	65,01	no
Mechelen	The Longines FEI World Cup Jumping - Mechelen round A	30.12.2017	CSI-W5*	3	1.60	390	99	12	08.06.2006	G	BWP	164	10	72	26	38	2171	177	M	2	2	no	vertical	vertical	straight	no	0	0	65,01	no
Mechelen	The Longines FEI World Cup Jumping - Mechelen round A	30.12.2017	CSI-W5*	3	1.60	390	99	12	08.06.2006	G	BWP	164	10	72	26	38	2171	177	M	3	3	no	spread	oxer	right	no	0	0	65,01	no
Mechelen	The Longines FEI World Cup Jumping - Mechelen round A	30.12.2017	CSI-W5*	3	1.60	390	99	12	08.06.2006	G	BWP	164	10	72	26	38	2171	177	M	4	4	no	vertical	vertical	right	no	0	0	65,01	no
Mechelen	The Longines FEI World Cup Jumping - Mechelen round A	30.12.2017	CSI-W5*	3	1.60	390	99	12	08.06.2006	G	BWP	164	10	72	26	38	2171	177	M	5	5a	yes	spread	oxer	left	no	0	0	65,01	no
Mechelen	The Longines FEI World Cup Jumping - Mechelen round A	30.12.2017	CSI-W5*	3	1.60	390	99	12	08.06.2006	G	BWP	164	10	72	26	38	2171	177	M	6	5b	yes	vertical	vertical	left	no	0	0	65,01	no
Mechelen	The Longines FEI World Cup Jumping - Mechelen round A	30.12.2017	CSI-W5*	3	1.60	390	99	12	08.06.2006	G	BWP	164	10	72	26	38	2171	177	M	7	6	no	spread	oxer	straight	no	0	0	65,01	no
Mechelen	The Longines FEI World Cup Jumping - Mechelen round A	30.12.2017	CSI-W5*	3	1.60	390	99	12	08.06.2006	G	BWP	164	10	72	26	38	2171	177	M	8	7	no	vertical	vertical with water	left	no	0	0	65,01	no
Mechelen	The Longines FEI World Cup Jumping - Mechelen round A	30.12.2017	CSI-W5*	3	1.60	390	99	12	08.06.2006	G	BWP	164	10	72	26	38	2171	177	M	9	8	no	spread	triple bar	left	no	0	0	65,01	no
Mechelen	The Longines FEI World Cup Jumping - Mechelen round A	30.12.2017	CSI-W5*	3	1.60	390	99	12	08.06.2006	G	BWP	164	10	72	26	38	2171	177	M	10	9	no	vertical	vertical	left	no	0	0	65,01	no
Mechelen	The Longines FEI World Cup Jumping - Mechelen round A	30.12.2017	CSI-W5*	3	1.60	390	99	12	08.06.2006	G	BWP	164	10	72	26	38	2171	177	M	11	10	no	spread	oxer	right	no	0	0	65,01	no
Mechelen	The Longines FEI World Cup Jumping - Mechelen round A	30.12.2017	CSI-W5*	3	1.60	390	99	12	08.06.2006	G	BWP	164	10	72	26	38	2171	177	M	12	11a	yes	vertical	vertical	right	no	0	0	65,01	no
Mechelen	The Longines FEI World Cup Jumping - Mechelen round A	30.12.2017	CSI-W5*	3	1.60	390	99	12	08.06.2006	G	BWP	164	10	72	26	38	2171	177	M	13	11b	yes	spread	oxer	right	no	0	0	65,01	no
Mechelen	The Longines FEI World Cup Jumping - Mechelen round A	30.12.2017	CSI-W5*	3	1.60	390	99	12	08.06.2006	G	BWP	164	10	72	26	38	2171	177	M	14	11c	yes	spread	oxer	right	no	0	0	65,01	no
Mechelen	The Longines FEI World Cup Jumping - Mechelen round A	30.12.2017	CSI-W5*	3	1.60	390	99	12	08.06.2006	G	BWP	164	10	72	26	38	2171	177	M	15	12	no	vertical	vertical	left	no	0	0	65,01	no
Mechelen	The Longines FEI World Cup Jumping - Mechelen round A	30.12.2017	CSI-W5*	3	1.60	390	99	12	08.06.2006	G	BWP	164	10	72	26	38	2171	177	M	16	13	no	spread	oxer	left	yes	4	0	65,01	no
Mechelen	The Longines FEI World Cup Jumping - Mechelen round A	30.12.2017	CSI-W5*	3	1.60	390	176	12	21.05.2006	M	SF	110	2	75	22	53	2104	161	M	1	1	no	spread	oxer	left	no	0	0	64,58	no
Mechelen	The Longines FEI World Cup Jumping - Mechelen round A	30.12.2017	CSI-W5*	3	1.60	390	176	12	21.05.2006	M	SF	110	2	75	22	53	2104	161	M	2	2	no	vertical	vertical	straight	no	0	0	64,58	no
Mechelen	The Longines FEI World Cup Jumping - Mechelen round A	30.12.2017	CSI-W5*	3	1.60	390	176	12	21.05.2006	M	SF	110	2	75	22	53	2104	161	M	3	3	no	spread	oxer	right	no	0	0	64,58	no
Mechelen	The Longines FEI World Cup Jumping - Mechelen round A	30.12.2017	CSI-W5*	3	1.60	390	176	12	21.05.2006	M	SF	110	2	75	22	53	2104	161	M	4	4	no	vertical	vertical	right	no	0	0	64,58	no
Mechelen	The Longines FEI World Cup Jumping - Mechelen round A	30.12.2017	CSI-W5*	3	1.60	390	176	12	21.05.2006	M	SF	110	2	75	22	53	2104	161	M	5	5a	yes	spread	oxer	left	no	0	0	64,58	no
Mechelen	The Longines FEI World Cup Jumping - Mechelen round A	30.12.2017	CSI-W5*	3	1.60	390	176	12	21.05.2006	M	SF	110	2	75	22	53	2104	161	M	6	5b	yes	vertical	vertical	left	no	0	0	64,58	no
Mechelen	The Longines FEI World Cup Jumping - Mechelen round A	30.12.2017	CSI-W5*	3	1.60	390	176	12	21.05.2006	M	SF	110	2	75	22	53	2104	161	M	7	6	no	spread	oxer	straight	no	0	0	64,58	no
Mechelen	The Longines FEI World Cup Jumping - Mechelen round A	30.12.2017	CSI-W5*	3	1.60	390	176	12	21.05.2006	M	SF	110	2	75	22	53	2104	161	M	8	7	no	vertical	vertical with water	left	no	0	0	64,58	no
Mechelen	The Longines FEI World Cup Jumping - Mechelen round A	30.12.2017	CSI-W5*	3	1.60	390	176	12	21.05.2006	M	SF	110	2	75	22	53	2104	161	M	9	8	no	spread	triple bar	left	no	0	0	64,58	no
Mechelen	The Longines FEI World Cup Jumping - Mechelen round A	30.12.2017	CSI-W5*	3	1.60	390	176	12	21.05.2006	M	SF	110	2	75	22	53	2104	161	M	10	9	no	vertical	vertical	left	no	0	0	64,58	no
Mechelen	The Longines FEI World Cup Jumping - Mechelen round A	30.12.2017	CSI-W5*	3	1.60	390	176	12	21.05.2006	M	SF	110	2	75	22	53	2104	161	M	11	10	no	spread	oxer	right	no	0	0	64,58	no
Mechelen	The Longines FEI World Cup Jumping - Mechelen round A	30.12.2017	CSI-W5*	3	1.60	390	176	12	21.05.2006	M	SF	110	2	75	22	53	2104	161	M	12	11a	yes	vertical	vertical	right	no	0	0	64,58	no
Mechelen	The Longines FEI World Cup Jumping - Mechelen round A	30.12.2017	CSI-W5*	3	1.60	390	176	12	21.05.2006	M	SF	110	2	75	22	53	2104	161	M	13	11b	yes	spread	oxer	right	no	0	0	64,58	no
Mechelen	The Longines FEI World Cup Jumping - Mechelen round A	30.12.2017	CSI-W5*	3	1.60	390	176	12	21.05.2006	M	SF	110	2	75	22	53	2104	161	M	14	11c	yes	spread	oxer	right	no	0	0	64,58	no
Mechelen	The Longines FEI World Cup Jumping - Mechelen round A	30.12.2017	CSI-W5*	3	1.60	390	176	12	21.05.2006	M	SF	110	2	75	22	53	2104	161	M	15	12	no	vertical	vertical	left	no	0	0	64,58	no
Mechelen	The Longines FEI World Cup Jumping - Mechelen round A	30.12.2017	CSI-W5*	3	1.60	390	176	12	21.05.2006	M	SF	110	2	75	22	53	2104	161	M	16	13	no	spread	oxer	left	no	0	0	64,58	no
Mechelen	The Longines FEI World Cup Jumping - Mechelen round B	30.12.2017	CSI-W5*	3	1.60	290	176	12	21.05.2006	M	SF	110	2	75	22	53	2104	161	M	1	1	no	spread	oxer	right	no	0	0	42,17	no
Mechelen	The Longines FEI World Cup Jumping - Mechelen round B	30.12.2017	CSI-W5*	3	1.60	290	176	12	21.05.2006	M	SF	110	2	75	22	53	2104	161	M	2	2	no	vertical	vertical	right	no	0	0	42,17	no
Mechelen	The Longines FEI World Cup Jumping - Mechelen round B	30.12.2017	CSI-W5*	3	1.60	290	176	12	21.05.2006	M	SF	110	2	75	22	53	2104	161	M	3	3	no	spread	oxer	left	no	0	0	42,17	no
Mechelen	The Longines FEI World Cup Jumping - Mechelen round B	30.12.2017	CSI-W5*	3	1.60	290	176	12	21.05.2006	M	SF	110	2	75	22	53	2104	161	M	4	5	no	vertical	vertical	left	no	0	0	42,17	no
Mechelen	The Longines FEI World Cup Jumping - Mechelen round B	30.12.2017	CSI-W5*	3	1.60	290	176	12	21.05.2006	M	SF	110	2	75	22	53	2104	161	M	5	6a	yes	vertical	vertical	right	no	0	0	42,17	no
Mechelen	The Longines FEI World Cup Jumping - Mechelen round B	30.12.2017	CSI-W5*	3	1.60	290	176	12	21.05.2006	M	SF	110	2	75	22	53	2104	161	M	6	6b	yes	spread	oxer	right	no	0	0	42,17	no
Mechelen	The Longines FEI World Cup Jumping - Mechelen round B	30.12.2017	CSI-W5*	3	1.60	290	176	12	21.05.2006	M	SF	110	2	75	22	53	2104	161	M	7	7	no	vertical	vertical	left	no	0	0	42,17	no
Mechelen	The Longines FEI World Cup Jumping - Mechelen round B	30.12.2017	CSI-W5*	3	1.60	290	176	12	21.05.2006	M	SF	110	2	75	22	53	2104	161	M	8	8	no	spread	triple bar	left	no	0	0	42,17	no
Mechelen	The Longines FEI World Cup Jumping - Mechelen round B	30.12.2017	CSI-W5*	3	1.60	290	176	12	21.05.2006	M	SF	110	2	75	22	53	2104	161	M	9	9	no	vertical	vertical	left	no	0	0	42,17	no
Mechelen	The Longines FEI World Cup Jumping - Mechelen round A	30.12.2017	CSI-W5*	3	1.60	390	120	11	28.04.2007	S	HOLST	147	1	76	55	44	1886	48	M	1	1	no	spread	oxer	left	no	0	0	64,15	no
Mechelen	The Longines FEI World Cup Jumping - Mechelen round A	30.12.2017	CSI-W5*	3	1.60	390	120	11	28.04.2007	S	HOLST	147	1	76	55	44	1886	48	M	2	2	no	vertical	vertical	straight	no	0	0	64,15	no
Mechelen	The Longines FEI World Cup Jumping - Mechelen round A	30.12.2017	CSI-W5*	3	1.60	390	120	11	28.04.2007	S	HOLST	147	1	76	55	44	1886	48	M	3	3	no	spread	oxer	right	no	0	0	64,15	no
Mechelen	The Longines FEI World Cup Jumping - Mechelen round A	30.12.2017	CSI-W5*	3	1.60	390	120	11	28.04.2007	S	HOLST	147	1	76	55	44	1886	48	M	4	4	no	vertical	vertical	right	yes	4	0	64,15	no
Mechelen	The Longines FEI World Cup Jumping - Mechelen round A	30.12.2017	CSI-W5*	3	1.60	390	120	11	28.04.2007	S	HOLST	147	1	76	55	44	1886	48	M	5	5a	yes	spread	oxer	left	no	0	0	64,15	no
Mechelen	The Longines FEI World Cup Jumping - Mechelen round A	30.12.2017	CSI-W5*	3	1.60	390	120	11	28.04.2007	S	HOLST	147	1	76	55	44	1886	48	M	6	5b	yes	vertical	vertical	left	no	0	0	64,15	no
Mechelen	The Longines FEI World Cup Jumping - Mechelen round A	30.12.2017	CSI-W5*	3	1.60	390	120	11	28.04.2007	S	HOLST	147	1	76	55	44	1886	48	M	7	6	no	spread	oxer	straight	no	0	0	64,15	no
Mechelen	The Longines FEI World Cup Jumping - Mechelen round A	30.12.2017	CSI-W5*	3	1.60	390	120	11	28.04.2007	S	HOLST	147	1	76	55	44	1886	48	M	8	7	no	vertical	vertical with water	left	no	0	0	64,15	no
Mechelen	The Longines FEI World Cup Jumping - Mechelen round A	30.12.2017	CSI-W5*	3	1.60	390	120	11	28.04.2007	S	HOLST	147	1	76	55	44	1886	48	M	9	8	no	spread	triple bar	left	no	0	0	64,15	no
Mechelen	The Longines FEI World Cup Jumping - Mechelen round A	30.12.2017	CSI-W5*	3	1.60	390	120	11	28.04.2007	S	HOLST	147	1	76	55	44	1886	48	M	10	9	no	vertical	vertical	left	no	0	0	64,15	no
Mechelen	The Longines FEI World Cup Jumping - Mechelen round A	30.12.2017	CSI-W5*	3	1.60	390	120	11	28.04.2007	S	HOLST	147	1	76	55	44	1886	48	M	11	10	no	spread	oxer	right	yes	4	0	64,15	no
Mechelen	The Longines FEI World Cup Jumping - Mechelen round A	30.12.2017	CSI-W5*	3	1.60	390	120	11	28.04.2007	S	HOLST	147	1	76	55	44	1886	48	M	12	11a	yes	vertical	vertical	right	no	0	0	64,15	no
Mechelen	The Longines FEI World Cup Jumping - Mechelen round A	30.12.2017	CSI-W5*	3	1.60	390	120	11	28.04.2007	S	HOLST	147	1	76	55	44	1886	48	M	13	11b	yes	spread	oxer	right	no	0	0	64,15	no
Mechelen	The Longines FEI World Cup Jumping - Mechelen round A	30.12.2017	CSI-W5*	3	1.60	390	120	11	28.04.2007	S	HOLST	147	1	76	55	44	1886	48	M	14	11c	yes	spread	oxer	right	no	0	0	64,15	no
Mechelen	The Longines FEI World Cup Jumping - Mechelen round A	30.12.2017	CSI-W5*	3	1.60	390	120	11	28.04.2007	S	HOLST	147	1	76	55	44	1886	48	M	15	12	no	vertical	vertical	left	yes	4	0	64,15	no
Mechelen	The Longines FEI World Cup Jumping - Mechelen round A	30.12.2017	CSI-W5*	3	1.60	390	120	11	28.04.2007	S	HOLST	147	1	76	55	44	1886	48	M	16	13	no	spread	oxer	left	yes	4	0	64,15	no
Mechelen	The Longines FEI World Cup Jumping - Mechelen round A	30.12.2017	CSI-W5*	3	1.60	390	181	12	20.03.2006	G	ZANG	214	6	81	76	33	1923	71	M	1	1	no	spread	oxer	left	no	0	0	0,00	yes
Mechelen	The Longines FEI World Cup Jumping - Mechelen round A	30.12.2017	CSI-W5*	3	1.60	390	181	12	20.03.2006	G	ZANG	214	6	81	76	33	1923	71	M	2	2	no	vertical	vertical	straight	no	0	0	0,00	yes
Mechelen	The Longines FEI World Cup Jumping - Mechelen round A	30.12.2017	CSI-W5*	3	1.60	390	181	12	20.03.2006	G	ZANG	214	6	81	76	33	1923	71	M	3	3	no	spread	oxer	right	no	0	0	0,00	yes
Mechelen	The Longines FEI World Cup Jumping - Mechelen round A	30.12.2017	CSI-W5*	3	1.60	390	181	12	20.03.2006	G	ZANG	214	6	81	76	33	1923	71	M	4	4	no	vertical	vertical	right	no	0	0	0,00	yes
Mechelen	The Longines FEI World Cup Jumping - Mechelen round A	30.12.2017	CSI-W5*	3	1.60	390	181	12	20.03.2006	G	ZANG	214	6	81	76	33	1923	71	M	5	5a	yes	spread	oxer	left	no	0	0	0,00	yes
Mechelen	The Longines FEI World Cup Jumping - Mechelen round A	30.12.2017	CSI-W5*	3	1.60	390	181	12	20.03.2006	G	ZANG	214	6	81	76	33	1923	71	M	6	5b	yes	vertical	vertical	left	no	0	0	0,00	yes
Mechelen	The Longines FEI World Cup Jumping - Mechelen round A	30.12.2017	CSI-W5*	3	1.60	390	181	12	20.03.2006	G	ZANG	214	6	81	76	33	1923	71	M	7	6	no	spread	oxer	straight	yes	4	0	0,00	yes
Mechelen	The Longines FEI World Cup Jumping - Mechelen round A	30.12.2017	CSI-W5*	3	1.60	390	181	12	20.03.2006	G	ZANG	214	6	81	76	33	1923	71	M	8	7	no	vertical	vertical with water	left	no	0	0	0,00	yes
Mechelen	The Longines FEI World Cup Jumping - Mechelen round A	30.12.2017	CSI-W5*	3	1.60	390	181	12	20.03.2006	G	ZANG	214	6	81	76	33	1923	71	M	9	8	no	spread	triple bar	left	no	0	0	0,00	yes
Mechelen	The Longines FEI World Cup Jumping - Mechelen round A	30.12.2017	CSI-W5*	3	1.60	390	181	12	20.03.2006	G	ZANG	214	6	81	76	33	1923	71	M	10	9	no	vertical	vertical	left	yes	4	0	0,00	yes
Mechelen	The Longines FEI World Cup Jumping - Mechelen round A	30.12.2017	CSI-W5*	3	1.60	390	181	12	20.03.2006	G	ZANG	214	6	81	76	33	1923	71	M	11	10	no	spread	oxer	right	no	0	0	0,00	yes
Mechelen	The Longines FEI World Cup Jumping - Mechelen round A	30.12.2017	CSI-W5*	3	1.60	390	181	12	20.03.2006	G	ZANG	214	6	81	76	33	1923	71	M	12	11a	yes	vertical	vertical	right	no	0	0	0,00	yes
Mechelen	The Longines FEI World Cup Jumping - Mechelen round A	30.12.2017	CSI-W5*	3	1.60	390	181	12	20.03.2006	G	ZANG	214	6	81	76	33	1923	71	M	13	11b	yes	spread	oxer	right	no	0	0	0,00	yes
Mechelen	The Longines FEI World Cup Jumping - Mechelen round A	30.12.2017	CSI-W5*	3	1.60	390	181	12	20.03.2006	G	ZANG	214	6	81	76	33	1923	71	M	14	11c	yes	spread	oxer	right	no	0	0	0,00	yes
Mechelen	The Longines FEI World Cup Jumping - Mechelen round A	30.12.2017	CSI-W5*	3	1.60	390	181	12	20.03.2006	G	ZANG	214	6	81	76	33	1923	71	M	15	12	no	vertical	vertical	left	no	0	0	0,00	yes
Mechelen	The Longines FEI World Cup Jumping - Mechelen round A	30.12.2017	CSI-W5*	3	1.60	390	181	12	20.03.2006	G	ZANG	214	6	81	76	33	1923	71	M	16	13	no	spread	oxer	left	no	0	0	0,00	yes
Mechelen	The Longines FEI World Cup Jumping - Mechelen round A	30.12.2017	CSI-W5*	3	1.60	390	210	10	16.04.2008	G	SF	156	13	85	41	41	2052	191	M	1	1	no	spread	oxer	left	no	0	0	64	no
Mechelen	The Longines FEI World Cup Jumping - Mechelen round A	30.12.2017	CSI-W5*	3	1.60	390	210	10	16.04.2008	G	SF	156	13	85	41	41	2052	191	M	2	2	no	vertical	vertical	straight	no	0	0	64	no
Mechelen	The Longines FEI World Cup Jumping - Mechelen round A	30.12.2017	CSI-W5*	3	1.60	390	210	10	16.04.2008	G	SF	156	13	85	41	41	2052	191	M	3	3	no	spread	oxer	right	no	0	0	64	no
Mechelen	The Longines FEI World Cup Jumping - Mechelen round A	30.12.2017	CSI-W5*	3	1.60	390	210	10	16.04.2008	G	SF	156	13	85	41	41	2052	191	M	4	4	no	vertical	vertical	right	no	0	0	64	no
Mechelen	The Longines FEI World Cup Jumping - Mechelen round A	30.12.2017	CSI-W5*	3	1.60	390	210	10	16.04.2008	G	SF	156	13	85	41	41	2052	191	M	5	5a	yes	spread	oxer	left	no	0	0	64	no
Mechelen	The Longines FEI World Cup Jumping - Mechelen round A	30.12.2017	CSI-W5*	3	1.60	390	210	10	16.04.2008	G	SF	156	13	85	41	41	2052	191	M	6	5b	yes	vertical	vertical	left	no	0	0	64	no
Mechelen	The Longines FEI World Cup Jumping - Mechelen round A	30.12.2017	CSI-W5*	3	1.60	390	210	10	16.04.2008	G	SF	156	13	85	41	41	2052	191	M	7	6	no	spread	oxer	straight	no	0	0	64	no
Mechelen	The Longines FEI World Cup Jumping - Mechelen round A	30.12.2017	CSI-W5*	3	1.60	390	210	10	16.04.2008	G	SF	156	13	85	41	41	2052	191	M	8	7	no	vertical	vertical with water	left	no	0	0	64	no
Mechelen	The Longines FEI World Cup Jumping - Mechelen round A	30.12.2017	CSI-W5*	3	1.60	390	210	10	16.04.2008	G	SF	156	13	85	41	41	2052	191	M	9	8	no	spread	triple bar	left	no	0	0	64	no
Mechelen	The Longines FEI World Cup Jumping - Mechelen round A	30.12.2017	CSI-W5*	3	1.60	390	210	10	16.04.2008	G	SF	156	13	85	41	41	2052	191	M	10	9	no	vertical	vertical	left	no	0	0	64	no
Mechelen	The Longines FEI World Cup Jumping - Mechelen round A	30.12.2017	CSI-W5*	3	1.60	390	210	10	16.04.2008	G	SF	156	13	85	41	41	2052	191	M	11	10	no	spread	oxer	right	no	0	0	64	no
Mechelen	The Longines FEI World Cup Jumping - Mechelen round A	30.12.2017	CSI-W5*	3	1.60	390	210	10	16.04.2008	G	SF	156	13	85	41	41	2052	191	M	12	11a	yes	vertical	vertical	right	yes	4	0	64	no
Mechelen	The Longines FEI World Cup Jumping - Mechelen round A	30.12.2017	CSI-W5*	3	1.60	390	210	10	16.04.2008	G	SF	156	13	85	41	41	2052	191	M	13	11b	yes	spread	oxer	right	no	0	0	64	no
Mechelen	The Longines FEI World Cup Jumping - Mechelen round A	30.12.2017	CSI-W5*	3	1.60	390	210	10	16.04.2008	G	SF	156	13	85	41	41	2052	191	M	14	11c	yes	spread	oxer	right	no	0	0	64	no
Mechelen	The Longines FEI World Cup Jumping - Mechelen round A	30.12.2017	CSI-W5*	3	1.60	390	210	10	16.04.2008	G	SF	156	13	85	41	41	2052	191	M	15	12	no	vertical	vertical	left	no	0	0	64	no
Mechelen	The Longines FEI World Cup Jumping - Mechelen round A	30.12.2017	CSI-W5*	3	1.60	390	210	10	16.04.2008	G	SF	156	13	85	41	41	2052	191	M	16	13	no	spread	oxer	left	no	0	0	64	no
Mechelen	The Longines FEI World Cup Jumping - Mechelen round A	30.12.2017	CSI-W5*	3	1.60	390	102	11	28.07.2007	G	BWP	172	9	92	38	25	1918	72	M	1	1	no	spread	oxer	left	no	0	0	64,17	no
Mechelen	The Longines FEI World Cup Jumping - Mechelen round A	30.12.2017	CSI-W5*	3	1.60	390	102	11	28.07.2007	G	BWP	172	9	92	38	25	1918	72	M	2	2	no	vertical	vertical	straight	no	0	0	64,17	no
Mechelen	The Longines FEI World Cup Jumping - Mechelen round A	30.12.2017	CSI-W5*	3	1.60	390	102	11	28.07.2007	G	BWP	172	9	92	38	25	1918	72	M	3	3	no	spread	oxer	right	no	0	0	64,17	no
Mechelen	The Longines FEI World Cup Jumping - Mechelen round A	30.12.2017	CSI-W5*	3	1.60	390	102	11	28.07.2007	G	BWP	172	9	92	38	25	1918	72	M	4	4	no	vertical	vertical	right	no	0	0	64,17	no
Mechelen	The Longines FEI World Cup Jumping - Mechelen round A	30.12.2017	CSI-W5*	3	1.60	390	102	11	28.07.2007	G	BWP	172	9	92	38	25	1918	72	M	5	5a	yes	spread	oxer	left	no	0	0	64,17	no
Mechelen	The Longines FEI World Cup Jumping - Mechelen round A	30.12.2017	CSI-W5*	3	1.60	390	102	11	28.07.2007	G	BWP	172	9	92	38	25	1918	72	M	6	5b	yes	vertical	vertical	left	no	0	0	64,17	no
Mechelen	The Longines FEI World Cup Jumping - Mechelen round A	30.12.2017	CSI-W5*	3	1.60	390	102	11	28.07.2007	G	BWP	172	9	92	38	25	1918	72	M	7	6	no	spread	oxer	straight	no	0	0	64,17	no
Mechelen	The Longines FEI World Cup Jumping - Mechelen round A	30.12.2017	CSI-W5*	3	1.60	390	102	11	28.07.2007	G	BWP	172	9	92	38	25	1918	72	M	8	7	no	vertical	vertical with water	left	no	0	0	64,17	no
Mechelen	The Longines FEI World Cup Jumping - Mechelen round A	30.12.2017	CSI-W5*	3	1.60	390	102	11	28.07.2007	G	BWP	172	9	92	38	25	1918	72	M	9	8	no	spread	triple bar	left	no	0	0	64,17	no
Mechelen	The Longines FEI World Cup Jumping - Mechelen round A	30.12.2017	CSI-W5*	3	1.60	390	102	11	28.07.2007	G	BWP	172	9	92	38	25	1918	72	M	10	9	no	vertical	vertical	left	no	0	0	64,17	no
Mechelen	The Longines FEI World Cup Jumping - Mechelen round A	30.12.2017	CSI-W5*	3	1.60	390	102	11	28.07.2007	G	BWP	172	9	92	38	25	1918	72	M	11	10	no	spread	oxer	right	no	0	0	64,17	no
Mechelen	The Longines FEI World Cup Jumping - Mechelen round A	30.12.2017	CSI-W5*	3	1.60	390	102	11	28.07.2007	G	BWP	172	9	92	38	25	1918	72	M	12	11a	yes	vertical	vertical	right	no	0	0	64,17	no
Mechelen	The Longines FEI World Cup Jumping - Mechelen round A	30.12.2017	CSI-W5*	3	1.60	390	102	11	28.07.2007	G	BWP	172	9	92	38	25	1918	72	M	13	11b	yes	spread	oxer	right	no	0	0	64,17	no
Mechelen	The Longines FEI World Cup Jumping - Mechelen round A	30.12.2017	CSI-W5*	3	1.60	390	102	11	28.07.2007	G	BWP	172	9	92	38	25	1918	72	M	14	11c	yes	spread	oxer	right	no	0	0	64,17	no
Mechelen	The Longines FEI World Cup Jumping - Mechelen round A	30.12.2017	CSI-W5*	3	1.60	390	102	11	28.07.2007	G	BWP	172	9	92	38	25	1918	72	M	15	12	no	vertical	vertical	left	no	0	0	64,17	no
Mechelen	The Longines FEI World Cup Jumping - Mechelen round A	30.12.2017	CSI-W5*	3	1.60	390	102	11	28.07.2007	G	BWP	172	9	92	38	25	1918	72	M	16	13	no	spread	oxer	left	yes	4	0	64,17	no
Mechelen	The Longines FEI World Cup Jumping - Mechelen round A	30.12.2017	CSI-W5*	3	1.60	390	147	12	04.05.2006	G	OLDBG	239	5	93	106	55	1594	47	M	1	1	no	spread	oxer	left	no	0	0	66,49	no
Mechelen	The Longines FEI World Cup Jumping - Mechelen round A	30.12.2017	CSI-W5*	3	1.60	390	147	12	04.05.2006	G	OLDBG	239	5	93	106	55	1594	47	M	2	2	no	vertical	vertical	straight	no	0	0	66,49	no
Mechelen	The Longines FEI World Cup Jumping - Mechelen round A	30.12.2017	CSI-W5*	3	1.60	390	147	12	04.05.2006	G	OLDBG	239	5	93	106	55	1594	47	M	3	3	no	spread	oxer	right	no	0	0	66,49	no
Mechelen	The Longines FEI World Cup Jumping - Mechelen round A	30.12.2017	CSI-W5*	3	1.60	390	147	12	04.05.2006	G	OLDBG	239	5	93	106	55	1594	47	M	4	4	no	vertical	vertical	right	no	0	0	66,49	no
Mechelen	The Longines FEI World Cup Jumping - Mechelen round A	30.12.2017	CSI-W5*	3	1.60	390	147	12	04.05.2006	G	OLDBG	239	5	93	106	55	1594	47	M	5	5a	yes	spread	oxer	left	no	0	0	66,49	no
Mechelen	The Longines FEI World Cup Jumping - Mechelen round A	30.12.2017	CSI-W5*	3	1.60	390	147	12	04.05.2006	G	OLDBG	239	5	93	106	55	1594	47	M	6	5b	yes	vertical	vertical	left	no	0	0	66,49	no

Mechelen	The Longines FEI World Cup Jumping - Mechelen round A	30.12.2017	CSI-W5*	3	1.60	390	147	12	04.05.2006	G	OLDBG	239	5	93	106	55	1594	47	M	7	6	no	spread	oxer	straight	no	0	0	66,49	no
Mechelen	The Longines FEI World Cup Jumping - Mechelen round A	30.12.2017	CSI-W5*	3	1.60	390	147	12	04.05.2006	G	OLDBG	239	5	93	106	55	1594	47	M	8	7	no	vertical	vertical with water	left	no	0	0	66,49	no
Mechelen	The Longines FEI World Cup Jumping - Mechelen round A	30.12.2017	CSI-W5*	3	1.60	390	147	12	04.05.2006	G	OLDBG	239	5	93	106	55	1594	47	M	9	8	no	spread	triple bar	left	no	0	0	66,49	no
Mechelen	The Longines FEI World Cup Jumping - Mechelen round A	30.12.2017	CSI-W5*	3	1.60	390	147	12	04.05.2006	G	OLDBG	239	5	93	106	55	1594	47	M	10	9	no	vertical	vertical	left	no	0	0	66,49	no
Mechelen	The Longines FEI World Cup Jumping - Mechelen round A	30.12.2017	CSI-W5*	3	1.60	390	147	12	04.05.2006	G	OLDBG	239	5	93	106	55	1594	47	M	11	10	no	spread	oxer	right	yes	4	0	66,49	no
Mechelen	The Longines FEI World Cup Jumping - Mechelen round A	30.12.2017	CSI-W5*	3	1.60	390	147	12	04.05.2006	G	OLDBG	239	5	93	106	55	1594	47	M	12	11a	yes	vertical	vertical	right	no	0	0	66,49	no
Mechelen	The Longines FEI World Cup Jumping - Mechelen round A	30.12.2017	CSI-W5*	3	1.60	390	147	12	04.05.2006	G	OLDBG	239	5	93	106	55	1594	47	M	13	11b	yes	spread	oxer	right	no	0	0	66,49	no
Mechelen	The Longines FEI World Cup Jumping - Mechelen round A	30.12.2017	CSI-W5*	3	1.60	390	147	12	04.05.2006	G	OLDBG	239	5	93	106	55	1594	47	M	14	11c	yes	spread	oxer	right	no	0	0	66,49	no
Mechelen	The Longines FEI World Cup Jumping - Mechelen round A	30.12.2017	CSI-W5*	3	1.60	390	147	12	04.05.2006	G	OLDBG	239	5	93	106	55	1594	47	M	15	12	no	vertical	vertical	left	no	0	0	66,49	no
Mechelen	The Longines FEI World Cup Jumping - Mechelen round A	30.12.2017	CSI-W5*	3	1.60	390	147	12	04.05.2006	G	OLDBG	239	5	93	106	55	1594	47	M	16	13	no	spread	oxer	left	no	0	0	66,49	no
Mechelen	The Longines FEI World Cup Jumping - Mechelen round A	30.12.2017	CSI-W5*	3	1.60	390	118	12	26.01.2006	G	OS	262	11	96	97	55	1508	53	M	1	1	no	spread	oxer	left	no	0	0	0,00	yes
Mechelen	The Longines FEI World Cup Jumping - Mechelen round A	30.12.2017	CSI-W5*	3	1.60	390	118	12	26.01.2006	G	OS	262	11	96	97	55	1508	53	M	2	2	no	vertical	vertical	straight	no	0	0	0,00	yes
Mechelen	The Longines FEI World Cup Jumping - Mechelen round A	30.12.2017	CSI-W5*	3	1.60	390	118	12	26.01.2006	G	OS	262	11	96	97	55	1508	53	M	3	3	no	spread	oxer	right	no	0	0	0,00	yes
Mechelen	The Longines FEI World Cup Jumping - Mechelen round A	30.12.2017	CSI-W5*	3	1.60	390	118	12	26.01.2006	G	OS	262	11	96	97	55	1508	53	M	4	4	no	vertical	vertical	right	no	0	0	0,00	yes
Mechelen	The Longines FEI World Cup Jumping - Mechelen round A	30.12.2017	CSI-W5*	3	1.60	390	118	12	26.01.2006	G	OS	262	11	96	97	55	1508	53	M	5	5a	yes	spread	oxer	left	no	0	0	0,00	yes
Mechelen	The Longines FEI World Cup Jumping - Mechelen round A	30.12.2017	CSI-W5*	3	1.60	390	118	12	26.01.2006	G	OS	262	11	96	97	55	1508	53	M	6	5b	yes	vertical	vertical	left	no	0	0	0,00	yes
Mechelen	The Longines FEI World Cup Jumping - Mechelen round A	30.12.2017	CSI-W5*	3	1.60	390	118	12	26.01.2006	G	OS	262	11	96	97	55	1508	53	M	7	6	no	spread	oxer	straight	no	0	0	0,00	yes
Mechelen	The Longines FEI World Cup Jumping - Mechelen round A	30.12.2017	CSI-W5*	3	1.60	390	118	12	26.01.2006	G	OS	262	11	96	97	55	1508	53	M	8	7	no	vertical	vertical with water	left	no	0	0	0,00	yes
Mechelen	The Longines FEI World Cup Jumping - Mechelen round A	30.12.2017	CSI-W5*	3	1.60	390	118	12	26.01.2006	G	OS	262	11	96	97	55	1508	53	M	9	8	no	spread	triple bar	left	no	0	0	0,00	yes
Mechelen	The Longines FEI World Cup Jumping - Mechelen round A	30.12.2017	CSI-W5*	3	1.60	390	118	12	26.01.2006	G	OS	262	11	96	97	55	1508	53	M	10	9	no	vertical	vertical	left	no	0	0	0,00	yes
Mechelen	The Longines FEI World Cup Jumping - Mechelen round A	30.12.2017	CSI-W5*	3	1.60	390	118	12	26.01.2006	G	OS	262	11	96	97	55	1508	53	M	11	10	no	spread	oxer	right	no	0	0	0,00	yes
Mechelen	The Longines FEI World Cup Jumping - Mechelen round A	30.12.2017	CSI-W5*	3	1.60	390	118	12	26.01.2006	G	OS	262	11	96	97	55	1508	53	M	12	11a	yes	vertical	vertical	right	no	0	0	0,00	yes
Mechelen	The Longines FEI World Cup Jumping - Mechelen round A	30.12.2017	CSI-W5*	3	1.60	390	118	12	26.01.2006	G	OS	262	11	96	97	55	1508	53	M	13	11b	yes	spread	oxer	right	yes	4	0	0,00	yes
Mechelen	The Longines FEI World Cup Jumping - Mechelen round A	30.12.2017	CSI-W5*	3	1.60	390	118	12	26.01.2006	G	OS	262	11	96	97	55	1508	53	M	14	11c	yes	spread	oxer	right	no	0	0	0,00	yes
Mechelen	The Longines FEI World Cup Jumping - Mechelen round A	30.12.2017	CSI-W5*	3	1.60	390	118	12	26.01.2006	G	OS	262	11	96	97	55	1508	53	M	15	12	no	vertical	vertical	left	no	0	0	0,00	yes
Mechelen	The Longines FEI World Cup Jumping - Mechelen round A	30.12.2017	CSI-W5*	3	1.60	390	118	12	26.01.2006	G	OS	262	11	96	97	55	1508	53	M	16	13	no	spread	oxer	left	yes	4	0	0,00	yes
Mechelen	The Longines FEI World Cup Jumping - Mechelen round A	30.12.2017	CSI-W5*	3	1.60	390	144	14	28.02.2004	G	DSP	186	2	103	171	32	1274	37	M	1	1	no	spread	oxer	left	no	0	0	67,88	no
Mechelen	The Longines FEI World Cup Jumping - Mechelen round A	30.12.2017	CSI-W5*	3	1.60	390	144	14	28.02.2004	G	DSP	186	2	103	171	32	1274	37	M	2	2	no	vertical	vertical	straight	no	0	0	67,88	no
Mechelen	The Longines FEI World Cup Jumping - Mechelen round A	30.12.2017	CSI-W5*	3	1.60	390	144	14	28.02.2004	G	DSP	186	2	103	171	32	1274	37	M	3	3	no	spread	oxer	right	no	0	0	67,88	no
Mechelen	The Longines FEI World Cup Jumping - Mechelen round A	30.12.2017	CSI-W5*	3	1.60	390	144	14	28.02.2004	G	DSP	186	2	103	171	32	1274	37	M	4	4	no	vertical	vertical	right	no	0	0	67,88	no
Mechelen	The Longines FEI World Cup Jumping - Mechelen round A	30.12.2017	CSI-W5*	3	1.60	390	144	14	28.02.2004	G	DSP	186	2	103	171	32	1274	37	M	5	5a	yes	spread	oxer	left	no	0	0	67,88	no
Mechelen	The Longines FEI World Cup Jumping - Mechelen round A	30.12.2017	CSI-W5*	3	1.60	390	144	14	28.02.2004	G	DSP	186	2	103	171	32	1274	37	M	6	5b	yes	vertical	vertical	left	no	0	0	67,88	no
Mechelen	The Longines FEI World Cup Jumping - Mechelen round A	30.12.2017	CSI-W5*	3	1.60	390	144	14	28.02.2004	G	DSP	186	2	103	171	32	1274	37	M	7	6	no	spread	oxer	straight	no	0	0	67,88	no
Mechelen	The Longines FEI World Cup Jumping - Mechelen round A	30.12.2017	CSI-W5*	3	1.60	390	144	14	28.02.2004	G	DSP	186	2	103	171	32	1274	37	M	8	7	no	vertical	vertical with water	left	no	0	0	67,88	no
Mechelen	The Longines FEI World Cup Jumping - Mechelen round A	30.12.2017	CSI-W5*	3	1.60	390	144	14	28.02.2004	G	DSP	186	2	103	171	32	1274	37	M	9	8	no	spread	triple bar	left	no	0	0	67,88	no
Mechelen	The Longines FEI World Cup Jumping - Mechelen round A	30.12.2017	CSI-W5*	3	1.60	390	144	14	28.02.2004	G	DSP	186	2	103	171	32	1274	37	M	10	9	no	vertical	vertical	left	no	0	0	67,88	no
Mechelen	The Longines FEI World Cup Jumping - Mechelen round A	30.12.2017	CSI-W5*	3	1.60	390	144	14	28.02.2004	G	DSP	186	2	103	171	32	1274	37	M	11	10	no	spread	oxer	right	no	0	0	67,88	no
Mechelen	The Longines FEI World Cup Jumping - Mechelen round A	30.12.2017	CSI-W5*	3	1.60	390	144	14	28.02.2004	G	DSP	186	2	103	171	32	1274	37	M	12	11a	yes	vertical	vertical	right	no	0	0	67,88	no
Mechelen	The Longines FEI World Cup Jumping - Mechelen round A	30.12.2017	CSI-W5*	3	1.60	390	144	14	28.02.2004	G	DSP	186	2	103	171	32	1274	37	M	13	11b	yes	spread	oxer	right	no	0	0	67,88	no
Mechelen	The Longines FEI World Cup Jumping - Mechelen round A	30.12.2017	CSI-W5*	3	1.60	390	144	14	28.02.2004	G	DSP	186	2	103	171	32	1274	37	M	14	11c	yes	spread	oxer	right	no	0	0	67,88	no
Mechelen	The Longines FEI World Cup Jumping - Mechelen round A	30.12.2017	CSI-W5*	3	1.60	390	144	14	28.02.2004	G	DSP	186	2	103	171	32	1274	37	M	15	12	no	vertical	vertical	left	no	0	0	67,88	no
Mechelen	The Longines FEI World Cup Jumping - Mechelen round A	30.12.2017	CSI-W5*	3	1.60	390	144	14	28.02.2004	G	DSP	186	2	103	171	32	1274	37	M	16	13	no	spread	oxer	left	no	0	1	67,88	no
Mechelen	The Longines FEI World Cup Jumping - Mechelen round A	30.12.2017	CSI-W5*	3	1.60	390	217	15	17.04.2003	M	KWPN	227	2	105	112	37	1565	24	M	1	1	no	spread	oxer	left	no	0	0	66,1	no
Mechelen	The Longines FEI World Cup Jumping - Mechelen round A	30.12.2017	CSI-W5*	3	1.60	390	217	15	17.04.2003	M	KWPN	227	2	105	112	37	1565	24	M	2	2	no	vertical	vertical	straight	no	0	0	66,1	no
Mechelen	The Longines FEI World Cup Jumping - Mechelen round A	30.12.2017	CSI-W5*	3	1.60	390	217	15	17.04.2003	M	KWPN	227	2	105	112	37	1565	24	M	3	3	no	spread	oxer	right	no	0	0	66,1	no
Mechelen	The Longines FEI World Cup Jumping - Mechelen round A	30.12.2017	CSI-W5*	3	1.60	390	217	15	17.04.2003	M	KWPN	227	2	105	112	37	1565	24	M	4	4	no	vertical	vertical	right	no	0	0	66,1	no
Mechelen	The Longines FEI World Cup Jumping - Mechelen round A	30.12.2017	CSI-W5*	3	1.60	390	217	15	17.04.2003	M	KWPN	227	2	105	112	37	1565	24	M	5	5a	yes	spread	oxer	left	yes	4	0	66,1	no
Mechelen	The Longines FEI World Cup Jumping - Mechelen round A	30.12.2017	CSI-W5*	3	1.60	390	217	15	17.04.2003	M	KWPN	227	2	105	112	37	1565	24	M	6	5b	yes	vertical	vertical	left	no	0	0	66,1	no
Mechelen	The Longines FEI World Cup Jumping - Mechelen round A	30.12.2017	CSI-W5*	3	1.60	390	217	15	17.04.2003	M	KWPN	227	2	105	112	37	1565	24	M	7	6	no	spread	oxer	straight	no	0	0	66,1	no
Mechelen	The Longines FEI World Cup Jumping - Mechelen round A	30.12.2017	CSI-W5*	3	1.60	390	217	15	17.04.2003	M	KWPN	227	2	105	112	37	1565	24	M	8	7	no	vertical	vertical with water	left	no	0	0	66,1	no
Mechelen	The Longines FEI World Cup Jumping - Mechelen round A	30.12.2017	CSI-W5*	3	1.60	390	217	15	17.04.2003	M	KWPN	227	2	105	112	37	1565	24	M	9	8	no	spread	triple bar	left	no	0	0	66,1	no
Mechelen	The Longines FEI World Cup Jumping - Mechelen round A	30.12.2017	CSI-W5*	3	1.60	390	217	15	17.04.2003	M	KWPN	227	2	105	112	37	1565	24	M	10	9	no	vertical	vertical	left	no	0	0	66,1	no
Mechelen	The Longines FEI World Cup Jumping - Mechelen round A	30.12.2017	CSI-W5*	3	1.60	390	217	15	17.04.2003	M	KWPN	227	2	105	112	37	1565	24	M	11	10	no	spread	oxer	right	no	0	0	66,1	no
Mechelen	The Longines FEI World Cup Jumping - Mechelen round A	30.12.2017	CSI-W5*	3	1.60	390	217	15	17.04.2003	M	KWPN	227	2	105	112	37	1565	24	M	12	11a	yes	vertical	vertical	right	no	0	0	66,1	no
Mechelen	The Longines FEI World Cup Jumping - Mechelen round A	30.12.2017	CSI-W5*	3	1.60	390	217	15	17.04.2003	M	KWPN	227	2	105	112	37	1565	24	M	13	11b	yes	spread	oxer	right	no	0	0	66,1	no
Mechelen	The Longines FEI World Cup Jumping - Mechelen round A	30.12.2017	CSI-W5*	3	1.60	390	217	15	17.04.2003	M	KWPN	227	2	105	112	37	1565	24	M	14	11c	yes	spread	oxer	right	no	0	0	66,1	no
Mechelen	The Longines FEI World Cup Jumping - Mechelen round A	30.12.2017	CSI-W5*	3	1.60	390	217	15	17.04.2003	M	KWPN	227	2	105	112	37	1565	24	M	15	12	no	vertical	vertical	left	no	0	0	66,1	no
Mechelen	The Longines FEI World Cup Jumping - Mechelen round A	30.12.2017	CSI-W5*	3	1.60	390	217	15	17.04.2003	M	KWPN	227	2	105	112	37	1565	24	M	16	13	no	spread	oxer	left	no	0	0	66,1	no
Mechelen	The Longines FEI World Cup Jumping - Mechelen round A	30.12.2017	CSI-W5*	3	1.60	390	69	9	21.06.2009	S	SBS	123	5	115	62	39	1976	81	M	1	1	no	spread	oxer	left	no	0	0	69,93	no
Mechelen	The Longines FEI World Cup Jumping - Mechelen round A	30.12.2017	CSI-W5*	3	1.60	390	69	9	21.06.2009	S	SBS	123	5	115	62	39	1976	81	M	2	2	no	vertical	vertical	straight	no	0	0	69,93	no
Mechelen	The Longines FEI World Cup Jumping - Mechelen round A	30.12.2017	CSI-W5*	3	1.60	390	69	9	21.06.2009	S	SBS	123	5	115	62	39	1976	81	M	3	3	no	spread	oxer	right	no	0	0	69,93	no
Mechelen	The Longines FEI World Cup Jumping - Mechelen round A	30.12.2017	CSI-W5*	3	1.60	390	69	9	21.06.2009	S	SBS	123	5	115	62	39	1976	81	M	4	4	no	vertical	vertical	right	yes	4	0	69,93	no
Mechelen	The Longines FEI World Cup Jumping - Mechelen round A	30.12.2017	CSI-W5*	3	1.60	390	69	9	21.06.2009	S	SBS	123	5	115	62	39	1976	81	M	5	5a	yes	spread	oxer	left	no	0	0	69,93	no
Mechelen	The Longines FEI World Cup Jumping - Mechelen round A	30.12.2017	CSI-W5*	3	1.60	390	69	9	21.06.2009	S	SBS	123	5	115	62	39	1976	81	M	6	5b	yes	vertical	vertical	left	no	0	0	69,93	no
Mechelen	The Longines FEI World Cup Jumping - Mechelen round A	30.12.2017	CSI-W5*	3	1.60	390	69	9	21.06.2009	S	SBS	123	5	115	62	39	1976	81	M	7	6	no	spread	oxer	straight	no	0	0	69,93	no
Mechelen	The Longines FEI World Cup Jumping - Mechelen round A	30.12.2017	CSI-W5*	3	1.60	390	69	9	21.06.2009	S	SBS	123	5	115	62	39	1976	81	M	8	7	no	vertical	vertical with water	left	no	0	0	69,93	no
Mechelen	The Longines FEI World Cup Jumping - Mechelen round A	30.12.2017	CSI-W5*	3	1.60	390	69	9	21.06.2009	S	SBS	123	5	115	62	39	1976	81	M	9	8	no	spread	triple bar	left	no	0	0	69,93	no
Mechelen	The Longines FEI World Cup Jumping - Mechelen round A	30.12.2017	CSI-W5*	3	1.60	390	69	9	21.06.2009	S	SBS	123	5	115	62	39	1976	81	M	10	9	no	vertical	vertical	left	no	0	0	69,93	no
Mechelen	The Longines FEI World Cup Jumping - Mechelen round A	30.12.2017	CSI-W5*	3	1.60	390	69	9	21.06.2009	S	SBS	123	5	115	62	39	1976	81	M	11	10	no	spread	oxer	right	no	0	0	69,93	no
Mechelen	The Longines FEI World Cup Jumping - Mechelen round A	30.12.2017	CSI-W5*	3	1.60	390	69	9	21.06.2009	S	SBS	123	5	115	62	39	1976	81	M	12	11a	yes	vertical	vertical	right	no	0	0	69,93	no
Mechelen	The Longines FEI World Cup Jumping - Mechelen round A	30.12.2017	CSI-W5*	3	1.60	390	69	9	21.06.2009	S	SBS	123	5	115	62	39	1976	81	M	13	11b	yes	spread	oxer	right	no	0	0	69,93	no
Mechelen	The Longines FEI World Cup Jumping - Mechelen round A	30.12.2017	CSI-W5*	3	1.60	390	69	9	21.06.2009	S	SBS	123	5	115	62	39	1976	81	M	14	11c	yes	spread	oxer	right	yes	4	0	69,93	no
Mechelen	The Longines FEI World Cup Jumping - Mechelen round A	30.12.2017	CSI-W5*	3	1.60	390	69	9	21.06.2009	S	SBS	123	5	115	62	39	1976	81	M	15	12	no	vertical	vertical	left	no	0	0	69,93	no
Mechelen	The Longines FEI World Cup Jumping - Mechelen round A	30.12.2017	CSI-W5*	3	1.60	390	69	9	21.06.2009	S	SBS	123	5	115	62	39	1976	81	M	16	13	no	spread	oxer	left	no	0	1	69,93	no
Mechelen	The Longines FEI World Cup Jumping - Mechelen round A	30.12.2017	CSI-W5*	3	1.60	390	148	10	28.06.2008	G	Bezplemenné	125	0	120	550	27	986	18	F	1	1	no	spread	oxer	left	no	0	0	68,83	no
Mechelen	The Longines FEI World Cup Jumping - Mechelen round A	30.12.2017	CSI-W5*	3	1.60	390	148	10	28.06.2008	G	Bezplemenné	125	0	120	550	27	986	18	F	2	2	no	vertical	vertical	straight	no	0	0	68,83	no
Mechelen	The Longines FEI World Cup Jumping - Mechelen round A	30.12.2017	CSI-W5*	3	1.60	390	148	10	28.06.2008	G	Bezplemenné	125	0	120	550	27	986	18	F	3	3	no	spread	oxer	right	yes	4	0	68,83	no
Mechelen	The Longines FEI World Cup Jumping - Mechelen round A	30.12.2017	CSI-W5*	3	1.60	390	148	10	28.06.2008	G	Bezplemenné	125	0	120	550	27	986	18	F	4	4	no	vertical	vertical	right	no	0	0	68,83	no
Mechelen	The Longines FEI World Cup Jumping - Mechelen round A	30.12.2017	CSI-W5*	3	1.60	390	148	10	28.06.2008	G	Bezplemenné	125	0	120	550	27	986	18	F	5	5a	yes	spread	oxer	left	no	0	0	68,83	no
Mechelen	The Longines FEI World Cup Jumping - Mechelen round A	30.12.2017	CSI-W5*	3	1.60	390	148	10	28.06.2008	G	Bezplemenné	125	0	120	550	27	986	18	F	6	5b	yes	vertical	vertical	left	no	0	0	68,83	no
Mechelen	The Longines FEI World Cup Jumping - Mechelen round A	30.12.2017	CSI-W5*	3	1.60	390	148	10	28.06.2008	G	Bezplemenné	125	0	120	550	27	986	18	F	7	6	no	spread	oxer	straight	no	0	0	68,83	no
Mechelen	The Longines FEI World Cup Jumping - Mechelen round A	30.12.2017	CSI-W5*	3	1.60	390	148	10	28.06.2008	G	Bezplemenné	125	0	120	550	27	986	18	F	8	7	no	vertical	vertical with water	left	no	0	0	68,83	no
Mechelen	The Longines FEI World Cup Jumping - Mechelen round A	30.12.2017	CSI-W5*	3	1.60	390	148	10	28.06.2008	G	Bezplemenné	125	0	120	550	27	986	18	F	9	8	no	spread	triple bar	left	no	0	0	68,83	no
Mechelen	The Longines FEI World Cup Jumping - Mechelen round A	30.12.2017	CSI-W5*	3	1.60	390	148	10	28.06.2008	G	Bezplemenné	125	0	120	550	27	986	18	F	10	9	no	vertical	vertical	left	no	0	0	68,83	no
Mechelen	The Longines FEI World Cup Jumping - Mechelen round A	30.12.2017	CSI-W5*	3	1.60	390	148	10	28.06.2008	G	Bezplemenné	125	0	120	550	27	986	18	F	11	10	no	spread	oxer	right	no	0	0	68,83	no
Mechelen	The Longines FEI World Cup Jumping - Mechelen round A	30.12.2017	CSI-W5*	3	1.60	390	148	10	28.06.2008	G	Bezplemenné	125	0	120	550	27	986	18	F	12	11a	yes	vertical	vertical	right	no	0	0	68,83	no
Mechelen	The Longines FEI World Cup Jumping - Mechelen round A	30.12.2017	CSI-W5*	3	1.60	390	148	10	28.06.2008	G	Bezplemenné	125	0	120	550	27	986	18	F	13	11b	yes	spread	oxer	right	no	0	0	68,83	no
Mechelen	The Longines FEI World Cup Jumping - Mechelen round A	30.12.2017	CSI-W5*	3	1.60	390	148	10	28.06.2008	G	Bezplemenné	125	0	120	550	27	986	18	F	14	11c	yes	spread	oxer	right	no	0	0	68,83	no
Mechelen	The Longines FEI World Cup Jumping - Mechelen round A	30.12.2017	CSI-W5*	3	1.60	390	148	10	28.06.2008	G	Bezplemenné	125	0	120	550	27	986	18	F	15	12	no	vertical	vertical	left	yes	4	0	68,83	no
Mechelen	The Longines FEI World Cup Jumping - Mechelen round A	30.12.2017	CSI-W5*	3	1.60	390	148	10	28.06.2008	G	Bezplemenné	125	0	120	550	27	986	18	F	16	13	no	spread	oxer	left	no	0	1	68,83	no
Mechelen	The Longines FEI World Cup Jumping - Mechelen round A	30.12.2017	CSI-W5*	3	1.60	390	221	14	30.04.2004	G	KWPN	281	20	121	2	38	1958	103	M	1	1	no	spread	oxer	left	no	0	0	66,02	no
Mechelen	The Longines FEI World Cup Jumping - Mechelen round A	30.12.2017	CSI-W5*	3	1.60	390	221	14	30.04.2004	G	KWPN	281	20	121	2	38	1958	103	M	2	2	no	vertical	vertical	straight	no	0	0	66,02	no
Mechelen	The Longines FEI World Cup Jumping - Mechelen round A	30.12.2017	CSI-W5*	3	1.60	390	221	14	30.04.2004	G	KWPN	281	20	121	2	38	1958	103	M	3	3	no	spread	oxer	right	no	0	0	66,02	no
Mechelen	The Longines FEI World Cup Jumping - Mechelen round A	30.12.2017	CSI-W5*	3	1.60	390	221	14	30.04.2004	G	KWPN	281	20	121	2	38	1958	103	M	4	4	no	vertical	vertical	right	no	0	0	66,02	no
Mechelen	The Longines FEI World Cup Jumping - Mechelen round A	30.12.2017	CSI-W5*	3	1.60	390	221	14	30.04.2004	G	KWPN	281	20	121	2	38	1958	103	M	5	5a	yes	spread	oxer	left	no	0	0	66,02	no
Mechelen	The Longines FEI World Cup Jumping - Mechelen round A	30.12.2017	CSI-W5*	3	1.60	390	221	14	30.04.2004	G	KWPN	281	20	121	2	38	1958	103	M	6	5b	yes	vertical	vertical	left	no	0	0	66,02	no
Mechelen	The Longines FEI World Cup Jumping - Mechelen round A	30.12.2017	CSI-W5*	3	1.60	390	221	14	30.04.2004	G	KWPN	281	20	121	2	38	1958	103	M	7	6	no	spread	oxer	straight	no	0	0	66,02	no
Mechelen	The Longines FEI World Cup Jumping - Mechelen round A	30.12.2017	CSI-W5*	3	1.60	390	221	14	30.04.2004	G	KWPN	281	20	121	2	38	1958	103	M	8	7	no	vertical	vertical with water	left	no	0	0	66,02	no
Mechelen	The Longines FEI World Cup Jumping - Mechelen round A	30.12.2017	CSI-W5*	3	1.60	390	221	14	30.04.2004	G	KWPN	281	20	121	2	38	1958	103	M	9	8	no	spread	triple bar	left	no	0	0	66,02	no
Mechelen	The Longines FEI World Cup Jumping - Mechelen round A	30.12.2017	CSI-W5*	3	1.60	390	221	14	30.04.2004	G	KWPN	281	20	121	2	38	1958	103	M	10	9	no	vertical	vertical	left	no	0	0	66,02	no
Mechelen	The Longines FEI World Cup Jumping - Mechelen round A	30.12.2017	CSI-W5*	3	1.60	390	221	14	30.04.2004	G	KWPN	281	20	121	2	38	1958	103	M	11	10	no	spread	oxer	right	no	0	0	66,02	no
Mechelen	The Longines FEI World Cup Jumping - Mechelen round A	30.12.2017	CSI-W5*	3	1.60	390	221	14	30.04.2004	G	KWPN	281	20	121	2	38	1958	103	M	12	11a	yes	vertical	vertical	right	no	0	0	66,02	no
Mechelen	The Longines FEI World Cup Jumping - Mechelen round A	30.12.2017	CSI-W5*	3	1.60	390	221	14	30.04.2004	G	KWPN	281	20	121	2	38	1958	103	M	13	11b	yes	spread	oxer	right	no	0	0	66,02	no
Mechelen	The Longines FEI World Cup Jumping - Mechelen round A	30.12.2017	CSI-W5*	3	1.60	390	221	14	30.04.2004	G	KWPN	281	20	121	2	38	1958	103	M	14	11c	yes	spread	oxer	right	no	0	0	66,02	no
Mechelen	The Longines FEI World Cup Jumping - Mechelen round A	30.12.2017	CSI-W5*	3	1.60	390	221	14	30.04.2004	G	KWPN	281	20	121	2	38	1958	103	M	15	12	no	vertical	vertical	left	no	0	0	66,02	no
Mechelen	The Longines FEI World Cup Jumping - Mechelen round A	30.12.2017	CSI-W5*	3	1.60	390	221	14	30.04.2004	G	KWPN	281	20	121	2	38	1958	103	M	16	13	no	spread	oxer	left	no	0	0	66,02	no
Mechelen	The Longines FEI World Cup Jumping - Mechelen round B	30.12.2017	CSI-W5*	3	1.60	290	221	14	30.04.2004	G	KWPN	281	20	121	2	38	1958	103	M	1	1	no	spread	oxer	right	no	0	0	38,19	no
Mechelen	The Longines FEI World Cup Jumping - Mechelen round B	30.12.2017	CSI-W5*	3	1.60	290	221	14	30.04.2004	G	KWPN	281	20	121	2	38	1958	103	M	2	2	no	vertical	vertical	right	no	0	0	38,19	no
Mechelen	The Longines FEI World Cup Jumping - Mechelen round B	30.12.2017	CSI-W5*	3	1.60	290	221	14	30.04.2004	G	KWPN	281	20	121	2	38	1958	103	M	3	3	no	spread	oxer	left	no	0	0	38,19	no
Mechelen	The Longines FEI World Cup Jumping - Mechelen round B	30.12.2017	CSI-W5*	3	1.60	290	221	14	30.04.2004	G	KWPN	281	20	121	2	38	1958	103	M	4	5	no	vertical	vertical	left	no	0	0	38,19	no
Mechelen	The Longines FEI World Cup Jumping - Mechelen round B	30.12.2017	CSI-W5*	3	1.60	290	221	14	30.04.2004	G	KWPN	281	20	121	2	38	1958	103	M	5	6a	yes	vertical	vertical	right	no	0	0	38,19	no
Mechelen	The Longines FEI World Cup Jumping - Mechelen round B	30.12.2017	CSI-W5*	3	1.60	290	221	14	30.04.2004	G	KWPN	281	20	121	2	38	1958	103	M	6	6b	yes	spread	oxer	right	no	0	0	38,19	no
Mechelen	The Longines FEI World Cup Jumping - Mechelen round B	30.12.2017	CSI-W5*	3	1.60	290	221	14	30.04.2004	G	KWPN	281	20	121	2	38	1958	103	M	7	7	no	vertical	vertical	left	no	0	0	38,19	no
Mechelen	The Longines FEI World Cup Jumping - Mechelen round B	30.12.2017	CSI-W5*	3	1.60	290	221	14	30.04.2004	G	KWPN	281	20	121	2	38	1958	103	M	8	8	no	spread	triple bar	left	no	0	0	38,19	no
Mechelen	The Longines FEI World Cup Jumping - Mechelen round B	30.12.2017	CSI-W5*	3	1.60	290	221	14	30.04.2004	G	KWPN	281	20	121	2	38	1958	103	M	9	9	no	vertical	vertical	left	no	0	0	38,19	no
Mechelen	The Longines FEI World Cup Jumping - Mechelen round A	30.12.2017	CSI-W5*	3	1.60	390	86	15	01.04.2003	G	SWB	263	9	122	156	29	1538	43	M	1	1	no	spread	oxer	left	no	0	0	0,00	yes
Mechelen	The Longines FEI World Cup Jumping - Mechelen round A	30.12.2017	CSI-W5*	3	1.60	390	86	15	01.04.2003	G	SWB	263	9	122	156	29	1538	43	M	2	2	no	vertical	vertical	straight	yes	4	0	0,00	yes
Mechelen	The Longines FEI World Cup Jumping - Mechelen round A	30.12.2017	CSI-W5*	3	1.60	390	86	15	01.04.2003	G	SWB	263	9	122	156	29	1538	43	M	3	3	no	spread	oxer	right	no	0	0	0,00	yes
Mechelen	The Longines FEI World Cup Jumping - Mechelen round A	30.12.2017	CSI-W5*	3	1.60	390	86	15	01.04.2003	G	SWB	263	9	122	156	29	1538	43	M	4	4	no	vertical	vertical	right	no	0	0	0,00	yes
Mechelen	The Longines FEI World Cup Jumping - Mechelen round A	30.12.2017	CSI-W5*	3	1.60	390	86	15	01.04.2003	G	SWB	263	9	122	156	29	1538	43	M	5	5a	yes	spread	oxer	left	no	0	0	0,00	yes
Mechelen	The Longines FEI World Cup Jumping - Mechelen round A	30.12.2017	CSI-W5*	3	1.60	390	86	15	01.04.2003	G	SWB	263	9	122	156	29	1538	43	M	6	5b	yes	vertical	vertical	left	no	0	0	0,00	yes
Mechelen	The Longines FEI World Cup Jumping - Mechelen round A	30.12.2017	CSI-W5*	3	1.60	390	86	15	01.04.2003	G	SWB	263	9	122	156	29	1538	43	M	7	6	no	spread	oxer	straight	no	0	0	0,00	yes
Mechelen	The Longines FEI World Cup Jumping - Mechelen round A	30.12.2017	CSI-W5*	3	1.60	390	86	15	01.04.2003	G	SWB	263	9	122	156	29	1538	43	M	8	7	no	vertical	vertical with water	left	no	0	0	0,00	yes
Mechelen	The Longines FEI World Cup Jumping - Mechelen round A	30.12.2017	CSI-W5*	3	1.60	390	86	15	01.04.2003	G	SWB	263	9	122	156	29	1538	43	M	9	8	no	spread	triple bar	left	no	0	0	0,00	yes
Mechelen	The Longines FEI World Cup Jumping - Mechelen round A	30.12.2017	CSI-W5*	3	1.60	390	86	15	01.04.2003	G	SWB	263	9	122	156	29	1538	43	M	10	9	no	vertical	vertical	left	no	0	0	0,00	yes
Mechelen	The Longines FEI World Cup Jumping - Mechelen round A	30.12.2017	CSI-W5*	3	1.60	390	86	15	01.04.2003	G	SWB	263	9	122	156	29	1538	43	M	11	10	no	spread	oxer	right	no	0	0	0,00	yes
Mechelen	The Longines FEI World Cup Jumping - Mechelen round A	30.12.2017	CSI-W5*	3	1.60	390	86	15	01.04.2003	G	SWB	263	9	122	156	29	1538	43	M	12	11a	yes	vertical	vertical	right	no	0	0	0,00	yes
Mechelen	The Longines FEI World Cup Jumping - Mechelen round A	30.12.2017	CSI-W5*	3	1.60	390	86	15	01.04.2003	G	SWB	263	9	122	156	29	1538	43	M	13	11b	yes	spread	oxer	right	no	0	0	0,00	yes
Mechelen	The Longines FEI World Cup Jumping - Mechelen round A	30.12.2017	CSI-W5*	3	1.60	390	86	15	01.04.2003	G	SWB	263	9	122	156	29	1538	43	M	14	11c	yes	spread	oxer	right	no	0	0	0,00	yes
Mechelen	The Longines FEI World Cup Jumping - Mechelen round A	30.12.2017	CSI-W5*	3	1.60	390	86	15	01.04.2003	G	SWB	263	9	122	156	29	1538	43	M	15	12	no	vertical	vertical	left	no	0	0	0,00	yes
Mechelen	The Longines FEI World Cup Jumping - Mechelen round A	30.12.2017	CSI-W5*	3	1.60	390	86	15	01.04.2003	G	SWB	263	9	122	156	29	1538	43	M	16	13	no	spread	oxer	left	no	0	0	0,00	yes
Mechelen	The Longines FEI World Cup Jumping - Mechelen round A	30.12.2017	CSI-W5*	3	1.60	390	74	12	21.07.2006	S	AES	55	0	124	293	24	1007	0	M	1	1	no	spread	oxer	left	no	0	0	71,39	no
Mechelen	The Longines FEI World Cup Jumping - Mechelen round A	30.12.2017	CSI-W5*	3	1.60	390	74	12	21.07.2006	S	AES	55	0	124	293	24	1007	0	M	2	2	no	vertical	vertical	straight	yes	4	0	71,39	no
Mechelen	The Longines FEI World Cup Jumping - Mechelen round A	30.12.2017	CSI-W5*	3	1.60	390	74	12	21.07.2006	S	AES	55	0	124	293	24	1007	0	M	3	3	no	spread	oxer	right	no	0	0	71,39	no
Mechelen	The Longines FEI World Cup Jumping - Mechelen round A	30.12.2017	CSI-W5*	3	1.60	390	74	12	21.07.2006	S	AES	55	0	124	293	24	1007	0	M	4	4	no	vertical	vertical	right	no	0	0	71,39	no
Mechelen	The Longines FEI World Cup Jumping - Mechelen round A	30.12.2017	CSI-W5*	3	1.60	390	74	12	21.07.2006	S	AES	55	0	124	293	24	1007	0	M	5	5a	yes	spread	oxer	left	no	0	0	71,39	no
Mechelen	The Longines FEI World Cup Jumping - Mechelen round A	30.12.2017	CSI-W5*	3	1.60	390	74	12	21.07.2006	S	AES	55	0	124	293	24	1007	0	M	6	5b	yes	vertical	vertical	left	no	0	0	71,39	no
Mechelen	The Longines FEI World Cup Jumping - Mechelen round A	30.12.2017	CSI-W5*	3	1.60	390	74	12	21.07.2006	S	AES	55	0	124	293	24	1007	0	M	7	6	no	spread	oxer	straight	no	0	0	71,39	no
Mechelen	The Longines FEI World Cup Jumping - Mechelen round A	30.12.2017	CSI-W5*	3	1.60	390	74	12	21.07.2006	S	AES	55	0	124	293	24	1007	0	M	8	7	no	vertical	vertical with water	left	no	0	0	71,39	no
Mechelen	The Longines FEI World Cup Jumping - Mechelen round A	30.12.2017	CSI-W5*	3	1.60	390	74	12	21.07.2006	S	AES	55	0	124	293	24	1007	0	M	9	8	no	spread	triple bar	left	no	0	0	71,39	no
Mechelen	The Longines FEI World Cup Jumping - Mechelen round A	30.12.2017	CSI-W5*	3	1.60	390	74	12	21.07.2006	S	AES	55	0	124	293	24	1007	0	M	10	9	no	vertical	vertical	left	no	0	0	71,39	no
Mechelen	The Longines FEI World Cup Jumping - Mechelen round A	30.12.2017	CSI-W5*	3	1.60	390	74	12	21.07.2006	S	AES	55	0	124	293	24	1007	0	M	11	10	no	spread	oxer	right	no	0	0	71,39	no
Mechelen	The Longines FEI World Cup Jumping - Mechelen round A	30.12.2017	CSI-W5*	3	1.60	390	74	12	21.07.2006	S	AES	55	0	124	293	24	1007	0	M	12	11a	yes	vertical	vertical	right	no	0	0	71,39	no
Mechelen	The Longines FEI World Cup Jumping - Mechelen round A	30.12.2017	CSI-W5*	3	1.60	390	74	12	21.07.2006	S	AES	55	0	124	293	24	1007	0	M	13	11b	yes	spread	oxer	right	no	0	0	71,39	no
Mechelen	The Longines FEI World Cup Jumping - Mechelen round A	30.12.2017	CSI-W5*	3	1.60	390	74	12	21.07.2006	S	AES	55	0	124	293	24	1007	0	M	14	11c	yes	spread	oxer	right	no	0	0	71,39	no
Mechelen	The Longines FEI World Cup Jumping - Mechelen round A	30.12.2017	CSI-W5*	3	1.60	390	74	12	21.07.2006	S	AES	55	0	124	293	24	1007	0	M	15	12	no	vertical	vertical	left	no	0	0	71,39	no
Mechelen	The Longines FEI World Cup Jumping - Mechelen round A	30.12.2017	CSI-W5*	3	1.60	390	74	12	21.07.2006	S	AES	55	0	124	293	24	1007	0	M	16	13	no	spread	oxer	left	no	0	2	71,39	no
Mechelen	The Longines FEI World Cup Jumping - Mechelen round A	30.12.2017	CSI-W5*	3	1.60	390	206	10	22.04.2008	G	SF	137	5	126	319	33	82	2	M	1	1	no	spread	oxer	left	yes	4	0	69,84	no
Mechelen	The Longines FEI World Cup Jumping - Mechelen round A	30.12.2017	CSI-W5*	3	1.60	390	206	10	22.04.2008	G	SF	137	5	126	319	33	82	2	M	2	2	no	vertical	vertical	straight	no	0	0	69,84	no
Mechelen	The Longines FEI World Cup Jumping - Mechelen round A	30.12.2017	CSI-W5*	3	1.60	390	206	10	22.04.2008	G	SF	137	5	126	319	33	82	2	M	3	3	no	spread	oxer	right	no	0	0	69,84	no
Mechelen	The Longines FEI World Cup Jumping - Mechelen round A	30.12.2017	CSI-W5*	3	1.60	390	206	10	22.04.2008	G	SF	137	5	126	319	33	82	2	M	4	4	no	vertical	vertical	right	no	0	0	69,84	no
Mechelen	The Longines FEI World Cup Jumping - Mechelen round A	30.12.2017	CSI-W5*	3	1.60	390	206	10	22.04.2008	G	SF	137	5	126	319	33	82	2	M	5	5a	yes	spread	oxer	left	no	0	0	69,84	no
Mechelen	The Longines FEI World Cup Jumping - Mechelen round A	30.12.2017	CSI-W5*	3	1.60	390	206	10	22.04.2008	G	SF	137	5	126	319	33	82	2	M	6	5b	yes	vertical	vertical	left	no	0	0	69,84	no
Mechelen	The Longines FEI World Cup Jumping - Mechelen round A	30.12.2017	CSI-W5*	3	1.60	390	206	10	22.04.2008	G	SF	137	5	126	319	33	82	2	M	7	6	no	spread	oxer	straight	no	0	0	69,84	no
Mechelen	The Longines FEI World Cup Jumping - Mechelen round A	30.12.2017	CSI-W5*	3	1.60	390	206	10	22.04.2008	G	SF	137	5	126	319	33	82	2	M	8	7	no	vertical	vertical with water	left	no	0	0	69,84	no
Mechelen	The Longines FEI World Cup Jumping - Mechelen round A	30.12.2017	CSI-W5*	3	1.60	390	206	10	22.04.2008	G	SF	137	5	126	319	33	82	2	M	9	8	no	spread	triple bar	left	no	0	0	69,84	no
Mechelen	The Longines FEI World Cup Jumping - Mechelen round A	30.12.2017	CSI-W5*	3	1.60	390	206	10	22.04.2008	G	SF	137	5	126	319	33	82	2	M	10	9	no	vertical	vertical	left	no	0	0	69,84	no
Mechelen	The Longines FEI World Cup Jumping - Mechelen round A	30.12.2017	CSI-W5*	3	1.60	390	206	10	22.04.2008	G	SF	137	5	126	319	33	82	2	M	11	10	no	spread	oxer	right	no	0	0	69,84	no
Mechelen	The Longines FEI World Cup Jumping - Mechelen round A	30.12.2017	CSI-W5*	3	1.60	390	206	10	22.04.2008	G	SF	137	5	126	319	33	82	2	M	12	11a	yes	vertical	vertical	right	no	0	0	69,84	no
Mechelen	The Longines FEI World Cup Jumping - Mechelen round A	30.12.2017	CSI-W5*	3	1.60	390	206	10	22.04.2008	G	SF	137	5	126	319	33	82	2	M	13	11b	yes	spread	oxer	right	yes	4	0	69,84	no
Mechelen	The Longines FEI World Cup Jumping - Mechelen round A	30.12.2017	CSI-W5*	3	1.60	390	206	10	22.04.2008	G	SF	137	5	126	319	33	82	2	M	14	11c	yes	spread	oxer	right	yes	4	0	69,84	no
Mechelen	The Longines FEI World Cup Jumping - Mechelen round A	30.12.2017	CSI-W5*	3	1.60	390	206	10	22.04.2008	G	SF	137	5	126	319	33	82	2	M	15	12	no	vertical	vertical	left	no	0	0	69,84	no
Mechelen	The Longines FEI World Cup Jumping - Mechelen round A	30.12.2017	CSI-W5*	3	1.60	390	206	10	22.04.2008	G	SF	137	5	126	319	33	82	2	M	16	13	no	spread	oxer	left	no	0	1	69,84	no
Mechelen	The Longines FEI World Cup Jumping - Mechelen round A	30.12.2017	CSI-W5*	3	1.60	390	110	11	04.03.2007	G	BWP	91	3	127	615	26	880	39	M	1	1	no	spread	oxer	left	no	0	0	67,8	no
Mechelen	The Longines FEI World Cup Jumping - Mechelen round A	30.12.2017	CSI-W5*	3	1.60	390	110	11	04.03.2007	G	BWP	91	3	127	615	26	880	39	M	2	2	no	vertical	vertical	straight	no	0	0	67,8	no
Mechelen	The Longines FEI World Cup Jumping - Mechelen round A	30.12.2017	CSI-W5*	3	1.60	390	110	11	04.03.2007	G	BWP	91	3	127	615	26	880	39	M	3	3	no	spread	oxer	right	no	0	0	67,8	no
Mechelen	The Longines FEI World Cup Jumping - Mechelen round A	30.12.2017	CSI-W5*	3	1.60	390	110	11	04.03.2007	G	BWP	91	3	127	615	26	880	39	M	4	4	no	vertical	vertical	right	no	0	0	67,8	no
Mechelen	The Longines FEI World Cup Jumping - Mechelen round A	30.12.2017	CSI-W5*	3	1.60	390	110	11	04.03.2007	G	BWP	91	3	127	615	26	880	39	M	5	5a	yes	spread	oxer	left	yes	4	0	67,8	no
Mechelen	The Longines FEI World Cup Jumping - Mechelen round A	30.12.2017	CSI-W5*	3	1.60	390	110	11	04.03.2007	G	BWP	91	3	127	615	26	880	39	M	6	5b	yes	vertical	vertical	left	no	0	0	67,8	no
Mechelen	The Longines FEI World Cup Jumping - Mechelen round A	30.12.2017	CSI-W5*	3	1.60	390	110	11	04.03.2007	G	BWP	91	3	127	615	26	880	39	M	7	6	no	spread	oxer	straight	no	0	0	67,8	no
Mechelen	The Longines FEI World Cup Jumping - Mechelen round A	30.12.2017	CSI-W5*	3	1.60	390	110	11	04.03.2007	G	BWP	91	3	127	615	26	880	39	M	8	7	no	vertical	vertical with water	left	no	0	0	67,8	no
Mechelen	The Longines FEI World Cup Jumping - Mechelen round A	30.12.2017	CSI-W5*	3	1.60	390	110	11	04.03.2007	G	BWP	91	3	127	615	26	880	39	M	9	8	no	spread	triple bar	left	no	0	0	67,8	no
Mechelen	The Longines FEI World Cup Jumping - Mechelen round A	30.12.2017	CSI-W5*	3	1.60	390	110	11	04.03.2007	G	BWP	91	3	127	615	26	880	39	M	10	9	no	vertical	vertical	left	no	0	0	67,8	no
Mechelen	The Longines FEI World Cup Jumping - Mechelen round A	30.12.2017	CSI-W5*	3	1.60	390	110	11	04.03.2007	G	BWP	91	3	127	615	26	880	39	M	11	10	no	spread	oxer	right	no	0	0	67,8	no
Mechelen	The Longines FEI World Cup Jumping - Mechelen round A	30.12.2017	CSI-W5*	3	1.60	390	110	11	04.03.2007	G	BWP	91	3	127	615	26	880	39	M	12	11a	yes	vertical	vertical	right	no	0	0	67,8	no
Mechelen	The Longines FEI World Cup Jumping - Mechelen round A	30.12.2017	CSI-W5*	3	1.60	390	110	11	04.03.2007	G	BWP	91	3	127	615	26	880	39	M	13	11b	yes	spread	oxer	right	no	0	0	67,8	no
Mechelen	The Longines FEI World Cup Jumping - Mechelen round A	30.12.2017	CSI-W5*	3	1.60	390	110	11	04.03.2007	G	BWP	91	3	127	615	26	880	39	M	14	11c	yes	spread	oxer	right	no	0	0	67,8	no
Mechelen	The Longines FEI World Cup Jumping - Mechelen round A	30.12.2017	CSI-W5*	3	1.60	390	110	11	04.03.2007	G	BWP	91	3	127	615	26	880	39	M	15	12	no	vertical	vertical	left	no	0	0	67,8	no
Mechelen	The Longines FEI World Cup Jumping - Mechelen round A	30.12.2017	CSI-W5*	3	1.60	390	110	11	04.03.2007	G	BWP	91	3	127	615	26	880	39	M	16	13	no	spread	oxer	left	no	0	1	67,8	no
Mechelen	The Longines FEI World Cup Jumping - Mechelen round A	30.12.2017	CSI-W5*	3	1.60	390	30	13	17.05.2005	G	WESTF	153	1	128	78	33	838	18	M	1	1	no	spread	oxer	left	no	0	0	66,69	no
Mechelen	The Longines FEI World Cup Jumping - Mechelen round A	30.12.2017	CSI-W5*	3	1.60	390	30	13	17.05.2005	G	WESTF	153	1	128	78	33	838	18	M	2	2	no	vertical	vertical	straight	no	0	0	66,69	no
Mechelen	The Longines FEI World Cup Jumping - Mechelen round A	30.12.2017	CSI-W5*	3	1.60	390	30	13	17.05.2005	G	WESTF	153	1	128	78	33	838	18	M	3	3	no	spread	oxer	right	no	0	0	66,69	no
Mechelen	The Longines FEI World Cup Jumping - Mechelen round A	30.12.2017	CSI-W5*	3	1.60	390	30	13	17.05.2005	G	WESTF	153	1	128	78	33	838	18	M	4	4	no	vertical	vertical	right	yes	4	0	66,69	no
Mechelen	The Longines FEI World Cup Jumping - Mechelen round A	30.12.2017	CSI-W5*	3	1.60	390	30	13	17.05.2005	G	WESTF	153	1	128	78	33	838	18	M	5	5a	yes	spread	oxer	left	no	0	0	66,69	no
Mechelen	The Longines FEI World Cup Jumping - Mechelen round A	30.12.2017	CSI-W5*	3	1.60	390	30	13	17.05.2005	G	WESTF	153	1	128	78	33	838	18	M	6	5b	yes	vertical	vertical	left	no	0	0	66,69	no
Mechelen	The Longines FEI World Cup Jumping - Mechelen round A	30.12.2017	CSI-W5*	3	1.60	390	30	13	17.05.2005	G	WESTF	153	1	128	78	33	838	18	M	7	6	no	spread	oxer	straight	no	0	0	66,69	no
Mechelen	The Longines FEI World Cup Jumping - Mechelen round A	30.12.2017	CSI-W5*	3	1.60	390	30	13	17.05.2005	G	WESTF	153	1	128	78	33	838	18	M	8	7	no	vertical	vertical with water	left	no	0	0	66,69	no
Mechelen	The Longines FEI World Cup Jumping - Mechelen round A	30.12.2017	CSI-W5*	3	1.60	390	30	13	17.05.2005	G	WESTF	153	1	128	78	33	838	18	M	9	8	no	spread	triple bar	left	no	0	0	66,69	no
Mechelen	The Longines FEI World Cup Jumping - Mechelen round A	30.12.2017	CSI-W5*	3	1.60	390	30	13	17.05.2005	G	WESTF	153	1	128	78	33	838	18	M	10	9	no	vertical	vertical	left	no	0	0	66,69	no
Mechelen	The Longines FEI World Cup Jumping - Mechelen round A	30.12.2017	CSI-W5*	3	1.60	390	30	13	17.05.2005	G	WESTF	153	1	128	78	33	838	18	M	11	10	no	spread	oxer	right	no	0	0	66,69	no
Mechelen	The Longines FEI World Cup Jumping - Mechelen round A	30.12.2017	CSI-W5*	3	1.60	390	30	13	17.05.2005	G	WESTF	153	1	128	78	33	838	18	M	12	11a	yes	vertical	vertical	right	no	0	0	66,69	no
Mechelen	The Longines FEI World Cup Jumping - Mechelen round A	30.12.2017	CSI-W5*	3	1.60	390	30	13	17.05.2005	G	WESTF	153	1	128	78	33	838	18	M	13	11b	yes	spread	oxer	right	no	0	0	66,69	no
Mechelen	The Longines FEI World Cup Jumping - Mechelen round A	30.12.2017	CSI-W5*	3	1.60	390	30	13	17.05.2005	G	WESTF	153	1	128	78	33	838	18	M	14	11c	yes	spread	oxer	right	no	0	0	66,69	no
Mechelen	The Longines FEI World Cup Jumping - Mechelen round A	30.12.2017	CSI-W5*	3	1.60	390	30	13	17.05.2005	G	WESTF	153	1	128	78	33	838	18	M	15	12	no	vertical	vertical	left	no	0	0	66,69	no
Mechelen	The Longines FEI World Cup Jumping - Mechelen round A	30.12.2017	CSI-W5*	3	1.60	390	30	13	17.05.2005	G	WESTF	153	1	128	78	33	838	18	M	16	13	no	spread	oxer	left	no	0	0	66,69	no
Leipzig	The Longines FEI World Cup Jumping - Leipzig round A	21.01.2018	CSI-W5*	4	1.60	390	54	12	17.03.2006	M	WESTF	171	6	1	19	38	2803	179	M	1	1	no	vertical	vertical	left	no	0	0	69,08	no
Leipzig	The Longines FEI World Cup Jumping - Leipzig round A	21.01.2018	CSI-W5*	4	1.60	390	54	12	17.03.2006	M	WESTF	171	6	1	19	38	2803	179	M	2	2	no	spread	oxer	straight	no	0	0	69,08	no
Leipzig	The Longines FEI World Cup Jumping - Leipzig round A	21.01.2018	CSI-W5*	4	1.60	390	54	12	17.03.2006	M	WESTF	171	6	1	19	38	2803	179	M	3	3	no	spread	oxer	left	no	0	0	69,08	no
Leipzig	The Longines FEI World Cup Jumping - Leipzig round A	21.01.2018	CSI-W5*	4	1.60	390	54	12	17.03.2006	M	WESTF	171	6	1	19	38	2803	179	M	4	4	no	vertical	vertical	right	no	0	0	69,08	no
Leipzig	The Longines FEI World Cup Jumping - Leipzig round A	21.01.2018	CSI-W5*	4	1.60	390	54	12	17.03.2006	M	WESTF	171	6	1	19	38	2803	179	M	5	5a	yes	vertical	vertical	right	no	0	0	69,08	no
Leipzig	The Longines FEI World Cup Jumping - Leipzig round A	21.01.2018	CSI-W5*	4	1.60	390	54	12	17.03.2006	M	WESTF	171	6	1	19	38	2803	179	M	6	5b	yes	spread	oxer	right	no	0	0	69,08	no
Leipzig	The Longines FEI World Cup Jumping - Leipzig round A	21.01.2018	CSI-W5*	4	1.60	390	54	12	17.03.2006	M	WESTF	171	6	1	19	38	2803	179	M	7	5c	yes	spread	oxer	right	no	0	0	69,08	no
Leipzig	The Longines FEI World Cup Jumping - Leipzig round A	21.01.2018	CSI-W5*	4	1.60	390	54	12	17.03.2006	M	WESTF	171	6	1	19	38	2803	179	M	8	6	no	vertical	vertical	left	yes	4	0	69,08	no
Leipzig	The Longines FEI World Cup Jumping - Leipzig round A	21.01.2018	CSI-W5*	4	1.60	390	54	12	17.03.2006	M	WESTF	171	6	1	19	38	2803	179	M	9	7	no	spread	oxer with water	left	no	0	0	69,08	no
Leipzig	The Longines FEI World Cup Jumping - Leipzig round A	21.01.2018	CSI-W5*	4	1.60	390	54	12	17.03.2006	M	WESTF	171	6	1	19	38	2803	179	M	10	8	no	spread	oxer	straight	no	0	0	69,08	no
Leipzig	The Longines FEI World Cup Jumping - Leipzig round A	21.01.2018	CSI-W5*	4	1.60	390	54	12	17.03.2006	M	WESTF	171	6	1	19	38	2803	179	M	11	9	no	vertical	vertical	left	no	0	0	69,08	no
Leipzig	The Longines FEI World Cup Jumping - Leipzig round A	21.01.2018	CSI-W5*	4	1.60	390	54	12	17.03.2006	M	WESTF	171	6	1	19	38	2803	179	M	12	10	no	spread	oxer	left	no	0	0	69,08	no
Leipzig	The Longines FEI World Cup Jumping - Leipzig round A	21.01.2018	CSI-W5*	4	1.60	390	54	12	17.03.2006	M	WESTF	171	6	1	19	38	2803	179	M	13	11a	yes	spread	oxer	right	no	0	0	69,08	no
Leipzig	The Longines FEI World Cup Jumping - Leipzig round A	21.01.2018	CSI-W5*	4	1.60	390	54	12	17.03.2006	M	WESTF	171	6	1	19	38	2803	179	M	14	11b	yes	vertical	vertical	right	no	0	0	69,08	no
Leipzig	The Longines FEI World Cup Jumping - Leipzig round A	21.01.2018	CSI-W5*	4	1.60	390	54	12	17.03.2006	M	WESTF	171	6	1	19	38	2803	179	M	15	12	no	vertical	vertical	right	no	0	0	69,08	no
Leipzig	The Longines FEI World Cup Jumping - Leipzig round A	21.01.2018	CSI-W5*	4	1.60	390	82	14	17.04.2004	G	BWP	214	17	5	15	32	2092	63	M	1	1	no	vertical	vertical	left	no	0	0	69,7	no
Leipzig	The Longines FEI World Cup Jumping - Leipzig round A	21.01.2018	CSI-W5*	4	1.60	390	82	14	17.04.2004	G	BWP	214	17	5	15	32	2092	63	M	2	2	no	spread	oxer	straight	no	0	0	69,7	no
Leipzig	The Longines FEI World Cup Jumping - Leipzig round A	21.01.2018	CSI-W5*	4	1.60	390	82	14	17.04.2004	G	BWP	214	17	5	15	32	2092	63	M	3	3	no	spread	oxer	left	yes	4	0	69,7	no
Leipzig	The Longines FEI World Cup Jumping - Leipzig round A	21.01.2018	CSI-W5*	4	1.60	390	82	14	17.04.2004	G	BWP	214	17	5	15	32	2092	63	M	4	4	no	vertical	vertical	right	no	0	0	69,7	no
Leipzig	The Longines FEI World Cup Jumping - Leipzig round A	21.01.2018	CSI-W5*	4	1.60	390	82	14	17.04.2004	G	BWP	214	17	5	15	32	2092	63	M	5	5a	yes	vertical	vertical	right	no	0	0	69,7	no
Leipzig	The Longines FEI World Cup Jumping - Leipzig round A	21.01.2018	CSI-W5*	4	1.60	390	82	14	17.04.2004	G	BWP	214	17	5	15	32	2092	63	M	6	5b	yes	spread	oxer	right	no	0	0	69,7	no
Leipzig	The Longines FEI World Cup Jumping - Leipzig round A	21.01.2018	CSI-W5*	4	1.60	390	82	14	17.04.2004	G	BWP	214	17	5	15	32	2092	63	M	7	5c	yes	spread	oxer	right	no	0	0	69,7	no
Leipzig	The Longines FEI World Cup Jumping - Leipzig round A	21.01.2018	CSI-W5*	4	1.60	390	82	14	17.04.2004	G	BWP	214	17	5	15	32	2092	63	M	8	6	no	vertical	vertical	left	no	0	0	69,7	no
Leipzig	The Longines FEI World Cup Jumping - Leipzig round A	21.01.2018	CSI-W5*	4	1.60	390	82	14	17.04.2004	G	BWP	214	17	5	15	32	2092	63	M	9	7	no	spread	oxer with water	left	no	0	0	69,7	no
Leipzig	The Longines FEI World Cup Jumping - Leipzig round A	21.01.2018	CSI-W5*	4	1.60	390	82	14	17.04.2004	G	BWP	214	17	5	15	32	2092	63	M	10	8	no	spread	oxer	straight	no	0	0	69,7	no
Leipzig	The Longines FEI World Cup Jumping - Leipzig round A	21.01.2018	CSI-W5*	4	1.60	390	82	14	17.04.2004	G	BWP	214	17	5	15	32	2092	63	M	11	9	no	vertical	vertical	left	no	0	0	69,7	no
Leipzig	The Longines FEI World Cup Jumping - Leipzig round A	21.01.2018	CSI-W5*	4	1.60	390	82	14	17.04.2004	G	BWP	214	17	5	15	32	2092	63	M	12	10	no	spread	oxer	left	no	0	0	69,7	no
Leipzig	The Longines FEI World Cup Jumping - Leipzig round A	21.01.2018	CSI-W5*	4	1.60	390	82	14	17.04.2004	G	BWP	214	17	5	15	32	2092	63	M	13	11a	yes	spread	oxer	right	no	0	0	69,7	no
Leipzig	The Longines FEI World Cup Jumping - Leipzig round A	21.01.2018	CSI-W5*	4	1.60	390	82	14	17.04.2004	G	BWP	214	17	5	15	32	2092	63	M	14	11b	yes	vertical	vertical	right	no	0	0	69,7	no
Leipzig	The Longines FEI World Cup Jumping - Leipzig round A	21.01.2018	CSI-W5*	4	1.60	390	82	14	17.04.2004	G	BWP	214	17	5	15	32	2092	63	M	15	12	no	vertical	vertical	right	no	0	0	69,7	no
Leipzig	The Longines FEI World Cup Jumping - Leipzig round A	21.01.2018	CSI-W5*	4	1.60	390	195	11	25.04.2007	G	SF	164	16	9	103	48	1417	59	M	1	1	no	vertical	vertical	left	no	0	0	78,8	no
Leipzig	The Longines FEI World Cup Jumping - Leipzig round A	21.01.2018	CSI-W5*	4	1.60	390	195	11	25.04.2007	G	SF	164	16	9	103	48	1417	59	M	2	2	no	spread	oxer	straight	no	0	0	78,8	no
Leipzig	The Longines FEI World Cup Jumping - Leipzig round A	21.01.2018	CSI-W5*	4	1.60	390	195	11	25.04.2007	G	SF	164	16	9	103	48	1417	59	M	3	3	no	spread	oxer	left	no	0	0	78,8	no
Leipzig	The Longines FEI World Cup Jumping - Leipzig round A	21.01.2018	CSI-W5*	4	1.60	390	195	11	25.04.2007	G	SF	164	16	9	103	48	1417	59	M	4	4	no	vertical	vertical	right	no	0	0	78,8	no
Leipzig	The Longines FEI World Cup Jumping - Leipzig round A	21.01.2018	CSI-W5*	4	1.60	390	195	11	25.04.2007	G	SF	164	16	9	103	48	1417	59	M	5	5a	yes	vertical	vertical	right	no	0	0	78,8	no
Leipzig	The Longines FEI World Cup Jumping - Leipzig round A	21.01.2018	CSI-W5*	4	1.60	390	195	11	25.04.2007	G	SF	164	16	9	103	48	1417	59	M	6	5b	yes	spread	oxer	right	no	0	0	78,8	no
Leipzig	The Longines FEI World Cup Jumping - Leipzig round A	21.01.2018	CSI-W5*	4	1.60	390	195	11	25.04.2007	G	SF	164	16	9	103	48	1417	59	M	7	5c	yes	spread	oxer	right	yes	4	0	78,8	no
Leipzig	The Longines FEI World Cup Jumping - Leipzig round A	21.01.2018	CSI-W5*	4	1.60	390	195	11	25.04.2007	G	SF	164	16	9	103	48	1417	59	M	8	6	no	vertical	vertical	left	no	0	0	78,8	no
Leipzig	The Longines FEI World Cup Jumping - Leipzig round A	21.01.2018	CSI-W5*	4	1.60	390	195	11	25.04.2007	G	SF	164	16	9	103	48	1417	59	M	9	7	no	spread	oxer with water	left	no	0	0	78,8	no
Leipzig	The Longines FEI World Cup Jumping - Leipzig round A	21.01.2018	CSI-W5*	4	1.60	390	195	11	25.04.2007	G	SF	164	16	9	103	48	1417	59	M	10	8	no	spread	oxer	straight	no	0	0	78,8	no
Leipzig	The Longines FEI World Cup Jumping - Leipzig round A	21.01.2018	CSI-W5*	4	1.60	390	195	11	25.04.2007	G	SF	164	16	9	103	48	1417	59	M	11	9	no	vertical	vertical	left	no	0	0	78,8	no
Leipzig	The Longines FEI World Cup Jumping - Leipzig round A	21.01.2018	CSI-W5*	4	1.60	390	195	11	25.04.2007	G	SF	164	16	9	103	48	1417	59	M	12	10	no	spread	oxer	left	no	0	0	78,8	no
Leipzig	The Longines FEI World Cup Jumping - Leipzig round A	21.01.2018	CSI-W5*	4	1.60	390	195	11	25.04.2007	G	SF	164	16	9	103	48	1417	59	M	13	11a	yes	spread	oxer	right	no	0	0	78,8	no
Leipzig	The Longines FEI World Cup Jumping - Leipzig round A	21.01.2018	CSI-W5*	4	1.60	390	195	11	25.04.2007	G	SF	164	16	9	103	48	1417	59	M	14	11b	yes	vertical	vertical	right	no	0	0	78,8	no
Leipzig	The Longines FEI World Cup Jumping - Leipzig round A	21.01.2018	CSI-W5*	4	1.60	390	195	11	25.04.2007	G	SF	164	16	9	103	48	1417	59	M	15	12	no	vertical	vertical	right	yes	4	3	78,8	no
Leipzig	The Longines FEI World Cup Jumping - Leipzig round A	21.01.2018	CSI-W5*	4	1.60	390	122	13	04.05.2005	G	HOLST	228	19	14	5	37	1868	96	M	1	1	no	vertical	vertical	left	no	0	0	66,27	no
Leipzig	The Longines FEI World Cup Jumping - Leipzig round A	21.01.2018	CSI-W5*	4	1.60	390	122	13	04.05.2005	G	HOLST	228	19	14	5	37	1868	96	M	2	2	no	spread	oxer	straight	no	0	0	66,27	no
Leipzig	The Longines FEI World Cup Jumping - Leipzig round A	21.01.2018	CSI-W5*	4	1.60	390	122	13	04.05.2005	G	HOLST	228	19	14	5	37	1868	96	M	3	3	no	spread	oxer	left	no	0	0	66,27	no
Leipzig	The Longines FEI World Cup Jumping - Leipzig round A	21.01.2018	CSI-W5*	4	1.60	390	122	13	04.05.2005	G	HOLST	228	19	14	5	37	1868	96	M	4	4	no	vertical	vertical	right	no	0	0	66,27	no
Leipzig	The Longines FEI World Cup Jumping - Leipzig round A	21.01.2018	CSI-W5*	4	1.60	390	122	13	04.05.2005	G	HOLST	228	19	14	5	37	1868	96	M	5	5a	yes	vertical	vertical	right	no	0	0	66,27	no
Leipzig	The Longines FEI World Cup Jumping - Leipzig round A	21.01.2018	CSI-W5*	4	1.60	390	122	13	04.05.2005	G	HOLST	228	19	14	5	37	1868	96	M	6	5b	yes	spread	oxer	right	no	0	0	66,27	no
Leipzig	The Longines FEI World Cup Jumping - Leipzig round A	21.01.2018	CSI-W5*	4	1.60	390	122	13	04.05.2005	G	HOLST	228	19	14	5	37	1868	96	M	7	5c	yes	spread	oxer	right	no	0	0	66,27	no
Leipzig	The Longines FEI World Cup Jumping - Leipzig round A	21.01.2018	CSI-W5*	4	1.60	390	122	13	04.05.2005	G	HOLST	228	19	14	5	37	1868	96	M	8	6	no	vertical	vertical	left	no	0	0	66,27	no
Leipzig	The Longines FEI World Cup Jumping - Leipzig round A	21.01.2018	CSI-W5*	4	1.60	390	122	13	04.05.2005	G	HOLST	228	19	14	5	37	1868	96	M	9	7	no	spread	oxer with water	left	yes	4	0	66,27	no
Leipzig	The Longines FEI World Cup Jumping - Leipzig round A	21.01.2018	CSI-W5*	4	1.60	390	122	13	04.05.2005	G	HOLST	228	19	14	5	37	1868	96	M	10	8	no	spread	oxer	straight	no	0	0	66,27	no
Leipzig	The Longines FEI World Cup Jumping - Leipzig round A	21.01.2018	CSI-W5*	4	1.60	390	122	13	04.05.2005	G	HOLST	228	19	14	5	37	1868	96	M	11	9	no	vertical	vertical	left	no	0	0	66,27	no
Leipzig	The Longines FEI World Cup Jumping - Leipzig round A	21.01.2018	CSI-W5*	4	1.60	390	122	13	04.05.2005	G	HOLST	228	19	14	5	37	1868	96	M	12	10	no	spread	oxer	left	no	0	0	66,27	no
Leipzig	The Longines FEI World Cup Jumping - Leipzig round A	21.01.2018	CSI-W5*	4	1.60	390	122	13	04.05.2005	G	HOLST	228	19	14	5	37	1868	96	M	13	11a	yes	spread	oxer	right	no	0	0	66,27	no
Leipzig	The Longines FEI World Cup Jumping - Leipzig round A	21.01.2018	CSI-W5*	4	1.60	390	122	13	04.05.2005	G	HOLST	228	19	14	5	37	1868	96	M	14	11b	yes	vertical	vertical	right	no	0	0	66,27	no
Leipzig	The Longines FEI World Cup Jumping - Leipzig round A	21.01.2018	CSI-W5*	4	1.60	390	122	13	04.05.2005	G	HOLST	228	19	14	5	37	1868	96	M	15	12	no	vertical	vertical	right	no	0	0	66,27	no
Leipzig	The Longines FEI World Cup Jumping - Leipzig round A	21.01.2018	CSI-W5*	4	1.60	390	57	15	04.04.2003	G	WESTF	230	13	16	17	37	2215	106	M	1	1	no	vertical	vertical	left	no	0	0	67,04	no
Leipzig	The Longines FEI World Cup Jumping - Leipzig round A	21.01.2018	CSI-W5*	4	1.60	390	57	15	04.04.2003	G	WESTF	230	13	16	17	37	2215	106	M	2	2	no	spread	oxer	straight	no	0	0	67,04	no
Leipzig	The Longines FEI World Cup Jumping - Leipzig round A	21.01.2018	CSI-W5*	4	1.60	390	57	15	04.04.2003	G	WESTF	230	13	16	17	37	2215	106	M	3	3	no	spread	oxer	left	no	0	0	67,04	no
Leipzig	The Longines FEI World Cup Jumping - Leipzig round A	21.01.2018	CSI-W5*	4	1.60	390	57	15	04.04.2003	G	WESTF	230	13	16	17	37	2215	106	M	4	4	no	vertical	vertical	right	no	0	0	67,04	no
Leipzig	The Longines FEI World Cup Jumping - Leipzig round A	21.01.2018	CSI-W5*	4	1.60	390	57	15	04.04.2003	G	WESTF	230	13	16	17	37	2215	106	M	5	5a	yes	vertical	vertical	right	no	0	0	67,04	no
Leipzig	The Longines FEI World Cup Jumping - Leipzig round A	21.01.2018	CSI-W5*	4	1.60	390	57	15	04.04.2003	G	WESTF	230	13	16	17	37	2215	106	M	6	5b	yes	spread	oxer	right	no	0	0	67,04	no
Leipzig	The Longines FEI World Cup Jumping - Leipzig round A	21.01.2018	CSI-W5*	4	1.60	390	57	15	04.04.2003	G	WESTF	230	13	16	17	37	2215	106	M	7	5c	yes	spread	oxer	right	no	0	0	67,04	no
Leipzig	The Longines FEI World Cup Jumping - Leipzig round A	21.01.2018	CSI-W5*	4	1.60	390	57	15	04.04.2003	G	WESTF	230	13	16	17	37	2215	106	M	8	6	no	vertical	vertical	left	no	0	0	67,04	no

Leipzig	The Longines FEI World Cup Jumping - Leipzig round A	21.01.2018	CSI-W5*	4	1.60	390	57	15	04.04.2003	G	WESTF	230	13	16	17	37	2215	106	M	9	7	no	spread	oxer with water	left	no	0	0	67,04	no
Leipzig	The Longines FEI World Cup Jumping - Leipzig round A	21.01.2018	CSI-W5*	4	1.60	390	57	15	04.04.2003	G	WESTF	230	13	16	17	37	2215	106	M	10	8	no	spread	oxer	straight	no	0	0	67,04	no
Leipzig	The Longines FEI World Cup Jumping - Leipzig round A	21.01.2018	CSI-W5*	4	1.60	390	57	15	04.04.2003	G	WESTF	230	13	16	17	37	2215	106	M	11	9	no	vertical	vertical	left	no	0	0	67,04	no
Leipzig	The Longines FEI World Cup Jumping - Leipzig round A	21.01.2018	CSI-W5*	4	1.60	390	57	15	04.04.2003	G	WESTF	230	13	16	17	37	2215	106	M	12	10	no	spread	oxer	left	no	0	0	67,04	no
Leipzig	The Longines FEI World Cup Jumping - Leipzig round A	21.01.2018	CSI-W5*	4	1.60	390	57	15	04.04.2003	G	WESTF	230	13	16	17	37	2215	106	M	13	11a	yes	spread	oxer	right	no	0	0	67,04	no
Leipzig	The Longines FEI World Cup Jumping - Leipzig round A	21.01.2018	CSI-W5*	4	1.60	390	57	15	04.04.2003	G	WESTF	230	13	16	17	37	2215	106	M	14	11b	yes	vertical	vertical	right	yes	4	0	67,04	no
Leipzig	The Longines FEI World Cup Jumping - Leipzig round A	21.01.2018	CSI-W5*	4	1.60	390	57	15	04.04.2003	G	WESTF	230	13	16	17	37	2215	106	M	15	12	no	vertical	vertical	right	no	0	0	67,04	no
Leipzig	The Longines FEI World Cup Jumping - Leipzig round A	21.01.2018	CSI-W5*	4	1.60	390	104	11	23.03.2007	G	KWPN	95	0	17	3	46	1005	51	M	1	1	no	vertical	vertical	left	no	0	0	65,71	no
Leipzig	The Longines FEI World Cup Jumping - Leipzig round A	21.01.2018	CSI-W5*	4	1.60	390	104	11	23.03.2007	G	KWPN	95	0	17	3	46	1005	51	M	2	2	no	spread	oxer	straight	no	0	0	65,71	no
Leipzig	The Longines FEI World Cup Jumping - Leipzig round A	21.01.2018	CSI-W5*	4	1.60	390	104	11	23.03.2007	G	KWPN	95	0	17	3	46	1005	51	M	3	3	no	spread	oxer	left	no	0	0	65,71	no
Leipzig	The Longines FEI World Cup Jumping - Leipzig round A	21.01.2018	CSI-W5*	4	1.60	390	104	11	23.03.2007	G	KWPN	95	0	17	3	46	1005	51	M	4	4	no	vertical	vertical	right	no	0	0	65,71	no
Leipzig	The Longines FEI World Cup Jumping - Leipzig round A	21.01.2018	CSI-W5*	4	1.60	390	104	11	23.03.2007	G	KWPN	95	0	17	3	46	1005	51	M	5	5a	yes	vertical	vertical	right	no	0	0	65,71	no
Leipzig	The Longines FEI World Cup Jumping - Leipzig round A	21.01.2018	CSI-W5*	4	1.60	390	104	11	23.03.2007	G	KWPN	95	0	17	3	46	1005	51	M	6	5b	yes	spread	oxer	right	no	0	0	65,71	no
Leipzig	The Longines FEI World Cup Jumping - Leipzig round A	21.01.2018	CSI-W5*	4	1.60	390	104	11	23.03.2007	G	KWPN	95	0	17	3	46	1005	51	M	7	5c	yes	spread	oxer	right	no	0	0	65,71	no
Leipzig	The Longines FEI World Cup Jumping - Leipzig round A	21.01.2018	CSI-W5*	4	1.60	390	104	11	23.03.2007	G	KWPN	95	0	17	3	46	1005	51	M	8	6	no	vertical	vertical	left	no	0	0	65,71	no
Leipzig	The Longines FEI World Cup Jumping - Leipzig round A	21.01.2018	CSI-W5*	4	1.60	390	104	11	23.03.2007	G	KWPN	95	0	17	3	46	1005	51	M	9	7	no	spread	oxer with water	left	no	0	0	65,71	no
Leipzig	The Longines FEI World Cup Jumping - Leipzig round A	21.01.2018	CSI-W5*	4	1.60	390	104	11	23.03.2007	G	KWPN	95	0	17	3	46	1005	51	M	10	8	no	spread	oxer	straight	no	0	0	65,71	no
Leipzig	The Longines FEI World Cup Jumping - Leipzig round A	21.01.2018	CSI-W5*	4	1.60	390	104	11	23.03.2007	G	KWPN	95	0	17	3	46	1005	51	M	11	9	no	vertical	vertical	left	no	0	0	65,71	no
Leipzig	The Longines FEI World Cup Jumping - Leipzig round A	21.01.2018	CSI-W5*	4	1.60	390	104	11	23.03.2007	G	KWPN	95	0	17	3	46	1005	51	M	12	10	no	spread	oxer	left	no	0	0	65,71	no
Leipzig	The Longines FEI World Cup Jumping - Leipzig round A	21.01.2018	CSI-W5*	4	1.60	390	104	11	23.03.2007	G	KWPN	95	0	17	3	46	1005	51	M	13	11a	yes	spread	oxer	right	yes	4	0	65,71	no
Leipzig	The Longines FEI World Cup Jumping - Leipzig round A	21.01.2018	CSI-W5*	4	1.60	390	104	11	23.03.2007	G	KWPN	95	0	17	3	46	1005	51	M	14	11b	yes	vertical	vertical	right	no	0	0	65,71	no
Leipzig	The Longines FEI World Cup Jumping - Leipzig round A	21.01.2018	CSI-W5*	4	1.60	390	104	11	23.03.2007	G	KWPN	95	0	17	3	46	1005	51	M	15	12	no	vertical	vertical	right	no	0	0	65,71	no
Leipzig	The Longines FEI World Cup Jumping - Leipzig round A	21.01.2018	CSI-W5*	4	1.60	390	219	13	13.05.2005	G	SWB	110	2	21	99	28	1384	56	F	1	1	no	vertical	vertical	left	no	0	0	66,86	no
Leipzig	The Longines FEI World Cup Jumping - Leipzig round A	21.01.2018	CSI-W5*	4	1.60	390	219	13	13.05.2005	G	SWB	110	2	21	99	28	1384	56	F	2	2	no	spread	oxer	straight	no	0	0	66,86	no
Leipzig	The Longines FEI World Cup Jumping - Leipzig round A	21.01.2018	CSI-W5*	4	1.60	390	219	13	13.05.2005	G	SWB	110	2	21	99	28	1384	56	F	3	3	no	spread	oxer	left	no	0	0	66,86	no
Leipzig	The Longines FEI World Cup Jumping - Leipzig round A	21.01.2018	CSI-W5*	4	1.60	390	219	13	13.05.2005	G	SWB	110	2	21	99	28	1384	56	F	4	4	no	vertical	vertical	right	no	0	0	66,86	no
Leipzig	The Longines FEI World Cup Jumping - Leipzig round A	21.01.2018	CSI-W5*	4	1.60	390	219	13	13.05.2005	G	SWB	110	2	21	99	28	1384	56	F	5	5a	yes	vertical	vertical	right	no	0	0	66,86	no
Leipzig	The Longines FEI World Cup Jumping - Leipzig round A	21.01.2018	CSI-W5*	4	1.60	390	219	13	13.05.2005	G	SWB	110	2	21	99	28	1384	56	F	6	5b	yes	spread	oxer	right	no	0	0	66,86	no
Leipzig	The Longines FEI World Cup Jumping - Leipzig round A	21.01.2018	CSI-W5*	4	1.60	390	219	13	13.05.2005	G	SWB	110	2	21	99	28	1384	56	F	7	5c	yes	spread	oxer	right	no	0	0	66,86	no
Leipzig	The Longines FEI World Cup Jumping - Leipzig round A	21.01.2018	CSI-W5*	4	1.60	390	219	13	13.05.2005	G	SWB	110	2	21	99	28	1384	56	F	8	6	no	vertical	vertical	left	no	0	0	66,86	no
Leipzig	The Longines FEI World Cup Jumping - Leipzig round A	21.01.2018	CSI-W5*	4	1.60	390	219	13	13.05.2005	G	SWB	110	2	21	99	28	1384	56	F	9	7	no	spread	oxer with water	left	no	0	0	66,86	no
Leipzig	The Longines FEI World Cup Jumping - Leipzig round A	21.01.2018	CSI-W5*	4	1.60	390	219	13	13.05.2005	G	SWB	110	2	21	99	28	1384	56	F	10	8	no	spread	oxer	straight	no	0	0	66,86	no
Leipzig	The Longines FEI World Cup Jumping - Leipzig round A	21.01.2018	CSI-W5*	4	1.60	390	219	13	13.05.2005	G	SWB	110	2	21	99	28	1384	56	F	11	9	no	vertical	vertical	left	no	0	0	66,86	no
Leipzig	The Longines FEI World Cup Jumping - Leipzig round A	21.01.2018	CSI-W5*	4	1.60	390	219	13	13.05.2005	G	SWB	110	2	21	99	28	1384	56	F	12	10	no	spread	oxer	left	no	0	0	66,86	no
Leipzig	The Longines FEI World Cup Jumping - Leipzig round A	21.01.2018	CSI-W5*	4	1.60	390	219	13	13.05.2005	G	SWB	110	2	21	99	28	1384	56	F	13	11a	yes	spread	oxer	right	no	0	0	66,86	no
Leipzig	The Longines FEI World Cup Jumping - Leipzig round A	21.01.2018	CSI-W5*	4	1.60	390	219	13	13.05.2005	G	SWB	110	2	21	99	28	1384	56	F	14	11b	yes	vertical	vertical	right	no	0	0	66,86	no
Leipzig	The Longines FEI World Cup Jumping - Leipzig round A	21.01.2018	CSI-W5*	4	1.60	390	219	13	13.05.2005	G	SWB	110	2	21	99	28	1384	56	F	15	12	no	vertical	vertical	right	no	0	0	66,86	no
Leipzig	The Longines FEI World Cup Jumping - Leipzig round B	21.01.2018	CSI-W5*	4	1.60	300	219	13	13.05.2005	G	SWB	110	2	21	99	28	1384	56	F	1	1	no	vertical	vertical	left	no	0	0	46,56	no
Leipzig	The Longines FEI World Cup Jumping - Leipzig round B	21.01.2018	CSI-W5*	4	1.60	300	219	13	13.05.2005	G	SWB	110	2	21	99	28	1384	56	F	2	2	no	spread	oxer	left	no	0	0	46,56	no
Leipzig	The Longines FEI World Cup Jumping - Leipzig round B	21.01.2018	CSI-W5*	4	1.60	300	219	13	13.05.2005	G	SWB	110	2	21	99	28	1384	56	F	3	3	no	vertical	vertical	right	no	0	0	46,56	no
Leipzig	The Longines FEI World Cup Jumping - Leipzig round B	21.01.2018	CSI-W5*	4	1.60	300	219	13	13.05.2005	G	SWB	110	2	21	99	28	1384	56	F	4	4a	yes	vertical	vertical	right	no	0	0	46,56	no
Leipzig	The Longines FEI World Cup Jumping - Leipzig round B	21.01.2018	CSI-W5*	4	1.60	300	219	13	13.05.2005	G	SWB	110	2	21	99	28	1384	56	F	5	4b	yes	spread	oxer	right	no	0	0	46,56	no
Leipzig	The Longines FEI World Cup Jumping - Leipzig round B	21.01.2018	CSI-W5*	4	1.60	300	219	13	13.05.2005	G	SWB	110	2	21	99	28	1384	56	F	6	5	no	spread	oxer	left	no	0	0	46,56	no
Leipzig	The Longines FEI World Cup Jumping - Leipzig round B	21.01.2018	CSI-W5*	4	1.60	300	219	13	13.05.2005	G	SWB	110	2	21	99	28	1384	56	F	7	6	no	vertical	vertical	left	no	0	0	46,56	no
Leipzig	The Longines FEI World Cup Jumping - Leipzig round B	21.01.2018	CSI-W5*	4	1.60	300	219	13	13.05.2005	G	SWB	110	2	21	99	28	1384	56	F	8	7	no	vertical	vertical	right	no	0	0	46,56	no
Leipzig	The Longines FEI World Cup Jumping - Leipzig round B	21.01.2018	CSI-W5*	4	1.60	300	219	13	13.05.2005	G	SWB	110	2	21	99	28	1384	56	F	9	8	no	spread	oxer	right	no	0	0	46,56	no
Leipzig	The Longines FEI World Cup Jumping - Leipzig round A	21.01.2018	CSI-W5*	4	1.60	390	5	13	31.03.2005	G	KWPN	238	4	25	558	26	820	16	F	1	1	no	vertical	vertical	left	no	0	0	70,92	no
Leipzig	The Longines FEI World Cup Jumping - Leipzig round A	21.01.2018	CSI-W5*	4	1.60	390	5	13	31.03.2005	G	KWPN	238	4	25	558	26	820	16	F	2	2	no	spread	oxer	straight	no	0	0	70,92	no
Leipzig	The Longines FEI World Cup Jumping - Leipzig round A	21.01.2018	CSI-W5*	4	1.60	390	5	13	31.03.2005	G	KWPN	238	4	25	558	26	820	16	F	3	3	no	spread	oxer	left	no	0	0	70,92	no
Leipzig	The Longines FEI World Cup Jumping - Leipzig round A	21.01.2018	CSI-W5*	4	1.60	390	5	13	31.03.2005	G	KWPN	238	4	25	558	26	820	16	F	4	4	no	vertical	vertical	right	no	0	0	70,92	no
Leipzig	The Longines FEI World Cup Jumping - Leipzig round A	21.01.2018	CSI-W5*	4	1.60	390	5	13	31.03.2005	G	KWPN	238	4	25	558	26	820	16	F	5	5a	yes	vertical	vertical	right	no	0	0	70,92	no
Leipzig	The Longines FEI World Cup Jumping - Leipzig round A	21.01.2018	CSI-W5*	4	1.60	390	5	13	31.03.2005	G	KWPN	238	4	25	558	26	820	16	F	6	5b	yes	spread	oxer	right	no	0	0	70,92	no
Leipzig	The Longines FEI World Cup Jumping - Leipzig round A	21.01.2018	CSI-W5*	4	1.60	390	5	13	31.03.2005	G	KWPN	238	4	25	558	26	820	16	F	7	5c	yes	spread	oxer	right	yes	4	0	70,92	no
Leipzig	The Longines FEI World Cup Jumping - Leipzig round A	21.01.2018	CSI-W5*	4	1.60	390	5	13	31.03.2005	G	KWPN	238	4	25	558	26	820	16	F	8	6	no	vertical	vertical	left	no	0	0	70,92	no
Leipzig	The Longines FEI World Cup Jumping - Leipzig round A	21.01.2018	CSI-W5*	4	1.60	390	5	13	31.03.2005	G	KWPN	238	4	25	558	26	820	16	F	9	7	no	spread	oxer with water	left	no	0	0	70,92	no
Leipzig	The Longines FEI World Cup Jumping - Leipzig round A	21.01.2018	CSI-W5*	4	1.60	390	5	13	31.03.2005	G	KWPN	238	4	25	558	26	820	16	F	10	8	no	spread	oxer	straight	no	0	0	70,92	no
Leipzig	The Longines FEI World Cup Jumping - Leipzig round A	21.01.2018	CSI-W5*	4	1.60	390	5	13	31.03.2005	G	KWPN	238	4	25	558	26	820	16	F	11	9	no	vertical	vertical	left	no	0	0	70,92	no
Leipzig	The Longines FEI World Cup Jumping - Leipzig round A	21.01.2018	CSI-W5*	4	1.60	390	5	13	31.03.2005	G	KWPN	238	4	25	558	26	820	16	F	12	10	no	spread	oxer	left	no	0	0	70,92	no
Leipzig	The Longines FEI World Cup Jumping - Leipzig round A	21.01.2018	CSI-W5*	4	1.60	390	5	13	31.03.2005	G	KWPN	238	4	25	558	26	820	16	F	13	11a	yes	spread	oxer	right	no	0	0	70,92	no
Leipzig	The Longines FEI World Cup Jumping - Leipzig round A	21.01.2018	CSI-W5*	4	1.60	390	5	13	31.03.2005	G	KWPN	238	4	25	558	26	820	16	F	14	11b	yes	vertical	vertical	right	no	0	0	70,92	no
Leipzig	The Longines FEI World Cup Jumping - Leipzig round A	21.01.2018	CSI-W5*	4	1.60	390	5	13	31.03.2005	G	KWPN	238	4	25	558	26	820	16	F	15	12	no	vertical	vertical	right	no	0	1	70,92	no
Leipzig	The Longines FEI World Cup Jumping - Leipzig round A	21.01.2018	CSI-W5*	4	1.60	390	127	10	03.07.2008	M	BWP	131	3	26	14	30	1957	97	M	1	1	no	vertical	vertical	left	no	0	0	68,88	no
Leipzig	The Longines FEI World Cup Jumping - Leipzig round A	21.01.2018	CSI-W5*	4	1.60	390	127	10	03.07.2008	M	BWP	131	3	26	14	30	1957	97	M	2	2	no	spread	oxer	straight	no	0	0	68,88	no
Leipzig	The Longines FEI World Cup Jumping - Leipzig round A	21.01.2018	CSI-W5*	4	1.60	390	127	10	03.07.2008	M	BWP	131	3	26	14	30	1957	97	M	3	3	no	spread	oxer	left	no	0	0	68,88	no
Leipzig	The Longines FEI World Cup Jumping - Leipzig round A	21.01.2018	CSI-W5*	4	1.60	390	127	10	03.07.2008	M	BWP	131	3	26	14	30	1957	97	M	4	4	no	vertical	vertical	right	no	0	0	68,88	no
Leipzig	The Longines FEI World Cup Jumping - Leipzig round A	21.01.2018	CSI-W5*	4	1.60	390	127	10	03.07.2008	M	BWP	131	3	26	14	30	1957	97	M	5	5a	yes	vertical	vertical	right	no	0	0	68,88	no
Leipzig	The Longines FEI World Cup Jumping - Leipzig round A	21.01.2018	CSI-W5*	4	1.60	390	127	10	03.07.2008	M	BWP	131	3	26	14	30	1957	97	M	6	5b	yes	spread	oxer	right	yes	4	0	68,88	no
Leipzig	The Longines FEI World Cup Jumping - Leipzig round A	21.01.2018	CSI-W5*	4	1.60	390	127	10	03.07.2008	M	BWP	131	3	26	14	30	1957	97	M	7	5c	yes	spread	oxer	right	no	0	0	68,88	no
Leipzig	The Longines FEI World Cup Jumping - Leipzig round A	21.01.2018	CSI-W5*	4	1.60	390	127	10	03.07.2008	M	BWP	131	3	26	14	30	1957	97	M	8	6	no	vertical	vertical	left	no	0	0	68,88	no
Leipzig	The Longines FEI World Cup Jumping - Leipzig round A	21.01.2018	CSI-W5*	4	1.60	390	127	10	03.07.2008	M	BWP	131	3	26	14	30	1957	97	M	9	7	no	spread	oxer with water	left	no	0	0	68,88	no
Leipzig	The Longines FEI World Cup Jumping - Leipzig round A	21.01.2018	CSI-W5*	4	1.60	390	127	10	03.07.2008	M	BWP	131	3	26	14	30	1957	97	M	10	8	no	spread	oxer	straight	no	0	0	68,88	no
Leipzig	The Longines FEI World Cup Jumping - Leipzig round A	21.01.2018	CSI-W5*	4	1.60	390	127	10	03.07.2008	M	BWP	131	3	26	14	30	1957	97	M	11	9	no	vertical	vertical	left	no	0	0	68,88	no
Leipzig	The Longines FEI World Cup Jumping - Leipzig round A	21.01.2018	CSI-W5*	4	1.60	390	127	10	03.07.2008	M	BWP	131	3	26	14	30	1957	97	M	12	10	no	spread	oxer	left	no	0	0	68,88	no
Leipzig	The Longines FEI World Cup Jumping - Leipzig round A	21.01.2018	CSI-W5*	4	1.60	390	127	10	03.07.2008	M	BWP	131	3	26	14	30	1957	97	M	13	11a	yes	spread	oxer	right	no	0	0	68,88	no
Leipzig	The Longines FEI World Cup Jumping - Leipzig round A	21.01.2018	CSI-W5*	4	1.60	390	127	10	03.07.2008	M	BWP	131	3	26	14	30	1957	97	M	14	11b	yes	vertical	vertical	right	no	0	0	68,88	no
Leipzig	The Longines FEI World Cup Jumping - Leipzig round A	21.01.2018	CSI-W5*	4	1.60	390	127	10	03.07.2008	M	BWP	131	3	26	14	30	1957	97	M	15	12	no	vertical	vertical	right	no	0	0	68,88	no
Leipzig	The Longines FEI World Cup Jumping - Leipzig round A	21.01.2018	CSI-W5*	4	1.60	390	171	17	10.06.2001	G	SBS	295	13	28	4	38	2170	90	M	1	1	no	vertical	vertical	left	no	0	0	66,49	no
Leipzig	The Longines FEI World Cup Jumping - Leipzig round A	21.01.2018	CSI-W5*	4	1.60	390	171	17	10.06.2001	G	SBS	295	13	28	4	38	2170	90	M	2	2	no	spread	oxer	straight	no	0	0	66,49	no
Leipzig	The Longines FEI World Cup Jumping - Leipzig round A	21.01.2018	CSI-W5*	4	1.60	390	171	17	10.06.2001	G	SBS	295	13	28	4	38	2170	90	M	3	3	no	spread	oxer	left	no	0	0	66,49	no
Leipzig	The Longines FEI World Cup Jumping - Leipzig round A	21.01.2018	CSI-W5*	4	1.60	390	171	17	10.06.2001	G	SBS	295	13	28	4	38	2170	90	M	4	4	no	vertical	vertical	right	no	0	0	66,49	no
Leipzig	The Longines FEI World Cup Jumping - Leipzig round A	21.01.2018	CSI-W5*	4	1.60	390	171	17	10.06.2001	G	SBS	295	13	28	4	38	2170	90	M	5	5a	yes	vertical	vertical	right	no	0	0	66,49	no
Leipzig	The Longines FEI World Cup Jumping - Leipzig round A	21.01.2018	CSI-W5*	4	1.60	390	171	17	10.06.2001	G	SBS	295	13	28	4	38	2170	90	M	6	5b	yes	spread	oxer	right	no	0	0	66,49	no
Leipzig	The Longines FEI World Cup Jumping - Leipzig round A	21.01.2018	CSI-W5*	4	1.60	390	171	17	10.06.2001	G	SBS	295	13	28	4	38	2170	90	M	7	5c	yes	spread	oxer	right	no	0	0	66,49	no
Leipzig	The Longines FEI World Cup Jumping - Leipzig round A	21.01.2018	CSI-W5*	4	1.60	390	171	17	10.06.2001	G	SBS	295	13	28	4	38	2170	90	M	8	6	no	vertical	vertical	left	no	0	0	66,49	no
Leipzig	The Longines FEI World Cup Jumping - Leipzig round A	21.01.2018	CSI-W5*	4	1.60	390	171	17	10.06.2001	G	SBS	295	13	28	4	38	2170	90	M	9	7	no	spread	oxer with water	left	no	0	0	66,49	no
Leipzig	The Longines FEI World Cup Jumping - Leipzig round A	21.01.2018	CSI-W5*	4	1.60	390	171	17	10.06.2001	G	SBS	295	13	28	4	38	2170	90	M	10	8	no	spread	oxer	straight	no	0	0	66,49	no
Leipzig	The Longines FEI World Cup Jumping - Leipzig round A	21.01.2018	CSI-W5*	4	1.60	390	171	17	10.06.2001	G	SBS	295	13	28	4	38	2170	90	M	11	9	no	vertical	vertical	left	no	0	0	66,49	no
Leipzig	The Longines FEI World Cup Jumping - Leipzig round A	21.01.2018	CSI-W5*	4	1.60	390	171	17	10.06.2001	G	SBS	295	13	28	4	38	2170	90	M	12	10	no	spread	oxer	left	no	0	0	66,49	no
Leipzig	The Longines FEI World Cup Jumping - Leipzig round A	21.01.2018	CSI-W5*	4	1.60	390	171	17	10.06.2001	G	SBS	295	13	28	4	38	2170	90	M	13	11a	yes	spread	oxer	right	no	0	0	66,49	no
Leipzig	The Longines FEI World Cup Jumping - Leipzig round A	21.01.2018	CSI-W5*	4	1.60	390	171	17	10.06.2001	G	SBS	295	13	28	4	38	2170	90	M	14	11b	yes	vertical	vertical	right	no	0	0	66,49	no
Leipzig	The Longines FEI World Cup Jumping - Leipzig round A	21.01.2018	CSI-W5*	4	1.60	390	171	17	10.06.2001	G	SBS	295	13	28	4	38	2170	90	M	15	12	no	vertical	vertical	right	no	0	0	66,49	no
Leipzig	The Longines FEI World Cup Jumping - Leipzig round B	21.01.2018	CSI-W5*	4	1.60	300	171	17	10.06.2001	G	SBS	295	13	28	4	38	2170	90	M	1	1	no	vertical	vertical	left	no	0	0	44,39	no
Leipzig	The Longines FEI World Cup Jumping - Leipzig round B	21.01.2018	CSI-W5*	4	1.60	300	171	17	10.06.2001	G	SBS	295	13	28	4	38	2170	90	M	2	2	no	spread	oxer	left	no	0	0	44,39	no
Leipzig	The Longines FEI World Cup Jumping - Leipzig round B	21.01.2018	CSI-W5*	4	1.60	300	171	17	10.06.2001	G	SBS	295	13	28	4	38	2170	90	M	3	3	no	vertical	vertical	right	no	0	0	44,39	no
Leipzig	The Longines FEI World Cup Jumping - Leipzig round B	21.01.2018	CSI-W5*	4	1.60	300	171	17	10.06.2001	G	SBS	295	13	28	4	38	2170	90	M	4	4a	yes	vertical	vertical	right	no	0	0	44,39	no
Leipzig	The Longines FEI World Cup Jumping - Leipzig round B	21.01.2018	CSI-W5*	4	1.60	300	171	17	10.06.2001	G	SBS	295	13	28	4	38	2170	90	M	5	4b	yes	spread	oxer	right	no	0	0	44,39	no
Leipzig	The Longines FEI World Cup Jumping - Leipzig round B	21.01.2018	CSI-W5*	4	1.60	300	171	17	10.06.2001	G	SBS	295	13	28	4	38	2170	90	M	6	5	no	spread	oxer	left	no	0	0	44,39	no
Leipzig	The Longines FEI World Cup Jumping - Leipzig round B	21.01.2018	CSI-W5*	4	1.60	300	171	17	10.06.2001	G	SBS	295	13	28	4	38	2170	90	M	7	6	no	vertical	vertical	left	no	0	0	44,39	no
Leipzig	The Longines FEI World Cup Jumping - Leipzig round B	21.01.2018	CSI-W5*	4	1.60	300	171	17	10.06.2001	G	SBS	295	13	28	4	38	2170	90	M	8	7	no	vertical	vertical	right	no	0	0	44,39	no
Leipzig	The Longines FEI World Cup Jumping - Leipzig round B	21.01.2018	CSI-W5*	4	1.60	300	171	17	10.06.2001	G	SBS	295	13	28	4	38	2170	90	M	9	8	no	spread	oxer	right	no	0	0	44,39	no
Leipzig	The Longines FEI World Cup Jumping - Leipzig round A	21.01.2018	CSI-W5*	4	1.60	390	35	15	22.05.2003	G	HOLST	368	19	29	89	35	1502	55	M	1	1	no	vertical	vertical	left	no	0	0	69,29	no
Leipzig	The Longines FEI World Cup Jumping - Leipzig round A	21.01.2018	CSI-W5*	4	1.60	390	35	15	22.05.2003	G	HOLST	368	19	29	89	35	1502	55	M	2	2	no	spread	oxer	straight	no	0	0	69,29	no
Leipzig	The Longines FEI World Cup Jumping - Leipzig round A	21.01.2018	CSI-W5*	4	1.60	390	35	15	22.05.2003	G	HOLST	368	19	29	89	35	1502	55	M	3	3	no	spread	oxer	left	no	0	0	69,29	no
Leipzig	The Longines FEI World Cup Jumping - Leipzig round A	21.01.2018	CSI-W5*	4	1.60	390	35	15	22.05.2003	G	HOLST	368	19	29	89	35	1502	55	M	4	4	no	vertical	vertical	right	no	0	0	69,29	no
Leipzig	The Longines FEI World Cup Jumping - Leipzig round A	21.01.2018	CSI-W5*	4	1.60	390	35	15	22.05.2003	G	HOLST	368	19	29	89	35	1502	55	M	5	5a	yes	vertical	vertical	right	no	0	0	69,29	no
Leipzig	The Longines FEI World Cup Jumping - Leipzig round A	21.01.2018	CSI-W5*	4	1.60	390	35	15	22.05.2003	G	HOLST	368	19	29	89	35	1502	55	M	6	5b	yes	spread	oxer	right	no	0	0	69,29	no
Leipzig	The Longines FEI World Cup Jumping - Leipzig round A	21.01.2018	CSI-W5*	4	1.60	390	35	15	22.05.2003	G	HOLST	368	19	29	89	35	1502	55	M	7	5c	yes	spread	oxer	right	no	0	0	69,29	no
Leipzig	The Longines FEI World Cup Jumping - Leipzig round A	21.01.2018	CSI-W5*	4	1.60	390	35	15	22.05.2003	G	HOLST	368	19	29	89	35	1502	55	M	8	6	no	vertical	vertical	left	no	0	0	69,29	no
Leipzig	The Longines FEI World Cup Jumping - Leipzig round A	21.01.2018	CSI-W5*	4	1.60	390	35	15	22.05.2003	G	HOLST	368	19	29	89	35	1502	55	M	9	7	no	spread	oxer with water	left	no	0	0	69,29	no
Leipzig	The Longines FEI World Cup Jumping - Leipzig round A	21.01.2018	CSI-W5*	4	1.60	390	35	15	22.05.2003	G	HOLST	368	19	29	89	35	1502	55	M	10	8	no	spread	oxer	straight	no	0	0	69,29	no
Leipzig	The Longines FEI World Cup Jumping - Leipzig round A	21.01.2018	CSI-W5*	4	1.60	390	35	15	22.05.2003	G	HOLST	368	19	29	89	35	1502	55	M	11	9	no	vertical	vertical	left	yes	4	0	69,29	no
Leipzig	The Longines FEI World Cup Jumping - Leipzig round A	21.01.2018	CSI-W5*	4	1.60	390	35	15	22.05.2003	G	HOLST	368	19	29	89	35	1502	55	M	12	10	no	spread	oxer	left	no	0	0	69,29	no
Leipzig	The Longines FEI World Cup Jumping - Leipzig round A	21.01.2018	CSI-W5*	4	1.60	390	35	15	22.05.2003	G	HOLST	368	19	29	89	35	1502	55	M	13	11a	yes	spread	oxer	right	no	0	0	69,29	no
Leipzig	The Longines FEI World Cup Jumping - Leipzig round A	21.01.2018	CSI-W5*	4	1.60	390	35	15	22.05.2003	G	HOLST	368	19	29	89	35	1502	55	M	14	11b	yes	vertical	vertical	right	no	0	0	69,29	no
Leipzig	The Longines FEI World Cup Jumping - Leipzig round A	21.01.2018	CSI-W5*	4	1.60	390	35	15	22.05.2003	G	HOLST	368	19	29	89	35	1502	55	M	15	12	no	vertical	vertical	right	no	0	0	69,29	no
Leipzig	The Longines FEI World Cup Jumping - Leipzig round A	21.01.2018	CSI-W5*	4	1.60	390	59	15	22.04.2003	M	DSP	185	0	31	167	28	855	19	M	1	1	no	vertical	vertical	left	no	0	0	0,00	yes
Leipzig	The Longines FEI World Cup Jumping - Leipzig round A	21.01.2018	CSI-W5*	4	1.60	390	59	15	22.04.2003	M	DSP	185	0	31	167	28	855	19	M	2	2	no	spread	oxer	straight	no	0	0	0,00	yes
Leipzig	The Longines FEI World Cup Jumping - Leipzig round A	21.01.2018	CSI-W5*	4	1.60	390	59	15	22.04.2003	M	DSP	185	0	31	167	28	855	19	M	3	3	no	spread	oxer	left	no	0	0	0,00	yes
Leipzig	The Longines FEI World Cup Jumping - Leipzig round A	21.01.2018	CSI-W5*	4	1.60	390	59	15	22.04.2003	M	DSP	185	0	31	167	28	855	19	M	4	4	no	vertical	vertical	right	no	0	0	0,00	yes
Leipzig	The Longines FEI World Cup Jumping - Leipzig round A	21.01.2018	CSI-W5*	4	1.60	390	59	15	22.04.2003	M	DSP	185	0	31	167	28	855	19	M	5	5a	yes	vertical	vertical	right	no	0	0	0,00	yes
Leipzig	The Longines FEI World Cup Jumping - Leipzig round A	21.01.2018	CSI-W5*	4	1.60	390	59	15	22.04.2003	M	DSP	185	0	31	167	28	855	19	M	6	5b	yes	spread	oxer	right	no	0	0	0,00	yes
Leipzig	The Longines FEI World Cup Jumping - Leipzig round A	21.01.2018	CSI-W5*	4	1.60	390	59	15	22.04.2003	M	DSP	185	0	31	167	28	855	19	M	7	5c	yes	spread	oxer	right	yes	4	0	0,00	yes
Leipzig	The Longines FEI World Cup Jumping - Leipzig round A	21.01.2018	CSI-W5*	4	1.60	390	59	15	22.04.2003	M	DSP	185	0	31	167	28	855	19	M	8	6	no	vertical	vertical	left	no	0	0	0,00	yes
Leipzig	The Longines FEI World Cup Jumping - Leipzig round A	21.01.2018	CSI-W5*	4	1.60	390	59	15	22.04.2003	M	DSP	185	0	31	167	28	855	19	M	9	7	no	spread	oxer with water	left	no	0	0	0,00	yes
Leipzig	The Longines FEI World Cup Jumping - Leipzig round A	21.01.2018	CSI-W5*	4	1.60	390	59	15	22.04.2003	M	DSP	185	0	31	167	28	855	19	M	10	8	no	spread	oxer	straight	yes	4	0	0,00	yes
Leipzig	The Longines FEI World Cup Jumping - Leipzig round A	21.01.2018	CSI-W5*	4	1.60	390	59	15	22.04.2003	M	DSP	185	0	31	167	28	855	19	M	11	9	no	vertical	vertical	left	no	0	0	0,00	yes
Leipzig	The Longines FEI World Cup Jumping - Leipzig round A	21.01.2018	CSI-W5*	4	1.60	390	59	15	22.04.2003	M	DSP	185	0	31	167	28	855	19	M	12	10	no	spread	oxer	left	yes	4	0	0,00	yes
Leipzig	The Longines FEI World Cup Jumping - Leipzig round A	21.01.2018	CSI-W5*	4	1.60	390	59	15	22.04.2003	M	DSP	185	0	31	167	28	855	19	M	13	11a	yes	spread	oxer	right	no	0	0	0,00	yes
Leipzig	The Longines FEI World Cup Jumping - Leipzig round A	21.01.2018	CSI-W5*	4	1.60	390	59	15	22.04.2003	M	DSP	185	0	31	167	28	855	19	M	14	11b	yes	vertical	vertical	right	no	0	0	0,00	yes
Leipzig	The Longines FEI World Cup Jumping - Leipzig round A	21.01.2018	CSI-W5*	4	1.60	390	59	15	22.04.2003	M	DSP	185	0	31	167	28	855	19	M	15	12	no	vertical	vertical	right	no	0	0	0,00	yes
Leipzig	The Longines FEI World Cup Jumping - Leipzig round A	21.01.2018	CSI-W5*	4	1.60	390	75	14	01.05.2004	G	BWP	198	0	38	10	29	1642	46	M	1	1	no	vertical	vertical	left	no	0	0	69,72	no
Leipzig	The Longines FEI World Cup Jumping - Leipzig round A	21.01.2018	CSI-W5*	4	1.60	390	75	14	01.05.2004	G	BWP	198	0	38	10	29	1642	46	M	2	2	no	spread	oxer	straight	no	0	0	69,72	no
Leipzig	The Longines FEI World Cup Jumping - Leipzig round A	21.01.2018	CSI-W5*	4	1.60	390	75	14	01.05.2004	G	BWP	198	0	38	10	29	1642	46	M	3	3	no	spread	oxer	left	no	0	0	69,72	no
Leipzig	The Longines FEI World Cup Jumping - Leipzig round A	21.01.2018	CSI-W5*	4	1.60	390	75	14	01.05.2004	G	BWP	198	0	38	10	29	1642	46	M	4	4	no	vertical	vertical	right	no	0	0	69,72	no
Leipzig	The Longines FEI World Cup Jumping - Leipzig round A	21.01.2018	CSI-W5*	4	1.60	390	75	14	01.05.2004	G	BWP	198	0	38	10	29	1642	46	M	5	5a	yes	vertical	vertical	right	no	0	0	69,72	no
Leipzig	The Longines FEI World Cup Jumping - Leipzig round A	21.01.2018	CSI-W5*	4	1.60	390	75	14	01.05.2004	G	BWP	198	0	38	10	29	1642	46	M	6	5b	yes	spread	oxer	right	no	0	0	69,72	no
Leipzig	The Longines FEI World Cup Jumping - Leipzig round A	21.01.2018	CSI-W5*	4	1.60	390	75	14	01.05.2004	G	BWP	198	0	38	10	29	1642	46	M	7	5c	yes	spread	oxer	right	no	0	0	69,72	no
Leipzig	The Longines FEI World Cup Jumping - Leipzig round A	21.01.2018	CSI-W5*	4	1.60	390	75	14	01.05.2004	G	BWP	198	0	38	10	29	1642	46	M	8	6	no	vertical	vertical	left	no	0	0	69,72	no
Leipzig	The Longines FEI World Cup Jumping - Leipzig round A	21.01.2018	CSI-W5*	4	1.60	390	75	14	01.05.2004	G	BWP	198	0	38	10	29	1642	46	M	9	7	no	spread	oxer with water	left	no	0	0	69,72	no
Leipzig	The Longines FEI World Cup Jumping - Leipzig round A	21.01.2018	CSI-W5*	4	1.60	390	75	14	01.05.2004	G	BWP	198	0	38	10	29	1642	46	M	10	8	no	spread	oxer	straight	no	0	0	69,72	no
Leipzig	The Longines FEI World Cup Jumping - Leipzig round A	21.01.2018	CSI-W5*	4	1.60	390	75	14	01.05.2004	G	BWP	198	0	38	10	29	1642	46	M	11	9	no	vertical	vertical	left	no	0	0	69,72	no
Leipzig	The Longines FEI World Cup Jumping - Leipzig round A	21.01.2018	CSI-W5*	4	1.60	390	75	14	01.05.2004	G	BWP	198	0	38	10	29	1642	46	M	12	10	no	spread	oxer	left	no	0	0	69,72	no
Leipzig	The Longines FEI World Cup Jumping - Leipzig round A	21.01.2018	CSI-W5*	4	1.60	390	75	14	01.05.2004	G	BWP	198	0	38	10	29	1642	46	M	13	11a	yes	spread	oxer	right	no	0	0	69,72	no
Leipzig	The Longines FEI World Cup Jumping - Leipzig round A	21.01.2018	CSI-W5*	4	1.60	390	75	14	01.05.2004	G	BWP	198	0	38	10	29	1642	46	M	14	11b	yes	vertical	vertical	right	no	0	0	69,72	no
Leipzig	The Longines FEI World Cup Jumping - Leipzig round A	21.01.2018	CSI-W5*	4	1.60	390	75	14	01.05.2004	G	BWP	198	0	38	10	29	1642	46	M	15	12	no	vertical	vertical	right	no	0	0	69,72	no
Leipzig	The Longines FEI World Cup Jumping - Leipzig round B	21.01.2018	CSI-W5*	4	1.60	300	75	14	01.05.2004	G	BWP	198	0	38	10	29	1642	46	M	1	1	no	vertical	vertical	left	no	0	0	44,48	no
Leipzig	The Longines FEI World Cup Jumping - Leipzig round B	21.01.2018	CSI-W5*	4	1.60	300	75	14	01.05.2004	G	BWP	198	0	38	10	29	1642	46	M	2	2	no	spread	oxer	left	no	0	0	44,48	no
Leipzig	The Longines FEI World Cup Jumping - Leipzig round B	21.01.2018	CSI-W5*	4	1.60	300	75	14	01.05.2004	G	BWP	198	0	38	10	29	1642	46	M	3	3	no	vertical	vertical	right	no	0	0	44,48	no
Leipzig	The Longines FEI World Cup Jumping - Leipzig round B	21.01.2018	CSI-W5*	4	1.60	300	75	14	01.05.2004	G	BWP	198	0	38	10	29	1642	46	M	4	4a	yes	vertical	vertical	right	no	0	0	44,48	no
Leipzig	The Longines FEI World Cup Jumping - Leipzig round B	21.01.2018	CSI-W5*	4	1.60	300	75	14	01.05.2004	G	BWP	198	0	38	10	29	1642	46	M	5	4b	yes	spread	oxer	right	no	0	0	44,48	no
Leipzig	The Longines FEI World Cup Jumping - Leipzig round B	21.01.2018	CSI-W5*	4	1.60	300	75	14	01.05.2004	G	BWP	198	0	38	10	29	1642	46	M	6	5	no	spread	oxer	left	no	0	0	44,48	no
Leipzig	The Longines FEI World Cup Jumping - Leipzig round B	21.01.2018	CSI-W5*	4	1.60	300	75	14	01.05.2004	G	BWP	198	0	38	10	29	1642	46	M	7	6	no	vertical	vertical	left	no	0	0	44,48	no
Leipzig	The Longines FEI World Cup Jumping - Leipzig round B	21.01.2018	CSI-W5*	4	1.60	300	75	14	01.05.2004	G	BWP	198	0	38	10	29	1642	46	M	8	7	no	vertical	vertical	right	yes	4	0	44,48	no
Leipzig	The Longines FEI World Cup Jumping - Leipzig round B	21.01.2018	CSI-W5*	4	1.60	300	75	14	01.05.2004	G	BWP	198	0	38	10	29	1642	46	M	9	8	no	spread	oxer	right	no	0	0	44,48	no
Leipzig	The Longines FEI World Cup Jumping - Leipzig round A	21.01.2018	CSI-W5*	4	1.60	390	48	17	01.01.2001	G	WESTF	290	9	39	71	51	2525	123	M	1	1	no	vertical	vertical	left	no	0	0	71,49	no
Leipzig	The Longines FEI World Cup Jumping - Leipzig round A	21.01.2018	CSI-W5*	4	1.60	390	48	17	01.01.2001	G	WESTF	290	9	39	71	51	2525	123	M	2	2	no	spread	oxer	straight	no	0	0	71,49	no
Leipzig	The Longines FEI World Cup Jumping - Leipzig round A	21.01.2018	CSI-W5*	4	1.60	390	48	17	01.01.2001	G	WESTF	290	9	39	71	51	2525	123	M	3	3	no	spread	oxer	left	no	0	0	71,49	no
Leipzig	The Longines FEI World Cup Jumping - Leipzig round A	21.01.2018	CSI-W5*	4	1.60	390	48	17	01.01.2001	G	WESTF	290	9	39	71	51	2525	123	M	4	4	no	vertical	vertical	right	no	0	0	71,49	no
Leipzig	The Longines FEI World Cup Jumping - Leipzig round A	21.01.2018	CSI-W5*	4	1.60	390	48	17	01.01.2001	G	WESTF	290	9	39	71	51	2525	123	M	5	5a	yes	vertical	vertical	right	no	0	0	71,49	no
Leipzig	The Longines FEI World Cup Jumping - Leipzig round A	21.01.2018	CSI-W5*	4	1.60	390	48	17	01.01.2001	G	WESTF	290	9	39	71	51	2525	123	M	6	5b	yes	spread	oxer	right	no	0	0	71,49	no
Leipzig	The Longines FEI World Cup Jumping - Leipzig round A	21.01.2018	CSI-W5*	4	1.60	390	48	17	01.01.2001	G	WESTF	290	9	39	71	51	2525	123	M	7	5c	yes	spread	oxer	right	no	0	0	71,49	no
Leipzig	The Longines FEI World Cup Jumping - Leipzig round A	21.01.2018	CSI-W5*	4	1.60	390	48	17	01.01.2001	G	WESTF	290	9	39	71	51	2525	123	M	8	6	no	vertical	vertical	left	yes	4	0	71,49	no
Leipzig	The Longines FEI World Cup Jumping - Leipzig round A	21.01.2018	CSI-W5*	4	1.60	390	48	17	01.01.2001	G	WESTF	290	9	39	71	51	2525	123	M	9	7	no	spread	oxer with water	left	no	0	0	71,49	no
Leipzig	The Longines FEI World Cup Jumping - Leipzig round A	21.01.2018	CSI-W5*	4	1.60	390	48	17	01.01.2001	G	WESTF	290	9	39	71	51	2525	123	M	10	8	no	spread	oxer	straight	yes	4	0	71,49	no
Leipzig	The Longines FEI World Cup Jumping - Leipzig round A	21.01.2018	CSI-W5*	4	1.60	390	48	17	01.01.2001	G	WESTF	290	9	39	71	51	2525	123	M	11	9	no	vertical	vertical	left	no	0	0	71,49	no
Leipzig	The Longines FEI World Cup Jumping - Leipzig round A	21.01.2018	CSI-W5*	4	1.60	390	48	17	01.01.2001	G	WESTF	290	9	39	71	51	2525	123	M	12	10	no	spread	oxer	left	no	0	0	71,49	no
Leipzig	The Longines FEI World Cup Jumping - Leipzig round A	21.01.2018	CSI-W5*	4	1.60	390	48	17	01.01.2001	G	WESTF	290	9	39	71	51	2525	123	M	13	11a	yes	spread	oxer	right	yes	4	0	71,49	no
Leipzig	The Longines FEI World Cup Jumping - Leipzig round A	21.01.2018	CSI-W5*	4	1.60	390	48	17	01.01.2001	G	WESTF	290	9	39	71	51	2525	123	M	14	11b	yes	vertical	vertical	right	no	0	0	71,49	no
Leipzig	The Longines FEI World Cup Jumping - Leipzig round A	21.01.2018	CSI-W5*	4	1.60	390	48	17	01.01.2001	G	WESTF	290	9	39	71	51	2525	123	M	15	12	no	vertical	vertical	right	no	0	1	71,49	no
Leipzig	The Longines FEI World Cup Jumping - Leipzig round A	21.01.2018	CSI-W5*	4	1.60	390	146	11	20.02.2007	S	WESTF	153	1	41	122	28	897	18	F	1	1	no	vertical	vertical	left	no	0	0	68,57	no
Leipzig	The Longines FEI World Cup Jumping - Leipzig round A	21.01.2018	CSI-W5*	4	1.60	390	146	11	20.02.2007	S	WESTF	153	1	41	122	28	897	18	F	2	2	no	spread	oxer	straight	no	0	0	68,57	no
Leipzig	The Longines FEI World Cup Jumping - Leipzig round A	21.01.2018	CSI-W5*	4	1.60	390	146	11	20.02.2007	S	WESTF	153	1	41	122	28	897	18	F	3	3	no	spread	oxer	left	no	0	0	68,57	no
Leipzig	The Longines FEI World Cup Jumping - Leipzig round A	21.01.2018	CSI-W5*	4	1.60	390	146	11	20.02.2007	S	WESTF	153	1	41	122	28	897	18	F	4	4	no	vertical	vertical	right	no	0	0	68,57	no
Leipzig	The Longines FEI World Cup Jumping - Leipzig round A	21.01.2018	CSI-W5*	4	1.60	390	146	11	20.02.2007	S	WESTF	153	1	41	122	28	897	18	F	5	5a	yes	vertical	vertical	right	no	0	0	68,57	no
Leipzig	The Longines FEI World Cup Jumping - Leipzig round A	21.01.2018	CSI-W5*	4	1.60	390	146	11	20.02.2007	S	WESTF	153	1	41	122	28	897	18	F	6	5b	yes	spread	oxer	right	no	0	0	68,57	no
Leipzig	The Longines FEI World Cup Jumping - Leipzig round A	21.01.2018	CSI-W5*	4	1.60	390	146	11	20.02.2007	S	WESTF	153	1	41	122	28	897	18	F	7	5c	yes	spread	oxer	right	no	0	0	68,57	no
Leipzig	The Longines FEI World Cup Jumping - Leipzig round A	21.01.2018	CSI-W5*	4	1.60	390	146	11	20.02.2007	S	WESTF	153	1	41	122	28	897	18	F	8	6	no	vertical	vertical	left	no	0	0	68,57	no
Leipzig	The Longines FEI World Cup Jumping - Leipzig round A	21.01.2018	CSI-W5*	4	1.60	390	146	11	20.02.2007	S	WESTF	153	1	41	122	28	897	18	F	9	7	no	spread	oxer with water	left	no	0	0	68,57	no
Leipzig	The Longines FEI World Cup Jumping - Leipzig round A	21.01.2018	CSI-W5*	4	1.60	390	146	11	20.02.2007	S	WESTF	153	1	41	122	28	897	18	F	10	8	no	spread	oxer	straight	no	0	0	68,57	no
Leipzig	The Longines FEI World Cup Jumping - Leipzig round A	21.01.2018	CSI-W5*	4	1.60	390	146	11	20.02.2007	S	WESTF	153	1	41	122	28	897	18	F	11	9	no	vertical	vertical	left	no	0	0	68,57	no
Leipzig	The Longines FEI World Cup Jumping - Leipzig round A	21.01.2018	CSI-W5*	4	1.60	390	146	11	20.02.2007	S	WESTF	153	1	41	122	28	897	18	F	12	10	no	spread	oxer	left	no	0	0	68,57	no
Leipzig	The Longines FEI World Cup Jumping - Leipzig round A	21.01.2018	CSI-W5*	4	1.60	390	146	11	20.02.2007	S	WESTF	153	1	41	122	28	897	18	F	13	11a	yes	spread	oxer	right	no	0	0	68,57	no
Leipzig	The Longines FEI World Cup Jumping - Leipzig round A	21.01.2018	CSI-W5*	4	1.60	390	146	11	20.02.2007	S	WESTF	153	1	41	122	28	897	18	F	14	11b	yes	vertical	vertical	right	no	0	0	68,57	no
Leipzig	The Longines FEI World Cup Jumping - Leipzig round A	21.01.2018	CSI-W5*	4	1.60	390	146	11	20.02.2007	S	WESTF	153	1	41	122	28	897	18	F	15	12	no	vertical	vertical	right	no	0	0	68,57	no
Leipzig	The Longines FEI World Cup Jumping - Leipzig round B	21.01.2018	CSI-W5*	4	1.60	300	146	11	20.02.2007	S	WESTF	153	1	41	122	28	897	18	F	1	1	no	vertical	vertical	left	yes	4	0	46,91	no
Leipzig	The Longines FEI World Cup Jumping - Leipzig round B	21.01.2018	CSI-W5*	4	1.60	300	146	11	20.02.2007	S	WESTF	153	1	41	122	28	897	18	F	2	2	no	spread	oxer	left	no	0	0	46,91	no
Leipzig	The Longines FEI World Cup Jumping - Leipzig round B	21.01.2018	CSI-W5*	4	1.60	300	146	11	20.02.2007	S	WESTF	153	1	41	122	28	897	18	F	3	3	no	vertical	vertical	right	no	0	0	46,91	no
Leipzig	The Longines FEI World Cup Jumping - Leipzig round B	21.01.2018	CSI-W5*	4	1.60	300	146	11	20.02.2007	S	WESTF	153	1	41	122	28	897	18	F	4	4a	yes	vertical	vertical	right	no	0	0	46,91	no
Leipzig	The Longines FEI World Cup Jumping - Leipzig round B	21.01.2018	CSI-W5*	4	1.60	300	146	11	20.02.2007	S	WESTF	153	1	41	122	28	897	18	F	5	4b	yes	spread	oxer	right	no	0	0	46,91	no
Leipzig	The Longines FEI World Cup Jumping - Leipzig round B	21.01.2018	CSI-W5*	4	1.60	300	146	11	20.02.2007	S	WESTF	153	1	41	122	28	897	18	F	6	5	no	spread	oxer	left	no	0	0	46,91	no
Leipzig	The Longines FEI World Cup Jumping - Leipzig round B	21.01.2018	CSI-W5*	4	1.60	300	146	11	20.02.2007	S	WESTF	153	1	41	122	28	897	18	F	7	6	no	vertical	vertical	left	no	0	0	46,91	no
Leipzig	The Longines FEI World Cup Jumping - Leipzig round B	21.01.2018	CSI-W5*	4	1.60	300	146	11	20.02.2007	S	WESTF	153	1	41	122	28	897	18	F	8	7	no	vertical	vertical	right	no	0	0	46,91	no
Leipzig	The Longines FEI World Cup Jumping - Leipzig round B	21.01.2018	CSI-W5*	4	1.60	300	146	11	20.02.2007	S	WESTF	153	1	41	122	28	897	18	F	9	8	no	spread	oxer	right	no	0	0	46,91	no
Leipzig	The Longines FEI World Cup Jumping - Leipzig round A	21.01.2018	CSI-W5*	4	1.60	390	208	10	18.05.2008	G	SF	126	1	43	134	48	1808	64	M	1	1	no	vertical	vertical	left	no	0	0	71,06	no
Leipzig	The Longines FEI World Cup Jumping - Leipzig round A	21.01.2018	CSI-W5*	4	1.60	390	208	10	18.05.2008	G	SF	126	1	43	134	48	1808	64	M	2	2	no	spread	oxer	straight	no	0	0	71,06	no
Leipzig	The Longines FEI World Cup Jumping - Leipzig round A	21.01.2018	CSI-W5*	4	1.60	390	208	10	18.05.2008	G	SF	126	1	43	134	48	1808	64	M	3	3	no	spread	oxer	left	no	0	0	71,06	no
Leipzig	The Longines FEI World Cup Jumping - Leipzig round A	21.01.2018	CSI-W5*	4	1.60	390	208	10	18.05.2008	G	SF	126	1	43	134	48	1808	64	M	4	4	no	vertical	vertical	right	no	0	0	71,06	no
Leipzig	The Longines FEI World Cup Jumping - Leipzig round A	21.01.2018	CSI-W5*	4	1.60	390	208	10	18.05.2008	G	SF	126	1	43	134	48	1808	64	M	5	5a	yes	vertical	vertical	right	no	0	0	71,06	no
Leipzig	The Longines FEI World Cup Jumping - Leipzig round A	21.01.2018	CSI-W5*	4	1.60	390	208	10	18.05.2008	G	SF	126	1	43	134	48	1808	64	M	6	5b	yes	spread	oxer	right	no	0	0	71,06	no
Leipzig	The Longines FEI World Cup Jumping - Leipzig round A	21.01.2018	CSI-W5*	4	1.60	390	208	10	18.05.2008	G	SF	126	1	43	134	48	1808	64	M	7	5c	yes	spread	oxer	right	no	0	0	71,06	no
Leipzig	The Longines FEI World Cup Jumping - Leipzig round A	21.01.2018	CSI-W5*	4	1.60	390	208	10	18.05.2008	G	SF	126	1	43	134	48	1808	64	M	8	6	no	vertical	vertical	left	no	0	0	71,06	no
Leipzig	The Longines FEI World Cup Jumping - Leipzig round A	21.01.2018	CSI-W5*	4	1.60	390	208	10	18.05.2008	G	SF	126	1	43	134	48	1808	64	M	9	7	no	spread	oxer with water	left	no	0	0	71,06	no
Leipzig	The Longines FEI World Cup Jumping - Leipzig round A	21.01.2018	CSI-W5*	4	1.60	390	208	10	18.05.2008	G	SF	126	1	43	134	48	1808	64	M	10	8	no	spread	oxer	straight	no	0	0	71,06	no
Leipzig	The Longines FEI World Cup Jumping - Leipzig round A	21.01.2018	CSI-W5*	4	1.60	390	208	10	18.05.2008	G	SF	126	1	43	134	48	1808	64	M	11	9	no	vertical	vertical	left	no	0	0	71,06	no
Leipzig	The Longines FEI World Cup Jumping - Leipzig round A	21.01.2018	CSI-W5*	4	1.60	390	208	10	18.05.2008	G	SF	126	1	43	134	48	1808	64	M	12	10	no	spread	oxer	left	no	0	0	71,06	no
Leipzig	The Longines FEI World Cup Jumping - Leipzig round A	21.01.2018	CSI-W5*	4	1.60	390	208	10	18.05.2008	G	SF	126	1	43	134	48	1808	64	M	13	11a	yes	spread	oxer	right	no	0	0	71,06	no
Leipzig	The Longines FEI World Cup Jumping - Leipzig round A	21.01.2018	CSI-W5*	4	1.60	390	208	10	18.05.2008	G	SF	126	1	43	134	48	1808	64	M	14	11b	yes	vertical	vertical	right	no	0	0	71,06	no
Leipzig	The Longines FEI World Cup Jumping - Leipzig round A	21.01.2018	CSI-W5*	4	1.60	390	208	10	18.05.2008	G	SF	126	1	43	134	48	1808	64	M	15	12	no	vertical	vertical	right	no	0	1	71,06	no
Leipzig	The Longines FEI World Cup Jumping - Leipzig round A	21.01.2018	CSI-W5*	4	1.60	390	36	13	30.05.2005	S	HOLST	223	29	52	52	32	2035	202	M	1	1	no	vertical	vertical	left	no	0	0	70,05	no
Leipzig	The Longines FEI World Cup Jumping - Leipzig round A	21.01.2018	CSI-W5*	4	1.60	390	36	13	30.05.2005	S	HOLST	223	29	52	52	32	2035	202	M	2	2	no	spread	oxer	straight	no	0	0	70,05	no
Leipzig	The Longines FEI World Cup Jumping - Leipzig round A	21.01.2018	CSI-W5*	4	1.60	390	36	13	30.05.2005	S	HOLST	223	29	52	52	32	2035	202	M	3	3	no	spread	oxer	left	no	0	0	70,05	no
Leipzig	The Longines FEI World Cup Jumping - Leipzig round A	21.01.2018	CSI-W5*	4	1.60	390	36	13	30.05.2005	S	HOLST	223	29	52	52	32	2035	202	M	4	4	no	vertical	vertical	right	no	0	0	70,05	no
Leipzig	The Longines FEI World Cup Jumping - Leipzig round A	21.01.2018	CSI-W5*	4	1.60	390	36	13	30.05.2005	S	HOLST	223	29	52	52	32	2035	202	M	5	5a	yes	vertical	vertical	right	no	0	0	70,05	no
Leipzig	The Longines FEI World Cup Jumping - Leipzig round A	21.01.2018	CSI-W5*	4	1.60	390	36	13	30.05.2005	S	HOLST	223	29	52	52	32	2035	202	M	6	5b	yes	spread	oxer	right	no	0	0	70,05	no
Leipzig	The Longines FEI World Cup Jumping - Leipzig round A	21.01.2018	CSI-W5*	4	1.60	390	36	13	30.05.2005	S	HOLST	223	29	52	52	32	2035	202	M	7	5c	yes	spread	oxer	right	yes	4	0	70,05	no
Leipzig	The Longines FEI World Cup Jumping - Leipzig round A	21.01.2018	CSI-W5*	4	1.60	390	36	13	30.05.2005	S	HOLST	223	29	52	52	32	2035	202	M	8	6	no	vertical	vertical	left	no	0	0	70,05	no
Leipzig	The Longines FEI World Cup Jumping - Leipzig round A	21.01.2018	CSI-W5*	4	1.60	390	36	13	30.05.2005	S	HOLST	223	29	52	52	32	2035	202	M	9	7	no	spread	oxer with water	left	yes	4	0	70,05	no
Leipzig	The Longines FEI World Cup Jumping - Leipzig round A	21.01.2018	CSI-W5*	4	1.60	390	36	13	30.05.2005	S	HOLST	223	29	52	52	32	2035	202	M	10	8	no	spread	oxer	straight	no	0	0	70,05	no
Leipzig	The Longines FEI World Cup Jumping - Leipzig round A	21.01.2018	CSI-W5*	4	1.60	390	36	13	30.05.2005	S	HOLST	223	29	52	52	32	2035	202	M	11	9	no	vertical	vertical	left	no	0	0	70,05	no
Leipzig	The Longines FEI World Cup Jumping - Leipzig round A	21.01.2018	CSI-W5*	4	1.60	390	36	13	30.05.2005	S	HOLST	223	29	52	52	32	2035	202	M	12	10	no	spread	oxer	left	no	0	0	70,05	no
Leipzig	The Longines FEI World Cup Jumping - Leipzig round A	21.01.2018	CSI-W5*	4	1.60	390	36	13	30.05.2005	S	HOLST	223	29	52	52	32	2035	202	M	13	11a	yes	spread	oxer	right	no	0	0	70,05	no
Leipzig	The Longines FEI World Cup Jumping - Leipzig round A	21.01.2018	CSI-W5*	4	1.60	390	36	13	30.05.2005	S	HOLST	223	29	52	52	32	2035	202	M	14	11b	yes	vertical	vertical	right	no	0	0	70,05	no
Leipzig	The Longines FEI World Cup Jumping - Leipzig round A	21.01.2018	CSI-W5*	4	1.60	390	36	13	30.05.2005	S	HOLST	223	29	52	52	32	2035	202	M	15	12	no	vertical	vertical	right	no	0	1	70,05	no
Leipzig	The Longines FEI World Cup Jumping - Leipzig round A	21.01.2018	CSI-W5*	4	1.60	390	136	9	01.04.2009	G	BWP	75	0	56	56	44	2054	71	M	1	1	no	vertical	vertical	left	no	0	0	68,93	no
Leipzig	The Longines FEI World Cup Jumping - Leipzig round A	21.01.2018	CSI-W5*	4	1.60	390	136	9	01.04.2009	G	BWP	75	0	56	56	44	2054	71	M	2	2	no	spread	oxer	straight	no	0	0	68,93	no
Leipzig	The Longines FEI World Cup Jumping - Leipzig round A	21.01.2018	CSI-W5*	4	1.60	390	136	9	01.04.2009	G	BWP	75	0	56	56	44	2054	71	M	3	3	no	spread	oxer	left	no	0	0	68,93	no
Leipzig	The Longines FEI World Cup Jumping - Leipzig round A	21.01.2018	CSI-W5*	4	1.60	390	136	9	01.04.2009	G	BWP	75	0	56	56	44	2054	71	M	4	4	no	vertical	vertical	right	no	0	0	68,93	no
Leipzig	The Longines FEI World Cup Jumping - Leipzig round A	21.01.2018	CSI-W5*	4	1.60	390	136	9	01.04.2009	G	BWP	75	0	56	56	44	2054	71	M	5	5a	yes	vertical	vertical	right	no	0	0	68,93	no
Leipzig	The Longines FEI World Cup Jumping - Leipzig round A	21.01.2018	CSI-W5*	4	1.60	390	136	9	01.04.2009	G	BWP	75	0	56	56	44	2054	71	M	6	5b	yes	spread	oxer	right	no	0	0	68,93	no
Leipzig	The Longines FEI World Cup Jumping - Leipzig round A	21.01.2018	CSI-W5*	4	1.60	390	136	9	01.04.2009	G	BWP	75	0	56	56	44	2054	71	M	7	5c	yes	spread	oxer	right	no	0	0	68,93	no
Leipzig	The Longines FEI World Cup Jumping - Leipzig round A	21.01.2018	CSI-W5*	4	1.60	390	136	9	01.04.2009	G	BWP	75	0	56	56	44	2054	71	M	8	6	no	vertical	vertical	left	no	0	0	68,93	no
Leipzig	The Longines FEI World Cup Jumping - Leipzig round A	21.01.2018	CSI-W5*	4	1.60	390	136	9	01.04.2009	G	BWP	75	0	56	56	44	2054	71	M	9	7	no	spread	oxer with water	left	no	0	0	68,93	no
Leipzig	The Longines FEI World Cup Jumping - Leipzig round A	21.01.2018	CSI-W5*	4	1.60	390	136	9	01.04.2009	G	BWP	75	0	56	56	44	2054	71	M	10	8	no	spread	oxer	straight	no	0	0	68,93	no
Leipzig	The Longines FEI World Cup Jumping - Leipzig round A	21.01.2018	CSI-W5*	4	1.60	390	136	9	01.04.2009	G	BWP	75	0	56	56	44	2054	71	M	11	9	no	vertical	vertical	left	no	0	0	68,93	no
Leipzig	The Longines FEI World Cup Jumping - Leipzig round A	21.01.2018	CSI-W5*	4	1.60	390	136	9	01.04.2009	G	BWP	75	0	56	56	44	2054	71	M	12	10	no	spread	oxer	left	no	0	0	68,93	no
Leipzig	The Longines FEI World Cup Jumping - Leipzig round A	21.01.2018	CSI-W5*	4	1.60	390	136	9	01.04.2009	G	BWP	75	0	56	56	44	2054	71	M	13	11a	yes	spread	oxer	right	no	0	0	68,93	no
Leipzig	The Longines FEI World Cup Jumping - Leipzig round A	21.01.2018	CSI-W5*	4	1.60	390	136	9	01.04.2009	G	BWP	75	0	56	56	44	2054	71	M	14	11b	yes	vertical	vertical	right	no	0	0	68,93	no
Leipzig	The Longines FEI World Cup Jumping - Leipzig round A	21.01.2018	CSI-W5*	4	1.60	390	136	9	01.04.2009	G	BWP	75	0	56	56	44	2054	71	M	15	12	no	vertical	vertical	right	no	0	0	68,93	no
Leipzig	The Longines FEI World Cup Jumping - Leipzig round B	21.01.2018	CSI-W5*	4	1.60	300	136	9	01.04.2009	G	BWP	75	0	56	56	44	2054	71	M	1	1	no	vertical	vertical	left	no	0	0	50,11	no
Leipzig	The Longines FEI World Cup Jumping - Leipzig round B	21.01.2018	CSI-W5*	4	1.60	300	136	9	01.04.2009	G	BWP	75	0	56	56	44	2054	71	M	2	2	no	spread	oxer	left	no	0	0	50,11	no
Leipzig	The Longines FEI World Cup Jumping - Leipzig round B	21.01.2018	CSI-W5*	4	1.60	300	136	9	01.04.2009	G	BWP	75	0	56	56	44	2054	71	M	3	3	no	vertical	vertical	right	no	0	0	50,11	no
Leipzig	The Longines FEI World Cup Jumping - Leipzig round B	21.01.2018	CSI-W5*	4	1.60	300	136	9	01.04.2009	G	BWP	75	0	56	56	44	2054	71	M	4	4a	yes	vertical	vertical	right	no	0	0	50,11	no
Leipzig	The Longines FEI World Cup Jumping - Leipzig round B	21.01.2018	CSI-W5*	4	1.60	300	136	9	01.04.2009	G	BWP	75	0	56	56	44	2054	71	M	5	4b	yes	spread	oxer	right	no	0	0	50,11	no
Leipzig	The Longines FEI World Cup Jumping - Leipzig round B	21.01.2018	CSI-W5*	4	1.60	300	136	9	01.04.2009	G	BWP	75	0	56	56	44	2054	71	M	6	5	no	spread	oxer	left	yes	4	0	50,11	no
Leipzig	The Longines FEI World Cup Jumping - Leipzig round B	21.01.2018	CSI-W5*	4	1.60	300	136	9	01.04.2009	G	BWP	75	0	56	56	44	2054	71	M	7	6	no	vertical	vertical	left	no	0	0	50,11	no
Leipzig	The Longines FEI World Cup Jumping - Leipzig round B	21.01.2018	CSI-W5*	4	1.60	300	136	9	01.04.2009	G	BWP	75	0	56	56	44	2054	71	M	8	7	no	vertical	vertical	right	no	0	0	50,11	no
Leipzig	The Longines FEI World Cup Jumping - Leipzig round B	21.01.2018	CSI-W5*	4	1.60	300	136	9	01.04.2009	G	BWP	75	0	56	56	44	2054	71	M	9	8	no	spread	oxer	right	no	0	0	50,11	no
Leipzig	The Longines FEI World Cup Jumping - Leipzig round A	21.01.2018	CSI-W5*	4	1.60	390	194	18	07.04.2000	S	KWPN	239	27	64	20	44	2014	118	M	1	1	no	vertical	vertical	left	no	0	0	67,75	no
Leipzig	The Longines FEI World Cup Jumping - Leipzig round A	21.01.2018	CSI-W5*	4	1.60	390	194	18	07.04.2000	S	KWPN	239	27	64	20	44	2014	118	M	2	2	no	spread	oxer	straight	no	0	0	67,75	no
Leipzig	The Longines FEI World Cup Jumping - Leipzig round A	21.01.2018	CSI-W5*	4	1.60	390	194	18	07.04.2000	S	KWPN	239	27	64	20	44	2014	118	M	3	3	no	spread	oxer	left	no	0	0	67,75	no
Leipzig	The Longines FEI World Cup Jumping - Leipzig round A	21.01.2018	CSI-W5*	4	1.60	390	194	18	07.04.2000	S	KWPN	239	27	64	20	44	2014	118	M	4	4	no	vertical	vertical	right	no	0	0	67,75	no
Leipzig	The Longines FEI World Cup Jumping - Leipzig round A	21.01.2018	CSI-W5*	4	1.60	390	194	18	07.04.2000	S	KWPN	239	27	64	20	44	2014	118	M	5	5a	yes	vertical	vertical	right	no	0	0	67,75	no
Leipzig	The Longines FEI World Cup Jumping - Leipzig round A	21.01.2018	CSI-W5*	4	1.60	390	194	18	07.04.2000	S	KWPN	239	27	64	20	44	2014	118	M	6	5b	yes	spread	oxer	right	no	0	0	67,75	no
Leipzig	The Longines FEI World Cup Jumping - Leipzig round A	21.01.2018	CSI-W5*	4	1.60	390	194	18	07.04.2000	S	KWPN	239	27	64	20	44	2014	118	M	7	5c	yes	spread	oxer	right	no	0	0	67,75	no
Leipzig	The Longines FEI World Cup Jumping - Leipzig round A	21.01.2018	CSI-W5*	4	1.60	390	194	18	07.04.2000	S	KWPN	239	27	64	20	44	2014	118	M	8	6	no	vertical	vertical	left	no	0	0	67,75	no
Leipzig	The Longines FEI World Cup Jumping - Leipzig round A	21.01.2018	CSI-W5*	4	1.60	390	194	18	07.04.2000	S	KWPN	239	27	64	20	44	2014	118	M	9	7	no	spread	oxer with water	left	no	0	0	67,75	no
Leipzig	The Longines FEI World Cup Jumping - Leipzig round A	21.01.2018	CSI-W5*	4	1.60	390	194	18	07.04.2000	S	KWPN	239	27	64	20	44	2014	118	M	10	8	no	spread	oxer	straight	no	0	0	67,75	no
Leipzig	The Longines FEI World Cup Jumping - Leipzig round A	21.01.2018	CSI-W5*	4	1.60	390	194	18	07.04.2000	S	KWPN	239	27	64	20	44	2014	118	M	11	9	no	vertical	vertical	left	no	0	0	67,75	no
Leipzig	The Longines FEI World Cup Jumping - Leipzig round A	21.01.2018	CSI-W5*	4	1.60	390	194	18	07.04.2000	S	KWPN	239	27	64	20	44	2014	118	M	12	10	no	spread	oxer	left	no	0	0	67,75	no
Leipzig	The Longines FEI World Cup Jumping - Leipzig round A	21.01.2018	CSI-W5*	4	1.60	390	194	18	07.04.2000	S	KWPN	239	27	64	20	44	2014	118	M	13	11a	yes	spread	oxer	right	no	0	0	67,75	no
Leipzig	The Longines FEI World Cup Jumping - Leipzig round A	21.01.2018	CSI-W5*	4	1.60	390	194	18	07.04.2000	S	KWPN	239	27	64	20	44	2014	118	M	14	11b	yes	vertical	vertical	right	no	0	0	67,75	no
Leipzig	The Longines FEI World Cup Jumping - Leipzig round A	21.01.2018	CSI-W5*	4	1.60	390	194	18	07.04.2000	S	KWPN	239	27	64	20	44	2014	118	M	15	12	no	vertical	vertical	right	no	0	0	67,75	no
Leipzig	The Longines FEI World Cup Jumping - Leipzig round B	21.01.2018	CSI-W5*	4	1.60	300	194	18	07.04.2000	S	KWPN	239	27	64	20	44	2014	118	M	1	1	no	vertical	vertical	left	no	0	0	43,19	no

Leipzig	The Longines FEI World Cup Jumping - Leipzig round B	21.01.2018	CSI-W5*	4	1.60	300	194	18	07.04.2000	S	KWPN	239	27	64	20	44	2014	118	M	2	2	no	spread	oxer	left	no	0	0	43,19	no
Leipzig	The Longines FEI World Cup Jumping - Leipzig round B	21.01.2018	CSI-W5*	4	1.60	300	194	18	07.04.2000	S	KWPN	239	27	64	20	44	2014	118	M	3	3	no	vertical	vertical	right	no	0	0	43,19	no
Leipzig	The Longines FEI World Cup Jumping - Leipzig round B	21.01.2018	CSI-W5*	4	1.60	300	194	18	07.04.2000	S	KWPN	239	27	64	20	44	2014	118	M	4	4a	yes	vertical	vertical	right	no	0	0	43,19	no
Leipzig	The Longines FEI World Cup Jumping - Leipzig round B	21.01.2018	CSI-W5*	4	1.60	300	194	18	07.04.2000	S	KWPN	239	27	64	20	44	2014	118	M	5	4b	yes	spread	oxer	right	no	0	0	43,19	no
Leipzig	The Longines FEI World Cup Jumping - Leipzig round B	21.01.2018	CSI-W5*	4	1.60	300	194	18	07.04.2000	S	KWPN	239	27	64	20	44	2014	118	M	6	5	no	spread	oxer	left	no	0	0	43,19	no
Leipzig	The Longines FEI World Cup Jumping - Leipzig round B	21.01.2018	CSI-W5*	4	1.60	300	194	18	07.04.2000	S	KWPN	239	27	64	20	44	2014	118	M	7	6	no	vertical	vertical	left	no	0	0	43,19	no
Leipzig	The Longines FEI World Cup Jumping - Leipzig round B	21.01.2018	CSI-W5*	4	1.60	300	194	18	07.04.2000	S	KWPN	239	27	64	20	44	2014	118	M	8	7	no	vertical	vertical	right	no	0	0	43,19	no
Leipzig	The Longines FEI World Cup Jumping - Leipzig round B	21.01.2018	CSI-W5*	4	1.60	300	194	18	07.04.2000	S	KWPN	239	27	64	20	44	2014	118	M	9	8	no	spread	oxer	right	no	0	0	43,19	no
Leipzig	The Longines FEI World Cup Jumping - Leipzig round A	21.01.2018	CSI-W5*	4	1.60	390	71	10	25.05.2008	M	KWPN	131	2	65	70	43	1682	26	M	1	1	no	vertical	vertical	left	no	0	0	67,25	no
Leipzig	The Longines FEI World Cup Jumping - Leipzig round A	21.01.2018	CSI-W5*	4	1.60	390	71	10	25.05.2008	M	KWPN	131	2	65	70	43	1682	26	M	2	2	no	spread	oxer	straight	no	0	0	67,25	no
Leipzig	The Longines FEI World Cup Jumping - Leipzig round A	21.01.2018	CSI-W5*	4	1.60	390	71	10	25.05.2008	M	KWPN	131	2	65	70	43	1682	26	M	3	3	no	spread	oxer	left	no	0	0	67,25	no
Leipzig	The Longines FEI World Cup Jumping - Leipzig round A	21.01.2018	CSI-W5*	4	1.60	390	71	10	25.05.2008	M	KWPN	131	2	65	70	43	1682	26	M	4	4	no	vertical	vertical	right	no	0	0	67,25	no
Leipzig	The Longines FEI World Cup Jumping - Leipzig round A	21.01.2018	CSI-W5*	4	1.60	390	71	10	25.05.2008	M	KWPN	131	2	65	70	43	1682	26	M	5	5a	yes	vertical	vertical	right	no	0	0	67,25	no
Leipzig	The Longines FEI World Cup Jumping - Leipzig round A	21.01.2018	CSI-W5*	4	1.60	390	71	10	25.05.2008	M	KWPN	131	2	65	70	43	1682	26	M	6	5b	yes	spread	oxer	right	no	0	0	67,25	no
Leipzig	The Longines FEI World Cup Jumping - Leipzig round A	21.01.2018	CSI-W5*	4	1.60	390	71	10	25.05.2008	M	KWPN	131	2	65	70	43	1682	26	M	7	5c	yes	spread	oxer	right	no	0	0	67,25	no
Leipzig	The Longines FEI World Cup Jumping - Leipzig round A	21.01.2018	CSI-W5*	4	1.60	390	71	10	25.05.2008	M	KWPN	131	2	65	70	43	1682	26	M	8	6	no	vertical	vertical	left	no	0	0	67,25	no
Leipzig	The Longines FEI World Cup Jumping - Leipzig round A	21.01.2018	CSI-W5*	4	1.60	390	71	10	25.05.2008	M	KWPN	131	2	65	70	43	1682	26	M	9	7	no	spread	oxer with water	left	no	0	0	67,25	no
Leipzig	The Longines FEI World Cup Jumping - Leipzig round A	21.01.2018	CSI-W5*	4	1.60	390	71	10	25.05.2008	M	KWPN	131	2	65	70	43	1682	26	M	10	8	no	spread	oxer	straight	no	0	0	67,25	no
Leipzig	The Longines FEI World Cup Jumping - Leipzig round A	21.01.2018	CSI-W5*	4	1.60	390	71	10	25.05.2008	M	KWPN	131	2	65	70	43	1682	26	M	11	9	no	vertical	vertical	left	no	0	0	67,25	no
Leipzig	The Longines FEI World Cup Jumping - Leipzig round A	21.01.2018	CSI-W5*	4	1.60	390	71	10	25.05.2008	M	KWPN	131	2	65	70	43	1682	26	M	12	10	no	spread	oxer	left	no	0	0	67,25	no
Leipzig	The Longines FEI World Cup Jumping - Leipzig round A	21.01.2018	CSI-W5*	4	1.60	390	71	10	25.05.2008	M	KWPN	131	2	65	70	43	1682	26	M	13	11a	yes	spread	oxer	right	no	0	0	67,25	no
Leipzig	The Longines FEI World Cup Jumping - Leipzig round A	21.01.2018	CSI-W5*	4	1.60	390	71	10	25.05.2008	M	KWPN	131	2	65	70	43	1682	26	M	14	11b	yes	vertical	vertical	right	no	0	0	67,25	no
Leipzig	The Longines FEI World Cup Jumping - Leipzig round A	21.01.2018	CSI-W5*	4	1.60	390	71	10	25.05.2008	M	KWPN	131	2	65	70	43	1682	26	M	15	12	no	vertical	vertical	right	no	0	0	67,25	no
Leipzig	The Longines FEI World Cup Jumping - Leipzig round B	21.01.2018	CSI-W5*	4	1.60	300	71	10	25.05.2008	M	KWPN	131	2	65	70	43	1682	26	M	1	1	no	vertical	vertical	left	no	0	0	46,76	no
Leipzig	The Longines FEI World Cup Jumping - Leipzig round B	21.01.2018	CSI-W5*	4	1.60	300	71	10	25.05.2008	M	KWPN	131	2	65	70	43	1682	26	M	2	2	no	spread	oxer	left	no	0	0	46,76	no
Leipzig	The Longines FEI World Cup Jumping - Leipzig round B	21.01.2018	CSI-W5*	4	1.60	300	71	10	25.05.2008	M	KWPN	131	2	65	70	43	1682	26	M	3	3	no	vertical	vertical	right	no	0	0	46,76	no
Leipzig	The Longines FEI World Cup Jumping - Leipzig round B	21.01.2018	CSI-W5*	4	1.60	300	71	10	25.05.2008	M	KWPN	131	2	65	70	43	1682	26	M	4	4a	yes	vertical	vertical	right	no	0	0	46,76	no
Leipzig	The Longines FEI World Cup Jumping - Leipzig round B	21.01.2018	CSI-W5*	4	1.60	300	71	10	25.05.2008	M	KWPN	131	2	65	70	43	1682	26	M	5	4b	yes	spread	oxer	right	no	0	0	46,76	no
Leipzig	The Longines FEI World Cup Jumping - Leipzig round B	21.01.2018	CSI-W5*	4	1.60	300	71	10	25.05.2008	M	KWPN	131	2	65	70	43	1682	26	M	6	5	no	spread	oxer	left	no	0	0	46,76	no
Leipzig	The Longines FEI World Cup Jumping - Leipzig round B	21.01.2018	CSI-W5*	4	1.60	300	71	10	25.05.2008	M	KWPN	131	2	65	70	43	1682	26	M	7	6	no	vertical	vertical	left	no	0	0	46,76	no
Leipzig	The Longines FEI World Cup Jumping - Leipzig round B	21.01.2018	CSI-W5*	4	1.60	300	71	10	25.05.2008	M	KWPN	131	2	65	70	43	1682	26	M	8	7	no	vertical	vertical	right	yes	4	0	46,76	no
Leipzig	The Longines FEI World Cup Jumping - Leipzig round B	21.01.2018	CSI-W5*	4	1.60	300	71	10	25.05.2008	M	KWPN	131	2	65	70	43	1682	26	M	9	8	no	spread	oxer	right	no	0	0	46,76	no
Leipzig	The Longines FEI World Cup Jumping - Leipzig round A	21.01.2018	CSI-W5*	4	1.60	390	188	11	06.06.2007	G	KWPN	209	10	67	60	47	2033	59	M	1	1	no	vertical	vertical	left	no	0	0	71,26	no
Leipzig	The Longines FEI World Cup Jumping - Leipzig round A	21.01.2018	CSI-W5*	4	1.60	390	188	11	06.06.2007	G	KWPN	209	10	67	60	47	2033	59	M	2	2	no	spread	oxer	straight	no	0	0	71,26	no
Leipzig	The Longines FEI World Cup Jumping - Leipzig round A	21.01.2018	CSI-W5*	4	1.60	390	188	11	06.06.2007	G	KWPN	209	10	67	60	47	2033	59	M	3	3	no	spread	oxer	left	no	0	0	71,26	no
Leipzig	The Longines FEI World Cup Jumping - Leipzig round A	21.01.2018	CSI-W5*	4	1.60	390	188	11	06.06.2007	G	KWPN	209	10	67	60	47	2033	59	M	4	4	no	vertical	vertical	right	no	0	0	71,26	no
Leipzig	The Longines FEI World Cup Jumping - Leipzig round A	21.01.2018	CSI-W5*	4	1.60	390	188	11	06.06.2007	G	KWPN	209	10	67	60	47	2033	59	M	5	5a	yes	vertical	vertical	right	no	0	0	71,26	no
Leipzig	The Longines FEI World Cup Jumping - Leipzig round A	21.01.2018	CSI-W5*	4	1.60	390	188	11	06.06.2007	G	KWPN	209	10	67	60	47	2033	59	M	6	5b	yes	spread	oxer	right	no	0	0	71,26	no
Leipzig	The Longines FEI World Cup Jumping - Leipzig round A	21.01.2018	CSI-W5*	4	1.60	390	188	11	06.06.2007	G	KWPN	209	10	67	60	47	2033	59	M	7	5c	yes	spread	oxer	right	no	0	0	71,26	no
Leipzig	The Longines FEI World Cup Jumping - Leipzig round A	21.01.2018	CSI-W5*	4	1.60	390	188	11	06.06.2007	G	KWPN	209	10	67	60	47	2033	59	M	8	6	no	vertical	vertical	left	yes	4	0	71,26	no
Leipzig	The Longines FEI World Cup Jumping - Leipzig round A	21.01.2018	CSI-W5*	4	1.60	390	188	11	06.06.2007	G	KWPN	209	10	67	60	47	2033	59	M	9	7	no	spread	oxer with water	left	yes	4	0	71,26	no
Leipzig	The Longines FEI World Cup Jumping - Leipzig round A	21.01.2018	CSI-W5*	4	1.60	390	188	11	06.06.2007	G	KWPN	209	10	67	60	47	2033	59	M	10	8	no	spread	oxer	straight	no	0	0	71,26	no
Leipzig	The Longines FEI World Cup Jumping - Leipzig round A	21.01.2018	CSI-W5*	4	1.60	390	188	11	06.06.2007	G	KWPN	209	10	67	60	47	2033	59	M	11	9	no	vertical	vertical	left	no	0	0	71,26	no
Leipzig	The Longines FEI World Cup Jumping - Leipzig round A	21.01.2018	CSI-W5*	4	1.60	390	188	11	06.06.2007	G	KWPN	209	10	67	60	47	2033	59	M	12	10	no	spread	oxer	left	no	0	0	71,26	no
Leipzig	The Longines FEI World Cup Jumping - Leipzig round A	21.01.2018	CSI-W5*	4	1.60	390	188	11	06.06.2007	G	KWPN	209	10	67	60	47	2033	59	M	13	11a	yes	spread	oxer	right	no	0	0	71,26	no
Leipzig	The Longines FEI World Cup Jumping - Leipzig round A	21.01.2018	CSI-W5*	4	1.60	390	188	11	06.06.2007	G	KWPN	209	10	67	60	47	2033	59	M	14	11b	yes	vertical	vertical	right	no	0	0	71,26	no
Leipzig	The Longines FEI World Cup Jumping - Leipzig round A	21.01.2018	CSI-W5*	4	1.60	390	188	11	06.06.2007	G	KWPN	209	10	67	60	47	2033	59	M	15	12	no	vertical	vertical	right	no	0	1	71,26	no
Leipzig	The Longines FEI World Cup Jumping - Leipzig round A	21.01.2018	CSI-W5*	4	1.60	390	45	13	20.04.2005	S	WESTF	126	6	71	12	44	2177	93	M	1	1	no	vertical	vertical	left	no	0	0	69,16	no
Leipzig	The Longines FEI World Cup Jumping - Leipzig round A	21.01.2018	CSI-W5*	4	1.60	390	45	13	20.04.2005	S	WESTF	126	6	71	12	44	2177	93	M	2	2	no	spread	oxer	straight	no	0	0	69,16	no
Leipzig	The Longines FEI World Cup Jumping - Leipzig round A	21.01.2018	CSI-W5*	4	1.60	390	45	13	20.04.2005	S	WESTF	126	6	71	12	44	2177	93	M	3	3	no	spread	oxer	left	no	0	0	69,16	no
Leipzig	The Longines FEI World Cup Jumping - Leipzig round A	21.01.2018	CSI-W5*	4	1.60	390	45	13	20.04.2005	S	WESTF	126	6	71	12	44	2177	93	M	4	4	no	vertical	vertical	right	no	0	0	69,16	no
Leipzig	The Longines FEI World Cup Jumping - Leipzig round A	21.01.2018	CSI-W5*	4	1.60	390	45	13	20.04.2005	S	WESTF	126	6	71	12	44	2177	93	M	5	5a	yes	vertical	vertical	right	no	0	0	69,16	no
Leipzig	The Longines FEI World Cup Jumping - Leipzig round A	21.01.2018	CSI-W5*	4	1.60	390	45	13	20.04.2005	S	WESTF	126	6	71	12	44	2177	93	M	6	5b	yes	spread	oxer	right	no	0	0	69,16	no
Leipzig	The Longines FEI World Cup Jumping - Leipzig round A	21.01.2018	CSI-W5*	4	1.60	390	45	13	20.04.2005	S	WESTF	126	6	71	12	44	2177	93	M	7	5c	yes	spread	oxer	right	no	0	0	69,16	no
Leipzig	The Longines FEI World Cup Jumping - Leipzig round A	21.01.2018	CSI-W5*	4	1.60	390	45	13	20.04.2005	S	WESTF	126	6	71	12	44	2177	93	M	8	6	no	vertical	vertical	left	no	0	0	69,16	no
Leipzig	The Longines FEI World Cup Jumping - Leipzig round A	21.01.2018	CSI-W5*	4	1.60	390	45	13	20.04.2005	S	WESTF	126	6	71	12	44	2177	93	M	9	7	no	spread	oxer with water	left	no	0	0	69,16	no
Leipzig	The Longines FEI World Cup Jumping - Leipzig round A	21.01.2018	CSI-W5*	4	1.60	390	45	13	20.04.2005	S	WESTF	126	6	71	12	44	2177	93	M	10	8	no	spread	oxer	straight	no	0	0	69,16	no
Leipzig	The Longines FEI World Cup Jumping - Leipzig round A	21.01.2018	CSI-W5*	4	1.60	390	45	13	20.04.2005	S	WESTF	126	6	71	12	44	2177	93	M	11	9	no	vertical	vertical	left	no	0	0	69,16	no
Leipzig	The Longines FEI World Cup Jumping - Leipzig round A	21.01.2018	CSI-W5*	4	1.60	390	45	13	20.04.2005	S	WESTF	126	6	71	12	44	2177	93	M	12	10	no	spread	oxer	left	no	0	0	69,16	no
Leipzig	The Longines FEI World Cup Jumping - Leipzig round A	21.01.2018	CSI-W5*	4	1.60	390	45	13	20.04.2005	S	WESTF	126	6	71	12	44	2177	93	M	13	11a	yes	spread	oxer	right	no	0	0	69,16	no
Leipzig	The Longines FEI World Cup Jumping - Leipzig round A	21.01.2018	CSI-W5*	4	1.60	390	45	13	20.04.2005	S	WESTF	126	6	71	12	44	2177	93	M	14	11b	yes	vertical	vertical	right	no	0	0	69,16	no
Leipzig	The Longines FEI World Cup Jumping - Leipzig round A	21.01.2018	CSI-W5*	4	1.60	390	45	13	20.04.2005	S	WESTF	126	6	71	12	44	2177	93	M	15	12	no	vertical	vertical	right	no	0	0	69,16	no
Leipzig	The Longines FEI World Cup Jumping - Leipzig round B	21.01.2018	CSI-W5*	4	1.60	300	45	13	20.04.2005	S	WESTF	126	6	71	12	44	2177	93	M	1	1	no	vertical	vertical	left	no	0	0	44,29	no
Leipzig	The Longines FEI World Cup Jumping - Leipzig round B	21.01.2018	CSI-W5*	4	1.60	300	45	13	20.04.2005	S	WESTF	126	6	71	12	44	2177	93	M	2	2	no	spread	oxer	left	no	0	0	44,29	no
Leipzig	The Longines FEI World Cup Jumping - Leipzig round B	21.01.2018	CSI-W5*	4	1.60	300	45	13	20.04.2005	S	WESTF	126	6	71	12	44	2177	93	M	3	3	no	vertical	vertical	right	no	0	0	44,29	no
Leipzig	The Longines FEI World Cup Jumping - Leipzig round B	21.01.2018	CSI-W5*	4	1.60	300	45	13	20.04.2005	S	WESTF	126	6	71	12	44	2177	93	M	4	4a	yes	vertical	vertical	right	no	0	0	44,29	no
Leipzig	The Longines FEI World Cup Jumping - Leipzig round B	21.01.2018	CSI-W5*	4	1.60	300	45	13	20.04.2005	S	WESTF	126	6	71	12	44	2177	93	M	5	4b	yes	spread	oxer	right	no	0	0	44,29	no
Leipzig	The Longines FEI World Cup Jumping - Leipzig round B	21.01.2018	CSI-W5*	4	1.60	300	45	13	20.04.2005	S	WESTF	126	6	71	12	44	2177	93	M	6	5	no	spread	oxer	left	no	0	0	44,29	no
Leipzig	The Longines FEI World Cup Jumping - Leipzig round B	21.01.2018	CSI-W5*	4	1.60	300	45	13	20.04.2005	S	WESTF	126	6	71	12	44	2177	93	M	7	6	no	vertical	vertical	left	no	0	0	44,29	no
Leipzig	The Longines FEI World Cup Jumping - Leipzig round B	21.01.2018	CSI-W5*	4	1.60	300	45	13	20.04.2005	S	WESTF	126	6	71	12	44	2177	93	M	8	7	no	vertical	vertical	right	no	0	0	44,29	no
Leipzig	The Longines FEI World Cup Jumping - Leipzig round B	21.01.2018	CSI-W5*	4	1.60	300	45	13	20.04.2005	S	WESTF	126	6	71	12	44	2177	93	M	9	8	no	spread	oxer	right	no	0	0	44,29	no
Leipzig	The Longines FEI World Cup Jumping - Leipzig round A	21.01.2018	CSI-W5*	4	1.60	390	173	13	14.07.2005	G	SF	229	2	74	65	34	1260	61	M	1	1	no	vertical	vertical	left	no	0	0	68,34	no
Leipzig	The Longines FEI World Cup Jumping - Leipzig round A	21.01.2018	CSI-W5*	4	1.60	390	173	13	14.07.2005	G	SF	229	2	74	65	34	1260	61	M	2	2	no	spread	oxer	straight	no	0	0	68,34	no
Leipzig	The Longines FEI World Cup Jumping - Leipzig round A	21.01.2018	CSI-W5*	4	1.60	390	173	13	14.07.2005	G	SF	229	2	74	65	34	1260	61	M	3	3	no	spread	oxer	left	no	0	0	68,34	no
Leipzig	The Longines FEI World Cup Jumping - Leipzig round A	21.01.2018	CSI-W5*	4	1.60	390	173	13	14.07.2005	G	SF	229	2	74	65	34	1260	61	M	4	4	no	vertical	vertical	right	no	0	0	68,34	no
Leipzig	The Longines FEI World Cup Jumping - Leipzig round A	21.01.2018	CSI-W5*	4	1.60	390	173	13	14.07.2005	G	SF	229	2	74	65	34	1260	61	M	5	5a	yes	vertical	vertical	right	no	0	0	68,34	no
Leipzig	The Longines FEI World Cup Jumping - Leipzig round A	21.01.2018	CSI-W5*	4	1.60	390	173	13	14.07.2005	G	SF	229	2	74	65	34	1260	61	M	6	5b	yes	spread	oxer	right	no	0	0	68,34	no
Leipzig	The Longines FEI World Cup Jumping - Leipzig round A	21.01.2018	CSI-W5*	4	1.60	390	173	13	14.07.2005	G	SF	229	2	74	65	34	1260	61	M	7	5c	yes	spread	oxer	right	no	0	0	68,34	no
Leipzig	The Longines FEI World Cup Jumping - Leipzig round A	21.01.2018	CSI-W5*	4	1.60	390	173	13	14.07.2005	G	SF	229	2	74	65	34	1260	61	M	8	6	no	vertical	vertical	left	no	0	0	68,34	no
Leipzig	The Longines FEI World Cup Jumping - Leipzig round A	21.01.2018	CSI-W5*	4	1.60	390	173	13	14.07.2005	G	SF	229	2	74	65	34	1260	61	M	9	7	no	spread	oxer with water	left	no	0	0	68,34	no
Leipzig	The Longines FEI World Cup Jumping - Leipzig round A	21.01.2018	CSI-W5*	4	1.60	390	173	13	14.07.2005	G	SF	229	2	74	65	34	1260	61	M	10	8	no	spread	oxer	straight	no	0	0	68,34	no
Leipzig	The Longines FEI World Cup Jumping - Leipzig round A	21.01.2018	CSI-W5*	4	1.60	390	173	13	14.07.2005	G	SF	229	2	74	65	34	1260	61	M	11	9	no	vertical	vertical	left	no	0	0	68,34	no
Leipzig	The Longines FEI World Cup Jumping - Leipzig round A	21.01.2018	CSI-W5*	4	1.60	390	173	13	14.07.2005	G	SF	229	2	74	65	34	1260	61	M	12	10	no	spread	oxer	left	no	0	0	68,34	no
Leipzig	The Longines FEI World Cup Jumping - Leipzig round A	21.01.2018	CSI-W5*	4	1.60	390	173	13	14.07.2005	G	SF	229	2	74	65	34	1260	61	M	13	11a	yes	spread	oxer	right	no	0	0	68,34	no
Leipzig	The Longines FEI World Cup Jumping - Leipzig round A	21.01.2018	CSI-W5*	4	1.60	390	173	13	14.07.2005	G	SF	229	2	74	65	34	1260	61	M	14	11b	yes	vertical	vertical	right	yes	4	0	68,34	no
Leipzig	The Longines FEI World Cup Jumping - Leipzig round A	21.01.2018	CSI-W5*	4	1.60	390	173	13	14.07.2005	G	SF	229	2	74	65	34	1260	61	M	15	12	no	vertical	vertical	right	no	0	0	68,34	no
Leipzig	The Longines FEI World Cup Jumping - Leipzig round A	21.01.2018	CSI-W5*	4	1.60	390	120	11	28.04.2007	S	HOLST	147	1	76	55	44	1886	48	M	1	1	no	vertical	vertical	left	no	0	0	68,98	no
Leipzig	The Longines FEI World Cup Jumping - Leipzig round A	21.01.2018	CSI-W5*	4	1.60	390	120	11	28.04.2007	S	HOLST	147	1	76	55	44	1886	48	M	2	2	no	spread	oxer	straight	no	0	0	68,98	no
Leipzig	The Longines FEI World Cup Jumping - Leipzig round A	21.01.2018	CSI-W5*	4	1.60	390	120	11	28.04.2007	S	HOLST	147	1	76	55	44	1886	48	M	3	3	no	spread	oxer	left	no	0	0	68,98	no
Leipzig	The Longines FEI World Cup Jumping - Leipzig round A	21.01.2018	CSI-W5*	4	1.60	390	120	11	28.04.2007	S	HOLST	147	1	76	55	44	1886	48	M	4	4	no	vertical	vertical	right	no	0	0	68,98	no
Leipzig	The Longines FEI World Cup Jumping - Leipzig round A	21.01.2018	CSI-W5*	4	1.60	390	120	11	28.04.2007	S	HOLST	147	1	76	55	44	1886	48	M	5	5a	yes	vertical	vertical	right	no	0	0	68,98	no
Leipzig	The Longines FEI World Cup Jumping - Leipzig round A	21.01.2018	CSI-W5*	4	1.60	390	120	11	28.04.2007	S	HOLST	147	1	76	55	44	1886	48	M	6	5b	yes	spread	oxer	right	no	0	0	68,98	no
Leipzig	The Longines FEI World Cup Jumping - Leipzig round A	21.01.2018	CSI-W5*	4	1.60	390	120	11	28.04.2007	S	HOLST	147	1	76	55	44	1886	48	M	7	5c	yes	spread	oxer	right	no	0	0	68,98	no
Leipzig	The Longines FEI World Cup Jumping - Leipzig round A	21.01.2018	CSI-W5*	4	1.60	390	120	11	28.04.2007	S	HOLST	147	1	76	55	44	1886	48	M	8	6	no	vertical	vertical	left	no	0	0	68,98	no
Leipzig	The Longines FEI World Cup Jumping - Leipzig round A	21.01.2018	CSI-W5*	4	1.60	390	120	11	28.04.2007	S	HOLST	147	1	76	55	44	1886	48	M	9	7	no	spread	oxer with water	left	no	0	0	68,98	no
Leipzig	The Longines FEI World Cup Jumping - Leipzig round A	21.01.2018	CSI-W5*	4	1.60	390	120	11	28.04.2007	S	HOLST	147	1	76	55	44	1886	48	M	10	8	no	spread	oxer	straight	no	0	0	68,98	no
Leipzig	The Longines FEI World Cup Jumping - Leipzig round A	21.01.2018	CSI-W5*	4	1.60	390	120	11	28.04.2007	S	HOLST	147	1	76	55	44	1886	48	M	11	9	no	vertical	vertical	left	no	0	0	68,98	no
Leipzig	The Longines FEI World Cup Jumping - Leipzig round A	21.01.2018	CSI-W5*	4	1.60	390	120	11	28.04.2007	S	HOLST	147	1	76	55	44	1886	48	M	12	10	no	spread	oxer	left	no	0	0	68,98	no
Leipzig	The Longines FEI World Cup Jumping - Leipzig round A	21.01.2018	CSI-W5*	4	1.60	390	120	11	28.04.2007	S	HOLST	147	1	76	55	44	1886	48	M	13	11a	yes	spread	oxer	right	no	0	0	68,98	no
Leipzig	The Longines FEI World Cup Jumping - Leipzig round A	21.01.2018	CSI-W5*	4	1.60	390	120	11	28.04.2007	S	HOLST	147	1	76	55	44	1886	48	M	14	11b	yes	vertical	vertical	right	no	0	0	68,98	no
Leipzig	The Longines FEI World Cup Jumping - Leipzig round A	21.01.2018	CSI-W5*	4	1.60	390	120	11	28.04.2007	S	HOLST	147	1	76	55	44	1886	48	M	15	12	no	vertical	vertical	right	no	0	0	68,98	no
Leipzig	The Longines FEI World Cup Jumping - Leipzig round B	21.01.2018	CSI-W5*	4	1.60	300	120	11	28.04.2007	S	HOLST	147	1	76	55	44	1886	48	M	1	1	no	vertical	vertical	left	no	0	0	46,78	no
Leipzig	The Longines FEI World Cup Jumping - Leipzig round B	21.01.2018	CSI-W5*	4	1.60	300	120	11	28.04.2007	S	HOLST	147	1	76	55	44	1886	48	M	2	2	no	spread	oxer	left	no	0	0	46,78	no
Leipzig	The Longines FEI World Cup Jumping - Leipzig round B	21.01.2018	CSI-W5*	4	1.60	300	120	11	28.04.2007	S	HOLST	147	1	76	55	44	1886	48	M	3	3	no	vertical	vertical	right	no	0	0	46,78	no
Leipzig	The Longines FEI World Cup Jumping - Leipzig round B	21.01.2018	CSI-W5*	4	1.60	300	120	11	28.04.2007	S	HOLST	147	1	76	55	44	1886	48	M	4	4a	yes	vertical	vertical	right	no	0	0	46,78	no
Leipzig	The Longines FEI World Cup Jumping - Leipzig round B	21.01.2018	CSI-W5*	4	1.60	300	120	11	28.04.2007	S	HOLST	147	1	76	55	44	1886	48	M	5	4b	yes	spread	oxer	right	no	0	0	46,78	no
Leipzig	The Longines FEI World Cup Jumping - Leipzig round B	21.01.2018	CSI-W5*	4	1.60	300	120	11	28.04.2007	S	HOLST	147	1	76	55	44	1886	48	M	6	5	no	spread	oxer	left	no	0	0	46,78	no
Leipzig	The Longines FEI World Cup Jumping - Leipzig round B	21.01.2018	CSI-W5*	4	1.60	300	120	11	28.04.2007	S	HOLST	147	1	76	55	44	1886	48	M	7	6	no	vertical	vertical	left	no	0	0	46,78	no
Leipzig	The Longines FEI World Cup Jumping - Leipzig round B	21.01.2018	CSI-W5*	4	1.60	300	120	11	28.04.2007	S	HOLST	147	1	76	55	44	1886	48	M	8	7	no	vertical	vertical	right	no	0	0	46,78	no
Leipzig	The Longines FEI World Cup Jumping - Leipzig round B	21.01.2018	CSI-W5*	4	1.60	300	120	11	28.04.2007	S	HOLST	147	1	76	55	44	1886	48	M	9	8	no	spread	oxer	right	no	0	0	46,78	no
Leipzig	The Longines FEI World Cup Jumping - Leipzig round A	21.01.2018	CSI-W5*	4	1.60	390	222	11	07.05.2007	M	HOLST	43	1	87	199	55	1406	43	M	1	1	no	vertical	vertical	left	no	0	0	71,06	no
Leipzig	The Longines FEI World Cup Jumping - Leipzig round A	21.01.2018	CSI-W5*	4	1.60	390	222	11	07.05.2007	M	HOLST	43	1	87	199	55	1406	43	M	2	2	no	spread	oxer	straight	no	0	0	71,06	no
Leipzig	The Longines FEI World Cup Jumping - Leipzig round A	21.01.2018	CSI-W5*	4	1.60	390	222	11	07.05.2007	M	HOLST	43	1	87	199	55	1406	43	M	3	3	no	spread	oxer	left	no	0	0	71,06	no
Leipzig	The Longines FEI World Cup Jumping - Leipzig round A	21.01.2018	CSI-W5*	4	1.60	390	222	11	07.05.2007	M	HOLST	43	1	87	199	55	1406	43	M	4	4	no	vertical	vertical	right	no	0	0	71,06	no
Leipzig	The Longines FEI World Cup Jumping - Leipzig round A	21.01.2018	CSI-W5*	4	1.60	390	222	11	07.05.2007	M	HOLST	43	1	87	199	55	1406	43	M	5	5a	yes	vertical	vertical	right	no	0	0	71,06	no
Leipzig	The Longines FEI World Cup Jumping - Leipzig round A	21.01.2018	CSI-W5*	4	1.60	390	222	11	07.05.2007	M	HOLST	43	1	87	199	55	1406	43	M	6	5b	yes	spread	oxer	right	no	0	0	71,06	no
Leipzig	The Longines FEI World Cup Jumping - Leipzig round A	21.01.2018	CSI-W5*	4	1.60	390	222	11	07.05.2007	M	HOLST	43	1	87	199	55	1406	43	M	7	5c	yes	spread	oxer	right	no	0	0	71,06	no
Leipzig	The Longines FEI World Cup Jumping - Leipzig round A	21.01.2018	CSI-W5*	4	1.60	390	222	11	07.05.2007	M	HOLST	43	1	87	199	55	1406	43	M	8	6	no	vertical	vertical	left	yes	4	0	71,06	no
Leipzig	The Longines FEI World Cup Jumping - Leipzig round A	21.01.2018	CSI-W5*	4	1.60	390	222	11	07.05.2007	M	HOLST	43	1	87	199	55	1406	43	M	9	7	no	spread	oxer with water	left	no	0	0	71,06	no
Leipzig	The Longines FEI World Cup Jumping - Leipzig round A	21.01.2018	CSI-W5*	4	1.60	390	222	11	07.05.2007	M	HOLST	43	1	87	199	55	1406	43	M	10	8	no	spread	oxer	straight	no	0	0	71,06	no
Leipzig	The Longines FEI World Cup Jumping - Leipzig round A	21.01.2018	CSI-W5*	4	1.60	390	222	11	07.05.2007	M	HOLST	43	1	87	199	55	1406	43	M	11	9	no	vertical	vertical	left	no	0	0	71,06	no
Leipzig	The Longines FEI World Cup Jumping - Leipzig round A	21.01.2018	CSI-W5*	4	1.60	390	222	11	07.05.2007	M	HOLST	43	1	87	199	55	1406	43	M	12	10	no	spread	oxer	left	no	0	0	71,06	no
Leipzig	The Longines FEI World Cup Jumping - Leipzig round A	21.01.2018	CSI-W5*	4	1.60	390	222	11	07.05.2007	M	HOLST	43	1	87	199	55	1406	43	M	13	11a	yes	spread	oxer	right	no	0	0	71,06	no
Leipzig	The Longines FEI World Cup Jumping - Leipzig round A	21.01.2018	CSI-W5*	4	1.60	390	222	11	07.05.2007	M	HOLST	43	1	87	199	55	1406	43	M	14	11b	yes	vertical	vertical	right	yes	4	0	71,06	no
Leipzig	The Longines FEI World Cup Jumping - Leipzig round A	21.01.2018	CSI-W5*	4	1.60	390	222	11	07.05.2007	M	HOLST	43	1	87	199	55	1406	43	M	15	12	no	vertical	vertical	right	yes	4	1	71,06	no
Leipzig	The Longines FEI World Cup Jumping - Leipzig round A	21.01.2018	CSI-W5*	4	1.60	390	116	11	25.05.2007	S	WESTF	157	15	90	169	24	632	50	M	1	1	no	vertical	vertical	left	no	0	0	68,94	no
Leipzig	The Longines FEI World Cup Jumping - Leipzig round A	21.01.2018	CSI-W5*	4	1.60	390	116	11	25.05.2007	S	WESTF	157	15	90	169	24	632	50	M	2	2	no	spread	oxer	straight	no	0	0	68,94	no
Leipzig	The Longines FEI World Cup Jumping - Leipzig round A	21.01.2018	CSI-W5*	4	1.60	390	116	11	25.05.2007	S	WESTF	157	15	90	169	24	632	50	M	3	3	no	spread	oxer	left	no	0	0	68,94	no
Leipzig	The Longines FEI World Cup Jumping - Leipzig round A	21.01.2018	CSI-W5*	4	1.60	390	116	11	25.05.2007	S	WESTF	157	15	90	169	24	632	50	M	4	4	no	vertical	vertical	right	no	0	0	68,94	no
Leipzig	The Longines FEI World Cup Jumping - Leipzig round A	21.01.2018	CSI-W5*	4	1.60	390	116	11	25.05.2007	S	WESTF	157	15	90	169	24	632	50	M	5	5a	yes	vertical	vertical	right	no	0	0	68,94	no
Leipzig	The Longines FEI World Cup Jumping - Leipzig round A	21.01.2018	CSI-W5*	4	1.60	390	116	11	25.05.2007	S	WESTF	157	15	90	169	24	632	50	M	6	5b	yes	spread	oxer	right	no	0	0	68,94	no
Leipzig	The Longines FEI World Cup Jumping - Leipzig round A	21.01.2018	CSI-W5*	4	1.60	390	116	11	25.05.2007	S	WESTF	157	15	90	169	24	632	50	M	7	5c	yes	spread	oxer	right	no	0	0	68,94	no
Leipzig	The Longines FEI World Cup Jumping - Leipzig round A	21.01.2018	CSI-W5*	4	1.60	390	116	11	25.05.2007	S	WESTF	157	15	90	169	24	632	50	M	8	6	no	vertical	vertical	left	no	0	0	68,94	no
Leipzig	The Longines FEI World Cup Jumping - Leipzig round A	21.01.2018	CSI-W5*	4	1.60	390	116	11	25.05.2007	S	WESTF	157	15	90	169	24	632	50	M	9	7	no	spread	oxer with water	left	no	0	0	68,94	no
Leipzig	The Longines FEI World Cup Jumping - Leipzig round A	21.01.2018	CSI-W5*	4	1.60	390	116	11	25.05.2007	S	WESTF	157	15	90	169	24	632	50	M	10	8	no	spread	oxer	straight	no	0	0	68,94	no
Leipzig	The Longines FEI World Cup Jumping - Leipzig round A	21.01.2018	CSI-W5*	4	1.60	390	116	11	25.05.2007	S	WESTF	157	15	90	169	24	632	50	M	11	9	no	vertical	vertical	left	no	0	0	68,94	no
Leipzig	The Longines FEI World Cup Jumping - Leipzig round A	21.01.2018	CSI-W5*	4	1.60	390	116	11	25.05.2007	S	WESTF	157	15	90	169	24	632	50	M	12	10	no	spread	oxer	left	no	0	0	68,94	no
Leipzig	The Longines FEI World Cup Jumping - Leipzig round A	21.01.2018	CSI-W5*	4	1.60	390	116	11	25.05.2007	S	WESTF	157	15	90	169	24	632	50	M	13	11a	yes	spread	oxer	right	no	0	0	68,94	no
Leipzig	The Longines FEI World Cup Jumping - Leipzig round A	21.01.2018	CSI-W5*	4	1.60	390	116	11	25.05.2007	S	WESTF	157	15	90	169	24	632	50	M	14	11b	yes	vertical	vertical	right	no	0	0	68,94	no
Leipzig	The Longines FEI World Cup Jumping - Leipzig round A	21.01.2018	CSI-W5*	4	1.60	390	116	11	25.05.2007	S	WESTF	157	15	90	169	24	632	50	M	15	12	no	vertical	vertical	right	no	0	0	68,94	no
Leipzig	The Longines FEI World Cup Jumping - Leipzig round B	21.01.2018	CSI-W5*	4	1.60	300	116	11	25.05.2007	S	WESTF	157	15	90	169	24	632	50	M	1	1	no	vertical	vertical	left	no	0	0	45,96	no
Leipzig	The Longines FEI World Cup Jumping - Leipzig round B	21.01.2018	CSI-W5*	4	1.60	300	116	11	25.05.2007	S	WESTF	157	15	90	169	24	632	50	M	2	2	no	spread	oxer	left	no	0	0	45,96	no
Leipzig	The Longines FEI World Cup Jumping - Leipzig round B	21.01.2018	CSI-W5*	4	1.60	300	116	11	25.05.2007	S	WESTF	157	15	90	169	24	632	50	M	3	3	no	vertical	vertical	right	no	0	0	45,96	no
Leipzig	The Longines FEI World Cup Jumping - Leipzig round B	21.01.2018	CSI-W5*	4	1.60	300	116	11	25.05.2007	S	WESTF	157	15	90	169	24	632	50	M	4	4a	yes	vertical	vertical	right	no	0	0	45,96	no
Leipzig	The Longines FEI World Cup Jumping - Leipzig round B	21.01.2018	CSI-W5*	4	1.60	300	116	11	25.05.2007	S	WESTF	157	15	90	169	24	632	50	M	5	4b	yes	spread	oxer	right	yes	4	0	45,96	no
Leipzig	The Longines FEI World Cup Jumping - Leipzig round B	21.01.2018	CSI-W5*	4	1.60	300	116	11	25.05.2007	S	WESTF	157	15	90	169	24	632	50	M	6	5	no	spread	oxer	left	no	0	0	45,96	no
Leipzig	The Longines FEI World Cup Jumping - Leipzig round B	21.01.2018	CSI-W5*	4	1.60	300	116	11	25.05.2007	S	WESTF	157	15	90	169	24	632	50	M	7	6	no	vertical	vertical	left	no	0	0	45,96	no
Leipzig	The Longines FEI World Cup Jumping - Leipzig round B	21.01.2018	CSI-W5*	4	1.60	300	116	11	25.05.2007	S	WESTF	157	15	90	169	24	632	50	M	8	7	no	vertical	vertical	right	no	0	0	45,96	no
Leipzig	The Longines FEI World Cup Jumping - Leipzig round B	21.01.2018	CSI-W5*	4	1.60	300	116	11	25.05.2007	S	WESTF	157	15	90	169	24	632	50	M	9	8	no	spread	oxer	right	no	0	0	45,96	no
Leipzig	The Longines FEI World Cup Jumping - Leipzig round A	21.01.2018	CSI-W5*	4	1.60	390	168	9	25.03.2009	G	HOLST	89	2	91	93	45	1436	65	M	1	1	no	vertical	vertical	left	no	0	0	69,97	no
Leipzig	The Longines FEI World Cup Jumping - Leipzig round A	21.01.2018	CSI-W5*	4	1.60	390	168	9	25.03.2009	G	HOLST	89	2	91	93	45	1436	65	M	2	2	no	spread	oxer	straight	no	0	0	69,97	no
Leipzig	The Longines FEI World Cup Jumping - Leipzig round A	21.01.2018	CSI-W5*	4	1.60	390	168	9	25.03.2009	G	HOLST	89	2	91	93	45	1436	65	M	3	3	no	spread	oxer	left	no	0	0	69,97	no
Leipzig	The Longines FEI World Cup Jumping - Leipzig round A	21.01.2018	CSI-W5*	4	1.60	390	168	9	25.03.2009	G	HOLST	89	2	91	93	45	1436	65	M	4	4	no	vertical	vertical	right	no	0	0	69,97	no
Leipzig	The Longines FEI World Cup Jumping - Leipzig round A	21.01.2018	CSI-W5*	4	1.60	390	168	9	25.03.2009	G	HOLST	89	2	91	93	45	1436	65	M	5	5a	yes	vertical	vertical	right	no	0	0	69,97	no
Leipzig	The Longines FEI World Cup Jumping - Leipzig round A	21.01.2018	CSI-W5*	4	1.60	390	168	9	25.03.2009	G	HOLST	89	2	91	93	45	1436	65	M	6	5b	yes	spread	oxer	right	no	0	0	69,97	no
Leipzig	The Longines FEI World Cup Jumping - Leipzig round A	21.01.2018	CSI-W5*	4	1.60	390	168	9	25.03.2009	G	HOLST	89	2	91	93	45	1436	65	M	7	5c	yes	spread	oxer	right	no	0	0	69,97	no
Leipzig	The Longines FEI World Cup Jumping - Leipzig round A	21.01.2018	CSI-W5*	4	1.60	390	168	9	25.03.2009	G	HOLST	89	2	91	93	45	1436	65	M	8	6	no	vertical	vertical	left	no	0	0	69,97	no
Leipzig	The Longines FEI World Cup Jumping - Leipzig round A	21.01.2018	CSI-W5*	4	1.60	390	168	9	25.03.2009	G	HOLST	89	2	91	93	45	1436	65	M	9	7	no	spread	oxer with water	left	no	0	0	69,97	no
Leipzig	The Longines FEI World Cup Jumping - Leipzig round A	21.01.2018	CSI-W5*	4	1.60	390	168	9	25.03.2009	G	HOLST	89	2	91	93	45	1436	65	M	10	8	no	spread	oxer	straight	no	0	0	69,97	no
Leipzig	The Longines FEI World Cup Jumping - Leipzig round A	21.01.2018	CSI-W5*	4	1.60	390	168	9	25.03.2009	G	HOLST	89	2	91	93	45	1436	65	M	11	9	no	vertical	vertical	left	no	0	0	69,97	no
Leipzig	The Longines FEI World Cup Jumping - Leipzig round A	21.01.2018	CSI-W5*	4	1.60	390	168	9	25.03.2009	G	HOLST	89	2	91	93	45	1436	65	M	12	10	no	spread	oxer	left	no	0	0	69,97	no
Leipzig	The Longines FEI World Cup Jumping - Leipzig round A	21.01.2018	CSI-W5*	4	1.60	390	168	9	25.03.2009	G	HOLST	89	2	91	93	45	1436	65	M	13	11a	yes	spread	oxer	right	no	0	0	69,97	no
Leipzig	The Longines FEI World Cup Jumping - Leipzig round A	21.01.2018	CSI-W5*	4	1.60	390	168	9	25.03.2009	G	HOLST	89	2	91	93	45	1436	65	M	14	11b	yes	vertical	vertical	right	no	0	0	69,97	no
Leipzig	The Longines FEI World Cup Jumping - Leipzig round A	21.01.2018	CSI-W5*	4	1.60	390	168	9	25.03.2009	G	HOLST	89	2	91	93	45	1436	65	M	15	12	no	vertical	vertical	right	no	0	0	69,97	no
Leipzig	The Longines FEI World Cup Jumping - Leipzig round B	21.01.2018	CSI-W5*	4	1.60	300	168	9	25.03.2009	G	HOLST	89	2	91	93	45	1436	65	M	1	1	no	vertical	vertical	left	no	0	0	46,11	no
Leipzig	The Longines FEI World Cup Jumping - Leipzig round B	21.01.2018	CSI-W5*	4	1.60	300	168	9	25.03.2009	G	HOLST	89	2	91	93	45	1436	65	M	2	2	no	spread	oxer	left	no	0	0	46,11	no
Leipzig	The Longines FEI World Cup Jumping - Leipzig round B	21.01.2018	CSI-W5*	4	1.60	300	168	9	25.03.2009	G	HOLST	89	2	91	93	45	1436	65	M	3	3	no	vertical	vertical	right	no	0	0	46,11	no
Leipzig	The Longines FEI World Cup Jumping - Leipzig round B	21.01.2018	CSI-W5*	4	1.60	300	168	9	25.03.2009	G	HOLST	89	2	91	93	45	1436	65	M	4	4a	yes	vertical	vertical	right	yes	4	0	46,11	no
Leipzig	The Longines FEI World Cup Jumping - Leipzig round B	21.01.2018	CSI-W5*	4	1.60	300	168	9	25.03.2009	G	HOLST	89	2	91	93	45	1436	65	M	5	4b	yes	spread	oxer	right	no	0	0	46,11	no
Leipzig	The Longines FEI World Cup Jumping - Leipzig round B	21.01.2018	CSI-W5*	4	1.60	300	168	9	25.03.2009	G	HOLST	89	2	91	93	45	1436	65	M	6	5	no	spread	oxer	left	no	0	0	46,11	no
Leipzig	The Longines FEI World Cup Jumping - Leipzig round B	21.01.2018	CSI-W5*	4	1.60	300	168	9	25.03.2009	G	HOLST	89	2	91	93	45	1436	65	M	7	6	no	vertical	vertical	left	no	0	0	46,11	no
Leipzig	The Longines FEI World Cup Jumping - Leipzig round B	21.01.2018	CSI-W5*	4	1.60	300	168	9	25.03.2009	G	HOLST	89	2	91	93	45	1436	65	M	8	7	no	vertical	vertical	right	no	0	0	46,11	no
Leipzig	The Longines FEI World Cup Jumping - Leipzig round B	21.01.2018	CSI-W5*	4	1.60	300	168	9	25.03.2009	G	HOLST	89	2	91	93	45	1436	65	M	9	8	no	spread	oxer	right	no	0	0	46,11	no
Leipzig	The Longines FEI World Cup Jumping - Leipzig round A	21.01.2018	CSI-W5*	4	1.60	390	117	11	22.02.2007	S	OS	91	0	97	201	28	1198	35	M	1	1	no	vertical	vertical	left	yes	4	0	0,00	yes
Leipzig	The Longines FEI World Cup Jumping - Leipzig round A	21.01.2018	CSI-W5*	4	1.60	390	117	11	22.02.2007	S	OS	91	0	97	201	28	1198	35	M	2	2	no	spread	oxer	straight	no	0	0	0,00	yes
Leipzig	The Longines FEI World Cup Jumping - Leipzig round A	21.01.2018	CSI-W5*	4	1.60	390	117	11	22.02.2007	S	OS	91	0	97	201	28	1198	35	M	3	3	no	spread	oxer	left	no	0	0	0,00	yes
Leipzig	The Longines FEI World Cup Jumping - Leipzig round A	21.01.2018	CSI-W5*	4	1.60	390	117	11	22.02.2007	S	OS	91	0	97	201	28	1198	35	M	4	4	no	vertical	vertical	right	no	0	0	0,00	yes
Leipzig	The Longines FEI World Cup Jumping - Leipzig round A	21.01.2018	CSI-W5*	4	1.60	390	117	11	22.02.2007	S	OS	91	0	97	201	28	1198	35	M	5	5a	yes	vertical	vertical	right	no	0	0	0,00	yes
Leipzig	The Longines FEI World Cup Jumping - Leipzig round A	21.01.2018	CSI-W5*	4	1.60	390	117	11	22.02.2007	S	OS	91	0	97	201	28	1198	35	M	6	5b	yes	spread	oxer	right	no	0	0	0,00	yes
Leipzig	The Longines FEI World Cup Jumping - Leipzig round A	21.01.2018	CSI-W5*	4	1.60	390	117	11	22.02.2007	S	OS	91	0	97	201	28	1198	35	M	7	5c	yes	spread	oxer	right	yes	4	0	0,00	yes
Leipzig	The Longines FEI World Cup Jumping - Leipzig round A	21.01.2018	CSI-W5*	4	1.60	390	117	11	22.02.2007	S	OS	91	0	97	201	28	1198	35	M	8	6	no	vertical	vertical	left	no	0	0	0,00	yes
Leipzig	The Longines FEI World Cup Jumping - Leipzig round A	21.01.2018	CSI-W5*	4	1.60	390	117	11	22.02.2007	S	OS	91	0	97	201	28	1198	35	M	9	7	no	spread	oxer with water	left	no	0	0	0,00	yes
Leipzig	The Longines FEI World Cup Jumping - Leipzig round A	21.01.2018	CSI-W5*	4	1.60	390	117	11	22.02.2007	S	OS	91	0	97	201	28	1198	35	M	10	8	no	spread	oxer	straight	yes	4	0	0,00	yes
Leipzig	The Longines FEI World Cup Jumping - Leipzig round A	21.01.2018	CSI-W5*	4	1.60	390	117	11	22.02.2007	S	OS	91	0	97	201	28	1198	35	M	11	9	no	vertical	vertical	left	no	0	0	0,00	yes
Leipzig	The Longines FEI World Cup Jumping - Leipzig round A	21.01.2018	CSI-W5*	4	1.60	390	117	11	22.02.2007	S	OS	91	0	97	201	28	1198	35	M	12	10	no	spread	oxer	left	no	0	0	0,00	yes
Leipzig	The Longines FEI World Cup Jumping - Leipzig round A	21.01.2018	CSI-W5*	4	1.60	390	117	11	22.02.2007	S	OS	91	0	97	201	28	1198	35	M	13	11a	yes	spread	oxer	right	no	0	0	0,00	yes
Leipzig	The Longines FEI World Cup Jumping - Leipzig round A	21.01.2018	CSI-W5*	4	1.60	390	117	11	22.02.2007	S	OS	91	0	97	201	28	1198	35	M	14	11b	yes	vertical	vertical	right	no	0	0	0,00	yes
Leipzig	The Longines FEI World Cup Jumping - Leipzig round A	21.01.2018	CSI-W5*	4	1.60	390	117	11	22.02.2007	S	OS	91	0	97	201	28	1198	35	M	15	12	no	vertical	vertical	right	no	0	0	0,00	yes
Leipzig	The Longines FEI World Cup Jumping - Leipzig round A	21.01.2018	CSI-W5*	4	1.60	390	2	13	17.04.2005	G	KWPN	274	23	101	46	53	686	44	M	1	1	no	vertical	vertical	left	no	0	0	67,88	no
Leipzig	The Longines FEI World Cup Jumping - Leipzig round A	21.01.2018	CSI-W5*	4	1.60	390	2	13	17.04.2005	G	KWPN	274	23	101	46	53	686	44	M	2	2	no	spread	oxer	straight	no	0	0	67,88	no
Leipzig	The Longines FEI World Cup Jumping - Leipzig round A	21.01.2018	CSI-W5*	4	1.60	390	2	13	17.04.2005	G	KWPN	274	23	101	46	53	686	44	M	3	3	no	spread	oxer	left	no	0	0	67,88	no
Leipzig	The Longines FEI World Cup Jumping - Leipzig round A	21.01.2018	CSI-W5*	4	1.60	390	2	13	17.04.2005	G	KWPN	274	23	101	46	53	686	44	M	4	4	no	vertical	vertical	right	no	0	0	67,88	no
Leipzig	The Longines FEI World Cup Jumping - Leipzig round A	21.01.2018	CSI-W5*	4	1.60	390	2	13	17.04.2005	G	KWPN	274	23	101	46	53	686	44	M	5	5a	yes	vertical	vertical	right	no	0	0	67,88	no
Leipzig	The Longines FEI World Cup Jumping - Leipzig round A	21.01.2018	CSI-W5*	4	1.60	390	2	13	17.04.2005	G	KWPN	274	23	101	46	53	686	44	M	6	5b	yes	spread	oxer	right	no	0	0	67,88	no
Leipzig	The Longines FEI World Cup Jumping - Leipzig round A	21.01.2018	CSI-W5*	4	1.60	390	2	13	17.04.2005	G	KWPN	274	23	101	46	53	686	44	M	7	5c	yes	spread	oxer	right	no	0	0	67,88	no
Leipzig	The Longines FEI World Cup Jumping - Leipzig round A	21.01.2018	CSI-W5*	4	1.60	390	2	13	17.04.2005	G	KWPN	274	23	101	46	53	686	44	M	8	6	no	vertical	vertical	left	no	0	0	67,88	no
Leipzig	The Longines FEI World Cup Jumping - Leipzig round A	21.01.2018	CSI-W5*	4	1.60	390	2	13	17.04.2005	G	KWPN	274	23	101	46	53	686	44	M	9	7	no	spread	oxer with water	left	no	0	0	67,88	no
Leipzig	The Longines FEI World Cup Jumping - Leipzig round A	21.01.2018	CSI-W5*	4	1.60	390	2	13	17.04.2005	G	KWPN	274	23	101	46	53	686	44	M	10	8	no	spread	oxer	straight	no	0	0	67,88	no
Leipzig	The Longines FEI World Cup Jumping - Leipzig round A	21.01.2018	CSI-W5*	4	1.60	390	2	13	17.04.2005	G	KWPN	274	23	101	46	53	686	44	M	11	9	no	vertical	vertical	left	no	0	0	67,88	no
Leipzig	The Longines FEI World Cup Jumping - Leipzig round A	21.01.2018	CSI-W5*	4	1.60	390	2	13	17.04.2005	G	KWPN	274	23	101	46	53	686	44	M	12	10	no	spread	oxer	left	no	0	0	67,88	no
Leipzig	The Longines FEI World Cup Jumping - Leipzig round A	21.01.2018	CSI-W5*	4	1.60	390	2	13	17.04.2005	G	KWPN	274	23	101	46	53	686	44	M	13	11a	yes	spread	oxer	right	no	0	0	67,88	no
Leipzig	The Longines FEI World Cup Jumping - Leipzig round A	21.01.2018	CSI-W5*	4	1.60	390	2	13	17.04.2005	G	KWPN	274	23	101	46	53	686	44	M	14	11b	yes	vertical	vertical	right	no	0	0	67,88	no
Leipzig	The Longines FEI World Cup Jumping - Leipzig round A	21.01.2018	CSI-W5*	4	1.60	390	2	13	17.04.2005	G	KWPN	274	23	101	46	53	686	44	M	15	12	no	vertical	vertical	right	no	0	0	67,88	no
Leipzig	The Longines FEI World Cup Jumping - Leipzig round B	21.01.2018	CSI-W5*	4	1.60	300	2	13	17.04.2005	G	KWPN	274	23	101	46	53	686	44	M	1	1	no	vertical	vertical	left	no	0	0	43,39	no
Leipzig	The Longines FEI World Cup Jumping - Leipzig round B	21.01.2018	CSI-W5*	4	1.60	300	2	13	17.04.2005	G	KWPN	274	23	101	46	53	686	44	M	2	2	no	spread	oxer	left	no	0	0	43,39	no
Leipzig	The Longines FEI World Cup Jumping - Leipzig round B	21.01.2018	CSI-W5*	4	1.60	300	2	13	17.04.2005	G	KWPN	274	23	101	46	53	686	44	M	3	3	no	vertical	vertical	right	no	0	0	43,39	no
Leipzig	The Longines FEI World Cup Jumping - Leipzig round B	21.01.2018	CSI-W5*	4	1.60	300	2	13	17.04.2005	G	KWPN	274	23	101	46	53	686	44	M	4	4a	yes	vertical	vertical	right	no	0	0	43,39	no
Leipzig	The Longines FEI World Cup Jumping - Leipzig round B	21.01.2018	CSI-W5*	4	1.60	300	2	13	17.04.2005	G	KWPN	274	23	101	46	53	686	44	M	5	4b	yes	spread	oxer	right	no	0	0	43,39	no
Leipzig	The Longines FEI World Cup Jumping - Leipzig round B	21.01.2018	CSI-W5*	4	1.60	300	2	13	17.04.2005	G	KWPN	274	23	101	46	53	686	44	M	6	5	no	spread	oxer	left	no	0	0	43,39	no
Leipzig	The Longines FEI World Cup Jumping - Leipzig round B	21.01.2018	CSI-W5*	4	1.60	300	2	13	17.04.2005	G	KWPN	274	23	101	46	53	686	44	M	7	6	no	vertical	vertical	left	no	0	0	43,39	no
Leipzig	The Longines FEI World Cup Jumping - Leipzig round B	21.01.2018	CSI-W5*	4	1.60	300	2	13	17.04.2005	G	KWPN	274	23	101	46	53	686	44	M	8	7	no	vertical	vertical	right	no	0	0	43,39	no
Leipzig	The Longines FEI World Cup Jumping - Leipzig round B	21.01.2018	CSI-W5*	4	1.60	300	2	13	17.04.2005	G	KWPN	274	23	101	46	53	686	44	M	9	8	no	spread	oxer	right	no	0	0	43,39	no
Leipzig	The Longines FEI World Cup Jumping - Leipzig round A	21.01.2018	CSI-W5*	4	1.60	390	172	10	11.07.2008	S	SBS	149	2	104	24	42	2261	97	M	1	1	no	vertical	vertical	left	no	0	0	67,18	no
Leipzig	The Longines FEI World Cup Jumping - Leipzig round A	21.01.2018	CSI-W5*	4	1.60	390	172	10	11.07.2008	S	SBS	149	2	104	24	42	2261	97	M	2	2	no	spread	oxer	straight	no	0	0	67,18	no
Leipzig	The Longines FEI World Cup Jumping - Leipzig round A	21.01.2018	CSI-W5*	4	1.60	390	172	10	11.07.2008	S	SBS	149	2	104	24	42	2261	97	M	3	3	no	spread	oxer	left	no	0	0	67,18	no
Leipzig	The Longines FEI World Cup Jumping - Leipzig round A	21.01.2018	CSI-W5*	4	1.60	390	172	10	11.07.2008	S	SBS	149	2	104	24	42	2261	97	M	4	4	no	vertical	vertical	right	no	0	0	67,18	no
Leipzig	The Longines FEI World Cup Jumping - Leipzig round A	21.01.2018	CSI-W5*	4	1.60	390	172	10	11.07.2008	S	SBS	149	2	104	24	42	2261	97	M	5	5a	yes	vertical	vertical	right	no	0	0	67,18	no
Leipzig	The Longines FEI World Cup Jumping - Leipzig round A	21.01.2018	CSI-W5*	4	1.60	390	172	10	11.07.2008	S	SBS	149	2	104	24	42	2261	97	M	6	5b	yes	spread	oxer	right	no	0	0	67,18	no
Leipzig	The Longines FEI World Cup Jumping - Leipzig round A	21.01.2018	CSI-W5*	4	1.60	390	172	10	11.07.2008	S	SBS	149	2	104	24	42	2261	97	M	7	5c	yes	spread	oxer	right	no	0	0	67,18	no
Leipzig	The Longines FEI World Cup Jumping - Leipzig round A	21.01.2018	CSI-W5*	4	1.60	390	172	10	11.07.2008	S	SBS	149	2	104	24	42	2261	97	M	8	6	no	vertical	vertical	left	no	0	0	67,18	no
Leipzig	The Longines FEI World Cup Jumping - Leipzig round A	21.01.2018	CSI-W5*	4	1.60	390	172	10	11.07.2008	S	SBS	149	2	104	24	42	2261	97	M	9	7	no	spread	oxer with water	left	no	0	0	67,18	no
Leipzig	The Longines FEI World Cup Jumping - Leipzig round A	21.01.2018	CSI-W5*	4	1.60	390	172	10	11.07.2008	S	SBS	149	2	104	24	42	2261	97	M	10	8	no	spread	oxer	straight	yes	4	0	67,18	no
Leipzig	The Longines FEI World Cup Jumping - Leipzig round A	21.01.2018	CSI-W5*	4	1.60	390	172	10	11.07.2008	S	SBS	149	2	104	24	42	2261	97	M	11	9	no	vertical	vertical	left	no	0	0	67,18	no
Leipzig	The Longines FEI World Cup Jumping - Leipzig round A	21.01.2018	CSI-W5*	4	1.60	390	172	10	11.07.2008	S	SBS	149	2	104	24	42	2261	97	M	12	10	no	spread	oxer	left	no	0	0	67,18	no
Leipzig	The Longines FEI World Cup Jumping - Leipzig round A	21.01.2018	CSI-W5*	4	1.60	390	172	10	11.07.2008	S	SBS	149	2	104	24	42	2261	97	M	13	11a	yes	spread	oxer	right	no	0	0	67,18	no
Leipzig	The Longines FEI World Cup Jumping - Leipzig round A	21.01.2018	CSI-W5*	4	1.60	390	172	10	11.07.2008	S	SBS	149	2	104	24	42	2261	97	M	14	11b	yes	vertical	vertical	right	no	0	0	67,18	no
Leipzig	The Longines FEI World Cup Jumping - Leipzig round A	21.01.2018	CSI-W5*	4	1.60	390	172	10	11.07.2008	S	SBS	149	2	104	24	42	2261	97	M	15	12	no	vertical	vertical	right	no	0	0	67,18	no
Leipzig	The Longines FEI World Cup Jumping - Leipzig round A	21.01.2018	CSI-W5*	4	1.60	390	148	10	28.06.2008	G	Bezplemenné	125	0	120	550	27	986	18	F	1	1	no	vertical	vertical	left	no	0	0	71,76	no
Leipzig	The Longines FEI World Cup Jumping - Leipzig round A	21.01.2018	CSI-W5*	4	1.60	390	148	10	28.06.2008	G	Bezplemenné	125	0	120	550	27	986	18	F	2	2	no	spread	oxer	straight	no	0	0	71,76	no
Leipzig	The Longines FEI World Cup Jumping - Leipzig round A	21.01.2018	CSI-W5*	4	1.60	390	148	10	28.06.2008	G	Bezplemenné	125	0	120	550	27	986	18	F	3	3	no	spread	oxer	left	no	0	0	71,76	no
Leipzig	The Longines FEI World Cup Jumping - Leipzig round A	21.01.2018	CSI-W5*	4	1.60	390	148	10	28.06.2008	G	Bezplemenné	125	0	120	550	27	986	18	F	4	4	no	vertical	vertical	right	no	0	0	71,76	no
Leipzig	The Longines FEI World Cup Jumping - Leipzig round A	21.01.2018	CSI-W5*	4	1.60	390	148	10	28.06.2008	G	Bezplemenné	125	0	120	550	27	986	18	F	5	5a	yes	vertical	vertical	right	no	0	0	71,76	no
Leipzig	The Longines FEI World Cup Jumping - Leipzig round A	21.01.2018	CSI-W5*	4	1.60	390	148	10	28.06.2008	G	Bezplemenné	125	0	120	550	27	986	18	F	6	5b	yes	spread	oxer	right	no	0	0	71,76	no
Leipzig	The Longines FEI World Cup Jumping - Leipzig round A	21.01.2018	CSI-W5*	4	1.60	390	148	10	28.06.2008	G	Bezplemenné	125	0	120	550	27	986	18	F	7	5c	yes	spread	oxer	right	no	0	0	71,76	no
Leipzig	The Longines FEI World Cup Jumping - Leipzig round A	21.01.2018	CSI-W5*	4	1.60	390	148	10	28.06.2008	G	Bezplemenné	125	0	120	550	27	986	18	F	8	6	no	vertical	vertical	left	yes	4	0	71,76	no
Leipzig	The Longines FEI World Cup Jumping - Leipzig round A	21.01.2018	CSI-W5*	4	1.60	390	148	10	28.06.2008	G	Bezplemenné	125	0	120	550	27	986	18	F	9	7	no	spread	oxer with water	left	no	0	0	71,76	no
Leipzig	The Longines FEI World Cup Jumping - Leipzig round A	21.01.2018	CSI-W5*	4	1.60	390	148	10	28.06.2008	G	Bezplemenné	125	0	120	550	27	986	18	F	10	8	no	spread	oxer	straight	no	0	0	71,76	no
Leipzig	The Longines FEI World Cup Jumping - Leipzig round A	21.01.2018	CSI-W5*	4	1.60	390	148	10	28.06.2008	G	Bezplemenné	125	0	120	550	27	986	18	F	11	9	no	vertical	vertical	left	no	0	0	71,76	no
Leipzig	The Longines FEI World Cup Jumping - Leipzig round A	21.01.2018	CSI-W5*	4	1.60	390	148	10	28.06.2008	G	Bezplemenné	125	0	120	550	27	986	18	F	12	10	no	spread	oxer	left	no	0	0	71,76	no
Leipzig	The Longines FEI World Cup Jumping - Leipzig round A	21.01.2018	CSI-W5*	4	1.60	390	148	10	28.06.2008	G	Bezplemenné	125	0	120	550	27	986	18	F	13	11a	yes	spread	oxer	right	no	0	0	71,76	no
Leipzig	The Longines FEI World Cup Jumping - Leipzig round A	21.01.2018	CSI-W5*	4	1.60	390	148	10	28.06.2008	G	Bezplemenné	125	0	120	550	27	986	18	F	14	11b	yes	vertical	vertical	right	no	0	0	71,76	no
Leipzig	The Longines FEI World Cup Jumping - Leipzig round A	21.01.2018	CSI-W5*	4	1.60	390	148	10	28.06.2008	G	Bezplemenné	125	0	120	550	27	986	18	F	15	12	no	vertical	vertical	right	no	0	1	71,76	no
Leipzig	The Longines FEI World Cup Jumping - Leipzig round A	21.01.2018	CSI-W5*	4	1.60	390	221	14	30.04.2004	G	KWPN	281	20	121	2	38	1958	103	M	1	1	no	vertical	vertical	left	no	0	0	66,28	no
Leipzig	The Longines FEI World Cup Jumping - Leipzig round A	21.01.2018	CSI-W5*	4	1.60	390	221	14	30.04.2004	G	KWPN	281	20	121	2	38	1958	103	M	2	2	no	spread	oxer	straight	no	0	0	66,28	no
Leipzig	The Longines FEI World Cup Jumping - Leipzig round A	21.01.2018	CSI-W5*	4	1.60	390	221	14	30.04.2004	G	KWPN	281	20	121	2	38	1958	103	M	3	3	no	spread	oxer	left	no	0	0	66,28	no
Leipzig	The Longines FEI World Cup Jumping - Leipzig round A	21.01.2018	CSI-W5*	4	1.60	390	221	14	30.04.2004	G	KWPN	281	20	121	2	38	1958	103	M	4	4	no	vertical	vertical	right	no	0	0	66,28	no
Leipzig	The Longines FEI World Cup Jumping - Leipzig round A	21.01.2018	CSI-W5*	4	1.60	390	221	14	30.04.2004	G	KWPN	281	20	121	2	38	1958	103	M	5	5a	yes	vertical	vertical	right	no	0	0	66,28	no
Leipzig	The Longines FEI World Cup Jumping - Leipzig round A	21.01.2018	CSI-W5*	4	1.60	390	221	14	30.04.2004	G	KWPN	281	20	121	2	38	1958	103	M	6	5b	yes	spread	oxer	right	no	0	0	66,28	no
Leipzig	The Longines FEI World Cup Jumping - Leipzig round A	21.01.2018	CSI-W5*	4	1.60	390	221	14	30.04.2004	G	KWPN	281	20	121	2	38	1958	103	M	7	5c	yes	spread	oxer	right	no	0	0	66,28	no
Leipzig	The Longines FEI World Cup Jumping - Leipzig round A	21.01.2018	CSI-W5*	4	1.60	390	221	14	30.04.2004	G	KWPN	281	20	121	2	38	1958	103	M	8	6	no	vertical	vertical	left	no	0	0	66,28	no
Leipzig	The Longines FEI World Cup Jumping - Leipzig round A	21.01.2018	CSI-W5*	4	1.60	390	221	14	30.04.2004	G	KWPN	281	20	121	2	38	1958	103	M	9	7	no	spread	oxer with water	left	no	0	0	66,28	no
Leipzig	The Longines FEI World Cup Jumping - Leipzig round A	21.01.2018	CSI-W5*	4	1.60	390	221	14	30.04.2004	G	KWPN	281	20	121	2	38	1958	103	M	10	8	no	spread	oxer	straight	no	0	0	66,28	no
Leipzig	The Longines FEI World Cup Jumping - Leipzig round A	21.01.2018	CSI-W5*	4	1.60	390	221	14	30.04.2004	G	KWPN	281	20	121	2	38	1958	103	M	11	9	no	vertical	vertical	left	no	0	0	66,28	no
Leipzig	The Longines FEI World Cup Jumping - Leipzig round A	21.01.2018	CSI-W5*	4	1.60	390	221	14	30.04.2004	G	KWPN	281	20	121	2	38	1958	103	M	12	10	no	spread	oxer	left	no	0	0	66,28	no
Leipzig	The Longines FEI World Cup Jumping - Leipzig round A	21.01.2018	CSI-W5*	4	1.60	390	221	14	30.04.2004	G	KWPN	281	20	121	2	38	1958	103	M	13	11a	yes	spread	oxer	right	no	0	0	66,28	no
Leipzig	The Longines FEI World Cup Jumping - Leipzig round A	21.01.2018	CSI-W5*	4	1.60	390	221	14	30.04.2004	G	KWPN	281	20	121	2	38	1958	103	M	14	11b	yes	vertical	vertical	right	no	0	0	66,28	no
Leipzig	The Longines FEI World Cup Jumping - Leipzig round A	21.01.2018	CSI-W5*	4	1.60	390	221	14	30.04.2004	G	KWPN	281	20	121	2	38	1958	103	M	15	12	no	vertical	vertical	right	no	0	0	66,28	no
Leipzig	The Longines FEI World Cup Jumping - Leipzig round B	21.01.2018	CSI-W5*	4	1.60	300	221	14	30.04.2004	G	KWPN	281	20	121	2	38	1958	103	M	1	1	no	vertical	vertical	left	no	0	0	44,28	no
Leipzig	The Longines FEI World Cup Jumping - Leipzig round B	21.01.2018	CSI-W5*	4	1.60	300	221	14	30.04.2004	G	KWPN	281	20	121	2	38	1958	103	M	2	2	no	spread	oxer	left	no	0	0	44,28	no
Leipzig	The Longines FEI World Cup Jumping - Leipzig round B	21.01.2018	CSI-W5*	4	1.60	300	221	14	30.04.2004	G	KWPN	281	20	121	2	38	1958	103	M	3	3	no	vertical	vertical	right	no	0	0	44,28	no
Leipzig	The Longines FEI World Cup Jumping - Leipzig round B	21.01.2018	CSI-W5*	4	1.60	300	221	14	30.04.2004	G	KWPN	281	20	121	2	38	1958	103	M	4	4a	yes	vertical	vertical	right	no	0	0	44,28	no
Leipzig	The Longines FEI World Cup Jumping - Leipzig round B	21.01.2018	CSI-W5*	4	1.60	300	221	14	30.04.2004	G	KWPN	281	20	121	2	38	1958	103	M	5	4b	yes	spread	oxer	right	no	0	0	44,28	no
Leipzig	The Longines FEI World Cup Jumping - Leipzig round B	21.01.2018	CSI-W5*	4	1.60	300	221	14	30.04.2004	G	KWPN	281	20	121	2	38	1958	103	M	6	5	no	spread	oxer	left	no	0	0	44,28	no

Leipzig	The Longines FEI World Cup Jumping - Leipzig round B	21.01.2018	CSI-W5*	4	1.60	300	221	14	30.04.2004	G	KWPN	281	20	121	2	38	1958	103	M	7	6	no	vertical	vertical	left	no	0	0	44,28	no
Leipzig	The Longines FEI World Cup Jumping - Leipzig round B	21.01.2018	CSI-W5*	4	1.60	300	221	14	30.04.2004	G	KWPN	281	20	121	2	38	1958	103	M	8	7	no	vertical	vertical	right	no	0	0	44,28	no
Leipzig	The Longines FEI World Cup Jumping - Leipzig round B	21.01.2018	CSI-W5*	4	1.60	300	221	14	30.04.2004	G	KWPN	281	20	121	2	38	1958	103	M	9	8	no	spread	oxer	right	no	0	0	44,28	no
Leipzig	The Longines FEI World Cup Jumping - Leipzig round A	21.01.2018	CSI-W5*	4	1.60	390	142	10	01.06.2008	S	ISH	154	2	124	293	24	1007	0	M	1	1	no	vertical	vertical	left	no	0	0	70,29	no
Leipzig	The Longines FEI World Cup Jumping - Leipzig round A	21.01.2018	CSI-W5*	4	1.60	390	142	10	01.06.2008	S	ISH	154	2	124	293	24	1007	0	M	2	2	no	spread	oxer	straight	no	0	0	70,29	no
Leipzig	The Longines FEI World Cup Jumping - Leipzig round A	21.01.2018	CSI-W5*	4	1.60	390	142	10	01.06.2008	S	ISH	154	2	124	293	24	1007	0	M	3	3	no	spread	oxer	left	no	0	0	70,29	no
Leipzig	The Longines FEI World Cup Jumping - Leipzig round A	21.01.2018	CSI-W5*	4	1.60	390	142	10	01.06.2008	S	ISH	154	2	124	293	24	1007	0	M	4	4	no	vertical	vertical	right	no	0	0	70,29	no
Leipzig	The Longines FEI World Cup Jumping - Leipzig round A	21.01.2018	CSI-W5*	4	1.60	390	142	10	01.06.2008	S	ISH	154	2	124	293	24	1007	0	M	5	5a	yes	vertical	vertical	right	no	0	0	70,29	no
Leipzig	The Longines FEI World Cup Jumping - Leipzig round A	21.01.2018	CSI-W5*	4	1.60	390	142	10	01.06.2008	S	ISH	154	2	124	293	24	1007	0	M	6	5b	yes	spread	oxer	right	yes	4	0	70,29	no
Leipzig	The Longines FEI World Cup Jumping - Leipzig round A	21.01.2018	CSI-W5*	4	1.60	390	142	10	01.06.2008	S	ISH	154	2	124	293	24	1007	0	M	7	5c	yes	spread	oxer	right	no	0	0	70,29	no
Leipzig	The Longines FEI World Cup Jumping - Leipzig round A	21.01.2018	CSI-W5*	4	1.60	390	142	10	01.06.2008	S	ISH	154	2	124	293	24	1007	0	M	8	6	no	vertical	vertical	left	yes	4	0	70,29	no
Leipzig	The Longines FEI World Cup Jumping - Leipzig round A	21.01.2018	CSI-W5*	4	1.60	390	142	10	01.06.2008	S	ISH	154	2	124	293	24	1007	0	M	9	7	no	spread	oxer with water	left	no	0	0	70,29	no
Leipzig	The Longines FEI World Cup Jumping - Leipzig round A	21.01.2018	CSI-W5*	4	1.60	390	142	10	01.06.2008	S	ISH	154	2	124	293	24	1007	0	M	10	8	no	spread	oxer	straight	yes	4	0	70,29	no
Leipzig	The Longines FEI World Cup Jumping - Leipzig round A	21.01.2018	CSI-W5*	4	1.60	390	142	10	01.06.2008	S	ISH	154	2	124	293	24	1007	0	M	11	9	no	vertical	vertical	left	no	0	0	70,29	no
Leipzig	The Longines FEI World Cup Jumping - Leipzig round A	21.01.2018	CSI-W5*	4	1.60	390	142	10	01.06.2008	S	ISH	154	2	124	293	24	1007	0	M	12	10	no	spread	oxer	left	yes	4	0	70,29	no
Leipzig	The Longines FEI World Cup Jumping - Leipzig round A	21.01.2018	CSI-W5*	4	1.60	390	142	10	01.06.2008	S	ISH	154	2	124	293	24	1007	0	M	13	11a	yes	spread	oxer	right	no	0	0	70,29	no
Leipzig	The Longines FEI World Cup Jumping - Leipzig round A	21.01.2018	CSI-W5*	4	1.60	390	142	10	01.06.2008	S	ISH	154	2	124	293	24	1007	0	M	14	11b	yes	vertical	vertical	right	no	0	0	70,29	no
Leipzig	The Longines FEI World Cup Jumping - Leipzig round A	21.01.2018	CSI-W5*	4	1.60	390	142	10	01.06.2008	S	ISH	154	2	124	293	24	1007	0	M	15	12	no	vertical	vertical	right	yes	4	1	70,29	no
Leipzig	The Longines FEI World Cup Jumping - Leipzig round A	21.01.2018	CSI-W5*	4	1.60	390	110	11	04.03.2007	G	BWP	91	3	127	615	26	880	39	M	1	1	no	vertical	vertical	left	no	0	0	72,66	no
Leipzig	The Longines FEI World Cup Jumping - Leipzig round A	21.01.2018	CSI-W5*	4	1.60	390	110	11	04.03.2007	G	BWP	91	3	127	615	26	880	39	M	2	2	no	spread	oxer	straight	no	0	0	72,66	no
Leipzig	The Longines FEI World Cup Jumping - Leipzig round A	21.01.2018	CSI-W5*	4	1.60	390	110	11	04.03.2007	G	BWP	91	3	127	615	26	880	39	M	3	3	no	spread	oxer	left	no	0	0	72,66	no
Leipzig	The Longines FEI World Cup Jumping - Leipzig round A	21.01.2018	CSI-W5*	4	1.60	390	110	11	04.03.2007	G	BWP	91	3	127	615	26	880	39	M	4	4	no	vertical	vertical	right	no	0	0	72,66	no
Leipzig	The Longines FEI World Cup Jumping - Leipzig round A	21.01.2018	CSI-W5*	4	1.60	390	110	11	04.03.2007	G	BWP	91	3	127	615	26	880	39	M	5	5a	yes	vertical	vertical	right	no	0	0	72,66	no
Leipzig	The Longines FEI World Cup Jumping - Leipzig round A	21.01.2018	CSI-W5*	4	1.60	390	110	11	04.03.2007	G	BWP	91	3	127	615	26	880	39	M	6	5b	yes	spread	oxer	right	no	0	0	72,66	no
Leipzig	The Longines FEI World Cup Jumping - Leipzig round A	21.01.2018	CSI-W5*	4	1.60	390	110	11	04.03.2007	G	BWP	91	3	127	615	26	880	39	M	7	5c	yes	spread	oxer	right	yes	4	0	72,66	no
Leipzig	The Longines FEI World Cup Jumping - Leipzig round A	21.01.2018	CSI-W5*	4	1.60	390	110	11	04.03.2007	G	BWP	91	3	127	615	26	880	39	M	8	6	no	vertical	vertical	left	no	0	0	72,66	no
Leipzig	The Longines FEI World Cup Jumping - Leipzig round A	21.01.2018	CSI-W5*	4	1.60	390	110	11	04.03.2007	G	BWP	91	3	127	615	26	880	39	M	9	7	no	spread	oxer with water	left	no	0	0	72,66	no
Leipzig	The Longines FEI World Cup Jumping - Leipzig round A	21.01.2018	CSI-W5*	4	1.60	390	110	11	04.03.2007	G	BWP	91	3	127	615	26	880	39	M	10	8	no	spread	oxer	straight	no	0	0	72,66	no
Leipzig	The Longines FEI World Cup Jumping - Leipzig round A	21.01.2018	CSI-W5*	4	1.60	390	110	11	04.03.2007	G	BWP	91	3	127	615	26	880	39	M	11	9	no	vertical	vertical	left	no	0	0	72,66	no
Leipzig	The Longines FEI World Cup Jumping - Leipzig round A	21.01.2018	CSI-W5*	4	1.60	390	110	11	04.03.2007	G	BWP	91	3	127	615	26	880	39	M	12	10	no	spread	oxer	left	no	0	0	72,66	no
Leipzig	The Longines FEI World Cup Jumping - Leipzig round A	21.01.2018	CSI-W5*	4	1.60	390	110	11	04.03.2007	G	BWP	91	3	127	615	26	880	39	M	13	11a	yes	spread	oxer	right	no	0	0	72,66	no
Leipzig	The Longines FEI World Cup Jumping - Leipzig round A	21.01.2018	CSI-W5*	4	1.60	390	110	11	04.03.2007	G	BWP	91	3	127	615	26	880	39	M	14	11b	yes	vertical	vertical	right	no	0	0	72,66	no
Leipzig	The Longines FEI World Cup Jumping - Leipzig round A	21.01.2018	CSI-W5*	4	1.60	390	110	11	04.03.2007	G	BWP	91	3	127	615	26	880	39	M	15	12	no	vertical	vertical	right	yes	4	1	72,66	no
Leipzig	The Longines FEI World Cup Jumping - Leipzig round A	21.01.2018	CSI-W5*	4	1.60	390	204	11	16.04.2007	M	SF	206	3	129	118	24	1446	43	F	1	1	no	vertical	vertical	left	yes	4	0	71,76	no
Leipzig	The Longines FEI World Cup Jumping - Leipzig round A	21.01.2018	CSI-W5*	4	1.60	390	204	11	16.04.2007	M	SF	206	3	129	118	24	1446	43	F	2	2	no	spread	oxer	straight	no	0	0	71,76	no
Leipzig	The Longines FEI World Cup Jumping - Leipzig round A	21.01.2018	CSI-W5*	4	1.60	390	204	11	16.04.2007	M	SF	206	3	129	118	24	1446	43	F	3	3	no	spread	oxer	left	no	0	0	71,76	no
Leipzig	The Longines FEI World Cup Jumping - Leipzig round A	21.01.2018	CSI-W5*	4	1.60	390	204	11	16.04.2007	M	SF	206	3	129	118	24	1446	43	F	4	4	no	vertical	vertical	right	no	0	0	71,76	no
Leipzig	The Longines FEI World Cup Jumping - Leipzig round A	21.01.2018	CSI-W5*	4	1.60	390	204	11	16.04.2007	M	SF	206	3	129	118	24	1446	43	F	5	5a	yes	vertical	vertical	right	no	0	0	71,76	no
Leipzig	The Longines FEI World Cup Jumping - Leipzig round A	21.01.2018	CSI-W5*	4	1.60	390	204	11	16.04.2007	M	SF	206	3	129	118	24	1446	43	F	6	5b	yes	spread	oxer	right	no	0	0	71,76	no
Leipzig	The Longines FEI World Cup Jumping - Leipzig round A	21.01.2018	CSI-W5*	4	1.60	390	204	11	16.04.2007	M	SF	206	3	129	118	24	1446	43	F	7	5c	yes	spread	oxer	right	no	0	0	71,76	no
Leipzig	The Longines FEI World Cup Jumping - Leipzig round A	21.01.2018	CSI-W5*	4	1.60	390	204	11	16.04.2007	M	SF	206	3	129	118	24	1446	43	F	8	6	no	vertical	vertical	left	no	0	0	71,76	no
Leipzig	The Longines FEI World Cup Jumping - Leipzig round A	21.01.2018	CSI-W5*	4	1.60	390	204	11	16.04.2007	M	SF	206	3	129	118	24	1446	43	F	9	7	no	spread	oxer with water	left	yes	4	0	71,76	no
Leipzig	The Longines FEI World Cup Jumping - Leipzig round A	21.01.2018	CSI-W5*	4	1.60	390	204	11	16.04.2007	M	SF	206	3	129	118	24	1446	43	F	10	8	no	spread	oxer	straight	no	0	0	71,76	no
Leipzig	The Longines FEI World Cup Jumping - Leipzig round A	21.01.2018	CSI-W5*	4	1.60	390	204	11	16.04.2007	M	SF	206	3	129	118	24	1446	43	F	11	9	no	vertical	vertical	left	no	0	0	71,76	no
Leipzig	The Longines FEI World Cup Jumping - Leipzig round A	21.01.2018	CSI-W5*	4	1.60	390	204	11	16.04.2007	M	SF	206	3	129	118	24	1446	43	F	12	10	no	spread	oxer	left	no	0	0	71,76	no
Leipzig	The Longines FEI World Cup Jumping - Leipzig round A	21.01.2018	CSI-W5*	4	1.60	390	204	11	16.04.2007	M	SF	206	3	129	118	24	1446	43	F	13	11a	yes	spread	oxer	right	no	0	0	71,76	no
Leipzig	The Longines FEI World Cup Jumping - Leipzig round A	21.01.2018	CSI-W5*	4	1.60	390	204	11	16.04.2007	M	SF	206	3	129	118	24	1446	43	F	14	11b	yes	vertical	vertical	right	yes	4	0	71,76	no
Leipzig	The Longines FEI World Cup Jumping - Leipzig round A	21.01.2018	CSI-W5*	4	1.60	390	204	11	16.04.2007	M	SF	206	3	129	118	24	1446	43	F	15	12	no	vertical	vertical	right	yes	4	1	71,76	no
Leipzig	The Longines FEI World Cup Jumping - Leipzig round A	21.01.2018	CSI-W5*	4	1.60	390	14	10	26.07.2008	M	ZfDP	107	0	130	88	46	1851	131	M	1	1	no	vertical	vertical	left	no	0	0	70,49	no
Leipzig	The Longines FEI World Cup Jumping - Leipzig round A	21.01.2018	CSI-W5*	4	1.60	390	14	10	26.07.2008	M	ZfDP	107	0	130	88	46	1851	131	M	2	2	no	spread	oxer	straight	no	0	0	70,49	no
Leipzig	The Longines FEI World Cup Jumping - Leipzig round A	21.01.2018	CSI-W5*	4	1.60	390	14	10	26.07.2008	M	ZfDP	107	0	130	88	46	1851	131	M	3	3	no	spread	oxer	left	no	0	0	70,49	no
Leipzig	The Longines FEI World Cup Jumping - Leipzig round A	21.01.2018	CSI-W5*	4	1.60	390	14	10	26.07.2008	M	ZfDP	107	0	130	88	46	1851	131	M	4	4	no	vertical	vertical	right	no	0	0	70,49	no
Leipzig	The Longines FEI World Cup Jumping - Leipzig round A	21.01.2018	CSI-W5*	4	1.60	390	14	10	26.07.2008	M	ZfDP	107	0	130	88	46	1851	131	M	5	5a	yes	vertical	vertical	right	no	0	0	70,49	no
Leipzig	The Longines FEI World Cup Jumping - Leipzig round A	21.01.2018	CSI-W5*	4	1.60	390	14	10	26.07.2008	M	ZfDP	107	0	130	88	46	1851	131	M	6	5b	yes	spread	oxer	right	no	0	0	70,49	no
Leipzig	The Longines FEI World Cup Jumping - Leipzig round A	21.01.2018	CSI-W5*	4	1.60	390	14	10	26.07.2008	M	ZfDP	107	0	130	88	46	1851	131	M	7	5c	yes	spread	oxer	right	no	0	0	70,49	no
Leipzig	The Longines FEI World Cup Jumping - Leipzig round A	21.01.2018	CSI-W5*	4	1.60	390	14	10	26.07.2008	M	ZfDP	107	0	130	88	46	1851	131	M	8	6	no	vertical	vertical	left	no	0	0	70,49	no
Leipzig	The Longines FEI World Cup Jumping - Leipzig round A	21.01.2018	CSI-W5*	4	1.60	390	14	10	26.07.2008	M	ZfDP	107	0	130	88	46	1851	131	M	9	7	no	spread	oxer with water	left	no	0	0	70,49	no
Leipzig	The Longines FEI World Cup Jumping - Leipzig round A	21.01.2018	CSI-W5*	4	1.60	390	14	10	26.07.2008	M	ZfDP	107	0	130	88	46	1851	131	M	10	8	no	spread	oxer	straight	no	0	0	70,49	no
Leipzig	The Longines FEI World Cup Jumping - Leipzig round A	21.01.2018	CSI-W5*	4	1.60	390	14	10	26.07.2008	M	ZfDP	107	0	130	88	46	1851	131	M	11	9	no	vertical	vertical	left	no	0	0	70,49	no
Leipzig	The Longines FEI World Cup Jumping - Leipzig round A	21.01.2018	CSI-W5*	4	1.60	390	14	10	26.07.2008	M	ZfDP	107	0	130	88	46	1851	131	M	12	10	no	spread	oxer	left	no	0	0	70,49	no
Leipzig	The Longines FEI World Cup Jumping - Leipzig round A	21.01.2018	CSI-W5*	4	1.60	390	14	10	26.07.2008	M	ZfDP	107	0	130	88	46	1851	131	M	13	11a	yes	spread	oxer	right	no	0	0	70,49	no
Leipzig	The Longines FEI World Cup Jumping - Leipzig round A	21.01.2018	CSI-W5*	4	1.60	390	14	10	26.07.2008	M	ZfDP	107	0	130	88	46	1851	131	M	14	11b	yes	vertical	vertical	right	no	0	0	70,49	no
Leipzig	The Longines FEI World Cup Jumping - Leipzig round A	21.01.2018	CSI-W5*	4	1.60	390	14	10	26.07.2008	M	ZfDP	107	0	130	88	46	1851	131	M	15	12	no	vertical	vertical	right	no	0	1	70,49	no
Leipzig	The Longines FEI World Cup Jumping - Leipzig round A	21.01.2018	CSI-W5*	4	1.60	390	141	9	06.04.2009	G	OLDBG	43	2	131	160	36	1400	81	M	1	1	no	vertical	vertical	left	no	0	0	69,58	no
Leipzig	The Longines FEI World Cup Jumping - Leipzig round A	21.01.2018	CSI-W5*	4	1.60	390	141	9	06.04.2009	G	OLDBG	43	2	131	160	36	1400	81	M	2	2	no	spread	oxer	straight	no	0	0	69,58	no
Leipzig	The Longines FEI World Cup Jumping - Leipzig round A	21.01.2018	CSI-W5*	4	1.60	390	141	9	06.04.2009	G	OLDBG	43	2	131	160	36	1400	81	M	3	3	no	spread	oxer	left	no	0	0	69,58	no
Leipzig	The Longines FEI World Cup Jumping - Leipzig round A	21.01.2018	CSI-W5*	4	1.60	390	141	9	06.04.2009	G	OLDBG	43	2	131	160	36	1400	81	M	4	4	no	vertical	vertical	right	no	0	0	69,58	no
Leipzig	The Longines FEI World Cup Jumping - Leipzig round A	21.01.2018	CSI-W5*	4	1.60	390	141	9	06.04.2009	G	OLDBG	43	2	131	160	36	1400	81	M	5	5a	yes	vertical	vertical	right	no	0	0	69,58	no
Leipzig	The Longines FEI World Cup Jumping - Leipzig round A	21.01.2018	CSI-W5*	4	1.60	390	141	9	06.04.2009	G	OLDBG	43	2	131	160	36	1400	81	M	6	5b	yes	spread	oxer	right	no	0	0	69,58	no
Leipzig	The Longines FEI World Cup Jumping - Leipzig round A	21.01.2018	CSI-W5*	4	1.60	390	141	9	06.04.2009	G	OLDBG	43	2	131	160	36	1400	81	M	7	5c	yes	spread	oxer	right	no	0	0	69,58	no
Leipzig	The Longines FEI World Cup Jumping - Leipzig round A	21.01.2018	CSI-W5*	4	1.60	390	141	9	06.04.2009	G	OLDBG	43	2	131	160	36	1400	81	M	8	6	no	vertical	vertical	left	no	0	0	69,58	no
Leipzig	The Longines FEI World Cup Jumping - Leipzig round A	21.01.2018	CSI-W5*	4	1.60	390	141	9	06.04.2009	G	OLDBG	43	2	131	160	36	1400	81	M	9	7	no	spread	oxer with water	left	no	0	0	69,58	no
Leipzig	The Longines FEI World Cup Jumping - Leipzig round A	21.01.2018	CSI-W5*	4	1.60	390	141	9	06.04.2009	G	OLDBG	43	2	131	160	36	1400	81	M	10	8	no	spread	oxer	straight	no	0	0	69,58	no
Leipzig	The Longines FEI World Cup Jumping - Leipzig round A	21.01.2018	CSI-W5*	4	1.60	390	141	9	06.04.2009	G	OLDBG	43	2	131	160	36	1400	81	M	11	9	no	vertical	vertical	left	no	0	0	69,58	no
Leipzig	The Longines FEI World Cup Jumping - Leipzig round A	21.01.2018	CSI-W5*	4	1.60	390	141	9	06.04.2009	G	OLDBG	43	2	131	160	36	1400	81	M	12	10	no	spread	oxer	left	no	0	0	69,58	no
Leipzig	The Longines FEI World Cup Jumping - Leipzig round A	21.01.2018	CSI-W5*	4	1.60	390	141	9	06.04.2009	G	OLDBG	43	2	131	160	36	1400	81	M	13	11a	yes	spread	oxer	right	no	0	0	69,58	no
Leipzig	The Longines FEI World Cup Jumping - Leipzig round A	21.01.2018	CSI-W5*	4	1.60	390	141	9	06.04.2009	G	OLDBG	43	2	131	160	36	1400	81	M	14	11b	yes	vertical	vertical	right	no	0	0	69,58	no
Leipzig	The Longines FEI World Cup Jumping - Leipzig round A	21.01.2018	CSI-W5*	4	1.60	390	141	9	06.04.2009	G	OLDBG	43	2	131	160	36	1400	81	M	15	12	no	vertical	vertical	right	yes	4	0	69,58	no
Leipzig	The Longines FEI World Cup Jumping - Leipzig round A	21.01.2018	CSI-W5*	4	1.60	390	91	12	02.06.2006	M	SWB	168	4	132	216	27	588	22	F	1	1	no	vertical	vertical	left	no	0	0	68,9	no
Leipzig	The Longines FEI World Cup Jumping - Leipzig round A	21.01.2018	CSI-W5*	4	1.60	390	91	12	02.06.2006	M	SWB	168	4	132	216	27	588	22	F	2	2	no	spread	oxer	straight	no	0	0	68,9	no
Leipzig	The Longines FEI World Cup Jumping - Leipzig round A	21.01.2018	CSI-W5*	4	1.60	390	91	12	02.06.2006	M	SWB	168	4	132	216	27	588	22	F	3	3	no	spread	oxer	left	no	0	0	68,9	no
Leipzig	The Longines FEI World Cup Jumping - Leipzig round A	21.01.2018	CSI-W5*	4	1.60	390	91	12	02.06.2006	M	SWB	168	4	132	216	27	588	22	F	4	4	no	vertical	vertical	right	no	0	0	68,9	no
Leipzig	The Longines FEI World Cup Jumping - Leipzig round A	21.01.2018	CSI-W5*	4	1.60	390	91	12	02.06.2006	M	SWB	168	4	132	216	27	588	22	F	5	5a	yes	vertical	vertical	right	no	0	0	68,9	no
Leipzig	The Longines FEI World Cup Jumping - Leipzig round A	21.01.2018	CSI-W5*	4	1.60	390	91	12	02.06.2006	M	SWB	168	4	132	216	27	588	22	F	6	5b	yes	spread	oxer	right	no	0	0	68,9	no
Leipzig	The Longines FEI World Cup Jumping - Leipzig round A	21.01.2018	CSI-W5*	4	1.60	390	91	12	02.06.2006	M	SWB	168	4	132	216	27	588	22	F	7	5c	yes	spread	oxer	right	no	0	0	68,9	no
Leipzig	The Longines FEI World Cup Jumping - Leipzig round A	21.01.2018	CSI-W5*	4	1.60	390	91	12	02.06.2006	M	SWB	168	4	132	216	27	588	22	F	8	6	no	vertical	vertical	left	no	0	0	68,9	no
Leipzig	The Longines FEI World Cup Jumping - Leipzig round A	21.01.2018	CSI-W5*	4	1.60	390	91	12	02.06.2006	M	SWB	168	4	132	216	27	588	22	F	9	7	no	spread	oxer with water	left	no	0	0	68,9	no
Leipzig	The Longines FEI World Cup Jumping - Leipzig round A	21.01.2018	CSI-W5*	4	1.60	390	91	12	02.06.2006	M	SWB	168	4	132	216	27	588	22	F	10	8	no	spread	oxer	straight	yes	4	0	68,9	no
Leipzig	The Longines FEI World Cup Jumping - Leipzig round A	21.01.2018	CSI-W5*	4	1.60	390	91	12	02.06.2006	M	SWB	168	4	132	216	27	588	22	F	11	9	no	vertical	vertical	left	no	0	0	68,9	no
Leipzig	The Longines FEI World Cup Jumping - Leipzig round A	21.01.2018	CSI-W5*	4	1.60	390	91	12	02.06.2006	M	SWB	168	4	132	216	27	588	22	F	12	10	no	spread	oxer	left	no	0	0	68,9	no
Leipzig	The Longines FEI World Cup Jumping - Leipzig round A	21.01.2018	CSI-W5*	4	1.60	390	91	12	02.06.2006	M	SWB	168	4	132	216	27	588	22	F	13	11a	yes	spread	oxer	right	no	0	0	68,9	no
Leipzig	The Longines FEI World Cup Jumping - Leipzig round A	21.01.2018	CSI-W5*	4	1.60	390	91	12	02.06.2006	M	SWB	168	4	132	216	27	588	22	F	14	11b	yes	vertical	vertical	right	yes	4	0	68,9	no
Leipzig	The Longines FEI World Cup Jumping - Leipzig round A	21.01.2018	CSI-W5*	4	1.60	390	91	12	02.06.2006	M	SWB	168	4	132	216	27	588	22	F	15	12	no	vertical	vertical	right	no	0	0	68,9	no
Leipzig	The Longines FEI World Cup Jumping - Leipzig round A	21.01.2018	CSI-W5*	4	1.60	390	51	14	08.04.2005	G	HOLST	225	7	133	16	26	1689	128	M	1	1	no	vertical	vertical	left	no	0	0	68,7	no
Leipzig	The Longines FEI World Cup Jumping - Leipzig round A	21.01.2018	CSI-W5*	4	1.60	390	51	14	08.04.2005	G	HOLST	225	7	133	16	26	1689	128	M	2	2	no	spread	oxer	straight	no	0	0	68,7	no
Leipzig	The Longines FEI World Cup Jumping - Leipzig round A	21.01.2018	CSI-W5*	4	1.60	390	51	14	08.04.2005	G	HOLST	225	7	133	16	26	1689	128	M	3	3	no	spread	oxer	left	no	0	0	68,7	no
Leipzig	The Longines FEI World Cup Jumping - Leipzig round A	21.01.2018	CSI-W5*	4	1.60	390	51	14	08.04.2005	G	HOLST	225	7	133	16	26	1689	128	M	4	4	no	vertical	vertical	right	no	0	0	68,7	no
Leipzig	The Longines FEI World Cup Jumping - Leipzig round A	21.01.2018	CSI-W5*	4	1.60	390	51	14	08.04.2005	G	HOLST	225	7	133	16	26	1689	128	M	5	5a	yes	vertical	vertical	right	no	0	0	68,7	no
Leipzig	The Longines FEI World Cup Jumping - Leipzig round A	21.01.2018	CSI-W5*	4	1.60	390	51	14	08.04.2005	G	HOLST	225	7	133	16	26	1689	128	M	6	5b	yes	spread	oxer	right	no	0	0	68,7	no
Leipzig	The Longines FEI World Cup Jumping - Leipzig round A	21.01.2018	CSI-W5*	4	1.60	390	51	14	08.04.2005	G	HOLST	225	7	133	16	26	1689	128	M	7	5c	yes	spread	oxer	right	no	0	0	68,7	no
Leipzig	The Longines FEI World Cup Jumping - Leipzig round A	21.01.2018	CSI-W5*	4	1.60	390	51	14	08.04.2005	G	HOLST	225	7	133	16	26	1689	128	M	8	6	no	vertical	vertical	left	no	0	0	68,7	no
Leipzig	The Longines FEI World Cup Jumping - Leipzig round A	21.01.2018	CSI-W5*	4	1.60	390	51	14	08.04.2005	G	HOLST	225	7	133	16	26	1689	128	M	9	7	no	spread	oxer with water	left	no	0	0	68,7	no
Leipzig	The Longines FEI World Cup Jumping - Leipzig round A	21.01.2018	CSI-W5*	4	1.60	390	51	14	08.04.2005	G	HOLST	225	7	133	16	26	1689	128	M	10	8	no	spread	oxer	straight	no	0	0	68,7	no
Leipzig	The Longines FEI World Cup Jumping - Leipzig round A	21.01.2018	CSI-W5*	4	1.60	390	51	14	08.04.2005	G	HOLST	225	7	133	16	26	1689	128	M	11	9	no	vertical	vertical	left	no	0	0	68,7	no
Leipzig	The Longines FEI World Cup Jumping - Leipzig round A	21.01.2018	CSI-W5*	4	1.60	390	51	14	08.04.2005	G	HOLST	225	7	133	16	26	1689	128	M	12	10	no	spread	oxer	left	no	0	0	68,7	no
Leipzig	The Longines FEI World Cup Jumping - Leipzig round A	21.01.2018	CSI-W5*	4	1.60	390	51	14	08.04.2005	G	HOLST	225	7	133	16	26	1689	128	M	13	11a	yes	spread	oxer	right	no	0	0	68,7	no
Leipzig	The Longines FEI World Cup Jumping - Leipzig round A	21.01.2018	CSI-W5*	4	1.60	390	51	14	08.04.2005	G	HOLST	225	7	133	16	26	1689	128	M	14	11b	yes	vertical	vertical	right	no	0	0	68,7	no
Leipzig	The Longines FEI World Cup Jumping - Leipzig round A	21.01.2018	CSI-W5*	4	1.60	390	51	14	08.04.2005	G	HOLST	225	7	133	16	26	1689	128	M	15	12	no	vertical	vertical	right	no	0	0	68,7	no
Leipzig	The Longines FEI World Cup Jumping - Leipzig round B	21.01.2018	CSI-W5*	4	1.60	300	51	14	08.04.2005	G	HOLST	225	7	133	16	26	1689	128	M	1	1	no	vertical	vertical	left	no	0	0	44,02	no
Leipzig	The Longines FEI World Cup Jumping - Leipzig round B	21.01.2018	CSI-W5*	4	1.60	300	51	14	08.04.2005	G	HOLST	225	7	133	16	26	1689	128	M	2	2	no	spread	oxer	left	no	0	0	44,02	no
Leipzig	The Longines FEI World Cup Jumping - Leipzig round B	21.01.2018	CSI-W5*	4	1.60	300	51	14	08.04.2005	G	HOLST	225	7	133	16	26	1689	128	M	3	3	no	vertical	vertical	right	no	0	0	44,02	no
Leipzig	The Longines FEI World Cup Jumping - Leipzig round B	21.01.2018	CSI-W5*	4	1.60	300	51	14	08.04.2005	G	HOLST	225	7	133	16	26	1689	128	M	4	4a	yes	vertical	vertical	right	no	0	0	44,02	no
Leipzig	The Longines FEI World Cup Jumping - Leipzig round B	21.01.2018	CSI-W5*	4	1.60	300	51	14	08.04.2005	G	HOLST	225	7	133	16	26	1689	128	M	5	4b	yes	spread	oxer	right	no	0	0	44,02	no
Leipzig	The Longines FEI World Cup Jumping - Leipzig round B	21.01.2018	CSI-W5*	4	1.60	300	51	14	08.04.2005	G	HOLST	225	7	133	16	26	1689	128	M	6	5	no	spread	oxer	left	yes	4	0	44,02	no
Leipzig	The Longines FEI World Cup Jumping - Leipzig round B	21.01.2018	CSI-W5*	4	1.60	300	51	14	08.04.2005	G	HOLST	225	7	133	16	26	1689	128	M	7	6	no	vertical	vertical	left	no	0	0	44,02	no
Leipzig	The Longines FEI World Cup Jumping - Leipzig round B	21.01.2018	CSI-W5*	4	1.60	300	51	14	08.04.2005	G	HOLST	225	7	133	16	26	1689	128	M	8	7	no	vertical	vertical	right	no	0	0	44,02	no
Leipzig	The Longines FEI World Cup Jumping - Leipzig round B	21.01.2018	CSI-W5*	4	1.60	300	51	14	08.04.2005	G	HOLST	225	7	133	16	26	1689	128	M	9	8	no	spread	oxer	right	no	0	0	44,02	no
Leipzig	The Longines FEI World Cup Jumping - Leipzig round A	21.01.2018	CSI-W5*	4	1.60	390	121	13	27.03.2005	G	OS	90	0	134	329	24	1159	28	M	1	1	no	vertical	vertical	left	no	0	0	68,7	no
Leipzig	The Longines FEI World Cup Jumping - Leipzig round A	21.01.2018	CSI-W5*	4	1.60	390	121	13	27.03.2005	G	OS	90	0	134	329	24	1159	28	M	2	2	no	spread	oxer	straight	no	0	0	68,7	no
Leipzig	The Longines FEI World Cup Jumping - Leipzig round A	21.01.2018	CSI-W5*	4	1.60	390	121	13	27.03.2005	G	OS	90	0	134	329	24	1159	28	M	3	3	no	spread	oxer	left	no	0	0	68,7	no
Leipzig	The Longines FEI World Cup Jumping - Leipzig round A	21.01.2018	CSI-W5*	4	1.60	390	121	13	27.03.2005	G	OS	90	0	134	329	24	1159	28	M	4	4	no	vertical	vertical	right	no	0	0	68,7	no
Leipzig	The Longines FEI World Cup Jumping - Leipzig round A	21.01.2018	CSI-W5*	4	1.60	390	121	13	27.03.2005	G	OS	90	0	134	329	24	1159	28	M	5	5a	yes	vertical	vertical	right	no	0	0	68,7	no
Leipzig	The Longines FEI World Cup Jumping - Leipzig round A	21.01.2018	CSI-W5*	4	1.60	390	121	13	27.03.2005	G	OS	90	0	134	329	24	1159	28	M	6	5b	yes	spread	oxer	right	no	0	0	68,7	no
Leipzig	The Longines FEI World Cup Jumping - Leipzig round A	21.01.2018	CSI-W5*	4	1.60	390	121	13	27.03.2005	G	OS	90	0	134	329	24	1159	28	M	7	5c	yes	spread	oxer	right	no	0	0	68,7	no
Leipzig	The Longines FEI World Cup Jumping - Leipzig round A	21.01.2018	CSI-W5*	4	1.60	390	121	13	27.03.2005	G	OS	90	0	134	329	24	1159	28	M	8	6	no	vertical	vertical	left	no	0	0	68,7	no
Leipzig	The Longines FEI World Cup Jumping - Leipzig round A	21.01.2018	CSI-W5*	4	1.60	390	121	13	27.03.2005	G	OS	90	0	134	329	24	1159	28	M	9	7	no	spread	oxer with water	left	no	0	0	68,7	no
Leipzig	The Longines FEI World Cup Jumping - Leipzig round A	21.01.2018	CSI-W5*	4	1.60	390	121	13	27.03.2005	G	OS	90	0	134	329	24	1159	28	M	10	8	no	spread	oxer	straight	no	0	0	68,7	no
Leipzig	The Longines FEI World Cup Jumping - Leipzig round A	21.01.2018	CSI-W5*	4	1.60	390	121	13	27.03.2005	G	OS	90	0	134	329	24	1159	28	M	11	9	no	vertical	vertical	left	no	0	0	68,7	no
Leipzig	The Longines FEI World Cup Jumping - Leipzig round A	21.01.2018	CSI-W5*	4	1.60	390	121	13	27.03.2005	G	OS	90	0	134	329	24	1159	28	M	12	10	no	spread	oxer	left	no	0	0	68,7	no
Leipzig	The Longines FEI World Cup Jumping - Leipzig round A	21.01.2018	CSI-W5*	4	1.60	390	121	13	27.03.2005	G	OS	90	0	134	329	24	1159	28	M	13	11a	yes	spread	oxer	right	no	0	0	68,7	no
Leipzig	The Longines FEI World Cup Jumping - Leipzig round A	21.01.2018	CSI-W5*	4	1.60	390	121	13	27.03.2005	G	OS	90	0	134	329	24	1159	28	M	14	11b	yes	vertical	vertical	right	no	0	0	68,7	no
Leipzig	The Longines FEI World Cup Jumping - Leipzig round A	21.01.2018	CSI-W5*	4	1.60	390	121	13	27.03.2005	G	OS	90	0	134	329	24	1159	28	M	15	12	no	vertical	vertical	right	no	0	0	68,7	no
Leipzig	The Longines FEI World Cup Jumping - Leipzig round B	21.01.2018	CSI-W5*	4	1.60	300	121	13	27.03.2005	G	OS	90	0	134	329	24	1159	28	M	1	1	no	vertical	vertical	left	no	0	0	48,16	no
Leipzig	The Longines FEI World Cup Jumping - Leipzig round B	21.01.2018	CSI-W5*	4	1.60	300	121	13	27.03.2005	G	OS	90	0	134	329	24	1159	28	M	2	2	no	spread	oxer	left	no	0	0	48,16	no
Leipzig	The Longines FEI World Cup Jumping - Leipzig round B	21.01.2018	CSI-W5*	4	1.60	300	121	13	27.03.2005	G	OS	90	0	134	329	24	1159	28	M	3	3	no	vertical	vertical	right	no	0	0	48,16	no
Leipzig	The Longines FEI World Cup Jumping - Leipzig round B	21.01.2018	CSI-W5*	4	1.60	300	121	13	27.03.2005	G	OS	90	0	134	329	24	1159	28	M	4	4a	yes	vertical	vertical	right	no	0	0	48,16	no
Leipzig	The Longines FEI World Cup Jumping - Leipzig round B	21.01.2018	CSI-W5*	4	1.60	300	121	13	27.03.2005	G	OS	90	0	134	329	24	1159	28	M	5	4b	yes	spread	oxer	right	yes	4	0	48,16	no
Leipzig	The Longines FEI World Cup Jumping - Leipzig round B	21.01.2018	CSI-W5*	4	1.60	300	121	13	27.03.2005	G	OS	90	0	134	329	24	1159	28	M	6	5	no	spread	oxer	left	no	0	0	48,16	no
Leipzig	The Longines FEI World Cup Jumping - Leipzig round B	21.01.2018	CSI-W5*	4	1.60	300	121	13	27.03.2005	G	OS	90	0	134	329	24	1159	28	M	7	6	no	vertical	vertical	left	no	0	0	48,16	no
Leipzig	The Longines FEI World Cup Jumping - Leipzig round B	21.01.2018	CSI-W5*	4	1.60	300	121	13	27.03.2005	G	OS	90	0	134	329	24	1159	28	M	8	7	no	vertical	vertical	right	no	0	0	48,16	no
Leipzig	The Longines FEI World Cup Jumping - Leipzig round B	21.01.2018	CSI-W5*	4	1.60	300	121	13	27.03.2005	G	OS	90	0	134	329	24	1159	28	M	9	8	no	spread	oxer	right	no	0	0	48,16	no
Zurich	The Longines FEI World Cup Jumping - Zurich round A	28.01.2018	CSI-W5*	3	1.60	430	137	12	23.06.2006	M	AES	123	7	7	48	58	1857	49	M	1	1	no	vertical	vertical	right	no	0	0	73,75	no
Zurich	The Longines FEI World Cup Jumping - Zurich round A	28.01.2018	CSI-W5*	3	1.60	430	137	12	23.06.2006	M	AES	123	7	7	48	58	1857	49	M	2	2	no	spread	oxer	right	no	0	0	73,75	no
Zurich	The Longines FEI World Cup Jumping - Zurich round A	28.01.2018	CSI-W5*	3	1.60	430	137	12	23.06.2006	M	AES	123	7	7	48	58	1857	49	M	3	3	no	vertical	wall	right	no	0	0	73,75	no
Zurich	The Longines FEI World Cup Jumping - Zurich round A	28.01.2018	CSI-W5*	3	1.60	430	137	12	23.06.2006	M	AES	123	7	7	48	58	1857	49	M	4	4	no	spread	oxer with water	left	no	0	0	73,75	no
Zurich	The Longines FEI World Cup Jumping - Zurich round A	28.01.2018	CSI-W5*	3	1.60	430	137	12	23.06.2006	M	AES	123	7	7	48	58	1857	49	M	5	5	no	vertical	vertical	left	no	0	0	73,75	no
Zurich	The Longines FEI World Cup Jumping - Zurich round A	28.01.2018	CSI-W5*	3	1.60	430	137	12	23.06.2006	M	AES	123	7	7	48	58	1857	49	M	6	6a	yes	spread	oxer	left	no	0	0	73,75	no
Zurich	The Longines FEI World Cup Jumping - Zurich round A	28.01.2018	CSI-W5*	3	1.60	430	137	12	23.06.2006	M	AES	123	7	7	48	58	1857	49	M	7	6b	yes	spread	oxer	left	no	0	0	73,75	no
Zurich	The Longines FEI World Cup Jumping - Zurich round A	28.01.2018	CSI-W5*	3	1.60	430	137	12	23.06.2006	M	AES	123	7	7	48	58	1857	49	M	8	6c	yes	vertical	vertical	left	no	0	0	73,75	no
Zurich	The Longines FEI World Cup Jumping - Zurich round A	28.01.2018	CSI-W5*	3	1.60	430	137	12	23.06.2006	M	AES	123	7	7	48	58	1857	49	M	9	7	no	spread	oxer	right	no	0	0	73,75	no
Zurich	The Longines FEI World Cup Jumping - Zurich round A	28.01.2018	CSI-W5*	3	1.60	430	137	12	23.06.2006	M	AES	123	7	7	48	58	1857	49	M	10	8a	yes	vertical	vertical	right	no	0	0	73,75	no
Zurich	The Longines FEI World Cup Jumping - Zurich round A	28.01.2018	CSI-W5*	3	1.60	430	137	12	23.06.2006	M	AES	123	7	7	48	58	1857	49	M	11	8b	yes	spread	oxer	right	no	0	0	73,75	no
Zurich	The Longines FEI World Cup Jumping - Zurich round A	28.01.2018	CSI-W5*	3	1.60	430	137	12	23.06.2006	M	AES	123	7	7	48	58	1857	49	M	12	9	no	vertical	vertical	right	no	0	0	73,75	no
Zurich	The Longines FEI World Cup Jumping - Zurich round A	28.01.2018	CSI-W5*	3	1.60	430	137	12	23.06.2006	M	AES	123	7	7	48	58	1857	49	M	13	10	no	spread	triple bar	right	no	0	0	73,75	no
Zurich	The Longines FEI World Cup Jumping - Zurich round A	28.01.2018	CSI-W5*	3	1.60	430	137	12	23.06.2006	M	AES	123	7	7	48	58	1857	49	M	14	11	no	spread	oxer	left	no	0	0	73,75	no
Zurich	The Longines FEI World Cup Jumping - Zurich round A	28.01.2018	CSI-W5*	3	1.60	430	137	12	23.06.2006	M	AES	123	7	7	48	58	1857	49	M	15	12	no	vertical	vertical with water	left	no	0	0	73,75	no
Zurich	The Longines FEI World Cup Jumping - Zurich round A	28.01.2018	CSI-W5*	3	1.60	430	137	12	23.06.2006	M	AES	123	7	7	48	58	1857	49	M	16	13	no	spread	oxer	straight	no	0	0	73,75	no
Zurich	The Longines FEI World Cup Jumping - Zurich round B	28.01.2018	CSI-W5*	3	1.60	300	137	12	23.06.2006	M	AES	123	7	7	48	58	1857	49	M	1	1	no	spread	oxer	left	no	0	0	40,85	no
Zurich	The Longines FEI World Cup Jumping - Zurich round B	28.01.2018	CSI-W5*	3	1.60	300	137	12	23.06.2006	M	AES	123	7	7	48	58	1857	49	M	2	2	no	vertical	vertical	left	no	0	0	40,85	no
Zurich	The Longines FEI World Cup Jumping - Zurich round B	28.01.2018	CSI-W5*	3	1.60	300	137	12	23.06.2006	M	AES	123	7	7	48	58	1857	49	M	3	3	no	spread	oxer	left	no	0	0	40,85	no
Zurich	The Longines FEI World Cup Jumping - Zurich round B	28.01.2018	CSI-W5*	3	1.60	300	137	12	23.06.2006	M	AES	123	7	7	48	58	1857	49	M	4	4a	yes	vertical	vertical	right	no	0	0	40,85	no
Zurich	The Longines FEI World Cup Jumping - Zurich round B	28.01.2018	CSI-W5*	3	1.60	300	137	12	23.06.2006	M	AES	123	7	7	48	58	1857	49	M	5	4b	yes	spread	oxer	right	no	0	0	40,85	no
Zurich	The Longines FEI World Cup Jumping - Zurich round B	28.01.2018	CSI-W5*	3	1.60	300	137	12	23.06.2006	M	AES	123	7	7	48	58	1857	49	M	6	5	no	vertical	vertical	right	no	0	0	40,85	no
Zurich	The Longines FEI World Cup Jumping - Zurich round B	28.01.2018	CSI-W5*	3	1.60	300	137	12	23.06.2006	M	AES	123	7	7	48	58	1857	49	M	7	6	no	vertical	vertical	right	no	0	0	40,85	no
Zurich	The Longines FEI World Cup Jumping - Zurich round B	28.01.2018	CSI-W5*	3	1.60	300	137	12	23.06.2006	M	AES	123	7	7	48	58	1857	49	M	8	7	no	spread	oxer	left	no	0	0	40,85	no
Zurich	The Longines FEI World Cup Jumping - Zurich round B	28.01.2018	CSI-W5*	3	1.60	300	137	12	23.06.2006	M	AES	123	7	7	48	58	1857	49	M	9	8	no	spread	oxer with water	left	no	0	0	40,85	no
Zurich	The Longines FEI World Cup Jumping - Zurich round A	28.01.2018	CSI-W5*	3	1.60	430	159	11	15.04.2007	M	PZHK	161	4	11	414	32	1331	18	F	1	1	no	vertical	vertical	right	no	0	0	70,86	no
Zurich	The Longines FEI World Cup Jumping - Zurich round A	28.01.2018	CSI-W5*	3	1.60	430	159	11	15.04.2007	M	PZHK	161	4	11	414	32	1331	18	F	2	2	no	spread	oxer	right	no	0	0	70,86	no
Zurich	The Longines FEI World Cup Jumping - Zurich round A	28.01.2018	CSI-W5*	3	1.60	430	159	11	15.04.2007	M	PZHK	161	4	11	414	32	1331	18	F	3	3	no	vertical	wall	right	no	0	0	70,86	no
Zurich	The Longines FEI World Cup Jumping - Zurich round A	28.01.2018	CSI-W5*	3	1.60	430	159	11	15.04.2007	M	PZHK	161	4	11	414	32	1331	18	F	4	4	no	spread	oxer with water	left	no	0	0	70,86	no
Zurich	The Longines FEI World Cup Jumping - Zurich round A	28.01.2018	CSI-W5*	3	1.60	430	159	11	15.04.2007	M	PZHK	161	4	11	414	32	1331	18	F	5	5	no	vertical	vertical	left	no	0	0	70,86	no
Zurich	The Longines FEI World Cup Jumping - Zurich round A	28.01.2018	CSI-W5*	3	1.60	430	159	11	15.04.2007	M	PZHK	161	4	11	414	32	1331	18	F	6	6a	yes	spread	oxer	left	no	0	0	70,86	no
Zurich	The Longines FEI World Cup Jumping - Zurich round A	28.01.2018	CSI-W5*	3	1.60	430	159	11	15.04.2007	M	PZHK	161	4	11	414	32	1331	18	F	7	6b	yes	spread	oxer	left	no	0	0	70,86	no
Zurich	The Longines FEI World Cup Jumping - Zurich round A	28.01.2018	CSI-W5*	3	1.60	430	159	11	15.04.2007	M	PZHK	161	4	11	414	32	1331	18	F	8	6c	yes	vertical	vertical	left	no	0	0	70,86	no
Zurich	The Longines FEI World Cup Jumping - Zurich round A	28.01.2018	CSI-W5*	3	1.60	430	159	11	15.04.2007	M	PZHK	161	4	11	414	32	1331	18	F	9	7	no	spread	oxer	right	no	0	0	70,86	no
Zurich	The Longines FEI World Cup Jumping - Zurich round A	28.01.2018	CSI-W5*	3	1.60	430	159	11	15.04.2007	M	PZHK	161	4	11	414	32	1331	18	F	10	8a	yes	vertical	vertical	right	yes	4	0	70,86	no
Zurich	The Longines FEI World Cup Jumping - Zurich round A	28.01.2018	CSI-W5*	3	1.60	430	159	11	15.04.2007	M	PZHK	161	4	11	414	32	1331	18	F	11	8b	yes	spread	oxer	right	no	0	0	70,86	no
Zurich	The Longines FEI World Cup Jumping - Zurich round A	28.01.2018	CSI-W5*	3	1.60	430	159	11	15.04.2007	M	PZHK	161	4	11	414	32	1331	18	F	12	9	no	vertical	vertical	right	no	0	0	70,86	no
Zurich	The Longines FEI World Cup Jumping - Zurich round A	28.01.2018	CSI-W5*	3	1.60	430	159	11	15.04.2007	M	PZHK	161	4	11	414	32	1331	18	F	13	10	no	spread	triple bar	right	no	0	0	70,86	no
Zurich	The Longines FEI World Cup Jumping - Zurich round A	28.01.2018	CSI-W5*	3	1.60	430	159	11	15.04.2007	M	PZHK	161	4	11	414	32	1331	18	F	14	11	no	spread	oxer	left	no	0	0	70,86	no
Zurich	The Longines FEI World Cup Jumping - Zurich round A	28.01.2018	CSI-W5*	3	1.60	430	159	11	15.04.2007	M	PZHK	161	4	11	414	32	1331	18	F	15	12	no	vertical	vertical with water	left	yes	4	0	70,86	no
Zurich	The Longines FEI World Cup Jumping - Zurich round A	28.01.2018	CSI-W5*	3	1.60	430	159	11	15.04.2007	M	PZHK	161	4	11	414	32	1331	18	F	16	13	no	spread	oxer	straight	no	0	0	70,86	no
Zurich	The Longines FEI World Cup Jumping - Zurich round A	28.01.2018	CSI-W5*	3	1.60	430	212	12	10.04.2006	S	BWP	134	11	13	42	49	1996	131	M	1	1	no	vertical	vertical	right	no	0	0	73,95	no
Zurich	The Longines FEI World Cup Jumping - Zurich round A	28.01.2018	CSI-W5*	3	1.60	430	212	12	10.04.2006	S	BWP	134	11	13	42	49	1996	131	M	2	2	no	spread	oxer	right	no	0	0	73,95	no
Zurich	The Longines FEI World Cup Jumping - Zurich round A	28.01.2018	CSI-W5*	3	1.60	430	212	12	10.04.2006	S	BWP	134	11	13	42	49	1996	131	M	3	3	no	vertical	wall	right	no	0	0	73,95	no
Zurich	The Longines FEI World Cup Jumping - Zurich round A	28.01.2018	CSI-W5*	3	1.60	430	212	12	10.04.2006	S	BWP	134	11	13	42	49	1996	131	M	4	4	no	spread	oxer with water	left	no	0	0	73,95	no
Zurich	The Longines FEI World Cup Jumping - Zurich round A	28.01.2018	CSI-W5*	3	1.60	430	212	12	10.04.2006	S	BWP	134	11	13	42	49	1996	131	M	5	5	no	vertical	vertical	left	no	0	0	73,95	no
Zurich	The Longines FEI World Cup Jumping - Zurich round A	28.01.2018	CSI-W5*	3	1.60	430	212	12	10.04.2006	S	BWP	134	11	13	42	49	1996	131	M	6	6a	yes	spread	oxer	left	no	0	0	73,95	no
Zurich	The Longines FEI World Cup Jumping - Zurich round A	28.01.2018	CSI-W5*	3	1.60	430	212	12	10.04.2006	S	BWP	134	11	13	42	49	1996	131	M	7	6b	yes	spread	oxer	left	no	0	0	73,95	no
Zurich	The Longines FEI World Cup Jumping - Zurich round A	28.01.2018	CSI-W5*	3	1.60	430	212	12	10.04.2006	S	BWP	134	11	13	42	49	1996	131	M	8	6c	yes	vertical	vertical	left	no	0	0	73,95	no
Zurich	The Longines FEI World Cup Jumping - Zurich round A	28.01.2018	CSI-W5*	3	1.60	430	212	12	10.04.2006	S	BWP	134	11	13	42	49	1996	131	M	9	7	no	spread	oxer	right	no	0	0	73,95	no
Zurich	The Longines FEI World Cup Jumping - Zurich round A	28.01.2018	CSI-W5*	3	1.60	430	212	12	10.04.2006	S	BWP	134	11	13	42	49	1996	131	M	10	8a	yes	vertical	vertical	right	no	0	0	73,95	no
Zurich	The Longines FEI World Cup Jumping - Zurich round A	28.01.2018	CSI-W5*	3	1.60	430	212	12	10.04.2006	S	BWP	134	11	13	42	49	1996	131	M	11	8b	yes	spread	oxer	right	no	0	0	73,95	no
Zurich	The Longines FEI World Cup Jumping - Zurich round A	28.01.2018	CSI-W5*	3	1.60	430	212	12	10.04.2006	S	BWP	134	11	13	42	49	1996	131	M	12	9	no	vertical	vertical	right	no	0	0	73,95	no
Zurich	The Longines FEI World Cup Jumping - Zurich round A	28.01.2018	CSI-W5*	3	1.60	430	212	12	10.04.2006	S	BWP	134	11	13	42	49	1996	131	M	13	10	no	spread	triple bar	right	no	0	0	73,95	no
Zurich	The Longines FEI World Cup Jumping - Zurich round A	28.01.2018	CSI-W5*	3	1.60	430	212	12	10.04.2006	S	BWP	134	11	13	42	49	1996	131	M	14	11	no	spread	oxer	left	no	0	0	73,95	no
Zurich	The Longines FEI World Cup Jumping - Zurich round A	28.01.2018	CSI-W5*	3	1.60	430	212	12	10.04.2006	S	BWP	134	11	13	42	49	1996	131	M	15	12	no	vertical	vertical with water	left	no	0	0	73,95	no
Zurich	The Longines FEI World Cup Jumping - Zurich round A	28.01.2018	CSI-W5*	3	1.60	430	212	12	10.04.2006	S	BWP	134	11	13	42	49	1996	131	M	16	13	no	spread	oxer	straight	yes	4	0	73,95	no
Zurich	The Longines FEI World Cup Jumping - Zurich round A	28.01.2018	CSI-W5*	3	1.60	430	196	13	15.05.2005	M	ZANG	166	4	14	5	37	1868	96	M	1	1	no	vertical	vertical	right	no	0	0	74,6	no
Zurich	The Longines FEI World Cup Jumping - Zurich round A	28.01.2018	CSI-W5*	3	1.60	430	196	13	15.05.2005	M	ZANG	166	4	14	5	37	1868	96	M	2	2	no	spread	oxer	right	no	0	0	74,6	no
Zurich	The Longines FEI World Cup Jumping - Zurich round A	28.01.2018	CSI-W5*	3	1.60	430	196	13	15.05.2005	M	ZANG	166	4	14	5	37	1868	96	M	3	3	no	vertical	wall	right	yes	4	0	74,6	no
Zurich	The Longines FEI World Cup Jumping - Zurich round A	28.01.2018	CSI-W5*	3	1.60	430	196	13	15.05.2005	M	ZANG	166	4	14	5	37	1868	96	M	4	4	no	spread	oxer with water	left	no	0	0	74,6	no
Zurich	The Longines FEI World Cup Jumping - Zurich round A	28.01.2018	CSI-W5*	3	1.60	430	196	13	15.05.2005	M	ZANG	166	4	14	5	37	1868	96	M	5	5	no	vertical	vertical	left	no	0	0	74,6	no
Zurich	The Longines FEI World Cup Jumping - Zurich round A	28.01.2018	CSI-W5*	3	1.60	430	196	13	15.05.2005	M	ZANG	166	4	14	5	37	1868	96	M	6	6a	yes	spread	oxer	left	no	0	0	74,6	no
Zurich	The Longines FEI World Cup Jumping - Zurich round A	28.01.2018	CSI-W5*	3	1.60	430	196	13	15.05.2005	M	ZANG	166	4	14	5	37	1868	96	M	7	6b	yes	spread	oxer	left	yes	4	0	74,6	no
Zurich	The Longines FEI World Cup Jumping - Zurich round A	28.01.2018	CSI-W5*	3	1.60	430	196	13	15.05.2005	M	ZANG	166	4	14	5	37	1868	96	M	8	6c	yes	vertical	vertical	left	no	0	0	74,6	no
Zurich	The Longines FEI World Cup Jumping - Zurich round A	28.01.2018	CSI-W5*	3	1.60	430	196	13	15.05.2005	M	ZANG	166	4	14	5	37	1868	96	M	9	7	no	spread	oxer	right	yes	4	0	74,6	no
Zurich	The Longines FEI World Cup Jumping - Zurich round A	28.01.2018	CSI-W5*	3	1.60	430	196	13	15.05.2005	M	ZANG	166	4	14	5	37	1868	96	M	10	8a	yes	vertical	vertical	right	no	0	0	74,6	no
Zurich	The Longines FEI World Cup Jumping - Zurich round A	28.01.2018	CSI-W5*	3	1.60	430	196	13	15.05.2005	M	ZANG	166	4	14	5	37	1868	96	M	11	8b	yes	spread	oxer	right	no	0	0	74,6	no
Zurich	The Longines FEI World Cup Jumping - Zurich round A	28.01.2018	CSI-W5*	3	1.60	430	196	13	15.05.2005	M	ZANG	166	4	14	5	37	1868	96	M	12	9	no	vertical	vertical	right	no	0	0	74,6	no
Zurich	The Longines FEI World Cup Jumping - Zurich round A	28.01.2018	CSI-W5*	3	1.60	430	196	13	15.05.2005	M	ZANG	166	4	14	5	37	1868	96	M	13	10	no	spread	triple bar	right	no	0	0	74,6	no
Zurich	The Longines FEI World Cup Jumping - Zurich round A	28.01.2018	CSI-W5*	3	1.60	430	196	13	15.05.2005	M	ZANG	166	4	14	5	37	1868	96	M	14	11	no	spread	oxer	left	no	0	0	74,6	no
Zurich	The Longines FEI World Cup Jumping - Zurich round A	28.01.2018	CSI-W5*	3	1.60	430	196	13	15.05.2005	M	ZANG	166	4	14	5	37	1868	96	M	15	12	no	vertical	vertical with water	left	no	0	0	74,6	no
Zurich	The Longines FEI World Cup Jumping - Zurich round A	28.01.2018	CSI-W5*	3	1.60	430	196	13	15.05.2005	M	ZANG	166	4	14	5	37	1868	96	M	16	13	no	spread	oxer	straight	no	0	1	74,6	no
Zurich	The Longines FEI World Cup Jumping - Zurich round A	28.01.2018	CSI-W5*	3	1.60	430	104	11	23.03.2007	G	KWPN	95	0	17	3	46	1005	51	M	1	1	no	vertical	vertical	right	no	0	0	73,11	no
Zurich	The Longines FEI World Cup Jumping - Zurich round A	28.01.2018	CSI-W5*	3	1.60	430	104	11	23.03.2007	G	KWPN	95	0	17	3	46	1005	51	M	2	2	no	spread	oxer	right	no	0	0	73,11	no
Zurich	The Longines FEI World Cup Jumping - Zurich round A	28.01.2018	CSI-W5*	3	1.60	430	104	11	23.03.2007	G	KWPN	95	0	17	3	46	1005	51	M	3	3	no	vertical	wall	right	no	0	0	73,11	no
Zurich	The Longines FEI World Cup Jumping - Zurich round A	28.01.2018	CSI-W5*	3	1.60	430	104	11	23.03.2007	G	KWPN	95	0	17	3	46	1005	51	M	4	4	no	spread	oxer with water	left	yes	4	0	73,11	no
Zurich	The Longines FEI World Cup Jumping - Zurich round A	28.01.2018	CSI-W5*	3	1.60	430	104	11	23.03.2007	G	KWPN	95	0	17	3	46	1005	51	M	5	5	no	vertical	vertical	left	no	0	0	73,11	no
Zurich	The Longines FEI World Cup Jumping - Zurich round A	28.01.2018	CSI-W5*	3	1.60	430	104	11	23.03.2007	G	KWPN	95	0	17	3	46	1005	51	M	6	6a	yes	spread	oxer	left	no	0	0	73,11	no
Zurich	The Longines FEI World Cup Jumping - Zurich round A	28.01.2018	CSI-W5*	3	1.60	430	104	11	23.03.2007	G	KWPN	95	0	17	3	46	1005	51	M	7	6b	yes	spread	oxer	left	no	0	0	73,11	no
Zurich	The Longines FEI World Cup Jumping - Zurich round A	28.01.2018	CSI-W5*	3	1.60	430	104	11	23.03.2007	G	KWPN	95	0	17	3	46	1005	51	M	8	6c	yes	vertical	vertical	left	no	0	0	73,11	no
Zurich	The Longines FEI World Cup Jumping - Zurich round A	28.01.2018	CSI-W5*	3	1.60	430	104	11	23.03.2007	G	KWPN	95	0	17	3	46	1005	51	M	9	7	no	spread	oxer	right	no	0	0	73,11	no
Zurich	The Longines FEI World Cup Jumping - Zurich round A	28.01.2018	CSI-W5*	3	1.60	430	104	11	23.03.2007	G	KWPN	95	0	17	3	46	1005	51	M	10	8a	yes	vertical	vertical	right	no	0	0	73,11	no
Zurich	The Longines FEI World Cup Jumping - Zurich round A	28.01.2018	CSI-W5*	3	1.60	430	104	11	23.03.2007	G	KWPN	95	0	17	3	46	1005	51	M	11	8b	yes	spread	oxer	right	no	0	0	73,11	no
Zurich	The Longines FEI World Cup Jumping - Zurich round A	28.01.2018	CSI-W5*	3	1.60	430	104	11	23.03.2007	G	KWPN	95	0	17	3	46	1005	51	M	12	9	no	vertical	vertical	right	no	0	0	73,11	no
Zurich	The Longines FEI World Cup Jumping - Zurich round A	28.01.2018	CSI-W5*	3	1.60	430	104	11	23.03.2007	G	KWPN	95	0	17	3	46	1005	51	M	13	10	no	spread	triple bar	right	yes	4	0	73,11	no
Zurich	The Longines FEI World Cup Jumping - Zurich round A	28.01.2018	CSI-W5*	3	1.60	430	104	11	23.03.2007	G	KWPN	95	0	17	3	46	1005	51	M	14	11	no	spread	oxer	left	no	0	0	73,11	no
Zurich	The Longines FEI World Cup Jumping - Zurich round A	28.01.2018	CSI-W5*	3	1.60	430	104	11	23.03.2007	G	KWPN	95	0	17	3	46	1005	51	M	15	12	no	vertical	vertical with water	left	no	0	0	73,11	no
Zurich	The Longines FEI World Cup Jumping - Zurich round A	28.01.2018	CSI-W5*	3	1.60	430	104	11	23.03.2007	G	KWPN	95	0	17	3	46	1005	51	M	16	13	no	spread	oxer	straight	no	0	0	73,11	no
Zurich	The Longines FEI World Cup Jumping - Zurich round A	28.01.2018	CSI-W5*	3	1.60	430	154	14	29.04.2004	G	SWB	175	3	18	81	31	1327	0	M	1	1	no	vertical	vertical	right	no	0	0	74,99	no
Zurich	The Longines FEI World Cup Jumping - Zurich round A	28.01.2018	CSI-W5*	3	1.60	430	154	14	29.04.2004	G	SWB	175	3	18	81	31	1327	0	M	2	2	no	spread	oxer	right	no	0	0	74,99	no
Zurich	The Longines FEI World Cup Jumping - Zurich round A	28.01.2018	CSI-W5*	3	1.60	430	154	14	29.04.2004	G	SWB	175	3	18	81	31	1327	0	M	3	3	no	vertical	wall	right	no	0	0	74,99	no
Zurich	The Longines FEI World Cup Jumping - Zurich round A	28.01.2018	CSI-W5*	3	1.60	430	154	14	29.04.2004	G	SWB	175	3	18	81	31	1327	0	M	4	4	no	spread	oxer with water	left	no	0	0	74,99	no
Zurich	The Longines FEI World Cup Jumping - Zurich round A	28.01.2018	CSI-W5*	3	1.60	430	154	14	29.04.2004	G	SWB	175	3	18	81	31	1327	0	M	5	5	no	vertical	vertical	left	no	0	0	74,99	no
Zurich	The Longines FEI World Cup Jumping - Zurich round A	28.01.2018	CSI-W5*	3	1.60	430	154	14	29.04.2004	G	SWB	175	3	18	81	31	1327	0	M	6	6a	yes	spread	oxer	left	no	0	0	74,99	no
Zurich	The Longines FEI World Cup Jumping - Zurich round A	28.01.2018	CSI-W5*	3	1.60	430	154	14	29.04.2004	G	SWB	175	3	18	81	31	1327	0	M	7	6b	yes	spread	oxer	left	no	0	0	74,99	no
Zurich	The Longines FEI World Cup Jumping - Zurich round A	28.01.2018	CSI-W5*	3	1.60	430	154	14	29.04.2004	G	SWB	175	3	18	81	31	1327	0	M	8	6c	yes	vertical	vertical	left	no	0	0	74,99	no
Zurich	The Longines FEI World Cup Jumping - Zurich round A	28.01.2018	CSI-W5*	3	1.60	430	154	14	29.04.2004	G	SWB	175	3	18	81	31	1327	0	M	9	7	no	spread	oxer	right	no	0	0	74,99	no
Zurich	The Longines FEI World Cup Jumping - Zurich round A	28.01.2018	CSI-W5*	3	1.60	430	154	14	29.04.2004	G	SWB	175	3	18	81	31	1327	0	M	10	8a	yes	vertical	vertical	right	no	0	0	74,99	no
Zurich	The Longines FEI World Cup Jumping - Zurich round A	28.01.2018	CSI-W5*	3	1.60	430	154	14	29.04.2004	G	SWB	175	3	18	81	31	1327	0	M	11	8b	yes	spread	oxer	right	no	0	0	74,99	no
Zurich	The Longines FEI World Cup Jumping - Zurich round A	28.01.2018	CSI-W5*	3	1.60	430	154	14	29.04.2004	G	SWB	175	3	18	81	31	1327	0	M	12	9	no	vertical	vertical	right	no	0	0	74,99	no
Zurich	The Longines FEI World Cup Jumping - Zurich round A	28.01.2018	CSI-W5*	3	1.60	430	154	14	29.04.2004	G	SWB	175	3	18	81	31	1327	0	M	13	10	no	spread	triple bar	right	no	0	0	74,99	no
Zurich	The Longines FEI World Cup Jumping - Zurich round A	28.01.2018	CSI-W5*	3	1.60	430	154	14	29.04.2004	G	SWB	175	3	18	81	31	1327	0	M	14	11	no	spread	oxer	left	no	0	0	74,99	no
Zurich	The Longines FEI World Cup Jumping - Zurich round A	28.01.2018	CSI-W5*	3	1.60	430	154	14	29.04.2004	G	SWB	175	3	18	81	31	1327	0	M	15	12	no	vertical	vertical with water	left	no	0	0	74,99	no
Zurich	The Longines FEI World Cup Jumping - Zurich round A	28.01.2018	CSI-W5*	3	1.60	430	154	14	29.04.2004	G	SWB	175	3	18	81	31	1327	0	M	16	13	no	spread	oxer	straight	no	0	1	74,99	no
Zurich	The Longines FEI World Cup Jumping - Zurich round A	28.01.2018	CSI-W5*	3	1.60	430	215	14	20.07.2004	G	KWPN	236	9	19	54	42	2041	67	M	1	1	no	vertical	vertical	right	no	0	0	0,00	yes
Zurich	The Longines FEI World Cup Jumping - Zurich round A	28.01.2018	CSI-W5*	3	1.60	430	215	14	20.07.2004	G	KWPN	236	9	19	54	42	2041	67	M	2	2	no	spread	oxer	right	no	0	0	0,00	yes
Zurich	The Longines FEI World Cup Jumping - Zurich round A	28.01.2018	CSI-W5*	3	1.60	430	215	14	20.07.2004	G	KWPN	236	9	19	54	42	2041	67	M	3	3	no	vertical	wall	right	no	0	0	0,00	yes
Zurich	The Longines FEI World Cup Jumping - Zurich round A	28.01.2018	CSI-W5*	3	1.60	430	215	14	20.07.2004	G	KWPN	236	9	19	54	42	2041	67	M	4	4	no	spread	oxer with water	left	yes	4	0	0,00	yes
Zurich	The Longines FEI World Cup Jumping - Zurich round A	28.01.2018	CSI-W5*	3	1.60	430	215	14	20.07.2004	G	KWPN	236	9	19	54	42	2041	67	M	5	5	no	vertical	vertical	left	no	0	0	0,00	yes
Zurich	The Longines FEI World Cup Jumping - Zurich round A	28.01.2018	CSI-W5*	3	1.60	430	215	14	20.07.2004	G	KWPN	236	9	19	54	42	2041	67	M	6	6a	yes	spread	oxer	left	no	0	0	0,00	yes
Zurich	The Longines FEI World Cup Jumping - Zurich round A	28.01.2018	CSI-W5*	3	1.60	430	215	14	20.07.2004	G	KWPN	236	9	19	54	42	2041	67	M	7	6b	yes	spread	oxer	left	no	0	0	0,00	yes
Zurich	The Longines FEI World Cup Jumping - Zurich round A	28.01.2018	CSI-W5*	3	1.60	430	215	14	20.07.2004	G	KWPN	236	9	19	54	42	2041	67	M	8	6c	yes	vertical	vertical	left	no	0	0	0,00	yes
Zurich	The Longines FEI World Cup Jumping - Zurich round A	28.01.2018	CSI-W5*	3	1.60	430	215	14	20.07.2004	G	KWPN	236	9	19	54	42	2041	67	M	9	7	no	spread	oxer	right	no	0	0	0,00	yes
Zurich	The Longines FEI World Cup Jumping - Zurich round A	28.01.2018	CSI-W5*	3	1.60	430	215	14	20.07.2004	G	KWPN	236	9	19	54	42	2041	67	M	10	8a	yes	vertical	vertical	right	yes	4	0	0,00	yes
Zurich	The Longines FEI World Cup Jumping - Zurich round A	28.01.2018	CSI-W5*	3	1.60	430	215	14	20.07.2004	G	KWPN	236	9	19	54	42	2041	67	M	11	8b	yes	spread	oxer	right	no	0	0	0,00	yes
Zurich	The Longines FEI World Cup Jumping - Zurich round A	28.01.2018	CSI-W5*	3	1.60	430	215	14	20.07.2004	G	KWPN	236	9	19	54	42	2041	67	M	12	9	no	vertical	vertical	right	yes	4	0	0,00	yes
Zurich	The Longines FEI World Cup Jumping - Zurich round A	28.01.2018	CSI-W5*	3	1.60	430	215	14	20.07.2004	G	KWPN	236	9	19	54	42	2041	67	M	13	10	no	spread	triple bar	right	no	0	0	0,00	yes
Zurich	The Longines FEI World Cup Jumping - Zurich round A	28.01.2018	CSI-W5*	3	1.60	430	215	14	20.07.2004	G	KWPN	236	9	19	54	42	2041	67	M	14	11	no	spread	oxer	left	no	0	0	0,00	yes
Zurich	The Longines FEI World Cup Jumping - Zurich round A	28.01.2018	CSI-W5*	3	1.60	430	215	14	20.07.2004	G	KWPN	236	9	19	54	42	2041	67	M	15	12	no	vertical	vertical with water	left	no	0	0	0,00	yes
Zurich	The Longines FEI World Cup Jumping - Zurich round A	28.01.2018	CSI-W5*	3	1.60	430	215	14	20.07.2004	G	KWPN	236	9	19	54	42	2041	67	M	16	13	no	spread	oxer	straight	no	0	0	0,00	yes
Zurich	The Longines FEI World Cup Jumping - Zurich round A	28.01.2018	CSI-W5*	3	1.60	430	205	11	27.05.2007	M	SF	183	14	20	73	38	2030	91	M	1	1	no	vertical	vertical	right	no	0	0	72,71	no

Zurich	The Longines FEI World Cup Jumping - Zurich round A	28.01.2018	CSI-W5*	3	1.60	430	205	11	27.05.2007	M	SF	183	14	20	73	38	2030	91	M	2	2	no	spread	oxer	right	no	0	0	72,71	no
Zurich	The Longines FEI World Cup Jumping - Zurich round A	28.01.2018	CSI-W5*	3	1.60	430	205	11	27.05.2007	M	SF	183	14	20	73	38	2030	91	M	3	3	no	vertical	wall	right	no	0	0	72,71	no
Zurich	The Longines FEI World Cup Jumping - Zurich round A	28.01.2018	CSI-W5*	3	1.60	430	205	11	27.05.2007	M	SF	183	14	20	73	38	2030	91	M	4	4	no	spread	oxer with water	left	no	0	0	72,71	no
Zurich	The Longines FEI World Cup Jumping - Zurich round A	28.01.2018	CSI-W5*	3	1.60	430	205	11	27.05.2007	M	SF	183	14	20	73	38	2030	91	M	5	5	no	vertical	vertical	left	no	0	0	72,71	no
Zurich	The Longines FEI World Cup Jumping - Zurich round A	28.01.2018	CSI-W5*	3	1.60	430	205	11	27.05.2007	M	SF	183	14	20	73	38	2030	91	M	6	6a	yes	spread	oxer	left	no	0	0	72,71	no
Zurich	The Longines FEI World Cup Jumping - Zurich round A	28.01.2018	CSI-W5*	3	1.60	430	205	11	27.05.2007	M	SF	183	14	20	73	38	2030	91	M	7	6b	yes	spread	oxer	left	no	0	0	72,71	no
Zurich	The Longines FEI World Cup Jumping - Zurich round A	28.01.2018	CSI-W5*	3	1.60	430	205	11	27.05.2007	M	SF	183	14	20	73	38	2030	91	M	8	6c	yes	vertical	vertical	left	no	0	0	72,71	no
Zurich	The Longines FEI World Cup Jumping - Zurich round A	28.01.2018	CSI-W5*	3	1.60	430	205	11	27.05.2007	M	SF	183	14	20	73	38	2030	91	M	9	7	no	spread	oxer	right	no	0	0	72,71	no
Zurich	The Longines FEI World Cup Jumping - Zurich round A	28.01.2018	CSI-W5*	3	1.60	430	205	11	27.05.2007	M	SF	183	14	20	73	38	2030	91	M	10	8a	yes	vertical	vertical	right	no	0	0	72,71	no
Zurich	The Longines FEI World Cup Jumping - Zurich round A	28.01.2018	CSI-W5*	3	1.60	430	205	11	27.05.2007	M	SF	183	14	20	73	38	2030	91	M	11	8b	yes	spread	oxer	right	no	0	0	72,71	no
Zurich	The Longines FEI World Cup Jumping - Zurich round A	28.01.2018	CSI-W5*	3	1.60	430	205	11	27.05.2007	M	SF	183	14	20	73	38	2030	91	M	12	9	no	vertical	vertical	right	no	0	0	72,71	no
Zurich	The Longines FEI World Cup Jumping - Zurich round A	28.01.2018	CSI-W5*	3	1.60	430	205	11	27.05.2007	M	SF	183	14	20	73	38	2030	91	M	13	10	no	spread	triple bar	right	no	0	0	72,71	no
Zurich	The Longines FEI World Cup Jumping - Zurich round A	28.01.2018	CSI-W5*	3	1.60	430	205	11	27.05.2007	M	SF	183	14	20	73	38	2030	91	M	14	11	no	spread	oxer	left	no	0	0	72,71	no
Zurich	The Longines FEI World Cup Jumping - Zurich round A	28.01.2018	CSI-W5*	3	1.60	430	205	11	27.05.2007	M	SF	183	14	20	73	38	2030	91	M	15	12	no	vertical	vertical with water	left	no	0	0	72,71	no
Zurich	The Longines FEI World Cup Jumping - Zurich round A	28.01.2018	CSI-W5*	3	1.60	430	205	11	27.05.2007	M	SF	183	14	20	73	38	2030	91	M	16	13	no	spread	oxer	straight	no	0	0	72,71	no
Zurich	The Longines FEI World Cup Jumping - Zurich round B	28.01.2018	CSI-W5*	3	1.60	300	205	11	27.05.2007	M	SF	183	14	20	73	38	2030	91	M	1	1	no	spread	oxer	left	no	0	0	39,62	no
Zurich	The Longines FEI World Cup Jumping - Zurich round B	28.01.2018	CSI-W5*	3	1.60	300	205	11	27.05.2007	M	SF	183	14	20	73	38	2030	91	M	2	2	no	vertical	vertical	left	no	0	0	39,62	no
Zurich	The Longines FEI World Cup Jumping - Zurich round B	28.01.2018	CSI-W5*	3	1.60	300	205	11	27.05.2007	M	SF	183	14	20	73	38	2030	91	M	3	3	no	spread	oxer	left	no	0	0	39,62	no
Zurich	The Longines FEI World Cup Jumping - Zurich round B	28.01.2018	CSI-W5*	3	1.60	300	205	11	27.05.2007	M	SF	183	14	20	73	38	2030	91	M	4	4a	yes	vertical	vertical	right	no	0	0	39,62	no
Zurich	The Longines FEI World Cup Jumping - Zurich round B	28.01.2018	CSI-W5*	3	1.60	300	205	11	27.05.2007	M	SF	183	14	20	73	38	2030	91	M	5	4b	yes	spread	oxer	right	no	0	0	39,62	no
Zurich	The Longines FEI World Cup Jumping - Zurich round B	28.01.2018	CSI-W5*	3	1.60	300	205	11	27.05.2007	M	SF	183	14	20	73	38	2030	91	M	6	5	no	vertical	vertical	right	yes	4	0	39,62	no
Zurich	The Longines FEI World Cup Jumping - Zurich round B	28.01.2018	CSI-W5*	3	1.60	300	205	11	27.05.2007	M	SF	183	14	20	73	38	2030	91	M	7	6	no	vertical	vertical	right	no	0	0	39,62	no
Zurich	The Longines FEI World Cup Jumping - Zurich round B	28.01.2018	CSI-W5*	3	1.60	300	205	11	27.05.2007	M	SF	183	14	20	73	38	2030	91	M	8	7	no	spread	oxer	left	no	0	0	39,62	no
Zurich	The Longines FEI World Cup Jumping - Zurich round B	28.01.2018	CSI-W5*	3	1.60	300	205	11	27.05.2007	M	SF	183	14	20	73	38	2030	91	M	9	8	no	spread	oxer with water	left	no	0	0	39,62	no
Zurich	The Longines FEI World Cup Jumping - Zurich round A	28.01.2018	CSI-W5*	3	1.60	430	28	12	26.03.2006	M	ZANG	193	9	23	11	35	1800	65	M	1	1	no	vertical	vertical	right	no	0	0	74,33	no
Zurich	The Longines FEI World Cup Jumping - Zurich round A	28.01.2018	CSI-W5*	3	1.60	430	28	12	26.03.2006	M	ZANG	193	9	23	11	35	1800	65	M	2	2	no	spread	oxer	right	no	0	0	74,33	no
Zurich	The Longines FEI World Cup Jumping - Zurich round A	28.01.2018	CSI-W5*	3	1.60	430	28	12	26.03.2006	M	ZANG	193	9	23	11	35	1800	65	M	3	3	no	vertical	wall	right	no	0	0	74,33	no
Zurich	The Longines FEI World Cup Jumping - Zurich round A	28.01.2018	CSI-W5*	3	1.60	430	28	12	26.03.2006	M	ZANG	193	9	23	11	35	1800	65	M	4	4	no	spread	oxer with water	left	no	0	0	74,33	no
Zurich	The Longines FEI World Cup Jumping - Zurich round A	28.01.2018	CSI-W5*	3	1.60	430	28	12	26.03.2006	M	ZANG	193	9	23	11	35	1800	65	M	5	5	no	vertical	vertical	left	no	0	0	74,33	no
Zurich	The Longines FEI World Cup Jumping - Zurich round A	28.01.2018	CSI-W5*	3	1.60	430	28	12	26.03.2006	M	ZANG	193	9	23	11	35	1800	65	M	6	6a	yes	spread	oxer	left	no	0	0	74,33	no
Zurich	The Longines FEI World Cup Jumping - Zurich round A	28.01.2018	CSI-W5*	3	1.60	430	28	12	26.03.2006	M	ZANG	193	9	23	11	35	1800	65	M	7	6b	yes	spread	oxer	left	yes	4	0	74,33	no
Zurich	The Longines FEI World Cup Jumping - Zurich round A	28.01.2018	CSI-W5*	3	1.60	430	28	12	26.03.2006	M	ZANG	193	9	23	11	35	1800	65	M	8	6c	yes	vertical	vertical	left	no	0	0	74,33	no
Zurich	The Longines FEI World Cup Jumping - Zurich round A	28.01.2018	CSI-W5*	3	1.60	430	28	12	26.03.2006	M	ZANG	193	9	23	11	35	1800	65	M	9	7	no	spread	oxer	right	no	0	0	74,33	no
Zurich	The Longines FEI World Cup Jumping - Zurich round A	28.01.2018	CSI-W5*	3	1.60	430	28	12	26.03.2006	M	ZANG	193	9	23	11	35	1800	65	M	10	8a	yes	vertical	vertical	right	no	0	0	74,33	no
Zurich	The Longines FEI World Cup Jumping - Zurich round A	28.01.2018	CSI-W5*	3	1.60	430	28	12	26.03.2006	M	ZANG	193	9	23	11	35	1800	65	M	11	8b	yes	spread	oxer	right	no	0	0	74,33	no
Zurich	The Longines FEI World Cup Jumping - Zurich round A	28.01.2018	CSI-W5*	3	1.60	430	28	12	26.03.2006	M	ZANG	193	9	23	11	35	1800	65	M	12	9	no	vertical	vertical	right	yes	4	0	74,33	no
Zurich	The Longines FEI World Cup Jumping - Zurich round A	28.01.2018	CSI-W5*	3	1.60	430	28	12	26.03.2006	M	ZANG	193	9	23	11	35	1800	65	M	13	10	no	spread	triple bar	right	no	0	0	74,33	no
Zurich	The Longines FEI World Cup Jumping - Zurich round A	28.01.2018	CSI-W5*	3	1.60	430	28	12	26.03.2006	M	ZANG	193	9	23	11	35	1800	65	M	14	11	no	spread	oxer	left	no	0	0	74,33	no
Zurich	The Longines FEI World Cup Jumping - Zurich round A	28.01.2018	CSI-W5*	3	1.60	430	28	12	26.03.2006	M	ZANG	193	9	23	11	35	1800	65	M	15	12	no	vertical	vertical with water	left	no	0	0	74,33	no
Zurich	The Longines FEI World Cup Jumping - Zurich round A	28.01.2018	CSI-W5*	3	1.60	430	28	12	26.03.2006	M	ZANG	193	9	23	11	35	1800	65	M	16	13	no	spread	oxer	straight	no	0	1	74,33	no
Zurich	The Longines FEI World Cup Jumping - Zurich round A	28.01.2018	CSI-W5*	3	1.60	430	184	12	18.06.2006	S	SF	154	4	28	4	38	2170	90	M	1	1	no	vertical	vertical	right	no	0	0	72,81	no
Zurich	The Longines FEI World Cup Jumping - Zurich round A	28.01.2018	CSI-W5*	3	1.60	430	184	12	18.06.2006	S	SF	154	4	28	4	38	2170	90	M	2	2	no	spread	oxer	right	no	0	0	72,81	no
Zurich	The Longines FEI World Cup Jumping - Zurich round A	28.01.2018	CSI-W5*	3	1.60	430	184	12	18.06.2006	S	SF	154	4	28	4	38	2170	90	M	3	3	no	vertical	wall	right	no	0	0	72,81	no
Zurich	The Longines FEI World Cup Jumping - Zurich round A	28.01.2018	CSI-W5*	3	1.60	430	184	12	18.06.2006	S	SF	154	4	28	4	38	2170	90	M	4	4	no	spread	oxer with water	left	no	0	0	72,81	no
Zurich	The Longines FEI World Cup Jumping - Zurich round A	28.01.2018	CSI-W5*	3	1.60	430	184	12	18.06.2006	S	SF	154	4	28	4	38	2170	90	M	5	5	no	vertical	vertical	left	no	0	0	72,81	no
Zurich	The Longines FEI World Cup Jumping - Zurich round A	28.01.2018	CSI-W5*	3	1.60	430	184	12	18.06.2006	S	SF	154	4	28	4	38	2170	90	M	6	6a	yes	spread	oxer	left	no	0	0	72,81	no
Zurich	The Longines FEI World Cup Jumping - Zurich round A	28.01.2018	CSI-W5*	3	1.60	430	184	12	18.06.2006	S	SF	154	4	28	4	38	2170	90	M	7	6b	yes	spread	oxer	left	no	0	0	72,81	no
Zurich	The Longines FEI World Cup Jumping - Zurich round A	28.01.2018	CSI-W5*	3	1.60	430	184	12	18.06.2006	S	SF	154	4	28	4	38	2170	90	M	8	6c	yes	vertical	vertical	left	no	0	0	72,81	no
Zurich	The Longines FEI World Cup Jumping - Zurich round A	28.01.2018	CSI-W5*	3	1.60	430	184	12	18.06.2006	S	SF	154	4	28	4	38	2170	90	M	9	7	no	spread	oxer	right	no	0	0	72,81	no
Zurich	The Longines FEI World Cup Jumping - Zurich round A	28.01.2018	CSI-W5*	3	1.60	430	184	12	18.06.2006	S	SF	154	4	28	4	38	2170	90	M	10	8a	yes	vertical	vertical	right	no	0	0	72,81	no
Zurich	The Longines FEI World Cup Jumping - Zurich round A	28.01.2018	CSI-W5*	3	1.60	430	184	12	18.06.2006	S	SF	154	4	28	4	38	2170	90	M	11	8b	yes	spread	oxer	right	no	0	0	72,81	no
Zurich	The Longines FEI World Cup Jumping - Zurich round A	28.01.2018	CSI-W5*	3	1.60	430	184	12	18.06.2006	S	SF	154	4	28	4	38	2170	90	M	12	9	no	vertical	vertical	right	no	0	0	72,81	no
Zurich	The Longines FEI World Cup Jumping - Zurich round A	28.01.2018	CSI-W5*	3	1.60	430	184	12	18.06.2006	S	SF	154	4	28	4	38	2170	90	M	13	10	no	spread	triple bar	right	no	0	0	72,81	no
Zurich	The Longines FEI World Cup Jumping - Zurich round A	28.01.2018	CSI-W5*	3	1.60	430	184	12	18.06.2006	S	SF	154	4	28	4	38	2170	90	M	14	11	no	spread	oxer	left	no	0	0	72,81	no
Zurich	The Longines FEI World Cup Jumping - Zurich round A	28.01.2018	CSI-W5*	3	1.60	430	184	12	18.06.2006	S	SF	154	4	28	4	38	2170	90	M	15	12	no	vertical	vertical with water	left	no	0	0	72,81	no
Zurich	The Longines FEI World Cup Jumping - Zurich round A	28.01.2018	CSI-W5*	3	1.60	430	184	12	18.06.2006	S	SF	154	4	28	4	38	2170	90	M	16	13	no	spread	oxer	straight	no	0	0	72,81	no
Zurich	The Longines FEI World Cup Jumping - Zurich round B	28.01.2018	CSI-W5*	3	1.60	300	184	12	18.06.2006	S	SF	154	4	28	4	38	2170	90	M	1	1	no	spread	oxer	left	no	0	0	40,72	no
Zurich	The Longines FEI World Cup Jumping - Zurich round B	28.01.2018	CSI-W5*	3	1.60	300	184	12	18.06.2006	S	SF	154	4	28	4	38	2170	90	M	2	2	no	vertical	vertical	left	no	0	0	40,72	no
Zurich	The Longines FEI World Cup Jumping - Zurich round B	28.01.2018	CSI-W5*	3	1.60	300	184	12	18.06.2006	S	SF	154	4	28	4	38	2170	90	M	3	3	no	spread	oxer	left	no	0	0	40,72	no
Zurich	The Longines FEI World Cup Jumping - Zurich round B	28.01.2018	CSI-W5*	3	1.60	300	184	12	18.06.2006	S	SF	154	4	28	4	38	2170	90	M	4	4a	yes	vertical	vertical	right	no	0	0	40,72	no
Zurich	The Longines FEI World Cup Jumping - Zurich round B	28.01.2018	CSI-W5*	3	1.60	300	184	12	18.06.2006	S	SF	154	4	28	4	38	2170	90	M	5	4b	yes	spread	oxer	right	no	0	0	40,72	no
Zurich	The Longines FEI World Cup Jumping - Zurich round B	28.01.2018	CSI-W5*	3	1.60	300	184	12	18.06.2006	S	SF	154	4	28	4	38	2170	90	M	6	5	no	vertical	vertical	right	no	0	0	40,72	no
Zurich	The Longines FEI World Cup Jumping - Zurich round B	28.01.2018	CSI-W5*	3	1.60	300	184	12	18.06.2006	S	SF	154	4	28	4	38	2170	90	M	7	6	no	vertical	vertical	right	no	0	0	40,72	no
Zurich	The Longines FEI World Cup Jumping - Zurich round B	28.01.2018	CSI-W5*	3	1.60	300	184	12	18.06.2006	S	SF	154	4	28	4	38	2170	90	M	8	7	no	spread	oxer	left	no	0	0	40,72	no
Zurich	The Longines FEI World Cup Jumping - Zurich round B	28.01.2018	CSI-W5*	3	1.60	300	184	12	18.06.2006	S	SF	154	4	28	4	38	2170	90	M	9	8	no	spread	oxer with water	left	no	0	0	40,72	no
Zurich	The Longines FEI World Cup Jumping - Zurich round A	28.01.2018	CSI-W5*	3	1.60	430	207	10	08.08.2008	M	SF	116	0	30	9	36	2556	124	M	1	1	no	vertical	vertical	right	no	0	0	70,57	no
Zurich	The Longines FEI World Cup Jumping - Zurich round A	28.01.2018	CSI-W5*	3	1.60	430	207	10	08.08.2008	M	SF	116	0	30	9	36	2556	124	M	2	2	no	spread	oxer	right	no	0	0	70,57	no
Zurich	The Longines FEI World Cup Jumping - Zurich round A	28.01.2018	CSI-W5*	3	1.60	430	207	10	08.08.2008	M	SF	116	0	30	9	36	2556	124	M	3	3	no	vertical	wall	right	no	0	0	70,57	no
Zurich	The Longines FEI World Cup Jumping - Zurich round A	28.01.2018	CSI-W5*	3	1.60	430	207	10	08.08.2008	M	SF	116	0	30	9	36	2556	124	M	4	4	no	spread	oxer with water	left	no	0	0	70,57	no
Zurich	The Longines FEI World Cup Jumping - Zurich round A	28.01.2018	CSI-W5*	3	1.60	430	207	10	08.08.2008	M	SF	116	0	30	9	36	2556	124	M	5	5	no	vertical	vertical	left	no	0	0	70,57	no
Zurich	The Longines FEI World Cup Jumping - Zurich round A	28.01.2018	CSI-W5*	3	1.60	430	207	10	08.08.2008	M	SF	116	0	30	9	36	2556	124	M	6	6a	yes	spread	oxer	left	no	0	0	70,57	no
Zurich	The Longines FEI World Cup Jumping - Zurich round A	28.01.2018	CSI-W5*	3	1.60	430	207	10	08.08.2008	M	SF	116	0	30	9	36	2556	124	M	7	6b	yes	spread	oxer	left	no	0	0	70,57	no
Zurich	The Longines FEI World Cup Jumping - Zurich round A	28.01.2018	CSI-W5*	3	1.60	430	207	10	08.08.2008	M	SF	116	0	30	9	36	2556	124	M	8	6c	yes	vertical	vertical	left	no	0	0	70,57	no
Zurich	The Longines FEI World Cup Jumping - Zurich round A	28.01.2018	CSI-W5*	3	1.60	430	207	10	08.08.2008	M	SF	116	0	30	9	36	2556	124	M	9	7	no	spread	oxer	right	no	0	0	70,57	no
Zurich	The Longines FEI World Cup Jumping - Zurich round A	28.01.2018	CSI-W5*	3	1.60	430	207	10	08.08.2008	M	SF	116	0	30	9	36	2556	124	M	10	8a	yes	vertical	vertical	right	no	0	0	70,57	no
Zurich	The Longines FEI World Cup Jumping - Zurich round A	28.01.2018	CSI-W5*	3	1.60	430	207	10	08.08.2008	M	SF	116	0	30	9	36	2556	124	M	11	8b	yes	spread	oxer	right	yes	4	0	70,57	no
Zurich	The Longines FEI World Cup Jumping - Zurich round A	28.01.2018	CSI-W5*	3	1.60	430	207	10	08.08.2008	M	SF	116	0	30	9	36	2556	124	M	12	9	no	vertical	vertical	right	no	0	0	70,57	no
Zurich	The Longines FEI World Cup Jumping - Zurich round A	28.01.2018	CSI-W5*	3	1.60	430	207	10	08.08.2008	M	SF	116	0	30	9	36	2556	124	M	13	10	no	spread	triple bar	right	no	0	0	70,57	no
Zurich	The Longines FEI World Cup Jumping - Zurich round A	28.01.2018	CSI-W5*	3	1.60	430	207	10	08.08.2008	M	SF	116	0	30	9	36	2556	124	M	14	11	no	spread	oxer	left	no	0	0	70,57	no
Zurich	The Longines FEI World Cup Jumping - Zurich round A	28.01.2018	CSI-W5*	3	1.60	430	207	10	08.08.2008	M	SF	116	0	30	9	36	2556	124	M	15	12	no	vertical	vertical with water	left	no	0	0	70,57	no
Zurich	The Longines FEI World Cup Jumping - Zurich round A	28.01.2018	CSI-W5*	3	1.60	430	207	10	08.08.2008	M	SF	116	0	30	9	36	2556	124	M	16	13	no	spread	oxer	straight	no	0	0	70,57	no
Zurich	The Longines FEI World Cup Jumping - Zurich round A	28.01.2018	CSI-W5*	3	1.60	430	156	14	28.07.2004	M	AES	262	25	33	23	23	1854	199	M	1	1	no	vertical	vertical	right	no	0	0	69,35	no
Zurich	The Longines FEI World Cup Jumping - Zurich round A	28.01.2018	CSI-W5*	3	1.60	430	156	14	28.07.2004	M	AES	262	25	33	23	23	1854	199	M	2	2	no	spread	oxer	right	no	0	0	69,35	no
Zurich	The Longines FEI World Cup Jumping - Zurich round A	28.01.2018	CSI-W5*	3	1.60	430	156	14	28.07.2004	M	AES	262	25	33	23	23	1854	199	M	3	3	no	vertical	wall	right	no	0	0	69,35	no
Zurich	The Longines FEI World Cup Jumping - Zurich round A	28.01.2018	CSI-W5*	3	1.60	430	156	14	28.07.2004	M	AES	262	25	33	23	23	1854	199	M	4	4	no	spread	oxer with water	left	no	0	0	69,35	no
Zurich	The Longines FEI World Cup Jumping - Zurich round A	28.01.2018	CSI-W5*	3	1.60	430	156	14	28.07.2004	M	AES	262	25	33	23	23	1854	199	M	5	5	no	vertical	vertical	left	no	0	0	69,35	no
Zurich	The Longines FEI World Cup Jumping - Zurich round A	28.01.2018	CSI-W5*	3	1.60	430	156	14	28.07.2004	M	AES	262	25	33	23	23	1854	199	M	6	6a	yes	spread	oxer	left	no	0	0	69,35	no
Zurich	The Longines FEI World Cup Jumping - Zurich round A	28.01.2018	CSI-W5*	3	1.60	430	156	14	28.07.2004	M	AES	262	25	33	23	23	1854	199	M	7	6b	yes	spread	oxer	left	yes	4	0	69,35	no
Zurich	The Longines FEI World Cup Jumping - Zurich round A	28.01.2018	CSI-W5*	3	1.60	430	156	14	28.07.2004	M	AES	262	25	33	23	23	1854	199	M	8	6c	yes	vertical	vertical	left	no	0	0	69,35	no
Zurich	The Longines FEI World Cup Jumping - Zurich round A	28.01.2018	CSI-W5*	3	1.60	430	156	14	28.07.2004	M	AES	262	25	33	23	23	1854	199	M	9	7	no	spread	oxer	right	yes	4	0	69,35	no
Zurich	The Longines FEI World Cup Jumping - Zurich round A	28.01.2018	CSI-W5*	3	1.60	430	156	14	28.07.2004	M	AES	262	25	33	23	23	1854	199	M	10	8a	yes	vertical	vertical	right	no	0	0	69,35	no
Zurich	The Longines FEI World Cup Jumping - Zurich round A	28.01.2018	CSI-W5*	3	1.60	430	156	14	28.07.2004	M	AES	262	25	33	23	23	1854	199	M	11	8b	yes	spread	oxer	right	no	0	0	69,35	no
Zurich	The Longines FEI World Cup Jumping - Zurich round A	28.01.2018	CSI-W5*	3	1.60	430	156	14	28.07.2004	M	AES	262	25	33	23	23	1854	199	M	12	9	no	vertical	vertical	right	no	0	0	69,35	no
Zurich	The Longines FEI World Cup Jumping - Zurich round A	28.01.2018	CSI-W5*	3	1.60	430	156	14	28.07.2004	M	AES	262	25	33	23	23	1854	199	M	13	10	no	spread	triple bar	right	no	0	0	69,35	no
Zurich	The Longines FEI World Cup Jumping - Zurich round A	28.01.2018	CSI-W5*	3	1.60	430	156	14	28.07.2004	M	AES	262	25	33	23	23	1854	199	M	14	11	no	spread	oxer	left	no	0	0	69,35	no
Zurich	The Longines FEI World Cup Jumping - Zurich round A	28.01.2018	CSI-W5*	3	1.60	430	156	14	28.07.2004	M	AES	262	25	33	23	23	1854	199	M	15	12	no	vertical	vertical with water	left	no	0	0	69,35	no
Zurich	The Longines FEI World Cup Jumping - Zurich round A	28.01.2018	CSI-W5*	3	1.60	430	156	14	28.07.2004	M	AES	262	25	33	23	23	1854	199	M	16	13	no	spread	oxer	straight	no	0	0	69,35	no
Zurich	The Longines FEI World Cup Jumping - Zurich round A	28.01.2018	CSI-W5*	3	1.60	430	84	14	10.06.2004	G	BWP	248	6	35	44	30	2234	138	M	1	1	no	vertical	vertical	right	no	0	0	0,00	yes
Zurich	The Longines FEI World Cup Jumping - Zurich round A	28.01.2018	CSI-W5*	3	1.60	430	84	14	10.06.2004	G	BWP	248	6	35	44	30	2234	138	M	2	2	no	spread	oxer	right	no	0	0	0,00	yes
Zurich	The Longines FEI World Cup Jumping - Zurich round A	28.01.2018	CSI-W5*	3	1.60	430	84	14	10.06.2004	G	BWP	248	6	35	44	30	2234	138	M	3	3	no	vertical	wall	right	yes	4	0	0,00	yes
Zurich	The Longines FEI World Cup Jumping - Zurich round A	28.01.2018	CSI-W5*	3	1.60	430	84	14	10.06.2004	G	BWP	248	6	35	44	30	2234	138	M	4	4	no	spread	oxer with water	left	no	0	0	0,00	yes
Zurich	The Longines FEI World Cup Jumping - Zurich round A	28.01.2018	CSI-W5*	3	1.60	430	84	14	10.06.2004	G	BWP	248	6	35	44	30	2234	138	M	5	5	no	vertical	vertical	left	no	0	0	0,00	yes
Zurich	The Longines FEI World Cup Jumping - Zurich round A	28.01.2018	CSI-W5*	3	1.60	430	84	14	10.06.2004	G	BWP	248	6	35	44	30	2234	138	M	6	6a	yes	spread	oxer	left	no	0	0	0,00	yes
Zurich	The Longines FEI World Cup Jumping - Zurich round A	28.01.2018	CSI-W5*	3	1.60	430	84	14	10.06.2004	G	BWP	248	6	35	44	30	2234	138	M	7	6b	yes	spread	oxer	left	yes	4	0	0,00	yes
Zurich	The Longines FEI World Cup Jumping - Zurich round A	28.01.2018	CSI-W5*	3	1.60	430	84	14	10.06.2004	G	BWP	248	6	35	44	30	2234	138	M	8	6c	yes	vertical	vertical	left	yes	4	0	0,00	yes
Zurich	The Longines FEI World Cup Jumping - Zurich round A	28.01.2018	CSI-W5*	3	1.60	430	84	14	10.06.2004	G	BWP	248	6	35	44	30	2234	138	M	9	7	no	spread	oxer	right	no	0	0	0,00	yes
Zurich	The Longines FEI World Cup Jumping - Zurich round A	28.01.2018	CSI-W5*	3	1.60	430	84	14	10.06.2004	G	BWP	248	6	35	44	30	2234	138	M	10	8a	yes	vertical	vertical	right	no	0	0	0,00	yes
Zurich	The Longines FEI World Cup Jumping - Zurich round A	28.01.2018	CSI-W5*	3	1.60	430	84	14	10.06.2004	G	BWP	248	6	35	44	30	2234	138	M	11	8b	yes	spread	oxer	right	no	0	0	0,00	yes
Zurich	The Longines FEI World Cup Jumping - Zurich round A	28.01.2018	CSI-W5*	3	1.60	430	84	14	10.06.2004	G	BWP	248	6	35	44	30	2234	138	M	12	9	no	vertical	vertical	right	no	0	0	0,00	yes
Zurich	The Longines FEI World Cup Jumping - Zurich round A	28.01.2018	CSI-W5*	3	1.60	430	84	14	10.06.2004	G	BWP	248	6	35	44	30	2234	138	M	13	10	no	spread	triple bar	right	no	0	0	0,00	yes
Zurich	The Longines FEI World Cup Jumping - Zurich round A	28.01.2018	CSI-W5*	3	1.60	430	84	14	10.06.2004	G	BWP	248	6	35	44	30	2234	138	M	14	11	no	spread	oxer	left	no	0	0	0,00	yes
Zurich	The Longines FEI World Cup Jumping - Zurich round A	28.01.2018	CSI-W5*	3	1.60	430	84	14	10.06.2004	G	BWP	248	6	35	44	30	2234	138	M	15	12	no	vertical	vertical with water	left	yes	4	0	0,00	yes
Zurich	The Longines FEI World Cup Jumping - Zurich round A	28.01.2018	CSI-W5*	3	1.60	430	84	14	10.06.2004	G	BWP	248	6	35	44	30	2234	138	M	16	13	no	spread	oxer	straight	no	0	0	0,00	yes
Zurich	The Longines FEI World Cup Jumping - Zurich round A	28.01.2018	CSI-W5*	3	1.60	430	48	17	01.01.2001	G	WESTF	290	9	39	71	51	2525	123	M	1	1	no	vertical	vertical	right	yes	4	0	0,00	yes
Zurich	The Longines FEI World Cup Jumping - Zurich round A	28.01.2018	CSI-W5*	3	1.60	430	48	17	01.01.2001	G	WESTF	290	9	39	71	51	2525	123	M	2	2	no	spread	oxer	right	no	0	0	0,00	yes
Zurich	The Longines FEI World Cup Jumping - Zurich round A	28.01.2018	CSI-W5*	3	1.60	430	48	17	01.01.2001	G	WESTF	290	9	39	71	51	2525	123	M	3	3	no	vertical	wall	right	no	0	0	0,00	yes
Zurich	The Longines FEI World Cup Jumping - Zurich round A	28.01.2018	CSI-W5*	3	1.60	430	48	17	01.01.2001	G	WESTF	290	9	39	71	51	2525	123	M	4	4	no	spread	oxer with water	left	yes	4	0	0,00	yes
Zurich	The Longines FEI World Cup Jumping - Zurich round A	28.01.2018	CSI-W5*	3	1.60	430	48	17	01.01.2001	G	WESTF	290	9	39	71	51	2525	123	M	5	5	no	vertical	vertical	left	no	0	0	0,00	yes
Zurich	The Longines FEI World Cup Jumping - Zurich round A	28.01.2018	CSI-W5*	3	1.60	430	48	17	01.01.2001	G	WESTF	290	9	39	71	51	2525	123	M	6	6a	yes	spread	oxer	left	no	0	0	0,00	yes
Zurich	The Longines FEI World Cup Jumping - Zurich round A	28.01.2018	CSI-W5*	3	1.60	430	48	17	01.01.2001	G	WESTF	290	9	39	71	51	2525	123	M	7	6b	yes	spread	oxer	left	no	0	0	0,00	yes
Zurich	The Longines FEI World Cup Jumping - Zurich round A	28.01.2018	CSI-W5*	3	1.60	430	48	17	01.01.2001	G	WESTF	290	9	39	71	51	2525	123	M	8	6c	yes	vertical	vertical	left	no	0	0	0,00	yes
Zurich	The Longines FEI World Cup Jumping - Zurich round A	28.01.2018	CSI-W5*	3	1.60	430	48	17	01.01.2001	G	WESTF	290	9	39	71	51	2525	123	M	9	7	no	spread	oxer	right	no	0	0	0,00	yes
Zurich	The Longines FEI World Cup Jumping - Zurich round A	28.01.2018	CSI-W5*	3	1.60	430	48	17	01.01.2001	G	WESTF	290	9	39	71	51	2525	123	M	10	8a	yes	vertical	vertical	right	no	0	0	0,00	yes
Zurich	The Longines FEI World Cup Jumping - Zurich round A	28.01.2018	CSI-W5*	3	1.60	430	48	17	01.01.2001	G	WESTF	290	9	39	71	51	2525	123	M	11	8b	yes	spread	oxer	right	no	0	0	0,00	yes
Zurich	The Longines FEI World Cup Jumping - Zurich round A	28.01.2018	CSI-W5*	3	1.60	430	48	17	01.01.2001	G	WESTF	290	9	39	71	51	2525	123	M	12	9	no	vertical	vertical	right	no	0	0	0,00	yes
Zurich	The Longines FEI World Cup Jumping - Zurich round A	28.01.2018	CSI-W5*	3	1.60	430	48	17	01.01.2001	G	WESTF	290	9	39	71	51	2525	123	M	13	10	no	spread	triple bar	right	no	0	0	0,00	yes
Zurich	The Longines FEI World Cup Jumping - Zurich round A	28.01.2018	CSI-W5*	3	1.60	430	48	17	01.01.2001	G	WESTF	290	9	39	71	51	2525	123	M	14	11	no	spread	oxer	left	no	0	0	0,00	yes
Zurich	The Longines FEI World Cup Jumping - Zurich round A	28.01.2018	CSI-W5*	3	1.60	430	48	17	01.01.2001	G	WESTF	290	9	39	71	51	2525	123	M	15	12	no	vertical	vertical with water	left	no	0	0	0,00	yes
Zurich	The Longines FEI World Cup Jumping - Zurich round A	28.01.2018	CSI-W5*	3	1.60	430	48	17	01.01.2001	G	WESTF	290	9	39	71	51	2525	123	M	16	13	no	spread	oxer	straight	no	0	0	0,00	yes
Zurich	The Longines FEI World Cup Jumping - Zurich round A	28.01.2018	CSI-W5*	3	1.60	430	105	12	02.04.2006	M	DSP	186	8	50	30	25	1981	78	M	1	1	no	vertical	vertical	right	no	0	0	71,94	no
Zurich	The Longines FEI World Cup Jumping - Zurich round A	28.01.2018	CSI-W5*	3	1.60	430	105	12	02.04.2006	M	DSP	186	8	50	30	25	1981	78	M	2	2	no	spread	oxer	right	no	0	0	71,94	no
Zurich	The Longines FEI World Cup Jumping - Zurich round A	28.01.2018	CSI-W5*	3	1.60	430	105	12	02.04.2006	M	DSP	186	8	50	30	25	1981	78	M	3	3	no	vertical	wall	right	no	0	0	71,94	no
Zurich	The Longines FEI World Cup Jumping - Zurich round A	28.01.2018	CSI-W5*	3	1.60	430	105	12	02.04.2006	M	DSP	186	8	50	30	25	1981	78	M	4	4	no	spread	oxer with water	left	no	0	0	71,94	no
Zurich	The Longines FEI World Cup Jumping - Zurich round A	28.01.2018	CSI-W5*	3	1.60	430	105	12	02.04.2006	M	DSP	186	8	50	30	25	1981	78	M	5	5	no	vertical	vertical	left	no	0	0	71,94	no
Zurich	The Longines FEI World Cup Jumping - Zurich round A	28.01.2018	CSI-W5*	3	1.60	430	105	12	02.04.2006	M	DSP	186	8	50	30	25	1981	78	M	6	6a	yes	spread	oxer	left	no	0	0	71,94	no
Zurich	The Longines FEI World Cup Jumping - Zurich round A	28.01.2018	CSI-W5*	3	1.60	430	105	12	02.04.2006	M	DSP	186	8	50	30	25	1981	78	M	7	6b	yes	spread	oxer	left	no	0	0	71,94	no
Zurich	The Longines FEI World Cup Jumping - Zurich round A	28.01.2018	CSI-W5*	3	1.60	430	105	12	02.04.2006	M	DSP	186	8	50	30	25	1981	78	M	8	6c	yes	vertical	vertical	left	no	0	0	71,94	no
Zurich	The Longines FEI World Cup Jumping - Zurich round A	28.01.2018	CSI-W5*	3	1.60	430	105	12	02.04.2006	M	DSP	186	8	50	30	25	1981	78	M	9	7	no	spread	oxer	right	no	0	0	71,94	no
Zurich	The Longines FEI World Cup Jumping - Zurich round A	28.01.2018	CSI-W5*	3	1.60	430	105	12	02.04.2006	M	DSP	186	8	50	30	25	1981	78	M	10	8a	yes	vertical	vertical	right	no	0	0	71,94	no
Zurich	The Longines FEI World Cup Jumping - Zurich round A	28.01.2018	CSI-W5*	3	1.60	430	105	12	02.04.2006	M	DSP	186	8	50	30	25	1981	78	M	11	8b	yes	spread	oxer	right	no	0	0	71,94	no
Zurich	The Longines FEI World Cup Jumping - Zurich round A	28.01.2018	CSI-W5*	3	1.60	430	105	12	02.04.2006	M	DSP	186	8	50	30	25	1981	78	M	12	9	no	vertical	vertical	right	no	0	0	71,94	no
Zurich	The Longines FEI World Cup Jumping - Zurich round A	28.01.2018	CSI-W5*	3	1.60	430	105	12	02.04.2006	M	DSP	186	8	50	30	25	1981	78	M	13	10	no	spread	triple bar	right	no	0	0	71,94	no
Zurich	The Longines FEI World Cup Jumping - Zurich round A	28.01.2018	CSI-W5*	3	1.60	430	105	12	02.04.2006	M	DSP	186	8	50	30	25	1981	78	M	14	11	no	spread	oxer	left	no	0	0	71,94	no
Zurich	The Longines FEI World Cup Jumping - Zurich round A	28.01.2018	CSI-W5*	3	1.60	430	105	12	02.04.2006	M	DSP	186	8	50	30	25	1981	78	M	15	12	no	vertical	vertical with water	left	no	0	0	71,94	no
Zurich	The Longines FEI World Cup Jumping - Zurich round A	28.01.2018	CSI-W5*	3	1.60	430	105	12	02.04.2006	M	DSP	186	8	50	30	25	1981	78	M	16	13	no	spread	oxer	straight	no	0	0	71,94	no
Zurich	The Longines FEI World Cup Jumping - Zurich round B	28.01.2018	CSI-W5*	3	1.60	300	105	12	02.04.2006	M	DSP	186	8	50	30	25	1981	78	M	1	1	no	spread	oxer	left	no	0	0	40,98	no
Zurich	The Longines FEI World Cup Jumping - Zurich round B	28.01.2018	CSI-W5*	3	1.60	300	105	12	02.04.2006	M	DSP	186	8	50	30	25	1981	78	M	2	2	no	vertical	vertical	left	no	0	0	40,98	no
Zurich	The Longines FEI World Cup Jumping - Zurich round B	28.01.2018	CSI-W5*	3	1.60	300	105	12	02.04.2006	M	DSP	186	8	50	30	25	1981	78	M	3	3	no	spread	oxer	left	no	0	0	40,98	no
Zurich	The Longines FEI World Cup Jumping - Zurich round B	28.01.2018	CSI-W5*	3	1.60	300	105	12	02.04.2006	M	DSP	186	8	50	30	25	1981	78	M	4	4a	yes	vertical	vertical	right	no	0	0	40,98	no
Zurich	The Longines FEI World Cup Jumping - Zurich round B	28.01.2018	CSI-W5*	3	1.60	300	105	12	02.04.2006	M	DSP	186	8	50	30	25	1981	78	M	5	4b	yes	spread	oxer	right	no	0	0	40,98	no
Zurich	The Longines FEI World Cup Jumping - Zurich round B	28.01.2018	CSI-W5*	3	1.60	300	105	12	02.04.2006	M	DSP	186	8	50	30	25	1981	78	M	6	5	no	vertical	vertical	right	no	0	0	40,98	no
Zurich	The Longines FEI World Cup Jumping - Zurich round B	28.01.2018	CSI-W5*	3	1.60	300	105	12	02.04.2006	M	DSP	186	8	50	30	25	1981	78	M	7	6	no	vertical	vertical	right	no	0	0	40,98	no
Zurich	The Longines FEI World Cup Jumping - Zurich round B	28.01.2018	CSI-W5*	3	1.60	300	105	12	02.04.2006	M	DSP	186	8	50	30	25	1981	78	M	8	7	no	spread	oxer	left	no	0	0	40,98	no
Zurich	The Longines FEI World Cup Jumping - Zurich round B	28.01.2018	CSI-W5*	3	1.60	300	105	12	02.04.2006	M	DSP	186	8	50	30	25	1981	78	M	9	8	no	spread	oxer with water	left	no	0	0	40,98	no
Zurich	The Longines FEI World Cup Jumping - Zurich round A	28.01.2018	CSI-W5*	3	1.60	430	118	12	26.01.2006	G	OS	262	11	53	32	33	1848	55	M	1	1	no	vertical	vertical	right	no	0	0	0,00	yes
Zurich	The Longines FEI World Cup Jumping - Zurich round A	28.01.2018	CSI-W5*	3	1.60	430	118	12	26.01.2006	G	OS	262	11	53	32	33	1848	55	M	2	2	no	spread	oxer	right	no	0	0	0,00	yes
Zurich	The Longines FEI World Cup Jumping - Zurich round A	28.01.2018	CSI-W5*	3	1.60	430	118	12	26.01.2006	G	OS	262	11	53	32	33	1848	55	M	3	3	no	vertical	wall	right	no	0	0	0,00	yes
Zurich	The Longines FEI World Cup Jumping - Zurich round A	28.01.2018	CSI-W5*	3	1.60	430	118	12	26.01.2006	G	OS	262	11	53	32	33	1848	55	M	4	4	no	spread	oxer with water	left	yes	4	0	0,00	yes
Zurich	The Longines FEI World Cup Jumping - Zurich round A	28.01.2018	CSI-W5*	3	1.60	430	118	12	26.01.2006	G	OS	262	11	53	32	33	1848	55	M	5	5	no	vertical	vertical	left	no	0	0	0,00	yes
Zurich	The Longines FEI World Cup Jumping - Zurich round A	28.01.2018	CSI-W5*	3	1.60	430	118	12	26.01.2006	G	OS	262	11	53	32	33	1848	55	M	6	6a	yes	spread	oxer	left	yes	4	0	0,00	yes
Zurich	The Longines FEI World Cup Jumping - Zurich round A	28.01.2018	CSI-W5*	3	1.60	430	118	12	26.01.2006	G	OS	262	11	53	32	33	1848	55	M	7	6b	yes	spread	oxer	left	no	0	0	0,00	yes
Zurich	The Longines FEI World Cup Jumping - Zurich round A	28.01.2018	CSI-W5*	3	1.60	430	118	12	26.01.2006	G	OS	262	11	53	32	33	1848	55	M	8	6c	yes	vertical	vertical	left	no	0	0	0,00	yes
Zurich	The Longines FEI World Cup Jumping - Zurich round A	28.01.2018	CSI-W5*	3	1.60	430	118	12	26.01.2006	G	OS	262	11	53	32	33	1848	55	M	9	7	no	spread	oxer	right	yes	4	0	0,00	yes
Zurich	The Longines FEI World Cup Jumping - Zurich round A	28.01.2018	CSI-W5*	3	1.60	430	118	12	26.01.2006	G	OS	262	11	53	32	33	1848	55	M	10	8a	yes	vertical	vertical	right	no	0	0	0,00	yes
Zurich	The Longines FEI World Cup Jumping - Zurich round A	28.01.2018	CSI-W5*	3	1.60	430	118	12	26.01.2006	G	OS	262	11	53	32	33	1848	55	M	11	8b	yes	spread	oxer	right	no	0	0	0,00	yes
Zurich	The Longines FEI World Cup Jumping - Zurich round A	28.01.2018	CSI-W5*	3	1.60	430	118	12	26.01.2006	G	OS	262	11	53	32	33	1848	55	M	12	9	no	vertical	vertical	right	no	0	0	0,00	yes
Zurich	The Longines FEI World Cup Jumping - Zurich round A	28.01.2018	CSI-W5*	3	1.60	430	118	12	26.01.2006	G	OS	262	11	53	32	33	1848	55	M	13	10	no	spread	triple bar	right	no	0	0	0,00	yes
Zurich	The Longines FEI World Cup Jumping - Zurich round A	28.01.2018	CSI-W5*	3	1.60	430	118	12	26.01.2006	G	OS	262	11	53	32	33	1848	55	M	14	11	no	spread	oxer	left	no	0	0	0,00	yes
Zurich	The Longines FEI World Cup Jumping - Zurich round A	28.01.2018	CSI-W5*	3	1.60	430	118	12	26.01.2006	G	OS	262	11	53	32	33	1848	55	M	15	12	no	vertical	vertical with water	left	no	0	0	0,00	yes
Zurich	The Longines FEI World Cup Jumping - Zurich round A	28.01.2018	CSI-W5*	3	1.60	430	118	12	26.01.2006	G	OS	262	11	53	32	33	1848	55	M	16	13	no	spread	oxer	straight	no	0	0	0,00	yes
Zurich	The Longines FEI World Cup Jumping - Zurich round A	28.01.2018	CSI-W5*	3	1.60	430	152	12	20.05.2006	M	WESTF	94	7	55	6	37	1750	61	M	1	1	no	vertical	vertical	right	no	0	0	71,62	no
Zurich	The Longines FEI World Cup Jumping - Zurich round A	28.01.2018	CSI-W5*	3	1.60	430	152	12	20.05.2006	M	WESTF	94	7	55	6	37	1750	61	M	2	2	no	spread	oxer	right	no	0	0	71,62	no
Zurich	The Longines FEI World Cup Jumping - Zurich round A	28.01.2018	CSI-W5*	3	1.60	430	152	12	20.05.2006	M	WESTF	94	7	55	6	37	1750	61	M	3	3	no	vertical	wall	right	no	0	0	71,62	no
Zurich	The Longines FEI World Cup Jumping - Zurich round A	28.01.2018	CSI-W5*	3	1.60	430	152	12	20.05.2006	M	WESTF	94	7	55	6	37	1750	61	M	4	4	no	spread	oxer with water	left	no	0	0	71,62	no
Zurich	The Longines FEI World Cup Jumping - Zurich round A	28.01.2018	CSI-W5*	3	1.60	430	152	12	20.05.2006	M	WESTF	94	7	55	6	37	1750	61	M	5	5	no	vertical	vertical	left	no	0	0	71,62	no
Zurich	The Longines FEI World Cup Jumping - Zurich round A	28.01.2018	CSI-W5*	3	1.60	430	152	12	20.05.2006	M	WESTF	94	7	55	6	37	1750	61	M	6	6a	yes	spread	oxer	left	no	0	0	71,62	no
Zurich	The Longines FEI World Cup Jumping - Zurich round A	28.01.2018	CSI-W5*	3	1.60	430	152	12	20.05.2006	M	WESTF	94	7	55	6	37	1750	61	M	7	6b	yes	spread	oxer	left	no	0	0	71,62	no
Zurich	The Longines FEI World Cup Jumping - Zurich round A	28.01.2018	CSI-W5*	3	1.60	430	152	12	20.05.2006	M	WESTF	94	7	55	6	37	1750	61	M	8	6c	yes	vertical	vertical	left	no	0	0	71,62	no
Zurich	The Longines FEI World Cup Jumping - Zurich round A	28.01.2018	CSI-W5*	3	1.60	430	152	12	20.05.2006	M	WESTF	94	7	55	6	37	1750	61	M	9	7	no	spread	oxer	right	no	0	0	71,62	no
Zurich	The Longines FEI World Cup Jumping - Zurich round A	28.01.2018	CSI-W5*	3	1.60	430	152	12	20.05.2006	M	WESTF	94	7	55	6	37	1750	61	M	10	8a	yes	vertical	vertical	right	no	0	0	71,62	no
Zurich	The Longines FEI World Cup Jumping - Zurich round A	28.01.2018	CSI-W5*	3	1.60	430	152	12	20.05.2006	M	WESTF	94	7	55	6	37	1750	61	M	11	8b	yes	spread	oxer	right	no	0	0	71,62	no
Zurich	The Longines FEI World Cup Jumping - Zurich round A	28.01.2018	CSI-W5*	3	1.60	430	152	12	20.05.2006	M	WESTF	94	7	55	6	37	1750	61	M	12	9	no	vertical	vertical	right	no	0	0	71,62	no
Zurich	The Longines FEI World Cup Jumping - Zurich round A	28.01.2018	CSI-W5*	3	1.60	430	152	12	20.05.2006	M	WESTF	94	7	55	6	37	1750	61	M	13	10	no	spread	triple bar	right	no	0	0	71,62	no
Zurich	The Longines FEI World Cup Jumping - Zurich round A	28.01.2018	CSI-W5*	3	1.60	430	152	12	20.05.2006	M	WESTF	94	7	55	6	37	1750	61	M	14	11	no	spread	oxer	left	no	0	0	71,62	no
Zurich	The Longines FEI World Cup Jumping - Zurich round A	28.01.2018	CSI-W5*	3	1.60	430	152	12	20.05.2006	M	WESTF	94	7	55	6	37	1750	61	M	15	12	no	vertical	vertical with water	left	no	0	0	71,62	no
Zurich	The Longines FEI World Cup Jumping - Zurich round A	28.01.2018	CSI-W5*	3	1.60	430	152	12	20.05.2006	M	WESTF	94	7	55	6	37	1750	61	M	16	13	no	spread	oxer	straight	no	0	0	71,62	no
Zurich	The Longines FEI World Cup Jumping - Zurich round B	28.01.2018	CSI-W5*	3	1.60	300	152	12	20.05.2006	M	WESTF	94	7	55	6	37	1750	61	M	1	1	no	spread	oxer	left	no	0	0	39,61	no
Zurich	The Longines FEI World Cup Jumping - Zurich round B	28.01.2018	CSI-W5*	3	1.60	300	152	12	20.05.2006	M	WESTF	94	7	55	6	37	1750	61	M	2	2	no	vertical	vertical	left	no	0	0	39,61	no
Zurich	The Longines FEI World Cup Jumping - Zurich round B	28.01.2018	CSI-W5*	3	1.60	300	152	12	20.05.2006	M	WESTF	94	7	55	6	37	1750	61	M	3	3	no	spread	oxer	left	no	0	0	39,61	no
Zurich	The Longines FEI World Cup Jumping - Zurich round B	28.01.2018	CSI-W5*	3	1.60	300	152	12	20.05.2006	M	WESTF	94	7	55	6	37	1750	61	M	4	4a	yes	vertical	vertical	right	no	0	0	39,61	no
Zurich	The Longines FEI World Cup Jumping - Zurich round B	28.01.2018	CSI-W5*	3	1.60	300	152	12	20.05.2006	M	WESTF	94	7	55	6	37	1750	61	M	5	4b	yes	spread	oxer	right	no	0	0	39,61	no
Zurich	The Longines FEI World Cup Jumping - Zurich round B	28.01.2018	CSI-W5*	3	1.60	300	152	12	20.05.2006	M	WESTF	94	7	55	6	37	1750	61	M	6	5	no	vertical	vertical	right	no	0	0	39,61	no
Zurich	The Longines FEI World Cup Jumping - Zurich round B	28.01.2018	CSI-W5*	3	1.60	300	152	12	20.05.2006	M	WESTF	94	7	55	6	37	1750	61	M	7	6	no	vertical	vertical	right	no	0	0	39,61	no
Zurich	The Longines FEI World Cup Jumping - Zurich round B	28.01.2018	CSI-W5*	3	1.60	300	152	12	20.05.2006	M	WESTF	94	7	55	6	37	1750	61	M	8	7	no	spread	oxer	left	yes	4	0	39,61	no
Zurich	The Longines FEI World Cup Jumping - Zurich round B	28.01.2018	CSI-W5*	3	1.60	300	152	12	20.05.2006	M	WESTF	94	7	55	6	37	1750	61	M	9	8	no	spread	oxer with water	left	no	0	0	39,61	no
Zurich	The Longines FEI World Cup Jumping - Zurich round A	28.01.2018	CSI-W5*	3	1.60	430	65	10	11.03.2008	G	KWPN	156	6	56	56	44	2054	71	M	1	1	no	vertical	vertical	right	no	0	0	71,38	no
Zurich	The Longines FEI World Cup Jumping - Zurich round A	28.01.2018	CSI-W5*	3	1.60	430	65	10	11.03.2008	G	KWPN	156	6	56	56	44	2054	71	M	2	2	no	spread	oxer	right	no	0	0	71,38	no
Zurich	The Longines FEI World Cup Jumping - Zurich round A	28.01.2018	CSI-W5*	3	1.60	430	65	10	11.03.2008	G	KWPN	156	6	56	56	44	2054	71	M	3	3	no	vertical	wall	right	no	0	0	71,38	no
Zurich	The Longines FEI World Cup Jumping - Zurich round A	28.01.2018	CSI-W5*	3	1.60	430	65	10	11.03.2008	G	KWPN	156	6	56	56	44	2054	71	M	4	4	no	spread	oxer with water	left	no	0	0	71,38	no
Zurich	The Longines FEI World Cup Jumping - Zurich round A	28.01.2018	CSI-W5*	3	1.60	430	65	10	11.03.2008	G	KWPN	156	6	56	56	44	2054	71	M	5	5	no	vertical	vertical	left	no	0	0	71,38	no
Zurich	The Longines FEI World Cup Jumping - Zurich round A	28.01.2018	CSI-W5*	3	1.60	430	65	10	11.03.2008	G	KWPN	156	6	56	56	44	2054	71	M	6	6a	yes	spread	oxer	left	no	0	0	71,38	no
Zurich	The Longines FEI World Cup Jumping - Zurich round A	28.01.2018	CSI-W5*	3	1.60	430	65	10	11.03.2008	G	KWPN	156	6	56	56	44	2054	71	M	7	6b	yes	spread	oxer	left	no	0	0	71,38	no
Zurich	The Longines FEI World Cup Jumping - Zurich round A	28.01.2018	CSI-W5*	3	1.60	430	65	10	11.03.2008	G	KWPN	156	6	56	56	44	2054	71	M	8	6c	yes	vertical	vertical	left	no	0	0	71,38	no
Zurich	The Longines FEI World Cup Jumping - Zurich round A	28.01.2018	CSI-W5*	3	1.60	430	65	10	11.03.2008	G	KWPN	156	6	56	56	44	2054	71	M	9	7	no	spread	oxer	right	no	0	0	71,38	no
Zurich	The Longines FEI World Cup Jumping - Zurich round A	28.01.2018	CSI-W5*	3	1.60	430	65	10	11.03.2008	G	KWPN	156	6	56	56	44	2054	71	M	10	8a	yes	vertical	vertical	right	no	0	0	71,38	no
Zurich	The Longines FEI World Cup Jumping - Zurich round A	28.01.2018	CSI-W5*	3	1.60	430	65	10	11.03.2008	G	KWPN	156	6	56	56	44	2054	71	M	11	8b	yes	spread	oxer	right	no	0	0	71,38	no
Zurich	The Longines FEI World Cup Jumping - Zurich round A	28.01.2018	CSI-W5*	3	1.60	430	65	10	11.03.2008	G	KWPN	156	6	56	56	44	2054	71	M	12	9	no	vertical	vertical	right	no	0	0	71,38	no
Zurich	The Longines FEI World Cup Jumping - Zurich round A	28.01.2018	CSI-W5*	3	1.60	430	65	10	11.03.2008	G	KWPN	156	6	56	56	44	2054	71	M	13	10	no	spread	triple bar	right	no	0	0	71,38	no
Zurich	The Longines FEI World Cup Jumping - Zurich round A	28.01.2018	CSI-W5*	3	1.60	430	65	10	11.03.2008	G	KWPN	156	6	56	56	44	2054	71	M	14	11	no	spread	oxer	left	no	0	0	71,38	no
Zurich	The Longines FEI World Cup Jumping - Zurich round A	28.01.2018	CSI-W5*	3	1.60	430	65	10	11.03.2008	G	KWPN	156	6	56	56	44	2054	71	M	15	12	no	vertical	vertical with water	left	no	0	0	71,38	no
Zurich	The Longines FEI World Cup Jumping - Zurich round A	28.01.2018	CSI-W5*	3	1.60	430	65	10	11.03.2008	G	KWPN	156	6	56	56	44	2054	71	M	16	13	no	spread	oxer	straight	no	0	0	71,38	no
Zurich	The Longines FEI World Cup Jumping - Zurich round B	28.01.2018	CSI-W5*	3	1.60	300	65	10	11.03.2008	G	KWPN	156	6	56	56	44	2054	71	M	1	1	no	spread	oxer	left	no	0	0	41,3	no
Zurich	The Longines FEI World Cup Jumping - Zurich round B	28.01.2018	CSI-W5*	3	1.60	300	65	10	11.03.2008	G	KWPN	156	6	56	56	44	2054	71	M	2	2	no	vertical	vertical	left	no	0	0	41,3	no
Zurich	The Longines FEI World Cup Jumping - Zurich round B	28.01.2018	CSI-W5*	3	1.60	300	65	10	11.03.2008	G	KWPN	156	6	56	56	44	2054	71	M	3	3	no	spread	oxer	left	no	0	0	41,3	no
Zurich	The Longines FEI World Cup Jumping - Zurich round B	28.01.2018	CSI-W5*	3	1.60	300	65	10	11.03.2008	G	KWPN	156	6	56	56	44	2054	71	M	4	4a	yes	vertical	vertical	right	yes	4	0	41,3	no
Zurich	The Longines FEI World Cup Jumping - Zurich round B	28.01.2018	CSI-W5*	3	1.60	300	65	10	11.03.2008	G	KWPN	156	6	56	56	44	2054	71	M	5	4b	yes	spread	oxer	right	yes	4	0	41,3	no
Zurich	The Longines FEI World Cup Jumping - Zurich round B	28.01.2018	CSI-W5*	3	1.60	300	65	10	11.03.2008	G	KWPN	156	6	56	56	44	2054	71	M	6	5	no	vertical	vertical	right	no	0	0	41,3	no
Zurich	The Longines FEI World Cup Jumping - Zurich round B	28.01.2018	CSI-W5*	3	1.60	300	65	10	11.03.2008	G	KWPN	156	6	56	56	44	2054	71	M	7	6	no	vertical	vertical	right	no	0	0	41,3	no
Zurich	The Longines FEI World Cup Jumping - Zurich round B	28.01.2018	CSI-W5*	3	1.60	300	65	10	11.03.2008	G	KWPN	156	6	56	56	44	2054	71	M	8	7	no	spread	oxer	left	no	0	0	41,3	no
Zurich	The Longines FEI World Cup Jumping - Zurich round B	28.01.2018	CSI-W5*	3	1.60	300	65	10	11.03.2008	G	KWPN	156	6	56	56	44	2054	71	M	9	8	no	spread	oxer with water	left	no	0	0	41,3	no
Zurich	The Longines FEI World Cup Jumping - Zurich round A	28.01.2018	CSI-W5*	3	1.60	430	71	10	25.05.2008	M	KWPN	131	2	65	70	43	1682	26	M	1	1	no	vertical	vertical	right	no	0	0	75,48	no
Zurich	The Longines FEI World Cup Jumping - Zurich round A	28.01.2018	CSI-W5*	3	1.60	430	71	10	25.05.2008	M	KWPN	131	2	65	70	43	1682	26	M	2	2	no	spread	oxer	right	no	0	0	75,48	no
Zurich	The Longines FEI World Cup Jumping - Zurich round A	28.01.2018	CSI-W5*	3	1.60	430	71	10	25.05.2008	M	KWPN	131	2	65	70	43	1682	26	M	3	3	no	vertical	wall	right	no	0	0	75,48	no
Zurich	The Longines FEI World Cup Jumping - Zurich round A	28.01.2018	CSI-W5*	3	1.60	430	71	10	25.05.2008	M	KWPN	131	2	65	70	43	1682	26	M	4	4	no	spread	oxer with water	left	no	0	0	75,48	no
Zurich	The Longines FEI World Cup Jumping - Zurich round A	28.01.2018	CSI-W5*	3	1.60	430	71	10	25.05.2008	M	KWPN	131	2	65	70	43	1682	26	M	5	5	no	vertical	vertical	left	no	0	0	75,48	no
Zurich	The Longines FEI World Cup Jumping - Zurich round A	28.01.2018	CSI-W5*	3	1.60	430	71	10	25.05.2008	M	KWPN	131	2	65	70	43	1682	26	M	6	6a	yes	spread	oxer	left	yes	4	0	75,48	no
Zurich	The Longines FEI World Cup Jumping - Zurich round A	28.01.2018	CSI-W5*	3	1.60	430	71	10	25.05.2008	M	KWPN	131	2	65	70	43	1682	26	M	7	6b	yes	spread	oxer	left	no	0	0	75,48	no
Zurich	The Longines FEI World Cup Jumping - Zurich round A	28.01.2018	CSI-W5*	3	1.60	430	71	10	25.05.2008	M	KWPN	131	2	65	70	43	1682	26	M	8	6c	yes	vertical	vertical	left	no	0	0	75,48	no
Zurich	The Longines FEI World Cup Jumping - Zurich round A	28.01.2018	CSI-W5*	3	1.60	430	71	10	25.05.2008	M	KWPN	131	2	65	70	43	1682	26	M	9	7	no	spread	oxer	right	no	0	0	75,48	no
Zurich	The Longines FEI World Cup Jumping - Zurich round A	28.01.2018	CSI-W5*	3	1.60	430	71	10	25.05.2008	M	KWPN	131	2	65	70	43	1682	26	M	10	8a	yes	vertical	vertical	right	no	0	0	75,48	no
Zurich	The Longines FEI World Cup Jumping - Zurich round A	28.01.2018	CSI-W5*	3	1.60	430	71	10	25.05.2008	M	KWPN	131	2	65	70	43	1682	26	M	11	8b	yes	spread	oxer	right	yes	4	0	75,48	no
Zurich	The Longines FEI World Cup Jumping - Zurich round A	28.01.2018	CSI-W5*	3	1.60	430	71	10	25.05.2008	M	KWPN	131	2	65	70	43	1682	26	M	12	9	no	vertical	vertical	right	no	0	0	75,48	no
Zurich	The Longines FEI World Cup Jumping - Zurich round A	28.01.2018	CSI-W5*	3	1.60	430	71	10	25.05.2008	M	KWPN	131	2	65	70	43	1682	26	M	13	10	no	spread	triple bar	right	no	0	0	75,48	no
Zurich	The Longines FEI World Cup Jumping - Zurich round A	28.01.2018	CSI-W5*	3	1.60	430	71	10	25.05.2008	M	KWPN	131	2	65	70	43	1682	26	M	14	11	no	spread	oxer	left	no	0	0	75,48	no
Zurich	The Longines FEI World Cup Jumping - Zurich round A	28.01.2018	CSI-W5*	3	1.60	430	71	10	25.05.2008	M	KWPN	131	2	65	70	43	1682	26	M	15	12	no	vertical	vertical with water	left	yes	4	0	75,48	no
Zurich	The Longines FEI World Cup Jumping - Zurich round A	28.01.2018	CSI-W5*	3	1.60	430	71	10	25.05.2008	M	KWPN	131	2	65	70	43	1682	26	M	16	13	no	spread	oxer	straight	no	0	1	75,48	no
Zurich	The Longines FEI World Cup Jumping - Zurich round A	28.01.2018	CSI-W5*	3	1.60	430	12	12	01.04.2006	S	KWPN	105	2	68	67	31	1737	39	F	1	1	no	vertical	vertical	right	no	0	0	71,64	no
Zurich	The Longines FEI World Cup Jumping - Zurich round A	28.01.2018	CSI-W5*	3	1.60	430	12	12	01.04.2006	S	KWPN	105	2	68	67	31	1737	39	F	2	2	no	spread	oxer	right	no	0	0	71,64	no
Zurich	The Longines FEI World Cup Jumping - Zurich round A	28.01.2018	CSI-W5*	3	1.60	430	12	12	01.04.2006	S	KWPN	105	2	68	67	31	1737	39	F	3	3	no	vertical	wall	right	no	0	0	71,64	no
Zurich	The Longines FEI World Cup Jumping - Zurich round A	28.01.2018	CSI-W5*	3	1.60	430	12	12	01.04.2006	S	KWPN	105	2	68	67	31	1737	39	F	4	4	no	spread	oxer with water	left	no	0	0	71,64	no
Zurich	The Longines FEI World Cup Jumping - Zurich round A	28.01.2018	CSI-W5*	3	1.60	430	12	12	01.04.2006	S	KWPN	105	2	68	67	31	1737	39	F	5	5	no	vertical	vertical	left	no	0	0	71,64	no
Zurich	The Longines FEI World Cup Jumping - Zurich round A	28.01.2018	CSI-W5*	3	1.60	430	12	12	01.04.2006	S	KWPN	105	2	68	67	31	1737	39	F	6	6a	yes	spread	oxer	left	no	0	0	71,64	no
Zurich	The Longines FEI World Cup Jumping - Zurich round A	28.01.2018	CSI-W5*	3	1.60	430	12	12	01.04.2006	S	KWPN	105	2	68	67	31	1737	39	F	7	6b	yes	spread	oxer	left	no	0	0	71,64	no
Zurich	The Longines FEI World Cup Jumping - Zurich round A	28.01.2018	CSI-W5*	3	1.60	430	12	12	01.04.2006	S	KWPN	105	2	68	67	31	1737	39	F	8	6c	yes	vertical	vertical	left	no	0	0	71,64	no
Zurich	The Longines FEI World Cup Jumping - Zurich round A	28.01.2018	CSI-W5*	3	1.60	430	12	12	01.04.2006	S	KWPN	105	2	68	67	31	1737	39	F	9	7	no	spread	oxer	right	no	0	0	71,64	no
Zurich	The Longines FEI World Cup Jumping - Zurich round A	28.01.2018	CSI-W5*	3	1.60	430	12	12	01.04.2006	S	KWPN	105	2	68	67	31	1737	39	F	10	8a	yes	vertical	vertical	right	no	0	0	71,64	no
Zurich	The Longines FEI World Cup Jumping - Zurich round A	28.01.2018	CSI-W5*	3	1.60	430	12	12	01.04.2006	S	KWPN	105	2	68	67	31	1737	39	F	11	8b	yes	spread	oxer	right	no	0	0	71,64	no
Zurich	The Longines FEI World Cup Jumping - Zurich round A	28.01.2018	CSI-W5*	3	1.60	430	12	12	01.04.2006	S	KWPN	105	2	68	67	31	1737	39	F	12	9	no	vertical	vertical	right	no	0	0	71,64	no
Zurich	The Longines FEI World Cup Jumping - Zurich round A	28.01.2018	CSI-W5*	3	1.60	430	12	12	01.04.2006	S	KWPN	105	2	68	67	31	1737	39	F	13	10	no	spread	triple bar	right	no	0	0	71,64	no
Zurich	The Longines FEI World Cup Jumping - Zurich round A	28.01.2018	CSI-W5*	3	1.60	430	12	12	01.04.2006	S	KWPN	105	2	68	67	31	1737	39	F	14	11	no	spread	oxer	left	no	0	0	71,64	no
Zurich	The Longines FEI World Cup Jumping - Zurich round A	28.01.2018	CSI-W5*	3	1.60	430	12	12	01.04.2006	S	KWPN	105	2	68	67	31	1737	39	F	15	12	no	vertical	vertical with water	left	yes	4	0	71,64	no
Zurich	The Longines FEI World Cup Jumping - Zurich round A	28.01.2018	CSI-W5*	3	1.60	430	12	12	01.04.2006	S	KWPN	105	2	68	67	31	1737	39	F	16	13	no	spread	oxer	straight	no	0	0	71,64	no
Zurich	The Longines FEI World Cup Jumping - Zurich round A	28.01.2018	CSI-W5*	3	1.60	430	211	13	11.05.2005	S	SBS	150	4	70	43	38	1891	85	F	1	1	no	vertical	vertical	right	no	0	0	72,07	no
Zurich	The Longines FEI World Cup Jumping - Zurich round A	28.01.2018	CSI-W5*	3	1.60	430	211	13	11.05.2005	S	SBS	150	4	70	43	38	1891	85	F	2	2	no	spread	oxer	right	no	0	0	72,07	no
Zurich	The Longines FEI World Cup Jumping - Zurich round A	28.01.2018	CSI-W5*	3	1.60	430	211	13	11.05.2005	S	SBS	150	4	70	43	38	1891	85	F	3	3	no	vertical	wall	right	no	0	0	72,07	no
Zurich	The Longines FEI World Cup Jumping - Zurich round A	28.01.2018	CSI-W5*	3	1.60	430	211	13	11.05.2005	S	SBS	150	4	70	43	38	1891	85	F	4	4	no	spread	oxer with water	left	no	0	0	72,07	no
Zurich	The Longines FEI World Cup Jumping - Zurich round A	28.01.2018	CSI-W5*	3	1.60	430	211	13	11.05.2005	S	SBS	150	4	70	43	38	1891	85	F	5	5	no	vertical	vertical	left	no	0	0	72,07	no
Zurich	The Longines FEI World Cup Jumping - Zurich round A	28.01.2018	CSI-W5*	3	1.60	430	211	13	11.05.2005	S	SBS	150	4	70	43	38	1891	85	F	6	6a	yes	spread	oxer	left	no	0	0	72,07	no
Zurich	The Longines FEI World Cup Jumping - Zurich round A	28.01.2018	CSI-W5*	3	1.60	430	211	13	11.05.2005	S	SBS	150	4	70	43	38	1891	85	F	7	6b	yes	spread	oxer	left	no	0	0	72,07	no
Zurich	The Longines FEI World Cup Jumping - Zurich round A	28.01.2018	CSI-W5*	3	1.60	430	211	13	11.05.2005	S	SBS	150	4	70	43	38	1891	85	F	8	6c	yes	vertical	vertical	left	no	0	0	72,07	no
Zurich	The Longines FEI World Cup Jumping - Zurich round A	28.01.2018	CSI-W5*	3	1.60	430	211	13	11.05.2005	S	SBS	150	4	70	43	38	1891	85	F	9	7	no	spread	oxer	right	no	0	0	72,07	no
Zurich	The Longines FEI World Cup Jumping - Zurich round A	28.01.2018	CSI-W5*	3	1.60	430	211	13	11.05.2005	S	SBS	150	4	70	43	38	1891	85	F	10	8a	yes	vertical	vertical	right	no	0	0	72,07	no
Zurich	The Longines FEI World Cup Jumping - Zurich round A	28.01.2018	CSI-W5*	3	1.60	430	211	13	11.05.2005	S	SBS	150	4	70	43	38	1891	85	F	11	8b	yes	spread	oxer	right	no	0	0	72,07	no

Zurich	The Longines FEI World Cup Jumping - Zurich round A	28.01.2018	CSI-W5*	3	1.60	430	211	13	11.05.2005	S	SBS	150	4	70	43	38	1891	85	F	12	9	no	vertical	vertical	right	yes	4	0	72,07	no
Zurich	The Longines FEI World Cup Jumping - Zurich round A	28.01.2018	CSI-W5*	3	1.60	430	211	13	11.05.2005	S	SBS	150	4	70	43	38	1891	85	F	13	10	no	spread	triple bar	right	no	0	0	72,07	no
Zurich	The Longines FEI World Cup Jumping - Zurich round A	28.01.2018	CSI-W5*	3	1.60	430	211	13	11.05.2005	S	SBS	150	4	70	43	38	1891	85	F	14	11	no	spread	oxer	left	no	0	0	72,07	no
Zurich	The Longines FEI World Cup Jumping - Zurich round A	28.01.2018	CSI-W5*	3	1.60	430	211	13	11.05.2005	S	SBS	150	4	70	43	38	1891	85	F	15	12	no	vertical	vertical with water	left	no	0	0	72,07	no
Zurich	The Longines FEI World Cup Jumping - Zurich round A	28.01.2018	CSI-W5*	3	1.60	430	211	13	11.05.2005	S	SBS	150	4	70	43	38	1891	85	F	16	13	no	spread	oxer	straight	no	0	0	72,07	no
Zurich	The Longines FEI World Cup Jumping - Zurich round A	28.01.2018	CSI-W5*	3	1.60	430	164	15	15.03.2003	G	SF	197	13	71	12	44	2177	93	M	1	1	no	vertical	vertical	right	no	0	0	70,48	no
Zurich	The Longines FEI World Cup Jumping - Zurich round A	28.01.2018	CSI-W5*	3	1.60	430	164	15	15.03.2003	G	SF	197	13	71	12	44	2177	93	M	2	2	no	spread	oxer	right	no	0	0	70,48	no
Zurich	The Longines FEI World Cup Jumping - Zurich round A	28.01.2018	CSI-W5*	3	1.60	430	164	15	15.03.2003	G	SF	197	13	71	12	44	2177	93	M	3	3	no	vertical	wall	right	no	0	0	70,48	no
Zurich	The Longines FEI World Cup Jumping - Zurich round A	28.01.2018	CSI-W5*	3	1.60	430	164	15	15.03.2003	G	SF	197	13	71	12	44	2177	93	M	4	4	no	spread	oxer with water	left	no	0	0	70,48	no
Zurich	The Longines FEI World Cup Jumping - Zurich round A	28.01.2018	CSI-W5*	3	1.60	430	164	15	15.03.2003	G	SF	197	13	71	12	44	2177	93	M	5	5	no	vertical	vertical	left	no	0	0	70,48	no
Zurich	The Longines FEI World Cup Jumping - Zurich round A	28.01.2018	CSI-W5*	3	1.60	430	164	15	15.03.2003	G	SF	197	13	71	12	44	2177	93	M	6	6a	yes	spread	oxer	left	no	0	0	70,48	no
Zurich	The Longines FEI World Cup Jumping - Zurich round A	28.01.2018	CSI-W5*	3	1.60	430	164	15	15.03.2003	G	SF	197	13	71	12	44	2177	93	M	7	6b	yes	spread	oxer	left	no	0	0	70,48	no
Zurich	The Longines FEI World Cup Jumping - Zurich round A	28.01.2018	CSI-W5*	3	1.60	430	164	15	15.03.2003	G	SF	197	13	71	12	44	2177	93	M	8	6c	yes	vertical	vertical	left	no	0	0	70,48	no
Zurich	The Longines FEI World Cup Jumping - Zurich round A	28.01.2018	CSI-W5*	3	1.60	430	164	15	15.03.2003	G	SF	197	13	71	12	44	2177	93	M	9	7	no	spread	oxer	right	no	0	0	70,48	no
Zurich	The Longines FEI World Cup Jumping - Zurich round A	28.01.2018	CSI-W5*	3	1.60	430	164	15	15.03.2003	G	SF	197	13	71	12	44	2177	93	M	10	8a	yes	vertical	vertical	right	no	0	0	70,48	no
Zurich	The Longines FEI World Cup Jumping - Zurich round A	28.01.2018	CSI-W5*	3	1.60	430	164	15	15.03.2003	G	SF	197	13	71	12	44	2177	93	M	11	8b	yes	spread	oxer	right	no	0	0	70,48	no
Zurich	The Longines FEI World Cup Jumping - Zurich round A	28.01.2018	CSI-W5*	3	1.60	430	164	15	15.03.2003	G	SF	197	13	71	12	44	2177	93	M	12	9	no	vertical	vertical	right	yes	4	0	70,48	no
Zurich	The Longines FEI World Cup Jumping - Zurich round A	28.01.2018	CSI-W5*	3	1.60	430	164	15	15.03.2003	G	SF	197	13	71	12	44	2177	93	M	13	10	no	spread	triple bar	right	no	0	0	70,48	no
Zurich	The Longines FEI World Cup Jumping - Zurich round A	28.01.2018	CSI-W5*	3	1.60	430	164	15	15.03.2003	G	SF	197	13	71	12	44	2177	93	M	14	11	no	spread	oxer	left	no	0	0	70,48	no
Zurich	The Longines FEI World Cup Jumping - Zurich round A	28.01.2018	CSI-W5*	3	1.60	430	164	15	15.03.2003	G	SF	197	13	71	12	44	2177	93	M	15	12	no	vertical	vertical with water	left	no	0	0	70,48	no
Zurich	The Longines FEI World Cup Jumping - Zurich round A	28.01.2018	CSI-W5*	3	1.60	430	164	15	15.03.2003	G	SF	197	13	71	12	44	2177	93	M	16	13	no	spread	oxer	straight	no	0	0	70,48	no
Zurich	The Longines FEI World Cup Jumping - Zurich round A	28.01.2018	CSI-W5*	3	1.60	430	99	12	08.06.2006	G	BWP	164	10	72	26	38	2171	177	M	1	1	no	vertical	vertical	right	no	0	0	70,02	no
Zurich	The Longines FEI World Cup Jumping - Zurich round A	28.01.2018	CSI-W5*	3	1.60	430	99	12	08.06.2006	G	BWP	164	10	72	26	38	2171	177	M	2	2	no	spread	oxer	right	no	0	0	70,02	no
Zurich	The Longines FEI World Cup Jumping - Zurich round A	28.01.2018	CSI-W5*	3	1.60	430	99	12	08.06.2006	G	BWP	164	10	72	26	38	2171	177	M	3	3	no	vertical	wall	right	no	0	0	70,02	no
Zurich	The Longines FEI World Cup Jumping - Zurich round A	28.01.2018	CSI-W5*	3	1.60	430	99	12	08.06.2006	G	BWP	164	10	72	26	38	2171	177	M	4	4	no	spread	oxer with water	left	no	0	0	70,02	no
Zurich	The Longines FEI World Cup Jumping - Zurich round A	28.01.2018	CSI-W5*	3	1.60	430	99	12	08.06.2006	G	BWP	164	10	72	26	38	2171	177	M	5	5	no	vertical	vertical	left	no	0	0	70,02	no
Zurich	The Longines FEI World Cup Jumping - Zurich round A	28.01.2018	CSI-W5*	3	1.60	430	99	12	08.06.2006	G	BWP	164	10	72	26	38	2171	177	M	6	6a	yes	spread	oxer	left	no	0	0	70,02	no
Zurich	The Longines FEI World Cup Jumping - Zurich round A	28.01.2018	CSI-W5*	3	1.60	430	99	12	08.06.2006	G	BWP	164	10	72	26	38	2171	177	M	7	6b	yes	spread	oxer	left	no	0	0	70,02	no
Zurich	The Longines FEI World Cup Jumping - Zurich round A	28.01.2018	CSI-W5*	3	1.60	430	99	12	08.06.2006	G	BWP	164	10	72	26	38	2171	177	M	8	6c	yes	vertical	vertical	left	no	0	0	70,02	no
Zurich	The Longines FEI World Cup Jumping - Zurich round A	28.01.2018	CSI-W5*	3	1.60	430	99	12	08.06.2006	G	BWP	164	10	72	26	38	2171	177	M	9	7	no	spread	oxer	right	no	0	0	70,02	no
Zurich	The Longines FEI World Cup Jumping - Zurich round A	28.01.2018	CSI-W5*	3	1.60	430	99	12	08.06.2006	G	BWP	164	10	72	26	38	2171	177	M	10	8a	yes	vertical	vertical	right	no	0	0	70,02	no
Zurich	The Longines FEI World Cup Jumping - Zurich round A	28.01.2018	CSI-W5*	3	1.60	430	99	12	08.06.2006	G	BWP	164	10	72	26	38	2171	177	M	11	8b	yes	spread	oxer	right	no	0	0	70,02	no
Zurich	The Longines FEI World Cup Jumping - Zurich round A	28.01.2018	CSI-W5*	3	1.60	430	99	12	08.06.2006	G	BWP	164	10	72	26	38	2171	177	M	12	9	no	vertical	vertical	right	no	0	0	70,02	no
Zurich	The Longines FEI World Cup Jumping - Zurich round A	28.01.2018	CSI-W5*	3	1.60	430	99	12	08.06.2006	G	BWP	164	10	72	26	38	2171	177	M	13	10	no	spread	triple bar	right	no	0	0	70,02	no
Zurich	The Longines FEI World Cup Jumping - Zurich round A	28.01.2018	CSI-W5*	3	1.60	430	99	12	08.06.2006	G	BWP	164	10	72	26	38	2171	177	M	14	11	no	spread	oxer	left	no	0	0	70,02	no
Zurich	The Longines FEI World Cup Jumping - Zurich round A	28.01.2018	CSI-W5*	3	1.60	430	99	12	08.06.2006	G	BWP	164	10	72	26	38	2171	177	M	15	12	no	vertical	vertical with water	left	no	0	0	70,02	no
Zurich	The Longines FEI World Cup Jumping - Zurich round A	28.01.2018	CSI-W5*	3	1.60	430	99	12	08.06.2006	G	BWP	164	10	72	26	38	2171	177	M	16	13	no	spread	oxer	straight	no	0	0	70,02	no
Zurich	The Longines FEI World Cup Jumping - Zurich round B	28.01.2018	CSI-W5*	3	1.60	300	99	12	08.06.2006	G	BWP	164	10	72	26	38	2171	177	M	1	1	no	spread	oxer	left	no	0	0	40,36	no
Zurich	The Longines FEI World Cup Jumping - Zurich round B	28.01.2018	CSI-W5*	3	1.60	300	99	12	08.06.2006	G	BWP	164	10	72	26	38	2171	177	M	2	2	no	vertical	vertical	left	no	0	0	40,36	no
Zurich	The Longines FEI World Cup Jumping - Zurich round B	28.01.2018	CSI-W5*	3	1.60	300	99	12	08.06.2006	G	BWP	164	10	72	26	38	2171	177	M	3	3	no	spread	oxer	left	yes	4	0	40,36	no
Zurich	The Longines FEI World Cup Jumping - Zurich round B	28.01.2018	CSI-W5*	3	1.60	300	99	12	08.06.2006	G	BWP	164	10	72	26	38	2171	177	M	4	4a	yes	vertical	vertical	right	no	0	0	40,36	no
Zurich	The Longines FEI World Cup Jumping - Zurich round B	28.01.2018	CSI-W5*	3	1.60	300	99	12	08.06.2006	G	BWP	164	10	72	26	38	2171	177	M	5	4b	yes	spread	oxer	right	no	0	0	40,36	no
Zurich	The Longines FEI World Cup Jumping - Zurich round B	28.01.2018	CSI-W5*	3	1.60	300	99	12	08.06.2006	G	BWP	164	10	72	26	38	2171	177	M	6	5	no	vertical	vertical	right	no	0	0	40,36	no
Zurich	The Longines FEI World Cup Jumping - Zurich round B	28.01.2018	CSI-W5*	3	1.60	300	99	12	08.06.2006	G	BWP	164	10	72	26	38	2171	177	M	7	6	no	vertical	vertical	right	no	0	0	40,36	no
Zurich	The Longines FEI World Cup Jumping - Zurich round B	28.01.2018	CSI-W5*	3	1.60	300	99	12	08.06.2006	G	BWP	164	10	72	26	38	2171	177	M	8	7	no	spread	oxer	left	no	0	0	40,36	no
Zurich	The Longines FEI World Cup Jumping - Zurich round B	28.01.2018	CSI-W5*	3	1.60	300	99	12	08.06.2006	G	BWP	164	10	72	26	38	2171	177	M	9	8	no	spread	oxer with water	left	no	0	0	40,36	no
Zurich	The Longines FEI World Cup Jumping - Zurich round A	28.01.2018	CSI-W5*	3	1.60	430	173	13	14.07.2005	G	SF	229	2	74	65	34	1260	61	M	1	1	no	vertical	vertical	right	no	0	0	72,35	no
Zurich	The Longines FEI World Cup Jumping - Zurich round A	28.01.2018	CSI-W5*	3	1.60	430	173	13	14.07.2005	G	SF	229	2	74	65	34	1260	61	M	2	2	no	spread	oxer	right	no	0	0	72,35	no
Zurich	The Longines FEI World Cup Jumping - Zurich round A	28.01.2018	CSI-W5*	3	1.60	430	173	13	14.07.2005	G	SF	229	2	74	65	34	1260	61	M	3	3	no	vertical	wall	right	no	0	0	72,35	no
Zurich	The Longines FEI World Cup Jumping - Zurich round A	28.01.2018	CSI-W5*	3	1.60	430	173	13	14.07.2005	G	SF	229	2	74	65	34	1260	61	M	4	4	no	spread	oxer with water	left	no	0	0	72,35	no
Zurich	The Longines FEI World Cup Jumping - Zurich round A	28.01.2018	CSI-W5*	3	1.60	430	173	13	14.07.2005	G	SF	229	2	74	65	34	1260	61	M	5	5	no	vertical	vertical	left	no	0	0	72,35	no
Zurich	The Longines FEI World Cup Jumping - Zurich round A	28.01.2018	CSI-W5*	3	1.60	430	173	13	14.07.2005	G	SF	229	2	74	65	34	1260	61	M	6	6a	yes	spread	oxer	left	no	0	0	72,35	no
Zurich	The Longines FEI World Cup Jumping - Zurich round A	28.01.2018	CSI-W5*	3	1.60	430	173	13	14.07.2005	G	SF	229	2	74	65	34	1260	61	M	7	6b	yes	spread	oxer	left	no	0	0	72,35	no
Zurich	The Longines FEI World Cup Jumping - Zurich round A	28.01.2018	CSI-W5*	3	1.60	430	173	13	14.07.2005	G	SF	229	2	74	65	34	1260	61	M	8	6c	yes	vertical	vertical	left	no	0	0	72,35	no
Zurich	The Longines FEI World Cup Jumping - Zurich round A	28.01.2018	CSI-W5*	3	1.60	430	173	13	14.07.2005	G	SF	229	2	74	65	34	1260	61	M	9	7	no	spread	oxer	right	no	0	0	72,35	no
Zurich	The Longines FEI World Cup Jumping - Zurich round A	28.01.2018	CSI-W5*	3	1.60	430	173	13	14.07.2005	G	SF	229	2	74	65	34	1260	61	M	10	8a	yes	vertical	vertical	right	no	0	0	72,35	no
Zurich	The Longines FEI World Cup Jumping - Zurich round A	28.01.2018	CSI-W5*	3	1.60	430	173	13	14.07.2005	G	SF	229	2	74	65	34	1260	61	M	11	8b	yes	spread	oxer	right	no	0	0	72,35	no
Zurich	The Longines FEI World Cup Jumping - Zurich round A	28.01.2018	CSI-W5*	3	1.60	430	173	13	14.07.2005	G	SF	229	2	74	65	34	1260	61	M	12	9	no	vertical	vertical	right	no	0	0	72,35	no
Zurich	The Longines FEI World Cup Jumping - Zurich round A	28.01.2018	CSI-W5*	3	1.60	430	173	13	14.07.2005	G	SF	229	2	74	65	34	1260	61	M	13	10	no	spread	triple bar	right	no	0	0	72,35	no
Zurich	The Longines FEI World Cup Jumping - Zurich round A	28.01.2018	CSI-W5*	3	1.60	430	173	13	14.07.2005	G	SF	229	2	74	65	34	1260	61	M	14	11	no	spread	oxer	left	no	0	0	72,35	no
Zurich	The Longines FEI World Cup Jumping - Zurich round A	28.01.2018	CSI-W5*	3	1.60	430	173	13	14.07.2005	G	SF	229	2	74	65	34	1260	61	M	15	12	no	vertical	vertical with water	left	no	0	0	72,35	no
Zurich	The Longines FEI World Cup Jumping - Zurich round A	28.01.2018	CSI-W5*	3	1.60	430	173	13	14.07.2005	G	SF	229	2	74	65	34	1260	61	M	16	13	no	spread	oxer	straight	no	0	0	72,35	no
Zurich	The Longines FEI World Cup Jumping - Zurich round B	28.01.2018	CSI-W5*	3	1.60	300	173	13	14.07.2005	G	SF	229	2	74	65	34	1260	61	M	1	1	no	spread	oxer	left	no	0	0	43,41	no
Zurich	The Longines FEI World Cup Jumping - Zurich round B	28.01.2018	CSI-W5*	3	1.60	300	173	13	14.07.2005	G	SF	229	2	74	65	34	1260	61	M	2	2	no	vertical	vertical	left	no	0	0	43,41	no
Zurich	The Longines FEI World Cup Jumping - Zurich round B	28.01.2018	CSI-W5*	3	1.60	300	173	13	14.07.2005	G	SF	229	2	74	65	34	1260	61	M	3	3	no	spread	oxer	left	no	0	0	43,41	no
Zurich	The Longines FEI World Cup Jumping - Zurich round B	28.01.2018	CSI-W5*	3	1.60	300	173	13	14.07.2005	G	SF	229	2	74	65	34	1260	61	M	4	4a	yes	vertical	vertical	right	no	0	0	43,41	no
Zurich	The Longines FEI World Cup Jumping - Zurich round B	28.01.2018	CSI-W5*	3	1.60	300	173	13	14.07.2005	G	SF	229	2	74	65	34	1260	61	M	5	4b	yes	spread	oxer	right	no	0	0	43,41	no
Zurich	The Longines FEI World Cup Jumping - Zurich round B	28.01.2018	CSI-W5*	3	1.60	300	173	13	14.07.2005	G	SF	229	2	74	65	34	1260	61	M	6	5	no	vertical	vertical	right	yes	4	0	43,41	no
Zurich	The Longines FEI World Cup Jumping - Zurich round B	28.01.2018	CSI-W5*	3	1.60	300	173	13	14.07.2005	G	SF	229	2	74	65	34	1260	61	M	7	6	no	vertical	vertical	right	no	0	0	43,41	no
Zurich	The Longines FEI World Cup Jumping - Zurich round B	28.01.2018	CSI-W5*	3	1.60	300	173	13	14.07.2005	G	SF	229	2	74	65	34	1260	61	M	8	7	no	spread	oxer	left	yes	4	0	43,41	no
Zurich	The Longines FEI World Cup Jumping - Zurich round B	28.01.2018	CSI-W5*	3	1.60	300	173	13	14.07.2005	G	SF	229	2	74	65	34	1260	61	M	9	8	no	spread	oxer with water	left	no	0	0	43,41	no
Zurich	The Longines FEI World Cup Jumping - Zurich round A	28.01.2018	CSI-W5*	3	1.60	430	58	13	04.02.2005	G	DSP	255	4	76	55	44	1886	48	M	1	1	no	vertical	vertical	right	no	0	0	74,5	no
Zurich	The Longines FEI World Cup Jumping - Zurich round A	28.01.2018	CSI-W5*	3	1.60	430	58	13	04.02.2005	G	DSP	255	4	76	55	44	1886	48	M	2	2	no	spread	oxer	right	no	0	0	74,5	no
Zurich	The Longines FEI World Cup Jumping - Zurich round A	28.01.2018	CSI-W5*	3	1.60	430	58	13	04.02.2005	G	DSP	255	4	76	55	44	1886	48	M	3	3	no	vertical	wall	right	no	0	0	74,5	no
Zurich	The Longines FEI World Cup Jumping - Zurich round A	28.01.2018	CSI-W5*	3	1.60	430	58	13	04.02.2005	G	DSP	255	4	76	55	44	1886	48	M	4	4	no	spread	oxer with water	left	no	0	0	74,5	no
Zurich	The Longines FEI World Cup Jumping - Zurich round A	28.01.2018	CSI-W5*	3	1.60	430	58	13	04.02.2005	G	DSP	255	4	76	55	44	1886	48	M	5	5	no	vertical	vertical	left	no	0	0	74,5	no
Zurich	The Longines FEI World Cup Jumping - Zurich round A	28.01.2018	CSI-W5*	3	1.60	430	58	13	04.02.2005	G	DSP	255	4	76	55	44	1886	48	M	6	6a	yes	spread	oxer	left	no	0	0	74,5	no
Zurich	The Longines FEI World Cup Jumping - Zurich round A	28.01.2018	CSI-W5*	3	1.60	430	58	13	04.02.2005	G	DSP	255	4	76	55	44	1886	48	M	7	6b	yes	spread	oxer	left	no	0	0	74,5	no
Zurich	The Longines FEI World Cup Jumping - Zurich round A	28.01.2018	CSI-W5*	3	1.60	430	58	13	04.02.2005	G	DSP	255	4	76	55	44	1886	48	M	8	6c	yes	vertical	vertical	left	no	0	0	74,5	no
Zurich	The Longines FEI World Cup Jumping - Zurich round A	28.01.2018	CSI-W5*	3	1.60	430	58	13	04.02.2005	G	DSP	255	4	76	55	44	1886	48	M	9	7	no	spread	oxer	right	yes	4	0	74,5	no
Zurich	The Longines FEI World Cup Jumping - Zurich round A	28.01.2018	CSI-W5*	3	1.60	430	58	13	04.02.2005	G	DSP	255	4	76	55	44	1886	48	M	10	8a	yes	vertical	vertical	right	no	0	0	74,5	no
Zurich	The Longines FEI World Cup Jumping - Zurich round A	28.01.2018	CSI-W5*	3	1.60	430	58	13	04.02.2005	G	DSP	255	4	76	55	44	1886	48	M	11	8b	yes	spread	oxer	right	no	0	0	74,5	no
Zurich	The Longines FEI World Cup Jumping - Zurich round A	28.01.2018	CSI-W5*	3	1.60	430	58	13	04.02.2005	G	DSP	255	4	76	55	44	1886	48	M	12	9	no	vertical	vertical	right	no	0	0	74,5	no
Zurich	The Longines FEI World Cup Jumping - Zurich round A	28.01.2018	CSI-W5*	3	1.60	430	58	13	04.02.2005	G	DSP	255	4	76	55	44	1886	48	M	13	10	no	spread	triple bar	right	no	0	0	74,5	no
Zurich	The Longines FEI World Cup Jumping - Zurich round A	28.01.2018	CSI-W5*	3	1.60	430	58	13	04.02.2005	G	DSP	255	4	76	55	44	1886	48	M	14	11	no	spread	oxer	left	no	0	0	74,5	no
Zurich	The Longines FEI World Cup Jumping - Zurich round A	28.01.2018	CSI-W5*	3	1.60	430	58	13	04.02.2005	G	DSP	255	4	76	55	44	1886	48	M	15	12	no	vertical	vertical with water	left	no	0	0	74,5	no
Zurich	The Longines FEI World Cup Jumping - Zurich round A	28.01.2018	CSI-W5*	3	1.60	430	58	13	04.02.2005	G	DSP	255	4	76	55	44	1886	48	M	16	13	no	spread	oxer	straight	no	0	1	74,5	no
Zurich	The Longines FEI World Cup Jumping - Zurich round A	28.01.2018	CSI-W5*	3	1.60	430	90	14	01.03.2004	M	HANN	185	6	86	36	48	1157	25	F	1	1	no	vertical	vertical	right	no	0	0	0,00	yes
Zurich	The Longines FEI World Cup Jumping - Zurich round A	28.01.2018	CSI-W5*	3	1.60	430	90	14	01.03.2004	M	HANN	185	6	86	36	48	1157	25	F	2	2	no	spread	oxer	right	no	0	0	0,00	yes
Zurich	The Longines FEI World Cup Jumping - Zurich round A	28.01.2018	CSI-W5*	3	1.60	430	90	14	01.03.2004	M	HANN	185	6	86	36	48	1157	25	F	3	3	no	vertical	wall	right	no	0	0	0,00	yes
Zurich	The Longines FEI World Cup Jumping - Zurich round A	28.01.2018	CSI-W5*	3	1.60	430	90	14	01.03.2004	M	HANN	185	6	86	36	48	1157	25	F	4	4	no	spread	oxer with water	left	no	0	0	0,00	yes
Zurich	The Longines FEI World Cup Jumping - Zurich round A	28.01.2018	CSI-W5*	3	1.60	430	90	14	01.03.2004	M	HANN	185	6	86	36	48	1157	25	F	5	5	no	vertical	vertical	left	no	0	0	0,00	yes
Zurich	The Longines FEI World Cup Jumping - Zurich round A	28.01.2018	CSI-W5*	3	1.60	430	90	14	01.03.2004	M	HANN	185	6	86	36	48	1157	25	F	6	6a	yes	spread	oxer	left	no	0	0	0,00	yes
Zurich	The Longines FEI World Cup Jumping - Zurich round A	28.01.2018	CSI-W5*	3	1.60	430	90	14	01.03.2004	M	HANN	185	6	86	36	48	1157	25	F	7	6b	yes	spread	oxer	left	no	0	0	0,00	yes
Zurich	The Longines FEI World Cup Jumping - Zurich round A	28.01.2018	CSI-W5*	3	1.60	430	90	14	01.03.2004	M	HANN	185	6	86	36	48	1157	25	F	8	6c	yes	vertical	vertical	left	no	0	0	0,00	yes
Zurich	The Longines FEI World Cup Jumping - Zurich round A	28.01.2018	CSI-W5*	3	1.60	430	90	14	01.03.2004	M	HANN	185	6	86	36	48	1157	25	F	9	7	no	spread	oxer	right	yes	4	0	0,00	yes
Zurich	The Longines FEI World Cup Jumping - Zurich round A	28.01.2018	CSI-W5*	3	1.60	430	90	14	01.03.2004	M	HANN	185	6	86	36	48	1157	25	F	10	8a	yes	vertical	vertical	right	no	0	0	0,00	yes
Zurich	The Longines FEI World Cup Jumping - Zurich round A	28.01.2018	CSI-W5*	3	1.60	430	90	14	01.03.2004	M	HANN	185	6	86	36	48	1157	25	F	11	8b	yes	spread	oxer	right	no	0	0	0,00	yes
Zurich	The Longines FEI World Cup Jumping - Zurich round A	28.01.2018	CSI-W5*	3	1.60	430	90	14	01.03.2004	M	HANN	185	6	86	36	48	1157	25	F	12	9	no	vertical	vertical	right	no	0	0	0,00	yes
Zurich	The Longines FEI World Cup Jumping - Zurich round A	28.01.2018	CSI-W5*	3	1.60	430	90	14	01.03.2004	M	HANN	185	6	86	36	48	1157	25	F	13	10	no	spread	triple bar	right	no	0	0	0,00	yes
Zurich	The Longines FEI World Cup Jumping - Zurich round A	28.01.2018	CSI-W5*	3	1.60	430	90	14	01.03.2004	M	HANN	185	6	86	36	48	1157	25	F	14	11	no	spread	oxer	left	no	0	0	0,00	yes
Zurich	The Longines FEI World Cup Jumping - Zurich round A	28.01.2018	CSI-W5*	3	1.60	430	90	14	01.03.2004	M	HANN	185	6	86	36	48	1157	25	F	15	12	no	vertical	vertical with water	left	no	0	0	0,00	yes
Zurich	The Longines FEI World Cup Jumping - Zurich round A	28.01.2018	CSI-W5*	3	1.60	430	90	14	01.03.2004	M	HANN	185	6	86	36	48	1157	25	F	16	13	no	spread	oxer	straight	no	0	0	0,00	yes
Zurich	The Longines FEI World Cup Jumping - Zurich round A	28.01.2018	CSI-W5*	3	1.60	430	147	12	04.05.2006	G	OLDBG	239	5	93	106	55	1594	47	M	1	1	no	vertical	vertical	right	no	0	0	72,27	no
Zurich	The Longines FEI World Cup Jumping - Zurich round A	28.01.2018	CSI-W5*	3	1.60	430	147	12	04.05.2006	G	OLDBG	239	5	93	106	55	1594	47	M	2	2	no	spread	oxer	right	no	0	0	72,27	no
Zurich	The Longines FEI World Cup Jumping - Zurich round A	28.01.2018	CSI-W5*	3	1.60	430	147	12	04.05.2006	G	OLDBG	239	5	93	106	55	1594	47	M	3	3	no	vertical	wall	right	no	0	0	72,27	no
Zurich	The Longines FEI World Cup Jumping - Zurich round A	28.01.2018	CSI-W5*	3	1.60	430	147	12	04.05.2006	G	OLDBG	239	5	93	106	55	1594	47	M	4	4	no	spread	oxer with water	left	no	0	0	72,27	no
Zurich	The Longines FEI World Cup Jumping - Zurich round A	28.01.2018	CSI-W5*	3	1.60	430	147	12	04.05.2006	G	OLDBG	239	5	93	106	55	1594	47	M	5	5	no	vertical	vertical	left	no	0	0	72,27	no
Zurich	The Longines FEI World Cup Jumping - Zurich round A	28.01.2018	CSI-W5*	3	1.60	430	147	12	04.05.2006	G	OLDBG	239	5	93	106	55	1594	47	M	6	6a	yes	spread	oxer	left	no	0	0	72,27	no
Zurich	The Longines FEI World Cup Jumping - Zurich round A	28.01.2018	CSI-W5*	3	1.60	430	147	12	04.05.2006	G	OLDBG	239	5	93	106	55	1594	47	M	7	6b	yes	spread	oxer	left	no	0	0	72,27	no
Zurich	The Longines FEI World Cup Jumping - Zurich round A	28.01.2018	CSI-W5*	3	1.60	430	147	12	04.05.2006	G	OLDBG	239	5	93	106	55	1594	47	M	8	6c	yes	vertical	vertical	left	no	0	0	72,27	no
Zurich	The Longines FEI World Cup Jumping - Zurich round A	28.01.2018	CSI-W5*	3	1.60	430	147	12	04.05.2006	G	OLDBG	239	5	93	106	55	1594	47	M	9	7	no	spread	oxer	right	no	0	0	72,27	no
Zurich	The Longines FEI World Cup Jumping - Zurich round A	28.01.2018	CSI-W5*	3	1.60	430	147	12	04.05.2006	G	OLDBG	239	5	93	106	55	1594	47	M	10	8a	yes	vertical	vertical	right	no	0	0	72,27	no
Zurich	The Longines FEI World Cup Jumping - Zurich round A	28.01.2018	CSI-W5*	3	1.60	430	147	12	04.05.2006	G	OLDBG	239	5	93	106	55	1594	47	M	11	8b	yes	spread	oxer	right	no	0	0	72,27	no
Zurich	The Longines FEI World Cup Jumping - Zurich round A	28.01.2018	CSI-W5*	3	1.60	430	147	12	04.05.2006	G	OLDBG	239	5	93	106	55	1594	47	M	12	9	no	vertical	vertical	right	no	0	0	72,27	no
Zurich	The Longines FEI World Cup Jumping - Zurich round A	28.01.2018	CSI-W5*	3	1.60	430	147	12	04.05.2006	G	OLDBG	239	5	93	106	55	1594	47	M	13	10	no	spread	triple bar	right	no	0	0	72,27	no
Zurich	The Longines FEI World Cup Jumping - Zurich round A	28.01.2018	CSI-W5*	3	1.60	430	147	12	04.05.2006	G	OLDBG	239	5	93	106	55	1594	47	M	14	11	no	spread	oxer	left	no	0	0	72,27	no
Zurich	The Longines FEI World Cup Jumping - Zurich round A	28.01.2018	CSI-W5*	3	1.60	430	147	12	04.05.2006	G	OLDBG	239	5	93	106	55	1594	47	M	15	12	no	vertical	vertical with water	left	no	0	0	72,27	no
Zurich	The Longines FEI World Cup Jumping - Zurich round A	28.01.2018	CSI-W5*	3	1.60	430	147	12	04.05.2006	G	OLDBG	239	5	93	106	55	1594	47	M	16	13	no	spread	oxer	straight	no	0	0	72,27	no
Zurich	The Longines FEI World Cup Jumping - Zurich round B	28.01.2018	CSI-W5*	3	1.60	300	147	12	04.05.2006	G	OLDBG	239	5	93	106	55	1594	47	M	1	1	no	spread	oxer	left	no	0	0	42,92	no
Zurich	The Longines FEI World Cup Jumping - Zurich round B	28.01.2018	CSI-W5*	3	1.60	300	147	12	04.05.2006	G	OLDBG	239	5	93	106	55	1594	47	M	2	2	no	vertical	vertical	left	no	0	0	42,92	no
Zurich	The Longines FEI World Cup Jumping - Zurich round B	28.01.2018	CSI-W5*	3	1.60	300	147	12	04.05.2006	G	OLDBG	239	5	93	106	55	1594	47	M	3	3	no	spread	oxer	left	no	0	0	42,92	no
Zurich	The Longines FEI World Cup Jumping - Zurich round B	28.01.2018	CSI-W5*	3	1.60	300	147	12	04.05.2006	G	OLDBG	239	5	93	106	55	1594	47	M	4	4a	yes	vertical	vertical	right	no	0	0	42,92	no
Zurich	The Longines FEI World Cup Jumping - Zurich round B	28.01.2018	CSI-W5*	3	1.60	300	147	12	04.05.2006	G	OLDBG	239	5	93	106	55	1594	47	M	5	4b	yes	spread	oxer	right	no	0	0	42,92	no
Zurich	The Longines FEI World Cup Jumping - Zurich round B	28.01.2018	CSI-W5*	3	1.60	300	147	12	04.05.2006	G	OLDBG	239	5	93	106	55	1594	47	M	6	5	no	vertical	vertical	right	no	0	0	42,92	no
Zurich	The Longines FEI World Cup Jumping - Zurich round B	28.01.2018	CSI-W5*	3	1.60	300	147	12	04.05.2006	G	OLDBG	239	5	93	106	55	1594	47	M	7	6	no	vertical	vertical	right	no	0	0	42,92	no
Zurich	The Longines FEI World Cup Jumping - Zurich round B	28.01.2018	CSI-W5*	3	1.60	300	147	12	04.05.2006	G	OLDBG	239	5	93	106	55	1594	47	M	8	7	no	spread	oxer	left	no	0	0	42,92	no
Zurich	The Longines FEI World Cup Jumping - Zurich round B	28.01.2018	CSI-W5*	3	1.60	300	147	12	04.05.2006	G	OLDBG	239	5	93	106	55	1594	47	M	9	8	no	spread	oxer with water	left	no	0	0	42,92	no
Zurich	The Longines FEI World Cup Jumping - Zurich round A	28.01.2018	CSI-W5*	3	1.60	430	64	11	26.06.2007	M	KWPN	163	22	101	46	53	686	44	M	1	1	no	vertical	vertical	right	no	0	0	72,31	no
Zurich	The Longines FEI World Cup Jumping - Zurich round A	28.01.2018	CSI-W5*	3	1.60	430	64	11	26.06.2007	M	KWPN	163	22	101	46	53	686	44	M	2	2	no	spread	oxer	right	no	0	0	72,31	no
Zurich	The Longines FEI World Cup Jumping - Zurich round A	28.01.2018	CSI-W5*	3	1.60	430	64	11	26.06.2007	M	KWPN	163	22	101	46	53	686	44	M	3	3	no	vertical	wall	right	no	0	0	72,31	no
Zurich	The Longines FEI World Cup Jumping - Zurich round A	28.01.2018	CSI-W5*	3	1.60	430	64	11	26.06.2007	M	KWPN	163	22	101	46	53	686	44	M	4	4	no	spread	oxer with water	left	no	0	0	72,31	no
Zurich	The Longines FEI World Cup Jumping - Zurich round A	28.01.2018	CSI-W5*	3	1.60	430	64	11	26.06.2007	M	KWPN	163	22	101	46	53	686	44	M	5	5	no	vertical	vertical	left	no	0	0	72,31	no
Zurich	The Longines FEI World Cup Jumping - Zurich round A	28.01.2018	CSI-W5*	3	1.60	430	64	11	26.06.2007	M	KWPN	163	22	101	46	53	686	44	M	6	6a	yes	spread	oxer	left	no	0	0	72,31	no
Zurich	The Longines FEI World Cup Jumping - Zurich round A	28.01.2018	CSI-W5*	3	1.60	430	64	11	26.06.2007	M	KWPN	163	22	101	46	53	686	44	M	7	6b	yes	spread	oxer	left	no	0	0	72,31	no
Zurich	The Longines FEI World Cup Jumping - Zurich round A	28.01.2018	CSI-W5*	3	1.60	430	64	11	26.06.2007	M	KWPN	163	22	101	46	53	686	44	M	8	6c	yes	vertical	vertical	left	no	0	0	72,31	no
Zurich	The Longines FEI World Cup Jumping - Zurich round A	28.01.2018	CSI-W5*	3	1.60	430	64	11	26.06.2007	M	KWPN	163	22	101	46	53	686	44	M	9	7	no	spread	oxer	right	no	0	0	72,31	no
Zurich	The Longines FEI World Cup Jumping - Zurich round A	28.01.2018	CSI-W5*	3	1.60	430	64	11	26.06.2007	M	KWPN	163	22	101	46	53	686	44	M	10	8a	yes	vertical	vertical	right	no	0	0	72,31	no
Zurich	The Longines FEI World Cup Jumping - Zurich round A	28.01.2018	CSI-W5*	3	1.60	430	64	11	26.06.2007	M	KWPN	163	22	101	46	53	686	44	M	11	8b	yes	spread	oxer	right	no	0	0	72,31	no
Zurich	The Longines FEI World Cup Jumping - Zurich round A	28.01.2018	CSI-W5*	3	1.60	430	64	11	26.06.2007	M	KWPN	163	22	101	46	53	686	44	M	12	9	no	vertical	vertical	right	no	0	0	72,31	no
Zurich	The Longines FEI World Cup Jumping - Zurich round A	28.01.2018	CSI-W5*	3	1.60	430	64	11	26.06.2007	M	KWPN	163	22	101	46	53	686	44	M	13	10	no	spread	triple bar	right	no	0	0	72,31	no
Zurich	The Longines FEI World Cup Jumping - Zurich round A	28.01.2018	CSI-W5*	3	1.60	430	64	11	26.06.2007	M	KWPN	163	22	101	46	53	686	44	M	14	11	no	spread	oxer	left	no	0	0	72,31	no
Zurich	The Longines FEI World Cup Jumping - Zurich round A	28.01.2018	CSI-W5*	3	1.60	430	64	11	26.06.2007	M	KWPN	163	22	101	46	53	686	44	M	15	12	no	vertical	vertical with water	left	no	0	0	72,31	no
Zurich	The Longines FEI World Cup Jumping - Zurich round A	28.01.2018	CSI-W5*	3	1.60	430	64	11	26.06.2007	M	KWPN	163	22	101	46	53	686	44	M	16	13	no	spread	oxer	straight	no	0	0	72,31	no
Zurich	The Longines FEI World Cup Jumping - Zurich round B	28.01.2018	CSI-W5*	3	1.60	300	64	11	26.06.2007	M	KWPN	163	22	101	46	53	686	44	M	1	1	no	spread	oxer	left	no	0	0	39,58	no
Zurich	The Longines FEI World Cup Jumping - Zurich round B	28.01.2018	CSI-W5*	3	1.60	300	64	11	26.06.2007	M	KWPN	163	22	101	46	53	686	44	M	2	2	no	vertical	vertical	left	no	0	0	39,58	no
Zurich	The Longines FEI World Cup Jumping - Zurich round B	28.01.2018	CSI-W5*	3	1.60	300	64	11	26.06.2007	M	KWPN	163	22	101	46	53	686	44	M	3	3	no	spread	oxer	left	no	0	0	39,58	no
Zurich	The Longines FEI World Cup Jumping - Zurich round B	28.01.2018	CSI-W5*	3	1.60	300	64	11	26.06.2007	M	KWPN	163	22	101	46	53	686	44	M	4	4a	yes	vertical	vertical	right	no	0	0	39,58	no
Zurich	The Longines FEI World Cup Jumping - Zurich round B	28.01.2018	CSI-W5*	3	1.60	300	64	11	26.06.2007	M	KWPN	163	22	101	46	53	686	44	M	5	4b	yes	spread	oxer	right	no	0	0	39,58	no
Zurich	The Longines FEI World Cup Jumping - Zurich round B	28.01.2018	CSI-W5*	3	1.60	300	64	11	26.06.2007	M	KWPN	163	22	101	46	53	686	44	M	6	5	no	vertical	vertical	right	no	0	0	39,58	no
Zurich	The Longines FEI World Cup Jumping - Zurich round B	28.01.2018	CSI-W5*	3	1.60	300	64	11	26.06.2007	M	KWPN	163	22	101	46	53	686	44	M	7	6	no	vertical	vertical	right	no	0	0	39,58	no
Zurich	The Longines FEI World Cup Jumping - Zurich round B	28.01.2018	CSI-W5*	3	1.60	300	64	11	26.06.2007	M	KWPN	163	22	101	46	53	686	44	M	8	7	no	spread	oxer	left	no	0	0	39,58	no
Zurich	The Longines FEI World Cup Jumping - Zurich round B	28.01.2018	CSI-W5*	3	1.60	300	64	11	26.06.2007	M	KWPN	163	22	101	46	53	686	44	M	9	8	no	spread	oxer with water	left	yes	4	0	39,58	no
Zurich	The Longines FEI World Cup Jumping - Zurich round A	28.01.2018	CSI-W5*	3	1.60	430	144	14	28.02.2004	G	DSP	186	2	103	171	32	1274	37	M	1	1	no	vertical	vertical	right	no	0	0	75,78	no
Zurich	The Longines FEI World Cup Jumping - Zurich round A	28.01.2018	CSI-W5*	3	1.60	430	144	14	28.02.2004	G	DSP	186	2	103	171	32	1274	37	M	2	2	no	spread	oxer	right	no	0	0	75,78	no
Zurich	The Longines FEI World Cup Jumping - Zurich round A	28.01.2018	CSI-W5*	3	1.60	430	144	14	28.02.2004	G	DSP	186	2	103	171	32	1274	37	M	3	3	no	vertical	wall	right	no	0	0	75,78	no
Zurich	The Longines FEI World Cup Jumping - Zurich round A	28.01.2018	CSI-W5*	3	1.60	430	144	14	28.02.2004	G	DSP	186	2	103	171	32	1274	37	M	4	4	no	spread	oxer with water	left	no	0	0	75,78	no
Zurich	The Longines FEI World Cup Jumping - Zurich round A	28.01.2018	CSI-W5*	3	1.60	430	144	14	28.02.2004	G	DSP	186	2	103	171	32	1274	37	M	5	5	no	vertical	vertical	left	no	0	0	75,78	no
Zurich	The Longines FEI World Cup Jumping - Zurich round A	28.01.2018	CSI-W5*	3	1.60	430	144	14	28.02.2004	G	DSP	186	2	103	171	32	1274	37	M	6	6a	yes	spread	oxer	left	no	0	0	75,78	no
Zurich	The Longines FEI World Cup Jumping - Zurich round A	28.01.2018	CSI-W5*	3	1.60	430	144	14	28.02.2004	G	DSP	186	2	103	171	32	1274	37	M	7	6b	yes	spread	oxer	left	no	0	0	75,78	no
Zurich	The Longines FEI World Cup Jumping - Zurich round A	28.01.2018	CSI-W5*	3	1.60	430	144	14	28.02.2004	G	DSP	186	2	103	171	32	1274	37	M	8	6c	yes	vertical	vertical	left	no	0	0	75,78	no
Zurich	The Longines FEI World Cup Jumping - Zurich round A	28.01.2018	CSI-W5*	3	1.60	430	144	14	28.02.2004	G	DSP	186	2	103	171	32	1274	37	M	9	7	no	spread	oxer	right	no	0	0	75,78	no
Zurich	The Longines FEI World Cup Jumping - Zurich round A	28.01.2018	CSI-W5*	3	1.60	430	144	14	28.02.2004	G	DSP	186	2	103	171	32	1274	37	M	10	8a	yes	vertical	vertical	right	no	0	0	75,78	no
Zurich	The Longines FEI World Cup Jumping - Zurich round A	28.01.2018	CSI-W5*	3	1.60	430	144	14	28.02.2004	G	DSP	186	2	103	171	32	1274	37	M	11	8b	yes	spread	oxer	right	yes	4	0	75,78	no
Zurich	The Longines FEI World Cup Jumping - Zurich round A	28.01.2018	CSI-W5*	3	1.60	430	144	14	28.02.2004	G	DSP	186	2	103	171	32	1274	37	M	12	9	no	vertical	vertical	right	yes	4	0	75,78	no
Zurich	The Longines FEI World Cup Jumping - Zurich round A	28.01.2018	CSI-W5*	3	1.60	430	144	14	28.02.2004	G	DSP	186	2	103	171	32	1274	37	M	13	10	no	spread	triple bar	right	no	0	0	75,78	no
Zurich	The Longines FEI World Cup Jumping - Zurich round A	28.01.2018	CSI-W5*	3	1.60	430	144	14	28.02.2004	G	DSP	186	2	103	171	32	1274	37	M	14	11	no	spread	oxer	left	yes	4	0	75,78	no
Zurich	The Longines FEI World Cup Jumping - Zurich round A	28.01.2018	CSI-W5*	3	1.60	430	144	14	28.02.2004	G	DSP	186	2	103	171	32	1274	37	M	15	12	no	vertical	vertical with water	left	no	0	0	75,78	no
Zurich	The Longines FEI World Cup Jumping - Zurich round A	28.01.2018	CSI-W5*	3	1.60	430	144	14	28.02.2004	G	DSP	186	2	103	171	32	1274	37	M	16	13	no	spread	oxer	straight	no	0	1	75,78	no
Zurich	The Longines FEI World Cup Jumping - Zurich round A	28.01.2018	CSI-W5*	3	1.60	430	4	12	19.06.2006	G	Bezplemenné	207	1	104	24	42	2261	97	M	1	1	no	vertical	vertical	right	no	0	0	73,83	no
Zurich	The Longines FEI World Cup Jumping - Zurich round A	28.01.2018	CSI-W5*	3	1.60	430	4	12	19.06.2006	G	Bezplemenné	207	1	104	24	42	2261	97	M	2	2	no	spread	oxer	right	no	0	0	73,83	no
Zurich	The Longines FEI World Cup Jumping - Zurich round A	28.01.2018	CSI-W5*	3	1.60	430	4	12	19.06.2006	G	Bezplemenné	207	1	104	24	42	2261	97	M	3	3	no	vertical	wall	right	no	0	0	73,83	no
Zurich	The Longines FEI World Cup Jumping - Zurich round A	28.01.2018	CSI-W5*	3	1.60	430	4	12	19.06.2006	G	Bezplemenné	207	1	104	24	42	2261	97	M	4	4	no	spread	oxer with water	left	no	0	0	73,83	no
Zurich	The Longines FEI World Cup Jumping - Zurich round A	28.01.2018	CSI-W5*	3	1.60	430	4	12	19.06.2006	G	Bezplemenné	207	1	104	24	42	2261	97	M	5	5	no	vertical	vertical	left	no	0	0	73,83	no
Zurich	The Longines FEI World Cup Jumping - Zurich round A	28.01.2018	CSI-W5*	3	1.60	430	4	12	19.06.2006	G	Bezplemenné	207	1	104	24	42	2261	97	M	6	6a	yes	spread	oxer	left	no	0	0	73,83	no
Zurich	The Longines FEI World Cup Jumping - Zurich round A	28.01.2018	CSI-W5*	3	1.60	430	4	12	19.06.2006	G	Bezplemenné	207	1	104	24	42	2261	97	M	7	6b	yes	spread	oxer	left	no	0	0	73,83	no
Zurich	The Longines FEI World Cup Jumping - Zurich round A	28.01.2018	CSI-W5*	3	1.60	430	4	12	19.06.2006	G	Bezplemenné	207	1	104	24	42	2261	97	M	8	6c	yes	vertical	vertical	left	no	0	0	73,83	no
Zurich	The Longines FEI World Cup Jumping - Zurich round A	28.01.2018	CSI-W5*	3	1.60	430	4	12	19.06.2006	G	Bezplemenné	207	1	104	24	42	2261	97	M	9	7	no	spread	oxer	right	no	0	0	73,83	no
Zurich	The Longines FEI World Cup Jumping - Zurich round A	28.01.2018	CSI-W5*	3	1.60	430	4	12	19.06.2006	G	Bezplemenné	207	1	104	24	42	2261	97	M	10	8a	yes	vertical	vertical	right	no	0	0	73,83	no
Zurich	The Longines FEI World Cup Jumping - Zurich round A	28.01.2018	CSI-W5*	3	1.60	430	4	12	19.06.2006	G	Bezplemenné	207	1	104	24	42	2261	97	M	11	8b	yes	spread	oxer	right	no	0	0	73,83	no
Zurich	The Longines FEI World Cup Jumping - Zurich round A	28.01.2018	CSI-W5*	3	1.60	430	4	12	19.06.2006	G	Bezplemenné	207	1	104	24	42	2261	97	M	12	9	no	vertical	vertical	right	no	0	0	73,83	no
Zurich	The Longines FEI World Cup Jumping - Zurich round A	28.01.2018	CSI-W5*	3	1.60	430	4	12	19.06.2006	G	Bezplemenné	207	1	104	24	42	2261	97	M	13	10	no	spread	triple bar	right	no	0	0	73,83	no
Zurich	The Longines FEI World Cup Jumping - Zurich round A	28.01.2018	CSI-W5*	3	1.60	430	4	12	19.06.2006	G	Bezplemenné	207	1	104	24	42	2261	97	M	14	11	no	spread	oxer	left	no	0	0	73,83	no
Zurich	The Longines FEI World Cup Jumping - Zurich round A	28.01.2018	CSI-W5*	3	1.60	430	4	12	19.06.2006	G	Bezplemenné	207	1	104	24	42	2261	97	M	15	12	no	vertical	vertical with water	left	no	0	0	73,83	no
Zurich	The Longines FEI World Cup Jumping - Zurich round A	28.01.2018	CSI-W5*	3	1.60	430	4	12	19.06.2006	G	Bezplemenné	207	1	104	24	42	2261	97	M	16	13	no	spread	oxer	straight	no	0	0	73,83	no
Zurich	The Longines FEI World Cup Jumping - Zurich round B	28.01.2018	CSI-W5*	3	1.60	300	4	12	19.06.2006	G	Bezplemenné	207	1	104	24	42	2261	97	M	1	1	no	spread	oxer	left	no	0	0	42,33	no
Zurich	The Longines FEI World Cup Jumping - Zurich round B	28.01.2018	CSI-W5*	3	1.60	300	4	12	19.06.2006	G	Bezplemenné	207	1	104	24	42	2261	97	M	2	2	no	vertical	vertical	left	no	0	0	42,33	no
Zurich	The Longines FEI World Cup Jumping - Zurich round B	28.01.2018	CSI-W5*	3	1.60	300	4	12	19.06.2006	G	Bezplemenné	207	1	104	24	42	2261	97	M	3	3	no	spread	oxer	left	no	0	0	42,33	no
Zurich	The Longines FEI World Cup Jumping - Zurich round B	28.01.2018	CSI-W5*	3	1.60	300	4	12	19.06.2006	G	Bezplemenné	207	1	104	24	42	2261	97	M	4	4a	yes	vertical	vertical	right	no	0	0	42,33	no
Zurich	The Longines FEI World Cup Jumping - Zurich round B	28.01.2018	CSI-W5*	3	1.60	300	4	12	19.06.2006	G	Bezplemenné	207	1	104	24	42	2261	97	M	5	4b	yes	spread	oxer	right	no	0	0	42,33	no
Zurich	The Longines FEI World Cup Jumping - Zurich round B	28.01.2018	CSI-W5*	3	1.60	300	4	12	19.06.2006	G	Bezplemenné	207	1	104	24	42	2261	97	M	6	5	no	vertical	vertical	right	no	0	0	42,33	no
Zurich	The Longines FEI World Cup Jumping - Zurich round B	28.01.2018	CSI-W5*	3	1.60	300	4	12	19.06.2006	G	Bezplemenné	207	1	104	24	42	2261	97	M	7	6	no	vertical	vertical	right	no	0	0	42,33	no
Zurich	The Longines FEI World Cup Jumping - Zurich round B	28.01.2018	CSI-W5*	3	1.60	300	4	12	19.06.2006	G	Bezplemenné	207	1	104	24	42	2261	97	M	8	7	no	spread	oxer	left	no	0	0	42,33	no
Zurich	The Longines FEI World Cup Jumping - Zurich round B	28.01.2018	CSI-W5*	3	1.60	300	4	12	19.06.2006	G	Bezplemenné	207	1	104	24	42	2261	97	M	9	8	no	spread	oxer with water	left	no	0	0	42,33	no
Zurich	The Longines FEI World Cup Jumping - Zurich round A	28.01.2018	CSI-W5*	3	1.60	430	190	14	22.03.2004	M	AES	269	11	112	74	33	1410	55	M	1	1	no	vertical	vertical	right	no	0	0	70,61	no
Zurich	The Longines FEI World Cup Jumping - Zurich round A	28.01.2018	CSI-W5*	3	1.60	430	190	14	22.03.2004	M	AES	269	11	112	74	33	1410	55	M	2	2	no	spread	oxer	right	no	0	0	70,61	no
Zurich	The Longines FEI World Cup Jumping - Zurich round A	28.01.2018	CSI-W5*	3	1.60	430	190	14	22.03.2004	M	AES	269	11	112	74	33	1410	55	M	3	3	no	vertical	wall	right	no	0	0	70,61	no
Zurich	The Longines FEI World Cup Jumping - Zurich round A	28.01.2018	CSI-W5*	3	1.60	430	190	14	22.03.2004	M	AES	269	11	112	74	33	1410	55	M	4	4	no	spread	oxer with water	left	no	0	0	70,61	no
Zurich	The Longines FEI World Cup Jumping - Zurich round A	28.01.2018	CSI-W5*	3	1.60	430	190	14	22.03.2004	M	AES	269	11	112	74	33	1410	55	M	5	5	no	vertical	vertical	left	no	0	0	70,61	no
Zurich	The Longines FEI World Cup Jumping - Zurich round A	28.01.2018	CSI-W5*	3	1.60	430	190	14	22.03.2004	M	AES	269	11	112	74	33	1410	55	M	6	6a	yes	spread	oxer	left	no	0	0	70,61	no
Zurich	The Longines FEI World Cup Jumping - Zurich round A	28.01.2018	CSI-W5*	3	1.60	430	190	14	22.03.2004	M	AES	269	11	112	74	33	1410	55	M	7	6b	yes	spread	oxer	left	no	0	0	70,61	no
Zurich	The Longines FEI World Cup Jumping - Zurich round A	28.01.2018	CSI-W5*	3	1.60	430	190	14	22.03.2004	M	AES	269	11	112	74	33	1410	55	M	8	6c	yes	vertical	vertical	left	no	0	0	70,61	no
Zurich	The Longines FEI World Cup Jumping - Zurich round A	28.01.2018	CSI-W5*	3	1.60	430	190	14	22.03.2004	M	AES	269	11	112	74	33	1410	55	M	9	7	no	spread	oxer	right	no	0	0	70,61	no
Zurich	The Longines FEI World Cup Jumping - Zurich round A	28.01.2018	CSI-W5*	3	1.60	430	190	14	22.03.2004	M	AES	269	11	112	74	33	1410	55	M	10	8a	yes	vertical	vertical	right	no	0	0	70,61	no
Zurich	The Longines FEI World Cup Jumping - Zurich round A	28.01.2018	CSI-W5*	3	1.60	430	190	14	22.03.2004	M	AES	269	11	112	74	33	1410	55	M	11	8b	yes	spread	oxer	right	no	0	0	70,61	no
Zurich	The Longines FEI World Cup Jumping - Zurich round A	28.01.2018	CSI-W5*	3	1.60	430	190	14	22.03.2004	M	AES	269	11	112	74	33	1410	55	M	12	9	no	vertical	vertical	right	no	0	0	70,61	no
Zurich	The Longines FEI World Cup Jumping - Zurich round A	28.01.2018	CSI-W5*	3	1.60	430	190	14	22.03.2004	M	AES	269	11	112	74	33	1410	55	M	13	10	no	spread	triple bar	right	no	0	0	70,61	no
Zurich	The Longines FEI World Cup Jumping - Zurich round A	28.01.2018	CSI-W5*	3	1.60	430	190	14	22.03.2004	M	AES	269	11	112	74	33	1410	55	M	14	11	no	spread	oxer	left	no	0	0	70,61	no
Zurich	The Longines FEI World Cup Jumping - Zurich round A	28.01.2018	CSI-W5*	3	1.60	430	190	14	22.03.2004	M	AES	269	11	112	74	33	1410	55	M	15	12	no	vertical	vertical with water	left	yes	4	0	70,61	no
Zurich	The Longines FEI World Cup Jumping - Zurich round A	28.01.2018	CSI-W5*	3	1.60	430	190	14	22.03.2004	M	AES	269	11	112	74	33	1410	55	M	16	13	no	spread	oxer	straight	no	0	0	70,61	no
Zurich	The Longines FEI World Cup Jumping - Zurich round A	28.01.2018	CSI-W5*	3	1.60	430	148	10	28.06.2008	G	Bezplemenné	125	0	120	550	27	986	18	F	1	1	no	vertical	vertical	right	no	0	0	72,44	no
Zurich	The Longines FEI World Cup Jumping - Zurich round A	28.01.2018	CSI-W5*	3	1.60	430	148	10	28.06.2008	G	Bezplemenné	125	0	120	550	27	986	18	F	2	2	no	spread	oxer	right	no	0	0	72,44	no
Zurich	The Longines FEI World Cup Jumping - Zurich round A	28.01.2018	CSI-W5*	3	1.60	430	148	10	28.06.2008	G	Bezplemenné	125	0	120	550	27	986	18	F	3	3	no	vertical	wall	right	no	0	0	72,44	no
Zurich	The Longines FEI World Cup Jumping - Zurich round A	28.01.2018	CSI-W5*	3	1.60	430	148	10	28.06.2008	G	Bezplemenné	125	0	120	550	27	986	18	F	4	4	no	spread	oxer with water	left	no	0	0	72,44	no
Zurich	The Longines FEI World Cup Jumping - Zurich round A	28.01.2018	CSI-W5*	3	1.60	430	148	10	28.06.2008	G	Bezplemenné	125	0	120	550	27	986	18	F	5	5	no	vertical	vertical	left	no	0	0	72,44	no
Zurich	The Longines FEI World Cup Jumping - Zurich round A	28.01.2018	CSI-W5*	3	1.60	430	148	10	28.06.2008	G	Bezplemenné	125	0	120	550	27	986	18	F	6	6a	yes	spread	oxer	left	no	0	0	72,44	no
Zurich	The Longines FEI World Cup Jumping - Zurich round A	28.01.2018	CSI-W5*	3	1.60	430	148	10	28.06.2008	G	Bezplemenné	125	0	120	550	27	986	18	F	7	6b	yes	spread	oxer	left	no	0	0	72,44	no
Zurich	The Longines FEI World Cup Jumping - Zurich round A	28.01.2018	CSI-W5*	3	1.60	430	148	10	28.06.2008	G	Bezplemenné	125	0	120	550	27	986	18	F	8	6c	yes	vertical	vertical	left	no	0	0	72,44	no
Zurich	The Longines FEI World Cup Jumping - Zurich round A	28.01.2018	CSI-W5*	3	1.60	430	148	10	28.06.2008	G	Bezplemenné	125	0	120	550	27	986	18	F	9	7	no	spread	oxer	right	no	0	0	72,44	no
Zurich	The Longines FEI World Cup Jumping - Zurich round A	28.01.2018	CSI-W5*	3	1.60	430	148	10	28.06.2008	G	Bezplemenné	125	0	120	550	27	986	18	F	10	8a	yes	vertical	vertical	right	no	0	0	72,44	no
Zurich	The Longines FEI World Cup Jumping - Zurich round A	28.01.2018	CSI-W5*	3	1.60	430	148	10	28.06.2008	G	Bezplemenné	125	0	120	550	27	986	18	F	11	8b	yes	spread	oxer	right	no	0	0	72,44	no
Zurich	The Longines FEI World Cup Jumping - Zurich round A	28.01.2018	CSI-W5*	3	1.60	430	148	10	28.06.2008	G	Bezplemenné	125	0	120	550	27	986	18	F	12	9	no	vertical	vertical	right	no	0	0	72,44	no
Zurich	The Longines FEI World Cup Jumping - Zurich round A	28.01.2018	CSI-W5*	3	1.60	430	148	10	28.06.2008	G	Bezplemenné	125	0	120	550	27	986	18	F	13	10	no	spread	triple bar	right	no	0	0	72,44	no
Zurich	The Longines FEI World Cup Jumping - Zurich round A	28.01.2018	CSI-W5*	3	1.60	430	148	10	28.06.2008	G	Bezplemenné	125	0	120	550	27	986	18	F	14	11	no	spread	oxer	left	no	0	0	72,44	no
Zurich	The Longines FEI World Cup Jumping - Zurich round A	28.01.2018	CSI-W5*	3	1.60	430	148	10	28.06.2008	G	Bezplemenné	125	0	120	550	27	986	18	F	15	12	no	vertical	vertical with water	left	yes	4	0	72,44	no
Zurich	The Longines FEI World Cup Jumping - Zurich round A	28.01.2018	CSI-W5*	3	1.60	430	148	10	28.06.2008	G	Bezplemenné	125	0	120	550	27	986	18	F	16	13	no	spread	oxer	straight	no	0	0	72,44	no
Zurich	The Longines FEI World Cup Jumping - Zurich round A	28.01.2018	CSI-W5*	3	1.60	430	22	13	01.03.2005	S	HOLST	155	8	121	2	38	1958	103	M	1	1	no	vertical	vertical	right	no	0	0	71,93	no
Zurich	The Longines FEI World Cup Jumping - Zurich round A	28.01.2018	CSI-W5*	3	1.60	430	22	13	01.03.2005	S	HOLST	155	8	121	2	38	1958	103	M	2	2	no	spread	oxer	right	no	0	0	71,93	no
Zurich	The Longines FEI World Cup Jumping - Zurich round A	28.01.2018	CSI-W5*	3	1.60	430	22	13	01.03.2005	S	HOLST	155	8	121	2	38	1958	103	M	3	3	no	vertical	wall	right	no	0	0	71,93	no
Zurich	The Longines FEI World Cup Jumping - Zurich round A	28.01.2018	CSI-W5*	3	1.60	430	22	13	01.03.2005	S	HOLST	155	8	121	2	38	1958	103	M	4	4	no	spread	oxer with water	left	no	0	0	71,93	no
Zurich	The Longines FEI World Cup Jumping - Zurich round A	28.01.2018	CSI-W5*	3	1.60	430	22	13	01.03.2005	S	HOLST	155	8	121	2	38	1958	103	M	5	5	no	vertical	vertical	left	no	0	0	71,93	no
Zurich	The Longines FEI World Cup Jumping - Zurich round A	28.01.2018	CSI-W5*	3	1.60	430	22	13	01.03.2005	S	HOLST	155	8	121	2	38	1958	103	M	6	6a	yes	spread	oxer	left	no	0	0	71,93	no
Zurich	The Longines FEI World Cup Jumping - Zurich round A	28.01.2018	CSI-W5*	3	1.60	430	22	13	01.03.2005	S	HOLST	155	8	121	2	38	1958	103	M	7	6b	yes	spread	oxer	left	yes	4	0	71,93	no
Zurich	The Longines FEI World Cup Jumping - Zurich round A	28.01.2018	CSI-W5*	3	1.60	430	22	13	01.03.2005	S	HOLST	155	8	121	2	38	1958	103	M	8	6c	yes	vertical	vertical	left	no	0	0	71,93	no
Zurich	The Longines FEI World Cup Jumping - Zurich round A	28.01.2018	CSI-W5*	3	1.60	430	22	13	01.03.2005	S	HOLST	155	8	121	2	38	1958	103	M	9	7	no	spread	oxer	right	no	0	0	71,93	no
Zurich	The Longines FEI World Cup Jumping - Zurich round A	28.01.2018	CSI-W5*	3	1.60	430	22	13	01.03.2005	S	HOLST	155	8	121	2	38	1958	103	M	10	8a	yes	vertical	vertical	right	no	0	0	71,93	no
Zurich	The Longines FEI World Cup Jumping - Zurich round A	28.01.2018	CSI-W5*	3	1.60	430	22	13	01.03.2005	S	HOLST	155	8	121	2	38	1958	103	M	11	8b	yes	spread	oxer	right	no	0	0	71,93	no
Zurich	The Longines FEI World Cup Jumping - Zurich round A	28.01.2018	CSI-W5*	3	1.60	430	22	13	01.03.2005	S	HOLST	155	8	121	2	38	1958	103	M	12	9	no	vertical	vertical	right	no	0	0	71,93	no
Zurich	The Longines FEI World Cup Jumping - Zurich round A	28.01.2018	CSI-W5*	3	1.60	430	22	13	01.03.2005	S	HOLST	155	8	121	2	38	1958	103	M	13	10	no	spread	triple bar	right	no	0	0	71,93	no
Zurich	The Longines FEI World Cup Jumping - Zurich round A	28.01.2018	CSI-W5*	3	1.60	430	22	13	01.03.2005	S	HOLST	155	8	121	2	38	1958	103	M	14	11	no	spread	oxer	left	no	0	0	71,93	no
Zurich	The Longines FEI World Cup Jumping - Zurich round A	28.01.2018	CSI-W5*	3	1.60	430	22	13	01.03.2005	S	HOLST	155	8	121	2	38	1958	103	M	15	12	no	vertical	vertical with water	left	no	0	0	71,93	no
Zurich	The Longines FEI World Cup Jumping - Zurich round A	28.01.2018	CSI-W5*	3	1.60	430	22	13	01.03.2005	S	HOLST	155	8	121	2	38	1958	103	M	16	13	no	spread	oxer	straight	no	0	0	71,93	no
Zurich	The Longines FEI World Cup Jumping - Zurich round A	28.01.2018	CSI-W5*	3	1.60	430	14	10	26.07.2008	M	ZfDP	107	0	130	88	46	1851	131	M	1	1	no	vertical	vertical	right	no	0	0	73,04	no
Zurich	The Longines FEI World Cup Jumping - Zurich round A	28.01.2018	CSI-W5*	3	1.60	430	14	10	26.07.2008	M	ZfDP	107	0	130	88	46	1851	131	M	2	2	no	spread	oxer	right	no	0	0	73,04	no
Zurich	The Longines FEI World Cup Jumping - Zurich round A	28.01.2018	CSI-W5*	3	1.60	430	14	10	26.07.2008	M	ZfDP	107	0	130	88	46	1851	131	M	3	3	no	vertical	wall	right	no	0	0	73,04	no
Zurich	The Longines FEI World Cup Jumping - Zurich round A	28.01.2018	CSI-W5*	3	1.60	430	14	10	26.07.2008	M	ZfDP	107	0	130	88	46	1851	131	M	4	4	no	spread	oxer with water	left	no	0	0	73,04	no
Zurich	The Longines FEI World Cup Jumping - Zurich round A	28.01.2018	CSI-W5*	3	1.60	430	14	10	26.07.2008	M	ZfDP	107	0	130	88	46	1851	131	M	5	5	no	vertical	vertical	left	no	0	0	73,04	no
Zurich	The Longines FEI World Cup Jumping - Zurich round A	28.01.2018	CSI-W5*	3	1.60	430	14	10	26.07.2008	M	ZfDP	107	0	130	88	46	1851	131	M	6	6a	yes	spread	oxer	left	no	0	0	73,04	no
Zurich	The Longines FEI World Cup Jumping - Zurich round A	28.01.2018	CSI-W5*	3	1.60	430	14	10	26.07.2008	M	ZfDP	107	0	130	88	46	1851	131	M	7	6b	yes	spread	oxer	left	no	0	0	73,04	no
Zurich	The Longines FEI World Cup Jumping - Zurich round A	28.01.2018	CSI-W5*	3	1.60	430	14	10	26.07.2008	M	ZfDP	107	0	130	88	46	1851	131	M	8	6c	yes	vertical	vertical	left	no	0	0	73,04	no
Zurich	The Longines FEI World Cup Jumping - Zurich round A	28.01.2018	CSI-W5*	3	1.60	430	14	10	26.07.2008	M	ZfDP	107	0	130	88	46	1851	131	M	9	7	no	spread	oxer	right	no	0	0	73,04	no
Zurich	The Longines FEI World Cup Jumping - Zurich round A	28.01.2018	CSI-W5*	3	1.60	430	14	10	26.07.2008	M	ZfDP	107	0	130	88	46	1851	131	M	10	8a	yes	vertical	vertical	right	no	0	0	73,04	no
Zurich	The Longines FEI World Cup Jumping - Zurich round A	28.01.2018	CSI-W5*	3	1.60	430	14	10	26.07.2008	M	ZfDP	107	0	130	88	46	1851	131	M	11	8b	yes	spread	oxer	right	no	0	0	73,04	no
Zurich	The Longines FEI World Cup Jumping - Zurich round A	28.01.2018	CSI-W5*	3	1.60	430	14	10	26.07.2008	M	ZfDP	107	0	130	88	46	1851	131	M	12	9	no	vertical	vertical	right	no	0	0	73,04	no
Zurich	The Longines FEI World Cup Jumping - Zurich round A	28.01.2018	CSI-W5*	3	1.60	430	14	10	26.07.2008	M	ZfDP	107	0	130	88	46	1851	131	M	13	10	no	spread	triple bar	right	no	0	0	73,04	no
Zurich	The Longines FEI World Cup Jumping - Zurich round A	28.01.2018	CSI-W5*	3	1.60	430	14	10	26.07.2008	M	ZfDP	107	0	130	88	46	1851	131	M	14	11	no	spread	oxer	left	no	0	0	73,04	no
Zurich	The Longines FEI World Cup Jumping - Zurich round A	28.01.2018	CSI-W5*	3	1.60	430	14	10	26.07.2008	M	ZfDP	107	0	130	88	46	1851	131	M	15	12	no	vertical	vertical with water	left	no	0	0	73,04	no
Zurich	The Longines FEI World Cup Jumping - Zurich round A	28.01.2018	CSI-W5*	3	1.60	430	14	10	26.07.2008	M	ZfDP	107	0	130	88	46	1851	131	M	16	13	no	spread	oxer	straight	no	0	0	73,04	no
Zurich	The Longines FEI World Cup Jumping - Zurich round B	28.01.2018	CSI-W5*	3	1.60	300	14	10	26.07.2008	M	ZfDP	107	0	130	88	46	1851	131	M	1	1	no	spread	oxer	left	no	0	0	42,08	no
Zurich	The Longines FEI World Cup Jumping - Zurich round B	28.01.2018	CSI-W5*	3	1.60	300	14	10	26.07.2008	M	ZfDP	107	0	130	88	46	1851	131	M	2	2	no	vertical	vertical	left	no	0	0	42,08	no
Zurich	The Longines FEI World Cup Jumping - Zurich round B	28.01.2018	CSI-W5*	3	1.60	300	14	10	26.07.2008	M	ZfDP	107	0	130	88	46	1851	131	M	3	3	no	spread	oxer	left	no	0	0	42,08	no
Zurich	The Longines FEI World Cup Jumping - Zurich round B	28.01.2018	CSI-W5*	3	1.60	300	14	10	26.07.2008	M	ZfDP	107	0	130	88	46	1851	131	M	4	4a	yes	vertical	vertical	right	no	0	0	42,08	no
[truncated: 357,883 more chars]
