# Supplementary figures and images for: Still beyond a chance: Distribution of faults in elite show-jumping horses
Source: PLoS One. 2022 Mar 16;17(3):e0264615. doi: 10.1371/journal.pone.0264615 (PMC8926200; doi:10.1371/journal.pone.0264615)

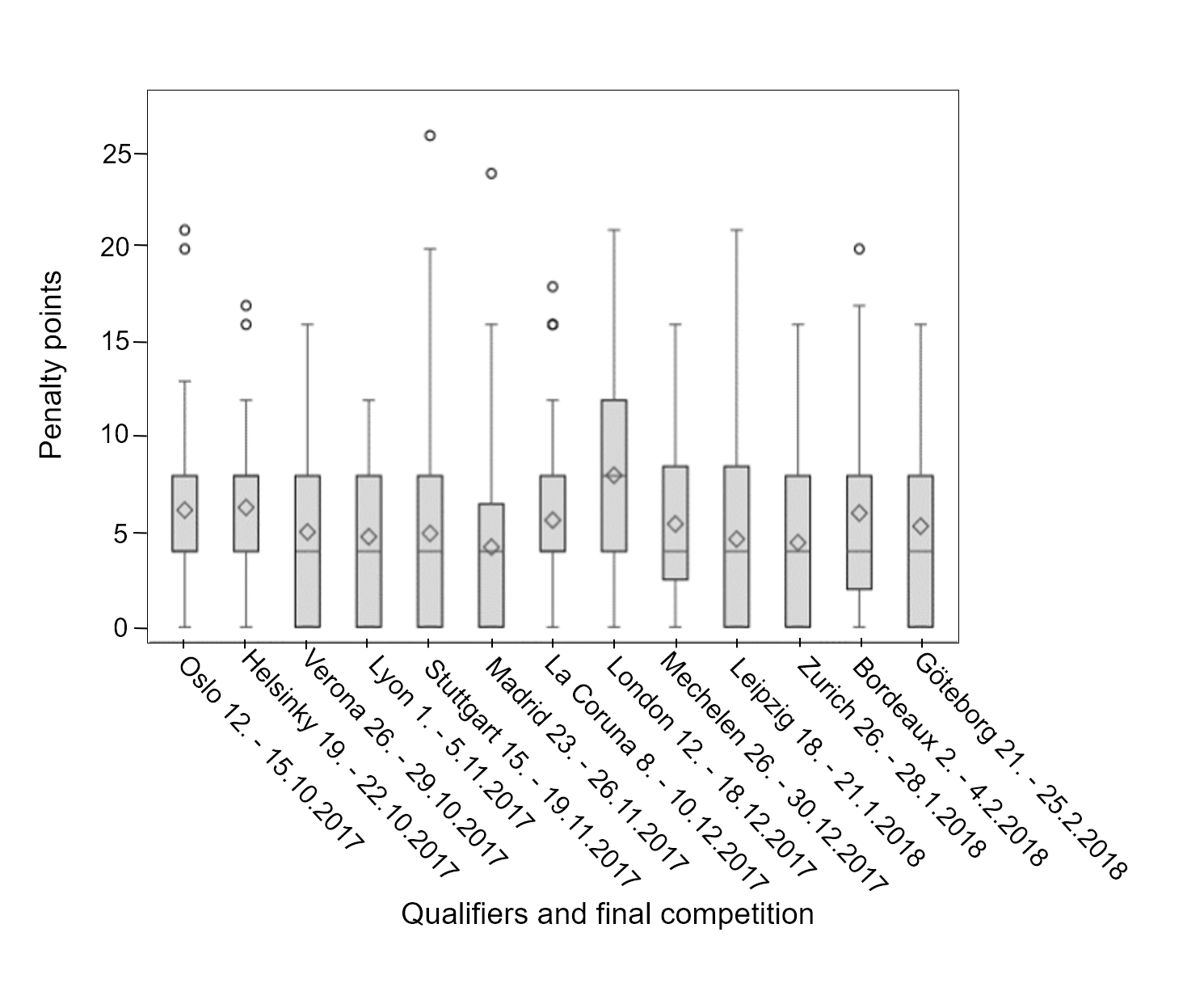

Supplement: S1 Fig — Distribution of the mean score of penalty points acquired by a rider-horse pair in particular qualifiers throughout the league. The central line in a box plot indicates the median, while the edges of the box indicate the first and third quartiles. Little circles beyond the whiskers are the outliers. (TIF) [file pone.0264615.s003.tif]

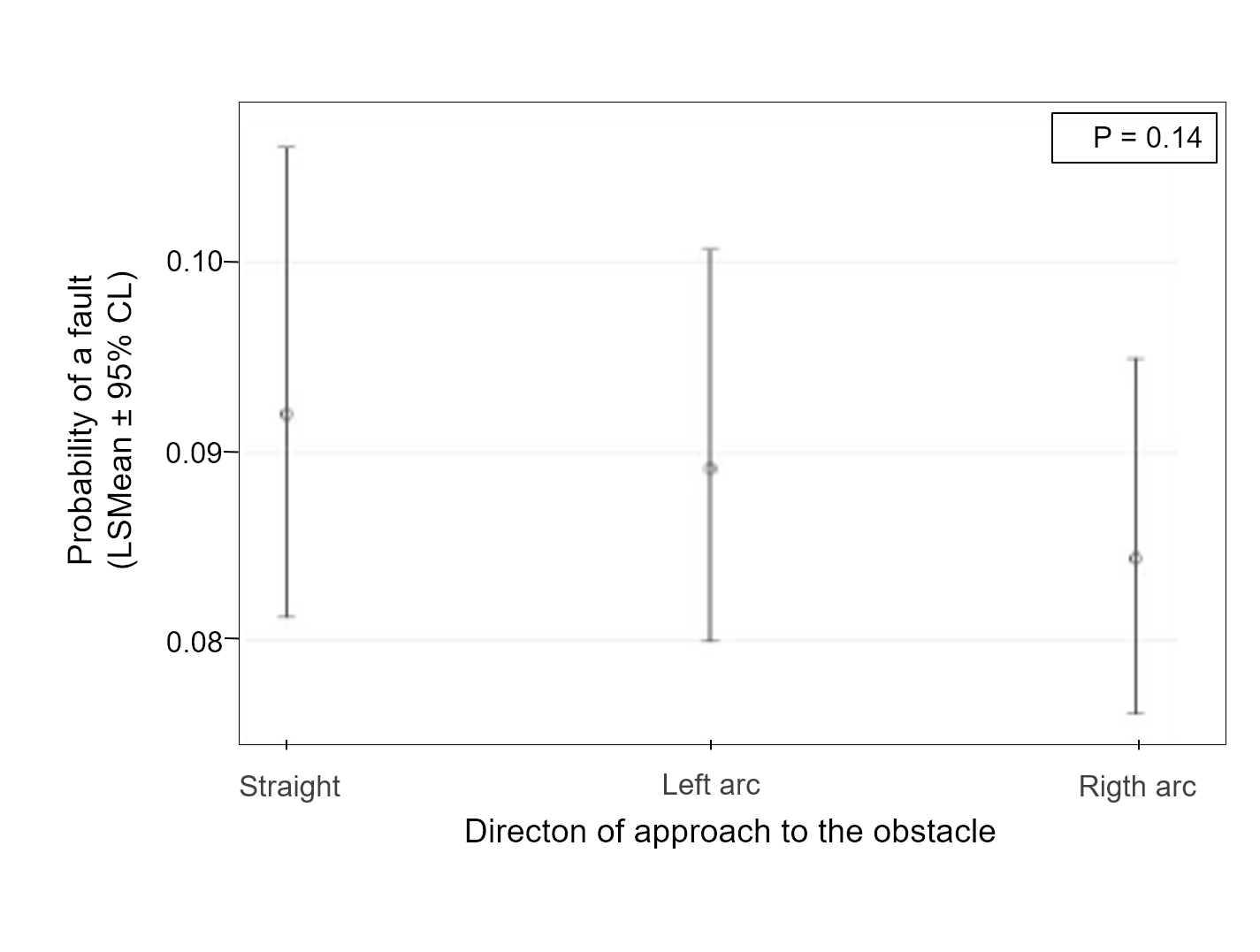

Supplement: S2 Fig — Predicted probability of a fault according to the direction of approach to the obstacle (LS MEANS ± 95% confidence limit). (TIF) [file pone.0264615.s004.tif]
